# Asymmetric Synthesis of Heterocyclic Chloroamines and Aziridines by Enantioselective Protonation of Catalytically Generated Enamines**

**DOI:** 10.1002/chem.202200060

**Published:** 2022-02-23

**Authors:** Liam A. McLean, Matthew W. Ashford, James W. B. Fyfe, Alexandra M. Z. Slawin, Andrew G. Leach, Allan J. B. Watson

**Affiliations:** ^1^ EaStCHEM, School of Chemistry University of St Andrews North Haugh St Andrews KY16 9ST, Fife UK; ^2^ School of Health Sciences University of Manchester Oxford Road Manchester M13 9PL UK

**Keywords:** asymmetric catalysis, aziridine, Brønsted acid, chloroamine, heterocycles

## Abstract

We report a method for the synthesis of chiral vicinal chloroamines via asymmetric protonation of catalytically generated prochiral chloroenamines using chiral Brønsted acids. The process is highly enantioselective, with the origin of asymmetry and catalyst substituent effects elucidated by DFT calculations. We show the utility of the method as an approach to the synthesis of a broad range of heterocycle‐substituted aziridines by treatment of the chloroamines with base in a one‐pot process, as well as the utility of the process to allow access to vicinal diamines.

## Graphical abstract

**Brønsted acid catalysis** enables the synthesis of chiral vicinal chloroamines via asymmetric protonation of catalytically generated prochiral chloroenamines. The process is highly enantioselective, with the origin of asymmetry and catalyst substituent effects elucidated by DFT calculations. The method allows access to rare heterocycle‐substituted vicinal chloroamines, aziridines, and vicinal diamines.

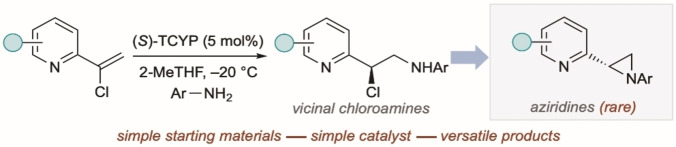

Vicinal chloroamines are key components of natural products and pharmaceuticals (e. g., Scheme 1a) and are broadly useful intermediates for chemical synthesis.[^1^, ^2^, ^3^, ^4^, ^5^, ^6^, ^7^] The value of this motif has driven the development of new methodologies for their preparation, which has been largely dominated by strategies for alkene aminohalogenation.[^6^, ^7^] Asymmetric processes are of particularly high value since nucleophilic substitution allows straightforward diversification to a range of new enantioenriched carbons bearing C‐heteroatom bonds. Most of these enantioselective methods use transition metal catalysis, with comparatively few examples of organocatalytic processes.^[7]^

**Scheme 1:**
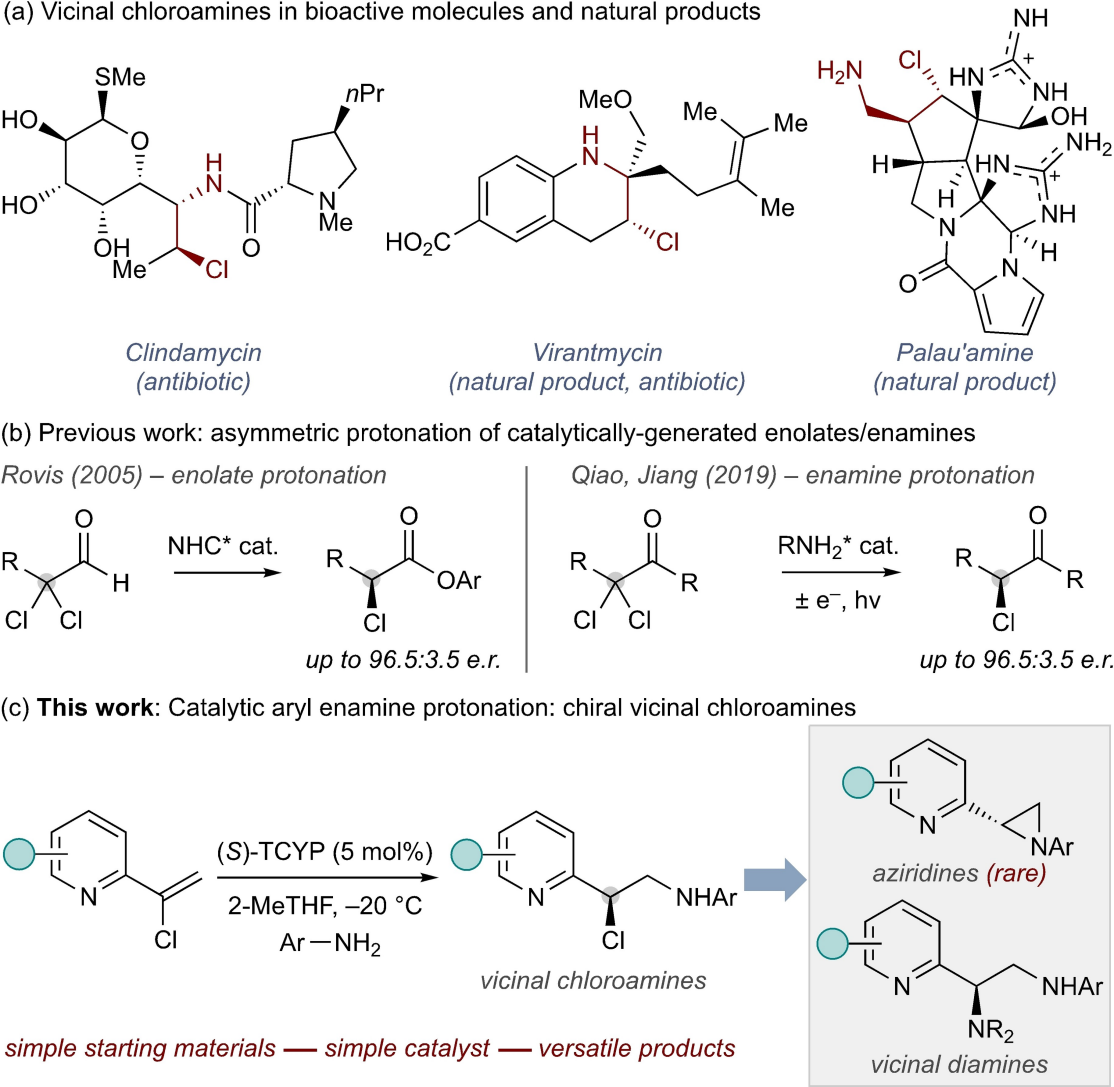
(a) Examples of vicinal chloroamines. (b) Previous work: catalytic enolate/enamine protonation. (c) This work: catalytic chloroenamine protonation – access to heterocyclic chloroamines, aziridines, and diamines.

To our knowledge, the formation of aryl vicinal chloramines via organocatalytic asymmetric protonation has not been reported. There are only four reports preparing chiral carbons with C−Cl bonds via enantioselective protonation, all of which generate α‐chlorocarbonyl products.[^8^, ^9^, ^10^, ^11^] Of these, only two proceed via asymmetric protonation of a catalytically generated species (Scheme 1b). Rovis employed NHC catalysis for the synthesis of α‐chloroacids.^[9]^ Qiao and Jiang used similar starting materials to generate α‐chloroketones using dual amine/photoredox catalysis.^[11]^ This latter process represents the only example of an enantioselective protonation of a catalytically generated prochiral chloroenamine.[^12^, ^13^]

Here we report the development of a new approach for the synthesis of chiral carbons bearing C−Cl bonds via enantioselective protonation of catalytically generated prochiral chloroenamines formed from an initial 1,4‐addition (Scheme 1c).[^14^, ^15^, ^16^, ^17^] This process allows simultaneous formation of a new C−N bond and generation of a new Cl‐substituted stereogenic centre under simple reaction conditions, and access to vicinal chloroamines.

Only three examples of catalytic asymmetric synthesis of heterocycle‐substituted aziridines have been reported,[^18^, ^19^, ^20^] and all of these prepare the same product. This new strategy therefore simultaneously addresses a limitation in aziridination methodologies, while also allowing access to diamines.

A benchmark system using chlorovinyl quinoline **1** and aniline was used for reaction development (Table 1). An initial screening campaign identified promising conditions using 20 mol% catalyst **3** at −20 °C in CPME (Entry 1; for full screening details, see Tables S1–S6).[^14^, ^15^] However, further attempts at reaction optimization did not improve asymmetric induction. We therefore conducted an extensive catalyst screen (see Table S1), which, in combination with DFT analysis (see below), allowed catalyst SAR to be rationalized and provided insight into substituent effects more broadly. The parent, unsubstituted BINOL catalyst **4** delivers low enantioselectivity (entry 2), as expected from prior work in this field.^[21]^ Systematic exploration of the aryl groups at the 3,3’‐substituents to understand the selectivity determinants of catalyst **3** was informative. As expected,^[21]^ 3,3’‐diphenyl catalyst **5** delivered improvement upon the enantioselectivity offered by unsubstituted catalyst **4** (entry 3). However, introduction of an *i*‐Pr group at the 4‐position of the 3,3’‐aryl units (**6**) had no effect on enantioselectivity (entry 4), suggesting this position has very little effect on enantiofacial discrimination.

**Table 1:** Reaction development.

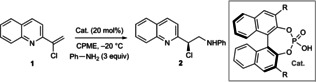		
Entry	R (catalyst)	**2** [%] (e.r.)^[a]^
1	2,4,6‐(*i*‐Pr)_3_C_6_H_2_ (**3**)	98 % (90 : 10)
2	H (**4**)	90 % (51 : 49)
3	Ph (**5**)	71 % (64 : 36)
4	4‐(*i*‐Pr)C_6_H_4_ (**6**)	97 % (67 : 33)
5	3,5‐(*i*‐Pr)_2_C_6_H_3_ (**7**)	73 % (74 : 26)
6	3,5‐(*t*‐Bu)_2_C_6_H_3_ (**8**)	70 % (71 : 29)
7	2,6‐(*i*‐Pr)_2_C_6_H_3_ (**9**)	77 % (88 : 12)
8	2,4,6‐(*c*‐Pent)_3_C_6_H_2_ (**10**)	95 % (90 : 10)
9	2,4,6‐(*c*‐Hex)_3_C_6_H_2_ (**11**)	96 % (96 : 4)
10	2,4,6‐(*c*‐Hept)_3_C_6_H_2_ (**12**)	95 % (97 : 3)
**11^[b]^**	**2,4,6‐(*c*‐Hex)_3_C_6_H_2_ (11)**	**96 % (97 : 3)**

[a] Determined by HPLC analysis. [b] Reaction conditions: **11** (5 mol%), 2‐MeTHF, PhNH_2_ (1 equiv).

Selectivity increased marginally by moving the *i*‐Pr groups to the 3,5‐positions (**7**, entry 5); however, selectivity remained significantly lower than for **3** even when the steric component was increased, for example, with *t*‐Bu‐substituted catalyst **8** (entry 6). These data suggested the 3‐,4‐, and 5‐positions of the 3,3’ aryl units exert a weak influence on stereoselectivity. This was compounded by assessment of catalyst **9**, which lacks the 4‐(*i*‐Pr) unit yet delivers approximately the same enantioinduction as **3** (entry 7 vs. entry 1). The lower importance of the 3‐, 4‐, and 5‐positions of the aryl unit was further supported by assessment of a range of functional groups at these positions on the BINOL scaffold as well as in alternative catalyst frameworks (Table S1). However, while having little to no effect on enantioinduction, the 4‐position of these aryl units was found to directly affect reactivity: increased conversion (ca. 20 %) to product was observed for those catalysts with 4‐*i*‐Pr compared to those without (**5** vs. **6**, **9** vs. **3**).

Second phase catalyst screening revealed the introduction of cycloalkyl groups to the 3,3’ aryl units improved enantioselectivity while remaining synthetically tractable. *c*‐Pent derivative **10** ^[22]^ was found to be equivalent to *i*‐Pr catalyst **3** (entry 8); however, *c*‐Hex catalyst **11** (TCYP),^[23]^ and *c*‐Hept catalyst **12** (entry 10) provided marked improvement, with **11** subsequently offering excellent reactivity and selectivity at 5 mol% loading and equistoichiometric PhNH_2_ in 2‐MeTHF (entry 11).

The reaction conditions identified in entry 11 were assessed for generality by application towards the synthesis of a range of vicinal chloroamines (Scheme 2a). Broad variation of the aniline nucleophile was generally accommodated, delivering the expected chloroamines in good yield and with high enantioselectivity throughout; however, **17** was noted as a clear exception, providing racemic product, which could be related to the p*K*_a_ of this specific aniline. Confirmation of product stereochemistry was achieved by crystallization of **20**, which also highlighted the solid‐state conformational preferences of the chloroamine, where the C−Cl is *gauche* to the quinoline nitrogen and *anti‐*periplanar to the aniline. Variation of the azaheterocycle was also generally successful (**26**–**35**), although significant variation in reactivity was noted and driven by changes in heteroaryl electronics. Other *N*‐nucleophiles (e. g., alkyl amines) were not tolerated in this process due to competing protonation events.

**Scheme 2:**
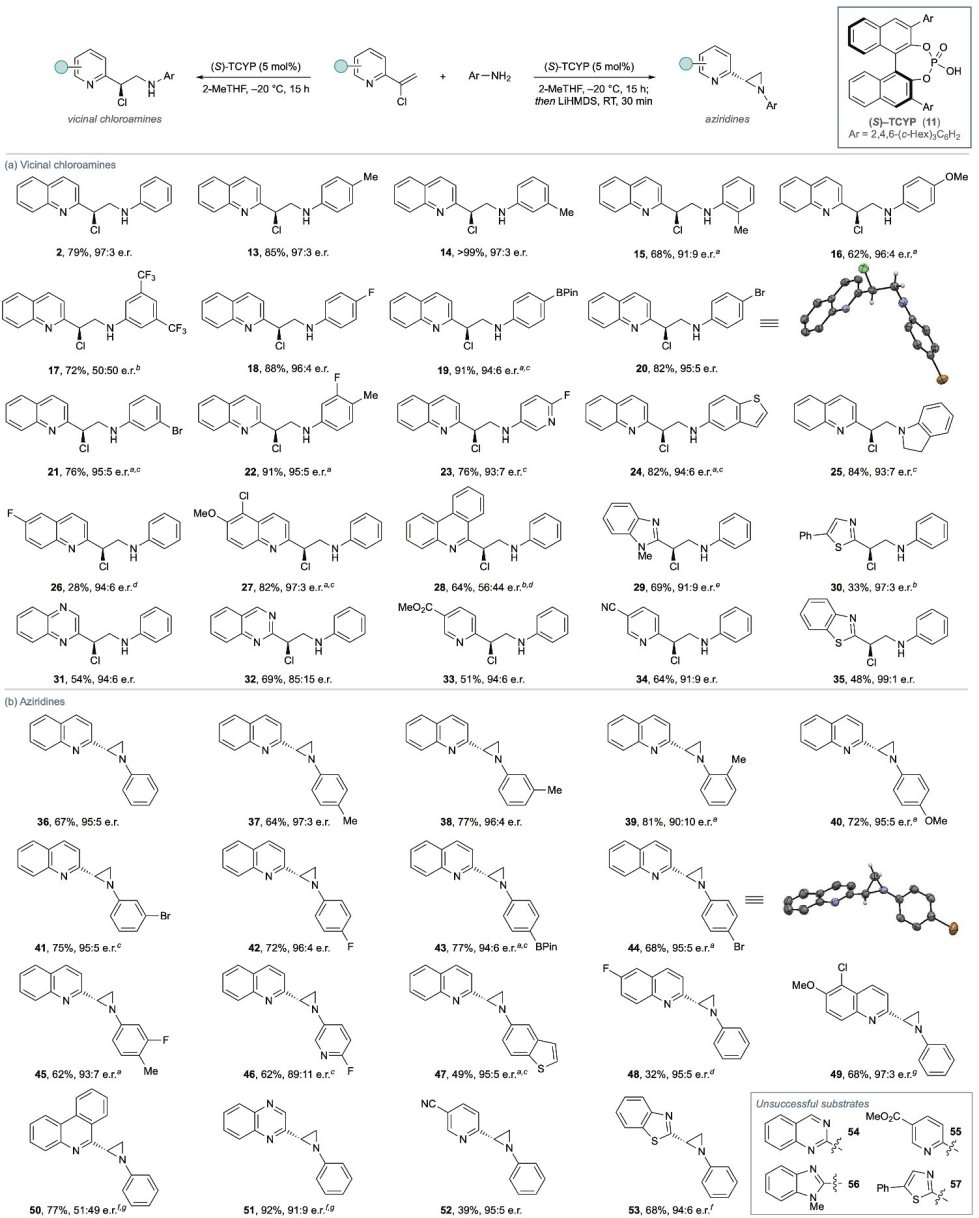
(a) Example scope of the chloroamine process. (b) Example scope of the aziridination process. Isolated yields. Enantiomeric ratios determined by HPLC analysis. ^[a]^ 39 h; ^[b]^ RT; ^[c]^ CPME; ^[d]^ 4 days; ^[e]^ 0 °C; ^[f]^ PhMe; ^[g]^ Accessed from isolated chloroamine.

As noted above, this approach to vicinal chloroamines can allow access to rare heterocycle‐substituted aziridines (Scheme 2b). Following the asymmetric protonation process, in situ treatment of the chloroamine with base delivered a library of products **36**–**53**. Variation in the *N*‐aryl component was straightforward, with structural data of complementary product **44** (100 % *es*) confirming the absolute stereochemistry. Variation of the N‐heterocycle was also tolerated; however, in this case the observed variation in reaction efficiency was due to the stability of the aziridine products, several of which were found to be particularly reactive and unstable to isolation (e. g., **54**–**57**). In addition, the S_N_i event was found to have high solvent dependency: a solvent switch was necessary for **51** and **53** where THF led to completely racemic products, presumably through a more ionic pathway, while toluene allowed for exclusive S_N_i.

Access to the aziridine products above allows further derivatization. For example, heterocyclic vicinal diamines are also relatively rare chemotypes. However, these can be prepared by treatment of the heterocyclic aziridines with, for example, a cyclic amine in the presence of catalytic TFA giving **58**–**60** with high fidelity for S_N_2 at the benzylic stereocenter (Scheme 3).

**Scheme 3:**
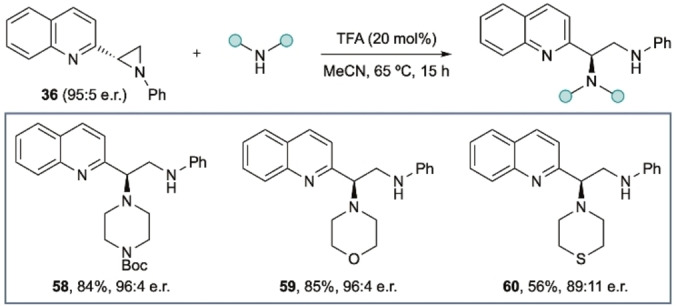
Synthesis of heterocyclic vicinal diamines.

To gain greater insight into catalyst SAR and rationalize the superior selectivity observed using TCYP (**11**) vs. TRIP (**3**), we undertook an in‐depth computational evaluation of the system using the ONIOM method with B3LYP/6‐31G**^[24]^ for the QM region and UFF^[25]^ for the MM region.^[26]^ Calculations were performed using Gaussian09 (see Supporting Information).[^14^, ^15^, ^27^] A full reaction profile is provided in the Supporting Information (Figure S1).

Increased enantioselectivity offered by catalysts **10**–**12** in comparison to **3** (see Figure 1 for comparison of **5** vs. **6** and **3** vs. **11**) results from a catalyst‐transition state complex, which adopts a preferred conformation where the C−Cl bond is *anti* with respect to the catalyst P=O (Figure S7) and this is not possible for the pro‐*S* transition state due to the 3,3’‐substituents: this is a key contributor to enantioselectivity. In the alternative conformation that must be adopted when the catalyst has a 3,3’‐substituent, the aniline unit adopts an *anti*‐orientation that places it between two of the 2,6‐alkyl substituents: these are then key to enantioselectivity in the rate‐determining proton transfer event. As the substituent bulk is increased from Ph (catalyst **5**) to substituted phenyl systems with *i*‐Pr (catalysts **3** and **6**) and cycloalkyl (catalysts **10**–**12**) groups, transition state‐stabilizing contacts increase between the alkyl group at the 4‐position and the aniline unit in the favoured transition state. This serves to strengthen preference for the observed enantiomer. In contrast, moving from *i*‐Pr to larger cycloalkyl groups increasingly destabilizes the transition state that leads to the disfavoured enantiomer by compressing the gap that the aniline occupies – the aniline is effectively extruded and must rotate away.

**Figure 1:**
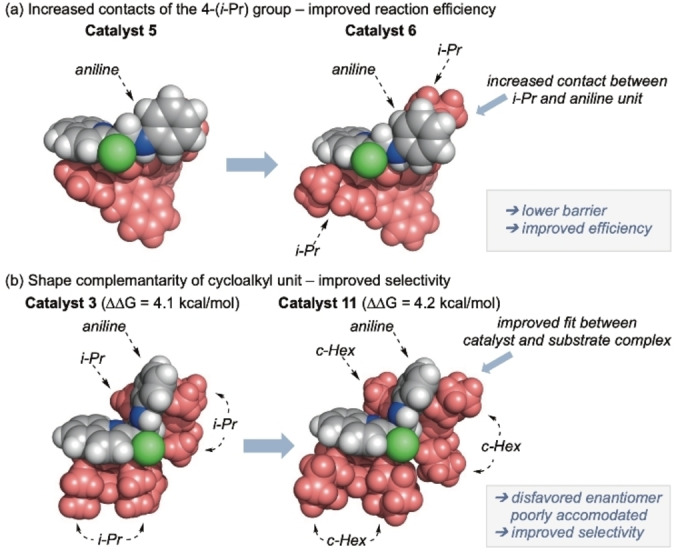
Space‐filling models of the effect of (a) the 4‐(*i*‐Pr) group and (b) cycloalkyl groups. The rate‐limiting transition states are shown in CPK representation to illustrate contacts. There is a small reduction in ▵G^≠^ on addition of 4‐(*i*‐Pr) from 23.2 to 23.1 kcal/mol corresponding to an increased reaction efficiency for catalyst **6** compared to catalyst **5**. There is also a lower ▵G^≠^ of 21.9 kcal/mol for catalyst **3** than for **11** with ▵G^≠^ of 23.1 kcal/mol. Selectivity (▵▵G^≠^) sees a computed improvement on going from **3** to **11** with a change from 4.1 to 4.2 kcal/mol. Further details are provided in the Supporting Information including links between computed ▵▵G^≠^ value and experimental selectivity and some rationalization for changes in yields.

Thus, good shape complementarity with the 4‐alkyl in the pro‐*R* transition state and space‐filling by the 2,6‐alkyl groups in the pro‐*S* transition state lead to the observed improvement in enantioselectivity with the cycloalkyl catalysts. Specifically, there is an increase in ΔΔG^≠^(*S−R*) on moving from **3** to **11**, consistent with the experimentally observed improvement in enantioinduction; the computed stereoselectivity for **11** is in excellent agreement with that measured while **3** is computed to be more selective than measured and thus agreement is qualitative.

In summary, a method for the synthesis of chiral heterocyclic vicinal chloroamines has been developed. The reaction relies upon asymmetric protonation of a catalytically generated aryl chloroenamine using a chiral Brønsted acid and represents the first example of this process. A range of chloramine products can be accessed and, in turn, provide access to rare heterocyclic aziridines and diamines, bridging a significant gap in synthetic methodologies for the preparation of these product classes. Computational analysis has assisted in rationalizing observations of increased reactivity and enantioselectivity with cycloalkyl CPA catalysts, providing insight into the specific function of these alkyl units in these processes.[^28^, ^29^]

## Conflict of interest

The authors declare no conflict of interest.

## Supporting information

As a service to our authors and readers, this journal provides supporting information supplied by the authors. Such materials are peer reviewed and may be re‐organized for online delivery, but are not copy‐edited or typeset. Technical support issues arising from supporting information (other than missing files) should be addressed to the authors.

Supporting Information

## Data Availability

The data that support the findings of this study are openly available in University of St Andrews at https://doi.org/10.17630/816af4fa‐ef6a‐4043‐b0f9‐b88f4f8a47f1, reference number 26.
