# Supplementary material for: Asymmetric Synthesis of Heterocyclic Chloroamines and Aziridines by Enantioselective Protonation of Catalytically Generated Enamines
Source: Chemistry. 2022 Feb 23;28(16):e202200060. doi: 10.1002/chem.202200060 (PMC9306946; doi:10.1002/chem.202200060)
Supplement: Supplementary file 1 — Supporting Information [file CHEM-28-0-s001.pdf]

Chemistry–A European Journal

Supporting Information

Asymmetric Synthesis of Heterocyclic Chloroamines and Aziridines by Enantioselective Protonation of Catalytically Generated Enamines

Liam A. McLean, Matthew W. Ashford, James W. B. Fyfe, Alexandra M. Z. Slawin, Andrew G. Leach, and Allan J. B. Watson*

Contents

1. General Information 2
2. General Experimental Procedures 2
3. Optimization Study 5
4. Characterization Data for Compounds 9
5. X-Ray Data 101
6. Computational Chemistry 102
7. References 115

1. General Information

Purification of Solvents

Dry THF, DCM, hexane, toluene, and Et_2O for reactions were obtained from a PureSolv SPS-400-5 solvent purification system. All other solvents for reactions were obtained from commercial suppliers and purification was carried out according to standard laboratory methods.[1] Petroleum ether refers to the petroleum ether fraction in the boiling point (b.p.) range 40-60 °C unless otherwise stated. Et_2O, EtOAc, toluene, cyclohexane, petroleum ether and hexane for purification purposes were used as obtained from suppliers without further purification.

Experimental Details

Reactions were carried out using conventional glassware (preparation of intermediates), or in 2 mL HPLC vials. The glassware was oven-dried (150 °C) and purged with nitrogen or argon before use. Purging refers to a vacuum/nitrogen-refilling procedure. TCYP refers to 3,3′-bis(2,4,6-tricyclohexylphenyl)-1,1′-binaphthyl-2,2′-diyl hydrogenphosphate. Room temperature (rt) was generally *ca.* 18 °C. Reactions were carried out at elevated temperatures using a temperature-regulated hotplate/stirrer with a sand bath. Reactions were carried out at 0, −10, −20, −50, and −78 °C using an acetone bath cooled by a Thermo Haake EK90 cryocooler. Reactions were carried out at 0 °C using an ice/water bath. Acid-washed zinc was prepared acorrding to the literature procedure.[1] $NiCl_2(PPh_3)_2$ was synthesised fresh, following the literature procedure.[2] Additionally, $NiCl_2(PPh_3)_2$ was recrystallised from AcOH, dried, and stored in a desiccator over Drierite™ drying agent.

Purification of Products

Thin layer chromatography was carried out using Merck silica plates coated with fluorescent indicator UV254. These were analyzed under 254 nm UV light, developed using potassium permanganate or vanillin solutions. Normal phase flash chromatography was carried out using ZEOprep 60 HYD 40-63 µm silica gel.

Analysis of Products

Fourier Transformed Infra-Red (FTIR) spectra were obtained on a Shimadzu IRAffinity-1 machine. ^{1}H and ^{13}C NMR spectra were obtained on either a Bruker AV 400 at 400 MHz and 101 MHz, respectively, or Bruker DRX 500 at 500 MHz and 126 MHz, respectively. ^{31}P NMR spectra were obtained on either a Bruker AV 400 at 400 MHz, 162 MHz or a Bruker AV 300 spectrometer at 121 MHz. ^{19}F NMR spectra were obtained on a Bruker AV 500 spectrometer at 470 MHz. ^{11}B NMR spectra were obtained on a Bruker AV 300 spectrometer at 96 MHz. Chemical shifts are reported in ppm and coupling constants are reported in Hz with $CDCl_3$ referenced at 7.26 (^{1}H) and 77.0 ppm (^{13}C). ^{11}B NMR spectra are referenced to $BF_3{\cdot}Et_2O$. Unless otherwise stated, J refers to $^{3}J_{HH}$ and J_{CF} in ^{1}H and ^{13}C NMR, respectively. All NMR spectra were collected at 298 K. High-resolution mass spectra were obtained through analysis at the University of St Andrews. High performance liquid chromatography (HPLC) was performed on an Agilent 1200 series HPLC using a chiral stationary phase column (column, Daicel Co. CHIRALCEL OJ-H, CHIRALPAK IA or CHIRAL ART Amylose-C; eluent: *n*-hexane/*i*-PrOH). All solvents used were HPLC-grade solvents purchased from Fisher. The column employed and the respective solvent mixture are indicated for each experiment. Optical rotations were obtained on a Perkin Elmer Model 341 polarimeter.

2. General Experimental Procedures

General Procedure A: Aza-Michael reaction catalyzed using a chiral acid.

A 2 mL HPLC vial, equipped with a stir-bar, was charged with vinyl chloride (1.0 equiv.), and (*S*)-TCYP (5 mol%). The vial was then capped and purged with nitrogen before addition of 2-MeTHF (0.5 M).

The reaction mixture was cooled to −20 °C for 15 min before the addition of aniline (1.0 equiv.). The resulting mixture was then allowed to stir at −20 °C for 15 h before being quenched by sat. aq. $NaHCO_3$ solution (2 mL) and diluted with EtOAc (2 mL). The phases were separated and the aqueous phase was washed with EtOAc (2 mL × 2). The organic phases were combined, dried over Na_2SO_4, filtered, and concentrated *in vacuo*. The crude product was purified using flash column chromatography (silica, EtOAc:hexane or Et_2O:hexane) to afford the desired product. The enantiomeric ratio was determined by chiral HPLC in comparison with the racemate.

Racemic chloroamines were synthesised by substituting (*S*)-TCYP in General Procedure A for TFA (20 mol%). These reactions were carried out at rt.

General Procedure B: **One-Pot Aza-Michael-Ring Closure Reaction catalyzed using a chiral acid.**

A 2 mL HPLC vial, equipped with a stir-bar, was charged with vinyl chloride (1.0 equiv.), and (*S*)-TCYP (5 mol%). The vial was then capped and purged with nitrogen before addition of 2-MeTHF (0.5 M). The reaction mixture was cooled to −20 °C for 15 min before the addition of aniline (1.0 equiv.). The resulting mixture was then allowed to stir at −20 °C for 15 h. The reaction was then allowed to warm to rt and a 0.6 M solution of LiHMDS in THF (1.2 equiv.) was added, and the reaction was allowed to stir for a further 30 min at rt before being quenched by sat. aq. NH_4Cl solution (2 mL) and diluted with EtOAc (2 mL). The phases were separated and the aqueous phase was washed with EtOAc (2 mL × 2). The organic phases were combined, dried over Na_2SO_4, filtered, and concentrated *in vacuo*. The crude product was purified using flash column chromatography (silica, EtOAc:hexane) to afford the desired product. The enantiomeric ratio was determined by chiral HPLC in comparison with the racemate.

Racemic aziridines were synthesised by treating racemic chloroamines with 0.6 M solution of LiHMDS in THF, in an analgous method described in General Procedure B.

Synthetic Routes to Starting Materials

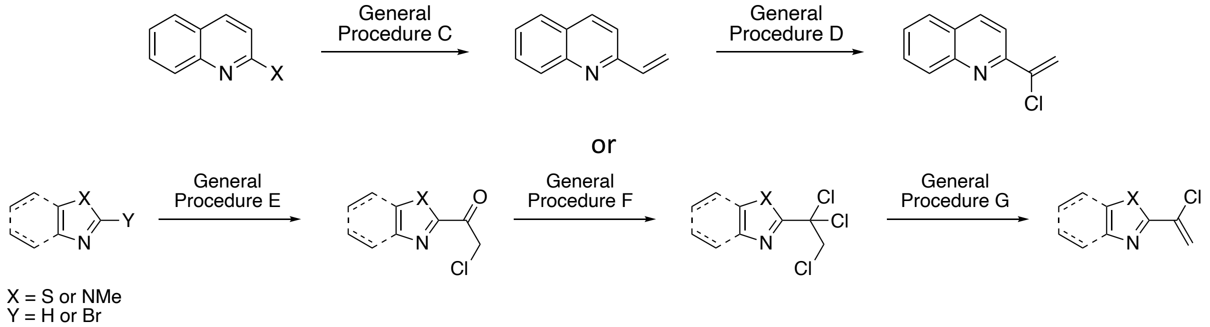

General Procedure C: Suzuki–Miyaura coupling.

Prepared according to literature procedure.[3]

An oven-dried 100 mL round bottomed flask, equipped with a reflux condensor, was charged with 2-chloro/bromoheterocycle (1.0 equiv.), vinylboronic acid pinacol ester (1.1 equiv.), $Pd(OAc)_2$ (4 mol%), S-Phos (8 mol%), K_3PO_4 (3.0 equiv.). The system was capped and purged. 1,4-Dioxane (20 mL) and water (5.0 equiv.) were added and the reaction was stirred at 80 °C for 6 h. The mixture was then allowed to cool to rt, filtered through Celite®, and concentrated *in vacuo*. The crude product was purified using flash column chromatography (silica, EtOAc:petroleum ether) to afford the desired product.

General Procedure D: Dichlorination and Dehydrochlorination of Vinylheterocycles.

A 100 mL oven-dried RBF, equipped with a stir-bar and wrapped in aluminium foil, was charged with 2-vinylheterocycle (1.0 equiv.) capped and purged with nitrogen. Dry DCM (0.5 M) was then added

and the solution was cooled to −78 °C (Flask A). Seperately, a 100 mL oven-dried RBF was charged with $KMnO_4$ (10.0 equiv.). The flask was capped and purged with nitrogen (Flask B). Rubber tubing, with syringes fitted with needles at both ends filled with calcium chloride, pierced both flasks A and B, and with the needle submerged within the solution of Flask A. An unfilled balloon was then fitted to Flask A, and 37% aq. HCl (10.0 equiv.) was then added dropwise to the Flask B. After 10 minutes the rubber tubing was removed. The reaction in Flask A was left to stir at −78 °C for 30 min. The reaction was then quenched with a sat. aq. solution of Na_2SO_3 (50 mL). The phases were separated and the aqueous phase was washed with DCM (50 mL × 2). The organic phases were combined, dried over Na_2SO_4, filtered, and concentrated *in vacuo* (25 °C bath). The crude was used directly in the next step.

A 100 mL RBF, equipped with a stir-bar, was charged with the crude dichloro species (assumed complete conversion) the flask was capped and purged with nitrogen. The flask was submerged in an ice bath and dry DCM (0.5 M) was added followed by KO^tBu (2 equiv.), and the reaction was stirred for 30 min at 0 °C (Caution! Reaction is exothermic). The reaction was then diluted with water, the phases were separated and the aqueous phase was washed with DCM (50 mL × 2). The organic phases were combined, dried over Na_2SO_4, filtered, and concentrated *in vacuo* (25 °C bath). The crude product was purified using flash column chromatography (silica, EtOAc:hexane or Et_2O:hexane) to afford the desired product.

General procedure E: Aryl Ketone Synthesis using Turbo Grignard®.

2-Chloro-*N*-methoxy-*N*-methylacetamide was prepared according to literature procedure.[4]

A flame-dried two-necked flask, equipped with stir-bar and purged with argon, was charged with 2-bromoheterocycle/heterocycle (1.0 equiv.) and THF (0.4 M). The mixture then cooled to 0 °C and *iso*propyl magnesium chloride lithium chloride complex (1.0 equiv.) was added dropwise to give a yellow/brown solution and the reaction was allowed to stir for 30 mins. To the yellow/brown solution was added a solution of 2-chloro-*N*-methoxy-*N*-methylacetamide (1.0 equiv.) in THF (0.4 M) dropwise which gave a dark solution which was stirred for a further 2 h. The reaction was allowed to warm to room temperature, and then quenched by addition of sat. aq. NH_4Cl and extracted with EtOAc (3 x). The organic phases were combined, dried over Na_2SO_4, filtered, and the solvent concentrated *in vacuo* to give the desired aryl ketone.

General Procedure F: Geminal Dichloride Synthesis.

An oven-dried microwave vial, equipped with a stir-bar, was charged with phosphorus pentachloride (1.3 equiv.) and benzene (4.70 M). To the resulting mixture was added aryl ketone (1.0 equiv.) and stirred for 2 h at reflux. The resulting dark mixture was cooled and poured into ice-water (20 mL) and extracted with Et_2O (3 x). The organic phases were combined, dried over Na_2SO_4, filtered, and had the solvent concentrated *in vacuo* to give a dark residue. The crude product was purified using flash column chromatography (silica, EtOAc:hexane) to afford the desired product.

General Procedure G: Vinyl Chloride Synthesis with Zn Metal.

A microwave vial, equipped with a stir-bar, was charged with geminal dichloride (1.0 equiv.) and was dissolved in EtOH (0.25 M) and acid-washed zinc dust (1.0 equiv.) was added, the reaction mixture was then stirred at reflux until the zinc dust was visibly consumed. The reaction was allowed to cool and then filtered through Celite®, and the solvent was removed *in vacuo* to give a brown residue. The crude product was purified using flash column chromatography (silica, EtOAc:hexane or Et_2O:toluene) to afford the desired product.

General Procedure H: Nucleophilic Ring-Opening Reaction.

A 2 mL HPLC vial, equipped with a stir-bar, was charged with (*R*)-2-(1-phenylaziridin-2-yl)quinoline (1.0 equiv.) and nucleophile (1.0 equiv.). The vial was then capped and purged with nitrogen before addition of MeCN (0.5 M) and TFA (20 mol%). The reaction mixture was heated to 65 °C and left to stir for 16 h before being quenched by sat. aq. $NaHCO_3$ solution (2 mL) and diluted with EtOAc (2 mL). The phases were separated and the aqueous phase was washed with EtOAc (2 mL × 2). The organic phases were combined, dried over Na_2SO_4, filtered, and concentrated *in vacuo*. The crude product was purified using flash column chromatography (silica, EtOAc:hexane) to afford the desired product. The enantiomeric ratio was determined by chiral HPLC in comparison with the racemate.

Racemates were synthesised by treating racemic aziridines with the appropriate nucleophile, as described in General Procedure H.

3. Optimization Study

Optimisation study of the aza-Michael reaction catalyzed using a chiral acid was carried out using general procedure A with modification of the appropriate reaction parameters.

Table S1: Catalyst Screening

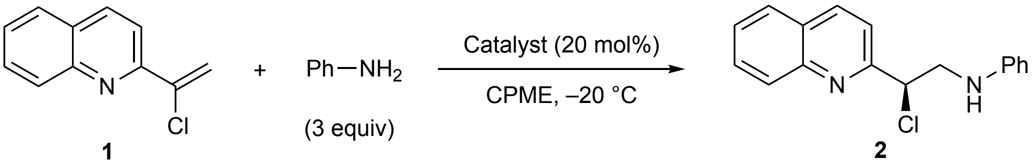

1 **2**

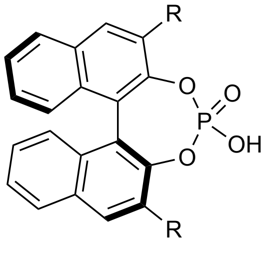

3: R = 2,4,6-(i-Pr)$_3C_6H_2$
4: R = H
5: R = C_6H_5
6: R = 4-(i-Pr)C_6H_4
7: R = 3,5-(i-Pr)$_2C_6H_3$
8: R = 3,5-(t-Bu)$_2C_6H_3$
9: R = 2,6-(i-Pr)$_2C_6H_3$
10: R = 2,4,6-(c-Pent)$_3C_6H_2$
11: R = 2,4,6-(c-Hex)$_3C_6H_2$
12: R = 2,4,6-(c-Hept)$_3C_6H_2$
S1: R = 3,5-$(CF_3)_2C_6H_3$
S2: R = $CHPh_2$

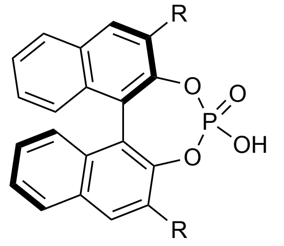

S3: R = 3,5-(Mes)$_2C_6H_3$
S4: R = 3,5-(MeO)$_2C_6H_3$
S5: R = 9-anthracene
S6: R = $SiPh_3$

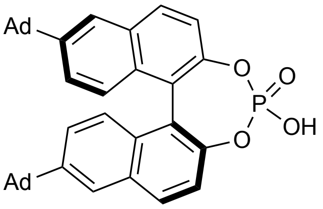

S7

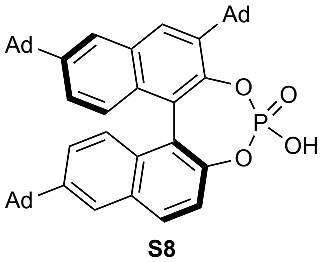

S8

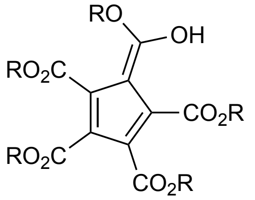

S9: R = Menthol

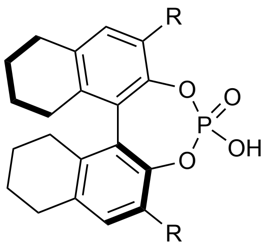

S10: R = 2,4,6-(i-Pr)$_3C_6H_2$
S11: R = 4-ClC_6H_4

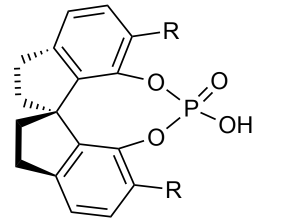

S12: R = 2,4,6-(i-Pr)$_3C_6H_2$

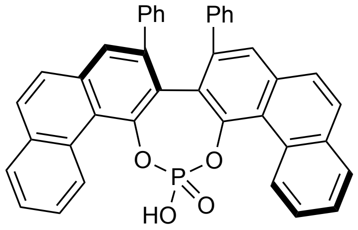

S13

Entry	*Catalyst*	*Time (h)*	*Conv. (%)*	*e.r.*
1	**3**	15	98	90:10
2	**4**	15	90	51:49

3	**5**	39	71	64:36
4	**6**	15	97	67:33
5	**7**	15	73	74:26
6	**8**	144	70	71:29
7	**9**	15	77	88:12
8	**10**	15	95	90:10
9	**11**	15	96	96:4
10	**12**	15	95	97:3
11	**S1**	15	90	70:30
12	**S2**	39	97	57:43
13	**S3**	15	90	44:56
14	**S4**	15	92	43:57
15	**S5**	15	84	18:82
16	**S6**	48	98	42:58
17	**S7**	39	91	53:47
18	**S8**	96	87	68:32
19	**S9**	15	95	52:48
20	**S10**	15	85	87:13
21	**S11**	96	92	65:35
22	**S12**	15	91	26:74
32	**S13**	24	95	68:32

The conversion was determined by ^{1}H NMR analysis with an internal standard, and e.r. was determined by HPLC analysis.

Table S2: Solvent Screening

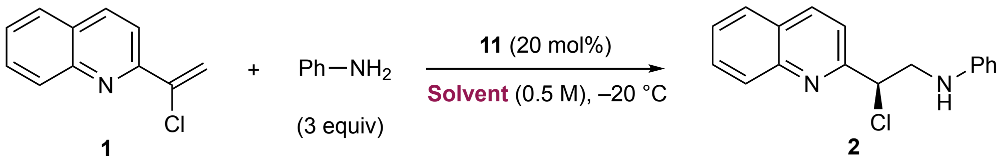

Entry	*Solvent*	*Time (h)*	*Conv. (%)*	e.r.
1	CPME	15	96	96:4
2	THF	15	74	94:6
3	Et_2O	15	99	91:9
4	2-MeTHF	15	91	96:4
5	DME	15	79	80:20
6	Anisole	15	97	89:11
7	TBME	15	95	95:5
8	Hexane	15	76	74:26
9	Toluene	15	98	71:29
10	DCM	15	98	68:32
11	DEC	15	92	91:9
12	EtOAc	15	93	88:12
13	IPA	15	87	51:49

The conversion was determined by ^{1}H NMR analysis with an internal standard, and e.r. was determined by HPLC analysis.

Table S3: Temperature Screening

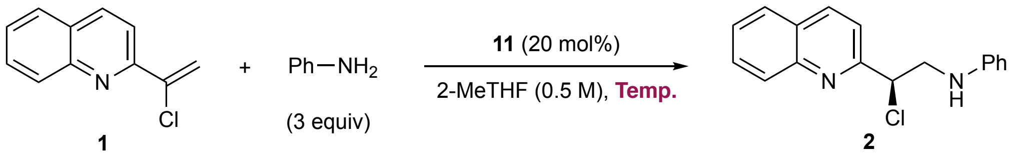

1 2

Entry	*Temp. (°C)*	*Time (h)*	*Conv. (%)*	e.r.
1	rt	15	93	90:10
2	0	15	97	95:5
3	−10	15	95	96:4
4	−20	15	96	96:4
5	−50	24	95	96:4
6	−70	39	96	96:4
7	−78		Trace Reaction Observed	

The conversion was determined by ^{1}H NMR analysis with an internal standard, and e.r. was determined by HPLC analysis.

Table S4: Nucleophile Stoichometry Screening

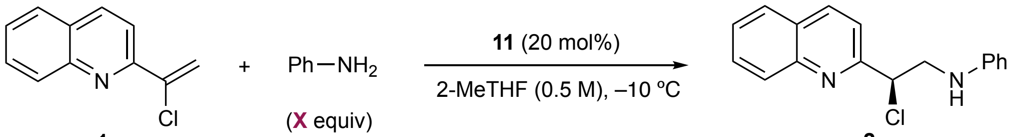

1 2

Entry	*Aniline Equiv.*	*Time (h)*	*Conv. (%)*	e.r.
1	5.0	15	94	95:5
2	3.0	15	95	96:4
3	2.0	15	97	96:4
4	1.5	15	97	96:4
5	1.0	15	94	96:4

The conversion was determined by ^{1}H NMR analysis with an internal standard, and e.r. was determined by HPLC analysis with an internal standard.

Table S5: Catalyst Stoichometry Screening

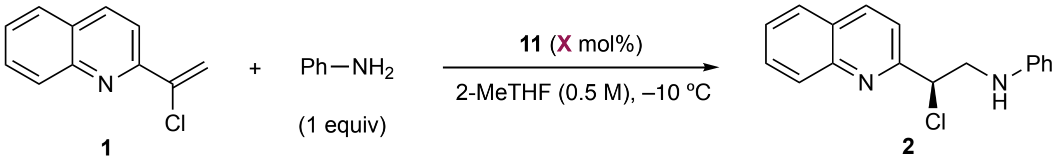

1 2

Entry	*Cat. Loading. (mol%)*	*Time (h)*	*Conv. (%)*	e.r.
1	1	15	92	92:8
2	2	15	94	94:6
3	5	15	97	95:5
4	10	15	94	95:5
5	20	15	94	96:4
6	5 (−20 °C)	15	94	96:4

The conversion was determined by ^{1}H NMR analysis with an internal standard, and e.r. was determined by HPLC analysis.

Table S6: Reaction Concentration Screening

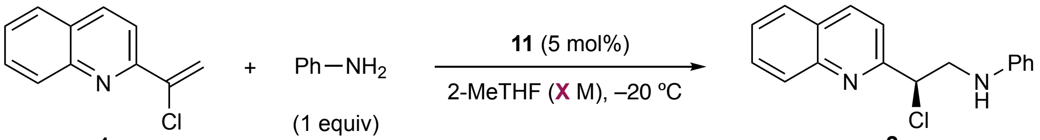

1 2

Entry	Concentration (M)	Time (h)	Conv. (%)	e.r.
1	0.50	15	94	96:4
2	0.25	15	93	96:4
3	0.10	24	90	95:5

The conversion was determined by ^{1}H NMR analysis with an internal standard, and e.r. was determined by HPLC analysis.

Table S7: Aziridination Optimization

Optimisation study of the one-pot aza-Michael-ring closure reaction catalyzed using a chiral acid was carried out using general procedure B with modification of the appropriate reaction parameters.

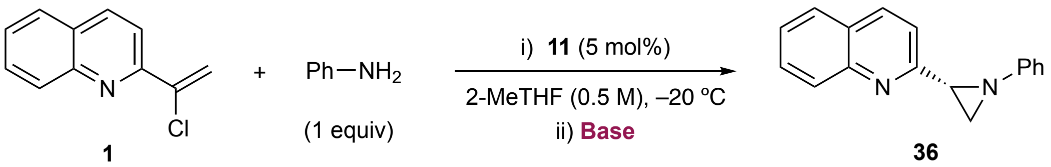

Entry	Base	Equivalents	Time	Conversion (%)	e.r.
1	NaH	2.0	5	28	91:9
2	KO^{t}Bu	2.0	5	81	73:27
3	K_2CO_3	2.0	5	0	--
4	K_3PO_4	2.0	5	0	--
5	TEA	2.0	5	0	--
6	DBU	2.0	5	0	--
7	DIPEA	2.0	5	0	--
8	Pyridine	2.0	5	0	--
9	NaH	1.05	5	trace	--
10	KO^{t}Bu	1.05	5	60	74:26
11	^{n}BuLi	1.05	5	22	95:5
12	LDA	1.05	5	43	95:5
13	LiHMDS	1.05	5	83	95:5
14	LiO^{t}Bu	1.05	5	0	--
15	LiHMDS	1.05	0.5	79	95:5
16	LiHMDS	1.2	0.5	95	95:5

The conversion was determined by ^{1}H NMR analysis with an internal standard, and e.r. was determined by HPLC analysis.

4. Characterization Data for Compounds

Catalysts **5**, **3**, **11**, **S1**, **S3**, **S4**, **S5**, **S6**, **S10**, **S11**, **S12**, and **S13** are commercially available.

The following catalysts were prepared according to literature procedures: **6**,[5] **7**,[6] **8**,[7] **9**,[6] **10**,[8] **S2**,[9] **S7**,[10] and **S9**.[11]

(11c*S*)-2,9,14-Tri(adamantan-1-yl)-4-hydroxydinaphtho[2,1-*d*:1',2'-*f*][1,3,2]dioxaphosphepine 4-oxide (S8)

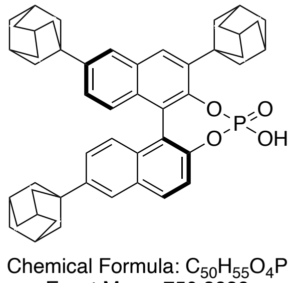

Chemical Formula: $C_{50}H_{55}O_4P$
Exact Mass: 750.3838
Molecular Weight: 750.9598

(*S*)-3,6,6'-tri(adamantan-1-yl)-[1,1'-binaphthalene]-2,2'-diol was prepared according to literature procedure.[12]

To a flame-dried 50 mL Schlenk-tube, equipped with a stir-bar, was charged with (*S*)-3,6,6'-tri(adamantan-1-yl)-[1,1'-binaphthalene]-2,2'-diol (555 mg, 1 mmol, 1.0 equiv.) and dissolved in pyridine (3 mL) and phosphorous oxychloride (187 mL, 2 mmol, 2.0 equiv.) was added. After stirring for 16 h at room temperature, H_2O (3 mL) was added. The reaction mixture was stirred for additional 6 h at room temperature followed by the addition of DCM (10 mL) and washed with 2 M aq. HCl (3 x 10 mL). The organic phase was washed with brine, dried over Na_2SO_4, and concentrated *in vacuo* to give a grey residue. The crude product was purified using flash column chromatography (silica, MeOH:DCM 20:80) to afford the desired product as a white solid (487 mg, 66%).

ν_{max} (film): 2901, 2847, 1449, 1211, 1020 cm^{-1}.

^{1}H NMR (300 MHz, $CDCl_3$): δ 7.92 (s, 1H), 7.87 (d, *J* = 8.9 Hz, 1H), 7.78 (d, *J* = 7.6 Hz, 1H), 7.46 (d, *J* = 8.9 Hz, 1H), 7.37 – 7.28 (m, 2H), 7.15 (t, *J* = 8.1 Hz, 2H), 2.25 (s, 7H), 2.17 – 2.05 (m, 10H), 2.03 – 1.92 (m, 12H), 1.87 – 1.69 (m, 18H). ^{1}H resonance for phosphoric acid not observed.

^{13}C NMR (126 MHz, $CDCl_3$): δ 148.4, 148.3, 146.5 (d, $^2J_{CP}$ = 6.9 Hz), 145.9 (d, $^2J_{CP}$ = 7.7 Hz), 145.8, 140.8, 131.8, 131.1, 130.9, 130.8, 129.4, 128.2, 126.8, 126.5, 124.7, 124.2, 123.3, 123.1, 121.9, 121.8, 120.1, 43.1, 43.0, 41.8, 37.8, 36.8, 36.7, 36.20, 36.15, 29.0, 28.9, 28.8.

^{31}P NMR (121 MHz, $CDCl_3$): δ 2.65.

HRMS (ESI): exact mass calculated for $[M-H]^-$ ($C_{50}H_{54}O_4P$) requires *m/z* 749.3760, found *m/z* 749.3745.

$[\alpha]_D^{23}$= +4.3° (*c* = 1.0, $CHCl_3$).

Cycloheptylzinc chloride (S14)

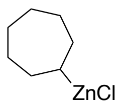

Chemical Formula: $C_7H_{13}ClZn$
Exact Mass: 195.9997
Molecular Weight: 198.0110

To a flame-dried three-necked flask, equipped with a stir-bar and a reflux condenser, was added magnesium turnings (668 mg, 27.5 mmol, 1.1 equiv.), THF (25.0 mL), and two crystals of iodine was added under an argon atmosphere. The mixture was then sonicated until the colour disappeared. The mixture was stirred while bromocycloheptane (3.43 mL, 25.0 mmol, 1 equiv.) was added dropwise *via*

syringe at a pace sufficient to avoid an uncontrollable exotherm and maintain a steady reflux. The reaction mixture was then heated to reflux for 16 h. The mixture was allowed to cool to room temperature and then oven-dried zinc chloride (4.09 g, 30.0 mmol, 1.2 equiv.) in THF (30 mL) was added *via* syringe and the mixture was allowed to stir for 1 h at room temperature. The white precipitate formed was allowed to settle before use of the solution. I_2 titration was carried out and gave a concentration of 0.35 – 0.39 M.

1,3,5-Tricycloheptylbenzene (S15)

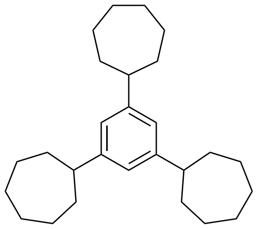

Chemical Formula: $C_{27}H_{42}$
Exact Mass: 366.3287
Molecular Weight: 366.6330

A flame-dried Schlenk-flask, equipped with a stir-bar, was charged with 1,3,5-tribromobenzene (3.63 g, 11.5 mmol, 1.0 equiv.), $Pd(OAc)_2$ (25.9 mg, 0.115 mmol, 0.01 equiv.) and S-Phos (94.7 mg, 0.231 mmol, 0.02 equiv.) and THF (69.2 mL, 0.167 M) under an argon atmosphere. The resulting brown mixture was allowed to stir at room temperature before addition of the cycloheptylzinc chloride **S14** (94.6 mL, 36.9 mmol, 0.39 M, 3.2 equiv.) dropwise *via* syringe. The resulting brown heterogenous mixture was allowed to stir for 3.5 h. The dark brown reaction mixture was quenched by addition of sat. aq. NH_4Cl and extracted with Et_2O (3x). The organics were combined, dried over Na_2SO_4, filtered, and had the solvent concentrated *in vacuo* to give a brown residue. The crude product was purified using flash column chromatography (Silica, hexane) to afford a white solid, upon standing. The white solid was washed with MeOH (400 mL) and dried to give the desired product as a white solid (3.31 g, 78 %).

ν_{max} (film): 2916, 2849, 1597, 1447 cm^{-1}.

^{1}H NMR (500 MHz, $CDCl_3$): δ 6.80 (s, 3H), 2.65 – 2.55 (m, 3H), 1.95 – 1.87 (m, 6H), 1.85 – 1.46 (m, 30H).

^{13}C NMR (126 MHz, $CDCl_3$): δ 149.7, 122.3, 47.2, 36.9, 27.9, 27.3.

HRMS (ESI): exact mass calculated for $[M+Na]^+$ ($C_{27}H_{42}Na$) requires *m/z* 389.3177, found *m/z* 389.3179.

2-Bromo-1,3,5-tricycloheptylbenzene (S16)

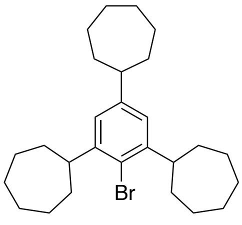

Chemical Formula: $C_{27}H_{41}Br$
Exact Mass: 444.2392
Molecular Weight: 445.5290

In a round-bottom flask, equipped with a stir-bar, 1,3,5-tricycloheptylbenzene **S15** (2.76 g, 7.53 mmol, 1.0 equiv.) was dissolved in $CHCl_3$ (4.89 mL, 1.54 M) under a nitrogen atmosphere and cooled to 0 °C. To the stirred solution, bromine (386 mL, 7.53 mmol, 1.0 equiv.) was added slowly over 15 min *via* syringe. The solution was warmed to 40 °C, where it was stirred for 3 h. The reaction mixture was allowed to cool to room temperature before diluting with DCM, and the resulting solution was washed with water. The aqueous phase was extracted with further DCM (2x) and the organic phases were

combined and washed successively with 2 M aq. NaOH solution (until the organics were colourless), brine and then dried over Na_2SO_4, filtered, and concentrated to afford a yellow oil. The crude product was purified using flash column chromatography (silica, hexane) to afford a white solid. This white solid was recrystallised in boiling EtOH to give white flakes (2.17 g, 65 %).

ν_{max} (film): 2916, 2851, 1456, 1443, 1015 cm^{-1}.

^{1}H NMR (400 MHz, CDCl$_3$): δ 6.87 (s, 2H), 3.28 – 3.16 (m, 2H), 2.57 (m, 1H), 1.99 – 1.47 (m, 36H).

^{13}C NMR (126 MHz, CDCl$_3$): δ 148.8, 148.3, 123.0, 122.6, 46.9, 45.6, 36.8, 36.0, 27.8, 27.8, 27.5, 27.2.

HRMS (ESI): exact mass calculated for [M]$^+$ ($C_{27}H_{41}Br$) requires *m/z* 444.2386, found *m/z* 444.2382, 446.2358.

(2,4,6-Tricycloheptylphenyl)magnesium bromide (S17)

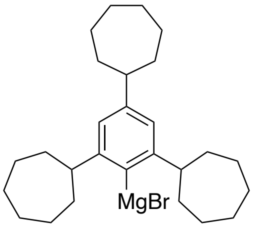

Chemical Formula: $C_{27}H_{41}BrMg$
Exact Mass: 468.2242
Molecular Weight: 469.8340

To a two-necked flask, equipped with a stir-bar and reflux condenser, was added magnesium turnings (44.4 mg, 1.83 mmol, 1.10 equiv.). The flask was heated under vacuum to 200 °C and the magnesium was ground, to a black powder, overnight by using the stir-bar at a moderate rpm. The flask was then purged with argon, the heating was halted, and iodine (1 crystal) was immediately added, which was allowed to sublime. Upon reaching room temperature, THF (0.83 mL, 2 M) was added followed by dropwise addition of 2-bromo-1,3,5-tricycloheptylbenzene **S16** (740 mg, 1.66 mmol, 1.0 equiv.) dissolved in THF (4.15 mL, 0.4 M) *via* syringe. The reaction mixture was then heated to reflux (ensure vigorous boiling) and 1,2-dibromoethane (1 drop) was added *via* syringe. The mixture was allowed to stir for a further 16 h. The reaction mixture was then allowed to cool to room temperature.

Quantitative Grignard reagent formation was assumed.

(*S*)-2,2'-Bis(methoxymethoxy)-3,3'-bis(2,4,6-tricycloheptylphenyl)-1,1'-binaphthalene (S18)

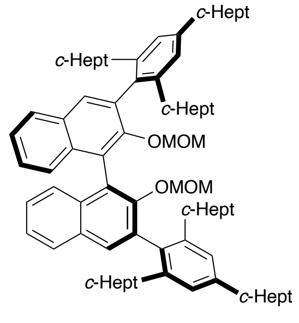

Chemical Formula: $C_{78}H_{102}O_4$
Exact Mass: 1102.7778
Molecular Weight: 1103.6700

(*S*)-3,3'-diiodo-2,2'-bis(methoxymethoxy)-1,1'-binaphthalene was prepared according to literature procedure.[13]

To a flame-dried two-necked flask, equipped with a stir-bar and reflux condenser, was added $NiCl_2(PPh_3)_2$ (16.4 mg, 0.025 mmol, 10 mol%), (*S*)-3,3'-diiodo-2,2'-bis(methoxymethoxy)-1,1'-binaphthalene (157 mg, 0.250 mmol, 1 equiv.) and the flask was evacuated and purged with argon

(3x). THF (0.88 mL, 0.28 M) was added, and the mixture was stirred at room temperature prior to the dropwise addition of the requisite (2,4,6-tricycloheptylphenyl)magnesium bromide **S17** in THF (1 mmol, 4.0 equiv.). Upon addition of the Grignard reagent, the reaction mixture turned red and was heated to reflux for 16 h. The reaction was allowed to cool to room temperature before quenching with sat. aq. NH_4Cl and extracted with Et_2O (3x). The organic phases were combined, dried over Na_2SO_4, filtered, and had the solvent concentrated *in vacuo* to give a yellow residue. The crude product was purified using flash column chromatography (silica, DCM:hexane 10:90 to 15:85) to afford the desired product as a white solid (147 mg, 53 %).

ν_{max} (film): 2914, 2851, 993, 746 cm^{-1}.

^{1}H NMR (500 MHz, $CDCl_3$): δ 7.83 (d, *J* = 8.4 Hz, 2H), 7.71 (s, 2H), 7.40 (ddd, *J* = 8.1, 5.8, 2.2 Hz, 2H), 7.29 – 7.23 (m, 4H), 6.98 – 6.93 (m, 4H), 4.39 (d, *J* = 5.1 Hz, 2H), 4.29 (d, *J* = 5.1 Hz, 2H), 2.69 – 2.61 (m, 2H), 2.55 – 2.45 (m, 4H), 2.28 (s, 6H), 1.96 – 1.58 (m, 40H), 1.51 – 1.06 (m, 32H).

^{13}C NMR (126 MHz, $CDCl_3$): δ 151.8, 149.5, 148.5, 148.3, 134.6, 133.7, 131.7, 131.0, 130.4, 127.7, 125.9, 125.8, 124.9, 121.4, 121.3, 97.1, 77.3, 55.1, 47.3, 43.7, 43.1, 38.4, 38.1, 37.0, 35.3, 35.2, 28.2, 28.1, 27.9, 27.7, 27.5, 27.3, 27.1, 27.0.

HRMS (ESI): exact mass calculated for $[M]^+$ ($C_{78}H_{102}O_4$) requires *m/z* 1102.7778, found *m/z* 1102.7760.

$[\alpha]_D^{23}$ = −51° (*c* = 0.5, $CHCl_3$).

(*S*)-3,3'-Bis(2,4,6-tricycloheptylphenyl)-[1,1'-binaphthalene]-2,2'-diol (S19)

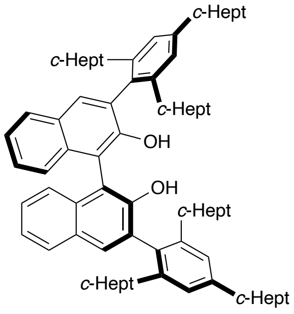

Chemical Formula: $C_{74}H_{94}O_2$
Exact Mass: 1014.7254
Molecular Weight: 1015.5640

In a round-bottom flask, equipped with a stir-bar, (*S*)-2,2'-Bis(methoxymethoxy)-3,3'-bis(2,4,6-tricycloheptylphenyl)-1,1'-binaphthalene **S18** (197 mg, 0.178 mmol, 1.0 equiv.) was dissolved in 1,4-dioxane (6.70 mL 0.027 M) to which conc. aq. hydrochloric acid (669 µL, 45.0 equiv.) was added. The mixture was allowed to stir at 75 °C for 16 h. The reaction was allowed to cool to room temperature before concentrating *in vacuo* to give a clear residue which was then quenched by addition of saturated aqueous $NaHCO_3$ and extracted with DCM (3x). The organic phases were combined, dried over Na_2SO_4, filtered, and the solvent concentrated *in vacuo* to give a white residue. The crude product was purified using flash column chromatography (silica, DCM:hexane 30:70) to afford the desired product as a white solid (165 mg, 91 %).

ν_{max} (film): 3522, 2917, 2851, 1601, 1450, 1423, 1256, 745 cm^{-1}.

^{1}H NMR (500 MHz, $CDCl_3$): δ 7.86 (d, J = 8.2 Hz, 2H), 7.77 – 7.66 (m, 2H), 7.40 – 7.32 (m, 2H), 7.31 – 7.27 (m, 4H), 7.05 – 6.99 (m, 4H), 4.88 – 4.81 (m, 2H), 2.73 – 2.65 (m, 2H), 2.61 – 2.52 (m, 2H), 2.49 – 2.41 (m, 2H), 2.03 – 1.55 (m, 54H), 1.43 – 1.23 (m, 14H), 1.16 – 1.01 (m, 4H).

^{13}C NMR (126 MHz, $CDCl_3$): δ 150.6, 150.2, 148.9, 148.8, 133.3, 130.6, 129.4, 129.0, 128.9, 128.2, 126.3, 124.4, 123.5, 122.0, 121.9, 113.0, 47.3, 43.5, 43.4, 37.0, 36.8, 36.4, 27.9, 27.7, 27.64, 27.59, 27.5, 27.4, 27.3, 27.2.

HRMS (ESI): exact mass calculated for $[M]^+$ ($C_{74}H_{94}O_2$) requires *m/z* 1014.7248, found *m/z* 1014.7241.

$[\alpha]_D^{23}$ = −101° (*c* = 0.5, $CHCl_3$).

(*S*)-4-hydroxy-2,6-bis(2,4,6-tricycloheptylphenyl)dinaphtho[2,1-*d*:1',2'-*f*][1,3,2]dioxaphosphepine 4-oxide (12)

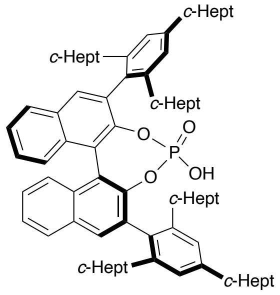

Chemical Formula: $C_{74}H_{93}O_4P$
Exact Mass: 1076.6811
Molecular Weight: 1077.5278

In a round-bottom flask, equipped with a stir-bar and reflux condenser, (*S*)-3,3'-bis(2,4,6-tricycloheptylphenyl)-[1,1'-binaphthalene]-2,2'-diol **S19** (125 mg, 0.123 mmol, 1.0 equiv.) was dissolved in pyridine (880 µL, 0.14 M) and phosphoroxychloride (23 µL, 2.0 equiv.) was added. The resulting mixture was then heated to reflux for 16 h. The reaction mixture was allowed to cool to room temperature and then H_2O (22 µL, 10.0 equiv.) was added, at which point a precipitate started to from. The reaction mixture was allowed to reflux for 4 h. The reaction mixture was cooled to room temperature and diluted with DCM and washed with 2 M aq. HCl (3x). The organic phases were combined, dried over Na_2SO_4, filtered, and had the solvent concentrated *in vacuo* to give a white residue. The crude product was purified using flash column chromatography (silica, MeOH:DCM 2.5:97.5) to afford a white solid. The white solid was then stirred in 2M aq. HCl (5 mL) for 1 h and extracted with DCM (3x). The organics were combined, dried over Na_2SO_4, filtered, and had the solvent concentrated *in vacuo* to give the desired product as a white solid (97 mg, 73 %).

ν_{max} (film): 2914, 2849, 1458, 1443, 1273, 1207, 1016, 897, 748 cm^{-1}.

^{1}H NMR (500 MHz, $CDCl_3$): δ 7.86 (d, *J* = 8.2 Hz, 2H), 7.73 (s, 2H), 7.47 – 7.41 (m, 2H), 7.29 – 7.22 (m, 4H), 6.87 (d, *J* = 24.9 Hz, 4H), 2.63 – 2.54 (m, 2H), 2.49 – 2.41 (m, 2H), 2.39 – 2.30 (m, 2H), 2.03 – 1.87 (m, 6H), 1.83 – 0.79 (m, 66H). ^{1}H resonance for the phosphoric acid P(O)(O**H**) was not observed.

^{13}C NMR (126 MHz, $CDCl_3$): δ 149.5, 148.7, 148.5, 146.3 (d, $^2J_{CP}$ = 9.1 Hz), 132.6, 132.3, 132.1, 131.1, 130.0, 128.1, 126.9, 126.2, 125.6, 121.9, 121.9, 121.2, 47.5, 43.2, 42.9, 39.2, 37.6, 37.2, 36.7, 35.4, 35.1, 28.1, 28.0, 27.9, 27.9, 27.9, 27.7, 27.6, 27.5, 27.1.

^{31}P NMR (162 MHz, $CDCl_3$): δ 1.12.

HRMS (ESI): exact mass calculated for $[M-H]^-$ ($C_{74}H_{92}O_4$) requires *m/z* 1075.6739, found *m/z* 1075.6687.

$[\alpha]_D^{23}$= +4.3° (*c* = 1.0, $CHCl_3$).

2-Vinylquinoline (S20)

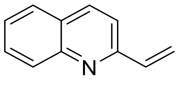

Chemical Formula: $C_{11}H_9N$
Exact Mass: 155.07
Molecular Weight: 155.20

Prepared according to adapted literature procedure.[14]

A 500 mL oven-dried round bottom flask, equipped with a stir-bar and reflux condenser, was charged with quinoline-2-carboxaldehyde (15.7 g, 100 mmol, 1.0 equiv.), potassium carbonate (22.1 g, 160 mmol, 1.6 equiv.), and methyltriphenylphosphonium bromide (42.9 g, 120 mmol, 1.2 equiv.) in anhydrous THF (150 mL), and heated at reflux for 16 h. The reaction mixture was cooled, filtered and

concentrated *in vacuo*. The crude product was purified using flash chromatography (silica, EtOAc:hexane 15:85) to give 2-vinylquinoline as a yellow liquid (14.5 g, 93%).

^{1}H NMR (500 MHz, CDCl$_3$): δ 8.12 (d, *J* = 8.6 Hz, 1H), 8.08 (d, *J* = 8.5 Hz, 1H), 7.78 (d, *J* = 8.1 Hz, 1H), 7.70 (ddd, *J* = 8.4, 6.9, 1.5 Hz, 1H), 7.61 (d, *J* = 8.6 Hz, 1H), 7.50 (ddd, *J* = 8.1, 6.9, 1.2 Hz, 1H), 7.05 (dd, *J* = 17.7, 10.9 Hz, 1H), 6.29 (dd, *J* = 17.7, 0.8 Hz, 1H), 5.68 (dd, *J* = 10.9, 0.8 Hz, 1H).

^{13}C NMR (126 MHz, CDCl$_3$): δ 156.2, 148.1, 138.1, 136.5, 129.8, 129.5, 127.6, 126.4, 120.0, 118.5.

Spectroscopic data in agreement with literature values.[14]

6-Fluoro-2-vinylquinoline (S21)

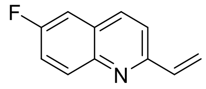

Prepared according to General Procedure C using 2-bromo-6-fluoroquinoline (968 mg, 5.00 mmol, 1.0 equiv.), vinylboronic acid pinacol ester (847 mg, 5.50 mmol, 1.1 equiv.), Pd(OAc)$_2$ (44.9 mg, 0.20 mmol, 4 mol%), S-Phos (164 mg, 0.40 mmol, 8 mol%), K_3PO_4 (3.18 g, 15.0 mmol, 3.0 equiv.), water (450 µL, 25.0 mmol, 5.0 equiv.), and 1,4-dioxane (20 mL) at 80 °C for 3 h. The reaction mixture was subjected to the purification outlined in General Procedure C (silica, EtOAc:hexane, 10:90) to afford the desired product as a yellow liquid (745 mg, 88%).

^{1}H NMR (500 MHz, CDCl$_3$): δ 7.98 (dd, *J* = 9.2, 5.3 Hz, 1H), 7.85 (d, *J* = 8.6 Hz, 1H), 7.44 (dd, *J* = 8.6, 1.0 Hz, 1H), 7.37 (ddd, *J* = 9.3, 8.3, 2.9 Hz, 1H), 7.24 (dd, *J* = 8.8, 2.9 Hz, 1H), 6.94 (dd, *J* = 17.7, 10.9 Hz, 1H), 6.19 (dd, *J* = 17.7, 0.9 Hz, 1H), 5.59 (dd, *J* = 10.9, 0.9 Hz, 1H).

^{13}C NMR (126 MHz, CDCl$_3$): δ 160.2 (d, J_{CF} = 248.1 Hz), 155.4 (d, J_{CF} = 2.8 Hz), 145.0, 137.6, 135.5 (d, J_{CF} = 5.4 Hz), 131.7 (d, J_{CF} = 9.1 Hz), 127.9 (d, J_{CF} = 10.0 Hz), 119.7, 119.5, 119.0, 110.5 (d, J_{CF} = 21.7 Hz).

^{19}F NMR (470 MHz, CDCl$_3$): δ −113.60.

6-Methoxy-2-vinylquinoline (S22)

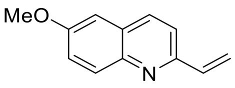

Prepared according to General Procedure C using 2-chloro-6-methoxyquinoline (968 mg, 5.00 mmol, 1.0 equiv.), vinylboronic acid pinacol ester (847 mg, 5.50 mmol, 1.1 equiv.), Pd(OAc)$_2$ (44.9 mg, 0.20 mmol, 4 mol%), S-Phos (164 mg, 0.40 mmol, 8 mol%), K_3PO_4 (3.18 g, 15.0 mmol, 3.0 equiv.), water (450 µL, 25.0 mmol, 5.0 equiv.), and 1,4-dioxane (20 mL) at 80 °C for 3 h. The reaction mixture was subjected to the purification outlined in General Procedure C (silica, EtOAc:hexane, 10:90) to afford the desired product as a yellow liquid (745 mg, 88%).

^{1}H NMR (500 MHz, CDCl$_3$): 7.98 (d, *J* = 8.6 Hz, 1H), 7.95 (d, *J* = 9.2 Hz, 1H), 7.55 (d, *J* = 8.5 Hz, 1H), 7.34 (dt, *J* = 9.2, 2.1 Hz, 1H), 7.04 – 6.95 (m, 2H), 6.20 (dd, *J* = 17.7, 1.0 Hz, 1H), 5.59 (dd, *J* = 11.0, 0.9 Hz, 1H), 3.91 (s, 3H).

^{13}C NMR (126 MHz, CDCl$_3$): 157.8, 154.0, 144.1, 138.0, 135.2, 130.9, 128.6, 122.4, 118.8, 118.7, 105.3, 55.6.

Spectroscopic data in agreement with literature values.[15]

2-Chloro-1-(1-methyl-1*H*-benzo[*d*]imidazol-2-yl)ethan-1-one (S23)

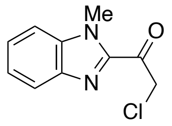

Chemical Formula: $C_{10}H_9ClN_2O$
Exact Mass: 208.0403
Molecular Weight: 208.6450

N-Methylbenzimidazole was prepared according to literature procedure.[16]

Prepared according to General Procedure E using *N*-methylbenzimidazole (925 mg, 7.00 mmol, 1.0 equiv.), THF (17.5 mL), *iso*propyl magnesium chloride lithium chloride complex (7 mL, 1.0 M in THF, 7.00 mmol, 1.0 equiv.), 2-chloro-*N*-methoxy-*N*-methylacetamide (963 mg, 7.0 mmoL) and THF (17.5 mL). The reaction mixture was subjected to the purification outlined in General Procedure E (silica, EtOAc:hexane, 20:80) to afford the desired product as a white solid (304 mg, 21%).

ν_{max} (film): 3053, 2941, 1699, 1481, 737 cm^{-1}.

^1H NMR (500 MHz, $CDCl_3$): δ 7.88 (d, *J* = 8.3 Hz, 1H), 7.52 – 7.46 (m, 2H), 7.44 – 7.38 (m, 1H), 5.15 (s, 2H), 4.18 (s, 3H).

^{13}C NMR (101 MHz, $CDCl_3$): δ 185.7, 143.8, 141.6, 136.9, 126.6, 124.2, 122.0, 110.7, 47.4, 32.2.

HRMS (ESI): exact mass calculated for $[M+H]^+$ ($C_{10}H_{10}ClN_2O$) requires *m/z* 209.0476, found *m/z* 209.0473.

1-Methyl-2-(1,1,2-trichloroethyl)-1*H*-benzo[*d*]imidazole (S24)

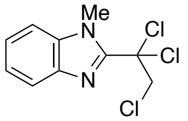

Chemical Formula: $C_{10}H_9Cl_3N_2$
Exact Mass: 261.9831
Molecular Weight: 263.5460

Prepared according to General Procedure F using phosphorous pentachloride (541 mg, 2.60 mmol, 1.3 equiv.), benzene (423 mL) and aryl ketone **S23** (417 mg, 2.00 mmol, 1.0 equiv.). The crude mixture was taken on without further purification as a yellow gum.

2-Chloro-1-(5-phenylthiazol-2-yl)ethan-1-one (S25)

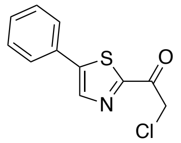

Chemical Formula: $C_{11}H_8ClNOS$
Exact Mass: 237.0015
Molecular Weight: 237.7010

5-Phenylthiazole was prepared according to literature procedure.[17]

Prepared according to General Procedure E using 5-phenylthiazole (806 mg, 5.00 mmol, 1.0 equiv.), THF (25 mL), *iso*propyl magnesium chloride lithium chloride complex (5 mL, 1.0 M in THF, 5.00 mmol, 1.0 equiv.), 2-chloro-*N*-methoxy-*N*-methylacetamide (688 mg, 5.00 mmoL, 1.0 equiv.) and THF (12.5 mL). The reaction mixture was subjected to the purification outlined in General Procedure E (silica, EtOAc:hexane, 5:95) to afford the desired product as a yellow solid (820 mg, 69 %).

ν_{max} (film): 2994, 1703, 1406, 1393, 760 cm^{-1}.

^1H NMR (500 MHz, $CDCl_3$): δ 8.16 (s, 1H), 7.69 – 7.60 (m, 2H), 7.52 – 7.40 (m, 3H), 4.97 (s, 2H).

^{13}C NMR (126 MHz, $CDCl_3$): δ 184.6, 162.0, 148.2, 140.5, 130.0, 130.0, 129.5, 127.3, 45.8.

HRMS (ESI): exact mass calculated for $[M+H]^+$ ($C_{11}H_9ClNOS$) requires *m/z* 238.0093, found *m/z* 238.0084.

5-Phenyl-2-(1,1,2-trichloroethyl)thiazole (S26)

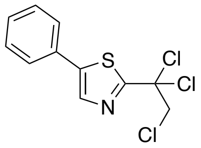

Chemical Formula: $C_{11}H_8Cl_3NS$
Exact Mass: 290.9443
Molecular Weight: 292.6020

Prepared according to General Procedure F using phosphorous pentachloride (942 mg, 4.52 mmol, 1.3 equiv.), benzene (740 μL) and aryl ketone **S25** (827 mg, 3.48 mmol, 1.0 equiv.). The reaction mixture was subjected to the purification outlined in General Procedure F (silica, EtOAc:hexane, 5:95) to afford the desired product as a yellow oil (723 mg, 71%).

ν_{max} (film): 3024, 2965, 1410, 754, 729, 687 cm^{-1}.

^{1}H NMR (500 MHz, $CDCl_3$): δ 7.97 (s, 1H), 7.59 – 7.56 (m, 2H), 7.46 – 7.36 (m, 3H), 4.58 (s, 2H).

^{13}C NMR (126 MHz, $CDCl_3$): δ 166.9, 143.2, 138.8, 130.3, 129.3, 129.1, 127.0, 83.6, 54.0.

HRMS (ESI): exact mass calculated for $[M+H]^+$ ($C_{11}H_9Cl_3NS$) requires *m/z* 291.9521, found *m/z* 291.9511.

2-Vinylquinoxaline (S27)

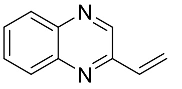

Chemical Formula: $C_{10}H_8N_2$
Exact Mass: 156.0687
Molecular Weight: 156.1880

Prepared according to General Procedure C using 2-chloroquinoxaline (823 mg, 5.00 mmol, 1.0 equiv.), vinylboronic acid pinacol ester (847 mg, 5.50 mmol, 1.1 equiv.), $Pd(OAc)_2$ (44.9 mg, 0.20 mmol, 4 mol%), S-Phos (164 mg, 0.40 mmol, 8 mol%), K_3PO_4 (3.18 g, 15.0 mmol, 3.0 equiv.), water (450 µL, 25.0 mmol, 5.0 equiv.), and 1,4-dioxane (20 mL) at 80 °C for 3 h. The reaction mixture was subjected to the purification outlined in General Procedure C (silica, EtOAc:hexane, 10:90) to afford the desired product as a yellow liquid (527 mg, 67%).

^{1}H NMR (500 MHz, $CDCl_3$): 9.01 (s, 1H), 8.17 – 7.97 (m, 2H), 7.85 – 7.59 (m, 2H), 7.05 (dd, *J* = 17.7, 11.0 Hz, 1H), 6.49 (d, *J* = 17.7 Hz, 1H), 5.80 (d, *J* = 11.1 Hz, 1H).

^{13}C NMR (126 MHz, $CDCl_3$): 150.6, 143.7, 142.3, 141.9, 134.9, 130.4, 129.7, 129.5, 129.3, 122.3.

Spectroscopic data in agreement with literature values. [18]

2-Vinylquinazoline (S28)

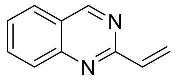

Chemical Formula: $C_{10}H_8N_2$
Exact Mass: 156.07
Molecular Weight: 156.19

Prepared according to General Procedure C using 2-chloroquinazoline (823 mg, 5.00 mmol, 1.0 equiv.), vinylboronic acid pinacol ester (847 mg, 5.50 mmol, 1.1 equiv.), $Pd(OAc)_2$ (44.9 mg, 0.20 mmol, 4 mol%), S-Phos (164 mg, 0.40 mmol, 8 mol%), K_3PO_4 (3.18 g, 15.0 mmol, 3.0 equiv.), water (450 µL, 25.0

mmol, 5.0 equiv.), and 1,4-dioxane (20 mL) at 80 °C for 3 h. The reaction mixture was subjected to the purification outlined in General Procedure C (silica, EtOAc:hexane, 10:90) to afford the desired product as a yellow liquid (614 mg, 79%).

^{1}H NMR (400 MHz, CDCl$_3$): 9.37 (s, 1H), 8.01 (d, *J* = 8.2 Hz, 1H), 7.92 – 7.86 (m, 2H), 7.63 – 7.58 (m, 1H), 7.05 (dd, *J* = 17.3, 10.5 Hz, 1H), 6.79 (d, *J* = 17.3 Hz, 1H), 5.85 (d, *J* = 10.5 Hz, 1H).

^{13}C NMR (126 MHz, CDCl$_3$): 160.9, 160.4, 150.5, 137.1, 134.3, 128.4, 127.5, 127.3, 124.4, 123.7.

Spectroscopic data in agreement with literature values. [19]

Methyl 6-vinylnicotinate (S29)

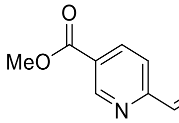

Chemical Formula: $C_9H_9NO_2$
Exact Mass: 163.06
Molecular Weight: 163.18

Prepared according to General Procedure C using methyl 6-bromonicotinate (2.16 mg, 10.0 mmol, 1.0 equiv.), vinylboronic acid pinacol ester (1.69 mg, 11.0 mmol, 1.1 equiv.), Pd(OAc)$_2$ (89.8 mg, 0.40 mmol, 4 mol%), S-Phos (328 mg, 0.80 mmol, 8 mol%), K_3PO_4 (6.37 g, 15.0 mmol, 3.0 equiv.), water (900 µL, 25.0 mmol, 5.0 equiv.), and 1,4-dioxane (40 mL) at 80 °C for 3 h. The reaction mixture was subjected to the purification outlined in General Procedure C (silica, EtOAc:hexane, 10:90) to afford the desired product as a yellow liquid (1.30 g, 80%).

^{1}H NMR (MHz, CDCl$_3$): 8.87 (d, *J* = 2.0 Hz, 1H), 7.91 (dd, *J* = 8.2, 2.2 Hz, 1H), 7.09 (d, *J* = 8.2 Hz, 1H), 6.57 (dd, *J* = 17.4, 10.8 Hz, 1H), 6.09 (dd, *J* = 17.4, 1.1 Hz, 1H), 5.33 (dd, *J* = 10.8, 1.1 Hz, 1H), 3.65 (s, 3H).

^{13}C NMR (MHz, CDCl$_3$): 165.1, 158.7, 150.3, 137.1, 135.8, 124.0, 120.5, 120.3, 51.8.

Spectroscopic data in agreement with literature values. [20]

6-Vinylnicotinonitrile (S30)

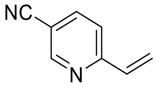

Chemical Formula: $C_8H_6N_2$
Exact Mass: 130.0531
Molecular Weight: 130.1500

Prepared according to General Procedure C using 2-bromo-5-cyanopyridine (915 mg, 5.00 mmol, 1.0 equiv.), vinylboronic acid pinacol ester (847 mg, 5.50 mmol, 1.1 equiv.), Pd(OAc)$_2$ (44.9 mg, 0.20 mmol, 4 mol%), S-Phos (164 mg, 0.40 mmol, 8 mol%), K_3PO_4 (3.18 g, 15.0 mmol, 3.0 equiv.), water (450 µL, 25.0 mmol, 5.0 equiv.), and 1,4-dioxane (20 mL) at 80 °C for 3 h. The reaction mixture was subjected to the purification outlined in General Procedure C (silica, EtOAc:hexane, 10:90) to afford the desired product as a yellow liquid (461 mg, 71%).

^{1}H NMR (400 MHz, CDCl$_3$): 8.83 (dd, *J* = 2.1, 0.7 Hz, 1H), 7.91 (dd, *J* = 8.2, 2.2 Hz, 1H), 7.42 (dd, *J* = 8.2, 0.8 Hz, 1H), 6.84 (dd, *J* = 17.4, 10.7 Hz, 1H), 6.39 (dd, *J* = 17.4, 1.0 Hz, 1H), 5.69 (dd, *J* = 10.7, 1.0 Hz, 1H).

^{13}C NMR (126 MHz, CDCl$_3$): 158.9, 152.4, 139.8, 135.6, 122.7, 121.1, 117.0, 108.1.

Spectroscopic data in agreement with literature values. [21]

1-(Benzo[*d*]thiazol-2-yl)-2-chloroethan-1-one (S31)

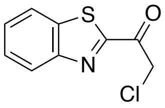

Chemical Formula: C_9H_6ClNOS
Exact Mass: 210.9859
Molecular Weight: 211.6630

Prepared according to General Procedure E using 2-bromothiazole (856 mg, 4.00 mmol, 1.0 equiv.), THF (10 mL), *iso*propyl magnesium chloride lithium chloride complex (4 mL, 1 M in THF, 4.00 mmol), 2-chloro-*N*-methoxy-*N*-methylacetamide (550 mg, 4.0 mmol, 1.0 equiv.) and THF (10.0 mL). The reaction mixture was subjected to the purification outlined in General Procedure E (silica, EtOAc:hexane, 5:95) to afford the desired product as a brown solid (846 mg, 100%).

ν_{max} (film): 3053, 2940, 1709, 762 cm^{-1}.

^{1}H NMR (500 MHz, $CDCl_3$): δ 8.23 – 8.16 (m, 1H), 8.06 – 7.97 (m, 1H), 7.66 – 7.54 (m, 2H), 5.10 (s, 2H).

^{13}C NMR (101 MHz, $CDCl_3$): δ 186.2, 163.2, 153.3, 137.3, 128.3, 127.4, 125.6, 122.5, 46.3.

HRMS (ESI): exact mass calculated for $[M+H]^+$ (C_9H_7ClNOS) requires *m/z* 211.9937, found *m/z* 211.9930.

2-(1,1,2-Trichloroethyl)benzo[*d*]thiazole (S32)

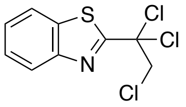

Chemical Formula: $C_9H_6Cl_3NS$
Exact Mass: 264.9287
Molecular Weight: 266.5640

Prepared according to General Procedure F using phosphorous pentachloride (1.08 g, 5.20 mmol, 1.3 equiv.), benzene (850 µL) and aryl ketone **S31** (846 mg, 4.00 mmol, 1.0 equiv.). The reaction mixture was subjected to the purification outlined in General Procedure F (silica, EtOAc:hexane, 5:95) to give the crude product as a yellow oil. The crude product was taken on without further purification.

6-Bromophenanthridine (S33)

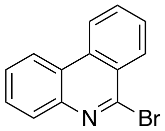

Chemical Formula: $C_{13}H_8BrN$
Exact Mass: 256.9840
Molecular Weight: 258.1180

2-Chlorophenanthridine was prepared according to literature procedure.[21]

In a 100 mL round-bottom flask, equipped with a stir-bar and a reflux condenser, a mixture of 2-chlorophenanthridine (1.82 g, 8.50 mmol, 1.0 equiv.), bromotrimethylsilane (2.24 mL, 17.0 mmol, 2.0 equiv.) and propionitrile (17.0 mL) was heated to reflux for 16 h. The reaction was allowed to cool and ice-cold 2M aq NaOH (40 mL) was added and extracted with Et_2O (3 x 30 mL). The organic phases were combined, dried over Na_2SO_4, filtered, and had the solvent concentrated *in vacuo* to give a tan residue. The crude product was purified using flash column chromatography (silica, EtOAc:hexane 5:95) to afford a crude white solid which was taken on without further purification.

6-Vinylphenanthridine (S34)

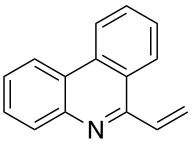

Chemical Formula: $C_{15}H_{11}N$
Exact Mass: 205.0891
Molecular Weight: 205.2600

To a flame-dried two-necked flask, equipped with a stir-bar and a reflux condenser, was charged with crude 6-bromophenanthridine **33** (1.10 g, 4.26 mmol, 1.0 equiv.), $Pd(PPh_3)_4$ (246 mg, 0.213 mmol, 5 mol%) and K_2CO_3 (727 µL, 12.8 mmol, 3.0 equiv.) and then THF (42.6 mL) was added. To the resulting mixture was added vinylboronic acid pinacol ester (867 µL, 5.11 mmol, 1.2 equiv.) and water (384 µL, 21.3 mmol, 5.0 equiv.) and the mixture was allowed to heat to reflux for 8 h. The now brown/yellow reaction mixture was allowed to cool to room temperature and was filtered through celite. The filtrate had the solvent concentrated *in vacuo* to give an orange residue. The crude product was purified using flash column chromatography (silica, EtOAc:hexane 10:90) to afford the desired product as an orange oil (550 mg, 37% over two steps).

Product was used immediately after obtaining ^{1}H NMR spectrum, due to stability issues.

^{1}H NMR (400 MHz, CDCl$_3$) δ 8.67 (d, *J* = 8.3 Hz, 1H), 8.56 (dd, *J* = 8.2, 1.4 Hz, 1H), 8.36 (d, *J* = 8.3 Hz, 1H), 8.19 (ddd, *J* = 8.2, 1.4, 0.6 Hz, 1H), 7.86 (ddd, *J* = 8.3, 7.0, 1.3 Hz, 1H), 7.78 – 7.60 (m, 4H), 6.59 (dd, *J* = 17.0, 2.0 Hz, 1H), 5.82 (dd, *J* = 10.8, 2.0 Hz, 1H).

2-(1-Chlorovinyl)quinoline (1)

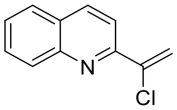

Chemical Formula: $C_{11}H_8ClN$
Exact Mass: 189.03
Molecular Weight: 189.64

Prepared according to General Procedure D using 2-vinylquinoline **S20** (3.88 g, 25.0 mmol, 1.0 equiv.), $KMnO_4$ (15.8 g, 250 mmol, 10 equiv.), conc. aq. HCl (20.9 mL, 250 mmol, 10 equiv.), KO^{t}Bu (5.61g, 50.0 mmol, 2.0 equiv.) and DCM (100 mL). The reaction mixture was subjected to the purification outlined in General Procedure D (silica, Et_2O:hexane, 1:99) to afford the desired product as a yellow solid (1.28 g, 27%).

ν_{max} (film): 1589, 1489, 750 cm^{-1}.

^{1}H NMR (500 MHz, CDCl$_3$): δ 8.21 (dd, *J* = 8.7, 0.9 Hz, 1H), 8.13 (d, *J* = 8.5 Hz, 1H), 7.88 (d, *J* = 8.6 Hz, 1H), 7.82 (dd, *J* = 8.3, 1.4 Hz, 1H), 7.74 (ddd, *J* = 8.5, 6.9, 1.5 Hz, 1H), 7.56 (ddd, *J* = 8.1, 6.8, 1.2 Hz, 1H), 6.64 (d, *J* = 1.4 Hz, 1H), 5.85 (d, *J* = 1.3 Hz, 1H).

^{13}C NMR (126 MHz, CDCl$_3$): δ 153.3, 147.5, 139.1, 137.1, 130.2, 129.9, 127.8, 127.5, 127.3, 118.7, 117.4.

HRMS (ESI): exact mass calculated for [M+H]$^+$ ($C_{11}H_9ClN$) requires 190.0418 *m/z*, found 190.0412 *m/z*.

2-(1-Chlorovinyl)-6-fluoroquinoline (S35)

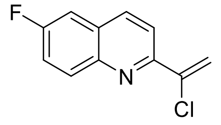

Chemical Formula: $C_{11}H_7ClFN$
Exact Mass: 207.0251
Molecular Weight: 207.6324

Prepared according to General Procedure D using 2-vinyl-6-fluoro-quinoline **S21** (450 mg, 2.60 mmol, 1.0 equiv.), $KMnO_4$ (4.11 g, 26.0 mmol, 10 equiv.), conc. aq. HCl (4.33 mL, 52.0 mmol, 20 equiv.), KO^tBu (583 mg, 5.20 mmol, 2.0 equiv.) and DCM (5.20 mL). The reaction mixture was subjected to the purification outlined in General Procedure D (silica, EtOAc:hexane, 10:90) to afford the desired product as a green solid (226 mg, 42%).

Product was used immediately after obtaining ^1H NMR spectrum, due to stability issues.

^1H NMR (500 MHz, $CDCl_3$): 8.19 – 8.07 (m, 2H), 7.90 (dd, *J* = 8.7, 0.7 Hz, 1H), 7.51 (ddd, *J* = 9.2, 8.3, 2.8 Hz, 1H), 7.44 (dd, *J* = 8.7, 2.8 Hz, 1H), 6.60 (d, *J* = 1.3 Hz, 1H), 5.84 (d, *J* = 1.3 Hz, 1H).

5-Chloro-2-(1-chlorovinyl)-6-methoxyquinoline (S36)

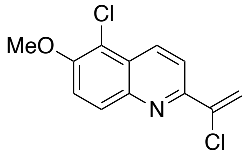

Chemical Formula: $C_{12}H_9Cl_2NO$
Exact Mass: 253.0061
Molecular Weight: 254.1100

Prepared according to General Procedure D using 2-vinyl-6-methoxyquinoline **S22** (741 mg, 4.00 mmol, 1.0 equiv.), $KMnO_4$ (3.16 g, 20.0 mmol, 5.0 equiv.), conc. aq. HCl (3.33 mL, 40.0 mmol, 10 equiv.), KO^tBu (898 mg, 8.00 mmol, 2.0 equiv.) and DCM (8 mL). The reaction mixture was subjected to the purification outlined in General Procedure D (silica, EtOAc:hexane, 15:85) to afford the desired product as a white solid (232 mg, 23%).

Product was used immediately after obtaining ^1H NMR spectrum, due to stability issues.

^1H NMR (500 MHz, $CDCl_3$): δ 8.56 (dd, *J* = 8.9, 0.8 Hz, 1H), 8.07 (dd, *J* = 9.4, 0.8 Hz, 1H), 7.93 (d, *J* = 9.0 Hz, 1H), 7.55 (d, *J* = 9.3 Hz, 1H), 6.57 (d, *J* = 1.4 Hz, 1H), 5.84 (d, *J* = 1.4 Hz, 1H), 4.08 (s, 3H).

2-(1-Chlorovinyl)-1-methyl-1*H*-benzo[*d*]imidazole (S37)

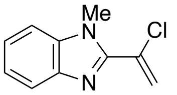

Chemical Formula: $C_{10}H_9ClN_2$
Exact Mass: 192.0454
Molecular Weight: 192.6460

Prepared according to General Procedure G using geminal dichloride **S24** (424 mg, 1.61 mmol, 1.0 equiv.), EtOH (6.44 mL) and acid-washed zinc dust (110 mg, 1.61 mmol, 1.0 equiv.). The reaction mixture was subjected to the purification outlined in General Procedure G (silica, Et_2O:toluene:Et_3N, 20:79:1) to afford the desired product as a white solid (136 mg, 37% over two steps).

Product was used immediately after obtaining ^1H NMR spectrum, due to stability issues.

^1H NMR (500 MHz, $CDCl_3$): δ 7.81 (dt, *J* = 7.8, 1.1 Hz, 1H), 7.40 – 7.35 (m, 2H), 7.35 – 7.30 (m, 1H), 6.07 (d, *J* = 1.8 Hz, 1H), 6.03 (d, *J* = 1.8 Hz, 1H), 3.91 (s, 3H).

2-(1-Chlorovinyl)-5-phenylthiazole (S38)

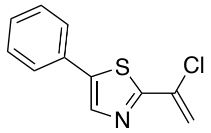

Chemical Formula: $C_{11}H_8ClNS$
Exact Mass: 221.0066
Molecular Weight: 221.7020

Prepared according to General Procedure G using geminal dichloride **S26** (683 mg, 2.33 mmol, 1.0 equiv.), EtOH (9.3 mL) and acid-washed zinc dust (153 mg, 2.33 mmol, 1.0 equiv.). The reaction mixture was subjected to the purification outlined in General Procedure G (silica, EtOAc:hexane, 5:95) to afford the desired product as a red oil (70.0 mg, 14%).

Product was used immediately after obtaining ^{1}H NMR spectrum, due to stability issues.

^{1}H NMR (500 MHz, $CDCl_3$): δ 7.99 (s, 1H), 7.58 – 7.56 (m, 2H), 7.43 – 7.39 (m, 2H), 7.39 – 7.33 (m, 1H), 6.43 (d, *J* = 2.1 Hz, 1H), 5.66 (d, *J* = 2.1 Hz, 1H).

2-(1-Chlorovinyl)quinoxaline (S39)

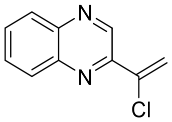

Chemical Formula: $C_{10}H_7ClN_2$
Exact Mass: 190.0298
Molecular Weight: 190.6300

Prepared according to General Procedure D using 2-vinylquinoxaline **S27** (400 mg, 2.56 mmol, 1.0 equiv.), $KMnO_4$ (4.05 g, 25.6 mmol, 10 equiv.), conc. aq. HCl (4.27 mL, 51.2 mmol, 20 equiv.), KO^{t}Bu (575 mg, 5.12 mmol, 2.0 equiv.) and DCM (5.12 mL). The reaction mixture was subjected to the purification outlined in General Procedure D (silica, EtOAc:hexane, 10:90) to afford the desired product as a black solid (187 mg, 38%).

Product was used immediately after obtaining ^{1}H NMR spectrum, due to stability issues.

^{1}H NMR (500 MHz, $CDCl_3$): 9.29 (s, 1H), 8.20 – 7.95 (m, 2H), 7.80 (dt, *J* = 6.2, 2.6 Hz, 2H), 6.66 (d, *J* = 1.6 Hz, 1H), 5.96 (d, *J* = 1.6 Hz, 1H).

2-(1-Chlorovinyl)quinazoline (S40)

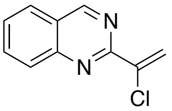

Chemical Formula: $C_{10}H_7ClN_2$
Exact Mass: 190.0298
Molecular Weight: 190.6300

Prepared according to General Procedure D using 2-vinylquinazoline **S28** (500 mg, 3.20 mmol, 1.0 equiv.), $KMnO_4$ (5.06 g, 32.0 mmol, 10 equiv.), conc. aq. HCl (5.34 mL, 64.0 mmol, 20 equiv.), KO^{t}Bu (718 mg, 6.40 mmol, 2.0 equiv.) and DCM (6.4 mL). The reaction mixture was subjected to the purification outlined in General Procedure D (silica, EtOAc:hexane, 15:85) to afford the desired product as a white solid (163 mg, 27 %).

Product was used immediately after obtaining ^{1}H NMR spectrum, due to stability issues.

^{1}H NMR (500 MHz, $CDCl_3$): δ 9.40 (d, *J* = 0.9 Hz, 1H), 8.17 – 8.10 (m, 1H), 7.96 (d, *J* = 7.5 Hz, 2H), 7.72 – 7.65 (m, 1H), 7.03 (d, *J* = 1.0 Hz, 1H), 6.10 (d, *J* = 1.0 Hz, 1H).

Methyl 6-(1-chlorovinyl)nicotinate (S41)

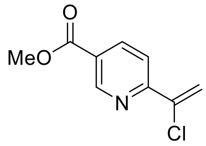

Chemical Formula: $C_9H_8ClNO_2$
Exact Mass: 197.0244
Molecular Weight: 197.6180

Prepared according to General Procedure D using methyl 6-vinylnicotinate **S29** (4.08 g, 25.0 mmol, 1.0 equiv.), $KMnO_4$ (15.8 g, 250 mmol, 10 equiv.), conc. aq. HCl (20.9 mL, 250 mmol, 10 equiv.), KO^{t}Bu (5.61 g, 50.0 mmol, 2 equiv.) and DCM (100 mL). The reaction mixture was subjected to the purification outlined in General Procedure D (silica, Et_2O:hexane, 1:99) to afford the desired product as a yellow solid (1.38 g, 28%).

ν_{max} (film): 791, 1125, 1285, 1296, 1730 cm^{-1}.

^{1}H NMR (500 MHz, CDCl$_3$): 9.17 (d, *J* = 1.6 Hz, 1H), 8.34 (dd, *J* = 8.3, 2.1 Hz, 1H), 7.84 (d, *J* = 8.3 Hz, 1H), 6.70 (d, *J* = 0.9 Hz, 1H), 5.82 (d, *J* = 0.9 Hz, 1H), 3.97 (s, 3H).

^{13}C NMR (126 MHz, CDCl$_3$): 165.5, 156.6, 150.4, 138.4, 137.7, 125.6, 120.4, 118.6, 52.6.

HRMS (ESI): exact mass calculated for $[M+H]^+$ ($C_9H_9ClNO_2$) requires *m/z* 198.0316, found *m/z* 198.0311.

6-(1-Chlorovinyl)nicotinonitrile (S42)

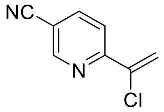

Chemical Formula: $C_8H_5ClN_2$
Exact Mass: 164.0141
Molecular Weight: 164.5920

Prepared according to General Procedure D using methyl 2-vinyl-5-cyanopyridine **S30** (350 mg, 2.69 mmol, 1.0 equiv.), $KMnO_4$ (4.25 g, 26.9 mmol, 10 equiv.), conc. aq. HCl (4.48 mL, 53.8 mmol, 20 equiv.), KO^{t}Bu (604 mg, 5.38 mmol, 2 equiv.) and DCM (100 mL). The reaction mixture was subjected to the purification outlined in General Procedure D (silica, EtOAc:hexane, 10:90) to afford the desired product as a white solid (80.0 mg, 18%).

Product was used immediately after obtaining ^{1}H NMR spectrum, due to stability issues.

^{1}H NMR (500 MHz, CDCl$_3$): 9.09 – 8.29 (m, 1H), 8.02 (dd, *J* = 8.3, 2.1 Hz, 1H), 7.88 (d, *J* = 8.3 Hz, 1H), 6.74 (d, *J* = 1.3 Hz, 1H), 5.87 (d, *J* = 1.2 Hz, 1H).

2-(1-Chlorovinyl)benzo[*d*]thiazole (S43)

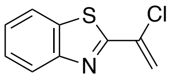

Chemical Formula: C_9H_6ClNS
Exact Mass: 194.9909
Molecular Weight: 195.6640

Prepared according to General Procedure G using geminal dichloride **S32** (630 mg, 2.36 mmol, 1.0 equiv.), EtOH (9.45 mL) and acid-washed zinc (155 mg, 2.36 mmol, 1.0 equiv.). The reaction mixture was subjected to the purification outlined in General Procedure G (silica, Et_2O:toluene, 1:99) to afford the desired product as an orange oil (49 mg, 6.5 % over two steps).

Product was used immediately after obtaining ^{1}H NMR spectrum, due to stability issues.

^{1}H NMR (500 MHz, CDCl$_3$): δ 8.07 (d, *J* = 8.1 Hz, 1H), 7.89 (d, *J* = 8.1 Hz, 1H), 7.52 (ddd, *J* = 8.3, 7.2, 1.3 Hz, 1H), 7.43 (ddd, *J* = 8.2, 7.2, 1.2 Hz, 1H), 6.56 (d, *J* = 2.1 Hz, 1H), 5.85 (d, *J* = 2.1 Hz, 1H).

6-(1-Chlorovinyl)phenanthridine (S44)

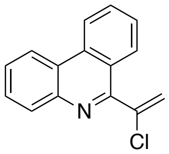

Chemical Formula: $C_{15}H_{10}ClN$
Exact Mass: 239.0502
Molecular Weight: 239.7020

Prepared according to General Procedure D using 6-vinylphenanthridine **S34** (513 mg, 2.50 mmol, 1.0 equiv.), $KMnO_4$ (1.98 g, 12.5 mmol, 5.0 equiv.), conc. aq. HCl (2.08 mL, 25 mmol, 10.0 equiv.), KotBu (561 mg, 5.00 mmol, 2.0 equiv.) and DCM (5 mL). The reaction mixture was subjected to the purification outlined in General Procedure D (silica, EtOAC:hexane, 1:9) to afford the desired product as a yellow solid (81.2 mg, 14%).

Product was used immediately after obtaining ^{1}H NMR spectrum, due to stability issues.

^{1}H NMR (400 MHz, CDCl$_3$): δ 8.67 (d, *J* = 8.4 Hz, 1H), 8.59 (dd, *J* = 8.1, 1.6 Hz, 1H), 8.37 (ddd, *J* = 8.3, 1.4, 0.7 Hz, 1H), 8.27 – 8.21 (m, 1H), 7.89 (ddd, *J* = 8.3, 7.0, 1.3 Hz, 1H), 7.80 – 7.68 (m, 3H), 6.07 (d, *J* = 1.7 Hz, 1H), 5.86 (d, *J* = 1.7 Hz, 1H).

(*R*)-*N*-(2-Chloro-2-(quinolin-2-yl)ethyl)aniline (2)

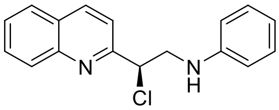

Chemical Formula: $C_{17}H_{15}ClN_2$
Exact Mass: 282.09
Molecular Weight: 282.77

Prepared according to General Procedure A using 2-(1-chlorovinyl)quinoline **1** (47.4 mg, 0.25 mmol, 1.0 equiv.), aniline (23 µL, 0.25 mmol, 1.0 equiv.), (*S*)-TCYP (12.4 mg, 12.5 µmol, 5 mol%), and 2-MeTHF (500 µL, 0.5 M). The reaction mixture was subjected to the purification outlined in General Procedure A (silica, EtOAc:petroleum ether, 5:95 to 10:90) to afford the desired product as a pale yellow solid (55.9 mg, 79%). The enantiomeric ratio (97:3) was determined by chiral HPLC in comparison with the racemate.

ν_{max} (film): 3302, 1599, 1497, 770 cm^{-1}.

^{1}H NMR (500 MHz, CDCl$_3$): δ 8.21 (d, *J* = 8.5 Hz, 1H), 8.14 (d, *J* = 8.5 Hz, 1H), 7.84 (d, *J* = 8.1 Hz, 1H), 7.81 – 7.74 (m, 1H), 7.64 – 7.55 (m, 2H), 7.21 – 7.14 (m, 2H), 6.73 (t, *J* = 7.3 Hz, 1H), 6.68 (d, *J* = 7.7 Hz, 2H), 5.38 (app. t, *J* = 6.7 Hz, 1H), 4.13 (dd, *J* = 14.0, 6.5 Hz, 1H), 3.88 (dd, *J* = 14.0, 7.0 Hz, 1H). ^{1}H resonance for NH not observed.

^{13}C NMR (126 MHz, CDCl$_3$): δ 158.1, 147.2, 147.1, 137.6, 130.2, 129.5, 129.5, 127.8, 127.7, 127.3, 120.5, 118.3, 113.5, 61.0, 49.6.

HRMS (ESI): exact mass calculated for $[M+H]^+$ ($C_{17}H_{16}ClN_2$) requires *m/z* 283.0997, found *m/z* 283.0994.

HPLC analysis using a chiral column: Chiralcel® OJ-H 5 µm column, 1.0 mL/min, 50:50 hexane:*iso*propanol, 250 nm, t_{major} = 19.4 min, t_{minor} = 30.9 min.

$[\alpha]_D^{23}$ = −72.8° (*c* = 1.0, CHCl$_3$).

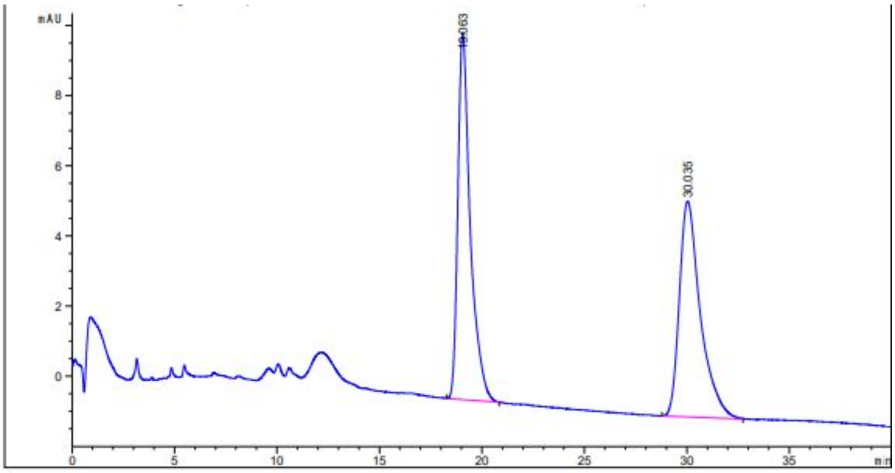

===

Area Percent Report

===

Sorted By : Signal
Multiplier : 1.0000
Dilution : 1.0000
Use Multiplier & Dilution Factor with ISTDs

Signal 1: VWD1 A, Wavelength=250 nm

Peak #	RetTime [min]	Type	Width [min]	Area [mAU*s]	Height [mAU]	Area %
1	19.063	BB	0.6256	440.55341	10.45397	50.2048
2	30.035	BB	0.9991	436.95993	6.15117	49.7952

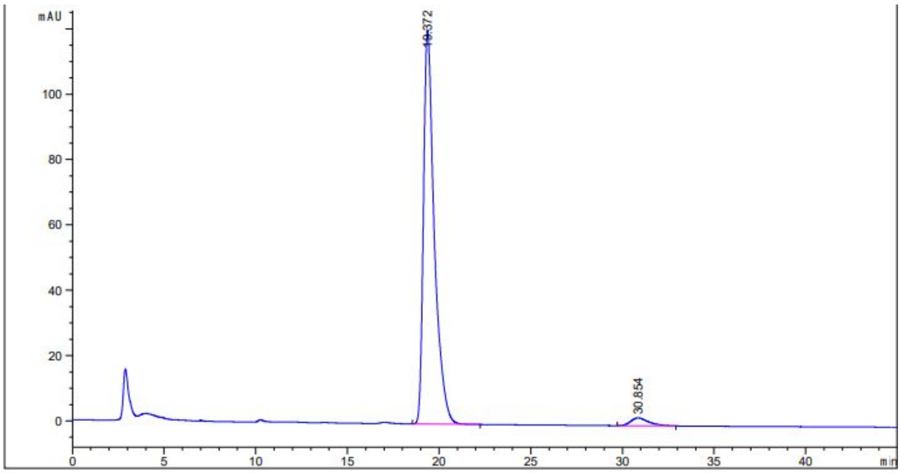

===

Area Percent Report

===

Sorted By : Signal
Multiplier : 1.0000
Dilution : 1.0000
Use Multiplier & Dilution Factor with ISTDs

Signal 1: VWD1 A, Wavelength=250 nm

Peak #	RetTime [min]	Type	Width [min]	Area [mAU*s]	Height [mAU]	Area %
1	19.372	BB	0.6096	4959.16016	120.54043	96.8012
2	30.854	BB	0.8721	163.87349	2.40941	3.1988

(*R*)-*N*-(2-Chloro-2-(quinolin-2-yl)ethyl)-4-methylaniline (13)

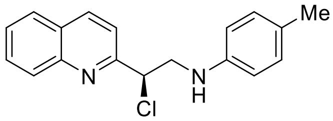

Chemical Formula: $C_{18}H_{17}ClN_2$
Exact Mass: 296.1080
Molecular Weight: 296.7980

Prepared according to General Procedure A using 2-(1-chlorovinyl)quinoline **1** (47.4 mg, 0.25 mmol, 1.0 equiv.), *p*-toluidine (26.8 mg, 0.25 mmol, 1.0 equiv.), (*S*)-TCYP (12.4 mg, 12.5 µmol, 5 mol%), and 2-MeTHF (500 µL, 0.5 M). The reaction mixture was subjected to the purification outlined in General Procedure A (silica, EtOAc:petroleum ether, 5:95 to 10:90) to afford the desired product as a pale yellow solid (62.8 mg, 85%). The enantiomeric ratio (97:3) was determined by chiral HPLC in comparison with the racemate.

ν_{max} (film): 1616, 1527, 770 cm^{-1}.

^{1}H NMR (500 MHz, $CDCl_3$): δ 8.19 (d, *J* = 8.5 Hz, 1H), 8.13 (d, *J* = 8.6 Hz, 1H), 7.83 (d, *J* = 8.1 Hz, 1H), 7.76 (ddd, *J* = 8.4, 6.9, 1.4 Hz, 1H), 7.62 – 7.55 (m, 2H), 7.01 (d, *J* = 8.1 Hz, 2H), 6.66 – 6.59 (m, 2H), 5.36 (app. t, *J* = 6.7 Hz, 1H), 4.11 (dd, *J* = 14.0, 6.4 Hz, 1H), 3.86 (dd, *J* = 14.0, 7.1 Hz, 1H), 2.26 (s, 3H). ^{1}H resonance for NH not observed.

^{13}C NMR (126 MHz, $CDCl_3$): δ 158.2, 147.4, 144.8, 137.4, 130.05, 130.00, 129.6, 127.7, 127.6, 127.2, 120.5, 113.7, 61.3, 50.0, 20.5.

HRMS (ESI): exact mass calculated for $[M+H]^+$ ($C_{18}H_{18}ClN_2$) requires *m/z* 297.1153, found *m/z* 297.1151.

HPLC analysis using a chiral column: Chiralcel® OJ-H 5 µm column, 1.0 mL/min, 50:50 hexane:*iso*propanol, 250 nm, t_{major} = 22.0 min, t_{minor} = 25.9 min.

$[\alpha]_D^{23}$ = −83.7° (*c* = 1.0, $CHCl_3$).

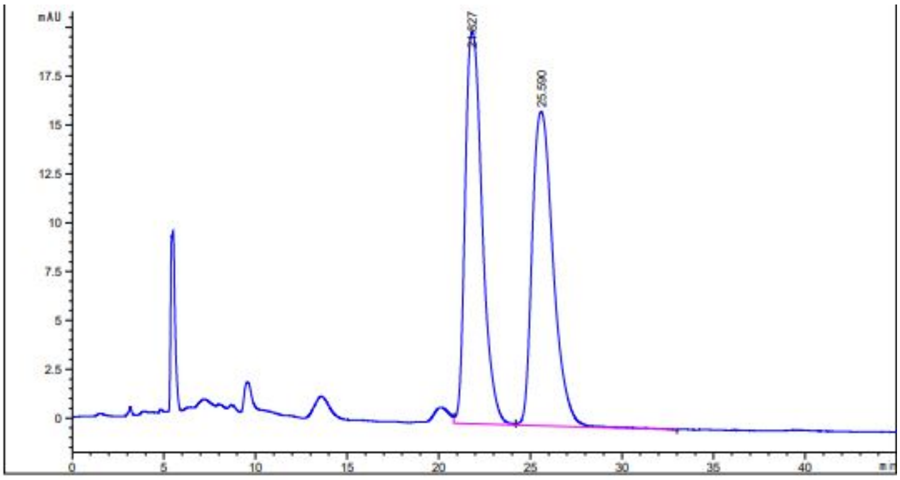


```
=====================================================================
                     Area Percent Report
=====================================================================

Sorted By             :      Signal
Multiplier            :      1.0000
Dilution              :      1.0000
Use Multiplier & Dilution Factor with ISTDs


Signal 1: VWD1 A, Wavelength=250 nm

Peak RetTime Type  Width     Area      Height     Area
  #   [min]        [min]   [mAU*s]     [mAU]        %
----|-------|----|-------|----------|----------|--------|
   1  21.827 VV    1.0167 1298.38367   20.07287  50.3300
   2  25.590 VB    1.2439 1281.35669   16.08544  49.6700
```

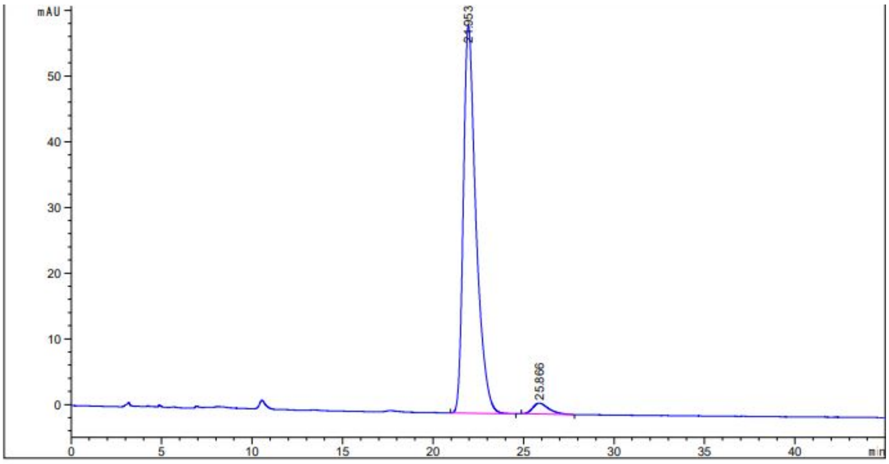

Area Percent Report

Sorted By : Signal
Multiplier : 1.0000
Dilution : 1.0000
Use Multiplier & Dilution Factor with ISTDs

Signal 1: VWD1 A, Wavelength=250 nm

Peak #	RetTime [min]	Type	Width [min]	Area [mAU*s]	Height [mAU]	Area %
1	21.953	BB	0.7246	2868.87549	58.99772	96.5300
2	25.866	BB	0.9038	103.12813	1.65677	3.4700

(*R*)-*N*-(2-Chloro-2-(quinolin-2-yl)ethyl)-3-methylaniline (14)

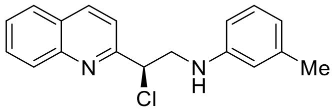

Chemical Formula: $C_{18}H_{17}ClN_2$
Exact Mass: 296.11
Molecular Weight: 296.80

Prepared according to General Procedure A using 2-(1-chlorovinyl)quinoline **1** (47.4 mg, 0.25 mmol, 1.0 equiv.), *m*-toluidine (26 µL, 0.25 mmol, 1.0 equiv.), (*S*)-TCYP (12.4 mg, 12.5 µmol, 5 mol%), and 2-MeTHF (500 µL, 0.5 M). The reaction mixture was subjected to the purification outlined in General Procedure A (silica, EtOAc:petroleum ether, 5:95 to 10:90) to afford the desired product as a pale yellow solid (74.2 mg, >99%). The enantiomeric ratio (97:3) was determined by chiral HPLC in comparison with the racemate.

ν_{max} (film): 3298, 1607, 1491, 768 cm^{-1}.

^{1}H NMR (500 MHz, $CDCl_3$): δ 8.20 (d, *J* = 8.5 Hz, 1H), 8.15 (d, *J* = 8.5 Hz, 1H), 7.84 (d, *J* = 8.1 Hz, 1H), 7.77 (ddd, *J* = 8.4, 6.9, 1.4 Hz, 1H), 7.63 – 7.56 (m, 2H), 7.11 – 7.03 (m, 1H), 6.56 (d, *J* = 7.5 Hz, 1H), 6.53 – 6.48 (m, 2H), 5.40 (app. t, *J* = 6.7 Hz, 1H), 4.12 (dd, *J* = 14.0, 6.6 Hz, 1H), 3.89 (dd, *J* = 14.0, 6.9 Hz, 1H), 2.26 (s, 3H). ^{1}H resonance for NH not observed.

^{13}C NMR (126 MHz, $CDCl_3$): δ 158.1, 147.01, 146.96, 139.3, 137.6, 130.2, 129.4, 127.7, 127.6, 127.3, 120.5, 119.3, 114.3, 110.6, 61.0, 49.7, 21.7.

HRMS (ESI): exact mass calculated for $[M+H]^+$ ($C_{18}H_{18}ClN_2$) requires *m/z* 297.1153, found *m/z* 297.1148.

HPLC analysis using a chiral column: Chiralcel® OJ-H 5 µm column, 1.0 mL/min, 50:50 hexane:*iso*propanol, 250 nm, t_{major} 21.4 = min, t_{minor} 24.2 = min.

$[\alpha]_D^{23}$ = −62.2° (*c* = 1.0, $CHCl_3$).

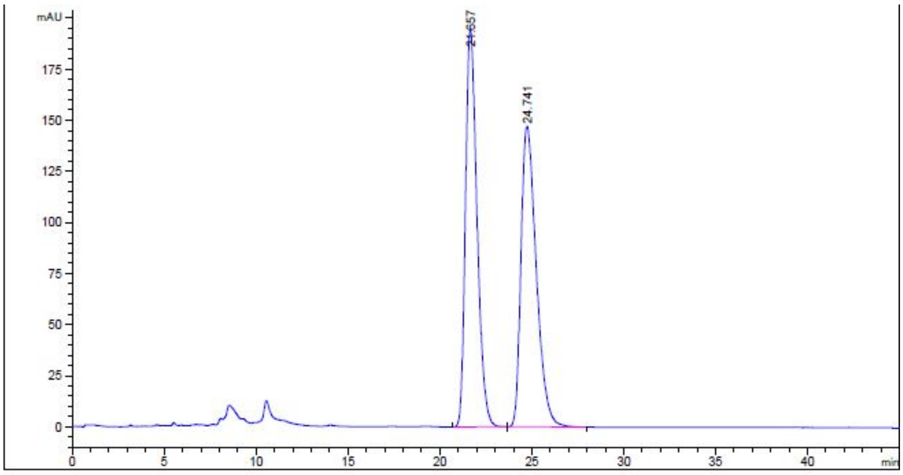

==

Area Percent Report

==

Sorted By : Signal
Multiplier : 1.0000
Dilution : 1.0000
Use Multiplier & Dilution Factor with ISTDs

Signal 1: VWD1 A, Wavelength=250 nm

Peak #	RetTime [min]	Type	Width [min]	Area [mAU*s]	Height [mAU]	Area %
1	21.657	BB	0.6727	8486.55371	194.26649	50.0288
2	24.741	BB	0.8839	8476.79492	147.00319	49.9712

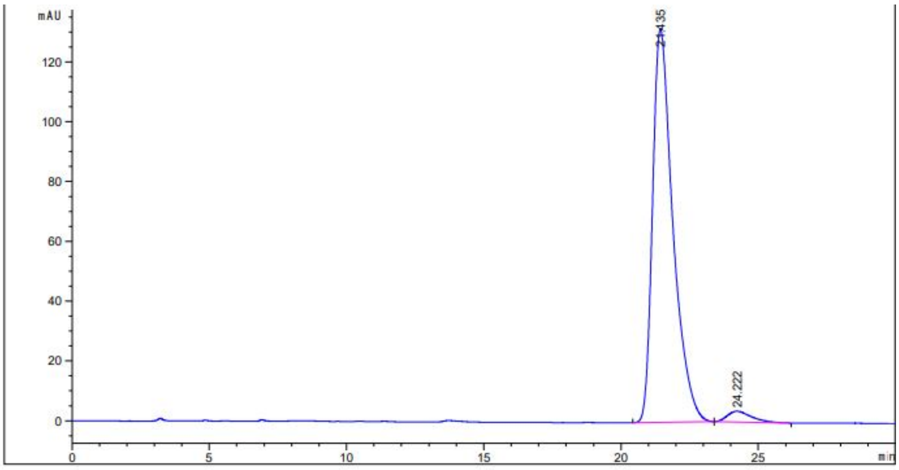

==

Area Percent Report

==

Sorted By : Signal
Multiplier : 1.0000
Dilution : 1.0000
Use Multiplier & Dilution Factor with ISTDs

Signal 1: VWD1 A, Wavelength=250 nm

Peak #	RetTime [min]	Type	Width [min]	Area [mAU*s]	Height [mAU]	Area %
1	21.435	BB	0.7719	6778.82275	131.46518	96.8647
2	24.222	BB	0.8426	219.41626	3.60480	3.1353

(*R*)-*N*-(2-Chloro-2-(quinolin-2-yl)ethyl)-2-methylaniline (15)

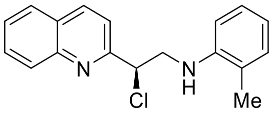

Chemical Formula: $C_{18}H_{17}ClN_2$
Exact Mass: 296.1080
Molecular Weight: 296.7980

Prepared according to General Procedure A using 2-(1-chlorovinyl)quinoline **1** (47.4 mg, 0.25 mmol, 1.0 equiv.), *o*-toluidine (27 µL, 0.25 mmol, 1.0 equiv.), (*S*)-TCYP (12.4 mg, 12.5 µmol, 5 mol%), and 2-MeTHF (500 µL, 0.5 M) for 39 h. The reaction mixture was subjected to the purification outlined in General Procedure A (silica, EtOAc:petroleum ether, 5:95 to 10:90) to afford the desired product as a pale yellow oil (50.8 mg, 68%). The enantiomeric ratio (91:9) was determined by chiral HPLC in comparison with the racemate.

ν_{max} (film): 1605, 1505, 746 cm^{-1}.

^{1}H NMR (500 MHz, $CDCl_3$): δ 8.21 (d, *J* = 8.5 Hz, 1H), 8.13 (d, *J* = 8.5 Hz, 1H), 7.84 (d, *J* = 8.1 Hz, 1H), 7.80 – 7.73 (m, 1H), 7.63 (d, *J* = 8.5 Hz, 1H), 7.59 (t, *J* = 7.5 Hz, 1H), 7.15 (t, *J* = 7.7 Hz, 1H), 7.06 (d, *J* = 7.3 Hz, 1H), 6.76 (d, *J* = 8.0 Hz, 1H), 6.70 (t, *J* = 7.3 Hz, 1H), 5.41 (app. t, *J* = 6.7 Hz, 1H), 4.43 (s, 1H), 4.14 (dd, *J* = 13.8, 6.5 Hz, 1H), 3.94 (dd, *J* = 13.8, 6.9 Hz, 1H), 2.10 (s, 3H).

^{13}C NMR (126 MHz, $CDCl_3$): δ 158.2, 147.3, 145.2, 137.5, 130.5, 130.2, 129.5, 127.74, 127.71, 127.32, 127.25, 122.8, 120.4, 117.8, 110.2, 61.0, 49.6, 17.5.

HRMS (ESI): exact mass calculated for $[M+H]^+$ ($C_{18}H_{18}ClN_2$) requires *m/z* 297.1153, found *m/z* 297.1150.

HPLC analysis using a chiral column: Chiralcel® OJ-H 5 µm column, 1.0 mL/min, 50:50 hexane:*iso*propanol, 250 nm, t_{major} = 10.8 min, t_{minor} = 12.6 min.

$[\alpha]_D^{23}$ = −58.9° (*c* = 1.0, $CHCl_3$).

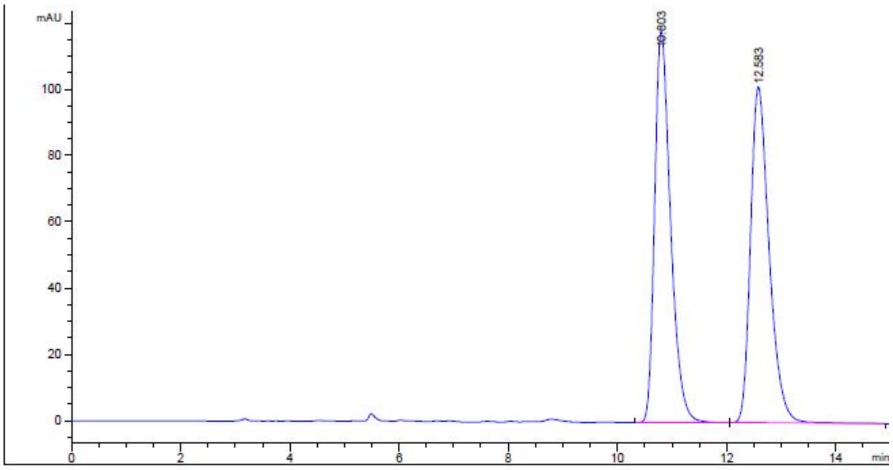


```
=====================================================================
                         Area Percent Report
=====================================================================

Sorted By             :      Signal
Multiplier            :      1.0000
Dilution              :      1.0000
Use Multiplier & Dilution Factor with ISTDs


Signal 1: VWD1 A, Wavelength=250 nm

Peak RetTime Type  Width     Area      Height     Area
  #   [min]        [min]   [mAU*s]     [mAU]       %
----|-------|----|-------|----------|----------|--------|
   1  10.803 BB    0.3074 2392.99902  118.26573  49.9152
   2  12.583 BB    0.3611 2401.12720  101.25949  50.0848
```

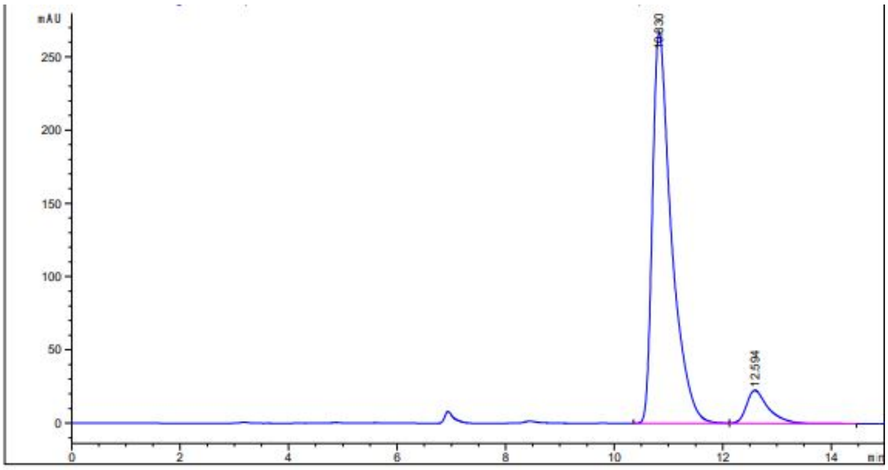


```
=====================================================================
                     Area Percent Report
=====================================================================

Sorted By              :      Signal
Multiplier             :      1.0000
Dilution               :      1.0000
Use Multiplier & Dilution Factor with ISTDs


Signal 1: VWD1 A, Wavelength=250 nm

Peak RetTime Type  Width     Area      Height     Area
  #   [min]        [min]   [mAU*s]     [mAU]        %
----|-------|----|-------|----------|----------|--------|
   1  10.830 BV    0.3588 6508.39795  266.82861  91.0994
   2  12.594 VB    0.4111  635.88080   22.70988   8.9006
```

(*R*)-*N*-(2-Chloro-2-(quinolin-2-yl)ethyl)-4-methoxyaniline (16)

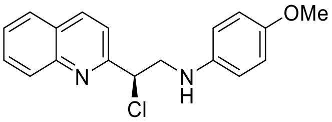

Chemical Formula: $C_{18}H_{17}ClN_2O$
Exact Mass: 312.1029
Molecular Weight: 312.7970

Prepared according to General Procedure A using 2-(1-chlorovinyl)quinoline **1** (47.4 mg, 0.25 mmol, 1.0 equiv.), *p*-anisidine (30.8 mg, 0.25 mmol, 1.0 equiv.), (*S*)-TCYP (12.4 mg, 12.5 µmol, 5 mol%), and 2-MeTHF (500 µL, 0.5 M) for 39 h. The reaction mixture was subjected to the purification outlined in General Procedure A (silica, EtOAc:petroleum ether, 5:95 to 10:90) to afford the desired product as a pale yellow solid (48.6 mg, 62%). The enantiomeric ratio (96:4) was determined by chiral HPLC in comparison with the racemate.

ν_{max} (film): 3292, 1530, 1506, 772 cm^{-1}.

^{1}H NMR (500 MHz, CDCl$_3$): δ 8.19 (d, *J* = 8.5 Hz, 1H), 8.11 (d, *J* = 8.5 Hz, 1H), 7.83 (d, *J* = 8.1 Hz, 1H), 7.75 (t, *J* = 7.7 Hz, 1H), 7.63 – 7.54 (m, 2H), 6.80 – 6.75 (m, 2H), 6.70 – 6.63 (m, 2H), 5.34 (app. T, *J* = 6.7 Hz, 1H), 4.09 (s, 1H), 4.05 (dd, *J* = 14.0, 6.2 Hz, 1H), 3.81 (dd, *J* = 13.9, 7.2 Hz, 1H), 3.75 (s, 3H).

^{13}C NMR (126 MHz, CDCl$_3$): δ 158.2, 152.8, 147.5, 141.2, 137.4, 130.1, 129.6, 127.7, 127.2, 120.4, 115.2, 115.1, 61.5, 55.9, 50.9.

HRMS (ESI): exact mass calculated for [M+H]$^+$ ($C_{18}H_{18}ClN_2O$) requires *m/z* 313.1102, found *m/z* 313.1101.

HPLC analysis using a chiral column: Chiralcel® OJ-H 5 µm column, 1.0 mL/min, 50:50 hexane:*iso*propanol, 250 nm, t_{major} = 39.5 min, t_{minor} = 34.5 min.

$[\alpha]_D^{23}$ = −79.0° (*c* = 1.0, CHCl$_3$).

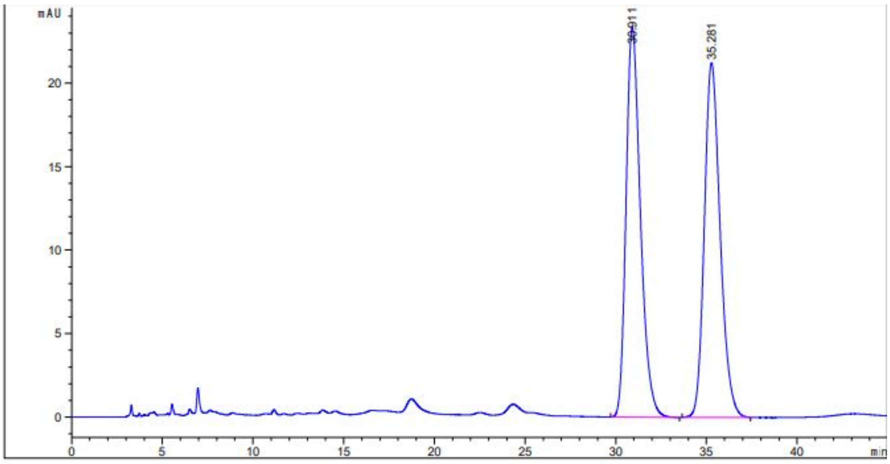


```
=====================================================================
                         Area Percent Report
=====================================================================

Sorted By             :      Signal
Multiplier            :      1.0000
Dilution              :      1.0000
Use Multiplier & Dilution Factor with ISTDs


Signal 1: VWD1 A, Wavelength=250 nm

Peak RetTime Type  Width     Area      Height     Area
  #   [min]        [min]   [mAU*s]     [mAU]       %
----|-------|----|-------|----------|----------|--------|
   1  30.911 BB    0.8367 1287.89966   23.36685  50.0175
   2  35.281 BB    0.9252 1286.99915   21.24463  49.9825

Totals :                  2574.89880   44.61148
```

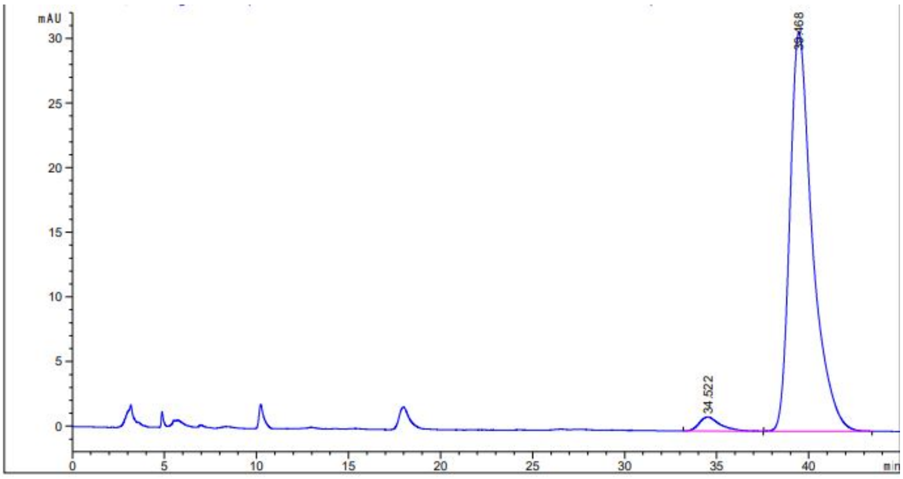


```
=====================================================================
                         Area Percent Report
=====================================================================

Sorted By             :      Signal
Multiplier            :      1.0000
Dilution              :      1.0000
Use Multiplier & Dilution Factor with ISTDs


Signal 1: VWD1 A, Wavelength=250 nm

Peak RetTime Type  Width     Area      Height     Area
  #   [min]        [min]   [mAU*s]     [mAU]       %
----|-------|----|-------|----------|----------|--------|
   1  34.522 BB    1.0145   85.46322    1.08916   3.1477
   2  39.468 BB    1.2679 2629.61621   30.85828  96.8523
```

(*R*)-*N*-(2-Chloro-2-(quinolin-2-yl)ethyl)-3,5-bis(trifluoromethyl)aniline (17)

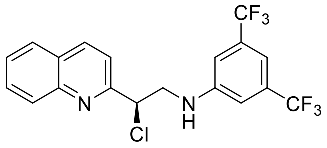

Chemical Formula: $C_{19}H_{13}ClF_6N_2$
Exact Mass: 418.07
Molecular Weight: 418.77

Prepared according to General Procedure A using 2-(1-chlorovinyl)quinoline **1** (47.4 mg, 0.25 mmol, 1.0 equiv.), 3,5-bis(trifluoromethyl)aniline (39 µL, 0.25 mmol, 1.0 equiv.), (*S*)-TCYP (12.4 mg, 12.5 µmol, 5 mol%), and 2-MeTHF (500 µL, 0.5 M) at RT. The reaction mixture was subjected to the purification outlined in General Procedure A (silica, EtOAc:petroleum ether, 5:95 to 10:90) to afford the desired product as a pale yellow solid (75.5 mg, 72%). The enantiomeric ratio (50:50) was determined by chiral HPLC in comparison with the racemate.

ν_{max} (film): 1620, 1393, 1273, 1121, 681 cm^{-1}.

^{1}H NMR (500 MHz, CDCl$_3$): δ 8.22 (d, *J* = 8.5 Hz, 1H), 8.11 (d, *J* = 8.4 Hz, 1H), 7.84 (d, *J* = 8.1 Hz, 1H), 7.78 (t, *J* = 7.6 Hz, 1H), 7.67 – 7.56 (m, 2H), 7.15 (s, 1H), 7.00 (s, 2H), 5.34 (app. t, *J* = 6.3 Hz, 1H), 4.92 (s, 1H), 4.17 (app. dt, *J* = 13.0, 6.1 Hz, 1H), 3.99 (app. dt, *J* = 13.1, 5.8 Hz, 1H).

^{13}C NMR (126 MHz, CDCl$_3$): δ 157.4, 148.1, 147.3, 137.8, 132.6 (q, ^2J = 32.7 Hz), 130.4, 129.5, 127.8, 127.5, 123.6 (q, ^1J = 272.7 Hz), 120.4, 112.7 – 112.1 (m), 111.1 – 110.9 (m), 60.6, 49.0.

^{19}F NMR (470 MHz, CDCl$_3$): δ −63.17.

HRMS (ESI): exact mass calculated for $[M+H]^+$ ($C_{19}H_{14}ClF_6N_2$) requires *m/z* 419.0744, found *m/z* 419.0738.

HPLC analysis using a chiral column: Chiral Art Amylose-C 5 µm column, 1.0 mL/min, 99:1 hexane:*iso*propanol, 250 nm, t_{major} = 14.9 min, t_{minor} = 12.1 min.

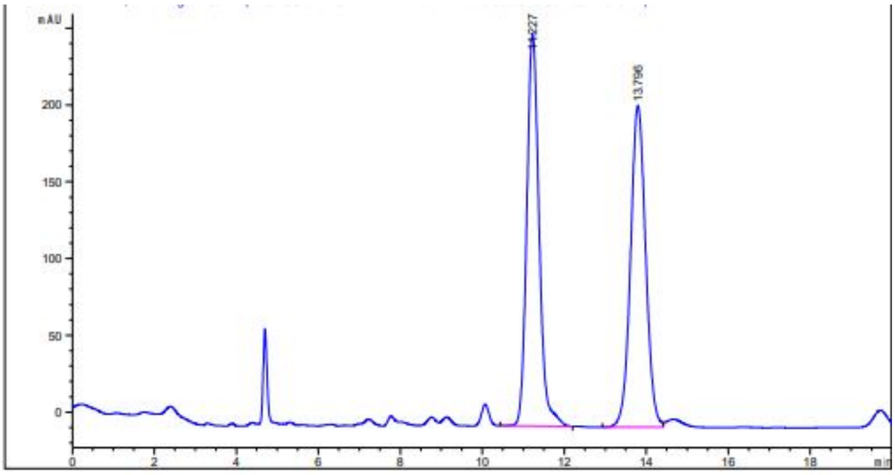


```
=====================================================================
                         Area Percent Report
=====================================================================

Sorted By             :      Signal
Multiplier            :      1.0000
Dilution              :      1.0000
Use Multiplier & Dilution Factor with ISTDs


Signal 1: VWD1 A, Wavelength=250 nm

Peak RetTime Type  Width     Area      Height     Area
  #   [min]        [min]   [mAU*s]     [mAU]        %
----|-------|----|-------|----------|----------|--------|
   1  11.227 BB    0.3221 5335.00146  255.58698  49.8586
   2  13.796 BV    0.3962 5365.27246  209.58109  50.1414
```

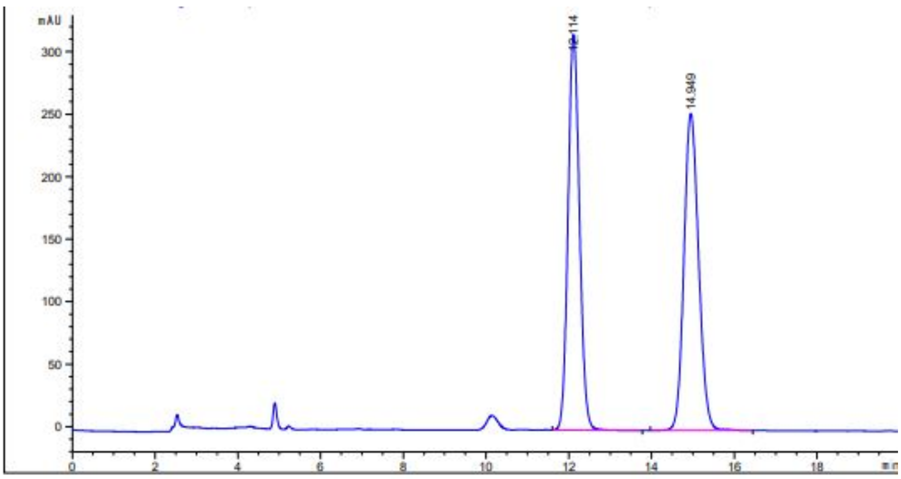


```
=====================================================================
                        Area Percent Report
=====================================================================

Sorted By             :      Signal
Multiplier            :      1.0000
Dilution              :      1.0000
Use Multiplier & Dilution Factor with ISTDs


Signal 1: VWD1 A, Wavelength=250 nm
```

Peak #	RetTime [min]	Type	Width [min]	Area [mAU*s]	Height [mAU]	Area %
1	12.114	VB	0.2984	6080.30811	316.47055	49.3371
2	14.949	BB	0.3819	6243.69092	253.55573	50.6629

(*R*)-*N*-(2-Chloro-2-(quinolin-2-yl)ethyl)-4-fluoroaniline (18)

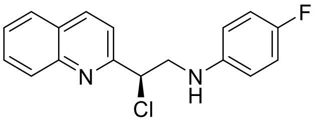

Chemical Formula: $C_{17}H_{14}ClFN_2$
Exact Mass: 300.08
Molecular Weight: 300.76

Prepared according to General Procedure A using 2-(1-chlorovinyl)quinoline **1** (47.4 mg, 0.25 mmol, 1.0 equiv.), 4-fluoroaniline (27 µL, 0.25 mmol, 1.0 equiv.), (*S*)-TCYP (12.4 mg, 12.5 µmol, 5 mol%), and 2-MeTHF (500 µL, 0.5 M). The reaction mixture was subjected to the purification outlined in General Procedure A (silica, EtOAc:petroleum ether, 5:95 to 10:90) to afford the desired product as a pale yellow solid (66.0 mg, 88%). The enantiomeric ratio (96:4) was determined by chiral HPLC in comparison with the racemate.

ν_{max} (film): 1506, 1223, 770 cm^{-1}.

^{1}H NMR (400 MHz, $CDCl_3$): δ 8.19 (d, *J* = 8.5 Hz, 1H), 8.11 (d, *J* = 8.5 Hz, 1H), 7.83 (d, *J* = 8.1 Hz, 1H), 7.78 – 7.72 (m, 1H), 7.63 – 7.56 (m, 2H), 6.95 – 6.85 (m, 2H), 6.66 – 6.58 (m, 2H), 5.34 (app. t, *J* = 6.7 Hz, 1H), 4.26 (s, 1H), 4.07 (dd, *J* = 14.0, 6.3 Hz, 1H), 3.83 (dd, *J* = 13.9, 7.1 Hz, 1H).

^{13}C NMR (126 MHz, $CDCl_3$): δ 158.0, 156.3 (d, ^1J = 235.8 Hz), 147.4, 143.4 (d, ^4J = 1.6 Hz), 137.5, 130.1, 129.6, 127.7, 127.3, 120.4, 116.0 (d, ^2J = 22.4 Hz), 114.5 (d, ^3J = 7.4 Hz), 61.3, 50.4.

^{19}F NMR (470 MHz, $CDCl_3$): δ −126.98.

HRMS (ESI): exact mass calculated for $[M+H]^+$ ($C_{17}H_{15}ClFN_2$) requires *m/z* 301.0902, found *m/z* 301.0898.

HPLC analysis using a chiral column: Chiralcel® OJ-H 5 µm column, 1.0 mL/min, 50:50 hexane:*iso*propanol, 250 nm, t_{major} = 14.2 min, t_{minor} = 19.9 min.

$[\alpha]_D^{23}$ = −51.8° (*c* = 1.0, $CHCl_3$).

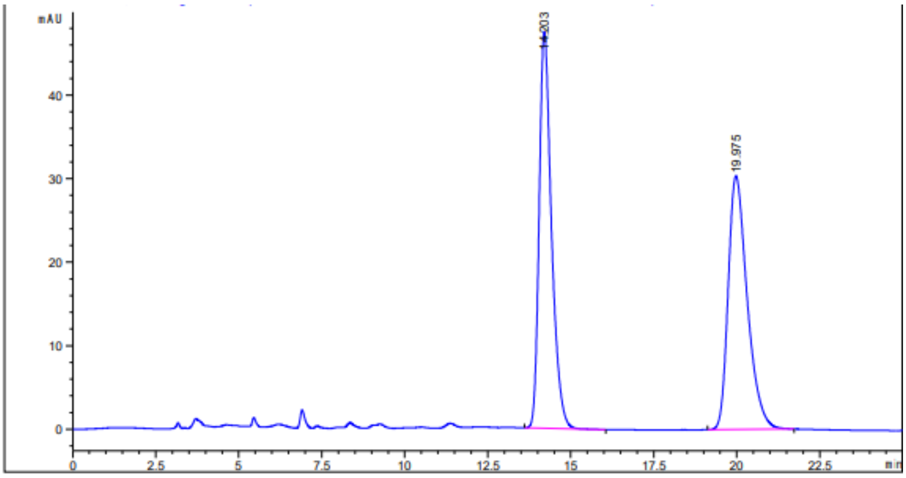

Area Percent Report

Sorted By : Signal
Multiplier : 1.0000
Dilution : 1.0000
Use Multiplier & Dilution Factor with ISTDs

Signal 1: VWD1 A, Wavelength=250 nm

Peak #	RetTime [min]	Type	Width [min]	Area [mAU*s]	Height [mAU]	Area %
1	14.203	BB	0.3878	1222.53479	47.47438	50.2507
2	19.975	BB	0.6034	1210.33704	30.36016	49.7493

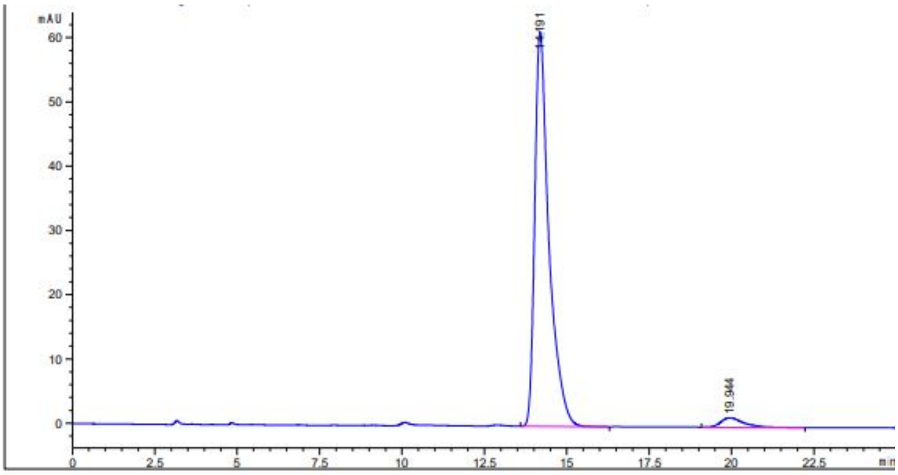

Area Percent Report

Sorted By : Signal
Multiplier : 1.0000
Dilution : 1.0000
Use Multiplier & Dilution Factor with ISTDs

Signal 1: VWD1 A, Wavelength=250 nm

Peak #	RetTime [min]	Type	Width [min]	Area [mAU*s]	Height [mAU]	Area %
1	14.191	VB	0.4406	1842.79871	61.36278	96.2827
2	19.944	BB	0.7161	71.14742	1.48522	3.7173
Totals :				1913.94612	62.84800	

(*R*)-*N*-(2-Chloro-2-(quinolin-2-yl)ethyl)-4-(4,4,5,5-tetramethyl-1,3,2-dioxaborolan-2-yl)aniline (19)

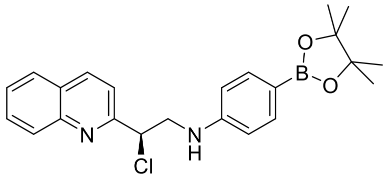

Chemical Formula: $C_{23}H_{26}BClN_2O_2$
Exact Mass: 408.1776
Molecular Weight: 408.7330

Prepared according to General Procedure A using 2-(1-chlorovinyl)quinoline **1** (47.4 mg, 0.25 mmol, 1.0 equiv.), 4-aminophenylboronic acid pinacol ester (54.8 mg, 0.25 mmol, 1.0 equiv.), (*S*)-TCYP (12.4 mg, 12.5 µmol, 5 mol%), and CPME (500 µL, 0.5 M) for 39 h. The reaction mixture was subjected to the purification outlined in General Procedure A (silica, EtOAc:petroleum ether, 5:95 to 10:90) to afford the desired product as a pale yellow solid (93.2 mg, 91%). The enantiomeric ratio (94:6) was determined by chiral HPLC in comparison with the racemate.

ν_{max} (film): 1605, 1358, 1142, 756, 656 cm^{-1}.

^{1}H NMR (300 MHz, $CDCl_3$): δ 8.19 (d, *J* = 8.5 Hz, 1H), 8.12 (d, *J* = 8.4 Hz, 1H), 7.83 (d, *J* = 8.1 Hz, 1H), 7.79 – 7.72 (m, 1H), 7.64 (d, *J* = 8.5 Hz, 2H), 7.61 – 7.51 (m, 2H), 6.65 (d, *J* = 8.6 Hz, 2H), 5.35 (app. t, *J* = 6.7 Hz, 1H), 4.55 (s, 1H), 4.17 (dd, *J* = 14.0, 6.7 Hz, 1H), 3.93 (dd, *J* = 14.0, 6.8 Hz, 1H), 1.32 (s, 12H).

^{13}C NMR (75 MHz, $CDCl_3$): δ 157.9, 149.7, 147.3, 137.5, 136.6, 130.2, 129.5, 127.7, 127.3, 120.6, 112.4, 83.4, 60.8, 48.9, 25.0.

n.b. the carbon bearing boron is not observed due to quadrupolar relaxation.

^{11}B NMR (96 MHz, $CDCl_3$): δ 30.67.

HRMS (ESI): exact mass calculated for $[M+H]^+$ ($C_{23}H_{27}BClN_2O_2$) requires *m/z* 409.1849, found *m/z* 409.1844.

HPLC analysis using a chiral column: Chiral Art Amylose-C 5 µm column, 1.0 mL/min, 90:10 hexane:*iso*propanol, 250 nm, t_{major} = 13.2 min, t_{minor} = 12.2 min.

$[\alpha]_D^{23}$ = −89.8° (*c* = 1.0, $CHCl_3$).

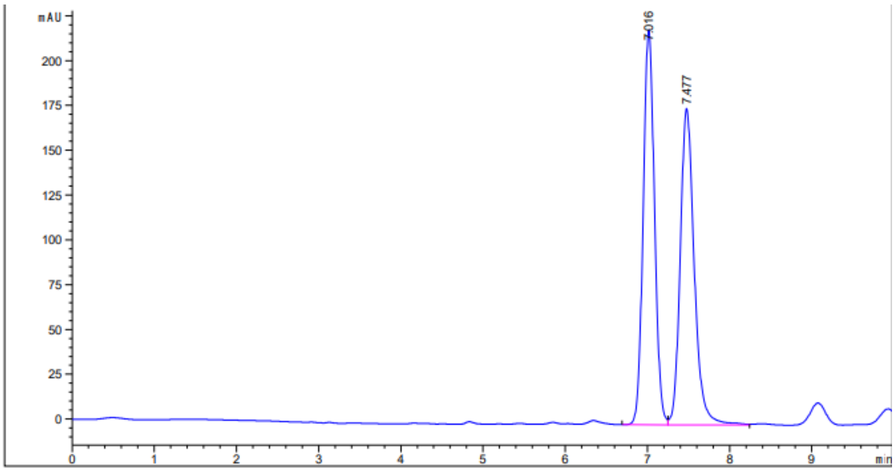

==
Area Percent Report
==

Sorted By : Signal
Multiplier : 1.0000
Dilution : 1.0000
Use Multiplier & Dilution Factor with ISTDs

Signal 1: VWD1 A, Wavelength=250 nm

Peak #	RetTime [min]	Type	Width [min]	Area [mAU*s]	Height [mAU]	Area %
1	7.016	BV	0.1479	2105.34839	220.19962	50.1230
2	7.477	VV	0.1807	2095.01221	176.55792	49.8770

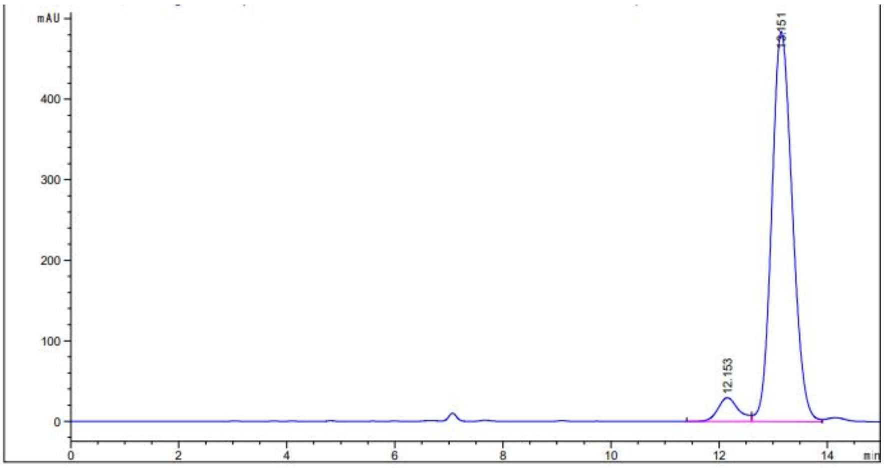

==
Area Percent Report
==

Sorted By : Signal
Multiplier : 1.0000
Dilution : 1.0000
Use Multiplier & Dilution Factor with ISTDs

Signal 1: VWD1 A, Wavelength=250 nm

Peak #	RetTime [min]	Type	Width [min]	Area [mAU*s]	Height [mAU]	Area %
1	12.153	BV	0.4014	790.22559	29.63072	5.7910
2	13.151	VV	0.4130	1.28556e4	484.37842	94.2090

(*R*)-4-Bromo-*N*-(2-chloro-2-(quinolin-2-yl)ethyl)aniline (20)

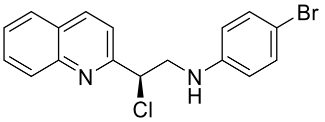

Chemical Formula: $C_{17}H_{14}BrClN_2$
Exact Mass: 360.0029
Molecular Weight: 361.6670

Prepared according to General Procedure A using 2-(1-chlorovinyl)quinoline **1** (47.4 mg, 0.25 mmol, 1.0 equiv.), 4-bromoaniline (43.0 mg, 0.25 mmol, 1.0 equiv.), (*S*)-TCYP (12.4 mg, 12.5 µmol, 5 mol%), and 2-MeTHF (500 µL, 0.5 M). The reaction mixture was subjected to the purification outlined in General Procedure A (silica, EtOAc:petroleum ether, 5:95 to 10:90) to afford the desired product as a pale yellow solid (74.0 mg, 82%). The enantiomeric ratio (95:5) was determined by chiral HPLC in comparison with the racemate.

ν_{max} (film): 1591, 1489, 812, 776 cm^{-1}.

^{1}H NMR (500 MHz, $CDCl_3$): δ 8.21 (d, *J* = 8.5 Hz, 1H), 8.11 (d, *J* = 8.5 Hz, 1H), 7.84 (d, *J* = 8.1 Hz, 1H), 7.80 – 7.73 (m, 1H), 7.62 – 7.56 (m, 2H), 7.24 (d, *J* = 8.8 Hz, 2H), 6.55 (d, *J* = 8.8 Hz, 2H), 5.33 (app. t, *J* = 6.7 Hz, 1H), 4.40 (s, 1H), 4.08 (dd, *J* = 14.1, 6.5 Hz, 1H), 3.85 (dd, *J* = 14.0, 7.0 Hz, 1H).

^{13}C NMR (126 MHz, $CDCl_3$): δ 157.8, 147.2, 146.1, 137.7, 132.2, 130.3, 129.4, 127.8, 127.7, 127.4, 120.5, 115.0, 109.8, 60.8, 49.5.

HRMS (ESI): exact mass calculated for $[M+H]^+$ ($C_{17}H_{15}BrClN_2$) requires *m/z* 361.0102, found *m/z* 361.0090.

HPLC analysis using a chiral column: Chiralcel® OJ-H 5 µm column, 1.0 mL/min, 50:50 hexane:*iso*propanol, 250 nm, t_{major} = 30.3 min, t_{minor} = 34.6 min.

$[\alpha]_D^{23}$ = −51.8° (*c* = 1.0, $CHCl_3$).

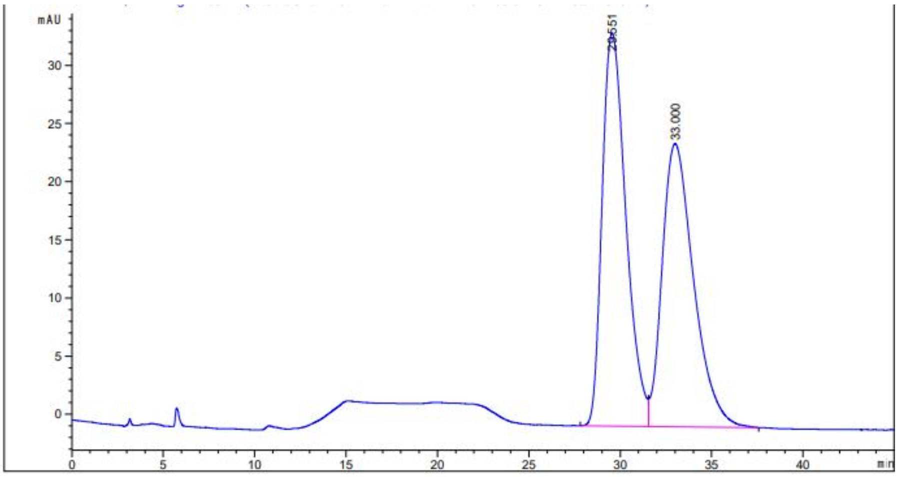


```
=====================================================================
                         Area Percent Report
=====================================================================

Sorted By              :      Signal
Multiplier             :      1.0000
Dilution               :      1.0000
Use Multiplier & Dilution Factor with ISTDs


Signal 1: VWD1 A, Wavelength=250 nm

Peak RetTime Type  Width     Area      Height     Area
  #   [min]        [min]   [mAU*s]     [mAU]        %
----|-------|----|-------|----------|----------|--------|
   1  29.551 BV     1.3735 3056.24243   33.76105  51.2296
   2  33.000 VB     1.7665 2909.53345   24.32852  48.7704
```

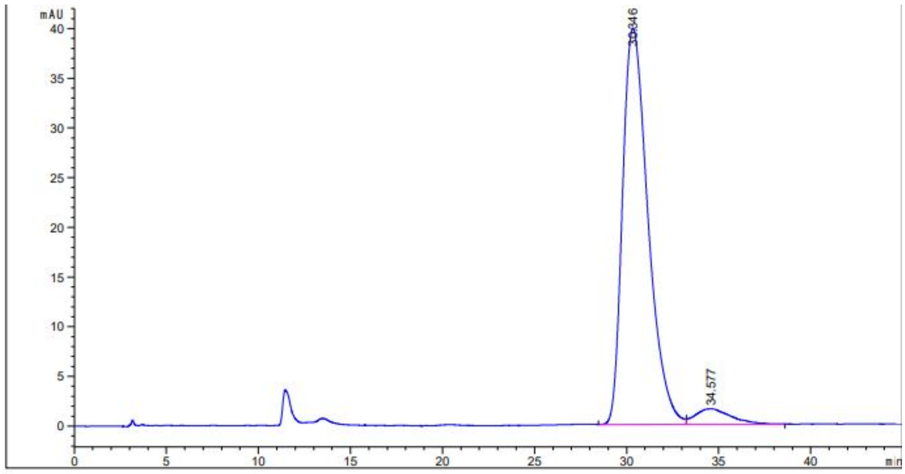


```
=====================================================================
                         Area Percent Report
=====================================================================

Sorted By             :      Signal
Multiplier            :      1.0000
Dilution              :      1.0000
Use Multiplier & Dilution Factor with ISTDs


Signal 1: VWD1 A, Wavelength=250 nm

Peak RetTime Type  Width     Area      Height     Area
  #   [min]        [min]   [mAU*s]     [mAU]       %
----|-------|----|-------|----------|----------|--------|
   1  30.346 BV    1.4894 3834.53394   39.85372  94.8562
   2  34.577 VB    1.5780  207.93488    1.55450   5.1438
```

(*R*)-3-Bromo-*N*-(2-chloro-2-(quinolin-2-yl)ethyl)aniline (21)

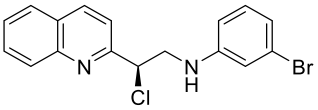

Chemical Formula: $C_{17}H_{14}BrClN_2$
Exact Mass: 360.0029
Molecular Weight: 361.6670

Prepared according to General Procedure A using 2-(1-chlorovinyl)quinoline **1** (47.4 mg, 0.25 mmol, 1.0 equiv.), 3-bromoaniline (27 µL, 0.25 mmol, 1.0 equiv.), (*S*)-TCYP (12.4 mg, 12.5 µmol, 5 mol%), and CPME (500 µL, 0.5 M) for 39 h. The reaction mixture was subjected to the purification outlined in General Procedure A (silica, EtOAc:petroleum ether, 5:95 to 10:90) to afford the desired product as a pale yellow solid (68.3 mg, 76%). The enantiomeric ratio (95:5) was determined by chiral HPLC in comparison with the racemate.

ν_{max} (film): 1595, 1503, 1481, 829, 762 cm^{-1}.

^{1}H NMR (500 MHz, $CDCl_3$): δ 8.20 (d, *J* = 8.5 Hz, 1H), 8.12 (d, *J* = 8.5 Hz, 1H), 7.84 (d, *J* = 8.1 Hz, 1H), 7.77 (ddd, *J* = 8.4, 7.0, 1.4 Hz, 1H), 7.69 – 7.51 (m, 2H), 7.00 (t, *J* = 7.9 Hz, 1H), 6.89 – 6.75 (m, 2H), 6.66 – 6.48 (m, 1H), 5.33 (app. t, *J* = 6.7 Hz, 1H), 4.45 (s, 1H), 4.10 (dd, *J* = 14.0, 6.5 Hz, 1H), 3.86 (dd, *J* = 14.0, 6.8 Hz, 1H).

^{13}C NMR (126 MHz, $CDCl_3$): δ 157.8, 148.5, 147.3, 137.6, 130.7, 130.2, 129.5, 127.7, 127.3, 123.5, 121.0, 120.5, 116.0, 112.0, 60.8, 49.2.

HRMS (ESI): exact mass calculated for $[M+H]^+$ ($C_{17}H_{15}BrClN_2$) requires *m/z* 361.0102, found *m/z* 361.0102.

HPLC analysis using a chiral column: Chiralcel® OJ-H 5 µm column, 1.0 mL/min, 50:50 hexane:*iso*propanol, 250 nm, t_{major} = 26.3 min, t_{minor} = 50.3 min.

$[\alpha]_D^{23}$ = −56.4° (c = 1.0, $CHCl_3$).

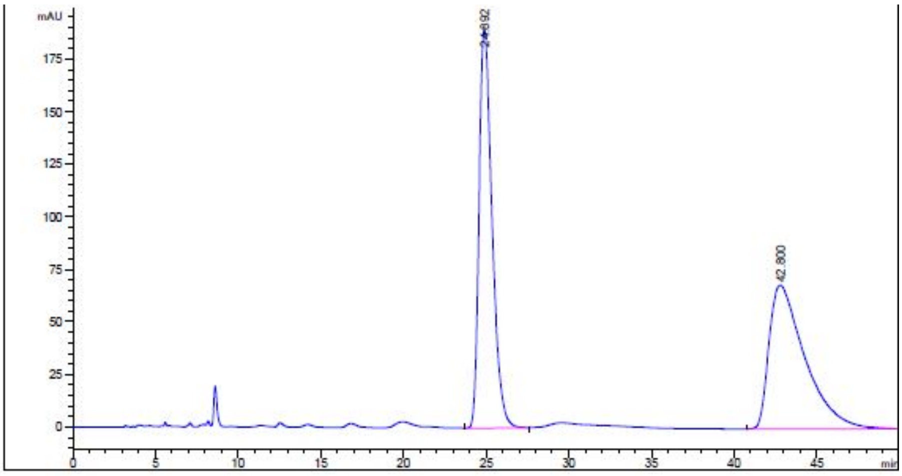

===

Area Percent Report

===

Sorted By : Signal
Multiplier : 1.0000
Dilution : 1.0000
Use Multiplier & Dilution Factor with ISTDs

Signal 1: VWD1 A, Wavelength=250 nm

Peak #	RetTime [min]	Type	Width [min]	Area [mAU*s]	Height [mAU]	Area %
1	24.892	BB	0.8401	1.03828e4	189.93922	50.1372
2	42.800	BBA	2.1648	1.03260e4	68.44814	49.8628

Totals : 2.07088e4 258.38737

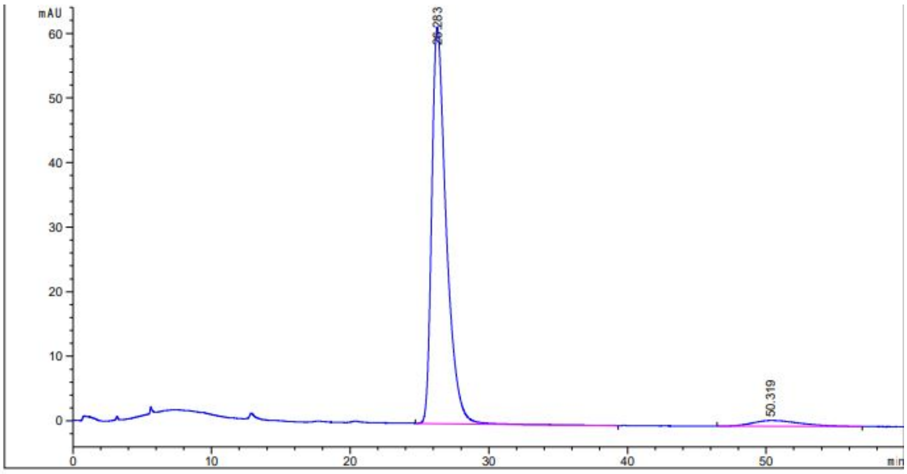

===

Area Percent Report

===

Sorted By : Signal
Multiplier : 1.0000
Dilution : 1.0000
Use Multiplier & Dilution Factor with ISTDs

Signal 1: VWD1 A, Wavelength=250 nm

Peak #	RetTime [min]	Type	Width [min]	Area [mAU*s]	Height [mAU]	Area %
1	26.283	VB	1.0889	4471.84326	61.36694	95.3081
2	50.319	BB	2.8918	220.14313	8.97178e-1	4.6919

(*R*)-*N*-(2-Chloro-2-(quinolin-2-yl)ethyl)-3-fluoro-4-methylaniline (22)

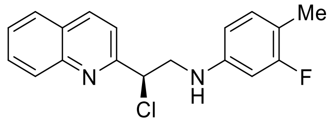

Chemical Formula: $C_{18}H_{16}ClFN_2$
Exact Mass: 314.0986
Molecular Weight: 314.7884

Prepared according to General Procedure A using 2-(1-chlorovinyl)quinoline **1** (47.4 mg, 0.25 mmol, 1.0 equiv.), 3-fluoro-4-methylaniline (31.3 mg, 0.25 mmol, 1.0 equiv.), (*S*)-TCYP (12.4 mg, 12.5 µmol, 5 mol%), and 2-MeTHF (500 µL, 0.5 M) for 39 h. The reaction mixture was subjected to the purification outlined in General Procedure A (silica, EtOAc:petroleum ether, 5:95 to 10:90) to afford the desired product as a pale yellow solid (72.0 mg, 91%). The enantiomeric ratio (95:5) was determined by chiral HPLC in comparison with the racemate.

ν_{max} (film): 1632, 1516, 1175, 775 cm^{-1}.

^{1}H NMR (500 MHz, $CDCl_3$): 8.19 (d, *J* = 8.5 Hz, 1H), 8.12 (d, *J* = 8.5 Hz, 1H), 7.83 (d, *J* = 8.1 Hz, 1H), 7.76 (t, *J* = 7.6 Hz, 1H), 7.59 (t, *J* = 8.6 Hz, 2H), 6.95 (t, *J* = 8.6 Hz, 1H), 6.42 – 6.31 (m, 2H), 5.34 (t, *J* = 6.7 Hz, 1H), 4.33 (s, 1H), 4.08 (dd, *J* = 14.0, 6.4 Hz, 1H), 3.84 (dd, *J* = 14.0, 7.0 Hz, 1H), 2.15 (s, 3H).

^{13}C NMR (126 MHz, $CDCl_3$): 162.22 (d, *J* = 242.6 Hz), 157.94, 147.40, 146.81 (d, *J* = 10.4 Hz), 137.47, 131.94 (d, *J* = 7.0 Hz), 130.13, 129.59, 127.72, 127.26, 120.46, 113.76 (d, *J* = 17.7 Hz), 109.17 (d, *J* = 2.5 Hz), 100.41 (d, *J* = 26.2 Hz), 61.00, 49.74, 13.76 (d, *J* = 3.0 Hz).

^{19}F NMR (470 MHz, $CDCl_3$): −116.53.

HRMS (ESI): exact mass calculated for $[M+H]^+$ ($C_{18}H_{16}ClFN_2$) requires *m/z* 315.1059, found *m/z* 315.1059.

HPLC analysis using a chiral column: CHIRAL ART Amylose-C 5 µm column, 1.0 mL/min, 50:50 hexane:*iso*propanol, 250 nm, t_{major} = 5.1 min, t_{minor} = 5.4 min.

$[\alpha]_D^{23}$ = −74.5° (*c* = 1.0, $CHCl_3$).

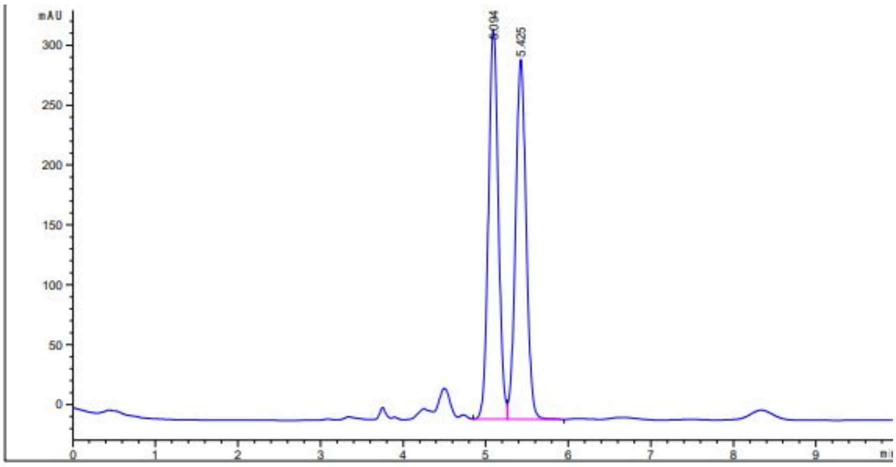

```
=====================================================================
                        Area Percent Report
=====================================================================

Sorted By             :      Signal
Multiplier            :      1.0000
Dilution              :      1.0000
Use Multiplier & Dilution Factor with ISTDs


Signal 1: VWD1 A, Wavelength=250 nm

Peak RetTime Type  Width     Area      Height     Area
  #   [min]        [min]   [mAU*s]     [mAU]       %
----|-------|----|-------|----------|----------|--------|
   1   5.094 BV    0.1294 2716.00220  325.47369  50.3868
   2   5.425 VB    0.1374 2674.30469  300.14532  49.6132
```

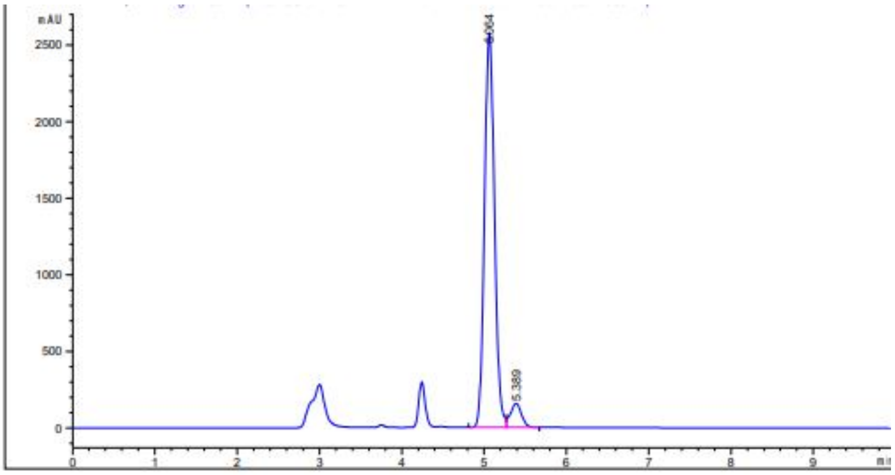

```
=====================================================================
                        Area Percent Report
=====================================================================

Sorted By             :      Signal
Multiplier            :      1.0000
Dilution              :      1.0000
Use Multiplier & Dilution Factor with ISTDs


Signal 1: VWD1 A, Wavelength=250 nm

Peak RetTime Type  Width     Area      Height     Area
  #   [min]        [min]   [mAU*s]     [mAU]       %
----|-------|----|-------|----------|----------|--------|
   1   5.064 BV    0.1320 2.17404e4  2574.05762  93.7576
   2   5.389 VB    0.1422 1447.49036  155.28909   6.2424
```

(*R*)-*N*-(2-Chloro-2-(quinolin-2-yl)ethyl)-6-fluoropyridin-3-amine (23)

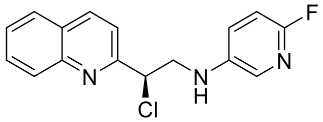

Chemical Formula: $C_{16}H_{13}ClFN_3$
Exact Mass: 301.0782
Molecular Weight: 301.7494

Prepared according to General Procedure A using 2-(1-chlorovinyl)quinoline **1** (47.4 mg, 0.25 mmol, 1.0 equiv.), 5-amino-2-fluoropyridine (28.0 mg, 0.25 mmol, 1.0 equiv.), (*S*)-TCYP (12.4 mg, 12.5 µmol, 5 mol%), and CPME (500 µL, 0.5 M). The reaction mixture was subjected to the purification outlined in General Procedure A (silica, EtOAc:petroleum ether, 5:95 to 10:90) to afford the desired product as a pale yellow solid (57.7 mg, 76%). The enantiomeric ratio (93:7) was determined by chiral HPLC in comparison with the racemate.

ν_{max} (film): 3308, 1602, 1497, 772 cm^{-1}.

^{1}H NMR (500 MHz, $CDCl_3$): δ 8.24 (d, *J* = 8.5 Hz, 1H), 8.13 (d, *J* = 8.4 Hz, 1H), 7.84 (d, *J* = 8.1 Hz, 1H), 7.77 (t, *J* = 7.7 Hz, 1H), 7.65 (d, *J* = 8.5 Hz, 1H), 7.62 – 7.55 (m, 2H), 7.13 – 7.06 (m, 1H), 6.71 (dd, *J* = 8.7, 2.7 Hz, 1H), 5.39 (app. t, *J* = 6.4 Hz, 1H), 4.05 (dd, *J* = 13.9, 6.4 Hz, 1H), 3.87 (dd, *J* = 13.9, 6.8 Hz, 1H). ^{1}H resonance for NH not observed

^{13}C NMR (126 MHz, $CDCl_3$): δ 157.5, 157.1 (d, ^{1}J = 230.0 Hz), 146.6, 141.5 (d, ^{4}J = 3.7 Hz), 138.2, 131.5 (d, ^{3}J = 14.8 Hz), 130.6, 128.9, 127.8, 127.7, 127.6, 126.0 (d, ^{3}J = 6.9 Hz), 120.3, 109.5 (d, ^{2}J = 39.5 Hz), 60.5, 50.1.

^{19}F NMR (470 MHz, $CDCl_3$): δ −81.45.

HRMS (ESI): exact mass calculated for $[M+H]^+$ ($C_{16}H_{13}ClFN_3$) requires *m/z* 302.0855, found *m/z* 302.0855.

HPLC analysis using a chiral column: Chiralcel® OJ-H 5 µm column, 2.0 mL/min, 50:50 hexane:*iso*propanol, 250 nm, t_{major} = 10.2 min, t_{minor} = 13.6 min.

$[\alpha]_D^{23}$ = −70.4° (*c* = 1.0, $CHCl_3$).

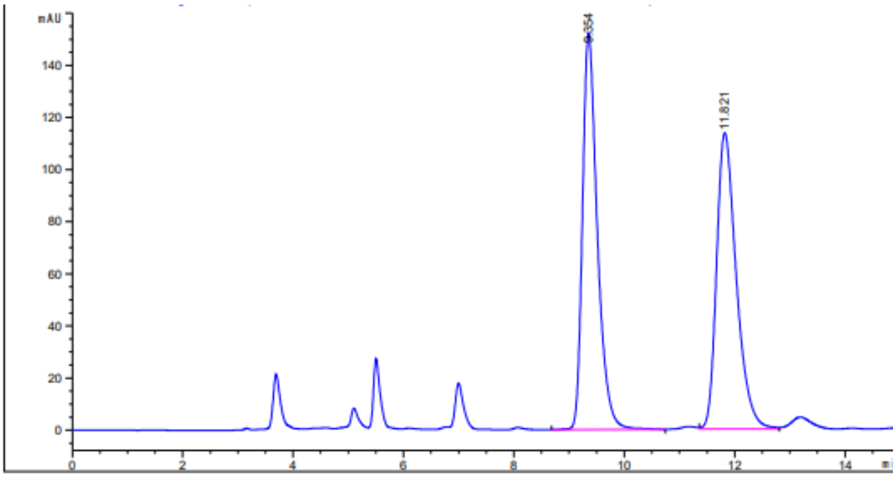

===

Area Percent Report

===

Sorted By : Signal
Multiplier : 1.0000
Dilution : 1.0000
Use Multiplier & Dilution Factor with ISTDs

Signal 1: VWD1 A, Wavelength=250 nm

Peak #	RetTime [min]	Type	Width [min]	Area [mAU*s]	Height [mAU]	Area %
1	9.354	BB	0.2801	2838.99683	152.30174	50.1613
2	11.821	VV	0.3768	2820.74316	113.70979	49.8387

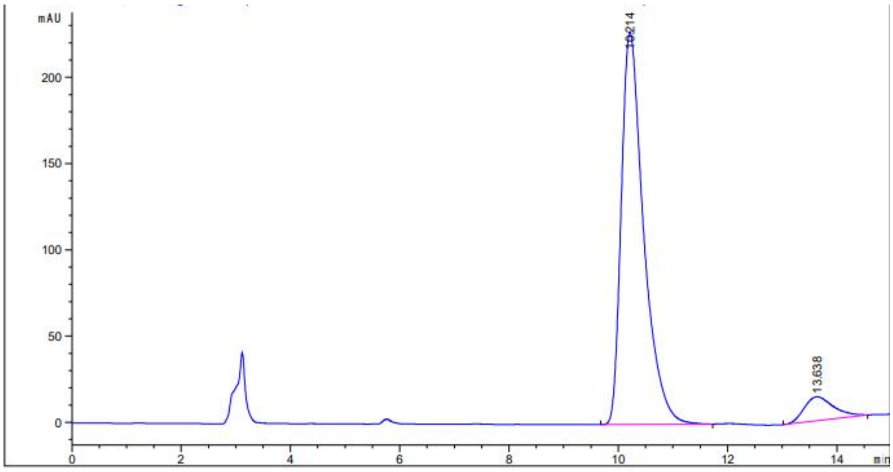

===

Area Percent Report

===

Sorted By : Signal
Multiplier : 1.0000
Dilution : 1.0000
Use Multiplier & Dilution Factor with ISTDs

Signal 1: VWD1 A, Wavelength=250 nm

Peak #	RetTime [min]	Type	Width [min]	Area [mAU*s]	Height [mAU]	Area %
1	10.214	BB	0.4262	6467.63379	227.57758	92.5909
2	13.638	BB	0.5680	517.53912	13.86512	7.4091

(*R*)-*N*-(2-Chloro-2-(quinolin-2-yl)ethyl)benzo[*b*]thiophen-5-amine (24)

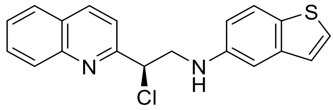

Chemical Formula: $C_{19}H_{15}ClN_2S$
Exact Mass: 338.0644
Molecular Weight: 338.8530

Prepared according to General Procedure A using 2-(1-chlorovinyl)quinoline **1** (47.4 mg, 0.25 mmol, 1.0 equiv.), 5-aminobenzothiophene (37.3 mg, 0.25 mmol, 1.0 equiv.), (*S*)-TCYP (12.4 mg, 12.5 µmol, 5 mol%), and 2-MeTHF (500 µL, 0.5 M). The reaction mixture was subjected to the purification outlined in General Procedure A (silica, EtOAc:petroleum ether, 5:95 to 10:90) to afford the desired product as a pale yellow solid (69.3 mg, 82%). The enantiomeric ratio (94:6) was determined by chiral HPLC in comparison with the racemate.

ν_{max} (film): 1604, 1506, 772 cm^{-1}.

^1H NMR (500 MHz, $CDCl_3$): δ 8.19 (d, *J* = 8.5 Hz, 1H), 8.14 (d, *J* = 8.3 Hz, 1H), 7.83 (dd, *J* = 8.1, 1.2 Hz, 1H), 7.77 (ddd, *J* = 8.4, 6.9, 1.4 Hz, 1H), 7.67 – 7.54 (m, 3H), 7.38 (d, *J* = 5.4 Hz, 1H), 7.16 (dd, *J* = 5.4, 0.6 Hz, 1H), 7.09 (d, *J* = 2.3 Hz, 1H), 6.82 – 6.73 (m, 1H), 5.42 (app. t, *J* = 6.7 Hz, 1H), 4.18 (dd, *J* = 14.0, 6.5 Hz, 1H), 3.94 (dd, *J* = 14.0, 7.0 Hz, 1H). ^1H resonance for NH not observed.

^{13}C NMR (126 MHz, $CDCl_3$): δ 158.1, 147.4, 144.6, 141.1, 137.5, 130.2, 130.1, 129.6, 127.7, 127.3, 127.2, 123.4, 123.2, 120.5, 114.5, 105.9, 61.1, 50.2.

HRMS (ESI): exact mass calculated for $[M+H]^+$ ($C_{19}H_{16}ClN_2S$) requires *m/z* 339.0717, found *m/z* 339.0717.

HPLC analysis using a chiral column: Chiralcel® OJ-H 5 µm column, 2.0 mL/min, 50:50 hexane:*iso*propanol, 250 nm, t_{major} = 57.2 min, t_{minor} = 42.4 min.

$[\alpha]_D^{23}$ = −119° (*c* = 1.0, $CHCl_3$).

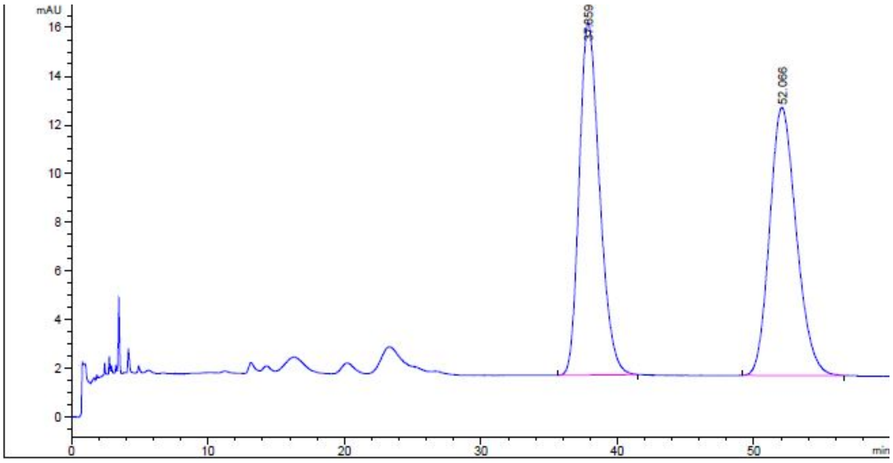

==
Area Percent Report
==

Sorted By : Signal
Multiplier : 1.0000
Dilution : 1.0000
Use Multiplier & Dilution Factor with ISTDs

Signal 1: VWD1 A, Wavelength=250 nm

Peak #	RetTime [min]	Type	Width [min]	Area [mAU*s]	Height [mAU]	Area %
1	37.859	BB	1.5821	1498.89111	14.44210	49.9608
2	52.066	BB	1.8824	1501.24329	11.00696	50.0392

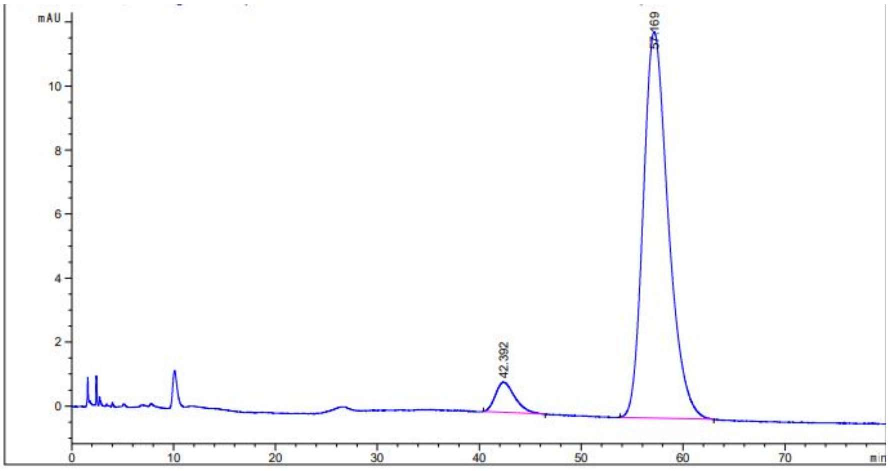

==
Area Percent Report
==

Sorted By : Signal
Multiplier : 1.0000
Dilution : 1.0000
Use Multiplier & Dilution Factor with ISTDs

Signal 1: VWD1 A, Wavelength=250 nm

Peak #	RetTime [min]	Type	Width [min]	Area [mAU*s]	Height [mAU]	Area %
1	42.392	BB	1.5583	124.08217	9.41317e-1	5.6936
2	57.169	BB	2.0809	2055.23291	12.07180	94.3064

(R)-2-(1-Chloro-2-(indolin-1-yl)ethyl)quinoline (25)

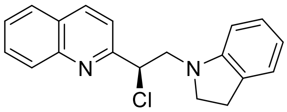

Chemical Formula: $C_{19}H_{17}ClN_2$
Exact Mass: 308.11
Molecular Weight: 308.81

Prepared according to General Procedure A using 2-(1-chlorovinyl)quinoline **1** (47.4 mg, 0.25 mmol, 1.0 equiv.), indoline (28 µL, 0.25 mmol, 1.0 equiv.), (*S*)-TCYP (12.4 mg, 12.5 µmol, 5 mol%), and CPME (500 µL, 0.5 M) for 15 h. The reaction mixture was subjected to the purification outlined in General Procedure A (silica, EtOAc:petroleum ether, 5:95 to 10:90) to afford the desired product as a pale yellow solid (65.0 mg, 84%). The enantiomeric ratio (93:7) was determined by chiral HPLC in comparison with the racemate.

ν_{max} (film): 1607, 1489, 745 cm^{-1}.

^{1}H NMR (500 MHz, $CDCl_3$): δ 8.20 (d, *J* = 8.5 Hz, 1H), 8.15 (d, *J* = 8.5 Hz, 1H), 7.83 (d, *J* = 8.1 Hz, 1H), 7.76 (ddd, *J* = 8.4, 7.0, 1.3 Hz, 1H), 7.66 (d, *J* = 8.5 Hz, 1H), 7.61 – 7.53 (m, 1H), 7.06 (t, *J* = 7.8 Hz, 2H), 6.66 (t, *J* = 7.3 Hz, 1H), 6.58 (d, *J* = 7.7 Hz, 1H), 5.38 (app. t, *J* = 6.9 Hz, 1H), 4.05 (dd, *J* = 14.4, 7.1 Hz, 1H), 3.84 (dd, *J* = 14.4, 6.7 Hz, 1H), 3.49 (app. q, *J* = 8.5 Hz, 1H), 3.39 (app. q, *J* = 8.5 Hz, 1H), 2.93 (app. t, *J* = 8.5 Hz, 2H).

^{13}C NMR (126 MHz, $CDCl_3$): δ 158.5, 151.9, 147.4, 137.3, 130.0, 129.5, 129.4, 127.8, 127.7, 127.4, 127.1, 124.6, 120.2, 117.8, 106.6, 61.4, 56.6, 54.2, 28.7.

HRMS (ESI): exact mass calculated for $[M+H]^+$ ($C_{19}H_{18}ClN_2$) requires *m/z* 309.1153, found *m/z* 309.1152.

$[\alpha]_D^{23}$ = +30.1° (*c* = 1.0, $CHCl_3$).

HPLC analysis using a chiral column: Chiralcel® OJ-H 5 µm column, 1.0 mL/min, 50:50 hexane:*iso*propanol, 250 nm, t_{major} = 13.2 min, t_{minor} = 15.4 min.

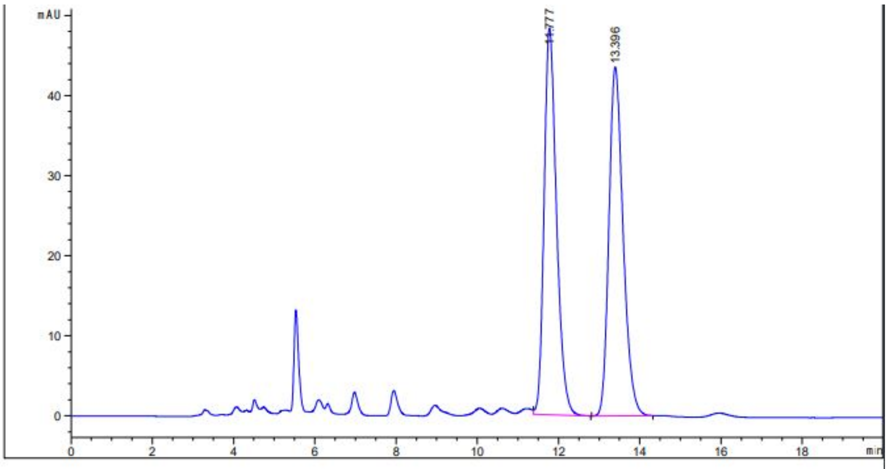

Area Percent Report

Sorted By : Signal
Multiplier : 1.0000
Dilution : 1.0000
Use Multiplier & Dilution Factor with ISTDs

Signal 1: VWD1 A, Wavelength=250 nm

Peak #	RetTime [min]	Type	Width [min]	Area [mAU*s]	Height [mAU]	Area %
1	11.777	VB	0.3233	1024.08643	48.26287	49.2142
2	13.396	BB	0.3719	1056.78955	43.54992	50.7858

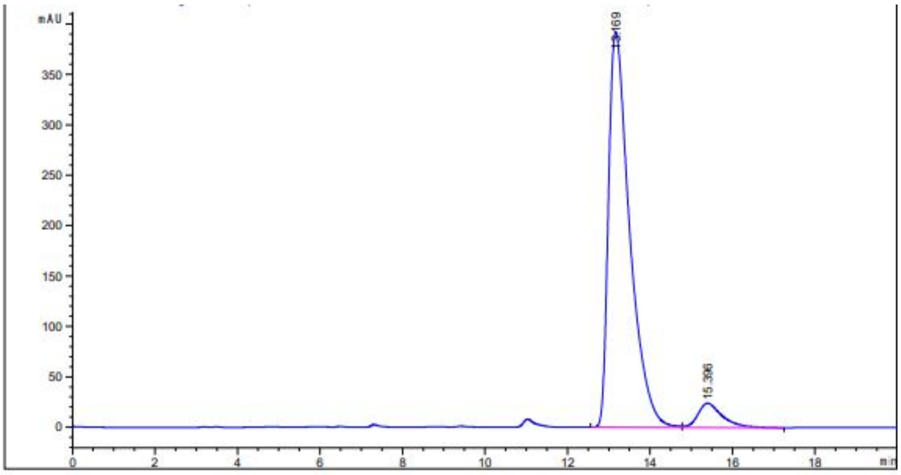


```
=====================================================================
                         Area Percent Report
=====================================================================

Sorted By             :      Signal
Multiplier            :      1.0000
Dilution              :      1.0000
Use Multiplier & Dilution Factor with ISTDs


Signal 1: VWD1 A, Wavelength=250 nm

Peak RetTime Type  Width     Area      Height     Area
  #   [min]        [min]   [mAU*s]     [mAU]        %
----|-------|----|-------|----------|----------|--------|
   1  13.169 BV    0.5337 1.39850e4  392.39307  93.3601
   2  15.396 VB    0.6103  994.63593   24.06757   6.6399
```

(*R*)-*N*-(2-Chloro-2-(6-fluoroquinolin-2-yl)ethyl)aniline (26)

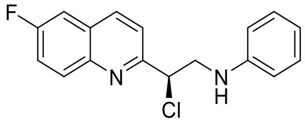

Chemical Formula: $C_{17}H_{14}ClFN_2$
Exact Mass: 300.0830
Molecular Weight: 300.7614

Prepared according to General Procedure A using 2-(1-chlorovinyl)-6-fluoroquinoline **S35** (51.9 mg, 0.25 mmol, 1.0 equiv.), aniline (23 µL, 0.25 mmol, 1.0 equiv.), (*S*)-TCYP (12.4 mg, 12.5 µmol, 5 mol%), and 2-MeTHF (500 µL, 0.5 M). The reaction mixture was subjected to the purification outlined in General Procedure A (silica, EtOAc:petroleum ether, 5:95 to 10:90) to afford the desired product as a pale yellow solid (21.4 mg, 28%). The enantiomeric ratio (94:6) was determined by chiral HPLC in comparison with the racemate.

ν_{max} (film): 1600, 1504, 1234, 750 cm^{-1}.

^{1}H NMR (500 MHz, $CDCl_3$): δ 8.16 – 8.09 (m, 2H), 7.61 (d, *J* = 8.5 Hz, 1H), 7.56 – 7.49 (m, 1H), 7.45 (dd, *J* = 8.7, 2.8 Hz, 1H), 7.19 (t, *J* = 7.9 Hz, 2H), 6.75 (t, *J* = 7.3 Hz, 1H), 6.68 (d, *J* = 7.7 Hz, 2H), 5.34 (t, *J* = 6.7 Hz, 1H), 4.33 (s, 1H), 4.12 (dd, *J* = 14.1, 6.4 Hz, 1H), 3.87 (dd, *J* = 14.1, 7.0 Hz, 1H).

^{13}C NMR (126 MHz, $CDCl_3$): δ 160.9 (d, ^1J = 249.2 Hz), 157.5, 147.1, 144.5, 136.8 (d, ^4J = 5.3 Hz), 132.1 (d, ^3J = 9.1 Hz), 129.6, 128.4 (d, ^3J = 10.2 Hz), 121.3, 120.4 (d, ^2J = 25.8 Hz), 118.4, 113.5, 110.8 (d, ^2J = 21.8 Hz), 61.0, 49.5.

^{19}F NMR (376 MHz, $CDCl_3$): δ −112.37.

HRMS (ESI): exact mass calculated for $[M+H]^+$ ($C_{17}H_{15}ClFN_2$) requires *m/z* 301.0908, found *m/z* 301.0898.

HPLC analysis using a chiral column: Chiralcel® OJ-H 5 μm column, 1.0 mL/min, 50:50 hexane:*iso*propanol, 250 nm, t_{major} = 17.4 min, t_{minor} = 27.6 min.

$[\alpha]_D^{23}$ = −8.4° (*c* = 1.0, $CHCl_3$).

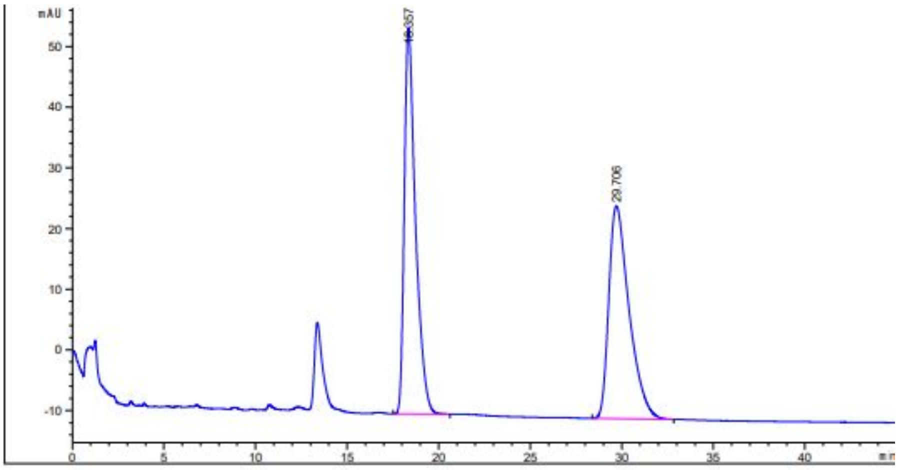

Area Percent Report

Sorted By : Signal
Multiplier : 1.0000
Dilution : 1.0000
Use Multiplier & Dilution Factor with ISTDs

Signal 1: VWD1 A, Wavelength=250 nm

Peak #	RetTime [min]	Type	Width [min]	Area [mAU*s]	Height [mAU]	Area %
1	18.357	BB	0.6276	2711.90625	63.71347	50.1083
2	29.706	BB	1.1318	2700.18848	35.10372	49.8917

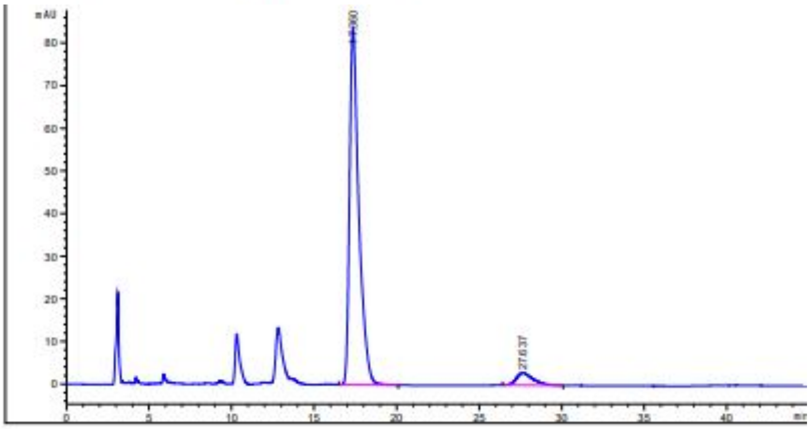

Area Percent Report

Sorted By : Signal
Multiplier : 1.0000
Dilution : 1.0000
Use Multiplier & Dilution Factor with ISTDs

Signal 1: VWD1 A, Wavelength=250 nm

Peak #	RetTime [min]	Type	Width [min]	Area [mAU*s]	Height [mAU]	Area %
1	17.360	MM	0.5741	3275.51611	83.77097	94.1416
2	27.637	MM	0.8746	203.83316	2.89224	5.8584

(*R*)-*N*-(2-Chloro-2-(5-chloro-6-methoxyquinolin-2-yl)ethyl)aniline (27)

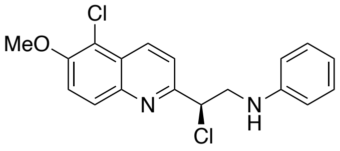

Chemical Formula: $C_{18}H_{16}Cl_2N_2O$
Exact Mass: 346.0640
Molecular Weight: 347.2390

Prepared according to General Procedure A using 5-chloro-2-(1-chlorovinyl)-6-methoxyquinoline **S36** (63.5 mg, 0.25 mmol, 1.0 equiv.), (*S*)-TCYP (12.4 mg, 0.0125 mmol, 5 mol%), CPME (0.5 mL) and aniline (23.3 mg, 0.25 mmol, 1.0 equiv.) at -20 °C. The reaction mixture was subjected to the purification outlined in General Procedure A (silica, EtOAc:hexane, 20:80) to afford the desired product as a white solid (71.2 mg, 82 %). The enantiomeric ratio (97:3) was determined by chiral HPLC in comparison with the racemate.

ν_{max}(film): 3337, 2868, 1593, 1497, 1069, 764 cm^{-1}.

^{1}H NMR (500 MHz, CDCl$_3$): δ 8.55 (dd, *J* = 8.9, 0.8 Hz, 1H), 8.06 (dd, *J* = 9.3, 0.8 Hz, 1H), 7.65 (d, *J* = 8.8 Hz, 1H), 7.55 (d, *J* = 9.3 Hz, 1H), 7.22 – 7.14 (m, 3H), 6.74 (tt, *J* = 7.3, 1.1 Hz, 1H), 6.70 – 6.64 (m, 2H), 5.34 (t, *J* = 6.7 Hz, 1H), 4.13 – 4.06 (m, 4H), 3.86 (dd, *J* = 14.1, 7.1 Hz, 1H).

^{13}C NMR (126 MHz, CDCl$_3$): δ 156.3, 153.2, 146.9, 142.8, 133.3, 129.4, 129.3, 126.7, 121.5, 118.2, 116.9, 115.9, 113.4, 60.7, 57.0, 49.4.

HRMS (ESI): exact mass calculated for [M+H]$^+$ ($C_{18}H_{17}Cl_2N_2O$) requires *m/z* 347.0694, found *m/z* 347.0704.

HPLC analysis using a chiral column: Chiralpak-IA 5 μm column, 1.0 mL/min, 50:50 hexane:*iso*propanol, 250 nm, t_{major} = 6.2 min, t_{minor} = 7.1 min.

$[\alpha]_D^{23}$ = −53.4° (*c* 0.5, CHCl$_3$).

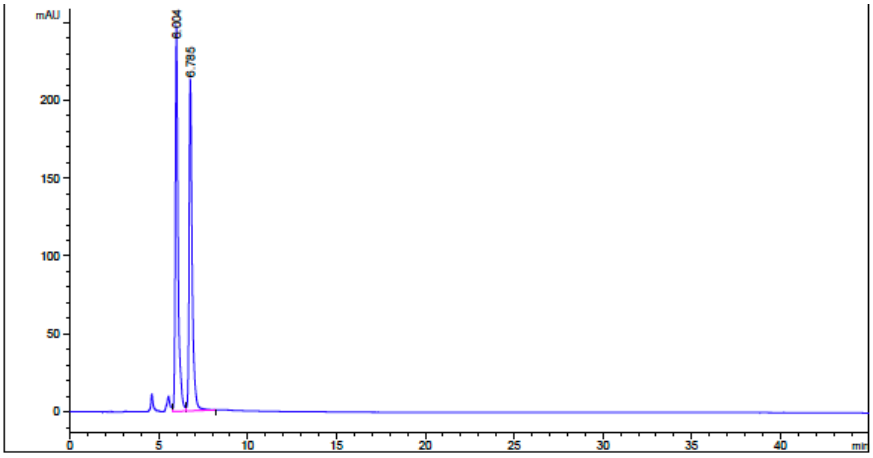

Area Percent Report

Sorted By : Signal
Multiplier : 1.0000
Dilution : 1.0000
Use Multiplier & Dilution Factor with ISTDs

Signal 1: VWD1 A, Wavelength=254 nm

Peak #	RetTime [min]	Type	Width [min]	Area [mAU*s]	Height [mAU]	Area %
1	6.004	VV	0.1553	2599.69702	245.99652	50.0643
2	6.785	VB	0.1804	2593.02075	212.21930	49.9357

Totals : 5192.71777 458.21582

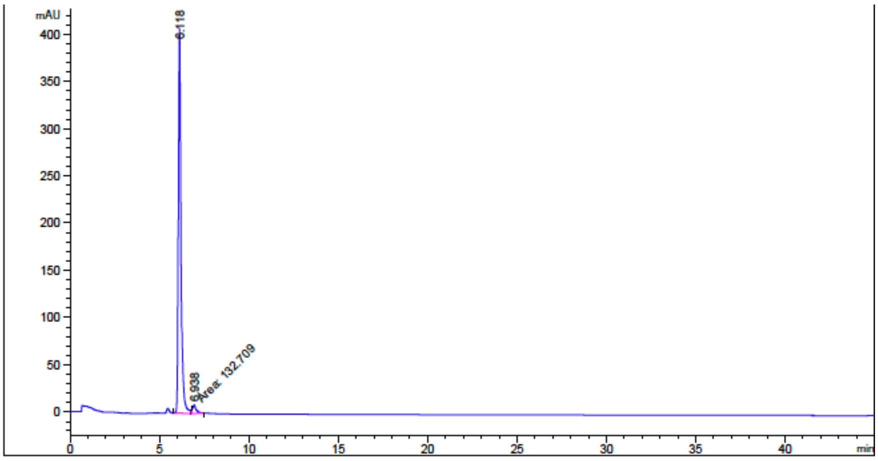

Area Percent Report

Sorted By : Signal
Multiplier : 1.0000
Dilution : 1.0000
Use Multiplier & Dilution Factor with ISTDs

Signal 1: VWD1 A, Wavelength=254 nm

Peak #	RetTime [min]	Type	Width [min]	Area [mAU*s]	Height [mAU]	Area %
1	6.118	BV	0.1541	4211.90527	407.54541	96.9454
2	6.938	MM	0.2559	132.70905	8.64335	3.0546

Totals : 4344.61432 416.18876

(*R*)-*N*-(2-Chloro-2-(phenanthridin-6-yl)ethyl)aniline (28)

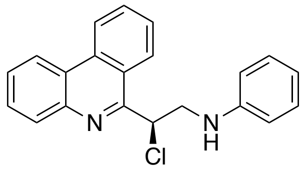

Chemical Formula: $C_{21}H_{17}ClN_2$
Exact Mass: 332.1080
Molecular Weight: 332.8310

Prepared according to General Procedure A using 6-(1-chlorovinyl)phenanthridine **S44** (36.0 mg, 0.15 mmol, 1.0 equiv.), (*S*)-TCYP (7.5 mg, 0.0075 mmol, 5 mol %), 2-MeTHF (0.3 mL) and aniline (13.7 mg, 0.15 mmol, 1.0 equiv.) at rt for 4 days. The reaction mixture was subjected to the purification outlined in General Procedure A (silica, EtOAc:hexane, 5:95) to afford the desired product as a white solid (32.1 mg, 64 %). The enantiomeric ratio (56:44) was determined by chiral HPLC in comparison with the racemate.

ν_{max} (film): 3397, 3053, 2851, 1601, 1508, 762, 723 cm^{-1}.

^{1}H NMR (500 MHz, $CDCl_3$) δ 8.67 (d, *J* = 8.3 Hz, 1H), 8.59 (dd, *J* = 8.1, 1.4 Hz, 1H), 8.32 (d, *J* = 8.2 Hz, 1H), 8.23 (dd, *J* = 8.1, 1.4 Hz, 1H), 7.86 (ddd, *J* = 8.3, 7.0, 1.3 Hz, 1H), 7.79 (ddd, *J* = 8.2, 7.0, 1.4 Hz, 1H), 7.72 (dddd, *J* = 8.3, 7.0, 4.6, 1.3 Hz, 2H), 7.23 – 7.16 (m, 2H), 6.77 – 6.70 (m, 3H), 5.92 (dd, *J* = 7.6, 5.9 Hz, 1H), 4.58 (dd, *J* = 14.3, 7.6 Hz, 1H), 4.21 (dd, *J* = 14.3, 5.9 Hz, 1H). ^{1}H resonance for NH not observed.

^{13}C NMR (126 MHz, $CDCl_3$) δ 156.0, 147.1, 142.9, 133.5, 130.8, 130.5, 129.5, 128.8, 127.8, 127.6, 125.7, 124.5, 124.2, 122.6, 122.0, 117.9, 113.1, 54.8, 47.1.

HRMS (ESI): exact mass calculated for $[M+H]^+$ ($C_{21}H_{18}ClN_2$) requires *m/z* 333.1159, found *m/z* 333.1146.

HPLC analysis using a chiral column: Chiralpak IA, 5 µm column, 1.0 mL/min, 50:50 hexane:*iso*propanol, 250 nm, t_{major} = 6.2 min, t_{minor} = 7.1 min.

$[\alpha]_D^{23}$ = −32.9° (*c* = 1.0, $CHCl_3$).

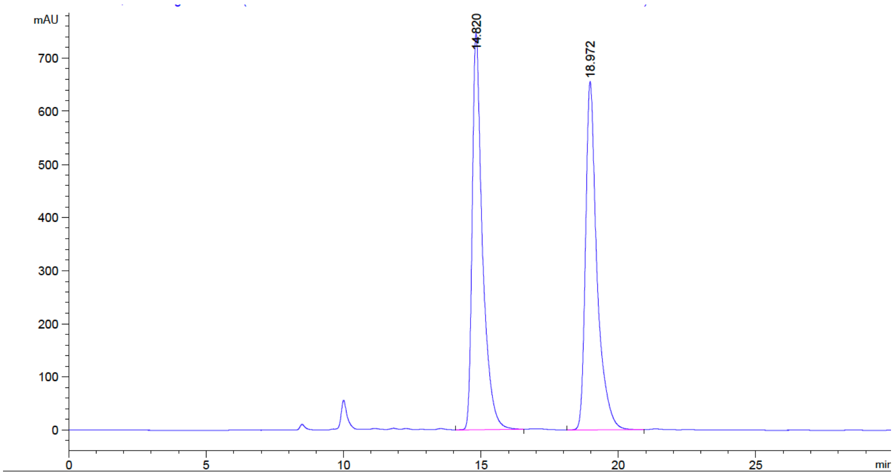

===

Area Percent Report

===

Sorted By : Signal

Multiplier : 1.0000

Dilution : 1.0000

Use Multiplier & Dilution Factor with ISTDs

Signal 1: VWD1 A, Wavelength=250 nm

Peak #	RetTime [min]	Type	Width [min]	Area [mAU*s]	Height [mAU]	Area %
1	14.820	BB	0.3772	1.92956e4	746.80817	50.4098
2	18.972	BB	0.4265	1.89819e4	655.74103	49.5902

Totals : 3.82776e4 1402.54919

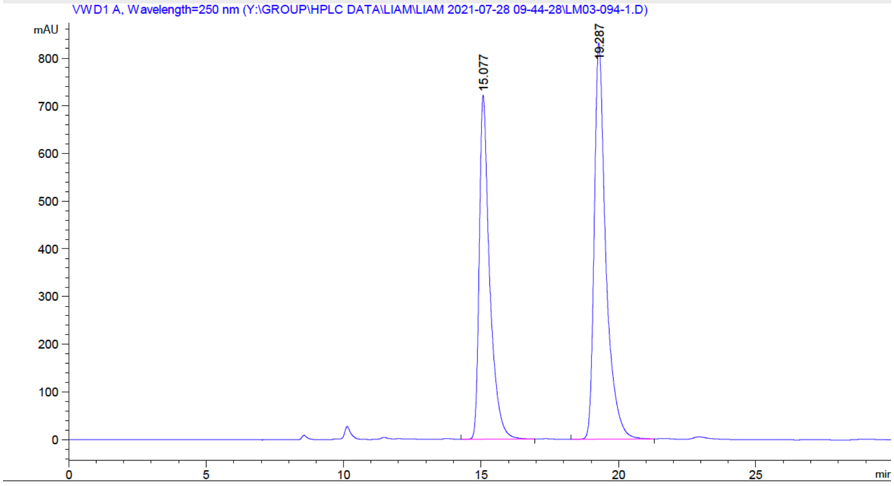

===

Area Percent Report

===

Sorted By : Signal

Multiplier : 1.0000

Dilution : 1.0000

Use Multiplier & Dilution Factor with ISTDs

Signal 1: VWD1 A, Wavelength=250 nm

Peak #	RetTime [min]	Type	Width [min]	Area [mAU*s]	Height [mAU]	Area %
1	15.077	BB	0.3838	1.89663e4	721.59155	43.6881
2	19.287	BB	0.4336	2.44468e4	830.75385	56.3119

Totals : 4.34131e4 1552.34540

(*R*)-*N*-(2-Chloro-2-(1-methyl-1*H*-benzo[*d*]imidazol-2-yl)ethyl)aniline (29)

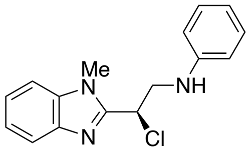

Chemical Formula: $C_{16}H_{16}ClN_3$
Exact Mass: 285.1033
Molecular Weight: 285.7750

Prepared according to General Procedure A using 2-(1-chlorovinyl)-1-methyl-1*H*-benzo[*d*]imidazole **S37** (48.2 mg, 0.25 mmol, 1.0 equiv.), (*S*)-TCYP (12.4 mg, 0.0125 mmol, 5 mol%), 2-MeTHF (0.5 mL) and aniline (23.3 mg, 0.25 mmol, 1.0 equiv.) at 0 °C. The reaction mixture was subjected to the purification outlined in General Procedure A (silica, EtOAc:hexane, 20:80) to afford the desired product as a white solid (49.0 mg, 69 %). The enantiomeric ratio (91:9) was determined by chiral HPLC in comparison with the racemate.

v_{max}(film): 3327, 3049, 2932, 1601, 750, cm^{-1}.

^{1}H NMR (500 MHz, $CDCl_3$): δ 7.85 – 7.81 (m, 1H), 7.40 – 7.30 (m, 3H), 7.22 – 7.16 (m, 2H), 6.74 (t, *J* = 7.3 Hz, 1H), 6.67 (d, *J* = 7.5 Hz, 2H), 5.25 (dd, *J* = 8.1, 5.8 Hz, 1H), 4.40 (dd, *J* = 14.5, 8.1 Hz, 1H), 4.09 (dd, *J* = 14.5, 5.8 Hz, 1H), 3.79 (s, 3H). ^{1}H resonance for NH not observed.

^{13}C NMR (126 MHz, $CDCl_3$): δ 150.4, 146.5, 141.7, 135.8, 129.5, 123.7, 122.8, 120.2, 118.2, 113.0, 109.6, 49.3, 47.4, 30.0.

HRMS (ESI): exact mass calculated for $[M+H]^+$ ($C_{16}H_{17}ClN_3$) requires *m/z* 286.1111, found *m/z* 286.1098.

HPLC analysis using a chiral column: Amylose-C 5 µm column, 0.5 mL/min, 90:10 hexane:*iso*propanol, 250 nm, t_{major} = 23.0 min, t_{minor} = 24.6 min.

$[\alpha]_D^{23}$ = −174° (*c* = 1.0, $CHCl_3$).

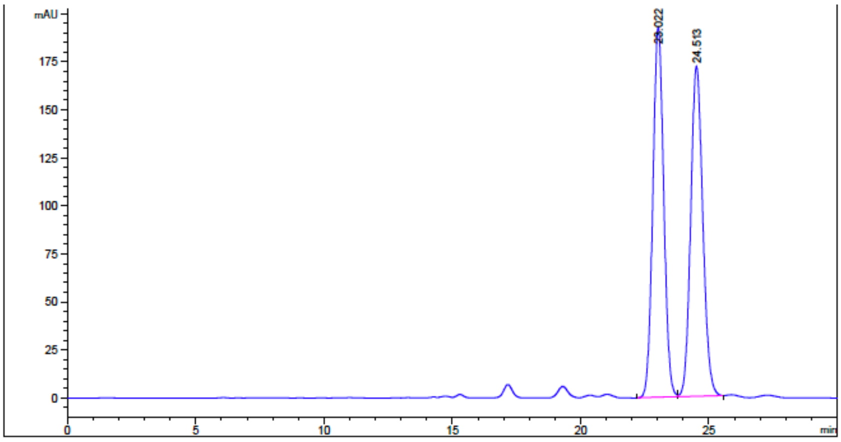

Area Percent Report

Sorted By : Signal
Multiplier : 1.0000
Dilution : 1.0000
Use Multiplier & Dilution Factor with ISTDs

Signal 1: VWD1 A, Wavelength=250 nm

Peak #	RetTime [min]	Type	Width [min]	Area [mAU*s]	Height [mAU]	Area %
1	23.022	BV	0.4589	5680.20850	192.68852	50.2212
2	24.513	VB	0.5060	5630.16895	171.86407	49.7788

Totals : 1.13104e4 364.55260

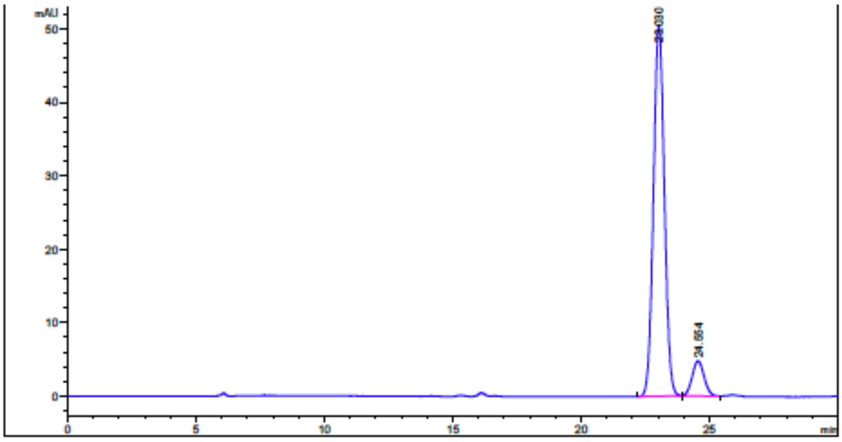

Area Percent Report

Sorted By : Signal
Multiplier : 1.0000
Dilution : 1.0000
Use Multiplier & Dilution Factor with ISTDs

Signal 1: VWD1 A, Wavelength=250 nm

Peak #	RetTime [min]	Type	Width [min]	Area [mAU*s]	Height [mAU]	Area %
1	23.030	BB	0.4598	1488.71692	50.37835	90.7420
2	24.554	BB	0.5031	151.88800	4.74409	9.2580

Totals : 1640.60492 55.12243

(*R*)-*N*-(2-Chloro-2-(5-phenylthiazol-2-yl)ethyl)aniline (30)

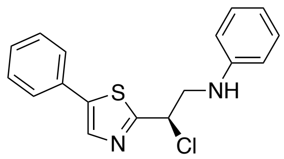

Chemical Formula: $C_{17}H_{15}ClN_2S$
Exact Mass: 314.0644
Molecular Weight: 314.8310

Prepared according to General Procedure A using 2-(1-chlorovinyl)-5-phenylthiazole **S38** (55.4 mg, 0.25 mmol, 1.0 equiv.), (*S*)-TCYP (12.4 mg, 0.0125 mmol, 5 mol%), 2-MeTHF (0.5 mL) and aniline (23.3 mg, 0.25 mmol, 1.0 equiv.) at rt. The reaction mixture was subjected to the purification outlined in General Procedure A (silica, EtOAc:hexane, 20:80) to afford the desired product as a white solid (26.0 mg, 33 %). The enantiomeric ratio (97:3) was determined by chiral HPLC in comparison with the racemate.

Product appears to be somewhat sensitive to silica and degrades on further attempts at purification.

ν_{max}(film): 3302, 2963, 2359, 1602, 1497, 1261, 750 cm^{-1}.

^{1}H NMR (500 MHz, CDCl$_3$): δ 7.91 (s, 1H), 7.59 – 7.51 (m, 2H), 7.44 – 7.39 (m, 2H), 7.39 – 7.34 (m, 1H), 7.23 (t, *J* = 8.1 Hz, 2H), 6.83 (t, *J* = 7.3 Hz, 1H), 6.78 (d, *J* = 7.9 Hz, 2H), 5.44 (t, *J* = 6.4 Hz, 1H), 4.07 (dd, *J* = 14.2, 6.1 Hz, 1H), 3.84 (dd, *J* = 14.1, 6.8 Hz, 1H).^{1}H resonance for NH not observed.

^{13}C NMR (176 MHz, CDCl$_3$): δ 167.2, 146.0, 141.3, 138.0, 130.8, 129.5, 129.2, 128.7, 126.9, 119.1, 113.9, 56.6, 50.7.

HRMS (ESI): exact mass calculated for $[M+H]^+$ ($C_{17}H_{16}ClN_2S$) requires *m/z* 315.0723, found *m/z* 315.0712.

HPLC analysis using a chiral column: Amylose-C 5 µm column, 1.0 mL/min, 50:50 hexane:*iso*propanol, 250 nm, t_{major} = 9.2 min, t_{minor} = 29.1 min.

$[\alpha]_D^{23}$= −48.6° (*c* = 1.0, CHCl$_3$).

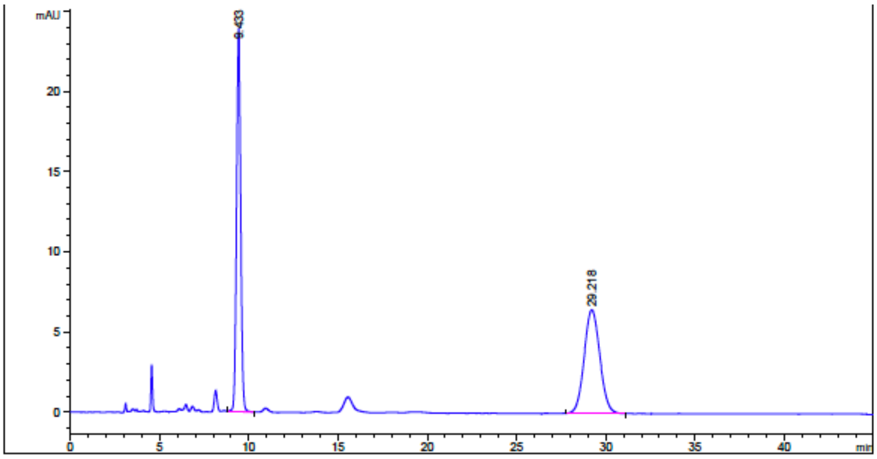


```
=====================================================================
                         Area Percent Report
=====================================================================

Sorted By             :      Signal
Multiplier            :      1.0000
Dilution              :      1.0000
Use Multiplier & Dilution Factor with ISTDs


Signal 1: VWD1 A, Wavelength=254 nm

Peak RetTime Type  Width     Area      Height     Area
  #   [min]        [min]   [mAU*s]     [mAU]       %
----|-------|----|-------|----------|----------|--------|
   1   9.433 BB    0.2589  399.49496   23.92410  50.3395
   2  29.218 BB    0.9222  394.10678    6.46600  49.6605

Totals :                   793.60175   30.39010
```

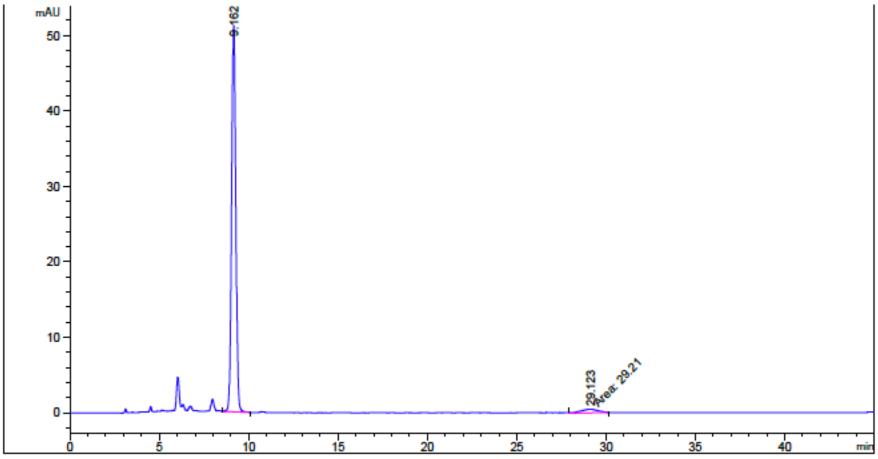


```
=====================================================================
                         Area Percent Report
=====================================================================

Sorted By             :      Signal
Multiplier            :      1.0000
Dilution              :      1.0000
Use Multiplier & Dilution Factor with ISTDs


Signal 1: VWD1 A, Wavelength=254 nm

Peak RetTime Type  Width     Area      Height     Area
  #   [min]        [min]   [mAU*s]     [mAU]       %
----|-------|----|-------|----------|----------|--------|
   1   9.162 BB    0.2563  843.84094   51.21504  96.6543
   2  29.123 MM    0.7395   29.21000 4.69593e-1   3.3457

Totals :                   873.05094   51.68463
```

(*R*)-*N*-(2-chloro-2-(quinoxalin-2-yl)ethyl)aniline (31)

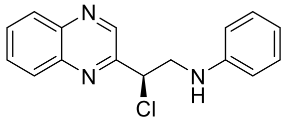

Chemical Formula: $C_{16}H_{14}ClN_3$
Exact Mass: 283.0876
Molecular Weight: 283.7590

Prepared according to General Procedure A using 2-(1-chlorovinyl)quinoxaline **S39** (47.7 mg, 0.25 mmol, 1.0 equiv.), aniline (23 µL, 0.25 mmol, 1.0 equiv.), (*S*)-TCYP (12.4 mg, 12.5 µmol, 5 mol%), and 2-MeTHF (500 µL, 0.5 M). The reaction mixture was subjected to the purification outlined in General Procedure A (silica, EtOAc:petroleum ether, 5:95 to 10:90) to afford the desired product as a brown oil (38.0 mg, 54%). The enantiomeric ratio (94:6) was determined by chiral HPLC in comparison with the racemate.

ν_{max} (film): 1491, 1198, 768 cm^{-1}.

^{1}H NMR (500 MHz, $CDCl_3$): δ 9.03 (s, 1H), 8.15 (ddt, *J* = 7.3, 4.0, 1.7 Hz, 2H), 7.90 – 7.77 (m, 2H), 7.22 (dd, *J* = 8.6, 7.4 Hz, 2H), 6.85 – 6.76 (m, 1H), 6.75 – 6.69 (m, 2H), 5.44 (t, *J* = 6.7 Hz, 1H), 4.22 (dd, *J* = 14.3, 6.6 Hz, 1H), 3.97 (dd, *J* = 14.3, 6.9 Hz, 1H). ^{1}H resonance for NH not observed.

^{13}C NMR (126 MHz, $CDCl_3$): δ 152.8, 146.3, 145.1, 142.1, 141.3, 130.7, 130.6, 129.5, 129.4, 129.4, 118.8, 113.6, 58.1, 49.1.

HRMS (ESI): exact mass calculated for $[M+H]^+$ ($C_{16}H_{15}ClN_3$) requires *m/z* 284.0.949, found *m/z* 284.0940.

HPLC analysis using a chiral column: Chiralcel® OJ-H 5 µm column, 1.0 mL/min, 50:50 hexane:*iso*propanol, 250 nm, t_{major} = 25.7 min, t_{minor} = 36.2 min.

$[\alpha]_D^{23}$ = −54.4° (*c* = 0.5, $CHCl_3$).

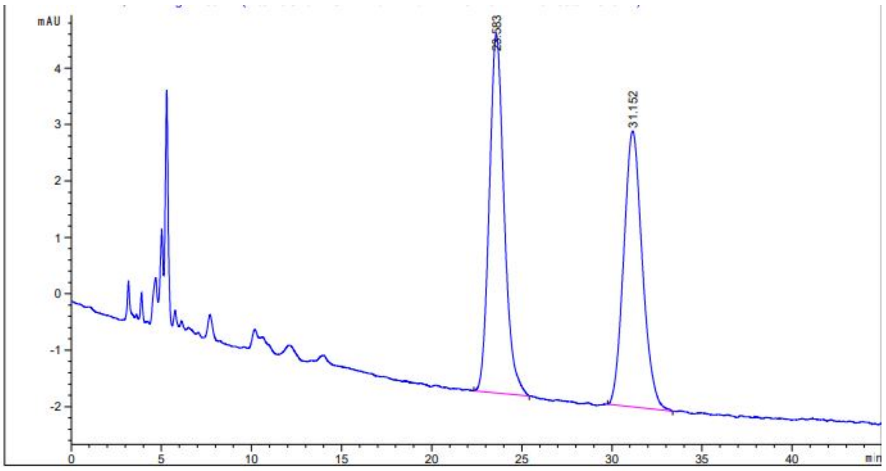

Area Percent Report

Sorted By : Signal
Multiplier : 1.0000
Dilution : 1.0000
Use Multiplier & Dilution Factor with ISTDs

Signal 1: VWD1 A, Wavelength=250 nm

Peak #	RetTime [min]	Type	Width [min]	Area [mAU*s]	Height [mAU]	Area %
1	23.583	BB	0.8284	365.00168	6.35851	50.9716
2	31.152	BB	0.9227	351.08710	4.89478	49.0284

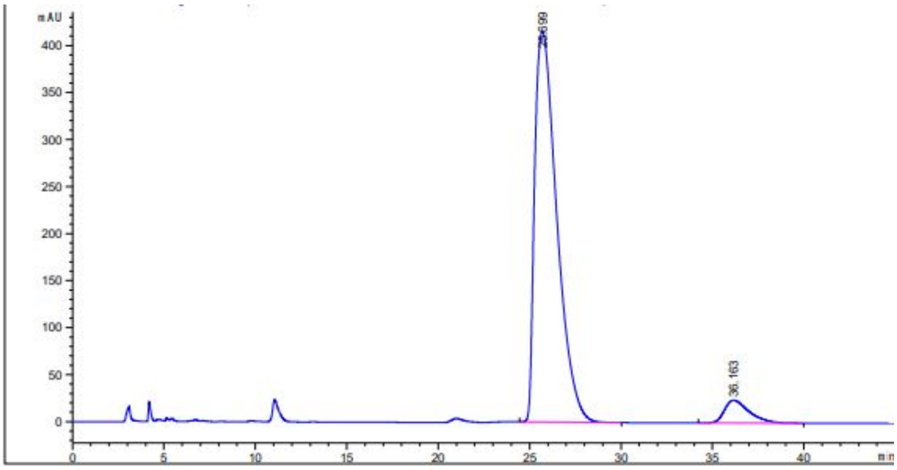


```
=====================================================================
                        Area Percent Report
=====================================================================

Sorted By             :      Signal
Multiplier            :      1.0000
Dilution              :      1.0000
Use Multiplier & Dilution Factor with ISTDs


Signal 1: VWD1 A, Wavelength=254 nm

Peak RetTime Type  Width      Area      Height      Area
  #   [min]        [min]   [mAU*s]      [mAU]        %
----|-------|----|-------|----------|----------|--------|
   1  25.699 BB     1.3034 3.48598e4  415.56088  93.9422
   2  36.163 BB     1.3505 2247.89771  24.06182   6.0578
```

(*R*)-*N*-(2-Chloro-2-(quinazolin-2-yl)ethyl)aniline (32)

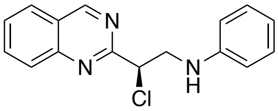

Chemical Formula: $C_{16}H_{14}ClN_3$
Exact Mass: 283.0876
Molecular Weight: 283.7590

Prepared according to General Procedure A using 2-(1-chlorovinyl)quinazoline **S40** (47.7 mg, 0.25 mmol, 1.0 equiv.), (*S*)-TCYP (12.4 mg, 0.0125 mmol, 5 mol%), 2-MeTHF (0.5 mL) and aniline (23.3 mg, 0.25 mmol, 1.0 equiv.) at -20 °C. The reaction mixture was subjected to the purification outlined in General Procedure A (silica, EtOAc:hexane, 20:80) to afford the desired product as a white solid (48.9 mg, 69 %). The enantiomeric ratio (85:15) was determined by chiral HPLC in comparison with the racemate.

ν_{max}(film): 3327, 3049, 2932, 1601, 750 cm^{-1}.

^{1}H NMR (400 MHz, $CDCl_3$): δ 9.45 (d, *J* = 0.8 Hz, 1H), 8.10 – 8.04 (m, 1H), 8.00 – 7.91 (m, 2H), 7.70 (ddd, *J* = 8.0, 6.9, 1.2 Hz, 1H), 7.22 – 7.15 (m, 2H), 6.76 – 6.65 (m, 3H), 5.43 (t, *J* = 6.8 Hz, 1H), 4.21 (dd, *J* = 14.1, 6.9 Hz, 1H), 3.97 (dd, *J* = 14.1, 6.8 Hz, 1H).^{1}H resonance for NH not observed.

^{13}C NMR (101 MHz, $CDCl_3$): δ 162.8, 161.2, 150.1, 146.9, 134.6, 129.4, 128.5, 128.4, 127.2, 123.8, 118.1, 113.2, 60.8, 48.7.

HRMS (ESI): exact mass calculated for $[M+H]^+$ ($C_{16}H_{15}ClN_3$) requires *m/z* 284.0.949, found *m/z* 284.0942.

HPLC analysis using a chiral column: Chiralpak-IA 5 µm column, 1.0 mL/min, 50:50 hexane:*iso*propanol, 250 nm, t_{major} = 8.7 min, t_{minor} = 10.0 min.

$[\alpha]_D^{23}$ = −38.0° (*c* = 0.7, $CHCl_3$).

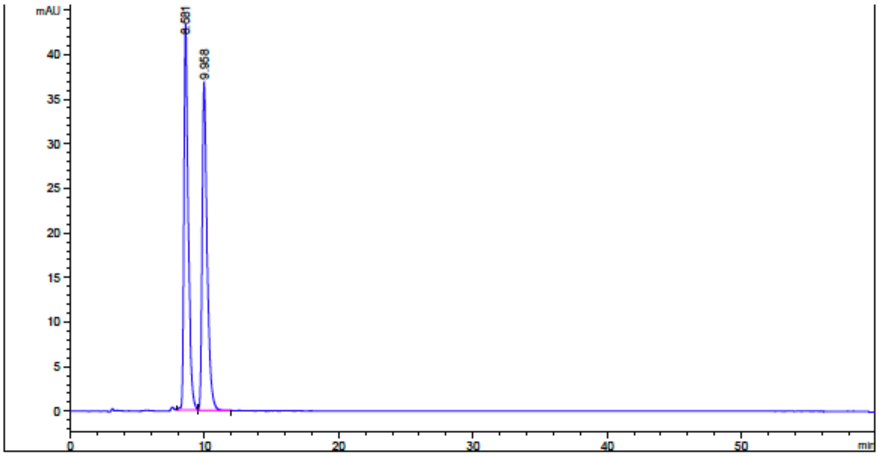

==

Area Percent Report

==

Sorted By : Signal
Multiplier : 1.0000
Dilution : 1.0000
Use Multiplier & Dilution Factor with ISTDs

Signal 1: VWD1 A, Wavelength=254 nm

Peak #	RetTime [min]	Type	Width [min]	Area [mAU*s]	Height [mAU]	Area %
1	8.581	BV	0.3220	956.96130	43.29205	50.0566
2	9.958	VB	0.3789	954.79767	36.74426	49.9434

Totals : 1911.75897 80.03631

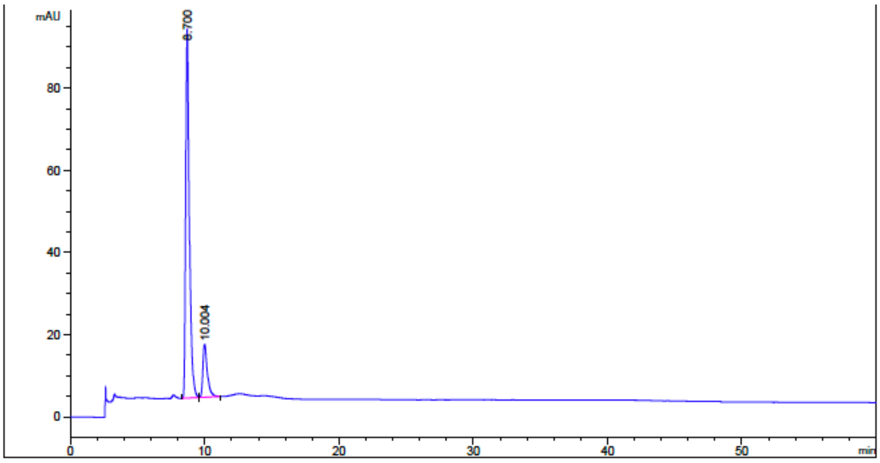

==

Area Percent Report

==

Sorted By : Signal
Multiplier : 1.0000
Dilution : 1.0000
Use Multiplier & Dilution Factor with ISTDs

Signal 1: VWD1 A, Wavelength=254 nm

Peak #	RetTime [min]	Type	Width [min]	Area [mAU*s]	Height [mAU]	Area %
1	8.700	BV	0.2821	1710.09497	89.72555	85.0967
2	10.004	VB	0.3408	299.49536	12.89796	14.9033

Totals : 2009.59033 102.62351

Methyl (*R*)-6-(1-chloro-2-(phenylamino)ethyl)nicotinate (33)

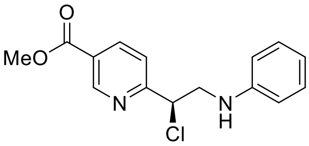

Chemical Formula: $C_{15}H_{15}ClN_2O_2$
Exact Mass: 290.0822
Molecular Weight: 290.7470

Prepared according to General Procedure A using methyl 6-(1-chlorovinyl)nicotinate **S41** (49.4 mg, 0.25 mmol, 1.0 equiv.), aniline (23 µL, 0.25 mmol, 1.0 equiv.), (*S*)-TCYP (12.4 mg, 12.5 µmol, 5 mol%), and 2-MeTHF (500 µL, 0.5 M). The reaction mixture was subjected to the purification outlined in General Procedure A (silica, EtOAc:petroleum ether, 5:95 to 10:90) to afford the desired product as a yellow oil (37.0 mg, 51%). The enantiomeric ratio (94:6) was determined by chiral HPLC in comparison with the racemate.

ν_{max} (film): 1726, 1599, 1506, 1288, 748 cm^{-1}.

^{1}H NMR (500 MHz, $CDCl_3$): δ 9.23 (dd, *J* = 2.2, 0.9 Hz, 1H), 8.33 (dd, *J* = 8.2, 2.2 Hz, 1H), 7.59 (dd, *J* = 8.1, 0.9 Hz, 1H), 7.24 – 7.18 (m, 2H), 6.77 (tt, *J* = 7.4, 1.1 Hz, 1H), 6.70 – 6.65 (m, 2H), 5.25 (dd, *J* = 7.2, 5.9 Hz, 1H), 4.03 (dd, *J* = 14.2, 6.0 Hz, 1H), 3.99 (s, 3H), 3.77 (dd, *J* = 14.3, 7.2 Hz, 1H). ^{1}H resonance for NH not observed.

^{13}C NMR (126 MHz, $CDCl_3$): δ 165.3, 162.0, 150.6, 146.7, 138.2, 129.5, 125.6, 122.5, 118.4, 113.3, 60.4, 52.6, 49.8.

HRMS (ESI): exact mass calculated for $[M+H]^+$ ($C_{15}H_{16}ClN_2O_2$) requires *m/z* 291.0895, found *m/z* 291.0887.

HPLC analysis using a chiral column: Chiralcel® OJ-H 5 µm column, 1.0 mL/min, 50:50 hexane:*iso*propanol, 250 nm, t_{major} = 20.9 min, t_{minor} = 35.1 min.

$[\alpha]_D^{23}$ = −26.8° (*c* = 0.5, $CHCl_3$).

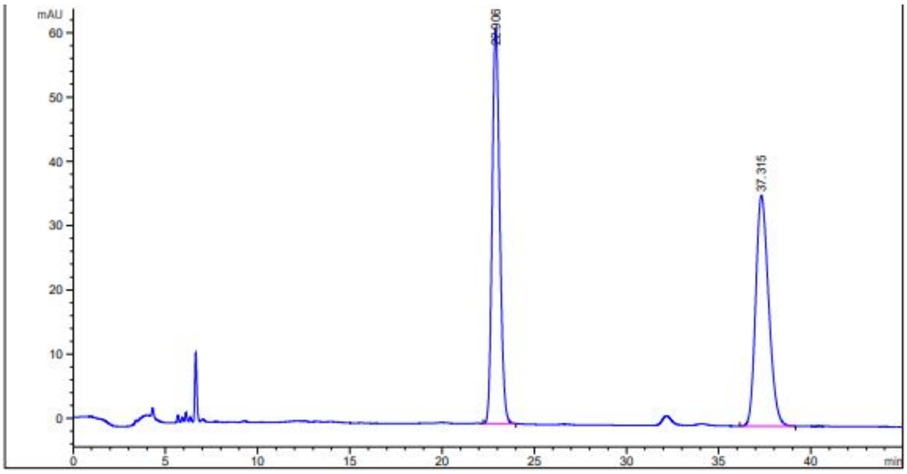

==

Area Percent Report

==

Sorted By : Signal
Multiplier : 1.0000
Dilution : 1.0000
Use Multiplier & Dilution Factor with ISTDs

Signal 1: VWD1 A, Wavelength=250 nm

Peak #	RetTime [min]	Type	Width [min]	Area [mAU*s]	Height [mAU]	Area %
1	22.906	BB	0.4407	1748.06372	61.52238	49.9957
2	37.315	BB	0.7562	1748.36353	35.88901	50.0043

Totals : 3496.42725 97.41140

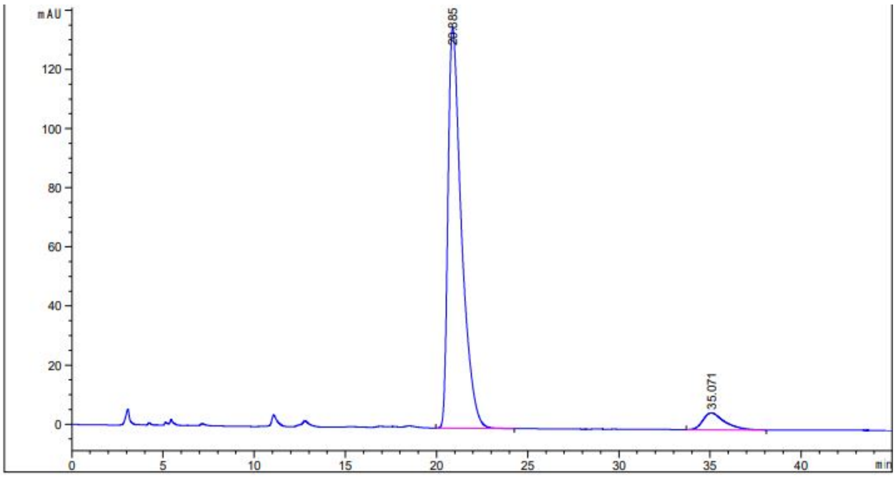

==

Area Percent Report

==

Sorted By : Signal
Multiplier : 1.0000
Dilution : 1.0000
Use Multiplier & Dilution Factor with ISTDs

Signal 1: VWD1 A, Wavelength=254 nm

Peak #	RetTime [min]	Type	Width [min]	Area [mAU*s]	Height [mAU]	Area %
1	20.885	BB	0.7661	6969.77686	135.49152	93.7230
2	35.071	BB	1.1289	466.79010	5.68113	6.2770

(*R*)-6-(1-Chloro-2-(phenylamino)ethyl)nicotinonitrile (34)

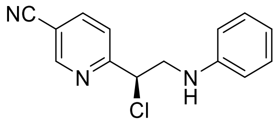

Chemical Formula: $C_{14}H_{12}ClN_3$
Exact Mass: 257.0720
Molecular Weight: 257.7210

Prepared according to General Procedure A using 2-(1-chlorovinyl)nicotinonitrile **S42** (19.6 mg, 0.25 mmol, 1.0 equiv.), (23 µL, 0.25 mmol, 1.0 equiv.), aniline (23 µL, 0.25 mmol, 1.0 equiv.), (*S*)-TCYP (12.4 mg, 12.5 µmol, 5 mol%), and 2-MeTHF (500 µL, 0.5 M). The reaction mixture was subjected to the purification outlined in General Procedure A (silica, EtOAc:petroleum ether, 5:95 to 10:90) to afford the desired product as a yellow solid (41.0 mg, 64%). The enantiomeric ratio (91:9) was determined by chiral HPLC in comparison with the racemate.

ν_{max} (film): 2232, 1601, 1479, 755 cm^{-1}.

^{1}H NMR (500 MHz, CDCl$_3$): δ 8.87 (dd, *J* = 2.1, 0.9 Hz, 1H), 7.95 (dd, *J* = 8.1, 2.1 Hz, 1H), 7.63 (dd, *J* = 8.2, 0.9 Hz, 1H), 7.25 – 7.20 (m, 2H), 6.86 (t, *J* = 7.3 Hz, 1H), 6.78 (d, *J* = 7.9 Hz, 2H), 5.28 (app. t, *J* = 6.5 Hz, 1H), 4.04 (dd, *J* = 14.2, 6.1 Hz, 1H), 3.76 (dd, *J* = 14.1, 7.0 Hz, 1H). ^{1}H resonance for NH not observed.

^{13}C NMR (126 MHz, CDCl$_3$): δ 162.1, 152.1, 146.4, 140.4, 129.6, 123.1, 118.8, 116.4, 113.5, 109.5, 60.1, 49.9.

HRMS (ESI): exact mass calculated for $[M+H]^+$ ($C_{14}H_{13}ClN_3$) requires *m/z* 258.0793, found *m/z* 258.0786.

HPLC analysis using a chiral column: Chiralcel® OJ-H 5 µm column, 1.0 mL/min, 50:50 hexane:*iso*propanol, 250 nm, t_{major} = 22.1 min, t_{minor} = 33.9 min.

$[\alpha]_D^{23}$ = −17.6° (*c* = 0.5, CHCl$_3$).

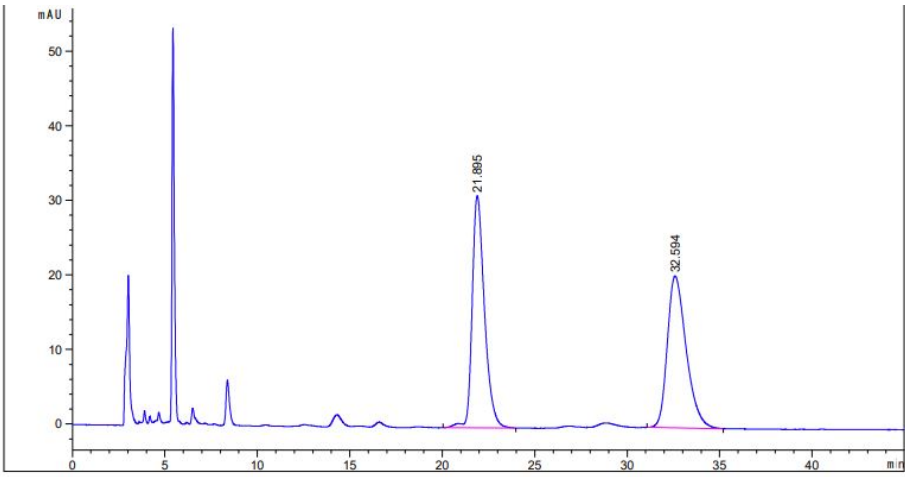


```
=====================================================================
                         Area Percent Report
=====================================================================

Sorted By             :      Signal
Multiplier            :      1.0000
Dilution              :      1.0000
Use Multiplier & Dilution Factor with ISTDs


Signal 1: VWD1 A, Wavelength=250 nm
```

Peak #	RetTime [min]	Type	Width [min]	Area [mAU*s]	Height [mAU]	Area %
1	21.895	BB	0.7014	1440.15808	31.12913	49.9468
2	32.594	BB	1.0300	1443.22522	20.38183	50.0532

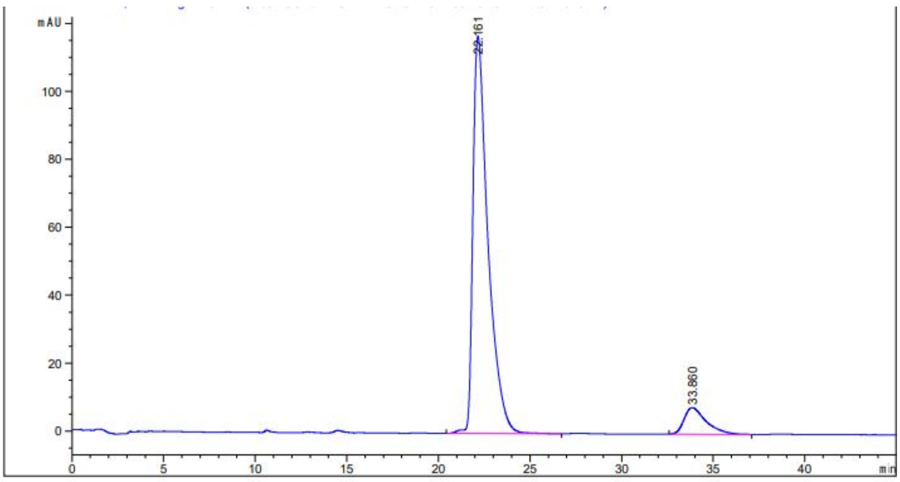

==

Area Percent Report

==

Sorted By : Signal
Multiplier : 1.0000
Dilution : 1.0000
Use Multiplier & Dilution Factor with ISTDs

Signal 1: VWD1 A, Wavelength=254 nm

Peak #	RetTime [min]	Type	Width [min]	Area [mAU*s]	Height [mAU]	Area %
1	22.161	BB	0.8446	6683.55859	116.91071	91.1596
2	33.860	BB	1.1494	648.15302	7.78115	8.8404

(*R*)-*N*-(2-(Benzo[*d*]thiazol-2-yl)-2-chloroethyl)aniline (35)

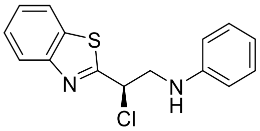

Chemical Formula: $C_{15}H_{13}ClN_2S$
Exact Mass: 288.0488
Molecular Weight: 288.7930

Prepared according to General Procedure A using 2-(1-chlorovinyl)benzo[d]thiazole **S43** (16.0 mg, 0.08 mmol, 1.0 equiv.), aniline (7.5 µL, 0.08 mmol, 1.0 equiv.), (*S*)-TCYP (4.1 mg, 4.1 µmol, 5 mol%), and 2-MeTHF (160 µL, 0.5 M). The reaction mixture was subjected to the purification outlined in General Procedure A (silica gel, EtOAc:petroleum ether, 5:95 to 10:90) to afford the desired product as a yellow oil (11.4 mg, 48%). The enantiomeric ratio (99:1) was determined by chiral HPLC in comparison with the racemate.

ν_{max} (film): 1600, 1499, 750 cm^{-1}.

^{1}H NMR (500 MHz, $CDCl_3$): δ 8.06 (d, *J* = 8.2 Hz, 1H), 7.90 (d, *J* = 8.0 Hz, 1H), 7.53 (ddd, *J* = 8.3, 7.3, 1.2 Hz, 1H), 7.44 (td, *J* = 7.8, 7.3, 1.1 Hz, 1H), 7.25 – 7.18 (m, 2H), 6.84 – 6.77 (m, 1H), 6.77 – 6.66 (m, 2H), 5.48 (dd, *J* = 6.9, 6.0 Hz, 1H), 4.15 (dd, *J* = 14.3, 5.9 Hz, 1H), 3.87 (dd, *J* = 14.3, 7.0 Hz, 1H). ^{1}H resonance for NH not observed.

^{13}C NMR (126 MHz, $CDCl_3$): δ 169.2, 152.9, 146.4, 135.5, 129.7, 126.6, 125.9, 123.7, 121.9, 118.9, 113.7, 57.3, 50.5.

HRMS (ESI): exact mass calculated for $[M+H]^+$ ($C_{15}H_{14}ClN_2S$) requires *m/z* 289.0561, found *m/z* 289.0557.

HPLC analysis using a chiral column: Chiralcel® OJ-H 5 µm column, 1.0 mL/min, 50:50 hexane:*iso*propanol, 250 nm, t_{major} = 18.8 min, t_{minor} = 23.8 min.

$[\alpha]_D^{23}$ = −30.3° (*c* = 0.5, $CHCl_3$).

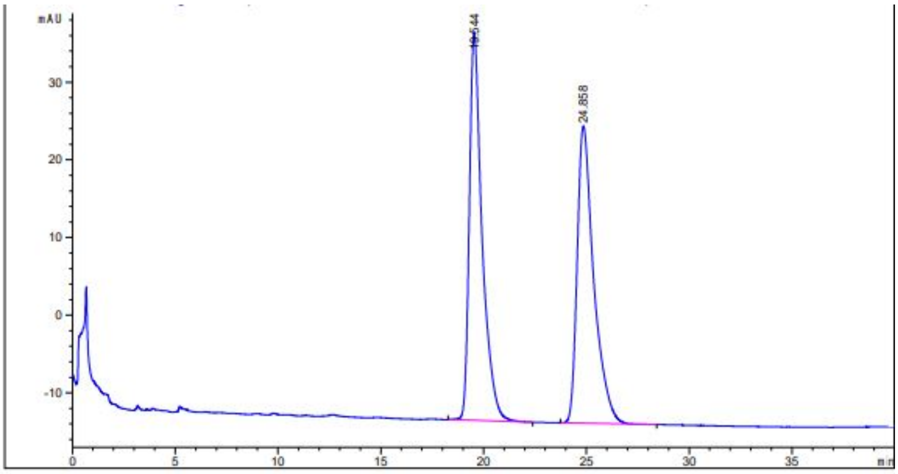

Area Percent Report

Sorted By : Signal
Multiplier : 1.0000
Dilution : 1.0000
Use Multiplier & Dilution Factor with ISTDs

Signal 1: VWD1 A, Wavelength=250 nm

Peak #	RetTime [min]	Type	Width [min]	Area [mAU*s]	Height [mAU]	Area %
1	19.544	BB	0.6324	2156.61963	49.89727	50.1721
2	24.858	BB	0.8216	2141.82593	38.28201	49.8279

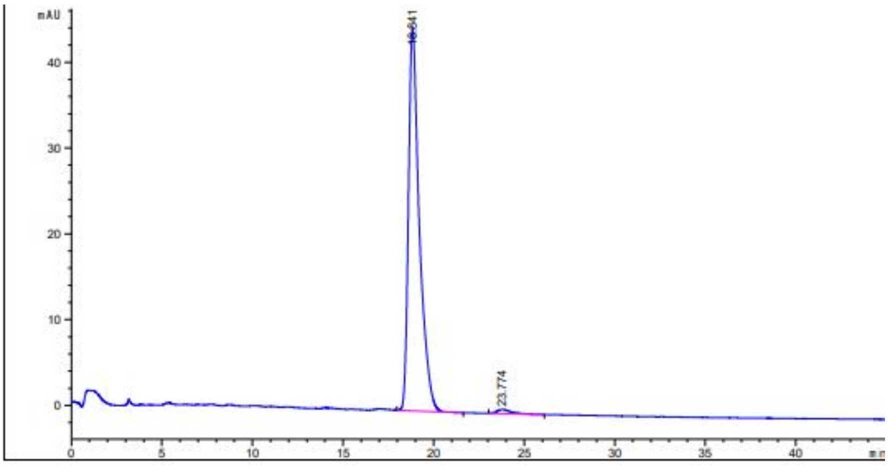


```
=====================================================================
                     Area Percent Report
=====================================================================

Sorted By             :      Signal
Multiplier            :      1.0000
Dilution              :      1.0000
Use Multiplier & Dilution Factor with ISTDs


Signal 1: VWD1 A, Wavelength=250 nm

Peak RetTime Type  Width     Area      Height     Area
  #   [min]        [min]   [mAU*s]     [mAU]        %
----|-------|----|-------|----------|----------|--------|
   1  18.841 BB    0.6149 1860.93738   44.60484  98.6500
   2  23.774 BB    0.6725   25.46739 4.72502e-1   1.3500
```

(*S*)-2-(1-Phenylaziridin-2-yl)quinoline (36)

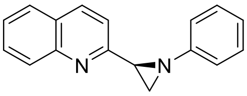

Chemical Formula: $C_{17}H_{14}N_2$
Exact Mass: 246.1157
Molecular Weight: 246.3130

Prepared according to General Procedure B using 2-(1-chlorovinyl)quinoline **1** (47.4 mg, 0.25 mmol, 1.0 equiv.), aniline (23 µL, 0.25 mmol, 1.0 equiv.), (*S*)-TCYP (12.4 mg, 12.5 µmol, 5 mol%), 0.6 M LiHMDS in THF (0.5 mL, 0.3 mmol, 1.2 equiv.), and 2-MeTHF (500 µL, 0.5 M). The reaction mixture was subjected to the purification outlined in General Procedure B (silica, EtOAc:cyclohexane, 5:95 to 10:90) to afford the desired product as a pale yellow solid (41.1 mg, 67%). The enantiomeric ratio (95:5) was determined by chiral HPLC in comparison with the racemate.

ν_{max} (film): 1597, 1489 cm^{-1}.

^{1}H NMR (500 MHz, $CDCl_3$): δ 8.16 (d, *J* = 8.5 Hz, 1H), 8.11 (d, *J* = 8.5 Hz, 1H), 7.82 (d, *J* = 8.0 Hz, 1H), 7.75 – 7.69 (m, 1H), 7.56 – 7.50 (m, 1H), 7.48 (d, *J* = 8.5 Hz, 1H), 7.31 – 7.23 (m, 2H), 7.11 (d, *J* = 7.5 Hz, 2H), 7.02 (t, *J* = 7.4 Hz, 1H), 3.52 (dd, *J* = 6.5, 3.2 Hz, 1H), 2.64 – 2.59 (m, 2H).

^{13}C NMR (126 MHz, $CDCl_3$): δ 159.6, 154.0, 147.8, 137.1, 129.8, 129.2, 129.0, 127.8, 127.7, 126.4, 123.0, 120.8, 117.9, 43.2, 37.3.

HRMS (ESI): exact mass calculated for $[M+H]^+$ ($C_{17}H_{15}N_2$) requires *m/z* 247.1230, found *m/z* 247.1225.

HPLC analysis using a chiral column: Chiral Art Amylose-C 5 µm column, 1.0 mL/min, 90:10 hexane:*iso*propanol, 250 nm, t_{major} = 6.9 min, t_{minor} = 7.4 min.

$[\alpha]_D^{23}$ = −174° (*c* = 1.0, $CHCl_3$).

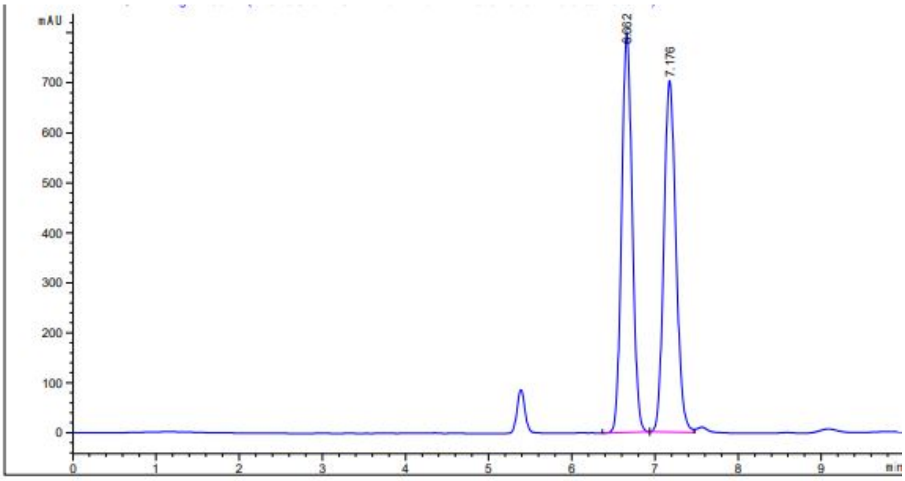

Area Percent Report

Sorted By : Signal
Multiplier : 1.0000
Dilution : 1.0000
Use Multiplier & Dilution Factor with ISTDs

Signal 1: VWD1 A, Wavelength=250 nm

Peak #	RetTime [min]	Type	Width [min]	Area [mAU*s]	Height [mAU]	Area %
1	6.662	BB	0.1382	7068.64160	798.46411	49.8931
2	7.176	BV	0.1556	7098.93604	703.50610	50.1069

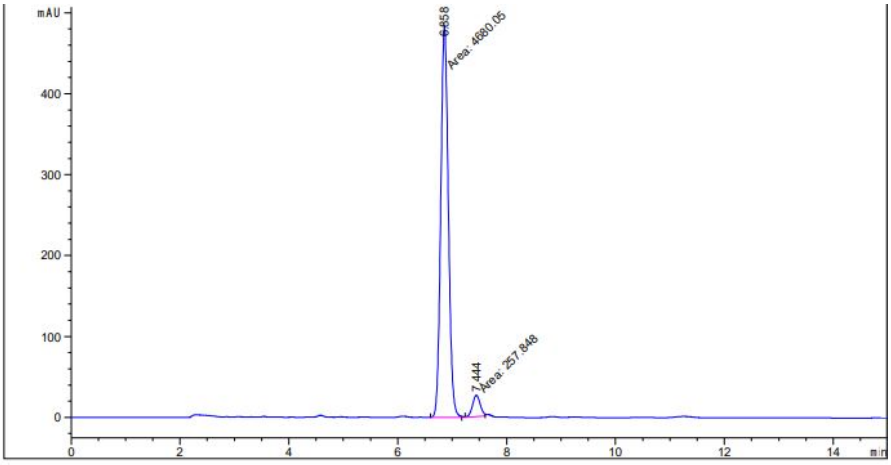


```
=====================================================================
                     Area Percent Report
=====================================================================

Sorted By             :      Signal
Multiplier            :      1.0000
Dilution              :      1.0000
Use Multiplier & Dilution Factor with ISTDs


Signal 1: VWD1 A, Wavelength=250 nm

Peak RetTime Type  Width     Area      Height     Area
  #   [min]        [min]   [mAU*s]     [mAU]       %
----|-------|----|-------|----------|----------|--------|
   1   6.858 MM    0.1614 4680.05420  483.23697  94.7782
   2   7.444 MM    0.1638  257.84833   26.23678   5.2218
```

(*S*)-2-(1-(*p*-Tolyl)aziridin-2-yl)quinoline (37)

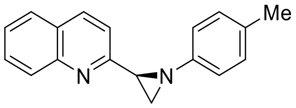

Chemical Formula: $C_{18}H_{16}N_2$
Exact Mass: 260.1313
Molecular Weight: 260.3400

Prepared according to General Procedure B using 2-(1-chlorovinyl)quinoline **1** (47.4 mg, 0.25 mmol, 1.0 equiv.), *p*-toluidine (26.8 mg, 0.25 mmol, 1.0 equiv.), (*S*)-TCYP (12.4 mg, 12.5 µmol, 5 mol%), 0.6 M LiHMDS in THF (0.5 mL, 0.3 mmol, 1.2 equiv.), and 2-MeTHF (500 µL, 0.5 M). The reaction mixture was subjected to the purification outlined in General Procedure B (silica, EtOAc:cyclohexane, 5:95 to 10:90) to afford the desired product as a pale yellow solid (41.6 mg, 64%). The enantiomeric ratio (97:3) was determined by chiral HPLC in comparison with the racemate.

ν_{max} (film): 1601, 1504, 1485 cm^{-1}.

^{1}H NMR (500 MHz, $CDCl_3$): δ 8.19 – 8.11 (m, 2H), 7.80 (d, *J* = 7.8 Hz, 1H), 7.76 – 7.70 (m, 1H), 7.52 (t, *J* = 7.4 Hz, 1H), 7.47 (d, *J* = 8.5 Hz, 1H), 7.08 (d, *J* = 8.2 Hz, 2H), 7.02 (d, *J* = 8.3 Hz, 2H), 3.49 (dd, *J* = 6.5, 3.2 Hz, 1H), 2.60 (app. d, *J* = 2.5 Hz, 1H), 2.57 (app. d, *J* = 6.6 Hz, 1H), 2.30 (s, 3H).

^{13}C NMR (126 MHz, $CDCl_3$): δ 159.6, 151.5, 147.7, 137.0, 132.3, 129.7, 129.7, 128.9, 127.7, 127.6, 126.2, 120.6, 117.8, 43.2, 37.4, 20.8.

HRMS (ESI): exact mass calculated for $[M+H]^+$ ($C_{18}H_{17}N_2$) requires *m/z* 261.1386, found *m/z* 261.1378.

HPLC analysis using a chiral column: Chiralcel® OJ-H 5 µm column, 1.0 mL/min, 90:10 hexane:*iso*propanol, 250 nm, t_{major} = 25.5 min, t_{minor} = 23.6 min.

$[\alpha]_D^{23}$ = −20.8° (*c* = 1.0, $CHCl_3$).

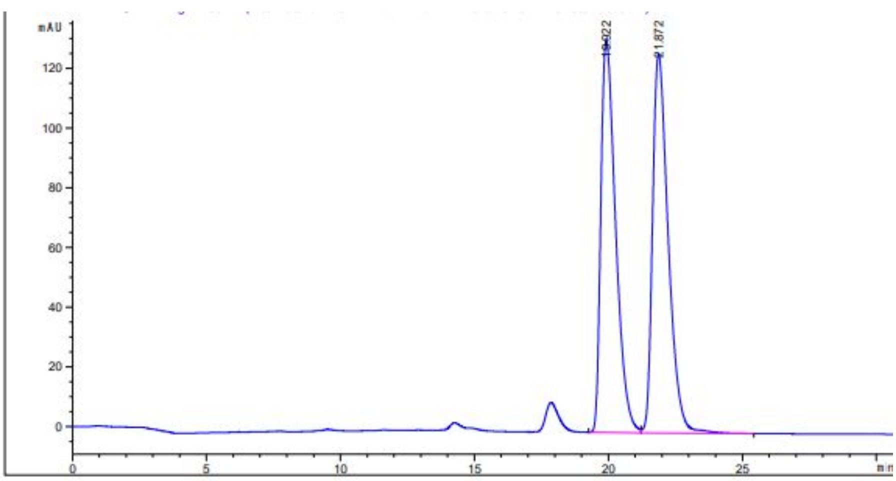

Area Percent Report

Sorted By : Signal
Multiplier : 1.0000
Dilution : 1.0000
Use Multiplier & Dilution Factor with ISTDs

Signal 1: VWD1 A, Wavelength=250 nm

Peak #	RetTime [min]	Type	Width [min]	Area [mAU*s]	Height [mAU]	Area %
1	19.922	BV	0.5901	5040.80664	131.45677	49.4185
2	21.872	VB	0.6196	5159.43945	127.00171	50.5815

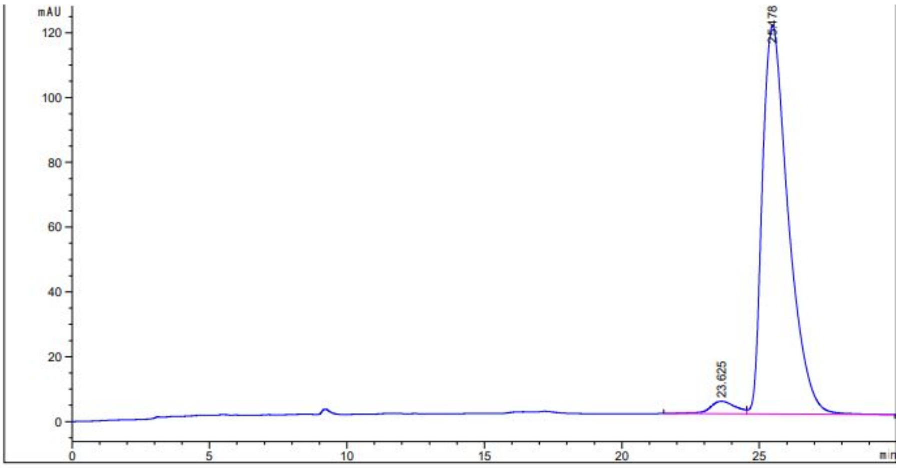

Area Percent Report

Sorted By : Signal
Multiplier : 1.0000
Dilution : 1.0000
Use Multiplier & Dilution Factor with ISTDs

Signal 1: VWD1 A, Wavelength=250 nm

Peak #	RetTime [min]	Type	Width [min]	Area [mAU*s]	Height [mAU]	Area %
1	23.625	BV	0.8964	254.11633	3.88838	3.1271
2	25.478	VBA	1.0030	7872.26904	119.94492	96.8729

(*S*)-2-(1-(*m*-Tolyl)aziridin-2-yl)quinoline (38)

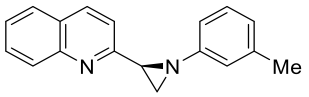

Chemical Formula: $C_{18}H_{16}N_2$
Exact Mass: 260.1313
Molecular Weight: 260.3400

Prepared according to General Procedure B using 2-(1-chlorovinyl)quinoline **1** (47.4 mg, 0.25 mmol, 1.0 equiv.), *m*-toluidine (26.8 mg, 0.25 mmol, 1.0 equiv.), (*S*)-TCYP (12.4 mg, 12.5 µmol, 5 mol%), 0.6 M LiHMDS in THF (0.5 mL, 0.3 mmol, 1.2 equiv.), and 2-MeTHF (500 µL, 0.5 M). The reaction mixture was subjected to the purification outlined in General Procedure B (silica, EtOAc:cyclohexane, 5:95 to 10:90) to afford the desired product as a pale yellow solid (50.1 mg, 77%). The enantiomeric ratio (96:4) was determined by chiral HPLC in comparison with the racemate.

ν_{max} (film): 1601, 1504, 1485 cm^{-1}.

^{1}H NMR (500 MHz, $CDCl_3$): δ 8.16 (d, *J* = 8.5 Hz, 1H), 8.11 (d, *J* = 8.4 Hz, 1H), 7.82 (d, *J* = 8.1 Hz, 1H), 7.73 (ddd, *J* = 8.4, 6.9, 1.4 Hz, 1H), 7.53 (ddd, *J* = 8.0, 7.0, 1.1 Hz, 1H), 7.47 (d, *J* = 8.5 Hz, 1H), 7.16 (t, *J* = 7.7 Hz, 1H), 6.95 – 6.89 (m, 2H), 6.84 (d, *J* = 7.5 Hz, 1H), 3.52 – 3.49 (m, 1H), 2.61 – 2.59 (m, 2H), 2.32 (s, 3H).

^{13}C NMR (126 MHz, $CDCl_3$): δ 159.7, 153.9, 147.8, 139.1, 137.1, 129.8, 129.1, 129.0, 127.8, 127.7, 126.3, 123.8, 121.5, 117.8, 117.8, 43.1, 37.4, 21.5.

HRMS (ESI): exact mass calculated for $[M+H]^+$ ($C_{18}H_{17}N_2$) requires *m/z* 261.1386, found *m/z* 261.1376.

HPLC analysis using a chiral column: Chiralcel® OJ-H 5 µm column, 1.0 mL/min, 50:50 hexane:*iso*propanol, 250 nm, t_{major} = 8.2 min, t_{minor} = 9.9 min.

$[\alpha]_D^{23}$ = −156° (*c* = 1.0, $CHCl_3$).

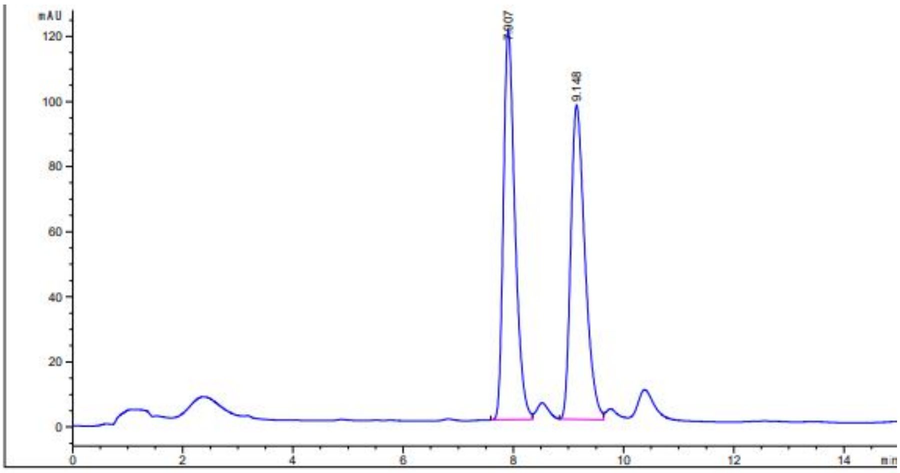

Area Percent Report

Sorted By : Signal
Multiplier : 1.0000
Dilution : 1.0000
Use Multiplier & Dilution Factor with ISTDs

Signal 1: VWD1 A, Wavelength=250 nm

Peak #	RetTime [min]	Type	Width [min]	Area [mAU*s]	Height [mAU]	Area %
1	7.907	BV	0.2205	1740.78992	119.77800	50.2732
2	9.148	BV	0.2737	1721.87036	96.48779	49.7268

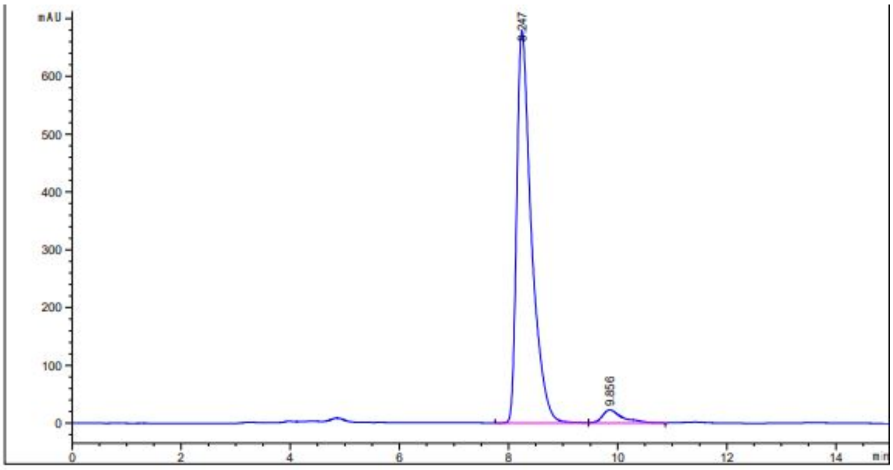

Area Percent Report

Sorted By : Signal
Multiplier : 1.0000
Dilution : 1.0000
Use Multiplier & Dilution Factor with ISTDs

Signal 1: VWD1 A, Wavelength=250 nm

Peak #	RetTime [min]	Type	Width [min]	Area [mAU*s]	Height [mAU]	Area %
1	8.247	BV	0.2781	1.27008e4	678.57654	95.7379
2	9.856	VB	0.3662	565.42218	22.48123	4.2621

(*S*)-2-(1-(*o*-Tolyl)aziridin-2-yl)quinoline (39)

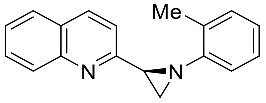

Chemical Formula: $C_{18}H_{16}N_2$
Exact Mass: 260.1313
Molecular Weight: 260.3400

Prepared according to General Procedure B using 2-(1-chlorovinyl)quinoline **1** (47.4 mg, 0.25 mmol, 1.0 equiv.), *o*-toluidine (26.8 mg, 0.25 mmol, 1.0 equiv.), (*S*)-TCYP (12.4 mg, 12.5 µmol, 5 mol%), 0.6 M LiHMDS in THF (0.5 mL, 0.3 mmol, 1.2 equiv.), and 2-MeTHF (500 µL, 0.5 M) for 39 h. The reaction mixture was subjected to the purification outlined in General Procedure B (silica, EtOAc:cyclohexane, 5:95 to 10:90) to afford the desired product as a pale yellow solid (52.4 mg, 81%). The enantiomeric ratio (90:10) was determined by chiral HPLC in comparison with the racemate.

ν_{max} (film): 1599, 1504, 1489 cm^{-1}.

^{1}H NMR (500 MHz, $CDCl_3$): δ 8.17 (d, *J* = 8.6 Hz, 1H), 8.11 (d, *J* = 8.5 Hz, 1H), 7.83 (dd, *J* = 8.1, 1.1 Hz, 1H), 7.73 (ddd, *J* = 8.4, 6.9, 1.4 Hz, 1H), 7.57 – 7.50 (m, 2H), 7.20 – 7.11 (m, 2H), 7.02 – 6.94 (m, 2H), 3.41 (dd, *J* = 6.5, 3.2 Hz, 1H), 2.66 (dd, *J* = 6.5, 1.0 Hz, 1H), 2.62 (dd, *J* = 3.2, 1.1 Hz, 1H), 2.25 (s, 3H).

^{13}C NMR (126 MHz, $CDCl_3$): δ 159.7, 151.4, 147.8, 137.1, 131.0, 130.7, 129.8, 129.0, 127.8, 127.7, 126.6, 126.3, 122.9, 119.1, 117.6, 43.5, 37.4, 18.5.

HRMS (ESI): exact mass calculated for $[M+H]^+$ ($C_{18}H_{17}N_2$) requires *m/z* 261.1386, found *m/z* 261.1377.

HPLC analysis using a chiral column: Chiral Art Amylose-C 5 µm column, 1.0 mL/min, 99:1 hexane:*iso*propanol, 250 nm, t_{major} = 12.2 min, t_{minor} = 13.9 min.

$[\alpha]_D^{23}$ = −144° (*c* = 1.0, $CHCl_3$).

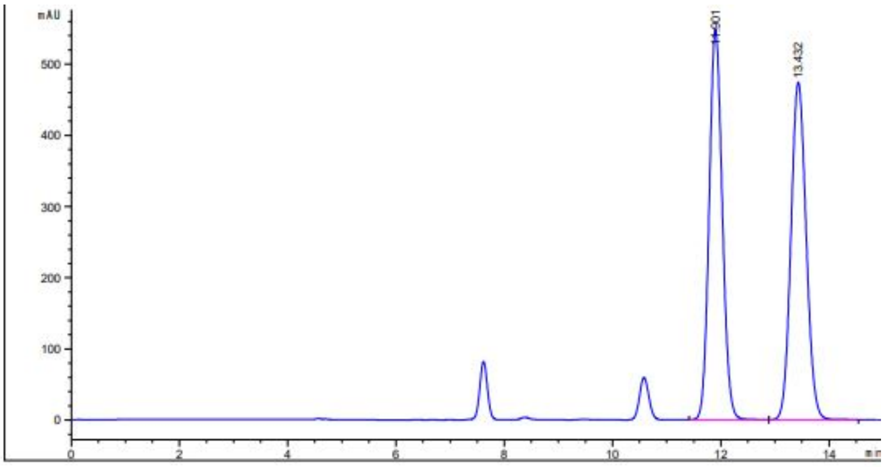

Area Percent Report

Sorted By : Signal
Multiplier : 1.0000
Dilution : 1.0000
Use Multiplier & Dilution Factor with ISTDs

Signal 1: VWD1 A, Wavelength=250 nm

Peak #	RetTime [min]	Type	Width [min]	Area [mAU*s]	Height [mAU]	Area %
1	11.901	VB	0.2618	9233.72168	548.90411	49.9473
2	13.432	BB	0.3033	9253.20020	474.24991	50.0527

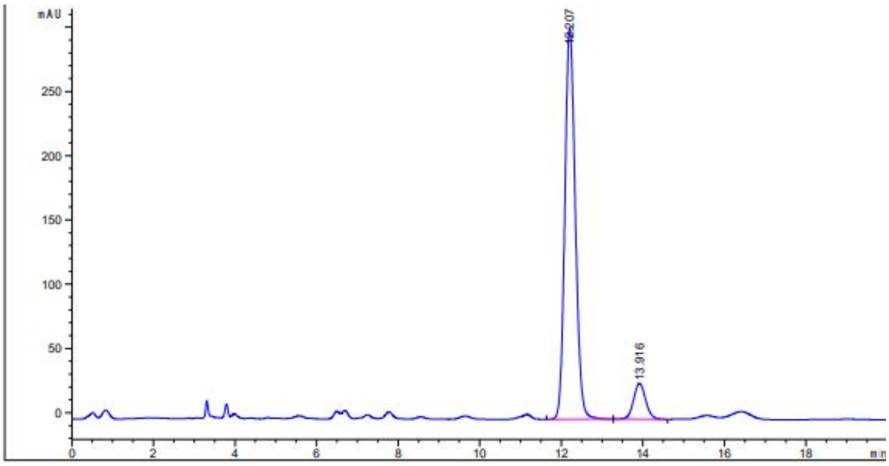

Area Percent Report

Sorted By : Signal
Multiplier : 1.0000
Dilution : 1.0000
Use Multiplier & Dilution Factor with ISTDs

Signal 1: VWD1 A, Wavelength=250 nm

Peak #	RetTime [min]	Type	Width [min]	Area [mAU*s]	Height [mAU]	Area %
1	12.207	BB	0.2736	5393.33887	304.45407	90.4184
2	13.916	BB	0.3173	571.52673	27.93714	9.5816

(*S*)-2-(1-(4-Methoxyphenyl)aziridin-2-yl)quinoline (40)

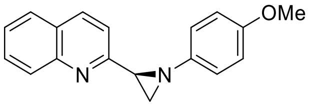

Chemical Formula: $C_{18}H_{16}N_2O$
Exact Mass: 276.13
Molecular Weight: 276.34

Prepared according to General Procedure B using 2-(1-chlorovinyl)quinoline **1** (47.4 mg, 0.25 mmol, 1.0 equiv.), *p*-anisidine (30.8 mg, 0.25 mmol, 1.0 equiv.), (*S*)-TCYP (12.4 mg, 12.5 µmol, 5 mol%), 0.6 M LiHMDS in THF (0.5 mL, 0.3 mmol, 1.2 equiv.), and 2-MeTHF (500 µL, 0.5 M) for 39 h. The reaction mixture was subjected to the purification outlined in General Procedure B (silica, EtOAc:cyclohexane, 5:95 to 10:90) to afford the desired product as a pale yellow solid (49.5 mg, 72%). The enantiomeric ratio (95:5) was determined by chiral HPLC in comparison with the racemate.

v_{max} (film): 1504, 1240 cm^{-1}.

^{1}H NMR (500 MHz, $CDCl_3$): δ 8.15 (d, *J* = 8.5 Hz, 1H), 8.10 (d, *J* = 8.5 Hz, 1H), 7.81 (d, *J* = 8.1 Hz, 1H), 7.72 (ddd, *J* = 8.4, 6.9, 1.4 Hz, 1H), 7.52 (ddd, *J* = 8.1, 7.0, 1.1 Hz, 1H), 7.46 (d, *J* = 8.5 Hz, 1H), 7.06 – 7.01 (m, 2H), 6.85 – 6.79 (m, 2H), 3.77 (s, 3H), 3.45 (dd, *J* = 6.5, 3.2 Hz, 1H), 2.59 (dd, *J* = 3.2, 1.1 Hz, 1H), 2.55 (dd, *J* = 6.6, 1.1 Hz, 1H).

^{13}C NMR (126 MHz, $CDCl_3$): δ 159.7, 155.5, 147.8, 147.3, 137.0, 129.8, 129.0, 127.8, 127.7, 126.3, 121.6, 117.9, 114.4, 55.6, 43.5, 37.6.

HRMS (ESI): exact mass calculated for $[M+H]^+$ ($C_{18}H_{17}N_2O$) requires *m/z* 277.1335, found *m/z* 277.1326.

HPLC analysis using a chiral column: Chiralcel® OJ-H 5 µm column, 1.0 mL/min, 50:50 hexane:*iso*propanol, 250 nm, t_{major} = 20.0 min, t_{minor} = 13.9 min.

$[\alpha]_D^{23}$ = −168° (*c* = 0.5, $CHCl_3$).

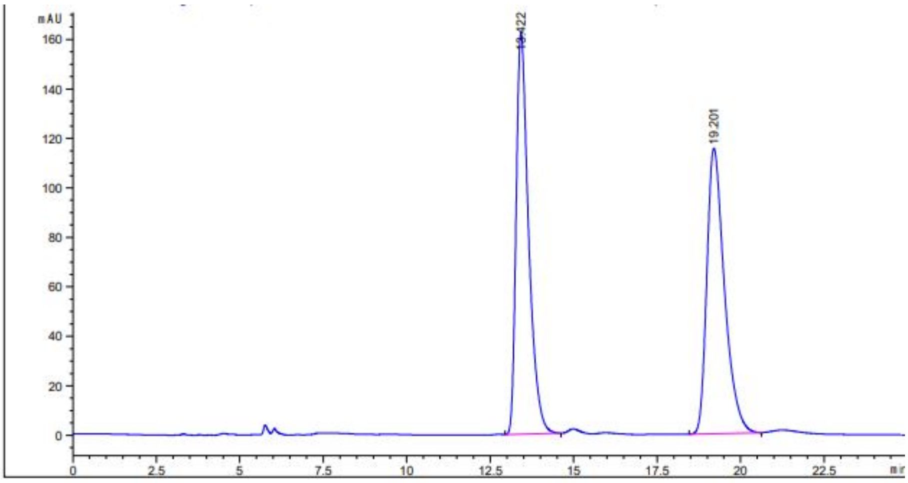

Area Percent Report

Sorted By	:	Signal
Multiplier	:	1.0000
Dilution	:	1.0000

Use Multiplier & Dilution Factor with ISTDs

Signal 1: VWD1 A, Wavelength=250 nm

Peak #	RetTime [min]	Type	Width [min]	Area [mAU*s]	Height [mAU]	Area %
1	13.422	BB	0.3880	4206.20264	162.42598	49.9030
2	19.201	BB	0.5551	4222.56250	115.38980	50.0970

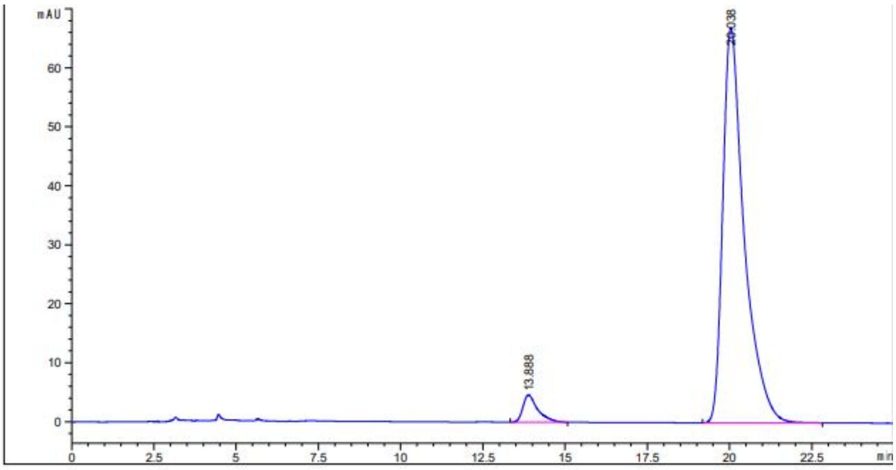

Area Percent Report

Sorted By	:	Signal
Multiplier	:	1.0000
Dilution	:	1.0000

Use Multiplier & Dilution Factor with ISTDs

Signal 1: VWD1 A, Wavelength=250 nm

Peak #	RetTime [min]	Type	Width [min]	Area [mAU*s]	Height [mAU]	Area %
1	13.888	BB	0.4569	144.37102	4.61202	4.5326
2	20.038	BB	0.6697	3040.76855	66.94771	95.4674

(*S*)-2-(1-(3-Bromophenyl)aziridin-2-yl)quinoline (41)

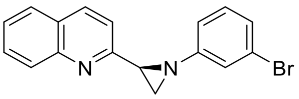

Chemical Formula: $C_{17}H_{13}BrN_2$
Exact Mass: 324.03
Molecular Weight: 325.21

Prepared according to General Procedure B using 2-(1-chlorovinyl)quinoline **1** (47.4 mg, 0.25 mmol, 1.0 equiv.), 3-bromoaniline (27 µL, 0.25 mmol, 1.0 equiv.), (*S*)-TCYP (12.4 mg, 12.5 µmol, 5 mol%), 0.6 M LiHMDS in THF (0.5 mL, 0.3 mmol, 1.2 equiv.), and CPME (500 µL, 0.5 M) for 39 h. The reaction mixture was subjected to the purification outlined in General Procedure B (silica, EtOAc:cyclohexane, 5:95 to 10:90) to afford the desired product as a pale yellow solid (67.4 mg, 75%). The enantiomeric ratio (95:5) was determined by chiral HPLC in comparison with the racemate.

ν_{max} (film): 1587, 1473, 831 cm^{-1}.

^{1}H NMR (500 MHz, $CDCl_3$): δ 8.16 (d, *J* = 8.5 Hz, 1H), 8.10 (d, *J* = 8.5 Hz, 1H), 7.81 (d, *J* = 8.1 Hz, 1H), 7.73 (t, *J* = 7.7 Hz, 1H), 7.53 (t, *J* = 7.5 Hz, 1H), 7.43 (d, *J* = 8.5 Hz, 1H), 7.29 (s, 1H), 7.16 – 7.09 (m, 2H), 7.02 (d, *J* = 7.3 Hz, 1H), 3.54 – 3.50 (m, 1H), 2.65 (app. S, 1H), 2.60 (app. D, *J* = 6.5 Hz, 1H).

^{13}C NMR (126 MHz, $CDCl_3$): δ 158.8, 155.4, 147.8, 137.2, 130.5, 129.9, 129.0, 127.8, 127.7, 126.5, 126.1, 124.0, 122.7, 119.6, 117.8, 43.3, 37.3.

HRMS (ESI): exact mass calculated for $[M+H]^+$ ($C_{17}H_{14}BrN_2$) requires *m/z* 325.0335, found *m/z* 325.0325.

HPLC analysis using a chiral column: Chiralcel® OJ-H 5 µm column, 1.0 mL/min, 50:50 hexane:*iso*propanol, 250 nm, t_{major} = 10.2 min, t_{minor} = 9.1 min.

$[\alpha]_D^{23}$ = −141° (*c* = 1.0, $CHCl_3$).

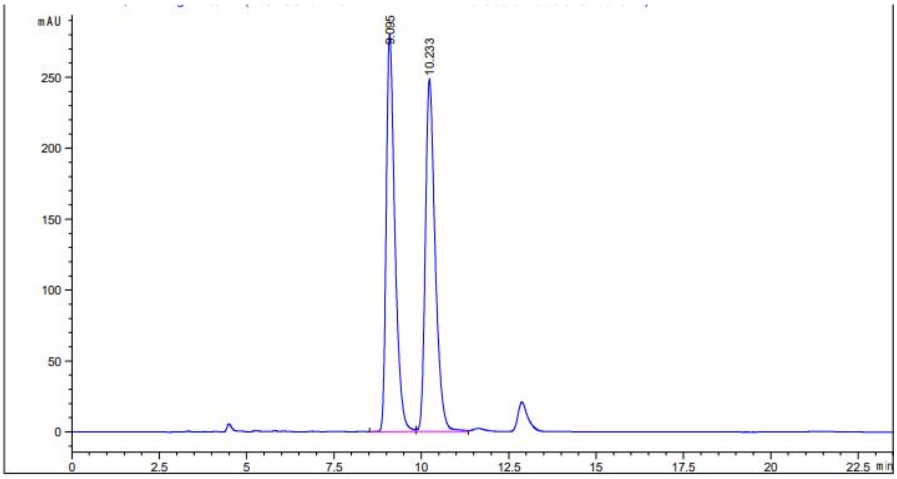

==

Area Percent Report

==

Sorted By : Signal
Multiplier : 1.0000
Dilution : 1.0000
Use Multiplier & Dilution Factor with ISTDs

Signal 1: VWD1 A, Wavelength=250 nm

Peak #	RetTime [min]	Type	Width [min]	Area [mAU*s]	Height [mAU]	Area %
1	9.095	BV	0.2588	4815.40674	280.04730	49.7402
2	10.233	VV	0.2969	4865.71826	248.49672	50.2598

Totals : 9681.12500 528.54402

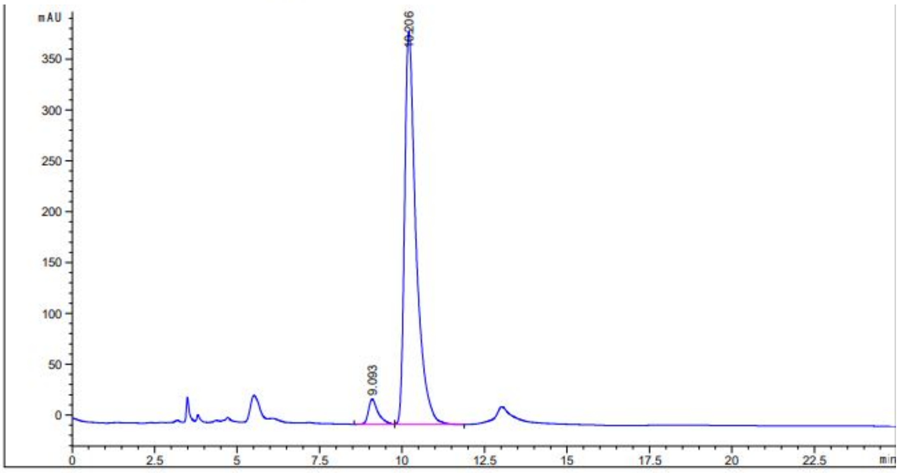

==

Area Percent Report

==

Sorted By : Signal
Multiplier : 1.0000
Dilution : 1.0000
Use Multiplier & Dilution Factor with ISTDs

Signal 1: VWD1 A, Wavelength=250 nm

Peak #	RetTime [min]	Type	Width [min]	Area [mAU*s]	Height [mAU]	Area %
1	9.093	BV	0.2964	500.63089	24.82938	5.0918
2	10.206	VB	0.3559	9331.48145	386.60541	94.9082

(*S*)-2-(1-(4-Fluorophenyl)aziridin-2-yl)quinoline (42)

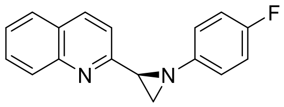

Chemical Formula: $C_{17}H_{13}FN_2$
Exact Mass: 264.1063
Molecular Weight: 264.3034

Prepared according to General Procedure B using 2-(1-chlorovinyl)quinoline **1** (47.4 mg, 0.25 mmol, 1.0 equiv.), 4-fluoroaniline (27 µL, 0.25 mmol, 1.0 equiv.), (*S*)-TCYP (12.4 mg, 12.5 µmol, 5 mol%), 0.6 M LiHMDS in THF (0.5 mL, 0.3 mmol, 1.2 equiv.), and 2-MeTHF (500 µL, 0.5 M). The reaction mixture was subjected to the purification outlined in General Procedure B (silica, EtOAc:cyclohexane, 5:95 to 10:90) to afford the desired product as a pale yellow solid (47.4 mg, 72%). The enantiomeric ratio (96:4) was determined by chiral HPLC in comparison with the racemate.

ν_{max} (film): 1501, 1215 cm^{-1}.

^{1}H NMR (500 MHz, CDCl$_3$): δ 8.16 (d, *J* = 8.5 Hz, 1H), 8.10 (d, *J* = 8.5 Hz, 1H), 7.81 (d, *J* = 8.1 Hz, 1H), 7.73 (ddd, *J* = 8.4, 7.0, 1.4 Hz, 1H), 7.56 – 7.50 (m, 1H), 7.44 (d, *J* = 8.5 Hz, 1H), 7.07 – 7.01 (m, 2H), 6.98 – 6.91 (m, 2H), 3.47 (dd, *J* = 6.6, 3.2 Hz, 1H), 2.63 (dd, *J* = 3.2, 1.0 Hz, 1H), 2.57 (dd, *J* = 6.6, 1.0 Hz, 1H).

^{13}C NMR (126 MHz, CDCl$_3$): δ 159.2, 158.9 (d, $^{1}J_{CF}$ = 240.8 Hz), 150.0 (d, $^{4}J_{CF}$ = 2.2 Hz), 147.8, 137.1, 129.9, 129.0, 127.8, 127.7, 126.4, 121.8 (d, $^{3}J_{CF}$ = 8.0 Hz), 117.8, 115.8 (d, $^{2}J_{CF}$ = 22.6 Hz), 43.5, 37.5.

^{19}F NMR (470 MHz, CDCl$_3$): δ −120.87.

HRMS (ESI): exact mass calculated for $[M+H]^+$ ($C_{17}H_{14}FN_2$) requires *m/z* 265.1136, found *m/z* 265.1129.

HPLC analysis using a chiral column: Chiralcel® OJ-H 5 µm column, 1.0 mL/min, 50:50 hexane:*iso*propanol, 250 nm, t_{major} = 11.1 min, t_{minor} = 8.5 min.

$[\alpha]_D^{23}$ = −182° (*c* = 1.0, CHCl$_3$).

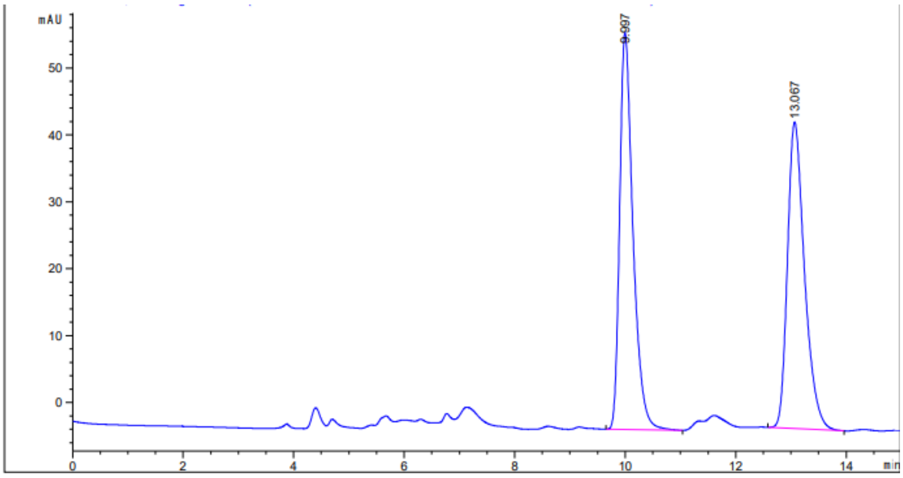

Area Percent Report

Sorted By	:	Signal
Multiplier	:	1.0000
Dilution	:	1.0000

Use Multiplier & Dilution Factor with ISTDs

Signal 1: VWD1 A, Wavelength=250 nm

Peak #	RetTime [min]	Type	Width [min]	Area [mAU*s]	Height [mAU]	Area %
1	9.997	BB	0.2508	993.60071	59.30369	50.3300
2	13.067	BB	0.3225	980.57117	45.81156	49.6700

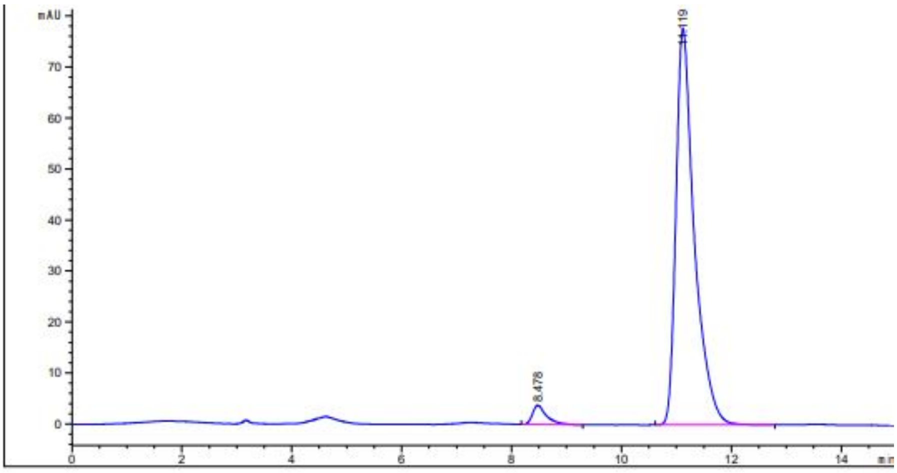


```
=====================================================================
                         Area Percent Report
=====================================================================

Sorted By             :      Signal
Multiplier            :      1.0000
Dilution              :      1.0000
Use Multiplier & Dilution Factor with ISTDs

Signal 1: VWD1 A, Wavelength=250 nm

Peak RetTime Type  Width     Area      Height     Area
  #   [min]        [min]   [mAU*s]     [mAU]       %
----|-------|----|-------|----------|----------|--------|
   1   8.478 BB    0.2592   67.20209    3.76458   3.5852
   2  11.119 BB    0.3428 1807.23425   77.66723  96.4148
```

(*S*)-2-(1-(4-(4,4,5,5-Tetramethyl-1,3,2-dioxaborolan-2-yl)phenyl)aziridin-2-yl)quinoline (43)

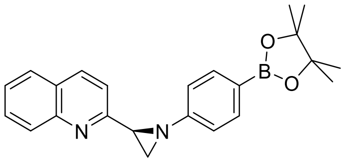

Chemical Formula: $C_{23}H_{25}BN_2O_2$
Exact Mass: 372.20
Molecular Weight: 372.28

Prepared according to General Procedure B using 2-(1-chlorovinyl)quinoline **1** (47.4 mg, 0.25 mmol, 1.0 equiv.), 4-aminophenylboronic acid pinacol ester (54.8 mg, 0.25 mmol, 1.0 equiv.), (*S*)-TCYP (12.4 mg, 12.5 µmol, 5 mol%), 0.6 M LiHMDS in THF (0.5 mL, 0.3 mmol, 1.2 equiv.), and CPME (500 µL, 0.5 M) for 39 h. The reaction mixture was subjected to the purification outlined in General Procedure B (silica, EtOAc:cyclohexane, 5:95 to 10:90) to afford the desired product as a pale yellow solid (44.0 mg, 77%). The enantiomeric ratio (94:6) was determined by chiral HPLC in comparison with the racemate.

ν_{max} (film): 2978, 1601, 1356, 1142, 1086 cm^{-1}.

^{1}H NMR (500 MHz, CDCl$_3$): δ 8.16 (d, *J* = 8.5 Hz, 1H), 8.11 (d, *J* = 8.5 Hz, 1H), 7.81 (d, *J* = 8.1 Hz, 1H), 7.76 – 7.69 (m, 3H), 7.53 (ddd, *J* = 8.1, 7.0, 1.1 Hz, 1H), 7.47 (d, *J* = 8.5 Hz, 1H), 7.11 (d, *J* = 8.4 Hz, 2H), 3.54 (dd, *J* = 6.5, 3.2 Hz, 1H), 2.65 (dd, *J* = 3.1, 1.1 Hz, 1H), 2.61 (dd, *J* = 6.5, 1.1 Hz, 1H), 1.33 (s, 12H).

^{13}C NMR (126 MHz, CDCl$_3$): δ 159.3, 156.8, 147.8, 137.1, 136.1, 129.8, 129.0, 127.8, 127.7, 126.4, 120.2, 117.9, 83.7, 43.1, 37.3, 25.0.

n.b. the carbon bearing boron is not observed due to quadrupolar relaxation.

^{11}B NMR (126 MHz, CDCl$_3$): δ 30.96.

HRMS (ESI): exact mass calculated for $[M+H]^+$ ($C_{23}H_{26}BN_2O_2$) requires *m/z* 373.2082, found *m/z* 373.2073.

HPLC analysis using a chiral column: Chiralcel® OJ-H 5 µm column, 1.0 mL/min, 50:50 hexane:*iso*propanol, 250 nm, t_{major} = 10.5 min, t_{minor} = 7.4 min.

$[\alpha]_D^{23}$ = −150° (*c* = 1.0, $CHCl_3$).

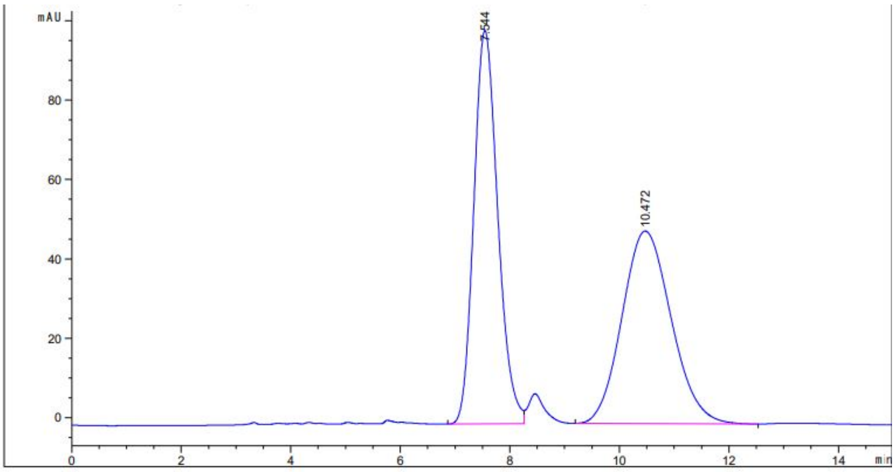

Area Percent Report

Sorted By : Signal
Multiplier : 1.0000
Dilution : 1.0000
Use Multiplier & Dilution Factor with ISTDs

Signal 1: VWD1 A, Wavelength=250 nm

Peak #	RetTime [min]	Type	Width [min]	Area [mAU*s]	Height [mAU]	Area %
1	7.544	BV	0.4710	3012.75562	99.14009	49.8692
2	10.472	BB	0.9668	3028.56104	48.53671	50.1308

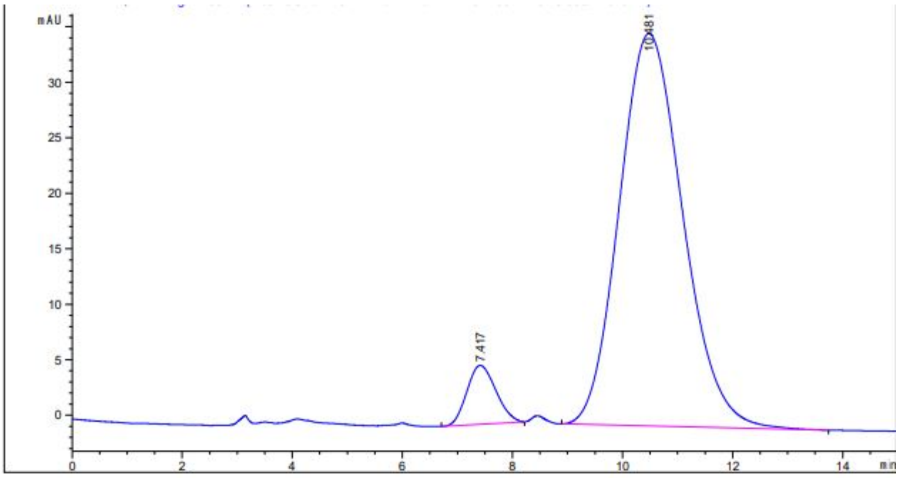


```
=====================================================================
                         Area Percent Report
=====================================================================

Sorted By             :      Signal
Multiplier            :      1.0000
Dilution              :      1.0000
Use Multiplier & Dilution Factor with ISTDs


Signal 1: VWD1 A, Wavelength=250 nm

Peak RetTime Type  Width     Area      Height     Area
  #   [min]        [min]   [mAU*s]     [mAU]        %
----|-------|----|-------|----------|----------|--------|
   1   7.417 BB    0.5705  194.19775    5.31403   6.4034
   2  10.481 BB    1.2251 2838.55054   35.35181  93.5966
```

(*S*)-2-(1-(4-Bromophenyl)aziridin-2-yl)quinoline (44)

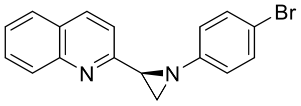

Chemical Formula: $C_{17}H_{13}BrN_2$
Exact Mass: 324.03
Molecular Weight: 325.21

Prepared according to General Procedure B using 2-(1-chlorovinyl)quinoline **1** (47.4 mg, 0.25 mmol, 1.0 equiv.), 4-bromoaniline (43.0 mg, 0.25 mmol, 1.0 equiv.), (*S*)-TCYP (12.4 mg, 12.5 µmol, 5 mol%), 0.6 M LiHMDS in THF (0.5 mL, 0.3 mmol, 1.2 equiv.), and 2-MeTHF (500 µL, 0.5 M). The reaction mixture was subjected to the purification outlined in General Procedure B (silica, EtOAc:cyclohexane, 5:95 to 10:90) to afford the desired product as a pale yellow solid (55.5 mg, 68%). The enantiomeric ratio (95:5) was determined by chiral HPLC in comparison with the racemate.

ν_{max} (film): 1483, 827 cm^{-1}.

^{1}H NMR (500 MHz, $CDCl_3$): δ 8.16 (d, *J* = 8.5 Hz, 1H), 8.10 (d, *J* = 8.5 Hz, 1H), 7.81 (d, *J* = 8.1 Hz, 1H), 7.73 (ddd, *J* = 8.4, 6.9, 1.4 Hz, 1H), 7.56 – 7.50 (m, 1H), 7.43 (d, *J* = 8.5 Hz, 1H), 7.39 – 7.34 (m, 2H), 7.00 – 6.94 (m, 2H), 3.48 (dd, *J* = 6.6, 3.2 Hz, 1H), 2.65 (dd, *J* = 3.2, 1.0 Hz, 1H), 2.56 (dd, *J* = 6.6, 1.0 Hz, 1H).

^{13}C NMR (126 MHz, $CDCl_3$): δ 158.9, 153.1, 147.8, 137.2, 132.1, 129.9, 129.0, 127.8, 127.7, 126.5, 122.6, 117.8, 115.5, 43.4, 37.4.

HRMS (ESI): exact mass calculated for $[M+H]^+$ ($C_{17}H_{14}BrN_2$) requires *m/z* 325.0335, found *m/z* 325.0326.

HPLC analysis using a chiral column: Chiralcel® OJ-H 5 µm column, 1.0 mL/min, 50:50 hexane:*iso*propanol, 250 nm, t_{major} = 12.6 min, t_{minor} = 9.0 min.

$[\alpha]_D^{23}$ = −180° (*c* = 1.0, $CHCl_3$).

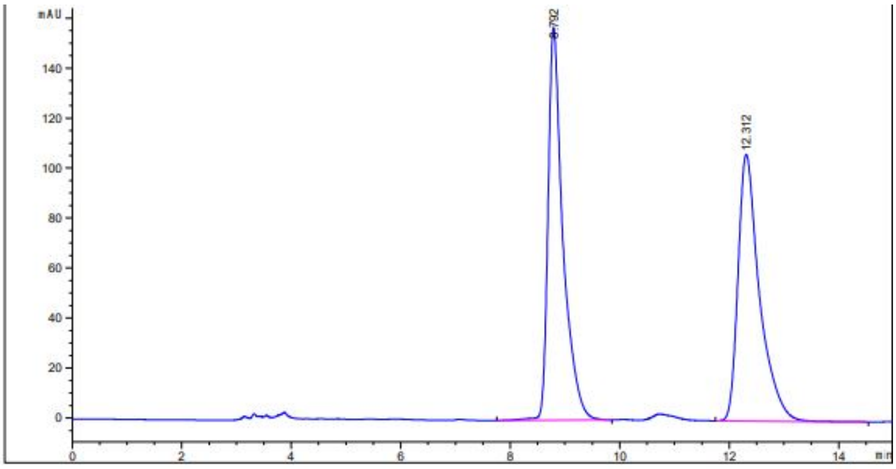

Area Percent Report

Sorted By : Signal
Multiplier : 1.0000
Dilution : 1.0000
Use Multiplier & Dilution Factor with ISTDs

Signal 1: VWD1 A, Wavelength=250 nm

Peak #	RetTime [min]	Type	Width [min]	Area [mAU*s]	Height [mAU]	Area %
1	8.792	BB	0.2759	2970.38843	157.11960	50.5503
2	12.312	BB	0.4008	2905.71851	106.65909	49.4497

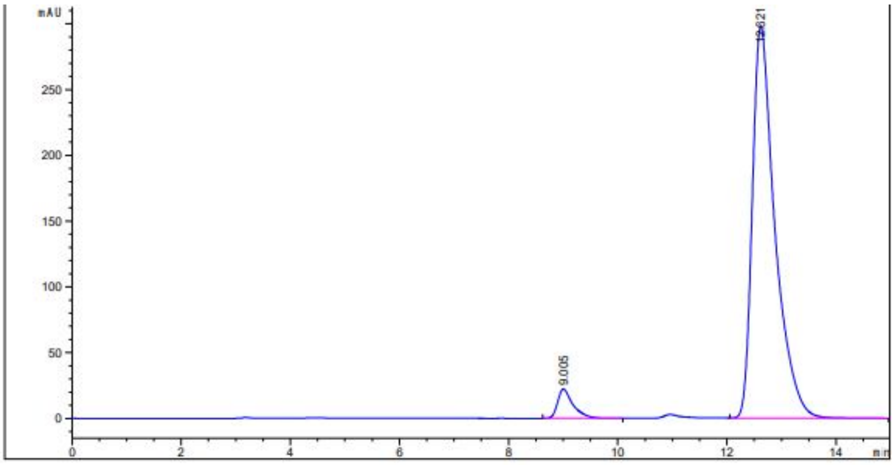

Area Percent Report

Sorted By : Signal
Multiplier : 1.0000
Dilution : 1.0000
Use Multiplier & Dilution Factor with ISTDs

Signal 1: VWD1 A, Wavelength=250 nm

Peak #	RetTime [min]	Type	Width [min]	Area [mAU*s]	Height [mAU]	Area %
1	9.005	BB	0.2843	436.12637	22.36214	4.8316
2	12.621	VBA	0.4266	8590.33594	298.01236	95.1684

(*S*)-2-(1-(3-Fluoro-4-methylphenyl)aziridin-2-yl)quinoline (45)

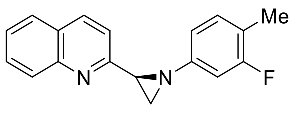

Chemical Formula: $C_{18}H_{15}FN_2$
Exact Mass: 278.1219
Molecular Weight: 278.3304

Prepared according to General Procedure B using 2-(1-chlorovinyl)quinoline **1** (47.4 mg, 0.25 mmol, 1.0 equiv.), 3-fluoro-4-methylaniline (31.3 mg, 0.25 mmol, 1.0 equiv.), (*S*)-TCYP (12.4 mg, 12.5 µmol, 5 mol%), 0.6 M LiHMDS in THF (0.5 mL, 0.3 mmol, 1.2 equiv.), and 2-MeTHF (500 µL, 0.5 M). The reaction mixture was subjected to the purification outlined in General Procedure B (silica, EtOAc:cyclohexane, 5:95 to 10:90) to afford the desired product as a pale yellow solid (43.1 mg, 62%). The enantiomeric ratio (93:7) was determined by chiral HPLC in comparison with the racemate.

ν_{max} (film): 1504, 1101 cm^{-1}.

^{1}H NMR (400 MHz, $CDCl_3$): δ 8.15 (d, *J* = 8.6 Hz, 1H), 8.11 (d, *J* = 8.4 Hz, 1H), 7.81 (dd, *J* = 8.1, 1.1 Hz, 1H), 7.72 (ddd, *J* = 8.4, 6.9, 1.5 Hz, 1H), 7.53 (ddd, *J* = 8.1, 6.9, 1.2 Hz, 1H), 7.44 (d, *J* = 8.5 Hz, 1H), 7.05 (t, *J* = 8.5 Hz, 1H), 6.82 – 6.75 (m, 3H), 3.48 (dd, *J* = 6.6, 3.2 Hz, 1H), 2.62 (dd, *J* = 3.3, 1.1 Hz, 1H), 2.57 (dd, *J* = 6.6, 1.2 Hz, 1H), 2.21 (d, *J* = 1.7 Hz, 3H).

^{13}C NMR (101 MHz, $CDCl_3$): δ 161.4 (d, $^1J_{CF}$ = 244.8 Hz), 159.1, 153.5 (d, $^3J_{CF}$ = 9.3 Hz), 147.8, 137.1, 131.7 (d, $^3J_{CF}$ = 6.4 Hz), 129.8, 129.0, 127.7, 127.7, 126.4, 119.1 (d, $^2J_{CF}$ = 17.5 Hz), 117.8, 116.1 (d, $^4J_{CF}$ = 3.0 Hz), 107.8 (d, $^2J_{CF}$ = 24.6 Hz), 43.5, 37.5, 14.1 (d, $^3J_{CF}$ = 3.2 Hz).

^{19}F NMR (376 MHz, $CDCl_3$): −116.30.

HRMS (ESI): exact mass calculated for $[M+H]^+$ ($C_{18}H_{16}FN_2$) requires *m/z* 279.1292, found *m/z* 279.1282.

HPLC analysis using a chiral column: Chiralcel® OJ-H 5 µm column, 1.0 mL/min, 50:50 hexane:*iso*propanol, 250 nm, t_{major} = 14.8 min, t_{minor} = 16.2 min.

$[\alpha]_D^{23}$ = −147° (*c* = 1.0, $CHCl_3$).

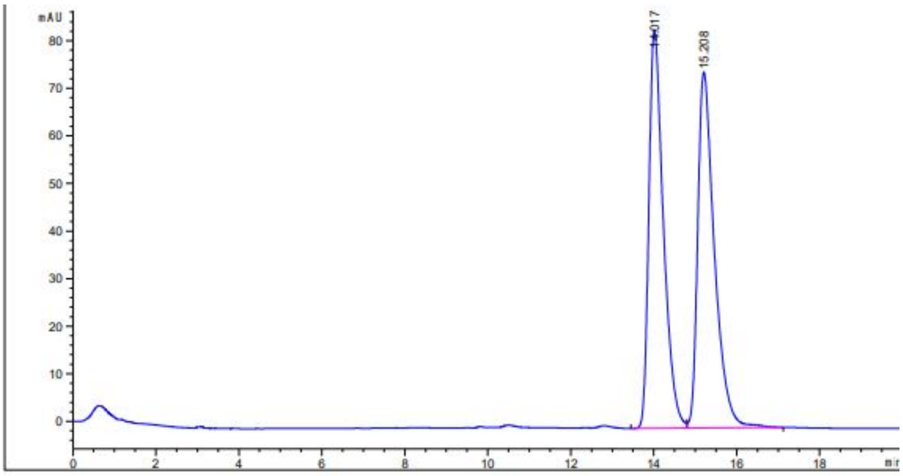

==
Area Percent Report
==

Sorted By : Signal
Multiplier : 1.0000
Dilution : 1.0000
Use Multiplier & Dilution Factor with ISTDs

Signal 1: VWD1 A, Wavelength=250 nm

Peak #	RetTime [min]	Type	Width [min]	Area [mAU*s]	Height [mAU]	Area %
1	14.017	BV	0.3642	2037.29785	83.66129	49.3771
2	15.208	VB	0.4144	2088.69751	74.84177	50.6229

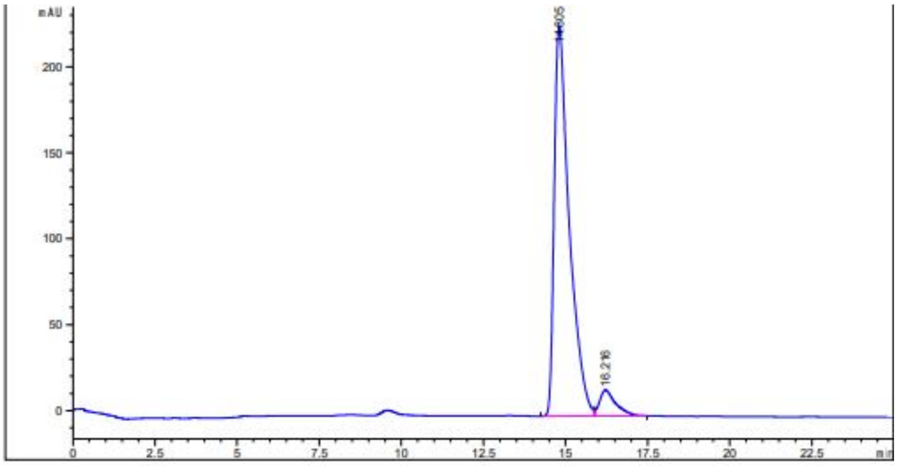

==
Area Percent Report
==

Sorted By : Signal
Multiplier : 1.0000
Dilution : 1.0000
Use Multiplier & Dilution Factor with ISTDs

Signal 1: VWD1 A, Wavelength=250 nm

Peak #	RetTime [min]	Type	Width [min]	Area [mAU*s]	Height [mAU]	Area %
1	14.805	BV	0.4670	7234.38818	226.66731	93.3912
2	16.216	VB	0.4871	511.93591	15.05146	6.6088

(*S*)-2-(1-(6-Fluoropyridin-3-yl)aziridin-2-yl)quinoline (46)

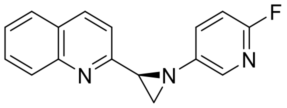

Chemical Formula: $C_{16}H_{12}FN_3$
Exact Mass: 265.10
Molecular Weight: 265.29

Prepared according to General Procedure B using 2-(1-chlorovinyl)quinoline **1** (47.4 mg, 0.25 mmol, 1.0 equiv.), 5-amino-2-fluoropyridine (28.0 mg, 0.25 mmol, 1.0 equiv.), (*S*)-TCYP (12.4 mg, 12.5 µmol, 5 mol%), 0.6 M LiHMDS in THF (0.5 mL, 0.3 mmol, 1.2 equiv.), and CPME (500 µL, 0.5 M). The reaction mixture was subjected to the purification outlined in General Procedure B (silica, EtOAc:cyclohexane, 5:95 to 10:90) to afford the desired product as a pale yellow solid (41.2 mg, 62%). The enantiomeric ratio (89:11) was determined by chiral HPLC in comparison with the racemate.

ν_{max} (film): 1477, 1244, 826 cm^{-1}.

^{1}H NMR (500 MHz, $CDCl_3$): δ 8.17 (d, *J* = 8.5 Hz, 1H), 8.08 (d, *J* = 8.5 Hz, 1H), 7.99 – 7.97 (m, 1H), 7.82 (d, *J* = 8.1 Hz, 1H), 7.75 – 7.70 (m, 1H), 7.57 – 7.47 (m, 2H), 7.42 (d, *J* = 8.5 Hz, 1H), 6.82 (dd, *J* = 8.7, 3.2 Hz, 1H), 3.49 (dd, *J* = 6.6, 3.3 Hz, 1H), 2.73 (app. d, *J* = 3.3 Hz, 1H), 2.59 (app. d, *J* = 6.6 Hz, 1H).

^{13}C NMR (126 MHz, $CDCl_3$): δ 159.6 (d, $^1J_{CF}$ = 234.8 Hz), 158.1, 148.0 (d, $^4J_{CF}$ = 4.2 Hz), 147.8, 139.4 (d, $^3J_{CF}$ = 15.2 Hz), 137.3, 133.7 (d, $^3J_{CF}$ = 7.9 Hz), 130.0, 129.0, 127.8, 127.8, 126.6, 117.8, 109.5 (d, $^2J_{CF}$ = 39.5 Hz), 43.3, 37.2.

^{19}F NMR (126 MHz, $CDCl_3$): δ −75.08.

HRMS (ESI): exact mass calculated for $[M+H]^+$ ($C_{16}H_{13}FN_3$) requires *m/z* 266.1080, found *m/z* 266.1080.

HPLC analysis using a chiral column: Chiralcel® OJ-H 5 µm column, 1.0 mL/min, 50:50 hexane:*iso*propanol, 250 nm, t_{major} = 13.3 min, t_{minor} = 10.0 min.

$[\alpha]_D^{23}$ = −144° (*c* = 1.0, $CHCl_3$)

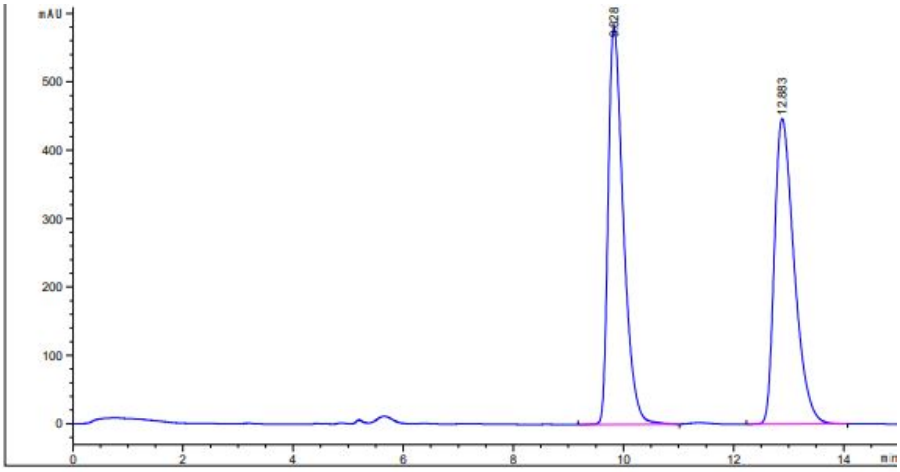

Area Percent Report

Sorted By : Signal
Multiplier : 1.0000
Dilution : 1.0000
Use Multiplier & Dilution Factor with ISTDs

Signal 1: VWD1 A, Wavelength=250 nm

Peak #	RetTime [min]	Type	Width [min]	Area [mAU*s]	Height [mAU]	Area %
1	9.828	BV	0.2916	1.11195e4	581.30688	49.9746
2	12.883	BB	0.3839	1.11307e4	446.66437	50.0254

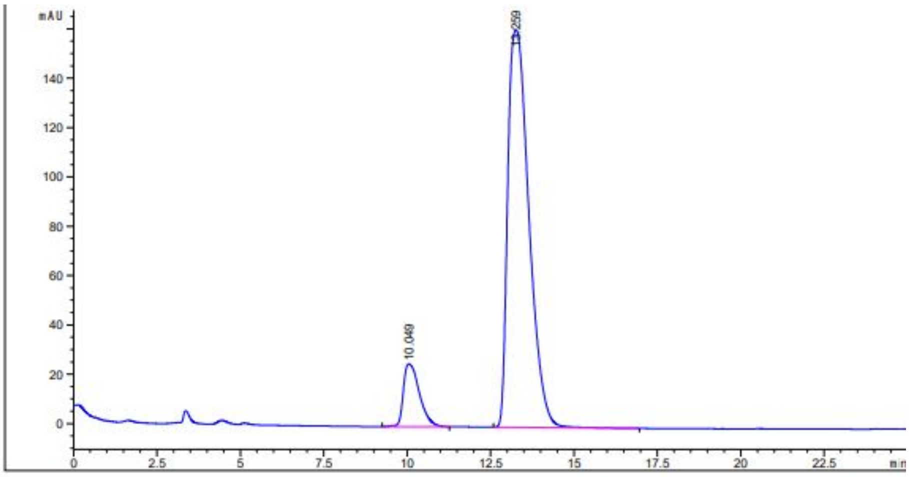


```
=====================================================================
                    Area Percent Report
=====================================================================

Sorted By             :      Signal
Multiplier            :      1.0000
Dilution              :      1.0000
Use Multiplier & Dilution Factor with ISTDs


Signal 1: VWD1 A, Wavelength=250 nm
```

Peak #	RetTime [min]	Type	Width [min]	Area [mAU*s]	Height [mAU]	Area %
1	10.049	BB	0.5187	849.50165	25.37230	10.7217
2	13.259	BB	0.6839	7073.71240	161.15097	89.2783

(*S*)-2-(1-(Benzo[*b*]thiophen-5-yl)aziridin-2-yl)quinoline (47)

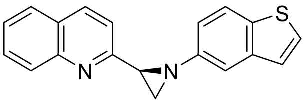

Chemical Formula: $C_{19}H_{14}N_2S$
Exact Mass: 302.09
Molecular Weight: 302.40

Prepared according to General Procedure B using 2-(1-chlorovinyl)quinoline **1** (47.4 mg, 0.25 mmol, 1.0 equiv.), benzo[*b*]thiophen-5-amine (37.3 mg, 0.25 mmol, 1.0 equiv.), (*S*)-TCYP (12.4 mg, 12.5 µmol, 5 mol%), 0.6 M LiHMDS in THF (0.5 mL, 0.3 mmol, 1.2 equiv.), and 2-MeTHF (500 µL, 0.5 M). The reaction mixture was subjected to the purification outlined in General Procedure B (silica, EtOAc:cyclohexane, 5:95 to 10:90) to afford the desired product as a pale yellow solid (37.3 mg, 49%). The enantiomeric ratio (95:5) was determined by chiral HPLC in comparison with the racemate.

ν_{max} (film): 1597, 1502, 1421 cm^{-1}.

^{1}H NMR (400 MHz, $CDCl_3$): δ 8.17 (d, *J* = 8.6 Hz, 1H), 8.13 (d, *J* = 8.4 Hz, 1H), 7.82 (dd, *J* = 8.2, 1.1 Hz, 1H), 7.74 (t, *J* = 7.7 Hz, 2H), 7.57 – 7.48 (m, 3H), 7.44 (d, *J* = 5.4 Hz, 1H), 7.26 – 7.21 (m, 1H), 7.19 (dd, *J* = 8.6, 2.0 Hz, 1H), 3.58 (dd, *J* = 6.5, 3.2 Hz, 1H), 2.69 (dd, *J* = 3.2, 1.2 Hz, 1H), 2.67 (dd, *J* = 6.6, 1.2 Hz, 1H).

^{13}C NMR (101 MHz, $CDCl_3$): δ 159.5, 151.2, 147.8, 140.4, 137.1, 134.4, 129.8, 129.0, 127.8, 127.7, 127.6, 126.4, 123.5, 123.0, 119.2, 117.9, 114.3, 43.6, 37.7.

HRMS (ESI): exact mass calculated for $[M+H]^+$ ($C_{19}H_{15}N_2S$) requires *m/z* 303.0950, found *m/z* 303.0943.

HPLC analysis using a chiral column: Chiralcel® OJ-H 5 µm column, 1.0 mL/min, 50:50 hexane:*iso*propanol, 250 nm, t_{major} = 21.7 min, t_{minor} = 16.7 min.

$[\alpha]_D^{23}$ = −181° (c = 1.0, $CHCl_3$).

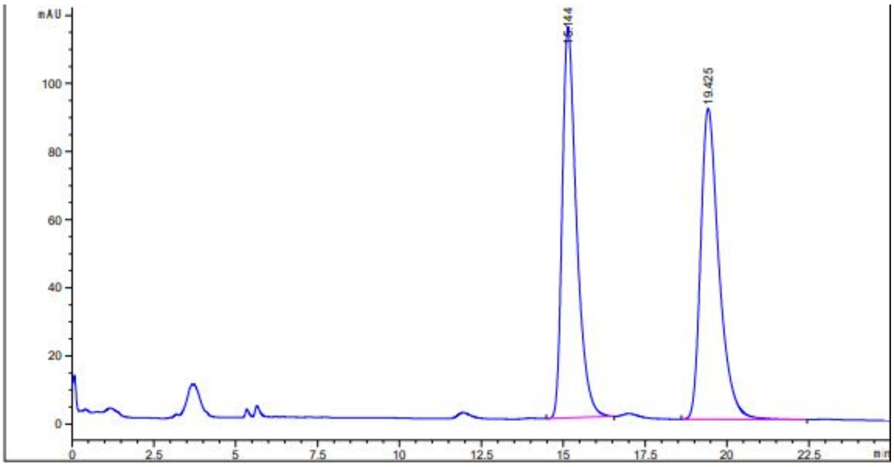


```
=====================================================================
                         Area Percent Report
=====================================================================

Sorted By             :      Signal
Multiplier            :      1.0000
Dilution              :      1.0000
Use Multiplier & Dilution Factor with ISTDs


Signal 1: VWD1 A, Wavelength=250 nm

Peak RetTime Type  Width     Area       Height      Area
  #   [min]        [min]    [mAU*s]     [mAU]         %
----|-------|----|-------|----------|----------|--------|
   1  15.144 BB    0.4559 3483.86987  114.83427  49.5592
   2  19.425 BB    0.5853 3545.84351   91.35070  50.4408
```

`

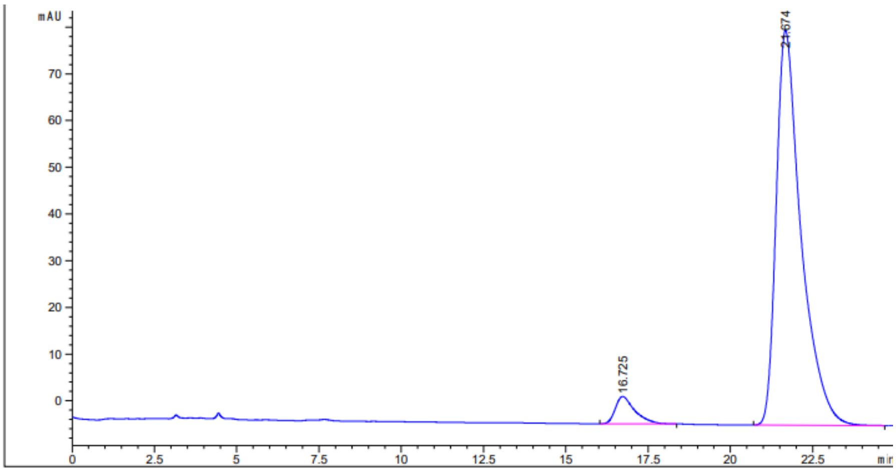


```
=====================================================================
                         Area Percent Report
=====================================================================

Sorted By             :      Signal
Multiplier            :      1.0000
Dilution              :      1.0000
Use Multiplier & Dilution Factor with ISTDs


Signal 1: VWD1 A, Wavelength=250 nm

Peak RetTime Type  Width     Area       Height      Area
  #   [min]        [min]    [mAU*s]     [mAU]         %
----|-------|----|-------|----------|----------|--------|
   1  16.725 BB    0.6031  240.30994    5.84907   5.1225
   2  21.674 BB    0.7744 4450.96240   84.54128  94.8775
```

(*R*)-*N*-(2-Chloro-2-(6-fluoroquinolin-2-yl)ethyl)aniline (48)

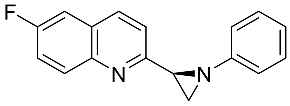

Chemical Formula: $C_{17}H_{13}FN_2$
Exact Mass: 264.1063
Molecular Weight: 264.3034

Prepared according to General Procedure B using 2-(1-chlorovinyl)-6-fluoroquinoline **S35** (51.9 mg, 0.25 mmol, 1.0 equiv.), aniline (23 µL, 0.25 mmol, 1.0 equiv.), (*S*)-TCYP (12.4 mg, 12.5 µmol, 5 mol%), 0.6 M LiHMDS in THF (0.5 mL, 0.3 mmol, 1.2 equiv.), and 2-MeTHF (500 µL, 0.5 M). The reaction mixture was subjected to the purification outlined in General Procedure B (silica, EtOAc:cyclohexane, 5:95 to 10:90) to afford the desired product as a pale yellow solid (21.4 mg, 32%). The enantiomeric ratio (95:5) was determined by chiral HPLC in comparison with the racemate.

ν_{max} (film): 1597, 1489, 1234 cm^{-1}.

^{1}H NMR (500 MHz, $CDCl_3$): δ 8.15 – 8.10 (m, 2H), 7.55 – 7.48 (m, 2H), 7.45 (dd, *J* = 8.8, 2.9 Hz, 1H), 7.33 – 7.27 (m, 2H), 7.16 – 7.10 (m, 2H), 7.05 (tt, *J* = 7.7, 1.2 Hz, 1H), 3.52 (dd, *J* = 6.5, 3.2 Hz, 1H), 2.66 – 2.62 (m, 2H).

^{13}C NMR (126 MHz, $CDCl_3$): δ 160.3 (d, $^1J_{CF}$ = 247.7 Hz), 158.8, 153.8, 144.8, 136.3 (d, $^4J_{CF}$ = 5.3 Hz), 131.4 (d, $^3J_{CF}$ = 9.1 Hz), 129.1, 128.2 (d, $^3J_{CF}$ = 10.0 Hz), 122.9, 120.6, 119.9 (d, $^2J_{CF}$ = 25.6 Hz), 118.6, 110.7 (d, $^2J_{CF}$ = 21.6 Hz), 42.9, 37.2.

^{19}F NMR (376 MHz, $CDCl_3$): δ −113.86.

HRMS (ESI): exact mass calculated for $[M+H]^+$ ($C_{17}H_{14}FN_2$) requires *m/z* 265.1136, found *m/z* 265.1131.

HPLC analysis using a chiral column: Chiralcel® OJ-H 5 µm column, 1.0 mL/min, 90:10 hexane:*iso*propanol, 250 nm, t_{major} = 25.4 min, t_{minor} = 28.4 min.

$[\alpha]_D^{23}$ = −154° (*c* = 0.5, $CHCl_3$).

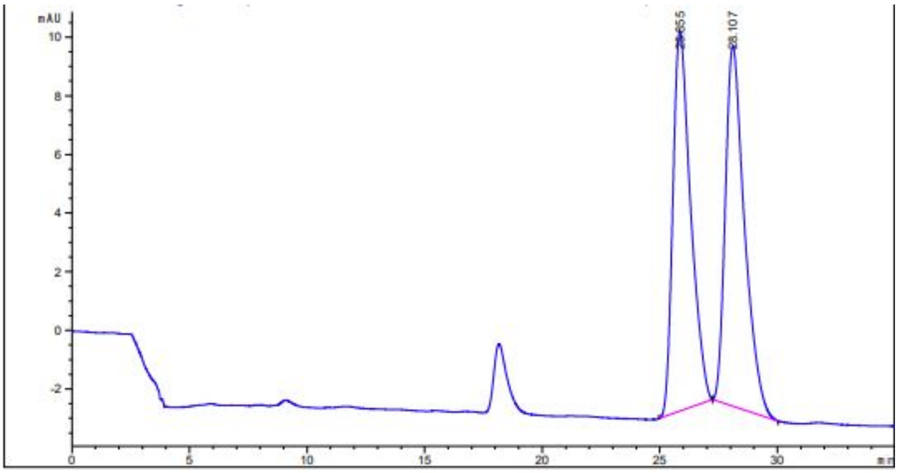


```
=====================================================================
                         Area Percent Report
=====================================================================

Sorted By             :      Signal
Multiplier            :      1.0000
Dilution              :      1.0000
Use Multiplier & Dilution Factor with ISTDs


Signal 1: VWD1 A, Wavelength=250 nm

Peak RetTime Type  Width     Area       Height     Area
  #   [min]        [min]   [mAU*s]      [mAU]        %
----|-------|----|-------|----------|----------|--------|
   1  25.855 BB    0.7868  679.63800   12.95368  49.3747
   2  28.107 BB    0.8267  696.85138   12.27798  50.6253
```

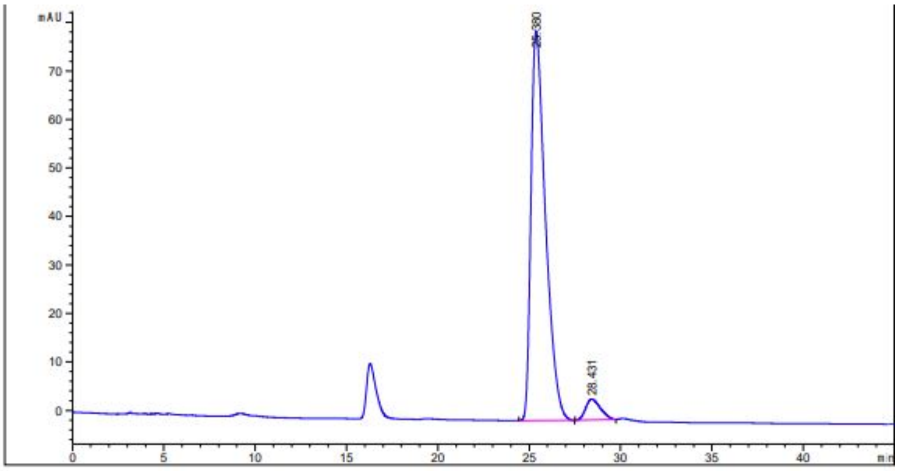

Area Percent Report

```
Sorted By             :      Signal
Multiplier            :      1.0000
Dilution              :      1.0000
Use Multiplier & Dilution Factor with ISTDs
```

Signal 1: VWD1 A, Wavelength=250 nm

Peak #	RetTime [min]	Type	Width [min]	Area [mAU*s]	Height [mAU]	Area %
1	25.380	BB	0.8235	4426.71533	80.32028	94.8030
2	28.431	BB	0.8129	242.66660	4.33755	5.1970

(*S*)-5-Chloro-6-methoxy-2-(1-phenylaziridin-2-yl)quinoline (49)

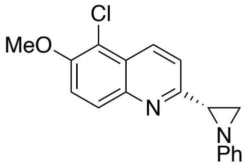

Chemical Formula: $C_{18}H_{15}ClN_2O$
Exact Mass: 310.0873
Molecular Weight: 310.7810

A 2 mL HPLC vial, equipped with a stir-bar, was charged with (*R*)-*N*-(2-chloro-2-(5-chloro-6-methoxyquinolin-2-yl)ethyl)aniline **27** (34.7 mg, 0.10 mmol, 1.0 equiv.) and dissolved in 2MeTHF (200 µL, 0.5 M). The suspension was treated with 0.6 M LiHMDS in THF (0.2 mL, 0.120 mmol, 1.2 equiv.) dropwise at room temperature. The red reaction was allowed to stir for 30 min. The reaction was then quenched by addition of sat. aq. NH_4Cl and extracted with EtOAc (3x). The organics were combined, dried over Na_2SO_4, filtered, and had the solvent concentrated *in vacuo* to give a yellow residue. The crude product was purified using flash column chromatography (silica, EtOAc:hexane, 20:80) to afford the desired product as a yellow oil (21.2 mg, 68%). The enantiomeric ratio (97:3) was determined by chiral HPLC in comparison with the racemate.

ν_{max}(film): 2959, 2359, 2340, 1593, 1489, 1269, cm^{-1}.

^{1}H NMR (400 MHz, $CDCl_3$): δ 8.52 (d, *J* = 8.9 Hz, 1H), 8.05 (dd, *J* = 9.3, 0.8 Hz, 1H), 7.53 (dd, *J* = 9.1, 1.6 Hz, 2H), 7.31 – 7.23 (m, 2H), 7.15 – 7.07 (m, 2H), 7.05 – 6.98 (m, 1H), 4.06 (s, 3H), 3.53 – 3.43 (m, 1H), 2.69 – 2.57 (m, 2H).

^{13}C NMR (101 MHz, $CDCl_3$): δ 157.8, 153.7, 152.5, 143.4, 132.8, 129.1, 128.8, 126.6, 122.9, 120.6, 119.0, 116.6, 116.0, 56.9, 42.7, 37.1.

HRMS (ESI): exact mass calculated for $[M+H]^+$ ($C_{18}H_{16}ClN_2O$) requires *m/z* 311.0951, found *m/z* 311.0939.

HPLC analysis using a chiral column: Amylose-C 5 µm column, 1.0 mL/min, 80:20 hexane:*iso*propanol, 250 nm, t_{major} = 7.1 min, t_{minor} = 7.9 min.

$[\alpha]_D^{23}$ = −177° (*c* = 1.0, $CHCl_3$).

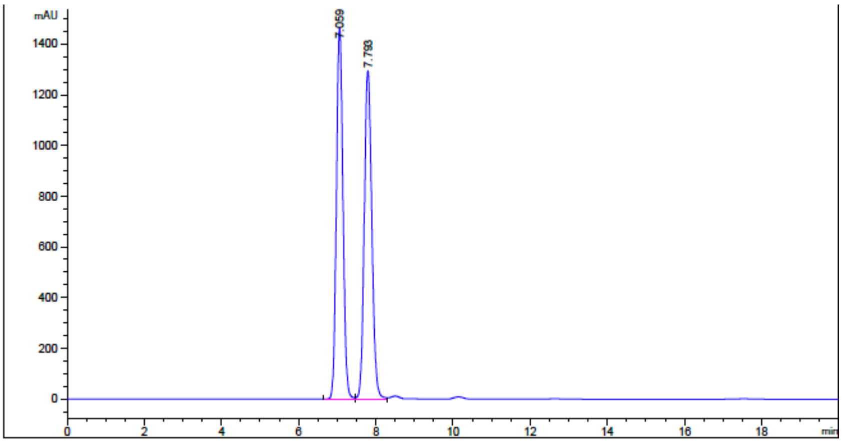

```
=====================================================================
                         Area Percent Report
=====================================================================

Sorted By             :      Signal
Multiplier            :      1.0000
Dilution              :      1.0000
Use Multiplier & Dilution Factor with ISTDs


Signal 1: VWD1 A, Wavelength=250 nm

Peak RetTime Type  Width     Area      Height     Area
  #   [min]        [min]   [mAU*s]     [mAU]        %
----|-------|----|-------|----------|----------|--------|
   1   7.059 BV    0.1844 1.72811e4  1463.10754  49.5723
   2   7.793 VV    0.2125 1.75793e4  1292.94360  50.4277

Totals :                  3.48604e4  2756.05115
```

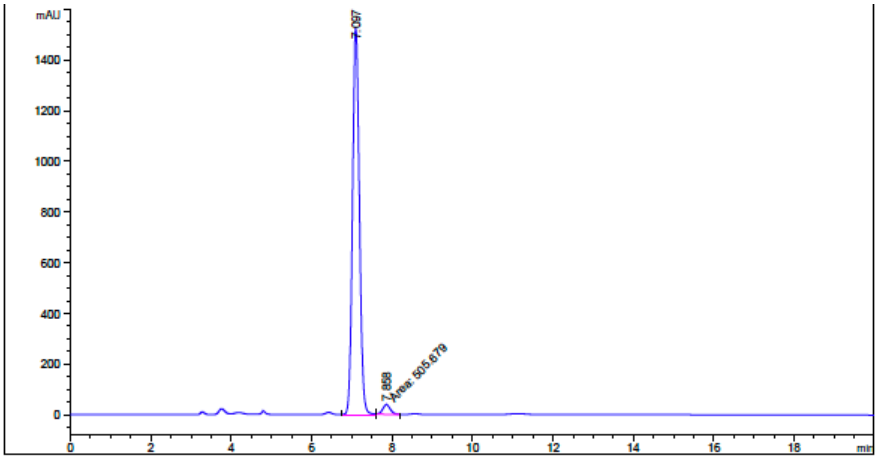

```
=====================================================================
                         Area Percent Report
=====================================================================

Sorted By             :      Signal
Multiplier            :      1.0000
Dilution              :      1.0000
Use Multiplier & Dilution Factor with ISTDs


Signal 1: VWD1 A, Wavelength=250 nm

Peak RetTime Type  Width     Area      Height     Area
  #   [min]        [min]   [mAU*s]     [mAU]        %
----|-------|----|-------|----------|----------|--------|
   1   7.097 BV    0.1895 1.82978e4  1525.29932  97.3107
   2   7.858 MM    0.2182  505.67947   38.62575   2.6893

Totals :                  1.88034e4  1563.92507
```

(*S*)-6-(1-{Phenylaziridin-2-yl)phenanthridine (50)

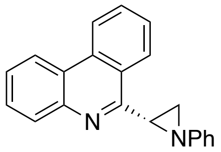

Chemical Formula: $C_{21}H_{16}N_2$
Exact Mass: 296.1313
Molecular Weight: 296.3730

A 2 mL HPLC vial, equipped with a stir-bar, was charged with (*R*)-*N*-(2-chloro-2-(phenanthridin-6-yl)ethyl)aniline **28** (18.0 mg, 0.0541 mmol) and dissolved in toluene (270 µL). The suspension was treated dropwise with LiHMDS (108 µL, 0.0649 mmol, 0.6 M in Toluene) at RT. The red reaction mixture was allowed to stir for 30 min. The reaction was then quenched by addition of sat. aq. NH_4Cl and extracted with EtOAc (3x). The organic phases were combined, dried over Na_2SO_4, filtered, and had the solvent concentrated *in vacuo* to give a yellow residue. The crude product was purified using flash column chromatography (silica, EtOAc:hexane, 10:90) to afford the desired product as a yellow oil (12.3 mg, 77%). The enantiomeric ratio (51:49) was determined by chiral HPLC in comparison with the racemate.

ν_{max}(film): 3063, 2959, 1595, 1489, 1300 cm^{-1}.

^{1}H NMR (400 MHz, $CDCl_3$) δ 8.71 (d, *J* = 8.3 Hz, 1H), 8.64 – 8.54 (m, 2H), 8.24 (dd, *J* = 8.5, 1.3 Hz, 1H), 7.90 (ddd, *J* = 8.3, 7.0, 1.3 Hz, 1H), 7.81 – 7.69 (m, 2H), 7.66 (ddd, *J* = 8.3, 7.0, 1.4 Hz, 1H), 7.34 – 7.28 (m, 2H), 7.24 – 7.20 (m, 2H), 7.04 (tt, *J* = 7.2, 1.2 Hz, 1H), 4.02 (dd, *J* = 6.5, 3.4 Hz, 1H), 3.17 (dd, *J* = 3.4, 1.6 Hz, 1H), 2.70 (dd, *J* = 6.4, 1.6 Hz, 1H).

^{13}C NMR (126 MHz, $CDCl_3$) δ 156.3, 154.4, 143.7, 133.0, 130.6, 130.4, 129.0, 128.7, 127.5, 126.9, 125.6, 125.3, 123.8, 122.8, 122.7, 121.9, 121.0, 40.5, 35.3.

HRMS (ESI): exact mass calculated for $[M+H]^+$ ($C_{21}H_{17}N_2$) requires *m/z* 297.1392, found *m/z* 297.1378.

HPLC analysis using a chiral column: Chiralcel® OJ-H 5 µm column, 0.5 mL/min, 90:10 hexane:*iso*propanol, 250 nm, t_{major} = 16.4 min, t_{minor} = 18.3min.

$[\alpha]_D^{23}$ = – 5.6° (*c* = 1.0, $CHCl_3$).

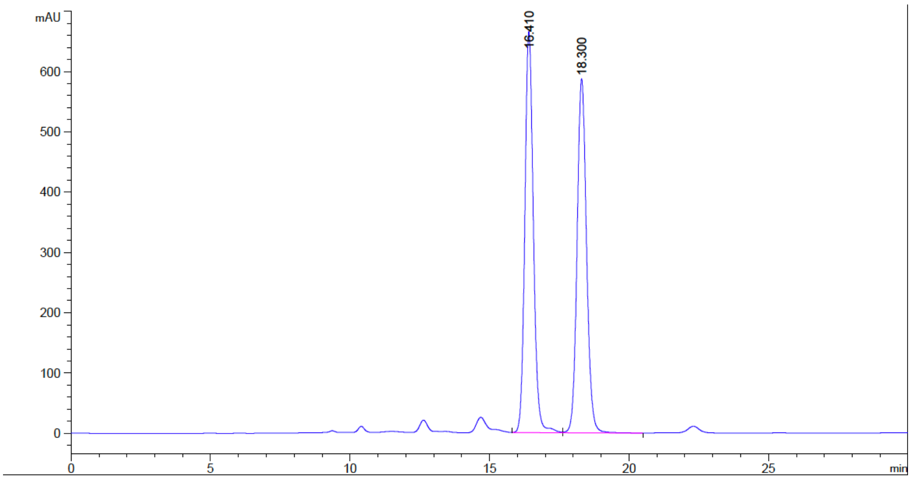

==

Area Percent Report

==

Sorted By : Signal
Multiplier : 1.0000
Dilution : 1.0000
Use Multiplier & Dilution Factor with ISTDs

Signal 1: VWD1 A, Wavelength=250 nm

Peak #	RetTime [min]	Type	Width [min]	Area [mAU*s]	Height [mAU]	Area %
1	16.410	BV	0.3262	1.41339e4	666.12408	50.5679
2	18.300	VB	0.3618	1.38165e4	587.27960	49.4321

Totals : 2.79504e4 1253.40369

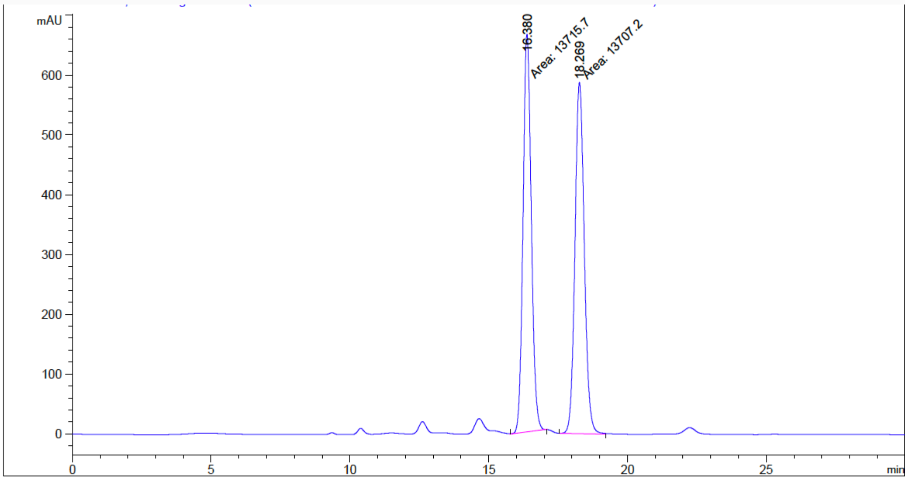

==

Area Percent Report

==

Sorted By : Signal
Multiplier : 1.0000
Dilution : 1.0000
Use Multiplier & Dilution Factor with ISTDs

Signal 1: VWD1 A, Wavelength=250 nm

Peak #	RetTime [min]	Type	Width [min]	Area [mAU*s]	Height [mAU]	Area %
1	16.380	MM	0.3436	1.37157e4	665.26233	50.0155
2	18.269	MM	0.3888	1.37072e4	587.63202	49.9845

Totals : 2.74230e4 1252.89435

(*S*)-2-(1-Phenylaziridin-2-yl)quinoxaline (51)

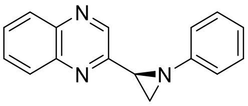

Chemical Formula: $C_{16}H_{13}N_3$
Exact Mass: 247.1109
Molecular Weight: 247.3010

A 2 mL HPLC vial was charged with (*R*)-*N*-(2-chloro-2-(quinoxalin-2-yl)ethyl)aniline **31** (25.0 mg, 0.088 mmol, 1.0 equiv.). The vial was then capped and purged with N_2 before addition of toluene (180 µL, 0.5 M). The reaction mixture was cooled to −20 °C for 15 min before the addition of a 0.6 M solution of LiHMDS in toluene (0.180 mL, 0.106 mmol, 1.2 equiv.), and the reaction was allowed to stir for a further 30 min before being quenched by sat. aq. NH_4Cl solution (2 mL) and diluted with EtOAc (2 mL). The phases were separated and the aqueous phase was washed with EtOAc (2 mL × 2). The organic phases were combined, dried over Na_2SO_4, filtered, and concentrated *in vacuo*. Purification of the crude using flash column chromatography (silica, EtOAc:petroleum ether, 5:95 to 10:90) gave the desired product as a yellow oil (20.1 mg, 92%). The enantiomeric ratio (91:9) was determined by chiral HPLC in comparison with the racemate.

ν_{max} (film): 1599, 1491 cm^{-1}.

^{1}H NMR (500 MHz, $CDCl_3$): δ 8.92 (s, 1H), 8.18 – 8.08 (m, 2H), 7.83 – 7.72 (m, 2H), 7.34 – 7.28 (m, 2H), 7.14 – 7.09 (m, 2H), 7.08 – 7.02 (m, 1H), 3.53 (dd, *J* = 6.5, 3.2 Hz, 1H), 2.78 (dd, *J* = 3.1, 1.2 Hz, 1H), 2.68 (dd, *J* = 6.5, 1.2 Hz, 1H).

^{13}C NMR (126 MHz, $CDCl_3$): δ 153.8, 153.5, 143.4, 142.3, 141.9, 130.4, 129.7, 129.4, 129.3, 129.1, 123.3, 120.8, 41.5, 37.3.

HRMS (ESI): exact mass calculated for $[M+H]^+$ ($C_{16}H_{14}N_3$) requires *m/z* 248.1182, found *m/z* 248.1175.

HPLC analysis using a chiral column: Chiralcel® OJ-H 5 µm column, 1.0 mL/min, 50:50 hexane:*iso*propanol, 250 nm, t_{major} = 8.4 min, t_{minor} = 7.9 min.

$[\alpha]_D^{23}$ = −125° (*c* = 1.0, $CHCl_3$).

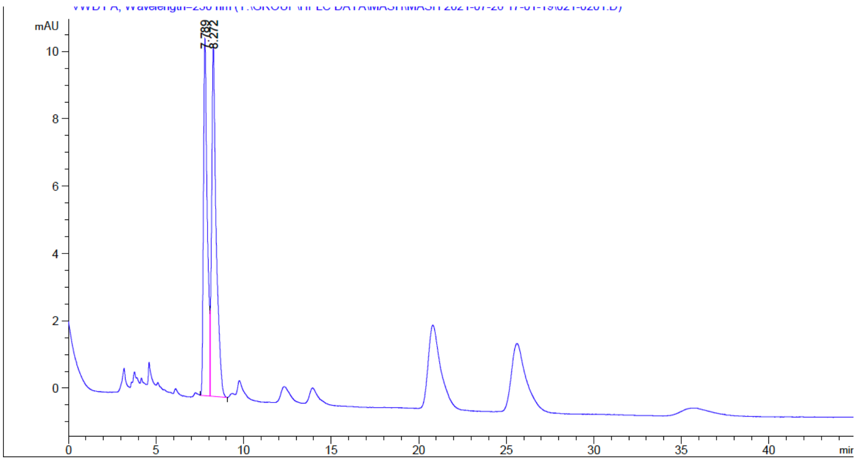

Area Percent Report

Sorted By	:	Signal
Multiplier	:	1.0000
Dilution	:	1.0000

Use Multiplier & Dilution Factor with ISTDs

Signal 1: VWD1 A, Wavelength=250 nm

Peak #	RetTime [min]	Type	Width [min]	Area [mAU*s]	Height [mAU]	Area %
1	7.789	BV	0.2205	160.91895	10.61583	45.6598
2	8.272	VB	0.2619	191.51125	10.37679	54.3402

Totals : 352.43019 20.99263

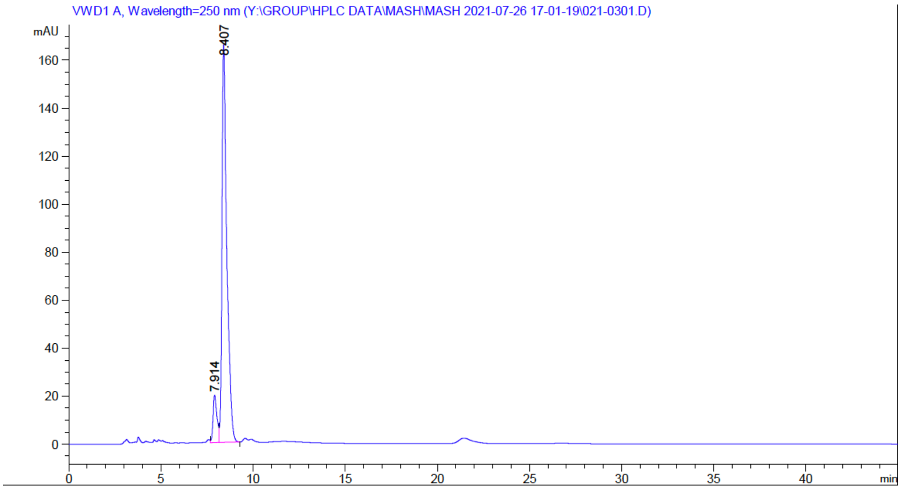

Area Percent Report

Sorted By	:	Signal
Multiplier	:	1.0000
Dilution	:	1.0000

Use Multiplier & Dilution Factor with ISTDs

Signal 1: VWD1 A, Wavelength=250 nm

Peak #	RetTime [min]	Type	Width [min]	Area [mAU*s]	Height [mAU]	Area %
1	7.914	VV	0.2189	295.32410	19.82631	8.8900
2	8.407	VB	0.2640	3026.66382	165.77171	91.1100

Totals : 3321.98792 185.59803

(*S*)-6-(1-Phenylaziridin-2-yl)nicotinonitrile (52)

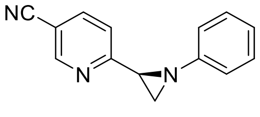

Chemical Formula: $C_{14}H_{11}N_3$
Exact Mass: 221.0953
Molecular Weight: 221.2630

Prepared according to General Procedure B using 6-(1-chlorovinyl)nicotinonitrile **S42** (51.9 mg, 0.25 mmol, 1.0 equiv.), aniline (23 µL, 0.25 mmol, 1.0 equiv.), (*S*)-TCYP (12.4 mg, 12.5 µmol, 5 mol%), 0.6 M LiHMDS in THF (0.5 mL, 0.3 mmol, 1.2 equiv.), and 2-MeTHF (500 µL, 0.5 M). The reaction mixture was subjected to the purification outlined in General Procedure B (silica, EtOAc:cyclohexane, 5:95 to 10:90) to afford the desired product as a pale yellow solid (21.4 mg, 39%). The enantiomeric ratio (95:5) was determined by chiral HPLC in comparison with the racemate.

ν_{max} (film): 2231, 1593, 1489 cm^{-1}.

^{1}H NMR (500 MHz, $CDCl_3$): δ 8.84 (dd, *J* = 2.1, 0.9 Hz, 1H), 7.94 (dd, *J* = 8.2, 2.1 Hz, 1H), 7.51 (dd, *J* = 8.1, 0.9 Hz, 1H), 7.37 – 7.20 (m, 2H), 7.06 – 6.96 (m, 3H), 3.36 (dd, *J* = 6.5, 3.1 Hz, 1H), 2.60 (dd, *J* = 6.5, 1.4 Hz, 1H), 2.56 (dd, *J* = 3.1, 1.4 Hz, 1H).

^{13}C NMR (126 MHz, $CDCl_3$): δ 163.6, 153.2, 152.1, 140.0, 129.3, 123.4, 120.6, 120.4, 116.9, 108.5, 42.3, 38.1.

HRMS (ESI): exact mass calculated for $[M+H]^+$ ($C_{14}H_{12}N_3$) requires *m/z* 222.1026, found *m/z* 222.1026.

HPLC analysis using a chiral column: Chiralcel® OJ-H 5 µm column, 1.0 mL/min, 50:50 hexane:isopropanol, 250 nm, t_{major} = 66.9 min, t_{minor} = 28.2 min.

$[\alpha]_D^{23}$ = −249° (*c* = 0.50, $CHCl_3$).

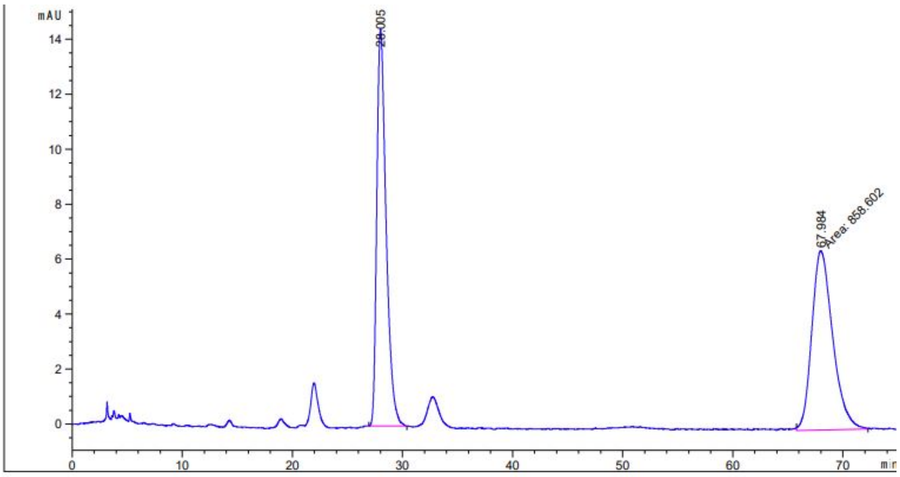


```
=====================================================================
                         Area Percent Report
=====================================================================

Sorted By             :      Signal
Multiplier            :      1.0000
Dilution              :      1.0000
Use Multiplier & Dilution Factor with ISTDs


Signal 1: VWD1 A, Wavelength=250 nm

Peak RetTime Type  Width     Area      Height     Area
  #   [min]        [min]   [mAU*s]     [mAU]        %
----|-------|----|-------|----------|----------|--------|
   1  28.005 BB    0.8890  846.41907   14.44311  49.6427
   2  67.984 MM    2.1959  858.60181    6.51674  50.3573
```

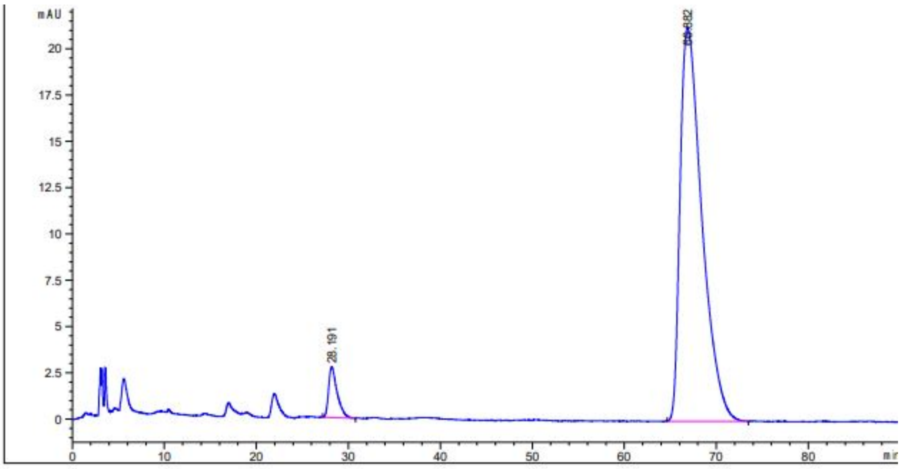


```
=====================================================================
                      Area Percent Report
=====================================================================

Sorted By            :      Signal
Multiplier           :      1.0000
Dilution             :      1.0000
Use Multiplier & Dilution Factor with ISTDs


Signal 1: VWD1 A, Wavelength=254 nm

Peak RetTime Type  Width     Area      Height     Area
  #   [min]        [min]   [mAU*s]     [mAU]        %
----|-------|----|-------|----------|----------|--------|
   1  28.191 BB    0.9408  177.28491    2.74143   4.8212
   2  66.882 BB    2.4293 3499.91724   21.23833  95.1788
```

(*S*)-2-(1-Phenylaziridin-2-yl)benzo[*d*]thiazole (53)

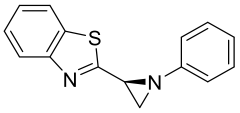

Chemical Formula: $C_{15}H_{12}N_2S$
Exact Mass: 252.0721
Molecular Weight: 252.3350

A 2 mL HPLC vial was charged with (*R*)-*N*-(2-(benzo[*d*]thiazol-2-yl)-2-chloroethyl)aniline **35**(11.2 mg, 0.039 mmol, 1.0 equiv.). The vial was then capped and purged with N_2 before addition of toluene (78 µL, 0.5 M). The reaction mixture was cooled to −20 °C for 15 min before the addition of a 0.6 M solution of LiHMDS in toluene (0.078 mL, 0.047 mmol, 1.2 equiv.), and the reaction was allowed to stir for a further 30 min before being quenched by sat. aq. NH_4Cl solution (2 mL) and diluted with EtOAc (2 mL). The phases were separated and the aqueous phase was washed with EtOAc (2 mL × 2). The organic phases were combined, dried over Na_2SO_4, filtered, and concentrated *in vacuo*. Purification of the crude using flash column chromatography (silica, EtOAc:petroleum ether, 5:95 to 10:90) gave the desired product as a pale yellow solid (6.7 mg, 68%). The enantiomeric ratio (94:6) was determined by chiral HPLC in comparison with the racemate.

ν_{max} (film): 1597, 1489 cm^{-1}.

^{1}H NMR (500 MHz, CDCl$_3$): δ 8.03 (d, *J* = 8.1 Hz, 1H), 7.93 – 7.89 (m, 1H), 7.51 (ddd, *J* = 8.3, 7.3, 1.2 Hz, 1H), 7.41 (td, *J* = 7.7, 7.3, 1.1 Hz, 1H), 7.32 (td, *J* = 7.4, 1.9 Hz, 2H), 7.13 (dd, *J* = 8.5, 1.1 Hz, 2H), 7.10 – 7.05 (m, 1H), 3.68 (dd, *J* = 6.3, 3.0 Hz, 1H), 2.71 (dd, *J* = 3.0, 1.2 Hz, 1H), 2.69 (dd, *J* = 6.4, 1.2 Hz, 1H).

^{13}C NMR (126 MHz, CDCl$_3$): δ 172.2, 153.7, 152.5, 134.7, 129.3, 126.2, 125.1, 123.5, 122.7, 121.9, 120.5, 40.0, 38.7.

HRMS (ESI): exact mass calculated for [M+H]$^+$ ($C_{15}H_{13}N_2S$) requires *m/z* 253.0794, found *m/z* 253.0796.

HPLC analysis using a chiral column: Chiralcel® OJ-H 5 µm column, 1.0 mL/min, 50:50 hexane:*iso*propanol, 250 nm, t_{major} = 13.9 min, t_{minor} = 12.2 min.

$[\alpha]_D^{23}$ = −233° (*c* = 0.2, $CHCl_3$).

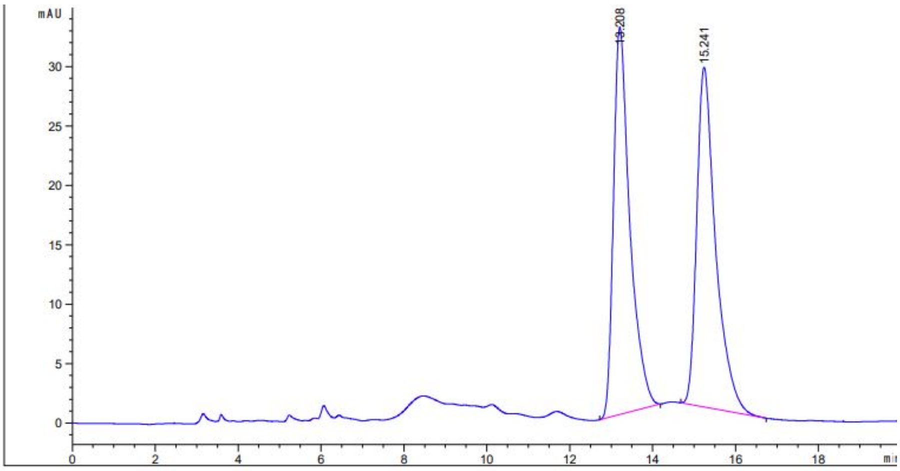


```
=====================================================================
                        Area Percent Report
=====================================================================

Sorted By             :      Signal
Multiplier            :      1.0000
Dilution              :      1.0000
Use Multiplier & Dilution Factor with ISTDs


Signal 1: VWD1 A, Wavelength=250 nm

Peak RetTime Type  Width     Area      Height     Area
  #   [min]        [min]   [mAU*s]     [mAU]        %
----|-------|----|-------|----------|----------|--------|
   1  13.208 BB    0.4066  895.79700   32.58640  49.7515
   2  15.241 BB    0.4683  904.74438   28.58516  50.2485
```

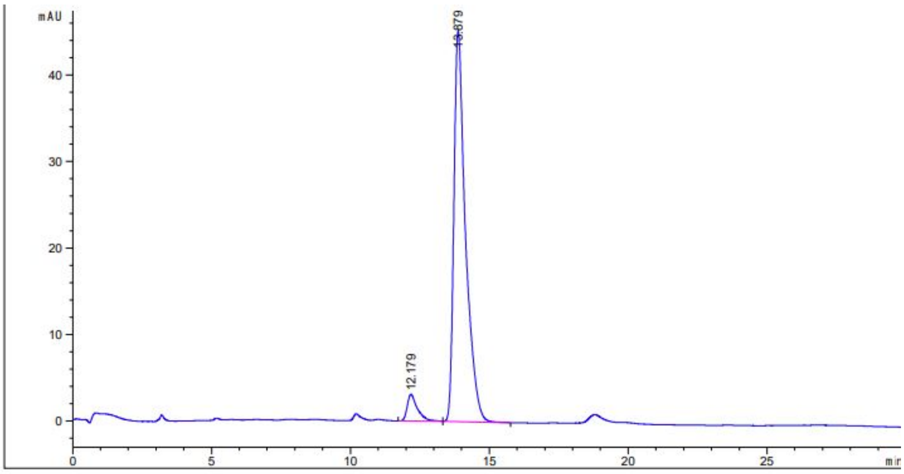


```
=====================================================================
                        Area Percent Report
=====================================================================

Sorted By             :      Signal
Multiplier            :      1.0000
Dilution              :      1.0000
Use Multiplier & Dilution Factor with ISTDs


Signal 1: VWD1 A, Wavelength=250 nm

Peak RetTime Type  Width     Area      Height     Area
  #   [min]        [min]   [mAU*s]     [mAU]        %
----|-------|----|-------|----------|----------|--------|
   1  12.179 BB    0.3665   78.58322    3.09135   5.6488
   2  13.879 BB    0.4246 1312.56775   45.20705  94.3512
```

***t*-Butyl (*R*)-4-(2-(phenylamino)-1-(quinolin-2-yl)ethyl)piperazine-1-carboxylate (58)**

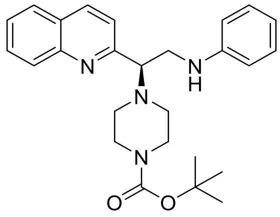

Chemical Formula: $C_{26}H_{32}N_4O_2$
Exact Mass: 432.2525
Molecular Weight: 432.5680

Prepared according to General Procedure H (*R*)-2-(1-phenylaziridin-2-yl)quinoline **36** (24.6 mg, 0.10 mmol, 1.0 equiv.), *N*-boc-piperazine (18.6 mg, 0.10 mmol, 1.0 equiv.), TFA (1.5 µL, 0.02 mmol, 20 mol%), and MeCN (200 µL, 0.5 M). The reaction mixture was subjected to the purification outlined in General Procedure H (silica, EtOAc:petroleum ether, 20:80) to afford the desired product as a pale yellow solid (36.4 mg, 84%). The enantiomeric ratio (96:4) was determined by chiral HPLC in comparison with the racemate.

ν_{max} (film): 1688, 1601, 1503, 1423, 1246, 1169 cm^{-1}.

^{1}H NMR (500 MHz, CDCl$_3$): δ 8.16 (d, *J* = 8.4 Hz, 1H), 8.12 (d, *J* = 8.5 Hz, 1H), 7.84 (d, *J* = 8.1 Hz, 1H), 7.75 (ddd, *J* = 8.3, 7.0, 1.3 Hz, 1H), 7.61 – 7.54 (m, 1H), 7.51 (d, *J* = 8.4 Hz, 1H), 7.22 – 7.15 (m, 2H), 6.74 – 6.69 (m, 1H), 6.67 (d, *J* = 7.7 Hz, 2H), 4.13 (app. S, 1H), 3.87 – 3.76 (m, 1H), 3.68 (dd, *J* = 12.3, 6.4 Hz, 1H), 3.49 (s, 4H), 2.64 (s, 4H,), 1.44 (s, 9H).

^{13}C NMR (126 MHz, CDCl$_3$): δ 159.1, 154.6, 148.2, 147.6, 136.3, 129.6, 129.5, 129.3, 127.6, 127.5, 126.6, 121.4, 117.5, 113.1, 79.7, 69.0, 49.8, 43.5, 28.4.

HRMS (ESI): exact mass calculated for [M+H]$^+$ ($C_{26}H_{33}N_4O_2$) requires *m/z* 433.2598, found *m/z* 433.2594.

HPLC analysis using a chiral column: Chiralcel® OJ-H 5 µm column, 1.0 mL/min, 90:10 hexane:*iso*propanol, 250 nm, t_{major} = 5.3 min, t_{minor} = 6.3 min.

$[\alpha]_D^{23}$ = +12.8° (*c* = 0.5, CHCl$_3$).

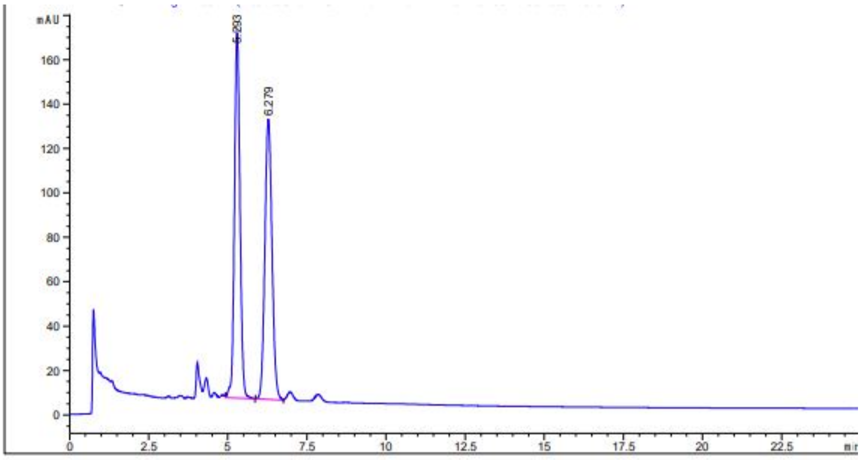

Area Percent Report

Sorted By : Signal
Multiplier : 1.0000
Dilution : 1.0000
Use Multiplier & Dilution Factor with ISTDs

Signal 1: VWD1 A, Wavelength=250 nm

Peak #	RetTime [min]	Type	Width [min]	Area [mAU*s]	Height [mAU]	Area %
1	5.293	VB	0.1891	2008.73975	164.40540	50.4430
2	6.279	BV	0.2435	1973.45483	126.23170	49.5570

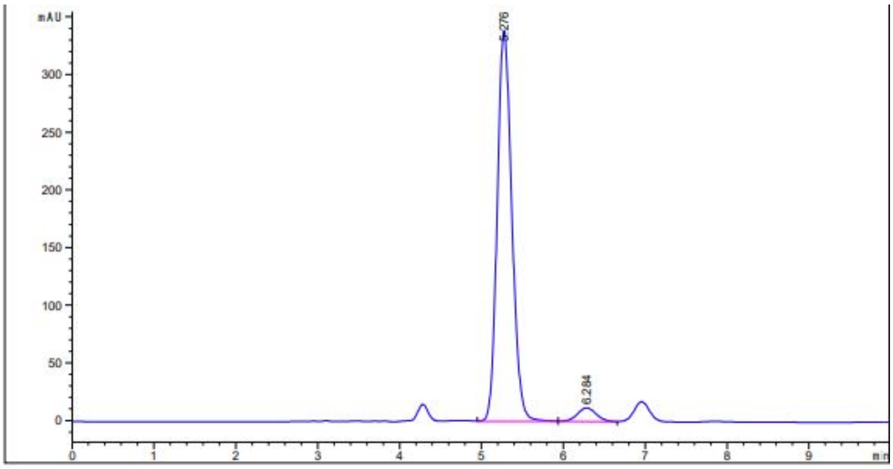

Area Percent Report

Sorted By : Signal
Multiplier : 1.0000
Dilution : 1.0000
Use Multiplier & Dilution Factor with ISTDs

Signal 1: VWD1 A, Wavelength=250 nm

Peak #	RetTime [min]	Type	Width [min]	Area [mAU*s]	Height [mAU]	Area %
1	5.276	VV	0.1927	4198.18311	338.54330	95.5284
2	6.284	VV	0.2546	196.51535	11.94362	4.4716

(*R*)-*N*-(2-Morpholino-2-(quinolin-2-yl)ethyl)aniline (59)

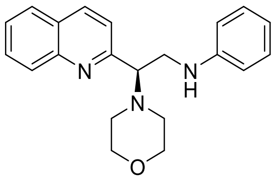

Chemical Formula: $C_{21}H_{23}N_3O$
Exact Mass: 333.18
Molecular Weight: 333.44

Prepared according to General Procedure H (*R*)-2-(1-phenylaziridin-2-yl)quinoline **36** (24.6 mg, 0.10 mmol, 1.0 equiv.), morpholine (8.8 µL, 0.10 mmol, 1.0 equiv.), TFA (1.5 µL, 0.02 mmol, 20 mol%), and MeCN (200 µL, 0.5 M). The reaction mixture was subjected to the purification outlined in General Procedure H (silica, EtOAc:petroleum ether, 20:80) to afford the desired product as a pale yellow solid (13.0 mg, 39%). The enantiomeric ratio (96:4) was determined by chiral HPLC in comparison with the racemate.

ν_{max} (film): 1601, 1504, 1115 cm^{-1}.

^{1}H NMR (400 MHz, $CDCl_3$): δ 8.23 – 8.03 (m, 2H), 7.85 (d, *J* = 8.1 Hz, 1H), 7.76 (t, *J* = 7.6 Hz, 1H), 7.61 – 7.51 (m, 2H), 7.18 (t, *J* = 7.7 Hz, 2H), 6.82 – 6.60 (m, 3H), 4.09 (app. s, 1H), 3.79 (app. s, 5H), 3.67 (dd, *J* = 12.3, 6.3 Hz, 1H), 2.70 (app. s, 4H).

^{13}C NMR (126 MHz, $CDCl_3$): δ 159.6, 148.1, 147.7, 136.5, 129.7, 129.4, 129.3, 127.6, 127.5, 126.7, 121.3, 117.5, 113.0, 69.5, 67.1, 50.6, 43.7.

HRMS (ESI): exact mass calculated for $[M+H]^+$ ($C_{21}H_{24}N_3O$) requires *m/z* 334.1914, found *m/z* 334.1907.

HPLC analysis using a chiral column: Chiralcel® OJ-H 5 µm column, 1.0 mL/min, 99:1 hexane:*iso*propanol, 250 nm, t_{major} = 36.9 min, t_{minor} = 44.8 min.

$[\alpha]_D^{23}$ = +15.8° (*c* = 0.5, $CHCl_3$).

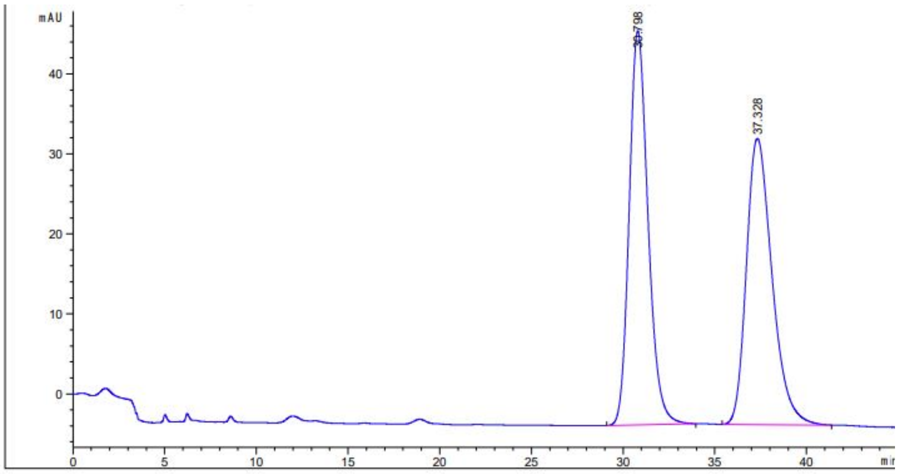


```
=====================================================================
                         Area Percent Report
=====================================================================

Sorted By              :      Signal
Multiplier             :      1.0000
Dilution               :      1.0000
Use Multiplier & Dilution Factor with ISTDs


Signal 1: VWD1 A, Wavelength=250 nm

Peak RetTime Type  Width     Area      Height     Area
  #   [min]        [min]   [mAU*s]     [mAU]        %
----|-------|----|-------|----------|----------|--------|
   1  30.798 BB    1.0980 3517.22778   49.26257  50.2979
   2  37.328 BB    1.4648 3475.57129   35.79520  49.7021
```

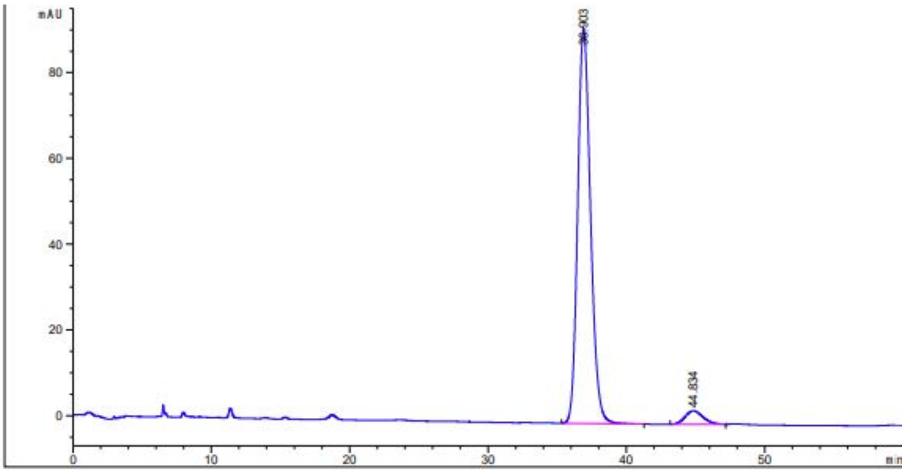

Area Percent Report

Sorted By : Signal
Multiplier : 1.0000
Dilution : 1.0000
Use Multiplier & Dilution Factor with ISTDs

Signal 1: VWD1 A, Wavelength=250 nm

Peak #	RetTime [min]	Type	Width [min]	Area [mAU*s]	Height [mAU]	Area %
1	36.903	BB	0.9912	5935.18115	92.20330	95.4792
2	44.834	BB	1.0755	281.02170	3.14207	4.5208

(*R*)-*N*-(2-(Quinolin-2-yl)-2-thiomorpholinoethyl)aniline (60)

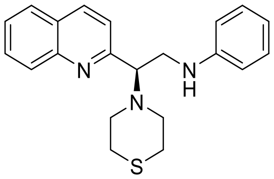

Chemical Formula: $C_{21}H_{23}N_3S$
Exact Mass: 349.1613
Molecular Weight: 349.4960

Prepared according to General Procedure H (*R*)-2-(1-phenylaziridin-2-yl)quinoline **36** (24.6 mg, 0.10 mmol, 1.0 equiv.), thiomorpholine (12 µL, 0.10 mmol, 1.0 equiv.), TFA (1.5 µL, 0.02 mmol, 20 mol%), and MeCN (200 µL, 0.5 M). The reaction mixture was subjected to the purification outlined in General Procedure H (silica, EtOAc:petroleum ether, 20:80) to afford the desired product as a pale yellow solid (13.9 mg, 40%). The enantiomeric ratio (89:11) was determined by chiral HPLC in comparison with the racemate.

ν_{max} (film): 1597, 1489 cm^{-1}.

^{1}H NMR (400 MHz, CDCl$_3$): δ 8.15 (d, *J* = 8.5 Hz, 1H), 8.12 (d, *J* = 8.1 Hz, 1H), 7.87 – 7.79 (m, 1H), 7.75 (ddd, *J* = 8.4, 6.9, 1.4 Hz, 1H), 7.57 (ddd, *J* = 8.1, 6.9, 1.2 Hz, 1H), 7.49 (d, *J* = 8.4 Hz, 1H), 7.20 (tt, *J* = 7.3, 2.1 Hz, 2H), 6.81 – 6.62 (m, 3H), 4.19 – 4.07 (m, 1H), 3.83 (dd, *J* = 12.4, 7.0 Hz, 1H), 3.71 (dd, *J* = 12.4, 6.7 Hz, 1H), 3.10 – 2.96 (m, 2H), 2.92 (s, 2H), 2.76 – 2.61 (m, 4H).

^{13}C NMR (101 MHz, CDCl$_3$): δ 159.2, 148.4, 147.6, 136.1, 129.5, 129.3, 127.6, 127.4, 126.5, 121.5, 117.4, 113.2, 69.6, 52.1, 42.6, 28.6.

HRMS (ESI): exact mass calculated for $[M+H]^+$ ($C_{21}H_{24}N_3S$) requires *m/z* 350.1685, found *m/z* 350.1681.

HPLC analysis using a chiral column: Chiralcel® OJ-H 5 µm column, 1.0 mL/min, 99:1 hexane:*iso*propanol, 250 nm, t_{major} = 26.9 min, t_{minor} = 31.2 min.

$[\alpha]_D^{23}$ = +6.6° (*c* = 0.5, CHCl$_3$).

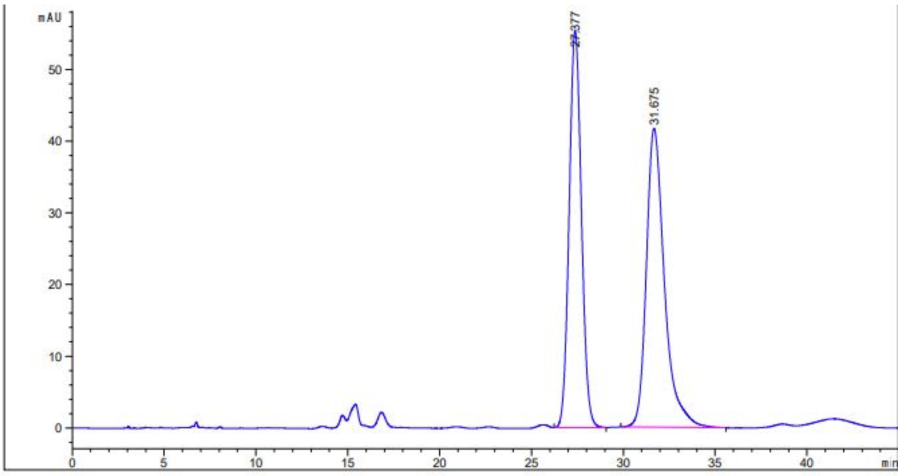

==

Area Percent Report

==

Sorted By : Signal
Multiplier : 1.0000
Dilution : 1.0000
Use Multiplier & Dilution Factor with ISTDs

Signal 1: VWD1 A, Wavelength=250 nm

Peak #	RetTime [min]	Type	Width [min]	Area [mAU*s]	Height [mAU]	Area %
1	27.377	BB	0.7183	2567.58594	55.40076	47.3292
2	31.675	BB	1.0384	2857.36475	41.69018	52.6708

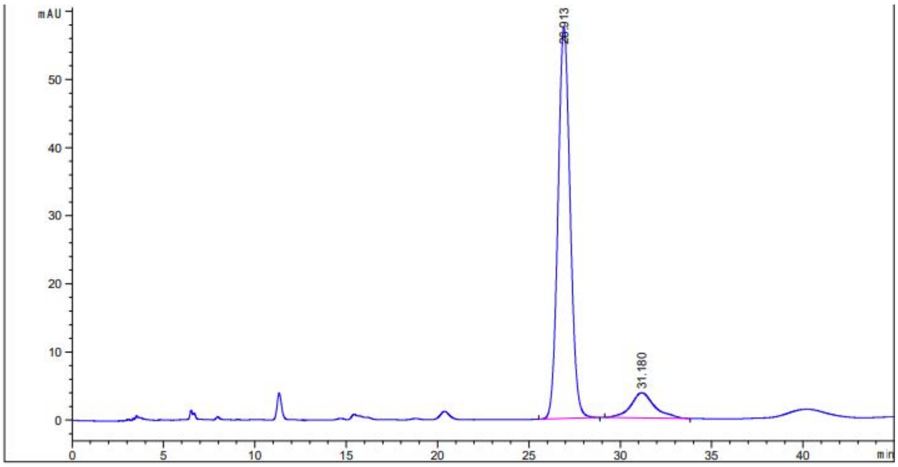

==

Area Percent Report

==

Sorted By : Signal
Multiplier : 1.0000
Dilution : 1.0000
Use Multiplier & Dilution Factor with ISTDs

Signal 1: VWD1 A, Wavelength=250 nm

Peak #	RetTime [min]	Type	Width [min]	Area [mAU*s]	Height [mAU]	Area %
1	26.913	BB	0.7200	2664.57495	57.47184	88.9031
2	31.180	BB	1.0601	332.59241	3.69580	11.0969

5. X-Ray Data

The dataset for the compound was collected using a Rigaku FR-X Ultrahigh Brilliance Microfocus RA generator/confocal optics with XtaLAB P200 diffractometer. The data was collected at 173 K with Mo radiation (λ = 0.71075 Å). Intensity data were collected using ω steps accumulating area detector images spanning at least a hemisphere of reciprocal space. All data was corrected for Lorentz and polarization effects. A multiscan absorption correction was applied by using REQAB. The structure was solved using direct methods (SHELXT). The structures were refined by full-matrix least-squares against F^2 (SHELXL). Non-hydrogen atoms were refined anisotropically, and some hydrogen atoms were refined isotropically and the rest were refined using a riding model. All calculations were performed using the CrystalStructure interface except for refinement, which was performed using SHELXL. Selected crystallographic data are presented in the table below.

Crystal Data		
Compound Number	**20**	**44**
Chemical formula	$C_{17}H_{14}BrClN_2$	$C_{17}H_{13}BrN_2$
M_r	361.67	325.21
Crystal system, space group	Orthorombic, $P2_12_12_1$ (#19)	Monoclinic, $P2_1$ (#4)
Temperature (K)	173	173
a, *b*, *c* (Å)	5.3565(13), 14.760(3), 19.254(5)	9.9059(12), 30.379(4), 14.388(2)
α, β, γ (°)	90.0000, 90.0000, 90.0000	90.0000, 92.268(4), 90.0000
V (Å^3)	1522.3(6)	4326.4(10)
Z	4	12
Radiation type	MoK	MoK
μ (mm^{-1})	2.877	2.849
Crystal size (mm)	0.120 x 0.030 x 0.030	0.050 x 0.050 x 0.050
Data Collection		
T_{min}, T_{max}	0.546, 0.917	0.681, 0.867
No. of measured, independent and observed reflections	9412, 2782, 2265,	57050, 15760, 10674
R_{int}	0.0481	0.0478
(sin θ/λ)$_{max}$ (Å^{-1})		
Refinement		
$R[F^2 > 2\sigma(F^2)]$, $wR(F^2)$, S	0.0353, 0.0542, 0.864	0.0909, 0.1048, 1.04
No. of reflections	2782	15760
No. of parameters	194	1081
No. of restraints	1	1
$\Delta\rangle_{max}$, $\Delta\rangle_{min}$ (e Å^{-3})	0.39, -0.42	0.76, -0.49
Absolute structure parameter	-0.001(7)	0.036(4)

Computer programs: *CrystalStructure* 4.3 (Rigaku, 2019), REQAB (Rigaku, 1998), *SHELXL* Version 2018/3 (Sheldrick, 2008), *SHELXT* Version 2018/2 (Sheldrick, 2014).

6. Computational Chemistry

Calculations were performed using the ONIOM approach in which the quantum mechanical region included all atoms of the quinoline and aniline substrates and the PO_4 unit of the catalyst.[23] This region was described using the B3LYP/6-31G** level of theory.[24] The remainder of the catalyst was described using the UFF forcefield.[25] This mixed QM/MM approach has been demonstrated to provide useful rationalisation of experimentally observed selectivities of reactions catalysed by other BINAP-derived species.[26] All calculations were performed in Gaussian 09 and free energies were computed using the goodvibes code with temperature set to 253 K and concentration 1 M.[27] Consistent bonding patterns were maintained in the job setup with alternating single and double bonds being used throughout the catalyst structure.

As in our previous study of reactions catalysed by the TRIP catalyst, the reaction profile was found to proceed through 1) complexation of the quinoline substrate to the catalyst (held together by a hydrogen bond), 2) addition of aniline to the quinoline complex with either concerted or subsequent proton transfer to the catalyst, 3) proton transfer from the catalyst to the resulting prochiral enamine and 4) product dissociation. The isolated catalyst is assumed to be in the POH form, and this strong hydrogen bond donor likely binds to a solvent molecule, here modelled as THF for computational simplicity. Mapping the reaction profile (Figure S1) revealed that the rate-limiting barrier for reaction initiation corresponds to the difference in energy between the quinoline complex (the resting state) and the transition state for protonation of the enamine whereas the barrier for the catalytic cycle corresponds to the energy difference between the product complex and the same transition state. Exploration of the details of the reaction involved consideration of significant conformational variation in both the substrate/reactant and catalyst components. In general, full substrate conformation exploration (scanning each relevant dihedral in 120° increments) was performed for the system involving the TRIP catalyst and for the catalyst lacking the flanking aromatic rings (**4**). The reactions catalysed by other catalysts (all featuring the aromatic rings present in TRIP) were obtained by altering the catalyst structure in each of the low energy conformations identified for TRIP. In the case of 4-alkyl or 3,5-dialkyl substituents on the phenyl rings in the catalyst, alternative conformations for the alkyl groups in the catalyst were considered. For catalysts containing cycloalkyl groups, the cyclohexyl version with well-defined chair conformations was studied first and then the rings expanded to give cycloheptyl in low energy conformations. Ring contraction to give cyclopentyl equivalents was also performed but, in this case, complemented by cyclopentyl geometries created directly in Gaussview.[28]

The detailed procedure for identifying the key transition states involved systematic exploration of the product-catalyst complexes for both enantiomers. All the resulting conformations that were within 5 kcal/mol of the lowest energy conformation were subjected to a scan using the redundant coordinate facility in Gaussian 09 in which H attached to the chiral centre was translated towards the oxygen of a P=O function. Exceptions to this were for those complexes in which the relevant CH was remote from the P=O group. Scans that led to plausible proton transfer pathways were then optimized to yield an array of transition state conformations and those that were identified as the key lowest energy transition states then saw an exploration of the catalyst conformation although it is noted that *i*-Pr groups (or cycloalkyl groups) at the 2 and 6 positions of the phenyl in catalysts like TRIP do not have low energy alternative orientations. All geometries were cross-checked to remove duplicate conformations. Complexes for the quinoline reactant with catalyst were set up by analogy to the approach described by the Goodman group.[26] Given the nature of the reaction energy profile, other species are not critical to governing reactivity and so although they have been identified, less extensive conformational sampling has been applied.

Reaction profile

The reaction profile for the TRIP-catalyzed reaction is in Figure S1. Conformations of the immediate precursor to the rate-limiting transition state were obtained by manually deleting the proton from the

chiral centre in each of the low energy (within 5 kcal/mol) product conformations and adding it to the phosphate of the catalyst. Subsequent optimization in some cases saw proton transfer back to the aniline nitrogen. Although on the electronic energy surface of this process there is a transition state for aniline addition to the quinoline-catalyst complex followed by an anilinium intermediate that then proton transfers to the catalyst, we find that this is effectively concerted aniline addition-proton transfer when free energies are considered. Geometries for individual species are provided in the geometries and energies section below for those interested but summarised as a single state in the energy profile.

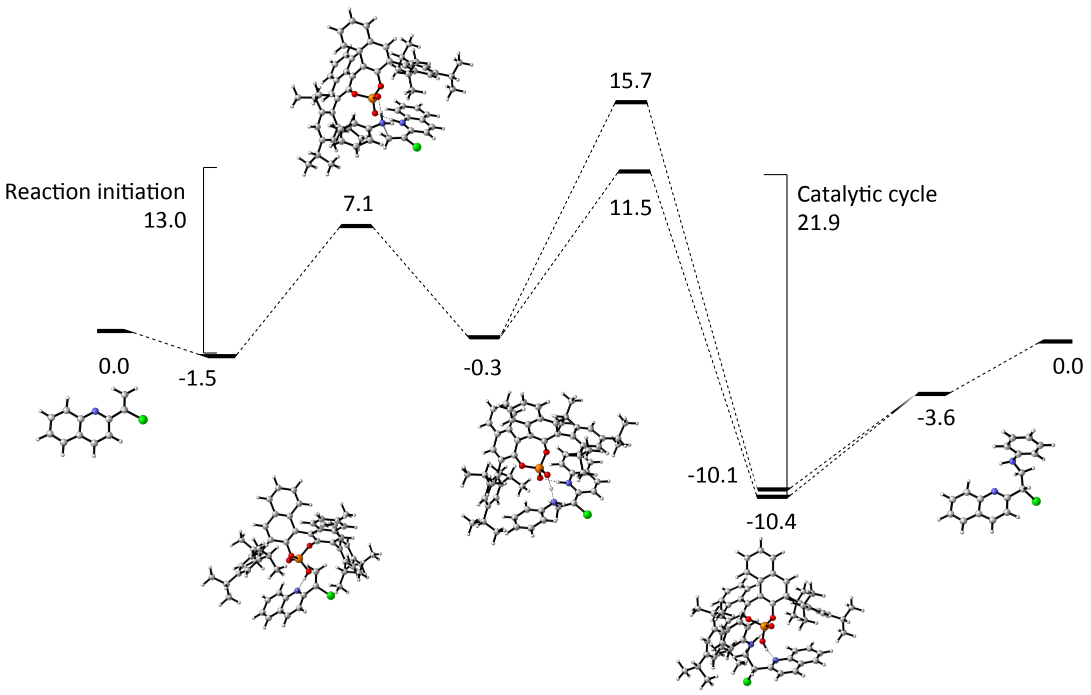

Figure S1. Energy profile for the TRIP-catalyzed reaction. Free energies in kcal/mol are indicated from ONIOM(B3LYP/6-31G**:UFF) calculations.

In its unbound form, the quinoline prefers the s-*trans* conformation (Figure S2). This is preferred by 2.2 kcal/mol to the s-*cis* form. Once bound to the catalyst, this preference is enhanced to 2.9 kcal/mol. This presumably reflects repulsion between the chlorine and the phosphate oxygens in the bound state. As found in previous work, the complex is held together by a dynamic hydrogen bonding that is retained even during proton transfer between catalyst and substrate and back again.

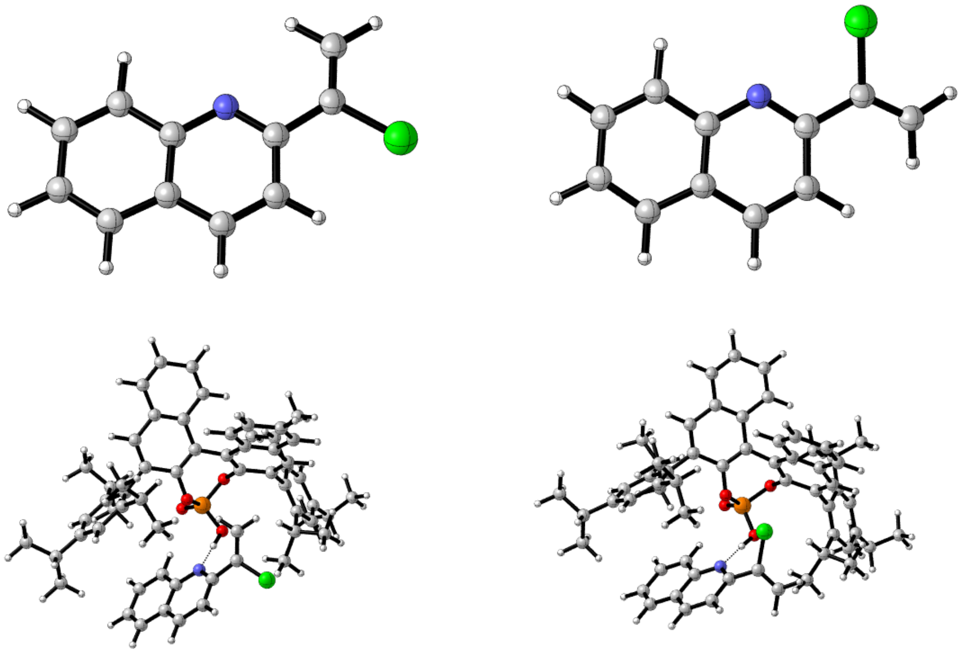

Figure S2. The lowest energy s-*trans* conformation of quinoline substrate on the left and higher energy s-*cis* conformation on the right. At top is shown the free form and at bottom the catalyst bound form with the hydrogen bond between quinoline nitrogen and POH shown in dashes.

Do barriers for initiation and catalytic cycle correspond to reaction efficiency?

Before investigating whether the calculations are able to provide insight into reaction efficiency, it is first necessary to establish that they provide energetics that are in line with experimental observation.

Given the reaction profile that is computed, it would be expected that reaction initiation would depend on the energy difference between the quinoline-catalyst complex and the transition state for protonation of the catalyst-bound enamine. Turnover in the catalytic cycle, should be the process on which the main flux of product depends, and this would depend on the displacement of product from the catalyst i.e., the resting state would be the catalyst-product complex. Given the substantial difference in binding free energy for substrate (1.5 kcal/mol) and product (6.8 kcal/mol), this ground state would be expected to become established very early in reaction evolution (even when quinoline is present at much higher concentrations than product). This allows us to rationalise the variation in reaction effectiveness, at least qualitatively by considering effects on ground state destabilisation and transition state stabilisation, both of which would be expected to decrease the barrier for the catalytic cycle. Whereas in the transition state in which the stereocenter is set, the NHPh group is free to rotate (Figure S3), the product of the reaction benefits from being held to the catalyst by two hydrogen bonds. These impose significant additional conformational restriction on the structure and therefore it is possible that the catalyst structure could have contrasting impacts on reaction efficiency and stereoselectivity.

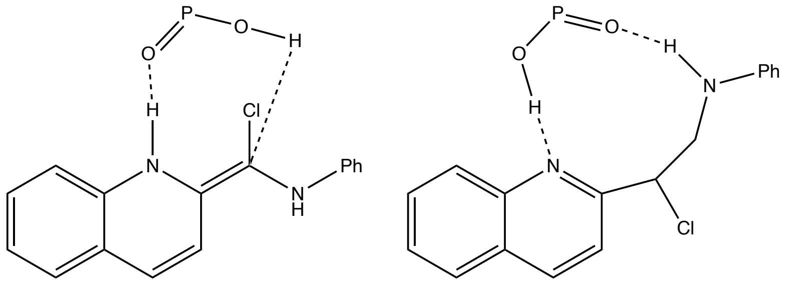

Figure S3. Left: Schematic of the two interactions holding the rate-limiting transition state together contrasted with Right: The two hydrogen bonds holding together the product-catalyst complex. In the transition state complex, the NHPh remains able to rotate whereas in the product complex the degree of flexibility is reduced.

Table S8. Activation free energies for entry into the catalytic cycle (initiation) and for subsequent catalytic cycles.

**Catalyst**	**Free energy barrier for initiation (kcal/mol)**	**Free energy barrier for catalytic cycle (kcal/mol)**
TRIP	13.1	21.9
**H**	15.6	22.5
**Ph**	11.8	23.2
4-(*i*-Pr)	14.1	23.1
**3,5-(*i*-Pr)$_2$**	15.5	23.8
2,4,6-C5	11.4	21.0
2,4,6-C6	17.3	23.1
2,4,6-C7	15.4	26.0

The barriers to the catalytic cycle (apart from that for the cycloheptyl substituted catalyst) reveal that, in line with experimental observation, the catalysts lacking a 4-substituent (in bold) have higher barriers for the catalytic cycle than the others, although in line with the small reduction in yield, this difference is minimal and below the accuracy of the calculations but supports the computed structures as providing a basis to explore the catalytic efficiency. This is explored in the section below.

Do differences in barrier correspond to selectivity?

As with reaction efficiency, it is necessary to consider whether differences in barrier height correspond to stereoselectivity before exploring the origin of this at a structural level.

The lowest energy transition state found computationally for the stereochemistry-determining step in the TRIP-catalysed reaction is one that would yield the observed major isomer (Figure S1). The lowest energy transition state that would give the alternative stereoisomer is 4.1 kcal/mol higher in free energy. This is not in quantitative agreement with observation – the computed stereochemical preference based on this difference in lowest energy transition states would suggest much greater than 99:1 preference. The conformational variation in the transition states (Figure S4) reveals one possible reason for this difference (albeit one that still does not quite provide quantitative agreement with experiment). The transition state leading to the major stereoisomer has two dominant low energy forms (that differ only by rotation of one of the *i*-Pr groups in the catalyst); there is only one other structure within 1 kcal/mol of the lowest energy conformation. By contrast, for the transition states leading to the minor stereoisomer, there are 8 alternative conformations within 1 kcal/mol of

the lowest. This suggests that there will be a sharp temperature dependence of the stereoselectivity and that improvements in stereoselectivity might be achieved with more rigid catalysts.

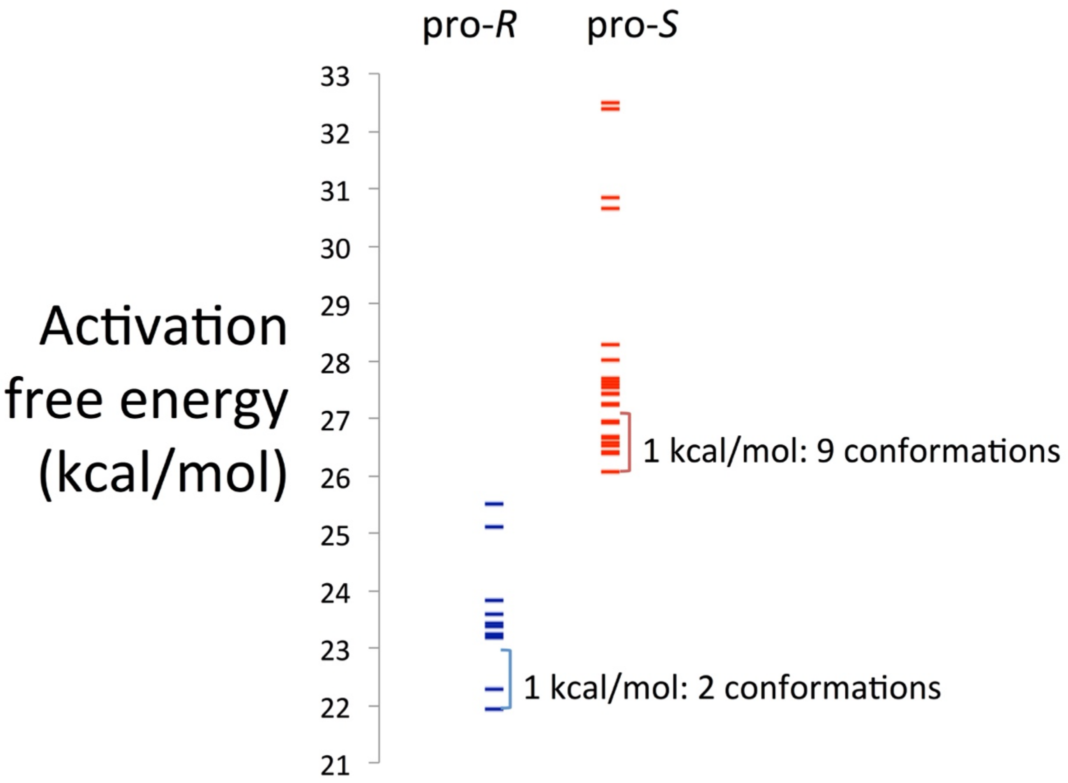

Figure S4. Free energies (for catalytic turnover) for a range of computed transition states leading to *R* enantiomer (blue) and *S* enantiomer (red).

The exploration of alternative catalysts computationally led to the computed barriers and stereoselectivity summarised in Table S9. As shown in Figure S5, the preferences track approximately with the measured enantiomeric preferences and support the computational approach as recapitulating the key features of the reaction in terms of stereoselectivity. One exception to the trend is the 3,5-(*i*-Pr)$_2$ catalyst that is computed to have a much lower stereoselectivity than is observed. The low level of stereoselectivity measured experimentally would correspond to a small energy difference that may be at the edge of what can be computed reliably. One other feature is clear from Tables S8 and S9. There is likely a lower energy transition state for the cycloheptyl catalyst leading to the *R* stereoisomer involving an alternative ring conformation of one or more of the flexible cycloheptyl groups; the conformational possibilities for this system are too large for systematic exploration. A lower energy transition state would bring the overall barrier (Table S8) and the stereoselectivity (Table S9) into line with those computed for the other examples. Given the likely missing structure, the cycloheptyl example is not discussed further but is expected to follow the trends on going from cyclopentyl to cyclohexyl.

Table S9. Activation free energy barriers for accessing the two stereoisomers *via* the rate-determining transition state.

Catalyst	Barrier to access *R* stereoisomer (kcal/mol)	Barrier to access *S* stereoisomer (kcal/mol)	Preference for *R* (kcal/mol)
TRIP	21.9	26.1	+4.1
H	22.5	22.5	0.0

Ph	23.2	25.8	+2.6
4-iPr	23.1	24.9	+1.8
3,5-(*i*-Pr)$_2$	24.4	23.8	-0.6
2,4,6-C5	21.0	24.4	+3.5
2,4,6-C6	23.1	27.2	+4.2
2,4,6-C7	26.0	29.5	+3.5

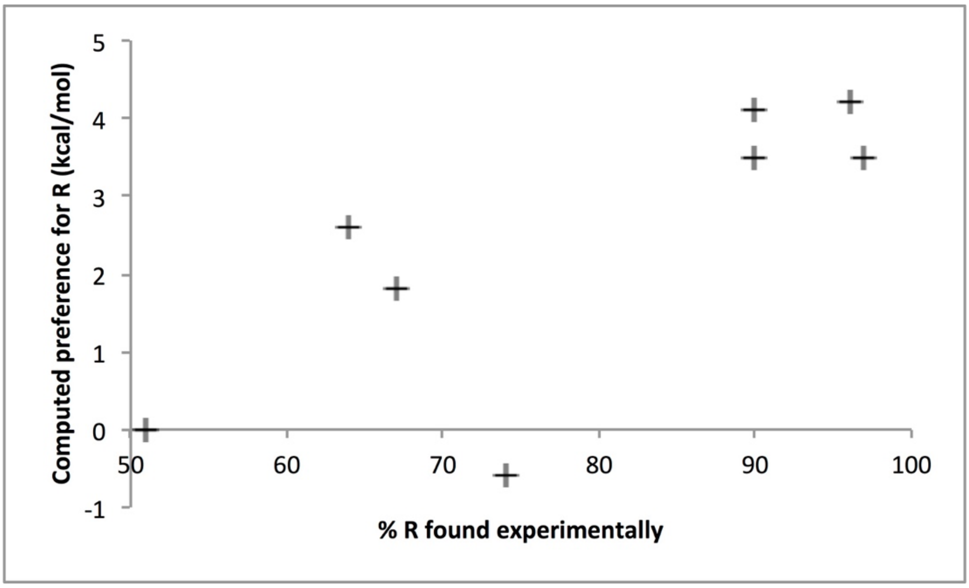

Figure S5. Computed preference for the *R* stereoisomer is plotted against the experimentally observed preference.

Rationale for changes in ground state (de)stabilisation.

Having established that the calculated energies correspond to reaction efficiency, we now examine the computed structures to explore the origin of these effects. Turning first to the effect of changing catalyst structure on reaction efficiency, the values in Table S9 reveal that across the set of catalysts studied, the catalytic cycle barrier corresponds well to catalyst efficiency; if the barrier is below 23.2 kcal/mol, the conversion is high. In principle, reaction efficiency can be increased by stabilising transition states or destabilising ground states (in this case the product-catalyst complex). To explore this, the binding free energy of the product and the free energy of transition states relative to substrates and catalyst have been computed (Table S10). These can be combined and related to the catalytic cycle barriers in Table S9 by the overall free energy change of the reaction of –3.6 kcal/mol. Table S10 shows that the H-substituted catalyst manages to achieve a low overall barrier despite poor transition state stabilisation by also benefiting from ground state destabilisation. The extra ground state stabilisation (4.5 kcal/mol) that occurs upon addition of the phenyl groups to the catalyst is not matched by a corresponding transition state stabilisation (only 3.8 kcal/mol) and thus the phenyl substituted catalyst is not as efficient. When this is supplemented with a 4-(*i*-Pr) group, there is an increased ground state destabilisation of 0.6 kcal/mol whereas the transition state free energy increases by only 0.5 kcal/mol and this small gain gives an increased efficiency. Adding two *i*-Pr groups at 3 and 5 sees a 1 kcal/mol increase in ground state destabilisation but a 1.6 kcal/mol increase in transition state free energy and thus this too is a less effective catalyst. The 4-cycloalkyl substituted examples evidently provide the most pronounced transition state stabilisation and this offsets any increasing ground state stabilisation.

Table S10. The rate-limiting barrier is decomposed into ground state effects (product complexation energies) and transition state effects (barriers relative to reactants).

Catalyst	Product complexation free energy (kcal/mol)	Lowest three catalyst-bound transition state free energies relative to reactants (kcal/mol)	Conversion observed after 15 hours
TRIP	-6.8	11.5 (*R*), 11.9 (*R*), 12.8 (*R*)	98
H	-3.3	15.6 (*S*), 15.6 (*R*), 16.6 (*R*)	90
Ph	-7.8	11.8 (*R*), 12.2 (*R*), 14.4 (*S*)	71
4-(*i*-Pr)	-7.2	12.3 (*R*), 14.0 (*R*), 14.1 (*S*)	97
3,5-(*i*-Pr)$_2$	-6.8	13.4 (*S*), 13.5 (*S*), 13.6 (*R*),	73
2,4,6-C5	-5.9	11.4 (*R*), 11.8 (*R*), 11.8 (*R*)	95
2,4,6-C6	-8.4	11.0 (*R*), 11.1 (*R*), 12.1 (*R*)	96

To explore the structural origins of the ground state effects, the complexes of the product with the different catalysts have been visualised using spheres of approximately van der Waals radii in pymol (Figure S6). This reveals that there are few contacts for the unsubstituted catalyst and that once the phenyl ring is added, the quinoline and aniline general stack around it. The shape complementarity of this arrangement is better with the 4-(*i*-Pr) than with either Ph or 3,5-(*i*-Pr)$_2$. It is interesting that the cyclopentyl system makes good contact with the chlorine, but this is at the cost of leaving less contact of the two aromatic rings. When the cyclopentyl ring is expanded to give the cyclohexyl, the shape complementarity improves, and the two aromatic groups (phenyl and quinoline) make contact with one of the cyclohexyl rings while the chlorine retains its good contacts. A similar set of contacts are possible for the TRIP catalyst.

TRIP

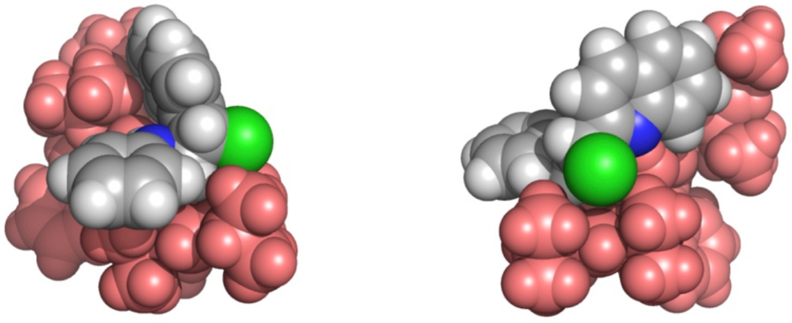

H

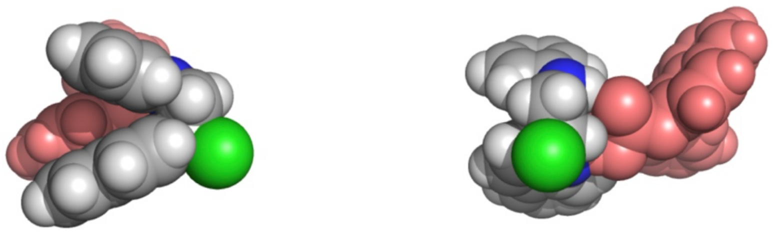

Ph

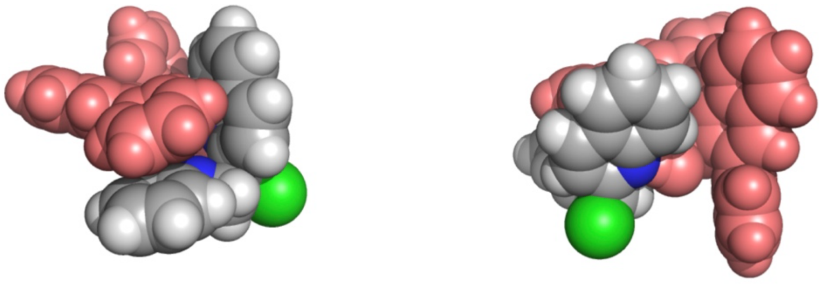

4-iPrPh

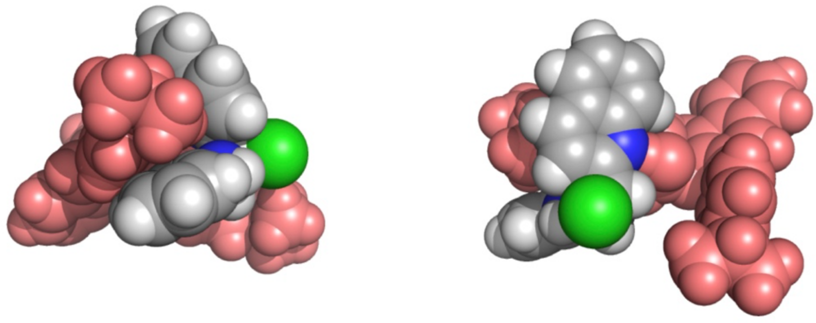

3,5-di-iPrPh

2,4,6-C5

2,4,6-C6

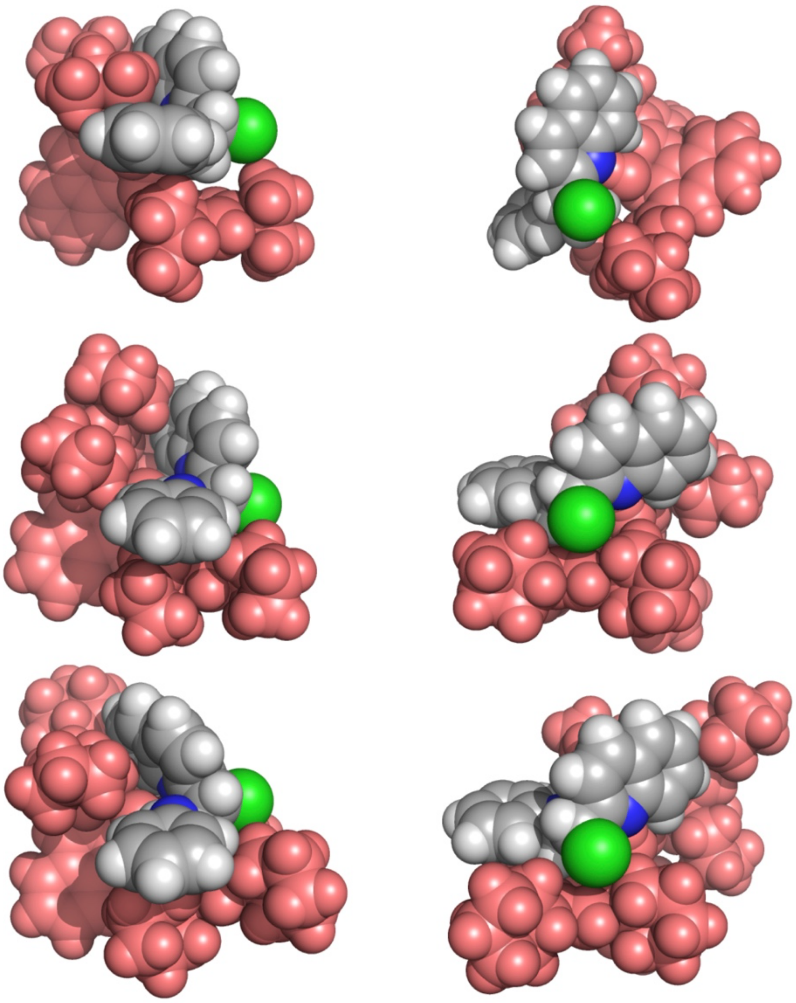

Figure S6. A visualisation of catalyst-product complexes (the ground state in the catalytic cycle). On the left the complexes are visualised with the chlorine protruding rightwards to visualise the contact between the quinoline group and the catalyst. On the right the chlorine is towards the viewer to visualise its interactions and those of the aniline group.

Rationale for changes in Transition State free energies.

In order to understand the transition state effects, it has been helpful to provide a systematic means of describing the transition state structure. Our inspiration was the classification system of Reid and Goodman into Type I and Type II that depend upon the orientation of an imine relative to the 3 and 3' substituents. In our case, the functionality proximal to the OH has three substituents as opposed to the two in the imine structure. Therefore, we propose classifying the transition state structures according to the position of these three groups along the O-H-C axis (Figure S7). In particular, the dihedral angle formed by P-O-C-Cl is a useful way of classifying the transition states noting that the positioning of the other two groups (Q for quinoline and An for CH_2NHPh) depends upon whether transition state is leading to the *R* or *S* product. Given that quinoline is the largest group of the three, there is an approximate equivalence between Reid and Goodman's Type I and pro-*R* anti and pro-*S* g-; similarly, their Type II is most similar to pro-*R* g+ and pro-*S* anti. One other feature of the structures is that generally either the quinoline-derived enamine NH or the aniline NH makes a hydrogen bond to the flanking P=O. The transition state is formally for protonation of the enamine but those examples in which the quinoline NH hydrogen bonds with the phosphate allows it then to deprotonate the quinoline nitrogen without rearrangement. There is a variation on this theme present in transition states for the unsubstituted catalyst in which aniline NH interacts with the phosphate but in turn, the

aniline nitrogen's lone pair interacts with the quinoline enamine NH; presumably a rapid proton transfer relay would follow protonation of the enamine by the POH. We note that a concerted protonation-deprotonation process is presumably avoided because it would involve a transition state with anti-aromatic character.

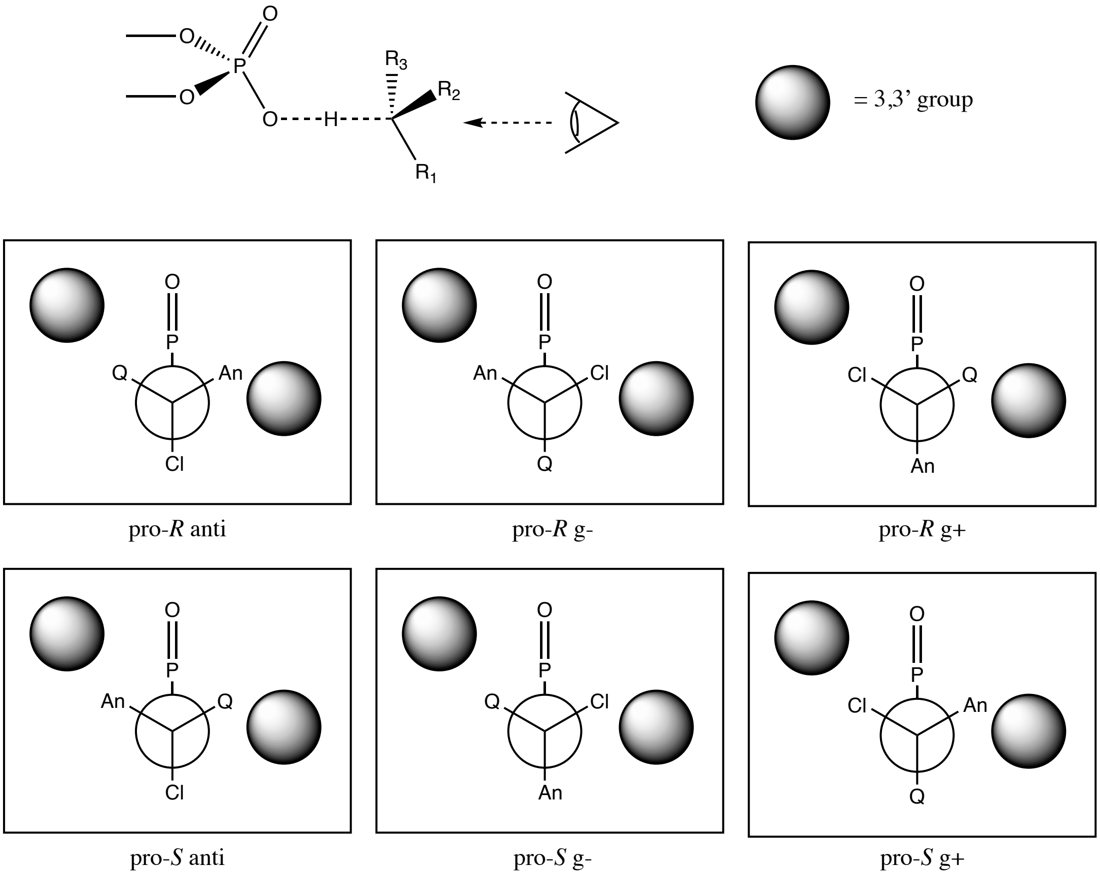

Figure S7. Classification of the transition states according to the positioning of groups around the O-H-C axis. Labels are according to the stereochemical outcome (pro-*R* or pro-*S*) and the relative orientation of Cl and the P=O group (anti, g- or g+).

It is useful to first examine the transition states for the unsubstituted catalyst in which the effects of the 3 and 3' groups are minimal and in which both experiment and theory agree that stereoselectivity would be low. The lowest free energy transition states of each type are shown in Table S11 for the unsubstituted catalyst and for TRIP. It is notable that two conformations are significantly higher in energy for the unsubstituted catalyst and that these are consequently not found for the TRIP catalyst. These are the two possibilities in which the quinoline is away from the P=O group and this reflects the benefit that arises from the stabilising electrostatic interaction in the transition state as the enamine gets protonated, and the phosphate deprotonated and facilitates rapid post-transition state proton transfer to regenerate the POH. It is also clear that in the absence of 3 and 3' groups, the reaction prefers the *anti* transition states. This likely reflects an orientation in which the dipole of the phosphate opposes that of the C-Cl bond. It is clear from Table S11 that catalysts like TRIP exert their stereoselective effect by strongly disfavouring the set of pro-*S* anti transition states as seen for the full set of transition states in Figure AGL8 where the free energy barrier corresponding to each transition state is plotted against the P-O-C-Cl dihedral. Going from TRIP to H catalyst, all transition states generally go down in energy apart from the pro-S anti. Therefore, understanding this is at the heart of understanding the stereoselectivity achieved by the catalysts.

Table S11. The lowest free energy transition state of each type for the unsubstituted and TRIP catalysts.

Stereochemistry	Conformation	Lowest free energy transition state	
		H	TRIP
Pro-*R*	anti	15.6	11.5
	g-	27.1	n.f.
	g+	17.8	15.1
Pro-*S*	anti	15.6	20.3
	g-	18.2	15.7
	g+	24.2	n.f.

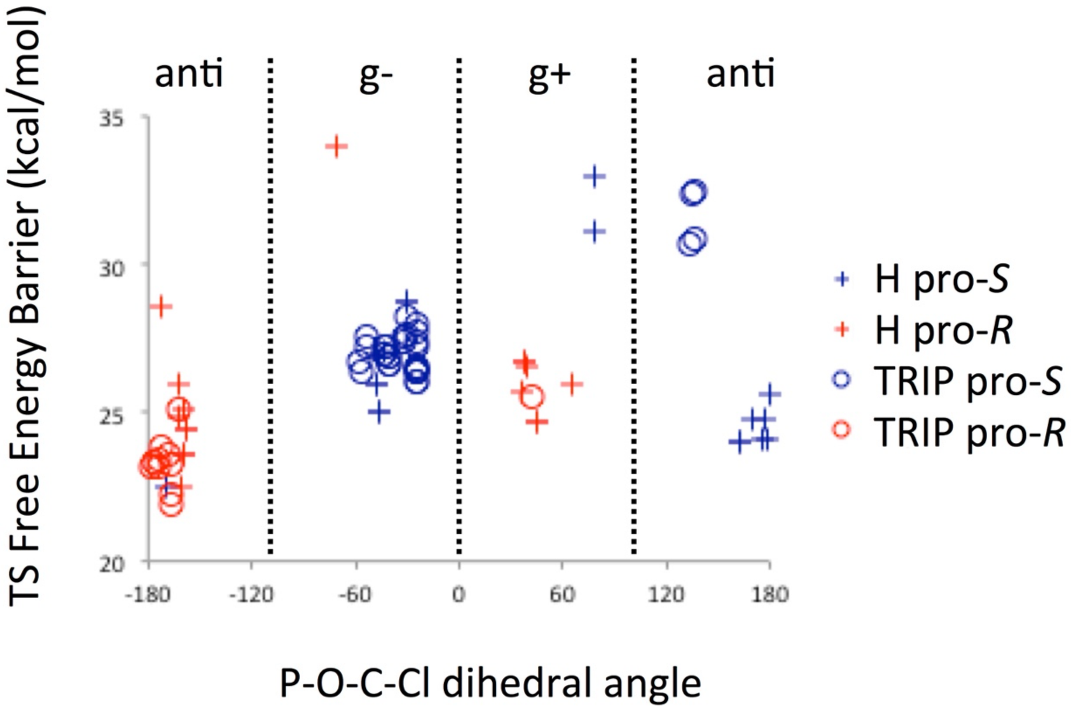

Figure S8. All free energy barriers for the transition states identified for the unsubstituted (H) and TRIP catalysts are plotted against the corresponding P-O-C-Cl dihedral angle.

The first rationale that was examined was whether the AREA(θ) measure proposed by Reid and Goodman might correspond with selectivity.[29] This descriptor attempts to capture the effects of steric bulk remote from the phosphate core of the catalyst. The values for all of the catalysts examined have not been provided previously (to the best of our knowledge) and therefore, we generated our own versions by following the published procedure but applying it to all of the low energy conformations (within 5 kcal/mol of the lowest) of the catalyst-THF complex that had been obtained in our ONIOM calculations (by contrast Reid and Goodman employed forcefield level calculations for the free catalyst). These values (Table S12 and plotted against the % of the *R* enantiomer formed in Figure S9) reveal that increasing the remote steric demand, as assessed by decreasing AREA(θ), corresponds to increasing enantioselectivity until an area of about 55-60˙ is reached and below that area a range of selectivities is observed and therefore among this group a more subtle explanation is required.

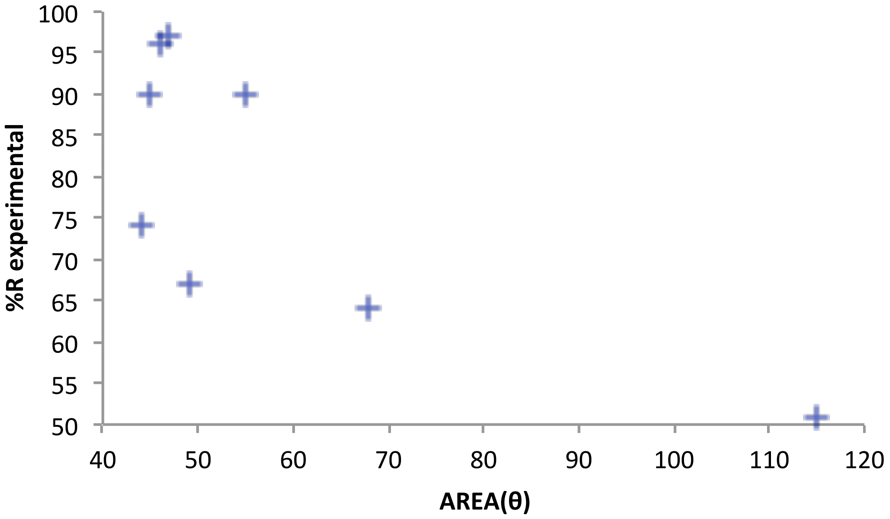

Figure S9. Experimentally observed % of the *R* enantiomer formed is plotted against the AREA(θ) descriptor for the catalyst.

Table S12. AREA(θ) values obtained for the catalysts studied computationally.

Catalyst	AREA(θ)
TRIP	55
H	115
Ph	68
4iPrPh	49
35iPr	44
C5	45
C6	46
C7	47

Considering the geometry of the transition states that have been found and consistent with the observation above that the quinoline has a distinct preference as the largest group, the lowest energy transition states leading to both *R* and *S* stereoisomers for the TRIP catalyst reveal the key impact of catalyst structure on transition state free energy both in terms of reaction efficiency and stereoselectivity. In Figure S10, the pro-*R* transition state and the pro-*S* transition state for TRIP are shown in such a way that the similar placement of the quinoline (largest group) is made clear. The second largest group is the CH_2NHPh and its placement reveals the differences. In the pro-*R* transition state, this is placed in such a way that it nestles against the 4-(*i*-Pr) group. Optimising this shape complementarity will stabilise this transition state and this would lead to an increase in both efficiency and stereoselectivity, as observed experimentally. Secondary alkyl groups appear to provide good contacts and therefore van der Waals-type stabilisation by lying against the aromatic ring of the aniline. This improves as increased contact area is made possible by expanding to cycloalkyl groups but would presumably be disrupted by expanding to tertiary alkyl groups. By contrast, in the pro-*S* transition state, the CH_2NHPh is placed not just on one of the ortho substituents but between two of them. This means that as these groups are expanded, there comes a point where the aniline would be squeezed out from this region requiring alternative, less favourable conformations to be adopted. This disfavouring is key for stereoselectivity and expanding from cyclopentyl through to cycloheptyl exploits this effect. This structure also shows why the pro-*S* transition state has access to a range of

alternative conformations; whereas the ortho substituents in the catalyst are locked in conformation due to their orientation with respect to the catalyst, the para groups are in principle free to rotate in the free catalyst. In the pro-*R* transition state complex, the contact with the CH_2NHPh restricts the conformational freedom of one of these groups. By contrast, in the pro-*S* transition state, the corresponding para group is protruding away from the substrate and retains its ability to rotate freely.

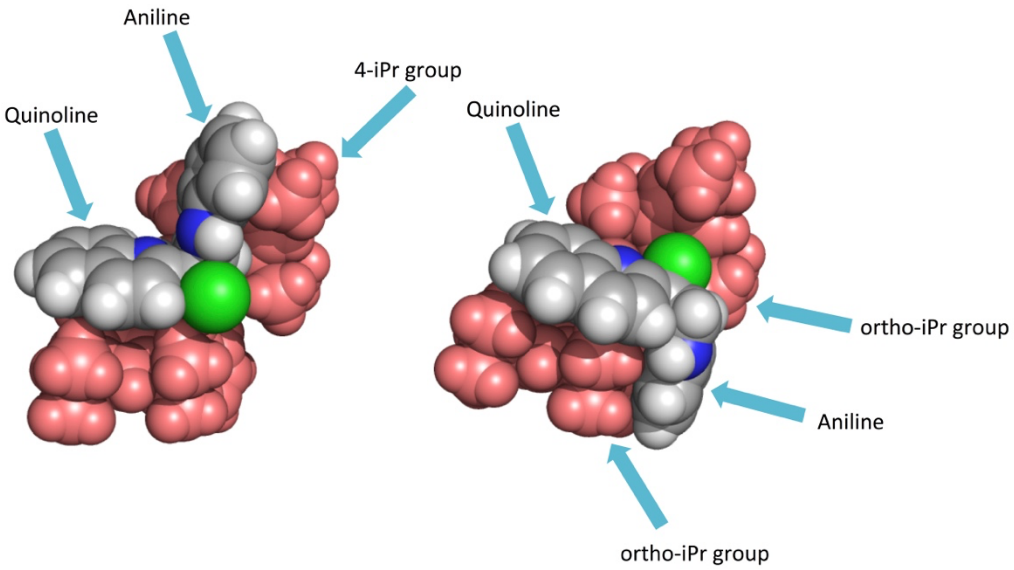

Figure S10. The lowest free energy transition states leading to *R* (left) and *S* (right) enantiomers of product are orientated so as to place the quinoline in a similar position with respect to the viewer.

7. References

[1] Armarego, W. L. F.; Chai, C. *Purification of Laboratory Chemicals, 7th ed.*, Elsevier, Oxford, **2013**.

[2] Venanzi, L. M. *J. Chem. Soc.* **1958**, *74*, 719-724.

[3] Cain, D. L.; McLaughlin, C.; Molloy, J. J.; Carpenter-Warren, C.; Anderson, N. A.; Watson, A. J. B. *Synlett* **2019**, *30*, 787–791.

[4] Komatsuki, K.; Sadamitsu, Y.; Sekine, K.; Saito, K.; Yamada, T. *Angew. Chem. Int. Ed.* **2017**, *129*, 11752–11756.

[5] Neel, A. J.; Milo, A.; Sigman, M. S.; Toste, F. D. *J. Am. Chem. Soc.* **2016**, *138*, 3863–3875.

[6] Li, J.; Grosslight, S.; Miller, S. J.; Sigman, M. S.; Toste, F. D. *ACS Catal.* **2019**, *9*, 9794–9799.

[7] Huang, Y.-Y.; Chakrabarti, A.; Morita, N.; Schneider, U.; Kobayashi, S. *Angew. Chem. Int. Ed.* **2011**, *50*, 11121–11124.

[8] Romanov-Michailidis, F.; Guénée, L.; Alexakis, A. *Angew. Chem. Int. Ed.* **2013**, *52*, 9266–9270.

[9] Yue, T.; Wang, M.-X.; Wang, D.-X.; Masson, G.; Zhu, J. *Angew. Chem. Int. Ed.* **2009**, *48*, 6717–6721.

[10] Yang, J.; Wu, S.; Chen, F.-X. *Synlett* **2010**, 2725–2728.

[11] Gheewala, C. D.; Hirschi, J. S.; Lee, W.-H.; Paley, D. W.; Vetticatt, M. J.; Lambert, T. H. *J. Am. Chem. Soc.* **2018**, *140*, 3523–3527.

[12] Navarro, R.; Monterde, C.; Iglesias, M.; Sánchez, F. *ACS Omega* **2018**, *3*, 1197–1200.

[13] Milburn, R. R.; Hussain, S. M. S.; Prien, O.; Ahmed, Z.; Snieckus, V. *Org. Lett.* **2007**, *9*, 4403–4406.

[14] Greenhalgh, M. D.; Frank, D. J.; Thomas, S. P. *Adv. Synth. Catal.* **2014**, *356*, 584–590.

[15] Xiao, J.; Huang, Ye.; Song, Z.; Feng, W. *RSC Adv.* **2015**, *5*, 99095–99098.

[16] Nakatake, D.; Yokote, Y.; Matsushima, Y.; Yazaki, R.; Ohshima, T. *Green Chem.* **2016**, *18*, 1524–1530.

[17] Parisien, M.; Valette, D.; Fagnou, K. *J. Org. Chem.* **2005**, *70*, 7578–7584.

[18] Knapp, D. M.; Gillis, E. P.; Burke, M. D. *J. Am. Chem. Soc.* **2009**, *131*, 6961–6963.

[19] Zhang, Y.; Sheets, M. R.; Raja, E. K.; Boblak, K. N.; Klumpp, D. A. *J. Am. Chem. Soc.* **2011**, *133*, 8467–8469.

[20] Kyowa Hakko Kirin Co., Ltd. EP2433938, **2012**.

[21] Chen, J.; Li, J.; Wang, J.; Li, H.; Wang, W.; Guo, Y. *Org. Lett.* **2015**, *17*, 2214–2217.

[22] Todorov, A. R.; Wirtanen, T.; Helaja, J. *J. Org. Chem*. **2017**, *82*, 13756–13767.

[23] Dapprich, S.; Komáromi, I.; Byun, K. S.; Morokuma, K.; Frisch, M. J. *J. Mol. Struct. THEOCHEM* **1999**, *461–462*, 1–21.

[24] Becke, A. D. *Phys. Rev. A* **1988**, *38*, 3098–3100; Becke, A. D. *J. Chem. Phys.* **1993**, *98*, 5648–5652; Lee, C.; Yang, W.; Parr, R. G. *Phys. Rev. B* **1988**, *37*, 785–789.

[25] Rappe, A. K.; Casewit, C. J.; Colwell K. S.; Goddard, W. A.; Skiff, W. M.; *J. Am. Chem. Soc.* **1992**, *114*, 10024–10035.

[26] Grayson, M. N.; Pellegrinet, S. C.; Goodman, J. M. *J. Am. Chem. Soc.* **2012**, 134, 2716–2722.

[27] GoodVibes Luchini, G.; Alegre-Requena, J. V.; Funes-Ardoiz, I.; Paton, R. S. GoodVibes: Automated Thermochemistry for Heterogeneous Computational Chemistry Data [version 1; peer review: 2 approved with reservations]. F1000 Research **2020**, 9 (Chem Inf Sci), 291.

[28] Gaussian 03, Revision C.02, Frisch, M. J.; Trucks, G. W.; Schlegel, H. B.; Scuseria, G. E.; Robb, M. A.; Cheeseman, J. R.; Montgomery, Jr., J. A.; Vreven, T.; Kudin, K. N.; Burant, J. C.; Millam, J. M.; Iyengar, S. S.; Tomasi, J.; Barone, V.; Mennucci, B.; Cossi, M.; Scalmani, G.; Rega, N.; Petersson, G. A.; Nakatsuji, H.; Hada, M.; Ehara, M.; Toyota, K.; Fukuda, R.; Hasegawa, J.; Ishida, M.; Nakajima, T.; Honda, Y.; Kitao, O.; Nakai, H.; Klene, M.; Li, X.; Knox, J. E.; Hratchian, H. P.; Cross, J. B.; Bakken, V.; Adamo, C.; Jaramillo, J.; Gomperts, R.; Stratmann, R. E.; Yazyev, O.; Austin, A. J.; Cammi, R.; Pomelli, C.; Ochterski, J. W.; Ayala, P. Y.; Morokuma, K.; Voth, G. A.; Salvador, P.; Dannenberg, J. J.; Zakrzewski, V. G.; Dapprich, S.; Daniels, A. D.; Strain, M. C.; Farkas, O.; Malick, D. K.; Rabuck, A. D.; Raghavachari, K.; Foresman, J. B.; Ortiz, J. V.; Cui, Q.; Baboul, A. G.; Clifford, S.; Cioslowski, J.; Stefanov, B. B.; Liu, G.; Liashenko, A.; Piskorz, P.; Komaromi, I.; Martin, R. L.; Fox, D. J.; Keith, T.; Al-Laham, M. A.; Peng, C. Y.; Nanayakkara, A.; Challacombe, M.; Gill, P. M.

W.; Johnson, B.; Chen, W.; Wong, M. W.; Gonzalez, C.; and Pople, J. A.; Gaussian, Inc., Wallingford CT, **2004**.

[29] Reid, J. P.; Goodman, J. M. J. Am. Chem. Soc. **2016**, *138*, 7910–7917

Quinoline s-trans
Electronic energy (Hartrees): -938.940296
Free Energy at 1M concentration and 253 K (Hartrees): -938.807679
Coordinates:

C	-3.94706	-0.25198	0.00021
C	-2.97021	-1.22144	0.00005
C	-1.59872	-0.85759	0.00004
C	-1.24046	0.52908	0.00019
C	-2.27339	1.50640	0.00036
C	-3.59401	1.12182	0.00037
H	-0.76013	-2.85689	-0.00029
H	-4.99519	-0.53568	0.00021
H	-3.23523	-2.27556	-0.00007
C	-0.53647	-1.79314	-0.00013
H	-1.97986	2.55088	0.00049
H	-4.37780	1.87349	0.00049
C	1.02173	0.04565	0.00013
C	0.76537	-1.35491	-0.00012
H	1.58741	-2.05839	-0.00029
C	2.40360	0.60218	-0.00004
C	2.68984	1.90536	0.00021
H	1.87098	2.61435	0.00058
H	3.71018	2.26679	0.00009
N	0.05108	0.94828	0.00022
Cl	3.73534	-0.56503	-0.00061

Quinoline s-cis
Electronic energy (Hartrees): -938.936753
Free Energy at 1M concentration and 253 K (Hartrees): -938.804198
Coordinates:

C	-3.86781	-0.20572	-0.00664
C	-3.09395	0.92807	-0.11961
C	-1.67973	0.83595	-0.07900
C	-1.06436	-0.44622	0.07823
C	-1.88902	-1.59713	0.18967
C	-3.25975	-1.47629	0.14864
H	-1.23580	2.94411	-0.34610
H	-4.95060	-0.13004	-0.03673
H	-3.55572	1.90457	-0.24061
C	-0.81412	1.95284	-0.20087
H	-1.39932	-2.55790	0.30730
H	-3.88512	-2.35985	0.23528
C	1.06249	0.45243	0.02875
C	0.54402	1.77124	-0.15310
H	1.21744	2.61057	-0.27929
C	2.53325	0.24979	0.11727
C	3.41646	1.18209	0.48881
H	3.08647	2.17230	0.78118
H	4.47787	0.97288	0.53196
N	0.28638	-0.61094	0.12625
Cl	3.11266	-1.36250	-0.28174

Aniline
Electronic energy (Hartrees): -287.616504
Free Energy at 1M concentration and 253 K (Hartrees): -287.520707
Coordinates:

C	-1.17129	-1.20244	0.00333
C	0.22137	-1.20814	-0.00518
C	0.93906	0.00020	-0.00986
C	0.22127	1.20823	-0.00510

C	-1.17160	1.20224	0.00333
C	-1.88123	-0.00006	0.00811
H	-1.70505	-2.14894	0.00866
H	0.76320	-2.15102	-0.01267
H	0.76258	2.15139	-0.01264
H	-1.70530	2.14878	0.00867
H	-2.96651	-0.00028	0.01622
N	2.33564	-0.00003	-0.07679
H	2.77829	0.83577	0.28034
H	2.77788	-0.83569	0.28120

Product (lowest energy conformation)
Electronic energy (Hartrees): -1226.583707
Free Energy at 1M concentration and 253 K (Hartrees): -1226.334166
Coordinates:

C	-4.89011	-1.72387	-0.12684
C	-4.38018	-0.85965	-1.07031
C	-3.14448	-0.20094	-0.84641
C	-2.43357	-0.44468	0.36989
C	-2.98290	-1.34026	1.32305
C	-4.18579	-1.96469	1.07805
H	-3.06134	0.90509	-2.71291
H	-5.83694	-2.22495	-0.30366
H	-4.91695	-0.67131	-1.99633
C	-2.55545	0.69797	-1.77355
H	-2.42917	-1.50732	2.24119
H	-4.60183	-2.64749	1.81275
C	-0.72676	0.99327	-0.23290
C	-1.35672	1.29218	-1.47430
H	-0.88765	1.99117	-2.15848
C	0.60360	1.64079	0.10465
H	1.28675	1.54411	-0.74232
C	1.26948	1.11659	1.38645
H	2.14177	1.73480	1.61101
H	0.56937	1.22553	2.21672
N	1.65199	-0.27604	1.29694
H	0.85226	-0.89327	1.34579
C	2.70057	-0.73755	0.50363
C	3.72989	0.10030	0.03351
C	2.76613	-2.11060	0.19033
C	4.78267	-0.42737	-0.71468
H	3.71852	1.16211	0.25527
C	3.82053	-2.62222	-0.55693
H	1.98073	-2.77245	0.54779
C	4.84141	-1.78660	-1.01892
H	5.56472	0.24144	-1.06396
H	3.84334	-3.68512	-0.78177
H	5.66285	-2.18803	-1.60352
N	-1.23849	0.15669	0.64920
Cl	0.35701	3.46383	0.27418

cis-Enamine (lowest energy conformation)
Electronic energy (Hartrees): -1226.559351
Free Energy at 1M concentration and 253 K (Hartrees): -1226.311208
Coordinates:

C	5.84467	-1.25387	-0.32302
C	4.66595	-1.91511	0.00385
C	3.45382	-1.21787	0.11859
C	3.44366	0.17999	-0.10644
C	4.63246	0.84579	-0.43842

C	5.81995	0.12887	-0.54339
H	2.17995	-2.92348	0.59943
H	6.77577	-1.80440	-0.40732
H	4.66820	-2.98818	0.17611
C	2.19401	-1.84791	0.44643
H	4.61882	1.91874	-0.61059
H	6.73546	0.65358	-0.79961
C	1.02536	0.30219	0.34440
C	1.04769	-1.13779	0.55088
H	0.10929	-1.63313	0.76274
C	-0.11105	1.04800	0.46822
C	-1.46855	0.53739	0.83550
H	-1.36353	-0.27863	1.56186
H	-2.02202	1.33567	1.35250
N	2.24942	0.86775	0.00537
Cl	-0.02740	2.78616	0.09763
N	-2.20779	0.04297	-0.32920
H	2.25859	1.86617	-0.15102
C	-3.56839	-0.24492	-0.25425
C	-4.31692	-0.37371	-1.44213
C	-4.22393	-0.45541	0.97314
C	-5.66601	-0.70340	-1.40000
H	-3.82418	-0.21500	-2.39864
C	-5.57900	-0.78890	0.99979
H	-3.68051	-0.36323	1.90695
C	-6.31265	-0.91560	-0.17823
H	-6.21878	-0.79369	-2.33120
H	-6.06273	-0.94637	1.96011
H	-7.36673	-1.17228	-0.14869
H	-1.91957	0.47804	-1.19526

trans-Enamine (lowest energy conformation)
Electronic energy (Hartrees): -1226.558962
Free Energy at 1M concentration and 253 K (Hartrees): -1226.309857
Coordinates:

C	-4.46078	-2.64211	-0.05353
C	-4.61768	-1.27019	0.10742
C	-3.51232	-0.40635	0.07072
C	-2.22149	-0.95043	-0.14207
C	-2.06451	-2.33675	-0.29890
C	-3.17788	-3.16865	-0.25292
H	-4.58898	1.45448	0.44119
H	-5.32336	-3.29961	-0.02298
H	-5.60512	-0.84585	0.26980
C	-3.60757	1.02364	0.26172
H	-1.07254	-2.74972	-0.46075
H	-3.04505	-4.23938	-0.37679
C	-1.19328	1.28557	-0.03186
C	-2.51697	1.82340	0.22439
H	-2.60298	2.89259	0.36730
C	-0.04233	2.01396	-0.14282
C	1.28271	1.42471	-0.54413
H	1.19691	0.98365	-1.54846
H	2.03972	2.21712	-0.60671
N	-1.13220	-0.10312	-0.19032
Cl	-0.06797	3.76358	0.09204
N	1.68580	0.34486	0.37366
H	-0.20045	-0.50877	-0.15650
C	2.92236	-0.30733	0.20670
C	3.44821	-1.04915	1.28099

C	3.63323	-0.28291	-1.00411
C	4.64323	-1.74625	1.14458
H	2.90624	-1.07639	2.22351
C	4.82907	-0.99270	-1.13050
H	3.26270	0.28734	-1.84814
C	5.34452	-1.72710	-0.06453
H	5.02952	-2.30874	1.98996
H	5.36266	-0.96001	-2.07631
H	6.27702	-2.27230	-0.16938
H	1.51290	0.60446	1.33906

THF
Electronic energy (Hartrees): -232.460288
Free Energy at 1M concentration and 253 K (Hartrees): -232.363932
Coordinates:

C	-1.16563	-0.43033	-0.13198
C	0.73336	0.99631	-0.22725
C	-0.73336	0.99630	0.22726
H	-1.53581	-0.48110	-1.16752
H	-1.95028	-0.82142	0.52577
H	1.34359	1.76059	0.26205
H	0.79588	1.15402	-1.30992
H	-0.79588	1.15395	1.30995
H	-1.34359	1.76061	-0.26199
O	-0.00001	-1.25196	0.00004
C	1.16563	-0.43035	0.13193
H	1.53593	-0.48114	1.16743
H	1.95022	-0.82142	-0.52590

Catalyst (TRIP, 3)-THF complex (lowest energy conformation)
Electronic energy (Hartrees): -876.434398
Free Energy at 1M concentration and 253 K (Hartrees): -875.369895
Coordinates:

P	0.08445	-1.03788	0.26219
O	-0.97219	-0.05604	-0.52873
O	1.36953	-0.13698	0.69687
O	-0.48099	-1.73076	1.43825
O	0.59124	-1.92457	-0.94444
C	-1.22374	1.12592	0.11931
C	-2.59961	1.41605	0.59831
C	-2.86080	2.57994	1.21149
H	-3.86720	2.80684	1.54343
C	-1.78477	3.56619	1.43358
C	-2.07027	4.80877	2.18732
H	-3.07010	5.00494	2.55424
C	-1.09396	5.69248	2.42071
H	-1.30282	6.59938	2.97334
C	0.27438	5.42833	1.93619
H	1.06456	6.13727	2.14732
C	0.54129	4.31168	1.24906
H	1.55652	4.12750	0.92358
C	-0.54046	3.33226	0.95641
C	-0.26952	2.07928	0.18985
C	1.79079	0.79849	-0.21676
C	3.17951	0.71451	-0.73679
C	3.63288	1.64449	-1.58994
H	4.65579	1.60543	-1.94550
C	2.74820	2.73361	-2.04671
C	3.23342	3.69753	-3.06113
H	4.24182	3.61864	-3.44801

C	2.42752	4.66746	-3.50610
H	2.78209	5.37022	-4.24899
C	1.04781	4.77511	-2.99504
H	0.39344	5.54860	-3.37597
C	0.60207	3.91318	-2.07376
H	-0.42089	3.99681	-1.73178
C	1.49632	2.84611	-1.54629
C	1.02498	1.87388	-0.51373
C	4.09930	-0.33274	-0.23440
C	4.69617	-0.18029	1.13703
C	4.43899	-1.40157	-1.00810
C	5.55785	-1.11481	1.58094
C	5.43116	-2.38025	-0.49082
C	5.95560	-2.26201	0.73682
H	5.99402	-1.02471	2.56651
H	5.73416	-3.20558	-1.12167
C	3.85108	-1.63646	-2.39954
H	3.00684	-0.94382	-2.59831
C	3.27365	-3.05376	-2.53875
H	2.64339	-3.30581	-1.66122
H	2.64389	-3.11711	-3.45189
H	4.07762	-3.81472	-2.62265
C	4.90794	-1.37684	-3.47851
H	5.76034	-2.08236	-3.37978
H	4.46023	-1.49899	-4.48790
H	5.29347	-0.33901	-3.39418
C	4.36705	1.01843	2.02725
H	3.66505	1.70653	1.51708
C	3.67189	0.57822	3.32227
H	2.77503	-0.03257	3.08525
H	4.35338	-0.01981	3.96321
H	3.34122	1.46946	3.89730
C	5.62032	1.85078	2.32857
H	6.34660	1.28136	2.94591
H	6.11266	2.15154	1.37898
H	5.33967	2.77326	2.88056
C	-3.70351	0.46258	0.33690
C	-4.26677	0.37301	-0.90057
C	-4.26558	-0.34317	1.47460
C	-5.43994	-0.51878	-1.09671
C	-5.36112	-1.09713	1.26256
C	-5.98008	-1.19958	-0.07677
H	-5.88191	-0.59683	-2.08146
H	-5.79813	-1.65920	2.07631
C	-3.78193	1.19427	-2.09682
H	-2.93101	1.85101	-1.82750
C	-4.88439	2.13172	-2.60446
H	-5.24947	2.77216	-1.77309
H	-4.48234	2.79425	-3.40065
H	-5.74065	1.56302	-3.02364
C	-3.27668	0.28037	-3.21905
H	-2.47310	-0.38129	-2.83426
H	-4.09413	-0.34903	-3.62895
H	-2.85422	0.89068	-4.04563
C	-3.62814	-0.31467	2.86219
H	-2.66416	0.23320	2.82936
C	-4.52862	0.42326	3.85960
H	-4.02176	0.50126	4.84509
H	-4.73895	1.45128	3.49618
H	-5.49285	-0.11081	3.99752

C	-3.29336	-1.72546	3.37175
H	-2.73931	-2.29564	2.59879
H	-2.65498	-1.65774	4.27844
H	-4.21108	-2.29083	3.63754
C	-7.21428	-2.05732	-0.29331
H	-7.54462	-1.98743	-1.35434
C	-6.91471	-3.53459	-0.01719
H	-6.10674	-3.88861	-0.69081
H	-6.60291	-3.69278	1.03685
H	-7.81837	-4.14984	-0.21447
C	-8.38161	-1.56288	0.56892
H	-8.57639	-0.48806	0.36547
H	-9.30279	-2.13390	0.32487
H	-8.16515	-1.69055	1.65065
C	6.95463	-3.28416	1.24830
H	7.12867	-4.06586	0.47470
C	6.41623	-3.99998	2.49243
H	5.43216	-4.46444	2.26781
H	7.11715	-4.80486	2.80081
H	6.29473	-3.29446	3.34151
C	8.31091	-2.62921	1.53434
H	8.68217	-2.11071	0.62450
H	8.23551	-1.89240	2.36190
H	9.05424	-3.40403	1.81950
H	0.06548	-2.77878	-1.04198
O	-0.96406	-3.98768	-1.26190
C	-1.86767	-3.64080	-2.34484
C	-1.73632	-4.29138	-0.07004
C	-3.28482	-3.61769	-1.74530
H	-1.55713	-2.66282	-2.72482
H	-1.75538	-4.37875	-3.14588
C	-3.01961	-3.48816	-0.23666
H	-1.92657	-5.37249	-0.03979
H	-1.14161	-3.98808	0.79238
H	-3.80752	-4.55522	-1.96225
H	-3.88669	-2.79978	-2.14852
H	-3.83593	-3.86523	0.38435
H	-2.82800	-2.44835	0.03966

Catalyst (TRIP, 3)-THF complex (alternative conformation)
Electronic energy (Hartrees): -876.433504
Free Energy at 1M concentration and 253 K (Hartrees): -875.369115
Coordinates:

P	-0.10898	1.01161	-0.08490
O	1.01745	-0.03248	-0.66719
O	-1.30100	0.13081	0.59088
O	0.47521	1.65524	1.23549
O	-0.47916	1.93006	-1.18378
C	1.24378	-1.17392	0.06324
C	2.62985	-1.46402	0.51769
C	2.89854	-2.61197	1.15666
H	3.91090	-2.83562	1.47207
C	1.81993	-3.57351	1.45242
C	2.11553	-4.78724	2.24817
H	3.12586	-4.98468	2.58440
C	1.13562	-5.64291	2.55775
H	1.35192	-6.52872	3.14087
C	-0.24652	-5.37501	2.11707
H	-1.03921	-6.05919	2.39109
C	-0.52251	-4.28435	1.39285

H	-1.54744	-4.09545	1.10248
C	0.56270	-3.33835	1.01316
C	0.28286	-2.11885	0.19582
C	-1.77976	-0.85990	-0.22696
C	-3.18550	-0.79511	-0.70028
C	-3.67529	-1.77422	-1.47497
H	-4.70985	-1.74878	-1.79647
C	-2.81305	-2.89690	-1.89394
C	-3.34099	-3.92171	-2.82379
H	-4.36378	-3.86260	-3.17474
C	-2.55623	-4.92296	-3.23618
H	-2.94219	-5.67057	-3.91705
C	-1.15731	-5.00456	-2.77459
H	-0.52079	-5.80443	-3.13042
C	-0.67260	-4.08709	-1.92979
H	0.36309	-4.15297	-1.62415
C	-1.54236	-2.98428	-1.43695
C	-1.02832	-1.95116	-0.48751
C	-4.07685	0.29399	-0.23834
C	-4.62941	0.23982	1.15857
C	-4.43083	1.31336	-1.06991
C	-5.47061	1.20936	1.56526
C	-5.39999	2.33287	-0.58942
C	-5.88783	2.30052	0.65840
H	-5.87445	1.19032	2.56838
H	-5.71674	3.11697	-1.26456
C	-3.88047	1.45251	-2.48921
H	-3.04896	0.73903	-2.66692
C	-3.29278	2.85113	-2.73314
H	-2.63127	3.14701	-1.89299
H	-2.69278	2.85104	-3.66823
H	-4.09074	3.61589	-2.83731
C	-4.96970	1.13762	-3.52011
H	-5.81196	1.85783	-3.44323
H	-4.54925	1.19040	-4.54709
H	-5.36272	0.11164	-3.35937
C	-4.27472	-0.89607	2.11843
H	-3.59696	-1.62477	1.63240
C	-3.52779	-0.37117	3.35146
H	-2.63544	0.21096	3.03741
H	-4.17979	0.27925	3.97175
H	-3.18300	-1.22202	3.97714
C	-5.52065	-1.69394	2.52509
H	-6.21859	-1.07653	3.12879
H	-6.05158	-2.05549	1.61846
H	-5.22455	-2.57833	3.12892
C	3.73140	-0.51312	0.23089
C	4.24392	-0.39403	-1.02683
C	4.33517	0.27489	1.36146
C	5.39816	0.51517	-1.25338
C	5.40716	1.05255	1.11652
C	5.96757	1.18898	-0.24517
H	5.79976	0.61561	-2.25327
H	5.86881	1.60823	1.92101
C	3.71908	-1.19313	-2.22081
H	2.87182	-1.84786	-1.93660
C	4.80098	-2.13033	-2.77101
H	5.17592	-2.79240	-1.96125
H	4.37627	-2.77100	-3.57329
H	5.65496	-1.56081	-3.19401

C	3.19206	-0.25917	-3.31819
H	2.43360	0.43535	-2.89955
H	4.01114	0.33914	-3.76924
H	2.70951	-0.85402	-4.12287
C	3.76327	0.20914	2.77696
H	2.81838	-0.37089	2.78458
C	4.73321	-0.50600	3.72504
H	4.27074	-0.61903	4.72881
H	4.96974	-1.51918	3.33694
H	5.68000	0.06468	3.83331
C	3.40209	1.60126	3.31635
H	2.77119	2.14482	2.58524
H	2.82931	1.50292	4.26318
H	4.30922	2.20702	3.52246
C	7.17160	2.07869	-0.49918
H	7.46681	2.02232	-1.57132
C	6.84129	3.54596	-0.20553
H	6.00289	3.88081	-0.85227
H	6.55797	3.68980	0.85866
H	7.72149	4.18651	-0.42699
C	8.38139	1.61207	0.31909
H	8.59465	0.54245	0.10700
H	9.27878	2.20556	0.04223
H	8.20239	1.73404	1.40824
C	-6.86497	3.36225	1.12987
H	-7.05751	4.09217	0.31136
C	-6.28533	4.15495	2.30704
H	-5.30519	4.59474	2.02335
H	-6.97143	4.98471	2.58081
H	-6.14368	3.50679	3.19768
C	-8.21590	2.73843	1.49869
H	-8.61779	2.16464	0.63626
H	-8.11984	2.05661	2.37008
H	-8.94549	3.53622	1.75451
H	0.90886	2.54281	1.03106
O	1.72332	3.80797	0.48277
C	0.95220	4.74197	-0.32725
C	2.84928	3.30544	-0.28068
C	1.83389	5.05466	-1.53714
H	0.02360	4.24719	-0.62657
H	0.73092	5.61356	0.29514
C	2.60232	3.73719	-1.72453
H	3.76436	3.73872	0.13933
H	2.88079	2.22114	-0.15921
H	2.52406	5.87587	-1.31290
H	1.24270	5.33367	-2.41300
H	3.53142	3.84944	-2.28982
H	1.96806	3.00176	-2.22742

Catalyst (TRIP, 3)-Quinoline complex s-trans (lowest energy conformation)
Electronic energy (Hartrees): -1582.918343
Free Energy at 1M concentration and 253 K (Hartrees): -1581.816098
Coordinates:

P	0.28036	-0.26697	-1.17936
O	-0.77095	0.32155	-0.03942
O	1.62519	0.65368	-1.14458
O	-0.27051	-0.31705	-2.54764
O	0.69636	-1.60872	-0.47266
C	-0.92434	1.68253	-0.07353
C	-2.26357	2.25523	-0.35511

C	-2.42437	3.58603	-0.39617
H	-3.40413	4.01063	-0.58252
C	-1.27730	4.48510	-0.15563
C	-1.45338	5.95102	-0.27252
H	-2.42225	6.36429	-0.52397
C	-0.41596	6.77060	-0.07001
H	-0.54489	7.84172	-0.15722
C	0.91050	6.21700	0.26397
H	1.74968	6.88506	0.40890
C	1.07880	4.89441	0.37764
H	2.06386	4.50840	0.60450
C	-0.07083	3.97124	0.17862
C	0.09108	2.49205	0.29731
C	2.04784	1.01404	0.11174
C	3.36891	0.53657	0.59530
C	3.81537	0.91623	1.80152
H	4.79272	0.59928	2.14520
C	2.98045	1.76137	2.67656
C	3.44404	2.08194	4.04609
H	4.40687	1.72651	4.39160
C	2.67380	2.80721	4.86379
H	3.01206	3.03868	5.86552
C	1.35427	3.28334	4.40756
H	0.72589	3.85037	5.08222
C	0.92953	3.00539	3.16963
H	-0.05221	3.34405	2.86699
C	1.78747	2.22355	2.23719
C	1.33530	1.89470	0.85186
C	4.23043	-0.29561	-0.27773
C	4.97181	0.36034	-1.40912
C	4.40063	-1.62663	-0.03545
C	5.79692	-0.38579	-2.16779
C	5.35996	-2.39502	-0.87105
C	6.01553	-1.82163	-1.88956
H	6.33611	0.06763	-2.98840
H	5.53152	-3.44002	-0.64858
C	3.65307	-2.36976	1.07245
H	2.83963	-1.74181	1.48862
C	2.97520	-3.64561	0.54651
H	2.44667	-3.44479	-0.40784
H	2.23301	-4.01238	1.28712
H	3.71364	-4.45592	0.37154
C	4.59900	-2.70523	2.23019
H	5.41126	-3.38539	1.89596
H	4.03545	-3.19863	3.05091
H	5.05603	-1.77810	2.63416
C	4.82949	1.85370	-1.70373
H	4.13608	2.33213	-0.98496
C	4.23555	2.09338	-3.09800
H	3.27433	1.54668	-3.20224
H	4.92892	1.75409	-3.89613
H	4.03527	3.17634	-3.24420
C	6.16845	2.58504	-1.54055
H	6.90600	2.25566	-2.30236
H	6.58494	2.39154	-0.52878
H	6.01844	3.68048	-1.64977
C	-3.44544	1.37162	-0.48704
C	-4.08589	0.90136	0.61942
C	-4.01238	1.10011	-1.85018
C	-5.37533	0.18022	0.45518

C	-5.19891	0.47125	-1.95140
C	-5.92624	-0.00059	-0.75286
H	-5.89698	-0.17098	1.33600
H	-5.63743	0.29414	-2.92379
C	-3.56779	1.13144	2.03961
H	-2.58800	1.64920	2.03984
C	-4.52375	2.02993	2.83198
H	-4.67030	2.99180	2.29531
H	-4.09432	2.25282	3.83213
H	-5.51173	1.54355	2.97394
C	-3.33916	-0.20177	2.76141
H	-2.70969	-0.86442	2.13396
H	-4.29754	-0.71977	2.97450
H	-2.81194	-0.02875	3.72389
C	-3.27778	1.53579	-3.11479
H	-2.26030	1.89693	-2.85880
C	-4.00778	2.70013	-3.79333
H	-3.42862	3.05173	-4.67374
H	-4.11096	3.54917	-3.08457
H	-5.01977	2.39270	-4.13307
C	-3.07996	0.36842	-4.09452
H	-2.67402	-0.51675	-3.56289
H	-2.36038	0.65986	-4.88913
H	-4.03475	0.07991	-4.58214
C	-7.28140	-0.67222	-0.88633
H	-7.69240	-0.90305	0.12248
C	-7.16012	-2.00370	-1.63238
H	-6.48292	-2.68077	-1.07283
H	-6.76164	-1.85739	-2.65871
H	-8.15406	-2.49468	-1.70334
C	-8.29027	0.25530	-1.57410
H	-8.34840	1.22342	-1.03197
H	-9.29989	-0.20814	-1.56431
H	-8.00428	0.45030	-2.62924
C	6.98025	-2.63171	-2.73662
H	7.01193	-3.68440	-2.37518
C	6.52069	-2.67577	-4.19846
H	5.48541	-3.07451	-4.25969
H	7.18526	-3.34502	-4.78553
H	6.54392	-1.66505	-4.65816
C	8.40544	-2.07805	-2.62452
H	8.71901	-2.04826	-1.55901
H	8.47355	-1.05344	-3.04757
H	9.11232	-2.73432	-3.17572
H	-0.06508	-2.09118	0.01362
C	-2.28165	-3.35616	-0.05060
C	-1.18224	-3.27979	2.00962
C	-2.37434	-2.91292	-1.39702
C	-3.24825	-4.28387	0.45059
C	-2.09912	-4.20480	2.57660
C	-3.38643	-3.38549	-2.20234
H	-1.65370	-2.20471	-1.79119
C	-4.27080	-4.75466	-0.41162
C	-3.11893	-4.69486	1.79976
H	-1.98742	-4.50194	3.61168
C	-4.33357	-4.31804	-1.71516
H	-3.45596	-3.03829	-3.22835
H	-4.99841	-5.46344	-0.02612
H	-3.83688	-5.39838	2.21280
H	-5.11788	-4.67942	-2.37316

N	-1.26738	-2.89150	0.74235
C	-0.11553	-2.65608	2.83634
C	0.27348	-1.38387	2.74328
H	1.05762	-0.98752	3.37766
H	-0.19241	-0.72532	2.01924
Cl	0.62689	-3.71356	4.04461

Catalyst (TRIP, 3)-Quinoline complex s-trans (alternative conformation)
Electronic energy (Hartrees): -1582.917249
Free Energy at 1M concentration and 253 K (Hartrees): -1581.815461
Coordinates:

P	0.24296	-0.20499	-1.34530
O	-0.85795	0.28219	-0.20625
O	1.61580	0.65158	-1.12151
O	-0.20481	-0.06691	-2.74107
O	0.55967	-1.64183	-0.76497
C	-0.96427	1.64183	-0.07053
C	-2.26200	2.30132	-0.35687
C	-2.37547	3.63241	-0.24388
H	-3.32835	4.11541	-0.42705
C	-1.21634	4.44519	0.17841
C	-1.33504	5.92007	0.23943
H	-2.27004	6.40161	-0.01874
C	-0.28772	6.66366	0.61234
H	-0.37488	7.74164	0.65539
C	0.99327	6.01775	0.95767
H	1.84208	6.62680	1.24085
C	1.10946	4.68554	0.91155
H	2.06364	4.23423	1.14942
C	-0.05364	3.84308	0.52232
C	0.05142	2.35493	0.45935
C	1.98319	0.85645	0.18616
C	3.29516	0.33825	0.65663
C	3.68427	0.55682	1.92200
H	4.65184	0.20778	2.26256
C	2.79629	1.26269	2.86564
C	3.18281	1.38081	4.29040
H	4.12845	0.97924	4.63269
C	2.36129	1.97514	5.16223
H	2.64183	2.05634	6.20440
C	1.06456	2.51278	4.70832
H	0.39598	2.97556	5.42269
C	0.71077	2.42007	3.42133
H	-0.25434	2.80155	3.11533
C	1.62320	1.78011	2.43500
C	1.24183	1.64482	0.99776
C	4.21574	-0.33345	-0.29293
C	4.90922	0.49220	-1.34211
C	4.46262	-1.67251	-0.21193
C	5.73443	-0.11856	-2.21329
C	5.40969	-2.29203	-1.17545
C	6.00356	-1.57020	-2.13544
H	6.24083	0.45845	-2.97507
H	5.61895	-3.35112	-1.09947
C	3.81264	-2.57749	0.83477
H	3.05300	-2.02869	1.42120
C	3.07350	-3.75553	0.18424
H	2.42070	-3.39746	-0.63747
H	2.43612	-4.26458	0.93889
H	3.78326	-4.50173	-0.23021

C	4.85595	-3.08033	1.83854
H	5.61816	-3.71610	1.34019
H	4.36114	-3.67760	2.63414
H	5.36805	-2.22029	2.31965
C	4.73794	2.01018	-1.41457
H	4.04749	2.36651	-0.62560
C	4.12496	2.44294	-2.75329
H	3.17700	1.89458	-2.93619
H	4.81918	2.24734	-3.59743
H	3.89691	3.53006	-2.73185
C	6.06714	2.73284	-1.15866
H	6.80164	2.52857	-1.96594
H	6.49728	2.40274	-0.18880
H	5.89924	3.82996	-1.10759
C	-3.46113	1.47900	-0.63100
C	-4.14761	0.89838	0.39231
C	-3.93448	1.30534	-2.04516
C	-5.33075	0.05522	0.08003
C	-5.05029	0.58747	-2.27565
C	-5.77225	-0.09069	-1.17684
H	-5.84832	-0.44481	0.88835
H	-5.42794	0.48053	-3.28301
C	-3.76552	1.09066	1.86172
H	-2.91360	1.79147	1.97592
C	-4.92046	1.70935	2.65866
H	-5.25145	2.65350	2.17537
H	-4.58404	1.94814	3.69031
H	-5.78508	1.01653	2.72658
C	-3.31433	-0.23287	2.48370
H	-2.45662	-0.62983	1.90749
H	-4.13092	-0.98511	2.48094
H	-2.98089	-0.07231	3.53119
C	-3.19930	1.95543	-3.21440
H	-2.21398	2.33934	-2.87893
C	-3.98957	3.15565	-3.74639
H	-3.42028	3.65827	-4.55724
H	-4.15673	3.89206	-2.93167
H	-4.97522	2.83636	-4.14755
C	-2.90228	0.95401	-4.34258
H	-2.48840	0.01236	-3.92695
H	-2.15702	1.38587	-5.04424
H	-3.81792	0.70986	-4.92124
C	-6.98946	-0.94919	-1.46896
H	-7.39980	-1.36270	-0.52006
C	-6.61515	-2.14517	-2.35135
H	-5.82735	-2.74953	-1.85448
H	-6.24179	-1.81274	-3.34309
H	-7.50184	-2.79595	-2.50711
C	-8.10573	-0.11829	-2.11216
H	-8.35338	0.75105	-1.46605
H	-9.02139	-0.73686	-2.22668
H	-7.80511	0.25297	-3.11464
C	6.95466	-2.23053	-3.11721
H	7.02742	-3.32050	-2.90161
C	6.43904	-2.09367	-4.55439
H	5.41293	-2.51279	-4.63169
H	7.09717	-2.65752	-5.24948
H	6.41755	-1.03038	-4.87444
C	8.36860	-1.65346	-2.98276
H	8.72281	-1.75555	-1.93460

H	8.39422	-0.58030	-3.26744
H	9.06983	-2.20916	-3.64131
H	-0.19869	-2.07661	-0.24802
C	-0.79507	-2.80220	2.14409
C	-2.33553	-3.49889	0.54224
C	0.33388	-2.00951	2.47352
C	-1.45006	-3.54460	3.17346
C	-3.03410	-4.27966	1.50460
C	0.79707	-1.96820	3.76839
H	0.80145	-1.44141	1.68138
C	-0.94472	-3.48521	4.49734
C	-2.59132	-4.29903	2.80264
H	-3.91253	-4.83540	1.20122
C	0.15722	-2.71283	4.78891
H	1.66101	-1.35802	4.01331
H	-1.44509	-4.05412	5.27606
H	-3.11649	-4.87881	3.55691
H	0.53771	-2.66834	5.80457
N	-1.24258	-2.81197	0.85065
C	-2.84064	-3.37185	-0.84792
C	-2.83767	-2.23687	-1.54729
H	-3.21370	-2.18674	-2.56142
H	-2.46686	-1.32548	-1.09462
Cl	-3.51844	-4.84748	-1.54816

Catalyst (TRIP, 3)-Quinoline complex s-trans (alternative conformation)
Electronic energy (Hartrees): -1582.918004
Free Energy at 1M concentration and 253 K (Hartrees): -1581.815107
Coordinates:

P	0.05147	-0.17218	-0.60985
O	1.14902	1.00290	-0.89200
O	-1.17953	0.50638	0.23982
O	0.65538	-1.08388	0.52352
O	-0.31173	-0.81864	-1.88902
C	1.40861	1.83363	0.16820
C	2.78252	1.86911	0.73036
C	3.07469	2.71954	1.72477
H	4.08180	2.76821	2.12135
C	2.02785	3.59114	2.29447
C	2.34420	4.45633	3.45417
H	3.34728	4.47418	3.86192
C	1.39192	5.21653	4.00514
H	1.62344	5.84695	4.85389
C	0.01880	5.18796	3.46647
H	-0.75269	5.78762	3.93205
C	-0.27598	4.41452	2.41524
H	-1.29526	4.39073	2.05355
C	0.77981	3.58631	1.77081
C	0.47877	2.71889	0.59193
C	-1.60549	1.71847	-0.23942
C	-2.99976	1.84790	-0.72976
C	-3.44933	3.03545	-1.16081
H	-4.47581	3.14197	-1.49149
C	-2.55390	4.20922	-1.18332
C	-3.03666	5.49087	-1.74713
H	-4.05044	5.57420	-2.11871
C	-2.22194	6.54992	-1.80259
H	-2.57483	7.48566	-2.21649
C	-0.83492	6.44272	-1.31128
H	-0.17403	7.29718	-1.37965

C	-0.39088	5.29111	-0.79460
H	0.63683	5.22871	-0.46246
C	-1.29423	4.11211	-0.69807
C	-0.82526	2.81636	-0.12040
C	-3.92400	0.69272	-0.65160
C	-4.52074	0.32478	0.67824
C	-4.26579	-0.00982	-1.76713
C	-5.38919	-0.70276	0.74162
C	-5.27199	-1.09779	-1.65430
C	-5.80398	-1.43904	-0.47260
H	-5.82473	-0.98616	1.69016
H	-5.58582	-1.61837	-2.54955
C	-3.67111	0.28437	-3.14401
H	-2.81944	0.99220	-3.06832
C	-3.10456	-0.98524	-3.79761
H	-2.47532	-1.54557	-3.07600
H	-2.47625	-0.71187	-4.67218
H	-3.91408	-1.65679	-4.15276
C	-4.71834	0.93791	-4.05200
H	-5.57709	0.25517	-4.22640
H	-4.26460	1.19632	-5.03261
H	-5.09563	1.87346	-3.58786
C	-4.19256	1.11041	1.94835
H	-3.48538	1.93422	1.72898
C	-3.50569	0.22520	2.99578
H	-2.60280	-0.25040	2.55917
H	-4.18926	-0.56751	3.36553
H	-3.18441	0.84218	3.86198
C	-5.44561	1.77952	2.52820
H	-6.17687	1.02599	2.88928
H	-5.93139	2.41024	1.75308
H	-5.16648	2.43352	3.38189
C	3.84589	1.00798	0.15788
C	4.41444	1.30948	-1.04365
C	4.34422	-0.17329	0.94531
C	5.51951	0.45617	-1.55404
C	5.38253	-0.88537	0.46766
C	5.99635	-0.57275	-0.84054
H	5.96323	0.68907	-2.51328
H	5.77777	-1.71599	1.03605
C	4.00341	2.52400	-1.87851
H	3.18600	3.09643	-1.39688
C	5.16999	3.50870	-2.02369
H	5.54478	3.80227	-1.01970
H	4.82898	4.42699	-2.54805
H	6.00607	3.06492	-2.60386
C	3.47360	2.09410	-3.25239
H	2.64908	1.35988	-3.13231
H	4.27370	1.63433	-3.86946
H	3.07457	2.97595	-3.79777
C	3.71562	-0.56688	2.28114
H	2.78023	0.00604	2.44603
C	4.65826	-0.23466	3.44363
H	4.16303	-0.46050	4.41210
H	4.91729	0.84476	3.43082
H	5.59615	-0.82638	3.37732
C	3.31434	-2.05052	2.31773
H	2.76594	-2.32510	1.39577
H	2.65208	-2.24241	3.18878
H	4.20214	-2.71130	2.40673

C	7.15315	-1.40272	-1.36766
H	7.47347	-1.01987	-2.36294
C	6.73600	-2.86397	-1.56552
H	5.85086	-2.91985	-2.23432
H	6.48720	-3.34443	-0.59595
H	7.56347	-3.43471	-2.03836
C	8.36757	-1.29950	-0.43789
H	8.65434	-0.23445	-0.30389
H	9.23225	-1.83823	-0.88096
H	8.15027	-1.74079	0.55788
C	-6.84067	-2.54530	-0.38863
H	-7.04011	-2.95723	-1.40368
C	-6.33370	-3.70767	0.47057
H	-5.37614	-4.09132	0.05864
H	-7.07096	-4.53853	0.46085
H	-6.17507	-3.38998	1.52273
C	-8.17445	-2.00847	0.14370
H	-8.52111	-1.16033	-0.48479
H	-8.07834	-1.66138	1.19424
H	-8.94693	-2.80601	0.10620
H	0.54810	-2.08872	0.34083
C	-0.90868	-4.09915	0.80293
C	1.12745	-4.55025	-0.24587
C	-1.83501	-3.14074	1.29198
C	-1.20329	-5.49114	0.93703
C	0.91215	-5.95141	-0.13834
C	-3.00253	-3.55993	1.88817
H	-1.60569	-2.08725	1.18861
C	-2.41662	-5.88621	1.55573
C	-0.24341	-6.40905	0.44256
H	1.65740	-6.63531	-0.52457
C	-3.29773	-4.93830	2.02391
H	-3.70974	-2.82486	2.25770
H	-2.63566	-6.94588	1.65371
H	-0.43389	-7.47571	0.52605
H	-4.22633	-5.24370	2.49612
N	0.25259	-3.66853	0.22205
C	2.33456	-4.00832	-0.91995
C	2.34852	-2.93799	-1.71506
H	1.42986	-2.40113	-1.93628
H	3.26874	-2.58551	-2.16576
Cl	3.82827	-4.91051	-0.61412

Catalyst (TRIP, 3)-Quinoline complex s-trans (alternative conformation)
Electronic energy (Hartrees): -1582.915488
Free Energy at 1M concentration and 253 K (Hartrees): -1581.813075
Coordinates:

P	0.08260	0.14067	-0.54060
O	-0.84034	-1.13105	-0.99359
O	1.30520	-0.46756	0.35834
O	-0.73051	0.89455	0.57376
O	0.48262	0.91725	-1.73374
C	-0.94547	-2.15277	-0.08200
C	-2.28131	-2.46345	0.49013
C	-2.44525	-3.56007	1.24325
H	-3.42119	-3.80027	1.64806
C	-1.28912	-4.40321	1.60085
C	-1.47601	-5.54336	2.52789
H	-2.46641	-5.79797	2.88438
C	-0.42271	-6.25976	2.93354

H	-0.55995	-7.09099	3.61304
C	0.93259	-5.90639	2.47222
H	1.78469	-6.47048	2.82912
C	1.11138	-4.88204	1.63030
H	2.11879	-4.62335	1.33471
C	-0.05550	-4.09890	1.13560
C	0.11174	-2.96161	0.17746
C	1.99391	-1.46027	-0.28813
C	3.40396	-1.22166	-0.68433
C	4.10426	-2.20437	-1.27036
H	5.14422	-2.05086	-1.53153
C	3.46017	-3.49644	-1.58500
C	4.21512	-4.53195	-2.32754
H	5.24623	-4.35678	-2.60847
C	3.62583	-5.68633	-2.65739
H	4.17775	-6.44185	-3.20151
C	2.21684	-5.92866	-2.29249
H	1.73946	-6.85590	-2.58208
C	1.52749	-5.00189	-1.61695
H	0.48907	-5.19048	-1.37889
C	2.17830	-3.72565	-1.21502
C	1.43546	-2.68051	-0.44913
C	4.05338	0.07702	-0.37526
C	4.41334	0.39678	1.05017
C	4.33979	0.97340	-1.36242
C	5.01254	1.57114	1.32498
C	5.04928	2.23197	-1.01256
C	5.35698	2.53244	0.25604
H	5.27241	1.82113	2.34454
H	5.31731	2.92174	-1.80197
C	3.96390	0.74499	-2.82686
H	3.28714	-0.12794	-2.93381
C	3.19780	1.94323	-3.40980
H	2.40114	2.27429	-2.71319
H	2.72419	1.65620	-4.37295
H	3.87222	2.80366	-3.60286
C	5.21495	0.45148	-3.66239
H	5.91082	1.31739	-3.65582
H	4.92861	0.23341	-4.71344
H	5.74772	-0.43447	-3.25841
C	4.13794	-0.58413	2.18958
H	3.65658	-1.50515	1.80755
C	3.17160	0.01452	3.22141
H	2.24759	0.37279	2.72040
H	3.63528	0.86592	3.76263
H	2.88460	-0.75899	3.96538
C	5.44128	-1.04289	2.85670
H	5.95109	-0.20170	3.37187
H	6.12858	-1.46705	2.09362
H	5.22588	-1.83367	3.60684
C	-3.42293	-1.53448	0.29646
C	-4.10344	-1.48885	-0.88252
C	-3.89667	-0.71033	1.46428
C	-5.32255	-0.64156	-0.97967
C	-5.04107	-0.01189	1.34982
C	-5.78578	0.04397	0.07517
H	-5.86526	-0.60689	-1.91531
H	-5.42251	0.54493	2.19446
C	-3.70658	-2.31835	-2.10335
H	-2.79098	-2.91294	-1.91321

C	-4.80101	-3.33322	-2.45453
H	-5.02012	-3.97377	-1.57343
H	-4.45892	-3.98929	-3.28334
H	-5.73665	-2.82697	-2.77228
C	-3.39007	-1.41469	-3.30212
H	-2.62469	-0.66006	-3.02215
H	-4.29748	-0.88596	-3.66160
H	-2.98619	-2.02318	-4.13922
C	-3.11615	-0.65788	2.77751
H	-2.12531	-1.14060	2.65062
C	-3.85504	-1.42211	3.88288
H	-3.24363	-1.43533	4.81034
H	-4.03400	-2.47230	3.57279
H	-4.83362	-0.94760	4.10931
C	-2.82815	0.78609	3.22290
H	-2.44218	1.38870	2.37649
H	-2.06419	0.78709	4.02954
H	-3.74218	1.28044	3.61418
C	-7.06641	0.85293	-0.03203
H	-7.43668	0.83841	-1.08199
C	-6.83499	2.32340	0.34008
H	-6.00049	2.74537	-0.25503
H	-6.59433	2.43522	1.41813
H	-7.74876	2.91666	0.12315
C	-8.16464	0.23771	0.84188
H	-8.33500	-0.82054	0.54918
H	-9.11690	0.79319	0.70452
H	-7.88593	0.27174	1.91675
C	6.06059	3.83607	0.58886
H	6.25604	4.41174	-0.34397
C	5.18181	4.71999	1.48143
H	4.21682	4.93148	0.97361
H	5.68848	5.68969	1.67390
H	4.97596	4.22947	2.45634
C	7.42200	3.57372	1.24331
H	8.03875	2.92035	0.58966
H	7.30546	3.08451	2.23342
H	7.96525	4.53210	1.38675
H	-0.91571	1.87107	0.30884
C	-2.39977	3.49734	-0.95510
C	-0.64836	4.42709	0.27629
C	-3.04201	2.33826	-1.46472
C	-2.90772	4.78922	-1.29453
C	-1.11734	5.74412	0.02154
C	-4.11907	2.47139	-2.31138
H	-2.66319	1.36152	-1.18931
C	-4.03608	4.88696	-2.14781
C	-2.23445	5.91514	-0.75710
H	-0.57577	6.59021	0.42623
C	-4.62255	3.74978	-2.65492
H	-4.59387	1.58653	-2.72177
H	-4.41933	5.87132	-2.40192
H	-2.60173	6.91263	-0.98321
H	-5.47783	3.82740	-3.31886
N	-1.29280	3.35462	-0.16562
C	0.62867	4.18674	0.99541
C	1.53639	3.28304	0.62458
H	1.37515	2.68391	-0.26644
H	2.44819	3.13374	1.18916
Cl	0.92281	5.22811	2.39576

Catalyst (TRIP, 3)-Quinoline complex s-trans (alternative conformation)
Electronic energy (Hartrees): -1582.914073
Free Energy at 1M concentration and 253 K (Hartrees): -1581.812569
Coordinates:

P	0.19954	-0.22238	-1.21782
O	-0.91345	0.19361	-0.07402
O	1.52893	0.70281	-0.99089
O	-0.25317	-0.12451	-2.61479
O	0.62907	-1.64114	-0.64838
C	-0.99591	1.53529	0.20174
C	-2.27694	2.24585	-0.04404
C	-2.39410	3.54451	0.26706
H	-3.33325	4.06074	0.10483
C	-1.24901	4.28313	0.83328
C	-1.37148	5.73511	1.09889
H	-2.30587	6.24686	0.90443
C	-0.32809	6.42120	1.57708
H	-0.41776	7.48261	1.76908
C	0.95306	5.73598	1.83236
H	1.80043	6.30166	2.19781
C	1.07227	4.42346	1.60138
H	2.02897	3.94866	1.77269
C	-0.08852	3.63921	1.09684
C	0.01712	2.17181	0.83295
C	1.95853	0.76530	0.31183
C	3.29759	0.22503	0.66073
C	3.72708	0.27657	1.93058
H	4.71532	-0.08655	2.18668
C	2.86068	0.83037	2.99018
C	3.30310	0.78151	4.40263
H	4.27134	0.36773	4.65540
C	2.50437	1.23897	5.37291
H	2.82567	1.19742	6.40557
C	1.17732	1.79151	5.04000
H	0.52794	2.14169	5.83190
C	0.77354	1.85159	3.76582
H	-0.21109	2.24237	3.54544
C	1.65983	1.36735	2.67346
C	1.22676	1.41133	1.24574
C	4.20129	-0.26743	-0.40648
C	4.85809	0.73029	-1.32040
C	4.46242	-1.59728	-0.55471
C	5.67614	0.28491	-2.29272
C	5.40767	-2.03388	-1.61533
C	5.97397	-1.15452	-2.45293
H	6.15319	0.98704	-2.96290
H	5.63724	-3.08689	-1.71376
C	3.82678	-2.67206	0.32628
H	3.04641	-2.23980	0.98086
C	3.12468	-3.75016	-0.51342
H	2.49120	-3.28365	-1.29590
H	2.47364	-4.37178	0.13817
H	3.85776	-4.41992	-1.00988
C	4.87498	-3.30200	1.24966
H	5.66234	-3.82294	0.66448
H	4.39168	-4.03710	1.92823
H	5.35424	-2.51811	1.87346
C	4.64616	2.23545	-1.15246
H	3.97380	2.44553	-0.29796

C	3.97565	2.84725	-2.38953
H	3.03176	2.30881	-2.61837
H	4.64132	2.79520	-3.27669
H	3.72788	3.91330	-2.19854
C	5.96378	2.95196	-0.82822
H	6.67382	2.90083	-1.68030
H	6.43757	2.48861	0.06364
H	5.76802	4.02158	-0.60003
C	-3.44712	1.51301	-0.57783
C	-4.22299	0.75176	0.24252
C	-3.82095	1.67701	-2.02379
C	-5.41296	0.06392	-0.32370
C	-4.94254	1.08705	-2.47846
C	-5.76920	0.22536	-1.60574
H	-6.01298	-0.56515	0.32103
H	-5.25024	1.22625	-3.50564
C	-3.94641	0.59989	1.73924
H	-3.04865	1.17146	2.05067
C	-5.10763	1.15437	2.57253
H	-5.30169	2.21239	2.29412
H	-4.84921	1.12194	3.65263
H	-6.03587	0.56546	2.41707
C	-3.66348	-0.86168	2.09512
H	-2.84690	-1.24201	1.45120
H	-4.56056	-1.49930	1.95003
H	-3.33883	-0.94297	3.15458
C	-2.97790	2.52666	-2.97275
H	-2.01514	2.79577	-2.49180
C	-3.69508	3.83996	-3.30498
H	-3.04613	4.47830	-3.94182
H	-3.92104	4.39774	-2.37156
H	-4.64652	3.64836	-3.84557
C	-2.60931	1.76760	-4.25737
H	-2.22990	0.75447	-4.01331
H	-1.81432	2.31684	-4.80570
H	-3.48403	1.66287	-4.93318
C	-7.00416	-0.46626	-2.15420
H	-7.48036	-1.08168	-1.35777
C	-6.63765	-1.42058	-3.29669
H	-5.87187	-2.14775	-2.95396
H	-6.23866	-0.86623	-4.17236
H	-7.53574	-1.98927	-3.61942
C	-8.04729	0.56033	-2.60990
H	-8.29981	1.24372	-1.77100
H	-8.97739	0.04199	-2.92682
H	-7.67157	1.16345	-3.46347
C	6.92013	-1.62849	-3.54131
H	7.01875	-2.73693	-3.50319
C	6.37547	-1.27675	-4.93046
H	5.35673	-1.70155	-5.05814
H	7.03185	-1.70784	-5.71629
H	6.32664	-0.17688	-5.07671
C	8.32437	-1.04804	-3.33784
H	8.69980	-1.30733	-2.32476
H	8.32306	0.05703	-3.44831
H	9.02434	-1.47587	-4.08697
H	-0.08949	-2.16862	-0.15702
C	-0.77627	-3.04321	2.12957
C	-2.08178	-3.83307	0.36460
C	0.32013	-2.25652	2.56978

C	-1.60348	-3.68858	3.09977
C	-2.95216	-4.50274	1.26851
C	0.58411	-2.13219	3.91435
H	0.92465	-1.75875	1.82343
C	-1.30335	-3.53987	4.47780
C	-2.70638	-4.43354	2.61630
H	-3.79619	-5.05889	0.88247
C	-0.22952	-2.77754	4.87746
H	1.42600	-1.53317	4.24562
H	-1.93742	-4.03354	5.20897
H	-3.35806	-4.94168	3.32189
H	-0.00334	-2.66465	5.93309
N	-1.03305	-3.13633	0.79012
C	-2.30634	-3.88779	-1.10290
C	-1.35806	-3.77956	-2.03513
H	-1.59902	-3.80114	-3.09050
H	-0.32103	-3.64705	-1.75350
Cl	-3.98778	-4.11638	-1.60348

Catalyst (TRIP, 3)-Quinoline complex s-trans (alternative conformation)
Electronic energy (Hartrees): -1582.914162
Free Energy at 1M concentration and 253 K (Hartrees): -1581.812246
Coordinates:

P	0.26911	-0.40421	-0.96811
O	-0.67217	0.45259	0.06893
O	1.74907	0.27572	-0.99810
O	-0.24722	-0.52327	-2.34627
O	0.43809	-1.73125	-0.12165
C	-0.67352	1.79686	-0.18785
C	-1.93487	2.45634	-0.60296
C	-1.94461	3.77114	-0.86559
H	-2.86822	4.26380	-1.14601
C	-0.70264	4.56374	-0.74872
C	-0.70886	5.99936	-1.11246
H	-1.62353	6.47121	-1.44928
C	0.41562	6.71868	-1.02874
H	0.41063	7.76741	-1.29634
C	1.66971	6.08271	-0.58122
H	2.58020	6.66610	-0.53474
C	1.68510	4.78777	-0.24452
H	2.61983	4.33628	0.06081
C	0.43691	3.98061	-0.30732
C	0.42788	2.53378	0.06171
C	2.20321	0.82366	0.17802
C	3.49846	0.34600	0.72764
C	3.99387	0.89173	1.84785
H	4.95379	0.56843	2.23310
C	3.24445	1.94327	2.56104
C	3.75561	2.46318	3.85031
H	4.68809	2.09069	4.25588
C	3.06612	3.39192	4.52118
H	3.43893	3.76956	5.46451
C	1.78814	3.89580	3.98377
H	1.22348	4.63058	4.54305
C	1.32157	3.43791	2.81632
H	0.36992	3.80421	2.45512
C	2.09182	2.42538	2.04250
C	1.59256	1.89759	0.73621
C	4.30113	-0.64979	-0.02133
C	5.03210	-0.21083	-1.26033

C	4.41411	-1.93646	0.41346
C	5.77382	-1.11046	-1.93357
C	5.28293	-2.87877	-0.33998
C	5.91705	-2.50345	-1.45924
H	6.30341	-0.81438	-2.82889
H	5.40385	-3.89006	0.02532
C	3.69357	-2.45833	1.65666
H	2.97492	-1.70929	2.04716
C	2.86641	-3.71457	1.34448
H	2.21764	-3.54036	0.46157
H	2.21821	-3.96622	2.21077
H	3.51560	-4.59103	1.13961
C	4.69656	-2.73844	2.78088
H	5.40831	-3.54044	2.49107
H	4.15935	-3.05558	3.70022
H	5.27239	-1.81859	3.01660
C	4.97985	1.23530	-1.75468
H	4.35093	1.85690	-1.08803
C	4.34907	1.32722	-3.15042
H	3.35045	0.84137	-3.15132
H	4.98682	0.83572	-3.91498
H	4.21564	2.39231	-3.43696
C	6.37119	1.88201	-1.73443
H	7.05365	1.39890	-2.46499
H	6.81334	1.79783	-0.71855
H	6.29237	2.96049	-1.98961
C	-3.19976	1.68610	-0.62258
C	-3.84951	1.39212	0.53842
C	-3.78790	1.26090	-1.93793
C	-5.13476	0.64848	0.47787
C	-4.98050	0.63579	-1.94792
C	-5.68846	0.30397	-0.69254
H	-5.63946	0.39449	1.40096
H	-5.43440	0.34891	-2.88639
C	-3.32934	1.81759	1.91300
H	-2.39552	2.40970	1.83422
C	-4.33570	2.72631	2.62943
H	-4.58802	3.59493	1.98416
H	-3.89309	3.11265	3.57248
H	-5.26931	2.18118	2.88117
C	-2.98815	0.59227	2.76698
H	-2.25524	-0.04353	2.22955
H	-3.89196	-0.01297	2.98946
H	-2.53029	0.91072	3.72776
C	-3.07251	1.53964	-3.25766
H	-2.04072	1.89829	-3.06422
C	-3.79128	2.64435	-4.03989
H	-3.22743	2.88385	-4.96666
H	-3.85531	3.56685	-3.42471
H	-4.81929	2.32964	-4.31984
C	-2.92654	0.27161	-4.11461
H	-2.55336	-0.57128	-3.49815
H	-2.20181	0.45252	-4.93691
H	-3.89484	-0.02713	-4.56837
C	-7.01648	-0.43008	-0.72734
H	-7.40885	-0.55565	0.30715
C	-6.84376	-1.83488	-1.31182
H	-6.10620	-2.39954	-0.70539
H	-6.49061	-1.79425	-2.36412
H	-7.80910	-2.38360	-1.28147

C	-8.06803	0.36728	-1.50766
H	-8.16415	1.38893	-1.08167
H	-9.05663	-0.13393	-1.43255
H	-7.79725	0.44921	-2.58145
C	6.78386	-3.49048	-2.22015
H	6.76864	-4.48084	-1.71146
C	6.24627	-3.70971	-3.63897
H	5.18649	-4.04089	-3.59703
H	6.83632	-4.49957	-4.15098
H	6.30775	-2.77832	-4.24079
C	8.24480	-3.02683	-2.25101
H	8.61502	-2.86731	-1.21564
H	8.35479	-2.08034	-2.82149
H	8.87934	-3.80254	-2.73042
H	-0.44162	-2.10723	0.23717
C	-2.61431	-3.35334	-0.17011
C	-2.11242	-3.10648	2.10019
C	-2.33563	-3.02665	-1.52544
C	-3.73906	-4.19070	0.11902
C	-3.22285	-3.91812	2.45991
C	-3.13889	-3.51794	-2.52997
H	-1.50693	-2.37445	-1.77479
C	-4.52969	-4.69794	-0.94353
C	-4.01225	-4.46098	1.47848
H	-3.42915	-4.10920	3.50366
C	-4.23753	-4.36437	-2.24532
H	-2.92251	-3.24808	-3.55885
H	-5.37604	-5.33675	-0.70679
H	-4.85538	-5.09532	1.73870
H	-4.85105	-4.74259	-3.05712
N	-1.81774	-2.84996	0.82551
C	-1.23647	-2.52443	3.15640
C	-0.00344	-2.04048	2.99960
H	0.54239	-1.63048	3.84039
H	0.47585	-2.03855	2.03117
Cl	-1.93824	-2.46671	4.78663

Catalyst (TRIP, 3)-Quinoline complex s-cis (alternative conformation)
Electronic energy (Hartrees): -1582.913239
Free Energy at 1M concentration and 253 K (Hartrees): -1581.811408
Coordinates:

P	0.27995	-0.39016	-1.14851
O	-0.79055	0.25441	-0.07270
O	1.62930	0.52558	-1.14545
O	-0.23661	-0.52803	-2.52521
O	0.68468	-1.69762	-0.36533
C	-0.92509	1.61564	-0.15410
C	-2.25486	2.19218	-0.46978
C	-2.40078	3.52195	-0.56358
H	-3.37406	3.94933	-0.77590
C	-1.24690	4.41726	-0.34382
C	-1.40528	5.87923	-0.51951
H	-2.36637	6.29252	-0.79921
C	-0.36154	6.69509	-0.33546
H	-0.47757	7.76327	-0.46572
C	0.95447	6.14092	0.03669
H	1.79917	6.80520	0.16621
C	1.10665	4.82207	0.20372
H	2.08473	4.43500	0.45750
C	-0.05074	3.90395	0.02610

C	0.09348	2.42860	0.20326
C	2.04222	0.93416	0.09900
C	3.35932	0.47614	0.61099
C	3.79164	0.89370	1.80977
H	4.76622	0.59032	2.17339
C	2.94897	1.77136	2.64486
C	3.39776	2.13990	4.00729
H	4.35438	1.79266	4.37753
C	2.62160	2.89870	4.78823
H	2.94891	3.16520	5.78489
C	1.31050	3.36474	4.29842
H	0.67719	3.95954	4.94394
C	0.89932	3.04379	3.06634
H	-0.07661	3.37626	2.73903
C	1.76385	2.22334	2.17418
C	1.32473	1.84322	0.79765
C	4.23973	-0.36404	-0.23456
C	4.99611	0.28034	-1.36272
C	4.41334	-1.69007	0.02938
C	5.83805	-0.47136	-2.09702
C	5.39291	-2.46380	-0.77766
C	6.06161	-1.90126	-1.79361
H	6.38710	-0.02669	-2.91588
H	5.56824	-3.50400	-0.53663
C	3.64811	-2.42305	1.13164
H	2.82310	-1.79482	1.52537
C	2.98831	-3.70847	0.60575
H	2.47174	-3.51805	-0.35754
H	2.23859	-4.07671	1.33720
H	3.73538	-4.51427	0.44785
C	4.57374	-2.73934	2.31088
H	5.39830	-3.41547	1.99943
H	3.99893	-3.23001	3.12519
H	5.01417	-1.80374	2.71427
C	4.84805	1.76805	-1.68226
H	4.14229	2.25310	-0.98012
C	4.27040	1.98183	-3.08741
H	3.31267	1.42934	-3.19375
H	4.97489	1.63203	-3.87114
H	4.06745	3.06132	-3.25422
C	6.17989	2.51105	-1.51328
H	6.92899	2.17617	-2.26129
H	6.58524	2.33493	-0.49384
H	6.02367	3.60373	-1.63998
C	-3.44573	1.31747	-0.57431
C	-4.09666	0.89621	0.54561
C	-4.00698	0.99865	-1.92907
C	-5.39466	0.18593	0.40136
C	-5.20334	0.38615	-2.01344
C	-5.94550	-0.02583	-0.80183
H	-5.92481	-0.12623	1.29175
H	-5.63776	0.17609	-2.98116
C	-3.58140	1.17163	1.95893
H	-2.60832	1.70176	1.94549
C	-4.54650	2.08011	2.72903
H	-4.71388	3.02089	2.16196
H	-4.11255	2.34384	3.71724
H	-5.52460	1.58263	2.89743
C	-3.33612	-0.13936	2.71443
H	-2.66396	-0.79234	2.12124

H	-4.28475	-0.68320	2.90576
H	-2.84668	0.06832	3.68990
C	-3.25251	1.36323	-3.20427
H	-2.23497	1.72841	-2.95428
C	-3.96253	2.49738	-3.95169
H	-3.36728	2.79963	-4.83965
H	-4.06885	3.38178	-3.28795
H	-4.97183	2.18127	-4.29134
C	-3.05278	0.14455	-4.11847
H	-2.65206	-0.71132	-3.53689
H	-2.32766	0.39057	-4.92336
H	-4.00587	-0.16726	-4.59494
C	-7.31700	-0.66731	-0.91671
H	-7.73663	-0.85183	0.09807
C	-7.22938	-2.02665	-1.61549
H	-6.57705	-2.70309	-1.02698
H	-6.81950	-1.92791	-2.64296
H	-8.23670	-2.49087	-1.67748
C	-8.29945	0.26158	-1.63998
H	-8.33421	1.24901	-1.13174
H	-9.32078	-0.17503	-1.61831
H	-8.00498	0.41259	-2.70002
C	7.04517	-2.71757	-2.61261
H	7.07847	-3.76424	-2.23420
C	6.60882	-2.78785	-4.08050
H	5.57746	-3.19509	-4.15145
H	7.28714	-3.46135	-4.64661
H	6.63201	-1.78429	-4.55562
C	8.46458	-2.15212	-2.48708
H	8.76117	-2.10342	-1.41742
H	8.53230	-1.13383	-2.92517
H	9.18449	-2.81194	-3.01677
H	-0.05939	-2.10895	0.18831
C	-2.29746	-3.38534	0.22225
C	-1.15335	-3.30076	2.25090
C	-2.40682	-2.97281	-1.13351
C	-3.27314	-4.28498	0.76038
C	-2.08891	-4.17744	2.86271
C	-3.43761	-3.44976	-1.91112
H	-1.67858	-2.29323	-1.56123
C	-4.31884	-4.75743	-0.07365
C	-3.13435	-4.66415	2.11659
H	-1.97427	-4.43172	3.90955
C	-4.39249	-4.35443	-1.38663
H	-3.51558	-3.12953	-2.94519
H	-5.05196	-5.44395	0.34067
H	-3.86587	-5.33405	2.56071
H	-5.18993	-4.72211	-2.02513
N	-1.26096	-2.92273	0.98545
C	-0.00953	-2.79652	3.06142
C	0.75321	-3.57470	3.83061
H	1.56597	-3.17282	4.42304
H	0.58693	-4.64644	3.85173
Cl	0.28127	-1.06189	3.00329

Catalyst (TRIP, 3)-Quinoline complex s-trans (alternative conformation)
Electronic energy (Hartrees): -1582.913012
Free Energy at 1M concentration and 253 K (Hartrees): -1581.810822
Coordinates:

P	0.09289	-0.22751	-0.60105

O	1.18993	0.89216	-1.05954
O	-1.05008	0.56750	0.26224
O	0.77955	-1.05521	0.55565
O	-0.37359	-0.97285	-1.78642
C	1.31749	2.01283	-0.27705
C	2.64102	2.29332	0.33405
C	2.86793	3.47332	0.92721
H	3.83397	3.68034	1.37258
C	1.77894	4.45653	1.07641
C	2.02373	5.70609	1.83342
H	3.01517	5.93058	2.20689
C	1.02128	6.55964	2.06457
H	1.20064	7.47110	2.62008
C	-0.33729	6.25100	1.58182
H	-1.15134	6.92992	1.80095
C	-0.56787	5.12791	0.89227
H	-1.58040	4.90982	0.58238
C	0.54596	4.18568	0.58832
C	0.32227	2.93213	-0.19956
C	-1.66677	1.53434	-0.48726
C	-3.09815	1.37611	-0.83510
C	-3.69752	2.28666	-1.61615
H	-4.75221	2.19585	-1.84788
C	-2.93028	3.43426	-2.14625
C	-3.57756	4.38385	-3.08033
H	-4.61397	4.24744	-3.36311
C	-2.88411	5.41239	-3.58073
H	-3.35742	6.10486	-4.26480
C	-1.46804	5.60086	-3.21044
H	-0.90766	6.42420	-3.63426
C	-0.87515	4.75218	-2.36261
H	0.16917	4.89625	-2.11876
C	-1.64062	3.61984	-1.77609
C	-1.00219	2.65945	-0.82764
C	-3.88996	0.29387	-0.20450
C	-4.29637	0.43819	1.23598
C	-4.25487	-0.81591	-0.90425
C	-5.02800	-0.53607	1.80868
C	-5.10059	-1.84254	-0.23926
C	-5.46418	-1.72462	1.04548
H	-5.32666	-0.45091	2.84453
H	-5.43466	-2.69904	-0.80999
C	-3.84039	-1.05149	-2.35571
H	-3.09214	-0.30103	-2.68513
C	-3.17028	-2.42234	-2.53477
H	-2.39147	-2.57660	-1.75973
H	-2.68576	-2.47894	-3.53286
H	-3.90705	-3.24944	-2.46251
C	-5.04897	-0.91332	-3.28722
H	-5.81843	-1.68160	-3.06010
H	-4.73037	-1.03130	-4.34495
H	-5.50451	0.09319	-3.17263
C	-3.90621	1.66464	2.06156
H	-3.34358	2.39230	1.44489
C	-2.98154	1.27919	3.22355
H	-2.09511	0.73059	2.83962
H	-3.50764	0.63728	3.96124
H	-2.62383	2.19335	3.74378
C	-5.14335	2.41987	2.56547
H	-5.71676	1.81652	3.30015

H	-5.80677	2.67326	1.71095
H	-4.83490	3.36720	3.05729
C	3.67779	1.23362	0.39308
C	4.42689	0.91470	-0.69854
C	3.94674	0.55378	1.70909
C	5.52532	-0.07775	-0.55471
C	4.98211	-0.30081	1.80308
C	5.80598	-0.64667	0.62609
H	6.12691	-0.32482	-1.41971
H	5.21678	-0.76549	2.75074
C	4.22149	1.56485	-2.06598
H	3.37578	2.28124	-2.05403
C	5.45553	2.37680	-2.47662
H	5.69020	3.13046	-1.69460
H	5.25666	2.91506	-3.42800
H	6.34089	1.72297	-2.62267
C	3.87870	0.51102	-3.12780
H	3.01225	-0.10026	-2.79709
H	4.73747	-0.16591	-3.31940
H	3.60707	1.00950	-4.08270
C	3.08093	0.82042	2.94100
H	2.19486	1.42712	2.66457
C	3.86021	1.61816	3.99343
H	3.19636	1.87219	4.84724
H	4.23701	2.56585	3.55481
H	4.72423	1.03498	4.37717
C	2.52313	-0.47686	3.54799
H	2.05192	-1.10185	2.76321
H	1.75061	-0.23580	4.30924
H	3.32021	-1.07100	4.04210
C	6.96159	-1.62297	0.75627
H	7.42564	-1.79664	-0.24085
C	6.48460	-2.98723	1.27051
H	5.67269	-3.37955	0.62469
H	6.10874	-2.91743	2.31292
H	7.32416	-3.71436	1.24923
C	8.05309	-1.04866	1.66601
H	8.39599	-0.06677	1.27499
H	8.92621	-1.73504	1.69331
H	7.67866	-0.91073	2.70271
C	-6.34145	-2.77838	1.69788
H	-6.53843	-3.60606	0.97962
C	-5.65003	-3.40092	2.91702
H	-4.66248	-3.81524	2.62746
H	-6.26752	-4.23208	3.31946
H	-5.50158	-2.65239	3.72353
C	-7.70147	-2.18662	2.08459
H	-8.19214	-1.74548	1.19061
H	-7.58992	-1.39763	2.85848
H	-8.36339	-2.98381	2.48530
H	0.73075	-2.06296	0.34834
C	1.86451	-3.94727	-0.77680
C	-0.27479	-4.43938	0.02689
C	2.92568	-3.01077	-0.91296
C	1.97148	-5.22038	-1.41794
C	-0.23582	-5.72676	-0.57386
C	4.02993	-3.33411	-1.66782
H	2.83880	-2.04273	-0.43304
C	3.13496	-5.52684	-2.16864
C	0.87879	-6.10839	-1.27768

H	-1.08395	-6.39017	-0.47223
C	4.14312	-4.59939	-2.29410
H	4.83001	-2.61120	-1.78756
H	3.20886	-6.49862	-2.64883
H	0.92576	-7.08959	-1.74238
H	5.02854	-4.83106	-2.87778
N	0.75233	-3.60028	-0.06333
C	-1.46846	-3.97781	0.78585
C	-1.48624	-3.01562	1.71065
H	-0.57899	-2.49415	1.98626
H	-2.40811	-2.71778	2.19403
Cl	-2.98505	-4.80071	0.38920

Catalyst (TRIP, 3)-Quinoline complex s-trans (alternative conformation)
Electronic energy (Hartrees): -1582.913531
Free Energy at 1M concentration and 253 K (Hartrees): -1581.810523
Coordinates:

P	-0.00200	0.06525	-0.74378
O	-1.02196	-1.17714	-1.03629
O	1.20717	-0.52749	0.19171
O	-0.72843	0.95081	0.34392
O	0.39664	0.70305	-2.01174
C	-1.19015	-2.07390	-0.01047
C	-2.54068	-2.22311	0.59246
C	-2.76701	-3.19414	1.48828
H	-3.75538	-3.32231	1.91309
C	-1.66442	-4.05687	1.95281
C	-1.91426	-5.04834	3.02454
H	-2.91327	-5.18076	3.42110
C	-0.90705	-5.78051	3.51118
H	-1.09007	-6.50289	4.29615
C	0.46068	-5.59223	2.99285
H	1.27687	-6.16644	3.41206
C	0.69577	-4.70371	2.02058
H	1.71276	-4.56205	1.68148
C	-0.42138	-3.90782	1.43956
C	-0.19080	-2.91954	0.34036
C	1.79672	-1.64899	-0.32984
C	3.22071	-1.58966	-0.73975
C	3.82320	-2.68943	-1.21597
H	4.87185	-2.66217	-1.48709
C	3.06228	-3.94556	-1.37922
C	3.71177	-5.12355	-1.99894
H	4.74845	-5.07324	-2.30799
C	3.01997	-6.25344	-2.18225
H	3.49486	-7.11330	-2.63676
C	1.60319	-6.32797	-1.77661
H	1.04351	-7.23861	-1.94699
C	1.00850	-5.27123	-1.21079
H	-0.03685	-5.33982	-0.94012
C	1.77266	-4.01697	-0.97336
C	1.13494	-2.82685	-0.33499
C	3.99429	-0.34096	-0.53622
C	4.37884	0.06047	0.86182
C	4.35463	0.44433	-1.59023
C	5.05492	1.21006	1.04820
C	5.15604	1.67019	-1.33611
C	5.47467	2.05261	-0.09208
H	5.33288	1.51936	2.04660
H	5.48142	2.26933	-2.17637

C	3.96903	0.12330	-3.03448
H	3.23679	-0.70918	-3.07847
C	3.28531	1.31793	-3.71704
H	2.49797	1.74175	-3.06083
H	2.81072	0.98973	-4.66647
H	4.01250	2.12166	-3.95702
C	5.20040	-0.32027	-3.83173
H	5.95307	0.49468	-3.88968
H	4.90405	-0.60451	-4.86403
H	5.66951	-1.20412	-3.35050
C	4.05568	-0.82123	2.06866
H	3.51511	-1.73518	1.75372
C	3.13916	-0.09862	3.06520
H	2.22579	0.26619	2.55131
H	3.65472	0.76465	3.53568
H	2.82572	-0.79908	3.86848
C	5.33638	-1.31224	2.75630
H	5.90123	-0.47035	3.20923
H	5.98853	-1.82784	2.01911
H	5.08243	-2.03611	3.56003
C	-3.63731	-1.28826	0.23500
C	-4.29559	-1.39913	-0.95276
C	-4.08966	-0.26880	1.24725
C	-5.46607	-0.51934	-1.21503
C	-5.18815	0.46528	0.98901
C	-5.91060	0.34832	-0.29529
H	-5.98792	-0.60636	-2.15909
H	-5.55112	1.17346	1.72096
C	-3.92428	-2.43324	-2.01587
H	-3.04679	-3.03727	-1.71100
C	-5.06685	-3.43337	-2.22943
H	-5.33639	-3.91202	-1.26355
H	-4.74700	-4.23084	-2.93382
H	-5.96744	-2.93723	-2.64824
C	-3.53767	-1.75061	-3.33425
H	-2.73474	-1.00367	-3.15779
H	-4.40723	-1.23670	-3.79479
H	-3.15634	-2.50561	-4.05452
C	-3.34374	-0.06311	2.56586
H	-2.37851	-0.60966	2.54553
C	-4.15541	-0.61934	3.74206
H	-3.57269	-0.52870	4.68355
H	-4.38412	-1.69291	3.57996
H	-5.11202	-0.06726	3.86157
C	-2.98774	1.41333	2.81135
H	-2.53892	1.86575	1.90572
H	-2.25174	1.49138	3.63989
H	-3.88358	2.00695	3.09026
C	-7.14338	1.19333	-0.56511
H	-7.54786	0.96351	-1.57667
C	-6.80490	2.68881	-0.54961
H	-6.03333	2.91255	-1.31507
H	-6.42900	3.00793	0.44532
H	-7.70972	3.28590	-0.79221
C	-8.25382	0.87266	0.44207
H	-8.48169	-0.21469	0.42382
H	-9.18012	1.42504	0.17550
H	-7.95674	1.16021	1.47287
C	6.27383	3.32228	0.14043
H	6.50706	3.80851	-0.83371

C	5.46589	4.33374	0.96039
H	4.50011	4.54652	0.45403
H	6.02767	5.28795	1.04881
H	5.26121	3.94992	1.98213
C	7.61445	3.01161	0.81607
H	8.18147	2.27049	0.21299
H	7.46544	2.60422	1.83839
H	8.22479	3.93678	0.89177
H	-0.75847	1.95799	0.13245
C	0.18694	4.10262	0.86451
C	-1.66912	4.33246	-0.54254
C	1.11549	3.25059	1.52368
C	0.32247	5.51870	1.00530
C	-1.60096	5.74917	-0.43960
C	2.12604	3.79399	2.28287
H	1.01581	2.17796	1.41502
C	1.37545	6.04351	1.79786
C	-0.61994	6.32655	0.32715
H	-2.32304	6.35827	-0.96559
C	2.26015	5.19718	2.42504
H	2.83520	3.13747	2.77711
H	1.47041	7.12132	1.89639
H	-0.55808	7.40788	0.41575
H	3.06839	5.60084	3.02694
N	-0.80376	3.55142	0.09963
C	-2.71385	3.67138	-1.37214
C	-3.08764	2.39170	-1.31111
H	-2.64631	1.70791	-0.59981
H	-3.83627	1.99974	-1.98767
Cl	-3.51791	4.71189	-2.56151

Catalyst (TRIP, 3)-Quinoline complex s-cis (alternative conformation)
Electronic energy (Hartrees): -1582.911341
Free Energy at 1M concentration and 253 K (Hartrees): -1581.8097
Coordinates:

P	-0.03163	-0.06733	-1.36456
O	-1.06769	0.22313	-0.11100
O	1.21947	0.97653	-1.21360
O	-0.60279	0.04961	-2.71535
O	0.54071	-1.46077	-0.88368
C	-1.38653	1.54631	0.04397
C	-2.79480	1.97773	-0.13496
C	-3.12569	3.26539	0.03981
H	-4.15623	3.58254	-0.06878
C	-2.09148	4.26044	0.39056
C	-2.45541	5.69178	0.50142
H	-3.47994	6.00621	0.34574
C	-1.51994	6.60245	0.79204
H	-1.78638	7.64850	0.87138
C	-0.11899	6.18574	0.99471
H	0.63651	6.93039	1.20987
C	0.21934	4.89446	0.90232
H	1.25579	4.61309	1.03398
C	-0.81455	3.86628	0.60632
C	-0.46356	2.41851	0.50263
C	1.64145	1.21364	0.07202
C	3.02937	0.84362	0.45237
C	3.48958	1.14246	1.67553
H	4.51295	0.90954	1.94444
C	2.60880	1.78054	2.67133

C	3.10212	2.00296	4.05001
H	4.11912	1.73996	4.31301
C	2.29116	2.52075	4.97839
H	2.65058	2.67814	5.98703
C	0.89834	2.86372	4.63479
H	0.23908	3.25737	5.39760
C	0.44643	2.67620	3.38953
H	-0.58665	2.90915	3.16973
C	1.34608	2.13085	2.33577
C	0.86684	1.91092	0.93717
C	3.93864	0.22070	-0.53856
C	4.56621	1.08569	-1.59570
C	4.26227	-1.10181	-0.46213
C	5.44743	0.53424	-2.45140
C	5.28035	-1.64956	-1.39663
C	5.83676	-0.88862	-2.34923
H	5.90679	1.13987	-3.22076
H	5.58020	-2.68467	-1.30114
C	3.63278	-2.05068	0.55914
H	2.75750	-1.57638	1.04657
C	3.10429	-3.33422	-0.10211
H	2.49902	-3.09227	-0.99950
H	2.46436	-3.89260	0.61261
H	3.93266	-4.00642	-0.40816
C	4.63337	-2.39494	1.66691
H	5.51420	-2.93115	1.25374
H	4.14767	-3.03970	2.43074
H	4.98378	-1.47002	2.16995
C	4.24564	2.57616	-1.70818
H	3.52190	2.88285	-0.92814
C	3.58942	2.90598	-3.05530
H	2.69454	2.26643	-3.21011
H	4.29440	2.74590	-3.89807
H	3.26247	3.96769	-3.06832
C	5.49621	3.43472	-1.47768
H	6.24524	3.28466	-2.28347
H	5.95982	3.17437	-0.50202
H	5.21908	4.51037	-1.45349
C	-3.84583	0.97585	-0.42952
C	-4.32019	0.15694	0.55100
C	-4.42300	0.89646	-1.81490
C	-5.39561	-0.81493	0.22256
C	-5.42948	0.03460	-2.05438
C	-5.93104	-0.87749	-1.00404
H	-5.75703	-1.48027	0.99588
H	-5.87883	-0.01484	-3.03664
C	-3.81516	0.20722	1.99413
H	-3.01665	0.96487	2.12398
C	-4.93868	0.61516	2.95401
H	-5.37042	1.58923	2.63878
H	-4.53562	0.73126	3.98287
H	-5.74733	-0.14524	2.97818
C	-3.20151	-1.13567	2.40607
H	-2.44340	-1.44538	1.65907
H	-3.97296	-1.93047	2.47937
H	-2.70017	-1.03957	3.39290
C	-3.90611	1.78240	-2.94653
H	-2.98148	2.30709	-2.63040
C	-4.93424	2.86227	-3.30220
H	-4.52106	3.54104	-4.07857

H	-5.17605	3.46923	-2.40415
H	-5.87149	2.40823	-3.68895
C	-3.52244	0.96475	-4.19034
H	-2.90014	0.09221	-3.90368
H	-2.93793	1.59691	-4.89249
H	-4.42129	0.59481	-4.72707
C	-7.02701	-1.88090	-1.31396
H	-7.26772	-2.47459	-0.40324
C	-6.56910	-2.87578	-2.38671
H	-5.63452	-3.38138	-2.06220
H	-6.38323	-2.36540	-3.35543
H	-7.34757	-3.65310	-2.54128
C	-8.31771	-1.17029	-1.73704
H	-8.62944	-0.44875	-0.95173
H	-9.13375	-1.91242	-1.86949
H	-8.18109	-0.62322	-2.69377
C	6.86395	-1.47566	-3.30051
H	7.02705	-2.55207	-3.06703
C	6.37100	-1.40362	-4.75033
H	5.38731	-1.91185	-4.84316
H	7.09129	-1.91713	-5.42242
H	6.26434	-0.35074	-5.08721
C	8.21849	-0.77474	-3.14475
H	8.55694	-0.83208	-2.08802
H	8.15598	0.29287	-3.44428
H	8.98020	-1.27470	-3.78039
H	-0.01591	-1.96003	-0.21633
C	0.19470	-2.90493	2.21719
C	-0.70544	-4.33574	0.61987
C	0.42864	-1.57443	2.65221
C	0.59803	-3.99605	3.04698
C	-0.36822	-5.47776	1.40017
C	1.05721	-1.34769	3.85517
H	0.07825	-0.76339	2.02354
C	1.25661	-3.72621	4.27338
C	0.29442	-5.30288	2.58938
H	-0.65245	-6.46552	1.05798
C	1.48569	-2.42704	4.66626
H	1.22091	-0.33189	4.19841
H	1.56984	-4.55874	4.89723
H	0.56966	-6.15928	3.19912
H	1.98781	-2.22312	5.60678
N	-0.41986	-3.10552	1.01570
C	-1.40086	-4.50594	-0.68229
C	-1.20614	-5.53766	-1.50785
H	-1.73968	-5.62292	-2.44620
H	-0.47698	-6.30326	-1.26683
Cl	-2.53914	-3.23856	-1.11044

Catalyst (TRIP, 3)-Quinoline complex s-cis (alternative conformation)
Electronic energy (Hartrees): -1582.911368
Free Energy at 1M concentration and 253 K (Hartrees): -1581.809644
Coordinates:

P	-0.05349	-0.06800	-1.34553
O	-1.08132	0.21868	-0.08507
O	1.19726	0.97685	-1.20025
O	-0.63422	0.05020	-2.69205
O	0.52528	-1.46172	-0.87185
C	-1.39639	1.54177	0.07886
C	-2.80374	1.97807	-0.09497

C	-3.13227	3.26408	0.09565
H	-4.16211	3.58447	-0.00943
C	-2.09557	4.25381	0.45370
C	-2.45689	5.68458	0.57939
H	-3.48168	6.00185	0.43139
C	-1.51875	6.59127	0.87368
H	-1.78331	7.63690	0.96383
C	-0.11741	6.17085	1.06536
H	0.64020	6.91249	1.28342
C	0.21868	4.88005	0.95922
H	1.25556	4.59643	1.08235
C	-0.81818	3.85585	0.65939
C	-0.46993	2.40850	0.54076
C	1.63122	1.20738	0.08274
C	3.02476	0.84162	0.44630
C	3.49583	1.13218	1.66735
H	4.52309	0.90225	1.92393
C	2.62244	1.75869	2.67676
C	3.12858	1.97288	4.05213
H	4.14905	1.71157	4.30320
C	2.32497	2.48104	4.99216
H	2.69376	2.63245	5.99834
C	0.92765	2.82159	4.66468
H	0.27455	3.20759	5.43665
C	0.46417	2.64137	3.42258
H	-0.57172	2.87249	3.21430
C	1.35528	2.10681	2.35623
C	0.86323	1.89613	0.96057
C	3.92840	0.23561	-0.55971
C	4.54239	1.11685	-1.61131
C	4.26044	-1.08556	-0.50119
C	5.42173	0.58135	-2.47890
C	5.27562	-1.61584	-1.44878
C	5.82147	-0.83989	-2.39547
H	5.87143	1.19882	-3.24455
H	5.58260	-2.65008	-1.36735
C	3.64325	-2.05004	0.51287
H	2.77037	-1.58576	1.01409
C	3.11380	-3.32632	-0.16125
H	2.49888	-3.07397	-1.04926
H	2.48282	-3.89728	0.55147
H	3.94202	-3.99047	-0.48477
C	4.65432	-2.40486	1.60768
H	5.53391	-2.93189	1.18031
H	4.17758	-3.06170	2.36682
H	5.00490	-1.48504	2.12001
C	4.20913	2.60585	-1.70581
H	3.48926	2.89876	-0.91696
C	3.53931	2.94406	-3.04410
H	2.64808	2.29917	-3.19796
H	4.23854	2.79787	-3.89415
H	3.20407	4.00326	-3.04372
C	5.45445	3.47229	-1.47645
H	6.19821	3.33667	-2.28962
H	5.92791	3.20594	-0.50717
H	5.16871	4.54531	-1.43912
C	-3.85506	0.98288	-0.41016
C	-4.32772	0.14175	0.55222
C	-4.43183	0.93254	-1.79731
C	-5.39868	-0.82727	0.20149

C	-5.43470	0.07223	-2.05625
C	-5.93265	-0.86528	-1.02680
H	-5.75764	-1.51125	0.95954
H	-5.88387	0.04320	-3.03939
C	-3.82611	0.16478	1.99732
H	-3.03317	0.92506	2.14494
C	-4.95443	0.54532	2.96287
H	-5.39251	1.52246	2.66643
H	-4.55426	0.64359	3.99471
H	-5.75766	-0.22114	2.97012
C	-3.20449	-1.18235	2.38296
H	-2.43829	-1.46916	1.63512
H	-3.97005	-1.98463	2.43260
H	-2.71087	-1.10476	3.37528
C	-3.91967	1.84687	-2.90865
H	-2.99658	2.36745	-2.58157
C	-4.95238	2.93085	-3.23776
H	-4.54254	3.62950	-3.99811
H	-5.19603	3.51546	-2.32555
H	-5.88812	2.48237	-3.63450
C	-3.53442	1.06076	-4.17216
H	-2.90817	0.18428	-3.90751
H	-2.95347	1.71197	-4.85972
H	-4.43246	0.70016	-4.71652
C	-7.02331	-1.86689	-1.36018
H	-7.26220	-2.48169	-0.46310
C	-6.55909	-2.83552	-2.45406
H	-5.62232	-3.34342	-2.13961
H	-6.37461	-2.30290	-3.41102
H	-7.33343	-3.61313	-2.62682
C	-8.31694	-1.15339	-1.76932
H	-8.63298	-0.45055	-0.96893
H	-9.12924	-1.89634	-1.91890
H	-8.18190	-0.58501	-2.71378
C	6.84748	-1.40842	-3.35913
H	7.01943	-2.48631	-3.13920
C	6.34609	-1.32278	-4.80530
H	5.36550	-1.83681	-4.89879
H	7.06627	-1.82318	-5.48732
H	6.23018	-0.26679	-5.12913
C	8.19786	-0.69970	-3.20234
H	8.54240	-0.76696	-2.14817
H	8.12614	0.37089	-3.48893
H	8.95969	-1.18669	-3.84783
H	-0.03097	-1.97224	-0.21390
C	0.22893	-2.94199	2.20235
C	-0.69158	-4.36956	0.61381
C	0.45555	-1.61260	2.64460
C	0.66828	-4.03525	3.01078
C	-0.32127	-5.51422	1.37556
C	1.11242	-1.38896	3.83303
H	0.07885	-0.80012	2.03312
C	1.35595	-3.76830	4.22163
C	0.36940	-5.34167	2.54885
H	-0.60195	-6.50263	1.03245
C	1.57773	-2.46991	4.62124
H	1.27068	-0.37448	4.18252
H	1.69756	-4.60234	4.82836
H	0.67091	-6.19996	3.14332
H	2.10259	-2.26807	5.54972

N	-0.41229	-3.13939	1.01427
C	-1.41479	-4.53766	-0.67335
C	-1.24182	-5.57006	-1.50310
H	-1.79616	-5.65311	-2.42949
H	-0.51084	-6.33887	-1.27857
Cl	-2.55685	-3.26633	-1.07934

Catalyst (TRIP, 3)-Quinoline complex s-cis (alternative conformation)
Electronic energy (Hartrees): -1582.909702
Free Energy at 1M concentration and 253 K (Hartrees): -1581.808836
Coordinates:

P	0.23653	-0.52256	-0.81705
O	-0.70968	0.44336	0.11752
O	1.71385	0.15473	-0.93451
O	-0.27996	-0.79496	-2.17355
O	0.43087	-1.74086	0.17331
C	-0.68548	1.75992	-0.25815
C	-1.93283	2.40077	-0.73967
C	-1.91629	3.68304	-1.13075
H	-2.83051	4.16453	-1.45774
C	-0.66089	4.46123	-1.07950
C	-0.63825	5.85501	-1.57930
H	-1.54087	6.30678	-1.97159
C	0.49741	6.56095	-1.55035
H	0.51347	7.57895	-1.91753
C	1.73505	5.95123	-1.02685
H	2.65480	6.52184	-1.02439
C	1.72421	4.69487	-0.56668
H	2.64746	4.26049	-0.20648
C	0.46318	3.90527	-0.56870
C	0.42580	2.50003	-0.06583
C	2.18303	0.79218	0.18828
C	3.47897	0.35345	0.76484
C	3.97127	0.96669	1.85081
H	4.93320	0.67211	2.25399
C	3.22606	2.07103	2.48606
C	3.73796	2.68041	3.73511
H	4.66317	2.32725	4.17334
C	3.05850	3.66752	4.32874
H	3.43207	4.11100	5.24265
C	1.79139	4.14814	3.74561
H	1.23570	4.93291	4.24250
C	1.32418	3.60930	2.61354
H	0.38156	3.96228	2.21684
C	2.08251	2.52857	1.92586
C	1.58054	1.90815	0.66282
C	4.29863	-0.65784	0.05783
C	5.05864	-0.24352	-1.17174
C	4.39999	-1.93458	0.52084
C	5.82595	-1.15289	-1.80176
C	5.30406	-2.88545	-0.17880
C	5.96988	-2.53135	-1.28661
H	6.37332	-0.87590	-2.69247
H	5.42475	-3.88537	0.21645
C	3.62805	-2.43598	1.74055
H	2.87443	-1.69093	2.07029
C	2.84356	-3.71844	1.42262
H	2.24206	-3.58541	0.49955
H	2.15414	-3.95901	2.25881
H	3.51961	-4.58723	1.28236

C	4.57859	-2.66251	2.92057
H	5.32432	-3.45186	2.68660
H	4.00324	-2.97181	3.81927
H	5.11778	-1.72195	3.16151
C	4.99467	1.18573	-1.71182
H	4.35066	1.82030	-1.07228
C	4.37639	1.22411	-3.11525
H	3.38003	0.73324	-3.10608
H	5.02351	0.70822	-3.85554
H	4.23983	2.27740	-3.44125
C	6.37708	1.85148	-1.69825
H	7.07235	1.35774	-2.40928
H	6.81133	1.80213	-0.67669
H	6.28592	2.92115	-1.98454
C	-3.21626	1.66705	-0.65968
C	-3.86885	1.54739	0.53002
C	-3.81687	1.08202	-1.90559
C	-5.16389	0.81960	0.57307
C	-5.01800	0.47765	-1.82926
C	-5.72502	0.32547	-0.53887
H	-5.66892	0.70034	1.52285
H	-5.48147	0.07793	-2.72056
C	-3.33949	2.15395	1.83129
H	-2.40126	2.72197	1.66965
C	-4.33573	3.16099	2.41912
H	-4.58556	3.93359	1.66069
H	-3.88537	3.67116	3.29743
H	-5.27165	2.66323	2.74843
C	-3.00375	1.05574	2.84395
H	-2.26938	0.35050	2.40149
H	-3.91134	0.49015	3.14231
H	-2.54916	1.50084	3.75467
C	-3.10812	1.18388	-3.25378
H	-2.06953	1.54727	-3.11227
C	-3.81628	2.19577	-4.16104
H	-3.25785	2.30909	-5.11471
H	-3.85870	3.18811	-3.66369
H	-4.85213	1.86696	-4.39167
C	-2.98873	-0.18216	-3.94857
H	-2.62548	-0.94807	-3.23386
H	-2.26640	-0.11663	-4.79002
H	-3.96473	-0.51814	-4.35759
C	-7.06337	-0.38779	-0.47207
H	-7.45197	-0.37027	0.57129
C	-6.91494	-1.86007	-0.86714
H	-6.18944	-2.35467	-0.18923
H	-6.55974	-1.96388	-1.91440
H	-7.89008	-2.38293	-0.76903
C	-8.10765	0.31570	-1.34673
H	-8.18646	1.38592	-1.05879
H	-9.10306	-0.15605	-1.20246
H	-7.84178	0.25123	-2.42293
C	6.86961	-3.52795	-1.99501
H	6.85120	-4.50423	-1.45990
C	6.37555	-3.79228	-3.42196
H	5.31885	-4.13511	-3.40101
H	6.98899	-4.58856	-3.89505
H	6.44361	-2.87697	-4.04727
C	8.32534	-3.04731	-1.99707
H	8.66377	-2.85533	-0.95635

H	8.44059	-2.11526	-2.58986
H	8.98230	-3.82779	-2.43691
H	-0.41982	-2.10557	0.56018
C	-2.68289	-3.42118	-0.00492
C	-2.48710	-2.95875	2.26798
C	-2.17278	-3.30018	-1.32447
C	-3.92119	-4.11320	0.19277
C	-3.70743	-3.63964	2.54541
C	-2.86781	-3.83803	-2.38498
H	-1.25572	-2.75513	-1.50567
C	-4.60148	-4.66857	-0.92012
C	-4.41112	-4.20766	1.51638
H	-4.04891	-3.73119	3.56866
C	-4.08615	-4.52958	-2.18813
H	-2.47244	-3.72025	-3.38895
H	-5.53913	-5.19156	-0.75284
H	-5.33857	-4.74112	1.70697
H	-4.61398	-4.94582	-3.04067
N	-1.98718	-2.86928	1.04187
C	-1.77006	-2.33055	3.41002
C	-2.37578	-1.84271	4.49744
H	-1.81132	-1.40287	5.30991
H	-3.45673	-1.84329	4.57622
Cl	-0.02156	-2.23858	3.29790

Catalyst (TRIP, 3)-Quinoline complex s-cis (alternative conformation)
Electronic energy (Hartrees): -1582.909872
Free Energy at 1M concentration and 253 K (Hartrees): -1581.807967
Coordinates:

P	-0.13580	-0.30746	-0.37079
O	0.87164	0.89975	-0.84599
O	-1.39176	0.39728	0.40038
O	0.55705	-0.98874	0.87382
O	-0.45658	-1.15437	-1.53599
C	1.00661	1.94903	0.02983
C	2.35077	2.24290	0.59270
C	2.52836	3.32124	1.36916
H	3.51106	3.55245	1.76279
C	1.38695	4.18599	1.72291
C	1.58521	5.31192	2.66472
H	2.57198	5.52689	3.05568
C	0.54625	6.06523	3.03996
H	0.69152	6.88637	3.72994
C	-0.80464	5.76898	2.52738
H	-1.64591	6.36533	2.85629
C	-0.99308	4.75804	1.67143
H	-1.99755	4.54280	1.33300
C	0.15904	3.93174	1.21547
C	-0.02115	2.80341	0.25097
C	-1.96630	1.41972	-0.30834
C	-3.36734	1.27636	-0.77617
C	-3.96258	2.29214	-1.41912
H	-4.99544	2.20649	-1.73490
C	-3.21468	3.53227	-1.71020
C	-3.85276	4.60581	-2.50637
H	-4.87356	4.49449	-2.85032
C	-3.17014	5.71601	-2.80660
H	-3.63653	6.49932	-3.38999
C	-1.77436	5.87153	-2.35423
H	-1.22079	6.76314	-2.61898

C	-1.19044	4.90998	-1.62973
H	-0.15913	5.03474	-1.32737
C	-1.94619	3.68190	-1.26230
C	-1.32132	2.59839	-0.44575
C	-4.13082	0.04514	-0.45989
C	-4.59132	-0.18652	0.95328
C	-4.42542	-0.86896	-1.42734
C	-5.27551	-1.30996	1.24083
C	-5.23062	-2.06593	-1.06845
C	-5.61993	-2.29452	0.19308
H	-5.60913	-1.49688	2.25247
H	-5.49886	-2.77148	-1.84380
C	-3.96166	-0.72221	-2.87698
H	-3.23656	0.11089	-2.98356
C	-3.22805	-1.98005	-3.36723
H	-2.47409	-2.30557	-2.62190
H	-2.70459	-1.76374	-4.32304
H	-3.93205	-2.81996	-3.54425
C	-5.15015	-0.40194	-3.79010
H	-5.88952	-1.23099	-3.78828
H	-4.79832	-0.24182	-4.83166
H	-5.65557	0.52595	-3.44905
C	-4.32736	0.83197	2.06261
H	-3.76892	1.70426	1.67059
C	-3.46359	0.23201	3.18081
H	-2.53170	-0.19865	2.75757
H	-4.00826	-0.56596	3.72794
H	-3.18008	1.02369	3.90683
C	-5.63936	1.39645	2.62309
H	-6.22936	0.61174	3.14178
H	-6.25169	1.82125	1.79896
H	-5.42357	2.20945	3.34897
C	3.50647	1.36246	0.29288
C	4.13858	1.42279	-0.91247
C	4.04841	0.47043	1.37639
C	5.36995	0.61587	-1.12305
C	5.19488	-0.20032	1.15862
C	5.88583	-0.14318	-0.14647
H	5.86925	0.65935	-2.08223
H	5.61935	-0.81586	1.93971
C	3.66758	2.32383	-2.05442
H	2.75522	2.88984	-1.78070
C	4.72721	3.37883	-2.39419
H	4.98602	3.96205	-1.48452
H	4.33070	4.08350	-3.15619
H	5.65051	2.91202	-2.79682
C	3.30099	1.49377	-3.29115
H	2.55426	0.71649	-3.02234
H	4.19392	0.99622	-3.72419
H	2.85394	2.14896	-4.06899
C	3.33188	0.31539	2.71651
H	2.32741	0.78403	2.66678
C	4.10700	1.02340	3.83392
H	3.54302	0.95802	4.78882
H	4.24424	2.09656	3.58650
H	5.10691	0.56159	3.97909
C	3.09379	-1.16059	3.07644
H	2.69987	-1.71632	2.20204
H	2.35426	-1.23367	3.90221
H	4.03113	-1.65437	3.40884

C	7.16233	-0.93238	-0.37648
H	7.51964	-0.78129	-1.42013
C	6.92287	-2.43688	-0.20022
H	6.11632	-2.77807	-0.88142
H	6.63657	-2.68069	0.84454
H	7.84667	-3.00017	-0.45188
C	8.27807	-0.44326	0.55378
H	8.43939	0.64708	0.41347
H	9.22834	-0.96754	0.31648
H	8.02581	-0.63468	1.61838
C	-6.41202	-3.54226	0.53961
H	-6.58445	-4.15012	-0.37721
C	-5.63392	-4.42635	1.52073
H	-4.64361	-4.68752	1.09087
H	-6.19018	-5.36981	1.70652
H	-5.47924	-3.91048	2.49205
C	-7.79208	-3.17953	1.10023
H	-8.33577	-2.52973	0.38146
H	-7.70549	-2.64748	2.07115
H	-8.39227	-4.10118	1.25686
H	0.83407	-1.94588	0.68193
C	2.14591	-3.52390	-0.87483
C	0.58346	-4.54042	0.51791
C	2.85911	-2.35503	-1.25011
C	2.33235	-4.72798	-1.62405
C	0.71636	-5.77785	-0.16811
C	3.70873	-2.38940	-2.33131
H	2.70622	-1.44328	-0.68565
C	3.23463	-4.73247	-2.71855
C	1.58549	-5.86343	-1.22783
H	0.14765	-6.63704	0.16662
C	3.90620	-3.58373	-3.06668
H	4.23724	-1.49140	-2.63132
H	3.37623	-5.65220	-3.27936
H	1.71055	-6.79904	-1.76641
H	4.58800	-3.58512	-3.91139
N	1.28375	-3.46771	0.18242
C	-0.38605	-4.43208	1.64058
C	-1.61622	-4.94815	1.61158
H	-1.97382	-5.45062	0.71938
H	-2.29531	-4.85297	2.44990
Cl	0.17697	-3.56722	3.06450

Catalyst (TRIP, 3)-Quinoline complex s-cis (alternative conformation)
Electronic energy (Hartrees): -1582.908714
Free Energy at 1M concentration and 253 K (Hartrees): -1581.807664
Coordinates:

P	-0.17368	-0.24454	-0.55662
O	0.58745	1.14936	-0.97251
O	-1.49034	0.18783	0.30769
O	0.69058	-0.86458	0.60778
O	-0.38990	-1.05079	-1.77277
C	0.58858	2.13062	-0.01280
C	1.88504	2.55927	0.57301
C	1.92736	3.61831	1.39377
H	2.87354	3.95301	1.80131
C	0.68388	4.29954	1.80198
C	0.74306	5.39421	2.79815
H	1.69895	5.73301	3.17802
C	-0.38328	5.96532	3.23713

H	-0.33920	6.76334	3.96701
C	-1.69098	5.49645	2.74199
H	-2.60030	5.94282	3.12348
C	-1.75501	4.51241	1.83766
H	-2.72712	4.16566	1.51483
C	-0.50850	3.89157	1.30907
C	-0.54884	2.80226	0.28473
C	-2.26006	1.14481	-0.30222
C	-3.64083	0.79792	-0.71993
C	-4.42810	1.73651	-1.26639
H	-5.44766	1.49792	-1.54453
C	-3.90946	3.09892	-1.50528
C	-4.75380	4.09470	-2.20457
H	-5.75868	3.83478	-2.51349
C	-4.27777	5.31803	-2.46063
H	-4.89473	6.04457	-2.97344
C	-2.90496	5.67744	-2.05646
H	-2.51837	6.66194	-2.28634
C	-2.13602	4.78702	-1.41904
H	-1.12488	5.06377	-1.15146
C	-2.66168	3.43210	-1.09981
C	-1.82746	2.42327	-0.38034
C	-4.17123	-0.56123	-0.45505
C	-4.49232	-0.95688	0.96049
C	-4.36514	-1.45204	-1.46860
C	-4.94374	-2.20164	1.20371
C	-4.91486	-2.79662	-1.15237
C	-5.16836	-3.16962	0.10935
H	-5.17497	-2.50585	2.21546
H	-5.10090	-3.49241	-1.95991
C	-4.03658	-1.13438	-2.92794
H	-3.48577	-0.17546	-3.01636
C	-3.12019	-2.20012	-3.54785
H	-2.26587	-2.41605	-2.87465
H	-2.71805	-1.83506	-4.51710
H	-3.66644	-3.14755	-3.73826
C	-5.32370	-0.98816	-3.74695
H	-5.89615	-1.94000	-3.76417
H	-5.07999	-0.70114	-4.79218
H	-5.96624	-0.19447	-3.31089
C	-4.34915	0.02574	2.12311
H	-3.97875	1.00625	1.76549
C	-3.32925	-0.47221	3.15702
H	-2.36266	-0.70560	2.66307
H	-3.69251	-1.38391	3.67626
H	-3.14694	0.31539	3.91903
C	-5.70589	0.30914	2.78115
H	-6.11633	-0.59903	3.27078
H	-6.43044	0.66472	2.01748
H	-5.59628	1.10285	3.55085
C	3.13047	1.80742	0.27887
C	3.75772	1.93148	-0.92449
C	3.75425	0.96934	1.36415
C	5.04887	1.22551	-1.14145
C	4.96005	0.41315	1.14692
C	5.63561	0.52261	-0.16240
H	5.54039	1.31173	-2.10176
H	5.44739	-0.14754	1.93240
C	3.23105	2.82608	-2.04770
H	2.26990	3.30451	-1.77342

C	4.20526	3.97611	-2.32973
H	4.39142	4.55175	-1.39772
H	3.76990	4.66737	-3.08275
H	5.17463	3.59949	-2.71880
C	2.95765	2.01564	-3.32088
H	2.28966	1.15797	-3.09458
H	3.89908	1.63058	-3.76500
H	2.45378	2.65584	-4.07614
C	3.06591	0.75849	2.71311
H	2.01972	1.12458	2.66675
C	3.77695	1.55081	3.81707
H	3.22527	1.44685	4.77573
H	3.81556	2.62842	3.55650
H	4.81561	1.18480	3.96334
C	2.97692	-0.72913	3.09597
H	2.61975	-1.33389	2.24037
H	2.26403	-0.86106	3.93775
H	3.96311	-1.12726	3.41497
C	6.98317	-0.13897	-0.38861
H	7.28894	-0.02116	-1.45276
C	6.92101	-1.64502	-0.10505
H	6.08722	-2.10569	-0.67021
H	6.77081	-1.84830	0.97607
H	7.86782	-2.13033	-0.42430
C	8.06209	0.53174	0.46814
H	8.10968	1.61761	0.23752
H	9.05552	0.08618	0.24722
H	7.84893	0.40451	1.55099
C	-5.68646	-4.56420	0.41030
H	-5.79216	-5.14287	-0.53508
C	-4.69881	-5.33296	1.29596
H	-3.69812	-5.36157	0.81369
H	-5.04706	-6.37874	1.43457
H	-4.60135	-4.85857	2.29545
C	-7.07559	-4.50634	1.05622
H	-7.77437	-3.93888	0.40492
H	-7.03644	-4.01758	2.05266
H	-7.47791	-5.53395	1.18401
H	1.15189	-1.73061	0.35658
C	2.99354	-3.02660	-0.97629
C	1.99063	-4.18694	0.77400
C	3.10598	-1.83454	-1.73950
C	3.84159	-4.13715	-1.28750
C	2.81410	-5.32237	0.54797
C	3.99912	-1.77152	-2.78431
H	2.48418	-0.98497	-1.49007
C	4.74673	-4.03811	-2.37582
C	3.72516	-5.29310	-0.47950
H	2.68826	-6.20131	1.16878
C	4.81891	-2.87911	-3.11321
H	4.07680	-0.86137	-3.36899
H	5.38062	-4.88862	-2.61071
H	4.35287	-6.15549	-0.68773
H	5.51191	-2.80439	-3.94537
N	2.07924	-3.08921	0.03766
C	0.99681	-4.22844	1.88384
C	1.30234	-4.59991	3.12794
H	2.32643	-4.85245	3.38218
H	0.55736	-4.63659	3.91341
Cl	-0.65158	-3.79086	1.46193

Catalyst (TRIP, 3)-Quinoline complex s-cis (alternative conformation)
Electronic energy (Hartrees): -1582.909988
Free Energy at 1M concentration and 253 K (Hartrees): -1581.80696
Coordinates:

P	0.03086	-0.20995	-0.63067
O	1.18500	0.91875	-0.88905
O	-1.15098	0.50519	0.26271
O	0.60405	-1.16755	0.49114
O	-0.39257	-0.79881	-1.91375
C	1.47557	1.73601	0.17531
C	2.85805	1.73826	0.72051
C	3.18517	2.58323	1.70919
H	4.19827	2.60659	2.09281
C	2.16962	3.48116	2.29226
C	2.52283	4.33680	3.44852
H	3.53087	4.32781	3.84422
C	1.59773	5.12161	4.01113
H	1.85597	5.74518	4.85728
C	0.21806	5.12978	3.48881
H	-0.53173	5.74935	3.96367
C	-0.10944	4.36510	2.44083
H	-1.13321	4.36849	2.09124
C	0.91640	3.50955	1.78361
C	0.57903	2.65223	0.60707
C	-1.54395	1.73156	-0.20822
C	-2.93826	1.91327	-0.68405
C	-3.34857	3.11932	-1.10372
H	-4.37392	3.26546	-1.42243
C	-2.41123	4.25944	-1.13281
C	-2.85274	5.56012	-1.68668
H	-3.86714	5.68236	-2.04554
C	-1.99978	6.58825	-1.74907
H	-2.32252	7.53787	-2.15591
C	-0.61181	6.42769	-1.27522
H	0.07968	7.25713	-1.34917
C	-0.20488	5.25847	-0.76751
H	0.82375	5.15648	-0.44819
C	-1.15054	4.11372	-0.66375
C	-0.72400	2.79951	-0.09576
C	-3.90551	0.79352	-0.60886
C	-4.50715	0.43627	0.72167
C	-4.29774	0.12936	-1.73152
C	-5.43860	-0.53503	0.77654
C	-5.36619	-0.89818	-1.62592
C	-5.91108	-1.22200	-0.44536
H	-5.87920	-0.80996	1.72523
H	-5.71862	-1.38441	-2.52611
C	-3.70028	0.41044	-3.10994
H	-2.81226	1.07202	-3.03291
C	-3.20587	-0.87883	-3.78311
H	-2.59551	-1.47659	-3.07527
H	-2.57634	-0.62796	-4.66351
H	-4.05228	-1.50603	-4.13332
C	-4.71927	1.12839	-4.00160
H	-5.61249	0.49240	-4.17934
H	-4.25938	1.37701	-4.98186
H	-5.04616	2.07531	-3.52279
C	-4.11010	1.16871	2.00368
H	-3.35832	1.95364	1.79114

C	-3.45977	0.21642	3.01506
H	-2.59498	-0.30082	2.54902
H	-4.18326	-0.54415	3.37660
H	-3.08703	0.78960	3.89067
C	-5.30941	1.89753	2.62396
H	-6.07767	1.18094	2.98329
H	-5.77111	2.57433	1.87336
H	-4.97505	2.51332	3.48618
C	3.89548	0.85211	0.13944
C	4.45063	1.13179	-1.07376
C	4.39790	-0.31907	0.93974
C	5.54466	0.26478	-1.58406
C	5.42623	-1.04437	0.46010
C	6.02809	-0.75247	-0.85861
H	5.98019	0.48266	-2.55056
H	5.82491	-1.86771	1.03668
C	4.04359	2.34095	-1.91856
H	3.23550	2.92610	-1.43700
C	5.21760	3.31370	-2.08343
H	5.60295	3.61454	-1.08560
H	4.88050	4.22939	-2.61483
H	6.04515	2.85649	-2.66542
C	3.49921	1.90133	-3.28368
H	2.67027	1.17457	-3.14964
H	4.29071	1.42946	-3.90271
H	3.10238	2.78072	-3.83461
C	3.78795	-0.68480	2.29257
H	2.87699	-0.08035	2.47871
C	4.76783	-0.38146	3.43230
H	4.28309	-0.57511	4.41298
H	5.07254	0.68572	3.40177
H	5.67769	-1.01406	3.35544
C	3.33510	-2.15189	2.34429
H	2.70623	-2.38945	1.46500
H	2.73210	-2.33031	3.26008
H	4.20040	-2.84720	2.35859
C	7.18567	-1.58474	-1.38016
H	7.48655	-1.22687	-2.39070
C	6.78349	-3.05549	-1.52990
H	5.87313	-3.13980	-2.16133
H	6.58294	-3.51649	-0.54010
H	7.60107	-3.62431	-2.02190
C	8.41198	-1.44124	-0.47171
H	8.69219	-0.37015	-0.37798
H	9.27467	-1.98852	-0.90816
H	8.21114	-1.84939	0.54158
C	-7.01764	-2.25893	-0.36955
H	-7.25760	-2.63430	-1.39000
C	-6.57681	-3.47052	0.45708
H	-5.65855	-3.91259	0.01560
H	-7.37125	-4.24691	0.44944
H	-6.37228	-3.18527	1.51070
C	-8.30595	-1.64756	0.19367
H	-8.60308	-0.76345	-0.41012
H	-8.17405	-1.33311	1.25056
H	-9.13033	-2.39092	0.14853
H	0.55659	-2.15152	0.27397
C	-0.97207	-4.18353	0.75860
C	1.10486	-4.79711	-0.09484
C	-1.86736	-3.15884	1.16871

C	-1.37901	-5.55006	0.88361
C	0.76566	-6.17793	-0.02373
C	-3.10116	-3.48910	1.68153
H	-1.56028	-2.12441	1.08349
C	-2.65986	-5.85270	1.41128
C	-0.46322	-6.54371	0.46241
H	1.47116	-6.91890	-0.37881
C	-3.50293	-4.84092	1.80792
H	-3.77829	-2.70164	1.99451
H	-2.95807	-6.89370	1.50031
H	-0.74738	-7.59119	0.51848
H	-4.48060	-5.07527	2.21749
N	0.25932	-3.84255	0.26632
C	2.46569	-4.42799	-0.56990
C	3.55871	-5.13607	-0.27192
H	3.49028	-6.00554	0.37217
H	4.53613	-4.85630	-0.64050
Cl	2.59462	-2.98934	-1.56317

Catalyst (TRIP, 3)-Quinoline complex Aniline addition TS
Electronic energy (Hartrees): -1870.543277
Free Energy at 1M concentration and 253 K (Hartrees): -1869.322984
Coordinates:

P	0.31276	-0.15607	-0.49797
O	1.46783	0.99812	-0.73389
O	-1.06513	0.63601	-0.02421
O	0.65671	-1.00778	0.71183
O	0.16271	-0.80873	-1.84344
C	1.44475	2.03933	0.15673
C	2.60403	2.23416	1.05895
C	2.63400	3.28148	1.89456
H	3.49164	3.44124	2.53709
C	1.49860	4.22538	1.94696
C	1.50744	5.32958	2.93394
H	2.35724	5.46452	3.59155
C	0.46433	6.16193	3.02078
H	0.47058	6.96692	3.74429
C	-0.70433	5.97818	2.13926
H	-1.55460	6.64177	2.23033
C	-0.71962	4.98868	1.23884
H	-1.59765	4.85990	0.62027
C	0.44501	4.07036	1.11041
C	0.45172	2.95471	0.11688
C	-1.40354	1.68108	-0.84465
C	-2.67613	1.62713	-1.60192
C	-2.97322	2.60025	-2.47573
H	-3.92217	2.59029	-2.99960
C	-2.05406	3.74432	-2.65634
C	-2.36267	4.78552	-3.66318
H	-3.25357	4.70482	-4.27355
C	-1.54192	5.82954	-3.82342
H	-1.76739	6.58936	-4.56055
C	-0.32236	5.94608	-3.00046
H	0.34537	6.78532	-3.14671
C	-0.03764	5.01460	-2.08290
H	0.86990	5.11003	-1.50135
C	-0.95185	3.85951	-1.87778
C	-0.65079	2.80117	-0.86974
C	-3.71563	0.65010	-1.20942
C	-4.59993	0.97960	-0.03973

C	-3.87551	-0.52698	-1.87213
C	-5.52940	0.08569	0.34721
C	-4.97199	-1.44260	-1.45976
C	-5.75624	-1.16568	-0.40853
H	-6.15495	0.29128	1.20506
H	-5.14207	-2.34835	-2.02707
C	-2.98256	-0.94795	-3.03566
H	-2.12892	-0.24970	-3.15668
C	-2.37353	-2.33875	-2.79527
H	-1.93320	-2.39843	-1.77690
H	-1.56954	-2.53292	-3.53696
H	-3.13502	-3.14019	-2.89560
C	-3.76509	-0.91520	-4.35228
H	-4.60642	-1.64025	-4.33683
H	-3.09387	-1.16854	-5.20054
H	-4.17322	0.10384	-4.52465
C	-4.46649	2.30431	0.71437
H	-3.73514	2.97004	0.21536
C	-3.93551	2.08084	2.13252
H	-2.93978	1.59280	2.08092
H	-4.62571	1.44415	2.72552
H	-3.81237	3.05431	2.65354
C	-5.78782	3.08535	0.73130
H	-6.55574	2.57322	1.34802
H	-6.17348	3.20140	-0.30423
H	-5.62290	4.09931	1.15466
C	3.75035	1.30030	0.98835
C	4.67260	1.41148	-0.00764
C	3.89504	0.22613	2.03058
C	5.79144	0.43540	-0.06549
C	4.95680	-0.59952	1.97062
C	5.93141	-0.52349	0.86058
H	6.50667	0.50354	-0.87489
H	5.10439	-1.35047	2.73428
C	4.62676	2.52234	-1.05950
H	3.75649	3.19297	-0.90964
C	5.86690	3.41854	-0.95940
H	5.95647	3.82746	0.06991
H	5.77705	4.27241	-1.66468
H	6.79295	2.85723	-1.20482
C	4.48027	1.94552	-2.47246
H	3.60196	1.26806	-2.52023
H	5.38823	1.38425	-2.77561
H	4.32087	2.76790	-3.20225
C	2.89200	0.08686	3.17554
H	1.96855	0.65479	2.93817
C	3.46878	0.67699	4.46682
H	2.71495	0.62275	5.28113
H	3.73829	1.74306	4.31508
H	4.37802	0.12217	4.78333
C	2.44703	-1.36970	3.40131
H	2.24918	-1.87683	2.43593
H	1.51516	-1.39069	4.00617
H	3.22198	-1.95092	3.94448
C	7.06857	-1.52425	0.76560
H	7.67115	-1.32877	-0.15013
C	6.53097	-2.95602	0.64873
H	5.82492	-3.03143	-0.20484
H	6.00321	-3.26472	1.57581
H	7.36903	-3.66269	0.46880

C	8.01571	-1.38945	1.96310
H	8.39561	-0.34753	2.03176
H	8.88607	-2.06841	1.83743
H	7.50158	-1.64733	2.91318
C	-6.88643	-2.10398	-0.02441
H	-6.92320	-2.96428	-0.73039
C	-6.66353	-2.68584	1.37434
H	-5.70062	-3.23536	1.39897
H	-7.47468	-3.40249	1.62416
H	-6.64552	-1.88755	2.14627
C	-8.24306	-1.39539	-0.11550
H	-8.38107	-0.96689	-1.13132
H	-8.32182	-0.57869	0.63296
H	-9.06374	-2.12118	0.06872
C	-1.91188	-2.39575	2.32009
C	-1.09421	-4.15095	0.87577
C	-1.70519	-1.11318	2.87115
C	-3.02365	-3.17878	2.72330
C	-2.24037	-4.93581	1.23405
C	-2.59165	-0.64300	3.82119
H	-0.85582	-0.53003	2.53462
C	-3.91923	-2.65642	3.68255
C	-3.16226	-4.46391	2.12204
H	-2.34439	-5.91938	0.79924
C	-3.70657	-1.40606	4.22726
H	-2.42739	0.33629	4.26046
H	-4.77234	-3.25711	3.98529
H	-4.01714	-5.07931	2.38958
H	-4.39295	-1.00855	4.96784
C	-0.03162	-4.66115	0.07256
C	1.18206	-4.05290	-0.19741
H	2.02362	-4.67991	-0.46554
H	1.42297	-3.11115	0.28722
N	1.29480	-3.24433	-2.11516
N	-1.01758	-2.90130	1.40285
C	2.63980	-3.18303	-2.54372
C	3.26802	-4.32487	-3.06364
C	3.37900	-2.00349	-2.36198
C	4.61762	-4.28443	-3.40904
H	2.69470	-5.23889	-3.19945
C	4.72672	-1.97836	-2.71184
H	2.88931	-1.11865	-1.97008
C	5.35428	-3.11234	-3.23277
H	5.09251	-5.17242	-3.81600
H	5.29458	-1.06442	-2.57652
H	6.40649	-3.08166	-3.49800
H	0.73536	-3.91742	-2.62970
H	0.81565	-2.31404	-2.04223
H	-0.27635	-2.20435	1.08252
Cl	-0.26554	-6.26855	-0.66508

Catalyst (TRIP, 3)-Quinoline complex Aniline addition product
Electronic energy (Hartrees): -1870.554057
Free Energy at 1M concentration and 253 K (Hartrees): -1869.332857
Coordinates:

P	-0.36213	-0.23138	0.41738
O	-1.61647	0.76783	0.78197
O	0.89781	0.71991	-0.05214
O	-0.68203	-1.11103	-0.76148
O	-0.05351	-0.91747	1.74451

C	-1.74925	1.85664	-0.04064
C	-2.96342	1.97861	-0.88440
C	-3.15310	3.07682	-1.62910
H	-4.04837	3.17829	-2.23109
C	-2.13732	4.14774	-1.65360
C	-2.32272	5.31367	-2.54797
H	-3.21850	5.40019	-3.15033
C	-1.38307	6.26238	-2.61910
H	-1.51830	7.11286	-3.27474
C	-0.15205	6.14563	-1.81482
H	0.61551	6.90452	-1.89561
C	0.02232	5.09988	-0.99868
H	0.94508	5.02403	-0.43962
C	-1.02804	4.05052	-0.88442
C	-0.86072	2.87319	0.02079
C	1.16739	1.75263	0.81088
C	2.48582	1.80876	1.48690
C	2.73516	2.77391	2.38434
H	3.71045	2.84367	2.85239
C	1.71013	3.79466	2.68539
C	1.96969	4.81563	3.72627
H	2.90642	4.81255	4.26964
C	1.04779	5.74527	4.00014
H	1.23752	6.49038	4.76191
C	-0.23467	5.75399	3.26991
H	-0.98286	6.49885	3.50847
C	-0.47633	4.84004	2.32288
H	-1.42968	4.85301	1.81113
C	0.54850	3.81440	1.99086
C	0.30099	2.78022	0.94363
C	3.59188	0.94566	1.01700
C	4.35843	1.36229	-0.20661
C	3.94933	-0.17936	1.69262
C	5.40883	0.61901	-0.60243
C	5.15071	-0.93533	1.24914
C	5.85603	-0.55848	0.17301
H	5.96488	0.89525	-1.48785
H	5.47677	-1.79019	1.82719
C	3.18188	-0.68819	2.90995
H	2.24920	-0.10765	3.06662
C	2.74802	-2.14858	2.71980
H	2.24978	-2.27282	1.73604
H	2.02913	-2.43745	3.51594
H	3.61392	-2.84151	2.76630
C	4.01942	-0.52760	4.18269
H	4.94347	-1.14192	4.13535
H	3.42896	-0.84347	5.06915
H	4.30388	0.53801	4.31685
C	3.97566	2.61007	-1.00429
H	3.14671	3.15469	-0.51136
C	3.46733	2.23774	-2.40059
H	2.58763	1.56677	-2.30926
H	4.25460	1.72641	-2.99394
H	3.14713	3.15046	-2.94703
C	5.13665	3.61068	-1.08349
H	5.97472	3.21422	-1.69409
H	5.50996	3.84008	-0.06244
H	4.78830	4.55911	-1.54572
C	-3.97836	0.90043	-0.89105
C	-4.88923	0.79635	0.11563

C	-4.04214	-0.04178	-2.06138
C	-5.94501	-0.24547	0.02687
C	-5.04132	-0.94188	-2.11965
C	-6.02623	-1.07241	-1.02453
H	-6.66341	-0.33515	0.83139
H	-5.12738	-1.60256	-2.97115
C	-4.89565	1.72683	1.32942
H	-4.07610	2.47162	1.27931
C	-6.19560	2.53754	1.39264
H	-6.34228	3.09263	0.44134
H	-6.14224	3.27680	2.22032
H	-7.07472	1.88203	1.56583
C	-4.67522	0.93820	2.62627
H	-3.73907	0.34433	2.55693
H	-5.52209	0.25031	2.83106
H	-4.57767	1.63748	3.48393
C	-3.01565	0.02035	-3.19194
H	-2.15379	0.64888	-2.88571
C	-3.62717	0.66642	-4.44001
H	-2.85822	0.76025	-5.23635
H	-4.00336	1.68299	-4.20108
H	-4.47114	0.05717	-4.82858
C	-2.43174	-1.36240	-3.53215
H	-2.17401	-1.91945	-2.60886
H	-1.50924	-1.24374	-4.13986
H	-3.15099	-1.97380	-4.11692
C	-7.10321	-2.14035	-1.08117
H	-7.73690	-2.09016	-0.16700
C	-6.48211	-3.54189	-1.11695
H	-5.80724	-3.68277	-0.24596
H	-5.90042	-3.70040	-2.04964
H	-7.28051	-4.31253	-1.06453
C	-8.03106	-1.91915	-2.28108
H	-8.46754	-0.89818	-2.24099
H	-8.86427	-2.65359	-2.25731
H	-7.48396	-2.03840	-3.24015
C	7.12525	-1.29737	-0.21317
H	7.30753	-2.13631	0.49589
C	7.00327	-1.91330	-1.60970
H	6.14280	-2.61008	-1.63356
H	7.91998	-2.49123	-1.85383
H	6.85939	-1.13323	-2.38678
C	8.34268	-0.36939	-0.12886
H	8.41345	0.07387	0.88753
H	8.27481	0.45097	-0.87457
H	9.27342	-0.94458	-0.32148
C	2.16367	-2.30912	-2.45772
C	1.71996	-3.94491	-0.69209
C	1.73839	-1.16280	-3.15391
C	3.38672	-2.93336	-2.79902
C	2.99763	-4.55418	-1.01523
C	2.52348	-0.66113	-4.18167
H	0.80912	-0.68474	-2.86277
C	4.16005	-2.39893	-3.84491
C	3.77000	-4.08314	-2.02301
H	3.30294	-5.41975	-0.44362
C	3.73794	-1.27252	-4.53446
H	2.19226	0.22497	-4.71557
H	5.09886	-2.88326	-4.10128
H	4.70754	-4.58027	-2.25865

H	4.34108	-0.86452	-5.33908
C	0.86100	-4.44575	0.26200
C	-0.53375	-4.04046	0.53024
H	-1.20162	-4.90726	0.56741
H	-0.90486	-3.33183	-0.21122
N	-0.69318	-3.34133	1.89576
N	1.39014	-2.82794	-1.43898
C	-2.04336	-3.38348	2.43981
C	-2.30317	-4.13395	3.58585
C	-3.06011	-2.67310	1.79787
C	-3.59700	-4.17213	4.10550
H	-1.50067	-4.68398	4.07076
C	-4.35141	-2.73065	2.32079
H	-2.84643	-2.08495	0.91080
C	-4.62330	-3.47280	3.47096
H	-3.79910	-4.75143	5.00096
H	-5.15106	-2.18831	1.83116
H	-5.63209	-3.50434	3.87047
H	-0.05491	-3.81169	2.54010
H	-0.35416	-2.23242	1.80915
H	0.56937	-2.25709	-1.18004
Cl	1.38837	-5.80317	1.29585

Catalyst (TRIP, 3)-Quinoline complex Aniline addition Proton transfer TS
Electronic energy (Hartrees): -1870.553975
Free Energy at 1M concentration and 253 K (Hartrees): -1869.334416
Coordinates:

P	0.37078	-0.23044	-0.38857
O	1.62047	0.75780	-0.77852
O	-0.88660	0.72603	0.06638
O	0.68982	-1.10941	0.78631
O	0.04770	-0.92434	-1.71729
C	1.76806	1.85129	0.03511
C	2.98943	1.97081	0.86881
C	3.19395	3.07486	1.60101
H	4.09403	3.17437	2.19606
C	2.18557	4.15284	1.62531
C	2.38611	5.32460	2.50875
H	3.28818	5.41072	3.10174
C	1.45232	6.27893	2.58186
H	1.59855	7.13358	3.22968
C	0.21274	6.16275	1.79070
H	-0.54990	6.92639	1.87329
C	0.02463	5.11171	0.98445
H	-0.90405	5.03641	0.43516
C	1.06807	4.05579	0.86801
C	0.88536	2.87246	-0.02624
C	-1.15291	1.75658	-0.80097
C	-2.47424	1.81696	-1.47144
C	-2.72337	2.78058	-2.37052
H	-3.70013	2.85276	-2.83508
C	-1.69505	3.79498	-2.68049
C	-1.95458	4.81253	-3.72473
H	-2.89414	4.81198	-4.26320
C	-1.02906	5.73574	-4.00790
H	-1.21872	6.47823	-4.77223
C	0.25731	5.74062	-3.28467
H	1.00842	6.48007	-3.53081
C	0.49901	4.82966	-2.33478
H	1.45538	4.83960	-1.82868

C	-0.52980	3.81130	-1.99224
C	-0.28240	2.78036	-0.94158
C	-3.58180	0.95713	-0.99874
C	-4.34346	1.37477	0.22756
C	-3.94950	-0.16192	-1.67879
C	-5.40183	0.64063	0.61925
C	-5.15764	-0.90879	-1.23824
C	-5.86044	-0.52884	-0.16157
H	-5.95557	0.91849	1.50560
H	-5.49186	-1.75787	-1.82012
C	-3.18780	-0.67161	-2.89940
H	-2.24979	-0.09901	-3.05378
C	-2.76702	-2.13661	-2.71681
H	-2.26838	-2.26952	-1.73450
H	-2.05214	-2.42826	-3.51551
H	-3.63923	-2.82147	-2.76491
C	-4.02486	-0.49732	-4.17066
H	-4.95428	-1.10364	-4.12556
H	-3.43790	-0.81412	-5.05911
H	-4.29996	0.57138	-4.29946
C	-3.94739	2.61365	1.03242
H	-3.11125	3.15102	0.54375
C	-3.44590	2.22779	2.42758
H	-2.57485	1.54592	2.33410
H	-4.24044	1.72363	3.01737
H	-3.11483	3.13355	2.97909
C	-5.09679	3.62726	1.11514
H	-5.94078	3.23696	1.72155
H	-5.46516	3.86705	0.09468
H	-4.73843	4.56879	1.58376
C	3.99063	0.87980	0.88499
C	4.88223	0.73777	-0.13438
C	4.05983	-0.03651	2.07578
C	5.92132	-0.32018	-0.04031
C	5.04405	-0.95272	2.13666
C	6.00674	-1.12503	1.02775
H	6.62466	-0.43938	-0.85422
H	5.13416	-1.59542	3.00143
C	4.88509	1.64181	-1.36803
H	4.07853	2.40082	-1.32185
C	6.19656	2.43000	-1.46740
H	6.36571	3.00321	-0.53068
H	6.14283	3.15190	-2.31025
H	7.06265	1.75685	-1.63852
C	4.63343	0.82942	-2.64455
H	3.69047	0.25032	-2.54890
H	5.46722	0.12527	-2.84774
H	4.53243	1.51203	-3.51515
C	3.05570	0.06852	3.22337
H	2.20343	0.71265	2.92319
C	3.70484	0.71869	4.45030
H	2.95209	0.84583	5.25742
H	4.10346	1.72090	4.18826
H	4.53895	0.09397	4.83553
C	2.44513	-1.29406	3.59563
H	2.14895	-1.85446	2.68622
H	1.54185	-1.14388	4.22489
H	3.16292	-1.91728	4.16955
C	7.06694	-2.20927	1.09049
H	7.68221	-2.19353	0.16258

C	6.42429	-3.59897	1.17605
H	5.72670	-3.75052	0.32503
H	5.86240	-3.72462	2.12570
H	7.20878	-4.38381	1.12467
C	8.02275	-1.97228	2.26510
H	8.47564	-0.96059	2.18848
H	8.84265	-2.72165	2.24475
H	7.49323	-2.05589	3.23774
C	-7.13861	-1.25500	0.21918
H	-7.32761	-2.09047	-0.49221
C	-7.02798	-1.87501	1.61490
H	-6.17376	-2.57933	1.64119
H	-7.95088	-2.44525	1.85375
H	-6.88072	-1.09795	2.39433
C	-8.34561	-0.31366	0.13281
H	-8.40857	0.13194	-0.88308
H	-8.27084	0.50481	0.87995
H	-9.28317	-0.87888	0.32182
C	-2.16772	-2.31505	2.47064
C	-1.74558	-3.94153	0.68768
C	-1.72649	-1.18296	3.17969
C	-3.40219	-2.92214	2.80140
C	-3.03296	-4.53442	1.00481
C	-2.50772	-0.67696	4.20867
H	-0.78773	-0.71784	2.89814
C	-4.17095	-2.38411	3.84835
C	-3.80038	-4.06029	2.01447
H	-3.34999	-5.39045	0.42527
C	-3.73367	-1.27064	4.55016
H	-2.16374	0.19859	4.75186
H	-5.11864	-2.85521	4.09656
H	-4.74547	-4.54572	2.24414
H	-4.33388	-0.85993	5.35557
C	-0.89498	-4.44104	-0.27233
C	0.50453	-4.04314	-0.54373
H	1.16119	-4.91895	-0.58589
H	0.88198	-3.35472	0.21338
N	0.66162	-3.32833	-1.89202
N	-1.39960	-2.83737	1.44938
C	2.00155	-3.37571	-2.45008
C	2.22456	-4.02403	-3.66519
C	3.05317	-2.75979	-1.76605
C	3.50906	-4.05543	-4.20760
H	1.39829	-4.50153	-4.18589
C	4.33551	-2.81209	-2.31103
H	2.87161	-2.24269	-0.82883
C	4.56785	-3.45296	-3.52871
H	3.67897	-4.55601	-5.15579
H	5.15946	-2.34431	-1.78558
H	5.56975	-3.48108	-3.94544
H	0.01484	-3.78146	-2.53932
H	0.32057	-2.14859	-1.78573
H	-0.57071	-2.27993	1.19829
Cl	-1.44049	-5.78025	-1.31939

Catalyst (TRIP, 3)-Quinoline complex Aniline addition Proton transfer product
Electronic energy (Hartrees): -1870.554117
Free Energy at 1M concentration and 253 K (Hartrees): -1869.334787
Coordinates:

P	0.33521	-0.15173	-0.36399

O	1.57150	0.77919	-0.89498
O	-0.86817	0.83145	0.17006
O	0.72584	-1.06150	0.75362
O	-0.14706	-0.81636	-1.68580
C	1.84287	1.79086	-0.01594
C	3.13755	1.79041	0.72327
C	3.47360	2.85131	1.48066
H	4.42518	2.86819	2.00403
C	2.53771	3.98942	1.63908
C	2.88543	5.10639	2.55575
H	3.83948	5.10939	3.07429
C	2.02003	6.11376	2.75010
H	2.27377	6.92792	3.42137
C	0.71001	6.11028	2.06012
H	0.00541	6.91615	2.23914
C	0.38865	5.10828	1.22582
H	-0.58611	5.11442	0.75011
C	1.35310	3.99573	0.97632
C	1.02483	2.87037	0.04396
C	-1.10224	1.87500	-0.68812
C	-2.43285	1.98809	-1.35275
C	-2.68750	3.02804	-2.16875
H	-3.66649	3.13437	-2.62822
C	-1.65001	4.05325	-2.42677
C	-1.91905	5.14675	-3.39665
H	-2.87867	5.20404	-3.90156
C	-0.97615	6.06652	-3.65451
H	-1.17253	6.86373	-4.36426
C	0.33958	5.98977	-2.97975
H	1.10518	6.72434	-3.20821
C	0.58976	5.00910	-2.09703
H	1.56799	4.95956	-1.63093
C	-0.45933	3.99523	-1.77844
C	-0.20698	2.89156	-0.79759
C	-3.52830	1.03749	-1.02146
C	-4.28754	1.21839	0.26867
C	-3.90816	0.06480	-1.90233
C	-5.34152	0.41250	0.52768
C	-5.12129	-0.75021	-1.60475
C	-5.81005	-0.59060	-0.46108
H	-5.88929	0.51636	1.45785
H	-5.46368	-1.47302	-2.33821
C	-3.14991	-0.21680	-3.19996
H	-2.18444	0.33063	-3.22094
C	-2.80002	-1.70753	-3.32602
H	-2.41009	-2.09397	-2.36183
H	-2.02145	-1.85084	-4.10554
H	-3.68786	-2.31169	-3.60792
C	-3.96033	0.25414	-4.41192
H	-4.92313	-0.29486	-4.48630
H	-3.38173	0.08579	-5.34524
H	-4.17523	1.34028	-4.32873
C	-3.90322	2.30182	1.27926
H	-3.06005	2.91135	0.89932
C	-3.42576	1.68207	2.59578
H	-2.57468	1.00069	2.39422
H	-4.23707	1.10939	3.09265
H	-3.07610	2.47641	3.28913
C	-5.05638	3.28595	1.51563
H	-5.91341	2.79354	2.02124

H	-5.40155	3.70512	0.54626
H	-4.71169	4.12753	2.15387
C	4.06112	0.62567	0.63275
C	4.80746	0.40580	-0.49188
C	4.23561	-0.27187	1.83555
C	5.79135	-0.71525	-0.50075
C	5.17403	-1.24293	1.79764
C	5.97661	-1.49700	0.57728
H	6.37884	-0.89410	-1.39547
H	5.34396	-1.87004	2.66538
C	4.71790	1.28921	-1.73961
H	3.96649	2.09411	-1.61772
C	6.05105	1.99886	-2.00408
H	6.35558	2.57909	-1.10675
H	5.94266	2.70642	-2.85380
H	6.85424	1.27389	-2.25289
C	4.28003	0.47035	-2.96144
H	3.33223	-0.06635	-2.74486
H	5.05184	-0.27422	-3.24843
H	4.10575	1.14440	-3.82707
C	3.39052	-0.09507	3.09996
H	2.56729	0.62408	2.91087
C	4.23748	0.47479	4.24407
H	3.59730	0.67112	5.13055
H	4.70527	1.43308	3.93693
H	5.04083	-0.23475	4.53610
C	2.71511	-1.40651	3.53413
H	2.18882	-1.87618	2.67869
H	1.97251	-1.20132	4.33422
H	3.45284	-2.13240	3.93523
C	6.99125	-2.62896	0.54398
H	7.46484	-2.68404	-0.46215
C	6.31561	-3.98276	0.79526
H	5.48895	-4.13985	0.07084
H	5.90391	-4.04251	1.82485
H	7.05189	-4.80407	0.66339
C	8.11472	-2.38110	1.55647
H	8.59524	-1.39914	1.35801
H	8.89012	-3.17143	1.46520
H	7.72570	-2.38639	2.59680
C	-7.07438	-1.39658	-0.20586
H	-7.29638	-2.04293	-1.08489
C	-6.89637	-2.32654	0.99682
H	-6.06877	-3.03713	0.79527
H	-7.82183	-2.91771	1.16429
H	-6.66849	-1.75343	1.92061
C	-8.28483	-0.47490	-0.01526
H	-8.38458	0.20748	-0.88642
H	-8.18660	0.13318	0.90875
H	-9.21409	-1.07912	0.05948
C	-1.89798	-2.02316	2.94392
C	-2.02538	-3.56707	1.03818
C	-1.16202	-1.04947	3.64180
C	-3.17231	-2.41752	3.41427
C	-3.35341	-3.93145	1.50460
C	-1.68836	-0.49201	4.79932
H	-0.20440	-0.73018	3.24601
C	-3.68037	-1.83318	4.58638
C	-3.88073	-3.39833	2.63182
H	-3.90122	-4.66885	0.93406

C	-2.94785	-0.88054	5.28170
H	-1.11527	0.26166	5.33169
H	-4.66193	-2.13924	4.93929
H	-4.86550	-3.71282	2.96769
H	-3.34787	-0.43493	6.18668
C	-1.40643	-4.14490	-0.04281
C	0.01861	-4.01935	-0.45610
H	0.48557	-5.01204	-0.51428
H	0.57709	-3.42627	0.26876
N	0.17564	-3.35620	-1.80367
N	-1.38434	-2.59491	1.79726
C	1.45806	-3.51227	-2.43843
C	1.53442	-4.03533	-3.73306
C	2.62327	-3.08627	-1.78879
C	2.76568	-4.13013	-4.38013
H	0.62776	-4.36488	-4.23488
C	3.85099	-3.19960	-2.43999
H	2.56782	-2.65264	-0.79492
C	3.93011	-3.71632	-3.73364
H	2.81174	-4.53391	-5.38712
H	4.75407	-2.87227	-1.93816
H	4.89100	-3.79266	-4.23277
H	-0.54291	-3.74754	-2.41171
H	-0.53666	-2.15214	1.43084
Cl	-2.31347	-5.30258	-1.05326
H	-0.05530	-1.88324	-1.70937

Catalyst (TRIP, 3)-Enamine protonation TS (pro-R) lowest free energy conformation
Electronic energy (Hartrees): -1870.535033
Free Energy at 1M concentration and 253 K (Hartrees): -1869.315971
Coordinates:

P	0.01882	0.13568	-0.06621
O	-1.29314	0.85455	0.58745
O	0.98120	1.30814	-0.65479
O	-0.40448	-0.59466	-1.37316
O	0.63506	-0.69639	1.01391
C	-1.82723	1.88225	-0.14790
C	-3.22223	1.75701	-0.64177
C	-3.78402	2.77054	-1.31617
H	-4.80732	2.69176	-1.66420
C	-3.01312	3.99484	-1.60722
C	-3.61785	5.06240	-2.43695
H	-4.63422	4.96164	-2.79699
C	-2.91040	6.15352	-2.74844
H	-3.35226	6.93222	-3.35661
C	-1.51965	6.29249	-2.27671
H	-0.94397	7.16623	-2.55377
C	-0.96630	5.33654	-1.52157
H	0.06374	5.44800	-1.21057
C	-1.75166	4.13140	-1.13735
C	-1.16016	3.05214	-0.28897
C	1.15763	2.38098	0.17996
C	2.52147	2.67768	0.68466
C	2.73892	3.78522	1.40945
H	3.73805	4.02229	1.75516
C	1.61479	4.67007	1.77517
C	1.85256	5.82806	2.66746
H	2.85322	6.04803	3.01829
C	0.83226	6.60480	3.04693

H	1.00658	7.44966	3.70062
C	-0.53704	6.30403	2.58751
H	-1.36168	6.92247	2.91776
C	-0.76177	5.26359	1.77676
H	-1.77726	5.04720	1.47293
C	0.36745	4.40859	1.31914
C	0.14585	3.24977	0.40161
C	3.64783	1.77729	0.33757
C	4.16885	1.76925	-1.07386
C	4.21219	0.96132	1.27368
C	5.16749	0.92607	-1.39649
C	5.33777	0.07637	0.87333
C	5.78025	0.03344	-0.39085
H	5.55939	0.91075	-2.40437
H	5.81012	-0.54412	1.62228
C	3.75423	0.92718	2.73379
H	2.83359	1.52683	2.88308
C	3.39736	-0.49446	3.18962
H	2.67237	-0.95181	2.48892
H	2.92806	-0.46188	4.19612
H	4.29520	-1.14361	3.25041
C	4.82958	1.52955	3.64496
H	5.76082	0.92466	3.61820
H	4.46302	1.56960	4.69297
H	5.06779	2.56507	3.32212
C	3.62666	2.72494	-2.13694
H	2.81253	3.35145	-1.72301
C	3.02491	1.96235	-3.32600
H	2.27234	1.22707	-2.97163
H	3.80821	1.42177	-3.89778
H	2.51785	2.67245	-4.01365
C	4.71029	3.70516	-2.60468
H	5.53245	3.17780	-3.13291
H	5.13319	4.24710	-1.73171
H	4.27150	4.45411	-3.29818
C	-4.01326	0.53058	-0.37310
C	-4.55166	0.29746	0.85693
C	-4.32568	-0.39941	-1.51140
C	-5.48431	-0.84869	1.03058
C	-5.23303	-1.37636	-1.32798
C	-5.85094	-1.61484	-0.00651
H	-5.91851	-1.03042	2.00513
H	-5.50111	-2.02750	-2.14873
C	-4.29996	1.20525	2.06146
H	-3.62460	2.04592	1.80792
C	-5.60327	1.85101	2.54765
H	-6.09531	2.38888	1.70906
H	-5.38530	2.58734	3.35062
H	-6.30682	1.09323	2.95152
C	-3.61308	0.43139	3.19319
H	-2.66905	-0.02140	2.82373
H	-4.26620	-0.37491	3.58814
H	-3.36151	1.12028	4.02758
C	-3.62730	-0.26633	-2.86230
H	-2.81313	0.48481	-2.79740
C	-4.60339	0.21972	-3.93998
H	-4.06417	0.37625	-4.89857
H	-5.05812	1.18610	-3.63703
H	-5.41545	-0.51970	-4.10631
C	-2.95742	-1.58293	-3.28740

H	-2.37935	-2.01120	-2.44341
H	-2.26066	-1.39862	-4.13279
H	-3.70764	-2.33385	-3.61329
C	-6.89652	-2.70169	0.16954
H	-7.22808	-2.74149	1.23177
C	-6.32590	-4.08168	-0.17186
H	-5.42699	-4.27687	0.44436
H	-6.04873	-4.15283	-1.24469
H	-7.07612	-4.86985	0.05168
C	-8.13868	-2.40003	-0.67633
H	-8.54336	-1.39904	-0.41395
H	-8.92787	-3.15610	-0.47691
H	-7.89886	-2.41707	-1.76083
C	6.91606	-0.89612	-0.77887
H	7.21155	-1.51949	0.09469
C	6.48541	-1.85983	-1.89188
H	5.54760	-2.38046	-1.60844
H	7.27247	-2.62671	-2.05471
H	6.31564	-1.32086	-2.84779
C	8.15450	-0.09619	-1.19692
H	8.45943	0.58809	-0.37638
H	7.95478	0.50491	-2.10937
H	8.99990	-0.78603	-1.40577
H	0.02498	-2.33505	1.01654
C	-1.53800	-3.39994	1.81069
C	-0.62559	-3.65438	-0.42915
C	-1.47570	-2.72857	3.04642
C	-2.56283	-4.34156	1.55139
C	-1.68344	-4.58313	-0.71218
C	-2.42489	-3.00886	4.01324
H	-0.70015	-1.98726	3.20993
C	-3.50192	-4.61909	2.56779
C	-2.58982	-4.92720	0.24632
H	-1.72122	-5.01987	-1.70011
C	-3.43779	-3.96072	3.78182
H	-2.38739	-2.48437	4.96301
H	-4.28041	-5.35260	2.37828
H	-3.35816	-5.66155	0.01933
H	-4.16795	-4.17220	4.55603
N	-0.60592	-3.13467	0.83234
C	0.32397	-3.15414	-1.37079
C	1.78240	-2.98266	-0.97958
H	1.82676	-2.45319	-0.02768
H	2.27297	-2.32514	-1.71426
N	2.46928	-4.26914	-0.84873
H	2.35903	-4.83128	-1.68450
C	3.75912	-4.35225	-0.31545
C	4.62715	-5.36809	-0.76192
C	4.21376	-3.49207	0.70146
C	5.89297	-5.52293	-0.20658
H	4.29641	-6.03721	-1.55328
C	5.48255	-3.66097	1.25309
H	3.57890	-2.69360	1.06587
C	6.33547	-4.67029	0.80724
H	6.54068	-6.31360	-0.57566
H	5.80735	-2.98939	2.04267
H	7.32472	-4.78860	1.23767
H	-0.13976	-1.66327	-1.43977
Cl	0.18736	-3.86205	-3.03511

Catalyst (TRIP, 3)-Enamine protonation TS (pro-R) alternative conformation
Electronic energy (Hartrees): -1870.534679
Free Energy at 1M concentration and 253 K (Hartrees): -1869.315401
Coordinates:

P	0.01708	0.13769	-0.10407
O	-1.31871	0.82618	0.53331
O	0.97656	1.33472	-0.64560
O	-0.36693	-0.57011	-1.43553
O	0.62066	-0.70859	0.97198
C	-1.84601	1.86629	-0.18956
C	-3.22619	1.73773	-0.72320
C	-3.78465	2.76395	-1.38097
H	-4.79730	2.68211	-1.75827
C	-3.02293	4.00551	-1.61757
C	-3.62133	5.08913	-2.43095
H	-4.62796	4.98706	-2.81709
C	-2.92005	6.19650	-2.69550
H	-3.35723	6.98697	-3.29176
C	-1.54205	6.33767	-2.18836
H	-0.97054	7.22514	-2.42787
C	-0.99467	5.36711	-1.44763
H	0.02682	5.48124	-1.11051
C	-1.77428	4.14297	-1.11496
C	-1.18979	3.04724	-0.28287
C	1.12406	2.38762	0.21968
C	2.47337	2.68435	0.76214
C	2.66353	3.77543	1.51890
H	3.65233	4.01338	1.89254
C	1.52248	4.63927	1.88248
C	1.72876	5.77630	2.80892
H	2.71935	5.99752	3.18655
C	0.69227	6.53241	3.18621
H	0.84356	7.36192	3.86483
C	-0.66370	6.22935	2.69019
H	-1.50183	6.83050	3.01844
C	-0.85994	5.20785	1.84855
H	-1.86638	4.98879	1.51767
C	0.28795	4.37674	1.39338
C	0.09847	3.23997	0.44172
C	3.61594	1.80297	0.41906
C	4.16756	1.83282	-0.98044
C	4.16792	0.97059	1.34819
C	5.18089	1.00644	-1.30068
C	5.31007	0.10523	0.95194
C	5.78009	0.09589	-0.30297
H	5.59479	1.01843	-2.29978
H	5.77193	-0.52855	1.69631
C	3.67970	0.89888	2.79716
H	2.75011	1.48608	2.94029
C	3.32763	-0.53600	3.21356
H	2.62232	-0.98455	2.48762
H	2.83677	-0.53051	4.21025
H	4.23048	-1.17766	3.27925
C	4.72963	1.49070	3.74423
H	5.66721	0.89543	3.72363
H	4.34077	1.50354	4.78488
H	4.96431	2.53546	3.44985
C	3.63999	2.80875	-2.03245
H	2.81097	3.41784	-1.62209
C	3.07209	2.06951	-3.25253

H	2.31918	1.31889	-2.93276
H	3.87307	1.55022	-3.81954
H	2.57336	2.79127	-3.93411
C	4.72439	3.80956	-2.45237
H	5.56311	3.30237	-2.97429
H	5.12255	4.33432	-1.55753
H	4.29404	4.57087	-3.13768
C	-4.00459	0.49076	-0.51947
C	-4.56898	0.20361	0.68717
C	-4.27478	-0.40082	-1.69911
C	-5.49082	-0.95874	0.79299
C	-5.17507	-1.39419	-1.57627
C	-5.82601	-1.68557	-0.28417
H	-5.94318	-1.18548	1.74874
H	-5.40868	-2.02472	-2.42435
C	-4.35774	1.06529	1.93235
H	-3.68761	1.92348	1.72859
C	-5.68076	1.67481	2.41226
H	-6.15952	2.24049	1.58434
H	-5.49177	2.38024	3.24959
H	-6.38397	0.89270	2.76756
C	-3.68812	0.25425	3.04855
H	-2.73105	-0.17429	2.68364
H	-4.34087	-0.57383	3.39635
H	-3.46327	0.91215	3.91497
C	-3.53824	-0.21286	-3.02302
H	-2.73486	0.54379	-2.90708
C	-4.48718	0.30123	-4.11186
H	-3.92156	0.49647	-5.04789
H	-4.96110	1.25192	-3.78942
H	-5.28592	-0.43984	-4.32792
C	-2.84109	-1.50647	-3.47409
H	-2.28690	-1.96117	-2.62780
H	-2.11880	-1.28425	-4.28841
H	-3.57245	-2.25125	-3.85272
C	-6.85535	-2.79867	-0.20285
H	-7.00114	-3.23380	-1.21649
C	-8.22021	-2.26570	0.25171
H	-8.54343	-1.43358	-0.40983
H	-8.18017	-1.89801	1.29897
H	-8.98189	-3.07219	0.19153
C	-6.37635	-3.93562	0.70422
H	-5.41549	-4.33573	0.32078
H	-7.11791	-4.76269	0.70467
H	-6.23475	-3.58596	1.74892
C	6.93242	-0.81444	-0.68779
H	7.21484	-1.45547	0.17727
C	6.53442	-1.75551	-1.83188
H	5.59515	-2.29006	-1.58103
H	7.33139	-2.51201	-1.99505
H	6.38076	-1.19589	-2.77861
C	8.17218	0.00569	-1.06036
H	8.45326	0.67320	-0.21777
H	7.98657	0.62620	-1.96273
H	9.02811	-0.67179	-1.26685
H	0.04286	-2.35673	0.94141
C	-1.49585	-3.47304	1.71321
C	-0.58022	-3.65750	-0.53256
C	-1.44492	-2.83205	2.96550
C	-2.50026	-4.43053	1.43305

C	-1.62527	-4.59345	-0.83930
C	-2.38242	-3.16088	3.92852
H	-0.68697	-2.07653	3.14553
C	-3.42500	-4.76021	2.44664
C	-2.52354	-4.97813	0.11157
H	-1.65868	-5.00375	-1.83868
C	-3.37139	-4.13272	3.67738
H	-2.35358	-2.66025	4.89138
H	-4.18519	-5.50830	2.24058
H	-3.28253	-5.71643	-0.13347
H	-4.09161	-4.38347	4.44914
N	-0.57236	-3.16423	0.73986
C	0.36939	-3.12877	-1.45774
C	1.82248	-2.94646	-1.05129
H	1.85332	-2.42891	-0.09231
H	2.31166	-2.27403	-1.77318
N	2.52309	-4.22695	-0.93171
H	2.43695	-4.77252	-1.78122
C	3.80376	-4.30460	-0.37537
C	4.70007	-5.28837	-0.83715
C	4.22081	-3.47144	0.67948
C	5.95703	-5.43865	-0.26050
H	4.39890	-5.93591	-1.65774
C	5.48106	-3.63499	1.25158
H	3.56242	-2.69908	1.05812
C	6.36225	-4.61268	0.79018
H	6.62727	-6.20420	-0.64211
H	5.77650	-2.98482	2.07010
H	7.34459	-4.72709	1.23717
H	-0.10324	-1.63634	-1.50985
Cl	0.25567	-3.80946	-3.13497

Catalyst (TRIP, 3)-Enamine protonation TS (pro-R) alternative conformation
Electronic energy (Hartrees): -1870.531982
Free Energy at 1M concentration and 253 K (Hartrees): -1869.313961
Coordinates:

P	0.06408	-0.06939	0.06231
O	1.22694	0.90963	-0.51587
O	-1.26658	0.83602	0.34237
O	0.39743	-0.75181	1.34831
O	-0.18923	-0.97746	-1.18390
C	1.38974	2.06395	0.20543
C	2.66240	2.28812	0.93519
C	2.85039	3.42925	1.61338
H	3.78511	3.60647	2.13229
C	1.78395	4.44857	1.67200
C	1.97456	5.66322	2.49782
H	2.90576	5.81999	3.02797
C	0.99644	6.56984	2.59549
H	1.13574	7.45618	3.20086
C	-0.28158	6.35784	1.88980
H	-1.07803	7.08354	1.99277
C	-0.46171	5.26682	1.13647
H	-1.41662	5.12023	0.64984
C	0.62883	4.26390	0.99138
C	0.45593	3.03912	0.15261
C	-1.54146	1.80601	-0.58923
C	-2.84911	1.76897	-1.29204
C	-3.16950	2.74172	-2.15820
H	-4.13373	2.73486	-2.65275

C	-2.21477	3.82810	-2.45070
C	-2.54466	4.83846	-3.48226
H	-3.49451	4.79656	-4.00071
C	-1.66978	5.80498	-3.78022
H	-1.91067	6.54188	-4.53548
C	-0.36753	5.86238	-3.08930
H	0.34510	6.63392	-3.35092
C	-0.06258	4.95770	-2.15180
H	0.90744	5.00522	-1.67507
C	-1.03779	3.89360	-1.78751
C	-0.72584	2.87674	-0.73824
C	-3.83733	0.70459	-0.99024
C	-4.64016	0.78396	0.27866
C	-4.08473	-0.29204	-1.88518
C	-5.63416	-0.10288	0.47832
C	-5.22448	-1.21473	-1.64298
C	-5.97525	-1.12230	-0.53718
H	-6.21971	-0.06541	1.38682
H	-5.46907	-1.95585	-2.39277
C	-3.24912	-0.49425	-3.14799
H	-2.34968	0.15654	-3.13907
C	-2.72505	-1.93453	-3.24192
H	-2.28885	-2.24756	-2.27196
H	-1.93548	-2.00163	-4.02056
H	-3.53461	-2.64652	-3.50688
C	-4.05694	-0.12954	-4.39831
H	-4.94065	-0.79280	-4.51143
H	-3.42116	-0.22823	-5.30398
H	-4.40703	0.92200	-4.33459
C	-4.36021	1.84516	1.34182
H	-3.54271	2.51821	1.01916
C	-3.89650	1.20323	2.65601
H	-3.00998	0.56067	2.47152
H	-4.69901	0.58385	3.10933
H	-3.60641	1.99124	3.38324
C	-5.57897	2.74975	1.56717
H	-6.42505	2.18740	2.01498
H	-5.90880	3.18883	0.60125
H	-5.31163	3.58219	2.25273
C	3.74134	1.27331	0.89045
C	4.53443	1.14549	-0.20963
C	4.01895	0.44416	2.11316
C	5.67984	0.19900	-0.16553
C	5.11265	-0.34061	2.13556
C	5.98610	-0.47617	0.95304
H	6.30230	0.08598	-1.04263
H	5.35118	-0.91607	3.02050
C	4.34066	1.98072	-1.47615
H	3.48662	2.68041	-1.38005
C	5.56867	2.85765	-1.75029
H	5.79191	3.48546	-0.86111
H	5.36862	3.53309	-2.60947
H	6.46161	2.24322	-1.99035
C	4.02514	1.08460	-2.67905
H	3.12777	0.46646	-2.46509
H	4.87643	0.41317	-2.91826
H	3.80841	1.70879	-3.57210
C	3.09034	0.47697	3.32527
H	2.16335	1.03483	3.07893
C	3.75359	1.20322	4.50109

H	3.04349	1.27709	5.35226
H	4.04291	2.23234	4.20153
H	4.66220	0.66260	4.84194
C	2.63815	-0.93259	3.74225
H	2.31703	-1.51742	2.85581
H	1.78056	-0.86224	4.44514
H	3.45565	-1.48732	4.24912
C	7.20084	-1.38354	1.02456
H	7.27409	-1.81347	2.04812
C	8.49797	-0.60348	0.77591
H	8.56792	0.25284	1.48037
H	8.54258	-0.21783	-0.26463
H	9.37569	-1.26345	0.94386
C	7.06967	-2.56193	0.05527
H	6.13739	-3.12186	0.27569
H	7.92787	-3.25631	0.17836
H	7.04393	-2.21439	-0.99908
C	-7.18044	-2.02633	-0.34812
H	-7.29644	-2.69517	-1.23076
C	-7.00287	-2.93015	0.87452
H	-6.09127	-3.54822	0.74870
H	-7.87172	-3.61521	0.97316
H	-6.91278	-2.33545	1.80808
C	-8.47000	-1.20401	-0.24048
H	-8.57672	-0.54566	-1.12915
H	-8.47077	-0.57507	0.67490
H	-9.35026	-1.88073	-0.20304
H	-0.41078	-2.25577	1.57097
C	-2.16707	-2.92951	2.38923
C	-1.15480	-3.71955	0.31984
C	-2.08233	-2.06239	3.49479
C	-3.31005	-3.74207	2.19653
C	-2.33154	-4.51605	0.09819
C	-3.12717	-2.02288	4.40178
H	-1.21165	-1.42378	3.60378
C	-4.34649	-3.69347	3.15216
C	-3.34341	-4.54304	1.01020
H	-2.37254	-5.11497	-0.80055
C	-4.26055	-2.84345	4.23978
H	-3.07001	-1.34665	5.24918
H	-5.21708	-4.32865	3.01564
H	-4.20294	-5.18544	0.83826
H	-5.06543	-2.80420	4.96630
C	-0.07043	-3.57777	-0.59210
H	-0.23315	-2.05433	-0.97895
C	1.37615	-3.58492	-0.08396
H	1.70037	-4.62530	0.02284
H	1.39358	-3.13624	0.91697
N	2.30161	-2.90306	-0.96459
N	-1.13814	-2.98653	1.47482
C	3.44167	-3.45693	-1.52556
C	4.06037	-4.62082	-1.02946
C	4.03790	-2.81232	-2.62998
C	5.21057	-5.12370	-1.63739
H	3.66175	-5.12714	-0.15743
C	5.19328	-3.31523	-3.21446
H	3.57053	-1.91802	-3.03293
C	5.79170	-4.48198	-2.73005
H	5.66406	-6.02519	-1.23371
H	5.62564	-2.79268	-4.06365

H	6.69249	-4.87541	-3.18995
H	1.93312	-2.09127	-1.43863
Cl	-0.21663	-4.53386	-2.11511

Catalyst (TRIP, 3)-Enamine protonation TS (pro-R) alternative conformation
Electronic energy (Hartrees): -1870.532589
Free Energy at 1M concentration and 253 K (Hartrees): -1869.313953
Coordinates:

P	0.10799	-0.07554	-0.14371
O	1.21087	0.97818	-0.70332
O	-1.21474	0.77604	0.30275
O	0.55524	-0.85353	1.04996
O	-0.22637	-0.88608	-1.43772
C	1.36884	2.08950	0.08533
C	2.64958	2.29195	0.80955
C	2.85319	3.42141	1.50252
H	3.79492	3.58452	2.01274
C	1.78304	4.43081	1.61864
C	1.99115	5.62376	2.47153
H	2.94245	5.78067	2.96473
C	1.00413	6.50978	2.64038
H	1.15625	7.38001	3.26573
C	-0.30150	6.29483	1.98870
H	-1.10526	7.00114	2.15206
C	-0.49778	5.22351	1.21155
H	-1.47334	5.07286	0.76966
C	0.60245	4.24613	0.98354
C	0.41538	3.04683	0.11005
C	-1.59067	1.78510	-0.55030
C	-2.95691	1.75671	-1.13320
C	-3.36586	2.76011	-1.92464
H	-4.37116	2.75997	-2.32914
C	-2.45463	3.87258	-2.25505
C	-2.88546	4.91724	-3.21256
H	-3.87494	4.87870	-3.65100
C	-2.05328	5.91098	-3.54208
H	-2.36797	6.67314	-4.24320
C	-0.69858	5.96500	-2.95996
H	-0.02260	6.76020	-3.24676
C	-0.30227	5.02895	-2.08979
H	0.70271	5.07541	-1.69154
C	-1.22708	3.93305	-1.69122
C	-0.81308	2.88198	-0.71419
C	-3.90459	0.66941	-0.78490
C	-4.56613	0.67268	0.56604
C	-4.24803	-0.27692	-1.70267
C	-5.53073	-0.23189	0.82230
C	-5.34979	-1.22380	-1.38808
C	-5.97675	-1.19781	-0.20444
H	-6.01585	-0.24796	1.78876
H	-5.67153	-1.92613	-2.14605
C	-3.55819	-0.40011	-3.06029
H	-2.67650	0.27134	-3.12364
C	-3.01956	-1.82027	-3.27848
H	-2.45644	-2.15367	-2.38400
H	-2.33398	-1.83726	-4.15249
H	-3.84029	-2.54393	-3.46611
C	-4.50960	-0.00093	-4.19379
H	-5.38357	-0.68446	-4.24321
H	-3.97651	-0.03693	-5.16781

H	-4.87651	1.03525	-4.03859
C	-4.17942	1.67828	1.64979
H	-3.40437	2.37628	1.27987
C	-3.57682	0.97139	2.87133
H	-2.71522	0.34391	2.56024
H	-4.32589	0.32458	3.37503
H	-3.21067	1.72227	3.60352
C	-5.37102	2.55780	2.05127
H	-6.16262	1.96446	2.55519
H	-5.80327	3.04445	1.15076
H	-5.03567	3.35514	2.74857
C	3.70145	1.24873	0.77777
C	4.48022	1.07423	-0.32584
C	3.96788	0.43991	2.01800
C	5.55687	0.04817	-0.29298
C	5.02376	-0.39361	2.03766
C	5.83946	-0.63175	0.82806
H	6.15447	-0.11669	-1.18038
H	5.26837	-0.93461	2.94104
C	4.35198	1.94807	-1.57512
H	3.53568	2.69114	-1.47379
C	5.63151	2.76160	-1.80442
H	5.86437	3.36393	-0.90018
H	5.49131	3.45860	-2.65824
H	6.49598	2.10188	-2.02898
C	4.01071	1.10695	-2.80847
H	3.10288	0.49806	-2.61366
H	4.85058	0.43466	-3.08026
H	3.80190	1.77045	-3.67466
C	3.08419	0.56066	3.25922
H	2.17576	1.15237	3.02389
C	3.82223	1.29331	4.38614
H	3.14426	1.43708	5.25441
H	4.15763	2.29311	4.04051
H	4.71089	0.71703	4.72110
C	2.58075	-0.80899	3.74560
H	2.19055	-1.40686	2.89737
H	1.76042	-0.67051	4.48204
H	3.39093	-1.38809	4.23676
C	6.99753	-1.61280	0.86192
H	7.35289	-1.80893	-0.17508
C	6.58102	-2.96547	1.45727
H	5.61810	-3.30103	1.02243
H	6.46330	-2.90392	2.55973
H	7.35473	-3.73059	1.23367
C	8.17331	-1.01753	1.64238
H	8.49572	-0.06291	1.17397
H	9.03449	-1.71930	1.62947
H	7.88880	-0.81959	2.69823
C	-7.15428	-2.11853	0.06282
H	-7.36027	-2.74322	-0.83562
C	-6.85030	-3.08106	1.21369
H	-5.95560	-3.68563	0.96409
H	-7.70319	-3.77625	1.36616
H	-6.66578	-2.53351	2.16206
C	-8.42668	-1.31086	0.34461
H	-8.62643	-0.61054	-0.49456
H	-8.33258	-0.72737	1.28494
H	-9.29736	-1.99424	0.44015
H	-0.18260	-2.38004	1.21687

C	-1.83583	-3.10969	2.18325
C	-1.08175	-3.74704	-0.04172
C	-1.61323	-2.32224	3.32893
C	-3.00233	-3.90341	2.06910
C	-2.28255	-4.52586	-0.17880
C	-2.54557	-2.34327	4.35138
H	-0.72977	-1.69427	3.37864
C	-3.92094	-3.91869	3.13987
C	-3.18045	-4.61856	0.84225
H	-2.43495	-5.05837	-1.10668
C	-3.69940	-3.14733	4.26600
H	-2.38357	-1.72808	5.23111
H	-4.80831	-4.54032	3.06227
H	-4.05806	-5.24909	0.72756
H	-4.41406	-3.15640	5.08235
C	-0.11488	-3.53548	-1.06940
H	-0.27956	-1.97886	-1.31351
C	1.37966	-3.59734	-0.74948
H	1.69242	-4.64960	-0.72428
H	1.54594	-3.18520	0.25799
N	2.16446	-2.91730	-1.75395
N	-0.92051	-3.10508	1.15365
C	3.50276	-3.15272	-2.00877
C	4.26472	-4.10517	-1.30579
C	4.13818	-2.42702	-3.03880
C	5.60677	-4.31203	-1.62837
H	3.82170	-4.67315	-0.49534
C	5.48049	-2.62643	-3.33127
H	3.55376	-1.71681	-3.61738
C	6.23341	-3.57286	-2.62911
H	6.17129	-5.05251	-1.06758
H	5.94094	-2.04220	-4.12360
H	7.28214	-3.72950	-2.86005
H	1.77233	-2.06598	-2.12860
Cl	-0.46213	-4.39228	-2.62132

Catalyst (TRIP, 3)-Enamine protonation TS (pro-R) alternative conformation
Electronic energy (Hartrees): -1870.532148
Free Energy at 1M concentration and 253 K (Hartrees): -1869.313882
Coordinates:

P	0.05984	0.18318	-0.17235
O	-1.22648	0.84424	0.58461
O	0.97040	1.40055	-0.75065
O	-0.42168	-0.48732	-1.49259
O	0.74728	-0.69276	0.82695
C	-1.82008	1.89169	-0.07365
C	-3.23859	1.75988	-0.49398
C	-3.85499	2.78954	-1.09203
H	-4.89494	2.70554	-1.38513
C	-3.12307	4.03957	-1.37348
C	-3.79172	5.12821	-2.12291
H	-4.82475	5.02238	-2.43019
C	-3.12181	6.24486	-2.42681
H	-3.61042	7.03904	-2.97636
C	-1.70943	6.39155	-2.02722
H	-1.16531	7.28698	-2.29873
C	-1.09826	5.41643	-1.34466
H	-0.05447	5.53507	-1.08670
C	-1.84000	4.18146	-0.96853
C	-1.18349	3.08019	-0.19998

C	1.16801	2.43967	0.12149
C	2.55010	2.74469	0.56881
C	2.78449	3.82994	1.32153
H	3.79539	4.07300	1.62657
C	1.66449	4.67646	1.77872
C	1.92661	5.80484	2.70160
H	2.94012	6.03379	3.00683
C	0.91275	6.54345	3.16513
H	1.10498	7.36661	3.84110
C	-0.47303	6.22934	2.76840
H	-1.29103	6.81588	3.16639
C	-0.72009	5.21556	1.93084
H	-1.74597	4.98771	1.67395
C	0.39992	4.40437	1.38012
C	0.15273	3.27725	0.43004
C	3.67183	1.87462	0.13958
C	4.12033	1.91472	-1.29609
C	4.29177	1.03829	1.02111
C	5.10961	1.09281	-1.69438
C	5.39897	0.17277	0.53617
C	5.78084	0.17889	-0.74992
H	5.44995	1.10996	-2.72162
H	5.90309	-0.47910	1.23614
C	3.91360	0.95978	2.50203
H	3.00069	1.55000	2.71950
C	3.58645	-0.47589	2.93267
H	2.83093	-0.91493	2.25342
H	3.16663	-0.47642	3.96127
H	4.48983	-1.11955	2.92992
C	5.03515	1.53984	3.37095
H	5.96610	0.94166	3.27379
H	4.72698	1.54520	4.43833
H	5.25056	2.58629	3.06776
C	3.51652	2.89743	-2.29985
H	2.71594	3.49908	-1.82677
C	2.86734	2.16725	-3.48441
H	2.14373	1.40806	-3.12048
H	3.62886	1.65969	-4.11298
H	2.31695	2.89287	-4.12062
C	4.56502	3.90681	-2.78504
H	5.36776	3.40769	-3.36775
H	5.02179	4.42613	-1.91539
H	4.08524	4.67165	-3.43255
C	-3.99324	0.50901	-0.23368
C	-4.45712	0.21522	1.01360
C	-4.35898	-0.37573	-1.39195
C	-5.36397	-0.95113	1.18962
C	-5.24224	-1.37294	-1.20025
C	-5.78123	-1.67636	0.14228
H	-5.73985	-1.18053	2.17824
H	-5.54896	-1.99204	-2.03219
C	-4.15006	1.07554	2.23997
H	-3.50206	1.93709	1.98529
C	-5.43245	1.67893	2.82600
H	-5.97917	2.24299	2.04014
H	-5.17934	2.38478	3.64586
H	-6.10097	0.89356	3.23655
C	-3.38916	0.26615	3.29690
H	-2.46054	-0.15339	2.85610
H	-4.00673	-0.56779	3.69163

H	-3.10166	0.92292	4.14546
C	-3.74132	-0.17396	-2.77331
H	-2.93646	0.58822	-2.72276
C	-4.78472	0.33889	-3.77270
H	-4.30331	0.54563	-4.75232
H	-5.23707	1.28306	-3.40343
H	-5.59286	-0.40775	-3.92517
C	-3.07658	-1.45949	-3.29075
H	-2.44208	-1.90930	-2.50038
H	-2.43470	-1.22858	-4.16756
H	-3.83238	-2.21106	-3.60189
C	-6.80584	-2.78134	0.32884
H	-7.07559	-2.87156	1.40536
C	-6.24475	-4.13874	-0.10587
H	-5.31137	-4.35199	0.44989
H	-6.02778	-4.15917	-1.19456
H	-6.97507	-4.94375	0.12343
C	-8.09686	-2.45594	-0.43060
H	-8.49405	-1.47195	-0.10107
H	-8.86711	-3.22853	-0.22069
H	-7.91941	-2.42237	-1.52664
C	6.89678	-0.72001	-1.25100
H	7.04345	-0.54590	-2.34001
C	8.22841	-0.39397	-0.56348
H	8.46969	0.68278	-0.69327
H	9.04697	-0.99061	-1.01968
H	8.18784	-0.62498	0.52210
C	6.53516	-2.19970	-1.08182
H	5.56129	-2.41198	-1.57264
H	6.46541	-2.47037	-0.00813
H	7.31001	-2.83900	-1.55585
H	0.14840	-2.33624	0.78750
C	-1.35241	-3.45618	1.62331
C	-0.57301	-3.59558	-0.67781
C	-1.22380	-2.84123	2.88278
C	-2.38085	-4.39838	1.38188
C	-1.63671	-4.52488	-0.93927
C	-2.11003	-3.17833	3.89072
H	-0.44776	-2.09791	3.03457
C	-3.25375	-4.73492	2.43827
C	-2.47957	-4.92451	0.05440
H	-1.72844	-4.91736	-1.94213
C	-3.12434	-4.13205	3.67604
H	-2.02088	-2.69738	4.85992
H	-4.03383	-5.47002	2.26186
H	-3.25153	-5.65882	-0.15999
H	-3.80436	-4.38903	4.48152
N	-0.48197	-3.13554	0.60496
C	0.31648	-3.04858	-1.64729
C	1.79932	-2.87545	-1.33769
H	1.91094	-2.34135	-0.39503
H	2.23344	-2.21747	-2.10426
N	2.51525	-4.14582	-1.26083
H	2.61069	-4.59543	-2.16164
C	3.58625	-4.38427	-0.40075
C	4.57584	-5.31537	-0.77526
C	3.69466	-3.77951	0.86612
C	5.62173	-5.63425	0.08482
H	4.51585	-5.78725	-1.75354
C	4.75010	-4.10372	1.71595

H	2.94577	-3.07046	1.19846
C	5.72282	-5.03088	1.34008
H	6.36902	-6.35531	-0.23550
H	4.80678	-3.62459	2.68942
H	6.54244	-5.27527	2.00808
H	-0.16243	-1.54535	-1.62118
Cl	0.09678	-3.68736	-3.32857

Catalyst (TRIP, 3)-Enamine protonation TS (pro-R) alternative conformation
Electronic energy (Hartrees): -1870.532004
Free Energy at 1M concentration and 253 K (Hartrees): -1869.313681
Coordinates:

P	0.10180	-0.07833	-0.09604
O	1.21757	0.93656	-0.70158
O	-1.19362	0.81202	0.35594
O	0.55151	-0.83993	1.10751
O	-0.27275	-0.90958	-1.36559
C	1.41398	2.06573	0.05222
C	2.71641	2.26534	0.73716
C	2.95371	3.40541	1.40151
H	3.91086	3.56535	1.88336
C	1.90290	4.43471	1.51977
C	2.14978	5.64262	2.34065
H	3.11374	5.79331	2.81062
C	1.18233	6.55008	2.50911
H	1.36278	7.43130	3.11111
C	-0.13998	6.34456	1.88873
H	-0.92736	7.06919	2.05167
C	-0.37127	5.25976	1.14044
H	-1.35800	5.11730	0.72117
C	0.70627	4.25725	0.91338
C	0.47919	3.04138	0.07309
C	-1.56603	1.80773	-0.51396
C	-2.94517	1.79648	-1.06585
C	-3.34680	2.78500	-1.87938
H	-4.36073	2.79813	-2.26162
C	-2.41729	3.86606	-2.25965
C	-2.84357	4.89331	-3.23779
H	-3.84166	4.86438	-3.65702
C	-1.99674	5.85940	-3.60957
H	-2.30817	6.60882	-4.32571
C	-0.63072	5.90006	-3.05338
H	0.05642	6.67249	-3.37392
C	-0.23824	4.97976	-2.16487
H	0.77456	5.01551	-1.78579
C	-1.17836	3.91520	-1.72002
C	-0.76820	2.88180	-0.72281
C	-3.91368	0.75028	-0.65577
C	-4.54987	0.82854	0.70525
C	-4.29924	-0.22332	-1.52666
C	-5.53496	-0.03657	1.01606
C	-5.41855	-1.12606	-1.15220
C	-6.02400	-1.03135	0.04092
H	-6.00385	-0.00013	1.99082
H	-5.77395	-1.84864	-1.87400
C	-3.64277	-0.41816	-2.89219
H	-2.74539	0.22627	-3.00042
C	-3.14703	-1.85995	-3.06537
H	-2.57084	-2.17227	-2.17175
H	-2.48382	-1.93026	-3.95385

H	-3.99067	-2.56816	-3.20404
C	-4.60837	-0.03756	-4.02003
H	-5.50141	-0.69790	-4.02329
H	-4.09858	-0.12655	-5.00308
H	-4.94350	1.01379	-3.89818
C	-4.11394	1.86692	1.73838
H	-3.32790	2.52713	1.32475
C	-3.50506	1.19470	2.97600
H	-2.66474	0.53456	2.67432
H	-4.25966	0.58726	3.51887
H	-3.10684	1.96486	3.67049
C	-5.27237	2.79550	2.12604
H	-6.06863	2.24620	2.67081
H	-5.71041	3.25586	1.21457
H	-4.90068	3.61175	2.78182
C	3.75636	1.21101	0.69110
C	4.50328	1.01431	-0.43074
C	4.04949	0.41751	1.93482
C	5.57337	-0.01897	-0.41168
C	5.10029	-0.42276	1.93887
C	5.88213	-0.68379	0.71154
H	6.14571	-0.20110	-1.31234
H	5.36442	-0.95325	2.84305
C	4.34673	1.87006	-1.68945
H	3.54115	2.62308	-1.57591
C	5.62671	2.66687	-1.96968
H	5.89346	3.27929	-1.08172
H	5.46710	3.35342	-2.82861
H	6.47719	1.99515	-2.21159
C	3.96043	1.01325	-2.89853
H	3.04779	0.42374	-2.66956
H	4.78170	0.32178	-3.17944
H	3.74006	1.66484	-3.77090
C	3.19698	0.55964	3.19530
H	2.28797	1.15643	2.97519
C	3.96749	1.29896	4.29580
H	3.31183	1.45737	5.17861
H	4.30142	2.29245	3.93061
H	4.85983	0.71967	4.61554
C	2.69441	-0.80064	3.70815
H	2.27894	-1.40366	2.87544
H	1.89322	-0.64785	4.46271
H	3.51171	-1.38150	4.18533
C	7.03500	-1.67152	0.72861
H	7.35654	-1.88981	-0.31505
C	6.62970	-3.00994	1.36280
H	5.64815	-3.34371	0.97037
H	6.55369	-2.92829	2.46770
H	7.38800	-3.78593	1.12403
C	8.23796	-1.06860	1.46028
H	8.55167	-0.12582	0.96276
H	9.09404	-1.77635	1.43535
H	7.98708	-0.84736	2.52006
C	-7.22553	-1.89154	0.39075
H	-7.56804	-1.63714	1.41833
C	-8.40201	-1.61128	-0.55275
H	-8.64308	-0.52665	-0.54852
H	-9.30190	-2.16763	-0.21382
H	-8.16624	-1.92308	-1.59238
C	-6.86801	-3.38105	0.39681

H	-6.04443	-3.56136	1.11480
H	-6.55302	-3.72666	-0.61043
H	-7.74424	-3.98259	0.71985
H	-0.23561	-2.34050	1.31353
C	-1.91137	-2.99912	2.29241
C	-1.16759	-3.71683	0.08887
C	-1.67258	-2.18446	3.41551
C	-3.09769	-3.76542	2.19580
C	-2.38435	-4.47364	-0.02841
C	-2.61005	-2.14943	4.43289
H	-0.77227	-1.57977	3.45138
C	-4.02267	-3.72181	3.26046
C	-3.28700	-4.51533	0.99151
H	-2.54552	-5.03047	-0.94038
C	-3.78550	-2.92307	4.36419
H	-2.43552	-1.51302	5.29495
H	-4.92682	-4.32041	3.19681
H	-4.17656	-5.13157	0.89305
H	-4.50450	-2.88747	5.17603
C	-0.19838	-3.55086	-0.94488
H	-0.33818	-2.00002	-1.22053
C	1.29543	-3.63668	-0.62742
H	1.58649	-4.69436	-0.58618
H	1.47217	-3.21182	0.37281
N	2.09159	-2.98822	-1.64346
N	-0.99229	-3.04723	1.26696
C	3.41526	-3.27327	-1.92175
C	4.15412	-4.25497	-1.23428
C	4.05903	-2.56868	-2.96126
C	5.48091	-4.51180	-1.58304
H	3.70621	-4.80768	-0.41602
C	5.38727	-2.81723	-3.27895
H	3.49049	-1.83716	-3.52858
C	6.11669	-3.79374	-2.59342
H	6.02735	-5.27436	-1.03403
H	5.85460	-2.24800	-4.07813
H	7.15436	-3.98907	-2.84396
H	1.71900	-2.13214	-2.02700
Cl	-0.56851	-4.43220	-2.47739

Catalyst (TRIP, 3)-Enamine protonation TS (pro-R) alternative conformation
Electronic energy (Hartrees): -1870.531421
Free Energy at 1M concentration and 253 K (Hartrees): -1869.313598
Coordinates:

P	0.06461	-0.06315	0.09237
O	1.23434	0.88191	-0.52767
O	-1.24042	0.87436	0.38977
O	0.41254	-0.73303	1.38130
O	-0.23665	-0.98446	-1.13297
C	1.43136	2.05028	0.16187
C	2.72320	2.27063	0.85832
C	2.94512	3.42473	1.50352
H	3.89372	3.59831	1.99795
C	1.89802	4.46398	1.55850
C	2.12721	5.69540	2.34891
H	3.07206	5.84877	2.85538
C	1.16729	6.62155	2.44376
H	1.33480	7.52019	3.02330
C	-0.12892	6.41478	1.77046
H	-0.91030	7.15694	1.87150

C	-0.34392	5.30859	1.04908
H	-1.31143	5.16697	0.58649
C	0.72572	4.28292	0.90698
C	0.51375	3.04051	0.10376
C	-1.52319	1.82756	-0.55661
C	-2.85154	1.80203	-1.21982
C	-3.17384	2.75571	-2.10629
H	-4.15232	2.75825	-2.57212
C	-2.20379	3.81138	-2.45580
C	-2.53930	4.79931	-3.50706
H	-3.50260	4.76276	-4.00050
C	-1.65301	5.73891	-3.85361
H	-1.89807	6.45942	-4.62321
C	-0.33302	5.78881	-3.19652
H	0.38788	6.53823	-3.49691
C	-0.02270	4.90440	-2.24159
H	0.95942	4.94542	-1.78962
C	-1.00928	3.87088	-1.82410
C	-0.69086	2.87676	-0.75535
C	-3.85877	0.77822	-0.84932
C	-4.62208	0.92934	0.43765
C	-4.15924	-0.24424	-1.69726
C	-5.63367	0.07893	0.70035
C	-5.31454	-1.12505	-1.38575
C	-6.02972	-0.96880	-0.26179
H	-6.19321	0.16708	1.62257
H	-5.60285	-1.88451	-2.09952
C	-3.37024	-0.51565	-2.97673
H	-2.45498	0.11118	-3.02120
C	-2.88620	-1.97178	-3.03136
H	-2.42314	-2.25739	-2.06551
H	-2.12707	-2.09013	-3.83379
H	-3.72230	-2.67212	-3.23872
C	-4.20899	-0.17704	-4.21393
H	-5.11232	-0.82059	-4.27361
H	-3.60574	-0.32623	-5.13481
H	-4.53027	0.88506	-4.17925
C	-4.28277	2.02392	1.44861
H	-3.45822	2.66136	1.07548
C	-3.79584	1.42254	2.77334
H	-2.93028	0.75191	2.58917
H	-4.59914	0.84136	3.27338
H	-3.46602	2.23093	3.46019
C	-5.46996	2.96978	1.67281
H	-6.31605	2.44921	2.16849
H	-5.81788	3.37853	0.69995
H	-5.15995	3.82155	2.31533
C	3.78414	1.23715	0.81570
C	4.55380	1.07219	-0.29596
C	4.07289	0.43209	2.05170
C	5.68647	0.11050	-0.25129
C	5.15577	-0.36756	2.07206
C	6.00493	-0.54248	0.87706
H	6.29052	-0.03162	-1.13699
H	5.40276	-0.92611	2.96549
C	4.34730	1.88070	-1.57770
H	3.50537	2.59496	-1.48159
C	5.58217	2.73272	-1.89602
H	5.83199	3.37744	-1.02622
H	5.37491	3.39110	-2.76666

H	6.46122	2.09970	-2.13904
C	3.99520	0.96174	-2.75282
H	3.09285	0.36288	-2.50717
H	4.83153	0.27178	-2.99209
H	3.77067	1.56812	-3.65615
C	3.16737	0.50579	3.27931
H	2.24425	1.07195	3.03731
C	3.86265	1.24793	4.42634
H	3.16946	1.35137	5.28830
H	4.16135	2.26569	4.09843
H	4.76937	0.70146	4.76283
C	2.70232	-0.88723	3.73567
H	2.35514	-1.48592	2.86847
H	1.85978	-0.78846	4.45318
H	3.52116	-1.44342	4.23876
C	7.20896	-1.46408	0.94757
H	7.29589	-1.87084	1.97950
C	8.51115	-0.70746	0.65657
H	8.60543	0.16408	1.33920
H	8.54111	-0.34685	-0.39343
H	9.38328	-1.37500	0.82376
C	7.04437	-2.66297	0.00907
H	6.10911	-3.20488	0.25978
H	7.89557	-3.36580	0.13286
H	7.00358	-2.33988	-1.05254
C	-7.25973	-1.81388	0.01873
H	-7.69126	-1.51354	0.99930
C	-8.34643	-1.57796	-1.03762
H	-8.58607	-0.49489	-1.10151
H	-9.27352	-2.12217	-0.75739
H	-8.01811	-1.93294	-2.03755
C	-6.90863	-3.30148	0.12351
H	-6.14285	-3.44780	0.90987
H	-6.51845	-3.69487	-0.83885
H	-7.80936	-3.88697	0.40608
H	-0.43162	-2.21155	1.63359
C	-2.20040	-2.82451	2.47238
C	-1.21609	-3.68444	0.41808
C	-2.09272	-1.93011	3.55390
C	-3.36187	-3.61621	2.30430
C	-2.40730	-4.46637	0.22463
C	-3.13439	-1.84123	4.46094
H	-1.20729	-1.30911	3.64380
C	-4.39587	-3.51573	3.25888
C	-3.41449	-4.45060	1.14195
H	-2.46399	-5.08837	-0.65742
C	-4.28750	-2.63828	4.32248
H	-3.05967	-1.14360	5.28941
H	-5.28177	-4.13328	3.14139
H	-4.28484	-5.08386	0.99220
H	-5.08997	-2.55954	5.04849
C	-0.13988	-3.57748	-0.50899
H	-0.28602	-2.06035	-0.91451
C	1.31254	-3.60261	-0.01912
H	1.61993	-4.64683	0.10046
H	1.35160	-3.13778	0.97385
N	2.23694	-2.95174	-0.92429
N	-1.17626	-2.92736	1.55640
C	3.36200	-3.53427	-1.48652
C	3.97239	-4.69312	-0.96879

C	3.95069	-2.92652	-2.61554
C	5.10666	-5.22746	-1.57981
H	3.57982	-5.17094	-0.07809
C	5.09077	-3.46006	-3.20289
H	3.48913	-2.03700	-3.03539
C	5.68034	-4.62216	-2.69692
H	5.55401	-6.12425	-1.15918
H	5.51780	-2.96557	-4.07137
H	6.56908	-5.03961	-3.15908
H	1.87238	-2.14746	-1.41387
Cl	-0.32273	-4.55672	-2.01352

Catalyst (TRIP, 3)-Enamine protonation TS (pro-R) alternative conformation
Electronic energy (Hartrees): -1870.531781
Free Energy at 1M concentration and 253 K (Hartrees): -1869.313347
Coordinates:

P	0.05805	0.19225	-0.21287
O	-1.25476	0.81696	0.52901
O	0.96114	1.43619	-0.74378
O	-0.38393	-0.45654	-1.55769
O	0.73884	-0.69395	0.78175
C	-1.84693	1.87318	-0.11662
C	-3.25246	1.73281	-0.57643
C	-3.87027	2.77111	-1.15786
H	-4.90101	2.68024	-1.48002
C	-3.15106	4.03933	-1.38511
C	-3.81854	5.13975	-2.11821
H	-4.84292	5.02863	-2.45153
C	-3.15817	6.27370	-2.37527
H	-3.64588	7.07653	-2.91294
C	-1.75712	6.42807	-1.94034
H	-1.21994	7.33816	-2.17433
C	-1.14725	5.44222	-1.27219
H	-0.11108	5.56760	-0.98808
C	-1.87944	4.18704	-0.94758
C	-1.22463	3.07348	-0.19539
C	1.12742	2.45395	0.15961
C	2.49535	2.76190	0.64682
C	2.69991	3.82981	1.43236
H	3.70067	4.07594	1.76687
C	1.55994	4.65099	1.88642
C	1.78804	5.75742	2.84426
H	2.79182	5.99029	3.17739
C	0.75528	6.47116	3.30503
H	0.92263	7.27823	4.00653
C	-0.61764	6.15104	2.87024
H	-1.45129	6.71683	3.26580
C	-0.83395	5.15705	2.00104
H	-1.85116	4.92375	1.71612
C	0.30770	4.37449	1.45339
C	0.09520	3.27045	0.46845
C	3.63628	1.91395	0.22373
C	4.11808	1.99314	-1.19949
C	4.24475	1.06344	1.09980
C	5.12577	1.19169	-1.59335
C	5.37294	0.22192	0.62113
C	5.78488	0.26280	-0.65499
H	5.49012	1.23708	-2.61145
H	5.86794	-0.44083	1.31734
C	3.83307	0.94566	2.56921

H	2.90697	1.51820	2.77803
C	3.51640	-0.50383	2.95983
H	2.78539	-0.93885	2.25192
H	3.07034	-0.53313	3.97692
H	4.42907	-1.13424	2.96678
C	4.92577	1.52073	3.47735
H	5.86711	0.93769	3.38837
H	4.59286	1.49709	4.53702
H	5.13347	2.57672	3.20343
C	3.52727	2.99315	-2.19384
H	2.70879	3.57449	-1.72603
C	2.91503	2.28478	-3.41087
H	2.19188	1.50899	-3.08282
H	3.69716	1.80113	-4.03294
H	2.37152	3.01949	-4.04255
C	4.57550	4.02525	-2.62928
H	5.39765	3.54904	-3.20404
H	5.00530	4.52842	-1.73669
H	4.10269	4.80019	-3.26986
C	-3.99154	0.46094	-0.38068
C	-4.47898	0.11239	0.84318
C	-4.31452	-0.38605	-1.57970
C	-5.37226	-1.07176	0.95232
C	-5.18788	-1.40198	-1.44752
C	-5.75754	-1.75931	-0.13380
H	-5.76457	-1.34655	1.92186
H	-5.45935	-2.00138	-2.30681
C	-4.21201	0.92657	2.10943
H	-3.57131	1.80652	1.90407
C	-5.51607	1.48758	2.68984
H	-6.05314	2.07596	1.91526
H	-5.29192	2.16237	3.54356
H	-6.18223	0.67683	3.05220
C	-3.46323	0.08489	3.14998
H	-2.52044	-0.30638	2.71320
H	-4.07821	-0.77142	3.49828
H	-3.20198	0.71089	4.02969
C	-3.66095	-0.12762	-2.93488
H	-2.86776	0.64181	-2.83406
C	-4.68221	0.40834	-3.94492
H	-4.17621	0.65480	-4.90272
H	-5.15630	1.33377	-3.55611
H	-5.47651	-0.34163	-4.14603
C	-2.96519	-1.38557	-3.47872
H	-2.35204	-1.85962	-2.68556
H	-2.29772	-1.11464	-4.32442
H	-3.70204	-2.13188	-3.84336
C	-6.76376	-2.89251	-0.04142
H	-6.96008	-3.28850	-1.06250
C	-8.10845	-2.40187	0.51030
H	-8.48093	-1.54947	-0.09730
H	-8.01464	-2.07598	1.56786
H	-8.86021	-3.21825	0.45966
C	-6.21779	-4.05706	0.78951
H	-5.27481	-4.42520	0.33646
H	-6.94609	-4.89575	0.79732
H	-6.02214	-3.74779	1.83813
C	6.92229	-0.61203	-1.15036
H	7.09308	-0.41000	-2.23101
C	8.23347	-0.28909	-0.42320

H	8.46586	0.79299	-0.52088
H	9.06921	-0.86572	-0.87396
H	8.16963	-0.54720	0.65512
C	6.57280	-2.09903	-1.02561
H	5.61402	-2.30940	-1.54604
H	6.47885	-2.39627	0.03921
H	7.36627	-2.71871	-1.49489
H	0.17950	-2.34564	0.70998
C	-1.28744	-3.52348	1.52721
C	-0.51444	-3.59152	-0.77974
C	-1.16706	-2.93734	2.80117
C	-2.29277	-4.48564	1.26812
C	-1.56275	-4.53288	-1.06055
C	-2.03609	-3.32503	3.80599
H	-0.41072	-2.17699	2.96730
C	-3.14567	-4.87610	2.32193
C	-2.39162	-4.97540	-0.07314
H	-1.65312	-4.90019	-2.07304
C	-3.02379	-4.30243	3.57422
H	-1.95321	-2.86677	4.78667
H	-3.90559	-5.62849	2.13096
H	-3.15245	-5.71685	-0.30239
H	-3.68947	-4.60019	4.37761
N	-0.43143	-3.15648	0.51257
C	0.36848	-3.01314	-1.73628
C	1.84775	-2.81895	-1.41905
H	1.94822	-2.29615	-0.46876
H	2.27109	-2.14246	-2.17516
N	2.58891	-4.07500	-1.36008
H	2.71428	-4.50100	-2.26837
C	3.63370	-4.32572	-0.47275
C	4.64528	-5.23585	-0.84062
C	3.69635	-3.75354	0.81224
C	5.66876	-5.56477	0.04220
H	4.62045	-5.68259	-1.83214
C	4.73004	-4.08732	1.68482
H	2.92900	-3.06251	1.14030
C	5.72511	-4.99284	1.31493
H	6.43400	-6.26870	-0.27378
H	4.75143	-3.63319	2.67160
H	6.52736	-5.24493	2.00090
H	-0.12421	-1.51222	-1.69245
Cl	0.16653	-3.62687	-3.42890

Catalyst (TRIP, 3)-Enamine protonation TS (pro-R) alternative conformation
Electronic energy (Hartrees): -1870.531124
Free Energy at 1M concentration and 253 K (Hartrees): -1869.312946
Coordinates:

P	-0.04140	0.15611	-0.23024
O	0.84079	1.37682	-0.84374
O	-1.41639	0.80615	0.36198
O	0.63169	-0.55559	0.90222
O	-0.38161	-0.67306	-1.49703
C	0.87488	2.49803	-0.05596
C	2.15462	2.88814	0.58837
C	2.24727	4.06464	1.22418
H	3.18247	4.36200	1.68314
C	1.05750	4.92268	1.38721
C	1.15593	6.16277	2.19126
H	2.11071	6.47379	2.59680

C	0.06589	6.90074	2.42512
H	0.13840	7.80536	3.01494
C	-1.24304	6.47324	1.89697
H	-2.12683	7.05851	2.11600
C	-1.34190	5.35762	1.16500
H	-2.31830	5.04870	0.81781
C	-0.13107	4.54555	0.86054
C	-0.20769	3.30538	0.02733
C	-2.02615	1.71970	-0.46078
C	-3.39771	1.43206	-0.95189
C	-4.04572	2.34706	-1.68861
H	-5.05802	2.15779	-2.02477
C	-3.37919	3.60648	-2.07470
C	-4.06645	4.55231	-2.98405
H	-5.06906	4.33952	-3.33373
C	-3.45047	5.67117	-3.38051
H	-3.95254	6.36086	-4.04664
C	-2.07851	5.96215	-2.92256
H	-1.57689	6.85775	-3.26581
C	-1.45124	5.11681	-2.09653
H	-0.43687	5.33788	-1.79211
C	-2.13542	3.88385	-1.62024
C	-1.46333	2.93138	-0.68591
C	-4.07163	0.15636	-0.59775
C	-4.57187	-0.05313	0.80634
C	-4.28640	-0.80543	-1.54033
C	-5.25848	-1.17583	1.09410
C	-5.09822	-1.99853	-1.18561
C	-5.56583	-2.18208	0.05574
H	-5.61977	-1.34768	2.09888
H	-5.32399	-2.72774	-1.95276
C	-3.72566	-0.71705	-2.95954
H	-3.01343	0.12876	-3.05558
C	-2.92963	-1.97941	-3.32390
H	-2.24208	-2.25307	-2.49862
H	-2.32634	-1.79711	-4.23879
H	-3.60023	-2.84254	-3.51854
C	-4.85002	-0.47861	-3.97379
H	-5.56549	-1.32814	-3.98480
H	-4.42395	-0.36058	-4.99298
H	-5.40481	0.44895	-3.72182
C	-4.33248	0.97559	1.91070
H	-3.77342	1.84740	1.52056
C	-3.48310	0.38633	3.04653
H	-2.54238	-0.04211	2.64078
H	-4.03107	-0.41270	3.58903
H	-3.21541	1.18308	3.77285
C	-5.65581	1.53776	2.44646
H	-6.24838	0.75563	2.96602
H	-6.25910	1.94973	1.60925
H	-5.45500	2.35995	3.16626
C	3.30496	1.95039	0.60490
C	4.06886	1.74970	-0.50519
C	3.67168	1.26981	1.89758
C	5.26325	0.86812	-0.40947
C	4.79830	0.53620	1.95228
C	5.62722	0.30535	0.75142
H	5.86755	0.70448	-1.29188
H	5.10170	0.07743	2.88325
C	3.78986	2.43765	-1.84151

H	2.87898	3.06717	-1.79426
C	4.93583	3.38457	-2.21707
H	5.10054	4.12156	-1.40198
H	4.67952	3.94308	-3.14269
H	5.88004	2.82773	-2.39392
C	3.54388	1.40766	-2.95184
H	2.74897	0.69482	-2.64594
H	4.46648	0.83615	-3.18526
H	3.20975	1.92194	-3.87809
C	2.80325	1.40090	3.14936
H	1.83892	1.88671	2.89587
C	3.49110	2.28007	4.20086
H	2.81910	2.42614	5.07338
H	3.72641	3.27758	3.77554
H	4.43540	1.81351	4.55396
C	2.43842	0.03209	3.74855
H	2.08986	-0.66099	2.95743
H	1.62223	0.15060	4.49301
H	3.30721	-0.43196	4.26113
C	6.87320	-0.55771	0.83452
H	7.32697	-0.66699	-0.17574
C	6.53734	-1.96919	1.33278
H	5.70599	-2.39911	0.73744
H	6.23710	-1.95900	2.40175
H	7.42310	-2.63053	1.22271
C	7.92877	0.10017	1.72945
H	8.17296	1.11410	1.34625
H	8.86015	-0.50545	1.72721
H	7.56715	0.19011	2.77591
C	-6.41429	-3.39706	0.38562
H	-6.57396	-4.00965	-0.53041
C	-5.70667	-4.29548	1.40363
H	-4.72836	-4.62075	0.99398
H	-6.31586	-5.20291	1.60204
H	-5.53869	-3.76376	2.36419
C	-7.80133	-2.98018	0.88875
H	-8.29143	-2.31494	0.14595
H	-7.73402	-2.44650	1.86029
H	-8.44124	-3.87803	1.02509
H	0.33624	-2.20458	0.93470
C	-0.93541	-3.46202	1.93790
C	-0.20745	-3.71986	-0.36725
C	-0.88446	-2.69396	3.11745
C	-1.80662	-4.57361	1.83821
C	-1.11679	-4.82524	-0.48876
C	-1.68430	-3.05237	4.18784
H	-0.24172	-1.82097	3.15500
C	-2.59112	-4.92515	2.95784
C	-1.85914	-5.24401	0.57620
H	-1.17754	-5.32558	-1.44483
C	-2.53369	-4.17514	4.11750
H	-1.65717	-2.45565	5.09438
H	-3.25190	-5.78454	2.88650
H	-2.51801	-6.10175	0.46854
H	-3.14711	-4.44339	4.97134
C	0.54072	-3.13961	-1.44266
H	-0.06696	-1.75968	-1.51055
C	1.98410	-2.68066	-1.26594
H	2.05995	-2.08841	-0.34205
H	2.20696	-1.99511	-2.08634

N	2.94433	-3.76892	-1.30775
N	-0.15121	-3.12118	0.85762
C	4.30148	-3.58319	-1.54599
C	5.19496	-4.64108	-1.27768
C	4.82915	-2.39108	-2.07851
C	6.55380	-4.51245	-1.53980
H	4.80707	-5.56916	-0.86349
C	6.19254	-2.28181	-2.34881
H	4.18361	-1.54545	-2.27943
C	7.06948	-3.33191	-2.08180
H	7.21638	-5.34466	-1.31756
H	6.57036	-1.35098	-2.76320
H	8.13087	-3.23303	-2.28496
H	2.72216	-4.55322	-0.71096
Cl	0.30293	-3.93421	-3.04428

Catalyst (TRIP, 3)-Enamine protonation TS (pro-R) alternative conformation
Electronic energy (Hartrees): -1870.531379
Free Energy at 1M concentration and 253 K (Hartrees): -1869.310912
Coordinates:

P	-0.12231	0.08590	-0.03170
O	0.83720	1.27008	-0.59381
O	-1.53826	0.76266	0.39139
O	0.42274	-0.68107	1.11786
O	-0.37662	-0.71238	-1.36986
C	0.80890	2.42963	0.13607
C	2.03437	2.85360	0.85596
C	2.07115	4.04607	1.46726
H	2.96889	4.36555	1.98286
C	0.86996	4.90462	1.49992
C	0.89651	6.17181	2.26624
H	1.80833	6.49355	2.75396
C	-0.20618	6.92186	2.36353
H	-0.18636	7.84657	2.92569
C	-1.45833	6.48279	1.71915
H	-2.35416	7.08111	1.82445
C	-1.49189	5.34250	1.01999
H	-2.42895	5.02762	0.58110
C	-0.26417	4.51330	0.87206
C	-0.26979	3.24209	0.08475
C	-2.05254	1.65703	-0.51621
C	-3.36747	1.36473	-1.13911
C	-3.89547	2.22593	-2.02078
H	-4.87005	2.03329	-2.45371
C	-3.16138	3.44941	-2.40077
C	-3.71271	4.34178	-3.44639
H	-4.66207	4.11085	-3.91347
C	-3.03925	5.43491	-3.82052
H	-3.44186	6.08550	-4.58610
C	-1.73867	5.75099	-3.19988
H	-1.18809	6.62491	-3.52345
C	-1.23494	4.95476	-2.24979
H	-0.26877	5.19312	-1.82542
C	-1.98674	3.75151	-1.80002
C	-1.44826	2.84844	-0.73817
C	-4.14821	0.18295	-0.70229
C	-4.85264	0.22584	0.62547
C	-4.25975	-0.92028	-1.49369
C	-5.59156	-0.83323	1.00661
C	-5.12732	-2.04273	-1.04981

C	-5.75290	-2.01643	0.13481
H	-6.09966	-0.82388	1.96132
H	-5.25322	-2.89903	-1.69946
C	-3.52766	-1.05990	-2.82753
H	-2.78416	-0.24664	-2.96113
C	-2.73437	-2.37424	-2.89764
H	-2.17395	-2.53654	-1.95478
H	-2.00743	-2.33495	-3.73686
H	-3.40200	-3.24630	-3.06006
C	-4.51479	-0.96126	-3.99519
H	-5.25853	-1.78568	-3.95965
H	-3.96878	-1.01599	-4.96119
H	-5.05529	0.00801	-3.95711
C	-4.76012	1.44352	1.54524
H	-4.14280	2.23905	1.08438
C	-4.08066	1.08681	2.87404
H	-3.08962	0.62268	2.68353
H	-4.69878	0.37962	3.46634
H	-3.92198	2.00572	3.47797
C	-6.13952	2.07284	1.78110
H	-6.80468	1.39093	2.35149
H	-6.61832	2.31202	0.80736
H	-6.03222	3.01771	2.35573
C	3.20184	1.94277	0.92290
C	3.99607	1.74121	-0.16587
C	3.51788	1.24779	2.21991
C	5.13527	0.79134	-0.06163
C	4.60765	0.46146	2.28817
C	5.43455	0.18379	1.09512
H	5.74606	0.60372	-0.93516
H	4.88148	-0.00489	3.22433
C	3.80418	2.49549	-1.48284
H	2.92038	3.16371	-1.44606
C	5.00601	3.40489	-1.76443
H	5.15921	4.10393	-0.91440
H	4.82157	4.00719	-2.67968
H	5.93386	2.81324	-1.91396
C	3.56521	1.53129	-2.65098
H	2.74926	0.81984	-2.40503
H	4.48370	0.95838	-2.89215
H	3.26617	2.10114	-3.55647
C	2.65143	1.43603	3.46563
H	1.72102	1.98120	3.20910
C	3.38704	2.27443	4.51775
H	2.71900	2.47119	5.38333
H	3.69036	3.25114	4.08554
H	4.29528	1.74909	4.88258
C	2.19544	0.09514	4.06343
H	1.76384	-0.55442	3.27604
H	1.41478	0.27124	4.83409
H	3.03802	-0.44448	4.54442
C	6.60875	-0.77343	1.18186
H	7.01385	-0.96672	0.16293
C	6.18183	-2.12928	1.76097
H	5.26603	-2.49548	1.25225
H	5.97255	-2.05666	2.84902
H	6.99071	-2.87580	1.61099
C	7.74080	-0.15975	2.01133
H	8.06135	0.80431	1.56143
H	8.61584	-0.84408	2.02660

H	7.41396	0.02440	3.05715
C	-6.62090	-3.18207	0.57250
H	-6.62062	-3.97205	-0.21224
C	-6.06848	-3.82245	1.85014
H	-5.01074	-4.11737	1.69325
H	-6.65035	-4.73455	2.10220
H	-6.12099	-3.12053	2.70916
C	-8.07666	-2.74130	0.76283
H	-8.45780	-2.27519	-0.17098
H	-8.17044	-2.00995	1.59338
H	-8.71265	-3.62198	0.99558
H	0.37293	-2.52584	1.07347
C	-0.94165	-3.95268	1.70818
C	0.12354	-3.84750	-0.49779
C	-1.06766	-3.40380	2.99587
C	-1.76296	-5.03431	1.31173
C	-0.76441	-4.91378	-0.91468
C	-1.98266	-3.95146	3.88391
H	-0.45760	-2.54975	3.27352
C	-2.66790	-5.57917	2.24016
C	-1.63871	-5.48482	-0.04897
H	-0.68573	-5.25918	-1.93605
C	-2.77928	-5.04849	3.51671
H	-2.08015	-3.52264	4.87675
H	-3.29195	-6.41450	1.93385
H	-2.26992	-6.30404	-0.38313
H	-3.48537	-5.47019	4.22445
C	1.04267	-3.21110	-1.32959
H	0.03843	-1.63125	-1.39872
C	2.32799	-2.54438	-0.86695
H	2.20347	-2.20399	0.16860
H	2.51508	-1.64111	-1.45032
N	3.48821	-3.41140	-0.98658
N	-0.02141	-3.43464	0.81682
C	4.70587	-3.06543	-1.55993
C	5.85931	-3.79830	-1.21269
C	4.84224	-2.02857	-2.50141
C	7.09917	-3.48169	-1.75636
H	5.77496	-4.60954	-0.49271
C	6.09209	-1.71674	-3.03251
H	3.97038	-1.48203	-2.84023
C	7.23329	-2.42968	-2.66503
H	7.96987	-4.05918	-1.45759
H	6.16742	-0.90615	-3.75236
H	8.20287	-2.17758	-3.08209
H	3.53361	-4.15335	-0.30369
Cl	1.11491	-3.72537	-3.03628

Catalyst (TRIP, 3)-Enamine protonation TS (pro-R) alternative conformation
Electronic energy (Hartrees): -1870.530607
Free Energy at 1M concentration and 253 K (Hartrees): -1869.310272
Coordinates:

P	0.52370	-0.13931	-0.24852
O	0.14062	-1.55777	-0.96234
O	1.81309	-0.43881	0.71014
O	-0.55831	0.22107	0.80753
O	0.78277	0.86366	-1.32880
C	0.13622	-2.65085	-0.12971
C	-1.13678	-3.39234	0.08111
C	-1.12548	-4.56822	0.72720

H	-2.04592	-5.12393	0.86151
C	0.11307	-5.06499	1.35366
C	0.07266	-6.28714	2.18960
H	-0.84676	-6.85153	2.28412
C	1.16952	-6.69571	2.83600
H	1.13873	-7.58738	3.44873
C	2.42015	-5.92061	2.73205
H	3.29621	-6.23931	3.28206
C	2.46773	-4.81854	1.97481
H	3.38669	-4.24903	1.93943
C	1.26643	-4.37327	1.21555
C	1.29932	-3.16240	0.33801
C	2.79590	-1.21822	0.14891
C	4.15554	-0.64234	-0.02171
C	5.18358	-1.43580	-0.35940
H	6.18182	-1.02333	-0.45122
C	4.94644	-2.84655	-0.71813
C	6.06220	-3.66850	-1.24091
H	7.06158	-3.25539	-1.29891
C	5.83946	-4.92242	-1.64856
H	6.65476	-5.52179	-2.03266
C	4.47756	-5.48662	-1.59371
H	4.29854	-6.49219	-1.95195
C	3.46426	-4.75380	-1.11780
H	2.46901	-5.17783	-1.11424
C	3.70276	-3.36986	-0.62315
C	2.59470	-2.54080	-0.05484
C	4.37223	0.80479	0.20724
C	4.30593	1.33998	1.61088
C	4.60105	1.65529	-0.83533
C	4.40514	2.66711	1.80767
C	4.68348	3.11541	-0.56515
C	4.56836	3.60480	0.67755
H	4.38342	3.06917	2.81137
H	4.83687	3.79638	-1.39197
C	4.81594	1.17595	-2.27545
H	4.69370	0.08034	-2.36896
C	3.79041	1.77365	-3.24259
H	2.76869	1.47927	-2.93065
H	3.95628	1.37444	-4.26613
H	3.86541	2.87954	-3.28794
C	6.24426	1.49042	-2.73588
H	6.41753	2.58614	-2.78802
H	6.42556	1.05674	-3.74261
H	6.97754	1.04402	-2.03056
C	4.20386	0.41052	2.82036
H	4.10522	-0.64416	2.49534
C	2.96364	0.72014	3.67198
H	2.05171	0.73085	3.03945
H	3.05556	1.70392	4.17830
H	2.83525	-0.06150	4.45092
C	5.47994	0.47122	3.66951
H	5.61692	1.47652	4.12110
H	6.36517	0.23648	3.04027
H	5.42810	-0.27802	4.48825
C	-2.42737	-2.83011	-0.38714
C	-2.74190	-2.80079	-1.71335
C	-3.43489	-2.36159	0.63223
C	-4.07422	-2.28905	-2.12819
C	-4.64177	-1.94030	0.20875

C	-4.98505	-1.89529	-1.22857
H	-4.32262	-2.26387	-3.18117
H	-5.38988	-1.63022	0.92541
C	-1.81446	-3.33992	-2.80372
H	-0.85243	-3.69516	-2.38475
C	-2.43975	-4.55612	-3.49753
H	-2.68719	-5.33548	-2.74516
H	-1.71997	-4.99151	-4.22335
H	-3.36510	-4.27920	-4.04512
C	-1.46491	-2.24792	-3.82421
H	-1.06285	-1.35104	-3.30800
H	-2.35385	-1.94686	-4.41690
H	-0.68836	-2.62037	-4.52596
C	-3.14526	-2.40937	2.13552
H	-2.09495	-2.70682	2.32161
C	-4.03111	-3.45246	2.82929
H	-3.73389	-3.55705	3.89464
H	-3.91411	-4.44206	2.33953
H	-5.10154	-3.15890	2.78897
C	-3.30014	-1.03787	2.80584
H	-2.62692	-0.30262	2.32394
H	-3.01348	-1.10518	3.87708
H	-4.34552	-0.66946	2.75110
C	-6.35947	-1.43450	-1.67987
H	-6.42484	-1.46206	-2.79102
C	-6.62073	0.01268	-1.25428
H	-5.81452	0.67264	-1.63854
H	-6.66571	0.09898	-0.14884
H	-7.58692	0.36605	-1.67323
C	-7.45084	-2.36827	-1.14431
H	-7.24566	-3.41346	-1.46049
H	-8.44008	-2.07022	-1.55270
H	-7.50211	-2.33218	-0.03538
C	4.62620	5.10037	0.92632
H	4.63795	5.64516	-0.04466
C	3.38847	5.57966	1.69591
H	3.41325	5.23965	2.75275
H	3.34169	6.68949	1.68553
H	2.46616	5.18423	1.22235
C	5.91146	5.47515	1.67120
H	6.79786	5.14299	1.08942
H	5.94438	5.00141	2.67550
H	5.97099	6.57744	1.79623
H	-0.14955	2.33603	-1.30891
C	0.25215	4.34932	-1.01642
C	-1.65287	3.13896	-0.15646
C	1.39374	4.34080	-1.83765
C	-0.10860	5.51723	-0.29774
C	-1.99590	4.28825	0.62720
C	2.15295	5.48944	-1.95494
H	1.64704	3.42952	-2.36127
C	0.68126	6.67923	-0.44900
C	-1.25551	5.43393	0.54626
H	-2.89863	4.25270	1.22039
C	1.79503	6.66763	-1.26648
H	3.03529	5.48321	-2.58711
H	0.40255	7.57658	0.09580
H	-1.55352	6.31163	1.11355
H	2.40227	7.56038	-1.37366
N	-0.53056	3.22274	-0.91004

C	-2.38125	1.89660	-0.10285
C	-3.67380	1.86491	0.67425
H	-3.44481	2.12158	1.71678
H	-4.05033	0.83429	0.68149
N	-4.68569	2.79535	0.16925
H	-4.66315	2.86322	-0.84147
C	-5.98494	2.77077	0.68497
C	-7.03270	3.35336	-0.05479
C	-6.28501	2.20395	1.93676
C	-8.33072	3.36723	0.44127
H	-6.81520	3.79019	-1.02672
C	-7.59223	2.22793	2.42451
H	-5.50572	1.73656	2.52772
C	-8.62447	2.80507	1.68731
H	-9.12112	3.81758	-0.15293
H	-7.80066	1.78007	3.39239
H	-9.63949	2.81468	2.07141
H	-1.37472	0.94601	0.46295
Cl	-2.61059	1.13269	-1.75073

Catalyst (TRIP, 3)-Enamine protonation TS (pro-S) lowest free energy conformation
Electronic energy (Hartrees): -1870.526015
Free Energy at 1M concentration and 253 K (Hartrees): -1869.309379
Coordinates:

P	-0.20314	-0.37797	-0.30248
O	-1.56799	0.29655	0.30771
O	0.62967	0.80278	-1.06724
O	-0.45012	-1.49049	-1.27199
O	0.58955	-0.72684	0.98376
C	-2.10238	1.35741	-0.37858
C	-3.49934	1.24837	-0.86551
C	-4.09137	2.29712	-1.45272
H	-5.11479	2.21957	-1.80109
C	-3.34837	3.55632	-1.65288
C	-3.98921	4.67717	-2.37868
H	-5.01108	4.58705	-2.72573
C	-3.30803	5.80375	-2.61220
H	-3.77644	6.62143	-3.14463
C	-1.91053	5.93021	-2.15788
H	-1.35661	6.83506	-2.37243
C	-1.32423	4.92617	-1.49587
H	-0.29004	5.03348	-1.19813
C	-2.07872	3.67681	-1.19905
C	-1.44869	2.54124	-0.45705
C	0.89744	1.87482	-0.25401
C	2.30361	2.17887	0.10440
C	2.57267	3.17713	0.95842
H	3.59931	3.42564	1.20034
C	1.47217	3.96677	1.55023
C	1.77300	5.00576	2.56135
H	2.79721	5.19761	2.85593
C	0.78003	5.70399	3.12288
H	1.00013	6.45880	3.86663
C	-0.62008	5.44100	2.73782
H	-1.41973	5.99737	3.20947
C	-0.90192	4.51337	1.81568
H	-1.93605	4.32302	1.56035
C	0.19255	3.74330	1.16737
C	-0.09771	2.69114	0.14949

C	3.40303	1.47938	-0.59958
C	3.67677	1.82596	-2.03757
C	4.14289	0.51824	0.01876
C	4.62433	1.14078	-2.70567
C	5.21671	-0.16801	-0.74618
C	5.43035	0.09617	-2.04356
H	4.82711	1.36528	-3.74484
H	5.82025	-0.91567	-0.25068
C	3.90129	0.08410	1.46294
H	2.97716	0.54164	1.87286
C	3.70277	-1.43508	1.55810
H	2.98179	-1.77314	0.78645
H	3.30335	-1.70608	2.55610
H	4.65594	-1.98487	1.41348
C	5.05837	0.53732	2.35874
H	6.01795	0.08255	2.03288
H	4.86678	0.24045	3.41142
H	5.15551	1.64315	2.32217
C	2.91966	2.94768	-2.74994
H	2.20541	3.43948	-2.06063
C	2.08786	2.40493	-3.91918
H	1.40968	1.60025	-3.56456
H	2.73829	1.99730	-4.72146
H	1.46410	3.21695	-4.35034
C	3.87157	4.05663	-3.21760
H	4.55925	3.69376	-4.01013
H	4.47332	4.42635	-2.35983
H	3.28857	4.91018	-3.62509
C	-4.24498	-0.01783	-0.68628
C	-4.78898	-0.34076	0.51965
C	-4.41205	-0.94467	-1.85751
C	-5.52304	-1.62539	0.65899
C	-5.11892	-2.07896	-1.69523
C	-5.68433	-2.45479	-0.38121
H	-5.93555	-1.89325	1.62307
H	-5.27597	-2.74444	-2.53283
C	-4.69551	0.57210	1.74314
H	-4.12486	1.49670	1.52157
C	-6.08773	1.03380	2.18891
H	-6.60864	1.53436	1.34470
H	-5.99737	1.76333	3.02201
H	-6.70584	0.17948	2.53655
C	-3.95402	-0.12313	2.89036
H	-2.97783	-0.50373	2.52867
H	-4.53949	-0.97440	3.29641
H	-3.76343	0.59789	3.71383
C	-3.82604	-0.61194	-3.22908
H	-3.15623	0.26888	-3.15865
C	-4.93825	-0.24605	-4.21833
H	-4.49778	0.06273	-5.19038
H	-5.53492	0.60328	-3.82267
H	-5.61612	-1.10860	-4.39317
C	-2.95857	-1.75363	-3.78139
H	-2.23260	-2.09219	-3.01515
H	-2.38963	-1.40079	-4.66805
H	-3.57694	-2.62171	-4.09213
C	-6.42504	-3.76827	-0.20919
H	-6.77271	-3.87383	0.84333
C	-5.49976	-4.95681	-0.49485
H	-4.60036	-4.90334	0.15563

H	-5.17420	-4.96660	-1.55668
H	-6.02783	-5.91008	-0.27906
C	-7.67303	-3.81546	-1.09817
H	-8.32616	-2.94270	-0.88321
H	-8.25174	-4.74089	-0.89111
H	-7.40130	-3.80422	-2.17487
C	6.47905	-0.65670	-2.84183
H	6.49114	-0.26779	-3.88413
C	7.88473	-0.43874	-2.26818
H	8.10454	0.64840	-2.20289
H	8.64208	-0.90657	-2.93278
H	7.98092	-0.88671	-1.25657
C	6.14027	-2.15040	-2.92705
H	5.12476	-2.28369	-3.35727
H	6.17047	-2.62843	-1.92480
H	6.86714	-2.66913	-3.58771
H	0.11233	-2.97653	-0.44289
C	1.80494	-4.05828	-0.84929
C	0.85485	-3.70175	1.34849
C	1.72588	-3.79769	-2.23198
C	2.91414	-4.75592	-0.30600
C	1.96505	-4.41163	1.90826
C	2.74148	-4.24683	-3.05517
H	0.89108	-3.21782	-2.61131
C	3.92706	-5.21039	-1.17997
C	2.94343	-4.93297	1.10892
H	2.00124	-4.55737	2.97957
C	3.84183	-4.96190	-2.53552
H	2.69461	-4.03727	-4.11928
H	4.77592	-5.74702	-0.76594
H	3.76443	-5.49226	1.54969
H	4.62610	-5.30374	-3.20299
C	-0.10084	-2.96474	2.13105
C	-0.07751	-3.03348	3.66463
H	-0.03005	-4.06888	4.02860
H	-1.03530	-2.64189	4.01754
N	0.99296	-2.30019	4.31343
N	0.81688	-3.60602	-0.00560
C	1.07826	-0.91002	4.37695
C	0.02219	-0.06839	3.98789
C	2.25204	-0.31632	4.88118
C	0.14695	1.31538	4.09503
H	-0.88194	-0.48682	3.56796
C	2.36496	1.06606	4.98063
H	3.07996	-0.95137	5.18985
C	1.31296	1.89799	4.58979
H	-0.68168	1.94050	3.77323
H	3.28547	1.49579	5.36636
H	1.40297	2.97618	4.67140
H	1.86923	-2.78437	4.42845
H	0.26942	-1.65746	1.60206
Cl	-1.80542	-3.11899	1.50421

Catalyst (TRIP, 3)-Enamine protonation TS (pro-S) alternative conformation
Electronic energy (Hartrees): -1870.526015
Free Energy at 1M concentration and 253 K (Hartrees): -1869.309372
Coordinates:

P	-0.20328	-0.37796	-0.30208
O	-1.56801	0.29680	0.30810
O	0.62973	0.80266	-1.06685

O	-0.45047	-1.49038	-1.27164
O	0.58937	-0.72701	0.98413
C	-2.10222	1.35767	-0.37830
C	-3.49912	1.24873	-0.86548
C	-4.09098	2.29753	-1.45277
H	-5.11435	2.22006	-1.80131
C	-3.34785	3.55668	-1.65281
C	-3.98850	4.67758	-2.37870
H	-5.01034	4.58755	-2.72587
C	-3.30720	5.80410	-2.61214
H	-3.77546	6.62182	-3.14464
C	-1.90973	5.93044	-2.15765
H	-1.35571	6.83524	-2.37215
C	-1.32361	4.92635	-1.49556
H	-0.28944	5.03357	-1.19770
C	-2.07825	3.67706	-1.19881
C	-1.44841	2.54143	-0.45674
C	0.89763	1.87470	-0.25365
C	2.30384	2.17867	0.10465
C	2.57302	3.17692	0.95864
H	3.59970	3.42538	1.20048
C	1.47262	3.96666	1.55049
C	1.77358	5.00567	2.56156
H	2.79781	5.19745	2.85608
C	0.78069	5.70400	3.12311
H	1.00089	6.45882	3.86682
C	-0.61946	5.44111	2.73813
H	-1.41904	5.99756	3.20979
C	-0.90142	4.51348	1.81602
H	-1.93557	4.32321	1.56073
C	0.19296	3.74330	1.16769
C	-0.09743	2.69115	0.14984
C	3.40316	1.47915	-0.59944
C	3.67685	1.82583	-2.03742
C	4.14298	0.51790	0.01878
C	4.62436	1.14066	-2.70561
C	5.21673	-0.16834	-0.74626
C	5.43035	0.09595	-2.04362
H	4.82711	1.36524	-3.74477
H	5.82024	-0.91608	-0.25085
C	3.90139	0.08363	1.46292
H	2.97733	0.54125	1.87293
C	3.70267	-1.43553	1.55790
H	2.98156	-1.77340	0.78629
H	3.30331	-1.70660	2.55591
H	4.65575	-1.98543	1.41313
C	5.05856	0.53661	2.35872
H	6.01807	0.08174	2.03278
H	4.86698	0.23966	3.41138
H	5.15585	1.64243	2.32225
C	2.91973	2.94761	-2.74969
H	2.20551	3.43936	-2.06032
C	2.08788	2.40494	-3.91892
H	1.40972	1.60024	-3.56434
H	2.73828	1.99737	-4.72127
H	1.46410	3.21699	-4.35000
C	3.87164	4.05657	-3.21730
H	4.55929	3.69375	-4.00987
H	4.47341	4.42624	-2.35952
H	3.28863	4.91016	-3.62472

C	-4.24482	-0.01747	-0.68647
C	-4.78904	-0.34048	0.51934
C	-4.41169	-0.94422	-1.85780
C	-5.52309	-1.62514	0.65847
C	-5.11860	-2.07852	-1.69572
C	-5.68420	-2.45446	-0.38182
H	-5.93574	-1.89308	1.62247
H	-5.27553	-2.74394	-2.53340
C	-4.69581	0.57232	1.74289
H	-4.12518	1.49697	1.52146
C	-6.08812	1.03390	2.18848
H	-6.60894	1.53447	1.34422
H	-5.99793	1.76340	3.02163
H	-6.70623	0.17953	2.53600
C	-3.95444	-0.12294	2.89018
H	-2.97814	-0.50339	2.52862
H	-4.53989	-0.97431	3.29605
H	-3.76408	0.59803	3.71376
C	-3.82547	-0.61138	-3.22926
H	-3.15556	0.26935	-3.15862
C	-4.93752	-0.24522	-4.21859
H	-4.49691	0.06362	-5.19056
H	-5.53414	0.60413	-3.82289
H	-5.61548	-1.10767	-4.39361
C	-2.95806	-1.75309	-3.78164
H	-2.23228	-2.09191	-3.01534
H	-2.38893	-1.40016	-4.66814
H	-3.57651	-2.62102	-4.09265
C	-6.42488	-3.76799	-0.21000
H	-6.77270	-3.87364	0.84246
C	-5.49950	-4.95648	-0.49561
H	-4.60019	-4.90301	0.15501
H	-5.17379	-4.96617	-1.55739
H	-6.02756	-5.90978	-0.27995
C	-7.67274	-3.81517	-1.09917
H	-8.32594	-2.94246	-0.88425
H	-8.25144	-4.74065	-0.89226
H	-7.40085	-3.80384	-2.17583
C	6.47898	-0.65691	-2.84199
H	6.49102	-0.26793	-3.88427
C	7.88470	-0.43901	-2.26840
H	8.10454	0.64812	-2.20308
H	8.64200	-0.90683	-2.93306
H	7.98093	-0.88703	-1.25682
C	6.14016	-2.15060	-2.92727
H	5.12461	-2.28384	-3.35742
H	6.17043	-2.62869	-1.92506
H	6.86697	-2.66930	-3.58802
H	0.11176	-2.97651	-0.44259
C	1.80427	-4.05828	-0.84932
C	0.85446	-3.70198	1.34861
C	1.72509	-3.79745	-2.23196
C	2.91350	-4.75607	-0.30626
C	1.96465	-4.41204	1.90815
C	2.74061	-4.24647	-3.05531
H	0.89029	-3.21747	-2.61111
C	3.92631	-5.21044	-1.18040
C	2.94291	-4.93335	1.10862
H	2.00093	-4.55799	2.97943
C	3.84098	-4.96169	-2.53589

H	2.69366	-4.03672	-4.11938
H	4.77517	-5.74719	-0.76654
H	3.76390	-5.49278	1.54923
H	4.62517	-5.30344	-3.20349
C	-0.10104	-2.96493	2.13140
C	-0.07751	-3.03406	3.66497
H	-0.02980	-4.06954	4.02867
H	-1.03530	-2.64275	4.01813
N	0.99283	-2.30072	4.31400
N	0.81634	-3.60609	-0.00546
C	1.07791	-0.91046	4.37713
C	2.25184	-0.31644	4.88059
C	0.02143	-0.06913	3.98856
C	2.36453	1.06598	4.97977
H	3.08007	-0.95126	5.18891
C	0.14595	1.31468	4.09540
H	-0.88285	-0.48784	3.56925
C	1.31212	1.89761	4.58941
H	3.28517	1.49598	5.36489
H	-0.68299	1.93958	3.77394
H	1.40193	2.97584	4.67081
H	1.86962	-2.78442	4.42726
H	0.26922	-1.65765	1.60247
Cl	-1.80574	-3.11900	1.50479

Catalyst (TRIP, 3)-Enamine protonation TS (pro-S) alternative conformation
Electronic energy (Hartrees): -1870.525716
Free Energy at 1M concentration and 253 K (Hartrees): -1869.308862
Coordinates:

P	-0.20886	-0.39742	-0.25427
O	-1.56215	0.33133	0.31734
O	0.69411	0.74994	-0.99015
O	-0.47366	-1.49384	-1.23758
O	0.53172	-0.78515	1.05148
C	-2.03116	1.41420	-0.38240
C	-3.41344	1.36156	-0.91871
C	-3.94427	2.43591	-1.51805
H	-4.95693	2.39960	-1.90276
C	-3.14682	3.66617	-1.68515
C	-3.71920	4.81465	-2.42491
H	-4.73223	4.76670	-2.80477
C	-2.98677	5.91414	-2.63069
H	-3.40511	6.75200	-3.17338
C	-1.60045	5.98278	-2.13195
H	-1.00426	6.86545	-2.32454
C	-1.07592	4.95346	-1.45703
H	-0.04798	5.01813	-1.12717
C	-1.88869	3.73409	-1.19027
C	-1.32855	2.57121	-0.43427
C	0.98179	1.80929	-0.16723
C	2.38978	2.06156	0.22321
C	2.67563	3.05019	1.08319
H	3.70480	3.26062	1.34927
C	1.59170	3.87963	1.65050
C	1.90708	4.90699	2.66909
H	2.93062	5.06139	2.98712
C	0.92765	5.64141	3.20792
H	1.15807	6.38795	3.95688
C	-0.47203	5.42943	2.79093
H	-1.26144	6.01473	3.24445

C	-0.76634	4.51236	1.86211
H	-1.80054	4.35971	1.58270
C	0.31393	3.70303	1.23844
C	0.00909	2.66355	0.21202
C	3.47953	1.31808	-0.45089
C	3.79435	1.63835	-1.88641
C	4.17652	0.34240	0.19418
C	4.73576	0.91787	-2.52474
C	5.25354	-0.37778	-0.53543
C	5.50406	-0.13644	-1.82909
H	4.96408	1.12249	-3.56190
H	5.83060	-1.13029	-0.01434
C	3.87949	-0.07707	1.63237
H	2.95908	0.41150	2.01372
C	3.62718	-1.58804	1.72478
H	2.90821	-1.90048	0.94060
H	3.20093	-1.84457	2.71525
H	4.56312	-2.17052	1.59692
C	5.02349	0.34099	2.56142
H	5.97536	-0.15208	2.27076
H	4.78665	0.06127	3.60969
H	5.16311	1.44207	2.51944
C	3.08722	2.77254	-2.62961
H	2.37102	3.29270	-1.96365
C	2.26941	2.24027	-3.81363
H	1.56175	1.45722	-3.46856
H	2.92764	1.80770	-4.59628
H	1.67812	3.06424	-4.26727
C	4.08234	3.84794	-3.08565
H	4.77953	3.45553	-3.85554
H	4.67237	4.21116	-2.21703
H	3.53483	4.71253	-3.51831
C	-4.21140	0.12235	-0.78079
C	-4.81348	-0.19057	0.39980
C	-4.37073	-0.78502	-1.96833
C	-5.60396	-1.44538	0.49610
C	-5.12936	-1.89061	-1.84654
C	-5.75964	-2.25630	-0.55943
H	-6.06357	-1.70596	1.44068
H	-5.28139	-2.54028	-2.69737
C	-4.72901	0.70456	1.63681
H	-4.11219	1.60659	1.44825
C	-6.11676	1.21949	2.03570
H	-6.58360	1.75126	1.17900
H	-6.02790	1.93495	2.88108
H	-6.78290	0.38810	2.34853
C	-4.06108	-0.03384	2.80195
H	-3.09151	-0.45605	2.46977
H	-4.69879	-0.86081	3.17856
H	-3.86668	0.67049	3.63890
C	-3.71900	-0.46285	-3.31258
H	-3.01524	0.38736	-3.20557
C	-4.77600	-0.03843	-4.33835
H	-4.28596	0.26152	-5.28922
H	-5.35050	0.83156	-3.95499
H	-5.48304	-0.86895	-4.54924
C	-2.88060	-1.63468	-3.84652
H	-2.20052	-2.01403	-3.05763
H	-2.26265	-1.29639	-4.70563
H	-3.52326	-2.47110	-4.19286

C	-6.56029	-3.53933	-0.43146
H	-6.95190	-3.64141	0.60586
C	-5.67499	-4.76260	-0.69664
H	-4.79946	-4.75381	-0.01227
H	-5.31005	-4.77482	-1.74556
H	-6.25005	-5.69510	-0.51239
C	-7.77442	-3.52457	-1.36730
H	-8.39847	-2.62751	-1.16637
H	-8.39855	-4.42709	-1.19357
H	-7.46161	-3.51317	-2.43280
C	6.56470	-0.93216	-2.56780
H	7.03537	-1.66976	-1.87910
C	5.94182	-1.73156	-3.71801
H	5.13009	-2.38363	-3.33096
H	6.71042	-2.37898	-4.19147
H	5.52078	-1.05765	-4.49395
C	7.68504	-0.01723	-3.07546
H	8.11697	0.55993	-2.23000
H	7.30873	0.69399	-3.84083
H	8.49569	-0.62615	-3.52962
H	0.02508	-2.99988	-0.41413
C	1.70642	-4.10739	-0.79431
C	0.70789	-3.76603	1.38397
C	1.66923	-3.82012	-2.17354
C	2.78814	-4.83448	-0.23461
C	1.78871	-4.50762	1.96015
C	2.70009	-4.26975	-2.97721
H	0.85506	-3.21863	-2.56376
C	3.81692	-5.28982	-1.08957
C	2.77679	-5.03596	1.17731
H	1.79529	-4.66992	3.02974
C	3.77415	-5.01258	-2.44161
H	2.68635	-4.03857	-4.03780
H	4.64468	-5.84931	-0.66319
H	3.57544	-5.61794	1.62964
H	4.57081	-5.35477	-3.09406
C	-0.24817	-3.01609	2.15444
C	-0.26227	-3.10888	3.68713
H	-0.22676	-4.14972	4.03636
H	-1.22656	-2.71996	4.02397
N	0.79448	-2.38906	4.37448
N	0.70485	-3.65102	0.03112
C	0.88656	-0.99816	4.43684
C	2.05915	-0.40774	4.94713
C	-0.16448	-0.15366	4.04127
C	2.17683	0.97465	5.04279
H	2.88273	-1.04485	5.26298
C	-0.03554	1.22995	4.14426
H	-1.06891	-0.57125	3.62131
C	1.13034	1.80935	4.64304
H	3.09682	1.40219	5.43217
H	-0.85987	1.85706	3.81537
H	1.22390	2.88749	4.72140
H	1.67221	-2.87361	4.47974
H	0.16733	-1.71346	1.65192
Cl	-1.94004	-3.11418	1.48281

Catalyst (TRIP, 3)-Enamine protonation TS (pro-S) alternative conformation
Electronic energy (Hartrees): -1870.527156
Free Energy at 1M concentration and 253 K (Hartrees): -1869.308857

Coordinates:

P	0.51826	-0.06852	0.16794
O	1.75648	0.14361	-0.86904
O	0.08685	1.41894	0.68529
O	0.87447	-0.86006	1.38290
O	-0.62103	-0.60832	-0.74484
C	2.80856	0.79857	-0.28075
C	4.06766	0.05798	-0.01621
C	5.10547	0.69093	0.54920
H	6.03713	0.16492	0.71884
C	4.98053	2.10093	0.97148
C	6.09860	2.75236	1.69204
H	7.01953	2.21121	1.87038
C	5.97435	4.00993	2.12954
H	6.79108	4.48441	2.65802
C	4.71742	4.74964	1.90720
H	4.61414	5.75809	2.28657
C	3.70357	4.17249	1.25197
H	2.78199	4.72424	1.12355
C	3.83873	2.78634	0.72722
C	2.72507	2.12182	-0.01578
C	0.28525	2.46990	-0.17464
C	-0.89413	3.27547	-0.57808
C	-0.72044	4.44721	-1.20703
H	-1.57949	5.04220	-1.49385
C	0.62675	4.87398	-1.63000
C	0.78229	6.10433	-2.44007
H	-0.07722	6.72785	-2.65283
C	1.98292	6.44770	-2.91749
H	2.09509	7.34555	-3.51143
C	3.15488	5.59223	-2.65393
H	4.11802	5.85903	-3.06960
C	3.02595	4.48159	-1.91911
H	3.89399	3.85446	-1.76837
C	1.70391	4.10618	-1.34434
C	1.53638	2.88652	-0.49308
C	-2.26386	2.74113	-0.37242
C	-2.84782	2.69151	1.01363
C	-2.99864	2.29294	-1.43187
C	-4.09032	2.19829	1.18126
C	-4.39033	1.83071	-1.20082
C	-4.90877	1.75795	0.03131
H	-4.52484	2.14842	2.17036
H	-4.99572	1.54764	-2.05088
C	-2.45777	2.23995	-2.86361
H	-1.38072	2.49928	-2.90208
C	-2.55568	0.82569	-3.45296
H	-2.05963	0.09588	-2.78235
H	-2.04243	0.78990	-4.43764
H	-3.61051	0.51616	-3.60342
C	-3.18935	3.24936	-3.75586
H	-4.26480	2.99120	-3.85592
H	-2.73375	3.26217	-4.76903
H	-3.10591	4.27005	-3.32664
C	-2.08299	3.21344	2.22871
H	-1.07606	3.56738	1.93201
C	-1.86871	2.11051	3.27616
H	-1.41339	1.21622	2.80377
H	-2.82578	1.80959	3.75175
H	-1.18186	2.46963	4.07186

C	-2.79037	4.42583	2.84716
H	-3.78147	4.14652	3.26311
H	-2.93299	5.21453	2.07761
H	-2.17187	4.84912	3.66731
C	4.19024	-1.36569	-0.41291
C	4.32983	-1.71403	-1.72331
C	4.19490	-2.43240	0.64929
C	4.45952	-3.15167	-2.07841
C	4.37172	-3.71589	0.28368
C	4.48530	-4.10532	-1.13769
H	4.54213	-3.43046	-3.12089
H	4.41299	-4.49290	1.03442
C	4.39847	-0.68712	-2.85544
H	4.30923	0.34997	-2.47503
C	5.75370	-0.75158	-3.57019
H	6.57614	-0.61525	-2.83558
H	5.82470	0.06135	-4.32424
H	5.89291	-1.72311	-4.08924
C	3.24525	-0.88198	-3.84827
H	2.27185	-0.86375	-3.31427
H	3.33625	-1.84617	-4.39081
H	3.24500	-0.05946	-4.59509
C	4.02707	-2.09186	2.12978
H	3.70386	-1.03699	2.24354
C	5.35974	-2.23893	2.87328
H	5.24174	-1.92505	3.93242
H	6.13394	-1.59546	2.40590
H	5.71301	-3.29203	2.85123
C	2.93764	-2.94370	2.80417
H	2.02952	-2.99112	2.17041
H	2.66036	-2.49679	3.78268
H	3.28807	-3.98114	2.98801
C	4.61055	-5.56701	-1.52704
H	4.67870	-5.66115	-2.63441
C	3.37186	-6.35639	-1.08712
H	2.45520	-5.89358	-1.51185
H	3.28157	-6.37935	0.01962
H	3.43617	-7.40183	-1.45731
C	5.88971	-6.18159	-0.94737
H	6.77467	-5.59233	-1.27006
H	6.01234	-7.22198	-1.31718
H	5.85854	-6.20423	0.16249
C	-6.32217	1.24496	0.23924
H	-6.78521	0.99552	-0.74226
C	-6.31483	-0.04359	1.06730
H	-5.68679	-0.80933	0.56524
H	-7.34610	-0.44675	1.15537
H	-5.91685	0.13818	2.08807
C	-7.20736	2.31411	0.88994
H	-7.18626	3.24540	0.28439
H	-6.86626	2.54746	1.92063
H	-8.25816	1.95711	0.94035
H	-0.30525	-1.95774	2.02836
C	-1.62646	-1.64051	3.57033
C	-2.15714	-2.56335	1.39393
C	-0.63509	-1.14310	4.43805
C	-2.99375	-1.60883	3.94120
C	-3.54677	-2.53140	1.75279
C	-1.01301	-0.63197	5.66625
H	0.39932	-1.13804	4.11219

C	-3.34295	-1.08959	5.20675
C	-3.93886	-2.08674	2.98260
H	-4.26880	-2.86243	1.02022
C	-2.36729	-0.60863	6.05918
H	-0.25415	-0.23731	6.33474
H	-4.39065	-1.06672	5.49298
H	-4.99292	-2.09341	3.24740
H	-2.64078	-0.20525	7.02869
C	-1.68070	-2.90865	0.08622
C	-2.64556	-3.49663	-0.93362
H	-3.14623	-4.39553	-0.54303
H	-2.06156	-3.81803	-1.80803
N	-3.66717	-2.51718	-1.27884
N	-1.27758	-2.14339	2.33837
C	-4.71180	-2.81994	-2.15387
C	-5.57456	-1.78523	-2.56553
C	-4.96988	-4.12482	-2.61011
C	-6.65185	-2.04530	-3.40230
H	-5.39306	-0.77752	-2.20719
C	-6.06299	-4.37477	-3.44222
H	-4.31774	-4.94363	-2.32878
C	-6.91031	-3.34561	-3.84763
H	-7.29814	-1.22594	-3.70499
H	-6.24185	-5.39101	-3.78291
H	-7.75411	-3.54857	-4.49932
H	-3.30772	-1.57479	-1.36572
H	-1.09913	-1.55584	-0.41196
Cl	-0.12679	-3.84601	0.08711

Catalyst (TRIP, 3)-Enamine protonation TS (pro-S) alternative conformation
Electronic energy (Hartrees): -1870.525798
Free Energy at 1M concentration and 253 K (Hartrees): -1869.308844
Coordinates:

P	-0.18840	-0.36871	-0.35272
O	-1.57571	0.27517	0.23866
O	0.66948	0.84886	-1.02738
O	-0.39871	-1.43066	-1.38571
O	0.55724	-0.78260	0.94229
C	-2.08532	1.36932	-0.41338
C	-3.46117	1.28412	-0.96150
C	-4.03425	2.36247	-1.51280
H	-5.04247	2.30179	-1.90597
C	-3.28857	3.63178	-1.61567
C	-3.90505	4.78923	-2.30420
H	-4.91304	4.71693	-2.69347
C	-3.21876	5.92717	-2.45192
H	-3.66927	6.77169	-2.95723
C	-1.83937	6.03022	-1.93998
H	-1.28020	6.94550	-2.08567
C	-1.27535	4.99278	-1.31091
H	-0.25302	5.08481	-0.97020
C	-2.03704	3.72889	-1.10885
C	-1.43214	2.55592	-0.40483
C	0.90641	1.87946	-0.15315
C	2.29905	2.16857	0.26526
C	2.53658	3.12305	1.17677
H	3.55395	3.36153	1.46383
C	1.41448	3.87950	1.77137
C	1.67834	4.86733	2.84235
H	2.69169	5.04644	3.17944

C	0.66497	5.53465	3.40510
H	0.85789	6.25190	4.19230
C	-0.72090	5.28811	2.96190
H	-1.53759	5.81848	3.43431
C	-0.96905	4.40714	1.98588
H	-1.99354	4.22756	1.68745
C	0.14889	3.67303	1.33598
C	-0.10400	2.67361	0.25685
C	3.42325	1.50937	-0.43831
C	3.74232	1.93183	-1.84631
C	4.14569	0.51810	0.15223
C	4.71365	1.28470	-2.51822
C	5.24627	-0.12492	-0.61220
C	5.50140	0.20804	-1.88605
H	4.94919	1.56398	-3.53701
H	5.83574	-0.89684	-0.13734
C	3.85787	0.00750	1.56243
H	2.92014	0.44259	1.96581
C	3.65849	-1.51440	1.56939
H	2.96192	-1.80982	0.75876
H	3.22869	-1.83858	2.53845
H	4.61634	-2.05599	1.42478
C	4.98424	0.41203	2.51864
H	5.95446	-0.02608	2.20155
H	4.75787	0.06086	3.54744
H	5.08171	1.51821	2.54313
C	3.00403	3.08756	-2.52319
H	2.26627	3.54116	-1.83244
C	2.21196	2.60463	-3.74505
H	1.52565	1.78137	-3.45447
H	2.88917	2.24037	-4.54605
H	1.59941	3.43674	-4.15339
C	3.96605	4.22149	-2.90179
H	4.67989	3.90204	-3.68987
H	4.53888	4.54718	-2.00717
H	3.39299	5.09391	-3.28257
C	-4.20533	0.00669	-0.88624
C	-4.78939	-0.39080	0.27782
C	-4.32165	-0.85074	-2.11561
C	-5.50917	-1.68968	0.31603
C	-5.01966	-2.00039	-2.04489
C	-5.62206	-2.45934	-0.77697
H	-5.94962	-2.01888	1.24724
H	-5.14274	-2.61751	-2.92515
C	-4.75519	0.45127	1.55387
H	-4.19050	1.39432	1.40704
C	-6.17008	0.86850	1.97139
H	-6.66611	1.41066	1.13794
H	-6.12226	1.54937	2.84807
H	-6.78815	-0.01247	2.24489
C	-4.04754	-0.29958	2.68727
H	-3.05340	-0.64695	2.34182
H	-4.63552	-1.17986	3.02165
H	-3.89808	0.37530	3.55704
C	-3.69544	-0.42976	-3.44470
H	-3.04154	0.45390	-3.30026
C	-4.77926	-0.02002	-4.44822
H	-4.31114	0.35304	-5.38414
H	-5.40229	0.79560	-4.02328
H	-5.43707	-0.87962	-4.69801

C	-2.79276	-1.52492	-4.03326
H	-2.08312	-1.89370	-3.26568
H	-2.20464	-1.11415	-4.88165
H	-3.38671	-2.38390	-4.40986
C	-6.34218	-3.79467	-0.73335
H	-6.31469	-4.25592	-1.74544
C	-7.82016	-3.62062	-0.36226
H	-8.30600	-2.90605	-1.06073
H	-7.93242	-3.24144	0.67556
H	-8.34815	-4.59499	-0.43984
C	-5.64258	-4.77102	0.22087
H	-4.57414	-4.88130	-0.06390
H	-6.12287	-5.77092	0.16065
H	-5.69863	-4.41585	1.27173
C	6.57830	-0.49962	-2.68811
H	6.62325	-0.05590	-3.70738
C	7.96366	-0.31003	-2.05772
H	8.17748	0.77248	-1.92772
H	8.74401	-0.74064	-2.72086
H	8.02816	-0.81090	-1.06865
C	6.24764	-1.98726	-2.86346
H	5.24724	-2.09924	-3.33336
H	6.24660	-2.51775	-1.88750
H	6.99757	-2.46897	-3.52638
H	0.13838	-2.95731	-0.62140
C	1.84892	-4.00888	-1.03172
C	0.82933	-3.77378	1.15103
C	1.81003	-3.67647	-2.40062
C	2.94577	-4.72841	-0.49192
C	1.92651	-4.50667	1.70678
C	2.85304	-4.07664	-3.21471
H	0.98336	-3.08158	-2.77438
C	3.98794	-5.13092	-1.35700
C	2.93285	-4.97966	0.91195
H	1.93044	-4.70923	2.76938
C	3.94200	-4.81226	-2.69969
H	2.83699	-3.81183	-4.26736
H	4.82752	-5.68408	-0.94583
H	3.74388	-5.55685	1.34808
H	4.74842	-5.11451	-3.35981
C	-0.15463	-3.08406	1.94205
C	-0.17860	-3.24050	3.46929
H	-0.12520	-4.29422	3.77531
H	-1.15306	-2.88474	3.81353
N	0.85856	-2.52991	4.19451
N	0.83250	-3.60673	-0.19646
C	0.92100	-1.14240	4.32474
C	-0.14368	-0.30061	3.96085
C	2.07702	-0.55472	4.87446
C	-0.04409	1.07838	4.13455
H	-1.03594	-0.71470	3.51225
C	2.16513	0.82310	5.04080
H	2.91091	-1.19028	5.16550
C	1.10500	1.65542	4.67348
H	-0.87834	1.70460	3.82986
H	3.07259	1.24904	5.46018
H	1.17546	2.72980	4.80745
H	1.74425	-3.00174	4.28974
H	0.22243	-1.74825	1.49829
Cl	-1.83773	-3.20858	1.25277

Catalyst (TRIP, 3)-Enamine protonation TS (pro-S) alternative conformation
Electronic energy (Hartrees): -1870.525797
Free Energy at 1M concentration and 253 K (Hartrees): -1869.308813
Coordinates:

P	-0.18778	-0.36968	-0.35239
O	-1.57544	0.27433	0.23808
O	0.67102	0.84776	-1.02618
O	-0.39765	-1.43116	-1.38599
O	0.55690	-0.78416	0.94294
C	-2.08438	1.36858	-0.41433
C	-3.45995	1.28382	-0.96334
C	-4.03205	2.36218	-1.51568
H	-5.04007	2.30186	-1.90942
C	-3.28575	3.63111	-1.61850
C	-3.90111	4.78851	-2.30812
H	-4.90875	4.71643	-2.69835
C	-3.21424	5.92613	-2.45565
H	-3.66393	6.77060	-2.96175
C	-1.83533	6.02887	-1.94239
H	-1.27566	6.94388	-2.08791
C	-1.27232	4.99149	-1.31231
H	-0.25030	5.08327	-0.97064
C	-2.03470	3.72796	-1.11049
C	-1.43091	2.55504	-0.40547
C	0.90732	1.87835	-0.15177
C	2.29966	2.16782	0.26740
C	2.53651	3.12250	1.17888
H	3.55367	3.36124	1.46645
C	1.41396	3.87893	1.77265
C	1.67703	4.86708	2.84352
H	2.69015	5.04637	3.18123
C	0.66323	5.53452	3.40536
H	0.85557	6.25204	4.19246
C	-0.72233	5.28776	2.96130
H	-1.53937	5.81823	3.43298
C	-0.96975	4.40644	1.98540
H	-1.99403	4.22671	1.68630
C	0.14867	3.67222	1.33649
C	-0.10340	2.67256	0.25742
C	3.42444	1.50886	-0.43546
C	3.74454	1.93156	-1.84316
C	4.14680	0.51787	0.15563
C	4.71692	1.28507	-2.51418
C	5.24845	-0.12450	-0.60781
C	5.50472	0.20885	-1.88133
H	4.95322	1.56453	-3.53274
H	5.83787	-0.89614	-0.13246
C	3.85786	0.00702	1.56550
H	2.91965	0.44178	1.96809
C	3.65883	-1.51490	1.57199
H	2.96268	-1.81021	0.76096
H	3.22865	-1.83942	2.54076
H	4.61686	-2.05625	1.42769
C	4.98324	0.41168	2.52281
H	5.95380	-0.02642	2.20676
H	4.75579	0.06061	3.55142
H	5.08062	1.51786	2.54730
C	3.00611	3.08680	-2.52072
H	2.26747	3.53997	-1.83062

C	2.21540	2.60330	-3.74322
H	1.52932	1.77967	-3.45316
H	2.89352	2.23936	-4.54360
H	1.60271	3.43501	-4.15217
C	3.96774	4.22132	-2.89855
H	4.68241	3.90231	-3.68604
H	4.53963	4.54738	-2.00346
H	3.39445	5.09337	-3.27981
C	-4.20528	0.00710	-0.88738
C	-4.79091	-0.38853	0.27653
C	-4.32209	-0.85109	-2.11617
C	-5.51356	-1.68583	0.31499
C	-5.02272	-1.99917	-2.04531
C	-5.62739	-2.45589	-0.77765
H	-5.95557	-2.01343	1.24603
H	-5.14614	-2.61674	-2.92522
C	-4.75553	0.45406	1.55220
H	-4.18848	1.39569	1.40536
C	-6.16968	0.87486	1.96876
H	-6.66388	1.41800	1.13485
H	-6.12073	1.55587	2.84527
H	-6.79008	-0.00455	2.24213
C	-4.05027	-0.29810	2.68621
H	-3.05726	-0.64903	2.34093
H	-4.64110	-1.17623	3.02134
H	-3.89863	0.37707	3.55541
C	-3.69306	-0.43274	-3.44476
H	-3.03728	0.44955	-3.30015
C	-4.77454	-0.02135	-4.45014
H	-4.30421	0.34966	-5.38579
H	-5.39620	0.79620	-4.02685
H	-5.43408	-0.87963	-4.69999
C	-2.79211	-1.53060	-4.03106
H	-2.08479	-1.90080	-3.26201
H	-2.20142	-1.12186	-4.87868
H	-3.38763	-2.38830	-4.40815
C	-6.35089	-3.78942	-0.73385
H	-6.32351	-4.25145	-1.74558
C	-7.82885	-3.61138	-0.36438
H	-8.31220	-2.89609	-1.06385
H	-7.94122	-3.23113	0.67304
H	-8.35922	-4.58451	-0.44176
C	-5.65472	-4.76689	0.22176
H	-4.58620	-4.87994	-0.06173
H	-6.13738	-5.76569	0.16162
H	-5.71114	-4.41094	1.27233
C	6.58298	-0.49793	-2.68235
H	6.62883	-0.05391	-3.70144
C	7.96750	-0.30767	-2.05032
H	8.18049	0.77493	-1.91972
H	8.74888	-0.73759	-2.71271
H	8.03119	-0.80882	-1.06134
C	6.25343	-1.98571	-2.85849
H	5.25370	-2.09813	-3.32970
H	6.25144	-2.51644	-1.88265
H	7.00448	-2.46681	-3.52058
H	0.13991	-2.95769	-0.62298
C	1.85138	-4.00753	-1.03380
C	0.83111	-3.77517	1.14891
C	1.81242	-3.67411	-2.40246

C	2.94882	-4.72644	-0.49436
C	1.92889	-4.50744	1.70431
C	2.85591	-4.07273	-3.21668
H	0.98529	-3.07966	-2.77589
C	3.99151	-5.12732	-1.35957
C	2.93588	-4.97877	0.90931
H	1.93287	-4.71072	2.76678
C	3.94546	-4.80774	-2.70204
H	2.83979	-3.80715	-4.26913
H	4.83158	-5.67995	-0.94867
H	3.74745	-5.55540	1.34517
H	4.75228	-5.10874	-3.36225
C	-0.15357	-3.08695	1.94043
C	-0.17766	-3.24533	3.46751
H	-0.12147	-4.29927	3.77225
H	-1.15317	-2.89273	3.81193
N	0.85732	-2.53283	4.19415
N	0.83442	-3.60699	-0.19841
C	0.91635	-1.14503	4.32404
C	2.07132	-0.55412	4.87244
C	-0.15100	-0.30623	3.96117
C	2.15589	0.82400	5.03826
H	2.90721	-1.18733	5.16280
C	-0.05499	1.07308	4.13429
H	-1.04268	-0.72298	3.51393
C	1.09314	1.65335	4.67178
H	3.06265	1.25252	5.45653
H	-0.89123	1.69694	3.83022
H	1.16083	2.72798	4.80532
H	1.74472	-3.00195	4.28716
H	0.22246	-1.75059	1.49813
Cl	-1.83644	-3.21158	1.25053

Catalyst (TRIP, 3)-Enamine protonation TS (pro-S) alternative conformation
Electronic energy (Hartrees): -1870.525646
Free Energy at 1M concentration and 253 K (Hartrees): -1869.30868
Coordinates:

P	-0.21348	-0.40575	-0.22772
O	-1.56745	0.33035	0.33199
O	0.69617	0.73415	-0.96826
O	-0.47889	-1.50649	-1.20643
O	0.52202	-0.78883	1.08197
C	-2.02845	1.41124	-0.37631
C	-3.40660	1.36001	-0.92360
C	-3.93056	2.43390	-1.52984
H	-4.94006	2.39845	-1.92286
C	-3.12888	3.66144	-1.69572
C	-3.69301	4.80847	-2.44408
H	-4.70395	4.76220	-2.82970
C	-2.95563	5.90448	-2.65071
H	-3.36788	6.74123	-3.19977
C	-1.57192	5.97053	-2.14452
H	-0.97135	6.85001	-2.33823
C	-1.05493	4.94258	-1.46174
H	-0.02841	5.00514	-1.12718
C	-1.87362	3.72736	-1.19352
C	-1.32203	2.56588	-0.42888
C	0.98496	1.79880	-0.15238
C	2.39398	2.05679	0.23073
C	2.68127	3.05163	1.08313

H	3.71108	3.26590	1.34370
C	1.59808	3.88318	1.64853
C	1.91515	4.91838	2.65865
H	2.93936	5.07610	2.97288
C	0.93642	5.65646	3.19385
H	1.16805	6.40908	3.93633
C	-0.46418	5.43996	2.78203
H	-1.25298	6.02818	3.23285
C	-0.76000	4.51529	1.86124
H	-1.79466	4.35954	1.58515
C	0.31950	3.70237	1.24112
C	0.01327	2.65625	0.22198
C	3.48309	1.31267	-0.44346
C	3.79387	1.62765	-1.88096
C	4.18482	0.34264	0.20462
C	4.73766	0.90864	-2.51749
C	5.26330	-0.37679	-0.52353
C	5.51144	-0.13944	-1.81841
H	4.96351	1.10967	-3.55589
H	5.84403	-1.12480	-0.00001
C	3.89107	-0.07119	1.64493
H	2.97143	0.41857	2.02640
C	3.63825	-1.58130	1.74277
H	2.91216	-1.89376	0.96518
H	3.21992	-1.83495	2.73720
H	4.57242	-2.16512	1.60834
C	5.03653	0.35027	2.57058
H	5.98675	-0.14824	2.28379
H	4.79832	0.07985	3.62105
H	5.17975	1.45055	2.51998
C	3.07966	2.75461	-2.62831
H	2.36259	3.27486	-1.96336
C	2.26144	2.21213	-3.80737
H	1.55801	1.42798	-3.45612
H	2.91977	1.77795	-4.58906
H	1.66551	3.03094	-4.26428
C	4.06853	3.83231	-3.09248
H	4.76545	3.43929	-3.86230
H	4.65922	4.20265	-2.22731
H	3.51582	4.69216	-3.52798
C	-4.20719	0.12180	-0.79306
C	-4.82071	-0.19047	0.38172
C	-4.35892	-0.78361	-1.98311
C	-5.61747	-1.44197	0.46901
C	-5.12405	-1.88561	-1.87010
C	-5.76817	-2.25002	-0.58948
H	-6.08661	-1.70197	1.40907
H	-5.27085	-2.53353	-2.72319
C	-4.74248	0.70199	1.62099
H	-4.12131	1.60233	1.43880
C	-6.13127	1.22083	2.01112
H	-6.59032	1.75568	1.15215
H	-6.04587	1.93448	2.85838
H	-6.80238	0.39118	2.31787
C	-4.08519	-0.04132	2.78891
H	-3.11533	-0.46686	2.46163
H	-4.72870	-0.86647	3.15962
H	-3.89323	0.66053	3.62850
C	-3.69144	-0.46364	-3.32021
H	-2.98487	0.38321	-3.20516

C	-4.73540	-0.03376	-4.35702
H	-4.23375	0.26463	-5.30232
H	-5.31012	0.83855	-3.97933
H	-5.44383	-0.86095	-4.57615
C	-2.85274	-1.63900	-3.84589
H	-2.18235	-2.02152	-3.05024
H	-2.22453	-1.30317	-4.69852
H	-3.49548	-2.47250	-4.19903
C	-6.57745	-3.52856	-0.47135
H	-6.97913	-3.63033	0.56215
C	-5.69705	-4.75654	-0.73096
H	-4.82763	-4.75421	-0.03879
H	-5.32283	-4.76900	-1.77661
H	-6.27925	-5.68595	-0.55363
C	-7.78286	-3.50468	-1.41822
H	-8.40343	-2.60435	-1.22110
H	-8.41388	-4.40384	-1.25215
H	-7.46023	-3.49287	-2.48079
C	6.57526	-0.93293	-2.55496
H	7.04847	-1.66714	-1.86439
C	5.95596	-1.73730	-3.70364
H	5.14524	-2.39000	-3.31579
H	6.72681	-2.38433	-4.17399
H	5.53443	-1.06684	-4.48228
C	7.69221	-0.01483	-3.06434
H	8.12222	0.56525	-2.21990
H	7.31310	0.69381	-3.83073
H	8.50497	-0.62157	-3.51767
H	0.02863	-3.00665	-0.39006
C	1.71597	-4.09980	-0.78455
C	0.73105	-3.77068	1.40157
C	1.66409	-3.81289	-2.16343
C	2.80963	-4.81609	-0.23418
C	1.82454	-4.50052	1.96877
C	2.69234	-4.25238	-2.97587
H	0.84069	-3.21953	-2.54658
C	3.83587	-5.26074	-1.09788
C	2.81221	-5.01757	1.17770
H	1.84275	-4.66141	3.03851
C	3.77848	-4.98426	-2.44948
H	2.66702	-4.02169	-4.03636
H	4.67306	-5.81150	-0.67865
H	3.62146	-5.58989	1.62349
H	4.57295	-5.31826	-3.10883
C	-0.22748	-3.03242	2.18122
C	-0.22781	-3.13612	3.71367
H	-0.14821	-4.17690	4.05541
H	-1.20383	-2.78921	4.06136
N	0.80587	-2.38045	4.39988
N	0.71677	-3.65370	0.04927
C	0.85042	-0.98545	4.44404
C	-0.24403	-0.18564	4.07478
C	2.01584	-0.34594	4.90885
C	-0.16362	1.20296	4.15560
H	-1.14717	-0.64326	3.69547
C	2.08525	1.04114	4.98326
H	2.87213	-0.94799	5.20584
C	0.99608	1.83123	4.60754
H	-1.02092	1.79492	3.84650
H	3.00107	1.50758	5.33612

H	1.05171	2.91307	4.66910
H	1.70640	-2.82843	4.47413
H	0.16895	-1.72442	1.68136
Cl	-1.92265	-3.14380	1.51908

Catalyst (TRIP, 3)-Enamine protonation TS (pro-S) alternative conformation
Electronic energy (Hartrees): -1870.52549
Free Energy at 1M concentration and 253 K (Hartrees): -1869.308631
Coordinates:

P	-0.19161	-0.38154	-0.33362
O	-1.56692	0.30900	0.23246
O	0.72925	0.80957	-0.97096
O	-0.41709	-1.42133	-1.38562
O	0.50587	-0.83956	0.97305
C	-2.01767	1.42907	-0.41892
C	-3.38101	1.40274	-1.00323
C	-3.89863	2.50877	-1.55453
H	-4.89774	2.49113	-1.97432
C	-3.10323	3.74991	-1.62199
C	-3.65792	4.93827	-2.31040
H	-4.65780	4.90919	-2.72537
C	-2.92558	6.05089	-2.42627
H	-3.33096	6.91802	-2.93149
C	-1.55661	6.09501	-1.87917
H	-0.95972	6.98997	-1.99907
C	-1.04825	5.02926	-1.24999
H	-0.03206	5.07808	-0.88306
C	-1.86207	3.79302	-1.08323
C	-1.32006	2.58951	-0.37952
C	0.98369	1.81948	-0.07790
C	2.37543	2.04815	0.37961
C	2.62629	2.98381	1.30708
H	3.64418	3.17815	1.62388
C	1.51967	3.77705	1.88232
C	1.79407	4.74066	2.97255
H	2.80465	4.87606	3.33720
C	0.79321	5.43999	3.51855
H	0.99354	6.13953	4.31970
C	-0.58940	5.25292	3.03771
H	-1.39678	5.80887	3.49649
C	-0.84643	4.39403	2.04447
H	-1.86919	4.25791	1.71841
C	0.25853	3.62483	1.41319
C	-0.00528	2.64820	0.31604
C	3.49139	1.34514	-0.29522
C	3.85739	1.74679	-1.69781
C	4.16710	0.33458	0.31824
C	4.82248	1.06364	-2.34178
C	5.27121	-0.34279	-0.41189
C	5.56730	-0.02858	-1.68000
H	5.08774	1.32708	-3.35663
H	5.83081	-1.12364	0.08601
C	3.82013	-0.16599	1.71891
H	2.88503	0.29903	2.09402
C	3.57007	-1.68027	1.71705
H	2.88270	-1.94978	0.88981
H	3.10757	-1.99264	2.67473
H	4.51238	-2.25314	1.59261
C	4.92896	0.20039	2.71036
H	5.89271	-0.27237	2.42522

H	4.65716	-0.14063	3.73161
H	5.06482	1.30238	2.73733
C	3.17621	2.92227	-2.39996
H	2.43532	3.40367	-1.73186
C	2.40324	2.45962	-3.64195
H	1.68482	1.65816	-3.36896
H	3.09042	2.07284	-4.42367
H	1.82750	3.30865	-4.06858
C	4.18577	4.02154	-2.75649
H	4.91141	3.67364	-3.52146
H	4.74290	4.33388	-1.84730
H	3.65322	4.90990	-3.15860
C	-4.17376	0.15313	-0.96606
C	-4.80358	-0.23779	0.17614
C	-4.28960	-0.68205	-2.21075
C	-5.57360	-1.50810	0.17581
C	-5.03281	-1.80468	-2.17570
C	-5.68642	-2.25751	-0.93118
H	-6.05153	-1.83280	1.08995
H	-5.15567	-2.40425	-3.06803
C	-4.77123	0.58416	1.46523
H	-4.16520	1.50545	1.34821
C	-6.17936	1.05236	1.85020
H	-6.62945	1.62654	1.01214
H	-6.12893	1.71761	2.73863
H	-6.83983	0.19325	2.09235
C	-4.12619	-0.21068	2.60597
H	-3.13911	-0.59614	2.28134
H	-4.75967	-1.06909	2.91297
H	-3.97109	0.44606	3.48854
C	-3.61210	-0.26719	-3.51642
H	-2.92857	0.58806	-3.34145
C	-4.65194	0.19871	-4.54173
H	-4.14479	0.56630	-5.45930
H	-5.25412	1.03199	-4.12105
H	-5.33559	-0.63092	-4.82164
C	-2.73704	-1.38837	-4.09770
H	-2.06329	-1.79574	-3.31743
H	-2.11068	-0.98883	-4.92379
H	-3.35349	-2.21786	-4.50310
C	-6.45823	-3.56436	-0.92659
H	-6.42111	-4.01222	-1.94437
C	-7.93793	-3.33826	-0.59222
H	-8.37689	-2.59573	-1.29255
H	-8.06357	-2.96948	0.44781
H	-8.50066	-4.29023	-0.69832
C	-5.82273	-4.58028	0.03113
H	-4.75199	-4.72790	-0.22678
H	-6.33922	-5.55988	-0.05677
H	-5.89360	-4.23788	1.08533
C	6.65508	-0.78139	-2.42421
H	7.10252	-1.55662	-1.76187
C	6.07491	-1.51474	-3.63896
H	5.25166	-2.18801	-3.31819
H	6.86122	-2.13404	-4.12088
H	5.67979	-0.79819	-4.38992
C	7.79090	0.16065	-2.83946
H	8.19126	0.68947	-1.94809
H	7.44066	0.91367	-3.57669
H	8.61846	-0.42178	-3.29785

H	0.05260	-2.97333	-0.62586
C	1.74850	-4.05964	-1.00214
C	0.66897	-3.83497	1.15257
C	1.76414	-3.69599	-2.36360
C	2.80797	-4.81899	-0.44274
C	1.72669	-4.60991	1.72746
C	2.82502	-4.10240	-3.15109
H	0.96572	-3.07158	-2.75040
C	3.86845	-5.22869	-1.28192
C	2.74296	-5.09781	0.95454
H	1.69226	-4.83067	2.78586
C	3.87754	-4.87651	-2.61707
H	2.85217	-3.81211	-4.19679
H	4.67911	-5.81307	-0.85608
H	3.52321	-5.70641	1.40394
H	4.69858	-5.18370	-3.25660
C	-0.31454	-3.12483	1.92602
C	-0.38802	-3.29845	3.44974
H	-0.38149	-4.35680	3.74402
H	-1.35890	-2.91126	3.76971
N	0.65189	-2.63356	4.21270
N	0.71626	-3.64748	-0.19138
C	0.75846	-1.25157	4.36674
C	1.91126	-0.71365	4.97171
C	-0.25923	-0.36669	3.97175
C	2.04245	0.65789	5.16006
H	2.70873	-1.38301	5.28791
C	-0.11675	1.00549	4.16812
H	-1.14622	-0.74203	3.48076
C	1.02950	1.53327	4.76091
H	2.94660	1.04484	5.62219
H	-0.91445	1.66558	3.83812
H	1.13379	2.60274	4.91148
H	1.51310	-3.14130	4.34049
H	0.12066	-1.79766	1.50942
Cl	-1.98093	-3.18520	1.18867

Catalyst (TRIP, 3)-Enamine protonation TS (pro-S) alternative conformation
Electronic energy (Hartrees): -1870.525488
Free Energy at 1M concentration and 253 K (Hartrees): -1869.308568
Coordinates:

P	-0.19292	-0.38360	-0.32771
O	-1.56834	0.30871	0.23569
O	0.72953	0.80596	-0.96594
O	-0.41837	-1.42436	-1.37885
O	0.50315	-0.84089	0.97993
C	-2.01693	1.42892	-0.41698
C	-3.37926	1.40362	-1.00371
C	-3.89523	2.51011	-1.55565
H	-4.89359	2.49316	-1.97727
C	-3.09877	3.75061	-1.62193
C	-3.65154	4.93935	-2.31125
H	-4.65098	4.91115	-2.72735
C	-2.91799	6.05123	-2.42655
H	-3.32194	6.91865	-2.93243
C	-1.54955	6.09410	-1.87802
H	-0.95159	6.98839	-1.99760
C	-1.04295	5.02800	-1.24802
H	-0.02703	5.07589	-0.88023
C	-1.85823	3.79262	-1.08170

C	-1.31825	2.58871	-0.37699
C	0.98461	1.81683	-0.07410
C	2.37682	2.04662	0.38143
C	2.62850	2.98344	1.30751
H	3.64672	3.17854	1.62281
C	1.52232	3.77712	1.88299
C	1.79768	4.74248	2.97143
H	2.80862	4.87870	3.33478
C	0.79723	5.44249	3.51735
H	0.99825	6.14336	4.31717
C	-0.58587	5.25429	3.03831
H	-1.39290	5.81075	3.49710
C	-0.84375	4.39380	2.04667
H	-1.86679	4.25693	1.72179
C	0.26074	3.62400	1.41536
C	-0.00390	2.64627	0.31941
C	3.49227	1.34370	-0.29426
C	3.85703	1.74517	-1.69721
C	4.16874	0.33353	0.31889
C	4.82209	1.06241	-2.34165
C	5.27260	-0.34361	-0.41184
C	5.56786	-0.02935	-1.68013
H	5.08650	1.32573	-3.35675
H	5.83277	-1.12420	0.08584
C	3.82269	-0.16686	1.71981
H	2.88814	0.29860	2.09570
C	3.57170	-1.68091	1.71795
H	2.88247	-1.94953	0.89195
H	3.11079	-1.99324	2.67638
H	4.51338	-2.25436	1.59147
C	4.93241	0.19892	2.71048
H	5.89550	-0.27514	2.42522
H	4.66062	-0.14103	3.73210
H	5.06951	1.30079	2.73662
C	3.17444	2.91989	-2.39926
H	2.43386	3.40111	-1.73070
C	2.40057	2.45610	-3.64027
H	1.68282	1.65444	-3.36610
H	3.08726	2.06919	-4.42237
H	1.82401	3.30457	-4.06688
C	4.18294	4.01966	-2.75728
H	4.90804	3.67190	-3.52284
H	4.74077	4.33275	-1.84878
H	3.64940	4.90749	-3.15925
C	-4.17261	0.15433	-0.96916
C	-4.80505	-0.23716	0.17137
C	-4.28631	-0.67971	-2.21482
C	-5.57585	-1.50699	0.16834
C	-5.03031	-1.80189	-2.18230
C	-5.68687	-2.25535	-0.93956
H	-6.05588	-1.83219	1.08121
H	-5.15166	-2.40062	-3.07541
C	-4.77464	0.58357	1.46128
H	-4.16815	1.50480	1.34616
C	-6.18324	1.05181	1.84443
H	-6.63185	1.62686	1.00616
H	-6.13401	1.71627	2.73353
H	-6.84434	0.19269	2.08481
C	-4.13165	-0.21264	2.60220
H	-3.14425	-0.59810	2.27856

H	-4.76590	-1.07114	2.90733
H	-3.97762	0.44316	3.48567
C	-3.60575	-0.26418	-3.51869
H	-2.92217	0.59060	-3.34157
C	-4.64309	0.20304	-4.54593
H	-4.13374	0.57114	-5.46208
H	-5.24581	1.03626	-4.12592
H	-5.32651	-0.62604	-4.82805
C	-2.72996	-1.38530	-4.09891
H	-2.05783	-1.79331	-3.31758
H	-2.10186	-0.98551	-4.92355
H	-3.34589	-2.21436	-4.50598
C	-6.45967	-3.56161	-0.93778
H	-6.42057	-4.00869	-1.95582
C	-7.93994	-3.33463	-0.60663
H	-8.37674	-2.59127	-1.30744
H	-8.06767	-2.96647	0.43337
H	-8.50316	-4.28610	-0.71468
C	-5.82715	-4.57878	0.02060
H	-4.75588	-4.72687	-0.23483
H	-6.34407	-5.55796	-0.06942
H	-5.90035	-4.23726	1.07493
C	6.65569	-0.78162	-2.42483
H	7.10373	-1.55670	-1.76273
C	6.07541	-1.51513	-3.63944
H	5.25246	-2.18864	-3.31846
H	6.86178	-2.13418	-4.12158
H	5.67986	-0.79869	-4.39029
C	7.79091	0.16099	-2.84044
H	8.19145	0.68982	-1.94916
H	7.43997	0.91400	-3.57736
H	8.61850	-0.42100	-3.29932
H	0.05284	-2.97519	-0.62053
C	1.74982	-4.05925	-0.99866
C	0.67195	-3.83679	1.15704
C	1.76354	-3.69535	-2.36009
C	2.81103	-4.81706	-0.44046
C	1.73144	-4.61018	1.73077
C	2.82428	-4.09996	-3.14868
H	0.96377	-3.07212	-2.74597
C	3.87139	-5.22487	-1.28073
C	2.74783	-5.09625	0.95681
H	1.69842	-4.83124	2.78918
C	3.87859	-4.87246	-2.61581
H	2.84992	-3.80949	-4.19437
H	4.68342	-5.80799	-0.85578
H	3.52954	-5.70358	1.40537
H	4.69949	-5.17819	-3.25621
C	-0.31204	-3.12862	1.93190
C	-0.38296	-3.30469	3.45553
H	-0.36840	-4.36330	3.74852
H	-1.35617	-2.92518	3.77734
N	0.65306	-2.63323	4.21848
N	0.71774	-3.64878	-0.18683
C	0.75096	-1.24985	4.36804
C	-0.27679	-0.37389	3.97944
C	1.90444	-0.70160	4.96218
C	-0.14342	0.99985	4.17105
H	-1.16528	-0.75774	3.49779
C	2.02657	0.67143	5.14599

H	2.70958	-1.36397	5.27362
C	1.00362	1.53790	4.75299
H	-0.94886	1.65294	3.84596
H	2.93150	1.06657	5.59958
H	1.10072	2.60851	4.90015
H	1.52009	-3.13322	4.33797
H	0.11978	-1.80036	1.51593
Cl	-1.97904	-3.19124	1.19595

Catalyst (TRIP, 3)-Enamine protonation TS (pro-S) alternative conformation
Electronic energy (Hartrees): -1870.526335
Free Energy at 1M concentration and 253 K (Hartrees): -1869.308433
Coordinates:

P	-0.04714	0.02002	0.29815
O	1.08776	0.99379	-0.35280
O	-1.40066	0.91869	0.48566
O	0.27316	-0.51737	1.65491
O	-0.26892	-0.99847	-0.85350
C	1.27205	2.15375	0.35027
C	2.56605	2.37750	1.03540
C	2.77137	3.51148	1.71963
H	3.72551	3.69296	2.19963
C	1.69841	4.52448	1.81838
C	1.90432	5.73371	2.64817
H	2.84581	5.88781	3.16061
C	0.92758	6.63912	2.77076
H	1.07842	7.52164	3.37893
C	-0.36368	6.43239	2.08722
H	-1.15697	7.15876	2.20816
C	-0.55864	5.34639	1.33025
H	-1.52046	5.20360	0.85583
C	0.52772	4.34441	1.15996
C	0.33606	3.12545	0.31786
C	-1.63830	1.87156	-0.47121
C	-2.89073	1.77583	-1.26025
C	-3.21145	2.74868	-2.12469
H	-4.13762	2.69550	-2.68465
C	-2.29537	3.88461	-2.34567
C	-2.61908	4.89550	-3.37876
H	-3.54468	4.82606	-3.93665
C	-1.76829	5.89573	-3.63049
H	-2.00461	6.63259	-4.38725
C	-0.49680	5.98879	-2.88887
H	0.19987	6.78528	-3.11658
C	-0.19774	5.08454	-1.94915
H	0.75371	5.15820	-1.44024
C	-1.15016	3.98468	-1.63108
C	-0.84596	2.96679	-0.57850
C	-3.80530	0.62202	-1.07356
C	-4.69810	0.57663	0.13533
C	-3.87961	-0.36904	-2.00639
C	-5.59988	-0.41795	0.24361
C	-4.92847	-1.41287	-1.86925
C	-5.75131	-1.44190	-0.81241
H	-6.24676	-0.47122	1.10871
H	-5.03716	-2.15461	-2.64973
C	-2.93458	-0.45016	-3.20499
H	-2.09072	0.26264	-3.09252
C	-2.29589	-1.84392	-3.32744
H	-1.94030	-2.20106	-2.33951

H	-1.42697	-1.80310	-4.01860
H	-3.01728	-2.58821	-3.72529
C	-3.67051	-0.08104	-4.49753
H	-4.49729	-0.79399	-4.70285
H	-2.96488	-0.09778	-5.35545
H	-4.09466	0.94163	-4.41904
C	-4.60625	1.62602	1.24182
H	-3.83497	2.38310	1.00072
C	-4.18677	0.99160	2.57526
H	-3.23902	0.42720	2.44741
H	-4.96562	0.29805	2.95667
H	-4.01795	1.78286	3.33642
C	-5.92209	2.40130	1.38817
H	-6.74249	1.74424	1.74593
H	-6.21335	2.84137	0.41037
H	-5.79500	3.23007	2.11712
C	3.64943	1.37907	0.87801
C	4.33169	1.28174	-0.29779
C	3.96002	0.43319	2.00504
C	5.32352	0.18884	-0.46820
C	4.90440	-0.50944	1.82209
C	5.59091	-0.67278	0.52221
H	5.83179	0.08387	-1.41761
H	5.17000	-1.17635	2.63040
C	4.13773	2.26556	-1.45415
H	3.39104	3.04696	-1.20592
C	5.44055	3.01689	-1.75357
H	5.80725	3.52102	-0.83378
H	5.26194	3.79458	-2.52675
H	6.22893	2.32889	-2.12495
C	3.61657	1.55487	-2.70892
H	2.68461	0.99940	-2.47545
H	4.36778	0.84477	-3.11264
H	3.38303	2.30133	-3.49785
C	3.26875	0.55468	3.36234
H	2.38516	1.21991	3.28091
C	4.21468	1.18611	4.38955
H	3.68717	1.32977	5.35668
H	4.55967	2.17919	4.03185
H	5.10292	0.54065	4.55971
C	2.73534	-0.79361	3.87628
H	2.20263	-1.33650	3.07076
H	2.02404	-0.62400	4.71270
H	3.55649	-1.44044	4.25066
C	6.57451	-1.80849	0.30760
H	6.96325	-1.78283	-0.73556
C	5.88820	-3.16677	0.49436
H	4.99649	-3.23587	-0.16342
H	5.56624	-3.31369	1.54704
H	6.58591	-3.98637	0.21969
C	7.78329	-1.67029	1.23939
H	8.26584	-0.68048	1.09143
H	8.53146	-2.45879	1.00982
H	7.48373	-1.76815	2.30429
C	-6.83460	-2.50049	-0.71025
H	-6.81278	-3.15526	-1.61061
C	-6.60190	-3.40470	0.50347
H	-5.60323	-3.88161	0.42639
H	-7.36709	-4.20941	0.53291
H	-6.65612	-2.82962	1.45205

C	-8.22616	-1.85900	-0.65976
H	-8.37220	-1.19738	-1.54035
H	-8.36002	-1.26013	0.26589
H	-9.00877	-2.64710	-0.68485
H	-0.16590	-2.21046	1.86395
C	-1.94182	-3.02127	2.51252
C	-0.66373	-3.73315	0.57938
C	-2.05130	-2.13037	3.59765
C	-2.98917	-3.92756	2.21173
C	-1.73032	-4.63265	0.24609
C	-3.19135	-2.16378	4.37962
H	-1.25630	-1.41409	3.77604
C	-4.12770	-3.95128	3.04696
C	-2.83132	-4.73943	1.04718
H	-1.63347	-5.25381	-0.63388
C	-4.23006	-3.08039	4.11458
H	-3.28838	-1.46893	5.20800
H	-4.92568	-4.65512	2.82844
H	-3.61066	-5.45510	0.79917
H	-5.11225	-3.09384	4.74612
C	0.45871	-3.45173	-0.26567
C	0.71625	-4.22064	-1.55375
H	-0.25264	-4.47651	-2.00114
H	1.24334	-5.16153	-1.36878
N	1.47379	-3.44208	-2.52973
N	-0.82157	-3.00327	1.71500
C	2.86453	-3.53519	-2.67344
C	3.55668	-4.75620	-2.58644
C	3.59872	-2.37710	-2.99026
C	4.93466	-4.80244	-2.79157
H	3.02061	-5.67455	-2.37030
C	4.97103	-2.43744	-3.20868
H	3.07806	-1.42542	-3.04975
C	5.65584	-3.65004	-3.10587
H	5.44686	-5.75750	-2.71067
H	5.51124	-1.52552	-3.44732
H	6.72882	-3.69455	-3.26299
H	1.14753	-2.48324	-2.57293
H	0.04112	-2.08631	-0.62163
Cl	1.99975	-3.12907	0.62502

Catalyst (TRIP, 3)-Enamine protonation TS (pro-S) alternative conformation
Electronic energy (Hartrees): -1870.526335
Free Energy at 1M concentration and 253 K (Hartrees): -1869.30843
Coordinates:

P	0.04783	0.02020	-0.29822
O	-1.08688	0.99413	0.35282
O	1.40149	0.91864	-0.48563
O	-0.27262	-0.51690	-1.65507
O	0.26950	-0.99852	0.85322
C	-1.27120	2.15395	-0.35047
C	-2.56521	2.37759	-1.03567
C	-2.77047	3.51141	-1.72018
H	-3.72461	3.69283	-2.20022
C	-1.69745	4.52432	-1.81922
C	-1.90329	5.73333	-2.64935
H	-2.84479	5.88735	-3.16182
C	-0.92649	6.63863	-2.77224
H	-1.07728	7.52099	-3.38067
C	0.36479	6.43200	-2.08867

H	1.15813	7.15828	-2.20984
C	0.55968	5.34621	-1.33139
H	1.52151	5.20348	-0.85695
C	-0.52674	4.34436	-1.16078
C	-0.33514	3.12561	-0.31835
C	1.63914	1.87173	0.47102
C	2.89152	1.77605	1.26015
C	3.21239	2.74913	2.12427
H	4.13852	2.69597	2.68429
C	2.29646	3.88526	2.34490
C	2.62029	4.89641	3.37770
H	3.54590	4.82704	3.93560
C	1.76960	5.89680	3.62917
H	2.00600	6.63385	4.38572
C	0.49810	5.98978	2.88754
H	-0.19849	6.78641	3.11505
C	0.19894	5.08530	1.94808
H	-0.75252	5.15891	1.43917
C	1.15124	3.98525	1.63030
C	0.84691	2.96708	0.57801
C	3.80578	0.62189	1.07396
C	4.69832	0.57556	-0.13509
C	3.87980	-0.36880	2.00721
C	5.59949	-0.41959	-0.24320
C	4.92800	-1.41330	1.87020
C	5.75050	-1.44329	0.81312
H	6.24618	-0.47353	-1.10840
H	5.03643	-2.15483	2.65094
C	2.93506	-0.44889	3.20613
H	2.09156	0.26433	3.09361
C	2.29560	-1.84223	3.32939
H	1.93942	-2.19958	2.34174
H	1.42696	-1.80062	4.02086
H	3.01670	-2.58677	3.72730
C	3.67157	-0.07962	4.49829
H	4.49800	-0.79297	4.70369
H	2.96619	-0.09557	5.35643
H	4.09630	0.94277	4.41922
C	4.60696	1.62468	-1.24189
H	3.83611	2.38224	-1.00095
C	4.18708	0.99011	-2.57513
H	3.23910	0.42613	-2.44707
H	4.96560	0.29614	-2.95646
H	4.01852	1.78125	-3.33647
C	5.92321	2.39921	-1.38853
H	6.74325	1.74158	-1.74609
H	6.21474	2.83944	-0.41089
H	5.79655	3.22779	-2.11775
C	-3.64866	1.37927	-0.87805
C	-4.33093	1.28226	0.29778
C	-3.95943	0.43324	-2.00491
C	-5.32289	0.18952	0.46840
C	-4.90391	-0.50926	-1.82176
C	-5.59043	-0.67225	-0.52185
H	-5.83119	0.08482	1.41783
H	-5.16963	-1.17627	-2.62995
C	-4.13690	2.26635	1.45390
H	-3.39013	3.04763	1.20551
C	-5.43967	3.01789	1.75310
H	-5.80631	3.52181	0.83317

H	-5.26100	3.79576	2.52608
H	-6.22812	2.33005	2.12463
C	-3.61584	1.55592	2.70886
H	-2.68394	1.00029	2.47554
H	-4.36715	0.84601	3.11274
H	-3.38223	2.30256	3.49760
C	-3.26830	0.55450	-3.36231
H	-2.38468	1.21970	-3.28109
C	-4.21433	1.18582	-4.38952
H	-3.68692	1.32934	-5.35672
H	-4.55927	2.17895	-4.03191
H	-5.10260	0.54035	-4.55950
C	-2.73501	-0.79388	-3.87613
H	-2.20207	-1.33661	-3.07065
H	-2.02391	-0.62442	-4.71276
H	-3.55624	-1.44081	-4.25019
C	-6.57429	-1.80771	-0.30704
H	-6.96306	-1.78175	0.73610
C	-5.88827	-3.16618	-0.49351
H	-4.99667	-3.23537	0.16440
H	-5.56622	-3.31335	-1.54613
H	-6.58620	-3.98558	-0.21879
C	-7.78302	-1.66944	-1.23890
H	-8.26533	-0.67948	-1.09118
H	-8.53138	-2.45771	-1.00917
H	-7.48345	-1.76761	-2.30377
C	6.83294	-2.50274	0.71095
H	6.81085	-3.15727	1.61148
C	6.59921	-3.40709	-0.50249
H	5.60012	-3.88309	-0.42511
H	7.36368	-4.21247	-0.53185
H	6.65377	-2.83231	-1.45124
C	8.22499	-1.86234	0.65992
H	8.37178	-1.20063	1.54031
H	8.35905	-1.26379	-0.26591
H	9.00699	-2.65104	0.68498
H	0.16537	-2.21024	-1.86392
C	1.94069	-3.02224	-2.51259
C	0.66210	-3.73348	-0.57957
C	2.05078	-2.13130	-3.59763
C	2.98745	-3.92924	-2.21187
C	1.72800	-4.63388	-0.24647
C	3.19087	-2.16531	-4.37952
H	1.25623	-1.41450	-3.77598
C	4.12603	-3.95353	-3.04700
C	2.82898	-4.74124	-1.04750
H	1.63062	-5.25518	0.63335
C	4.22900	-3.08258	-4.11451
H	3.28838	-1.47043	-5.20782
H	4.92360	-4.65783	-2.82846
H	3.60781	-5.45749	-0.79961
H	5.11124	-3.09647	-4.74597
C	-0.46005	-3.45122	0.26562
C	-0.71802	-4.22018	1.55357
H	0.25072	-4.47672	2.00090
H	-1.24572	-5.16069	1.36845
N	-1.47510	-3.44124	2.52961
H	-1.14861	-2.48248	2.57257
C	-2.86580	-3.53405	2.67381
C	-3.59962	-2.37577	2.99081

C	-3.55826	-4.75490	2.58713
C	-4.97187	-2.43577	3.20966
H	-3.07870	-1.42423	3.05011
C	-4.93620	-4.80079	2.79269
H	-3.02249	-5.67340	2.37090
C	-5.65701	-3.64821	3.10712
H	-5.51178	-1.52370	3.44840
H	-5.44865	-5.75574	2.71201
H	-6.72996	-3.69245	3.26458
H	-0.04147	-2.08612	0.62155
N	0.82044	-3.00357	-1.71509
Cl	-2.00097	-3.12751	-0.62490

Catalyst (TRIP, 3)-Enamine protonation TS (pro-S) alternative conformation
Electronic energy (Hartrees): -1870.526593
Free Energy at 1M concentration and 253 K (Hartrees): -1869.308385
Coordinates:

P	0.51092	-0.07108	0.22241
O	1.77452	0.07536	-0.79524
O	0.07126	1.44578	0.63796
O	0.83516	-0.78768	1.49165
O	-0.60728	-0.66314	-0.68379
C	2.81352	0.76549	-0.22412
C	4.06590	0.04319	0.11340
C	5.09088	0.71003	0.66312
H	6.01801	0.19590	0.88585
C	4.95771	2.14314	0.99531
C	6.05953	2.83792	1.70027
H	6.97540	2.30895	1.93298
C	5.92670	4.12003	2.05647
H	6.73151	4.62631	2.57362
C	4.67623	4.84441	1.75973
H	4.56541	5.87431	2.07372
C	3.67718	4.22779	1.11783
H	2.75954	4.77042	0.93417
C	3.82278	2.81193	0.68281
C	2.72587	2.10264	-0.04351
C	0.29124	2.44163	-0.28027
C	-0.87674	3.22257	-0.75855
C	-0.68589	4.35290	-1.45477
H	-1.53669	4.93055	-1.79639
C	0.67148	4.75092	-1.87311
C	0.84839	5.92861	-2.75397
H	-0.00454	6.53869	-3.02440
C	2.06039	6.24050	-3.22423
H	2.18822	7.09976	-3.86984
C	3.22397	5.40178	-2.88155
H	4.19697	5.64138	-3.29078
C	3.07566	4.33889	-2.08258
H	3.93852	3.72143	-1.87344
C	1.73997	4.00111	-1.51611
C	1.55018	2.83666	-0.59514
C	-2.25240	2.70514	-0.54924
C	-2.86574	2.74351	0.82443
C	-2.96670	2.19453	-1.59436
C	-4.11390	2.26605	0.99562
C	-4.36521	1.75248	-1.36498
C	-4.91011	1.75849	-0.14217
H	-4.56946	2.27907	1.97640
H	-4.95390	1.41961	-2.20870

C	-2.39602	2.05125	-3.00822
H	-1.31728	2.30452	-3.03970
C	-2.48757	0.60345	-3.51070
H	-2.00778	-0.08461	-2.78629
H	-1.95485	0.50531	-4.48069
H	-3.54037	0.28787	-3.66291
C	-3.10429	3.00550	-3.97673
H	-4.17840	2.74460	-4.08381
H	-2.62714	2.95431	-4.97870
H	-3.02588	4.05059	-3.60977
C	-2.12428	3.33699	2.02103
H	-1.10980	3.66884	1.72458
C	-1.93694	2.30005	3.13868
H	-1.47540	1.37710	2.73204
H	-2.90517	2.03173	3.61118
H	-1.26568	2.70551	3.92536
C	-2.83906	4.58745	2.54852
H	-3.83980	4.33745	2.95997
H	-2.96216	5.32757	1.72896
H	-2.23590	5.05865	3.35383
C	4.19780	-1.40173	-0.19358
C	4.36707	-1.82838	-1.47695
C	4.18504	-2.40200	0.93168
C	4.51510	-3.28344	-1.73981
C	4.38242	-3.70402	0.64976
C	4.53245	-4.17758	-0.74015
H	4.62174	-3.62482	-2.76038
H	4.41248	-4.43516	1.44690
C	4.45445	-0.87294	-2.66868
H	4.35100	0.18503	-2.35535
C	5.82525	-0.97220	-3.34909
H	6.63085	-0.78507	-2.60699
H	5.90805	-0.20697	-4.15041
H	5.98116	-1.97304	-3.80384
C	3.32409	-1.13749	-3.67174
H	2.33925	-1.09240	-3.16074
H	3.43199	-2.13328	-4.15047
H	3.33560	-0.36341	-4.46864
C	3.97375	-1.97320	2.38338
H	3.64416	-0.91491	2.42379
C	5.28449	-2.06818	3.17300
H	5.13464	-1.68995	4.20681
H	6.07027	-1.45123	2.68939
H	5.64109	-3.11899	3.22667
C	2.86735	-2.78761	3.07575
H	1.97642	-2.87228	2.42160
H	2.56369	-2.28646	4.01957
H	3.21348	-3.81219	3.32724
C	4.68473	-5.66375	-1.00919
H	4.67511	-6.21161	-0.04086
C	6.02656	-5.97471	-1.68397
H	6.86224	-5.57261	-1.07196
H	6.07859	-5.52943	-2.70010
H	6.16019	-7.07393	-1.77423
C	3.51053	-6.20132	-1.83695
H	2.54928	-5.96315	-1.33278
H	3.58772	-7.30540	-1.93329
H	3.50099	-5.75860	-2.85554
C	-6.33056	1.26569	0.06660
H	-6.77404	0.95780	-0.90729

C	-6.34837	0.03112	0.97302
H	-5.71439	-0.76722	0.53308
H	-7.38363	-0.36107	1.06373
H	-5.97106	0.27419	1.98887
C	-7.22321	2.37717	0.63040
H	-7.18383	3.26890	-0.03120
H	-6.90278	2.67266	1.65174
H	-8.27690	2.02869	0.68015
H	-0.36437	-1.84019	2.17542
C	-1.71699	-1.42538	3.66624
C	-2.20549	-2.47527	1.53801
C	-0.74203	-0.88094	4.52431
C	-3.09181	-1.36577	4.00432
C	-3.60229	-2.41673	1.86398
C	-1.14372	-0.29538	5.71107
H	0.29932	-0.90009	4.22186
C	-3.46554	-0.76992	5.22846
C	-4.01841	-1.89710	3.05591
H	-4.30999	-2.78885	1.13720
C	-2.50609	-0.24263	6.07181
H	-0.39735	0.13538	6.37127
H	-4.51907	-0.72553	5.48967
H	-5.07792	-1.88407	3.29775
H	-2.79850	0.21950	7.00903
C	-1.70268	-2.90035	0.26389
C	-2.64751	-3.54593	-0.73983
H	-3.15761	-4.42036	-0.30809
H	-2.04548	-3.91801	-1.58130
N	-3.66024	-2.58840	-1.16338
N	-1.34429	-2.00266	2.47446
C	-4.68750	-2.94270	-2.03960
C	-5.54129	-1.93460	-2.52868
C	-4.93741	-4.27234	-2.42311
C	-6.60204	-2.24396	-3.36981
H	-5.36627	-0.90743	-2.22681
C	-6.01408	-4.57134	-3.26046
H	-4.29144	-5.07280	-2.08132
C	-6.85259	-3.56841	-3.74264
H	-7.24157	-1.44409	-3.73291
H	-6.18686	-5.60601	-3.54408
H	-7.68344	-3.81001	-4.39777
H	-3.29799	-1.65287	-1.29868
H	-1.10142	-1.58421	-0.30297
Cl	-0.15492	-3.84361	0.35583

Catalyst (TRIP, 3)-Enamine protonation TS (pro-S) alternative conformation
Electronic energy (Hartrees): -1870.525738
Free Energy at 1M concentration and 253 K (Hartrees): -1869.308024
Coordinates:

P	-0.03112	0.03184	0.32772
O	1.10428	0.97019	-0.37280
O	-1.36533	0.95781	0.52833
O	0.31148	-0.48030	1.68874
O	-0.29518	-1.00763	-0.79564
C	1.31846	2.14877	0.28935
C	2.62993	2.37643	0.93893
C	2.86555	3.52917	1.58070
H	3.83222	3.71247	2.03424
C	1.80942	4.56084	1.66715
C	2.05018	5.79434	2.45055

H	3.00429	5.95138	2.93815
C	1.08952	6.71824	2.56175
H	1.26586	7.61855	3.13605
C	-0.21854	6.50821	1.91195
H	-0.99838	7.25043	2.02378
C	-0.44506	5.40016	1.19688
H	-1.41847	5.25619	0.74709
C	0.62267	4.37629	1.03942
C	0.39562	3.13229	0.24409
C	-1.61639	1.88372	-0.45124
C	-2.89313	1.78494	-1.19969
C	-3.21638	2.72730	-2.09632
H	-4.15966	2.67289	-2.62696
C	-2.28456	3.83476	-2.38603
C	-2.61522	4.81023	-3.45047
H	-3.55448	4.73534	-3.98434
C	-1.75406	5.78506	-3.76010
H	-1.99553	6.49600	-4.53968
C	-0.46472	5.88516	-3.05079
H	0.23927	6.66044	-3.32456
C	-0.15913	5.01308	-2.08317
H	0.80437	5.09048	-1.59794
C	-1.12162	3.94262	-1.70179
C	-0.80910	2.96013	-0.61865
C	-3.83489	0.67110	-0.92721
C	-4.70368	0.72416	0.29885
C	-3.95955	-0.36817	-1.79952
C	-5.63760	-0.23011	0.47918
C	-5.03992	-1.36491	-1.58459
C	-5.84384	-1.30439	-0.51263
H	-6.26987	-0.21369	1.35745
H	-5.19113	-2.14231	-2.32087
C	-3.04203	-0.54905	-3.00794
H	-2.17149	0.13780	-2.94950
C	-2.45667	-1.97031	-3.06351
H	-2.09933	-2.28666	-2.06221
H	-1.59771	-1.99927	-3.76761
H	-3.21110	-2.70754	-3.41008
C	-3.78984	-0.22416	-4.30541
H	-4.64687	-0.91498	-4.45506
H	-3.10301	-0.31557	-5.17381
H	-4.17352	0.81703	-4.27630
C	-4.54918	1.82763	1.34413
H	-3.75965	2.54379	1.04414
C	-4.11561	1.25128	2.69893
H	-3.18689	0.65429	2.57877
H	-4.90409	0.60111	3.13327
H	-3.90652	2.07555	3.41367
C	-5.83414	2.65480	1.48012
H	-6.66571	2.04731	1.89503
H	-6.13715	3.05023	0.48694
H	-5.65981	3.51757	2.15808
C	3.69591	1.35807	0.79139
C	4.35336	1.21502	-0.39381
C	4.01492	0.44220	1.94051
C	5.32596	0.10283	-0.54975
C	4.94260	-0.51888	1.76870
C	5.60050	-0.73215	0.46133
H	5.81333	-0.03893	-1.50532
H	5.21450	-1.16481	2.59182

C	4.14962	2.16536	-1.57616
H	3.41913	2.96488	-1.33756
C	5.45644	2.88790	-1.92495
H	5.84889	3.41490	-1.02891
H	5.27297	3.64374	-2.71838
H	6.22735	2.17722	-2.29027
C	3.59292	1.42383	-2.79736
H	2.65847	0.88924	-2.52779
H	4.32583	0.69090	-3.19385
H	3.35336	2.14885	-3.60428
C	3.35029	0.61343	3.30564
H	2.47320	1.28669	3.21966
C	4.32230	1.26422	4.29571
H	3.81467	1.44296	5.26762
H	4.67154	2.24203	3.90187
H	5.20633	0.61377	4.46882
C	2.81075	-0.71214	3.87003
H	2.26040	-1.27471	3.09009
H	2.11419	-0.50850	4.71125
H	3.63087	-1.35564	4.25242
C	6.56141	-1.88984	0.26225
H	6.92838	-1.90292	-0.78899
C	5.85776	-3.23020	0.50578
H	4.94966	-3.30388	-0.12876
H	5.55830	-3.34016	1.56955
H	6.53553	-4.06929	0.24014
C	7.79156	-1.74256	1.16420
H	8.28670	-0.76610	0.97493
H	8.52198	-2.55030	0.94458
H	7.51288	-1.80175	2.23759
C	-6.97329	-2.29894	-0.31258
H	-7.50038	-2.06395	0.63870
C	-8.01169	-2.19309	-1.43630
H	-8.38311	-1.14877	-1.51375
H	-8.87749	-2.85376	-1.21701
H	-7.57787	-2.49359	-2.41364
C	-6.44245	-3.73112	-0.18613
H	-5.69960	-3.78287	0.63450
H	-5.96183	-4.06929	-1.12847
H	-7.27467	-4.42603	0.05567
H	-0.17307	-2.15992	1.94020
C	-1.97524	-2.88983	2.61038
C	-0.72900	-3.69301	0.69261
C	-2.05141	-1.96582	3.67044
C	-3.05432	-3.76666	2.33513
C	-1.82451	-4.56729	0.38787
C	-3.19106	-1.93670	4.45303
H	-1.23158	-1.27299	3.82778
C	-4.19278	-3.72535	3.17004
C	-2.92536	-4.61696	1.19463
H	-1.75137	-5.21489	-0.47515
C	-4.26245	-2.82207	4.21316
H	-3.26241	-1.21581	5.26155
H	-5.01587	-4.40597	2.97222
H	-3.72652	-5.31558	0.96879
H	-5.14390	-2.78596	4.84485
C	0.39273	-3.46205	-0.16893
C	0.61541	-4.26652	-1.44184
H	-0.36481	-4.51004	-1.87090
H	1.12230	-5.21528	-1.24171

N	1.37619	-3.52861	-2.44609
N	-0.85646	-2.93239	1.81150
C	2.76360	-3.64726	-2.60208
C	3.44012	-4.87399	-2.47989
C	3.50955	-2.51161	-2.96889
C	4.81454	-4.94692	-2.70062
H	2.89488	-5.77637	-2.22344
C	4.87801	-2.59893	-3.20166
H	3.00125	-1.55545	-3.05688
C	5.54740	-3.81672	-3.06454
H	5.31460	-5.90563	-2.59185
H	5.42749	-1.70376	-3.47935
H	6.61757	-3.88194	-3.23318
H	1.06372	-2.56667	-2.51457
H	0.00278	-2.09841	-0.54753
Cl	1.95111	-3.16061	0.69852

Catalyst (TRIP, 3)-Enamine protonation TS (pro-S) alternative conformation
Electronic energy (Hartrees): -1870.525738
Free Energy at 1M concentration and 253 K (Hartrees): -1869.308018
Coordinates:

P	-0.03197	0.03216	0.32841
O	1.10411	0.96984	-0.37192
O	-1.36561	0.95895	0.52873
O	0.31016	-0.48019	1.68946
O	-0.29661	-1.00717	-0.79496
C	1.31902	2.14811	0.29058
C	2.63047	2.37458	0.94066
C	2.86666	3.52684	1.58306
H	3.83334	3.70931	2.03693
C	1.81120	4.55921	1.66963
C	2.05256	5.79218	2.45366
H	3.00661	5.94833	2.94169
C	1.09252	6.71672	2.56492
H	1.26929	7.61665	3.13968
C	-0.21546	6.50792	1.91453
H	-0.99481	7.25066	2.02638
C	-0.44252	5.40036	1.19888
H	-1.41587	5.25729	0.74866
C	0.62453	4.37578	1.04137
C	0.39687	3.13228	0.24545
C	-1.61573	1.88516	-0.45078
C	-2.89213	1.78711	-1.19992
C	-3.21461	2.72993	-2.09635
H	-4.15765	2.67606	-2.62747
C	-2.28217	3.83709	-2.38529
C	-2.61193	4.81306	-3.44957
H	-3.55101	4.73879	-3.98383
C	-1.75015	5.78756	-3.75856
H	-1.99097	6.49886	-4.53803
C	-0.46104	5.88681	-3.04872
H	0.24345	6.66182	-3.32199
C	-0.15624	5.01428	-2.08125
H	0.80711	5.09105	-1.59563
C	-1.11942	3.94417	-1.70057
C	-0.80779	2.96118	-0.61761
C	-3.83422	0.67330	-0.92843
C	-4.70332	0.72575	0.29744
C	-3.95856	-0.36562	-1.80123
C	-5.63712	-0.22875	0.47719

C	-5.03859	-1.36283	-1.58672
C	-5.84270	-1.30291	-0.51488
H	-6.26963	-0.21274	1.35530
H	-5.18925	-2.14024	-2.32311
C	-3.04096	-0.54569	-3.00973
H	-2.17040	0.14109	-2.95075
C	-2.45563	-1.96694	-3.06639
H	-2.09879	-2.28434	-2.06525
H	-1.59635	-1.99525	-3.77013
H	-3.20992	-2.70380	-3.41407
C	-3.78872	-0.21984	-4.30699
H	-4.64583	-0.91047	-4.45710
H	-3.10189	-0.31074	-5.17545
H	-4.17228	0.82137	-4.27720
C	-4.54925	1.82880	1.34322
H	-3.75961	2.54509	1.04383
C	-4.11625	1.25192	2.69800
H	-3.18765	0.65469	2.57798
H	-4.90504	0.60184	3.13191
H	-3.90717	2.07593	3.41304
C	-5.83429	2.65589	1.47900
H	-6.66605	2.04820	1.89322
H	-6.13683	3.05178	0.48584
H	-5.66028	3.51835	2.15744
C	3.69590	1.35572	0.79261
C	4.35356	1.21329	-0.39255
C	4.01392	0.43855	1.94098
C	5.32518	0.10036	-0.54935
C	4.94074	-0.52324	1.76844
C	5.59871	-0.73587	0.46099
H	5.81260	-0.04093	-1.50497
H	5.21193	-1.17016	2.59102
C	4.15098	2.16503	-1.57400
H	3.42136	2.96514	-1.33466
C	5.45864	2.88647	-1.92196
H	5.85166	3.41206	-1.02534
H	5.27605	3.64338	-2.71460
H	6.22877	2.17532	-2.28803
C	3.59354	1.42534	-2.79596
H	2.65838	0.89165	-2.52703
H	4.32561	0.69188	-3.19301
H	3.35500	2.15141	-3.60225
C	3.34947	0.60941	3.30625
H	2.47283	1.28333	3.22069
C	4.32200	1.25898	4.29663
H	3.81457	1.43749	5.26868
H	4.67186	2.23678	3.90332
H	5.20561	0.60783	4.46928
C	2.80906	-0.71610	3.86998
H	2.25834	-1.27791	3.08977
H	2.11263	-0.51242	4.71130
H	3.62874	-1.36033	4.25205
C	6.55865	-1.89422	0.26100
H	6.92605	-1.90644	-0.79011
C	5.85363	-3.23421	0.50267
H	4.94593	-3.30641	-0.13263
H	5.55329	-3.34498	1.56612
H	6.53083	-4.07363	0.23667
C	7.78857	-1.74915	1.16364
H	8.28466	-0.77288	0.97580

H	8.51837	-2.55726	0.94332
H	7.50941	-1.80946	2.23686
C	-6.97136	-2.29837	-0.31489
H	-7.49935	-2.06318	0.63583
C	-8.00914	-2.19447	-1.43941
H	-8.38135	-1.15051	-1.51814
H	-8.87454	-2.85561	-1.21999
H	-7.57451	-2.49564	-2.41618
C	-6.43907	-3.72989	-0.18694
H	-5.69729	-3.78032	0.63480
H	-5.95673	-4.06802	-1.12844
H	-7.27083	-4.42562	0.05409
H	-0.17382	-2.15977	1.94054
C	-1.97531	-2.89030	2.61181
C	-0.72994	-3.69315	0.69332
C	-2.05124	-1.96616	3.67178
C	-3.05422	-3.76756	2.33728
C	-1.82526	-4.56796	0.38939
C	-3.19047	-1.93736	4.45499
H	-1.23157	-1.27302	3.82857
C	-4.19228	-3.72651	3.17274
C	-2.92556	-4.61800	1.19685
H	-1.75235	-5.21569	-0.47355
C	-4.26168	-2.82313	4.21581
H	-3.26162	-1.21641	5.26347
H	-5.01529	-4.40733	2.97530
H	-3.72656	-5.31703	0.97166
H	-5.14281	-2.78724	4.84797
C	0.39117	-3.46185	-0.16892
C	0.61320	-4.26600	-1.44213
H	-0.36724	-4.51035	-1.87022
H	1.12115	-5.21431	-1.24257
N	1.37238	-3.52730	-2.44704
H	1.05940	-2.56548	-2.51475
C	2.75965	-3.64525	-2.60473
C	3.50472	-2.50901	-2.97152
C	3.43680	-4.87180	-2.48427
C	4.87295	-2.59561	-3.20588
H	2.99591	-1.55299	-3.05816
C	4.81100	-4.94400	-2.70657
H	2.89218	-5.77460	-2.22796
C	5.54299	-3.81322	-3.07043
H	5.42177	-1.70003	-3.48356
H	5.31158	-5.90260	-2.59912
H	6.61299	-3.87786	-3.24035
H	0.00098	-2.09795	-0.54708
N	-0.85699	-2.93252	1.81226
Cl	1.95006	-3.16011	0.69748

Catalyst (TRIP, 3)-Enamine protonation TS (pro-S) alternative conformation
Electronic energy (Hartrees): -1870.524962
Free Energy at 1M concentration and 253 K (Hartrees): -1869.307988
Coordinates:

P	-0.04233	0.03289	0.30617
O	1.12694	0.96242	-0.34591
O	-1.37660	0.96787	0.44316
O	0.24512	-0.47249	1.68202
O	-0.27353	-1.01047	-0.82144
C	1.30486	2.14795	0.31498
C	2.56793	2.37345	1.05661

C	2.77310	3.54028	1.68319
H	3.70503	3.72176	2.20484
C	1.72649	4.58448	1.67465
C	1.92949	5.83267	2.44551
H	2.85407	5.99394	2.98583
C	0.97080	6.76444	2.48080
H	1.11938	7.67539	3.04610
C	-0.29848	6.54803	1.76038
H	-1.07897	7.29618	1.81222
C	-0.49029	5.42665	1.05608
H	-1.43783	5.27856	0.55551
C	0.57842	4.39400	0.98081
C	0.39150	3.13560	0.19721
C	-1.58597	1.87323	-0.56493
C	-2.82956	1.75819	-1.36536
C	-3.11972	2.68976	-2.28463
H	-4.03959	2.62495	-2.85352
C	-2.17824	3.79477	-2.55274
C	-2.46765	4.75502	-3.64274
H	-3.38607	4.67269	-4.21066
C	-1.59460	5.72515	-3.93343
H	-1.80591	6.42499	-4.73160
C	-0.33281	5.83503	-3.17744
H	0.38141	6.60646	-3.43510
C	-0.06451	4.97651	-2.18688
H	0.87960	5.06060	-1.66586
C	-1.04149	3.91190	-1.82721
C	-0.77207	2.94667	-0.71761
C	-3.76838	0.63257	-1.12964
C	-4.66160	0.65721	0.08010
C	-3.86083	-0.39848	-2.01678
C	-5.57863	-0.31714	0.23426
C	-4.92518	-1.41885	-1.83043
C	-5.74568	-1.38749	-0.77192
H	-6.22627	-0.31972	1.10043
H	-5.04569	-2.19486	-2.57505
C	-2.91859	-0.55112	-3.21078
H	-2.06458	0.15420	-3.13413
C	-2.29982	-1.95804	-3.26557
H	-1.94754	-2.27100	-2.26174
H	-1.43180	-1.96350	-3.95905
H	-3.03228	-2.71084	-3.62537
C	-3.65103	-0.23559	-4.51948
H	-4.48709	-0.94695	-4.68982
H	-2.94650	-0.30304	-5.37582
H	-4.06208	0.79494	-4.49075
C	-4.55499	1.75552	1.13681
H	-3.77059	2.48756	0.86346
C	-4.15115	1.17723	2.50069
H	-3.21698	0.58463	2.40350
H	-4.94635	0.52165	2.91420
H	-3.96480	2.00077	3.22252
C	-5.85786	2.55900	1.24135
H	-6.69090	1.93306	1.62482
H	-6.13765	2.95869	0.24307
H	-5.71937	3.41800	1.93225
C	3.61714	1.32602	1.04947
C	4.37734	1.11397	-0.06159
C	3.83044	0.47958	2.27524
C	5.38112	0.01891	-0.05085

C	4.78918	-0.46627	2.25576
C	5.58002	-0.73727	1.03888
H	5.95995	-0.17218	-0.94420
H	4.98556	-1.06127	3.13782
C	4.26315	1.97041	-1.32416
H	3.49648	2.76364	-1.21372
C	5.58083	2.70151	-1.60830
H	5.87817	3.30403	-0.72333
H	5.45460	3.39116	-2.47023
H	6.39710	1.98797	-1.84786
C	3.83650	1.12329	-2.52934
H	2.89611	0.57962	-2.30102
H	4.61920	0.38538	-2.80229
H	3.65206	1.77805	-3.40760
C	3.00913	0.69223	3.54618
H	2.13197	1.33504	3.32744
C	3.84375	1.41294	4.61066
H	3.22210	1.61832	5.50822
H	4.21397	2.38245	4.21646
H	4.71663	0.79627	4.91443
C	2.44026	-0.62365	4.10643
H	2.01880	-1.24829	3.29336
H	1.63139	-0.40521	4.83609
H	3.22234	-1.21460	4.62822
C	6.58716	-1.87272	1.04103
H	6.57043	-2.37243	2.03489
C	8.01329	-1.35173	0.82549
H	8.25631	-0.58210	1.58908
H	8.12933	-0.90474	-0.18466
H	8.74125	-2.18463	0.92805
C	6.22498	-2.93865	-0.00098
H	5.18065	-3.28597	0.15211
H	6.90093	-3.81441	0.09898
H	6.32209	-2.53639	-1.03067
C	-6.84112	-2.42699	-0.61680
H	-6.82844	-3.12532	-1.48400
C	-6.61682	-3.27328	0.63972
H	-5.62310	-3.76351	0.58614
H	-7.39023	-4.06766	0.70853
H	-6.66439	-2.65219	1.55924
C	-8.22499	-1.76745	-0.59615
H	-8.36496	-1.14837	-1.50811
H	-8.35008	-1.12213	0.29902
H	-9.01680	-2.54658	-0.58132
H	-0.18006	-2.14783	1.93499
C	-1.95573	-2.91230	2.63168
C	-0.71309	-3.70528	0.70689
C	-2.04012	-1.98028	3.68427
C	-3.02059	-3.81134	2.37435
C	-1.79537	-4.60338	0.42094
C	-3.17362	-1.96533	4.47627
H	-1.23224	-1.27072	3.82810
C	-4.15196	-3.78476	3.21907
C	-2.88711	-4.66727	1.23881
H	-1.71943	-5.25708	-0.43707
C	-4.23006	-2.87319	4.25431
H	-3.25163	-1.23848	5.27881
H	-4.96404	-4.48170	3.03257
H	-3.67858	-5.38086	1.02564
H	-5.10682	-2.84763	4.89301

C	0.39550	-3.46377	-0.16847
C	0.60978	-4.28042	-1.43465
H	-0.37491	-4.54822	-1.83901
H	1.13427	-5.21834	-1.23110
N	1.33340	-3.54576	-2.46306
H	1.00641	-2.59162	-2.55548
C	2.69855	-3.68109	-2.72589
C	3.38248	-2.59706	-3.31093
C	3.41487	-4.87094	-2.50311
C	4.72433	-2.70026	-3.65898
H	2.84458	-1.66927	-3.48864
C	4.76272	-4.95889	-2.84812
H	2.92544	-5.73533	-2.06782
C	5.43155	-3.88313	-3.43141
H	5.22307	-1.84411	-4.10515
H	5.29251	-5.88881	-2.65916
H	6.48135	-3.96089	-3.69551
H	0.00204	-2.09730	-0.56064
N	-0.84417	-2.93980	1.82195
Cl	1.96183	-3.12542	0.67235

Catalyst (TRIP, 3)-Enamine protonation TS (pro-S) alternative conformation
Electronic energy (Hartrees): -1870.524956
Free Energy at 1M concentration and 253 K (Hartrees): -1869.307963
Coordinates:

P	-0.03936	0.03206	0.29950
O	1.12577	0.96675	-0.35250
O	-1.37739	0.96152	0.43859
O	0.25081	-0.47324	1.67480
O	-0.26663	-1.01133	-0.82894
C	1.29868	2.15311	0.30821
C	2.56090	2.38417	1.04965
C	2.76185	3.55264	1.67457
H	3.69302	3.73814	2.19616
C	1.71129	4.59280	1.66516
C	1.90985	5.84266	2.43447
H	2.83420	6.00830	2.97385
C	0.94742	6.77057	2.46949
H	1.09276	7.68276	3.03365
C	-0.32158	6.54815	1.75044
H	-1.10512	7.29311	1.80218
C	-0.50939	5.42521	1.04754
H	-1.45682	5.27265	0.54811
C	0.56351	4.39687	0.97242
C	0.38123	3.13693	0.19013
C	-1.59227	1.86638	-0.56881
C	-2.83811	1.74803	-1.36527
C	-3.13312	2.67809	-2.28454
H	-4.05456	2.61069	-2.85065
C	-2.19536	3.78547	-2.55578
C	-2.49032	4.74445	-3.64541
H	-3.40986	4.65912	-4.21111
C	-1.62099	5.71723	-3.93841
H	-1.83639	6.41618	-4.73627
C	-0.35779	5.83130	-3.18537
H	0.35339	6.60492	-3.44488
C	-0.08449	4.97393	-2.19517
H	0.86051	5.06116	-1.67624
C	-1.05732	3.90641	-1.83292
C	-0.78238	2.94254	-0.72348

C	-3.77462	0.62152	-1.12478
C	-4.66485	0.64737	0.08718
C	-3.86828	-0.41106	-2.00992
C	-5.58115	-0.32714	0.24479
C	-4.93222	-1.43122	-1.82013
C	-5.75084	-1.39829	-0.76019
H	-6.22635	-0.32917	1.11282
H	-5.05423	-2.20813	-2.56360
C	-2.92785	-0.56557	-3.20507
H	-2.07470	0.14119	-3.13189
C	-2.30714	-1.97175	-3.25660
H	-1.95151	-2.28039	-2.25254
H	-1.44101	-1.97843	-3.95247
H	-3.03959	-2.72688	-3.61154
C	-3.66288	-0.25472	-4.51346
H	-4.49805	-0.96786	-4.68069
H	-2.95957	-0.32324	-5.37074
H	-4.07555	0.77526	-4.48668
C	-4.55568	1.74684	1.14247
H	-3.77276	2.47923	0.86582
C	-4.14680	1.17020	2.50555
H	-3.21205	0.57893	2.40584
H	-4.93980	0.51380	2.92199
H	-3.95937	1.99456	3.22620
C	-5.85883	2.54944	1.25062
H	-6.69007	1.92335	1.63778
H	-6.14245	2.94774	0.25285
H	-5.71860	3.40936	1.94004
C	3.61336	1.33997	1.04591
C	4.37542	1.12750	-0.06380
C	3.82918	0.49842	2.27461
C	5.38483	0.03767	-0.04810
C	4.79285	-0.44245	2.25955
C	5.58678	-0.71329	1.04468
H	5.96574	-0.15349	-0.94008
H	4.99091	-1.03368	3.14377
C	4.25814	1.97872	-1.32960
H	3.48715	2.76824	-1.22291
C	5.57224	2.71572	-1.61499
H	5.86540	3.32307	-0.73194
H	5.44332	3.40149	-2.47961
H	6.39253	2.00563	-1.85102
C	3.83728	1.12492	-2.53212
H	2.89958	0.57707	-2.30276
H	4.62418	0.39019	-2.80164
H	3.65024	1.77548	-3.41295
C	3.00433	0.71011	3.54343
H	2.12457	1.34824	3.32148
C	3.83332	1.43712	4.60803
H	3.20880	1.64165	5.50379
H	4.19989	2.40743	4.21244
H	4.70840	0.82524	4.91509
C	2.44060	-0.60723	4.10542
H	2.02369	-1.23564	3.29289
H	1.62928	-0.39104	4.83303
H	3.22444	-1.19333	4.63000
C	6.60058	-1.84276	1.05237
H	6.58533	-2.33900	2.04799
C	8.02390	-1.31410	0.83704
H	8.26127	-0.54043	1.59831

H	8.13872	-0.86990	-0.17447
H	8.75662	-2.14232	0.94347
C	6.24627	-2.91452	0.01368
H	5.20352	-3.26694	0.16595
H	6.92678	-3.78619	0.11824
H	6.34336	-2.51551	-1.01725
C	-6.84685	-2.43680	-0.60229
H	-6.83796	-3.13423	-1.47030
C	-6.61876	-3.28458	0.65251
H	-5.62646	-3.77726	0.59366
H	-7.39378	-4.07714	0.72435
H	-6.66048	-2.66401	1.57272
C	-8.23013	-1.77602	-0.57602
H	-8.37256	-1.15524	-1.48647
H	-8.35186	-1.13219	0.32072
H	-9.02261	-2.55447	-0.55996
H	-0.17368	-2.14847	1.92736
C	-1.94859	-2.91722	2.62183
C	-0.70209	-3.70655	0.69808
C	-2.03617	-1.98594	3.67482
C	-3.01080	-3.81905	2.36338
C	-1.78240	-4.60633	0.41000
C	-3.17000	-1.97473	4.46639
H	-1.23036	-1.27416	3.81936
C	-4.14224	-3.79668	3.20815
C	-2.87478	-4.67326	1.22684
H	-1.70439	-5.25885	-0.44872
C	-4.22345	-2.88589	4.24382
H	-3.25056	-1.24842	5.26917
H	-4.95194	-4.49633	3.02149
H	-3.66477	-5.38802	1.01205
H	-5.10041	-2.86353	4.88236
C	0.40787	-3.46333	-0.17510
C	0.62638	-4.28158	-1.43962
H	-0.35711	-4.54691	-1.84854
H	1.14724	-5.22086	-1.23286
N	1.35694	-3.55009	-2.46530
N	-0.83632	-2.94154	1.81305
C	2.72394	-3.68692	-2.71815
C	3.43725	-4.87726	-2.48840
C	3.41335	-2.60455	-3.29970
C	4.78756	-4.96720	-2.82309
H	2.94357	-5.74042	-2.05547
C	4.75770	-2.70969	-3.63746
H	2.87784	-1.67647	-3.48292
C	5.46191	-3.89298	-3.40284
H	5.31491	-5.89741	-2.62877
H	5.26077	-1.85462	-4.08084
H	6.51364	-3.97218	-3.65872
H	1.03124	-2.59589	-2.56181
H	0.01212	-2.09748	-0.56874
Cl	1.97168	-3.12223	0.66935

Catalyst (TRIP, 3)-Enamine protonation TS (pro-S) alternative conformation
Electronic energy (Hartrees): -1870.524327
Free Energy at 1M concentration and 253 K (Hartrees): -1869.307527
Coordinates:

P	-0.02562	0.04567	0.33449
O	1.14614	0.93522	-0.36730
O	-1.33808	1.01078	0.48359

O	0.28447	-0.43441	1.71445
O	-0.30293	-1.01727	-0.76383
C	1.35760	2.13794	0.25108
C	2.64094	2.36626	0.95606
C	2.87997	3.54970	1.53788
H	3.82644	3.73213	2.03233
C	1.85161	4.61168	1.51602
C	2.09352	5.88188	2.23813
H	3.03250	6.04479	2.75249
C	1.15240	6.83178	2.26127
H	1.32944	7.75890	2.79097
C	-0.13601	6.61395	1.57613
H	-0.90178	7.37774	1.61827
C	-0.36305	5.47253	0.91586
H	-1.32378	5.32475	0.44103
C	0.68525	4.41828	0.85428
C	0.45884	3.13702	0.11973
C	-1.56059	1.88787	-0.54643
C	-2.83015	1.77414	-1.30490
C	-3.12240	2.67324	-2.25524
H	-4.06045	2.61031	-2.79398
C	-2.16326	3.74373	-2.59230
C	-2.45948	4.66661	-3.71229
H	-3.39281	4.58158	-4.25493
C	-1.57438	5.60619	-4.06118
H	-1.79075	6.27870	-4.88119
C	-0.29307	5.71937	-3.33924
H	0.42980	6.46546	-3.64323
C	-0.01832	4.89473	-2.32203
H	0.93907	4.97981	-1.82597
C	-1.00719	3.86519	-1.89870
C	-0.72960	2.93736	-0.75980
C	-3.79800	0.69555	-0.98418
C	-4.66482	0.82223	0.23838
C	-3.94488	-0.38013	-1.80796
C	-5.61679	-0.10437	0.46286
C	-5.04214	-1.34646	-1.54462
C	-5.84272	-1.22124	-0.47587
H	-6.24817	-0.03464	1.33916
H	-5.20704	-2.15652	-2.24171
C	-3.03375	-0.63494	-3.00803
H	-2.15185	0.03930	-2.98541
C	-2.47205	-2.06665	-2.99523
H	-2.11665	-2.33893	-1.98055
H	-1.61625	-2.14446	-3.69944
H	-3.23954	-2.80758	-3.30296
C	-3.77984	-0.36208	-4.31849
H	-4.64771	-1.04608	-4.43286
H	-3.09668	-0.50632	-5.18262
H	-4.14758	0.68496	-4.33970
C	-4.48947	1.96964	1.23187
H	-3.68519	2.65500	0.90123
C	-4.07107	1.44795	2.61374
H	-3.15896	0.82050	2.52481
H	-4.87670	0.84108	3.07822
H	-3.84019	2.30014	3.28789
C	-5.75736	2.82845	1.32540
H	-6.60252	2.25777	1.76439
H	-6.04940	3.18436	0.31415
H	-5.56700	3.71743	1.96405

C	3.67217	1.30123	0.96068
C	4.40471	1.04083	-0.15861
C	3.89753	0.49075	2.20827
C	5.39029	-0.07040	-0.13356
C	4.83999	-0.47147	2.19939
C	5.59993	-0.79476	0.97560
H	5.94641	-0.29998	-1.03226
H	5.04527	-1.04136	3.09588
C	4.27710	1.85807	-1.44561
H	3.52603	2.66735	-1.34476
C	5.60004	2.55708	-1.78127
H	5.92632	3.18265	-0.92289
H	5.46640	3.22064	-2.66233
H	6.39914	1.82239	-2.01468
C	3.81050	0.98004	-2.61325
H	2.86668	0.45992	-2.34731
H	4.57501	0.22069	-2.87876
H	3.61748	1.60928	-3.50815
C	3.10668	0.75758	3.48824
H	2.23569	1.40787	3.26697
C	3.97506	1.49760	4.51165
H	3.37591	1.74169	5.41479
H	4.35231	2.44781	4.07897
H	4.84416	0.87638	4.81694
C	2.52836	-0.53004	4.10187
H	2.07985	-1.17294	3.31796
H	1.73866	-0.27488	4.84055
H	3.31150	-1.11725	4.62626
C	6.58729	-1.94729	0.99309
H	6.58385	-2.41442	2.00280
C	8.01706	-1.45910	0.73020
H	8.28998	-0.66976	1.46294
H	8.11855	-1.04704	-0.29622
H	8.73266	-2.30115	0.84422
C	6.18393	-3.03947	-0.00569
H	5.13727	-3.36272	0.18086
H	6.84658	-3.92363	0.10828
H	6.26544	-2.67234	-1.04976
C	-6.98526	-2.18873	-0.22420
H	-7.51074	-1.89534	0.71161
C	-8.02104	-2.13213	-1.35393
H	-8.37780	-1.08882	-1.49089
H	-8.89616	-2.76725	-1.09902
H	-7.59085	-2.49358	-2.31200
C	-6.47199	-3.61795	-0.01772
H	-5.73467	-3.63374	0.80961
H	-5.98969	-4.01142	-0.93747
H	-7.31358	-4.28948	0.25568
H	-0.19152	-2.09129	2.01182
C	-1.99152	-2.77093	2.73256
C	-0.78795	-3.65743	0.82417
C	-2.03803	-1.80742	3.75887
C	-3.08969	-3.63695	2.50280
C	-1.90104	-4.52642	0.56902
C	-3.16792	-1.72781	4.55205
H	-1.20440	-1.12403	3.88053
C	-4.21747	-3.54356	3.34784
C	-2.99015	-4.53014	1.39273
H	-1.85209	-5.20642	-0.27025
C	-4.25833	-2.60105	4.35722

H	-3.21657	-0.97610	5.33373
H	-5.05540	-4.21493	3.18336
H	-3.80526	-5.22351	1.20338
H	-5.13166	-2.52466	4.99656
C	0.31823	-3.47082	-0.06803
C	0.49373	-4.32356	-1.31632
H	-0.50261	-4.57769	-1.70019
H	0.99868	-5.26859	-1.09707
N	1.21849	-3.63227	-2.37342
H	0.90713	-2.67564	-2.49069
C	2.57722	-3.80095	-2.64966
C	3.27256	-2.74885	-3.27789
C	3.27489	-4.99623	-2.39839
C	4.60731	-2.88808	-3.64045
H	2.74931	-1.81727	-3.47808
C	4.61585	-5.12026	-2.75864
H	2.77618	-5.83681	-1.92841
C	5.29591	-4.07619	-3.38496
H	5.11530	-2.05592	-4.12031
H	5.13125	-6.05337	-2.54698
H	6.34030	-4.18184	-3.66055
H	-0.04404	-2.10670	-0.48513
N	-0.88462	-2.86102	1.92078
Cl	1.90339	-3.15925	0.74708

Catalyst (TRIP, 3)-Enamine protonation TS (pro-S) alternative conformation
Electronic energy (Hartrees): -1870.523731
Free Energy at 1M concentration and 253 K (Hartrees): -1869.30752
Coordinates:

P	-0.19796	0.12162	-0.57760
O	0.66214	1.50848	-0.63876
O	-1.56086	0.42141	0.27179
O	0.52448	-0.86304	0.39346
O	-0.40879	-0.33902	-1.98173
C	0.83764	2.11625	0.57789
C	2.21493	2.27482	1.10481
C	2.40875	2.82439	2.31173
H	3.41425	2.96731	2.69009
C	1.25520	3.27504	3.11791
C	1.47514	3.80514	4.48313
H	2.47859	3.87527	4.88427
C	0.43543	4.19707	5.22746
H	0.59690	4.58250	6.22588
C	-0.93665	4.09484	4.69454
H	-1.77502	4.39358	5.31053
C	-1.14605	3.62383	3.45976
H	-2.16186	3.53599	3.09794
C	0.00203	3.21089	2.60802
C	-0.20598	2.67657	1.22825
C	-2.13644	1.65031	0.09320
C	-3.53175	1.70644	-0.40460
C	-4.17454	2.88172	-0.46371
H	-5.20042	2.92426	-0.81018
C	-3.47898	4.13092	-0.09461
C	-4.16799	5.43119	-0.26253
H	-5.19117	5.46299	-0.61588
C	-3.52865	6.57181	0.01758
H	-4.03226	7.52166	-0.10746
C	-2.12944	6.54013	0.48390
H	-1.60995	7.46813	0.68519

C	-1.49966	5.37061	0.64460
H	-0.46557	5.37388	0.96224
C	-2.20731	4.09032	0.36808
C	-1.53069	2.76996	0.55094
C	-4.21903	0.45569	-0.80857
C	-4.66465	-0.51455	0.25160
C	-4.43944	0.17102	-2.12343
C	-5.28248	-1.65101	-0.12213
C	-5.16243	-1.07775	-2.48059
C	-5.55326	-1.95232	-1.54384
H	-5.60903	-2.36100	0.62542
H	-5.37248	-1.28459	-3.52177
C	-3.97267	1.08171	-3.26033
H	-3.33752	1.90851	-2.88205
C	-3.10222	0.32119	-4.27092
H	-2.29443	-0.22819	-3.74632
H	-2.63223	1.03676	-4.97907
H	-3.70015	-0.40442	-4.86077
C	-5.17367	1.72917	-3.95835
H	-5.81785	0.96314	-4.43987
H	-4.82408	2.43911	-4.73812
H	-5.78230	2.29578	-3.22212
C	-4.45533	-0.23392	1.73962
H	-3.93575	0.73305	1.88663
C	-3.57177	-1.30581	2.39575
H	-2.61829	-1.41617	1.83799
H	-4.08277	-2.29112	2.42199
H	-3.32896	-1.01025	3.43870
C	-5.79644	-0.10303	2.47302
H	-6.35298	-1.06390	2.47366
H	-6.42156	0.67440	1.98370
H	-5.62464	0.20321	3.52711
C	3.37367	1.95587	0.23814
C	3.73628	2.80118	-0.76809
C	4.16449	0.70185	0.48230
C	4.89931	2.44883	-1.62388
C	5.24383	0.45191	-0.28345
C	5.62126	1.34262	-1.40100
H	5.16566	3.10200	-2.44471
H	5.84466	-0.42877	-0.10458
C	3.00974	4.11822	-1.05104
H	2.18474	4.29601	-0.33247
C	3.95775	5.31352	-0.89480
H	4.42236	5.29903	0.11443
H	3.39159	6.26356	-1.00156
H	4.76100	5.29813	-1.66093
C	2.36877	4.10311	-2.44405
H	1.69457	3.22620	-2.54418
H	3.13821	4.05607	-3.24294
H	1.76350	5.02274	-2.59339
C	3.77732	-0.28740	1.57847
H	2.78809	-0.02142	2.00409
C	4.78517	-0.24332	2.73183
H	4.45553	-0.91729	3.55139
H	4.85359	0.78859	3.13733
H	5.79366	-0.56261	2.39264
C	3.63647	-1.71639	1.03166
H	2.98341	-1.71806	0.13509
H	3.18644	-2.37468	1.80200
H	4.61967	-2.14938	0.75335

C	6.79073	0.99219	-2.30279
H	6.92803	1.78476	-3.07271
C	6.52265	-0.31744	-3.05303
H	5.57978	-0.23178	-3.63387
H	6.43489	-1.17393	-2.35118
H	7.34988	-0.52625	-3.76438
C	8.09803	0.91409	-1.50593
H	8.26610	1.86519	-0.95672
H	8.95403	0.75651	-2.19631
H	8.07667	0.07678	-0.77688
C	-6.27208	-3.23047	-1.93651
H	-6.39411	-3.27388	-3.04240
C	-5.45687	-4.46319	-1.52929
H	-4.44782	-4.42039	-1.99186
H	-5.96090	-5.38707	-1.88498
H	-5.34422	-4.52515	-0.42613
C	-7.67955	-3.27671	-1.33049
H	-8.25133	-2.37145	-1.62726
H	-7.63917	-3.32873	-0.22190
H	-8.22496	-4.16926	-1.70477
H	0.15843	-2.06061	-2.18195
C	1.95753	-2.72290	-2.92275
C	0.84855	-3.54104	-0.93367
C	1.93693	-1.83133	-4.01237
C	3.09069	-3.53819	-2.67512
C	1.99550	-4.35400	-0.65673
C	3.03143	-1.77895	-4.85625
H	1.07809	-1.18256	-4.14899
C	4.18603	-3.46561	-3.56354
C	3.06234	-4.36285	-1.50933
H	2.00130	-4.94172	0.25068
C	4.15586	-2.60312	-4.64237
H	3.02568	-1.08694	-5.69260
H	5.05426	-4.09138	-3.37772
H	3.91791	-4.99887	-1.29861
H	5.00267	-2.54615	-5.31851
N	0.88177	-2.79446	-2.06662
C	-0.22512	-3.35208	-0.00165
H	0.15600	-1.95143	0.30203
C	-0.29543	-4.19384	1.26359
H	-0.14491	-5.26317	1.05447
H	-1.30596	-4.09719	1.68513
N	0.73632	-3.74356	2.19083
H	0.82996	-2.73486	2.19849
C	0.91035	-4.32872	3.44571
C	1.70572	-3.66376	4.40138
C	0.35593	-5.57482	3.78805
C	1.94155	-4.23110	5.64734
H	2.13831	-2.69803	4.15067
C	0.60764	-6.13622	5.04127
H	-0.27670	-6.10460	3.08484
C	1.39790	-5.47609	5.98007
H	2.55615	-3.69576	6.36600
H	0.16815	-7.10015	5.28331
H	1.58438	-5.91723	6.95400
Cl	-1.85533	-3.15672	-0.76094

Catalyst (TRIP, 3)-Enamine protonation TS (pro-S) alternative conformation
Electronic energy (Hartrees): -1870.524321
Free Energy at 1M concentration and 253 K (Hartrees): -1869.307518

Coordinates:

P	-0.02803	0.04605	0.32663
O	1.13923	0.94301	-0.37307
O	-1.34446	1.00484	0.47996
O	0.28479	-0.43679	1.70503
O	-0.30117	-1.01507	-0.77461
C	1.34476	2.14543	0.24785
C	2.62645	2.37807	0.95459
C	2.86075	3.56231	1.53671
H	3.80597	3.74793	2.03237
C	1.82845	4.62038	1.51480
C	2.06543	5.89126	2.23740
H	3.00397	6.05782	2.75140
C	1.12043	6.83725	2.26139
H	1.29390	7.76488	2.79140
C	-0.16744	6.61431	1.57692
H	-0.93644	7.37481	1.61996
C	-0.39005	5.47225	0.91624
H	-1.35057	5.32063	0.44219
C	0.66271	4.42246	0.85333
C	0.44160	3.14079	0.11769
C	-1.57317	1.88345	-0.54737
C	-2.84537	1.76752	-1.30099
C	-3.14405	2.66758	-2.24845
H	-4.08396	2.60280	-2.78375
C	-2.18953	3.74183	-2.58682
C	-2.49278	4.66617	-3.70372
H	-3.42776	4.57922	-4.24326
C	-1.61209	5.60955	-4.05356
H	-1.83364	6.28318	-4.87126
C	-0.32863	5.72548	-3.33581
H	0.39063	6.47473	-3.64060
C	-0.04754	4.89952	-2.32140
H	0.91123	4.98676	-1.82831
C	-1.03147	3.86574	-1.89695
C	-0.74694	2.93667	-0.76078
C	-3.80866	0.68548	-0.97809
C	-4.66752	0.80510	0.25095
C	-3.95719	-0.38817	-1.80421
C	-5.61327	-0.12678	0.47998
C	-5.04905	-1.35937	-1.53640
C	-5.84141	-1.24167	-0.46062
H	-6.23873	-0.06224	1.36096
H	-5.21633	-2.16729	-2.23543
C	-3.05247	-0.63673	-3.01053
H	-2.17411	0.04227	-2.99330
C	-2.48265	-2.06527	-3.00246
H	-2.11668	-2.33528	-1.99085
H	-1.63252	-2.13843	-3.71407
H	-3.24877	-2.81045	-3.30333
C	-3.80864	-0.36678	-4.31590
H	-4.67302	-1.05591	-4.42577
H	-3.13017	-0.50559	-5.18462
H	-4.18300	0.67804	-4.33324
C	-4.49140	1.95143	1.24567
H	-3.69264	2.64139	0.91124
C	-4.06186	1.42929	2.62401
H	-3.14783	0.80566	2.52833
H	-4.86200	0.81848	3.09279
H	-3.83007	2.28137	3.29801

C	-5.76286	2.80389	1.34887
H	-6.60253	2.22818	1.79186
H	-6.06290	3.16052	0.34021
H	-5.57288	3.69244	1.98826
C	3.66064	1.31588	0.96297
C	4.39739	1.05661	-0.15380
C	3.88590	0.50848	2.21263
C	5.38875	-0.04938	-0.12349
C	4.83355	-0.44863	2.20861
C	5.59930	-0.76997	0.98796
H	5.94896	-0.27769	-1.01997
H	5.03873	-1.01596	3.10675
C	4.26924	1.87040	-1.44290
H	3.51329	2.67560	-1.34615
C	5.58931	2.57606	-1.77596
H	5.90933	3.20540	-0.91800
H	5.45475	3.23683	-2.65898
H	6.39326	1.84532	-2.00514
C	3.81119	0.98722	-2.61003
H	2.86992	0.46169	-2.34552
H	4.58116	0.23216	-2.87206
H	3.61664	1.61350	-3.50668
C	3.08864	0.77254	3.48922
H	2.21469	1.41738	3.26357
C	3.94873	1.51905	4.51492
H	3.34466	1.76086	5.41540
H	4.32212	2.47085	4.08246
H	4.82025	0.90333	4.82443
C	2.51554	-0.51758	4.10258
H	2.07469	-1.16483	3.31789
H	1.72089	-0.26601	4.83719
H	3.29996	-1.09891	4.63162
C	6.59378	-1.91627	1.01129
H	6.58986	-2.38126	2.02199
C	8.02134	-1.41964	0.75220
H	8.28681	-0.62708	1.48419
H	8.12369	-1.00907	-0.27475
H	8.74184	-2.25692	0.87040
C	6.20074	-3.01308	0.01352
H	5.15558	-3.34264	0.19730
H	6.86868	-3.89272	0.13150
H	6.28338	-2.64751	-1.03097
C	-6.97778	-2.21499	-0.20342
H	-7.49318	-1.93090	0.74090
C	-8.02663	-2.15226	-1.32069
H	-8.38963	-1.10931	-1.44346
H	-8.89602	-2.79377	-1.06204
H	-7.60558	-2.50230	-2.28707
C	-6.45792	-3.64443	-0.01477
H	-5.70628	-3.66417	0.79947
H	-5.99068	-4.03106	-0.94516
H	-7.29344	-4.31943	0.26856
H	-0.18171	-2.09625	1.99460
C	-1.97581	-2.79586	2.71166
C	-0.76174	-3.66491	0.80198
C	-2.03321	-1.83526	3.74018
C	-3.06442	-3.67318	2.47934
C	-1.86608	-4.54392	0.54301
C	-3.16402	-1.77015	4.53336
H	-1.20710	-1.14309	3.86364

C	-4.19290	-3.59492	3.32499
C	-2.95553	-4.56138	1.36614
H	-1.80990	-5.22037	-0.29870
C	-4.24435	-2.65543	4.33667
H	-3.22127	-1.02075	5.31667
H	-5.02314	-4.27547	3.15913
H	-3.76391	-5.26179	1.17373
H	-5.11841	-2.59062	4.97631
C	0.34416	-3.46598	-0.08791
C	0.52959	-4.31680	-1.33617
H	-0.46406	-4.57147	-1.72675
H	1.03400	-5.26168	-1.11488
N	1.26026	-3.62400	-2.38790
N	-0.86756	-2.87223	1.90040
C	2.62148	-3.78848	-2.65446
C	3.32374	-4.97833	-2.39034
C	3.31563	-2.73741	-3.28579
C	4.66748	-5.09816	-2.74163
H	2.82698	-5.81786	-1.91653
C	4.65337	-2.87230	-3.63888
H	2.78897	-1.81003	-3.49629
C	5.34629	-4.05517	-3.37106
H	5.18626	-6.02704	-2.51983
H	5.16014	-2.04079	-4.12115
H	6.39296	-4.15741	-3.63921
H	0.94566	-2.66907	-2.51004
H	-0.03069	-2.10273	-0.50223
Cl	1.92486	-3.14239	0.73127

Catalyst (TRIP, 3)-Enamine protonation TS (pro-S) alternative conformation
Electronic energy (Hartrees): -1870.525474
Free Energy at 1M concentration and 253 K (Hartrees): -1869.307508
Coordinates:

P	0.51773	-0.05893	0.17489
O	1.73450	0.08312	-0.89915
O	0.12611	1.45895	0.63677
O	0.89090	-0.79541	1.41977
O	-0.64835	-0.62354	-0.68496
C	2.80807	0.75450	-0.37256
C	4.06296	0.01151	-0.09453
C	5.12622	0.66263	0.39811
H	6.05440	0.13324	0.57594
C	5.03375	2.09827	0.73306
C	6.18238	2.77599	1.37732
H	7.10148	2.23321	1.56021
C	6.08823	4.05962	1.74021
H	6.92732	4.55340	2.21307
C	4.83433	4.80262	1.51213
H	4.75580	5.83354	1.83245
C	3.79289	4.20153	0.92526
H	2.87462	4.75784	0.79319
C	3.89364	2.78416	0.48208
C	2.74884	2.09194	-0.18489
C	0.30929	2.46778	-0.27571
C	-0.86894	3.28643	-0.65473
C	-0.70165	4.42030	-1.35104
H	-1.55961	5.02701	-1.61574
C	0.63152	4.78939	-1.86371
C	0.77444	5.97176	-2.74441
H	-0.08125	6.60237	-2.95161

C	1.95882	6.26281	-3.29221
H	2.06171	7.12560	-3.93757
C	3.12472	5.39708	-3.03471
H	4.07344	5.62104	-3.50519
C	3.00737	4.32972	-2.23652
H	3.86912	3.69309	-2.08866
C	1.70529	4.01380	-1.58593
C	1.55308	2.84383	-0.66518
C	-2.23411	2.81581	-0.31257
C	-2.70531	2.88274	1.11442
C	-3.05910	2.31235	-1.27646
C	-3.93582	2.42723	1.41788
C	-4.42345	1.86119	-0.89497
C	-4.84325	1.90200	0.37875
H	-4.29579	2.47264	2.43758
H	-5.09256	1.49985	-1.66393
C	-2.65325	2.21030	-2.75004
H	-1.58708	2.47230	-2.89948
C	-2.79346	0.77973	-3.28320
H	-2.26398	0.06845	-2.61832
H	-2.33975	0.70710	-4.29477
H	-3.85744	0.48231	-3.36495
C	-3.47160	3.18761	-3.60169
H	-4.55142	2.92824	-3.58310
H	-3.11978	3.16235	-4.65520
H	-3.34697	4.22308	-3.22011
C	-1.84358	3.49622	2.21725
H	-0.85882	3.80657	1.81562
C	-1.55814	2.48595	3.33758
H	-1.13974	1.55108	2.91220
H	-2.47982	2.23615	3.90379
H	-0.81502	2.90581	4.04847
C	-2.48935	4.76912	2.77895
H	-3.44684	4.54291	3.29396
H	-2.68524	5.48946	1.95594
H	-1.80441	5.25303	3.50770
C	4.15021	-1.43878	-0.39224
C	4.24653	-1.88166	-1.67774
C	4.16644	-2.42767	0.74295
C	4.34105	-3.34311	-1.93284
C	4.30977	-3.73706	0.46500
C	4.37587	-4.22821	-0.92760
H	4.38966	-3.69653	-2.95464
H	4.35892	-4.45967	1.26781
C	4.30104	-0.93895	-2.88155
H	4.23771	0.12388	-2.57375
C	5.63586	-1.07760	-3.62363
H	6.47933	-0.90278	-2.92168
H	5.69926	-0.32233	-4.43605
H	5.74664	-2.08626	-4.07428
C	3.11925	-1.18371	-3.82882
H	2.16061	-1.11284	-3.27298
H	3.18206	-2.18454	-4.30498
H	3.11111	-0.41514	-4.63105
C	4.04903	-1.97989	2.19974
H	3.74312	-0.91476	2.24675
C	5.40232	-2.09392	2.91128
H	5.32148	-1.70403	3.94848
H	6.17005	-1.49719	2.37628
H	5.74103	-3.15123	2.95358

C	2.97048	-2.76460	2.96714
H	2.04408	-2.84989	2.36470
H	2.72539	-2.24090	3.91581
H	3.31576	-3.78919	3.22017
C	4.46321	-5.71604	-1.21508
H	4.49880	-5.88962	-2.31437
C	3.22271	-6.44674	-0.68756
H	2.30342	-5.99689	-1.12052
H	3.16232	-6.38879	0.41996
H	3.25763	-7.51705	-0.98323
C	5.74639	-6.31425	-0.62705
H	6.63284	-5.76732	-1.01382
H	5.83952	-7.38040	-0.92527
H	5.74552	-6.25811	0.48208
C	-6.24442	1.46567	0.76875
H	-6.35322	1.54155	1.87345
C	-7.30096	2.38849	0.14989
H	-7.10547	3.44163	0.44521
H	-8.31200	2.10738	0.51452
H	-7.29278	2.31954	-0.95879
C	-6.50734	0.00189	0.39624
H	-5.68764	-0.64279	0.77367
H	-6.58708	-0.12133	-0.70291
H	-7.45942	-0.34049	0.85467
H	-0.30849	-1.82197	2.13802
C	-1.64575	-1.36110	3.63115
C	-2.16528	-2.43442	1.52361
C	-0.65401	-0.84337	4.48645
C	-3.01821	-1.25788	3.96866
C	-3.55914	-2.33432	1.84852
C	-1.03702	-0.23957	5.67011
H	0.38643	-0.89858	4.18508
C	-3.37313	-0.64082	5.18822
C	-3.96044	-1.77479	3.02791
H	-4.27505	-2.69866	1.12509
C	-2.39747	-0.14023	6.02901
H	-0.27758	0.16986	6.32892
H	-4.42464	-0.56168	5.44923
H	-5.01958	-1.71959	3.26570
H	-2.67521	0.33675	6.96324
C	-1.67792	-2.88909	0.25137
C	-2.62810	-3.59576	-0.70765
H	-3.11408	-4.45465	-0.22154
H	-2.03034	-4.00131	-1.53616
N	-3.66634	-2.68560	-1.17595
N	-1.29043	-1.96175	2.44556
C	-4.65344	-3.11600	-2.06749
C	-5.48299	-2.15840	-2.68282
C	-4.88416	-4.47484	-2.34713
C	-6.50537	-2.54488	-3.53997
H	-5.31975	-1.10642	-2.47192
C	-5.92058	-4.85061	-3.20379
H	-4.25592	-5.24071	-1.90751
C	-6.73813	-3.89743	-3.80750
H	-7.12697	-1.78228	-4.00110
H	-6.07872	-5.90680	-3.40441
H	-7.53820	-4.19860	-4.47612
H	-3.31930	-1.75350	-1.36488
H	-1.11797	-1.57926	-0.32128
Cl	-0.12157	-3.82092	0.35049

Catalyst (TRIP, 3)-Enamine protonation TS (pro-S) alternative conformation
Electronic energy (Hartrees): -1870.524983
Free Energy at 1M concentration and 253 K (Hartrees): -1869.307211
Coordinates:

P	0.00895	-0.12826	-0.36217
O	-1.24905	0.71821	0.23136
O	1.23706	0.92439	-0.59287
O	-0.23824	-0.75439	-1.70008
O	0.36859	-1.06636	0.81849
C	-1.58051	1.79711	-0.54224
C	-2.87930	1.79820	-1.25736
C	-3.23442	2.86222	-1.99108
H	-4.19415	2.88172	-2.49325
C	-2.31651	4.01275	-2.12764
C	-2.68169	5.14128	-3.01449
H	-3.63125	5.13856	-3.53520
C	-1.83930	6.16691	-3.17877
H	-2.10632	6.99033	-3.82855
C	-0.53681	6.17315	-2.48567
H	0.14995	6.99510	-2.64177
C	-0.19920	5.16148	-1.67762
H	0.77106	5.17511	-1.19935
C	-1.13853	4.02824	-1.45877
C	-0.78869	2.88972	-0.55619
C	1.34233	1.96896	0.29017
C	2.62205	2.13330	1.02377
C	2.81008	3.20320	1.80997
H	3.75168	3.33893	2.32864
C	1.72842	4.18877	2.00147
C	1.91235	5.30216	2.96093
H	2.85110	5.41594	3.48877
C	0.91878	6.16908	3.18320
H	1.05311	6.98090	3.88637
C	-0.36955	6.01265	2.48202
H	-1.17840	6.70256	2.68565
C	-0.54357	5.01419	1.60851
H	-1.50611	4.90394	1.12731
C	0.56390	4.06019	1.32427
C	0.39885	2.94215	0.34525
C	3.70856	1.13573	0.85459
C	4.48057	1.09576	-0.43703
C	4.02388	0.26732	1.85718
C	5.48199	0.20530	-0.57175
C	5.17697	-0.65359	1.68008
C	5.86615	-0.69894	0.53270
H	6.04266	0.15592	-1.49528
H	5.46183	-1.30359	2.49696
C	3.23679	0.18728	3.16548
H	2.32015	0.81125	3.12346
C	2.75362	-1.24405	3.44059
H	2.24053	-1.65517	2.54699
H	2.03839	-1.24436	4.28982
H	3.59573	-1.91692	3.70479
C	4.07944	0.70710	4.33536
H	4.97959	0.07548	4.49185
H	3.47733	0.70391	5.26906
H	4.40592	1.74982	4.13790
C	4.17223	2.04926	-1.59115
H	3.32999	2.71954	-1.33171

C	3.74652	1.28357	-2.85295
H	2.90989	0.59185	-2.62040
H	4.58822	0.69274	-3.27181
H	3.39939	1.99727	-3.63027
C	5.36330	2.97121	-1.88306
H	6.23731	2.39767	-2.25752
H	5.65795	3.51352	-0.95908
H	5.08227	3.72301	-2.65137
C	-3.80385	0.64988	-1.09981
C	-4.49069	0.46882	0.06321
C	-3.97869	-0.32615	-2.23199
C	-5.37037	-0.71959	0.20463
C	-4.82552	-1.36219	-2.07479
C	-5.53156	-1.59451	-0.79868
H	-5.89032	-0.88010	1.13916
H	-4.99425	-2.05611	-2.88754
C	-4.42205	1.45280	1.23263
H	-3.73848	2.29882	1.01933
C	-5.79523	2.08283	1.49463
H	-6.16987	2.57198	0.56997
H	-5.71334	2.85694	2.28742
H	-6.53438	1.32193	1.82281
C	-3.88347	0.77118	2.49596
H	-2.91822	0.26599	2.28087
H	-4.60207	0.01910	2.88190
H	-3.70918	1.52683	3.29139
C	-3.24975	-0.14564	-3.56310
H	-2.45812	0.62447	-3.46257
C	-4.21905	0.34322	-4.64532
H	-3.66882	0.53732	-5.59075
H	-4.70416	1.28957	-4.32627
H	-5.00911	-0.41285	-4.84159
C	-2.53370	-1.42818	-4.01972
H	-1.96205	-1.87658	-3.18330
H	-1.82294	-1.19185	-4.84029
H	-3.25390	-2.18438	-4.39682
C	-6.41589	-2.81876	-0.64508
H	-6.39609	-3.40269	-1.59188
C	-7.87628	-2.42309	-0.39449
H	-8.23170	-1.74883	-1.20290
H	-7.99074	-1.90560	0.58154
H	-8.51957	-3.32882	-0.39029
C	-5.89664	-3.74593	0.46196
H	-4.84184	-4.02743	0.25924
H	-6.50251	-4.67646	0.49351
H	-5.95197	-3.25604	1.45715
C	7.02772	-1.66281	0.37192
H	7.19105	-2.22347	1.32000
C	6.72309	-2.70098	-0.71207
H	5.79532	-3.25234	-0.45000
H	7.55346	-3.43546	-0.78267
H	6.58976	-2.21998	-1.70439
C	8.33049	-0.91330	0.06863
H	8.52276	-0.15136	0.85412
H	8.28483	-0.40795	-0.91913
H	9.18384	-1.62459	0.05937
H	0.24197	-2.38082	-1.70638
C	2.03797	-3.22947	-2.24650
C	0.69430	-3.84923	-0.33173
C	2.19043	-2.38495	-3.36443

C	3.06771	-4.13079	-1.87565
C	1.74483	-4.73485	0.07921
C	3.35154	-2.46451	-4.11038
H	1.40906	-1.66860	-3.59466
C	4.22755	-4.20578	-2.67872
C	2.87206	-4.88357	-0.67915
H	1.61852	-5.30983	0.98640
C	4.36978	-3.38365	-3.77923
H	3.48178	-1.80557	-4.96321
H	5.01136	-4.90632	-2.40463
H	3.64321	-5.58462	-0.37074
H	5.26851	-3.43521	-4.38505
C	-0.45685	-3.55133	0.47699
C	-0.75165	-4.46886	1.67622
H	0.11047	-4.43961	2.35286
H	-0.85365	-5.51055	1.34010
N	-1.93893	-4.17635	2.44433
N	0.89281	-3.16964	-1.48858
C	-2.07718	-3.08920	3.30900
C	-1.00104	-2.27377	3.69475
C	-3.34422	-2.83522	3.87093
C	-1.19038	-1.25233	4.62586
H	-0.02420	-2.41348	3.25080
C	-3.51823	-1.81805	4.80138
H	-4.18790	-3.45590	3.57798
C	-2.44155	-1.01667	5.19228
H	-0.34521	-0.62681	4.89897
H	-4.50616	-1.64536	5.21979
H	-2.58093	-0.21753	5.91339
H	-2.79521	-4.43255	1.97381
H	-0.04485	-2.14165	0.78035
Cl	-1.97202	-3.29186	-0.49569

Catalyst (TRIP, 3)-Enamine protonation TS (pro-S) alternative conformation
Electronic energy (Hartrees): -1870.523145
Free Energy at 1M concentration and 253 K (Hartrees): -1869.307195
Coordinates:

P	-0.22503	0.08631	-0.52834
O	0.60221	1.48630	-0.67915
O	-1.55907	0.39339	0.36329
O	0.55542	-0.84479	0.45053
O	-0.48143	-0.43334	-1.90402
C	0.80957	2.14888	0.50340
C	2.20180	2.35846	0.96958
C	2.42866	2.96288	2.14408
H	3.44427	3.14278	2.47693
C	1.29652	3.42397	2.97438
C	1.55581	4.01655	4.30661
H	2.57182	4.12412	4.66566
C	0.53640	4.41899	5.07309
H	0.72670	4.85014	6.04745
C	-0.85213	4.26631	4.59794
H	-1.67315	4.57429	5.23248
C	-1.09730	3.73857	3.39305
H	-2.12380	3.61486	3.07444
C	0.02681	3.31265	2.51610
C	-0.22104	2.71534	1.16914
C	-2.16922	1.60069	0.15485
C	-3.58281	1.60560	-0.29169
C	-4.25350	2.76347	-0.37783

H	-5.29222	2.76923	-0.68654
C	-3.57305	4.04198	-0.08946
C	-4.29684	5.31896	-0.28717
H	-5.33270	5.31386	-0.60300
C	-3.67357	6.48387	-0.08042
H	-4.20268	7.41660	-0.22720
C	-2.25792	6.50190	0.33421
H	-1.75251	7.44850	0.47596
C	-1.59650	5.35398	0.52133
H	-0.55188	5.39276	0.79974
C	-2.28471	4.04831	0.32663
C	-1.57220	2.75168	0.54031
C	-4.25628	0.32383	-0.61284
C	-4.64348	-0.60484	0.50624
C	-4.51560	-0.02642	-1.90445
C	-5.24882	-1.77034	0.20755
C	-5.21934	-1.30631	-2.17743
C	-5.55730	-2.14471	-1.18655
H	-5.53578	-2.45214	0.99757
H	-5.45789	-1.56754	-3.19917
C	-4.11252	0.84066	-3.09822
H	-3.48156	1.69632	-2.78211
C	-3.26561	0.05253	-4.10790
H	-2.42975	-0.46130	-3.59151
H	-2.83464	0.74529	-4.86206
H	-3.87131	-0.70701	-4.64478
C	-5.35367	1.43095	-3.77641
H	-5.99977	0.63044	-4.19536
H	-5.05041	2.11008	-4.60170
H	-5.94488	2.01952	-3.04323
C	-4.38734	-0.25056	1.97106
H	-3.88428	0.73262	2.05409
C	-3.45691	-1.27203	2.64253
H	-2.52245	-1.38946	2.05466
H	-3.94503	-2.26501	2.73392
H	-3.18239	-0.92253	3.66065
C	-5.70358	-0.11271	2.74691
H	-6.23817	-1.08369	2.81288
H	-6.36309	0.62785	2.24567
H	-5.50056	0.24636	3.77855
C	3.33372	2.02819	0.07226
C	3.63942	2.83913	-0.98010
C	4.16020	0.80129	0.33515
C	4.77668	2.47605	-1.86551
C	5.21513	0.54254	-0.46108
C	5.53052	1.39467	-1.62690
H	4.99740	3.10059	-2.72143
H	5.84126	-0.31789	-0.27078
C	2.87369	4.12827	-1.28720
H	2.07288	4.31836	-0.54479
C	3.80025	5.34822	-1.21616
H	4.30322	5.38464	-0.22605
H	3.20951	6.28123	-1.33877
H	4.57383	5.31803	-2.01181
C	2.18061	4.04282	-2.65244
H	1.52247	3.14898	-2.69074
H	2.91992	3.97860	-3.47815
H	1.55007	4.94301	-2.81506
C	3.83587	-0.15075	1.48338
H	2.85753	0.11191	1.93546

C	4.88466	-0.03964	2.59496
H	4.60077	-0.68693	3.45228
H	4.94515	1.00889	2.95672
H	5.88669	-0.35130	2.23043
C	3.70624	-1.60319	0.99905
H	3.02133	-1.65425	0.12803
H	3.29874	-2.23884	1.81090
H	4.68788	-2.02710	0.70185
C	6.67222	1.03155	-2.55877
H	6.76293	1.79481	-3.36437
C	6.40430	-0.31261	-3.24531
H	5.43827	-0.26972	-3.79184
H	6.36210	-1.14152	-2.50706
H	7.20812	-0.53331	-3.97954
C	8.01023	1.01223	-1.81080
H	8.17840	1.98806	-1.30686
H	8.84260	0.84396	-2.52707
H	8.03473	0.20499	-1.04867
C	-6.25444	-3.46352	-1.46786
H	-6.43750	-3.99072	-0.50536
C	-7.62376	-3.24230	-2.12246
H	-8.24183	-2.56729	-1.49250
H	-8.15843	-4.21113	-2.22072
H	-7.51870	-2.79444	-3.13333
C	-5.37578	-4.38667	-2.32133
H	-4.39208	-4.53843	-1.82808
H	-5.20836	-3.96010	-3.33302
H	-5.86356	-5.37844	-2.43337
H	0.11361	-2.14752	-2.06655
C	1.89741	-2.79874	-2.85361
C	0.88697	-3.56227	-0.79173
C	1.81264	-1.95251	-3.97577
C	3.05880	-3.57761	-2.61991
C	2.06309	-4.33767	-0.52955
C	2.87146	-1.90963	-4.86454
H	0.93385	-1.32913	-4.10328
C	4.11636	-3.51611	-3.55376
C	3.09601	-4.35568	-1.42274
H	2.11821	-4.88911	0.39871
C	4.02298	-2.69892	-4.66378
H	2.81606	-1.25225	-5.72662
H	5.00605	-4.11418	-3.37845
H	3.97434	-4.96322	-1.22186
H	4.84092	-2.65032	-5.37519
N	0.85792	-2.86048	-1.95303
C	-0.15385	-3.36151	0.17445
H	0.20803	-1.94334	0.41291
C	-0.15577	-4.15682	1.47156
H	0.01220	-5.22933	1.29547
H	-1.15162	-4.06707	1.92815
N	0.89955	-3.64959	2.34081
H	0.96739	-2.63925	2.31131
C	1.14352	-4.18765	3.60485
C	1.95924	-3.46834	4.50241
C	0.64252	-5.43707	4.01186
C	2.26722	-3.98672	5.75381
H	2.35085	-2.49933	4.20199
C	0.96645	-5.94874	5.26981
H	-0.00435	-6.00861	3.35576
C	1.77717	-5.23526	6.15035

H	2.89626	-3.41004	6.42653
H	0.56738	-6.91630	5.56215
H	2.01985	-5.63824	7.12842
Cl	-1.81636	-3.22939	-0.52657

Catalyst (TRIP, 3)-Enamine protonation TS (pro-S) alternative conformation
Electronic energy (Hartrees): -1870.524885
Free Energy at 1M concentration and 253 K (Hartrees): -1869.307053
Coordinates:

P	0.51238	-0.06039	0.22112
O	1.75500	0.02504	-0.82908
O	0.10723	1.47951	0.58944
O	0.85609	-0.72650	1.51318
O	-0.63151	-0.67368	-0.63537
C	2.81298	0.73184	-0.31730
C	4.06482	0.01373	0.03188
C	5.11358	0.69896	0.50923
H	6.03988	0.18637	0.73879
C	5.00731	2.15111	0.75705
C	6.13810	2.87199	1.38588
H	7.05520	2.34611	1.62083
C	6.03015	4.17414	1.67053
H	6.85615	4.69959	2.13219
C	4.77846	4.89529	1.37130
H	4.68812	5.94275	1.62874
C	3.75322	4.25484	0.79766
H	2.83570	4.79721	0.61290
C	3.87023	2.81448	0.44079
C	2.74398	2.07773	-0.20989
C	0.30604	2.43611	-0.37456
C	-0.86599	3.22917	-0.82160
C	-0.68701	4.32190	-1.57831
H	-1.54050	4.91093	-1.89273
C	0.65512	4.66442	-2.08596
C	0.81322	5.79449	-3.03044
H	-0.03945	6.40937	-3.29065
C	2.00774	6.05721	-3.57052
H	2.12177	6.88166	-4.26246
C	3.17008	5.21160	-3.24019
H	4.12765	5.41126	-3.70365
C	3.03894	4.19168	-2.38419
H	3.89890	3.56758	-2.18225
C	1.72464	3.90950	-1.74282
C	1.55642	2.79397	-0.75954
C	-2.23681	2.77773	-0.47613
C	-2.73169	2.92545	0.93674
C	-3.04627	2.22092	-1.42372
C	-3.96765	2.48829	1.24483
C	-4.41731	1.79214	-1.04006
C	-4.85842	1.90568	0.22203
H	-4.34457	2.59162	2.25413
H	-5.07402	1.38815	-1.79842
C	-2.61640	2.03682	-2.88248
H	-1.54824	2.29202	-3.02929
C	-2.74626	0.57828	-3.33630
H	-2.22523	-0.09296	-2.62476
H	-2.27753	0.45000	-4.33542
H	-3.80837	0.27410	-3.41672
C	-3.42168	2.96299	-3.80114
H	-4.50130	2.70259	-3.78617

H	-3.05217	2.87955	-4.84556
H	-3.30489	4.01851	-3.47619
C	-1.88807	3.60058	2.01739
H	-0.89643	3.88779	1.61552
C	-1.62245	2.65551	3.19789
H	-1.19770	1.69807	2.83336
H	-2.55376	2.43808	3.76170
H	-0.89109	3.11511	3.89628
C	-2.54201	4.90330	2.49500
H	-3.50818	4.70668	3.00593
H	-2.72345	5.57572	1.62924
H	-1.86893	5.42786	3.20646
C	4.16690	-1.45119	-0.17654
C	4.29591	-1.96940	-1.43052
C	4.17168	-2.36986	1.01663
C	4.41947	-3.44130	-1.59367
C	4.34592	-3.69101	0.82232
C	4.45310	-4.26309	-0.53411
H	4.49511	-3.85507	-2.58998
H	4.38859	-4.36465	1.66808
C	4.36297	-1.10145	-2.68849
H	4.27785	-0.02291	-2.44816
C	5.71522	-1.26492	-3.39315
H	6.54093	-1.03459	-2.68612
H	5.78539	-0.55992	-4.24905
H	5.84986	-2.29750	-3.77853
C	3.20524	-1.42257	-3.64265
H	2.23409	-1.33159	-3.11192
H	3.29191	-2.45020	-4.05369
H	3.20366	-0.70574	-4.49147
C	4.00456	-1.83735	2.43966
H	3.68429	-0.77568	2.41335
C	5.33631	-1.88912	3.19771
H	5.21843	-1.43775	4.20605
H	6.11315	-1.31549	2.65073
H	5.68618	-2.93701	3.31544
C	2.91213	-2.58982	3.21928
H	2.00416	-2.71865	2.59654
H	2.63491	-2.01661	4.12974
H	3.25996	-3.59470	3.53898
C	4.58100	-5.76641	-0.70126
H	4.59067	-6.24456	0.30332
C	5.90087	-6.14209	-1.38640
H	6.75698	-5.70910	-0.82585
H	5.93118	-5.77023	-2.43249
H	6.01942	-7.24662	-1.40206
C	3.37915	-6.34509	-1.45864
H	2.43438	-6.05900	-0.94819
H	3.44096	-7.45416	-1.47852
H	3.34784	-5.97518	-2.50545
C	-6.26711	1.49467	0.61252
H	-6.39392	1.63279	1.70926
C	-7.31050	2.38346	-0.07470
H	-7.11700	3.45109	0.16458
H	-8.32832	2.12508	0.28790
H	-7.28385	2.25263	-1.17752
C	-6.52841	0.01283	0.31867
H	-5.71553	-0.61133	0.74254
H	-6.59380	-0.17112	-0.77287
H	-7.48752	-0.30180	0.78233

H	-0.35828	-1.71128	2.26028
C	-1.72433	-1.17212	3.69994
C	-2.20327	-2.35787	1.64344
C	-0.74935	-0.60651	4.54423
C	-3.10292	-1.05373	4.00592
C	-3.60306	-2.24394	1.93722
C	-1.15508	0.05997	5.68593
H	0.29659	-0.67545	4.26557
C	-3.48123	-0.37216	5.18333
C	-4.02684	-1.62312	3.07738
H	-4.30517	-2.64814	1.22152
C	-2.52209	0.17573	6.01357
H	-0.40846	0.50627	6.33539
H	-4.53754	-0.28134	5.42004
H	-5.09027	-1.55822	3.29255
H	-2.81780	0.70223	6.91512
C	-1.69212	-2.87896	0.40646
C	-2.62427	-3.63655	-0.53153
H	-3.11655	-4.47131	-0.01090
H	-2.01170	-4.08171	-1.32832
N	-3.65638	-2.75446	-1.06246
N	-1.34629	-1.83509	2.55532
C	-4.63017	-3.23155	-1.94471
C	-5.45417	-2.30805	-2.61699
C	-4.85380	-4.60324	-2.16002
C	-6.46495	-2.73948	-3.46637
H	-5.29613	-1.24649	-2.45640
C	-5.87862	-5.02401	-3.00973
H	-4.22918	-5.34451	-1.67533
C	-6.69114	-4.10428	-3.66949
H	-7.08272	-2.00247	-3.97213
H	-6.03165	-6.08928	-3.15994
H	-7.48235	-4.44046	-4.33196
H	-3.30905	-1.83166	-1.29183
H	-1.11600	-1.60302	-0.22841
Cl	-0.13805	-3.80243	0.58435

Catalyst (TRIP, 3)-Enamine protonation TS (pro-S) alternative conformation
Electronic energy (Hartrees): -1870.524285
Free Energy at 1M concentration and 253 K (Hartrees): -1869.306942
Coordinates:

P	-0.03719	-0.11902	-0.34354
O	-1.26872	0.71158	0.32398
O	1.14439	0.95566	-0.68845
O	-0.36459	-0.77990	-1.64697
O	0.42976	-1.02068	0.82754
C	-1.67234	1.77680	-0.43413
C	-3.01659	1.74679	-1.05932
C	-3.44234	2.79822	-1.77321
H	-4.43381	2.79445	-2.20987
C	-2.56006	3.96596	-1.97878
C	-3.00867	5.08043	-2.84491
H	-3.99201	5.05530	-3.29796
C	-2.20044	6.12092	-3.07412
H	-2.52851	6.93401	-3.70877
C	-0.85304	6.15749	-2.47415
H	-0.19514	6.99114	-2.68347
C	-0.43981	5.15874	-1.68540
H	0.56138	5.19471	-1.27724
C	-1.33902	4.00932	-1.39374

C	-0.90463	2.88446	-0.51072
C	1.30139	2.01001	0.17443
C	2.63331	2.20569	0.79899
C	2.86228	3.28473	1.56165
H	3.84037	3.44428	1.99969
C	1.77900	4.24944	1.83509
C	2.01640	5.37343	2.77012
H	2.99168	5.50935	3.22086
C	1.02656	6.22269	3.06545
H	1.20017	7.04230	3.75076
C	-0.30999	6.03644	2.46958
H	-1.11345	6.71261	2.73215
C	-0.53307	5.02816	1.61879
H	-1.52804	4.89611	1.21515
C	0.56679	4.09321	1.25396
C	0.34632	2.96476	0.29845
C	3.72377	1.23164	0.53998
C	4.37412	1.19377	-0.81766
C	4.14700	0.37829	1.51524
C	5.36649	0.31063	-1.04244
C	5.28971	-0.52891	1.23536
C	5.85853	-0.58461	0.02280
H	5.84549	0.26535	-2.01197
H	5.66478	-1.16236	2.02725
C	3.48966	0.29699	2.89291
H	2.56330	0.90632	2.93480
C	3.05870	-1.13962	3.22193
H	2.46582	-1.56394	2.38566
H	2.43210	-1.14508	4.13830
H	3.93307	-1.79830	3.40446
C	4.43211	0.83890	3.97320
H	5.35297	0.22219	4.04701
H	3.92221	0.83432	4.96028
H	4.72178	1.88475	3.73834
C	3.95642	2.14579	-1.93826
H	3.13361	2.80728	-1.60436
C	3.42958	1.37870	-3.16035
H	2.62363	0.67780	-2.85796
H	4.23737	0.79786	-3.65330
H	3.00844	2.09063	-3.90189
C	5.10822	3.08019	-2.33058
H	5.95198	2.51591	-2.78078
H	5.47663	3.62434	-1.43456
H	4.75395	3.83009	-3.06993
C	-3.90423	0.57945	-0.83995
C	-4.52279	0.39424	0.36020
C	-4.12609	-0.40507	-1.95594
C	-5.38838	-0.79878	0.55062
C	-4.95531	-1.44639	-1.75151
C	-5.60055	-1.67465	-0.44055
H	-5.85844	-0.95935	1.51218
H	-5.15313	-2.14728	-2.55053
C	-4.39219	1.37870	1.52396
H	-3.72917	2.23000	1.27070
C	-5.75158	1.99834	1.86939
H	-6.18693	2.48211	0.96879
H	-5.62680	2.77520	2.65387
H	-6.46262	1.23267	2.24488
C	-3.77183	0.70077	2.75127
H	-2.81280	0.21164	2.47825

H	-4.45519	-0.06420	3.17450
H	-3.56314	1.45602	3.53878
C	-3.46184	-0.22982	-3.32107
H	-2.67011	0.54474	-3.26233
C	-4.48388	0.24729	-4.35915
H	-3.98053	0.43535	-5.33152
H	-4.95646	1.19464	-4.02484
H	-5.27992	-0.51284	-4.51089
C	-2.76257	-1.51264	-3.80313
H	-2.15878	-1.95997	-2.98883
H	-2.08471	-1.27706	-4.65129
H	-3.49626	-2.27024	-4.15049
C	-6.48095	-2.89076	-0.21532
H	-6.88955	-2.87803	0.82042
C	-5.67057	-4.18399	-0.35825
H	-4.82393	-4.18178	0.36116
H	-5.26814	-4.29463	-1.38755
H	-6.31215	-5.06247	-0.13294
C	-7.68253	-2.88491	-1.16748
H	-8.24738	-1.93402	-1.06145
H	-8.36707	-3.72407	-0.91956
H	-7.36010	-2.99582	-2.22428
C	7.00940	-1.53061	-0.26795
H	7.28970	-1.44242	-1.34110
C	8.25064	-1.15985	0.55232
H	8.53165	-0.10231	0.35929
H	9.10613	-1.80544	0.25983
H	8.06583	-1.28922	1.63996
C	6.60746	-2.99179	-0.02968
H	5.67546	-3.22381	-0.58394
H	6.43851	-3.19306	1.04933
H	7.40825	-3.67032	-0.39339
H	0.17144	-2.38977	-1.65363
C	1.96329	-3.17726	-2.28930
C	0.76612	-3.81170	-0.28418
C	2.01231	-2.34717	-3.42712
C	3.04962	-4.02971	-1.96813
C	1.87260	-4.65123	0.07303
C	3.12897	-2.39214	-4.24066
H	1.18913	-1.66733	-3.61934
C	4.16251	-4.06973	-2.83785
C	2.95616	-4.76997	-0.75153
H	1.82481	-5.21532	0.99460
C	4.20360	-3.26179	-3.95711
H	3.18007	-1.74374	-5.10986
H	4.98997	-4.73307	-2.60212
H	3.77150	-5.43570	-0.48075
H	5.06561	-3.28682	-4.61569
C	-0.33945	-3.53395	0.59279
C	-0.53307	-4.43304	1.82453
H	0.37829	-4.38946	2.43186
H	-0.65571	-5.48045	1.51428
N	-1.65911	-4.12953	2.67722
N	0.86496	-3.14876	-1.46307
C	-1.72889	-3.02758	3.53227
C	-0.62166	-2.21808	3.83280
C	-2.95234	-2.75181	4.17475
C	-0.73795	-1.18000	4.75738
H	0.32229	-2.37799	3.32884
C	-3.05365	-1.71830	5.09803

H	-3.82013	-3.36733	3.94914
C	-1.94606	-0.92150	5.40181
H	0.12968	-0.55978	4.96383
H	-4.00940	-1.52891	5.57923
H	-2.02897	-0.10965	6.11740
H	-2.54840	-4.38297	2.27001
H	0.04646	-2.10988	0.84206
Cl	-1.92250	-3.33774	-0.28359

Catalyst (TRIP, 3)-Enamine protonation TS (pro-S) alternative conformation
Electronic energy (Hartrees): -1870.52445
Free Energy at 1M concentration and 253 K (Hartrees): -1869.306866
Coordinates:

P	-0.00741	-0.10602	-0.40444
O	-1.26900	0.68444	0.25507
O	1.17708	0.99179	-0.65228
O	-0.28005	-0.70709	-1.74890
O	0.42131	-1.06017	0.73940
C	-1.65734	1.77723	-0.47094
C	-2.97945	1.76182	-1.14160
C	-3.39165	2.83825	-1.82559
H	-4.36825	2.84445	-2.29449
C	-2.51422	4.02104	-1.95244
C	-2.94423	5.16712	-2.78610
H	-3.91099	5.15233	-3.27396
C	-2.13955	6.22374	-2.94274
H	-2.45402	7.06005	-3.55365
C	-0.81429	6.24722	-2.29466
H	-0.15857	7.09517	-2.44510
C	-0.41806	5.21967	-1.53468
H	0.56764	5.24749	-1.08979
C	-1.31447	4.05070	-1.32379
C	-0.89902	2.89364	-0.47400
C	1.28772	2.00939	0.26065
C	2.58974	2.18640	0.95025
C	2.77750	3.23361	1.76655
H	3.73446	3.37989	2.25320
C	1.67586	4.17904	2.03373
C	1.86424	5.26336	3.02511
H	2.81871	5.38662	3.52171
C	0.85546	6.09193	3.31500
H	0.99314	6.88235	4.04153
C	-0.45357	5.92182	2.65659
H	-1.27340	6.57989	2.91451
C	-0.63213	4.94971	1.75472
H	-1.60859	4.82778	1.30537
C	0.49036	4.03949	1.39596
C	0.31969	2.95129	0.38492
C	3.69664	1.22893	0.70007
C	4.40811	1.25221	-0.62677
C	4.07933	0.33538	1.65591
C	5.41438	0.38305	-0.84397
C	5.23782	-0.55566	1.38927
C	5.86160	-0.55660	0.20266
H	5.93722	0.38159	-1.79167
H	5.57914	-1.22239	2.16902
C	3.36099	0.19335	2.99785
H	2.42961	0.79594	3.02272
C	2.92525	-1.25777	3.24785
H	2.37793	-1.65210	2.36715

H	2.25384	-1.30340	4.13084
H	3.79465	-1.91812	3.44747
C	4.25013	0.69459	4.14112
H	5.17147	0.08058	4.23011
H	3.69679	0.64540	5.10328
H	4.54202	1.75106	3.96354
C	4.03622	2.25039	-1.72307
H	3.19519	2.89355	-1.39874
C	3.56971	1.53511	-2.99985
H	2.75457	0.81909	-2.76499
H	4.40221	0.97890	-3.47994
H	3.17893	2.27703	-3.72851
C	5.19925	3.20529	-2.02172
H	6.06583	2.66424	-2.45683
H	5.52339	3.71148	-1.08717
H	4.87463	3.98521	-2.74343
C	-3.86247	0.57998	-0.99355
C	-4.50481	0.33909	0.18393
C	-4.04289	-0.36449	-2.15131
C	-5.34124	-0.88151	0.31351
C	-4.85085	-1.43224	-2.00201
C	-5.50697	-1.72867	-0.71265
H	-5.82491	-1.08912	1.25818
H	-5.02381	-2.10486	-2.83161
C	-4.42873	1.28638	1.38279
H	-3.77922	2.16049	1.17630
C	-5.81170	1.86275	1.70897
H	-6.23148	2.36927	0.81351
H	-5.72832	2.61294	2.52429
H	-6.51553	1.06801	2.03462
C	-3.82807	0.58201	2.60502
H	-2.85470	0.11599	2.34305
H	-4.50964	-0.20534	2.98776
H	-3.65220	1.31666	3.41955
C	-3.36341	-0.11768	-3.49780
H	-2.59197	0.67253	-3.39586
C	-4.38156	0.37707	-4.53135
H	-3.86862	0.61857	-5.48677
H	-4.88317	1.29740	-4.16518
H	-5.15539	-0.39562	-4.72767
C	-2.62521	-1.36193	-4.02055
H	-2.01638	-1.82260	-3.21760
H	-1.94645	-1.07630	-4.85244
H	-3.33472	-2.12531	-4.40347
C	-6.34548	-2.98625	-0.57170
H	-6.33850	-3.53808	-1.53774
C	-7.80876	-2.64834	-0.26069
H	-8.21281	-1.96032	-1.03389
H	-7.90726	-2.16729	0.73550
H	-8.42172	-3.57486	-0.26633
C	-5.75942	-3.93098	0.48603
H	-4.70366	-4.17068	0.23927
H	-6.33372	-4.88163	0.50645
H	-5.79637	-3.47560	1.49833
C	7.02843	-1.48639	-0.07654
H	7.35835	-1.34882	-1.13022
C	8.22862	-1.15158	0.81704
H	8.51401	-0.08569	0.68634
H	9.09941	-1.78130	0.53530
H	7.99399	-1.33153	1.88775

C	6.62104	-2.95751	0.07567
H	5.71805	-3.16576	-0.53343
H	6.40053	-3.20763	1.13499
H	7.44118	-3.61757	-0.27881
H	0.26808	-2.31338	-1.80658
C	2.09122	-3.06214	-2.40040
C	0.81250	-3.79526	-0.48135
C	2.18524	-2.17855	-3.49430
C	3.16734	-3.92523	-2.07314
C	1.90680	-4.64751	-0.11697
C	3.33657	-2.18072	-4.25932
H	1.36753	-1.49319	-3.69016
C	4.31732	-3.91974	-2.89407
C	3.02538	-4.72348	-0.89867
H	1.82234	-5.25474	0.77403
C	4.40271	-3.05912	-3.97077
H	3.42184	-1.49138	-5.09363
H	5.13732	-4.59071	-2.65393
H	3.83166	-5.39902	-0.62507
H	5.29269	-3.04968	-4.59146
C	-0.33024	-3.56197	0.36043
C	-0.56761	-4.51944	1.53967
H	0.31533	-4.49436	2.18882
H	-0.66280	-5.55239	1.17588
N	-1.73366	-4.26933	2.35436
N	0.95796	-3.07714	-1.62243
C	-1.85660	-3.21293	3.25868
C	-0.77784	-2.39750	3.63723
C	-3.10758	-2.99290	3.86890
C	-0.94831	-1.40947	4.60717
H	0.18602	-2.51140	3.15890
C	-3.26264	-2.00910	4.83788
H	-3.95353	-3.61341	3.58225
C	-2.18314	-1.20757	5.22045
H	-0.10175	-0.78326	4.87425
H	-4.23837	-1.86233	5.29318
H	-2.30796	-0.43453	5.97204
H	-2.60129	-4.52027	1.90199
H	0.03984	-2.14916	0.68889
Cl	-1.87654	-3.33315	-0.57118

Catalyst (TRIP, 3)-Enamine protonation TS (pro-S) alternative conformation
Electronic energy (Hartrees): -1870.522728
Free Energy at 1M concentration and 253 K (Hartrees): -1869.30677
Coordinates:

P	-0.18251	0.11932	-0.54971
O	0.78576	1.43404	-0.55589
O	-1.53072	0.50232	0.28883
O	0.44327	-0.94949	0.39865
O	-0.40609	-0.27788	-1.97127
C	0.98085	1.99816	0.67855
C	2.35506	2.03765	1.23557
C	2.56604	2.55133	2.45542
H	3.57120	2.60643	2.85689
C	1.43493	3.07714	3.24756
C	1.66757	3.56476	4.62647
H	2.66533	3.55085	5.04724
C	0.64622	4.02182	5.35896
H	0.81680	4.37613	6.36735
C	-0.71890	4.03384	4.79922

H	-1.54416	4.38459	5.40542
C	-0.93908	3.60194	3.55207
H	-1.95138	3.59788	3.17034
C	0.19093	3.11791	2.71360
C	-0.02958	2.62636	1.31990
C	-2.00882	1.77439	0.12646
C	-3.38496	1.94650	-0.39763
C	-3.93653	3.16798	-0.44059
H	-4.94867	3.29567	-0.80595
C	-3.15677	4.35278	-0.02966
C	-3.74272	5.70470	-0.18027
H	-4.75358	5.82124	-0.55089
C	-3.02501	6.78752	0.13689
H	-3.45330	7.77517	0.02451
C	-1.64136	6.64028	0.62689
H	-1.05753	7.52189	0.85814
C	-1.10448	5.42336	0.77213
H	-0.07932	5.34171	1.10769
C	-1.90097	4.20636	0.45452
C	-1.32947	2.83498	0.62000
C	-4.15408	0.76102	-0.84893
C	-4.69224	-0.20120	0.17510
C	-4.36880	0.52901	-2.17516
C	-5.38679	-1.27677	-0.24171
C	-5.17721	-0.65083	-2.58035
C	-5.65185	-1.51784	-1.67590
H	-5.78062	-1.97994	0.47942
H	-5.38199	-0.81270	-3.63051
C	-3.81164	1.43129	-3.27761
H	-3.12159	2.19535	-2.86509
C	-2.98372	0.63334	-4.29517
H	-2.23228	0.00672	-3.77347
H	-2.44493	1.32940	-4.97314
H	-3.62503	-0.02539	-4.91707
C	-4.94565	2.18890	-3.97688
H	-5.63784	1.48856	-4.49072
H	-4.52757	2.89039	-4.73015
H	-5.52265	2.78095	-3.23527
C	-4.49109	0.02146	1.67397
H	-3.90204	0.94108	1.85784
C	-3.70516	-1.13319	2.31389
H	-2.75402	-1.30410	1.76737
H	-4.29211	-2.07557	2.30598
H	-3.45767	-0.88461	3.36794
C	-5.83228	0.23510	2.38753
H	-6.46092	-0.67947	2.35022
H	-6.38614	1.07195	1.91052
H	-5.65810	0.49748	3.45301
C	3.50348	1.63037	0.39166
C	3.95035	2.44396	-0.60683
C	4.19860	0.32477	0.65882
C	5.10966	2.00893	-1.42805
C	5.28498	0.00247	-0.06982
C	5.75372	0.86006	-1.17569
H	5.44318	2.63813	-2.24193
H	5.82201	-0.91556	0.12850
C	3.32976	3.80956	-0.91004
H	2.49782	4.04681	-0.21730
C	4.35667	4.93303	-0.72225
H	4.78615	4.88630	0.30146

H	3.86491	5.92158	-0.84651
H	5.18118	4.85794	-1.46190
C	2.73396	3.84025	-2.32287
H	2.00404	3.01254	-2.44755
H	3.52324	3.74053	-3.09722
H	2.19933	4.79998	-2.48849
C	3.70377	-0.63901	1.73425
H	2.72391	-0.30230	2.13057
C	4.67423	-0.67882	2.91952
H	4.26664	-1.32864	3.72337
H	4.80981	0.34261	3.33438
H	5.66516	-1.07462	2.61082
C	3.47298	-2.04951	1.17039
H	2.84090	-1.99683	0.26043
H	2.95995	-2.68007	1.92427
H	4.42862	-2.54939	0.90885
C	6.93413	0.41185	-2.01810
H	7.30860	-0.56163	-1.63080
C	8.09760	1.40665	-1.92449
H	8.37959	1.56291	-0.86123
H	7.82630	2.38621	-2.37207
H	8.98300	1.00671	-2.46326
C	6.51845	0.18079	-3.47614
H	5.66776	-0.53156	-3.51851
H	7.36500	-0.25442	-4.04880
H	6.21425	1.13145	-3.96317
C	-6.45844	-2.72581	-2.11729
H	-6.56189	-2.72966	-3.22593
C	-5.74793	-4.02823	-1.73140
H	-4.72994	-4.05087	-2.17542
H	-6.31432	-4.90059	-2.12167
H	-5.66145	-4.12843	-0.62869
C	-7.87687	-2.68006	-1.53731
H	-8.37191	-1.72576	-1.81796
H	-7.86216	-2.76512	-0.43011
H	-8.48151	-3.51749	-1.94633
H	0.05459	-2.01519	-2.22813
C	1.82494	-2.76473	-2.95488
C	0.61584	-3.58583	-1.02654
C	1.88998	-1.82984	-4.00587
C	2.89597	-3.66229	-2.71594
C	1.70128	-4.48093	-0.75580
C	3.00697	-1.81509	-4.82130
H	1.07854	-1.12115	-4.13433
C	4.01545	-3.62688	-3.57624
C	2.78584	-4.52717	-1.58456
H	1.64719	-5.10053	0.12855
C	4.06913	-2.72051	-4.61761
H	3.06809	-1.09016	-5.62702
H	4.83566	-4.31649	-3.39827
H	3.59355	-5.22477	-1.37991
H	4.93433	-2.69249	-5.27198
N	0.72526	-2.79989	-2.12738
C	-0.46615	-3.36310	-0.11150
H	0.00177	-2.00560	0.25595
C	-0.62252	-4.24906	1.11567
H	-0.52541	-5.31578	0.86618
H	-1.63858	-4.11192	1.51172
N	0.40261	-3.89536	2.09150
H	0.55318	-2.89466	2.14038

C	0.49534	-4.53237	3.32961
C	1.26954	-3.93547	4.34566
C	-0.12140	-5.76743	3.59820
C	1.42701	-4.55808	5.57755
H	1.74733	-2.97758	4.15383
C	0.05111	-6.38458	4.83843
H	-0.73991	-6.24637	2.84753
C	0.82219	-5.79223	5.83658
H	2.02717	-4.07425	6.34336
H	-0.43530	-7.33871	5.02282
H	0.94660	-6.27671	6.79977
Cl	-2.06085	-3.02938	-0.89740

Catalyst (TRIP, 3)-Enamine protonation TS (pro-S) alternative conformation
Electronic energy (Hartrees): -1870.522173
Free Energy at 1M concentration and 253 K (Hartrees): -1869.306296
Coordinates:

P	-0.20809	0.08904	-0.50114
O	0.73357	1.42045	-0.58560
O	-1.52917	0.47306	0.37923
O	0.47449	-0.93959	0.45218
O	-0.47977	-0.35391	-1.90057
C	0.96290	2.02933	0.62139
C	2.35600	2.11493	1.12347
C	2.60200	2.67292	2.31701
H	3.62026	2.76158	2.67739
C	1.49080	3.20291	3.13418
C	1.76494	3.74111	4.48640
H	2.77781	3.76036	4.86911
C	0.76265	4.20315	5.24175
H	0.96362	4.59446	6.23068
C	-0.62241	4.17045	4.73425
H	-1.43134	4.52583	5.35948
C	-0.88030	3.69263	3.51131
H	-1.90586	3.65664	3.16865
C	0.22711	3.20200	2.64693
C	-0.03541	2.65948	1.27968
C	-2.04003	1.72895	0.19277
C	-3.43770	1.85594	-0.28497
C	-4.01523	3.06435	-0.34992
H	-5.04261	3.15958	-0.68103
C	-3.24536	4.27763	-0.00920
C	-3.86399	5.61194	-0.18410
H	-4.88979	5.69601	-0.52094
C	-3.15765	6.71878	0.06926
H	-3.60990	7.69348	-0.06087
C	-1.75446	6.61539	0.51270
H	-1.18088	7.51561	0.69212
C	-1.18779	5.41484	0.67948
H	-0.14973	5.36469	0.97971
C	-1.97006	4.17241	0.43305
C	-1.36479	2.81891	0.62364
C	-4.19870	0.64033	-0.66366
C	-4.68203	-0.29174	0.41455
C	-4.45451	0.35368	-1.97167
C	-5.36929	-1.39563	0.06357
C	-5.24966	-0.85710	-2.30279
C	-5.67462	-1.69830	-1.34849
H	-5.72618	-2.07854	0.82356
H	-5.48492	-1.06479	-3.33748

C	-3.95766	1.22467	-3.12666
H	-3.26718	2.01550	-2.76881
C	-3.15488	0.40501	-4.14736
H	-2.37595	-0.19394	-3.63377
H	-2.65158	1.08576	-4.86687
H	-3.80810	-0.28275	-4.72384
C	-5.13274	1.93519	-3.80729
H	-5.83151	1.20356	-4.26579
H	-4.75842	2.61452	-4.60271
H	-5.69089	2.54496	-3.06564
C	-4.43204	-0.00777	1.89561
H	-3.85531	0.92908	2.02294
C	-3.60027	-1.12177	2.54928
H	-2.66648	-1.29590	1.97444
H	-4.16822	-2.07445	2.59903
H	-3.31955	-0.82795	3.58318
C	-5.75058	0.20823	2.64943
H	-6.36146	-0.71877	2.67031
H	-6.33807	1.01540	2.16161
H	-5.54334	0.51504	3.69698
C	3.48009	1.70488	0.24875
C	3.87188	2.49641	-0.78979
C	4.21185	0.42200	0.52760
C	5.00876	2.06001	-1.64109
C	5.27695	0.09957	-0.23175
C	5.68608	0.93257	-1.37931
H	5.29830	2.67077	-2.48529
H	5.84011	-0.80124	-0.02700
C	3.21106	3.83908	-1.11000
H	2.40091	4.08068	-0.39334
C	4.21986	4.98856	-0.99578
H	4.68874	4.98222	0.01156
H	3.70250	5.96255	-1.13065
H	5.01716	4.90756	-1.76408
C	2.56179	3.81374	-2.49916
H	1.84535	2.96810	-2.57036
H	3.32319	3.70564	-3.29986
H	2.00109	4.75695	-2.67326
C	3.77736	-0.51869	1.64852
H	2.80611	-0.18954	2.07150
C	4.79171	-0.50387	2.79704
H	4.42788	-1.13733	3.63425
H	4.92116	0.53201	3.17656
H	5.77858	-0.88901	2.46289
C	3.55516	-1.94970	1.13505
H	2.88871	-1.93683	0.24844
H	3.08375	-2.56741	1.92582
H	4.51064	-2.43824	0.85232
C	6.84377	0.48296	-2.25196
H	7.25300	-0.47057	-1.85044
C	7.98843	1.50345	-2.23165
H	8.30621	1.69753	-1.18484
H	7.67991	2.46331	-2.69742
H	8.86160	1.10533	-2.79133
C	6.37942	0.19946	-3.68586
H	5.54315	-0.53091	-3.67509
H	7.21329	-0.23570	-4.27688
H	6.03717	1.12857	-4.18895
C	-6.46690	-2.94758	-1.68910
H	-6.71241	-3.49009	-0.74928

C	-7.79955	-2.59677	-2.36249
H	-8.37650	-1.89595	-1.72179
H	-8.40651	-3.51628	-2.50480
H	-7.63696	-2.12555	-3.35495
C	-5.64480	-3.90894	-2.55663
H	-4.68762	-4.15498	-2.04946
H	-5.42133	-3.46368	-3.54926
H	-6.20671	-4.85441	-2.71296
H	0.00277	-2.08871	-2.12993
C	1.76120	-2.81887	-2.90397
C	0.64680	-3.61042	-0.90748
C	1.76420	-1.91332	-3.98229
C	2.86159	-3.68466	-2.68169
C	1.76213	-4.47360	-0.65481
C	2.84886	-1.89657	-4.84022
H	0.93214	-1.22686	-4.09892
C	3.94658	-3.64845	-3.58505
C	2.81499	-4.51926	-1.52353
H	1.75671	-5.06881	0.24777
C	3.93929	-2.77128	-4.65243
H	2.86218	-1.19374	-5.66744
H	4.78906	-4.31392	-3.41964
H	3.64600	-5.19261	-1.33128
H	4.77826	-2.74228	-5.34006
N	0.69507	-2.85500	-2.03367
C	-0.40291	-3.38442	0.04379
H	0.05008	-2.00828	0.35640
C	-0.49367	-4.24146	1.29765
H	-0.38700	-5.31232	1.07048
H	-1.49546	-4.11226	1.73093
N	0.56270	-3.84634	2.22303
H	0.69814	-2.84244	2.24070
C	0.71085	-4.44945	3.47303
C	1.51900	-3.81986	4.44175
C	0.11611	-5.68170	3.79781
C	1.72946	-4.40839	5.68250
H	1.98074	-2.86384	4.20643
C	0.34192	-6.26450	5.04609
H	-0.52697	-6.18459	3.08446
C	1.14595	-5.63979	5.99739
H	2.35473	-3.89984	6.41131
H	-0.12855	-7.21714	5.27426
H	1.31210	-6.09743	6.96739
Cl	-2.03429	-3.10374	-0.68668

Catalyst (TRIP, 3)-Enamine protonation TS (pro-S) alternative conformation
Electronic energy (Hartrees): -1870.522003
Free Energy at 1M concentration and 253 K (Hartrees): -1869.302068
Coordinates:

P	0.11332	-0.01623	-0.10279
O	1.37821	0.83718	-0.70736
O	-0.95476	1.07949	0.47773
O	0.52577	-0.94598	0.99446
O	-0.51667	-0.69032	-1.34409
C	1.78087	1.87653	0.09839
C	3.11574	1.82183	0.75268
C	3.55761	2.87498	1.45580
H	4.53631	2.84633	1.92082
C	2.70003	4.05649	1.65932
C	3.16888	5.16054	2.52852

H	4.15945	5.12639	2.96490
C	2.36959	6.20175	2.78253
H	2.71189	7.00696	3.41968
C	1.01131	6.24714	2.20972
H	0.35850	7.07857	2.44242
C	0.58099	5.25731	1.41894
H	-0.42977	5.29925	1.03664
C	1.47276	4.10898	1.09494
C	1.02482	2.99613	0.20221
C	-1.19434	2.13078	-0.37540
C	-2.54241	2.29218	-0.98403
C	-2.82452	3.39776	-1.69131
H	-3.81708	3.54513	-2.09859
C	-1.77337	4.39233	-1.97876
C	-2.07103	5.53939	-2.86738
H	-3.06954	5.67570	-3.26386
C	-1.10701	6.40907	-3.18784
H	-1.32466	7.24586	-3.83891
C	0.26060	6.22182	-2.66727
H	1.04188	6.91572	-2.94954
C	0.53808	5.19152	-1.85990
H	1.55311	5.06009	-1.50946
C	-0.53174	4.23365	-1.46749
C	-0.25596	3.08917	-0.54782
C	-3.58725	1.25728	-0.78631
C	-4.22924	1.11534	0.56648
C	-4.00653	0.47192	-1.82262
C	-5.17786	0.17623	0.74374
C	-5.09957	-0.50739	-1.58710
C	-5.65425	-0.66159	-0.37742
H	-5.64915	0.05835	1.70999
H	-5.46582	-1.09684	-2.41707
C	-3.44579	0.59191	-3.24358
H	-2.55648	1.25367	-3.27058
C	-2.96824	-0.75875	-3.79664
H	-2.24612	-1.22790	-3.10357
H	-2.45465	-0.60743	-4.77014
H	-3.81300	-1.45919	-3.96056
C	-4.49305	1.20589	-4.18086
H	-5.37496	0.53865	-4.28524
H	-4.05346	1.37163	-5.18764
H	-4.83591	2.18583	-3.78891
C	-3.88878	2.05976	1.71987
H	-3.11409	2.78978	1.41420
C	-3.31507	1.29890	2.92067
H	-2.43816	0.69894	2.60328
H	-4.07172	0.62219	3.37026
H	-2.97851	2.01548	3.69991
C	-5.10832	2.89534	2.13126
H	-5.90878	2.25834	2.56319
H	-5.51420	3.43378	1.24810
H	-4.81309	3.64996	2.89142
C	3.96238	0.60750	0.67775
C	4.72640	0.35311	-0.41933
C	4.11909	-0.23986	1.90995
C	5.75777	-0.71610	-0.34616
C	5.09976	-1.15989	1.95087
C	5.95945	-1.42017	0.77742
H	6.38222	-0.90255	-1.21000
H	5.25515	-1.74622	2.84608

C	4.61364	1.15792	-1.71319
H	3.82815	1.93620	-1.64292
C	5.91954	1.90218	-2.01602
H	6.20130	2.53972	-1.15068
H	5.78485	2.55969	-2.90141
H	6.74959	1.19632	-2.22849
C	4.20997	0.25146	-2.88287
H	3.26842	-0.28658	-2.63993
H	5.00046	-0.49477	-3.10845
H	4.03419	0.86219	-3.79402
C	3.18912	-0.07717	3.11058
H	2.34701	0.59643	2.84742
C	3.92727	0.56127	4.29324
H	3.22234	0.73007	5.13513
H	4.35232	1.54273	3.99739
H	4.75328	-0.09187	4.64697
C	2.55153	-1.41307	3.52894
H	2.17020	-1.96014	2.64228
H	1.70057	-1.22632	4.21849
H	3.28310	-2.06490	4.05149
C	7.05493	-2.46922	0.84534
H	7.55307	-2.56208	-0.14606
C	6.48455	-3.85107	1.19127
H	5.67215	-4.12209	0.48572
H	6.07991	-3.87390	2.22497
H	7.28230	-4.62000	1.11147
C	8.13355	-2.05726	1.85306
H	8.54676	-1.06211	1.58190
H	8.96585	-2.79305	1.84223
H	7.72011	-2.00519	2.88284
C	-6.79499	-1.64322	-0.17548
H	-7.03011	-2.15652	-1.13521
C	-6.40827	-2.73292	0.82773
H	-5.50839	-3.27061	0.46440
H	-7.23270	-3.47054	0.92836
H	-6.19541	-2.30152	1.82875
C	-8.07165	-0.91785	0.26587
H	-8.32868	-0.12143	-0.46500
H	-7.94762	-0.45846	1.26930
H	-8.91953	-1.63434	0.30992
H	1.09586	-2.71755	-0.30063
C	2.13774	-3.55776	-1.87090
C	-0.27123	-3.60726	-1.53381
C	3.40451	-3.17701	-1.39531
C	2.00621	-4.20454	-3.12586
C	-0.40788	-4.25000	-2.80375
C	4.52202	-3.44654	-2.16760
H	3.48762	-2.67371	-0.43715
C	3.16646	-4.46628	-3.88432
C	0.68807	-4.54345	-3.55972
H	-1.40655	-4.49770	-3.13500
C	4.40998	-4.09233	-3.41318
H	5.50462	-3.15929	-1.81180
H	3.06183	-4.96235	-4.84484
H	0.56803	-5.04578	-4.51576
H	5.30100	-4.29257	-3.99867
C	-1.34365	-3.10787	-0.72288
C	-1.27160	-3.38573	0.79015
H	-0.21430	-3.47893	1.06960
H	-1.72482	-4.36488	0.96999

N	-1.91539	-2.40355	1.63723
H	-1.27782	-1.64240	1.84809
C	-2.73959	-2.77602	2.70115
C	-2.70636	-2.03408	3.89750
C	-3.64521	-3.85000	2.61491
C	-3.56851	-2.33171	4.94833
H	-1.98750	-1.22543	3.99890
C	-4.49112	-4.15033	3.68042
H	-3.71381	-4.42838	1.69980
C	-4.46978	-3.39397	4.85305
H	-3.52314	-1.73458	5.85532
H	-5.18736	-4.97905	3.58134
H	-5.13890	-3.62820	5.67505
H	-1.02418	-1.73719	-1.03490
N	0.99976	-3.30823	-1.13159
Cl	-2.99768	-3.46482	-1.35410

Catalyst (TRIP, 3)-Enamine protonation TS (pro-S) alternative conformation
Electronic energy (Hartrees): -1870.521565
Free Energy at 1M concentration and 253 K (Hartrees): -1869.301774
Coordinates:

P	0.14475	-0.04861	-0.10650
O	1.40290	0.77884	-0.76007
O	-0.90942	1.06944	0.45927
O	0.57710	-0.94389	1.01251
O	-0.50779	-0.76231	-1.31495
C	1.81224	1.85754	-0.01098
C	3.15652	1.84112	0.62674
C	3.60774	2.93306	1.26150
H	4.59228	2.93037	1.71517
C	2.75176	4.12297	1.41219
C	3.23274	5.27443	2.21037
H	4.23138	5.26694	2.62929
C	2.43456	6.32590	2.42178
H	2.78570	7.16585	3.00715
C	1.06554	6.33570	1.87358
H	0.41371	7.17642	2.07334
C	0.62430	5.30269	1.14665
H	-0.39420	5.32047	0.78366
C	1.51446	4.14085	0.86861
C	1.05486	2.97936	0.04601
C	-1.17701	2.08683	-0.42714
C	-2.55069	2.24052	-0.97810
C	-2.85023	3.31067	-1.73187
H	-3.85991	3.45460	-2.09675
C	-1.80009	4.27131	-2.11990
C	-2.12071	5.37244	-3.05723
H	-3.13223	5.49852	-3.42290
C	-1.16155	6.21317	-3.45939
H	-1.39597	7.01645	-4.14588
C	0.22281	6.03965	-2.97958
H	0.99888	6.70979	-3.32622
C	0.52110	5.05089	-2.12880
H	1.54678	4.92807	-1.80712
C	-0.54160	4.12630	-1.64792
C	-0.24116	3.02940	-0.67974
C	-3.60675	1.25434	-0.64364
C	-4.13532	1.20257	0.76341
C	-4.12767	0.42588	-1.59548
C	-5.04708	0.26429	1.08436

C	-5.20341	-0.52417	-1.20979
C	-5.62370	-0.63054	0.05994
H	-5.44096	0.21158	2.09125
H	-5.66698	-1.13014	-1.97546
C	-3.69674	0.46361	-3.06519
H	-2.82492	1.13108	-3.21560
C	-3.24507	-0.91283	-3.56987
H	-2.42042	-1.29754	-2.94094
H	-2.86507	-0.82860	-4.61049
H	-4.07712	-1.64688	-3.56528
C	-4.83147	1.00764	-3.94189
H	-5.70594	0.32294	-3.93539
H	-4.48163	1.12350	-4.98998
H	-5.15760	2.00340	-3.57500
C	-3.74691	2.25792	1.80075
H	-3.01019	2.96970	1.37932
C	-3.08406	1.63244	3.03227
H	-2.21975	1.00977	2.72355
H	-3.80272	1.00585	3.60023
H	-2.70837	2.43055	3.70751
C	-4.95980	3.10677	2.20438
H	-5.71963	2.49786	2.73832
H	-5.42730	3.55361	1.30080
H	-4.63974	3.93388	2.87364
C	4.00098	0.62357	0.62066
C	4.76921	0.31041	-0.45764
C	4.16391	-0.14464	1.90256
C	5.81525	-0.73753	-0.31587
C	5.15668	-1.04672	2.00309
C	6.02405	-1.36670	0.85030
H	6.44552	-0.96769	-1.16484
H	5.31701	-1.57398	2.93343
C	4.64764	1.03191	-1.79874
H	3.84958	1.80038	-1.77756
C	5.94291	1.77572	-2.14491
H	6.21274	2.46985	-1.32028
H	5.80068	2.37498	-3.06957
H	6.78396	1.07060	-2.31170
C	4.26021	0.04905	-2.91067
H	3.32445	-0.48451	-2.63734
H	5.06095	-0.69958	-3.08667
H	4.08020	0.59930	-3.85877
C	3.22737	0.08007	3.08778
H	2.37900	0.72696	2.78109
C	3.95320	0.79739	4.23224
H	3.24271	1.00909	5.05962
H	4.36861	1.76387	3.87884
H	4.78473	0.17631	4.62818
C	2.60292	-1.23516	3.58356
H	2.22717	-1.83641	2.73021
H	1.74995	-1.01676	4.26116
H	3.34069	-1.84812	4.14310
C	7.13628	-2.39112	0.98729
H	7.63471	-2.54333	0.00342
C	6.58941	-3.75538	1.42862
H	5.77563	-4.08481	0.75009
H	6.19323	-3.71578	2.46505
H	7.39827	-4.51598	1.39318
C	8.20888	-1.89345	1.96215
H	8.60675	-0.91353	1.62150

H	9.05228	-2.61549	2.00164
H	7.79484	-1.77566	2.98626
C	-6.76449	-1.55770	0.44199
H	-6.85012	-1.58468	1.55124
C	-8.09543	-1.02808	-0.10308
H	-8.27549	0.00307	0.26977
H	-8.93224	-1.67278	0.24091
H	-8.09161	-1.01163	-1.21409
C	-6.52216	-3.00027	-0.02169
H	-5.51426	-3.33691	0.28632
H	-6.60396	-3.09101	-1.12535
H	-7.27197	-3.67702	0.44069
H	1.13909	-2.72616	-0.17367
C	2.21251	-3.66571	-1.66852
C	-0.20161	-3.70400	-1.37071
C	3.47124	-3.25698	-1.19447
C	2.10452	-4.39028	-2.88282
C	-0.31370	-4.42618	-2.60006
C	4.60207	-3.57484	-1.92843
H	3.53791	-2.69579	-0.26771
C	3.27756	-4.69813	-3.60301
C	0.79556	-4.76058	-3.31793
H	-1.30508	-4.70118	-2.93132
C	4.51262	-4.29568	-3.13372
H	5.57896	-3.26788	-1.57409
H	3.18934	-5.25344	-4.53223
H	0.69395	-5.32256	-4.24240
H	5.41418	-4.53157	-3.68901
C	-1.29242	-3.17158	-0.60745
C	-1.24619	-3.36131	0.92307
H	-0.19412	-3.43214	1.22230
H	-1.69958	-4.33074	1.15320
N	-1.89394	-2.33591	1.70821
H	-1.25603	-1.58425	1.94379
C	-2.88231	-2.60440	2.65188
C	-2.97643	-1.79715	3.80185
C	-3.82774	-3.63415	2.48978
C	-4.00643	-1.97529	4.71951
H	-2.22212	-1.03475	3.97408
C	-4.84626	-3.81325	3.42353
H	-3.78603	-4.27306	1.61499
C	-4.95651	-2.98287	4.53945
H	-4.05681	-1.32710	5.59047
H	-5.57127	-4.60703	3.26331
H	-5.76014	-3.12143	5.25582
H	-0.99154	-1.80683	-0.97538
N	1.06064	-3.37048	-0.96741
Cl	-2.92935	-3.59083	-1.24727

Catalyst (TRIP, 3)-Enamine protonation TS (pro-S) alternative conformation
Electronic energy (Hartrees): -1870.519522
Free Energy at 1M concentration and 253 K (Hartrees): -1869.299313
Coordinates:

P	0.07470	-0.08662	-0.05414
O	1.41065	0.67992	-0.62860
O	-0.95245	1.07449	0.46602
O	0.39732	-1.01563	1.07257
O	-0.55482	-0.75445	-1.30056
C	1.83871	1.71721	0.16823
C	3.14413	1.61289	0.87682

C	3.59124	2.64607	1.60613
H	4.54743	2.58037	2.11309
C	2.77732	3.86401	1.76578
C	3.25149	4.95033	2.65429
H	4.21417	4.87161	3.14404
C	2.49137	6.03088	2.85934
H	2.83737	6.82321	3.51048
C	1.17062	6.13823	2.21205
H	0.54768	7.00230	2.40422
C	0.73650	5.16483	1.40320
H	-0.24822	5.25385	0.96514
C	1.58715	3.97165	1.13465
C	1.13258	2.87307	0.22795
C	-1.10510	2.11632	-0.41726
C	-2.41848	2.33362	-1.08131
C	-2.61213	3.43311	-1.82688
H	-3.57914	3.62350	-2.27555
C	-1.49867	4.36425	-2.09305
C	-1.69954	5.50494	-3.01632
H	-2.67206	5.68221	-3.45853
C	-0.67981	6.31833	-3.31134
H	-0.82679	7.15052	-3.98759
C	0.65294	6.07534	-2.72701
H	1.47929	6.72421	-2.98711
C	0.84326	5.05028	-1.88832
H	1.83382	4.87739	-1.48906
C	-0.28899	4.15446	-1.52638
C	-0.11045	3.01758	-0.57456
C	-3.52660	1.36470	-0.89325
C	-4.22117	1.29843	0.43929
C	-3.95298	0.57226	-1.92120
C	-5.22546	0.41767	0.60909
C	-5.10598	-0.33868	-1.69738
C	-5.70881	-0.42656	-0.50422
H	-5.73495	0.35438	1.56096
H	-5.47591	-0.93197	-2.52295
C	-3.33713	0.61749	-3.32341
H	-2.40904	1.22429	-3.33514
C	-2.92377	-0.77487	-3.82266
H	-2.26435	-1.27110	-3.08736
H	-2.36038	-0.68186	-4.77571
H	-3.80399	-1.42461	-4.00761
C	-4.31240	1.26514	-4.31414
H	-5.22962	0.64951	-4.43087
H	-3.82971	1.37354	-5.30891
H	-4.60765	2.27530	-3.96239
C	-3.86829	2.25683	1.57736
H	-3.04486	2.93470	1.27911
C	-3.37799	1.50194	2.81826
H	-2.52568	0.84567	2.54875
H	-4.18568	0.88134	3.25991
H	-3.02832	2.22206	3.58841
C	-5.05316	3.16888	1.92227
H	-5.90151	2.58914	2.34329
H	-5.39889	3.70207	1.01079
H	-4.74258	3.92862	2.67111
C	3.97493	0.38696	0.80675
C	4.84664	0.19735	-0.22107
C	4.03832	-0.50743	2.01216
C	5.91462	-0.83018	-0.07966

C	5.04659	-1.39219	2.11928
C	6.04603	-1.55206	1.04504
H	6.63679	-0.95109	-0.87560
H	5.13131	-2.01570	2.99978
C	4.83632	1.06195	-1.48070
H	4.00405	1.79388	-1.46474
C	6.12632	1.88252	-1.59521
H	6.27228	2.48843	-0.67529
H	6.05932	2.57662	-2.46009
H	7.01147	1.22802	-1.73928
C	4.61436	0.20337	-2.73130
H	3.67264	-0.37749	-2.62939
H	5.45701	-0.50068	-2.89447
H	4.52285	0.85354	-3.62743
C	2.99044	-0.41950	3.11816
H	2.15823	0.24027	2.79538
C	3.58566	0.19759	4.38910
H	2.79613	0.31135	5.16232
H	4.00090	1.20331	4.16904
H	4.39651	-0.44072	4.80022
C	2.36164	-1.78989	3.42250
H	2.09217	-2.31429	2.48176
H	1.43887	-1.65648	4.02682
H	3.05927	-2.43730	3.99453
C	7.21221	-2.50331	1.25318
H	7.11531	-2.98114	2.25327
C	8.54794	-1.74951	1.24925
H	8.52923	-0.93849	2.00850
H	8.75399	-1.30373	0.25296
H	9.37720	-2.44370	1.50351
C	7.21684	-3.63586	0.22098
H	6.23335	-4.15262	0.21736
H	7.99737	-4.38203	0.48198
H	7.43328	-3.24830	-0.79670
C	-6.90746	-1.33929	-0.31505
H	-7.13761	-1.86804	-1.26756
C	-6.61471	-2.41729	0.73173
H	-5.73372	-3.01312	0.41588
H	-7.48102	-3.10638	0.82413
H	-6.41244	-1.96786	1.72700
C	-8.15691	-0.53375	0.06017
H	-8.34539	0.25281	-0.70160
H	-8.04199	-0.05154	1.05394
H	-9.04353	-1.20227	0.09454
H	0.90508	-2.76255	-0.17885
C	1.94900	-3.59278	-1.74924
C	-0.45654	-3.66605	-1.40765
C	3.20224	-3.12940	-1.31247
C	1.82554	-4.27313	-2.98735
C	-0.58580	-4.35267	-2.65548
C	4.31165	-3.34730	-2.11083
H	3.27946	-2.58512	-0.37636
C	2.98264	-4.49952	-3.76170
C	0.51379	-4.65981	-3.40041
H	-1.58181	-4.61864	-2.98078
C	4.21216	-4.03982	-3.33186
H	5.27521	-2.96479	-1.80177
H	2.88449	-5.02585	-4.70669
H	0.40051	-5.19678	-4.33831
H	5.09985	-4.20089	-3.93423

C	-1.53022	-3.11425	-0.63437
C	-1.50458	-3.34869	0.88675
H	-0.45859	-3.47128	1.19472
H	-1.99818	-4.30504	1.08364
N	-2.13063	-2.31913	1.68974
H	-1.46796	-1.58317	1.90993
C	-3.02321	-2.62197	2.71903
C	-3.01542	-1.84609	3.89427
C	-3.97214	-3.65650	2.61676
C	-3.94166	-2.07256	4.90748
H	-2.26576	-1.06780	4.00944
C	-4.88291	-3.88595	3.64588
H	-4.02285	-4.25761	1.71526
C	-4.88518	-3.09597	4.79625
H	-3.91402	-1.45063	5.79839
H	-5.61106	-4.68520	3.53467
H	-5.60451	-3.27483	5.58925
H	-1.13221	-1.75290	-0.98279
N	0.81325	-3.36961	-0.99930
Cl	-3.17962	-3.43013	-1.29833

Catalyst (TRIP, 3)-Enamine protonation TS (pro-S) alternative conformation
Electronic energy (Hartrees): -1870.519036
Free Energy at 1M concentration and 253 K (Hartrees): -1869.299124
Coordinates:

P	0.09823	-0.12025	-0.05216
O	1.42646	0.62423	-0.67261
O	-0.91778	1.05743	0.45474
O	0.44257	-1.01659	1.09549
O	-0.55358	-0.82571	-1.26741
C	1.86246	1.69863	0.06931
C	3.18201	1.63538	0.75759
C	3.63775	2.70557	1.42586
H	4.60270	2.66831	1.91912
C	2.82172	3.92650	1.54088
C	3.30797	5.05723	2.36517
H	4.28097	5.00709	2.83802
C	2.54616	6.14294	2.53299
H	2.90095	6.96782	3.13730
C	1.21168	6.21202	1.90924
H	0.58754	7.08116	2.07251
C	0.76668	5.19814	1.15809
H	-0.22791	5.26079	0.73826
C	1.61872	3.99730	0.92992
C	1.15236	2.85331	0.08728
C	-1.10033	2.06807	-0.45955
C	-2.44122	2.27766	-1.06915
C	-2.65420	3.34250	-1.85909
H	-3.63966	3.52903	-2.26808
C	-1.54190	4.24253	-2.21959
C	-1.76780	5.33976	-3.18855
H	-2.75480	5.50577	-3.60212
C	-0.75299	6.12779	-3.55985
H	-0.91827	6.92830	-4.26932
C	0.59852	5.90016	-3.01339
H	1.41968	6.52839	-3.33364
C	0.81150	4.91441	-2.13398
H	1.81425	4.75163	-1.76153
C	-0.31359	4.04753	-1.68941
C	-0.10832	2.95527	-0.69219

C	-3.56026	1.35748	-0.75140
C	-4.14423	1.37335	0.63457
C	-4.08697	0.52954	-1.70052
C	-5.11691	0.49411	0.94354
C	-5.22520	-0.35234	-1.33259
C	-5.70006	-0.39887	-0.07865
H	-5.55074	0.49028	1.93524
H	-5.68915	-0.95718	-2.09895
C	-3.59791	0.50187	-3.15200
H	-2.68329	1.11404	-3.28319
C	-3.20907	-0.91195	-3.60378
H	-2.44138	-1.33159	-2.92726
H	-2.77592	-0.87679	-4.62628
H	-4.08432	-1.59362	-3.62395
C	-4.66434	1.08568	-4.08688
H	-5.57770	0.45376	-4.09602
H	-4.26910	1.15022	-5.12318
H	-4.94431	2.10900	-3.76008
C	-3.73748	2.43354	1.65990
H	-2.94907	3.09445	1.24933
C	-3.15374	1.80578	2.92978
H	-2.31189	1.13276	2.66831
H	-3.92403	1.23017	3.48392
H	-2.76253	2.60014	3.60061
C	-4.91675	3.35540	1.99772
H	-5.72645	2.80178	2.51835
H	-5.32745	3.80207	1.06688
H	-4.57671	4.18201	2.65768
C	4.01789	0.41127	0.74074
C	4.89124	0.18344	-0.27747
C	4.10072	-0.41558	1.99185
C	5.98601	-0.80594	-0.07951
C	5.13012	-1.26826	2.14715
C	6.13626	-1.45921	1.08426
H	6.71239	-0.95107	-0.86756
H	5.22809	-1.84169	3.05975
C	4.85662	0.97413	-1.58397
H	4.00250	1.68042	-1.60820
C	6.12236	1.82375	-1.74644
H	6.25003	2.48534	-0.86286
H	6.03599	2.46493	-2.64959
H	7.02609	1.18777	-1.85343
C	4.66002	0.03943	-2.78328
H	3.73306	-0.55832	-2.64908
H	5.52044	-0.65153	-2.90451
H	4.55432	0.63423	-3.71558
C	3.04820	-0.29350	3.08973
H	2.20458	0.33341	2.73282
C	3.62692	0.39713	4.33007
H	2.83231	0.53475	5.09414
H	4.02439	1.39819	4.06099
H	4.44763	-0.20464	4.77527
C	2.44533	-1.65934	3.45873
H	2.18149	-2.22988	2.54351
H	1.52258	-1.51494	4.06049
H	3.15666	-2.26765	4.05619
C	7.33054	-2.36095	1.34712
H	7.24868	-2.77937	2.37480
C	8.64330	-1.56948	1.29406
H	8.60000	-0.71307	2.00064

H	8.83687	-1.18168	0.27139
H	9.49267	-2.22124	1.59069
C	7.36852	-3.55393	0.38622
H	6.40582	-4.10681	0.42730
H	8.17954	-4.25281	0.68274
H	7.55900	-3.22217	-0.65614
C	-6.89900	-1.25837	0.28288
H	-7.03961	-1.23488	1.38654
C	-8.17751	-0.69644	-0.34879
H	-8.32772	0.35730	-0.02983
H	-9.05800	-1.28791	-0.01849
H	-8.12164	-0.73041	-1.45792
C	-6.69789	-2.72787	-0.10973
H	-5.72643	-3.09532	0.27156
H	-6.71904	-2.85994	-1.21197
H	-7.50386	-3.35132	0.33256
H	0.93515	-2.76472	-0.04706
C	2.01250	-3.69087	-1.54289
C	-0.39774	-3.75920	-1.23895
C	3.25615	-3.19539	-1.11393
C	1.91525	-4.45087	-2.73635
C	-0.50049	-4.52537	-2.44254
C	4.38083	-3.46098	-1.87567
H	3.31400	-2.59208	-0.21338
C	3.08702	-4.72214	-3.47312
C	0.61355	-4.87170	-3.14723
H	-1.48858	-4.82059	-2.76644
C	4.30688	-4.23097	-3.05106
H	5.33762	-3.05716	-1.57416
H	3.00789	-5.30929	-4.38343
H	0.52036	-5.46901	-4.05021
H	5.20656	-4.42721	-3.62454
C	-1.49069	-3.17825	-0.51626
C	-1.48983	-3.32410	1.01843
H	-0.44912	-3.42602	1.34667
H	-1.98597	-4.26860	1.26370
N	-2.11723	-2.25198	1.75780
H	-1.45437	-1.52363	1.99632
C	-3.14155	-2.45476	2.67933
C	-3.23679	-1.61025	3.80227
C	-4.12119	-3.45239	2.51951
C	-4.29714	-1.72290	4.69537
H	-2.45894	-0.87170	3.97396
C	-5.17058	-3.56587	3.42909
H	-4.08134	-4.11740	1.66426
C	-5.27925	-2.69974	4.51763
H	-4.34652	-1.04776	5.54567
H	-5.92073	-4.33641	3.27113
H	-6.10647	-2.78738	5.21494
H	-1.10959	-1.82149	-0.92139
N	0.86217	-3.42438	-0.82979
Cl	-3.12263	-3.56180	-1.19004

Catalyst (TRIP, 3)-Product (R) lowest free energy conformation
Electronic energy (Hartrees): -1870.573745
Free Energy at 1M concentration and 253 K (Hartrees): -1869.350893
Coordinates:

P	0.18246	-0.19362	-0.01500
O	-0.89469	0.93154	0.46697
O	1.52496	0.57941	-0.53220

O	-0.32896	-0.77008	-1.39031
O	0.35518	-1.15628	1.10001
C	-0.91580	2.11103	-0.23245
C	-2.18460	2.52615	-0.88265
C	-2.26256	3.71429	-1.49866
H	-3.19185	4.02886	-1.95881
C	-1.08123	4.59601	-1.57371
C	-1.15868	5.86219	-2.33816
H	-2.08792	6.15682	-2.80985
C	-0.08083	6.64528	-2.45264
H	-0.13779	7.56934	-3.01329
C	1.19373	6.24518	-1.82691
H	2.06861	6.87173	-1.94362
C	1.27277	5.10629	-1.12918
H	2.22477	4.82149	-0.70153
C	0.07386	4.23852	-0.96590
C	0.13343	2.96492	-0.18591
C	2.00972	1.48502	0.37511
C	3.34767	1.26338	0.97716
C	3.81680	2.12459	1.89187
H	4.80708	1.98786	2.31091
C	3.00681	3.28763	2.30808
C	3.50317	4.18518	3.37660
H	4.46197	3.99692	3.84349
C	2.76840	5.23071	3.77111
H	3.13090	5.88513	4.55332
C	1.45585	5.48963	3.14888
H	0.85727	6.32550	3.48731
C	1.00123	4.68869	2.17816
H	0.02663	4.88436	1.75108
C	1.81924	3.53770	1.70873
C	1.33374	2.62736	0.62880
C	4.23258	0.19273	0.46044
C	5.04293	0.46034	-0.77593
C	4.39611	-0.97639	1.13806
C	5.97490	-0.43541	-1.15307
C	5.44621	-1.93012	0.68907
C	6.21260	-1.67424	-0.38128
H	6.57670	-0.25192	-2.03246
H	5.62222	-2.82827	1.26638
C	3.57963	-1.32874	2.37946
H	2.75028	-0.60606	2.52844
C	2.91960	-2.70889	2.24351
H	2.38553	-2.78536	1.27485
H	2.18132	-2.85777	3.06019
H	3.66719	-3.52742	2.29939
C	4.45528	-1.26653	3.63512
H	5.27344	-2.01641	3.58981
H	3.84034	-1.46612	4.53858
H	4.90242	-0.25477	3.73681
C	4.83457	1.72360	-1.61056
H	4.05237	2.36707	-1.16311
C	4.34274	1.37874	-3.02182
H	3.42212	0.75915	-2.96099
H	5.11441	0.82079	-3.59301
H	4.09793	2.30939	-3.57705
C	6.10554	2.58148	-1.65860
H	6.92233	2.06669	-2.20673
H	6.44967	2.80641	-0.62632
H	5.89381	3.54447	-2.17069

C	-3.37710	1.64554	-0.82481
C	-4.11299	1.54026	0.31746
C	-3.81599	0.91930	-2.06649
C	-5.33798	0.69645	0.31235
C	-4.96563	0.21836	-2.04139
C	-5.76618	0.09091	-0.80446
H	-5.91615	0.60368	1.22255
H	-5.31904	-0.27468	-2.93639
C	-3.77757	2.31215	1.59530
H	-2.87185	2.93880	1.47033
C	-4.90697	3.28252	1.96250
H	-5.12239	3.95531	1.10486
H	-4.60206	3.91057	2.82681
H	-5.83582	2.73968	2.23646
C	-3.47856	1.35579	2.75452
H	-2.64296	0.68041	2.47858
H	-4.36697	0.74359	3.01565
H	-3.17176	1.93052	3.65424
C	-3.00582	0.98836	-3.36033
H	-2.03247	1.48753	-3.17817
C	-3.74177	1.81654	-4.42022
H	-3.10791	1.92588	-5.32601
H	-3.96206	2.83149	-4.02741
H	-4.69765	1.33205	-4.71261
C	-2.66429	-0.40735	-3.90311
H	-2.22018	-1.03267	-3.10378
H	-1.92493	-0.32202	-4.72787
H	-3.56451	-0.92314	-4.29802
C	-7.07206	-0.68542	-0.80710
H	-7.51414	-0.68834	0.21482
C	-6.85538	-2.15123	-1.20236
H	-6.14301	-2.63620	-0.50474
H	-6.46287	-2.23733	-2.23725
H	-7.81654	-2.70522	-1.14532
C	-8.09361	-0.01718	-1.73426
H	-8.24326	1.04232	-1.43480
H	-9.07188	-0.53872	-1.66222
H	-7.75447	-0.04682	-2.79160
C	7.34515	-2.61334	-0.76060
H	7.33625	-3.50603	-0.09521
C	7.19876	-3.12785	-2.19823
H	6.21982	-3.63007	-2.33043
H	7.99508	-3.87105	-2.41657
H	7.28148	-2.30192	-2.93526
C	8.69985	-1.92285	-0.56743
H	8.79931	-1.56688	0.48045
H	8.80714	-1.05495	-1.25261
H	9.52516	-2.63847	-0.76988
H	-0.46178	-1.79280	-1.34693
C	0.88376	-3.83808	-1.09998
C	-1.35925	-4.14028	-0.54471
C	1.89586	-3.02573	-1.67676
C	1.24479	-5.10041	-0.53538
C	-1.08448	-5.41979	0.00030
C	3.20678	-3.44097	-1.65859
H	1.62350	-2.07276	-2.11389
C	2.60722	-5.49742	-0.53414
C	0.20630	-5.88840	0.01401
H	-1.89586	-6.00388	0.41978
C	3.57087	-4.68011	-1.07684

H	3.96826	-2.80134	-2.09198
H	2.87393	-6.45310	-0.09166
H	0.44155	-6.85597	0.44906
H	4.61226	-4.98446	-1.05854
C	-2.78034	-3.61328	-0.48542
H	-3.39376	-4.33160	0.05955
C	-2.92351	-2.23025	0.17327
H	-3.97816	-1.95649	0.17831
H	-2.41625	-1.47897	-0.43440
N	-2.39276	-2.25471	1.51561
N	-0.40858	-3.38611	-1.07740
C	-3.16909	-2.18477	2.66441
C	-4.55665	-2.42200	2.67649
C	-2.53706	-1.87142	3.88675
C	-5.28010	-2.32009	3.86489
H	-5.07901	-2.68936	1.76446
C	-3.27051	-1.77885	5.06271
H	-1.46713	-1.68040	3.88901
C	-4.65184	-1.99718	5.06641
H	-6.35170	-2.50031	3.84322
H	-2.75753	-1.52507	5.98663
H	-5.22236	-1.91943	5.98638
H	-1.43524	-1.92290	1.60244
Cl	-3.50420	-3.57073	-2.17473

Catalyst (TRIP, 3)-Product (S) lowest free energy conformation
Electronic energy (Hartrees): -1870.573028
Free Energy at 1M concentration and 253 K (Hartrees): -1869.350501
Coordinates:

P	0.28464	-0.02312	-0.35566
O	1.45554	0.95676	-0.91772
O	-0.90457	0.92568	0.22797
O	0.68506	-0.98119	0.70483
O	-0.22178	-0.64259	-1.70942
C	1.79663	1.97116	-0.06321
C	3.15716	1.98160	0.52996
C	3.54929	3.02349	1.27702
H	4.54744	3.04650	1.69732
C	2.60766	4.11931	1.58320
C	3.02145	5.20528	2.50147
H	4.02650	5.22353	2.90439
C	2.15460	6.16501	2.84142
H	2.45712	6.95672	3.51458
C	0.77745	6.13845	2.31344
H	0.07299	6.90340	2.61372
C	0.39505	5.16508	1.47887
H	-0.62787	5.15076	1.12755
C	1.35790	4.10883	1.06244
C	0.96062	3.01741	0.12122
C	-1.20814	2.03542	-0.51965
C	-2.58572	2.17136	-1.05849
C	-2.93968	3.29067	-1.70718
H	-3.95318	3.41267	-2.06962
C	-1.94893	4.35591	-1.95656
C	-2.32661	5.53211	-2.77366
H	-3.33600	5.62894	-3.15383
C	-1.42209	6.47777	-3.04884
H	-1.69835	7.33597	-3.64770
C	-0.04016	6.34674	-2.54962
H	0.69358	7.10303	-2.79701

C	0.30946	5.29103	-1.80564
H	1.33495	5.20311	-1.47265
C	-0.69437	4.24692	-1.46139
C	-0.33402	3.06597	-0.61903
C	-3.59100	1.10444	-0.81857
C	-4.17863	0.94747	0.55787
C	-4.02077	0.30023	-1.83360
C	-5.12352	0.00984	0.76227
C	-5.10257	-0.68502	-1.56546
C	-5.63342	-0.82863	-0.34315
H	-5.55804	-0.11540	1.74461
H	-5.48994	-1.27631	-2.38458
C	-3.47609	0.40075	-3.26020
H	-2.59220	1.06977	-3.30580
C	-2.99447	-0.95887	-3.78698
H	-2.28307	-1.42018	-3.07574
H	-2.46983	-0.82354	-4.75688
H	-3.84011	-1.65910	-3.94851
C	-4.53510	0.99169	-4.19824
H	-5.41898	0.32334	-4.27445
H	-4.10854	1.13204	-5.21442
H	-4.87003	1.98191	-3.82488
C	-3.76423	1.85252	1.71834
H	-3.00765	2.59123	1.38990
C	-3.11747	1.04568	2.85095
H	-2.26248	0.46180	2.45431
H	-3.84266	0.34636	3.31775
H	-2.73291	1.73028	3.63667
C	-4.95063	2.67631	2.23648
H	-5.72144	2.02836	2.70422
H	-5.41429	3.24105	1.39943
H	-4.60154	3.40778	2.99643
C	4.07253	0.83391	0.31070
C	4.66143	0.62659	-0.90153
C	4.37457	-0.09561	1.45637
C	5.56757	-0.53985	-1.07420
C	5.25735	-1.09455	1.27054
C	5.86093	-1.35522	-0.05239
H	6.01157	-0.72275	-2.04404
H	5.52110	-1.74568	2.09239
C	4.47889	1.57313	-2.08922
H	3.78709	2.40455	-1.84697
C	5.81048	2.23402	-2.46514
H	6.23681	2.75630	-1.58187
H	5.64915	2.98567	-3.26728
H	6.54540	1.48531	-2.82886
C	3.87221	0.84348	-3.29538
H	2.92320	0.34704	-3.00694
H	4.56616	0.07706	-3.69916
H	3.64767	1.57107	-4.10429
C	3.72016	0.07672	2.82715
H	2.90753	0.82905	2.77358
C	4.73768	0.58345	3.85623
H	4.23299	0.77853	4.82653
H	5.19642	1.53233	3.50793
H	5.54460	-0.16242	4.01933
C	3.05662	-1.21991	3.31803
H	2.41566	-1.65143	2.52258
H	2.42276	-1.00654	4.20513
H	3.81126	-1.97878	3.61292

C	6.79244	-2.53682	-0.25169
H	7.08977	-2.60917	-1.32230
C	6.09555	-3.85462	0.10911
H	5.12599	-3.93192	-0.42427
H	5.90503	-3.92475	1.20088
H	6.73025	-4.71486	-0.19273
C	8.07848	-2.35739	0.56203
H	8.57727	-1.40576	0.27902
H	8.78033	-3.19284	0.35312
H	7.86408	-2.33936	1.65179
C	-6.79629	-1.78018	-0.11476
H	-7.04498	-2.31047	-1.06167
C	-6.44818	-2.85739	0.91935
H	-5.59746	-3.46965	0.55864
H	-7.31391	-3.53782	1.06627
H	-6.18322	-2.40903	1.89998
C	-8.05193	-1.00705	0.30553
H	-8.28815	-0.22573	-0.44837
H	-7.91173	-0.52132	1.29452
H	-8.91992	-1.69730	0.37169
H	-0.13945	-1.68671	-1.69661
C	1.24096	-3.75562	-1.88178
C	-0.84106	-3.91246	-0.83558
C	2.18155	-2.96064	-2.58349
C	1.55690	-5.10878	-1.55819
C	-0.59847	-5.26044	-0.46851
C	3.39000	-3.50270	-2.95949
H	1.92866	-1.93023	-2.80624
C	2.80717	-5.63776	-1.96836
C	0.58802	-5.84804	-0.83103
H	-1.35090	-5.80768	0.08700
C	3.70182	-4.85133	-2.65973
H	4.11364	-2.89056	-3.48893
H	3.04731	-6.66909	-1.72585
H	0.79899	-6.87918	-0.56105
H	4.65970	-5.25967	-2.96654
N	0.04242	-3.20480	-1.52060
C	-2.12822	-3.20154	-0.47719
H	-2.06302	-2.16798	-0.81193
C	-2.52469	-3.23951	1.00105
H	-2.79053	-4.26871	1.27971
H	-3.43922	-2.63828	1.10845
N	-1.45307	-2.74055	1.83649
H	-0.74011	-2.17043	1.38274
C	-1.72282	-2.35701	3.14815
C	-0.79294	-1.54016	3.82446
C	-2.88416	-2.76060	3.83162
C	-1.02189	-1.14738	5.13605
H	0.08382	-1.19900	3.28502
C	-3.09842	-2.35781	5.15154
H	-3.62135	-3.38771	3.34162
C	-2.17630	-1.55231	5.81593
H	-0.29421	-0.50970	5.63135
H	-4.00380	-2.67987	5.65940
H	-2.35214	-1.24061	6.84078
Cl	-3.49342	-3.92824	-1.46546

Catalyst (TRIP, 3)-Product (S) alternative conformation
Electronic energy (Hartrees): -1870.572705
Free Energy at 1M concentration and 253 K (Hartrees): -1869.350318

Coordinates:

P	-0.18536	-1.24251	-0.74508
O	0.86959	-0.31011	0.13342
O	-1.52328	-1.44672	0.16505
O	0.36523	-2.53849	-1.17834
O	-0.62301	-0.20267	-1.85012
C	0.92248	-0.62148	1.46823
C	2.18011	-1.17135	2.03787
C	2.28258	-1.36761	3.36026
H	3.20245	-1.75419	3.78288
C	1.13019	-1.11336	4.24679
C	1.23293	-1.42798	5.69043
H	2.16521	-1.79385	6.10245
C	0.17397	-1.26627	6.49070
H	0.24930	-1.49859	7.54511
C	-1.10553	-0.78563	5.93593
H	-1.96579	-0.68074	6.58434
C	-1.20728	-0.49329	4.63434
H	-2.16284	-0.16711	4.24619
C	-0.02841	-0.63641	3.73610
C	-0.11070	-0.30365	2.28192
C	-2.00727	-0.28855	0.72505
C	-3.34164	0.21128	0.31022
C	-3.78896	1.38477	0.77923
H	-4.77464	1.74121	0.50364
C	-2.97432	2.16794	1.72987
C	-3.44735	3.49732	2.17922
H	-4.37838	3.89986	1.80013
C	-2.72449	4.20909	3.05016
H	-3.06772	5.18328	3.37325
C	-1.45321	3.67157	3.56908
H	-0.86490	4.25980	4.26138
C	-1.01659	2.47167	3.16949
H	-0.06663	2.11253	3.54111
C	-1.81181	1.66326	2.20585
C	-1.32791	0.33759	1.71306
C	-4.24743	-0.66183	-0.47248
C	-4.92381	-1.80976	0.22553
C	-4.49695	-0.42816	-1.79073
C	-5.75436	-2.60300	-0.47710
C	-5.46339	-1.30084	-2.50835
C	-6.05150	-2.34072	-1.90147
H	-6.24060	-3.44026	0.00483
H	-5.69169	-1.09165	-3.54529
C	-3.82053	0.69257	-2.57662
H	-3.03160	1.18291	-1.97338
C	-3.12392	0.14891	-3.83382
H	-2.50148	-0.73487	-3.58047
H	-2.46241	0.92606	-4.26955
H	-3.85930	-0.14918	-4.61000
C	-4.83347	1.78203	-2.94311
H	-5.62890	1.38251	-3.60737
H	-4.32183	2.61790	-3.46600
H	-5.30563	2.18662	-2.02269
C	-4.70488	-2.08682	1.71376
H	-4.03773	-1.32582	2.16305
C	-4.01986	-3.44169	1.93191
H	-3.07086	-3.48515	1.35622
H	-4.67334	-4.27996	1.61052
H	-3.77771	-3.57607	3.00786

C	-6.01912	-1.99791	2.50111
H	-6.72063	-2.80914	2.21409
H	-6.50670	-1.01698	2.31506
H	-5.81521	-2.08223	3.59005
C	3.31477	-1.53449	1.15397
C	4.16062	-0.58255	0.67026
C	3.58987	-2.98788	0.87867
C	5.33004	-1.00248	-0.14686
C	4.70101	-3.32447	0.19733
C	5.60700	-2.29672	-0.35771
H	5.99270	-0.24524	-0.54534
H	4.94181	-4.36573	0.03350
C	4.00385	0.90333	0.98952
H	3.10811	1.08670	1.61314
C	5.19697	1.41999	1.80121
H	5.32408	0.80751	2.71951
H	5.01897	2.47277	2.10904
H	6.13573	1.38119	1.20959
C	3.81462	1.72529	-0.28875
H	2.93513	1.34349	-0.84054
H	4.70560	1.67086	-0.94816
H	3.62939	2.79127	-0.03879
C	2.66030	-4.08818	1.38979
H	1.72107	-3.64440	1.77892
C	3.31065	-4.85448	2.54779
H	2.59871	-5.60276	2.95696
H	3.58467	-4.15517	3.36513
H	4.22734	-5.38347	2.21020
C	2.23666	-5.05548	0.27271
H	1.90067	-4.49366	-0.62173
H	1.39522	-5.69096	0.62271
H	3.07165	-5.72395	-0.02515
C	6.84643	-2.70246	-1.13509
H	7.36958	-1.79605	-1.51532
C	6.48285	-3.55378	-2.35726
H	5.73824	-3.02255	-2.98368
H	6.06021	-4.53514	-2.05530
H	7.38800	-3.73667	-2.97477
C	7.83432	-3.44403	-0.22784
H	8.09641	-2.81047	0.64652
H	8.76844	-3.66887	-0.78558
H	7.40213	-4.39949	0.13855
C	-7.02060	-3.22780	-2.66199
H	-7.11530	-2.87367	-3.71339
C	-6.50879	-4.67160	-2.72218
H	-5.49167	-4.69613	-3.16874
H	-7.18125	-5.28682	-3.35741
H	-6.46747	-5.12634	-1.70979
C	-8.42142	-3.16614	-2.04223
H	-8.77345	-2.11305	-2.00238
H	-8.42521	-3.58512	-1.01370
H	-9.13691	-3.74762	-2.66203
H	0.09501	0.39164	-2.27955
C	2.29827	0.69268	-3.44315
C	1.22765	2.69793	-2.91576
C	2.29415	-0.72748	-3.44751
C	3.44706	1.38541	-3.94238
C	2.32738	3.44982	-3.40195
C	3.38663	-1.41560	-3.92509
H	1.44033	-1.27281	-3.06351

C	4.55227	0.64288	-4.43105
C	3.42533	2.79883	-3.90645
H	2.28864	4.53274	-3.36280
C	4.52211	-0.73173	-4.42323
H	3.37366	-2.50077	-3.91503
H	5.41837	1.17974	-4.80770
H	4.28243	3.35653	-4.27400
H	5.36974	-1.29627	-4.79873
N	1.21785	1.37125	-2.93737
C	0.03609	3.46113	-2.36868
H	0.33581	4.49998	-2.22645
C	-0.57234	2.93382	-1.05730
H	-0.88648	1.90127	-1.18917
H	-1.47247	3.51671	-0.83702
N	0.36529	3.00240	0.03441
H	0.85856	2.15162	0.25797
C	0.62738	4.13446	0.78948
C	1.58238	4.07671	1.82517
C	-0.04953	5.35242	0.58164
C	1.83899	5.18475	2.62307
H	2.10509	3.14522	2.01614
C	0.21737	6.45550	1.39228
H	-0.79490	5.44134	-0.20098
C	1.15971	6.38837	2.41714
H	2.57572	5.10307	3.41748
H	-0.32577	7.37948	1.21383
H	1.36104	7.25190	3.04241
Cl	-1.27725	3.54125	-3.65209

Catalyst (TRIP, 3)-Product (S) alternative conformation
Electronic energy (Hartrees): -1870.5703
Free Energy at 1M concentration and 253 K (Hartrees): -1869.349536
Coordinates:

P	-0.20905	0.12568	0.22786
O	-1.54771	0.77120	0.89878
O	0.57126	1.32833	-0.55711
O	-0.69922	-0.73184	-0.99194
O	0.58144	-0.57066	1.27153
C	-2.29772	1.53331	0.03775
C	-3.66943	1.08647	-0.31676
C	-4.41749	1.82632	-1.14781
H	-5.42863	1.52036	-1.38861
C	-3.87259	3.06211	-1.74425
C	-4.67618	3.81572	-2.73417
H	-5.67162	3.47921	-2.99616
C	-4.17473	4.91275	-3.31160
H	-4.76171	5.46164	-4.03660
C	-2.81392	5.37075	-2.97261
H	-2.40497	6.24564	-3.46139
C	-2.08228	4.70069	-2.07485
H	-1.07770	5.04030	-1.86088
C	-2.63802	3.49628	-1.39946
C	-1.84330	2.73209	-0.39077
C	0.56961	2.55216	0.06306
C	1.87072	3.16503	0.43323
C	1.90905	4.41901	0.90836
H	2.85852	4.87698	1.15991
C	0.65930	5.16728	1.14339
C	0.71368	6.50708	1.77248
H	1.66890	6.95453	2.01767

C	-0.41635	7.16835	2.04422
H	-0.37585	8.14647	2.50579
C	-1.72288	6.55987	1.72990
H	-2.63437	7.08952	1.97528
C	-1.78251	5.35237	1.15670
H	-2.75229	4.91524	0.96006
C	-0.53295	4.61738	0.81712
C	-0.57093	3.27470	0.16091
C	3.12994	2.40131	0.25948
C	3.67016	2.17741	-1.12658
C	3.81195	1.92173	1.33886
C	4.82319	1.49846	-1.27554
C	5.10399	1.21969	1.12203
C	5.58341	1.00063	-0.10984
H	5.22654	1.32648	-2.26416
H	5.66499	0.87455	1.98039
C	3.31267	2.08070	2.77689
H	2.30002	2.53135	2.80783
C	3.17900	0.72413	3.48158
H	2.54688	0.04143	2.87890
H	2.69292	0.85782	4.47157
H	4.16840	0.24855	3.64465
C	4.23562	3.01383	3.56879
H	5.25297	2.58028	3.67081
H	3.82172	3.18624	4.58527
H	4.31541	3.99608	3.05670
C	2.96188	2.72466	-2.36568
H	2.03222	3.25585	-2.08312
C	2.54394	1.59476	-3.31785
H	1.95886	0.82833	-2.76874
H	3.42766	1.10412	-3.77726
H	1.90620	2.00033	-4.13202
C	3.83114	3.76078	-3.08976
H	4.75022	3.29779	-3.50684
H	4.12340	4.56872	-2.38519
H	3.25942	4.21904	-3.92494
C	-4.24103	-0.13617	0.29717
C	-4.66989	-0.12998	1.59080
C	-4.41502	-1.37231	-0.54181
C	-5.29504	-1.35753	2.15019
C	-5.03900	-2.44029	-0.01025
C	-5.48750	-2.44985	1.39827
H	-5.61630	-1.35987	3.18367
H	-5.19989	-3.32688	-0.60823
C	-4.58026	1.10342	2.49161
H	-4.12280	1.96468	1.96538
C	-5.97622	1.57262	2.91936
H	-6.60534	1.75641	2.02210
H	-5.89935	2.52349	3.48892
H	-6.47839	0.81981	3.56251
C	-3.69454	0.82849	3.71310
H	-2.69483	0.47080	3.38720
H	-4.14821	0.06445	4.37855
H	-3.55374	1.76274	4.29761
C	-3.90592	-1.44131	-1.98015
H	-3.26201	-0.56469	-2.19797
C	-5.07293	-1.40032	-2.97318
H	-4.68624	-1.39143	-4.01463
H	-5.67505	-0.48050	-2.81941
H	-5.73458	-2.28374	-2.84655

C	-3.03210	-2.68372	-2.22139
H	-2.33553	-2.83538	-1.37266
H	-2.43632	-2.55533	-3.15041
H	-3.64949	-3.60040	-2.32960
C	-6.14353	-3.68489	1.98880
H	-6.41619	-3.49903	3.05222
C	-5.17742	-4.87496	1.97720
H	-4.24725	-4.61324	2.52544
H	-4.91241	-5.16830	0.93927
H	-5.64298	-5.74793	2.48245
C	-7.44248	-4.02329	1.24818
H	-8.12554	-3.14703	1.25855
H	-7.95692	-4.86976	1.75121
H	-7.24208	-4.31010	0.19419
C	6.89578	0.26361	-0.30605
H	7.32259	-0.02295	0.68092
C	6.68175	-1.03291	-1.09596
H	5.92066	-1.66598	-0.59255
H	7.63106	-1.60755	-1.14693
H	6.34160	-0.82086	-2.13169
C	7.93167	1.16120	-0.99259
H	8.06797	2.09802	-0.41093
H	7.61741	1.42221	-2.02525
H	8.91118	0.63957	-1.04416
H	-0.22841	-1.65592	-1.13421
C	0.90487	-3.09765	-2.78835
C	0.45774	-4.04168	-0.69913
C	0.82910	-1.97074	-3.64508
C	1.49782	-4.30651	-3.26002
C	1.01793	-5.28206	-1.10085
C	1.33815	-2.04841	-4.92243
H	0.36663	-1.06347	-3.27266
C	2.00899	-4.35200	-4.58169
C	1.53455	-5.40694	-2.36517
H	1.02293	-6.11445	-0.40712
C	1.93238	-3.24347	-5.39520
H	1.28383	-1.18324	-5.57608
H	2.46102	-5.27304	-4.93896
H	1.97313	-6.34532	-2.69356
H	2.32711	-3.28253	-6.40568
C	-0.09589	-3.84254	0.69547
C	0.95339	-4.04622	1.79810
H	1.33002	-5.07204	1.74202
H	0.46159	-3.93413	2.77719
N	2.03694	-3.11952	1.56611
N	0.40314	-3.00403	-1.51743
C	3.29817	-3.28749	2.13363
C	4.26876	-2.28957	1.90847
C	3.66641	-4.41299	2.89283
C	5.55084	-2.41399	2.42666
H	3.99426	-1.42392	1.31291
C	4.96418	-4.53260	3.39423
H	2.94255	-5.18989	3.11383
C	5.91596	-3.54022	3.17140
H	6.27710	-1.62772	2.24436
H	5.22272	-5.41173	3.97871
H	6.92073	-3.63668	3.57047
H	1.73052	-2.15187	1.47163
H	-0.52182	-2.84465	0.77594
Cl	-1.49764	-4.99007	0.97552

Catalyst (TRIP, 3)-Product (S) alternative conformation
Electronic energy (Hartrees): -1870.571721
Free Energy at 1M concentration and 253 K (Hartrees): -1869.34926
Coordinates:

P	0.27545	-0.08396	-0.21912
O	-0.84369	0.96773	0.32397
O	1.53493	0.78402	-0.77907
O	-0.26196	-0.68829	-1.57041
O	0.57759	-1.05175	0.86398
C	-1.08512	2.04987	-0.48363
C	-2.46038	2.25348	-1.00772
C	-2.74003	3.34310	-1.73819
H	-3.74689	3.51184	-2.10092
C	-1.67794	4.31232	-2.07236
C	-1.97836	5.45055	-2.97117
H	-2.98113	5.59032	-3.35551
C	-1.01189	6.30856	-3.31501
H	-1.23169	7.13899	-3.97346
C	0.36079	6.11800	-2.80889
H	1.14339	6.80256	-3.10970
C	0.64108	5.09619	-1.99177
H	1.65941	4.96136	-1.65211
C	-0.43025	4.15109	-1.57444
C	-0.14602	3.00933	-0.65359
C	1.91031	1.83410	0.01801
C	3.26126	1.81893	0.63408
C	3.69843	2.89203	1.30929
H	4.69366	2.89754	1.73736
C	2.81300	4.05332	1.52168
C	3.27240	5.18002	2.36627
H	4.27167	5.17496	2.78366
C	2.45404	6.20630	2.62112
H	2.78972	7.02815	3.24031
C	1.08458	6.21326	2.07325
H	0.41784	7.03350	2.30624
C	0.66197	5.20311	1.30440
H	-0.35604	5.21671	0.93928
C	1.57288	4.07059	0.98045
C	1.13247	2.93732	0.10986
C	4.13429	0.62760	0.49298
C	4.79841	0.35198	-0.82817
C	4.36649	-0.19854	1.55330
C	5.61679	-0.71228	-0.93785
C	5.32411	-1.32390	1.39567
C	5.91482	-1.58074	0.22125
H	6.09923	-0.92997	-1.88096
H	5.55127	-1.94513	2.25201
C	3.69455	-0.01295	2.91526
H	2.89452	0.75442	2.86708
C	3.00546	-1.30184	3.38768
H	2.36355	-1.71502	2.58372
H	2.36615	-1.08615	4.27039
H	3.74417	-2.07621	3.68188
C	4.71277	0.46358	3.95729
H	5.50557	-0.29760	4.11822
H	4.20440	0.65667	4.92607
H	5.19024	1.40841	3.62341
C	4.59675	1.26688	-2.03575
H	3.90977	2.09905	-1.78640

C	3.95636	0.51428	-3.21026
H	3.00843	0.03707	-2.88675
H	4.63397	-0.27069	-3.60698
H	3.72207	1.22336	-4.03270
C	5.91591	1.92627	-2.45892
H	6.64154	1.17494	-2.83572
H	6.36677	2.46042	-1.59520
H	5.72870	2.66738	-3.26521
C	-3.53997	1.28782	-0.67947
C	-4.07108	1.23402	0.57578
C	-4.10775	0.41608	-1.76809
C	-5.20984	0.31590	0.84030
C	-5.17044	-0.36520	-1.49151
C	-5.76194	-0.41547	-0.13667
H	-5.63495	0.28032	1.83490
H	-5.61642	-0.96976	-2.26921
C	-3.60452	2.13660	1.72028
H	-2.77877	2.80500	1.40690
C	-4.73339	3.06585	2.18324
H	-5.12418	3.64637	1.32017
H	-4.34784	3.78464	2.93769
H	-5.56846	2.49519	2.64079
C	-3.06478	1.30508	2.89003
H	-2.25372	0.63768	2.53469
H	-3.86150	0.68445	3.35098
H	-2.64385	1.97308	3.67143
C	-3.53832	0.43460	-3.18739
H	-2.61674	1.04944	-3.22564
C	-4.53592	1.06590	-4.16667
H	-4.07332	1.16332	-5.17202
H	-4.82448	2.08026	-3.81937
H	-5.45312	0.44613	-4.25813
C	-3.12268	-0.96404	-3.66596
H	-2.41383	-1.42065	-2.94854
H	-2.61125	-0.89052	-4.64944
H	-3.99947	-1.63459	-3.78179
C	-7.01157	-1.23647	0.13470
H	-7.30726	-1.13645	1.20353
C	-6.77067	-2.72729	-0.11972
H	-5.93345	-3.09032	0.51173
H	-6.53513	-2.91894	-1.18751
H	-7.67783	-3.30962	0.14865
C	-8.18917	-0.72308	-0.70202
H	-8.35194	0.35850	-0.50633
H	-9.11753	-1.26815	-0.42758
H	-8.00322	-0.86749	-1.78750
C	6.89144	-2.73564	0.08975
H	7.01558	-3.24278	1.07324
C	6.36100	-3.78742	-0.88871
H	5.36967	-4.15123	-0.54491
H	7.05281	-4.65562	-0.92867
H	6.25852	-3.36910	-1.91239
C	8.27928	-2.23940	-0.33268
H	8.63928	-1.46537	0.37848
H	8.25887	-1.80664	-1.35516
H	9.00278	-3.08241	-0.32288
H	-0.23973	-1.73264	-1.54877
C	1.03216	-3.88479	-1.69171
C	-1.05639	-3.91273	-0.65043
C	2.00702	-3.15957	-2.42123

C	1.28416	-5.24047	-1.32475
C	-0.87625	-5.25678	-0.23617
C	3.18514	-3.77209	-2.78458
H	1.80247	-2.12614	-2.67728
C	2.50603	-5.84209	-1.71973
C	0.28287	-5.90954	-0.57414
H	-1.65461	-5.75083	0.33320
C	3.43363	-5.12322	-2.44018
H	3.93353	-3.21425	-3.33899
H	2.69703	-6.87544	-1.44429
H	0.44598	-6.93970	-0.26949
H	4.36802	-5.58813	-2.73894
C	-2.32121	-3.14200	-0.34203
C	-2.76622	-3.13199	1.12208
H	-3.08130	-4.14314	1.41615
H	-3.66135	-2.49497	1.18611
N	-1.69759	-2.65689	1.97440
N	-0.13912	-3.26655	-1.35125
C	-1.97345	-2.22491	3.26865
C	-0.99212	-1.48617	3.96291
C	-3.18859	-2.50621	3.91907
C	-1.22856	-1.04391	5.25730
H	-0.06477	-1.24715	3.45266
C	-3.40878	-2.05546	5.22242
H	-3.96401	-3.07387	3.41571
C	-2.43883	-1.32313	5.90284
H	-0.46072	-0.46803	5.76741
H	-4.35665	-2.28144	5.70397
H	-2.61932	-0.97322	6.91445
H	-0.93506	-2.15005	1.52670
H	-2.20327	-2.11855	-0.69312
Cl	-3.67512	-3.84011	-1.36414

Catalyst (TRIP, 3)-Product (R) alternative conformation
Electronic energy (Hartrees): -1870.571852
Free Energy at 1M concentration and 253 K (Hartrees): -1869.349142
Coordinates:

P	0.41329	-1.19001	-0.88753
O	-0.71881	-0.38437	0.01281
O	1.71803	-1.42301	0.06136
O	-0.06033	-2.46249	-1.46254
O	0.85179	-0.03434	-1.87094
C	-0.83966	-0.83022	1.30390
C	-2.11377	-1.45949	1.74127
C	-2.27467	-1.81398	3.02435
H	-3.20614	-2.26069	3.35219
C	-1.17287	-1.65006	3.99189
C	-1.33760	-2.14018	5.37986
H	-2.27996	-2.56937	5.69696
C	-0.32231	-2.05935	6.24628
H	-0.44284	-2.41975	7.25967
C	0.97105	-1.49351	5.81869
H	1.79699	-1.45468	6.51718
C	1.12816	-1.04287	4.56882
H	2.09385	-0.65688	4.27107
C	-0.00327	-1.09360	3.60210
C	0.14088	-0.58411	2.20480
C	2.11377	-0.31271	0.76728
C	3.44689	0.28038	0.48895
C	3.83483	1.38433	1.14317

H	4.81862	1.80357	0.96875
C	2.94964	2.00982	2.14421
C	3.35929	3.27593	2.79446
H	4.29830	3.74509	2.52826
C	2.56861	3.84921	3.70755
H	2.86612	4.77708	4.17853
C	1.28867	3.21745	4.07718
H	0.65005	3.68825	4.81350
C	0.91473	2.06906	3.50214
H	-0.03150	1.62993	3.78450
C	1.77794	1.42078	2.47846
C	1.36145	0.16135	1.78712
C	4.40430	-0.42423	-0.39746
C	5.06220	-1.68224	0.10152
C	4.72568	0.06647	-1.62797
C	5.93864	-2.32111	-0.69614
C	5.74107	-0.64742	-2.44674
C	6.30642	-1.78631	-2.02419
H	6.41564	-3.23084	-0.35767
H	6.02760	-0.23698	-3.40595
C	4.09247	1.33006	-2.20948
H	3.26020	1.68818	-1.57085
C	3.48530	1.06910	-3.59690
H	2.81282	0.18695	-3.56539
H	2.89444	1.95001	-3.92566
H	4.27085	0.88753	-4.35931
C	5.11908	2.46569	-2.27655
H	5.95199	2.21150	-2.96605
H	4.63221	3.39761	-2.63578
H	5.53885	2.66185	-1.26748
C	4.78816	-2.23359	1.50175
H	4.07884	-1.58401	2.04995
C	4.14038	-3.62290	1.43826
H	3.22460	-3.58913	0.81099
H	4.83829	-4.37430	1.01256
H	3.84856	-3.95246	2.45835
C	6.06492	-2.25834	2.35282
H	6.80606	-2.98159	1.95238
H	6.52463	-1.24704	2.37582
H	5.81982	-2.55110	3.39623
C	-3.20560	-1.72828	0.77284
C	-4.08393	-0.75080	0.41428
C	-3.43548	-3.14167	0.31392
C	-5.25916	-1.10831	-0.42599
C	-4.56102	-3.43463	-0.36390
C	-5.51972	-2.38081	-0.75820
H	-5.95533	-0.33139	-0.71413
H	-4.77161	-4.45641	-0.64770
C	-3.97407	0.68704	0.91972
H	-3.09591	0.81465	1.58115
C	-5.19338	1.06929	1.76747
H	-5.33055	0.33300	2.58821
H	-5.03908	2.07051	2.22405
H	-6.11854	1.10421	1.15494
C	-3.78591	1.66533	-0.24410
H	-2.82398	1.45113	-0.75070
H	-4.61190	1.58921	-0.98117
H	-3.75163	2.71038	0.12875
C	-2.43767	-4.25354	0.63175
H	-1.50035	-3.81750	1.03550

C	-2.99588	-5.19772	1.70306
H	-2.23536	-5.96209	1.97047
H	-3.25130	-4.62882	2.62121
H	-3.90892	-5.71625	1.34028
C	-2.03197	-5.04104	-0.62496
H	-1.13665	-5.66228	-0.40874
H	-1.78611	-4.34907	-1.45617
H	-2.84673	-5.71624	-0.96159
C	-6.80624	-2.74036	-1.48183
H	-7.36654	-1.81398	-1.74201
C	-6.52955	-3.47815	-2.79697
H	-5.86440	-2.87194	-3.44192
H	-6.05405	-4.46500	-2.61701
H	-7.48117	-3.64356	-3.34576
C	-7.71561	-3.57497	-0.57321
H	-7.92089	-3.02343	0.36930
H	-8.68544	-3.76952	-1.07899
H	-7.24446	-4.54944	-0.32295
C	7.32615	-2.50598	-2.88846
H	7.47112	-1.95439	-3.84478
C	6.83764	-3.91219	-3.25462
H	5.84607	-3.85257	-3.75242
H	7.55101	-4.39077	-3.95907
H	6.74865	-4.55458	-2.35303
C	8.69164	-2.56161	-2.19372
H	9.02649	-1.53534	-1.93077
H	8.64755	-3.17318	-1.26774
H	9.44658	-3.00862	-2.87523
H	0.07101	0.44439	-2.34605
C	-2.23589	0.83046	-3.41803
C	-0.95107	2.68029	-2.79730
C	-2.35834	-0.57897	-3.51528
C	-3.33228	1.66745	-3.78979
C	-1.98960	3.57681	-3.14432
C	-3.53389	-1.12679	-3.97599
H	-1.53461	-1.21477	-3.20566
C	-4.52636	1.06613	-4.26298
C	-3.16599	3.06677	-3.63919
H	-1.84459	4.64049	-3.01091
C	-4.62286	-0.30353	-4.35520
H	-3.62231	-2.20596	-4.04576
H	-5.36027	1.70294	-4.54452
H	-3.98390	3.73011	-3.90719
H	-5.53985	-0.75717	-4.71775
N	-1.07261	1.36914	-2.94225
C	0.35211	3.11291	-2.15153
C	0.25138	3.27118	-0.62838
H	-0.15161	2.32368	-0.26273
H	1.27026	3.37190	-0.22072
N	-0.62117	4.36228	-0.24877
H	-0.27953	5.25704	-0.57674
C	-1.24148	4.43755	1.00299
C	-1.64908	5.69560	1.48590
C	-1.53216	3.30316	1.77769
C	-2.33582	5.80835	2.68985
H	-1.42725	6.58625	0.90225
C	-2.22903	3.42770	2.97913
H	-1.23089	2.31939	1.43660
C	-2.63597	4.67485	3.44920
H	-2.63737	6.79258	3.03725

H	-2.45187	2.53231	3.55316
H	-3.17386	4.76401	4.38732
H	1.10071	2.35410	-2.35820
Cl	1.01157	4.64632	-2.90717

Catalyst (TRIP, 3)-Product (S) alternative conformation
Electronic energy (Hartrees): -1870.569832
Free Energy at 1M concentration and 253 K (Hartrees): -1869.349005
Coordinates:

P	0.11021	-0.00071	0.26777
O	-0.87552	1.27305	0.50260
O	1.56292	0.58163	-0.22389
O	-0.36426	-0.69161	-1.06118
O	0.12270	-0.82213	1.50155
C	-0.79772	2.26604	-0.44053
C	-1.99846	2.56861	-1.25618
C	-1.96318	3.55959	-2.15766
H	-2.84726	3.80293	-2.73464
C	-0.72119	4.33159	-2.36706
C	-0.66918	5.36438	-3.42730
H	-1.54639	5.57277	-4.02719
C	0.46213	6.04138	-3.65126
H	0.49990	6.79426	-4.42794
C	1.66677	5.75519	-2.84909
H	2.58546	6.29067	-3.05116
C	1.62887	4.82901	-1.88419
H	2.53127	4.61924	-1.32569
C	0.36944	4.08750	-1.60217
C	0.30064	3.04836	-0.53036
C	2.02754	1.64428	0.50753
C	3.29406	1.49554	1.26266
C	3.77075	2.52308	1.97972
H	4.71355	2.42930	2.50590
C	3.03176	3.80129	2.03137
C	3.52659	4.90226	2.88946
H	4.43508	4.77680	3.46534
C	2.85262	6.05535	2.95850
H	3.21406	6.85890	3.58711
C	1.60779	6.23233	2.18642
H	1.05627	7.16049	2.26326
C	1.15546	5.24639	1.40301
H	0.22909	5.39005	0.86289
C	1.90885	3.96776	1.29331
C	1.42579	2.85189	0.42533
C	4.09807	0.26290	1.10620
C	4.92491	0.09194	-0.13701
C	4.09624	-0.70700	2.06114
C	5.63572	-1.04039	-0.29652
C	4.95543	-1.90501	1.87231
C	5.67097	-2.08275	0.75287
H	6.22576	-1.18793	-1.19087
H	4.99633	-2.65153	2.65452
C	3.25157	-0.61341	3.33093
H	2.51280	0.21194	3.25727
C	2.43240	-1.89308	3.56434
H	1.94512	-2.22167	2.62304
H	1.64166	-1.70290	4.32126
H	3.07005	-2.72260	3.93567
C	4.14127	-0.31249	4.54115
H	4.87672	-1.12851	4.70706

H	3.51854	-0.20231	5.45456
H	4.69313	0.63799	4.38098
C	5.00006	1.18513	-1.20405
H	4.38699	2.06106	-0.91345
C	4.44091	0.70011	-2.54658
H	3.40313	0.33007	-2.41362
H	5.06538	-0.11236	-2.97297
H	4.41534	1.54030	-3.27298
C	6.43229	1.71257	-1.36133
H	7.10944	0.93144	-1.76656
H	6.82143	2.05159	-0.37735
H	6.44357	2.57990	-2.05563
C	-3.25525	1.82559	-1.00730
C	-4.01478	2.10218	0.08961
C	-3.68968	0.75508	-1.97017
C	-5.22183	1.27740	0.35647
C	-4.83260	0.08659	-1.72707
C	-5.61539	0.31976	-0.49458
H	-5.79613	1.46095	1.25515
H	-5.18935	-0.65292	-2.43042
C	-3.70564	3.26117	1.04114
H	-2.81081	3.82997	0.71615
C	-4.85705	4.27417	1.05720
H	-5.07223	4.62005	0.02343
H	-4.57620	5.15966	1.66673
H	-5.77985	3.83191	1.48802
C	-3.40199	2.75778	2.45643
H	-2.55082	2.04654	2.43302
H	-4.28377	2.25522	2.90486
H	-3.11601	3.61001	3.10921
C	-2.89503	0.45108	-3.23936
H	-1.89211	0.92134	-3.18047
C	-3.59893	1.03972	-4.46701
H	-2.98476	0.86972	-5.37701
H	-3.73701	2.13416	-4.34163
H	-4.59499	0.57045	-4.61586
C	-2.64597	-1.05523	-3.43132
H	-2.31793	-1.52235	-2.48212
H	-1.85145	-1.21291	-4.19165
H	-3.56181	-1.57839	-3.77844
C	-6.84420	-0.51871	-0.19535
H	-7.24836	-0.25461	0.80812
C	-6.49523	-2.01149	-0.15844
H	-5.62749	-2.18249	0.51050
H	-6.24072	-2.39207	-1.17015
H	-7.35731	-2.59393	0.23074
C	-7.95261	-0.24095	-1.21599
H	-8.20383	0.84134	-1.21847
H	-8.86834	-0.80946	-0.94686
H	-7.63886	-0.53839	-2.23920
C	6.52169	-3.32724	0.57510
H	6.43643	-3.97803	1.47453
C	6.03485	-4.15332	-0.61962
H	4.96267	-4.41280	-0.48722
H	6.61345	-5.09887	-0.69081
H	6.15640	-3.59294	-1.57072
C	8.00373	-2.96405	0.42645
H	8.33515	-2.35559	1.29502
H	8.18609	-2.38873	-0.50577
H	8.61965	-3.88809	0.39524

H	-0.14984	-1.71049	-1.10489
C	1.27712	-3.66703	-1.82625
C	-0.73823	-4.11710	-0.73145
C	2.20902	-2.70640	-2.29290
C	1.54171	-5.05763	-2.00970
C	-0.54247	-5.51573	-0.86825
C	3.35586	-3.12236	-2.93154
H	2.00005	-1.65575	-2.12595
C	2.73046	-5.44982	-2.67639
C	0.58429	-5.97447	-1.50504
H	-1.29251	-6.19685	-0.48474
C	3.61630	-4.49982	-3.13247
H	4.07344	-2.38904	-3.28434
H	2.92901	-6.50839	-2.81876
H	0.75381	-7.04093	-1.62747
H	4.52688	-4.80385	-3.63910
C	-1.89752	-3.53092	0.04308
C	-1.66119	-3.59965	1.58896
H	-0.57723	-3.56239	1.73482
H	-2.01006	-4.56572	1.96660
N	-2.21130	-2.50535	2.33735
N	0.13684	-3.24003	-1.20139
C	-3.54280	-2.34242	2.70602
C	-4.43762	-3.41842	2.85569
C	-4.00891	-1.04535	2.99716
C	-5.74643	-3.19270	3.27767
H	-4.11873	-4.42969	2.62983
C	-5.31646	-0.83438	3.42096
H	-3.32782	-0.20834	2.87833
C	-6.20196	-1.90490	3.56363
H	-6.41928	-4.04031	3.37762
H	-5.64877	0.17831	3.63239
H	-7.22545	-1.73811	3.88396
H	-1.65107	-1.66207	2.25984
H	-2.01792	-2.48612	-0.23528
Cl	-3.46092	-4.33605	-0.45873

Catalyst (TRIP, 3)-Product (S) alternative conformation
Electronic energy (Hartrees): -1870.569858
Free Energy at 1M concentration and 253 K (Hartrees): -1869.348975
Coordinates:

P	0.11009	-0.00015	0.25196
O	-0.88494	1.26500	0.49475
O	1.55861	0.59591	-0.23421
O	-0.35796	-0.68676	-1.08169
O	0.12692	-0.82903	1.48062
C	-0.81241	2.26629	-0.44000
C	-2.01481	2.56854	-1.25323
C	-1.98513	3.56640	-2.14728
H	-2.87059	3.80908	-2.72239
C	-0.74775	4.34757	-2.35000
C	-0.70173	5.38938	-3.40162
H	-1.57994	5.59707	-4.00023
C	0.42530	6.07558	-3.61918
H	0.45858	6.83515	-4.38952
C	1.63128	5.79059	-2.81859
H	2.54657	6.33377	-3.01556
C	1.59879	4.85619	-1.86143
H	2.50221	4.64777	-1.30398
C	0.34398	4.10427	-1.58637

C	0.28112	3.05621	-0.52286
C	2.01519	1.65508	0.50711
C	3.27950	1.50687	1.26600
C	3.74900	2.53190	1.99127
H	4.69021	2.43892	2.52045
C	3.00348	3.80602	2.04947
C	3.49027	4.90337	2.91689
H	4.39802	4.77861	3.49415
C	2.80989	6.05226	2.99273
H	3.16541	6.85313	3.62811
C	1.56594	6.22808	2.21896
H	1.00912	7.15262	2.30131
C	1.12086	5.24546	1.42727
H	0.19494	5.38802	0.88617
C	1.88147	3.97180	1.30984
C	1.40655	2.85971	0.43261
C	4.08828	0.27761	1.10692
C	4.91833	0.11378	-0.13510
C	4.08824	-0.69547	2.05874
C	5.63341	-1.01553	-0.29698
C	4.95225	-1.88964	1.86772
C	5.67043	-2.06117	0.74904
H	6.22566	-1.15804	-1.19068
H	4.99429	-2.63866	2.64749
C	3.24058	-0.60952	3.32710
H	2.49820	0.21267	3.25428
C	2.42680	-1.89364	3.55529
H	1.94407	-2.22277	2.61187
H	1.63281	-1.70887	4.31015
H	3.06725	-2.72090	3.92687
C	4.12652	-0.30825	4.54001
H	4.86534	-1.12145	4.70486
H	3.50151	-0.20370	5.45252
H	4.67444	0.64516	4.38391
C	4.99193	1.21076	-1.19828
H	4.37541	2.08369	-0.90593
C	4.43710	0.72823	-2.54348
H	3.40018	0.35470	-2.41378
H	5.06480	-0.08119	-2.97093
H	4.41058	1.57053	-3.26739
C	6.42270	1.74346	-1.35097
H	7.10317	0.96603	-1.75776
H	6.80891	2.08020	-0.36505
H	6.43242	2.61332	-2.04213
C	-3.26756	1.81734	-1.00883
C	-4.02962	2.08474	0.08860
C	-3.69368	0.74709	-1.97551
C	-5.23136	1.25097	0.35146
C	-4.83221	0.07001	-1.73577
C	-5.61717	0.29304	-0.50282
H	-5.80748	1.42736	1.25036
H	-5.18317	-0.66962	-2.44188
C	-3.72756	3.24085	1.04588
H	-2.83614	3.81663	0.72376
C	-4.88505	4.24678	1.06707
H	-5.10234	4.59664	0.03507
H	-4.60953	5.13083	1.68111
H	-5.80517	3.79680	1.49563
C	-3.42083	2.73206	2.45859
H	-2.56549	2.02589	2.43141

H	-4.29967	2.22204	2.90456
H	-3.13972	3.58261	3.11566
C	-2.89494	0.45198	-3.24413
H	-1.89422	0.92644	-3.18085
C	-3.59864	1.04300	-4.47071
H	-2.98231	0.87859	-5.38028
H	-3.74056	2.13649	-4.34128
H	-4.59288	0.57098	-4.62305
C	-2.63896	-1.05251	-3.44183
H	-2.31557	-1.52339	-2.49267
H	-1.83906	-1.20352	-4.19785
H	-3.55069	-1.57719	-3.79744
C	-6.83882	-0.55676	-0.20627
H	-7.24532	-0.29912	0.79792
C	-6.47719	-2.04676	-0.17384
H	-5.60849	-2.21302	0.49519
H	-6.21885	-2.42194	-1.18660
H	-7.33465	-2.63764	0.21282
C	-7.94948	-0.28526	-1.22614
H	-8.20977	0.79489	-1.22539
H	-8.86043	-0.86221	-0.95875
H	-7.63322	-0.57699	-2.25021
C	6.52531	-3.30237	0.56845
H	6.44123	-3.95614	1.46582
C	6.04206	-4.12620	-0.62921
H	4.97060	-4.38940	-0.49835
H	6.62368	-5.06969	-0.70299
H	6.16247	-3.56232	-1.57839
C	8.00635	-2.93413	0.42227
H	8.33503	-2.32708	1.29286
H	8.18787	-2.35567	-0.50817
H	8.62517	-3.85617	0.38908
H	-0.13900	-1.70438	-1.12753
C	1.30848	-3.64956	-1.84226
C	-0.68820	-4.11766	-0.72153
C	2.21530	-2.68018	-2.33910
C	1.60103	-5.03795	-1.99775
C	-0.46369	-5.51464	-0.82933
C	3.36458	-3.08575	-2.98011
H	1.98485	-1.63081	-2.19396
C	2.79159	-5.41960	-2.66723
C	0.66784	-5.96355	-1.46442
H	-1.19619	-6.20291	-0.42508
C	3.65239	-4.46147	-3.15334
H	4.06233	-2.34533	-3.35709
H	3.01154	-6.47663	-2.78796
H	0.85953	-7.02862	-1.56415
H	4.56426	-4.75744	-3.66242
C	-1.85655	-3.54175	0.04671
C	-1.62580	-3.59838	1.59321
H	-0.54345	-3.53986	1.74341
H	-1.95858	-4.56856	1.97488
N	-2.19908	-2.51024	2.33334
H	-1.65361	-1.65787	2.25248
C	-3.53476	-2.36880	2.69543
C	-4.02537	-1.07843	2.97585
C	-4.41072	-3.45969	2.84903
C	-5.33798	-0.88805	3.39375
H	-3.35923	-0.23009	2.85298
C	-5.72492	-3.25464	3.26468

H	-4.07269	-4.46662	2.63173
C	-6.20457	-1.97338	3.54059
H	-5.68911	0.12004	3.59672
H	-6.38277	-4.11354	3.36809
H	-7.23208	-1.82304	3.85614
H	-1.99026	-2.50064	-0.23926
N	0.16480	-3.23273	-1.21647
Cl	-3.40724	-4.37159	-0.45613

Catalyst (TRIP, 3)-Product (S) alternative conformation
Electronic energy (Hartrees): -1870.571207
Free Energy at 1M concentration and 253 K (Hartrees): -1869.34869
Coordinates:

P	-0.18410	-1.60044	0.23345
O	0.81949	-0.28846	0.33352
O	-1.56595	-1.24976	1.01960
O	0.37923	-2.86146	0.74962
O	-0.55538	-1.52957	-1.30248
C	0.94674	0.21281	1.60257
C	2.27600	0.19571	2.26582
C	2.40438	0.67281	3.51260
H	3.37438	0.67714	3.99662
C	1.23216	1.20271	4.23602
C	1.37513	1.64855	5.64114
H	2.34140	1.60981	6.12857
C	0.31110	2.09837	6.31490
H	0.41587	2.42239	7.34228
C	-1.01217	2.14236	5.66394
H	-1.87363	2.48560	6.22215
C	-1.15038	1.74788	4.39292
H	-2.13449	1.76573	3.94371
C	0.03011	1.27412	3.61985
C	-0.09663	0.83071	2.19907
C	-2.03070	0.03789	0.89719
C	-3.40242	0.25300	0.36544
C	-3.90552	1.49422	0.29320
H	-4.91649	1.65213	-0.06383
C	-3.10060	2.65348	0.72154
C	-3.64369	4.02224	0.56383
H	-4.62514	4.17155	0.13149
C	-2.92382	5.07980	0.95221
H	-3.31918	6.08003	0.83213
C	-1.57985	4.88779	1.52788
H	-0.99147	5.74953	1.81530
C	-1.07705	3.65585	1.66754
H	-0.07446	3.54309	2.05754
C	-1.87701	2.46590	1.26658
C	-1.34634	1.07772	1.42956
C	-4.26326	-0.90959	0.03729
C	-4.81314	-1.74693	1.16020
C	-4.58514	-1.21119	-1.25273
C	-5.58067	-2.81281	0.86431
C	-5.46479	-2.37847	-1.52388
C	-5.92692	-3.15202	-0.53226
H	-5.98416	-3.42670	1.65807
H	-5.73365	-2.60811	-2.54668
C	-4.08648	-0.39750	-2.44608
H	-3.37337	0.38494	-2.12645
C	-3.32990	-1.27762	-3.45043
H	-2.54740	-1.86849	-2.93211

H	-2.83629	-0.64040	-4.21407
H	-4.01334	-1.97710	-3.97526
C	-5.24893	0.33050	-3.12968
H	-5.97532	-0.38981	-3.56197
H	-4.86398	0.97846	-3.94609
H	-5.77808	0.97562	-2.39649
C	-4.56399	-1.39068	2.62663
H	-3.93399	-0.48395	2.71023
C	-3.80950	-2.51000	3.35687
H	-2.87634	-2.76332	2.81125
H	-4.43130	-3.42564	3.44478
H	-3.53202	-2.17541	4.37931
C	-5.87559	-1.05061	3.34666
H	-6.54096	-1.93676	3.41707
H	-6.40878	-0.24269	2.80112
H	-5.66162	-0.69077	4.37585
C	3.48199	-0.27657	1.54520
C	4.19207	0.57275	0.75147
C	4.02278	-1.64514	1.84522
C	5.52508	0.14524	0.24830
C	5.24768	-1.97810	1.39594
C	6.04811	-1.04420	0.57549
H	6.10798	0.83394	-0.34898
H	5.66879	-2.94492	1.63488
C	3.72181	1.99066	0.42480
H	2.70263	2.18372	0.81727
C	4.63899	3.03157	1.07586
H	4.69018	2.85778	2.17214
H	4.23573	4.05287	0.90581
H	5.66558	2.98360	0.65540
C	3.63563	2.20686	-1.09031
H	2.93014	1.47231	-1.52631
H	4.62368	2.09288	-1.58155
H	3.26097	3.22817	-1.31190
C	3.22663	-2.64662	2.67681
H	2.19236	-2.27517	2.82977
C	3.85060	-2.81612	4.06646
H	3.22599	-3.49616	4.68417
H	3.90636	-1.83353	4.58132
H	4.87465	-3.24069	3.99457
C	3.09307	-4.00428	1.96883
H	2.77671	-3.86031	0.91519
H	2.32816	-4.62467	2.48281
H	4.05219	-4.56347	1.97569
C	7.45462	-1.41291	0.13684
H	7.91007	-0.56922	-0.42939
C	7.43931	-2.62172	-0.80293
H	6.84511	-2.38089	-1.70730
H	7.00272	-3.51457	-0.30743
H	8.47293	-2.86630	-1.12830
C	8.35654	-1.67098	1.34972
H	8.34222	-0.79157	2.02870
H	9.40377	-1.83405	1.01664
H	8.02429	-2.56728	1.91501
C	-6.81057	-4.34806	-0.83758
H	-6.95670	-4.44200	-1.93741
C	-6.15342	-5.64898	-0.36234
H	-5.14624	-5.75517	-0.81934
H	-6.76849	-6.52065	-0.67231
H	-6.04961	-5.66657	0.74329

C	-8.20109	-4.17505	-0.21560
H	-8.65883	-3.22522	-0.56605
H	-8.14697	-4.15970	0.89354
H	-8.86209	-5.01295	-0.52418
H	0.20577	-1.23445	-1.91257
C	2.49718	-1.69206	-2.89828
C	1.57815	0.32642	-3.61717
C	2.39155	-2.86865	-2.11017
C	3.68094	-1.48008	-3.67346
C	2.72746	0.60963	-4.40235
C	3.41513	-3.79020	-2.11031
H	1.51455	-3.03930	-1.49791
C	4.71011	-2.45464	-3.65570
C	3.76445	-0.28648	-4.43048
H	2.76469	1.53122	-4.97285
C	4.57700	-3.59146	-2.89188
H	3.32609	-4.68282	-1.49953
H	5.60200	-2.28556	-4.25263
H	4.65345	-0.09109	-5.02408
H	5.36748	-4.33545	-2.87890
N	1.46895	-0.78089	-2.89736
C	0.47014	1.35927	-3.59864
H	0.91429	2.34903	-3.72103
C	-0.45346	1.36362	-2.37360
H	0.16948	1.33224	-1.47597
H	-1.04145	0.44937	-2.35895
N	-1.35811	2.49220	-2.30817
H	-2.09972	2.44909	-2.99475
C	-0.91658	3.79032	-2.04236
C	-1.71150	4.88545	-2.43110
C	0.27369	4.04882	-1.33958
C	-1.32846	6.18606	-2.12290
H	-2.63837	4.70372	-2.96995
C	0.65000	5.35786	-1.04075
H	0.88682	3.22623	-0.99244
C	-0.14192	6.43771	-1.42840
H	-1.96350	7.01170	-2.43156
H	1.57168	5.52688	-0.49053
H	0.15471	7.45435	-1.19223
Cl	-0.55242	1.15455	-5.12610

Catalyst (TRIP, 3)-Product (S) alternative conformation
Electronic energy (Hartrees): -1870.569574
Free Energy at 1M concentration and 253 K (Hartrees): -1869.348217
Coordinates:

P	0.22566	-0.06844	-0.10270
O	-0.96033	0.98261	0.27352
O	1.47711	0.80941	-0.68216
O	-0.22498	-0.80752	-1.42168
O	0.53176	-0.91835	1.06885
C	-1.17929	1.97915	-0.64510
C	-2.50151	2.05379	-1.31551
C	-2.75530	3.04349	-2.18357
H	-3.72883	3.12007	-2.65285
C	-1.71287	4.03784	-2.50796
C	-1.97633	5.06776	-3.53921
H	-2.93938	5.10957	-4.03272
C	-1.02513	5.94844	-3.86789
H	-1.21777	6.69954	-4.62303
C	0.29492	5.89092	-3.21157

H	1.06774	6.59231	-3.49854
C	0.54111	4.96824	-2.27435
H	1.52377	4.92979	-1.82351
C	-0.51692	4.00056	-1.87508
C	-0.26971	2.96449	-0.82718
C	1.76892	1.92364	0.06116
C	3.05766	1.98517	0.79329
C	3.40242	3.10577	1.44388
H	4.35698	3.16861	1.95203
C	2.47217	4.25118	1.50294
C	2.82483	5.44071	2.31189
H	3.77956	5.48890	2.82077
C	1.96430	6.45834	2.42145
H	2.22187	7.32611	3.01483
C	0.65413	6.39479	1.74656
H	-0.04794	7.21072	1.86025
C	0.32809	5.32749	1.00842
H	-0.64887	5.29217	0.54556
C	1.28807	4.20086	0.84863
C	0.95418	3.00148	0.02148
C	3.97681	0.82262	0.77022
C	4.76096	0.53634	-0.48000
C	4.11894	0.01663	1.86133
C	5.57387	-0.53662	-0.50876
C	5.07738	-1.11742	1.79918
C	5.75192	-1.40235	0.67695
H	6.14119	-0.76256	-1.40139
H	5.22436	-1.73087	2.67824
C	3.34085	0.23318	3.16117
H	2.51557	0.96052	3.01351
C	2.67436	-1.06090	3.65717
H	2.17651	-1.59254	2.82238
H	1.90818	-0.82081	4.42535
H	3.41401	-1.75031	4.11570
C	4.26158	0.80602	4.24418
H	5.08222	0.09640	4.48367
H	3.68196	1.00659	5.17054
H	4.70898	1.76249	3.90272
C	4.68929	1.45560	-1.69942
H	3.98981	2.29514	-1.51680
C	4.15954	0.71427	-2.93370
H	3.17863	0.24878	-2.70637
H	4.86503	-0.07637	-3.26446
H	4.01570	1.42823	-3.77254
C	6.05113	2.10062	-1.98805
H	6.80205	1.34141	-2.29264
H	6.41995	2.62849	-1.08255
H	5.95320	2.84492	-2.80710
C	-3.56604	1.08252	-0.96617
C	-4.23495	1.18232	0.21732
C	-3.94380	0.01012	-1.95141
C	-5.31330	0.20569	0.52205
C	-4.96640	-0.81515	-1.65651
C	-5.68164	-0.72877	-0.36478
H	-5.83118	0.27320	1.47003
H	-5.28009	-1.56575	-2.36864
C	-3.96937	2.29756	1.23131
H	-3.17003	2.98420	0.88799
C	-5.21413	3.17287	1.42151
H	-5.55092	3.57073	0.44023

H	-4.97538	4.03463	2.08094
H	-6.04640	2.60064	1.88244
C	-3.49418	1.72332	2.57003
H	-2.59996	1.08621	2.41190
H	-4.28787	1.11728	3.05361
H	-3.21273	2.54685	3.26037
C	-3.22663	-0.13109	-3.29368
H	-2.31725	0.50369	-3.30900
C	-4.12674	0.34427	-4.44017
H	-3.57122	0.30717	-5.40154
H	-4.44785	1.39266	-4.26635
H	-5.03121	-0.29494	-4.52717
C	-2.74189	-1.56749	-3.54933
H	-2.20324	-1.95912	-2.66507
H	-2.04519	-1.58247	-4.41456
H	-3.58785	-2.24945	-3.77691
C	-6.83217	-1.67025	-0.05573
H	-7.24324	-1.44952	0.95531
C	-6.36188	-3.12812	-0.03619
H	-5.55346	-3.25255	0.71278
H	-5.98414	-3.44403	-1.03154
H	-7.20216	-3.79567	0.25073
C	-7.97860	-1.47712	-1.05501
H	-8.30225	-0.41422	-1.06177
H	-8.84861	-2.10222	-0.76081
H	-7.66955	-1.76507	-2.08215
C	6.69782	-2.58842	0.62964
H	6.70543	-3.10869	1.61404
C	6.23407	-3.61220	-0.41167
H	5.19050	-3.92617	-0.19493
H	6.88194	-4.51378	-0.37521
H	6.27733	-3.18815	-1.43716
C	8.13579	-2.13443	0.35346
H	8.45044	-1.38594	1.11191
H	8.22878	-1.68295	-0.65688
H	8.82634	-3.00244	0.41558
H	-0.04738	-1.82261	-1.37508
C	1.52601	-3.75945	-1.78276
C	-0.37807	-4.20261	-0.51034
C	2.27054	-2.83831	-2.56217
C	2.03804	-5.07375	-1.56198
C	0.06474	-5.52305	-0.23845
C	3.47518	-3.22081	-3.10817
H	1.87128	-1.84105	-2.70842
C	3.27942	-5.43609	-2.14444
C	1.26083	-5.94865	-0.76148
H	-0.55303	-6.17865	0.36341
C	3.98048	-4.52829	-2.90575
H	4.04673	-2.51409	-3.70134
H	3.66624	-6.43752	-1.97791
H	1.62159	-6.95542	-0.56856
H	4.93206	-4.80866	-3.34650
C	-1.65173	-3.64711	0.08929
C	-1.52100	-3.35181	1.58528
H	-0.67453	-2.66529	1.66654
H	-1.26666	-4.27803	2.12798
N	-2.71560	-2.70303	2.08345
N	0.32654	-3.36575	-1.25458
C	-2.71739	-1.94431	3.26204
C	-1.57947	-1.26664	3.73606

C	-3.92462	-1.81163	3.97330
C	-1.66380	-0.48929	4.89034
H	-0.64630	-1.30145	3.18634
C	-3.99317	-1.02919	5.12176
H	-4.81372	-2.32349	3.61060
C	-2.86111	-0.36311	5.59579
H	-0.77387	0.03118	5.23434
H	-4.93923	-0.94279	5.64944
H	-2.91376	0.24573	6.49296
H	-3.53394	-3.29216	2.00094
H	-1.93795	-2.73129	-0.42354
Cl	-3.04460	-4.81287	-0.22608

Catalyst (TRIP, 3)-Product (S) alternative conformation
Electronic energy (Hartrees): -1870.570281
Free Energy at 1M concentration and 253 K (Hartrees): -1869.347498
Coordinates:

P	0.19337	-0.19006	-0.13195
O	-0.86570	0.93018	0.40318
O	1.56750	0.57898	-0.56755
O	-0.30814	-0.64502	-1.55786
O	0.31269	-1.23440	0.91293
C	-0.86132	2.13919	-0.24404
C	-2.11596	2.60471	-0.88726
C	-2.16285	3.81472	-1.46256
H	-3.08190	4.16658	-1.91615
C	-0.96286	4.67378	-1.49489
C	-1.00565	5.96746	-2.21479
H	-1.92257	6.29679	-2.68761
C	0.08856	6.73277	-2.28742
H	0.05701	7.67697	-2.81561
C	1.34647	6.28614	-1.65935
H	2.23457	6.89940	-1.74187
C	1.39412	5.12204	-1.00144
H	2.33419	4.80387	-0.57085
C	0.17691	4.27255	-0.88550
C	0.20199	2.97054	-0.15232
C	2.04215	1.43798	0.38944
C	3.36481	1.17407	1.00840
C	3.83007	1.99511	1.96123
H	4.80904	1.82641	2.39505
C	3.03097	3.15576	2.40321
C	3.52081	4.00663	3.51209
H	4.46773	3.78657	3.98926
C	2.79485	5.04909	3.93035
H	3.15262	5.66948	4.74190
C	1.49825	5.35073	3.29403
H	0.90613	6.18345	3.65120
C	1.04981	4.59205	2.28718
H	0.08633	4.81812	1.85001
C	1.85895	3.44560	1.79174
C	1.38042	2.58234	0.67087
C	4.24023	0.10240	0.47823
C	5.08102	0.38945	-0.73305
C	4.37255	-1.08553	1.12907
C	6.01172	-0.50771	-1.10997
C	5.42059	-2.04161	0.68030
C	6.21699	-1.76769	-0.36347
H	6.63624	-0.30996	-1.97020
H	5.57190	-2.95590	1.23904

C	3.52250	-1.45731	2.34208
H	2.70285	-0.72413	2.49405
C	2.84009	-2.81910	2.15053
H	2.32370	-2.85254	1.17010
H	2.08351	-2.97991	2.94797
H	3.57145	-3.65299	2.19304
C	4.37058	-1.44850	3.61804
H	5.17434	-2.21357	3.56970
H	3.73141	-1.66061	4.50160
H	4.83546	-0.44947	3.75842
C	4.90488	1.67374	-1.54236
H	4.11858	2.31447	-1.09821
C	4.44313	1.36674	-2.97246
H	3.51573	0.75477	-2.94776
H	5.22283	0.81484	-3.53860
H	4.21997	2.31244	-3.51114
C	6.18504	2.51919	-1.54059
H	7.00945	2.00865	-2.08123
H	6.50681	2.71668	-0.49557
H	5.99502	3.49587	-2.03496
C	-3.33115	1.75460	-0.85797
C	-4.07332	1.63638	0.27901
C	-3.78561	1.07699	-2.12104
C	-5.32564	0.83481	0.24417
C	-4.95676	0.41251	-2.12064
C	-5.76816	0.27759	-0.89170
H	-5.90983	0.73232	1.14946
H	-5.31950	-0.04639	-3.02994
C	-3.71279	2.34807	1.58438
H	-2.78612	2.94755	1.48297
C	-4.80833	3.34191	1.98930
H	-4.99978	4.05412	1.15821
H	-4.48239	3.92535	2.87689
H	-5.75559	2.82150	2.24291
C	-3.44755	1.33740	2.70515
H	-2.64299	0.63781	2.39803
H	-4.35949	0.75337	2.94893
H	-3.11248	1.86474	3.62360
C	-2.96380	1.15114	-3.40693
H	-1.97299	1.60592	-3.20302
C	-3.65868	2.03813	-4.44666
H	-3.01582	2.14565	-5.34631
H	-3.83876	3.05023	-4.02705
H	-4.63235	1.60211	-4.75624
C	-2.67447	-0.24124	-3.98839
H	-2.27511	-0.91131	-3.20215
H	-1.91641	-0.16339	-4.79677
H	-3.58900	-0.70324	-4.41571
C	-7.09563	-0.45986	-0.92152
H	-7.55539	-0.45626	0.09260
C	-6.90917	-1.92800	-1.32051
H	-6.23677	-2.43389	-0.59924
H	-6.47981	-2.01976	-2.34033
H	-7.88699	-2.45479	-1.30113
C	-8.08345	0.24046	-1.86168
H	-8.20398	1.30396	-1.56331
H	-9.07829	-0.25049	-1.80266
H	-7.73257	0.20036	-2.91472
C	7.35190	-2.70730	-0.73464
H	7.31622	-3.61660	-0.09309

C	7.24365	-3.18345	-2.18880
H	6.26573	-3.67477	-2.36235
H	8.04081	-3.92689	-2.40325
H	7.35363	-2.33962	-2.90156
C	8.70612	-2.03422	-0.48459
H	8.77815	-1.70638	0.57465
H	8.84024	-1.14982	-1.14346
H	9.53112	-2.75164	-0.68187
H	-0.42729	-1.66825	-1.61086
C	0.88692	-3.72167	-1.35074
C	-1.38303	-3.95178	-0.85986
C	1.92897	-2.96080	-1.93994
C	1.20556	-4.93494	-0.67107
C	-1.14764	-5.16232	-0.16386
C	3.23330	-3.38729	-1.83949
H	1.68303	-2.03495	-2.44728
C	2.55959	-5.35126	-0.59535
C	0.13546	-5.64354	-0.07400
H	-1.97808	-5.68189	0.29394
C	3.55471	-4.58923	-1.16266
H	4.02174	-2.78630	-2.28027
H	2.79633	-6.27247	-0.07045
H	0.34145	-6.56310	0.46717
H	4.58942	-4.90616	-1.08374
C	-2.75949	-3.30488	-0.95254
C	-2.93846	-2.14362	0.05244
H	-3.99246	-1.86947	0.08097
H	-2.41314	-1.28318	-0.37784
N	-2.44629	-2.42810	1.38228
N	-0.40232	-3.26387	-1.42788
C	-3.22837	-2.25537	2.52639
C	-4.62568	-2.41634	2.53017
C	-2.59034	-1.93385	3.74097
C	-5.35347	-2.22890	3.70460
H	-5.14390	-2.71185	1.62498
C	-3.32737	-1.76160	4.90741
H	-1.51169	-1.79912	3.74880
C	-4.71803	-1.89870	4.90145
H	-6.43287	-2.35319	3.67896
H	-2.80941	-1.50505	5.82776
H	-5.29195	-1.75571	5.81159
H	-1.48015	-2.13123	1.50041
H	-2.89377	-2.91656	-1.96342
Cl	-4.09365	-4.53964	-0.76591

Catalyst (TRIP, 3)-Product (R) alternative conformation
Electronic energy (Hartrees): -1870.566459
Free Energy at 1M concentration and 253 K (Hartrees): -1869.346731
Coordinates:

P	-0.43918	0.22044	0.04555
O	0.39216	-1.16619	0.26020
O	-1.86800	-0.16250	-0.63661
O	0.22169	0.99083	-1.15773
O	-0.49880	0.92230	1.35080
C	0.30042	-2.09960	-0.74286
C	1.54884	-2.54825	-1.41065
C	1.50114	-3.50698	-2.34657
H	2.41310	-3.85437	-2.81745
C	0.20685	-4.09132	-2.74956
C	0.15822	-5.07708	-3.85386

H	1.07158	-5.38980	-4.34450
C	-1.01394	-5.58293	-4.25198
H	-1.04885	-6.30193	-5.06026
C	-2.26518	-5.15257	-3.59950
H	-3.21252	-5.54860	-3.94191
C	-2.23154	-4.26694	-2.59707
H	-3.16343	-3.94760	-2.14978
C	-0.93231	-3.71790	-2.12143
C	-0.86706	-2.73444	-0.99784
C	-2.51902	-1.20056	-0.02181
C	-3.81650	-0.94138	0.65033
C	-4.49513	-1.95598	1.20545
H	-5.45777	-1.77797	1.66986
C	-3.93330	-3.32133	1.20063
C	-4.64731	-4.41126	1.90483
H	-5.59483	-4.21549	2.39130
C	-4.12331	-5.64086	1.94654
H	-4.64454	-6.43626	2.46339
C	-2.82551	-5.91402	1.30043
H	-2.39517	-6.90544	1.35914
C	-2.17559	-4.93795	0.65610
H	-1.21318	-5.15554	0.21271
C	-2.76077	-3.57174	0.57260
C	-2.05902	-2.46520	-0.14631
C	-4.39673	0.42229	0.64423
C	-5.02309	0.94373	-0.61909
C	-4.39358	1.19256	1.76868
C	-5.57465	2.17206	-0.61494
C	-5.05941	2.52108	1.73492
C	-5.60754	3.00015	0.60977
H	-6.02581	2.56619	-1.51540
H	-5.09808	3.11327	2.63978
C	-3.73837	0.74684	3.07647
H	-3.12296	-0.16416	2.92351
C	-2.77794	1.81471	3.62436
H	-2.12591	2.20556	2.81691
H	-2.13189	1.37308	4.41315
H	-3.32993	2.66822	4.07090
C	-4.80628	0.39916	4.11913
H	-5.42438	1.28810	4.36790
H	-4.32459	0.02892	5.04927
H	-5.47278	-0.40026	3.73293
C	-5.06538	0.10828	-1.89861
H	-4.59032	-0.87935	-1.73894
C	-4.28317	0.78395	-3.03300
H	-3.24627	1.00658	-2.70298
H	-4.76853	1.73098	-3.34990
H	-4.22849	0.10666	-3.91193
C	-6.50952	-0.18788	-2.32396
H	-7.04339	0.73962	-2.61990
H	-7.05993	-0.66869	-1.48703
H	-6.51508	-0.88570	-3.18850
C	2.85341	-1.98677	-0.98660
C	3.40398	-2.33677	0.20985
C	3.58230	-1.03649	-1.89516
C	4.67514	-1.69324	0.63500
C	4.76815	-0.53665	-1.50016
C	5.32642	-0.83769	-0.16525
H	5.08792	-1.93358	1.60623
H	5.32712	0.11574	-2.15668

C	2.80012	-3.41151	1.11671
H	1.86536	-3.82958	0.69180
C	3.75880	-4.59944	1.26175
H	4.01619	-5.00303	0.25901
H	3.27456	-5.40996	1.84747
H	4.69474	-4.30297	1.78064
C	2.43037	-2.84027	2.48879
H	1.77203	-1.95488	2.37095
H	3.33668	-2.54474	3.05499
H	1.88169	-3.60176	3.08297
C	3.02466	-0.64819	-3.26293
H	1.98598	-1.01858	-3.37557
C	3.85037	-1.28086	-4.38889
H	3.39176	-1.04886	-5.37382
H	3.87583	-2.38464	-4.26936
H	4.89290	-0.89736	-4.38350
C	2.94628	0.87471	-3.43441
H	2.48069	1.32852	-2.53794
H	2.32693	1.12566	-4.32183
H	3.95159	1.32421	-3.57548
C	6.62719	-0.19827	0.28511
H	6.82877	-0.45976	1.34865
C	6.55335	1.33292	0.20618
H	5.64859	1.70514	0.72897
H	6.51975	1.68197	-0.84739
H	7.44588	1.77989	0.69354
C	7.80213	-0.73003	-0.54209
H	7.85640	-1.83656	-0.45705
H	8.75681	-0.30566	-0.16436
H	7.69186	-0.45815	-1.61359
C	-6.26203	4.36976	0.59561
H	-6.19038	4.83588	1.60427
C	-5.54444	5.30828	-0.38138
H	-4.46511	5.37104	-0.12499
H	-5.97670	6.32947	-0.31409
H	-5.64442	4.95186	-1.42848
C	-7.75368	4.26107	0.25890
H	-8.25537	3.57297	0.97258
H	-7.90664	3.88153	-0.77355
H	-8.23550	5.25862	0.34224
H	0.55527	1.94086	-0.95891
C	1.46827	3.94701	-2.19646
C	1.97161	3.86506	0.08052
C	0.68161	3.47916	-3.28026
C	2.43576	4.97252	-2.42177
C	2.95635	4.87578	-0.06331
C	0.86113	4.01062	-4.53785
H	-0.04482	2.69527	-3.09598
C	2.59551	5.49634	-3.73021
C	3.19019	5.41213	-1.30517
H	3.49498	5.21872	0.81150
C	1.82351	5.02387	-4.76729
H	0.25877	3.64891	-5.36547
H	3.33343	6.27610	-3.89714
H	3.94352	6.18342	-1.44170
H	1.94886	5.42779	-5.76706
C	1.66079	3.21751	1.41538
H	0.64306	2.82903	1.38398
C	2.61692	2.06550	1.77124
H	3.59530	2.48177	2.02706

H	2.74933	1.45670	0.85743
N	2.09497	1.31700	2.88393
N	1.27788	3.40580	-0.95174
C	2.87074	0.51509	3.71089
C	2.22872	-0.21260	4.73593
C	4.26898	0.40669	3.60024
C	2.95873	-1.00938	5.60829
H	1.14911	-0.13677	4.83583
C	4.99113	-0.39016	4.48944
H	4.79986	0.92816	2.81374
C	4.35022	-1.10549	5.49866
H	2.43529	-1.55982	6.38553
H	6.06998	-0.45722	4.37528
H	4.91766	-1.72821	6.18289
H	1.13405	1.01715	2.74854
Cl	1.64787	4.47147	2.74385

Catalyst (TRIP, 3)-Product (S) alternative conformation
Electronic energy (Hartrees): -1870.566876
Free Energy at 1M concentration and 253 K (Hartrees): -1869.346581
Coordinates:

P	0.45962	-0.90790	-1.28255
O	1.36491	-0.31658	-0.03435
O	-0.67828	-1.89822	-0.65945
O	1.22470	-1.59265	-2.33972
O	-0.34085	0.40452	-1.66447
C	1.65961	-1.22540	0.95040
C	3.07976	-1.56726	1.21919
C	3.38684	-2.43297	2.19566
H	4.42317	-2.67070	2.40630
C	2.32077	-3.06605	2.99602
C	2.67323	-4.07863	4.01788
H	3.71055	-4.33350	4.19616
C	1.71211	-4.68501	4.72268
H	1.97033	-5.42635	5.46781
C	0.29538	-4.35478	4.47776
H	-0.47873	-4.86581	5.03562
C	-0.03322	-3.43694	3.56136
H	-1.07978	-3.22973	3.38249
C	1.02624	-2.73125	2.78913
C	0.68646	-1.70199	1.76037
C	-1.34444	-1.37894	0.42393
C	-2.80399	-1.12358	0.32306
C	-3.46511	-0.60594	1.36902
H	-4.53584	-0.44841	1.31318
C	-2.74749	-0.27641	2.61668
C	-3.47660	0.35845	3.73802
H	-4.53762	0.55549	3.65414
C	-2.82904	0.68717	4.86070
H	-3.36252	1.15431	5.67834
C	-1.38475	0.41471	4.98840
H	-0.86295	0.69912	5.89297
C	-0.72020	-0.17546	3.98826
H	0.34243	-0.34808	4.09561
C	-1.43046	-0.56216	2.73897
C	-0.70988	-1.21383	1.60514
C	-3.55142	-1.57448	-0.87411
C	-3.80064	-3.04635	-1.05939
C	-4.02696	-0.68523	-1.79049
C	-4.46503	-3.46323	-2.15356

C	-4.79110	-1.19406	-2.96015
C	-4.98998	-2.50553	-3.14990
H	-4.64835	-4.51804	-2.30679
H	-5.19012	-0.48700	-3.67566
C	-3.81579	0.82378	-1.67308
H	-3.12459	1.06952	-0.84172
C	-3.17428	1.40334	-2.94291
H	-2.31095	0.78353	-3.26158
H	-2.81053	2.43440	-2.74532
H	-3.90132	1.44889	-3.78067
C	-5.14397	1.52701	-1.37216
H	-5.89742	1.31778	-2.16060
H	-4.99172	2.62453	-1.32280
H	-5.54362	1.18423	-0.39428
C	-3.34834	-4.07525	-0.02199
H	-2.84688	-3.57980	0.83207
C	-2.32537	-5.05346	-0.61401
H	-1.47272	-4.49448	-1.05419
H	-2.78285	-5.68766	-1.40229
H	-1.92857	-5.71662	0.18419
C	-4.54444	-4.82480	0.57991
H	-5.05190	-5.45600	-0.17959
H	-5.27741	-4.09908	0.99307
H	-4.20284	-5.48162	1.40839
C	4.16694	-0.90967	0.45882
C	4.62666	0.31825	0.82641
C	4.83953	-1.65792	-0.65651
C	5.78050	0.90550	0.09514
C	5.92518	-1.11893	-1.24269
C	6.41542	0.22555	-0.86982
H	6.13429	1.88959	0.37390
H	6.45189	-1.66387	-2.01383
C	4.04380	1.10277	2.00283
H	3.19998	0.56281	2.47866
C	5.09290	1.30228	3.10269
H	5.49855	0.31821	3.42156
H	4.62835	1.78849	3.98712
H	5.93100	1.94051	2.75205
C	3.47540	2.44477	1.53617
H	2.72390	2.26389	0.74397
H	4.26844	3.11011	1.13555
H	2.97322	2.96325	2.38049
C	4.34293	-3.03062	-1.10413
H	3.34490	-3.23494	-0.66406
C	5.28674	-4.13317	-0.61168
H	4.88604	-5.13101	-0.89082
H	5.37646	-4.09289	0.49447
H	6.29845	-4.01801	-1.05611
C	4.15879	-3.11273	-2.62847
H	3.62665	-2.21495	-3.00497
H	3.55987	-4.01159	-2.88855
H	5.13508	-3.18683	-3.15228
C	7.62872	0.82165	-1.56085
H	7.82993	1.84176	-1.16249
C	7.38862	0.97034	-3.06730
H	6.47662	1.57499	-3.24719
H	7.26535	-0.01895	-3.55635
H	8.24811	1.49218	-3.53952
C	8.87952	-0.02079	-1.28627
H	9.03948	-0.11804	-0.19105

H	9.77355	0.47180	-1.72476
H	8.78455	-1.03603	-1.72661
C	-5.75826	-2.99972	-4.36235
H	-6.07046	-2.13646	-4.99252
C	-4.87742	-3.89676	-5.23962
H	-3.95354	-3.35323	-5.53214
H	-5.42332	-4.17617	-6.16597
H	-4.59228	-4.82735	-4.70468
C	-7.03824	-3.72995	-3.93963
H	-7.66039	-3.06751	-3.30039
H	-6.80480	-4.65750	-3.37523
H	-7.63305	-4.00416	-4.83697
H	0.19004	1.27093	-1.55286
C	2.03950	2.99940	-2.00324
C	0.47616	3.48805	-0.34281
C	2.53266	2.07978	-2.96411
C	2.73329	4.22711	-1.77231
C	1.10007	4.73040	-0.06025
C	3.67427	2.38402	-3.67084
H	2.01580	1.13752	-3.11533
C	3.90255	4.50847	-2.52351
C	2.21400	5.09326	-0.77744
H	0.71264	5.35976	0.73236
C	4.36062	3.60453	-3.45493
H	4.05788	1.67599	-4.39856
H	4.43026	5.44165	-2.34773
H	2.71724	6.03522	-0.57702
H	5.25840	3.82125	-4.02547
N	0.92097	2.67617	-1.28715
C	-0.72001	2.99925	0.44334
H	-0.98273	1.99529	0.11510
C	-1.93839	3.91250	0.35667
H	-2.18212	3.99067	-0.71084
H	-1.67850	4.92697	0.70356
N	-3.06514	3.34107	1.05876
H	-2.81140	2.93332	1.95016
C	-4.30339	3.99070	1.07391
C	-5.29462	3.54173	1.96697
C	-4.61751	5.04540	0.19999
C	-6.55661	4.12237	1.97735
H	-5.06565	2.72471	2.64498
C	-5.89202	5.61496	0.21515
H	-3.87789	5.42114	-0.49816
C	-6.86998	5.16380	1.09865
H	-7.30331	3.75562	2.67606
H	-6.11378	6.42598	-0.47300
H	-7.85766	5.61333	1.10594
Cl	-0.21762	2.79544	2.21052

Catalyst (TRIP, 3)-Product (R) alternative conformation
Electronic energy (Hartrees): -1870.567291
Free Energy at 1M concentration and 253 K (Hartrees): -1869.346522
Coordinates:

P	0.36853	-0.39453	-1.30733
O	1.72822	0.46897	-1.51563
O	-0.68082	0.54030	-0.41753
O	0.72788	-1.49172	-0.23730
O	-0.15286	-0.83097	-2.61664
C	2.06060	1.38229	-0.54645
C	3.37404	1.24365	0.13201

C	3.79459	2.18944	0.98257
H	4.76075	2.09736	1.46468
C	2.93492	3.34449	1.29889
C	3.37394	4.32483	2.31872
H	4.34682	4.22703	2.78413
C	2.56664	5.32809	2.67774
H	2.88553	6.04040	3.42761
C	1.23093	5.45644	2.06612
H	0.57022	6.25302	2.38306
C	0.83034	4.58393	1.13449
H	-0.16587	4.68282	0.72586
C	1.73057	3.48024	0.69742
C	1.30656	2.48843	-0.34005
C	-0.93855	1.75979	-0.98897
C	-2.33076	2.11279	-1.35841
C	-2.57547	3.25635	-2.01525
H	-3.59341	3.53658	-2.26036
C	-1.46885	4.18342	-2.33258
C	-1.73701	5.41376	-3.11177
H	-2.73966	5.62982	-3.45919
C	-0.74159	6.26312	-3.38974
H	-0.93790	7.16158	-3.96042
C	0.62945	5.97515	-2.92508
H	1.43285	6.65882	-3.16729
C	0.88108	4.86961	-2.21443
H	1.89552	4.66723	-1.89632
C	-0.21934	3.92767	-1.87857
C	0.03727	2.68585	-1.09186
C	-3.45754	1.32510	-0.80854
C	-4.03575	1.72605	0.52155
C	-3.99996	0.28651	-1.49930
C	-5.08295	1.03629	1.01139
C	-5.14577	-0.45364	-0.90804
C	-5.65418	-0.11856	0.28550
H	-5.53721	1.33064	1.94692
H	-5.57591	-1.28222	-1.45505
C	-3.49759	-0.14278	-2.87509
H	-2.57200	0.40224	-3.15280
C	-3.14132	-1.63457	-2.89281
H	-2.51682	-1.87702	-2.00968
H	-2.56695	-1.87976	-3.81159
H	-4.04736	-2.27539	-2.87118
C	-4.53711	0.18248	-3.95214
H	-5.48038	-0.37868	-3.78113
H	-4.14212	-0.08455	-4.95563
H	-4.76110	1.27081	-3.94495
C	-3.51672	2.95070	1.27813
H	-2.53724	3.26845	0.86735
C	-3.26860	2.66958	2.76818
H	-2.71035	1.72321	2.89418
H	-4.22065	2.59736	3.33466
H	-2.66559	3.49057	3.21200
C	-4.47741	4.13166	1.10492
H	-5.47142	3.90130	1.54467
H	-4.60871	4.36131	0.02617
H	-4.06616	5.03514	1.60387
C	4.23170	0.06648	-0.13671
C	4.97734	-0.00819	-1.27407
C	4.33078	-1.02036	0.89808
C	5.88169	-1.17194	-1.46818

C	5.20535	-2.02553	0.70543
C	6.00702	-2.12411	-0.53331
H	6.46338	-1.23970	-2.37833
H	5.32039	-2.79571	1.45548
C	4.96108	1.07816	-2.35066
H	4.25918	1.89742	-2.09568
C	6.34026	1.73446	-2.48585
H	6.66815	2.13254	-1.50163
H	6.28932	2.58187	-3.20259
H	7.09910	1.01140	-2.85197
C	4.48694	0.51348	-3.69566
H	3.49982	0.01864	-3.57584
H	5.20958	-0.22417	-4.10305
H	4.37392	1.33624	-4.43363
C	3.48020	-0.99487	2.16784
H	2.69488	-0.21654	2.08408
C	4.33829	-0.64147	3.38769
H	3.70442	-0.58429	4.29761
H	4.82328	0.34627	3.24117
H	5.12721	-1.40572	3.55358
C	2.73635	-2.32095	2.39636
H	2.23442	-2.65205	1.46517
H	1.96415	-2.19070	3.18288
H	3.42788	-3.12365	2.72773
C	6.94841	-3.29512	-0.74949
H	7.45758	-3.19679	-1.73481
C	6.17645	-4.61944	-0.77604
H	5.38233	-4.58112	-1.55227
H	5.70653	-4.83100	0.20779
H	6.86453	-5.45538	-1.02486
C	8.04666	-3.31984	0.31976
H	8.59075	-2.35112	0.32841
H	8.77739	-4.12589	0.09498
H	7.62142	-3.50191	1.32940
C	-6.80604	-0.90899	0.87945
H	-7.10439	-1.72671	0.18510
C	-6.38931	-1.57346	2.19669
H	-5.51701	-2.23978	2.02654
H	-7.22318	-2.19227	2.59139
H	-6.11906	-0.81512	2.96186
C	-8.03938	-0.01991	1.07654
H	-8.31567	0.46569	0.11614
H	-7.85163	0.76753	1.83664
H	-8.90061	-0.63522	1.41396
H	0.16508	-2.34633	-0.28513
C	-1.14390	-4.19117	-1.52319
C	-1.74263	-3.75521	0.68871
C	-0.21562	-4.02991	-2.58062
C	-2.33455	-4.94953	-1.74360
C	-2.95690	-4.47704	0.54195
C	-0.46324	-4.60920	-3.80489
H	0.66693	-3.42521	-2.41592
C	-2.55851	-5.53291	-3.01593
C	-3.24690	-5.06078	-0.66483
H	-3.62658	-4.57194	1.38864
C	-1.63832	-5.36646	-4.02614
H	0.24662	-4.47443	-4.61468
H	-3.46595	-6.10791	-3.17799
H	-4.17047	-5.61643	-0.80358
H	-1.81403	-5.81101	-5.00082

C	-1.38881	-3.08932	2.00223
H	-0.33594	-2.81447	1.99690
C	-2.24688	-1.81636	2.32960
H	-2.66898	-1.92414	3.33283
H	-3.08921	-1.77070	1.63286
N	-1.53113	-0.57644	2.23343
N	-0.88610	-3.60263	-0.31019
C	-0.59346	-0.15463	3.17258
C	0.08664	1.06086	2.94943
C	-0.32796	-0.85667	4.36187
C	0.98958	1.55273	3.88234
H	-0.12768	1.61772	2.04334
C	0.58349	-0.34931	5.28965
H	-0.81478	-1.80378	4.56497
C	1.24937	0.85322	5.06422
H	1.48870	2.49815	3.69353
H	0.77567	-0.91461	6.19741
H	1.95901	1.23836	5.78926
H	-1.40583	-0.19739	1.30352
Cl	-1.54176	-4.33866	3.34619

Catalyst (TRIP, 3)-Product (R) alternative conformation
Electronic energy (Hartrees): -1870.566261
Free Energy at 1M concentration and 253 K (Hartrees): -1869.345585
Coordinates:

P	0.23191	0.26318	0.57303
O	1.54394	0.61123	-0.34541
O	-0.53378	1.66720	0.86470
O	0.50464	-0.42026	1.85718
O	-0.64733	-0.51667	-0.47540
C	2.38022	1.50558	0.26668
C	3.72363	1.05712	0.70115
C	4.53573	1.91038	1.34081
H	5.52878	1.59644	1.64008
C	4.08901	3.29084	1.62631
C	4.96195	4.19967	2.40430
H	5.93256	3.86164	2.74541
C	4.55429	5.44077	2.69126
H	5.19220	6.10367	3.26140
C	3.22838	5.90709	2.24188
H	2.89633	6.90545	2.49596
C	2.43407	5.09635	1.53326
H	1.45698	5.45052	1.23237
C	2.88486	3.72328	1.18061
C	2.01679	2.79920	0.39098
C	-0.40984	2.66991	-0.06545
C	-1.65192	3.21888	-0.66456
C	-1.59630	4.30343	-1.45016
H	-2.50507	4.71840	-1.86976
C	-0.29520	4.90262	-1.80267
C	-0.24565	6.02886	-2.76345
H	-1.16281	6.44571	-3.16082
C	0.93126	6.53275	-3.14860
H	0.96649	7.35400	-3.85272
C	2.18635	5.95873	-2.62887
H	3.13639	6.35211	-2.96691
C	2.15249	4.94549	-1.75577
H	3.08744	4.52265	-1.41425
C	0.84939	4.39666	-1.28681
C	0.78247	3.27514	-0.29801

C	-2.95456	2.56794	-0.38310
C	-3.58519	2.74474	0.97057
C	-3.58017	1.81085	-1.32994
C	-4.75274	2.12754	1.23256
C	-4.88435	1.17831	-1.00056
C	-5.43593	1.30224	0.21432
H	-5.22495	2.24838	2.19800
H	-5.39768	0.60595	-1.76086
C	-3.01105	1.59798	-2.73501
H	-1.97753	1.99172	-2.81595
C	-2.91332	0.10907	-3.09670
H	-2.38893	-0.44952	-2.29725
H	-2.33580	-0.01367	-4.03783
H	-3.91562	-0.34142	-3.24949
C	-3.85568	2.34749	-3.77131
H	-4.89139	1.94742	-3.80676
H	-3.40113	2.24565	-4.77993
H	-3.89966	3.42808	-3.51922
C	-2.95618	3.63919	2.03845
H	-1.99921	4.06535	1.67804
C	-2.62508	2.84873	3.31247
H	-2.00810	1.95952	3.06488
H	-3.54733	2.50914	3.82904
H	-2.04676	3.48682	4.01431
C	-3.85754	4.83944	2.35529
H	-4.81244	4.51624	2.82099
H	-4.08389	5.39975	1.42285
H	-3.34040	5.52845	3.05687
C	4.19626	-0.29942	0.33812
C	4.60248	-0.57425	-0.93346
C	4.24295	-1.37438	1.38707
C	5.06492	-1.94831	-1.26212
C	4.74013	-2.58230	1.06165
C	5.14708	-2.90099	-0.32330
H	5.35446	-2.17693	-2.27961
H	4.82002	-3.35610	1.81271
C	4.64286	0.48261	-2.03967
H	4.31393	1.47532	-1.67179
C	6.07397	0.68435	-2.55261
H	6.74830	0.93450	-1.70566
H	6.10257	1.52572	-3.27772
H	6.45572	-0.22652	-3.05967
C	3.69532	0.11638	-3.18772
H	2.66292	-0.01197	-2.80165
H	4.01186	-0.82119	-3.69039
H	3.68026	0.93127	-3.94265
C	3.75140	-1.12344	2.81030
H	3.20802	-0.15768	2.86049
C	4.93303	-1.02338	3.78104
H	4.56798	-0.78104	4.80191
H	5.62375	-0.21564	3.45867
H	5.49668	-1.97986	3.82190
C	2.75365	-2.19936	3.27413
H	2.00447	-2.40597	2.48121
H	2.21728	-1.85102	4.18250
H	3.26903	-3.15096	3.52282
C	5.63300	-4.29373	-0.67995
H	5.85443	-4.35141	-1.76967
C	4.55316	-5.34152	-0.38716
H	3.60587	-5.05630	-0.88796

H	4.36489	-5.43397	0.70335
H	4.86908	-6.33288	-0.77617
C	6.93342	-4.62414	0.06064
H	7.70520	-3.85783	-0.16655
H	7.31812	-5.61266	-0.26958
H	6.77284	-4.65680	1.15917
C	-6.75500	0.62262	0.53414
H	-7.09091	0.01680	-0.33691
C	-6.60482	-0.34019	1.71820
H	-5.75835	-1.03509	1.54165
H	-7.53061	-0.94257	1.83716
H	-6.41813	0.21105	2.66396
C	-7.85040	1.65908	0.80635
H	-7.95232	2.34150	-0.06450
H	-7.61696	2.26235	1.70925
H	-8.82489	1.14939	0.96387
H	-0.45878	-1.53613	-0.41235
C	0.50971	-3.66194	-1.34439
C	-0.71196	-3.83388	0.64157
C	1.11765	-2.83004	-2.31866
C	0.64829	-5.07878	-1.43873
C	-0.61736	-5.24667	0.61803
C	1.83536	-3.39600	-3.34814
H	1.01377	-1.75523	-2.22142
C	1.37936	-5.62894	-2.52341
C	0.04756	-5.85651	-0.41851
H	-1.06523	-5.82339	1.41589
C	1.96402	-4.80280	-3.45633
H	2.30860	-2.75888	-4.08866
H	1.48104	-6.70821	-2.59505
H	0.12494	-6.93970	-0.46039
H	2.53131	-5.22687	-4.27894
N	-0.18495	-3.08543	-0.31701
C	-1.41856	-3.03689	1.72962
C	-2.79968	-2.52630	1.30368
H	-2.64458	-1.90632	0.41890
H	-3.18242	-1.86133	2.09295
N	-3.71138	-3.61706	0.98993
H	-3.90542	-4.17415	1.81416
C	-4.86926	-3.38929	0.22894
C	-6.06374	-4.04947	0.56837
C	-4.86761	-2.55542	-0.90314
C	-7.21422	-3.88290	-0.19753
H	-6.08531	-4.69121	1.44641
C	-6.02546	-2.39324	-1.66054
H	-3.95952	-2.04428	-1.20078
C	-7.20805	-3.04926	-1.31683
H	-8.12418	-4.40127	0.09181
H	-5.99986	-1.74598	-2.53250
H	-8.10696	-2.91289	-1.90942
H	-0.78802	-2.18389	1.99631
Cl	-1.57266	-3.99704	3.28367

Catalyst (TRIP, 3)-Product (R) alternative conformation
Electronic energy (Hartrees): -1870.568055
Free Energy at 1M concentration and 253 K (Hartrees): -1869.345551
Coordinates:

P	0.11553	0.08155	-0.90781
O	1.06448	1.42393	-0.84747
O	-1.29956	0.43922	-0.15618

O	0.69232	-0.92474	0.16477
O	0.04698	-0.36621	-2.31224
C	0.91121	2.24214	0.24494
C	2.08829	2.50485	1.11179
C	1.99369	3.37629	2.12601
H	2.85828	3.58953	2.74334
C	0.71158	4.04441	2.41978
C	0.60122	4.93335	3.59925
H	1.46722	5.12106	4.22184
C	-0.56895	5.50454	3.90329
H	-0.64932	6.15203	4.76686
C	-1.75790	5.24295	3.07032
H	-2.70769	5.68914	3.33571
C	-1.66705	4.44739	1.99847
H	-2.55906	4.25120	1.41905
C	-0.36431	3.82718	1.62886
C	-0.23416	2.93638	0.43557
C	-1.91530	1.55139	-0.67467
C	-3.24866	1.42396	-1.31232
C	-3.79372	2.48030	-1.93467
H	-4.78543	2.40584	-2.36601
C	-3.09465	3.78219	-1.94220
C	-3.67728	4.92832	-2.67711
H	-4.60639	4.81034	-3.22074
C	-3.05790	6.11392	-2.67272
H	-3.48336	6.95083	-3.21125
C	-1.78873	6.28369	-1.93884
H	-1.28492	7.24155	-1.95282
C	-1.25767	5.25637	-1.26561
H	-0.31799	5.39657	-0.74753
C	-1.94617	3.93826	-1.24305
C	-1.36868	2.77383	-0.50971
C	-4.07877	0.22967	-1.04457
C	-4.97430	0.23538	0.16431
C	-4.07174	-0.84230	-1.88171
C	-5.74568	-0.84113	0.40819
C	-4.96936	-1.99043	-1.58931
C	-5.75890	-2.00414	-0.50644
H	-6.39561	-0.85698	1.27236
H	-4.99038	-2.82887	-2.27291
C	-3.21013	-0.90143	-3.14041
H	-2.50509	-0.04612	-3.18334
C	-2.34859	-2.17277	-3.17111
H	-1.80553	-2.29311	-2.21069
H	-1.60006	-2.10231	-3.98895
H	-2.96367	-3.07985	-3.34603
C	-4.08656	-0.80759	-4.39358
H	-4.78154	-1.67136	-4.46224
H	-3.44973	-0.79139	-5.30379
H	-4.68178	0.13044	-4.36967
C	-5.06357	1.45177	1.09032
H	-4.36687	2.24834	0.76387
C	-4.65641	1.11288	2.52973
H	-3.61645	0.72749	2.55053
H	-5.33670	0.35935	2.97806
H	-4.68801	2.02873	3.15798
C	-6.46566	2.07238	1.04771
H	-7.22529	1.38094	1.46968
H	-6.74130	2.31396	-0.00127
H	-6.48308	3.01506	1.63550

C	3.38282	1.84280	0.83082
C	4.18384	2.28470	-0.17846
C	3.84539	0.72185	1.72039
C	5.49263	1.61661	-0.40472
C	5.06582	0.19157	1.51855
C	5.92376	0.63323	0.39830
H	6.11799	1.94933	-1.22300
H	5.43615	-0.58196	2.17687
C	3.82454	3.48675	-1.05520
H	2.83661	3.91086	-0.78512
C	4.83442	4.62390	-0.86000
H	4.89212	4.89849	0.21515
H	4.51162	5.52143	-1.42982
H	5.84641	4.33124	-1.21084
C	3.72541	3.09022	-2.53226
H	3.01925	2.24174	-2.65380
H	4.71657	2.80080	-2.93906
H	3.34184	3.94535	-3.12882
C	2.98365	0.20925	2.87401
H	1.95411	0.61353	2.78852
C	3.54487	0.68197	4.21999
H	2.87494	0.36115	5.04610
H	3.61277	1.78970	4.24003
H	4.55724	0.26059	4.39844
C	2.83816	-1.32106	2.85913
H	2.59655	-1.67571	1.83972
H	2.01715	-1.63072	3.54055
H	3.77075	-1.82315	3.19217
C	7.28158	-0.00686	0.17250
H	7.75212	0.41504	-0.74421
C	7.15300	-1.51910	-0.04666
H	6.44298	-1.72473	-0.87211
H	6.79201	-2.03199	0.86955
H	8.13951	-1.94785	-0.32388
C	8.22388	0.29676	1.34236
H	8.31375	1.39530	1.48268
H	9.23597	-0.10980	1.13119
H	7.85056	-0.15586	2.28555
C	-6.67368	-3.18613	-0.23949
H	-6.57763	-3.93411	-1.05870
C	-6.28111	-3.89715	1.05878
H	-5.22976	-4.24757	0.98987
H	-6.92954	-4.78461	1.21962
H	-6.38220	-3.22105	1.93413
C	-8.14327	-2.75002	-0.20690
H	-8.40334	-2.21812	-1.14708
H	-8.34582	-2.07868	0.65411
H	-8.80143	-3.64072	-0.11936
H	1.38978	-1.56439	-0.21829
C	3.43406	-2.36919	-1.66142
C	2.32350	-3.97072	-0.38249
C	3.61536	-1.00842	-2.01522
C	4.25587	-3.36579	-2.27436
C	3.11314	-5.01494	-0.93207
C	4.56045	-0.66651	-2.95692
H	3.00914	-0.24822	-1.53759
C	5.21815	-2.97750	-3.24037
C	4.06259	-4.71079	-1.87357
H	2.96274	-6.03249	-0.59163
C	5.36376	-1.65174	-3.57838

H	4.69465	0.37304	-3.23176
H	5.83617	-3.74217	-3.70252
H	4.67527	-5.49169	-2.31605
H	6.09973	-1.35456	-4.31879
C	1.20739	-4.27722	0.59099
H	0.87151	-3.36340	1.07807
C	0.00995	-4.97960	-0.10070
H	0.36833	-5.88452	-0.60062
H	-0.34198	-4.30305	-0.88812
N	-1.08951	-5.34947	0.75700
H	-0.88705	-6.16448	1.32086
C	-1.85573	-4.39286	1.44069
C	-2.61194	-4.79483	2.55699
C	-1.92545	-3.05239	1.03207
C	-3.40714	-3.88082	3.24031
H	-2.56972	-5.83128	2.88397
C	-2.72208	-2.14528	1.72696
H	-1.34570	-2.69245	0.19237
C	-3.46983	-2.54566	2.83256
H	-3.98171	-4.21602	4.09942
H	-2.72378	-1.11657	1.38731
H	-4.08833	-1.83609	3.37197
N	2.47756	-2.70392	-0.73810
Cl	1.83592	-5.33547	1.95169

Catalyst (TRIP, 3)-Product (R) alternative conformation
Electronic energy (Hartrees): -1870.565423
Free Energy at 1M concentration and 253 K (Hartrees): -1869.34554
Coordinates:

P	-0.48042	-0.19887	0.22317
O	-1.89553	0.31788	0.83856
O	0.37450	1.11909	-0.22313
O	-0.55980	-1.09983	-0.95193
O	0.17891	-0.77808	1.52916
C	-2.52432	1.26215	0.06923
C	-3.81649	0.92382	-0.57798
C	-4.47786	1.85792	-1.27588
H	-5.43106	1.62199	-1.73393
C	-3.90186	3.20565	-1.45302
C	-4.59931	4.19436	-2.30712
H	-5.54895	3.94469	-2.76385
C	-4.05763	5.39795	-2.52167
H	-4.56691	6.11908	-3.14788
C	-2.75599	5.74124	-1.91833
H	-2.31052	6.70742	-2.11743
C	-2.12069	4.85752	-1.14015
H	-1.15436	5.12180	-0.73241
C	-2.72639	3.52593	-0.86325
C	-2.04174	2.52266	0.00796
C	0.31930	2.19992	0.62170
C	1.58929	2.72930	1.18008
C	1.57326	3.82014	1.96042
H	2.50041	4.23063	2.34239
C	0.29519	4.47389	2.30368
C	0.28321	5.61491	3.24783
H	1.21104	5.98383	3.66709
C	-0.87367	6.19282	3.58876
H	-0.88191	7.02558	4.28012
C	-2.14426	5.68921	3.03295
H	-3.07884	6.14757	3.32963

C	-2.14349	4.66271	2.17468
H	-3.08834	4.29521	1.79684
C	-0.86193	4.02804	1.76247
C	-0.83332	2.88720	0.79833
C	2.88284	2.11653	0.79135
C	3.40942	2.34142	-0.59977
C	3.60566	1.37563	1.67751
C	4.58048	1.77830	-0.95336
C	4.92470	0.83322	1.25878
C	5.39011	1.00784	0.01470
H	4.97004	1.92013	-1.95225
H	5.52190	0.28880	1.97844
C	3.12303	1.07635	3.09652
H	2.07103	1.39886	3.23964
C	3.14121	-0.43009	3.38750
H	2.65850	-0.98165	2.55698
H	2.58365	-0.64161	4.32467
H	4.17616	-0.81235	3.50900
C	3.96859	1.83519	4.12499
H	5.02904	1.50633	4.09163
H	3.57387	1.65722	5.14813
H	3.92778	2.92624	3.92311
C	2.67239	3.22747	-1.60468
H	1.74828	3.64359	-1.15805
C	2.23460	2.43050	-2.83957
H	1.61323	1.56517	-2.53074
H	3.10969	2.05826	-3.41272
H	1.62257	3.07145	-3.50931
C	3.52184	4.44082	-2.00576
H	4.42501	4.13358	-2.57390
H	3.83929	4.99796	-1.09835
H	2.92511	5.12809	-2.64297
C	-4.38596	-0.43779	-0.44323
C	-4.98948	-0.83130	0.71309
C	-4.35051	-1.36889	-1.62416
C	-5.61005	-2.18067	0.77974
C	-4.97569	-2.55782	-1.53649
C	-5.62277	-2.99843	-0.28205
H	-6.07697	-2.49960	1.70257
H	-4.99239	-3.22538	-2.38703
C	-5.09830	0.07112	1.94338
H	-4.61653	1.05482	1.77442
C	-6.56501	0.37567	2.27113
H	-7.06675	0.81958	1.38465
H	-6.62384	1.10757	3.10498
H	-7.11257	-0.54203	2.57211
C	-4.38424	-0.55383	3.14875
H	-3.33095	-0.79179	2.88872
H	-4.88917	-1.48509	3.48064
H	-4.37717	0.16198	3.99837
C	-3.63478	-0.98955	-2.92014
H	-3.02735	-0.07417	-2.76581
C	-4.64745	-0.67749	-4.02797
H	-4.11768	-0.33982	-4.94423
H	-5.33160	0.13473	-3.70471
H	-5.25332	-1.57468	-4.27733
C	-2.64891	-2.07731	-3.37950
H	-2.03189	-2.43082	-2.52874
H	-1.97000	-1.66780	-4.15796
H	-3.18083	-2.95108	-3.81147

C	-6.27926	-4.36428	-0.19329
H	-6.70551	-4.51593	0.82411
C	-5.25255	-5.48044	-0.41806
H	-4.41557	-5.37858	0.30559
H	-4.84138	-5.44729	-1.44924
H	-5.72851	-6.47181	-0.26041
C	-7.44203	-4.47839	-1.18573
H	-8.17229	-3.65930	-1.01157
H	-7.96758	-5.44705	-1.04533
H	-7.08255	-4.42185	-2.23500
C	6.74664	0.45394	-0.38296
H	7.21647	-0.06173	0.48488
C	6.60589	-0.58797	-1.49638
H	5.94692	-1.41309	-1.15450
H	7.59899	-1.02026	-1.74299
H	6.17772	-0.13794	-2.41701
C	7.69954	1.58024	-0.80042
H	7.77634	2.33432	0.01196
H	7.34794	2.08334	-1.72590
H	8.71427	1.16885	-0.98828
H	0.52968	-1.74714	1.46512
C	1.36731	-3.55543	2.97992
C	1.90990	-3.83228	0.72350
C	0.62188	-2.86815	3.97281
C	2.23529	-4.62095	3.36771
C	2.79587	-4.89530	1.03146
C	0.74521	-3.22954	5.29578
H	-0.02960	-2.05637	3.66903
C	2.33728	-4.96698	4.73970
C	2.95734	-5.27711	2.34069
H	3.32117	-5.39575	0.22821
C	1.60695	-4.28388	5.68512
H	0.17481	-2.69946	6.05235
H	2.99951	-5.77858	5.02809
H	3.63371	-6.08751	2.59949
H	1.68812	-4.55107	6.73410
N	1.23839	-3.18275	1.66755
C	1.69567	-3.31142	-0.68456
C	2.57269	-2.06883	-0.99883
H	3.62056	-2.36431	-1.06923
H	2.49008	-1.40795	-0.12408
N	2.15973	-1.36182	-2.17573
H	1.20081	-1.03419	-2.13830
C	2.78167	-1.34273	-3.41358
C	2.13027	-0.68071	-4.47636
C	4.04382	-1.91099	-3.66779
C	2.73144	-0.56640	-5.72356
H	1.14683	-0.25036	-4.30397
C	4.63577	-1.78651	-4.92355
H	4.56372	-2.46617	-2.89588
C	3.99584	-1.11172	-5.96255
H	2.20363	-0.04503	-6.51783
H	5.61334	-2.23282	-5.08695
H	4.46509	-1.01994	-6.93684
H	0.65162	-3.01554	-0.79237
Cl	1.95630	-4.60832	-1.93676

Catalyst (TRIP, 3)-Product (R) alternative conformation
Electronic energy (Hartrees): -1870.568022
Free Energy at 1M concentration and 253 K (Hartrees): -1869.345475

Coordinates:

P	0.10811	0.07925	-0.91528
O	1.05584	1.42249	-0.85303
O	-1.30640	0.43205	-0.16024
O	0.68845	-0.92900	0.15413
O	0.03784	-0.36519	-2.32064
C	0.90529	2.23720	0.24247
C	2.08594	2.50241	1.10404
C	1.99325	3.37151	2.12061
H	2.86011	3.58645	2.73408
C	0.71016	4.03352	2.42317
C	0.60216	4.91817	3.60620
H	1.47052	5.10757	4.22494
C	-0.56890	5.48317	3.91830
H	-0.64763	6.12732	4.78449
C	-1.76100	5.21913	3.09064
H	-2.71132	5.66024	3.36250
C	-1.67227	4.42757	2.01569
H	-2.56641	4.22942	1.44023
C	-0.36880	3.81418	1.63727
C	-0.24103	2.92774	0.44041
C	-1.92447	1.54650	-0.67128
C	-3.26198	1.42251	-1.30151
C	-3.81129	2.48248	-1.91384
H	-4.80591	2.41054	-2.33905
C	-3.11278	3.78449	-1.91812
C	-3.70101	4.93504	-2.64136
H	-4.63402	4.82015	-3.17894
C	-3.08222	6.12093	-2.63338
H	-3.51204	6.96124	-3.16309
C	-1.80787	6.28647	-1.90752
H	-1.30442	7.24455	-1.91883
C	-1.27173	5.25500	-1.24476
H	-0.32838	5.39209	-0.73256
C	-1.95982	3.93664	-1.22570
C	-1.37793	2.76840	-0.50224
C	-4.09161	0.22748	-1.03547
C	-4.98495	0.22996	0.17502
C	-4.09036	-0.83972	-1.87860
C	-5.76544	-0.84182	0.41051
C	-4.99565	-1.98378	-1.59377
C	-5.78791	-1.99784	-0.51283
H	-6.41434	-0.85954	1.27543
H	-5.02198	-2.81761	-2.28281
C	-3.22863	-0.89640	-3.13728
H	-2.52121	-0.04291	-3.17693
C	-2.37038	-2.16973	-3.17175
H	-1.82463	-2.29195	-2.21308
H	-1.62407	-2.10034	-3.99171
H	-2.98833	-3.07510	-3.34542
C	-4.10416	-0.79570	-4.39056
H	-4.80029	-1.65816	-4.46371
H	-3.46670	-0.77629	-5.30025
H	-4.69805	0.14306	-4.36277
C	-5.05889	1.43715	1.11420
H	-4.35377	2.22922	0.79497
C	-4.65234	1.07783	2.54881
H	-3.61969	0.67263	2.56182
H	-5.34491	0.33308	2.99304
H	-4.66473	1.98842	3.18534

C	-6.45397	2.07376	1.08153
H	-7.22055	1.38574	1.49647
H	-6.72857	2.33094	0.03601
H	-6.45987	3.00942	1.68065
C	3.38164	1.84518	0.81695
C	4.17524	2.28812	-0.19780
C	3.85619	0.73171	1.70967
C	5.48825	1.62891	-0.42599
C	5.08007	0.21019	1.50591
C	5.93076	0.65389	0.38103
H	6.10816	1.96286	-1.24790
H	5.45862	-0.55762	2.16625
C	3.80471	3.48469	-1.07737
H	2.81482	3.90292	-0.80545
C	4.80730	4.62953	-0.88943
H	4.86667	4.90825	0.18455
H	4.47633	5.52281	-1.46126
H	5.82012	4.34275	-1.24270
C	3.70354	3.08253	-2.55277
H	3.00276	2.22895	-2.66926
H	4.69533	2.79844	-2.96175
H	3.31232	3.93304	-3.15096
C	3.00227	0.21623	2.86783
H	1.97140	0.61836	2.78939
C	3.57018	0.68787	4.21148
H	2.90430	0.36636	5.04059
H	3.63803	1.79562	4.23206
H	4.58336	0.26643	4.38493
C	2.85972	-1.31411	2.85075
H	2.60389	-1.66549	1.83371
H	2.04890	-1.62739	3.54262
H	3.79788	-1.81536	3.16898
C	7.29451	0.02632	0.15570
H	7.75702	0.44507	-0.76652
C	7.18169	-1.48909	-0.04938
H	6.46724	-1.70974	-0.86690
H	6.83417	-1.99827	0.87408
H	8.17080	-1.90885	-0.33106
C	8.23761	0.35055	1.31933
H	8.31728	1.45125	1.44849
H	9.25290	-0.04831	1.10886
H	7.87154	-0.09622	2.26816
C	-6.71503	-3.17272	-0.25693
H	-6.62575	-3.91452	-1.08250
C	-6.33164	-3.89907	1.03556
H	-5.28452	-4.26155	0.96418
H	-6.99065	-4.78009	1.18871
H	-6.42534	-3.22913	1.91645
C	-8.18011	-2.72178	-0.22224
H	-8.43354	-2.17896	-1.15798
H	-8.37679	-2.05598	0.64444
H	-8.84755	-3.60636	-0.14328
H	1.39188	-1.56174	-0.22813
C	3.45653	-2.36284	-1.65533
C	2.35841	-3.96034	-0.36102
C	3.62087	-1.00544	-2.02974
C	4.29879	-3.35663	-2.24483
C	3.17033	-5.00076	-0.88482
C	4.56866	-0.66476	-2.96924
H	2.99949	-0.24669	-1.56958

C	5.26299	-2.96982	-3.20953
C	4.12374	-4.69748	-1.82251
H	3.03295	-6.01458	-0.52823
C	5.39156	-1.64774	-3.56822
H	4.68949	0.37206	-3.26018
H	5.89653	-3.73256	-3.65351
H	4.75327	-5.47572	-2.24559
H	6.12920	-1.35161	-4.30738
C	1.23511	-4.26971	0.60326
H	0.88175	-3.35500	1.07607
C	0.05576	-4.99466	-0.09696
H	0.43326	-5.89859	-0.58453
H	-0.29521	-4.32957	-0.89463
N	-1.04955	-5.37308	0.74883
N	2.49620	-2.69707	-0.73562
C	-1.83904	-4.42330	1.41500
C	-2.61320	-4.83178	2.51654
C	-1.91423	-3.08413	1.00307
C	-3.43060	-3.92510	3.18298
H	-2.56723	-5.86753	2.84524
C	-2.73367	-2.18434	1.68088
H	-1.32129	-2.71942	0.17467
C	-3.49860	-2.59112	2.77226
H	-4.01907	-4.26511	4.03070
H	-2.73948	-1.15587	1.34040
H	-4.13546	-1.88770	3.29806
H	-0.84547	-6.18289	1.31946
Cl	1.85909	-5.30511	1.98323

Catalyst (TRIP, 3)-Product (R) alternative conformation
Electronic energy (Hartrees): -1870.566375
Free Energy at 1M concentration and 253 K (Hartrees): -1869.343853
Coordinates:

P	-0.36179	0.09689	0.23003
O	0.35144	1.27619	-0.65936
O	-1.72585	0.72881	0.86032
O	0.47140	-0.48733	1.30341
O	-0.82351	-0.85927	-0.93557
C	0.44134	2.49135	-0.02754
C	1.78402	3.07333	0.23787
C	1.88605	4.29443	0.78423
H	2.86171	4.73056	0.96443
C	0.67680	5.04216	1.17757
C	0.81098	6.33931	1.87991
H	1.79224	6.75633	2.06970
C	-0.27881	6.99932	2.28620
H	-0.18008	7.94615	2.80123
C	-1.61919	6.43136	2.04705
H	-2.49610	6.95824	2.40060
C	-1.75081	5.26301	1.40834
H	-2.74193	4.85275	1.26764
C	-0.54903	4.53254	0.92003
C	-0.66870	3.23222	0.19315
C	-2.45355	1.54578	0.02947
C	-3.85239	1.16538	-0.30159
C	-4.63664	2.02073	-0.97510
H	-5.66812	1.76384	-1.18521
C	-4.08837	3.29018	-1.48843
C	-4.92961	4.16147	-2.34110
H	-5.95791	3.89163	-2.54740

C	-4.42188	5.28278	-2.86356
H	-5.03650	5.91789	-3.48836
C	-3.01694	5.64940	-2.60330
H	-2.60433	6.54450	-3.05068
C	-2.25075	4.87191	-1.82948
H	-1.21580	5.14649	-1.67546
C	-2.81115	3.63862	-1.21139
C	-1.98378	2.76415	-0.32450
C	-4.39842	-0.13231	0.16531
C	-4.67493	-0.32143	1.63236
C	-4.66940	-1.14229	-0.71278
C	-5.14029	-1.50918	2.06089
C	-5.20278	-2.42778	-0.18792
C	-5.40970	-2.61803	1.12260
H	-5.35693	-1.65934	3.10973
H	-5.43335	-3.22505	-0.88195
C	-4.49837	-1.00059	-2.22892
H	-4.01207	-0.04136	-2.49575
C	-3.59253	-2.09079	-2.81644
H	-2.59576	-2.05604	-2.33818
H	-3.44836	-1.91731	-3.90430
H	-4.02452	-3.10403	-2.68427
C	-5.86621	-1.00964	-2.92162
H	-6.37329	-1.98940	-2.79283
H	-5.74528	-0.81442	-4.00864
H	-6.51479	-0.21372	-2.49818
C	-4.50764	0.81907	2.63739
H	-4.11289	1.72629	2.13999
C	-3.50317	0.45806	3.74134
H	-2.54542	0.11699	3.29574
H	-3.89572	-0.34625	4.39833
H	-3.29435	1.35039	4.36934
C	-5.85885	1.22815	3.23806
H	-6.29472	0.40934	3.84859
H	-6.57096	1.48903	2.42596
H	-5.73171	2.12065	3.88744
C	3.01702	2.33037	-0.11888
C	3.41457	2.21362	-1.41758
C	3.89083	1.79276	0.98133
C	4.71724	1.56227	-1.71789
C	5.08220	1.25127	0.66440
C	5.52642	1.13139	-0.74069
H	5.03239	1.46750	-2.74884
H	5.73302	0.87924	1.44377
C	2.61694	2.79010	-2.58870
H	1.68025	3.27428	-2.24913
C	3.41359	3.88607	-3.30712
H	3.71878	4.67003	-2.58122
H	2.78370	4.36370	-4.08787
H	4.32194	3.47439	-3.79491
C	2.19835	1.68545	-3.56715
H	1.62702	0.89823	-3.03280
H	3.08003	1.21876	-4.05405
H	1.54380	2.10792	-4.35913
C	3.46293	1.85711	2.44707
H	2.43204	2.25534	2.53356
C	4.37098	2.80024	3.24575
H	3.98894	2.90788	4.28336
H	4.38408	3.80631	2.77567
H	5.41095	2.41306	3.29108

C	3.42339	0.46429	3.08779
H	2.79081	-0.20887	2.47701
H	2.98222	0.52517	4.10548
H	4.43870	0.02355	3.17385
C	6.88255	0.53453	-1.07178
H	7.05094	0.55939	-2.17215
C	6.95387	-0.93385	-0.64331
H	6.16490	-1.51204	-1.16562
H	6.81797	-1.04126	0.45355
H	7.93806	-1.36673	-0.92232
C	8.01163	1.34968	-0.43046
H	7.93487	2.41436	-0.73881
H	8.99740	0.96223	-0.76563
H	7.97023	1.29235	0.67784
C	-5.94873	-3.94154	1.63482
H	-6.04776	-4.66288	0.79271
C	-4.99018	-4.57024	2.65440
H	-3.96768	-4.64465	2.22767
H	-5.33327	-5.59408	2.91529
H	-4.94258	-3.96828	3.58637
C	-7.34702	-3.76266	2.23656
H	-8.02983	-3.31330	1.48398
H	-7.31807	-3.10579	3.13174
H	-7.76128	-4.74919	2.53550
H	-0.40335	-1.79843	-0.93654
C	-0.59711	-4.35350	-1.12346
C	1.45397	-3.37994	-1.66182
C	-1.90455	-4.21265	-0.59350
C	-0.15945	-5.63320	-1.58133
C	1.96900	-4.61907	-2.11652
C	-2.74926	-5.29941	-0.55635
H	-2.21786	-3.24085	-0.22743
C	-1.05267	-6.73312	-1.51947
C	1.16242	-5.72900	-2.08251
H	2.98120	-4.66943	-2.49422
C	-2.32503	-6.56703	-1.02234
H	-3.75530	-5.18856	-0.16798
H	-0.71505	-7.70395	-1.87143
H	1.53015	-6.68956	-2.43311
H	-3.00868	-7.40917	-0.98045
N	0.22107	-3.25751	-1.19378
C	2.28237	-2.10817	-1.59756
C	2.99169	-1.94013	-0.25188
H	2.20249	-1.85907	0.49918
H	3.51919	-0.97622	-0.25713
N	3.86283	-3.06770	0.03982
H	4.57334	-3.17807	-0.67384
C	4.39905	-3.22440	1.32955
C	5.71901	-3.68456	1.47889
C	3.63858	-2.98204	2.48644
C	6.26414	-3.88896	2.74349
H	6.32083	-3.87259	0.59211
C	4.19912	-3.17750	3.74694
H	2.61166	-2.64212	2.40714
C	5.51153	-3.63054	3.89020
H	7.28862	-4.24058	2.82985
H	3.59559	-2.97266	4.62677
H	5.93988	-3.77871	4.87646
H	1.63368	-1.24884	-1.76101
Cl	3.50244	-2.02341	-2.96867

Catalyst (TRIP, 3)-Product (R) alternative conformation
Electronic energy (Hartrees): -1870.566801
Free Energy at 1M concentration and 253 K (Hartrees): -1869.343712
Coordinates:

P	-0.28116	-0.16798	0.44811
O	0.14413	-1.66398	0.91947
O	-1.61104	-0.32652	-0.47827
O	0.77944	0.30871	-0.61135
O	-0.44466	0.68381	1.65366
C	-0.20416	-2.73324	0.12748
C	0.87895	-3.64625	-0.31649
C	0.58338	-4.83086	-0.86833
H	1.37871	-5.49710	-1.18277
C	-0.81516	-5.17138	-1.18498
C	-1.11259	-6.41605	-1.93095
H	-0.31736	-7.11252	-2.16627
C	-2.36255	-6.68253	-2.32278
H	-2.57855	-7.59090	-2.87026
C	-3.44726	-5.72734	-2.03082
H	-4.45050	-5.93130	-2.38225
C	-3.18988	-4.60338	-1.35216
H	-3.99597	-3.90212	-1.18540
C	-1.81143	-4.31288	-0.86604
C	-1.50103	-3.07883	-0.07386
C	-2.65202	-0.92559	0.18441
C	-3.89539	-0.15034	0.42956
C	-4.94813	-0.74578	1.01136
H	-5.87107	-0.19768	1.15939
C	-4.84277	-2.13530	1.49924
C	-5.96239	-2.73452	2.26143
H	-6.87181	-2.17016	2.42630
C	-5.85340	-3.97365	2.75289
H	-6.67110	-4.41021	3.31166
C	-4.61207	-4.74452	2.54766
H	-4.52130	-5.73759	2.96849
C	-3.59733	-4.21420	1.85523
H	-2.68684	-4.78663	1.73618
C	-3.71617	-2.84988	1.27341
C	-2.60526	-2.24331	0.47908
C	-3.99453	1.25242	-0.04271
C	-4.04157	1.52268	-1.52269
C	-4.03273	2.29374	0.83806
C	-4.06140	2.79786	-1.95306
C	-4.08330	3.68287	0.30965
C	-4.07036	3.93273	-1.00682
H	-4.10252	3.01132	-3.01247
H	-4.12404	4.50998	1.00606
C	-4.03978	2.10328	2.35694
H	-3.89386	1.04085	2.63544
C	-2.89078	2.86148	3.03111
H	-1.93002	2.57412	2.56505
H	-2.84246	2.59790	4.10928
H	-3.01731	3.96078	2.94942
C	-5.38866	2.53286	2.94481
H	-5.56395	3.61910	2.79325
H	-5.41534	2.31892	4.03469
H	-6.21305	1.96729	2.46094
C	-4.12888	0.38958	-2.54619
H	-4.10325	-0.59670	-2.04322

C	-2.94092	0.41305	-3.51916
H	-1.98236	0.42789	-2.95980
H	-2.97876	1.30302	-4.18188
H	-2.95527	-0.49723	-4.15590
C	-5.46079	0.43283	-3.30655
H	-5.54047	1.34708	-3.93176
H	-6.30807	0.41377	-2.58798
H	-5.54880	-0.45428	-3.96969
C	2.28731	-3.20831	-0.20703
C	2.92821	-3.21168	0.99363
C	2.98629	-2.68701	-1.43363
C	4.28512	-2.61467	1.08518
C	4.21193	-2.14405	-1.30077
C	4.89098	-2.08430	0.01334
H	4.79242	-2.61010	2.04073
H	4.73675	-1.77079	-2.16980
C	2.32889	-3.84691	2.24987
H	1.31170	-4.24585	2.05738
C	3.17295	-5.04483	2.70112
H	3.26923	-5.77558	1.86959
H	2.68171	-5.55668	3.55618
H	4.18826	-4.72645	3.01881
C	2.18371	-2.82632	3.38585
H	1.55829	-1.97252	3.05628
H	3.17084	-2.44286	3.71822
H	1.68231	-3.29849	4.25749
C	2.37365	-2.83367	-2.83014
H	1.36479	-3.28203	-2.77422
C	3.21272	-3.77909	-3.69933
H	2.70063	-3.96218	-4.66818
H	3.33834	-4.75580	-3.18469
H	4.21585	-3.35030	-3.90724
C	2.17272	-1.48413	-3.52611
H	1.52802	-0.82987	-2.90472
H	1.66125	-1.63277	-4.50115
H	3.14140	-0.98231	-3.71900
C	6.27476	-1.47343	0.14192
H	6.58608	-1.46182	1.21082
C	6.28658	-0.01863	-0.34061
H	5.44987	0.55002	0.11790
H	6.19299	0.03184	-1.44416
H	7.24118	0.46920	-0.04972
C	7.30947	-2.31033	-0.61787
H	7.30317	-3.35549	-0.24091
H	8.32636	-1.89093	-0.46212
H	7.09380	-2.32144	-1.70753
C	-4.09568	5.36112	-1.52027
H	-4.05851	6.07278	-0.66473
C	-2.87204	5.65369	-2.39767
H	-1.93842	5.41507	-1.84617
H	-2.84880	6.73073	-2.66882
H	-2.89548	5.05671	-3.33375
C	-5.39559	5.64222	-2.28221
H	-6.27091	5.42971	-1.63170
H	-5.46826	5.01641	-3.19695
H	-5.43731	6.71128	-2.58148
H	1.14238	1.25907	-0.48900
C	2.95021	2.81563	-1.45555
C	1.49177	3.88768	0.00780
C	3.41473	1.61467	-2.04603

C	3.59679	4.04586	-1.78731
C	2.10087	5.14485	-0.25089
C	4.45000	1.64712	-2.95443
H	2.94894	0.68308	-1.75208
C	4.66146	4.04413	-2.72299
C	3.13215	5.22187	-1.14919
H	1.72268	6.02758	0.24990
C	5.07665	2.86667	-3.30117
H	4.80424	0.72558	-3.40471
H	5.14278	4.98628	-2.97036
H	3.60305	6.17476	-1.37569
H	5.89208	2.86704	-4.01769
C	0.39599	3.76203	1.03989
H	-0.19000	2.86330	0.85887
C	0.92627	3.71258	2.49993
H	0.05939	3.81957	3.15986
H	1.57353	4.57067	2.69328
N	1.60521	2.48032	2.79446
N	1.89156	2.76837	-0.58086
C	2.97551	2.26155	2.78068
C	3.43897	0.93798	2.94351
C	3.93513	3.28453	2.65774
C	4.79756	0.65661	2.99702
H	2.71081	0.13919	3.04098
C	5.29719	2.98517	2.70661
H	3.62879	4.31578	2.52099
C	5.74608	1.67682	2.87883
H	5.11916	-0.37163	3.13493
H	6.01468	3.79572	2.60767
H	6.80755	1.45379	2.91527
H	1.01757	1.65846	2.68725
Cl	-0.79333	5.15421	0.90425

Catalyst (TRIP, 3)-Product (R) alternative conformation
Electronic energy (Hartrees): -1870.56572
Free Energy at 1M concentration and 253 K (Hartrees): -1869.343618
Coordinates:

P	0.07400	-0.13500	-1.22248
O	-0.80116	0.46215	0.03011
O	1.49237	0.65856	-1.28495
O	-0.59684	-0.05272	-2.53787
O	0.42986	-1.57008	-0.66192
C	-0.88961	1.82906	0.03288
C	-2.21817	2.47689	-0.12942
C	-2.31794	3.81336	-0.06758
H	-3.28509	4.29232	-0.16673
C	-1.11422	4.64847	0.10372
C	-1.23222	6.12441	0.06204
H	-2.20366	6.58968	-0.05022
C	-0.13930	6.88884	0.15868
H	-0.22586	7.96724	0.12593
C	1.19020	6.26407	0.29331
H	2.07355	6.88761	0.34278
C	1.30628	4.93187	0.33824
H	2.29399	4.49607	0.40688
C	0.09559	4.06777	0.26926
C	0.19534	2.57754	0.32842
C	2.06657	1.02653	-0.09121
C	3.47985	0.63939	0.15662
C	4.11625	1.07609	1.25339

H	5.15866	0.82700	1.41423
C	3.41194	1.91388	2.24095
C	4.11640	2.35871	3.46541
H	5.15016	2.08135	3.62951
C	3.47922	3.10703	4.37261
H	3.99400	3.43105	5.26775
C	2.06211	3.46482	4.17196
H	1.54034	4.03585	4.92893
C	1.41978	3.07282	3.06572
H	0.37441	3.32638	2.94852
C	2.13264	2.29004	2.01953
C	1.46330	1.91311	0.73711
C	4.23132	-0.11768	-0.87105
C	4.70197	0.60037	-2.10558
C	4.53314	-1.43378	-0.69388
C	5.40699	-0.08167	-3.02770
C	5.35265	-2.13037	-1.72024
C	5.75787	-1.50506	-2.83423
H	5.75200	0.41689	-3.92331
H	5.62821	-3.16455	-1.56145
C	4.07652	-2.22813	0.52898
H	3.36750	-1.63983	1.14540
C	3.32109	-3.50119	0.12549
H	2.48872	-3.25223	-0.56497
H	2.89592	-3.98609	1.02849
H	3.98898	-4.23592	-0.36945
C	5.26994	-2.56815	1.42757
H	5.99258	-3.22632	0.90010
H	4.91990	-3.08795	2.34507
H	5.79181	-1.63753	1.73585
C	4.42622	2.08856	-2.32378
H	3.85242	2.51110	-1.47602
C	3.57406	2.32252	-3.57831
H	2.64317	1.71914	-3.52644
H	4.12812	2.04688	-4.50018
H	3.28812	3.39382	-3.64811
C	5.73091	2.89351	-2.38508
H	6.33365	2.62110	-3.27692
H	6.33479	2.70611	-1.47140
H	5.50468	3.98020	-2.43392
C	-3.44613	1.67151	-0.34132
C	-4.04783	1.02635	0.69661
C	-4.12346	1.71745	-1.68291
C	-5.40868	0.45813	0.50180
C	-5.36808	1.21760	-1.80508
C	-6.06451	0.59556	-0.65833
H	-5.90584	-0.01413	1.33902
H	-5.88254	1.26863	-2.75493
C	-3.42900	0.93403	2.09045
H	-2.43124	1.41268	2.13114
C	-4.28738	1.67038	3.12636
H	-4.45179	2.72097	2.80407
H	-3.76622	1.68714	4.10753
H	-5.27227	1.17710	3.26359
C	-3.21508	-0.52969	2.49135
H	-2.55318	-1.03405	1.75505
H	-4.17582	-1.08265	2.54757
H	-2.73006	-0.57664	3.48693
C	-3.43593	2.33512	-2.89929
H	-2.39052	2.61043	-2.64995

C	-4.14494	3.62278	-3.33685
H	-3.58168	4.10497	-4.16413
H	-4.19547	4.33907	-2.49007
H	-5.17735	3.41188	-3.68798
C	-3.34081	1.34326	-4.06694
H	-2.87151	0.39894	-3.72694
H	-2.71091	1.77026	-4.87631
H	-4.34114	1.11310	-4.48973
C	-7.51581	0.16200	-0.77626
H	-7.88559	-0.20028	0.20965
C	-7.66810	-0.99917	-1.76075
H	-7.11353	-1.87909	-1.37799
H	-7.28333	-0.73016	-2.76725
H	-8.73721	-1.28645	-1.85335
C	-8.40984	1.34244	-1.17548
H	-8.26872	2.18473	-0.46468
H	-9.47786	1.03820	-1.14340
H	-8.17644	1.69494	-2.20240
C	6.57948	-2.24352	-3.87541
H	6.74151	-3.29736	-3.55455
C	5.84187	-2.29466	-5.21835
H	4.83932	-2.75452	-5.08420
H	6.41329	-2.91394	-5.94234
H	5.71592	-1.27792	-5.64712
C	7.96503	-1.60656	-4.03278
H	8.48170	-1.57255	-3.04973
H	7.89005	-0.57404	-4.43489
H	8.58420	-2.21144	-4.72915
H	-0.37833	-2.18436	-0.58124
C	-2.81772	-3.16431	-1.01536
C	-1.61739	-3.97480	0.81274
C	-2.85588	-2.35993	-2.18365
C	-3.95546	-3.96230	-0.67506
C	-2.72068	-4.75983	1.23863
C	-3.96979	-2.39019	-2.99413
H	-2.00763	-1.73051	-2.43610
C	-5.08508	-3.96284	-1.53098
C	-3.87728	-4.74667	0.50130
H	-2.63061	-5.39094	2.11504
C	-5.08318	-3.20343	-2.67872
H	-3.99132	-1.78745	-3.89616
H	-5.94093	-4.58011	-1.27209
H	-4.73006	-5.35286	0.79544
H	-5.94254	-3.21729	-3.34228
N	-1.67694	-3.19321	-0.25462
C	-0.27787	-4.07516	1.51055
C	-0.29360	-4.02344	3.04783
H	0.69170	-4.33493	3.40895
H	-1.00875	-4.75556	3.43470
N	-0.62699	-2.70495	3.54696
H	-1.51976	-2.63936	4.01088
C	0.34187	-1.82806	4.04150
C	-0.03099	-0.81851	4.94862
C	1.69157	-1.91705	3.65815
C	0.92119	0.04487	5.47917
H	-1.06988	-0.73147	5.25710
C	2.63765	-1.04971	4.20253
H	2.00782	-2.66489	2.94111
C	2.26687	-0.07301	5.12494
H	0.60982	0.80641	6.18868

H	3.67639	-1.14536	3.89879
H	3.01125	0.58002	5.56682
H	0.39021	-3.31081	1.11709
Cl	0.47171	-5.69133	1.02265

Catalyst (TRIP, 3)-Product (R) alternative conformation
Electronic energy (Hartrees): -1870.565472
Free Energy at 1M concentration and 253 K (Hartrees): -1869.34356
Coordinates:

P	-0.29468	0.11058	0.35916
O	-1.74340	0.68473	0.87653
O	0.45927	1.33237	-0.42055
O	-0.59067	-0.83192	-0.86847
O	0.43268	-0.48365	1.50214
C	-2.34875	1.62127	0.07216
C	-3.68560	1.31618	-0.50285
C	-4.34290	2.25185	-1.20241
H	-5.32569	2.03862	-1.60657
C	-3.72555	3.56469	-1.46386
C	-4.42801	4.54551	-2.32341
H	-5.41157	4.31888	-2.71595
C	-3.84929	5.71297	-2.62258
H	-4.36211	6.42810	-3.25275
C	-2.50208	6.02257	-2.10875
H	-2.02695	6.95709	-2.37808
C	-1.86222	5.14595	-1.32627
H	-0.86286	5.38441	-0.98885
C	-2.50792	3.85619	-0.95283
C	-1.82027	2.86185	-0.07178
C	0.55850	2.46723	0.34245
C	1.89869	2.94942	0.75843
C	2.00715	4.06814	1.49089
H	2.98430	4.44755	1.76511
C	0.79689	4.79942	1.91948
C	0.92461	5.97856	2.80640
H	1.90292	6.31479	3.12675
C	-0.16790	6.63142	3.21775
H	-0.07388	7.49197	3.86744
C	-1.50616	6.17449	2.79582
H	-2.38685	6.69635	3.14740
C	-1.63222	5.11346	1.99027
H	-2.62316	4.78292	1.70803
C	-0.42534	4.39263	1.50356
C	-0.53864	3.20973	0.59956
C	3.11370	2.26687	0.25558
C	3.51552	2.46948	-1.17898
C	3.87436	1.48300	1.07063
C	4.61910	1.85314	-1.64061
C	5.08662	0.82556	0.51229
C	5.44124	0.98628	-0.77065
H	4.93121	1.99903	-2.66555
H	5.70334	0.21718	1.16081
C	3.55801	1.28064	2.55308
H	2.55597	1.68278	2.80833
C	3.51974	-0.20521	2.93269
H	2.89251	-0.76888	2.21563
H	3.08266	-0.32582	3.94685
H	4.53707	-0.64684	2.94376
C	4.57541	2.02941	3.42087
H	5.60027	1.62830	3.26894

H	4.30928	1.92783	4.49471
H	4.57469	3.10987	3.16478
C	2.72381	3.38463	-2.11411
H	1.86328	3.83921	-1.58569
C	2.13755	2.60077	-3.29570
H	1.53106	1.74748	-2.92460
H	2.93874	2.20918	-3.95730
H	1.47565	3.25970	-3.89736
C	3.57901	4.56170	-2.60173
H	4.41059	4.21772	-3.25198
H	4.00378	5.10728	-1.73196
H	2.95182	5.27116	-3.18290
C	-4.31027	-0.01481	-0.31459
C	-4.94806	-0.32946	0.84636
C	-4.36383	-0.96528	-1.47834
C	-5.72305	-1.59679	0.92256
C	-5.12326	-2.07219	-1.38397
C	-5.82971	-2.41729	-0.13244
H	-6.23499	-1.84506	1.84318
H	-5.20957	-2.74336	-2.22732
C	-4.94988	0.59091	2.06690
H	-4.35494	1.50871	1.88848
C	-6.37009	1.06706	2.39430
H	-6.81946	1.55736	1.50417
H	-6.34069	1.80916	3.22062
H	-7.01958	0.22235	2.70594
C	-4.30826	-0.10202	3.27504
H	-3.29029	-0.46128	3.01182
H	-4.91662	-0.96553	3.61643
H	-4.21485	0.61503	4.11842
C	-3.58508	-0.69458	-2.76395
H	-2.87909	0.14748	-2.60827
C	-4.53183	-0.28785	-3.89910
H	-3.94781	-0.03510	-4.80971
H	-5.12022	0.60671	-3.60691
H	-5.23545	-1.11167	-4.14485
C	-2.72493	-1.89903	-3.18278
H	-2.17747	-2.31069	-2.31104
H	-1.98260	-1.58578	-3.94764
H	-3.34659	-2.71005	-3.61733
C	-6.67183	-3.67788	-0.04729
H	-7.08751	-3.78796	0.97973
C	-5.83119	-4.93029	-0.32551
H	-4.96483	-4.97202	0.36534
H	-5.45606	-4.94189	-1.37044
H	-6.44463	-5.84211	-0.16337
C	-7.86366	-3.59682	-1.00756
H	-8.46687	-2.68962	-0.78912
H	-8.51678	-4.48638	-0.87981
H	-7.52445	-3.55602	-2.06453
C	6.69489	0.32378	-1.31221
H	7.10344	-0.38413	-0.55635
C	6.39680	-0.49136	-2.57788
H	5.51979	-1.15030	-2.41553
H	7.27135	-1.12718	-2.83249
H	6.18358	0.16978	-3.44404
C	7.77946	1.37103	-1.58382
H	8.00783	1.93156	-0.65203
H	7.45162	2.09141	-2.36360
H	8.71168	0.87384	-1.92736

H	-0.52852	-1.82635	-0.58832
C	-1.19826	-3.91425	0.74252
C	0.75858	-3.98872	-0.52832
C	-2.31453	-3.17229	1.20505
C	-0.97965	-5.23740	1.23343
C	1.04682	-5.30448	-0.08709
C	-3.16345	-3.72719	2.13626
H	-2.46650	-2.16420	0.83680
C	-1.89430	-5.78902	2.16667
C	0.17411	-5.92117	0.77536
H	1.94694	-5.79478	-0.43265
C	-2.96137	-5.04456	2.61383
H	-4.00091	-3.15256	2.51549
H	-1.72781	-6.79883	2.53129
H	0.37056	-6.92905	1.13123
H	-3.65220	-5.46218	3.33955
C	1.72334	-3.17946	-1.37543
H	1.15902	-2.46427	-1.97501
C	2.72041	-2.38616	-0.50086
H	2.12659	-1.60918	-0.00487
H	3.43890	-1.88408	-1.15341
N	3.41165	-3.17816	0.50579
N	-0.32843	-3.33755	-0.14294
C	4.81151	-3.23225	0.57826
C	5.60935	-3.33998	-0.57289
C	5.44451	-3.22035	1.83375
C	6.99784	-3.39059	-0.46387
H	5.13910	-3.41186	-1.54706
C	6.83137	-3.28612	1.93142
H	4.83995	-3.14612	2.73488
C	7.62254	-3.35940	0.78331
H	7.59466	-3.46504	-1.36877
H	7.29544	-3.26541	2.91361
H	8.70444	-3.39893	0.86021
H	2.98623	-3.08151	1.41794
Cl	2.58601	-4.23475	-2.59128

Catalyst (TRIP, 3)-Product (R) alternative conformation
Electronic energy (Hartrees): -1870.564892
Free Energy at 1M concentration and 253 K (Hartrees): -1869.343229
Coordinates:

P	0.04509	-0.09664	-0.23219
O	-1.14462	0.89389	0.30717
O	1.36738	0.81554	-0.51468
O	-0.27591	-0.88616	-1.43969
O	0.37031	-0.89914	1.08853
C	-1.39306	1.92993	-0.55319
C	-2.69518	1.99551	-1.25847
C	-2.92486	2.97747	-2.14198
H	-3.88400	3.04680	-2.64190
C	-1.88653	3.99454	-2.41274
C	-2.12561	5.03463	-3.43951
H	-3.06245	5.05798	-3.98220
C	-1.18485	5.94836	-3.70230
H	-1.35982	6.70723	-4.45394
C	0.09912	5.91933	-2.97551
H	0.86327	6.65010	-3.20699
C	0.32318	4.98779	-2.04148
H	1.27898	4.97216	-1.53456
C	-0.72319	3.98070	-1.71930

C	-0.49626	2.92879	-0.68455
C	1.54219	1.92439	0.27795
C	2.79585	2.03699	1.06435
C	3.03503	3.13581	1.79364
H	3.96576	3.23528	2.33956
C	2.03701	4.22042	1.85876
C	2.27627	5.38447	2.74281
H	3.19361	5.45425	3.31411
C	1.35941	6.35260	2.84235
H	1.53408	7.20180	3.49029
C	0.10029	6.25946	2.07977
H	-0.64840	7.03420	2.18382
C	-0.12246	5.21565	1.27292
H	-1.06464	5.15765	0.74480
C	0.90199	4.14461	1.12593
C	0.68051	2.97028	0.22700
C	3.82767	0.97896	0.96580
C	4.73234	0.95816	-0.23384
C	3.98959	0.05867	1.95739
C	5.68626	0.01089	-0.31493
C	5.09165	-0.93265	1.84619
C	5.89421	-0.97021	0.77307
H	6.34461	-0.02245	-1.17233
H	5.25692	-1.62852	2.65788
C	3.11436	0.03362	3.21149
H	2.21625	0.67297	3.08024
C	2.58444	-1.37612	3.51823
H	2.18101	-1.85003	2.60271
H	1.76685	-1.31707	4.26823
H	3.38035	-2.03118	3.93013
C	3.89043	0.58497	4.41224
H	4.77809	-0.04515	4.63453
H	3.23688	0.61005	5.31030
H	4.23270	1.62044	4.20494
C	4.62498	2.01544	-1.33385
H	3.80910	2.73149	-1.11258
C	4.28272	1.38861	-2.69066
H	3.34695	0.79651	-2.61216
H	5.10222	0.73130	-3.04813
H	4.12022	2.18626	-3.44665
C	5.90611	2.85496	-1.42259
H	6.76979	2.24116	-1.75475
H	6.13892	3.29697	-0.43008
H	5.76707	3.68545	-2.14742
C	-3.78056	1.05571	-0.89419
C	-4.51986	1.25715	0.23241
C	-4.10520	-0.09221	-1.80651
C	-5.63281	0.32082	0.54206
C	-5.17226	-0.86484	-1.52928
C	-5.96789	-0.66805	-0.29866
H	-6.20401	0.46444	1.45006
H	-5.44856	-1.66774	-2.19883
C	-4.29113	2.44242	1.17354
H	-3.47691	3.10305	0.81350
C	-5.53908	3.33026	1.25482
H	-5.84502	3.64819	0.23505
H	-5.31802	4.24228	1.84968
H	-6.38623	2.79781	1.73561
C	-3.86520	1.96407	2.56473
H	-2.94018	1.35596	2.48519

H	-4.66041	1.35538	3.04381
H	-3.64717	2.83590	3.21781
C	-3.26268	-0.38961	-3.04347
H	-2.33673	0.22120	-3.02844
C	-4.02324	-0.01598	-4.32035
H	-3.37775	-0.17906	-5.20968
H	-4.31054	1.05660	-4.29195
H	-4.94280	-0.62953	-4.43049
C	-2.80686	-1.85846	-3.08777
H	-2.41963	-2.17624	-2.09681
H	-1.99630	-1.98056	-3.83759
H	-3.64150	-2.53445	-3.36941
C	-7.15091	-1.56986	0.00466
H	-7.60047	-1.29004	0.98415
C	-6.71528	-3.03511	0.11549
H	-5.90857	-3.13209	0.86872
H	-6.34503	-3.42257	-0.85699
H	-7.57150	-3.66296	0.44205
C	-8.24560	-1.40368	-1.05509
H	-8.55375	-0.33810	-1.11934
H	-9.13706	-2.00639	-0.77892
H	-7.89055	-1.73531	-2.05396
C	7.03058	-1.97513	0.69936
H	7.04193	-2.60424	1.61797
C	6.84955	-2.92648	-0.48761
H	5.87959	-3.45895	-0.39970
H	7.65888	-3.68731	-0.49327
H	6.87678	-2.37619	-1.45163
C	8.38691	-1.26357	0.63069
H	8.49682	-0.57015	1.49186
H	8.49081	-0.68536	-0.31178
H	9.20966	-2.00855	0.67864
H	-0.30203	-1.68224	1.22745
C	-2.38733	-2.62843	2.26876
C	-1.37716	-3.88911	0.57343
C	-2.36240	-1.47954	3.09986
C	-3.46348	-3.55823	2.38512
C	-2.41375	-4.85237	0.62767
C	-3.37367	-1.27231	4.01059
H	-1.54220	-0.77770	2.99503
C	-4.48951	-3.31219	3.33422
C	-3.44094	-4.68318	1.52552
H	-2.38489	-5.70145	-0.04166
C	-4.44623	-2.19007	4.12894
H	-3.35236	-0.39269	4.64659
H	-5.30922	-4.02050	3.41667
H	-4.24708	-5.41014	1.58035
H	-5.23449	-2.00310	4.85134
N	-1.37602	-2.82597	1.37067
C	-0.17287	-3.96235	-0.35454
C	1.17340	-4.12967	0.43806
H	1.46834	-5.18125	0.41995
H	0.97147	-3.87662	1.48281
N	2.26246	-3.28923	0.00291
H	2.09002	-2.31124	0.19915
C	2.99743	-3.49496	-1.17162
C	3.49652	-2.37662	-1.86238
C	3.32084	-4.77726	-1.64648
C	4.30511	-2.53776	-2.98295
H	3.23326	-1.38279	-1.51203

C	4.12682	-4.92544	-2.77323
H	2.94718	-5.65927	-1.13780
C	4.62981	-3.81298	-3.44922
H	4.67731	-1.65955	-3.50168
H	4.36459	-5.92647	-3.12288
H	5.25739	-3.93709	-4.32608
H	-0.13720	-3.03168	-0.92832
Cl	-0.35807	-5.26741	-1.61554

Catalyst (TRIP, 3)-Product (S) alternative conformation
Electronic energy (Hartrees): -1870.563472
Free Energy at 1M concentration and 253 K (Hartrees): -1869.342667
Coordinates:

P	-0.34835	0.69428	-1.15792
O	-1.76357	-0.08564	-1.37390
O	0.74180	-0.36823	-0.52437
O	-0.58545	1.62174	0.10454
O	0.05473	1.33166	-2.42676
C	-2.06846	-1.09934	-0.49886
C	-3.35656	-1.03633	0.23640
C	-3.68790	-2.01266	1.09337
H	-4.63406	-1.97741	1.62064
C	-2.77076	-3.14653	1.32183
C	-3.08548	-4.14816	2.36619
H	-4.00182	-4.06856	2.93760
C	-2.23174	-5.14593	2.61904
H	-2.45784	-5.87212	3.38898
C	-0.97367	-5.25446	1.85610
H	-0.27923	-6.05466	2.07721
C	-0.68745	-4.36373	0.89988
H	0.24918	-4.45307	0.36598
C	-1.63263	-3.25427	0.59849
C	-1.31238	-2.21953	-0.42907
C	0.86965	-1.51339	-1.26678
C	2.17264	-1.84096	-1.89199
C	2.26510	-2.88156	-2.73340
H	3.22477	-3.15608	-3.15587
C	1.08888	-3.74527	-2.97552
C	1.18592	-4.87253	-3.93075
H	2.10784	-5.05030	-4.47034
C	0.13521	-5.67589	-4.13091
H	0.20648	-6.49879	-4.83036
C	-1.12679	-5.44069	-3.40245
H	-1.97716	-6.08626	-3.58030
C	-1.22318	-4.42956	-2.53130
H	-2.16185	-4.26454	-2.01868
C	-0.05791	-3.53968	-2.28410
C	-0.14632	-2.39387	-1.33283
C	3.39892	-1.21302	-1.35647
C	3.99547	-1.78556	-0.10222
C	3.93551	-0.09692	-1.91731
C	4.98841	-1.11627	0.51111
C	5.08663	0.56305	-1.24519
C	5.56321	0.11392	-0.07500
H	5.40345	-1.49100	1.43669
H	5.52507	1.44390	-1.69548
C	3.39676	0.51977	-3.20524
H	2.45121	0.03041	-3.51884
C	3.07161	2.00791	-3.01791
H	2.49742	2.15001	-2.08121

H	2.45831	2.37494	-3.86837
H	3.99173	2.62656	-2.96305
C	4.39522	0.32127	-4.34957
H	5.35721	0.83340	-4.13408
H	3.97832	0.73038	-5.29463
H	4.59143	-0.76252	-4.49651
C	3.50060	-3.10907	0.48455
H	2.76786	-3.58996	-0.19166
C	2.76983	-2.88453	1.80820
H	1.90830	-2.20433	1.64186
H	3.44861	-2.44238	2.56720
H	2.37588	-3.84682	2.19947
C	4.64004	-4.12529	0.64349
H	5.36032	-3.81048	1.42733
H	5.18138	-4.24249	-0.31963
H	4.22646	-5.11570	0.93086
C	-4.31508	0.06010	-0.03685
C	-5.08948	0.04212	-1.15760
C	-4.47470	1.16800	0.96528
C	-6.08408	1.12756	-1.36453
C	-5.42837	2.09692	0.76641
C	-6.26319	2.09505	-0.45433
H	-6.68639	1.12432	-2.26375
H	-5.58225	2.88070	1.49528
C	-5.00921	-1.07001	-2.20539
H	-4.25589	-1.83663	-1.93450
C	-6.34304	-1.81854	-2.31551
H	-6.64363	-2.20531	-1.31818
H	-6.23718	-2.68356	-3.00473
H	-7.14781	-1.15946	-2.70337
C	-4.57794	-0.51092	-3.56655
H	-3.61734	0.03769	-3.46599
H	-5.34261	0.17868	-3.98156
H	-4.42700	-1.34232	-4.28791
C	-3.58787	1.25141	2.20384
H	-2.76283	0.51907	2.12277
C	-4.37957	0.89925	3.46763
H	-3.70460	0.89303	4.35014
H	-4.82703	-0.11225	3.36757
H	-5.19281	1.63473	3.64559
C	-2.91494	2.62521	2.34498
H	-2.43908	2.92409	1.38811
H	-2.12925	2.57858	3.12804
H	-3.64532	3.40822	2.63786
C	-7.29711	3.18298	-0.68154
H	-7.81901	3.01511	-1.65067
C	-6.63128	4.56139	-0.76595
H	-5.85424	4.56029	-1.56018
H	-6.15736	4.83910	0.19932
H	-7.38772	5.33366	-1.02206
C	-8.36971	3.15628	0.41332
H	-8.83686	2.14941	0.46336
H	-9.16548	3.89664	0.18349
H	-7.93716	3.40126	1.40642
C	6.68623	0.84911	0.63258
H	6.97782	1.75086	0.04811
C	6.23140	1.33906	2.01233
H	5.32328	1.96852	1.91015
H	7.02919	1.95461	2.47979
H	6.00093	0.48757	2.68708

C	7.93369	-0.03430	0.74652
H	8.24456	-0.38483	-0.26092
H	7.74340	-0.91823	1.39139
H	8.77217	0.54761	1.18526
H	0.11310	2.34826	0.22740
C	1.47192	4.32919	-0.76642
C	2.34818	3.49108	1.22019
C	0.41312	4.36404	-1.71028
C	2.61913	5.15960	-0.97780
C	3.50997	4.29307	1.08670
C	0.50080	5.18147	-2.81310
H	-0.44229	3.71906	-1.56477
C	2.67795	5.98791	-2.12838
C	3.64855	5.10729	-0.01087
H	4.27451	4.26049	1.85522
C	1.63814	5.99787	-3.02806
H	-0.30976	5.19282	-3.53465
H	3.55476	6.61089	-2.28163
H	4.53495	5.72299	-0.13837
H	1.68572	6.63045	-3.90905
C	2.21969	2.60590	2.44407
C	2.12966	1.09672	2.14603
H	2.96051	0.86086	1.46609
H	1.21924	0.87159	1.59499
N	2.21329	0.28735	3.34016
N	1.37758	3.49525	0.31672
C	1.22178	-0.58351	3.79302
C	1.57747	-1.59670	4.70470
C	-0.12198	-0.49154	3.38769
C	0.63083	-2.50087	5.17415
H	2.61245	-1.67887	5.03054
C	-1.05940	-1.40289	3.87173
H	-0.43563	0.29458	2.71189
C	-0.69903	-2.41764	4.75787
H	0.93920	-3.27819	5.86793
H	-2.09468	-1.31115	3.55790
H	-1.43956	-3.12258	5.12183
H	3.14833	0.02697	3.61564
H	3.08279	2.77930	3.08954
Cl	0.79694	3.15324	3.45336

Catalyst (TRIP, 3)-Product (R) alternative conformation
Electronic energy (Hartrees): -1870.562333
Free Energy at 1M concentration and 253 K (Hartrees): -1869.342038
Coordinates:

P	-0.65478	-0.26616	-0.09126
O	0.03758	1.17723	-0.43361
O	-2.16305	0.02865	0.45091
O	-0.02167	-0.80761	1.24939
O	-0.52123	-1.12457	-1.28923
C	-0.29685	2.24829	0.36016
C	0.79858	3.00538	1.02073
C	0.52243	4.11318	1.72349
H	1.32333	4.67791	2.18661
C	-0.87356	4.56048	1.89021
C	-1.17350	5.72375	2.75632
H	-0.36896	6.26335	3.24035
C	-2.43915	6.11109	2.94680
H	-2.65772	6.96030	3.58122
C	-3.54304	5.37349	2.30359

H	-4.56656	5.67753	2.48101
C	-3.28213	4.32496	1.51446
H	-4.10895	3.78395	1.07373
C	-1.87676	3.90066	1.26657
C	-1.55837	2.74016	0.37932
C	-2.89463	0.83022	-0.38601
C	-4.09362	0.27234	-1.05929
C	-4.83026	1.05173	-1.86446
H	-5.72507	0.65944	-2.33322
C	-4.43307	2.45170	-2.11552
C	-5.20151	3.27191	-3.08012
H	-6.06745	2.85555	-3.57970
C	-4.82741	4.52910	-3.34095
H	-5.38811	5.12719	-4.04764
C	-3.64088	5.10089	-2.67617
H	-3.32986	6.11104	-2.90950
C	-2.94370	4.37048	-1.79821
H	-2.06273	4.80174	-1.34188
C	-3.36517	2.98043	-1.47362
C	-2.60646	2.14474	-0.49433
C	-4.53508	-1.11006	-0.76182
C	-5.22140	-1.38950	0.54618
C	-4.35408	-2.11609	-1.66241
C	-5.64655	-2.64046	0.80551
C	-4.88720	-3.46818	-1.35039
C	-5.48670	-3.73062	-0.18084
H	-6.13647	-2.86235	1.74384
H	-4.78508	-4.25633	-2.08479
C	-3.62788	-1.91546	-2.99237
H	-3.12819	-0.92498	-3.02861
C	-2.51423	-2.95573	-3.19198
H	-1.90008	-3.05067	-2.27276
H	-1.85028	-2.64325	-4.02621
H	-2.92982	-3.95487	-3.43994
C	-4.62222	-1.95935	-4.15745
H	-5.11830	-2.95129	-4.22108
H	-4.09558	-1.76270	-5.11569
H	-5.40137	-1.17951	-4.02423
C	-5.46187	-0.28731	1.57809
H	-5.08070	0.68554	1.21079
C	-4.71402	-0.57567	2.88641
H	-3.63396	-0.73454	2.68146
H	-5.11892	-1.47727	3.39247
H	-4.81133	0.28862	3.57767
C	-6.96032	-0.07727	1.83282
H	-7.41793	-0.96541	2.31716
H	-7.48391	0.11928	0.87271
H	-7.11303	0.80044	2.49669
C	2.20442	2.57583	0.83654
C	2.83943	2.77068	-0.35336
C	2.92952	1.90660	1.97069
C	4.21196	2.23134	-0.53468
C	4.17730	1.44498	1.76414
C	4.84063	1.57436	0.44966
H	4.70525	2.36292	-1.48799
H	4.72169	0.97284	2.57044
C	2.21205	3.55115	-1.51073
H	1.18311	3.88520	-1.26818
C	3.00938	4.82935	-1.79505
H	3.07099	5.44952	-0.87513

H	2.50243	5.42688	-2.58274
H	4.03892	4.59510	-2.13925
C	2.09715	2.68818	-2.77373
H	1.62019	1.71383	-2.53643
H	3.09332	2.50239	-3.22533
H	1.46880	3.20682	-3.52896
C	2.30099	1.77062	3.35659
H	1.24575	2.10680	3.34111
C	3.02916	2.65306	4.37704
H	2.51223	2.60556	5.35928
H	3.02699	3.70990	4.03451
H	4.08079	2.32291	4.51426
C	2.26430	0.31133	3.82221
H	1.81015	-0.31199	3.02958
H	1.64532	0.21792	4.74001
H	3.28016	-0.07694	4.04537
C	6.21436	0.97184	0.22026
H	6.52616	1.12872	-0.83666
C	6.19344	-0.54298	0.46118
H	5.38470	-1.01307	-0.13513
H	6.02347	-0.77883	1.53312
H	7.16089	-0.99099	0.14946
C	7.26491	1.65355	1.10309
H	7.26895	2.74806	0.91208
H	8.27502	1.25617	0.86655
H	7.05977	1.47801	2.18040
C	-6.00436	-5.12505	0.12238
H	-5.79577	-5.80303	-0.73589
C	-5.29233	-5.71899	1.34323
H	-4.19303	-5.71473	1.18162
H	-5.61600	-6.77048	1.49751
H	-5.52204	-5.14122	2.26348
C	-7.52411	-5.11375	0.32344
H	-8.02223	-4.67495	-0.56754
H	-7.80705	-4.52299	1.22030
H	-7.89774	-6.15188	0.45342
H	0.55300	-1.63684	1.13014
C	2.05263	-3.11432	2.54662
C	2.57472	-3.13150	0.27505
C	1.08869	-2.89850	3.56509
C	3.34846	-3.59829	2.90001
C	3.88187	-3.62024	0.54387
C	1.41281	-3.13868	4.88172
H	0.10830	-2.53044	3.28275
C	3.65204	-3.82773	4.26607
C	4.26656	-3.82642	1.84429
H	4.55796	-3.79239	-0.28232
C	2.70295	-3.60124	5.23729
H	0.67257	-2.97027	5.65778
H	4.64184	-4.18971	4.53024
H	5.26903	-4.17666	2.07548
H	2.93956	-3.78010	6.28160
C	2.07555	-2.90112	-1.13665
H	1.04068	-2.56157	-1.10249
C	2.86094	-1.90240	-1.98953
H	2.94346	-0.98790	-1.38916
H	2.24592	-1.65028	-2.86673
N	4.19080	-2.36784	-2.33409
N	1.71067	-2.85958	1.24388
C	5.10671	-1.52334	-2.97529

C	6.32790	-2.06156	-3.42456
C	4.87635	-0.15014	-3.14959
C	7.28532	-1.24730	-4.01740
H	6.52031	-3.12347	-3.29107
C	5.84888	0.65967	-3.73837
H	3.93640	0.28363	-2.83350
C	7.05826	0.12369	-4.17476
H	8.22032	-1.68621	-4.35459
H	5.64816	1.72043	-3.86071
H	7.81033	0.75787	-4.63312
H	4.17884	-3.30631	-2.71650
Cl	1.99345	-4.53376	-2.00117

Catalyst (TRIP, 3)-Product (R) alternative conformation
Electronic energy (Hartrees): -1870.562319
Free Energy at 1M concentration and 253 K (Hartrees): -1869.341973
Coordinates:

P	0.65221	-0.28329	0.07775
O	-0.05525	1.15606	0.40824
O	2.16185	0.02012	-0.45624
O	0.03359	-0.83876	-1.26396
O	0.51706	-1.13514	1.28034
C	0.28094	2.23059	-0.38043
C	-0.81314	2.98764	-1.04343
C	-0.53602	4.09637	-1.74444
H	-1.33630	4.66199	-2.20770
C	0.85994	4.54638	-1.90431
C	1.16216	5.71094	-2.76802
H	0.35931	6.24793	-3.25776
C	2.42788	6.10264	-2.94923
H	2.64818	6.95270	-3.58198
C	3.52960	5.36909	-2.29761
H	4.55331	5.67717	-2.46687
C	3.26655	4.31951	-1.51056
H	4.09182	3.78190	-1.06280
C	1.86097	3.88973	-1.27392
C	1.54045	2.72757	-0.38985
C	2.88235	0.82641	0.38571
C	4.07859	0.27648	1.07014
C	4.79711	1.05798	1.88943
H	5.69023	0.67178	2.36646
C	4.38552	2.45389	2.14000
C	5.13471	3.27692	3.11712
H	5.99616	2.86507	3.62804
C	4.74878	4.53111	3.37511
H	5.29551	5.13130	4.09091
C	3.56828	5.09677	2.69449
H	3.24747	6.10440	2.92535
C	2.88853	4.36382	1.80508
H	2.01145	4.79044	1.33698
C	3.32360	2.97722	1.48390
C	2.58351	2.13833	0.49337
C	4.54388	-1.09536	0.76196
C	5.26538	-1.34438	-0.53298
C	4.35637	-2.11731	1.64273
C	5.71734	-2.58426	-0.79981
C	4.91896	-3.45599	1.32455
C	5.55194	-3.69121	0.16683
H	6.23262	-2.78446	-1.72940
H	4.81197	-4.25639	2.04489

C	3.59464	-1.94718	2.95676
H	3.07776	-0.96552	2.99367
C	2.49422	-3.00861	3.11440
H	1.90531	-3.10157	2.17842
H	1.80400	-2.71847	3.93527
H	2.92049	-4.00383	3.36001
C	4.56071	-1.99036	4.14530
H	5.07312	-2.97408	4.20708
H	4.00723	-1.81720	5.09290
H	5.32835	-1.19461	4.04227
C	5.50929	-0.22364	-1.54394
H	5.10567	0.73786	-1.17074
C	4.79151	-0.50435	-2.87043
H	3.70981	-0.67983	-2.68856
H	5.21833	-1.39388	-3.37982
H	4.89077	0.37027	-3.54830
C	7.00896	0.01334	-1.76580
H	7.48986	-0.85986	-2.25467
H	7.51059	0.20294	-0.79265
H	7.16075	0.90362	-2.41295
C	-2.21941	2.56295	-0.85298
C	-2.84804	2.76275	0.33941
C	-2.94901	1.88550	-1.97902
C	-4.21483	2.21390	0.53434
C	-4.19043	1.41261	-1.75958
C	-4.84474	1.54418	-0.44064
H	-4.70204	2.34788	1.49040
H	-4.73743	0.93220	-2.55924
C	-2.21848	3.55756	1.48591
H	-1.19463	3.89925	1.23241
C	-3.02516	4.83068	1.76676
H	-3.10024	5.44286	0.84250
H	-2.51722	5.43915	2.54537
H	-4.04958	4.58976	2.12149
C	-2.08545	2.70591	2.75462
H	-1.60096	1.73435	2.52145
H	-3.07625	2.51457	3.21536
H	-1.45629	3.23659	3.50077
C	-2.33362	1.75581	-3.37128
H	-1.28549	2.11351	-3.36990
C	-3.09024	2.61887	-4.38774
H	-2.58148	2.57929	-5.37458
H	-3.10751	3.67666	-4.04841
H	-4.13566	2.26574	-4.51457
C	-2.27296	0.29551	-3.82990
H	-1.78401	-0.31116	-3.04506
H	-1.67397	0.21145	-4.76176
H	-3.28421	-0.11857	-4.02596
C	-6.21177	0.93203	-0.19700
H	-6.51565	1.09080	0.86201
C	-6.18131	-0.58345	-0.43185
H	-5.36315	-1.04405	0.15866
H	-6.01963	-0.82265	-1.50434
H	-7.14203	-1.03790	-0.10887
C	-7.27497	1.60179	-1.07371
H	-7.28639	2.69693	-0.88674
H	-8.27984	1.19707	-0.82745
H	-7.07709	1.42380	-2.15198
C	6.10121	-5.07202	-0.14295
H	5.88241	-5.76613	0.69975

C	5.43129	-5.65975	-1.39036
H	4.32826	-5.67610	-1.25758
H	5.77668	-6.70333	-1.55106
H	5.67499	-5.06488	-2.29600
C	7.62521	-5.03186	-0.30428
H	8.09257	-4.59873	0.60593
H	7.92080	-4.42232	-1.18435
H	8.02003	-6.06133	-0.44034
H	-0.54813	-1.66215	-1.14126
C	-2.07790	-3.11775	-2.55000
C	-2.57923	-3.14289	-0.27399
C	-1.11997	-2.91039	-3.57583
C	-3.38354	-3.58131	-2.89464
C	-3.89443	-3.61466	-0.53383
C	-1.45937	-3.13749	-4.89095
H	-0.13162	-2.55892	-3.30018
C	-3.70305	-3.79674	-4.25933
C	-4.29446	-3.80550	-1.83192
H	-4.56459	-3.78518	0.29747
C	-2.75960	-3.57788	-5.23774
H	-0.72359	-2.97550	-5.67259
H	-4.70052	-4.14223	-4.51671
H	-5.30361	-4.14084	-2.05599
H	-3.00842	-3.74597	-6.28101
C	-2.06560	-2.92644	1.13474
H	-1.02734	-2.59867	1.09366
C	-2.83125	-1.92235	1.99935
H	-2.90562	-1.00331	1.40505
H	-2.20615	-1.68374	2.87310
N	-4.16474	-2.37168	2.35091
N	-1.72071	-2.87520	-1.24894
C	-5.06741	-1.51713	2.99748
C	-6.29449	-2.04068	3.44813
C	-4.81859	-0.14761	3.17557
C	-7.23967	-1.21596	4.04611
H	-6.50135	-3.09943	3.31126
C	-5.77893	0.67276	3.76982
H	-3.87388	0.27497	2.85838
C	-6.99413	0.15134	4.20757
H	-8.17961	-1.64360	4.38404
H	-5.56419	1.73036	3.89557
H	-7.73649	0.79390	4.67006
H	-4.16339	-3.31138	2.73036
Cl	-1.99697	-4.56456	1.99008

Catalyst (TRIP, 3)-Product (S) alternative conformation
Electronic energy (Hartrees): -1870.561919
Free Energy at 1M concentration and 253 K (Hartrees): -1869.34167
Coordinates:

P	0.32907	0.19859	-0.94367
O	1.60682	1.22382	-0.95350
O	-0.87174	0.94652	-0.12061
O	0.67030	-0.94533	0.09166
O	0.02662	-0.22242	-2.32590
C	1.70148	2.11825	0.08564
C	2.90934	2.07303	0.94863
C	3.11234	3.02605	1.86846
H	3.99770	2.99891	2.49288
C	2.10188	4.07592	2.09381
C	2.29928	5.05667	3.18622

H	3.21005	5.04255	3.77199
C	1.35199	5.95980	3.45824
H	1.49592	6.67417	4.25853
C	0.09906	5.97281	2.68086
H	-0.67681	6.68663	2.92595
C	-0.08632	5.09543	1.68794
H	-1.02792	5.10604	1.15657
C	0.97491	4.10690	1.34578
C	0.80049	3.12236	0.23219
C	-1.22540	2.13247	-0.71090
C	-2.56215	2.26377	-1.33631
C	-2.88930	3.39440	-1.97900
H	-3.87913	3.51259	-2.40403
C	-1.91688	4.50444	-2.07417
C	-2.25793	5.71739	-2.85234
H	-3.21969	5.79567	-3.34388
C	-1.37889	6.72061	-2.95276
H	-1.62838	7.60617	-3.52284
C	-0.06189	6.62002	-2.29441
H	0.65131	7.42796	-2.39498
C	0.25419	5.53113	-1.58385
H	1.23165	5.46879	-1.12387
C	-0.72240	4.41858	-1.44126
C	-0.39338	3.19308	-0.65483
C	-3.58183	1.21835	-1.09555
C	-4.23800	1.15314	0.25487
C	-3.90962	0.31071	-2.05571
C	-5.10556	0.15640	0.51116
C	-4.93375	-0.72269	-1.74961
C	-5.48580	-0.82093	-0.53199
H	-5.57097	0.08064	1.48455
H	-5.22801	-1.41794	-2.52471
C	-3.27096	0.30810	-3.44356
H	-2.40402	0.99971	-3.48554
C	-2.72402	-1.07941	-3.81439
H	-2.13086	-1.49970	-2.97674
H	-2.06432	-0.99972	-4.70470
H	-3.54208	-1.78974	-4.05618
C	-4.27709	0.79079	-4.49315
H	-5.15239	0.10899	-4.54939
H	-3.79443	0.83403	-5.49286
H	-4.63474	1.81060	-4.23635
C	-3.96548	2.20873	1.32766
H	-3.26888	2.98316	0.95026
C	-3.29500	1.59295	2.56000
H	-2.37624	1.04644	2.25836
H	-3.98200	0.89329	3.08055
H	-3.00216	2.39293	3.27308
C	-5.24758	2.95849	1.71341
H	-5.97413	2.28715	2.21765
H	-5.72344	3.38741	0.80559
H	-5.00535	3.79366	2.40499
C	3.86363	0.94317	0.84753
C	4.79700	0.89787	-0.14242
C	3.85492	-0.11427	1.91761
C	5.81143	-0.18972	-0.12738
C	4.82334	-1.04862	1.92033
C	5.83991	-1.10813	0.84903
H	6.55680	-0.22366	-0.91120
H	4.85839	-1.78994	2.70672

C	4.88050	1.94642	-1.25137
H	4.07740	2.70478	-1.15755
C	6.20223	2.71964	-1.17484
H	6.31713	3.17408	-0.16741
H	6.20793	3.53765	-1.92665
H	7.07127	2.05725	-1.37155
C	4.69935	1.30206	-2.63156
H	3.75504	0.71784	-2.66026
H	5.54564	0.62736	-2.87763
H	4.64265	2.08959	-3.41304
C	2.78193	-0.14088	3.00590
H	1.97031	0.57375	2.75798
C	3.36583	0.28923	4.35657
H	2.56302	0.32772	5.12352
H	3.81564	1.30075	4.27593
H	4.14872	-0.42201	4.69625
C	2.10848	-1.51833	3.12214
H	1.85181	-1.91041	2.11855
H	1.17285	-1.43437	3.71559
H	2.77172	-2.25380	3.62433
C	6.90670	-2.18850	0.86072
H	7.54044	-2.10551	-0.05100
C	6.28227	-3.58980	0.84459
H	5.58673	-3.69200	-0.01318
H	5.72374	-3.79428	1.78214
H	7.07787	-4.35752	0.73679
C	7.83321	-2.01875	2.06963
H	8.28687	-1.00451	2.06135
H	8.65411	-2.76616	2.02816
H	7.27786	-2.15537	3.02194
C	-6.50322	-1.90729	-0.23471
H	-6.67794	-2.52442	-1.14487
C	-5.98406	-2.85352	0.85196
H	-5.01622	-3.29415	0.53275
H	-6.70494	-3.68290	1.01399
H	-5.83759	-2.31924	1.81461
C	-7.85542	-1.30300	0.16110
H	-8.20773	-0.60778	-0.63073
H	-7.78389	-0.74854	1.12062
H	-8.61160	-2.10860	0.27675
H	0.91419	-1.81505	-0.41549
C	2.35695	-3.42972	-1.80279
C	0.16900	-4.05208	-1.26740
C	3.37726	-2.44469	-1.76781
C	2.57919	-4.65423	-2.50382
C	0.31443	-5.29343	-1.92951
C	4.56645	-2.67324	-2.42187
H	3.19049	-1.51560	-1.24026
C	3.82792	-4.86528	-3.14438
C	1.51564	-5.58866	-2.53175
H	-0.51984	-5.98191	-1.96472
C	4.79913	-3.89185	-3.10639
H	5.34224	-1.91547	-2.41507
H	3.99687	-5.79962	-3.67237
H	1.65340	-6.53464	-3.04873
H	5.74971	-4.05061	-3.60580
C	-1.10011	-3.58163	-0.57100
C	-0.95987	-3.58871	0.97001
H	0.08370	-3.33510	1.19001
H	-1.12899	-4.59875	1.34953

N	-1.84418	-2.65115	1.61793
H	-1.46804	-1.71341	1.66696
C	-2.73940	-2.98340	2.63299
C	-3.05908	-2.02087	3.60803
C	-3.37286	-4.23790	2.70185
C	-3.99852	-2.29147	4.59756
H	-2.54897	-1.06220	3.58906
C	-4.29857	-4.50297	3.70928
H	-3.17907	-4.98742	1.94232
C	-4.62690	-3.53682	4.66113
H	-4.22979	-1.52481	5.33208
H	-4.78145	-5.47611	3.73543
H	-5.35721	-3.74923	5.43531
H	-1.29436	-2.56008	-0.89752
N	1.16430	-3.17876	-1.18849
Cl	-2.57630	-4.50980	-1.09017

Catalyst (TRIP, 3)-Product (R) alternative conformation
Electronic energy (Hartrees): -1870.561532
Free Energy at 1M concentration and 253 K (Hartrees): -1869.341558
Coordinates:

P	-0.08909	0.46972	-0.91215
O	1.14462	-0.28798	-0.10987
O	-1.20255	-0.67113	-1.25904
O	0.31168	1.20757	-2.12563
O	-0.69917	1.29101	0.28395
C	1.44432	-1.55593	-0.54332
C	2.78991	-1.82518	-1.11342
C	3.13734	-3.07590	-1.44760
H	4.12131	-3.27632	-1.85573
C	2.17143	-4.18233	-1.31407
C	2.54200	-5.53825	-1.78104
H	3.53520	-5.72751	-2.16904
C	1.65072	-6.53359	-1.73017
H	1.92156	-7.52357	-2.07378
C	0.28959	-6.28136	-1.22118
H	-0.43571	-7.08469	-1.20821
C	-0.05289	-5.06163	-0.79107
H	-1.06560	-4.89563	-0.44941
C	0.93896	-3.95057	-0.80728
C	0.58665	-2.58093	-0.32189
C	-1.57451	-1.43692	-0.18120
C	-2.97371	-1.36553	0.30832
C	-3.33505	-2.06147	1.39586
H	-4.36332	-2.04299	1.73745
C	-2.35344	-2.91270	2.09780
C	-2.75024	-3.62126	3.33632
H	-3.75088	-3.50370	3.73331
C	-1.87392	-4.40603	3.97206
H	-2.16364	-4.92058	4.87908
C	-0.50845	-4.57197	3.44008
H	0.19923	-5.20088	3.96466
C	-0.14208	-3.94203	2.31823
H	0.86922	-4.06824	1.95600
C	-1.10546	-3.06693	1.59629
C	-0.71425	-2.32948	0.35591
C	-4.00596	-0.67623	-0.50112
C	-4.49226	-1.33492	-1.76346
C	-4.54088	0.51305	-0.10717
C	-5.44012	-0.72119	-2.49645

C	-5.62958	1.11816	-0.91974
C	-6.04913	0.55301	-2.05990
H	-5.79388	-1.17367	-3.41286
H	-6.09160	2.03623	-0.58183
C	-4.07491	1.25883	1.14230
H	-3.16792	0.78778	1.57415
C	-3.68863	2.71024	0.81764
H	-2.96631	2.73769	-0.02488
H	-3.21840	3.18523	1.70350
H	-4.57468	3.31927	0.54409
C	-5.15605	1.21823	2.22708
H	-6.07713	1.74159	1.89318
H	-4.78481	1.70786	3.15279
H	-5.41186	0.16545	2.47117
C	-3.95414	-2.69132	-2.22243
H	-3.21809	-3.08999	-1.49788
C	-3.21821	-2.57362	-3.56317
H	-2.41700	-1.80768	-3.49214
H	-3.91363	-2.29019	-4.38112
H	-2.74555	-3.54483	-3.82345
C	-5.06989	-3.74249	-2.29461
H	-5.80526	-3.50073	-3.09049
H	-5.59977	-3.80023	-1.31969
H	-4.63590	-4.74183	-2.51254
C	3.74916	-0.72043	-1.34619
C	4.56661	-0.28231	-0.34940
C	3.91305	-0.18197	-2.74039
C	5.62191	0.71580	-0.66816
C	4.90899	0.68803	-2.99173
C	5.79781	1.17452	-1.91505
H	6.27740	1.05945	0.12120
H	5.06885	1.05422	-3.99641
C	4.48934	-0.81733	1.07997
H	3.62997	-1.50509	1.20872
C	5.74405	-1.62651	1.42437
H	5.87346	-2.45308	0.69312
H	5.64294	-2.07225	2.43712
H	6.65200	-0.98721	1.40848
C	4.27834	0.32018	2.08667
H	3.39066	0.91819	1.79663
H	5.16042	0.99125	2.13794
H	4.10711	-0.09588	3.10165
C	3.00114	-0.63437	-3.87981
H	2.12739	-1.18282	-3.47082
C	3.74184	-1.59716	-4.81468
H	3.05368	-1.96656	-5.60473
H	4.11567	-2.47332	-4.24449
H	4.60496	-1.09366	-5.30033
C	2.42330	0.55081	-4.67121
H	2.04798	1.33437	-3.98233
H	1.57800	0.20570	-5.30422
H	3.18685	1.00614	-5.33658
C	6.89227	2.18220	-2.21694
H	7.44985	2.42867	-1.28521
C	6.29794	3.49444	-2.74157
H	5.55952	3.89503	-2.01433
H	5.79350	3.34496	-3.71967
H	7.10166	4.25030	-2.87166
C	7.91048	1.60479	-3.20686
H	8.32558	0.65234	-2.81307

H	8.75093	2.31779	-3.34643
H	7.44485	1.41159	-4.19642
C	-7.14746	1.20287	-2.88209
H	-7.47976	2.14698	-2.39422
C	-6.63631	1.57831	-4.27775
H	-5.74401	2.23483	-4.19193
H	-7.42232	2.13256	-4.83381
H	-6.36224	0.67467	-4.86235
C	-8.37638	0.29087	-2.97322
H	-8.72877	0.02480	-1.95364
H	-8.14442	-0.64286	-3.52797
H	-9.20161	0.81698	-3.49896
H	-0.50122	2.30930	0.35213
C	-0.21048	4.81878	-0.09430
C	-0.40575	4.04223	2.10650
C	-0.10751	4.51976	-1.47779
C	-0.19286	6.17856	0.34517
C	-0.38196	5.35989	2.61837
C	-0.00082	5.55107	-2.38417
H	-0.09489	3.48197	-1.80147
C	-0.08485	7.21283	-0.62081
C	-0.28118	6.41340	1.73839
H	-0.45470	5.52044	3.68619
C	0.00698	6.90307	-1.95828
H	0.08269	5.32457	-3.44259
H	-0.07399	8.24634	-0.28593
H	-0.26691	7.43601	2.10631
H	0.09074	7.69589	-2.69519
N	-0.32314	3.79509	0.80396
C	-0.47174	2.78328	2.96115
C	0.89623	2.04846	3.02534
H	1.46977	2.40111	3.88657
H	1.44700	2.34903	2.12651
N	0.76882	0.60991	3.04310
H	0.81541	0.20140	2.11802
C	1.24429	-0.21143	4.06179
C	1.67887	-1.50964	3.73638
C	1.27907	0.18619	5.41008
C	2.12541	-2.38312	4.72120
H	1.68101	-1.80894	2.69221
C	1.74291	-0.69369	6.38711
H	0.91602	1.16750	5.69621
C	2.16565	-1.98255	6.05862
H	2.45619	-3.37880	4.43806
H	1.75951	-0.36645	7.42325
H	2.51812	-2.66146	6.82846
H	-1.18218	2.09282	2.51209
Cl	-1.13772	3.10632	4.62680

Catalyst (TRIP, 3)-Product (R) alternative conformation
Electronic energy (Hartrees): -1870.560715
Free Energy at 1M concentration and 253 K (Hartrees): -1869.341041
Coordinates:

P	0.39927	-0.14833	0.06639
O	-0.33046	1.24319	0.51031
O	1.82974	0.24465	-0.60866
O	-0.31004	-0.69754	-1.23234
O	0.46711	-1.03203	1.25154
C	-0.14058	2.33567	-0.30058
C	-1.33551	3.01261	-0.86919

C	-1.19076	4.13593	-1.58678
H	-2.06106	4.64050	-1.98986
C	0.15250	4.68194	-1.85835
C	0.29993	5.86444	-2.73808
H	-0.57669	6.34843	-3.15049
C	1.51616	6.33735	-3.03013
H	1.62324	7.20022	-3.67470
C	2.71699	5.67538	-2.48623
H	3.69934	6.04840	-2.74591
C	2.59409	4.61000	-1.68613
H	3.48957	4.12568	-1.32031
C	1.24612	4.09063	-1.32533
C	1.08101	2.90869	-0.42526
C	2.58495	1.06978	0.18299
C	3.85060	0.55863	0.76518
C	4.61549	1.36845	1.51175
H	5.55756	1.01239	1.91115
C	4.17987	2.74943	1.80187
C	4.98836	3.59959	2.70580
H	5.91177	3.22158	3.12673
C	4.57998	4.83637	3.00904
H	5.17034	5.45626	3.67136
C	3.31586	5.35513	2.45280
H	2.97921	6.34816	2.72132
C	2.58111	4.59744	1.63038
H	1.64536	4.98936	1.25487
C	3.03784	3.23074	1.25755
C	2.24019	2.36629	0.33597
C	4.31009	-0.81561	0.45432
C	4.89847	-1.09885	-0.89979
C	4.23443	-1.81012	1.38333
C	5.34731	-2.33932	-1.16951
C	4.79160	-3.14829	1.05444
C	5.30637	-3.41354	-0.15408
H	5.76835	-2.56378	-2.14010
H	4.77684	-3.92405	1.80873
C	3.60380	-1.61009	2.76183
H	3.05421	-0.64679	2.81062
C	2.56876	-2.70263	3.07864
H	1.90881	-2.88353	2.20553
H	1.93653	-2.38758	3.93638
H	3.05903	-3.66175	3.34778
C	4.68689	-1.56727	3.84507
H	5.24314	-2.52790	3.88976
H	4.22584	-1.37606	4.83762
H	5.40722	-0.74923	3.63504
C	5.01262	-0.01223	-1.96902
H	4.62366	0.95350	-1.59142
C	4.17538	-0.35857	-3.20751
H	3.12278	-0.55744	-2.91375
H	4.57640	-1.25333	-3.72855
H	4.18054	0.49410	-3.91967
C	6.47672	0.24821	-2.34695
H	6.92944	-0.63334	-2.84764
H	7.06634	0.48731	-1.43603
H	6.54159	1.11497	-3.03901
C	-2.69351	2.46636	-0.63036
C	-3.30397	2.60800	0.58042
C	-3.42729	1.80494	-1.76471
C	-4.69286	2.10462	0.75195

C	-4.69724	1.40100	-1.57432
C	-5.36157	1.53586	-0.26063
H	-5.17159	2.20060	1.71776
H	-5.24804	0.94501	-2.38548
C	-2.63730	3.29086	1.77570
H	-1.61321	3.63638	1.53132
C	-3.41272	4.54691	2.19055
H	-3.51391	5.23336	1.32270
H	-2.86463	5.08342	2.99440
H	-4.42544	4.29245	2.56770
C	-2.49396	2.31953	2.95598
H	-1.95141	1.40436	2.63820
H	-3.48427	2.02249	3.36015
H	-1.91265	2.79804	3.77280
C	-2.76588	1.58170	-3.12404
H	-1.68697	1.83260	-3.07270
C	-3.38737	2.49135	-4.19001
H	-2.84956	2.37002	-5.15450
H	-4.45930	2.24650	-4.34800
H	-3.30659	3.55437	-3.87955
C	-2.83293	0.11006	-3.55845
H	-2.51945	-0.54544	-2.72339
H	-2.15042	-0.06503	-4.41744
H	-3.85959	-0.17919	-3.86653
C	-6.77107	1.01172	-0.05420
H	-7.09911	1.20470	0.99216
C	-6.82613	-0.50614	-0.26661
H	-6.11300	-1.01277	0.41804
H	-7.84642	-0.88456	-0.04337
H	-6.57116	-0.77604	-1.31333
C	-7.76415	1.73238	-0.97307
H	-7.70272	2.83015	-0.81297
H	-8.80063	1.40591	-0.74221
H	-7.55625	1.51287	-2.04165
C	5.84865	-4.79505	-0.47280
H	5.73365	-5.46199	0.41138
C	5.06389	-5.43848	-1.62190
H	3.98140	-5.46871	-1.37302
H	5.41195	-6.48118	-1.78260
H	5.19893	-4.87246	-2.56785
C	7.34622	-4.73499	-0.79507
H	7.89723	-4.26103	0.04533
H	7.53543	-4.15313	-1.72193
H	7.74581	-5.76192	-0.93629
H	-0.92905	-1.48424	-1.09888
C	-1.84275	-3.40487	-2.62286
C	-2.89558	-3.31231	-0.54971
C	-0.81256	-2.94521	-3.48680
C	-2.76221	-4.39290	-3.09892
C	-3.84383	-4.28970	-0.94471
C	-0.71036	-3.44836	-4.76366
H	-0.12397	-2.19185	-3.12129
C	-2.62621	-4.88882	-4.42176
C	-3.77600	-4.82231	-2.20955
H	-4.61596	-4.59679	-0.24743
C	-1.62039	-4.42563	-5.23773
H	0.07812	-3.09143	-5.41921
H	-3.32919	-5.63787	-4.77578
H	-4.49625	-5.56669	-2.53865
H	-1.51993	-4.80694	-6.24915

C	-2.93224	-2.76030	0.85652
H	-3.84699	-3.07731	1.35752
C	-1.68424	-3.22036	1.67391
H	-0.80030	-3.11341	1.04489
H	-1.82690	-4.29338	1.86523
N	-1.41168	-2.50364	2.88590
H	-0.81000	-1.70379	2.71925
C	-2.28857	-2.41627	3.95578
C	-2.15189	-1.35466	4.87438
C	-3.29702	-3.37115	4.19021
C	-2.98826	-1.25910	5.97947
H	-1.38388	-0.60521	4.70120
C	-4.13076	-3.26151	5.30317
H	-3.41968	-4.21141	3.51377
C	-3.98910	-2.20941	6.20680
H	-2.86182	-0.42784	6.66810
H	-4.89978	-4.01326	5.46163
H	-4.64366	-2.12803	7.06872
N	-1.93367	-2.89017	-1.35906
Cl	-3.06139	-0.94616	0.80266

Catalyst (TRIP, 3)-Product (R) alternative conformation
Electronic energy (Hartrees): -1870.562825
Free Energy at 1M concentration and 253 K (Hartrees): -1869.340958
Coordinates:

P	0.34181	0.01088	0.16786
O	-0.56522	1.30914	0.56423
O	1.77714	0.57196	-0.37874
O	-0.23439	-0.56382	-1.18217
O	0.39652	-0.90236	1.32954
C	-0.43939	2.42366	-0.22793
C	-1.65859	2.96625	-0.88298
C	-1.59554	4.12089	-1.56231
H	-2.48635	4.52980	-2.02456
C	-0.30827	4.82298	-1.72505
C	-0.23548	6.03421	-2.57444
H	-1.13096	6.42680	-3.04004
C	0.93679	6.64590	-2.77367
H	0.98957	7.52966	-3.39630
C	2.16494	6.10898	-2.15770
H	3.11452	6.59417	-2.34332
C	2.10868	5.01878	-1.38404
H	3.02621	4.62802	-0.96478
C	0.80618	4.34539	-1.12515
C	0.71380	3.13463	-0.25312
C	2.36785	1.47055	0.47015
C	3.64165	1.10580	1.13888
C	4.25891	1.99777	1.92763
H	5.20585	1.75054	2.39236
C	3.64803	3.31733	2.18444
C	4.29076	4.25071	3.13814
H	5.22385	3.98175	3.61751
C	3.71979	5.42797	3.41461
H	4.18892	6.10853	4.11336
C	2.44047	5.79678	2.77923
H	1.97115	6.74032	3.02640
C	1.85479	4.96336	1.91164
H	0.90404	5.24356	1.47833
C	2.49410	3.66379	1.56782
C	1.86641	2.71879	0.59482

C	4.26608	-0.21484	0.88441
C	4.95847	-0.45737	-0.42818
C	4.25098	-1.19285	1.83371
C	5.55404	-1.64646	-0.64192
C	4.97909	-2.46193	1.57434
C	5.59055	-2.69105	0.40461
H	6.04998	-1.84264	-1.58279
H	5.01755	-3.21415	2.35128
C	3.52341	-1.04082	3.16971
H	2.87455	-0.14021	3.16947
C	2.59049	-2.23102	3.44517
H	1.98939	-2.47317	2.54492
H	1.89489	-1.98174	4.27484
H	3.16166	-3.13718	3.73711
C	4.52975	-0.87202	4.31330
H	5.17440	-1.77121	4.41298
H	3.99287	-0.71119	5.27255
H	5.17733	0.01015	4.12760
C	5.03172	0.62204	-1.50820
H	4.51419	1.54376	-1.17784
C	4.33181	0.17460	-2.79798
H	3.28714	-0.12982	-2.57918
H	4.86337	-0.67730	-3.27138
H	4.30103	1.01490	-3.52397
C	6.48350	1.03488	-1.78398
H	7.06420	0.20026	-2.23027
H	6.97423	1.34931	-0.83797
H	6.50696	1.89368	-2.48847
C	-2.95373	2.25089	-0.76573
C	-3.64933	2.25098	0.40716
C	-3.53150	1.56858	-1.97740
C	-4.96378	1.56004	0.46792
C	-4.74240	0.98500	-1.88228
C	-5.49401	0.96480	-0.60839
H	-5.51599	1.56445	1.39820
H	-5.18720	0.51457	-2.74834
C	-3.17177	2.98654	1.66095
H	-2.18741	3.46997	1.50333
C	-4.13679	4.12032	2.02817
H	-4.25935	4.80806	1.16407
H	-3.72959	4.70485	2.88081
H	-5.13359	3.72670	2.31824
C	-2.99261	2.01799	2.83787
H	-2.31716	1.18501	2.55174
H	-3.96402	1.59239	3.16516
H	-2.53714	2.55009	3.70016
C	-2.79734	1.55858	-3.31967
H	-1.78801	2.00322	-3.21432
C	-3.54635	2.40415	-4.35720
H	-2.95314	2.47151	-5.29411
H	-3.70077	3.43396	-3.97101
H	-4.53575	1.95999	-4.59680
C	-2.56511	0.13457	-3.84438
H	-2.01278	-0.46353	-3.09319
H	-1.95144	0.16756	-4.76992
H	-3.52144	-0.37650	-4.08109
C	-6.85986	0.30482	-0.52494
H	-7.26412	0.39678	0.50863
C	-6.77296	-1.19380	-0.83426
H	-6.06998	-1.68705	-0.13179

H	-6.42741	-1.37110	-1.87459
H	-7.77009	-1.66677	-0.70755
C	-7.85836	0.99950	-1.45765
H	-7.90615	2.08457	-1.22340
H	-8.87299	0.56963	-1.31635
H	-7.56783	0.87418	-2.52223
C	6.33017	-3.99566	0.16837
H	6.27665	-4.63377	1.07931
C	5.68293	-4.79043	-0.96935
H	4.61536	-4.98403	-0.73141
H	6.19301	-5.76989	-1.08944
H	5.74597	-4.23865	-1.93112
C	7.81642	-3.74355	-0.11115
H	8.26441	-3.15340	0.71681
H	7.95939	-3.19184	-1.06423
H	8.35888	-4.71059	-0.18040
H	-0.43103	-1.56135	-1.13198
C	0.62695	-3.82218	-1.74466
C	-1.43198	-4.01187	-0.67346
C	1.66691	-2.99243	-2.24355
C	0.73790	-5.24139	-1.88820
C	-1.38900	-5.42719	-0.76392
C	2.75512	-3.55627	-2.86929
H	1.58248	-1.91940	-2.12124
C	1.87219	-5.78720	-2.54343
C	-0.31330	-6.03281	-1.36698
H	-2.21134	-6.01349	-0.36769
C	2.85709	-4.96014	-3.03075
H	3.54842	-2.91817	-3.24551
H	1.94604	-6.86552	-2.65409
H	-0.26398	-7.11465	-1.45800
H	3.72313	-5.38080	-3.53229
C	-2.60540	-3.35953	0.02569
H	-3.42870	-4.07141	0.09606
C	-2.22817	-2.85051	1.44683
H	-1.32366	-2.23965	1.39182
H	-1.97292	-3.73852	2.03953
N	-3.24338	-2.07861	2.11924
N	-0.45814	-3.24628	-1.14265
C	-4.50072	-2.56895	2.45779
C	-4.76766	-3.94351	2.59908
C	-5.54555	-1.65903	2.71512
C	-6.03815	-4.38070	2.97536
H	-3.98195	-4.67289	2.43139
C	-6.80699	-2.10754	3.08526
H	-5.35402	-0.59434	2.61606
C	-7.06935	-3.47449	3.21705
H	-6.21824	-5.44756	3.07793
H	-7.59402	-1.38113	3.26835
H	-8.05652	-3.82311	3.50274
H	-3.23860	-1.10264	1.85827
Cl	-3.27650	-2.00308	-0.98489

Catalyst (TRIP, 3)-Product (S) alternative conformation
Electronic energy (Hartrees): -1870.562501
Free Energy at 1M concentration and 253 K (Hartrees): -1869.340925
Coordinates:

P	0.28060	0.09982	-0.63923
O	1.59980	1.05997	-0.81704
O	-0.85764	0.97003	0.15508

O	0.63249	-0.93022	0.50083
O	-0.10299	-0.44753	-1.95609
C	1.87581	1.90120	0.23311
C	3.17479	1.76249	0.94325
C	3.50862	2.63868	1.90182
H	4.46375	2.55524	2.40677
C	2.57159	3.70514	2.30208
C	2.91852	4.60065	3.42981
H	3.87348	4.50004	3.93055
C	2.05267	5.53449	3.83739
H	2.30601	6.18709	4.66288
C	0.74262	5.66814	3.17285
H	0.03622	6.40939	3.52374
C	0.42212	4.86771	2.14970
H	-0.55497	4.96718	1.69647
C	1.38821	3.84457	1.66227
C	1.06045	2.94495	0.51437
C	-1.08608	2.19949	-0.40734
C	-2.41174	2.49208	-1.00789
C	-2.64358	3.69418	-1.55603
H	-3.61863	3.92537	-1.96907
C	-1.58085	4.71850	-1.59194
C	-1.82569	6.00844	-2.27726
H	-2.78777	6.20872	-2.73226
C	-0.85885	6.93038	-2.33958
H	-1.03784	7.87209	-2.84220
C	0.45934	6.66210	-1.73351
H	1.24413	7.40401	-1.80591
C	0.68858	5.50225	-1.10692
H	1.66932	5.31775	-0.68902
C	-0.38624	4.47797	-1.00424
C	-0.15810	3.17686	-0.30747
C	-3.51729	1.50940	-0.91390
C	-4.31731	1.43123	0.35557
C	-3.88774	0.76885	-1.99434
C	-5.43194	0.67470	0.37934
C	-5.14880	-0.01553	-1.93388
C	-5.89979	-0.04732	-0.82445
H	-6.02295	0.60834	1.28273
H	-5.48734	-0.53440	-2.82114
C	-3.08452	0.74753	-3.29286
H	-2.10019	1.24328	-3.15821
C	-2.77736	-0.69117	-3.73616
H	-2.38691	-1.28526	-2.88336
H	-2.00925	-0.68344	-4.53879
H	-3.68302	-1.19758	-4.13077
C	-3.82362	1.50911	-4.39833
H	-4.79689	1.02811	-4.63323
H	-3.20691	1.53263	-5.32207
H	-4.01096	2.55649	-4.08075
C	-3.90748	2.20682	1.60692
H	-2.98147	2.78593	1.42427
C	-3.59297	1.26179	2.77291
H	-2.82141	0.52412	2.46604
H	-4.50157	0.71904	3.10779
H	-3.19394	1.84030	3.63331
C	-4.97598	3.23489	2.00025
H	-5.91833	2.73917	2.31510
H	-5.19123	3.90502	1.14045
H	-4.61002	3.85947	2.84308

C	4.11624	0.66948	0.59779
C	4.88893	0.73817	-0.52209
C	4.30389	-0.46639	1.56576
C	5.92147	-0.30775	-0.75162
C	5.29142	-1.35449	1.34844
C	6.13381	-1.28934	0.13622
H	6.53349	-0.25555	-1.64266
H	5.46884	-2.15155	2.05719
C	4.78577	1.87982	-1.53403
H	3.99947	2.60779	-1.25221
C	6.09287	2.67924	-1.59451
H	6.35955	3.04597	-0.58010
H	5.96868	3.56039	-2.25978
H	6.92877	2.06161	-1.98482
C	4.39979	1.35059	-2.92075
H	3.46773	0.74976	-2.85465
H	5.20423	0.71787	-3.35065
H	4.21561	2.19971	-3.61303
C	3.40637	-0.62799	2.79098
H	2.55043	0.07551	2.73088
C	4.17326	-0.29938	4.07738
H	3.49096	-0.35663	4.95224
H	4.58519	0.72991	4.02740
H	5.01212	-1.01064	4.23388
C	2.79326	-2.03526	2.87256
H	2.38732	-2.33952	1.88701
H	1.96516	-2.04400	3.61297
H	3.54510	-2.78912	3.18762
C	7.23156	-2.31298	-0.09311
H	7.72187	-2.12986	-1.07583
C	6.66592	-3.73808	-0.13470
H	5.86627	-3.81090	-0.89913
H	6.24727	-4.03631	0.84942
H	7.46851	-4.45651	-0.40622
C	8.31790	-2.18892	0.98088
H	8.72423	-1.15484	0.99288
H	9.15330	-2.88713	0.75994
H	7.91448	-2.42737	1.98802
C	-7.23638	-0.76895	-0.82328
H	-7.43069	-1.21122	-1.82652
C	-7.23978	-1.92522	0.17921
H	-6.43081	-2.63994	-0.07466
H	-8.20628	-2.47061	0.13105
H	-7.09330	-1.55800	1.21655
C	-8.38382	0.20720	-0.53817
H	-8.36144	1.04439	-1.26836
H	-8.31114	0.62402	0.48874
H	-9.35995	-0.31373	-0.63830
H	0.78379	-1.87103	0.13390
C	2.04190	-3.54140	-1.41000
C	0.15894	-4.35885	-0.30883
C	2.91675	-2.45597	-1.68073
C	2.25407	-4.79296	-2.07077
C	0.31429	-5.63485	-0.90530
C	3.93231	-2.60851	-2.59590
H	2.76235	-1.51218	-1.17206
C	3.32804	-4.92006	-2.98976
C	1.35568	-5.84618	-1.77760
H	-0.38935	-6.42598	-0.66927
C	4.14402	-3.84539	-3.25394

H	4.58477	-1.77218	-2.82113
H	3.48466	-5.87393	-3.48572
H	1.49465	-6.81400	-2.25199
H	4.95765	-3.93983	-3.96622
C	-1.03967	-4.08055	0.57050
C	-1.99917	-3.04680	-0.09549
H	-2.46113	-3.56066	-0.94776
H	-1.41840	-2.22866	-0.52019
N	-3.01442	-2.48157	0.75394
N	0.99972	-3.36275	-0.54643
C	-4.00518	-3.24213	1.37180
C	-4.64368	-2.73653	2.52049
C	-4.43458	-4.48166	0.86265
C	-5.67982	-3.43730	3.12592
H	-4.31798	-1.78455	2.93039
C	-5.47586	-5.17517	1.48000
H	-3.97575	-4.89579	-0.02917
C	-6.10834	-4.66465	2.61252
H	-6.15562	-3.02047	4.00928
H	-5.79377	-6.12711	1.06329
H	-6.91670	-5.21075	3.08785
H	-2.67294	-1.71414	1.31596
H	-1.57730	-5.00848	0.76630
Cl	-0.50248	-3.52793	2.22627

Catalyst (TRIP, 3)-Product (S) alternative conformation
Electronic energy (Hartrees): -1870.561358
Free Energy at 1M concentration and 253 K (Hartrees): -1869.340671
Coordinates:

P	0.29610	-0.59818	-1.18130
O	1.29817	-0.23140	0.07415
O	-0.85340	-1.61720	-0.63088
O	0.96865	-1.15050	-2.37165
O	-0.45571	0.78976	-1.33048
C	1.48344	-1.21534	1.01255
C	2.85716	-1.73108	1.24056
C	3.08637	-2.62522	2.21165
H	4.09016	-2.99649	2.38325
C	1.97230	-3.14349	3.02788
C	2.23278	-4.19619	4.03678
H	3.24272	-4.54754	4.20811
C	1.22212	-4.72077	4.73741
H	1.41315	-5.49167	5.47271
C	-0.15902	-4.26206	4.49855
H	-0.97587	-4.71013	5.04953
C	-0.40433	-3.30706	3.59426
H	-1.42909	-3.00989	3.41779
C	0.71383	-2.68692	2.82979
C	0.46826	-1.61231	1.81847
C	-1.52015	-1.13770	0.46925
C	-2.96644	-0.82803	0.35852
C	-3.61285	-0.28022	1.39769
H	-4.67661	-0.08202	1.33450
C	-2.89194	0.00526	2.65599
C	-3.59972	0.66950	3.77442
H	-4.64261	0.94153	3.67325
C	-2.95298	0.94047	4.91294
H	-3.46997	1.43059	5.72769
C	-1.53156	0.57439	5.06168
H	-1.01045	0.80910	5.98078

C	-0.88628	-0.03882	4.06293
H	0.16073	-0.28214	4.18620
C	-1.59434	-0.35706	2.79404
C	-0.89071	-1.02470	1.65819
C	-3.72330	-1.29833	-0.82446
C	-4.03705	-2.76475	-0.94003
C	-4.11791	-0.44074	-1.80583
C	-4.68148	-3.21037	-2.03474
C	-4.86224	-0.97706	-2.97613
C	-5.12204	-2.28574	-3.10090
H	-4.90524	-4.26318	-2.14061
H	-5.19304	-0.29340	-3.74711
C	-3.82856	1.05807	-1.76631
H	-3.19422	1.32526	-0.89575
C	-3.05939	1.51092	-3.01623
H	-2.20424	0.83159	-3.21435
H	-2.66396	2.53824	-2.86426
H	-3.71271	1.51997	-3.91389
C	-5.13250	1.84882	-1.62224
H	-5.80418	1.68295	-2.49067
H	-4.91120	2.93416	-1.55682
H	-5.66359	1.53810	-0.69771
C	-3.65660	-3.75612	0.16105
H	-3.17779	-3.23467	1.01291
C	-2.63534	-4.78316	-0.34384
H	-1.74666	-4.26329	-0.76061
H	-3.07282	-5.43288	-1.13094
H	-2.29644	-5.42645	0.49622
C	-4.89489	-4.44875	0.74576
H	-5.38337	-5.10761	-0.00251
H	-5.62801	-3.68780	1.08932
H	-4.60457	-5.06955	1.62031
C	3.97782	-1.27309	0.38753
C	4.66679	-0.13868	0.68966
C	4.40584	-2.12286	-0.77632
C	5.81976	0.25413	-0.16273
C	5.48371	-1.75215	-1.49307
C	6.21326	-0.50019	-1.19784
H	6.35909	1.16361	0.06732
H	5.83413	-2.37161	-2.30676
C	4.33363	0.73506	1.89850
H	3.43049	0.37029	2.42965
C	5.47576	0.70576	2.91983
H	5.68307	-0.34229	3.22524
H	5.19043	1.28107	3.82645
H	6.40374	1.14774	2.49940
C	4.02022	2.17128	1.46908
H	3.21845	2.15709	0.70563
H	4.91067	2.67954	1.04469
H	3.66651	2.76238	2.34021
C	3.68382	-3.42620	-1.11564
H	2.70623	-3.46484	-0.59214
C	4.50061	-4.63120	-0.63675
H	3.94478	-5.57243	-0.83510
H	4.68213	-4.56012	0.45641
H	5.48051	-4.67792	-1.15846
C	3.36393	-3.55094	-2.61474
H	2.94591	-2.60159	-3.00517
H	2.61549	-4.35625	-2.77500
H	4.27027	-3.80363	-3.20445

C	7.39114	-0.07430	-2.05533
H	7.80467	0.88971	-1.68180
C	6.95379	0.16064	-3.50561
H	6.13416	0.90903	-3.53790
H	6.59880	-0.78057	-3.97630
H	7.80532	0.55391	-4.10082
C	8.52407	-1.10433	-1.98194
H	8.82138	-1.26776	-0.92392
H	9.41130	-0.73299	-2.53793
H	8.21443	-2.07569	-2.42221
C	-5.86905	-2.80958	-4.31416
H	-6.11613	-1.96865	-5.00089
C	-5.00178	-3.79349	-5.10781
H	-4.04260	-3.31050	-5.39282
H	-5.52684	-4.09751	-6.03846
H	-4.78179	-4.70564	-4.51365
C	-7.19737	-3.45511	-3.90293
H	-7.80940	-2.73018	-3.32463
H	-7.02966	-4.35985	-3.28106
H	-7.77194	-3.75059	-4.80667
H	0.15504	1.60085	-1.20836
C	2.15180	3.07561	-1.83202
C	0.93276	3.67808	0.06609
C	2.36495	2.15251	-2.88860
C	3.05479	4.16986	-1.65620
C	1.77748	4.79543	0.29909
C	3.44168	2.32045	-3.73053
H	1.70039	1.30267	-3.00377
C	4.14487	4.31748	-2.55092
C	2.82013	5.03814	-0.56094
H	1.61868	5.42349	1.16796
C	4.33408	3.40790	-3.56667
H	3.61397	1.60216	-4.52578
H	4.83000	5.14935	-2.41310
H	3.48776	5.87983	-0.39702
H	5.17634	3.51591	-4.24311
N	1.10765	2.87566	-0.97054
C	-0.16058	3.29753	1.04387
H	-0.74470	2.47754	0.62782
C	-1.08826	4.43656	1.49712
H	-0.48920	5.29414	1.82381
H	-1.64094	4.09836	2.37634
N	-2.01516	4.82740	0.45356
H	-1.76437	5.66746	-0.04653
C	-3.39574	4.63650	0.58461
C	-4.28297	5.50761	-0.07267
C	-3.93207	3.58384	1.34651
C	-5.65928	5.34248	0.04801
H	-3.88431	6.32336	-0.67163
C	-5.31279	3.43842	1.47242
H	-3.27527	2.86745	1.82675
C	-6.18844	4.31303	0.82983
H	-6.32255	6.03153	-0.46711
H	-5.70436	2.61804	2.06663
H	-7.26202	4.18913	0.92628
Cl	0.64841	2.60932	2.55316

Catalyst (TRIP, 3)-Product (S) alternative conformation
Electronic energy (Hartrees): -1870.562206
Free Energy at 1M concentration and 253 K (Hartrees): -1869.340525

Coordinates:

P	0.34174	0.14014	-1.51540
O	1.24423	1.47935	-1.32024
O	-1.09232	0.37899	-0.74405
O	0.97266	-0.90960	-0.51089
O	0.27512	-0.22837	-2.94209
C	1.14377	2.09543	-0.09839
C	2.35109	2.18270	0.76358
C	2.28252	2.81890	1.94194
H	3.16398	2.90678	2.56522
C	1.00152	3.38145	2.41455
C	0.91292	3.96206	3.77404
H	1.79442	4.01661	4.40064
C	-0.25816	4.41032	4.23852
H	-0.32340	4.82769	5.23496
C	-1.46861	4.32338	3.39995
H	-2.41788	4.66632	3.79077
C	-1.39701	3.81278	2.16553
H	-2.30280	3.73837	1.57909
C	-0.09661	3.33011	1.62505
C	0.00333	2.72530	0.26335
C	-1.70673	1.56626	-1.04739
C	-3.05966	1.54445	-1.64780
C	-3.63516	2.69057	-2.03972
H	-4.64410	2.68562	-2.43572
C	-2.94611	3.97998	-1.81383
C	-3.56366	5.23935	-2.28850
H	-4.50674	5.21595	-2.82024
C	-2.95905	6.41101	-2.06227
H	-3.41042	7.33101	-2.41065
C	-1.67199	6.45366	-1.34092
H	-1.18204	7.40418	-1.17363
C	-1.10844	5.32230	-0.90146
H	-0.15747	5.37286	-0.38745
C	-1.77763	4.01423	-1.12880
C	-1.16264	2.73790	-0.66064
C	-3.86241	0.30717	-1.54388
C	-4.55894	0.02208	-0.24338
C	-3.89833	-0.60704	-2.54955
C	-5.14481	-1.17614	-0.06693
C	-4.64351	-1.87723	-2.34066
C	-5.20168	-2.17283	-1.15775
H	-5.61258	-1.41660	0.87792
H	-4.70751	-2.58984	-3.15240
C	-3.18946	-0.39228	-3.88410
H	-2.52727	0.49777	-3.84685
C	-2.28682	-1.58386	-4.23989
H	-1.67721	-1.88415	-3.36151
H	-1.59984	-1.30395	-5.06689
H	-2.88153	-2.46122	-4.57004
C	-4.21321	-0.13490	-4.99385
H	-4.88532	-1.00937	-5.12735
H	-3.69238	0.06415	-5.95478
H	-4.82907	0.75486	-4.74139
C	-4.59768	1.05473	0.88443
H	-4.17126	2.01987	0.54824
C	-3.74314	0.60209	2.06903
H	-2.70155	0.43715	1.72481
H	-4.13579	-0.33783	2.51064
H	-3.72612	1.38708	2.85507

C	-6.03424	1.37095	1.32269
H	-6.50839	0.50119	1.82366
H	-6.64564	1.65834	0.44067
H	-6.03310	2.22302	2.03580
C	3.63774	1.59748	0.30893
C	4.36669	2.19790	-0.67432
C	4.16133	0.34863	0.96933
C	5.65220	1.58328	-1.09903
C	5.35283	-0.14419	0.58300
C	6.12826	0.47793	-0.51035
H	6.21751	2.04557	-1.89775
H	5.75874	-1.02235	1.06585
C	3.94590	3.50963	-1.34019
H	2.98103	3.88144	-0.94149
C	4.96741	4.61738	-1.05569
H	5.10274	4.73513	0.04089
H	4.60401	5.58406	-1.46551
H	5.95150	4.38960	-1.51661
C	3.74498	3.32465	-2.84992
H	3.03227	2.49574	-3.04394
H	4.70346	3.09760	-3.36177
H	3.32442	4.25282	-3.29255
C	3.38858	-0.35523	2.08334
H	2.36622	0.05971	2.14848
C	4.05846	-0.12416	3.44233
H	3.44451	-0.57611	4.25056
H	4.15154	0.96297	3.64555
H	5.07198	-0.57839	3.46773
C	3.21617	-1.85892	1.81645
H	2.89296	-2.02584	0.77234
H	2.44372	-2.27989	2.49504
H	4.16207	-2.41577	1.98307
C	7.44011	-0.13394	-0.96750
H	7.87865	0.47641	-1.78903
C	7.21726	-1.54303	-1.52843
H	6.48544	-1.50573	-2.36314
H	6.83502	-2.23261	-0.74603
H	8.17166	-1.95162	-1.92355
C	8.46578	-0.15065	0.17176
H	8.60363	0.87563	0.57454
H	9.44692	-0.50933	-0.20629
H	8.14138	-0.81991	0.99640
C	-5.89311	-3.50666	-0.94334
H	-5.84070	-4.11600	-1.87374
C	-5.19284	-4.31158	0.15791
H	-4.12243	-4.45421	-0.09999
H	-5.66186	-5.31397	0.25381
H	-5.25888	-3.79551	1.13917
C	-7.37882	-3.30967	-0.62079
H	-7.86657	-2.71541	-1.42293
H	-7.51442	-2.78456	0.34833
H	-7.88813	-4.29516	-0.56095
H	0.80527	-1.88642	-0.74077
C	2.18485	-3.76006	-1.25691
C	0.45303	-4.30084	0.21809
C	2.65947	-2.94366	-2.31739
C	3.02303	-4.79024	-0.73405
C	1.22047	-5.35116	0.78015
C	3.92612	-3.14488	-2.81779
H	2.01025	-2.17599	-2.72667

C	4.32484	-4.96608	-1.27126
C	2.49580	-5.57508	0.31761
H	0.80225	-5.95641	1.57185
C	4.76770	-4.15581	-2.29078
H	4.28636	-2.51968	-3.62887
H	4.96048	-5.74733	-0.86382
H	3.10640	-6.36117	0.75409
H	5.76531	-4.28858	-2.69794
C	-1.00880	-4.03945	0.59505
C	-1.37852	-2.58037	0.92883
H	-2.41693	-2.57093	1.27085
H	-1.36600	-2.02483	-0.01485
N	-0.51655	-1.92372	1.89645
N	0.93497	-3.52259	-0.74398
C	-1.02719	-1.34815	3.06728
C	-0.52489	-0.10891	3.50799
C	-2.02272	-1.97409	3.83782
C	-1.00018	0.48186	4.67441
H	0.21905	0.40685	2.90901
C	-2.50385	-1.36465	4.99559
H	-2.40553	-2.94313	3.53798
C	-1.99991	-0.13695	5.42734
H	-0.59713	1.44158	4.98578
H	-3.27774	-1.86550	5.57096
H	-2.37774	0.32828	6.33220
H	0.15515	-1.32397	1.43268
H	-1.61433	-4.33989	-0.26530
Cl	-1.58065	-5.14913	1.93231

Catalyst (TRIP, 3)-Product (R) alternative conformation
Electronic energy (Hartrees): -1870.560215
Free Energy at 1M concentration and 253 K (Hartrees): -1869.340392
Coordinates:

P	-0.51778	0.02532	-0.35943
O	-1.97826	0.13132	0.37511
O	-0.06600	1.54484	-0.74473
O	-0.44672	-0.84749	-1.55079
O	0.37758	-0.36997	0.87510
C	-2.87270	0.96822	-0.24117
C	-4.13083	0.39861	-0.78394
C	-5.05040	1.20806	-1.32896
H	-5.97749	0.79851	-1.71229
C	-4.80053	2.66001	-1.43086
C	-5.78261	3.52741	-2.12138
H	-6.69635	3.10439	-2.52000
C	-5.54368	4.83601	-2.25898
H	-6.26089	5.46884	-2.76554
C	-4.29487	5.41933	-1.73280
H	-4.09717	6.47490	-1.86790
C	-3.40372	4.64548	-1.10244
H	-2.48397	5.09014	-0.74643
C	-3.66886	3.19304	-0.91386
C	-2.69367	2.30832	-0.20758
C	-0.28247	2.47836	0.23822
C	0.88658	3.15560	0.85523
C	0.69364	4.13064	1.75527
H	1.54017	4.66058	2.17443
C	-0.67107	4.48257	2.19473
C	-0.85974	5.48640	3.26731
H	-0.00284	5.98731	3.70043

C	-2.08842	5.77569	3.70859
H	-2.22521	6.50767	4.49406
C	-3.26076	5.08693	3.13650
H	-4.25030	5.30745	3.51565
C	-3.10173	4.18322	2.16270
H	-3.97382	3.67220	1.77758
C	-1.74369	3.87406	1.63684
C	-1.53869	2.87691	0.54228
C	2.25924	2.81812	0.40620
C	2.72432	3.31443	-0.93492
C	3.10702	2.09589	1.19553
C	3.97138	3.01763	-1.34397
C	4.47907	1.80424	0.70010
C	4.89231	2.22449	-0.50384
H	4.32759	3.37747	-2.29953
H	5.16149	1.24997	1.33063
C	2.72656	1.61222	2.59732
H	1.63549	1.71718	2.77069
C	3.04221	0.12289	2.80732
H	2.66904	-0.47803	1.95844
H	2.54615	-0.24199	3.73215
H	4.13294	-0.05495	2.91103
C	3.43772	2.45687	3.66044
H	4.54037	2.34278	3.58548
H	3.11532	2.14267	4.67615
H	3.18384	3.52975	3.53413
C	1.83394	4.18274	-1.82461
H	0.84777	4.35167	-1.35002
C	1.55264	3.50078	-3.17036
H	1.13407	2.48555	-3.00488
H	2.47698	3.41120	-3.77896
H	0.80934	4.09178	-3.74697
C	2.44118	5.57693	-2.02915
H	3.38900	5.52602	-2.60526
H	2.64350	6.05014	-1.04439
H	1.72860	6.22369	-2.58444
C	-4.38554	-1.05879	-0.69446
C	-4.72593	-1.63718	0.49195
C	-4.30288	-1.89782	-1.93978
C	-5.00522	-3.09672	0.52924
C	-4.61098	-3.20639	-1.86665
C	-4.96254	-3.84604	-0.58078
H	-5.25924	-3.55945	1.47406
H	-4.58691	-3.81955	-2.75700
C	-4.87252	-0.84593	1.79314
H	-4.65202	0.23014	1.64578
C	-6.31431	-0.90807	2.31140
H	-7.01445	-0.54878	1.52695
H	-6.42647	-0.25322	3.20194
H	-6.59760	-1.94213	2.59968
C	-3.88531	-1.34551	2.85641
H	-2.85035	-1.32802	2.45589
H	-4.12463	-2.38060	3.17828
H	-3.92169	-0.68467	3.74869
C	-3.89148	-1.29739	-3.28341
H	-3.53306	-0.25706	-3.14640
C	-5.08755	-1.23551	-4.24051
H	-4.79376	-0.73038	-5.18536
H	-5.91518	-0.65539	-3.78040
H	-5.45785	-2.25440	-4.48286

C	-2.72175	-2.06206	-3.92281
H	-1.90868	-2.21323	-3.18396
H	-2.31321	-1.48174	-4.77759
H	-3.04321	-3.05461	-4.30252
C	-5.25650	-5.33383	-0.51589
H	-5.49811	-5.62849	0.53036
C	-4.03038	-6.15169	-0.93798
H	-3.15588	-5.87766	-0.30957
H	-3.77580	-5.97360	-2.00427
H	-4.23068	-7.23563	-0.79971
C	-6.47703	-5.68944	-1.37254
H	-7.35272	-5.08267	-1.05710
H	-6.73338	-6.76242	-1.24135
H	-6.28072	-5.50377	-2.44972
C	6.29818	1.91898	-0.98694
H	6.80556	1.23806	-0.26771
C	6.27499	1.20488	-2.34424
H	5.56727	0.35131	-2.31617
H	7.28717	0.81571	-2.58474
H	5.96272	1.89319	-3.15762
C	7.13490	3.20035	-1.05946
H	7.16160	3.69310	-0.06397
H	6.71258	3.91242	-1.80025
H	8.17795	2.95730	-1.35483
H	0.64840	-1.37147	0.90788
C	0.88894	-3.30834	2.42548
C	1.93809	-3.49601	0.34253
C	0.04638	-2.56472	3.29142
C	1.43509	-4.55046	2.86645
C	2.53492	-4.72630	0.71680
C	-0.24473	-3.05265	4.54562
H	-0.35468	-1.61925	2.94366
C	1.11526	-5.02192	4.16580
C	2.28096	-5.24249	1.96420
H	3.17603	-5.24004	0.01343
C	0.29026	-4.28773	4.98752
H	-0.89358	-2.48496	5.20559
H	1.53094	-5.96928	4.49769
H	2.72796	-6.18460	2.27052
H	0.04644	-4.65294	5.98033
N	1.15942	-2.82033	1.17525
C	2.17920	-2.81940	-0.99854
C	3.25066	-1.70927	-0.90790
H	2.75865	-0.85789	-0.42249
H	3.50609	-1.38535	-1.91969
N	4.44926	-2.07327	-0.16855
H	4.44635	-1.73094	0.78126
C	5.73212	-2.05787	-0.72935
C	6.82511	-1.67698	0.07008
C	5.97404	-2.44140	-2.05968
C	8.11688	-1.67008	-0.44719
H	6.64990	-1.36762	1.09856
C	7.27098	-2.41508	-2.57023
H	5.15269	-2.77345	-2.68449
C	8.35182	-2.03211	-1.77506
H	8.94222	-1.36386	0.18962
H	7.43499	-2.70833	-3.60347
H	9.35810	-2.01668	-2.18126
H	1.24686	-2.36469	-1.34533
Cl	2.60776	-4.04296	-2.28588

Catalyst (TRIP, 3)-Product (R) alternative conformation
Electronic energy (Hartrees): -1870.560913
Free Energy at 1M concentration and 253 K (Hartrees): -1869.339825
Coordinates:

P	0.57336	0.03381	-0.93052
O	-0.22722	0.92973	0.18933
O	2.16451	0.36356	-0.79717
O	0.13754	0.24735	-2.32609
O	0.38590	-1.41322	-0.31996
C	0.09331	2.26167	0.14135
C	-0.93508	3.25141	-0.26756
C	-0.63106	4.55705	-0.29842
H	-1.38680	5.28641	-0.56457
C	0.73298	5.01320	0.03655
C	1.07897	6.44799	-0.08651
H	0.32750	7.16432	-0.39462
C	2.32179	6.86467	0.17863
H	2.57453	7.91303	0.08611
C	3.35630	5.89467	0.58568
H	4.36584	6.23536	0.77631
C	3.05239	4.59725	0.70662
H	3.83316	3.90094	0.98219
C	1.66667	4.12184	0.44374
C	1.30099	2.67887	0.57114
C	2.65924	0.62536	0.45919
C	3.83794	-0.14866	0.92936
C	4.36692	0.10068	2.13661
H	5.24850	-0.43599	2.46714
C	3.77676	1.13122	3.01151
C	4.30935	1.32935	4.37920
H	5.13632	0.72664	4.73363
C	3.76988	2.25208	5.18304
H	4.15835	2.39538	6.18305
C	2.63630	3.07113	4.71277
H	2.19134	3.80232	5.37528
C	2.15258	2.90414	3.47643
H	1.30726	3.50077	3.15969
C	2.75963	1.89983	2.56074
C	2.24031	1.69698	1.17478
C	4.52626	-1.08378	0.00847
C	5.35868	-0.52053	-1.11119
C	4.45040	-2.43212	0.18514
C	5.99851	-1.36826	-1.93868
C	5.21531	-3.32361	-0.72654
C	5.93703	-2.83235	-1.74301
H	6.59737	-0.98234	-2.75240
H	5.18764	-4.39288	-0.56333
C	3.61589	-3.07678	1.29100
H	2.99450	-2.32405	1.81905
C	2.63090	-4.10553	0.72073
H	2.02798	-3.64780	-0.09165
H	1.94469	-4.45107	1.52217
H	3.15507	-4.99628	0.31696
C	4.52388	-3.72210	2.34358
H	5.12013	-4.55002	1.90460
H	3.91186	-4.12910	3.17671
H	5.21932	-2.96468	2.76318
C	5.52259	0.98674	-1.31285
H	4.96326	1.55092	-0.54173

C	4.95895	1.43440	-2.66792
H	3.90356	1.10458	-2.77092
H	5.54891	1.01372	-3.50926
H	4.98334	2.54271	-2.74147
C	6.98767	1.41510	-1.15617
H	7.62259	0.99247	-1.96305
H	7.37911	1.07396	-0.17383
H	7.06534	2.52281	-1.19419
C	-2.31848	2.80657	-0.54667
C	-3.16694	2.51025	0.47679
C	-2.79575	2.71260	-1.96952
C	-4.52978	2.00965	0.16065
C	-4.05353	2.29500	-2.20693
C	-4.95684	1.89807	-1.10511
H	-5.19046	1.73692	0.97318
H	-4.43039	2.25474	-3.21920
C	-2.79554	2.71967	1.94722
H	-1.76712	3.11939	2.05780
C	-3.71778	3.75650	2.59972
H	-3.68782	4.70597	2.02332
H	-3.37766	3.96953	3.63578
H	-4.76680	3.39518	2.64297
C	-2.82353	1.39937	2.72212
H	-2.16977	0.65680	2.22165
H	-3.85407	0.99408	2.79013
H	-2.44130	1.55511	3.75349
C	-1.91839	3.16589	-3.13632
H	-0.87010	3.29354	-2.79738
C	-2.39010	4.52579	-3.66365
H	-1.70746	4.88274	-4.46417
H	-2.38645	5.27630	-2.84541
H	-3.41889	4.45555	-4.07737
C	-1.86944	2.13580	-4.27571
H	-1.66557	1.12533	-3.87444
H	-1.05696	2.39791	-4.98661
H	-2.82377	2.10560	-4.84212
C	-6.35191	1.38089	-1.40461
H	-6.87990	1.13798	-0.45544
C	-6.28740	0.08928	-2.22756
H	-5.65470	-0.66229	-1.70903
H	-5.86307	0.27832	-3.23633
H	-7.30579	-0.33766	-2.34723
C	-7.19240	2.44619	-2.11775
H	-7.21871	3.37759	-1.51255
H	-8.23535	2.08472	-2.24346
H	-6.77899	2.68138	-3.12119
C	6.69677	-3.76553	-2.66854
H	6.52928	-4.82320	-2.36341
C	6.19538	-3.63309	-4.11114
H	5.10026	-3.81652	-4.15136
H	6.69672	-4.38438	-4.75801
H	6.40519	-2.62149	-4.51867
C	8.20617	-3.51057	-2.58510
H	8.55004	-3.60492	-1.53278
H	8.46429	-2.49667	-2.95761
H	8.75235	-4.26008	-3.19676
H	-0.48689	-1.87299	-0.59244
C	-2.74269	-2.64073	-1.82413
C	-2.13964	-3.66628	0.18448
C	-2.47297	-1.66996	-2.82276

C	-3.89979	-3.47295	-1.94372
C	-3.25727	-4.53306	0.13136
C	-3.33225	-1.53813	-3.89188
H	-1.59069	-1.04231	-2.73243
C	-4.75833	-3.30929	-3.06100
C	-4.12569	-4.43208	-0.92727
H	-3.41243	-5.25518	0.92212
C	-4.47911	-2.35928	-4.01637
H	-3.12684	-0.79301	-4.65393
H	-5.63647	-3.94346	-3.14516
H	-4.99532	-5.08050	-0.99083
H	-5.14030	-2.23226	-4.86809
N	-1.88708	-2.76753	-0.75981
C	-1.16001	-3.63720	1.34230
C	-1.46682	-2.56818	2.42622
H	-1.53637	-1.60783	1.90922
H	-0.58223	-2.51191	3.06846
N	-2.61507	-2.77885	3.26497
H	-2.50461	-3.51522	3.94650
C	-3.93639	-2.46620	2.95363
C	-4.95537	-2.93986	3.80474
C	-4.30410	-1.66665	1.85663
C	-6.28793	-2.62130	3.56978
H	-4.68728	-3.55701	4.65940
C	-5.64438	-1.35339	1.63631
H	-3.55477	-1.29071	1.16949
C	-6.64834	-1.82214	2.48202
H	-7.05004	-3.00150	4.24454
H	-5.90213	-0.73810	0.78160
H	-7.68825	-1.57294	2.29745
H	-0.17662	-3.40193	0.94266
Cl	-0.97515	-5.27176	2.13575

Catalyst (TRIP, 3)-Product (R) alternative conformation
Electronic energy (Hartrees): -1870.560954
Free Energy at 1M concentration and 253 K (Hartrees): -1869.339673
Coordinates:

P	-0.14092	0.07715	0.15979
O	1.01549	-1.04218	0.40125
O	-1.43263	-0.69353	-0.49240
O	0.31364	0.93566	-1.08346
O	-0.39638	0.80012	1.42398
C	1.12454	-2.03961	-0.53608
C	2.42150	-2.21183	-1.23958
C	2.59747	-3.24338	-2.07810
H	3.54972	-3.38402	-2.57594
C	1.48891	-4.17737	-2.35501
C	1.66597	-5.24875	-3.36221
H	2.61649	-5.36912	-3.86701
C	0.65208	-6.06998	-3.65516
H	0.78170	-6.85157	-4.39257
C	-0.65131	-5.90437	-2.98425
H	-1.47480	-6.55736	-3.24312
C	-0.81982	-4.94312	-2.06889
H	-1.79148	-4.82472	-1.60818
C	0.30821	-4.04103	-1.70827
C	0.15014	-2.96838	-0.67948
C	-1.81290	-1.80227	0.21907
C	-3.12232	-1.80439	0.91668
C	-3.51296	-2.89075	1.59871

H	-4.48330	-2.91164	2.08046
C	-2.62803	-4.06848	1.69879
C	-3.03358	-5.22264	2.53369
H	-3.98870	-5.21601	3.04399
C	-2.22057	-6.27636	2.66405
H	-2.51664	-7.11845	3.27621
C	-0.91097	-6.28858	1.98496
H	-0.24752	-7.13396	2.11427
C	-0.53803	-5.25382	1.22301
H	0.43737	-5.27390	0.75575
C	-1.44501	-4.08752	1.04168
C	-1.05516	-2.92061	0.19341
C	-4.04033	-0.65007	0.78038
C	-4.83249	-0.49572	-0.48738
C	-4.20919	0.23766	1.79967
C	-5.70171	0.52769	-0.59269
C	-5.23008	1.30833	1.66127
C	-5.93662	1.45929	0.53270
H	-6.27363	0.66107	-1.50091
H	-5.41005	1.97378	2.49538
C	-3.40562	0.16898	3.09803
H	-2.55339	-0.53580	2.99959
C	-2.78856	1.53095	3.45655
H	-2.32794	2.00013	2.56262
H	-2.00049	1.39739	4.22842
H	-3.55102	2.22906	3.86156
C	-4.28360	-0.34180	4.24537
H	-5.13181	0.34991	4.43585
H	-3.68168	-0.43194	5.17480
H	-4.69116	-1.34451	3.99878
C	-4.69951	-1.49165	-1.63999
H	-3.96738	-2.28479	-1.39163
C	-4.17760	-0.81325	-2.91207
H	-3.22508	-0.28431	-2.69763
H	-4.91523	-0.08653	-3.31210
H	-3.97928	-1.57634	-3.69472
C	-6.02565	-2.21626	-1.90532
H	-6.80336	-1.51526	-2.27507
H	-6.38846	-2.69635	-0.97123
H	-5.87867	-3.01028	-2.66843
C	3.53683	-1.27012	-0.97969
C	4.22748	-1.31659	0.19465
C	3.92731	-0.27549	-2.03967
C	5.33158	-0.34837	0.42209
C	4.95982	0.55707	-1.80297
C	5.69453	0.53321	-0.51897
H	5.86911	-0.37545	1.36109
H	5.27664	1.25785	-2.56304
C	3.96125	-2.36622	1.27595
H	3.12984	-3.04336	0.99509
C	5.18509	-3.26917	1.47182
H	5.47323	-3.73194	0.50360
H	4.94529	-4.08455	2.18745
H	6.05063	-2.69843	1.86913
C	3.55156	-1.70781	2.59814
H	2.67425	-1.04875	2.43774
H	4.37966	-1.10460	3.02485
H	3.26727	-2.48505	3.33911
C	3.22231	-0.23195	-3.39668
H	2.34042	-0.90269	-3.39754

C	4.15520	-0.72654	-4.50883
H	3.60529	-0.77938	-5.47272
H	4.52997	-1.74401	-4.26820
H	5.02406	-0.04590	-4.63378
C	2.67677	1.16422	-3.72732
H	2.01955	1.52029	-2.91042
H	2.07196	1.12330	-4.65837
H	3.49484	1.89877	-3.87903
C	6.86510	1.47066	-0.27959
H	7.29140	1.29543	0.73408
C	6.41806	2.93490	-0.32528
H	5.61259	3.10699	0.41859
H	6.04521	3.20968	-1.33452
H	7.26994	3.60093	-0.07095
C	7.99019	1.20842	-1.28733
H	8.29771	0.14157	-1.24491
H	8.87456	1.83401	-1.04086
H	7.66708	1.44815	-2.32248
C	-6.98430	2.55276	0.42486
H	-7.04361	3.11871	1.38197
C	-6.60987	3.56118	-0.66470
H	-5.61517	4.00185	-0.44114
H	-7.35362	4.38576	-0.69403
H	-6.57734	3.07829	-1.66430
C	-8.37542	1.95962	0.17245
H	-8.62246	1.21920	0.96318
H	-8.42654	1.45904	-0.81750
H	-9.14145	2.76380	0.19928
H	0.19159	1.93531	-0.96201
C	-1.45629	3.84986	-1.48086
C	0.49593	4.57425	-0.44274
C	-2.25246	2.77969	-1.97120
C	-1.93766	5.19158	-1.61404
C	0.08069	5.92762	-0.52127
C	-3.45703	3.04482	-2.58175
H	-1.89425	1.76343	-1.86027
C	-3.18334	5.42629	-2.25171
C	-1.12579	6.23082	-1.10330
H	0.72439	6.70792	-0.12982
C	-3.92343	4.37345	-2.73478
H	-4.06252	2.22602	-2.95476
H	-3.53610	6.44879	-2.35360
H	-1.46191	7.26113	-1.18323
H	-4.87423	4.55485	-3.22620
C	1.84025	4.26052	0.18465
H	2.31177	5.20217	0.47703
C	1.74089	3.34167	1.39970
H	1.23772	2.41228	1.13158
H	1.06746	3.85490	2.11300
N	3.03947	3.05517	1.96303
N	-0.24620	3.57734	-0.90102
C	3.18106	2.27737	3.11317
C	2.12084	1.52337	3.64790
C	4.43864	2.22296	3.74479
C	2.33022	0.74619	4.78787
H	1.14990	1.50854	3.16537
C	4.62783	1.44493	4.88114
H	5.26796	2.78993	3.32702
C	3.57354	0.70030	5.41747
H	1.50050	0.16478	5.18079

H	5.60820	1.41884	5.34938
H	3.72305	0.09155	6.30372
H	3.71726	3.80047	1.88379
Cl	2.96630	3.58889	-1.09430

Catalyst (TRIP, 3)-Product (R) alternative conformation
Electronic energy (Hartrees): -1870.56098
Free Energy at 1M concentration and 253 K (Hartrees): -1869.339664
Coordinates:

P	0.13773	-0.07952	0.18452
O	-1.00791	1.05346	0.41414
O	1.44041	0.67269	-0.46871
O	-0.32266	-0.93961	-1.05562
O	0.38119	-0.79789	1.45373
C	-1.10295	2.04617	-0.52980
C	-2.39545	2.22859	-1.23926
C	-2.55694	3.25729	-2.08428
H	-3.50570	3.40556	-2.58650
C	-1.43665	4.17635	-2.36384
C	-1.59728	5.24193	-3.37996
H	-2.54453	5.36999	-3.88897
C	-0.57225	6.04810	-3.67619
H	-0.68970	6.82519	-4.42038
C	0.72678	5.87170	-2.99966
H	1.55922	6.51223	-3.26110
C	0.88038	4.91586	-2.07607
H	1.84908	4.78902	-1.61139
C	-0.25988	4.03049	-1.71226
C	-0.11796	2.96318	-0.67559
C	1.83092	1.78308	0.23473
C	3.14312	1.78059	0.92706
C	3.54300	2.86739	1.60321
H	4.51534	2.88440	2.08125
C	2.66725	4.05235	1.69824
C	3.08361	5.20904	2.52422
H	4.03915	5.19780	3.03370
C	2.28024	6.27106	2.64698
H	2.58441	7.11522	3.25224
C	0.97027	6.28989	1.96862
H	0.31460	7.14226	2.09172
C	0.58753	5.25272	1.21490
H	-0.38788	5.27793	0.74779
C	1.48396	4.07711	1.04180
C	1.08362	2.90810	0.20155
C	4.05627	0.62321	0.78673
C	4.84161	0.46601	-0.48492
C	4.22799	-0.26388	1.80583
C	5.70989	-0.55790	-0.59264
C	5.24700	-1.33592	1.66410
C	5.94966	-1.48779	0.53319
H	6.27696	-0.69335	-1.50359
H	5.42986	-2.00064	2.49819
C	3.42977	-0.19285	3.10722
H	2.58243	0.51854	3.01454
C	2.80375	-1.55121	3.46278
H	2.32887	-2.00878	2.57024
H	2.02527	-1.41568	4.24395
H	3.56382	-2.25941	3.85447
C	4.31553	0.30796	4.25297
H	5.15851	-0.39112	4.43958

H	3.71724	0.40114	5.18443
H	4.73052	1.30787	4.00696
C	4.70191	1.45890	-1.63946
H	3.97256	2.25372	-1.38840
C	4.17013	0.77755	-2.90589
H	3.21810	0.25104	-2.68338
H	4.90362	0.04816	-3.30866
H	3.96778	1.53860	-3.68950
C	6.02684	2.18140	-1.91621
H	6.80100	1.47877	-2.29023
H	6.39705	2.66321	-0.98593
H	5.87501	2.97390	-2.67997
C	-3.52201	1.30032	-0.97915
C	-4.21611	1.35939	0.19256
C	-3.92050	0.30617	-2.03666
C	-5.33100	0.40369	0.42068
C	-4.96225	-0.51472	-1.79952
C	-5.70034	-0.47798	-0.51781
H	-5.87138	0.44067	1.35770
H	-5.28459	-1.21479	-2.55791
C	-3.94342	2.41205	1.26929
H	-3.10373	3.07885	0.98834
C	-5.15855	3.32886	1.45482
H	-5.43730	3.78964	0.48291
H	-4.91350	4.14536	2.16739
H	-6.03191	2.76930	1.85093
C	-3.54641	1.75680	2.59687
H	-2.67539	1.08766	2.44399
H	-4.38274	1.16488	3.02333
H	-3.25712	2.53520	3.33468
C	-3.21363	0.25096	-3.39229
H	-2.32648	0.91462	-3.39495
C	-4.14070	0.74778	-4.50838
H	-3.58847	0.79291	-5.47131
H	-4.50876	1.76882	-4.27250
H	-5.01414	0.07286	-4.63260
C	-2.67845	-1.15084	-3.71537
H	-2.02358	-1.50694	-2.89648
H	-2.07348	-1.11949	-4.64668
H	-3.50199	-1.88010	-3.86296
C	-6.88218	-1.40131	-0.27869
H	-7.30742	-1.21998	0.73435
C	-6.45282	-2.87089	-0.32258
H	-5.64873	-3.05144	0.42051
H	-6.08437	-3.15185	-1.33170
H	-7.31237	-3.52622	-0.06624
C	-8.00285	-1.12673	-1.28804
H	-8.29836	-0.05646	-1.24639
H	-8.89457	-1.74220	-1.04259
H	-7.68093	-1.37041	-2.32264
C	6.99834	-2.58009	0.42321
H	7.06261	-3.14377	1.38133
C	6.62074	-3.59149	-0.66244
H	5.62837	-4.03416	-0.43271
H	7.36628	-4.41436	-0.69401
H	6.58185	-3.11069	-1.66281
C	8.38752	-1.98553	0.16345
H	8.63696	-1.24303	0.95148
H	8.43361	-1.48700	-0.82779
H	9.15482	-2.78853	0.18863

H	-0.20351	-1.93940	-0.93438
C	1.42661	-3.86875	-1.45568
C	-0.54666	-4.57902	-0.44797
C	2.24692	-2.80332	-1.91555
C	1.88537	-5.21593	-1.61185
C	-0.15476	-5.93777	-0.55078
C	3.45329	-3.07759	-2.51852
H	1.90615	-1.78308	-1.78771
C	3.13314	-5.46003	-2.24173
C	1.05055	-6.25072	-1.12982
H	-0.81541	-6.71406	-0.18021
C	3.89739	-4.41101	-2.69453
H	4.07784	-2.26227	-2.86694
H	3.46846	-6.48647	-2.36142
H	1.36897	-7.28504	-1.22825
H	4.85008	-4.59950	-3.17959
C	-1.89065	-4.25509	0.17426
H	-2.36801	-5.19312	0.46876
C	-1.79419	-3.33164	1.38576
H	-1.28996	-2.40334	1.11631
H	-1.12383	-3.84234	2.10364
N	-3.09523	-3.04287	1.94194
N	0.21602	-3.58642	-0.88137
C	-3.24303	-2.26255	3.08966
C	-4.50613	-2.20118	3.70951
C	-2.18398	-1.51337	3.63310
C	-4.70157	-1.42188	4.84387
H	-5.33457	-2.76371	3.28411
C	-2.39954	-0.73513	4.77125
H	-1.20847	-1.50391	3.15958
C	-3.64815	-0.68291	5.38971
H	-5.68605	-1.39034	5.30306
H	-1.57043	-0.15794	5.17155
H	-3.80242	-0.07337	6.27460
H	-3.77231	-3.78867	1.86142
Cl	-3.00895	-3.58471	-1.11304

Catalyst (TRIP, 3)-Product (S) alternative conformation
Electronic energy (Hartrees): -1870.559691
Free Energy at 1M concentration and 253 K (Hartrees): -1869.339631
Coordinates:

P	-0.28330	0.37146	1.19363
O	-1.67196	1.21846	1.12831
O	0.74704	0.99348	0.06705
O	-0.59409	-1.02584	0.52303
O	0.22442	0.36134	2.58121
C	-2.00174	1.86953	-0.03746
C	-3.36070	1.64629	-0.59716
C	-3.76020	2.31696	-1.68659
H	-4.75636	2.16396	-2.08548
C	-2.84575	3.25484	-2.36242
C	-3.26637	3.90877	-3.62310
H	-4.25453	3.72669	-4.02705
C	-2.42656	4.72323	-4.27044
H	-2.73365	5.19862	-5.19303
C	-1.07298	4.96477	-3.73676
H	-0.38874	5.60455	-4.27905
C	-0.68637	4.38477	-2.59476
H	0.32092	4.55590	-2.23958
C	-1.62099	3.50027	-1.84397

C	-1.21420	2.84221	-0.56347
C	1.01602	2.31729	0.29006
C	2.39495	2.73714	0.63350
C	2.64627	4.02006	0.93280
H	3.65898	4.34103	1.14786
C	1.55474	5.01661	0.91133
C	1.82715	6.41693	1.30870
H	2.82336	6.71161	1.61441
C	0.84359	7.32334	1.29255
H	1.04309	8.34634	1.58433
C	-0.51928	6.93014	0.88501
H	-1.31414	7.66476	0.89145
C	-0.77478	5.66892	0.51816
H	-1.78465	5.39643	0.24084
C	0.31383	4.65553	0.50719
C	0.05363	3.24143	0.10517
C	3.51489	1.78762	0.45517
C	4.05780	1.56641	-0.92873
C	4.06349	1.13365	1.51416
C	5.07634	0.70179	-1.09883
C	5.22373	0.23409	1.28309
C	5.70632	0.01648	0.05173
H	5.47445	0.52171	-2.08821
H	5.69267	-0.24685	2.13152
C	3.54768	1.29588	2.94207
H	2.59961	1.87234	2.96251
C	3.23207	-0.06531	3.57863
H	2.62958	-0.68077	2.88002
H	2.64890	0.07829	4.51320
H	4.15766	-0.62371	3.83097
C	4.55662	2.07458	3.79203
H	5.51919	1.52622	3.87326
H	4.15116	2.23394	4.81401
H	4.74863	3.06919	3.33565
C	3.50400	2.32780	-2.13513
H	2.70647	3.03112	-1.82471
C	2.85874	1.38034	-3.15236
H	2.05992	0.78534	-2.66227
H	3.60958	0.69212	-3.59353
H	2.39330	1.96529	-3.97424
C	4.58615	3.19261	-2.79501
H	5.37930	2.56738	-3.25626
H	5.04898	3.86268	-2.03912
H	4.13464	3.82576	-3.58858
C	-4.29883	0.71527	0.07241
C	-4.93810	1.07480	1.22092
C	-4.58799	-0.61723	-0.55993
C	-5.90162	0.12537	1.83779
C	-5.50101	-1.42605	0.01003
C	-6.18094	-1.05583	1.26965
H	-6.39637	0.40286	2.75950
H	-5.74201	-2.37701	-0.44491
C	-4.74364	2.43715	1.88939
H	-4.01393	3.06395	1.33874
C	-6.05420	3.23316	1.89810
H	-6.44415	3.33178	0.86230
H	-5.87636	4.25372	2.29973
H	-6.82457	2.73898	2.52660
C	-4.18726	2.28069	3.31031
H	-3.25413	1.67893	3.29222

H	-4.91989	1.78365	3.98002
H	-3.94656	3.27867	3.73510
C	-3.88063	-1.06613	-1.83725
H	-3.08064	-0.34844	-2.10986
C	-4.85808	-1.11141	-3.01738
H	-4.31415	-1.36339	-3.95274
H	-5.33831	-0.11901	-3.15219
H	-5.64996	-1.87259	-2.85203
C	-3.18172	-2.41968	-1.65484
H	-2.54980	-2.40001	-0.74295
H	-2.53202	-2.62984	-2.53109
H	-3.91308	-3.24974	-1.56271
C	-7.17493	-2.00440	1.91498
H	-7.57579	-1.55549	2.85178
C	-6.49766	-3.32314	2.30595
H	-5.62990	-3.12257	2.97028
H	-6.14318	-3.87478	1.40952
H	-7.21397	-3.96939	2.85680
C	-8.37280	-2.25394	0.99145
H	-8.84667	-1.28803	0.71399
H	-9.13173	-2.87515	1.51330
H	-8.06330	-2.78144	0.06445
C	6.90900	-0.88811	-0.15084
H	7.24819	-1.29435	0.82882
C	6.55229	-2.09052	-1.02992
H	5.69372	-2.63961	-0.58934
H	7.41497	-2.78790	-1.08857
H	6.28922	-1.76980	-2.05979
C	8.08818	-0.10651	-0.74150
H	8.33044	0.76321	-0.09388
H	7.85573	0.26069	-1.76357
H	8.98624	-0.75812	-0.79719
H	0.19563	-1.66366	0.67744
C	1.62472	-3.35151	2.01684
C	2.20208	-3.00781	-0.22037
C	0.87685	-2.94200	3.15096
C	2.54150	-4.44163	2.12864
C	3.12544	-4.07876	-0.19184
C	1.03342	-3.60999	4.34461
H	0.23321	-2.07248	3.07065
C	2.66946	-5.11076	3.37337
C	3.28251	-4.78993	0.97386
H	3.69115	-4.32004	-1.08150
C	1.92625	-4.70449	4.45770
H	0.47155	-3.28730	5.21563
H	3.36444	-5.94197	3.45408
H	3.97989	-5.62215	1.02023
H	2.02845	-5.21645	5.40963
C	1.92386	-2.16185	-1.45235
C	0.65198	-2.58662	-2.20761
H	0.49814	-1.87146	-3.02604
H	-0.18528	-2.45858	-1.52009
N	0.69102	-3.94910	-2.70051
N	1.46979	-2.67787	0.83501
C	-0.30873	-4.88941	-2.42607
C	-0.97242	-4.93629	-1.18711
C	-0.63839	-5.85248	-3.39699
C	-1.95473	-5.89603	-0.95066
H	-0.71927	-4.22987	-0.40405
C	-1.60658	-6.81935	-3.14129

```
H          -0.13482       -5.83058       -4.36106
C          -2.28178       -6.84529       -1.91949
H          -2.46160       -5.90247        0.01027
H          -1.84369       -7.54851       -3.91116
H          -3.04523       -7.59191       -1.72595
H           1.07816       -4.03043       -3.62972
H           1.81471       -1.12300       -1.13673
Cl          3.33217       -2.15924       -2.61623
```

Catalyst (TRIP, 3)-Product (S) alternative conformation
Electronic energy (Hartrees): -1870.558746
Free Energy at 1M concentration and 253 K (Hartrees): -1869.339486
Coordinates:

```
P          -0.23530        0.46651       -1.12638
O           0.37599        1.98354       -1.14904
O          -1.46817        0.48444       -0.05731
O           0.78574       -0.40098       -0.29165
O          -0.53475        0.03602       -2.50535
C           0.38458        2.64494        0.05388
C           1.69198        3.00857        0.65888
C           1.73333        3.85207        1.69976
H           2.68537        4.12879        2.13668
C           0.48589        4.30198        2.34542
C           0.55727        5.12204        3.57675
H           1.51581        5.46156        3.94925
C          -0.55993        5.44073        4.23798
H          -0.50639        6.03739        5.13938
C          -1.86714        4.95990        3.75393
H          -2.76446        5.19171        4.31307
C          -1.94294        4.22330        2.63964
H          -2.90954        3.85204        2.32841
C          -0.71289        3.89716        1.86497
C          -0.76922        3.09900        0.59954
C          -2.38521        1.47597       -0.29068
C          -3.77600        1.09485       -0.64159
C          -4.70982        2.04750       -0.78726
H          -5.73317        1.77628       -1.01703
C          -4.34452        3.47457       -0.68114
C          -5.35771        4.51612       -0.96774
H          -6.37362        4.23348       -1.21435
C          -5.02137        5.80996       -0.92731
H          -5.76178        6.57050       -1.13887
C          -3.63587        6.20471       -0.60837
H          -3.36478        7.25254       -0.59995
C          -2.71452        5.27153       -0.34266
H          -1.69898        5.58097       -0.13366
C          -3.08107        3.82953       -0.35130
C          -2.07600        2.76930       -0.04033
C          -4.13976       -0.33650       -0.79977
C          -4.20061       -1.22500        0.41493
C          -4.43649       -0.85073       -2.02802
C          -4.56850       -2.51183        0.26429
C          -4.88150       -2.26509       -2.13199
C          -4.94075       -3.06100       -1.05642
H          -4.61678       -3.16578        1.12433
H          -5.16481       -2.65749       -3.09990
C          -4.32926       -0.03732       -3.31912
H          -3.86357        0.95243       -3.13508
C          -3.42702       -0.73362       -4.34839
H          -2.46514       -1.02865       -3.88146
```

H	-3.20833	-0.04114	-5.18927
H	-3.91000	-1.64133	-4.76670
C	-5.71922	0.22525	-3.91005
H	-6.21513	-0.72395	-4.20446
H	-5.63471	0.87386	-4.80805
H	-6.35965	0.74524	-3.16712
C	-3.87764	-0.70478	1.81585
H	-3.59575	0.36497	1.78174
C	-2.68382	-1.45225	2.43108
H	-1.81450	-1.42997	1.74058
H	-2.93722	-2.51179	2.64507
H	-2.38141	-0.96687	3.38350
C	-5.10499	-0.78109	2.73329
H	-5.40676	-1.83325	2.92001
H	-5.95773	-0.23892	2.27125
H	-4.87885	-0.30310	3.71044
C	2.94044	2.35323	0.19564
C	3.52878	2.70418	-0.98194
C	3.59336	1.32240	1.08083
C	4.80390	2.04657	-1.37576
C	4.77304	0.79419	0.70638
C	5.40453	1.14917	-0.58131
H	5.26896	2.32057	-2.31370
H	5.27843	0.08740	1.34978
C	2.97294	3.79834	-1.89378
H	2.01797	4.20602	-1.50568
C	3.93943	4.98642	-1.96530
H	4.14465	5.36969	-0.94271
H	3.48757	5.80937	-2.55944
H	4.90084	4.69757	-2.43996
C	2.67060	3.25497	-3.29672
H	2.00789	2.36692	-3.23243
H	3.60084	2.96882	-3.82992
H	2.15021	4.03038	-3.89842
C	2.97010	0.88924	2.40988
H	1.94743	1.30391	2.51072
C	3.78822	1.41687	3.59490
H	3.27817	1.16875	4.55023
H	3.88772	2.52062	3.53255
H	4.80445	0.96857	3.60919
C	2.80529	-0.63413	2.50665
H	2.29698	-1.02264	1.60418
H	2.18296	-0.88975	3.39059
H	3.78386	-1.14753	2.61220
C	6.73070	0.52835	-0.98358
H	7.00239	0.85066	-2.01417
C	6.65143	-1.00376	-1.00109
H	5.80199	-1.33778	-1.62891
H	6.51946	-1.41451	0.02189
H	7.58501	-1.42628	-1.42981
C	7.85058	1.00364	-0.05184
H	7.90923	2.11315	-0.06339
H	8.82777	0.60101	-0.39419
H	7.67318	0.66511	0.99129
C	-5.39481	-4.50344	-1.19098
H	-5.65225	-4.72387	-2.25163
C	-4.27198	-5.46846	-0.79540
H	-3.37555	-5.28130	-1.42376
H	-4.59616	-6.51814	-0.96021
H	-3.99459	-5.34797	0.27330

C	-6.65941	-4.76081	-0.36324
H	-7.45713	-4.04445	-0.65478
H	-6.45855	-4.65220	0.72356
H	-7.03278	-5.79023	-0.55054
H	1.27716	-1.11224	-0.83868
C	3.00091	-2.09395	-2.48104
C	1.82329	-3.57368	-1.11585
C	3.22553	-0.75898	-2.90225
C	3.74012	-3.16045	-3.07788
C	2.51303	-4.68840	-1.65683
C	4.15317	-0.50081	-3.88573
H	2.65595	0.03809	-2.43955
C	4.69249	-2.85710	-4.08476
C	3.46552	-4.47569	-2.62459
H	2.26851	-5.68752	-1.31550
C	4.89256	-1.55345	-4.47968
H	4.32319	0.52026	-4.21098
H	5.25598	-3.66780	-4.53803
H	4.00936	-5.31138	-3.05685
H	5.62091	-1.32542	-5.25156
C	0.79324	-3.70862	-0.01458
C	1.39646	-4.09116	1.33946
H	2.23418	-3.40746	1.50985
H	1.81871	-5.10945	1.29034
N	0.42448	-3.90777	2.38797
N	2.06996	-2.33534	-1.51096
C	0.75744	-3.80746	3.73577
C	-0.28138	-3.73187	4.68540
C	2.08543	-3.73575	4.19242
C	0.00170	-3.57481	6.03625
H	-1.31318	-3.78152	4.34632
C	2.35443	-3.56555	5.55129
H	2.91272	-3.81153	3.49553
C	1.32324	-3.48396	6.48455
H	-0.81916	-3.51622	6.74569
H	3.38912	-3.50545	5.87782
H	1.54099	-3.35522	7.53986
H	-0.48439	-4.30597	2.19487
H	0.25864	-2.76636	0.09519
Cl	-0.49184	-4.93508	-0.48616

Catalyst (TRIP, 3)-Product (S) alternative conformation
Electronic energy (Hartrees): -1870.557925
Free Energy at 1M concentration and 253 K (Hartrees): -1869.338545
Coordinates:

P	0.14555	-0.28733	-1.20542
O	1.33136	-0.47923	-0.07680
O	-0.65307	-1.71049	-1.32195
O	0.57783	0.14393	-2.54377
O	-0.82310	0.65297	-0.37614
C	2.08841	-1.60766	-0.25142
C	3.53767	-1.46972	-0.53836
C	4.29865	-2.56558	-0.67308
H	5.36128	-2.47072	-0.86343
C	3.69735	-3.91020	-0.56109
C	4.53245	-5.11089	-0.79399
H	5.58531	-5.00483	-1.02399
C	3.98940	-6.33113	-0.72373
H	4.60007	-7.20803	-0.89564
C	2.55317	-6.48584	-0.42286

H	2.11600	-7.47540	-0.38740
C	1.79096	-5.40775	-0.20568
H	0.73585	-5.54294	-0.00773
C	2.38613	-4.04478	-0.25349
C	1.55852	-2.82595	-0.00936
C	-0.85888	-2.37219	-0.13565
C	-2.25244	-2.61351	0.32091
C	-2.47713	-3.32220	1.43680
H	-3.49155	-3.53142	1.75430
C	-1.35011	-3.80988	2.25326
C	-1.61858	-4.48951	3.54166
H	-2.63784	-4.66866	3.86054
C	-0.60369	-4.87641	4.32143
H	-0.80040	-5.36823	5.26529
C	0.78777	-4.61961	3.90492
H	1.60414	-4.91183	4.55269
C	1.04072	-4.01571	2.73813
H	2.06836	-3.81379	2.46795
C	-0.07712	-3.60654	1.84371
C	0.17761	-2.92647	0.53717
C	-3.40010	-2.13843	-0.48819
C	-3.76586	-2.88194	-1.74256
C	-4.15678	-1.07926	-0.08137
C	-4.83973	-2.48093	-2.44897
C	-5.36145	-0.70866	-0.86914
C	-5.68481	-1.35440	-1.99795
H	-5.11628	-3.00106	-3.35598
H	-5.98930	0.10097	-0.52122
C	-3.82549	-0.25255	1.16200
H	-2.81588	-0.50355	1.54340
C	-3.79768	1.25258	0.85381
H	-3.21230	1.44831	-0.06699
H	-3.32695	1.80445	1.69471
H	-4.82040	1.65957	0.71113
C	-4.81839	-0.54900	2.29034
H	-5.85013	-0.25828	1.99873
H	-4.53320	0.01415	3.20494
H	-4.80981	-1.63136	2.53531
C	-2.96609	-4.09420	-2.21982
H	-2.12839	-4.30968	-1.52832
C	-2.33401	-3.84073	-3.59484
H	-1.72581	-2.91172	-3.57012
H	-3.10916	-3.74022	-4.38345
H	-1.66505	-4.68515	-3.86631
C	-3.83066	-5.36168	-2.23482
H	-4.64168	-5.29028	-2.98980
H	-4.28419	-5.52276	-1.23333
H	-3.20357	-6.24616	-2.47750
C	4.15622	-0.12531	-0.61175
C	4.40351	0.59053	0.52125
C	4.55890	0.42864	-1.94971
C	5.06169	1.91840	0.40683
C	5.19865	1.61227	-2.00050
C	5.45094	2.40639	-0.77891
H	5.24029	2.49572	1.30468
H	5.52510	2.01163	-2.95088
C	4.06406	0.07623	1.92157
H	3.58860	-0.92436	1.88770
C	5.33208	-0.09293	2.76650
H	6.04791	-0.76352	2.24452

H	5.07741	-0.55249	3.74549
H	5.82654	0.88287	2.95608
C	3.06073	1.00350	2.61670
H	2.17791	1.15570	1.96408
H	3.51009	1.99298	2.84309
H	2.71488	0.54715	3.56883
C	4.27277	-0.32751	-3.24573
H	3.61288	-1.19614	-3.04561
C	5.56881	-0.88032	-3.84968
H	5.34037	-1.48684	-4.75197
H	6.08414	-1.53370	-3.11426
H	6.25659	-0.05747	-4.13938
C	3.52365	0.54303	-4.26701
H	2.66434	1.05242	-3.78461
H	3.13620	-0.09167	-5.09245
H	4.18835	1.31490	-4.70878
C	6.13358	3.75936	-0.86715
H	6.23026	4.20347	0.14931
C	5.30308	4.73875	-1.70470
H	4.27998	4.82858	-1.28167
H	5.22686	4.40168	-2.76013
H	5.77323	5.74515	-1.68940
C	7.55258	3.62041	-1.43024
H	8.13734	2.90356	-0.81483
H	8.07000	4.60312	-1.40294
H	7.53514	3.26084	-2.48086
C	-6.90987	-0.94383	-2.79492
H	-7.41554	-0.08448	-2.29931
C	-6.51626	-0.47859	-4.20160
H	-5.77393	0.34553	-4.13563
H	-7.41032	-0.09756	-4.73983
H	-6.07756	-1.31044	-4.79238
C	-7.93043	-2.08604	-2.85875
H	-8.19891	-2.41383	-1.83164
H	-7.52669	-2.95571	-3.41917
H	-8.85656	-1.73996	-3.36543
H	-0.41943	1.17848	0.39000
C	-0.67106	1.46857	2.93076
C	-0.40954	3.43977	1.72529
C	-0.48927	0.06299	3.00466
C	-1.18122	2.16916	4.06680
C	-0.88575	4.21237	2.81594
C	-0.83157	-0.61474	4.15198
H	-0.04983	-0.44132	2.15222
C	-1.53911	1.43644	5.22828
C	-1.28925	3.57756	3.96574
H	-0.93964	5.29124	2.72261
C	-1.37263	0.07132	5.26715
H	-0.67847	-1.68693	4.21407
H	-1.93658	1.97238	6.08571
H	-1.67643	4.14625	4.80693
H	-1.64352	-0.48567	6.15860
N	-0.33813	2.12065	1.77684
C	0.01117	4.14668	0.45000
H	0.13539	5.20703	0.67301
C	-0.98917	3.98998	-0.71926
H	-1.10871	2.93064	-0.95130
H	-0.56039	4.46973	-1.60690
N	-2.27786	4.55053	-0.37354
H	-3.05030	3.90382	-0.36659

C	-2.62820	5.88477	-0.55768
C	-3.97245	6.26951	-0.37988
C	-1.68829	6.87760	-0.89091
C	-4.35847	7.59577	-0.53283
H	-4.71066	5.51345	-0.12179
C	-2.09105	8.20539	-1.04090
H	-0.64594	6.62066	-1.04583
C	-3.42185	8.57950	-0.86376
H	-5.40252	7.86273	-0.39405
H	-1.34695	8.95247	-1.30309
H	-3.72607	9.61436	-0.98211
Cl	1.65588	3.57807	-0.09485

Catalyst (TRIP, 3)-Product (S) alternative conformation
Electronic energy (Hartrees): -1870.559536
Free Energy at 1M concentration and 253 K (Hartrees): -1869.3385
Coordinates:

P	-0.32344	0.04690	-0.72951
O	0.49011	1.43439	-0.99672
O	-1.58143	0.40665	0.24868
O	0.60655	-0.78896	0.23597
O	-0.69229	-0.58174	-2.01810
C	0.20089	2.57468	-0.27900
C	1.35442	3.35643	0.23925
C	1.16753	4.56914	0.77698
H	2.01366	5.13320	1.15297
C	-0.19232	5.09513	0.97322
C	-0.38168	6.37650	1.69209
H	0.47725	6.96065	1.99837
C	-1.61289	6.81465	1.97217
H	-1.75032	7.74928	2.50038
C	-2.78720	6.01317	1.58286
H	-3.78037	6.35460	1.84489
C	-2.62629	4.85830	0.92696
H	-3.50387	4.27271	0.69019
C	-1.26202	4.37668	0.56464
C	-1.05382	3.09905	-0.19271
C	-2.51457	1.15231	-0.42363
C	-3.85880	0.58259	-0.68578
C	-4.74045	1.26943	-1.42787
H	-5.73477	0.87359	-1.59858
C	-4.36793	2.57732	-2.00583
C	-5.31210	3.28329	-2.90211
H	-6.27928	2.84951	-3.12368
C	-4.97309	4.45854	-3.44353
H	-5.66287	4.97314	-4.09990
C	-3.65316	5.05297	-3.15625
H	-3.37811	5.99504	-3.61272
C	-2.79510	4.42585	-2.34320
H	-1.82455	4.86803	-2.15973
C	-3.16830	3.13168	-1.71252
C	-2.22951	2.41517	-0.79831
C	-4.26629	-0.67729	-0.02324
C	-4.56036	-0.66089	1.45126
C	-4.37297	-1.83982	-0.72362
C	-4.90045	-1.81198	2.06132
C	-4.80478	-3.07291	-0.01466
C	-5.03691	-3.07551	1.30514
H	-5.10273	-1.82271	3.12370
H	-4.91909	-3.99025	-0.57745

C	-4.03927	-1.94424	-2.21068
H	-3.60709	-0.99779	-2.59559
C	-2.97827	-3.02312	-2.46348
H	-2.13975	-2.90188	-1.74704
H	-2.57852	-2.92857	-3.49571
H	-3.39715	-4.04444	-2.34598
C	-5.30391	-2.21834	-3.03113
H	-5.75782	-3.19364	-2.75482
H	-5.05832	-2.23586	-4.11449
H	-6.05060	-1.41446	-2.85752
C	-4.49823	0.63049	2.26793
H	-4.24845	1.49378	1.62055
C	-3.40079	0.56046	3.33772
H	-2.42483	0.31434	2.86817
H	-3.63356	-0.20882	4.10388
H	-3.30168	1.54392	3.84497
C	-5.85914	0.96434	2.89351
H	-6.14888	0.21480	3.65963
H	-6.64153	0.99477	2.10516
H	-5.81843	1.96254	3.37974
C	2.71310	2.76851	0.24275
C	3.47514	2.75086	-0.88520
C	3.27416	2.25114	1.53876
C	4.87101	2.24424	-0.80341
C	4.54188	1.79754	1.56445
C	5.39362	1.81512	0.35433
H	5.48833	2.26001	-1.69214
H	4.96890	1.43662	2.49010
C	2.98234	3.29688	-2.22580
H	1.93177	3.64579	-2.16308
C	3.80742	4.51614	-2.65434
H	3.78492	5.28836	-1.85568
H	3.37783	4.96044	-3.57761
H	4.86333	4.23963	-2.85749
C	3.00583	2.21066	-3.30839
H	2.40212	1.33636	-2.98621
H	4.04167	1.87379	-3.52181
H	2.56539	2.60344	-4.24958
C	2.45779	2.28301	2.83381
H	1.43119	2.65241	2.64333
C	3.08196	3.24605	3.85170
H	2.42173	3.34238	4.74003
H	3.20049	4.25343	3.39851
H	4.07625	2.88476	4.18960
C	2.27819	0.88671	3.43916
H	1.76204	0.22566	2.71382
H	1.64455	0.94566	4.34981
H	3.25310	0.44006	3.72319
C	6.85684	1.41384	0.43504
H	7.34431	1.55194	-0.55651
C	7.00844	-0.06520	0.80171
H	6.57364	-0.69220	-0.00147
H	6.50431	-0.29927	1.76316
H	8.08358	-0.33008	0.89064
C	7.61340	2.30524	1.42746
H	7.47401	3.37507	1.16167
H	8.70033	2.07854	1.38991
H	7.25704	2.14313	2.46670
C	-5.44148	-4.35631	2.01215
H	-5.49410	-5.19412	1.28063

C	-4.40056	-4.74974	3.06666
H	-3.39773	-4.83886	2.59637
H	-4.66344	-5.73312	3.51166
H	-4.34910	-3.99673	3.88140
C	-6.83471	-4.22135	2.63730
H	-7.57334	-3.92932	1.86035
H	-6.84171	-3.45842	3.44429
H	-7.15263	-5.19404	3.06973
H	0.73002	-1.78456	-0.02558
C	0.96442	-4.10276	-1.30073
C	1.58262	-3.95350	0.94380
C	0.49942	-3.46862	-2.47958
C	1.29281	-5.49452	-1.33712
C	1.94062	-5.32419	0.98176
C	0.35216	-4.20206	-3.63669
H	0.24253	-2.41437	-2.45252
C	1.12355	-6.21774	-2.54507
C	1.78382	-6.08561	-0.14861
H	2.31750	-5.75111	1.90273
C	0.66028	-5.58273	-3.67439
H	-0.01055	-3.71020	-4.53407
H	1.37009	-7.27583	-2.56090
H	2.04150	-7.14148	-0.14517
H	0.53243	-6.13823	-4.59850
C	1.83095	-3.03351	2.12088
C	3.26627	-2.43843	2.12679
H	3.99640	-3.24909	2.14086
H	3.38820	-1.89262	3.06757
N	3.51447	-1.56011	1.00919
N	1.09809	-3.37123	-0.14580
C	4.09787	-1.93590	-0.20327
C	4.95561	-3.04039	-0.34320
C	3.86987	-1.11805	-1.32779
C	5.56966	-3.30090	-1.56873
H	5.16614	-3.68830	0.50070
C	4.49193	-1.38696	-2.54074
H	3.19980	-0.26844	-1.23230
C	5.35015	-2.48147	-2.67511
H	6.23345	-4.15739	-1.65115
H	4.29794	-0.73833	-3.39006
H	5.83408	-2.69182	-3.62344
H	2.82250	-0.83044	0.89733
H	1.10494	-2.22170	2.10569
Cl	1.55025	-3.87766	3.72115

Catalyst (TRIP, 3)-Product (S) alternative conformation
Electronic energy (Hartrees): -1870.556723
Free Energy at 1M concentration and 253 K (Hartrees): -1869.338344
Coordinates:

P	0.56103	0.13964	-0.08082
O	0.27194	-1.31916	-0.76019
O	2.13027	0.16722	0.37242
O	-0.10193	0.14321	1.35005
O	0.13144	1.19691	-1.02438
C	0.86435	-2.39697	-0.14924
C	0.00247	-3.48785	0.37872
C	0.56327	-4.57993	0.91849
H	-0.05957	-5.38792	1.28395
C	2.03017	-4.69100	1.03046
C	2.62787	-5.85861	1.71843

H	1.99256	-6.64374	2.10926
C	3.95433	-5.94668	1.86334
H	4.39034	-6.79912	2.36803
C	4.82718	-4.87487	1.34798
H	5.89824	-4.94044	1.48974
C	4.29725	-3.81908	0.71972
H	4.95503	-3.03319	0.37327
C	2.82477	-3.72159	0.52228
C	2.20795	-2.55679	-0.18191
C	3.00050	-0.27082	-0.59239
C	4.00147	0.67533	-1.14562
C	4.89627	0.24843	-2.04894
H	5.64887	0.92682	-2.43292
C	4.86345	-1.14673	-2.53093
C	5.79587	-1.57781	-3.59785
H	6.51791	-0.88025	-4.00383
C	5.74822	-2.82938	-4.06664
H	6.42869	-3.14319	-4.84763
C	4.75891	-3.78323	-3.52981
H	4.71064	-4.78795	-3.92950
C	3.91604	-3.40537	-2.56174
H	3.18310	-4.11422	-2.20011
C	3.97674	-2.02510	-2.00810
C	3.05280	-1.58004	-0.92155
C	4.06986	2.06546	-0.63711
C	4.66254	2.31721	0.72168
C	3.63372	3.11262	-1.39223
C	4.74809	3.58338	1.17160
C	3.79339	4.49500	-0.86954
C	4.30577	4.72805	0.34661
H	5.16780	3.78400	2.14798
H	3.48685	5.32943	-1.48656
C	2.98970	2.93121	-2.76700
H	2.77856	1.86116	-2.97212
C	1.63464	3.64970	-2.85650
H	1.01159	3.41582	-1.96922
H	1.08908	3.31569	-3.76491
H	1.76034	4.75102	-2.91704
C	3.93366	3.42128	-3.87038
H	4.13606	4.50878	-3.76856
H	3.48228	3.23569	-4.86841
H	4.89814	2.87350	-3.81953
C	5.18926	1.17633	1.59251
H	5.06743	0.20205	1.08026
C	4.40752	1.07148	2.90896
H	3.31984	0.98342	2.70214
H	4.57824	1.96053	3.55185
H	4.72740	0.16693	3.46909
C	6.69397	1.32324	1.85426
H	6.91283	2.22125	2.46949
H	7.23994	1.40557	0.89003
H	7.07469	0.42904	2.39268
C	-1.47475	-3.40511	0.27126
C	-2.10271	-3.62554	-0.91809
C	-2.28949	-3.17962	1.51494
C	-3.58940	-3.64810	-0.95691
C	-3.63144	-3.26127	1.44179
C	-4.32138	-3.49537	0.15533
H	-4.08833	-3.81100	-1.90333
H	-4.23142	-3.13122	2.33200

C	-1.34811	-3.90075	-2.21953
H	-0.25053	-3.88314	-2.06983
C	-1.67187	-5.30004	-2.75684
H	-1.45413	-6.06372	-1.97964
H	-1.04204	-5.52072	-3.64513
H	-2.73794	-5.38414	-3.05497
C	-1.65462	-2.82404	-3.26816
H	-1.43714	-1.81581	-2.85580
H	-2.71816	-2.85799	-3.58460
H	-1.01720	-2.97579	-4.16533
C	-1.62901	-2.86257	2.85517
H	-0.54451	-2.67782	2.71244
C	-1.76124	-4.04396	3.82321
H	-1.21847	-3.82473	4.76742
H	-1.31973	-4.95832	3.37446
H	-2.82687	-4.24464	4.06444
C	-2.19638	-1.58040	3.48294
H	-2.25271	-0.77690	2.72268
H	-1.53798	-1.23883	4.31000
H	-3.21414	-1.74380	3.89553
C	-5.83754	-3.55058	0.09306
H	-6.16832	-3.74352	-0.95244
C	-6.45509	-2.21241	0.51631
H	-6.07990	-1.39655	-0.13681
H	-6.20899	-1.97067	1.57191
H	-7.56035	-2.25385	0.41256
C	-6.37818	-4.70058	0.95064
H	-5.91331	-5.65990	0.63739
H	-7.47767	-4.78892	0.81823
H	-6.16602	-4.53332	2.02785
C	4.44161	6.14839	0.86485
H	4.05925	6.86816	0.10621
C	3.60412	6.35015	2.13298
H	2.54364	6.08441	1.93461
H	3.64174	7.41494	2.44763
H	3.98117	5.72268	2.96818
C	5.91222	6.50220	1.11466
H	6.50397	6.34449	0.18759
H	6.34381	5.87912	1.92640
H	6.00171	7.57066	1.40550
H	-0.88442	0.79888	1.43290
C	-2.16944	2.17580	3.12933
C	-3.11217	2.11626	0.99571
C	-1.09069	1.83507	3.98795
C	-3.28871	2.89078	3.65690
C	-4.25698	2.82666	1.44137
C	-1.13527	2.18905	5.31710
H	-0.24916	1.28962	3.57493
C	-3.29941	3.24128	5.03192
C	-4.33718	3.20706	2.75967
H	-5.04814	3.04172	0.73298
C	-2.24437	2.89605	5.84492
H	-0.31094	1.92391	5.97202
H	-4.15295	3.78398	5.42876
H	-5.20529	3.74972	3.12480
H	-2.25715	3.16474	6.89671
C	-2.93749	1.66890	-0.44181
C	-3.66405	2.46589	-1.52374
H	-3.22758	2.18994	-2.49565
H	-3.41381	3.52040	-1.35003

N	-5.10795	2.30084	-1.49968
N	-2.11380	1.80978	1.81404
C	-5.91277	2.93819	-2.45358
C	-7.22880	2.47635	-2.64926
C	-5.47549	4.04875	-3.19514
C	-8.07669	3.10879	-3.55068
H	-7.57877	1.61669	-2.08236
C	-6.33902	4.67797	-4.09375
H	-4.46532	4.42507	-3.07846
C	-7.64115	4.21869	-4.28095
H	-9.08632	2.73045	-3.68483
H	-5.97902	5.53493	-4.65643
H	-8.30487	4.71113	-4.98439
H	-5.37031	1.33314	-1.34749
H	-1.87132	1.66316	-0.68338
Cl	-3.41130	-0.11497	-0.56240

Catalyst (TRIP, 3)-Product (S) alternative conformation
Electronic energy (Hartrees): -1870.558509
Free Energy at 1M concentration and 253 K (Hartrees): -1869.337675
Coordinates:

P	0.54353	-0.33143	0.22440
O	-0.32130	1.06097	0.24424
O	1.95398	-0.03400	-0.52958
O	-0.09097	-1.27230	-0.88211
O	0.60249	-0.83125	1.61472
C	-0.14638	1.96086	-0.78060
C	-1.35464	2.46224	-1.48988
C	-1.23849	3.41298	-2.42843
H	-2.12085	3.79297	-2.93064
C	0.08985	3.93003	-2.80545
C	0.21272	4.89521	-3.92215
H	-0.67244	5.24503	-4.43879
C	1.41738	5.33604	-4.29980
H	1.50671	6.03965	-5.11738
C	2.63010	4.85377	-3.61212
H	3.60382	5.19593	-3.93826
C	2.52890	3.98680	-2.59806
H	3.43352	3.62745	-2.12568
C	1.19284	3.51043	-2.14496
C	1.05284	2.55070	-1.00697
C	2.64739	1.00492	0.03331
C	3.94897	0.73717	0.69357
C	4.63736	1.74672	1.24618
H	5.60339	1.56276	1.70152
C	4.09063	3.11847	1.23135
C	4.81666	4.20696	1.92528
H	5.75734	4.00329	2.42164
C	4.31223	5.44543	1.94456
H	4.84231	6.23998	2.45364
C	3.02463	5.73024	1.28285
H	2.61083	6.72965	1.32200
C	2.36399	4.75530	0.64776
H	1.40970	4.98134	0.19087
C	2.92698	3.37875	0.59101
C	2.21093	2.27307	-0.11432
C	4.53854	-0.62054	0.64585
C	5.16630	-1.09630	-0.63451
C	4.54418	-1.42468	1.74520
C	5.72579	-2.32041	-0.67101

C	5.21912	-2.74692	1.66815
C	5.76854	-3.18606	0.52725
H	6.17597	-2.68305	-1.58509
H	5.26410	-3.36698	2.55387
C	3.88800	-1.02429	3.06628
H	3.27850	-0.10436	2.94703
C	2.92023	-2.10761	3.56754
H	2.25964	-2.45029	2.74425
H	2.28308	-1.69834	4.38057
H	3.46596	-2.98737	3.96835
C	4.95427	-0.72071	4.12402
H	5.56680	-1.62120	4.34299
H	4.47159	-0.38127	5.06532
H	5.62562	0.08887	3.76757
C	5.19767	-0.22006	-1.88706
H	4.72653	0.76304	-1.69156
C	4.40146	-0.85777	-3.03301
H	3.36528	-1.07971	-2.69995
H	4.87630	-1.80008	-3.37872
H	4.34556	-0.15635	-3.89277
C	6.63758	0.08795	-2.31866
H	7.16565	-0.82922	-2.65436
H	7.19907	0.53740	-1.47173
H	6.63465	0.81633	-3.15765
C	-2.69845	1.96399	-1.11153
C	-3.28824	2.36540	0.04967
C	-3.42564	1.02652	-2.03455
C	-4.60632	1.78784	0.42689
C	-4.63060	0.55167	-1.66586
C	-5.24858	0.92676	-0.37613
H	-5.06069	2.08180	1.36419
H	-5.17698	-0.10849	-2.32536
C	-2.68839	3.44823	0.95054
H	-1.72343	3.82302	0.55288
C	-3.61314	4.66968	1.01791
H	-3.82417	5.04244	-0.00738
H	-3.12312	5.48718	1.58894
H	-4.57440	4.42236	1.51564
C	-2.39256	2.91237	2.35490
H	-1.69937	2.04946	2.29286
H	-3.32252	2.59837	2.87269
H	-1.90147	3.69888	2.96664
C	-2.85234	0.63577	-3.39633
H	-1.84441	1.07174	-3.53807
C	-3.72294	1.17261	-4.53908
H	-3.23774	0.96499	-5.51671
H	-3.84404	2.27243	-4.43888
H	-4.72730	0.69879	-4.53733
C	-2.66488	-0.87850	-3.51244
H	-2.03741	-1.22534	-2.67047
H	-2.14425	-1.13012	-4.46104
H	-3.63475	-1.41825	-3.48915
C	-6.61251	0.38111	0.01063
H	-6.88545	0.73183	1.03158
C	-6.61590	-1.15315	0.05029
H	-5.85130	-1.52311	0.76204
H	-6.41140	-1.58601	-0.95145
H	-7.60586	-1.52102	0.39454
C	-7.69027	0.90173	-0.94667
H	-7.68157	2.01273	-0.96121

H	-8.69424	0.56774	-0.60802
H	-7.52281	0.52803	-1.97925
C	6.43541	-4.54850	0.46899
H	6.36946	-5.04689	1.46249
C	5.72532	-5.46227	-0.53651
H	4.64684	-5.54268	-0.28156
H	6.16676	-6.48114	-0.50156
H	5.82122	-5.07239	-1.57201
C	7.92553	-4.41528	0.13420
H	8.42195	-3.74594	0.86907
H	8.07343	-4.00121	-0.88566
H	8.41662	-5.41051	0.18473
H	-0.69835	-2.00955	-0.57118
C	-2.07381	-3.86025	-1.86533
C	-3.02099	-3.37526	0.20037
C	-0.95229	-3.79651	-2.73575
C	-3.25083	-4.54980	-2.29825
C	-4.21974	-4.04783	-0.14893
C	-1.01153	-4.37876	-3.98113
H	-0.06456	-3.27129	-2.40224
C	-3.27870	-5.13381	-3.59137
C	-4.33846	-4.61295	-1.39523
H	-5.02737	-4.10814	0.57275
C	-2.18165	-5.04873	-4.41664
H	-0.15099	-4.32425	-4.64084
H	-4.17834	-5.65086	-3.91363
H	-5.25130	-5.12323	-1.69074
H	-2.20671	-5.49748	-5.40479
C	-2.88393	-2.76408	1.57706
C	-2.69964	-1.22234	1.52333
H	-3.66655	-0.80331	1.23288
H	-2.02038	-0.97937	0.71019
N	-2.23064	-0.60222	2.71897
N	-2.00140	-3.25606	-0.64021
C	-3.01672	-0.19523	3.77858
C	-2.39369	0.34375	4.92491
C	-4.42462	-0.25044	3.75569
C	-3.14984	0.82025	5.98779
H	-1.30845	0.39229	4.95884
C	-5.16967	0.23644	4.82923
H	-4.94534	-0.66407	2.89931
C	-4.54744	0.77618	5.95350
H	-2.64050	1.23306	6.85456
H	-6.25443	0.18687	4.77968
H	-5.13434	1.15046	6.78610
H	-1.22284	-0.59235	2.82275
H	-3.76133	-3.00750	2.17796
Cl	-1.50047	-3.57383	2.44748

Catalyst (TRIP, 3)-Product (S) alternative conformation
Electronic energy (Hartrees): -1870.557656
Free Energy at 1M concentration and 253 K (Hartrees): -1869.337331
Coordinates:

P	-0.48041	-0.19057	0.03146
O	-1.87302	0.28980	0.72357
O	0.37714	1.15083	-0.33553
O	-0.57987	-1.00584	-1.19694
O	0.19411	-0.83895	1.30835
C	-2.53707	1.24909	0.00561
C	-3.85030	0.91783	-0.60149

C	-4.53861	1.86222	-1.25827
H	-5.50713	1.63171	-1.68591
C	-3.97448	3.21631	-1.42663
C	-4.70407	4.21910	-2.23631
H	-5.66585	3.97325	-2.66907
C	-4.17685	5.43099	-2.43988
H	-4.70976	6.16249	-3.03348
C	-2.85947	5.76975	-1.86896
H	-2.42664	6.74337	-2.05942
C	-2.19483	4.87312	-1.13104
H	-1.21815	5.13467	-0.74663
C	-2.78336	3.53159	-0.86602
C	-2.06615	2.51421	-0.03819
C	0.30934	2.20206	0.54432
C	1.57891	2.73777	1.09929
C	1.55880	3.82366	1.88684
H	2.48463	4.23610	2.26988
C	0.27777	4.45708	2.25382
C	0.26377	5.58428	3.21460
H	1.19266	5.96275	3.62290
C	-0.89621	6.13711	3.58499
H	-0.90596	6.95932	4.28880
C	-2.16791	5.61904	3.04565
H	-3.10461	6.05591	3.36695
C	-2.16531	4.60508	2.17258
H	-3.11082	4.22559	1.80851
C	-0.88077	3.99909	1.72693
C	-0.85000	2.87401	0.74377
C	2.87620	2.11963	0.72681
C	3.41408	2.31739	-0.66498
C	3.60032	1.40797	1.63641
C	4.60661	1.77995	-0.98646
C	4.93717	0.88654	1.24845
C	5.42302	1.05684	0.01181
H	5.00734	1.90739	-1.98284
H	5.53509	0.37070	1.98840
C	3.10220	1.12133	3.05315
H	2.04975	1.44665	3.18448
C	3.11153	-0.38296	3.35050
H	2.61879	-0.92901	2.52334
H	2.55579	-0.58957	4.29008
H	4.14447	-0.77282	3.46516
C	3.93824	1.88309	4.08750
H	4.99618	1.54529	4.07534
H	3.52576	1.71825	5.10581
H	3.90988	2.97242	3.87470
C	2.66602	3.14893	-1.70745
H	1.71806	3.54080	-1.29068
C	2.28529	2.30591	-2.93214
H	1.69523	1.42093	-2.61766
H	3.18535	1.95840	-3.48161
H	1.66016	2.90594	-3.62720
C	3.47871	4.38291	-2.12116
H	4.40648	4.09542	-2.65925
H	3.75282	4.97518	-1.22189
H	2.87237	5.03012	-2.79043
C	-4.41493	-0.44532	-0.46024
C	-4.96522	-0.85473	0.71721
C	-4.42780	-1.36323	-1.65179
C	-5.57126	-2.20994	0.79769

C	-5.03792	-2.55874	-1.54803
C	-5.62254	-3.01823	-0.27005
H	-5.99282	-2.54240	1.73741
H	-5.08796	-3.21781	-2.40385
C	-5.02651	0.03408	1.96075
H	-4.56193	1.02407	1.78080
C	-6.47968	0.32182	2.35703
H	-7.02460	0.77007	1.49868
H	-6.50747	1.04460	3.20039
H	-7.00521	-0.60386	2.67254
C	-4.25390	-0.59660	3.12652
H	-3.21163	-0.82386	2.81735
H	-4.73627	-1.53502	3.47157
H	-4.21399	0.11107	3.98201
C	-3.77672	-0.96452	-2.97548
H	-3.17424	-0.04274	-2.84194
C	-4.84255	-0.65659	-4.03348
H	-4.35935	-0.30404	-4.96966
H	-5.52160	0.14377	-3.67181
H	-5.44756	-1.55949	-4.26352
C	-2.79952	-2.03644	-3.48698
H	-2.13736	-2.38395	-2.66800
H	-2.16486	-1.61442	-4.29542
H	-3.33947	-2.91590	-3.89683
C	-6.25646	-4.39347	-0.16412
H	-6.63510	-4.55904	0.86980
C	-5.22569	-5.49347	-0.44356
H	-4.35906	-5.38589	0.24348
H	-4.86074	-5.44608	-1.49145
H	-5.68074	-6.49262	-0.27386
C	-7.46042	-4.51590	-1.10512
H	-8.19307	-3.70819	-0.89220
H	-7.96625	-5.49269	-0.94943
H	-7.14860	-4.44673	-2.16877
C	6.81122	0.55448	-0.34459
H	7.28575	0.08373	0.54593
C	6.74345	-0.52106	-1.43246
H	6.14331	-1.38064	-1.06926
H	7.76373	-0.89192	-1.66793
H	6.28736	-0.12445	-2.36420
C	7.72107	1.71240	-0.77208
H	7.74254	2.49190	0.01941
H	7.36992	2.17196	-1.72011
H	8.75825	1.34434	-0.92369
H	0.60442	-1.76037	1.21236
C	1.44829	-3.52282	2.90048
C	2.37961	-3.80438	0.78495
C	0.50593	-2.86936	3.73965
C	2.31110	-4.51508	3.46486
C	3.26727	-4.79884	1.26933
C	0.43423	-3.19146	5.07577
H	-0.14096	-2.11380	3.30946
C	2.20919	-4.82132	4.84680
C	3.23576	-5.14475	2.59815
H	3.95606	-5.27797	0.58195
C	1.28920	-4.17203	5.63666
H	-0.28786	-2.68726	5.71074
H	2.86844	-5.57630	5.26628
H	3.90742	-5.90266	2.99240
H	1.21404	-4.40895	6.69339

N	1.51432	-3.18304	1.57606
C	2.40104	-3.43540	-0.68242
H	3.17264	-4.01353	-1.19182
C	2.64534	-1.91155	-0.91474
H	3.71525	-1.74168	-0.74241
H	2.12721	-1.35146	-0.13836
N	2.25635	-1.37344	-2.18089
H	1.26484	-1.16654	-2.25036
C	2.96761	-1.51404	-3.35980
C	2.43081	-0.97311	-4.54762
C	4.22703	-2.13858	-3.42796
C	3.13887	-1.03626	-5.74044
H	1.45643	-0.49300	-4.51275
C	4.93160	-2.18588	-4.63109
H	4.65925	-2.59621	-2.54490
C	4.40130	-1.63674	-5.79670
H	2.70103	-0.60735	-6.63790
H	5.90501	-2.66918	-4.65135
H	4.95252	-1.68036	-6.73057
Cl	0.84574	-4.01204	-1.44190

Catalyst (TRIP, 3)-Product (S) alternative conformation
Electronic energy (Hartrees): -1870.559323
Free Energy at 1M concentration and 253 K (Hartrees): -1869.337294
Coordinates:

P	-0.12114	0.11182	0.12263
O	0.73524	1.25412	-0.67230
O	-1.45464	0.83575	0.72450
O	0.59567	-0.58964	1.21084
O	-0.62858	-0.77179	-1.08527
C	0.99091	2.37219	0.08098
C	2.39569	2.72290	0.41844
C	2.65596	3.83680	1.11944
H	3.67957	4.11191	1.34509
C	1.55296	4.69161	1.60019
C	1.84886	5.85934	2.46190
H	2.87319	6.10278	2.71535
C	0.85205	6.61596	2.93368
H	1.06874	7.46785	3.56517
C	-0.54768	6.28393	2.60584
H	-1.35087	6.88673	3.00969
C	-0.82489	5.23461	1.82321
H	-1.85932	4.99473	1.61568
C	0.27486	4.40123	1.26550
C	-0.00727	3.22891	0.38344
C	-2.01370	1.85295	-0.01316
C	-3.44833	1.74751	-0.38987
C	-4.06410	2.77847	-0.98819
H	-5.11824	2.71630	-1.23207
C	-3.30721	3.98920	-1.35454
C	-3.97170	5.06608	-2.12428
H	-5.01994	4.98258	-2.38317
C	-3.27935	6.14443	-2.50616
H	-3.76491	6.93016	-3.07037
C	-1.84700	6.25931	-2.17278
H	-1.28587	7.12240	-2.50709
C	-1.23957	5.29454	-1.47241
H	-0.18219	5.38795	-1.26374
C	-2.00482	4.10363	-1.00994
C	-1.35492	3.02014	-0.20993

C	-4.22261	0.53630	-0.03036
C	-4.58769	0.30616	1.41101
C	-4.63508	-0.34578	-0.98561
C	-5.28985	-0.79424	1.73831
C	-5.42510	-1.53572	-0.57209
C	-5.72553	-1.76588	0.71405
H	-5.57332	-0.96938	2.76714
H	-5.77260	-2.22549	-1.32993
C	-4.36157	-0.14598	-2.47937
H	-3.71358	0.73463	-2.66118
C	-3.61096	-1.33615	-3.08967
H	-2.64191	-1.47645	-2.57354
H	-3.39495	-1.13930	-4.16153
H	-4.19938	-2.27433	-3.02199
C	-5.67118	0.11136	-3.23408
H	-6.33585	-0.77764	-3.19922
H	-5.45789	0.35059	-4.29785
H	-6.20585	0.97595	-2.78640
C	-4.23394	1.31669	2.50306
H	-3.66161	2.16624	2.08263
C	-3.34347	0.68888	3.58465
H	-2.45182	0.21567	3.12236
H	-3.89421	-0.08071	4.16528
H	-2.99280	1.47339	4.28880
C	-5.49720	1.93145	3.11971
H	-6.09367	1.16986	3.66486
H	-6.12833	2.37945	2.32245
H	-5.21867	2.73564	3.83396
C	3.51482	1.89255	-0.08429
C	3.86876	1.92760	-1.40027
C	4.30509	1.05266	0.88194
C	4.99416	1.07600	-1.86715
C	5.35104	0.34036	0.42178
C	5.70544	0.32669	-1.01382
H	5.25171	1.07505	-2.91827
H	5.94748	-0.24931	1.10414
C	3.20463	2.86153	-2.41586
H	2.40923	3.47668	-1.94940
C	4.22089	3.86125	-2.98172
H	4.70944	4.41586	-2.15200
H	3.70627	4.59859	-3.63443
H	5.00247	3.34935	-3.58152
C	2.52954	2.07197	-3.54359
H	1.77380	1.37725	-3.12322
H	3.26920	1.48798	-4.13003
H	2.00608	2.76777	-4.23360
C	3.97700	1.02272	2.37487
H	3.03915	1.57747	2.57695
C	5.08107	1.70571	3.19088
H	4.78749	1.75685	4.26112
H	5.23967	2.74242	2.82540
H	6.03894	1.14878	3.11309
C	3.73084	-0.40467	2.88077
H	2.96341	-0.89444	2.25346
H	3.35696	-0.37933	3.92657
H	4.65678	-1.01635	2.85713
C	6.86244	-0.52000	-1.51340
H	6.98549	-0.38554	-2.61182
C	6.59440	-2.01017	-1.27434
H	5.66084	-2.31350	-1.79283

H	6.49428	-2.23357	-0.19096
H	7.42836	-2.61820	-1.68515
C	8.18078	-0.08666	-0.86169
H	8.35047	0.99825	-1.03116
H	9.02881	-0.64449	-1.31317
H	8.17364	-0.28305	0.23127
C	-6.53866	-2.98540	1.11022
H	-6.71899	-3.62699	0.21829
C	-5.78837	-3.84534	2.13605
H	-4.77328	-4.09595	1.76297
H	-6.33871	-4.79485	2.30810
H	-5.68887	-3.31817	3.10825
C	-7.91162	-2.56784	1.64835
H	-8.44739	-1.95764	0.88994
H	-7.81243	-1.97334	2.58145
H	-8.52520	-3.46811	1.86564
H	-0.21718	-1.69153	-1.12876
C	-0.91451	-4.21192	-1.36161
C	1.35409	-3.87013	-1.72669
C	-2.16371	-3.72128	-0.89761
C	-0.83725	-5.55691	-1.84621
C	1.51489	-5.19253	-2.21158
C	-3.27929	-4.52597	-0.94440
H	-2.21802	-2.70852	-0.51480
C	-2.00748	-6.35858	-1.87621
C	0.42584	-6.02495	-2.27718
H	2.49659	-5.52961	-2.52513
C	-3.20738	-5.85073	-1.43792
H	-4.23341	-4.14474	-0.60137
H	-1.93559	-7.37666	-2.24891
H	0.52148	-7.04091	-2.65081
H	-4.10325	-6.46326	-1.46281
N	0.18377	-3.39357	-1.32992
C	2.58062	-2.98698	-1.61898
H	3.42800	-3.51800	-2.05711
C	2.91795	-2.58485	-0.17405
H	2.05199	-2.11173	0.28734
H	3.71411	-1.83159	-0.19947
N	3.30794	-3.77780	0.55942
H	4.24613	-4.08488	0.33678
C	2.98330	-4.01234	1.90523
C	3.80823	-4.87617	2.64938
C	1.84814	-3.46449	2.52787
C	3.50993	-5.18456	3.97317
H	4.69147	-5.30504	2.17958
C	1.56836	-3.77577	3.85769
H	1.19802	-2.77531	2.00130
C	2.38575	-4.63554	4.59230
H	4.16677	-5.85267	4.52379
H	0.69362	-3.32897	4.32207
H	2.15570	-4.86867	5.62722
Cl	2.40592	-1.48768	-2.64830

Catalyst (TRIP, 3)-Product (R) alternative conformation
Electronic energy (Hartrees): -1870.558341
Free Energy at 1M concentration and 253 K (Hartrees): -1869.337223
Coordinates:

P	0.68588	-0.12183	1.01769
O	2.30707	0.02026	1.01970
O	0.32947	-1.32374	-0.02980

O	0.11245	1.12638	0.23953
O	0.22396	-0.31472	2.40942
C	3.00660	-0.40502	-0.08332
C	3.88726	0.57634	-0.76041
C	4.79838	0.16442	-1.65176
H	5.44041	0.88583	-2.14430
C	4.82275	-1.24607	-2.08512
C	5.74085	-1.65975	-3.17132
H	6.44931	-0.95205	-3.58368
C	5.69511	-2.90629	-3.65227
H	6.36465	-3.20662	-4.44786
C	4.71650	-3.86992	-3.11525
H	4.66245	-4.86762	-3.53151
C	3.88620	-3.50834	-2.13045
H	3.15516	-4.22344	-1.77881
C	3.95208	-2.13676	-1.55275
C	3.05733	-1.71539	-0.42755
C	0.87881	-2.51527	0.37133
C	-0.01379	-3.60657	0.83380
C	0.51512	-4.75620	1.27727
H	-0.13097	-5.56974	1.58534
C	1.98089	-4.92510	1.34902
C	2.54972	-6.15990	1.93652
H	1.89438	-6.94910	2.28361
C	3.87487	-6.30424	2.04430
H	4.28972	-7.20603	2.47575
C	4.77573	-5.22835	1.58863
H	5.84625	-5.34112	1.70172
C	4.27221	-4.11174	1.05010
H	4.94986	-3.32523	0.74589
C	2.80143	-3.95049	0.89091
C	2.21385	-2.71518	0.28921
C	-1.48385	-3.46887	0.70411
C	-2.11267	-3.61803	-0.65354
C	-2.26892	-3.24139	1.79377
C	-3.45229	-3.52884	-0.76634
C	-3.74482	-3.20736	1.62553
C	-4.31514	-3.33562	0.41993
H	-3.92246	-3.62398	-1.73583
H	-4.37035	-3.08626	2.50027
C	-1.69966	-3.02500	3.19509
H	-0.59688	-2.90482	3.16319
C	-2.24900	-1.73817	3.83208
H	-2.20958	-0.89849	3.10713
H	-1.63945	-1.46443	4.71973
H	-3.30120	-1.86425	4.16297
C	-1.99104	-4.23671	4.08663
H	-3.08447	-4.38376	4.21596
H	-1.53085	-4.09254	5.08746
H	-1.55898	-5.15492	3.63572
C	-1.26916	-3.88266	-1.90101
H	-0.19750	-3.97727	-1.63831
C	-1.36596	-2.71915	-2.89495
H	-1.09927	-1.76462	-2.39320
H	-2.39038	-2.63346	-3.31120
H	-0.65755	-2.87758	-3.73589
C	-1.65093	-5.21190	-2.56605
H	-2.67945	-5.17712	-2.98272
H	-1.58980	-6.03772	-1.82522
H	-0.94632	-5.43727	-3.39501

C	3.66869	2.01951	-0.51609
C	4.08170	2.60274	0.64266
C	2.83914	2.80256	-1.49730
C	3.63394	3.98538	0.95196
C	2.41816	4.03457	-1.15402
C	2.80947	4.65261	0.13226
H	3.94964	4.44474	1.87955
H	1.80842	4.60885	-1.83830
C	4.98903	1.89352	1.64845
H	5.19890	0.84926	1.33770
C	6.34969	2.59470	1.72795
H	6.81678	2.63059	0.72036
H	7.02881	2.03353	2.40494
H	6.24620	3.63163	2.11214
C	4.33850	1.81119	3.03721
H	3.32067	1.37574	2.96604
H	4.26266	2.81227	3.51061
H	4.94693	1.16023	3.70072
C	2.51199	2.25753	-2.89007
H	2.91503	1.23689	-3.01986
C	3.16573	3.11375	-3.98224
H	3.00861	2.64676	-4.97806
H	4.25986	3.18609	-3.80270
H	2.73600	4.13760	-4.00252
C	1.00389	2.13060	-3.12331
H	0.55467	1.48906	-2.33781
H	0.81030	1.64888	-4.10549
H	0.51055	3.12417	-3.12335
C	2.29100	6.02594	0.51781
H	2.69953	6.32185	1.51042
C	0.76408	6.01061	0.65148
H	0.45036	5.22379	1.37100
H	0.28254	5.81301	-0.32776
H	0.40428	6.99107	1.02973
C	2.74597	7.09005	-0.48750
H	3.85304	7.07671	-0.58041
H	2.43942	8.09858	-0.13667
H	2.29903	6.91610	-1.48905
C	-5.82710	-3.31647	0.28269
H	-6.29645	-3.12960	1.27495
C	-6.28443	-2.18664	-0.64555
H	-5.83918	-1.21976	-0.32638
H	-7.39046	-2.08990	-0.60965
H	-5.98818	-2.39489	-1.69338
C	-6.34444	-4.67271	-0.20968
H	-6.01592	-5.47825	0.48147
H	-5.96693	-4.89960	-1.22948
H	-7.45508	-4.66951	-0.23615
H	-0.53620	1.71055	0.77686
C	-2.02047	2.93067	2.64385
C	-2.19103	3.62801	0.42393
C	-1.45289	2.05464	3.60365
C	-2.96927	3.91493	3.06521
C	-3.13961	4.62200	0.76151
C	-1.82068	2.16409	4.92676
H	-0.75216	1.29021	3.28105
C	-3.31773	4.00131	4.43780
C	-3.51885	4.76127	2.07402
H	-3.53968	5.26684	-0.00962
C	-2.75334	3.14129	5.35136

H	-1.38937	1.48412	5.65512
H	-4.03730	4.75338	4.74958
H	-4.24260	5.51935	2.36159
H	-3.02428	3.20671	6.40075
C	-1.71718	3.37289	-1.00061
H	-0.64884	3.17062	-0.96530
C	-2.40813	2.19156	-1.68698
H	-2.18141	1.30753	-1.08073
H	-1.93814	2.03758	-2.67071
N	-3.84367	2.37604	-1.77584
N	-1.65018	2.82128	1.32716
C	-4.61478	1.45829	-2.50237
C	-5.93227	1.80795	-2.85540
C	-4.13256	0.19328	-2.87863
C	-6.73478	0.92592	-3.56856
H	-6.31926	2.78051	-2.56024
C	-4.94738	-0.68011	-3.60106
H	-3.12602	-0.11152	-2.61666
C	-6.24869	-0.32746	-3.95167
H	-7.74735	1.22105	-3.82944
H	-4.55668	-1.65181	-3.88839
H	-6.87503	-1.01531	-4.51054
H	-4.09586	3.33378	-1.98911
Cl	-1.87437	4.86683	-2.05396

Catalyst (TRIP, 3)-Product (R) alternative conformation
Electronic energy (Hartrees): -1870.559018
Free Energy at 1M concentration and 253 K (Hartrees): -1869.336984
Coordinates:

P	0.08606	-0.36920	1.19884
O	1.00287	-1.71589	1.29372
O	-1.12539	-0.71264	0.15499
O	0.92449	0.63817	0.31072
O	-0.30551	0.07320	2.55048
C	0.98591	-2.57896	0.22479
C	2.26790	-2.85727	-0.46873
C	2.35548	-3.87424	-1.33661
H	3.29039	-4.07178	-1.84814
C	1.14911	-4.62800	-1.72351
C	1.24408	-5.65844	-2.78339
H	2.20821	-5.92414	-3.19897
C	0.14017	-6.25955	-3.23777
H	0.21030	-7.00974	-4.01479
C	-1.17966	-5.88307	-2.69964
H	-2.07322	-6.34525	-3.09910
C	-1.27574	-4.95826	-1.73770
H	-2.25968	-4.67227	-1.39361
C	-0.05161	-4.31252	-1.18407
C	-0.12470	-3.29332	-0.08753
C	-1.90341	-1.75293	0.59280
C	-3.32795	-1.50041	0.91677
C	-4.10073	-2.50815	1.35032
H	-5.15117	-2.33993	1.55623
C	-3.52551	-3.85481	1.55114
C	-4.35720	-4.93210	2.13502
H	-5.39049	-4.74047	2.39632
C	-3.83472	-6.14560	2.34429
H	-4.44269	-6.93210	2.77235
C	-2.42416	-6.41356	2.00265
H	-2.00255	-7.39215	2.19265

C	-1.66577	-5.44859	1.46955
H	-0.62859	-5.65830	1.24292
C	-2.24064	-4.10296	1.20425
C	-1.41840	-3.01367	0.59906
C	-3.91546	-0.16632	0.64416
C	-4.10898	0.26582	-0.78468
C	-4.27232	0.66953	1.65960
C	-4.61297	1.48881	-1.03460
C	-4.87230	1.98993	1.32948
C	-5.02563	2.39266	0.05996
H	-4.75700	1.81631	-2.05530
H	-5.19513	2.63673	2.13472
C	-4.09480	0.30648	3.13487
H	-3.54366	-0.64841	3.25381
C	-3.26252	1.35864	3.87954
H	-2.30478	1.53314	3.34921
H	-3.02887	1.00024	4.90486
H	-3.80464	2.32324	3.96583
C	-5.45777	0.11662	3.80984
H	-6.03779	1.06374	3.81560
H	-5.31964	-0.21992	4.85958
H	-6.04448	-0.65834	3.27241
C	-3.78953	-0.66317	-1.95715
H	-3.39499	-1.63230	-1.59608
C	-2.70819	-0.06779	-2.86929
H	-1.82756	0.24962	-2.27135
H	-3.09690	0.80762	-3.42930
H	-2.37148	-0.82912	-3.60486
C	-5.05451	-1.00508	-2.75524
H	-5.47602	-0.10573	-3.25175
H	-5.82378	-1.43618	-2.07925
H	-4.81828	-1.75854	-3.53688
C	3.40247	-1.91439	-0.33060
C	4.20163	-1.92400	0.77118
C	3.68643	-0.95524	-1.45599
C	5.35069	-0.98196	0.83604
C	4.75546	-0.14284	-1.36602
C	5.62049	-0.13497	-0.16776
H	5.99209	-0.99513	1.70750
H	4.99807	0.52540	-2.18064
C	3.99771	-2.89497	1.93354
H	3.10009	-3.52806	1.78169
C	5.18272	-3.86073	2.04860
H	5.32606	-4.40017	1.08780
H	4.98487	-4.61271	2.84217
H	6.12031	-3.32203	2.30103
C	3.77442	-2.14208	3.25188
H	2.95802	-1.39795	3.13763
H	4.69509	-1.61494	3.57783
H	3.48251	-2.85670	4.05075
C	2.81121	-0.90581	-2.71046
H	1.91530	-1.54567	-2.58776
C	3.57335	-1.43943	-3.92925
H	2.89988	-1.48033	-4.81191
H	3.94289	-2.46723	-3.72808
H	4.43989	-0.78949	-4.17502
C	2.26868	0.50295	-2.98300
H	1.75839	0.89784	-2.08283
H	1.52702	0.46847	-3.80972
H	3.07890	1.20363	-3.27332

C	6.81331	0.80116	-0.08763
H	7.29520	0.71577	0.91251
C	6.38796	2.26606	-0.25499
H	5.58962	2.52227	0.47054
H	6.01192	2.46333	-1.28086
H	7.25403	2.93539	-0.06556
C	7.86855	0.41862	-1.13098
H	8.17833	-0.63882	-0.98816
H	8.76727	1.06172	-1.01731
H	7.47388	0.54024	-2.16226
C	-5.66080	3.73686	-0.25065
H	-5.89751	4.27297	0.69605
C	-4.70355	4.63474	-1.04343
H	-3.76510	4.78887	-0.47255
H	-5.16791	5.62972	-1.21185
H	-4.45705	4.19079	-2.03079
C	-6.98411	3.55124	-1.00201
H	-7.66492	2.89616	-0.41726
H	-6.81805	3.09574	-2.00130
H	-7.48372	4.53373	-1.14098
H	1.01006	1.56865	0.71295
C	2.57196	3.06476	1.99985
C	0.72852	4.21109	1.14852
C	3.27333	1.84202	2.17306
C	3.08808	4.25267	2.60649
C	1.18894	5.43294	1.70646
C	4.41987	1.81047	2.93537
H	2.89206	0.94259	1.70571
C	4.28478	4.18670	3.36440
C	2.35771	5.45191	2.42310
H	0.60121	6.33361	1.56698
C	4.93505	2.98612	3.53103
H	4.94196	0.87145	3.08145
H	4.66920	5.09652	3.81693
H	2.72732	6.37511	2.86122
H	5.84488	2.93478	4.12079
C	-0.60785	4.22255	0.43042
H	-0.78608	5.23067	0.05410
C	-0.80574	3.23662	-0.72387
H	-1.80836	3.40800	-1.11180
H	-0.77093	2.19901	-0.37781
N	0.17024	3.53287	-1.75719
N	1.40565	3.07918	1.27459
C	-0.08242	3.37677	-3.12508
C	-1.36758	3.19889	-3.66405
C	1.00760	3.44359	-4.01529
C	-1.54369	3.07839	-5.04282
H	-2.23601	3.13926	-3.01865
C	0.82064	3.31410	-5.38601
H	2.00743	3.59557	-3.61521
C	-0.45903	3.12900	-5.91627
H	-2.54808	2.93255	-5.43088
H	1.68346	3.35948	-6.04485
H	-0.60479	3.02660	-6.98676
H	1.11041	3.26473	-1.49759
Cl	-1.92026	3.95191	1.68727

Catalyst (TRIP, 3)-Product (R) alternative conformation
Electronic energy (Hartrees): -1870.55706
Free Energy at 1M concentration and 253 K (Hartrees): -1869.336341

Coordinates:

P	-0.35536	0.08453	-0.55902
O	0.26503	1.53822	-0.96878
O	-1.64022	0.35810	0.42747
O	0.64597	-0.53817	0.50208
O	-0.63029	-0.69627	-1.78024
C	0.21773	2.52295	-0.01355
C	1.49155	3.14646	0.42881
C	1.46922	4.18923	1.27152
H	2.39543	4.65816	1.58191
C	0.19039	4.68612	1.81324
C	0.19302	5.77060	2.82207
H	1.12645	6.22592	3.12894
C	-0.95656	6.18929	3.36166
H	-0.95365	6.98022	4.10050
C	-2.23147	5.56364	2.96279
H	-3.15584	5.88974	3.42190
C	-2.24493	4.58736	2.04796
H	-3.18910	4.12555	1.79210
C	-0.97691	4.13208	1.41350
C	-0.96445	3.05113	0.38092
C	-2.54592	1.24101	-0.10292
C	-3.92159	0.77785	-0.42006
C	-4.80804	1.63680	-0.94593
H	-5.82263	1.31505	-1.15106
C	-4.42122	3.03303	-1.22893
C	-5.38386	3.94397	-1.89009
H	-6.37433	3.59241	-2.15112
C	-5.03316	5.20391	-2.16988
H	-5.73641	5.86862	-2.65473
C	-3.68207	5.68890	-1.82873
H	-3.39762	6.70338	-2.07665
C	-2.80730	4.87542	-1.22583
H	-1.81475	5.24488	-1.00466
C	-3.19227	3.47951	-0.88251
C	-2.23590	2.55190	-0.20812
C	-4.36860	-0.58492	-0.04471
C	-4.78491	-0.84645	1.37515
C	-4.53379	-1.55302	-0.98777
C	-5.38833	-2.01255	1.67500
C	-5.21494	-2.82075	-0.61026
C	-5.64684	-3.03744	0.64065
H	-5.70686	-2.21172	2.68894
H	-5.40602	-3.56245	-1.37473
C	-4.09676	-1.36992	-2.44004
H	-3.51460	-0.43324	-2.56712
C	-3.16767	-2.50538	-2.88978
H	-2.33527	-2.62413	-2.16752
H	-2.73297	-2.26895	-3.88434
H	-3.70970	-3.47083	-2.96822
C	-5.31513	-1.26655	-3.36391
H	-5.90595	-2.20694	-3.35522
H	-4.98662	-1.06467	-4.40579
H	-5.96964	-0.43013	-3.03927
C	-4.54636	0.18032	2.48143
H	-4.06840	1.09250	2.07575
C	-3.58502	-0.36651	3.54421
H	-2.63801	-0.69609	3.06495
H	-4.03083	-1.22647	4.08715
H	-3.34196	0.42794	4.28181

C	-5.86567	0.64551	3.11153
H	-5.67296	1.47411	3.82617
H	-6.55065	1.02162	2.32162
H	-6.36674	-0.17922	3.66045
C	2.79268	2.62877	-0.05917
C	3.22801	2.90605	-1.31983
C	3.68025	1.87007	0.88924
C	4.59387	2.47848	-1.72134
C	4.92557	1.53916	0.49639
C	5.41644	1.86191	-0.86145
H	4.94454	2.71154	-2.71831
H	5.58923	1.02136	1.17572
C	2.39957	3.70008	-2.33093
H	1.40816	3.97769	-1.92158
C	3.08879	5.02288	-2.68687
H	3.28743	5.60687	-1.76266
H	2.43003	5.62929	-3.34472
H	4.04903	4.85087	-3.21681
C	2.12459	2.86702	-3.58935
H	3.06004	2.65857	-4.14961
H	1.42863	3.41328	-4.26129
H	1.65160	1.90076	-3.31276
C	3.20570	1.49170	2.29207
H	2.12438	1.71117	2.40377
C	3.94281	2.31276	3.35706
H	5.03061	2.08742	3.35225
H	3.53693	2.08108	4.36493
H	3.80374	3.39795	3.16924
C	3.35644	-0.01098	2.56791
H	2.95480	-0.60547	1.72282
H	2.79505	-0.28591	3.48623
H	4.41954	-0.28251	2.72224
C	6.83996	1.52644	-1.27073
H	7.01820	1.84690	-2.32210
C	7.09149	0.01688	-1.21855
H	6.32921	-0.51557	-1.82476
H	7.05773	-0.35720	-0.17484
H	8.09271	-0.21551	-1.63987
C	7.84943	2.28052	-0.39769
H	7.65619	3.37340	-0.45081
H	8.88210	2.09282	-0.76179
H	7.78459	1.95583	0.66257
C	-6.44014	-4.28926	0.97657
H	-6.50550	-4.94839	0.08161
C	-5.76690	-5.10872	2.08473
H	-4.74166	-5.39938	1.78042
H	-6.34246	-6.04093	2.26875
H	-5.71313	-4.53857	3.03564
C	-7.87603	-3.92343	1.36924
H	-7.89407	-3.32151	2.30280
H	-8.35559	-3.33805	0.55562
H	-8.47408	-4.84561	1.53097
H	0.93002	-1.47007	0.22245
C	-0.06003	-3.70362	-0.42369
C	2.00714	-3.23901	-1.38265
C	-1.01578	-3.41773	0.58286
C	-0.33721	-4.71877	-1.38807
C	1.80365	-4.21995	-2.38870
C	-2.19931	-4.11684	0.62365
H	-0.80169	-2.63306	1.29961

C	-1.56746	-5.42071	-1.31761
C	0.64014	-4.94800	-2.38853
H	2.55286	-4.36462	-3.15903
C	-2.48090	-5.12402	-0.33154
H	-2.92393	-3.88249	1.39560
H	-1.77994	-6.18621	-2.05874
H	0.45403	-5.69677	-3.15393
H	-3.42577	-5.65645	-0.29235
C	3.29480	-2.44949	-1.30508
H	3.23646	-1.77087	-0.45508
C	4.54780	-3.34377	-1.19790
H	5.43676	-2.70683	-1.18488
H	4.61998	-3.96257	-2.09790
N	4.51345	-4.19291	-0.02820
N	1.10706	-2.99064	-0.44819
C	5.20325	-3.92220	1.15067
C	5.65933	-2.63166	1.46950
C	5.44617	-4.96394	2.06628
C	6.36348	-2.40575	2.65173
H	5.44567	-1.80017	0.80802
C	6.13547	-4.72142	3.24938
H	5.09471	-5.96762	1.83741
C	6.60911	-3.44190	3.55137
H	6.71354	-1.40072	2.87064
H	6.31099	-5.54365	3.93756
H	7.15284	-3.25729	4.47208
H	4.30575	-5.16523	-0.19234
Cl	3.48389	-1.37478	-2.77747

Catalyst (TRIP, 3)-Product (S) alternative conformation
Electronic energy (Hartrees): -1870.557166
Free Energy at 1M concentration and 253 K (Hartrees): -1869.336243
Coordinates:

P	-0.86706	0.01000	0.76108
O	-0.77300	-1.60806	1.02620
O	-2.03673	0.22841	-0.35517
O	0.39445	0.39487	-0.11368
O	-1.03187	0.68802	2.06080
C	-1.08813	-2.43212	-0.02758
C	-0.04445	-3.35238	-0.54203
C	-0.36635	-4.31447	-1.41747
H	0.39606	-4.98982	-1.78759
C	-1.73143	-4.38808	-1.97181
C	-2.03862	-5.38155	-3.02666
H	-1.28733	-6.09827	-3.33437
C	-3.24244	-5.39453	-3.60797
H	-3.46513	-6.11922	-4.38048
C	-4.26422	-4.40736	-3.21283
H	-5.22590	-4.40280	-3.70960
C	-3.99882	-3.50933	-2.25720
H	-4.75373	-2.77541	-2.01077
C	-2.67714	-3.50671	-1.56979
C	-2.36113	-2.52948	-0.48028
C	-3.23908	-0.30091	0.04228
C	-4.37650	0.61014	0.32261
C	-5.55292	0.10463	0.72221
H	-6.39721	0.76118	0.89583
C	-5.71013	-1.34990	0.92593
C	-6.98262	-1.88377	1.46377
H	-7.80428	-1.21334	1.68319

C	-7.12136	-3.19566	1.68382
H	-8.05094	-3.58485	2.07869
C	-6.00306	-4.11576	1.40097
H	-6.11453	-5.17345	1.60198
C	-4.85139	-3.64425	0.90924
H	-4.03655	-4.33308	0.72987
C	-4.69221	-2.19145	0.62987
C	-3.41926	-1.64048	0.07545
C	-4.24442	2.06206	0.05770
C	-4.24553	2.54544	-1.36626
C	-4.14053	2.95843	1.07862
C	-4.12823	3.86489	-1.60740
C	-4.06814	4.40881	0.76088
C	-4.04262	4.84695	-0.50528
H	-4.11888	4.23076	-2.62512
H	-4.02813	5.12378	1.57215
C	-4.09372	2.53930	2.54786
H	-3.98352	1.43945	2.64686
C	-2.88308	3.14949	3.27124
H	-1.96168	3.01692	2.66721
H	-2.73544	2.64659	4.25088
H	-3.02540	4.23413	3.46034
C	-5.39642	2.92456	3.25701
H	-5.54106	4.02597	3.25528
H	-5.37646	2.57021	4.30977
H	-6.26262	2.45099	2.74869
C	-4.39780	1.58022	-2.54233
H	-4.51302	0.53863	-2.18414
C	-3.15295	1.59267	-3.43938
H	-2.24451	1.38377	-2.83556
H	-3.02916	2.57392	-3.94410
H	-3.23869	0.80527	-4.21843
C	-5.66537	1.88227	-3.35269
H	-5.59859	2.86863	-3.85805
H	-6.55245	1.87973	-2.68360
H	-5.81467	1.10139	-4.12881
C	1.36687	-3.14807	-0.14702
C	1.82013	-3.56332	1.06692
C	2.29608	-2.44340	-1.09795
C	3.21396	-3.23282	1.46608
C	3.55162	-2.17039	-0.69628
C	4.03701	-2.56272	0.64583
H	3.56828	-3.54114	2.44106
H	4.24288	-1.69037	-1.37314
C	0.97133	-4.40982	2.01754
H	-0.04488	-4.58575	1.60884
C	1.59596	-5.79745	2.20557
H	1.73032	-6.28952	1.21838
H	0.92772	-6.43589	2.82224
H	2.58258	-5.73076	2.71085
C	0.77376	-3.71489	3.36905
H	0.31616	-2.71521	3.22219
H	1.73672	-3.60026	3.90824
H	0.08788	-4.31314	4.00608
C	1.88175	-2.14214	-2.54110
H	0.78938	-2.27437	-2.66590
C	2.56213	-3.11995	-3.50627
H	2.19951	-2.94938	-4.54247
H	2.32118	-4.16642	-3.22287
H	3.66563	-2.99109	-3.48927

C	2.16339	-0.69297	-2.95797
H	1.76287	0.01109	-2.20403
H	1.66539	-0.47603	-3.92711
H	3.24962	-0.51368	-3.08885
C	5.45965	-2.24498	1.07438
H	5.66089	-2.68424	2.07751
C	5.66699	-0.73279	1.20168
H	4.99450	-0.32778	1.98388
H	5.45573	-0.21663	0.24383
H	6.71324	-0.51130	1.50188
C	6.48062	-2.85914	0.10841
H	6.29129	-3.94799	-0.00515
H	7.50835	-2.72614	0.50872
H	6.43219	-2.37801	-0.89111
C	-3.93829	6.33171	-0.80349
H	-3.88670	6.90793	0.14788
C	-2.65587	6.64332	-1.58368
H	-1.77048	6.27304	-1.02361
H	-2.54852	7.74120	-1.71537
H	-2.67009	6.16794	-2.58731
C	-5.17646	6.82763	-1.55925
H	-6.09531	6.58775	-0.98247
H	-5.24998	6.35852	-2.56320
H	-5.12619	7.92988	-1.68858
H	1.17297	0.73569	0.44747
C	2.68937	1.09734	2.57405
C	3.05146	2.50258	0.75231
C	2.08448	-0.06706	3.10887
C	3.55361	1.87958	3.40109
C	3.92106	3.32942	1.50477
C	2.32017	-0.42290	4.41760
H	1.44390	-0.66671	2.47553
C	3.77341	1.48476	4.74507
C	4.16019	3.01923	2.81978
H	4.37666	4.19158	1.03720
C	3.16623	0.35539	5.24323
H	1.84699	-1.30733	4.82958
H	4.42980	2.08605	5.36792
H	4.82135	3.63623	3.42225
H	3.33487	0.05455	6.27256
C	2.81019	2.71105	-0.73112
C	3.90707	2.07722	-1.59722
H	3.59108	2.10988	-2.65207
H	3.94081	1.02619	-1.29494
N	5.20517	2.66763	-1.36264
N	2.45272	1.43965	1.26826
C	6.38834	2.05019	-1.78562
C	6.43651	0.73377	-2.27436
C	7.59643	2.76667	-1.67818
C	7.65867	0.15016	-2.61150
H	5.52767	0.15880	-2.39994
C	8.80557	2.17938	-2.03046
H	7.57539	3.78637	-1.30091
C	8.85078	0.86117	-2.49444
H	7.66797	-0.87211	-2.97948
H	9.72205	2.75523	-1.93616
H	9.79702	0.40253	-2.76264
H	5.20972	3.67336	-1.47793
H	1.85244	2.26673	-1.00046
Cl	2.63391	4.48930	-1.14547

Catalyst (TRIP, 3)-Product (R) alternative conformation
Electronic energy (Hartrees): -1870.558767
Free Energy at 1M concentration and 253 K (Hartrees): -1869.336167
Coordinates:

P	0.26787	0.40129	-0.88940
O	1.86332	0.69713	-1.02333
O	-0.29701	1.37792	0.28511
O	0.09740	-1.01236	-0.18396
O	-0.37423	0.54314	-2.21304
C	2.56104	1.23923	0.03446
C	3.84878	0.59300	0.40829
C	4.66785	1.17611	1.29523
H	5.61188	0.70910	1.55260
C	4.24728	2.39497	2.00624
C	5.09364	2.93775	3.09400
H	6.04789	2.47898	3.32146
C	4.68095	3.99306	3.80357
H	5.29865	4.38774	4.59991
C	3.37278	4.61030	3.51606
H	3.03023	5.44486	4.11418
C	2.60440	4.13060	2.53139
H	1.63516	4.57980	2.36115
C	3.06783	2.98168	1.70363
C	2.24079	2.44559	0.57602
C	-0.12184	2.70175	-0.02335
C	-1.30733	3.54424	-0.32255
C	-1.15646	4.86274	-0.51829
H	-2.02184	5.49061	-0.69189
C	0.19483	5.45535	-0.57438
C	0.35560	6.88427	-0.92914
H	-0.51618	7.50553	-1.09285
C	1.57789	7.41235	-1.05375
H	1.69460	8.45610	-1.31538
C	2.77185	6.56948	-0.85233
H	3.75802	6.99638	-0.98192
C	2.63740	5.27894	-0.52563
H	3.52609	4.67282	-0.41050
C	1.28445	4.68510	-0.34607
C	1.10659	3.25200	0.04321
C	-2.65118	2.92128	-0.34282
C	-3.22385	2.40498	0.94638
C	-3.33199	2.74023	-1.51097
C	-4.32654	1.63430	0.90778
C	-4.58174	1.93542	-1.49284
C	-5.02670	1.35868	-0.36670
H	-4.75093	1.25022	1.82555
H	-5.12463	1.78540	-2.41621
C	-2.88184	3.35192	-2.84149
H	-1.85237	3.75477	-2.77282
C	-2.83818	2.32549	-3.98138
H	-2.28420	1.42031	-3.66777
H	-2.31420	2.76073	-4.85917
H	-3.85783	2.03513	-4.30807
C	-3.79740	4.52277	-3.21573
H	-4.84030	4.17491	-3.37671
H	-3.43597	5.00898	-4.14704
H	-3.79813	5.28294	-2.40628
C	-2.64141	2.82828	2.29595
H	-1.73709	3.45252	2.14962

C	-2.20372	1.62613	3.14142
H	-1.55036	0.95160	2.55033
H	-3.08174	1.05604	3.50724
H	-1.62807	1.97567	4.02489
C	-3.63499	3.70490	3.06900
H	-4.54896	3.13477	3.33972
H	-3.92815	4.58071	2.45121
H	-3.16486	4.08103	4.00279
C	4.22879	-0.70623	-0.19351
C	4.64402	-0.78556	-1.48898
C	4.14729	-1.95213	0.64572
C	4.96470	-2.11848	-2.06309
C	4.44041	-3.13870	0.08009
C	4.86237	-3.23791	-1.33370
H	5.29148	-2.18249	-3.09281
H	4.38886	-4.04622	0.66611
C	4.84083	0.44518	-2.37579
H	4.54494	1.37675	-1.85250
C	6.32051	0.61875	-2.73761
H	6.93180	0.68145	-1.81181
H	6.46424	1.55891	-3.31196
H	6.68804	-0.22909	-3.35340
C	3.97530	0.36727	-3.64119
H	2.91445	0.17984	-3.37398
H	4.31883	-0.44192	-4.31890
H	4.02751	1.32920	-4.19457
C	3.77174	-1.90170	2.12822
H	3.56113	-0.86498	2.45086
C	4.92426	-2.39869	3.01069
H	4.67687	-2.24369	4.08267
H	5.84955	-1.82729	2.78321
H	5.11950	-3.47980	2.84930
C	2.48555	-2.67992	2.41090
H	1.65407	-2.23883	1.82477
H	2.21965	-2.60473	3.48696
H	2.59517	-3.75306	2.14977
C	5.19813	-4.58632	-1.94492
H	5.47566	-4.45983	-3.01578
C	3.98626	-5.52378	-1.90628
H	3.11155	-5.03790	-2.38958
H	3.71993	-5.78920	-0.86171
H	4.21289	-6.46034	-2.45906
C	6.40427	-5.21939	-1.24188
H	7.27283	-4.52757	-1.28043
H	6.68978	-6.16343	-1.75325
H	6.17548	-5.44913	-0.17960
C	-6.25472	0.46698	-0.39564
H	-6.62032	0.35855	-1.44186
C	-5.92042	-0.94259	0.10611
H	-5.05604	-1.35635	-0.45404
H	-6.78935	-1.61712	-0.04805
H	-5.67197	-0.93153	1.18658
C	-7.39795	1.08246	0.41875
H	-7.62647	2.10251	0.04232
H	-7.13480	1.14784	1.49579
H	-8.31357	0.46164	0.31686
H	-0.52012	-1.64981	-0.69163
C	-2.66687	-2.73262	-1.95924
C	-1.33810	-4.11978	-0.64546
C	-2.89431	-1.46311	-2.54779

C	-3.61515	-3.78301	-2.17265
C	-2.21001	-5.22016	-0.84150
C	-4.03423	-1.25401	-3.29420
H	-2.15546	-0.67878	-2.41377
C	-4.77641	-3.52808	-2.94451
C	-3.34298	-5.04578	-1.59296
H	-1.96959	-6.17675	-0.39295
C	-4.98508	-2.28277	-3.49003
H	-4.21105	-0.28470	-3.74504
H	-5.49291	-4.33105	-3.09374
H	-4.03925	-5.86554	-1.74835
H	-5.87671	-2.08586	-4.07727
C	-0.16492	-4.23183	0.30427
H	0.52415	-3.40986	0.11879
C	-0.63116	-4.24040	1.77447
H	0.23799	-4.41337	2.42505
H	-1.30485	-5.09168	1.91466
N	-1.35036	-3.02549	2.05948
N	-1.54675	-2.92838	-1.18701
C	-2.44215	-2.93410	2.91115
C	-3.26433	-1.79169	2.85072
C	-2.76825	-3.94524	3.83372
C	-4.35942	-1.66068	3.69635
H	-3.04234	-1.02517	2.11349
C	-3.88406	-3.80976	4.66044
H	-2.13947	-4.82447	3.92694
C	-4.68502	-2.67017	4.60697
H	-4.97499	-0.76744	3.63330
H	-4.11515	-4.60344	5.36588
H	-5.54589	-2.56798	5.25985
H	-0.99647	-2.16629	1.66271
Cl	0.81315	-5.74353	-0.00327

Catalyst (TRIP, 3)-Product (S) alternative conformation
Electronic energy (Hartrees): -1870.556508
Free Energy at 1M concentration and 253 K (Hartrees): -1869.336091
Coordinates:

P	-0.77002	-0.11054	0.80259
O	-0.66059	-1.73550	1.00004
O	-1.91323	0.14176	-0.33373
O	0.50257	0.31985	-0.03397
O	-0.96388	0.51641	2.12323
C	-0.85757	-2.50006	-0.12389
C	0.26048	-3.34528	-0.61308
C	0.04702	-4.24305	-1.58511
H	0.85805	-4.87221	-1.93186
C	-1.26448	-4.31566	-2.25712
C	-1.44565	-5.22913	-3.40898
H	-0.64222	-5.88933	-3.71138
C	-2.59944	-5.23884	-4.08432
H	-2.72995	-5.90478	-4.92753
C	-3.69131	-4.32732	-3.69461
H	-4.61151	-4.31826	-4.26447
C	-3.53997	-3.50243	-2.65212
H	-4.34344	-2.82114	-2.40786
C	-2.27617	-3.50688	-1.86376
C	-2.08732	-2.61072	-0.68002
C	-3.10467	-0.47197	-0.04101
C	-4.29840	0.36417	0.23765
C	-5.47635	-0.22140	0.49951

H	-6.35909	0.38302	0.67075
C	-5.58059	-1.69354	0.56218
C	-6.86259	-2.32363	0.95318
H	-7.72865	-1.70884	1.16466
C	-6.95545	-3.65432	1.04982
H	-7.89222	-4.11396	1.33732
C	-5.77742	-4.50000	0.77749
H	-5.85349	-5.57488	0.87937
C	-4.61617	-3.94096	0.41782
H	-3.75891	-4.57796	0.24484
C	-4.50526	-2.46397	0.27514
C	-3.22331	-1.81559	-0.13434
C	-4.20695	1.84004	0.13324
C	-4.09803	2.46954	-1.22841
C	-4.22485	2.62781	1.24530
C	-3.99729	3.80888	-1.32187
C	-4.16620	4.10450	1.08463
C	-4.03534	4.67204	-0.12213
H	-3.91093	4.27914	-2.29197
H	-4.21889	4.73207	1.96455
C	-4.29901	2.06017	2.66299
H	-4.16212	0.95899	2.66120
C	-3.18116	2.61951	3.55663
H	-2.20433	2.57196	3.03321
H	-3.10828	2.02084	4.48973
H	-3.37580	3.67488	3.84076
C	-5.67327	2.34019	3.28067
H	-5.85241	3.43233	3.37655
H	-5.73897	1.87912	4.28932
H	-6.47353	1.90277	2.64737
C	-4.12562	1.63080	-2.50652
H	-4.23116	0.55442	-2.26815
C	-2.81802	1.77234	-3.29767
H	-1.94907	1.53527	-2.64819
H	-2.69600	2.80187	-3.69517
H	-2.81381	1.06347	-4.15308
C	-5.33803	1.98367	-3.37825
H	-5.26966	3.02150	-3.76699
H	-6.27281	1.88192	-2.78627
H	-5.39858	1.28925	-4.24344
C	1.62269	-3.15797	-0.06085
C	1.94886	-3.63538	1.17214
C	2.65462	-2.43199	-0.88308
C	3.31200	-3.37841	1.70755
C	3.88172	-2.23885	-0.36386
C	4.23047	-2.71130	0.99391
H	3.56603	-3.74332	2.69423
H	4.64663	-1.74953	-0.94994
C	0.98752	-4.47656	2.01402
H	-0.00105	-4.58070	1.52267
C	1.52547	-5.90213	2.18348
H	1.70209	-6.36104	1.18704
H	0.78425	-6.52698	2.72625
H	2.47738	-5.90895	2.75535
C	0.72039	-3.82905	3.37830
H	0.39650	-2.77519	3.24917
H	1.62523	-3.85385	4.01991
H	-0.09060	-4.37684	3.90401
C	2.36784	-1.98979	-2.32036
H	1.28488	-2.06943	-2.53985

C	3.09625	-2.89938	-3.31699
H	2.82049	-2.62414	-4.35741
H	2.80805	-3.95869	-3.15112
H	4.19800	-2.80904	-3.20526
C	2.72910	-0.51959	-2.57429
H	2.30042	0.12639	-1.78536
H	2.31430	-0.19150	-3.55126
H	3.82731	-0.37256	-2.60503
C	5.62119	-2.47590	1.55736
H	5.69707	-2.91518	2.57766
C	5.91585	-0.97784	1.69504
H	5.17529	-0.50601	2.37097
H	5.87881	-0.46669	0.71129
H	6.92447	-0.82597	2.13490
C	6.68451	-3.16549	0.69453
H	6.44707	-4.24573	0.58862
H	7.68134	-3.07579	1.17667
H	6.74126	-2.70862	-0.31617
C	-3.93622	6.18080	-0.25599
H	-3.98695	6.65595	0.74972
C	-2.59275	6.58623	-0.87349
H	-1.75666	6.17280	-0.26962
H	-2.49805	7.69304	-0.88461
H	-2.50047	6.21407	-1.91583
C	-5.10814	6.73776	-1.07245
H	-6.07184	6.42708	-0.61499
H	-5.07570	6.37613	-2.12197
H	-5.07073	7.84803	-1.08158
H	1.22034	0.75658	0.54194
C	2.76979	1.12740	2.61097
C	2.72366	2.79678	0.98634
C	2.39893	-0.17842	3.01709
C	3.55406	1.94145	3.48428
C	3.48226	3.67446	1.80175
C	2.80667	-0.65416	4.24315
H	1.80180	-0.78779	2.34963
C	3.95462	1.42031	4.74015
C	3.89246	3.24428	3.03791
H	3.72385	4.66730	1.44050
C	3.58962	0.14556	5.10956
H	2.52560	-1.65372	4.55477
H	4.55279	2.04150	5.40084
H	4.48029	3.89150	3.68310
H	3.89913	-0.25228	6.07094
C	2.30796	3.18040	-0.41731
C	3.49470	3.47567	-1.34579
H	3.95580	4.42429	-1.04354
H	3.10577	3.61916	-2.36526
N	4.47533	2.40929	-1.26565
N	2.37899	1.58046	1.37895
C	5.54253	2.33228	-2.15788
C	5.90803	3.41326	-2.97978
C	6.30818	1.15140	-2.22284
C	7.00663	3.30615	-3.83398
H	5.34130	4.33744	-2.95544
C	7.39617	1.05755	-3.08162
H	6.04126	0.30754	-1.59286
C	7.75821	2.13441	-3.89654
H	7.26978	4.15426	-4.46013
H	7.96598	0.13304	-3.11456

H	8.60845	2.05812	-4.56657
H	4.13004	1.51847	-0.93907
H	1.68680	2.38430	-0.82716
Cl	1.22414	4.65197	-0.39155

Catalyst (TRIP, 3)-Product (S) alternative conformation
Electronic energy (Hartrees): -1870.558457
Free Energy at 1M concentration and 253 K (Hartrees): -1869.336077
Coordinates:

P	0.28693	-0.13552	-0.24460
O	-1.10263	0.67957	0.01131
O	1.40507	0.93361	-0.76157
O	0.10868	-0.97638	-1.56194
O	0.64089	-0.88406	0.98247
C	-1.40409	1.73084	-0.82327
C	-2.76805	1.77934	-1.42808
C	-3.15579	2.87042	-2.10511
H	-4.17885	2.96350	-2.45065
C	-2.20022	3.95804	-2.38236
C	-2.58720	5.07010	-3.28052
H	-3.57595	5.09430	-3.72154
C	-1.71522	6.04688	-3.55376
H	-1.99723	6.85831	-4.21216
C	-0.36162	6.01587	-2.96668
H	0.34570	6.79922	-3.20664
C	-0.00604	5.01859	-2.14842
H	0.99715	5.00328	-1.74258
C	-0.97444	3.93930	-1.81496
C	-0.61117	2.83053	-0.88405
C	1.51418	2.02982	0.04903
C	2.75689	2.23095	0.83511
C	2.91636	3.35373	1.55063
H	3.83525	3.52499	2.09785
C	1.82510	4.34436	1.63854
C	1.97395	5.52174	2.52471
H	2.89963	5.68403	3.06281
C	0.96451	6.38681	2.66831
H	1.07320	7.24555	3.31811
C	-0.30763	6.16335	1.95589
H	-1.13134	6.85048	2.10059
C	-0.44990	5.10684	1.14740
H	-1.40341	4.94683	0.66269
C	0.67777	4.15562	0.94544
C	0.55160	2.97439	0.03720
C	3.82958	1.20694	0.80826
C	4.69238	1.07947	-0.41669
C	4.05345	0.39708	1.88320
C	5.66923	0.15242	-0.43313
C	5.18584	-0.56396	1.83865
C	5.94536	-0.69986	0.74348
H	6.29602	0.04125	-1.30744
H	5.39724	-1.16831	2.71094
C	3.19476	0.43864	3.14918
H	2.27242	1.03364	2.98376
C	2.71997	-0.96322	3.56722
H	2.35100	-1.53241	2.69076
H	1.89199	-0.87766	4.30333
H	3.53838	-1.54638	4.03923
C	3.96422	1.10282	4.29650
H	4.87336	0.51920	4.55560

H	3.31812	1.17436	5.19747
H	4.27281	2.12957	4.01082
C	4.51365	2.00003	-1.62388
H	3.68417	2.71400	-1.45283
C	4.14804	1.20817	-2.88642
H	4.97667	0.53898	-3.19957
H	3.24208	0.59493	-2.70264
H	3.92694	1.90650	-3.72160
C	5.76351	2.85909	-1.85633
H	6.63469	2.23563	-2.14850
H	6.01603	3.41918	-0.93050
H	5.57300	3.59593	-2.66575
C	-3.78079	0.76780	-1.03763
C	-4.33539	0.81828	0.21021
C	-4.21510	-0.31178	-2.00455
C	-5.30537	-0.23005	0.61799
C	-5.11607	-1.22420	-1.58781
C	-5.68805	-1.19478	-0.22606
H	-5.72856	-0.19686	1.61359
H	-5.45207	-2.00431	-2.25882
C	-4.04645	1.94443	1.20931
H	-3.32933	2.68537	0.80448
C	-5.32137	2.73764	1.52118
H	-5.76991	3.11899	0.57883
H	-5.07853	3.60913	2.16624
H	-6.07102	2.11103	2.04836
C	-3.41432	1.39909	2.49447
H	-2.50706	0.80880	2.25213
H	-4.12535	0.75492	3.05106
H	-3.11199	2.23858	3.15625
C	-3.71002	-0.43578	-3.44844
H	-4.18568	-1.32561	-3.92099
C	-4.11603	0.74611	-4.33878
H	-3.36384	1.55798	-4.31267
H	-5.11061	1.14162	-4.04115
H	-4.18389	0.41098	-5.39615
C	-2.20912	-0.71286	-3.52431
H	-1.61597	0.16151	-3.19328
H	-1.92018	-0.94388	-4.57194
H	-1.96369	-1.59679	-2.89941
C	-6.73201	-2.21510	0.19491
H	-7.01986	-2.04786	1.25753
C	-6.18542	-3.64406	0.10237
H	-5.26960	-3.73950	0.71826
H	-5.94415	-3.91922	-0.94583
H	-6.93701	-4.36407	0.49064
C	-8.00664	-2.06310	-0.64282
H	-8.39246	-1.02418	-0.56301
H	-8.79231	-2.75402	-0.26920
H	-7.81365	-2.29313	-1.71217
C	7.08544	-1.70184	0.71978
H	7.14985	-2.22827	1.69883
C	6.84490	-2.77443	-0.34692
H	5.86998	-3.27511	-0.16468
H	7.64370	-3.54493	-0.30046
H	6.83961	-2.33259	-1.36580
C	8.43089	-0.99990	0.50196
H	8.58166	-0.21792	1.27672
H	8.48057	-0.52612	-0.50119
H	9.26004	-1.73462	0.58496

H	0.39343	-1.96431	-1.45740
C	2.16127	-3.72483	-1.76084
C	0.18995	-4.37942	-0.69560
C	2.90239	-2.70227	-2.40579
C	2.74241	-5.01645	-1.58312
C	0.69988	-5.68344	-0.46977
C	4.17201	-2.96643	-2.86725
H	2.44827	-1.72445	-2.51778
C	4.05092	-5.25532	-2.07642
C	1.96267	-5.99314	-0.91373
H	0.08254	-6.41708	0.03409
C	4.74784	-4.25140	-2.71004
H	4.74191	-2.18157	-3.35475
H	4.49123	-6.23970	-1.94476
H	2.37455	-6.98639	-0.75625
H	5.75038	-4.43751	-3.08246
C	-1.15986	-3.92993	-0.17407
C	-1.11385	-3.58325	1.31724
H	-0.34902	-2.80727	1.40987
H	-0.78055	-4.46532	1.89095
N	-2.38877	-3.05680	1.75862
N	0.89641	-3.45022	-1.31869
C	-2.51900	-2.34787	2.96198
C	-3.76756	-2.34906	3.61153
C	-1.46979	-1.59739	3.52190
C	-3.95957	-1.63126	4.78787
H	-4.59077	-2.91546	3.18108
C	-1.67718	-0.88597	4.70295
H	-0.51356	-1.52567	3.01756
C	-2.91329	-0.89694	5.35031
H	-4.93448	-1.64954	5.26761
H	-0.85401	-0.30812	5.11494
H	-3.06188	-0.33887	6.26948
H	-3.13364	-3.72877	1.62893
H	-1.50059	-3.05979	-0.73378
Cl	-2.43465	-5.21802	-0.50443

Catalyst (TRIP, 3)-Product (R) alternative conformation
Electronic energy (Hartrees): -1870.558991
Free Energy at 1M concentration and 253 K (Hartrees): -1869.335924
Coordinates:

P	-0.06772	0.18635	0.74762
O	-0.94454	1.57023	0.80050
O	1.28806	0.49834	-0.11122
O	-0.81478	-0.76364	-0.28903
O	0.10081	-0.31179	2.12412
C	-0.83113	2.43434	-0.26164
C	-2.06119	2.80536	-1.00469
C	-2.03257	3.80035	-1.90268
H	-2.93539	4.07902	-2.43412
C	-0.75484	4.45288	-2.24729
C	-0.71700	5.46563	-3.32738
H	-1.63192	5.76881	-3.82108
C	0.44734	6.00664	-3.70110
H	0.47429	6.74509	-4.49194
C	1.70137	5.58615	-3.04813
H	2.64335	6.00873	-3.37326
C	1.67756	4.67574	-2.06794
H	2.61309	4.36342	-1.62357
C	0.38569	4.08848	-1.61631

C	0.32813	3.08723	-0.50633
C	2.00340	1.54820	0.41440
C	3.34597	1.30537	1.00418
C	4.00931	2.31305	1.59068
H	5.00291	2.14994	1.99259
C	3.41328	3.66087	1.66216
C	4.12201	4.74086	2.38682
H	5.08151	4.54672	2.84988
C	3.57897	5.95980	2.47451
H	4.09636	6.74812	3.00584
C	2.26662	6.23080	1.85738
H	1.82201	7.21314	1.95164
C	1.62198	5.26363	1.19447
H	0.65007	5.47831	0.77064
C	2.22765	3.91047	1.06115
C	1.52793	2.81217	0.32872
C	4.03582	0.01032	0.81005
C	4.83650	-0.19701	-0.44410
C	4.07726	-0.91863	1.80342
C	5.62805	-1.28255	-0.54119
C	4.96869	-2.09837	1.65184
C	5.72205	-2.27017	0.55683
H	6.22248	-1.44150	-1.43074
H	5.03938	-2.81075	2.46334
C	3.28973	-0.77124	3.10284
H	2.58686	0.08538	3.04789
C	2.42986	-2.01413	3.37670
H	1.84311	-2.28414	2.47279
H	1.71975	-1.80725	4.20575
H	3.05366	-2.88540	3.66637
C	4.23326	-0.49102	4.27732
H	4.92854	-1.34080	4.44492
H	3.64632	-0.32481	5.20581
H	4.82917	0.42454	4.07524
C	4.79859	0.81491	-1.58997
H	4.10267	1.64482	-1.35791
C	4.27579	0.18226	-2.88519
H	3.28686	-0.29038	-2.70431
H	4.98038	-0.58238	-3.27400
H	4.14628	0.96437	-3.66354
C	6.17237	1.46212	-1.80532
H	6.91962	0.71786	-2.15271
H	6.52998	1.91567	-0.85606
H	6.09797	2.26715	-2.56740
C	-3.31154	2.05304	-0.76046
C	-4.03721	2.26045	0.37278
C	-3.74119	1.00215	-1.74792
C	-5.18679	1.36461	0.66460
C	-4.78147	0.20572	-1.43576
C	-5.53613	0.37841	-0.17406
H	-5.75415	1.51707	1.57344
H	-5.11721	-0.54593	-2.13690
C	-3.76060	3.42363	1.32887
H	-2.88272	4.01827	1.00240
C	-4.94493	4.39765	1.34338
H	-5.16480	4.74303	0.31037
H	-4.69881	5.28832	1.96031
H	-5.85496	3.91996	1.76389
C	-3.44433	2.93454	2.74756
H	-2.55333	2.27558	2.73721

H	-4.30017	2.38215	3.18764
H	-3.21430	3.80034	3.40452
C	-3.10421	0.89701	-3.13685
H	-2.24673	1.58913	-3.22986
C	-4.10621	1.30548	-4.22428
H	-3.60522	1.33145	-5.21559
H	-4.50395	2.32097	-4.01165
H	-4.95473	0.59070	-4.27663
C	-2.53052	-0.49530	-3.42076
H	-1.74371	-0.73442	-2.67900
H	-2.05651	-0.51287	-4.42542
H	-3.31887	-1.27576	-3.39811
C	-6.73186	-0.50524	0.13604
H	-7.17417	-0.21656	1.11624
C	-6.31692	-1.97545	0.25181
H	-5.56684	-2.09305	1.05955
H	-5.88573	-2.34736	-0.70071
H	-7.19728	-2.60179	0.50974
C	-7.83071	-0.32737	-0.91827
H	-8.10935	0.74508	-1.00040
H	-8.73654	-0.89872	-0.62293
H	-7.49625	-0.68918	-1.91351
C	6.68579	-3.44074	0.46549
H	6.64006	-4.04420	1.40014
C	6.31021	-4.37814	-0.68564
H	5.27037	-4.74367	-0.54967
H	6.98574	-5.25994	-0.69454
H	6.39190	-3.86290	-1.66585
C	8.13142	-2.94981	0.32447
H	8.38401	-2.26477	1.16200
H	8.28220	-2.41353	-0.63631
H	8.83080	-3.81229	0.35936
H	-1.58973	-1.25543	0.15141
C	-3.00763	-2.15819	2.20034
C	-2.79302	-3.53940	0.34139
C	-3.00106	-0.85145	2.74414
C	-3.38026	-3.26059	3.02971
C	-3.14425	-4.68653	1.09627
C	-3.33584	-0.65334	4.06433
H	-2.74115	-0.02156	2.10056
C	-3.70966	-3.02422	4.38813
C	-3.41883	-4.54523	2.43276
H	-3.20897	-5.65076	0.60581
C	-3.68669	-1.74455	4.89454
H	-3.32643	0.34957	4.47797
H	-3.98752	-3.86655	5.01547
H	-3.68689	-5.40672	3.03840
H	-3.94207	-1.56609	5.93445
C	-2.54167	-3.62752	-1.14761
H	-2.48464	-2.61372	-1.53340
C	-1.31029	-4.42465	-1.58376
H	-1.35050	-4.53658	-2.67771
H	-1.36820	-5.43486	-1.15813
N	-0.09272	-3.75751	-1.15292
N	-2.69925	-2.33075	0.87544
C	1.11472	-4.10386	-1.76659
C	2.17689	-3.17872	-1.78341
C	1.30583	-5.36001	-2.36959
C	3.37522	-3.50264	-2.40738
H	2.04552	-2.20436	-1.31886

C	2.52074	-5.67297	-2.98011
H	0.51092	-6.09771	-2.36142
C	3.56185	-4.74874	-3.01259
H	4.17627	-2.77270	-2.42888
H	2.64442	-6.65020	-3.43900
H	4.50324	-4.99062	-3.49559
H	-0.18747	-2.75816	-1.00639
Cl	-4.01091	-4.35648	-1.96575

Catalyst (TRIP, 3)-Product (S) alternative conformation
Electronic energy (Hartrees): -1870.556359
Free Energy at 1M concentration and 253 K (Hartrees): -1869.33586
Coordinates:

P	-0.07624	0.09496	-0.48839
O	0.99455	-0.77793	0.39353
O	-1.41998	-0.81955	-0.68197
O	0.42426	0.57498	-1.79356
O	-0.47216	1.18079	0.59060
C	0.94918	-2.14344	0.25400
C	2.16065	-2.85344	-0.22986
C	2.18742	-4.19302	-0.26401
H	3.07307	-4.71067	-0.61422
C	0.99891	-4.97428	0.12362
C	1.01777	-6.44940	-0.00935
H	1.91740	-6.95457	-0.33841
C	-0.07692	-7.16370	0.27119
H	-0.06314	-8.24131	0.17114
C	-1.31257	-6.48338	0.70131
H	-2.20386	-7.06479	0.89925
C	-1.33780	-5.15208	0.83124
H	-2.26596	-4.67711	1.11779
C	-0.11648	-4.34355	0.55707
C	-0.11396	-2.85396	0.70022
C	-1.96078	-1.26563	0.49964
C	-3.33927	-0.85994	0.86833
C	-3.83913	-1.20690	2.06395
H	-4.85839	-0.94669	2.32512
C	-3.02759	-2.00470	3.00677
C	-3.55513	-2.31572	4.35504
H	-4.53135	-1.95463	4.65382
C	-2.82706	-3.04294	5.20979
H	-3.21244	-3.26872	6.19568
C	-1.49271	-3.53449	4.81432
H	-0.90281	-4.10937	5.51649
C	-1.00965	-3.26433	3.59599
H	-0.02274	-3.61967	3.32966
C	-1.81644	-2.47526	2.62777
C	-1.29653	-2.15457	1.26644
C	-4.22526	-0.25930	-0.15439
C	-4.78814	-1.14582	-1.23111
C	-4.54450	1.06330	-0.12831
C	-5.58491	-0.60438	-2.17152
C	-5.47520	1.60119	-1.15568
C	-5.95866	0.82583	-2.13607
H	-5.98695	-1.22289	-2.96242
H	-5.76184	2.64374	-1.11229
C	-3.97662	2.03026	0.90751
H	-3.21060	1.53656	1.53927
C	-3.27063	3.21294	0.23145
H	-2.52620	2.83909	-0.50204

H	-2.74278	3.82525	0.99175
H	-3.98932	3.87348	-0.29609
C	-5.07853	2.52026	1.85223
H	-5.85030	3.09927	1.30216
H	-4.64236	3.17062	2.64041
H	-5.56577	1.65382	2.34821
C	-4.48810	-2.64521	-1.27533
H	-3.86809	-2.94908	-0.40974
C	-3.68637	-3.01462	-2.52946
H	-2.75978	-2.40426	-2.58214
H	-4.28102	-2.84492	-3.45177
H	-3.39248	-4.08542	-2.49073
C	-5.77192	-3.47969	-1.17304
H	-6.41530	-3.34743	-2.06809
H	-6.34587	-3.18288	-0.26914
H	-5.51721	-4.55745	-1.08294
C	3.33505	-2.09091	-0.70818
C	4.24245	-1.58054	0.16863
C	3.57350	-1.97742	-2.18733
C	5.45036	-0.89423	-0.36402
C	4.71937	-1.42212	-2.62235
C	5.69891	-0.83841	-1.68134
H	6.16410	-0.47104	0.33096
H	4.93281	-1.38143	-3.68144
C	4.11259	-1.75062	1.68355
H	3.20092	-2.32111	1.95452
C	5.29216	-2.55112	2.24891
H	5.38017	-3.52295	1.71748
H	5.12719	-2.76033	3.32754
H	6.24730	-1.99509	2.14351
C	3.98569	-0.39439	2.38189
H	3.09920	0.14356	1.98909
H	4.89097	0.22812	2.22437
H	3.84228	-0.53759	3.47408
C	2.56253	-2.51104	-3.20144
H	1.62798	-2.81792	-2.68906
C	3.10806	-3.75415	-3.91394
H	2.33386	-4.17644	-4.58956
H	3.37919	-4.53210	-3.16909
H	4.00793	-3.50705	-4.51641
C	2.14736	-1.43679	-4.21772
H	1.84313	-0.50833	-3.69295
H	1.28366	-1.79676	-4.81672
H	2.97588	-1.19525	-4.91613
C	6.98567	-0.21973	-2.19853
H	7.53938	0.25754	-1.35888
C	6.70993	0.87683	-3.23670
H	5.97762	1.61056	-2.84327
H	6.31017	0.44957	-4.18024
H	7.65085	1.41852	-3.47176
C	7.90001	-1.30123	-2.78349
H	8.11435	-2.07420	-2.01458
H	8.86494	-0.85291	-3.10313
H	7.42720	-1.79106	-3.66152
C	-6.88782	1.40503	-3.18751
H	-7.05273	2.48902	-2.99368
C	-6.26961	1.29104	-4.58570
H	-5.27306	1.78220	-4.60196
H	-6.91866	1.79893	-5.33065
H	-6.15126	0.22889	-4.88783

C	-8.26241	0.72816	-3.13688
H	-8.69197	0.81586	-2.11595
H	-8.19168	-0.34714	-3.40549
H	-8.95635	1.22400	-3.84877
H	-0.21716	2.15316	0.45628
C	-0.06554	4.51095	-0.62398
C	0.11032	4.43953	1.69913
C	-0.07143	3.81833	-1.86353
C	-0.21465	5.93444	-0.61424
C	-0.02147	5.84798	1.78766
C	-0.23213	4.52261	-3.03599
H	0.06053	2.73960	-1.87911
C	-0.37866	6.62439	-1.84299
C	-0.18964	6.58526	0.64127
H	0.00644	6.32555	2.76092
C	-0.39002	5.93042	-3.03060
H	-0.23528	3.98792	-3.98081
H	-0.49277	7.70479	-1.82671
H	-0.29958	7.66553	0.68615
H	-0.51615	6.46046	-3.96973
N	0.07900	3.80000	0.53828
C	0.28998	3.64659	2.97903
H	0.47375	4.35062	3.79207
C	1.42506	2.60819	2.96490
H	1.30888	1.92708	2.12343
H	1.33794	2.00212	3.87470
N	2.70678	3.29092	2.91757
H	3.08957	3.49465	3.83001
C	3.69512	3.06612	1.95187
C	5.03684	3.32430	2.28787
C	3.40324	2.62566	0.64970
C	6.05139	3.14470	1.35346
H	5.27938	3.66034	3.29402
C	4.43030	2.43781	-0.27292
H	2.38138	2.43062	0.34954
C	5.75911	2.69703	0.06432
H	7.07947	3.34801	1.64038
H	4.17191	2.08276	-1.26589
H	6.55604	2.55599	-0.65735
Cl	-1.28952	2.83232	3.45409

Catalyst (TRIP, 3)-Product (S) alternative conformation
Electronic energy (Hartrees): -1870.556333
Free Energy at 1M concentration and 253 K (Hartrees): -1869.335154
Coordinates:

P	-0.75278	0.05142	0.81083
O	-0.34898	-1.51557	1.10199
O	-1.92511	0.02871	-0.32282
O	0.41144	0.66541	-0.06078
O	-1.06283	0.69286	2.10321
C	-0.47802	-2.40484	0.06157
C	0.72540	-3.15654	-0.37770
C	0.60484	-4.18257	-1.23147
H	1.48246	-4.74089	-1.53742
C	-0.70168	-4.49076	-1.84277
C	-0.79323	-5.54187	-2.88237
H	0.08040	-6.12601	-3.14375
C	-1.95316	-5.77068	-3.50668
H	-2.01846	-6.53722	-4.26800
C	-3.14306	-4.96562	-3.17334

H	-4.07130	-5.13545	-3.70357
C	-3.07241	-4.01791	-2.23154
H	-3.95057	-3.42020	-2.02826
C	-1.79838	-3.77628	-1.49858
C	-1.69810	-2.74027	-0.42294
C	-3.00685	-0.72323	0.05892
C	-4.30764	-0.05191	0.30240
C	-5.37136	-0.77583	0.68102
H	-6.33214	-0.29680	0.82780
C	-5.24503	-2.23158	0.89634
C	-6.40109	-3.00198	1.40961
H	-7.34377	-2.50497	1.60224
C	-6.28507	-4.31425	1.64024
H	-7.12979	-4.87632	2.01708
C	-5.00099	-4.99804	1.39408
H	-4.90735	-6.05561	1.60462
C	-3.95238	-4.31231	0.92442
H	-3.01365	-4.82852	0.77329
C	-4.07473	-2.85832	0.63253
C	-2.92130	-2.07115	0.10142
C	-4.45764	1.39499	0.02132
C	-4.52851	1.85535	-1.40839
C	-4.55063	2.30335	1.03241
C	-4.67343	3.16928	-1.66416
C	-4.76299	3.73611	0.69791
C	-4.80502	4.15865	-0.57314
H	-4.71857	3.52035	-2.68613
H	-4.87994	4.45210	1.50081
C	-4.44560	1.91494	2.50700
H	-4.12209	0.85943	2.62073
C	-3.39068	2.75867	3.23931
H	-2.45113	2.80279	2.65040
H	-3.16375	2.30508	4.22781
H	-3.74617	3.79622	3.41118
C	-5.80981	2.04137	3.19338
H	-6.16931	3.09230	3.17633
H	-5.73693	1.70759	4.25053
H	-6.55690	1.40096	2.67882
C	-4.45930	0.86876	-2.57448
H	-4.36989	-0.17146	-2.20512
C	-3.22388	1.12359	-3.44814
H	-2.30485	1.10691	-2.82467
H	-3.28927	2.10530	-3.96282
H	-3.13330	0.32833	-4.21861
C	-5.74453	0.90205	-3.41210
H	-5.86711	1.87713	-3.92877
H	-6.62666	0.72669	-2.75967
H	-5.71737	0.10027	-4.18077
C	2.06001	-2.75699	0.12208
C	2.45591	-3.07495	1.38558
C	2.94617	-1.91422	-0.75261
C	3.74213	-2.53110	1.89665
C	4.06501	-1.38369	-0.22270
C	4.50314	-1.71769	1.15045
H	4.06714	-2.79843	2.89371
H	4.70188	-0.75194	-0.82620
C	1.65114	-4.00702	2.29292
H	0.70366	-4.32547	1.81198
C	2.43491	-5.29443	2.57371
H	2.70923	-5.78734	1.61639

H	1.80932	-6.00101	3.16005
H	3.36185	-5.08580	3.14846
C	1.25578	-3.31369	3.60236
H	0.71359	-2.36931	3.38958
H	2.14670	-3.08684	4.22391
H	0.58082	-3.97171	4.19022
C	2.65400	-1.71018	-2.24214
H	1.76577	-2.29046	-2.55389
C	3.81108	-2.22898	-3.10956
H	3.52226	-2.20979	-4.18223
H	4.05096	-3.27871	-2.83576
H	4.72133	-1.60668	-2.98471
C	2.33275	-0.24834	-2.56877
H	1.30507	-0.00860	-2.23570
H	2.36886	-0.07277	-3.66516
H	3.04258	0.44029	-2.07242
C	5.83295	-1.20745	1.67871
H	6.02623	-1.62687	2.69189
C	5.81757	0.31686	1.82427
H	5.08533	0.60658	2.60312
H	5.54394	0.81117	0.86975
H	6.81544	0.68485	2.14462
C	6.99211	-1.66309	0.78317
H	6.96955	-2.76740	0.66317
H	7.96239	-1.38546	1.24746
H	6.93785	-1.19127	-0.22017
C	-4.99576	5.63122	-0.88832
H	-5.07584	6.21502	0.05644
C	-3.78975	6.18652	-1.65490
H	-2.85685	6.00603	-1.07882
H	-3.90241	7.28243	-1.79801
H	-3.69305	5.70919	-2.65302
C	-6.29621	5.86114	-1.66689
H	-7.15725	5.44754	-1.09937
H	-6.25849	5.37676	-2.66556
H	-6.46594	6.94971	-1.80974
H	1.13302	1.13730	0.47266
C	2.56808	1.78940	2.56635
C	2.72087	3.17547	0.70095
C	2.16817	0.54973	3.12593
C	3.25910	2.73720	3.38450
C	3.40291	4.17201	1.44800
C	2.43969	0.27531	4.44717
H	1.66135	-0.17419	2.50091
C	3.51807	2.42352	4.74268
C	3.66192	3.95217	2.77780
H	3.69043	5.10095	0.96857
C	3.11484	1.21499	5.26261
H	2.12877	-0.67149	4.87472
H	4.04143	3.14880	5.35947
H	4.17689	4.70066	3.37421
H	3.31454	0.97567	6.30246
C	2.45810	3.36033	-0.77776
C	3.71866	3.72649	-1.59077
H	4.05338	4.73040	-1.29855
H	3.44000	3.79360	-2.64241
N	4.77275	2.75538	-1.41099
N	2.30954	2.04172	1.24568
C	5.37198	1.98193	-2.40351
C	4.83990	1.83378	-3.69678

C	6.55309	1.28183	-2.08699
C	5.46632	1.00306	-4.62511
H	3.92157	2.33713	-3.97889
C	7.16538	0.45482	-3.02132
H	6.98503	1.38779	-1.09455
C	6.62951	0.30565	-4.30256
H	5.02717	0.89945	-5.61341
H	8.07080	-0.07710	-2.74259
H	7.10604	-0.34291	-5.03022
H	5.32430	2.86940	-0.57438
H	1.98984	2.46007	-1.17096
Cl	1.22299	4.69789	-1.02453

Catalyst (TRIP, 3)-Product (R) alternative conformation
Electronic energy (Hartrees): -1870.557452
Free Energy at 1M concentration and 253 K (Hartrees): -1869.334962
Coordinates:

P	0.35703	0.34241	-0.84902
O	1.97071	0.48169	-1.02586
O	-0.07826	1.40865	0.30505
O	0.08742	-1.02670	-0.09738
O	-0.30419	0.51538	-2.15974
C	2.73621	0.96132	0.01455
C	3.94210	0.17793	0.39771
C	4.82988	0.68242	1.26631
H	5.71496	0.11473	1.53065
C	4.55969	1.95942	1.94846
C	5.47290	2.42642	3.01715
H	6.36984	1.86591	3.24985
C	5.19125	3.53883	3.70318
H	5.85755	3.87813	4.48574
C	3.96048	4.29612	3.40897
H	3.72181	5.17834	3.98888
C	3.13298	3.88549	2.44111
H	2.22041	4.43943	2.26661
C	3.45320	2.67171	1.63855
C	2.55904	2.20747	0.53052
C	0.22842	2.69675	-0.04786
C	-0.86773	3.64892	-0.36010
C	-0.58359	4.93989	-0.58878
H	-1.38128	5.64941	-0.77164
C	0.82108	5.38865	-0.66915
C	1.12417	6.78514	-1.05835
H	0.31917	7.48893	-1.22994
C	2.39291	7.18189	-1.20423
H	2.61350	8.20203	-1.49077
C	3.49603	6.22547	-0.99215
H	4.51949	6.54590	-1.13848
C	3.23312	4.96311	-0.63492
H	4.05602	4.27166	-0.51195
C	1.82826	4.51567	-0.43157
C	1.50809	3.11802	-0.00664
C	-2.26942	3.16714	-0.35834
C	-2.88329	2.74322	0.94542
C	-2.97768	3.03583	-1.51736
C	-4.06844	2.10660	0.93261
C	-4.30680	2.36931	-1.47250
C	-4.80097	1.87059	-0.32967
H	-4.52695	1.79614	1.86177
H	-4.87026	2.25478	-2.38850

C	-2.47643	3.56627	-2.86441
H	-1.40936	3.85836	-2.81121
C	-2.55166	2.51617	-3.98084
H	-2.09979	1.56198	-3.64859
H	-1.98698	2.87134	-4.86938
H	-3.59878	2.33424	-4.29884
C	-3.26521	4.81944	-3.26008
H	-4.34053	4.58122	-3.40668
H	-2.86193	5.24330	-4.20459
H	-3.17827	5.59332	-2.46854
C	-2.23515	3.10215	2.28238
H	-1.25471	3.59346	2.12192
C	-1.96310	1.85885	3.13749
H	-1.42351	1.08988	2.54613
H	-2.91077	1.42404	3.51562
H	-1.33333	2.13015	4.01144
C	-3.09315	4.11536	3.05057
H	-4.07588	3.68024	3.33100
H	-3.26560	5.01718	2.42479
H	-2.56995	4.43165	3.97828
C	4.16236	-1.17040	-0.17527
C	4.55580	-1.32650	-1.47059
C	3.93466	-2.37985	0.69047
C	4.70345	-2.70002	-2.01890
C	4.07258	-3.60448	0.14743
C	4.46575	-3.78327	-1.26679
H	5.01096	-2.82457	-3.04901
H	3.91418	-4.48681	0.75248
C	4.89643	-0.14743	-2.38376
H	4.72190	0.82384	-1.87832
C	6.38346	-0.16553	-2.75646
H	7.00477	-0.16002	-1.83524
H	6.63790	0.73774	-3.35128
H	6.63879	-1.06470	-3.35597
C	4.01859	-0.14267	-3.64307
H	2.94474	-0.19261	-3.36703
H	4.25488	-1.00104	-4.30599
H	4.18485	0.79454	-4.21602
C	3.58409	-2.25478	2.17474
H	3.50594	-1.19359	2.47666
C	4.67713	-2.87133	3.05739
H	4.46231	-2.66647	4.12802
H	5.66256	-2.42154	2.81024
H	4.73750	-3.97110	2.91660
C	2.21532	-2.86430	2.48546
H	1.43948	-2.33564	1.89711
H	1.97232	-2.73475	3.56175
H	2.18715	-3.94774	2.24627
C	4.61949	-5.17480	-1.85363
H	4.90074	-5.10508	-2.92872
C	3.29716	-5.94709	-1.78786
H	2.48795	-5.36180	-2.27511
H	3.00816	-6.15544	-0.73640
H	3.39604	-6.91560	-2.32290
C	5.74073	-5.94488	-1.14683
H	6.69065	-5.37147	-1.20480
H	5.89767	-6.92699	-1.64183
H	5.49428	-6.12382	-0.07876
C	-6.10963	1.10166	-0.32521
H	-6.48043	0.97780	-1.36784

C	-5.91612	-0.30734	0.25071
H	-5.11019	-0.83948	-0.29557
H	-6.85280	-0.89474	0.14416
H	-5.64697	-0.26636	1.32662
C	-7.18813	1.86575	0.45029
H	-7.31703	2.88216	0.02050
H	-6.91933	1.96125	1.52372
H	-8.15948	1.33178	0.37594
H	-0.66994	-1.56749	-0.50044
C	-2.96983	-2.44701	-1.78859
C	-1.80638	-3.95247	-0.45282
C	-3.06264	-1.16320	-2.38288
C	-4.00344	-3.40659	-2.03505
C	-2.76663	-4.97194	-0.68360
C	-4.15618	-0.85197	-3.16226
H	-2.25682	-0.45175	-2.23096
C	-5.11502	-3.04644	-2.83710
C	-3.85908	-4.69358	-1.46314
H	-2.61885	-5.95181	-0.24505
C	-5.19316	-1.78738	-3.38492
H	-4.22917	0.12694	-3.61999
H	-5.89714	-3.78068	-3.00982
H	-4.61780	-5.44909	-1.64972
H	-6.04593	-1.50894	-3.99647
C	-0.66915	-4.17681	0.51618
H	0.10322	-3.43321	0.33506
C	-1.12945	-4.13252	1.99415
H	-0.27543	-4.41510	2.62148
H	-1.89934	-4.89054	2.15731
N	-1.63823	-2.83266	2.36126
N	-1.89316	-2.74380	-0.98760
C	-2.92511	-2.57210	2.83175
C	-4.04584	-3.32767	2.44371
C	-3.12285	-1.48982	3.70872
C	-5.31441	-2.99890	2.91985
H	-3.93605	-4.15106	1.74713
C	-4.39579	-1.16007	4.16165
H	-2.25998	-0.91912	4.04210
C	-5.50587	-1.91435	3.77570
H	-6.16548	-3.59437	2.60071
H	-4.51711	-0.31433	4.83279
H	-6.49877	-1.65910	4.13143
H	-0.94299	-2.15517	2.63587
Cl	0.14795	-5.79121	0.22846

Catalyst (TRIP, 3)-Product (S) alternative conformation
Electronic energy (Hartrees): -1870.555025
Free Energy at 1M concentration and 253 K (Hartrees): -1869.33414
Coordinates:

P	0.55175	-0.38316	-1.14764
O	1.70722	0.74787	-1.33460
O	-0.76063	0.34759	-0.46882
O	1.02551	-1.24008	0.09519
O	0.30904	-1.07147	-2.43000
C	1.71904	1.79127	-0.44243
C	2.94015	2.01183	0.37355
C	3.02628	3.08331	1.17456
H	3.92017	3.25229	1.76304
C	1.88602	4.01020	1.30676
C	1.95125	5.11546	2.29047

H	2.85144	5.27447	2.87108
C	0.89618	5.91630	2.47276
H	0.94314	6.72079	3.19528
C	-0.34046	5.69722	1.69947
H	-1.19917	6.33406	1.86881
C	-0.40619	4.70822	0.80107
H	-1.33311	4.55363	0.26619
C	0.76857	3.82280	0.56728
C	0.71583	2.69962	-0.41776
C	-1.21884	1.42285	-1.18594
C	-2.60044	1.41224	-1.72059
C	-2.99432	2.37959	-2.56333
H	-4.01680	2.40545	-2.92232
C	-2.08179	3.49749	-2.88791
C	-2.50645	4.54322	-3.84643
H	-3.47173	4.47570	-4.33261
C	-1.69966	5.57609	-4.11472
H	-2.01024	6.33968	-4.81610
C	-0.38119	5.67655	-3.45856
H	0.27181	6.50790	-3.69088
C	0.00933	4.74083	-2.58528
H	0.98515	4.82384	-2.12468
C	-0.88462	3.59781	-2.26255
C	-0.46614	2.53440	-1.30391
C	-3.60895	0.52784	-1.09632
C	-4.36816	1.04043	0.09735
C	-3.84015	-0.72957	-1.55989
C	-5.25040	0.22696	0.70697
C	-4.86017	-1.57228	-0.88045
C	-5.52622	-1.13329	0.19708
H	-5.80058	0.57531	1.57036
H	-5.07237	-2.55881	-1.27130
C	-3.10963	-1.31203	-2.76649
H	-2.31619	-0.62516	-3.12596
C	-2.41138	-2.62955	-2.40535
H	-1.80055	-2.49271	-1.48994
H	-1.73931	-2.94435	-3.23203
H	-3.14224	-3.44489	-2.22384
C	-4.07592	-1.51004	-3.93884
H	-4.86606	-2.24925	-3.68847
H	-3.52222	-1.87287	-4.83108
H	-4.55823	-0.54337	-4.19881
C	-4.18349	2.46939	0.61552
H	-3.46137	3.02751	-0.01004
C	-3.59975	2.48537	2.03107
H	-2.60934	1.98958	2.02407
H	-4.26540	1.96739	2.75287
H	-3.45163	3.53202	2.37301
C	-5.49495	3.26299	0.54830
H	-6.24911	2.86032	1.25664
H	-5.90941	3.22349	-0.48186
H	-5.30816	4.32769	0.80518
C	4.05649	1.03768	0.31935
C	4.88632	0.98353	-0.76028
C	4.28947	0.12015	1.48992
C	5.99687	-0.00457	-0.76804
C	5.33319	-0.72922	1.45500
C	6.21743	-0.81806	0.27357
H	6.64384	-0.06158	-1.63373
H	5.53454	-1.37555	2.29815

C	4.75552	1.92498	-1.95876
H	3.89399	2.61332	-1.84652
C	5.99317	2.82140	-2.08381
H	6.15234	3.38129	-1.13733
H	5.84758	3.55870	-2.90214
H	6.90361	2.22600	-2.30625
C	4.51423	1.14109	-3.25585
H	3.64539	0.45988	-3.13854
H	5.40302	0.53840	-3.53694
H	4.29282	1.84393	-4.08726
C	3.38743	0.14794	2.72397
H	2.50106	0.78447	2.53639
C	4.12364	0.75388	3.92421
H	3.43247	0.84127	4.78974
H	4.49400	1.77001	3.67321
H	4.98818	0.12287	4.22153
C	2.83750	-1.24344	3.07295
H	2.41770	-1.73047	2.17065
H	2.02814	-1.15090	3.82849
H	3.62716	-1.90066	3.49344
C	7.35218	-1.82544	0.23587
H	7.90044	-1.74481	-0.72989
C	6.81276	-3.25789	0.32461
H	6.07505	-3.43750	-0.48684
H	6.32115	-3.44251	1.30328
H	7.64402	-3.98511	0.20496
C	8.36822	-1.54955	1.35014
H	8.74140	-0.50576	1.27445
H	9.23603	-2.23605	1.25104
H	7.91569	-1.69733	2.35347
C	-6.58578	-2.00052	0.85373
H	-6.64579	-2.98458	0.33627
C	-6.23845	-2.28801	2.31878
H	-5.25247	-2.79080	2.38292
H	-6.99769	-2.96615	2.76343
H	-6.20682	-1.35399	2.91839
C	-7.96871	-1.34928	0.73746
H	-8.20412	-1.14419	-0.32889
H	-8.01230	-0.39534	1.30468
H	-8.74651	-2.03381	1.13830
H	0.77631	-2.22737	0.09914
C	0.75285	-4.27801	-1.32668
C	-0.76219	-4.39467	0.43835
C	1.93421	-3.71530	-1.87358
C	0.09501	-5.33256	-2.03238
C	-1.46923	-5.44773	-0.20227
C	2.42598	-4.17559	-3.07302
H	2.43964	-2.92719	-1.32924
C	0.61981	-5.77457	-3.27378
C	-1.05697	-5.89365	-1.43349
H	-2.32721	-5.89276	0.29080
C	1.76351	-5.20563	-3.78387
H	3.33046	-3.73868	-3.48422
H	0.10802	-6.57134	-3.80629
H	-1.59154	-6.69116	-1.94258
H	2.16496	-5.54593	-4.73332
C	-1.26224	-3.97652	1.81292
C	-2.33759	-2.84419	1.84701
H	-2.84976	-2.93776	2.80569
H	-3.06575	-3.10134	1.06338

N	-1.89137	-1.48655	1.70717
N	0.28409	-3.80971	-0.12502
C	-1.70376	-0.62647	2.79954
C	-0.58881	0.23159	2.82911
C	-2.64276	-0.53997	3.84177
C	-0.42792	1.15058	3.86124
H	0.14789	0.15305	2.03923
C	-2.45924	0.36936	4.88261
H	-3.53549	-1.15778	3.82309
C	-1.35743	1.22561	4.90119
H	0.44159	1.80327	3.85978
H	-3.20006	0.42043	5.67604
H	-1.22661	1.93804	5.70971
H	-1.26866	-1.29821	0.93652
H	-1.74906	-4.85887	2.23584
Cl	0.10235	-3.61660	2.94759

Catalyst (TRIP, 3)-Product (S) alternative conformation
Electronic energy (Hartrees): -1870.553996
Free Energy at 1M concentration and 253 K (Hartrees): -1869.333179
Coordinates:

P	0.57532	0.12463	-0.65169
O	-0.16713	-1.28929	-0.98307
O	1.87594	-0.21795	0.27463
O	-0.35472	0.86644	0.38818
O	0.86649	0.84143	-1.91443
C	0.16782	-2.43625	-0.29793
C	-0.94913	-3.28050	0.20606
C	-0.70666	-4.50660	0.69017
H	-1.52401	-5.11817	1.05530
C	0.67507	-4.98627	0.84893
C	0.92435	-6.28607	1.51461
H	0.09333	-6.91579	1.80747
C	2.17559	-6.68506	1.76299
H	2.35697	-7.63341	2.25208
C	3.31207	-5.82234	1.39231
H	4.32137	-6.13361	1.62897
C	3.09681	-4.65005	0.78470
H	3.94689	-4.02039	0.56085
C	1.70995	-4.21025	0.45756
C	1.44110	-2.91513	-0.24808
C	2.82023	-0.90548	-0.44417
C	4.14366	-0.28503	-0.70025
C	5.03850	-0.91832	-1.47379
H	6.01860	-0.48638	-1.63946
C	4.70185	-2.21719	-2.09180
C	5.65747	-2.86168	-3.02153
H	6.60703	-2.38894	-3.23945
C	5.35071	-4.02951	-3.59717
H	6.04897	-4.49943	-4.27761
C	4.05468	-4.67652	-3.31469
H	3.80496	-5.61184	-3.79876
C	3.18675	-4.10496	-2.47172
H	2.23345	-4.58467	-2.29201
C	3.52548	-2.82047	-1.80279
C	2.57661	-2.16694	-0.85263
C	4.52362	0.96042	0.00434
C	4.83890	0.89755	1.47330
C	4.59915	2.14856	-0.65591
C	5.16748	2.03250	2.11887

C	5.01556	3.36447	0.09102
C	5.26975	3.32430	1.40624
H	5.38688	2.00923	3.17767
H	5.10501	4.30304	-0.44037
C	4.25358	2.29587	-2.13669
H	3.84191	1.35311	-2.55199
C	3.16509	3.35562	-2.34717
H	2.32670	3.18045	-1.64189
H	2.77330	3.29462	-3.38476
H	3.55585	4.38171	-2.18371
C	5.50598	2.62908	-2.95429
H	5.93592	3.60668	-2.64935
H	5.25377	2.67473	-4.03529
H	6.27449	1.84006	-2.81043
C	4.81552	-0.42321	2.24395
H	4.57136	-1.26775	1.57042
C	3.73550	-0.41349	3.33347
H	2.74710	-0.17032	2.88912
H	3.96643	0.33230	4.12305
H	3.66415	-1.41632	3.80633
C	6.19313	-0.75226	2.83450
H	6.48165	-0.02504	3.62227
H	6.96221	-0.73906	2.03269
H	6.17991	-1.76786	3.28493
C	-2.32955	-2.74508	0.26860
C	-3.10553	-2.66007	-0.84600
C	-2.90615	-2.39194	1.61184
C	-4.52410	-2.23901	-0.70507
C	-4.20852	-2.06065	1.69802
C	-5.06658	-1.97569	0.49312
H	-5.14825	-2.18888	-1.58848
H	-4.63597	-1.84033	2.66471
C	-2.60603	-3.05199	-2.23632
H	-1.53619	-3.34083	-2.21950
C	-3.36463	-4.27583	-2.76324
H	-3.27123	-5.11749	-2.04383
H	-2.93415	-4.60062	-3.73462
H	-4.44140	-4.05087	-2.91447
C	-2.71686	-1.87508	-3.21354
H	-2.22579	-0.97365	-2.78878
H	-3.77715	-1.63546	-3.43436
H	-2.21138	-2.12795	-4.16991
C	-2.05054	-2.41766	2.88067
H	-1.00038	-2.67619	2.64080
C	-2.54688	-3.48436	3.86500
H	-1.84695	-3.56386	4.72409
H	-2.59161	-4.47415	3.36281
H	-3.55547	-3.23403	4.25615
C	-1.98409	-1.03939	3.54845
H	-1.59140	-0.29490	2.82611
H	-1.29242	-1.07035	4.41728
H	-2.98169	-0.71145	3.90828
C	-6.55521	-1.66818	0.58135
H	-6.83965	-1.05867	-0.30473
C	-6.94670	-0.84527	1.81815
H	-6.27604	0.03019	1.93250
H	-6.90258	-1.46055	2.74185
H	-7.98592	-0.46913	1.70518
C	-7.36405	-2.96857	0.53107
H	-7.14950	-3.51986	-0.40939

H	-8.45209	-2.74549	0.55869
H	-7.10770	-3.62014	1.39420
C	5.66827	4.58531	2.15136
H	5.69303	5.44968	1.44989
C	4.64333	4.92364	3.24002
H	3.62986	5.01326	2.79339
H	4.90001	5.89459	3.71495
H	4.62042	4.14126	4.02795
C	7.07585	4.45046	2.74360
H	7.80309	4.19917	1.94204
H	7.11110	3.65865	3.52153
H	7.38726	5.41172	3.20534
H	-0.69866	1.77470	0.06870
C	-1.20263	4.14905	-1.14288
C	-2.26370	3.63758	0.86945
C	-0.40980	3.72968	-2.24069
C	-1.72817	5.47929	-1.12454
C	-2.82772	4.93207	0.95520
C	-0.14450	4.61129	-3.26596
H	-0.02196	2.71514	-2.25856
C	-1.42398	6.36162	-2.19294
C	-2.54717	5.84535	-0.03071
H	-3.46495	5.18368	1.79203
C	-0.64560	5.93516	-3.24401
H	0.45884	4.28327	-4.10685
H	-1.82305	7.37195	-2.16767
H	-2.96254	6.84882	0.01174
H	-0.41795	6.61059	-4.06310
C	-2.58038	2.52103	1.85258
C	-3.58941	1.52374	1.26099
H	-3.79678	0.75307	2.01407
H	-3.08884	1.02874	0.42821
N	-4.79683	2.17744	0.79603
N	-1.46785	3.26682	-0.12500
C	-5.50294	1.76098	-0.33928
C	-6.89556	1.96057	-0.38468
C	-4.87371	1.19746	-1.46364
C	-7.63231	1.60650	-1.51039
H	-7.39840	2.38928	0.47950
C	-5.62401	0.84350	-2.58360
H	-3.79951	1.04923	-1.48071
C	-7.00456	1.03933	-2.62082
H	-8.70683	1.76725	-1.51287
H	-5.11503	0.41685	-3.44258
H	-7.57893	0.75765	-3.49739
H	-5.40156	2.46519	1.55418
H	-1.66300	1.99229	2.10752
Cl	-3.21170	3.14645	3.45308

Catalyst (TRIP, 3)-Product (R) alternative conformation
Electronic energy (Hartrees): -1870.554526
Free Energy at 1M concentration and 253 K (Hartrees): -1869.332901
Coordinates:

P	0.64481	-0.40431	0.05424
O	-0.15165	0.99554	0.36480
O	2.10908	-0.00683	-0.53177
O	0.02245	-1.04583	-1.24185
O	0.61332	-1.21682	1.29180
C	0.22110	2.14194	-0.29883
C	-0.84954	2.93591	-0.95998

C	-0.59957	4.18022	-1.38851
H	-1.39923	4.79137	-1.78792
C	0.77773	4.70495	-1.37764
C	1.06893	6.01195	-2.01029
H	0.26448	6.60741	-2.42384
C	2.32697	6.45951	-2.07947
H	2.53947	7.41344	-2.54455
C	3.43166	5.64565	-1.53827
H	4.45074	6.00053	-1.62260
C	3.17781	4.46669	-0.95881
H	4.00724	3.87472	-0.59553
C	1.77839	3.97082	-0.83999
C	1.46358	2.67260	-0.16499
C	2.83713	0.71454	0.37776
C	4.04959	0.11036	0.98300
C	4.74940	0.79519	1.89921
H	5.65379	0.37316	2.32151
C	4.29606	2.13039	2.33887
C	5.02188	2.83318	3.42184
H	5.89864	2.38286	3.87048
C	4.59588	4.02543	3.85268
H	5.12562	4.53754	4.64555
C	3.39366	4.64048	3.25843
H	3.03985	5.59396	3.62895
C	2.73455	4.01681	2.27509
H	1.84055	4.47379	1.87221
C	3.21541	2.70561	1.76093
C	2.50008	1.99074	0.66038
C	4.54552	-1.19798	0.49692
C	5.23688	-1.26377	-0.83643
C	4.40092	-2.32823	1.24352
C	5.69871	-2.45049	-1.27382
C	4.97572	-3.60346	0.74050
C	5.57751	-3.67583	-0.45477
H	6.19060	-2.51945	-2.23455
H	4.90083	-4.49212	1.35346
C	3.66952	-2.34655	2.58544
H	3.14966	-1.38379	2.77096
C	2.57786	-3.42787	2.61819
H	1.96134	-3.38652	1.69599
H	1.91134	-3.26580	3.49224
H	3.01448	-4.44503	2.70286
C	4.66290	-2.54577	3.73499
H	5.17831	-3.52629	3.65197
H	4.13110	-2.50503	4.70956
H	5.42652	-1.73952	3.72308
C	5.44039	-0.01673	-1.69747
H	5.03951	0.88204	-1.18939
C	4.68694	-0.13044	-3.02907
H	3.61230	-0.34057	-2.84256
H	5.10604	-0.94114	-3.66158
H	4.75971	0.82612	-3.58944
C	6.93058	0.26763	-1.92800
H	7.40491	-0.52574	-2.54306
H	7.45936	0.33210	-0.95291
H	7.05459	1.23832	-2.45413
C	-2.21354	2.37563	-1.05846
C	-3.03460	2.39280	0.03077
C	-2.66812	1.70544	-2.33500
C	-4.34932	1.70640	-0.04341

C	-3.86671	1.08929	-2.34260
C	-4.75078	1.08324	-1.15816
H	-5.00260	1.72715	0.81902
H	-4.23275	0.62857	-3.25170
C	-2.68408	3.13372	1.32512
H	-1.68409	3.60955	1.26769
C	-3.67568	4.27545	1.57971
H	-3.70742	4.95325	0.69980
H	-3.35422	4.86920	2.46209
H	-4.69827	3.88812	1.77247
C	-2.63289	2.18026	2.52521
H	-1.89192	1.37609	2.34306
H	-3.62388	1.72325	2.72286
H	-2.31716	2.73257	3.43584
C	-1.92529	1.78808	-3.67788
H	-2.30687	0.97372	-4.33553
C	-2.25207	3.09446	-4.40822
H	-1.83228	3.96984	-3.87814
H	-3.35265	3.22124	-4.49290
H	-1.82716	3.07187	-5.43460
C	-0.40967	1.55175	-3.61508
H	-0.16515	0.76894	-2.87723
H	0.14703	2.47730	-3.36910
H	-0.04336	1.21042	-4.60703
C	-6.12846	0.44792	-1.22857
H	-6.62161	0.49892	-0.23184
C	-6.04095	-1.03377	-1.60684
H	-5.36495	-1.56369	-0.90709
H	-5.66135	-1.16367	-2.64191
H	-7.04499	-1.50399	-1.53993
C	-7.02472	1.20865	-2.21188
H	-7.08675	2.27885	-1.91968
H	-8.05166	0.78509	-2.19680
H	-6.62989	1.14211	-3.24800
C	6.13655	-4.99524	-0.95576
H	5.95338	-5.79582	-0.20387
C	5.43704	-5.42949	-2.24892
H	4.33897	-5.48297	-2.08768
H	5.79224	-6.43722	-2.55307
H	5.64407	-4.71846	-3.07658
C	7.65418	-4.90760	-1.15392
H	8.14300	-4.58646	-0.20924
H	7.91422	-4.18520	-1.95637
H	8.05898	-5.90398	-1.43244
H	-0.80620	-1.62833	-1.08612
C	-2.71381	-2.71134	-2.35853
C	-2.70888	-3.16124	-0.06687
C	-2.14993	-2.01739	-3.45975
C	-3.83956	-3.56399	-2.57104
C	-3.83001	-4.01992	-0.19617
C	-2.70125	-2.15405	-4.71383
H	-1.28737	-1.38461	-3.28991
C	-4.37595	-3.68992	-3.87842
C	-4.37810	-4.22375	-1.43890
H	-4.23123	-4.50022	0.68623
C	-3.82166	-2.99333	-4.92786
H	-2.27107	-1.61249	-5.55079
H	-5.23483	-4.33724	-4.03234
H	-5.23774	-4.87710	-1.56244
H	-4.24026	-3.08603	-5.92511

C	-2.08771	-2.80642	1.27147
H	-1.00678	-2.70482	1.16327
C	-2.61048	-1.45782	1.80656
H	-2.31149	-0.70252	1.06872
H	-2.06683	-1.22224	2.72184
N	-4.04419	-1.40186	2.03126
N	-2.17226	-2.54174	-1.11126
C	-4.60481	-0.99720	3.24836
C	-4.00204	-1.27680	4.48688
C	-5.83497	-0.31393	3.24083
C	-4.60876	-0.86128	5.67094
H	-3.07595	-1.83917	4.52617
C	-6.43707	0.08344	4.43052
H	-6.31280	-0.08905	2.28962
C	-5.82622	-0.17939	5.65827
H	-4.12292	-1.08607	6.61650
H	-7.38548	0.61254	4.39440
H	-6.29207	0.13712	6.58602
H	-4.55296	-1.03076	1.24277
Cl	-2.32247	-4.12621	2.50500

Catalyst (TRIP, 3)-Product (S) alternative conformation
Electronic energy (Hartrees): -1870.551825
Free Energy at 1M concentration and 253 K (Hartrees): -1869.332319
Coordinates:

P	-0.67025	-0.22565	-0.18730
O	0.06834	1.20817	-0.46138
O	-2.17463	0.09470	0.36300
O	-0.07432	-0.84484	1.13711
O	-0.57619	-1.04632	-1.41609
C	-0.17088	2.20610	0.45152
C	0.98280	2.82285	1.16181
C	0.77720	3.84056	2.01082
H	1.61516	4.30406	2.51844
C	-0.58786	4.33448	2.27189
C	-0.80503	5.39124	3.28677
H	0.03787	5.82121	3.81346
C	-2.04316	5.81628	3.55992
H	-2.20139	6.58690	4.30326
C	-3.19903	5.22607	2.85859
H	-4.20039	5.55752	3.10142
C	-3.01253	4.27536	1.93577
H	-3.87684	3.83906	1.45299
C	-1.63803	3.81341	1.59798
C	-1.40199	2.75757	0.56727
C	-2.85792	1.02306	-0.37837
C	-4.10029	0.61405	-1.08022
C	-4.81567	1.52443	-1.75734
H	-5.73951	1.23804	-2.24571
C	-4.34900	2.92213	-1.84993
C	-5.09692	3.89338	-2.68125
H	-5.99902	3.58748	-3.19640
C	-4.65985	5.15151	-2.80189
H	-5.20580	5.86020	-3.41106
C	-3.42422	5.57433	-2.11550
H	-3.06355	6.58770	-2.23599
C	-2.74451	4.70623	-1.35733
H	-1.82807	5.03149	-0.88323
C	-3.23453	3.31100	-1.18815
C	-2.49961	2.32448	-0.33985

C	-4.59420	-0.77823	-0.96294
C	-5.22416	-1.22327	0.32802
C	-4.50505	-1.64655	-2.00957
C	-5.68171	-2.48549	0.42948
C	-5.06799	-3.01444	-1.86224
C	-5.61098	-3.42682	-0.70859
H	-6.13412	-2.82510	1.35127
H	-5.03286	-3.69149	-2.70561
C	-3.85208	-1.27539	-3.34148
H	-3.33306	-0.29685	-3.27488
C	-2.77477	-2.29271	-3.74677
H	-2.09672	-2.50014	-2.89400
H	-2.16847	-1.88728	-4.58489
H	-3.22402	-3.25143	-4.08043
C	-4.91216	-1.14575	-4.44065
H	-5.42816	-2.11409	-4.61386
H	-4.43786	-0.82083	-5.39130
H	-5.66898	-0.38549	-4.15400
C	-5.38071	-0.27312	1.51555
H	-4.97382	0.72823	1.27414
C	-4.59982	-0.77271	2.73877
H	-3.53732	-0.95044	2.46886
H	-5.03022	-1.71651	3.13479
H	-4.62873	-0.00885	3.54496
C	-6.85931	-0.04948	1.85899
H	-7.33584	-0.98268	2.22632
H	-7.40767	0.30494	0.95994
H	-6.95245	0.72563	2.64954
C	2.36957	2.35706	0.91178
C	3.02480	2.68868	-0.23704
C	3.09405	1.60120	1.99176
C	4.45299	2.30310	-0.39212
C	4.40374	1.32826	1.83398
C	5.11875	1.67385	0.58589
H	4.96670	2.54702	-1.31287
H	4.94657	0.80895	2.61187
C	2.37061	3.47492	-1.37328
H	1.31633	3.72649	-1.14465
C	3.08036	4.81637	-1.59265
H	3.09881	5.39450	-0.64395
H	2.53400	5.41509	-2.35255
H	4.12289	4.67144	-1.94579
C	2.34562	2.64811	-2.66575
H	1.85750	1.66706	-2.48371
H	3.37072	2.46853	-3.05223
H	1.76583	3.18359	-3.44754
C	2.37282	1.12158	3.25021
H	1.28420	1.31667	3.16675
C	2.86851	1.87744	4.48847
H	2.28051	1.57311	5.38055
H	2.73972	2.97106	4.34539
H	3.94160	1.66661	4.68272
C	2.51268	-0.39635	3.44485
H	2.25718	-0.92871	2.50702
H	1.81946	-0.74275	4.24095
H	3.54623	-0.67565	3.73859
C	6.57621	1.29131	0.39967
H	6.92761	1.61111	-0.60748
C	6.76059	-0.22915	0.47296
H	6.09302	-0.72990	-0.25785

H	6.53021	-0.61468	1.48852
H	7.80907	-0.49724	0.22219
C	7.46175	2.00136	1.42915
H	7.31855	3.10096	1.35947
H	8.53134	1.77744	1.22911
H	7.21929	1.67249	2.46194
C	-6.15581	-4.83741	-0.57568
H	-6.01517	-5.39099	-1.53148
C	-5.39829	-5.61588	0.50623
H	-4.30966	-5.61473	0.28368
H	-5.74632	-6.67064	0.52939
H	-5.55968	-5.16961	1.51036
C	-7.66146	-4.81887	-0.28779
H	-8.19252	-4.24557	-1.07761
H	-7.87709	-4.35589	0.69838
H	-8.06024	-5.85576	-0.28458
H	0.47372	-1.68630	0.96545
C	1.19691	-3.91639	2.01129
C	2.27049	-3.36920	0.01235
C	0.19382	-3.61122	2.96967
C	2.04450	-5.04881	2.21722
C	3.15988	-4.46418	0.15303
C	0.04583	-4.40709	4.08232
H	-0.43626	-2.74421	2.80301
C	1.86125	-5.84899	3.37505
C	3.04054	-5.29463	1.24256
H	3.94221	-4.61562	-0.57752
C	0.88167	-5.53361	4.28818
H	-0.72017	-4.17084	4.81462
H	2.50786	-6.70884	3.52743
H	3.71549	-6.13685	1.37110
H	0.74544	-6.14659	5.17378
C	2.32542	-2.38928	-1.14402
C	3.04842	-2.79754	-2.43897
H	2.77831	-2.05865	-3.20166
H	2.60098	-3.74546	-2.76309
N	4.49074	-2.96342	-2.36729
N	1.33138	-3.11575	0.91509
C	5.42104	-2.00183	-2.79455
C	5.08523	-0.67077	-3.09391
C	6.77142	-2.38756	-2.89359
C	6.07758	0.23793	-3.46127
H	4.06369	-0.32570	-3.00650
C	7.75088	-1.47225	-3.26325
H	7.04916	-3.41351	-2.66063
C	7.41447	-0.14668	-3.54753
H	5.79203	1.26430	-3.67456
H	8.78547	-1.79846	-3.32463
H	8.17992	0.57011	-3.82696
H	4.80314	-3.90482	-2.55285
H	1.29798	-2.11716	-1.40727
Cl	3.03835	-0.84033	-0.48508

Catalyst (TRIP, 3)-Product (S) alternative conformation
Electronic energy (Hartrees): -1870.551776
Free Energy at 1M concentration and 253 K (Hartrees): -1869.332302
Coordinates:

P	0.58404	-0.25456	0.25841
O	-0.12044	1.18692	0.59249
O	2.04420	0.05712	-0.39280

O	-0.14123	-0.84284	-1.01976
O	0.55238	-1.07190	1.48774
C	0.28917	2.31495	-0.07995
C	-0.76070	3.17668	-0.68545
C	-0.43884	4.36694	-1.21117
H	-1.20827	4.99672	-1.64332
C	0.96932	4.79654	-1.27481
C	1.32017	6.05678	-1.96975
H	0.54110	6.68566	-2.38252
C	2.60039	6.42151	-2.09463
H	2.85618	7.34137	-2.60451
C	3.66955	5.56209	-1.55298
H	4.70532	5.84869	-1.68199
C	3.36235	4.42495	-0.91841
H	4.16580	3.79731	-0.55698
C	1.93939	4.02211	-0.73802
C	1.56841	2.76537	-0.01425
C	2.85098	0.73793	0.48088
C	4.05715	0.07230	1.03179
C	4.82831	0.71793	1.91895
H	5.72785	0.24994	2.30138
C	4.46551	2.07568	2.37353
C	5.27318	2.74082	3.42166
H	6.14004	2.24300	3.83816
C	4.93382	3.95783	3.86031
H	5.52342	4.44251	4.62773
C	3.74690	4.63897	3.30853
H	3.46492	5.61414	3.68414
C	3.01421	4.04936	2.35673
H	2.13446	4.55634	1.98302
C	3.39638	2.70872	1.83638
C	2.59667	2.03014	0.77224
C	4.47347	-1.25160	0.51526
C	5.11841	-1.33279	-0.84022
C	4.30500	-2.38188	1.25611
C	5.51833	-2.53288	-1.30139
C	4.80902	-3.67511	0.72394
C	5.37240	-3.76006	-0.48913
H	5.97682	-2.61257	-2.27768
H	4.71547	-4.56597	1.33114
C	3.61657	-2.38220	2.62037
H	3.15129	-1.39768	2.83451
C	2.47427	-3.40830	2.67254
H	1.83206	-3.31807	1.77176
H	1.84520	-3.22926	3.57066
H	2.86166	-4.44731	2.72496
C	4.63379	-2.64593	3.73550
H	5.09634	-3.64981	3.62535
H	4.13512	-2.59127	4.72676
H	5.43611	-1.87845	3.70840
C	5.34233	-0.08727	-1.69839
H	4.99390	0.82216	-1.17103
C	4.54097	-0.16030	-3.00449
H	3.46573	-0.32953	-2.78267
H	4.90617	-0.98173	-3.65630
H	4.63275	0.79722	-3.56041
C	6.83402	0.14098	-1.97652
H	7.25649	-0.66505	-2.61254
H	7.39678	0.17694	-1.01913
H	6.97832	1.11048	-2.49968

C	-2.16982	2.72066	-0.71099
C	-2.93652	2.75002	0.41480
C	-2.75108	2.20872	-2.00008
C	-4.33490	2.25277	0.34746
C	-4.00995	1.73097	-2.00313
C	-4.84376	1.74397	-0.78262
H	-4.94808	2.29342	1.23698
H	-4.44277	1.34955	-2.91776
C	-2.43125	3.29233	1.75256
H	-1.37269	3.61585	1.68827
C	-3.22596	4.53516	2.17006
H	-3.17713	5.30293	1.36833
H	-2.79095	4.97166	3.09453
H	-4.29046	4.28809	2.36659
C	-2.48737	2.21493	2.84489
H	-1.93707	1.30733	2.51990
H	-3.53392	1.93013	3.08185
H	-2.01030	2.59259	3.77428
C	-1.95978	2.24040	-3.30792
H	-0.93740	2.63067	-3.14143
C	-2.61793	3.17619	-4.32946
H	-1.97676	3.26542	-5.23237
H	-2.74108	4.18947	-3.89063
H	-3.61355	2.79585	-4.64150
C	-1.77303	0.83657	-3.88846
H	-1.27618	0.19577	-3.13478
H	-1.12336	0.87711	-4.78871
H	-2.74229	0.37785	-4.17567
C	-6.25746	1.19239	-0.81040
H	-6.73780	1.32482	0.18466
C	-6.24401	-0.31142	-1.10516
H	-5.62407	-0.83887	-0.34954
H	-5.83162	-0.51877	-2.11556
H	-7.27535	-0.72041	-1.05212
C	-7.12681	1.94600	-1.82364
H	-7.10491	3.03563	-1.60759
H	-8.17978	1.59933	-1.75100
H	-6.77399	1.77799	-2.86295
C	5.86481	-5.09551	-1.01674
H	5.67125	-5.89539	-0.26673
C	5.11342	-5.48987	-2.29347
H	4.01895	-5.50162	-2.10225
H	5.42009	-6.50791	-2.61558
H	5.32570	-4.77999	-3.12077
C	7.37870	-5.06508	-1.25651
H	7.90563	-4.77229	-0.32315
H	7.64456	-4.34553	-2.05963
H	7.73643	-6.07368	-1.55476
H	-0.73423	-1.63338	-0.84415
C	-2.40118	-2.78605	-2.43455
C	-3.01171	-3.18495	-0.23048
C	-1.37609	-2.51550	-3.37897
C	-3.71709	-3.08391	-2.90993
C	-4.33843	-3.50744	-0.62205
C	-1.65906	-2.51168	-4.72624
H	-0.37628	-2.30734	-3.01654
C	-3.98010	-3.04893	-4.30377
C	-4.69372	-3.42711	-1.94525
H	-5.05858	-3.80098	0.13348
C	-2.97084	-2.76703	-5.19492

H	-0.86730	-2.30585	-5.44001
H	-4.98678	-3.26293	-4.65229
H	-5.70907	-3.64765	-2.26379
H	-3.17379	-2.74802	-6.26121
C	-2.65615	-3.32715	1.23958
C	-2.50712	-2.00054	2.01365
H	-1.70186	-1.40605	1.58518
H	-2.19246	-2.23256	3.03716
N	-3.74786	-1.25440	1.99511
N	-2.09321	-2.78932	-1.09870
C	-4.76090	-1.40637	2.93466
C	-4.77730	-2.46322	3.86495
C	-5.83100	-0.48988	2.95295
C	-5.82702	-2.58399	4.77609
H	-3.97062	-3.18742	3.88950
C	-6.87006	-0.62251	3.86578
H	-5.83736	0.33019	2.24252
C	-6.88115	-1.67211	4.78873
H	-5.81226	-3.40664	5.48586
H	-7.67796	0.10407	3.85593
H	-7.69356	-1.77426	5.50083
H	-3.72216	-0.36579	1.52128
H	-3.44431	-3.91631	1.71053
Cl	-1.14716	-4.32814	1.44548

Catalyst (TRIP, 3)-Product (R) alternative conformation
Electronic energy (Hartrees): -1870.553453
Free Energy at 1M concentration and 253 K (Hartrees): -1869.331419
Coordinates:

P	0.23205	0.19764	0.99343
O	-0.63874	1.53238	1.30181
O	1.32455	0.61536	-0.14278
O	-0.76035	-0.72192	0.18215
O	0.79919	-0.35933	2.24207
C	-0.48363	2.66302	0.53098
C	-1.72709	3.31656	0.05062
C	-1.69030	4.55734	-0.45128
H	-2.60021	5.02192	-0.81456
C	-0.39956	5.22160	-0.69420
C	-0.37761	6.53196	-1.38503
H	-1.30464	7.04772	-1.60290
C	0.78484	7.08045	-1.75018
H	0.79951	8.03612	-2.25807
C	2.04908	6.36982	-1.48892
H	2.98414	6.79716	-1.82762
C	2.04019	5.18958	-0.85916
H	2.97925	4.67118	-0.72737
C	0.75728	4.58835	-0.39164
C	0.71878	3.28446	0.35257
C	2.29524	1.41194	0.40108
C	3.68053	0.89737	0.49273
C	4.62517	1.63322	1.09692
H	5.64729	1.27649	1.14329
C	4.27215	2.92892	1.71747
C	5.29308	3.68197	2.48136
H	6.30260	3.29890	2.56406
C	4.97175	4.83684	3.07505
H	5.71811	5.38568	3.63464
C	3.59539	5.36108	2.97959
H	3.33751	6.28571	3.47969

C	2.66658	4.69169	2.28701
H	1.65791	5.08145	2.24421
C	3.01585	3.42214	1.59617
C	1.99823	2.66257	0.80914
C	4.01710	-0.38227	-0.17520
C	3.98034	-0.44464	-1.67789
C	4.25902	-1.51387	0.54401
C	4.10931	-1.63933	-2.28481
C	4.47393	-2.79533	-0.17836
C	4.35893	-2.87433	-1.51095
H	4.06552	-1.70650	-3.36329
H	4.68526	-3.69087	0.39118
C	4.26780	-1.54301	2.07217
H	3.93762	-0.57395	2.49782
C	3.28940	-2.59287	2.61668
H	2.31662	-2.50660	2.09368
H	3.12178	-2.43106	3.70294
H	3.67397	-3.62469	2.47594
C	5.68502	-1.79666	2.59564
H	6.06163	-2.78832	2.26602
H	5.69246	-1.76496	3.70615
H	6.37440	-1.01018	2.22120
C	3.84011	0.81846	-2.52918
H	3.77605	1.71816	-1.88518
C	2.55828	0.79427	-3.37378
H	1.67057	0.65145	-2.72414
H	2.58526	-0.02147	-4.12639
H	2.43797	1.76073	-3.90823
C	5.07541	1.03004	-3.41452
H	5.16689	0.23053	-4.17965
H	5.99379	1.03654	-2.78913
H	5.00544	2.00809	-3.93700
C	-2.97916	2.53192	-0.05843
C	-3.78377	2.32295	1.01900
C	-3.37526	1.99486	-1.40730
C	-5.08214	1.62250	0.82415
C	-4.55294	1.35435	-1.53344
C	-5.46801	1.18551	-0.38356
H	-5.74579	1.50137	1.67037
H	-4.86004	0.97149	-2.49706
C	-3.43634	2.82375	2.41961
H	-2.44130	3.31286	2.44085
C	-4.44113	3.88350	2.88616
H	-4.48328	4.71510	2.15043
H	-4.12413	4.30259	3.86505
H	-5.45848	3.45419	3.00181
C	-3.36304	1.65900	3.41582
H	-2.65965	0.88228	3.04714
H	-4.35937	1.19387	3.56854
H	-2.99316	2.02284	4.39811
C	-2.48676	2.18413	-2.64058
H	-1.56415	2.73925	-2.38354
C	-3.19829	3.01597	-3.71565
H	-2.49485	3.24981	-4.54321
H	-3.55114	3.97565	-3.28089
H	-4.06760	2.47033	-4.13914
C	-2.00445	0.84469	-3.20684
H	-1.37797	0.32998	-2.45098
H	-1.37310	1.01326	-4.10534
H	-2.85495	0.19089	-3.49158

C	-6.85281	0.59170	-0.58174
H	-7.39820	0.56699	0.38867
C	-6.77979	-0.85306	-1.08732
H	-6.27191	-1.48841	-0.33648
H	-6.23325	-0.92055	-2.05131
H	-7.80364	-1.25865	-1.23305
C	-7.68099	1.46112	-1.53527
H	-7.71887	2.50677	-1.16133
H	-8.72183	1.07714	-1.59406
H	-7.24681	1.46060	-2.55764
C	4.48459	-4.21318	-2.21481
H	4.69110	-5.01582	-1.47122
C	3.17152	-4.58227	-2.91496
H	2.33923	-4.59419	-2.17879
H	3.25051	-5.59537	-3.36366
H	2.92813	-3.85677	-3.72001
C	5.65591	-4.20990	-3.20387
H	6.59376	-3.92288	-2.68184
H	5.47723	-3.49985	-4.03867
H	5.79401	-5.22602	-3.63118
H	-0.57637	-1.72546	0.22912
C	-0.30585	-4.07434	1.42640
C	-0.57912	-4.14095	-0.88252
C	-0.24947	-3.31859	2.62583
C	-0.17572	-5.49931	1.49650
C	-0.45312	-5.55380	-0.89484
C	-0.06174	-3.95651	3.83115
H	-0.30658	-2.23804	2.57927
C	0.01849	-6.12186	2.75644
C	-0.24661	-6.22409	0.28525
H	-0.51315	-6.08804	-1.83716
C	0.07292	-5.36416	3.90274
H	-0.00771	-3.36655	4.74073
H	0.11939	-7.20300	2.79621
H	-0.13801	-7.30534	0.30005
H	0.22170	-5.84240	4.86608
C	-0.88578	-3.45342	-2.19360
H	-0.72258	-4.16622	-3.00442
C	-2.34806	-2.93464	-2.33892
H	-2.98089	-3.81878	-2.45277
H	-2.39036	-2.39863	-3.29418
N	-2.87757	-2.10472	-1.29246
N	-0.48795	-3.42732	0.23121
C	-3.62134	-2.55321	-0.19898
C	-4.30843	-3.77955	-0.17401
C	-3.74164	-1.70042	0.91908
C	-5.09195	-4.12730	0.92746
H	-4.25195	-4.46565	-1.01250
C	-4.52836	-2.05882	2.00651
H	-3.20566	-0.75730	0.92107
C	-5.21426	-3.27656	2.02460
H	-5.61637	-5.07923	0.91766
H	-4.60295	-1.37744	2.84948
H	-5.82820	-3.55477	2.87519
H	-2.34183	-1.27097	-1.09606
Cl	0.31876	-2.12298	-2.54228

Catalyst (TRIP, 3)-Product (R) alternative conformation
Electronic energy (Hartrees): -1870.553205
Free Energy at 1M concentration and 253 K (Hartrees): -1869.331116

Coordinates:

P	-0.22498	0.25021	-0.79893
O	0.91503	1.36256	-1.10946
O	-1.25655	0.90443	0.27728
O	0.50463	-0.83731	0.09191
O	-0.83679	-0.21223	-2.06396
C	1.08005	2.45116	-0.28197
C	2.47352	2.79267	0.11384
C	2.74310	3.96846	0.69789
H	3.76060	4.21340	0.98113
C	1.64969	4.86283	1.10841
C	1.95851	6.07492	1.90225
H	2.98768	6.35230	2.09384
C	0.96882	6.82787	2.39210
H	1.19513	7.71154	2.97468
C	-0.43495	6.44075	2.16273
H	-1.23191	7.03099	2.59641
C	-0.72315	5.35166	1.44114
H	-1.75998	5.06702	1.32615
C	0.36727	4.53003	0.84138
C	0.07261	3.32734	-0.00535
C	-1.94727	1.95783	-0.25843
C	-3.42055	1.85441	-0.41433
C	-4.11744	2.91083	-0.86142
H	-5.19679	2.85736	-0.94125
C	-3.41333	4.12648	-1.31761
C	-4.17765	5.22759	-1.94748
H	-5.25507	5.16165	-2.03443
C	-3.53999	6.30640	-2.41509
H	-4.09896	7.10999	-2.87688
C	-2.07048	6.39837	-2.31631
H	-1.55876	7.26369	-2.71741
C	-1.37140	5.41122	-1.74428
H	-0.29273	5.48662	-1.70108
C	-2.06921	4.22030	-1.18963
C	-1.31307	3.11870	-0.52005
C	-4.11231	0.60556	-0.00931
C	-4.12667	0.21910	1.44566
C	-4.68376	-0.21913	-0.93460
C	-4.62831	-0.97894	1.79795
C	-5.25277	-1.51836	-0.48723
C	-5.19891	-1.90198	0.79531
H	-4.65048	-1.27242	2.83860
H	-5.70092	-2.17739	-1.21903
C	-4.77135	0.12516	-2.42386
H	-4.23021	1.06111	-2.66162
C	-4.11721	-0.95005	-3.29748
H	-3.07300	-1.11085	-2.96907
H	-4.09596	-0.61768	-4.35739
H	-4.66616	-1.91308	-3.24862
C	-6.23102	0.34435	-2.83709
H	-6.82357	-0.58837	-2.72508
H	-6.28270	0.67093	-3.89776
H	-6.68948	1.13707	-2.20827
C	-3.65346	1.17946	2.53787
H	-3.25929	2.11420	2.09270
C	-2.51274	0.57726	3.37225
H	-1.69424	0.22260	2.71312
H	-2.86715	-0.27683	3.98663
H	-2.09584	1.34812	4.05496

C	-4.82056	1.60644	3.43734
H	-5.22780	0.74445	4.00695
H	-5.63361	2.04560	2.82032
H	-4.48035	2.37715	4.16172
C	3.56056	1.79460	-0.02828
C	4.12413	1.52509	-1.23868
C	4.09224	1.12764	1.21174
C	5.27394	0.58323	-1.30502
C	5.14942	0.30116	1.10593
C	5.78515	0.02296	-0.19943
H	5.73724	0.38656	-2.26310
H	5.55970	-0.16815	1.98952
C	3.67978	2.20942	-2.53136
H	2.81530	2.88174	-2.36007
C	4.79848	3.09686	-3.08987
H	5.11567	3.83453	-2.32182
H	4.43268	3.65634	-3.97732
H	5.67983	2.49406	-3.39432
C	3.22477	1.18028	-3.57444
H	2.44743	0.51418	-3.14410
H	4.07378	0.55750	-3.92598
H	2.78679	1.69999	-4.45319
C	3.47760	1.38574	2.58976
H	2.60785	2.06644	2.51469
C	4.48161	2.06751	3.52818
H	3.98217	2.34874	4.47989
H	4.87388	2.99377	3.05679
H	5.33356	1.39568	3.76433
C	2.92566	0.10065	3.21199
H	2.16378	-0.33461	2.53457
H	2.43162	0.32622	4.18120
H	3.72970	-0.64271	3.39279
C	7.03555	-0.83584	-0.27873
H	7.34609	-0.95702	-1.34113
C	6.78648	-2.24213	0.27566
H	5.94506	-2.71390	-0.26528
H	6.54830	-2.21662	1.35966
H	7.68757	-2.87468	0.12777
C	8.19974	-0.16075	0.45492
H	8.37497	0.85397	0.03750
H	9.12915	-0.75492	0.32300
H	7.98906	-0.06994	1.54186
C	-5.72753	-3.26105	1.21558
H	-6.08938	-3.82055	0.32347
C	-4.61538	-4.10393	1.85002
H	-3.74698	-4.16021	1.16219
H	-4.97920	-5.13654	2.03778
H	-4.27782	-3.66695	2.81364
C	-6.91925	-3.11596	2.16841
H	-7.71307	-2.50091	1.69303
H	-6.61574	-2.63521	3.12242
H	-7.34556	-4.11586	2.39783
H	0.10217	-1.76337	0.07500
C	-1.20171	-4.02953	-0.75792
C	-0.12796	-4.18536	1.29484
C	-1.42717	-3.32151	-1.96603
C	-1.80487	-5.31904	-0.58877
C	-0.70164	-5.45821	1.54329
C	-2.21008	-3.87998	-2.95251
H	-0.98476	-2.34041	-2.10382

C	-2.62845	-5.84834	-1.61496
C	-1.54047	-6.01557	0.61216
H	-0.46723	-5.97658	2.46682
C	-2.82423	-5.14302	-2.77867
H	-2.35952	-3.33975	-3.88205
H	-3.08835	-6.82158	-1.46683
H	-1.99426	-6.98880	0.77941
H	-3.44605	-5.55294	-3.56868
C	0.85015	-3.64432	2.31591
H	1.05157	-4.43518	3.04130
C	2.18172	-3.14352	1.73149
H	2.82244	-2.82641	2.56490
H	1.99983	-2.26814	1.11531
N	2.79615	-4.18945	0.93877
N	-0.39058	-3.48024	0.20385
C	3.39401	-3.98884	-0.31165
C	4.24237	-4.99611	-0.81165
C	3.17116	-2.84921	-1.10558
C	4.85185	-4.86551	-2.05508
H	4.42207	-5.88557	-0.21117
C	3.79072	-2.73257	-2.34981
H	2.51973	-2.05072	-0.77290
C	4.63498	-3.72921	-2.83752
H	5.50544	-5.65753	-2.41036
H	3.60543	-1.84091	-2.94094
H	5.11411	-3.62407	-3.80554
H	3.24289	-4.91553	1.48117
Cl	0.07304	-2.31902	3.32056

Catalyst (TRIP, 3)-Product (S) alternative conformation
Electronic energy (Hartrees): -1870.549753
Free Energy at 1M concentration and 253 K (Hartrees): -1869.329422
Coordinates:

P	-0.40534	0.20742	1.12849
O	-1.92576	0.79074	1.11143
O	0.46964	1.12429	0.09727
O	-0.45411	-1.16239	0.34082
O	0.09062	0.17090	2.52286
C	-2.32875	1.65420	0.11770
C	-3.61712	1.35875	-0.56086
C	-4.17125	2.25644	-1.38675
H	-5.10994	2.03308	-1.88112
C	-3.45503	3.49822	-1.72380
C	-4.02347	4.41627	-2.73792
H	-4.99562	4.21453	-3.17044
C	-3.33691	5.49453	-3.12871
H	-3.75171	6.16322	-3.87187
C	-2.00183	5.76383	-2.56418
H	-1.43591	6.61931	-2.90991
C	-1.48285	4.94432	-1.64281
H	-0.48642	5.14554	-1.27414
C	-2.25291	3.76143	-1.16273
C	-1.70588	2.84073	-0.11415
C	0.59902	2.40334	0.57514
C	1.93617	2.90295	0.97657
C	2.05439	4.11376	1.54116
H	3.03249	4.49926	1.80460
C	0.85524	4.94414	1.77866
C	0.98413	6.24058	2.48276
H	1.95575	6.58541	2.81405

C	-0.09934	6.99011	2.71405
H	-0.00468	7.93683	3.23002
C	-1.42932	6.52426	2.27580
H	-2.30423	7.12662	2.48371
C	-1.55581	5.35727	1.63340
H	-2.54188	5.02371	1.33783
C	-0.35688	4.52596	1.34394
C	-0.47203	3.22084	0.62681
C	3.14504	2.13509	0.60518
C	3.59093	2.14144	-0.82989
C	3.84453	1.42461	1.53130
C	4.66071	1.40495	-1.18388
C	5.05140	0.67089	1.10093
C	5.43207	0.63548	-0.18366
H	4.98850	1.38650	-2.21447
H	5.62377	0.12725	1.84119
C	3.43295	1.35024	3.00013
H	2.45036	1.83708	3.16899
C	3.26636	-0.10499	3.45721
H	2.65133	-0.66589	2.72307
H	2.75562	-0.13482	4.44311
H	4.24611	-0.61649	3.56029
C	4.44721	2.08627	3.88149
H	5.44926	1.61081	3.82187
H	4.11112	2.07419	4.94035
H	4.53368	3.14518	3.55688
C	2.87227	2.98006	-1.88749
H	2.03462	3.54884	-1.43853
C	2.25567	2.09406	-2.97575
H	1.60941	1.31593	-2.51619
H	3.04551	1.59967	-3.57715
H	1.62864	2.70847	-3.65656
C	3.80801	4.03260	-2.49644
H	4.62928	3.55938	-3.07477
H	4.24898	4.65849	-1.69122
H	3.23750	4.69792	-3.17933
C	-4.25313	0.03304	-0.39201
C	-4.95043	-0.27419	0.73621
C	-4.12466	-0.97969	-1.49667
C	-5.57387	-1.61842	0.85391
C	-4.70644	-2.18468	-1.34645
C	-5.46719	-2.52810	-0.12494
H	-6.13589	-1.86036	1.74660
H	-4.63117	-2.92407	-2.13221
C	-5.15632	0.71936	1.88011
H	-4.63106	1.67691	1.68794
C	-6.64013	1.07458	2.03063
H	-7.03439	1.46886	1.06950
H	-6.76629	1.86085	2.80540
H	-7.23996	0.18892	2.32846
C	-4.58704	0.17459	3.19693
H	-3.52476	-0.12021	3.06422
H	-5.15970	-0.70749	3.55226
H	-4.63262	0.95901	3.98234
C	-3.37038	-0.66453	-2.79061
H	-2.92529	0.34834	-2.75141
C	-4.31590	-0.67314	-3.99810
H	-3.77284	-0.34245	-4.90917
H	-5.15938	0.02872	-3.82386
H	-4.72531	-1.68888	-4.18200

C	-2.19022	-1.61670	-3.00795
H	-1.52177	-1.58493	-2.12408
H	-1.60105	-1.29895	-3.89469
H	-2.53330	-2.65938	-3.17222
C	-6.13519	-3.88472	0.01170
H	-6.65765	-3.95399	0.99253
C	-5.09962	-5.01386	-0.02163
H	-4.33168	-4.84891	0.76421
H	-4.59836	-5.06938	-1.01095
H	-5.59411	-5.98866	0.17670
C	-7.19837	-4.08233	-1.07490
H	-7.93822	-3.25421	-1.03798
H	-7.73933	-5.03820	-0.90789
H	-6.73981	-4.10916	-2.08609
C	6.64264	-0.17711	-0.60451
H	7.08665	-0.68569	0.28089
C	6.24176	-1.27470	-1.59566
H	5.44572	-1.91276	-1.15536
H	7.11628	-1.91974	-1.82593
H	5.86738	-0.83711	-2.54464
C	7.73148	0.72610	-1.19481
H	8.00383	1.51923	-0.46595
H	7.38994	1.20564	-2.13652
H	8.64228	0.12899	-1.41411
H	-0.11054	-1.96868	0.83226
C	1.05431	-3.67814	2.70262
C	0.77564	-4.56622	0.57678
C	0.81421	-2.63117	3.62941
C	1.75066	-4.84989	3.14892
C	1.44712	-5.76034	0.94383
C	1.25616	-2.74707	4.92847
H	0.30858	-1.72972	3.30018
C	2.19891	-4.92988	4.49296
C	1.94445	-5.89289	2.21648
H	1.55198	-6.56079	0.21874
C	1.95527	-3.89736	5.36750
H	1.06953	-1.93562	5.62516
H	2.72944	-5.82175	4.81517
H	2.46678	-6.79601	2.52092
H	2.29676	-3.96098	6.39622
C	0.21466	-4.48255	-0.83020
C	0.82179	-3.40684	-1.73696
H	0.67680	-2.42228	-1.28718
H	0.27474	-3.42203	-2.69161
N	2.24488	-3.66851	-1.92029
N	0.60091	-3.55344	1.41349
C	2.94126	-3.02115	-2.94770
C	4.15760	-3.56600	-3.40194
C	2.47464	-1.83411	-3.53796
C	4.87720	-2.94883	-4.41832
H	4.53332	-4.47961	-2.94634
C	3.20471	-1.22927	-4.56198
H	1.54729	-1.38236	-3.20386
C	4.40549	-1.77458	-5.01189
H	5.81273	-3.39038	-4.75028
H	2.81674	-0.32478	-5.02112
H	4.96468	-1.29632	-5.80954
H	2.49037	-4.64837	-1.85555
H	0.35368	-5.45592	-1.30773
Cl	-1.59722	-4.27897	-0.76749

Catalyst (TRIP, 3)-Product (R) alternative conformation
Electronic energy (Hartrees): -1870.550195
Free Energy at 1M concentration and 253 K (Hartrees): -1869.32738
Coordinates:

P	0.73088	-0.17860	-0.73367
O	-0.00275	-1.57527	-1.14026
O	1.71510	-0.50894	0.52490
O	-0.35656	0.72017	-0.01774
O	1.39908	0.40622	-1.91674
C	-0.13115	-2.58507	-0.21013
C	-1.47110	-3.21831	-0.05988
C	-1.61737	-4.33196	0.67219
H	-2.59301	-4.79541	0.76787
C	-0.48717	-4.86461	1.44991
C	-0.70639	-5.99752	2.37882
H	-1.67535	-6.47827	2.43173
C	0.28920	-6.42803	3.16032
H	0.12782	-7.25411	3.84079
C	1.60489	-5.76377	3.11277
H	2.39728	-6.09493	3.77158
C	1.81361	-4.74402	2.27216
H	2.77644	-4.25117	2.28106
C	0.72822	-4.28073	1.36212
C	0.94596	-3.16409	0.38734
C	2.67261	-1.43294	0.20186
C	4.09893	-1.01953	0.15286
C	5.05413	-1.94683	-0.01766
H	6.09858	-1.66029	-0.01375
C	4.68893	-3.34784	-0.30524
C	5.74668	-4.32920	-0.63938
H	6.78865	-4.03509	-0.61592
C	5.41936	-5.58130	-0.97650
H	6.19272	-6.29735	-1.22277
C	4.00161	-5.98602	-1.03107
H	3.74225	-6.99325	-1.33065
C	3.03900	-5.10821	-0.72585
H	2.00486	-5.41789	-0.79861
C	3.38866	-3.72173	-0.31288
C	2.33440	-2.73048	0.06321
C	4.46055	0.41082	0.31096
C	4.27557	1.07013	1.65082
C	4.94771	1.13440	-0.73998
C	4.54041	2.38401	1.77380
C	5.24803	2.57705	-0.54261
C	5.02604	3.18477	0.63107
H	4.42778	2.87186	2.73235
H	5.64719	3.14949	-1.36935
C	5.22948	0.52310	-2.11672
H	4.83106	-0.50790	-2.19168
C	4.54861	1.30048	-3.25081
H	3.47671	1.45973	-3.02194
H	4.61481	0.72141	-4.19675
H	5.03548	2.28282	-3.42089
C	6.74066	0.43830	-2.36147
H	7.19658	1.45084	-2.39267
H	6.94309	-0.07212	-3.32732
H	7.22913	-0.14512	-1.55314
C	3.85993	0.27484	2.88866
H	3.66370	-0.78326	2.62661

C	2.56167	0.81949	3.50299
H	1.76817	0.89388	2.73045
H	2.71593	1.82403	3.94991
H	2.20458	0.13426	4.30120
C	4.98737	0.24523	3.92864
H	5.19487	1.26010	4.32892
H	5.91597	-0.15652	3.46935
H	4.70575	-0.41580	4.77608
C	-2.66168	-2.60965	-0.69600
C	-2.88797	-2.73520	-2.03308
C	-3.63048	-1.84268	0.16031
C	-4.09336	-2.09495	-2.62374
C	-4.68293	-1.23969	-0.42605
C	-4.94847	-1.38568	-1.87351
H	-4.28690	-2.21464	-3.68180
H	-5.37448	-0.65663	0.16725
C	-1.97689	-3.54585	-2.95553
H	-1.10423	-3.95684	-2.40862
C	-2.72302	-4.75633	-3.52878
H	-3.12772	-5.37812	-2.70158
H	-2.02734	-5.38422	-4.12561
H	-3.56175	-4.44136	-4.18466
C	-1.40619	-2.67151	-4.08000
H	-0.90518	-1.77648	-3.65491
H	-2.20262	-2.33649	-4.77698
H	-0.65335	-3.24578	-4.66113
C	-3.45235	-1.73496	1.67637
H	-2.60857	-2.35808	2.02648
C	-4.68886	-2.24621	2.43012
H	-4.47692	-2.29252	3.51974
H	-4.94752	-3.26994	2.08460
H	-5.56391	-1.58103	2.27657
C	-3.10303	-0.30605	2.08741
H	-2.10041	-0.04417	1.69601
H	-3.07503	-0.21127	3.19386
H	-3.84233	0.40782	1.68123
C	-6.19325	-0.77511	-2.49401
H	-6.24504	-1.03402	-3.57564
C	-6.15848	0.75287	-2.40260
H	-5.25005	1.14205	-2.90961
H	-6.15410	1.08707	-1.34499
H	-7.04864	1.18659	-2.90609
C	-7.46358	-1.33285	-1.84019
H	-7.46448	-2.44252	-1.89513
H	-8.36188	-0.95842	-2.37593
H	-7.53773	-1.02505	-0.77546
C	5.30234	4.66833	0.79516
H	5.57431	5.11361	-0.18858
C	4.05694	5.41348	1.29362
H	3.18276	5.17497	0.65390
H	4.22957	6.51001	1.25180
H	3.81352	5.13689	2.34110
C	6.48832	4.89612	1.73830
H	7.38614	4.36601	1.35434
H	6.26340	4.52527	2.76099
H	6.72428	5.98007	1.79808
H	-0.32441	1.69299	-0.27149
C	0.52989	4.26310	-0.97125
C	-1.53804	4.07003	0.08054
C	1.66943	3.64043	-1.54625

C	0.42119	5.69178	-1.01685
C	-1.72991	5.47493	0.04792
C	2.64917	4.41761	-2.12382
H	1.73336	2.55621	-1.55868
C	1.46099	6.45754	-1.60402
C	-0.74863	6.27556	-0.48264
H	-2.63842	5.91407	0.43947
C	2.55649	5.83022	-2.14703
H	3.51150	3.94527	-2.57776
H	1.36831	7.53993	-1.62361
H	-0.86770	7.35554	-0.50855
H	3.35180	6.41284	-2.60161
C	-2.59680	3.14644	0.65705
H	-2.09409	2.32504	1.16900
C	-3.60140	3.82877	1.61195
H	-4.21440	4.54255	1.05722
H	-3.00344	4.41574	2.32253
N	-4.46426	2.96018	2.37116
N	-0.44590	3.48917	-0.40060
C	-5.71838	2.47729	1.98991
C	-6.27982	1.41597	2.72594
C	-6.46965	3.02323	0.93497
C	-7.53315	0.90676	2.40426
H	-5.71381	0.98062	3.54687
C	-7.72822	2.50866	0.62823
H	-6.06821	3.82811	0.33092
C	-8.27179	1.44609	1.34956
H	-7.93052	0.07679	2.98192
H	-8.28156	2.94069	-0.20102
H	-9.24629	1.04373	1.09286
H	-4.00393	2.41843	3.08730
Cl	-3.47170	2.34360	-0.73686

Catalyst (H, 4)-THF complex
Electronic energy (Hartrees): -876.524279
Free Energy at 1M concentration and 253 K (Hartrees): -876.137746
Coordinates:

P	1.83106	-0.56863	-0.61274
O	0.61120	-0.83084	-1.66301
O	1.17172	0.08026	0.74859
O	2.65242	-1.73707	-0.22635
O	2.54723	0.61375	-1.38479
C	-0.48669	-1.43309	-1.10290
C	-0.83292	-2.80796	-1.51674
C	-1.91354	-3.41303	-1.01708
H	-2.15571	-4.42122	-1.32961
C	-2.75965	-2.72125	-0.02220
C	-3.91572	-3.42754	0.57667
H	-4.16310	-4.43284	0.25899
C	-4.65749	-2.82818	1.51408
H	-5.50117	-3.34562	1.95223
C	-4.31970	-1.46474	1.96477
H	-4.90999	-0.99489	2.74091
C	-3.28044	-0.81564	1.42700
H	-3.03771	0.17328	1.79154
C	-2.46402	-1.45510	0.35885
C	-1.30126	-0.74974	-0.26598
C	0.20017	1.02511	0.52814
C	0.45754	2.41541	0.95449
C	-0.47423	3.35760	0.79002

H	-0.27140	4.37291	1.10747
C	-1.76505	3.02320	0.15319
C	-2.76344	4.09064	-0.08719
H	-2.56459	5.10455	0.23717
C	-3.91349	3.80787	-0.70808
H	-4.64181	4.58882	-0.88468
C	-4.18732	2.43313	-1.16761
H	-5.10970	2.21641	-1.69091
C	-3.29450	1.46017	-0.95126
H	-3.50717	0.46588	-1.31989
C	-2.02359	1.75022	-0.23199
C	-1.01064	0.67997	0.03096
H	3.49868	0.72754	-1.07141
O	4.94369	0.79638	-0.37780
C	5.71455	-0.42043	-0.60344
C	4.81549	1.04061	1.04661
C	6.28128	-0.81080	0.76283
H	5.03305	-1.18665	-0.98436
H	6.47992	-0.19590	-1.35180
C	5.21128	-0.26978	1.72410
H	5.48604	1.86615	1.31760
H	3.78334	1.34280	1.24528
H	7.24468	-0.31920	0.93963
H	6.42758	-1.89024	0.85010
H	5.57938	-0.11850	2.74247
H	4.35379	-0.94797	1.75037
H	1.40779	2.67041	1.40623
H	-0.20763	-3.32608	-2.23269

Catalyst (H, 4)-Quinoline complex
Electronic energy (Hartrees): -1583.002551
Free Energy at 1M concentration and 253 K (Hartrees): -1582.579005
Coordinates:

P	-0.40787	-1.38380	1.54870
O	-0.06414	-1.22795	-0.06587
O	0.92416	-0.92170	2.37155
O	-0.83519	-2.74079	1.94220
O	-1.41097	-0.18086	1.74359
C	1.18671	-1.66056	-0.42417
C	1.30600	-2.82712	-1.32147
C	2.50629	-3.24434	-1.73255
H	2.57924	-4.10141	-2.39047
C	3.73046	-2.55414	-1.27456
C	5.05892	-3.06260	-1.68758
H	5.13284	-3.91685	-2.34905
C	6.17290	-2.47052	-1.24392
H	7.14324	-2.84352	-1.54527
C	6.08331	-1.31447	-0.33179
H	6.98923	-0.85337	0.04010
C	4.88832	-0.84431	0.04446
H	4.84816	-0.00962	0.73118
C	3.63819	-1.47302	-0.46323
C	2.28909	-0.96295	-0.06596
C	1.48658	0.24524	1.91453
C	1.42128	1.44931	2.76721
C	1.93627	2.60631	2.34381
H	1.87693	3.48277	2.97705
C	2.55673	2.69842	1.00570
C	3.05099	4.00522	0.51431
H	2.99598	4.88111	1.14874

C	3.56256	4.11058	-0.71663
H	3.92094	5.06658	-1.07594
C	3.62787	2.92675	-1.59429
H	4.02224	3.02417	-2.59756
C	3.19027	1.73990	-1.15813
H	3.22611	0.89230	-1.82890
C	2.64490	1.59839	0.21969
C	2.13752	0.28630	0.72878
H	-2.11060	-0.05342	1.01075
C	-4.30747	-0.75228	-0.09852
C	-3.46688	1.33113	-0.73604
C	-4.12547	-1.97504	0.60165
C	-5.53063	-0.52533	-0.80553
C	-4.65773	1.63435	-1.44951
C	-5.12233	-2.92520	0.58548
H	-3.20106	-2.16104	1.13840
C	-6.53533	-1.52596	-0.79628
C	-5.67192	0.71053	-1.48213
H	-4.74523	2.58276	-1.96401
C	-6.33387	-2.70392	-0.11393
H	-4.97481	-3.85962	1.11779
H	-7.46099	-1.34531	-1.33573
H	-6.58817	0.91712	-2.02880
H	-7.10371	-3.46936	-0.10944
N	-3.31172	0.18685	-0.07814
C	-2.31938	2.27650	-0.73256
C	-1.03431	1.92063	-0.75140
H	-0.24626	2.66466	-0.74664
H	-0.75816	0.87252	-0.77309
Cl	-2.74926	3.99373	-0.74097
H	0.94838	1.39317	3.73938
H	0.41351	-3.34430	-1.65017

Catalyst (H, 4)-Enamine protonation TS (pro-S) lowest free energy conformation
Electronic energy (Hartrees): -1870.615112
Free Energy at 1M concentration and 253 K (Hartrees): -1870.077309
Coordinates:

P	0.19902	-0.98148	0.85542
O	-0.49155	-0.04359	-0.32289
O	-1.01006	-1.46414	1.84673
O	1.20542	-0.22704	1.66165
O	0.68262	-2.21080	0.04945
C	-1.38222	0.88247	0.15466
C	-1.05313	2.31662	0.03363
C	-1.91454	3.24616	0.45378
H	-1.66025	4.29448	0.35785
C	-3.19702	2.84608	1.07007
C	-4.11494	3.88312	1.59518
H	-3.86465	4.93202	1.49558
C	-5.25550	3.52884	2.19679
H	-5.92503	4.28728	2.58151
C	-5.59871	2.10228	2.35058
H	-6.51242	1.82235	2.85888
C	-4.77895	1.15724	1.87571
H	-5.04030	0.11781	2.02131
C	-3.52096	1.53458	1.17515
C	-2.59006	0.49933	0.62674
C	-2.16131	-1.85059	1.20660
C	-2.58121	-3.26356	1.29848
C	-3.71490	-3.67208	0.72328

H	-4.00995	-4.71129	0.79909
C	-4.54890	-2.71766	-0.03625
C	-5.76682	-3.19726	-0.72954
H	-6.06654	-4.23467	-0.64764
C	-6.50012	-2.35233	-1.46195
H	-7.38902	-2.70405	-1.96956
C	-6.09176	-0.94092	-1.59273
H	-6.67632	-0.26832	-2.20715
C	-4.99621	-0.49585	-0.96645
H	-4.70350	0.53682	-1.09891
C	-4.18673	-1.41477	-0.11952
C	-2.96087	-0.94267	0.59821
H	3.32510	0.53255	-0.55907
C	2.36621	1.84014	-1.90549
C	2.52655	-0.57283	-2.04759
C	2.67296	3.01519	-1.19434
C	1.59529	1.89465	-3.09360
C	1.77090	-0.53181	-3.26267
C	2.20386	4.22914	-1.66369
H	3.26007	2.95371	-0.28347
C	1.13452	3.15021	-3.54569
C	1.32620	0.65855	-3.75921
H	1.55155	-1.46789	-3.75742
C	1.43161	4.30153	-2.84091
H	2.43210	5.13706	-1.11438
H	0.54070	3.19449	-4.45416
H	0.74492	0.67802	-4.67706
H	1.07194	5.26342	-3.19113
C	2.86011	-1.77604	-1.32677
C	4.02949	-1.76086	-0.33215
H	4.96729	-1.58886	-0.87574
H	4.10175	-2.75455	0.13008
N	3.85766	-0.69655	0.66331
N	2.81485	0.61722	-1.46486
C	4.87743	-0.43828	1.60860
C	4.52561	0.28123	2.76650
C	6.21406	-0.82213	1.42778
C	5.49348	0.61570	3.70652
H	3.48482	0.55589	2.91411
C	7.17661	-0.47533	2.37929
H	6.51342	-1.39427	0.55677
C	6.82912	0.24510	3.51972
H	5.20088	1.16672	4.59610
H	8.20715	-0.78207	2.22254
H	7.58206	0.50728	4.25645
H	2.93279	-0.71735	1.11469
H	1.67824	-2.00914	-0.59807
Cl	2.99002	-3.24520	-2.37294
H	-1.96606	-3.97142	1.83939
H	-0.11036	2.61536	-0.40272

Catalyst (H, 4)-Enamine protonation TS (pro-S) alternative conformation
Electronic energy (Hartrees): -1870.612173
Free Energy at 1M concentration and 253 K (Hartrees): -1870.074897
Coordinates:

P	-0.32950	-0.40844	-0.20898
O	0.74770	0.58768	0.50455
O	0.52365	-1.60427	-0.93152
O	-0.98166	0.27224	-1.44288
O	-1.25645	-0.88615	0.87329

C	1.82265	0.91549	-0.28196
C	1.97040	2.31085	-0.74132
C	3.02026	2.67058	-1.48383
H	3.11611	3.69747	-1.81408
C	4.03134	1.66666	-1.87717
C	5.15481	2.06191	-2.75776
H	5.25701	3.09051	-3.08068
C	6.04632	1.15003	-3.16018
H	6.86613	1.43946	-3.80491
C	5.90739	-0.25678	-2.73840
H	6.61911	-0.99394	-3.08720
C	4.89903	-0.62392	-1.93902
H	4.80414	-1.66545	-1.66334
C	3.91655	0.38422	-1.45491
C	2.78372	0.00362	-0.55427
C	1.56384	-2.09723	-0.18374
C	1.49796	-3.49726	0.28173
C	2.50636	-4.02764	0.97804
H	2.44400	-5.05570	1.31266
C	3.68910	-3.20831	1.31473
C	4.76896	-3.78583	2.14803
H	4.71108	-4.81635	2.47583
C	5.81865	-3.03676	2.50174
H	6.60646	-3.45914	3.11203
C	5.89925	-1.62742	2.07366
H	6.73959	-1.01995	2.38416
C	4.93187	-1.09705	1.31641
H	5.00116	-0.05457	1.03696
C	3.77279	-1.92610	0.88504
C	2.68018	-1.36146	0.03176
H	-2.00114	0.74703	-1.26140
C	-3.34158	1.29362	-0.84991
C	-2.99889	1.98077	0.47004
H	-2.73148	1.20517	1.19438
H	-3.88369	2.51205	0.86699
N	-1.86202	2.87225	0.30839
H	-1.86765	3.35669	-0.57997
C	-1.39465	3.64837	1.36583
C	-0.60652	4.78442	1.09125
C	-1.64705	3.32335	2.71116
C	-0.08134	5.55366	2.12257
H	-0.40845	5.05379	0.05616
C	-1.11366	4.10426	3.73656
H	-2.24803	2.45635	2.96110
C	-0.32848	5.22206	3.45800
H	0.52320	6.42363	1.88010
H	-1.32027	3.82978	4.76763
H	0.08109	5.82509	4.26217
C	-4.17036	0.11915	-0.81359
C	-5.31546	-0.12588	-1.64021
C	-4.44401	-2.09972	0.12548
C	-6.00669	-1.29988	-1.54877
H	-5.63006	0.65588	-2.31689
C	-3.94679	-3.09251	0.99311
C	-5.60109	-2.33877	-0.65699
H	-6.89013	-1.45446	-2.16274
C	-4.61710	-4.29849	1.08738
H	-3.04174	-2.89644	1.55869
C	-6.26422	-3.57938	-0.53095
C	-5.78234	-4.54545	0.33144

H	-4.23795	-5.06739	1.75348
H	-7.15396	-3.76128	-1.12707
H	-6.29408	-5.49771	0.42485
N	-3.80013	-0.88783	0.02363
H	-2.86065	-0.82755	0.48508
Cl	-3.87444	2.52975	-2.07630
H	0.62649	-4.09764	0.05309
H	1.22334	3.04555	-0.47269

Catalyst (H, 4)-Enamine protonation TS (pro-S) alternative conformation
Electronic energy (Hartrees): -1870.612172
Free Energy at 1M concentration and 253 K (Hartrees): -1870.074897
Coordinates:

P	0.32994	0.40838	-0.20960
O	-0.52303	1.60414	-0.93242
O	-0.74742	-0.58752	0.50400
O	1.25680	0.88621	0.87269
O	0.98220	-0.27258	-1.44331
C	-1.56303	2.09749	-0.18464
C	-1.49674	3.49756	0.28068
C	-2.50490	4.02825	0.97709
H	-2.44225	5.05633	1.31160
C	-3.68779	3.20924	1.31405
C	-4.76738	3.78710	2.14746
H	-4.70921	4.81764	2.47514
C	-5.81719	3.03832	2.50140
H	-6.60482	3.46095	3.11177
C	-5.89820	1.62896	2.07348
H	-6.73864	1.02171	2.38418
C	-4.93107	1.09827	1.31614
H	-5.00065	0.05577	1.03682
C	-3.77186	1.92700	0.88451
C	-2.67950	1.36200	0.03113
C	-1.82248	-0.91517	-0.28242
C	-1.97051	-2.31052	-0.74170
C	-3.02052	-2.67013	-1.48405
H	-3.11657	-3.69701	-1.81425
C	-4.03149	-1.66607	-1.87731
C	-5.15516	-2.06119	-2.75771
H	-5.25758	-3.08980	-3.08054
C	-6.04660	-1.14921	-3.16004
H	-6.86655	-1.43855	-3.80461
C	-5.90738	0.25761	-2.73837
H	-6.61904	0.99486	-3.08710
C	-4.89883	0.62465	-1.93918
H	-4.80373	1.66619	-1.66359
C	-3.91643	-0.38361	-1.45515
C	-2.78340	-0.00314	-0.55470
H	2.86094	0.82692	0.48486
C	4.44496	2.09830	0.12544
C	4.17075	-0.12078	-0.81300
C	3.94777	3.09161	0.99250
C	5.60250	2.33657	-0.65658
C	5.31633	0.12348	-1.63919
C	4.61856	4.29732	1.08665
H	3.04239	2.89613	1.55773
C	6.26611	3.57694	-0.53068
C	6.00805	1.29720	-1.54783
H	5.63089	-0.65864	-2.31546
C	5.78426	4.54352	0.33116

H	4.23944	5.06663	1.75231
H	7.15620	3.75825	-1.12646
H	6.89183	1.45118	-2.16145
H	6.29636	5.49560	0.42447
C	3.34149	-1.29490	-0.84930
C	2.99799	-1.98154	0.47072
H	2.73049	-1.20562	1.19468
H	3.88245	-2.51296	0.86823
N	1.86092	-2.87272	0.30884
N	3.80060	0.88665	0.02371
C	1.39227	-3.64775	1.36652
C	0.60404	-4.78378	1.09217
C	1.64339	-3.32160	2.71182
C	0.07747	-5.55187	2.12364
H	0.40697	-5.05404	0.05711
C	1.10862	-4.10136	3.73737
H	2.24443	-2.45460	2.96160
C	0.32330	-5.21912	3.45902
H	-0.52713	-6.42183	1.88132
H	1.31424	-3.82600	4.76840
H	-0.08736	-5.82124	4.26331
H	1.86719	-3.35794	-0.57908
H	2.00133	-0.74790	-1.26143
Cl	3.87440	-2.53163	-2.07509
H	-1.22354	-3.04534	-0.47313
H	-0.62517	4.09770	0.05185

Catalyst (H, 4)-Enamine protonation TS (pro-S) alternative conformation
Electronic energy (Hartrees): -1870.612209
Free Energy at 1M concentration and 253 K (Hartrees): -1870.074836
Coordinates:

P	-0.15743	-0.46347	-0.08521
O	-1.16652	0.62843	-0.77504
O	-1.08564	-1.57050	0.67295
O	0.75769	0.14183	0.93599
O	0.49963	-1.12925	-1.32788
C	-2.13545	1.08990	0.07795
C	-2.08097	2.49550	0.52746
C	-3.01907	2.97906	1.34563
H	-2.96764	4.01142	1.66869
C	-4.10645	2.10248	1.83049
C	-5.10334	2.63133	2.78987
H	-5.05885	3.66634	3.10538
C	-6.06103	1.83263	3.27281
H	-6.78815	2.21961	3.97499
C	-6.12129	0.41725	2.86112
H	-6.88486	-0.22927	3.27413
C	-5.22870	-0.06989	1.99129
H	-5.27836	-1.11660	1.72344
C	-4.17601	0.81319	1.41874
C	-3.16915	0.29719	0.43945
C	-2.23115	-1.93641	0.00905
C	-2.36816	-3.33762	-0.43717
C	-3.48265	-3.74938	-1.04640
H	-3.56810	-4.78005	-1.36766
C	-4.58282	-2.79798	-1.30581
C	-5.78410	-3.24924	-2.04567
H	-5.87315	-4.28155	-2.36061
C	-6.76268	-2.38365	-2.33051
H	-7.63940	-2.71392	-2.87257

C	-6.64393	-0.97189	-1.91976
H	-7.42857	-0.27134	-2.17509
C	-5.56539	-0.55469	-1.24652
H	-5.49018	0.49052	-0.97905
C	-4.48171	-1.51213	-0.89128
C	-3.26797	-1.07496	-0.13164
H	2.11007	0.97554	0.20613
C	2.52128	2.86545	-0.48043
C	3.17128	0.78204	-1.55008
C	1.89069	3.49278	0.61129
C	3.02456	3.63127	-1.56017
C	3.65396	1.54862	-2.66362
C	1.78472	4.87221	0.62509
H	1.48707	2.88334	1.41342
C	2.90783	5.03707	-1.50964
C	3.59675	2.91155	-2.65478
H	4.08191	1.01303	-3.49916
C	2.29870	5.65151	-0.43135
H	1.29744	5.36001	1.46376
H	3.29843	5.62566	-2.33495
H	3.99195	3.47263	-3.49756
H	2.20975	6.73239	-0.39784
C	3.10591	-0.64670	-1.47959
C	3.48627	-1.35782	-0.19211
H	3.15940	-2.40702	-0.25962
H	2.91771	-0.91629	0.63099
N	4.91427	-1.24131	0.09743
N	2.64329	1.49462	-0.51494
C	5.46714	-1.86356	1.21910
C	6.86114	-2.06565	1.27467
C	4.69213	-2.26062	2.32441
C	7.45478	-2.63471	2.39503
H	7.47352	-1.76402	0.42782
C	5.30208	-2.82767	3.44424
H	3.61589	-2.12969	2.31644
C	6.68151	-3.02127	3.49386
H	8.53178	-2.77936	2.40890
H	4.68149	-3.12581	4.28510
H	7.14680	-3.46662	4.36752
H	5.48170	-1.35270	-0.73420
H	1.58500	-0.92302	-1.47688
Cl	3.82279	-1.49939	-2.91378
H	-1.55918	-4.03619	-0.26491
H	-1.27603	3.13634	0.18999

Catalyst (H, 4)-Enamine protonation TS (pro-S) alternative conformation
Electronic energy (Hartrees): -1870.6123
Free Energy at 1M concentration and 253 K (Hartrees): -1870.074762
Coordinates:

P	0.40930	-0.54137	-0.27021
O	1.24853	0.32459	0.83710
O	1.49760	-1.22341	-1.27740
O	-0.52375	0.28804	-1.09788
O	-0.22584	-1.65790	0.60998
C	2.20789	1.13456	0.28604
C	2.02322	2.59871	0.34005
C	2.94864	3.41501	-0.17048
H	2.80119	4.48691	-0.12510
C	4.15438	2.85829	-0.82001
C	5.14118	3.77122	-1.44186

H	5.00012	4.84355	-1.38715
C	6.20914	3.27555	-2.07593
H	6.92890	3.94343	-2.53135
C	6.40256	1.81602	-2.16941
H	7.25671	1.42150	-2.70457
C	5.51991	0.98213	-1.60713
H	5.67016	-0.08398	-1.71022
C	4.34302	1.51720	-0.86919
C	3.34379	0.60852	-0.22494
C	2.63625	-1.70323	-0.67738
C	2.90071	-3.15536	-0.72884
C	4.01611	-3.66061	-0.19640
H	4.19597	-4.72755	-0.24208
C	4.98707	-2.77284	0.47598
C	6.18532	-3.35346	1.12495
H	6.36944	-4.41939	1.07304
C	7.04359	-2.56472	1.78015
H	7.91807	-2.99007	2.25542
C	6.79363	-1.11373	1.87006
H	7.47954	-0.48470	2.42276
C	5.71699	-0.57803	1.28315
H	5.54237	0.48442	1.38500
C	4.76879	-1.43648	0.52085
C	3.56088	-0.86316	-0.15251
H	-2.02840	0.59459	-0.28926
C	-2.95378	2.01427	0.87590
C	-3.25734	-0.39513	1.04547
C	-2.34791	3.09203	0.20227
C	-3.76119	2.24186	2.01704
C	-4.04217	-0.17261	2.22840
C	-2.56766	4.38134	0.65649
H	-1.71021	2.89252	-0.65313
C	-3.97050	3.56817	2.44852
C	-4.29388	1.08897	2.67764
H	-4.45128	-1.03682	2.73242
C	-3.38512	4.62686	1.77712
H	-2.10131	5.21430	0.13915
H	-4.59486	3.74290	3.32028
H	-4.91689	1.23404	3.55626
H	-3.54918	5.64505	2.11439
C	-2.88546	-1.66498	0.51427
C	-2.97926	-1.97036	-0.99183
H	-2.28907	-2.78729	-1.21941
H	-2.61743	-1.10832	-1.55648
N	-4.28719	-2.37731	-1.48483
N	-2.75212	0.72343	0.44114
C	-5.37643	-1.50928	-1.58316
C	-6.68140	-2.02758	-1.46636
C	-5.23101	-0.13835	-1.86450
C	-7.79359	-1.20945	-1.63360
H	-6.81354	-3.08435	-1.24563
C	-6.35329	0.67300	-2.02428
H	-4.24351	0.29549	-1.97310
C	-7.64219	0.15121	-1.91149
H	-8.78707	-1.63939	-1.53892
H	-6.21208	1.72804	-2.24318
H	-8.51058	0.79008	-2.03697
H	-4.55044	-3.29110	-1.13845
H	-1.32961	-1.66465	0.64494
Cl	-3.46450	-3.08839	1.47376

H	2.18538	-3.81360	-1.20543
H	1.13225	3.01088	0.79711

Catalyst (H, 4)-Enamine protonation TS (pro-S) alternative conformation
Electronic energy (Hartrees): -1870.611266
Free Energy at 1M concentration and 253 K (Hartrees): -1870.073737
Coordinates:

P	0.22056	-0.16138	0.11998
O	-0.66040	-1.34613	0.82163
O	-0.82946	0.92120	-0.50597
O	1.10791	-0.63045	-0.98942
O	0.91298	0.48349	1.36305
C	-1.68830	-1.80844	0.03967
C	-1.61834	-3.18608	-0.48769
C	-2.61277	-3.67589	-1.23242
H	-2.54878	-4.68769	-1.61309
C	-3.78169	-2.83238	-1.55954
C	-4.84590	-3.36286	-2.44271
H	-4.78747	-4.37782	-2.81582
C	-5.88209	-2.59020	-2.78553
H	-6.65849	-2.97820	-3.43220
C	-5.96326	-1.20124	-2.29515
H	-6.79250	-0.57398	-2.59600
C	-5.00995	-0.71334	-1.49283
H	-5.07874	0.31550	-1.16646
C	-3.86660	-1.57000	-1.07447
C	-2.79009	-1.05315	-0.17252
C	-1.92054	1.20100	0.27964
C	-2.07601	2.57090	0.80850
C	-3.14129	2.89657	1.54450
H	-3.24124	3.90543	1.92528
C	-4.16469	1.87909	1.86143
C	-5.30551	2.23339	2.73721
H	-5.41039	3.24406	3.11180
C	-6.20945	1.30648	3.07151
H	-7.04191	1.56588	3.71279
C	-6.06707	-0.07677	2.57924
H	-6.78899	-0.82764	2.87358
C	-5.04309	-0.40651	1.78344
H	-4.94657	-1.43285	1.45633
C	-4.04601	0.62062	1.37388
C	-2.89533	0.28200	0.47822
H	2.74431	-0.97521	-0.56593
C	3.90367	-2.61732	-0.13464
C	4.03540	-0.44560	0.95419
C	3.31245	-3.41000	-1.13700
C	4.84159	-3.17782	0.76661
C	4.95723	-1.01819	1.89573
C	3.67603	-4.74111	-1.24511
H	2.57352	-2.96744	-1.79721
C	5.19519	-4.53636	0.62387
C	5.35177	-2.31892	1.79028
H	5.34770	-0.37659	2.67278
C	4.62373	-5.30926	-0.37034
H	3.22170	-5.35561	-2.01629
H	5.91868	-4.96471	1.31185
H	6.07370	-2.72153	2.49598
H	4.89813	-6.35377	-0.47544
C	3.52277	0.88678	1.00384
C	3.44212	1.70457	-0.28272

H	3.19080	1.02921	-1.11502
H	4.43256	2.12972	-0.50074
N	2.49374	2.79374	-0.16997
N	3.56560	-1.28710	-0.01440
C	2.29843	3.72080	-1.18336
C	1.26190	4.66939	-1.05623
C	3.11531	3.77922	-2.32841
C	1.05456	5.63318	-2.03418
H	0.63150	4.64570	-0.17128
C	2.90076	4.75984	-3.29811
H	3.91328	3.05915	-2.47038
C	1.87348	5.69203	-3.16671
H	0.24719	6.34984	-1.90856
H	3.54608	4.78323	-4.17240
H	1.71045	6.44787	-3.92839
H	1.69177	2.61045	0.41616
H	2.01083	0.61266	1.29264
Cl	4.15400	1.88959	2.36779
H	-1.32174	3.31630	0.59462
H	-0.75568	-3.80194	-0.26673

Catalyst (H, 4)-Enamine protonation TS (pro-S) alternative conformation
Electronic energy (Hartrees): -1870.612065
Free Energy at 1M concentration and 253 K (Hartrees): -1870.073717
Coordinates:

P	0.26355	-0.77811	-0.46384
O	0.96448	0.03104	0.77414
O	1.44714	-1.20278	-1.50216
O	-0.73844	0.04409	-1.21597
O	-0.25712	-2.06684	0.23752
C	1.82872	1.01379	0.36576
C	1.46735	2.42603	0.60187
C	2.29498	3.40572	0.22981
H	2.01825	4.43739	0.40826
C	3.56992	3.08514	-0.44653
C	4.44867	4.18160	-0.91498
H	4.17702	5.21338	-0.72953
C	5.57993	3.90258	-1.57128
H	6.22069	4.70452	-1.91454
C	5.95155	2.50082	-1.84227
H	6.85671	2.28320	-2.39447
C	5.16752	1.50226	-1.41945
H	5.44846	0.48383	-1.65123
C	3.92145	1.79372	-0.65912
C	3.02946	0.69699	-0.16865
C	2.62480	-1.60992	-0.92399
C	3.07112	-3.00067	-1.14291
C	4.23068	-3.42833	-0.63770
H	4.54420	-4.45099	-0.80707
C	5.06894	-2.51992	0.17145
C	6.31768	-3.02655	0.78631
H	6.63533	-4.04692	0.61105
C	7.05694	-2.22740	1.56273
H	7.96843	-2.59894	2.01291
C	6.62477	-0.84082	1.82049
H	7.21503	-0.20716	2.46987
C	5.50111	-0.37135	1.26594
H	5.19227	0.64036	1.49111
C	4.68354	-1.23658	0.37138
C	3.42656	-0.73515	-0.26906

H	-2.27869	0.19642	-0.19151
C	-3.03033	1.35400	1.32697
C	-3.24965	-1.06470	1.11142
C	-2.55630	2.55734	0.77265
C	-3.67086	1.34657	2.58923
C	-3.87490	-1.07825	2.40794
C	-2.73426	3.73956	1.47106
H	-2.05263	2.53752	-0.18852
C	-3.84296	2.56876	3.27092
C	-4.08526	0.07478	3.10107
H	-4.19356	-2.03258	2.80272
C	-3.38260	3.75229	2.72110
H	-2.36850	4.66914	1.04564
H	-4.33961	2.56422	4.23721
H	-4.58441	0.03983	4.06586
H	-3.51741	4.68939	3.25126
C	-2.91499	-2.21219	0.34484
C	-3.14076	-2.33581	-1.19563
H	-4.00858	-2.98915	-1.34076
H	-2.28548	-2.86591	-1.62579
N	-3.33648	-1.12447	-1.94000
N	-2.85738	0.16465	0.65489
C	-4.53351	-0.42904	-2.02743
C	-4.57055	0.77887	-2.75511
C	-5.72403	-0.88622	-1.43074
C	-5.75671	1.49099	-2.88841
H	-3.65900	1.14198	-3.22378
C	-6.90719	-0.16234	-1.57929
H	-5.72754	-1.79919	-0.84543
C	-6.93968	1.02789	-2.30477
H	-5.75707	2.41642	-3.45819
H	-7.81368	-0.53769	-1.11187
H	-7.86462	1.58522	-2.41296
H	-2.49493	-0.64311	-2.22959
H	-1.34856	-2.13836	0.36472
Cl	-3.32910	-3.79667	1.11267
H	2.45381	-3.67656	-1.72101
H	0.52717	2.66664	1.08173

Catalyst (H, 4)-Enamine protonation TS (pro-S) alternative conformation
Electronic energy (Hartrees): -1870.610442
Free Energy at 1M concentration and 253 K (Hartrees): -1870.073237
Coordinates:

P	-0.55529	0.42799	-0.11496
O	-1.35180	-0.51382	-1.19126
O	-1.61573	0.78867	1.08038
O	-0.06507	1.71924	-0.69010
O	0.49710	-0.54941	0.48464
C	-2.59118	-0.03715	-1.53461
C	-2.81346	0.44859	-2.91139
C	-4.01625	0.89251	-3.28545
H	-4.16583	1.24585	-4.29814
C	-5.13076	0.92739	-2.31508
C	-6.44119	1.48563	-2.72136
H	-6.59408	1.83163	-3.73609
C	-7.44045	1.56742	-1.83645
H	-8.39722	1.97690	-2.13382
C	-7.23776	1.11295	-0.44768
H	-8.04349	1.20403	0.26933
C	-6.05953	0.60248	-0.07114

H	-5.92732	0.29887	0.95849
C	-4.94683	0.47369	-1.05164
C	-3.62126	-0.09998	-0.66048
C	-2.42169	-0.24915	1.47909
C	-2.28043	-0.76371	2.85621
C	-3.07224	-1.74406	3.29775
H	-2.95693	-2.11102	4.31009
C	-4.08218	-2.34429	2.40163
C	-4.90627	-3.47845	2.87973
H	-4.79677	-3.84080	3.89436
C	-5.78708	-4.06183	2.06004
H	-6.38773	-4.89155	2.40976
C	-5.93268	-3.58596	0.67140
H	-6.63004	-4.07859	0.00610
C	-5.19687	-2.55888	0.23066
H	-5.30355	-2.24509	-0.79882
C	-4.23557	-1.87583	1.13965
C	-3.40546	-0.71957	0.67614
H	1.63708	2.02587	-0.55240
C	2.67469	3.39310	0.58773
C	3.29753	1.08394	0.21707
C	1.77715	4.44185	0.30717
C	3.73516	3.57619	1.51116
C	4.35445	1.24325	1.17262
C	1.95925	5.66458	0.92690
H	0.94909	4.26239	-0.37036
C	3.89679	4.84143	2.11802
C	4.56848	2.44802	1.77992
H	5.01872	0.40760	1.34447
C	3.02439	5.87287	1.82824
H	1.26857	6.47546	0.71721
H	4.71356	4.98596	2.81949
H	5.39857	2.56455	2.47173
H	3.15101	6.84252	2.29861
C	2.96454	-0.17238	-0.39430
C	3.84701	-1.36888	-0.14590
H	3.33447	-2.26690	-0.52168
H	3.93182	-1.49822	0.94096
N	5.18858	-1.23014	-0.71479
N	2.52282	2.17054	-0.02448
C	6.09409	-2.29422	-0.68810
C	7.20075	-2.28066	-1.56046
C	5.96935	-3.36577	0.21453
C	8.14791	-3.29711	-1.52382
H	7.30946	-1.45923	-2.26498
C	6.92951	-4.37830	0.24299
H	5.12552	-3.41408	0.89367
C	8.02364	-4.35672	-0.62001
H	8.99004	-3.26235	-2.20967
H	6.81079	-5.19614	0.94865
H	8.76408	-5.14983	-0.59423
H	5.17223	-0.74437	-1.60371
H	1.56074	-0.42619	0.15402
Cl	2.53852	-0.02688	-2.16463
H	-1.53119	-0.33951	3.51255
H	-1.99619	0.44130	-3.62137

Catalyst (H, 4)-Enamine protonation TS (pro-S) alternative conformation
Electronic energy (Hartrees): -1870.610442
Free Energy at 1M concentration and 253 K (Hartrees): -1870.073234

Coordinates:

P	-0.55532	0.42802	-0.11496
O	-1.35185	-0.51374	-1.19127
O	-1.61576	0.78873	1.08037
O	-0.06502	1.71924	-0.69007
O	0.49702	-0.54945	0.48466
C	-2.59123	-0.03705	-1.53461
C	-2.81352	0.44869	-2.91139
C	-4.01632	0.89259	-3.28546
H	-4.16590	1.24593	-4.29814
C	-5.13084	0.92745	-2.31509
C	-6.44130	1.48564	-2.72139
H	-6.59419	1.83163	-3.73612
C	-7.44057	1.56739	-1.83649
H	-8.39736	1.97684	-2.13387
C	-7.23788	1.11294	-0.44770
H	-8.04363	1.20399	0.26930
C	-6.05963	0.60251	-0.07116
H	-5.92743	0.29892	0.95849
C	-4.94692	0.47376	-1.05164
C	-3.62133	-0.09990	-0.66048
C	-2.42174	-0.24907	1.47909
C	-2.28051	-0.76361	2.85623
C	-3.07234	-1.74394	3.29778
H	-2.95703	-2.11088	4.31013
C	-4.08229	-2.34417	2.40166
C	-4.90641	-3.47831	2.87977
H	-4.79692	-3.84064	3.89442
C	-5.78723	-4.06169	2.06008
H	-6.38789	-4.89140	2.40982
C	-5.93280	-3.58585	0.67143
H	-6.63016	-4.07848	0.00613
C	-5.19696	-2.55879	0.23067
H	-5.30363	-2.24503	-0.79881
C	-4.23566	-1.87574	1.13967
C	-3.40553	-0.71949	0.67615
H	1.63708	2.02579	-0.55220
C	2.67489	3.39303	0.58777
C	3.29756	1.08382	0.21717
C	1.77744	4.44185	0.30715
C	3.73540	3.57608	1.51114
C	4.35450	1.24308	1.17271
C	1.95970	5.66461	0.92678
H	0.94936	4.26244	-0.37035
C	3.89719	4.84135	2.11790
C	4.56863	2.44786	1.77995
H	5.01869	0.40737	1.34459
C	3.02488	5.87286	1.82808
H	1.26911	6.47556	0.71703
H	4.71400	4.98585	2.81933
H	5.39874	2.56434	2.47175
H	3.15163	6.84254	2.29836
C	2.96454	-0.17248	-0.39422
C	3.84699	-1.36898	-0.14583
H	3.33444	-2.26702	-0.52158
H	3.93185	-1.49832	0.94103
N	5.18855	-1.23022	-0.71475
N	2.52287	2.17044	-0.02436
C	6.09405	-2.29428	-0.68811
C	7.20072	-2.28068	-1.56047

C	5.96930	-3.36589	0.21445
C	8.14786	-3.29715	-1.52391
H	7.30946	-1.45920	-2.26492
C	6.92943	-4.37844	0.24283
H	5.12548	-3.41423	0.89360
C	8.02357	-4.35683	-0.62018
H	8.98999	-3.26235	-2.20974
H	6.81071	-5.19634	0.94844
H	8.76399	-5.14996	-0.59445
H	5.17224	-0.74436	-1.60363
H	1.56064	-0.42625	0.15405
Cl	2.53853	-0.02693	-2.16455
H	-1.53126	-0.33941	3.51256
H	-1.99625	0.44142	-3.62137

Catalyst (H, 4)-Enamine protonation TS (pro-S) alternative conformation
Electronic energy (Hartrees): -1870.608052
Free Energy at 1M concentration and 253 K (Hartrees): -1870.072317
Coordinates:

P	0.24793	-0.28733	-0.27610
O	1.26329	0.53692	0.71261
O	1.15204	-1.35492	-1.11718
O	-0.44983	0.59554	-1.26842
O	-0.64045	-1.06378	0.73274
C	2.38074	1.01026	0.07486
C	2.53244	2.46702	-0.11356
C	3.61750	2.96005	-0.71593
H	3.71671	4.03022	-0.84877
C	4.66456	2.04845	-1.22435
C	5.83141	2.60226	-1.94934
H	5.93697	3.67293	-2.07292
C	6.75829	1.78275	-2.45685
H	7.60984	2.18831	-2.98779
C	6.61604	0.32201	-2.30727
H	7.35782	-0.33619	-2.74103
C	5.56840	-0.18901	-1.65007
H	5.47319	-1.26374	-1.57466
C	4.54446	0.70980	-1.05049
C	3.36642	0.16648	-0.30480
C	2.15207	-1.97071	-0.40482
C	2.08021	-3.43313	-0.21146
C	3.05243	-4.08003	0.43594
H	2.98548	-5.15261	0.56965
C	4.20023	-3.33358	0.99113
C	5.23616	-4.05223	1.76858
H	5.17349	-5.12580	1.89620
C	6.25181	-3.37854	2.31867
H	7.00747	-3.90430	2.88794
C	6.33781	-1.91412	2.16393
H	7.14923	-1.37217	2.63243
C	5.41019	-1.25559	1.45938
H	5.48143	-0.17921	1.38194
C	4.29097	-1.99379	0.81164
C	3.24396	-1.28400	0.01093
H	-1.78800	1.42062	-0.57032
C	-2.17055	3.22758	0.32875
C	-3.12011	1.08245	0.97479
C	-1.34262	3.94097	-0.55916
C	-2.76881	3.87746	1.43599
C	-3.70190	1.72818	2.11740

C	-1.13674	5.29348	-0.34873
H	-0.86919	3.41474	-1.38182
C	-2.54546	5.25913	1.61565
C	-3.54490	3.06726	2.32388
H	-4.28496	1.12148	2.79587
C	-1.74280	5.96075	0.73446
H	-0.49710	5.84655	-1.02973
H	-3.00866	5.75940	2.46146
H	-4.01607	3.53936	3.18199
H	-1.57280	7.02271	0.87834
C	-3.17011	-0.31956	0.69098
C	-3.43760	-0.80311	-0.74682
H	-3.14631	-1.85119	-0.82008
H	-2.75851	-0.26347	-1.41498
N	-4.79243	-0.65354	-1.23563
N	-2.39683	1.88233	0.13413
C	-5.80082	-1.61214	-1.22119
C	-7.12196	-1.20211	-1.49354
C	-5.56560	-2.98300	-1.00483
C	-8.16086	-2.12455	-1.55348
H	-7.32435	-0.14669	-1.66416
C	-6.61639	-3.89581	-1.06684
H	-4.56895	-3.34125	-0.77531
C	-7.92095	-3.48378	-1.34169
H	-9.16749	-1.77448	-1.76620
H	-6.40558	-4.94752	-0.89223
H	-8.73188	-4.20361	-1.38624
H	-5.12404	0.29942	-1.27704
H	-1.72614	-0.73739	0.79536
Cl	-4.08802	-1.29744	1.89809
H	1.23378	-3.98411	-0.60145
H	1.75903	3.13754	0.23976

Catalyst (H, 4)-Enamine protonation TS (pro-S) alternative conformation
Electronic energy (Hartrees): -1870.608838
Free Energy at 1M concentration and 253 K (Hartrees): -1870.071832
Coordinates:

P	-0.35585	-0.66214	0.35352
O	-1.34567	-0.04758	1.50310
O	-1.17481	-0.60152	-1.06411
O	0.04675	-2.08096	0.59074
O	0.77448	0.41125	0.27488
C	-2.62352	-0.54408	1.45865
C	-3.09361	-1.40620	2.56173
C	-4.34158	-1.88190	2.56297
H	-4.67363	-2.51294	3.37802
C	-5.25671	-1.57159	1.44438
C	-6.61563	-2.16052	1.42211
H	-6.95131	-2.78511	2.24064
C	-7.43305	-1.92591	0.39008
H	-8.42533	-2.35792	0.37394
C	-6.97994	-1.08891	-0.73708
H	-7.63826	-0.92259	-1.58005
C	-5.75610	-0.54790	-0.72501
H	-5.43771	0.04182	-1.57394
C	-4.84523	-0.77181	0.43096
C	-3.47438	-0.17341	0.47475
C	-1.90052	0.54590	-1.26975
C	-1.50831	1.44032	-2.37763
C	-2.21031	2.54633	-2.63631

H	-1.91054	3.19267	-3.45191
C	-3.37364	2.90152	-1.79731
C	-4.10176	4.16650	-2.04971
H	-3.80749	4.80919	-2.87017
C	-5.12380	4.52205	-1.26415
H	-5.65428	5.44735	-1.44836
C	-5.52248	3.66134	-0.13443
H	-6.33491	3.96727	0.51210
C	-4.87484	2.51400	0.09958
H	-5.16944	1.91304	0.94913
C	-3.75639	2.08609	-0.78524
C	-3.01921	0.80430	-0.55184
H	1.79086	-2.45317	0.65162
C	2.96671	-3.43836	-0.72201
C	3.49555	-1.34491	0.37618
C	2.07055	-4.51581	-0.85911
C	4.12157	-3.34981	-1.53947
C	4.63499	-1.21147	-0.48521
C	2.34641	-5.50520	-1.78546
H	1.17172	-4.53797	-0.25197
C	4.37670	-4.38242	-2.46840
C	4.94211	-2.18979	-1.38709
H	5.23956	-0.31983	-0.39560
C	3.50502	-5.44825	-2.58760
H	1.65755	-6.33679	-1.89719
H	5.26483	-4.31989	-3.09084
H	5.82972	-2.09353	-2.00686
H	3.70484	-6.23873	-3.30365
C	3.09135	-0.34161	1.31575
C	4.00090	0.85062	1.57682
H	5.02759	0.53295	1.81302
H	3.62500	1.38050	2.46333
N	4.03738	1.70878	0.39696
N	2.72616	-2.45062	0.20492
C	4.78052	2.89198	0.37962
C	4.54911	3.82786	-0.64869
C	5.78152	3.17391	1.32645
C	5.29635	4.99667	-0.72748
H	3.77705	3.62251	-1.38656
C	6.52969	4.34858	1.23052
H	5.97519	2.48599	2.14149
C	6.29809	5.26840	0.20980
H	5.09446	5.70227	-1.52879
H	7.29741	4.54474	1.97417
H	6.88082	6.18176	0.14572
H	3.13190	1.79146	-0.05022
H	1.76457	0.11767	0.70359
Cl	2.44524	-1.01178	2.87169
H	-0.64677	1.19452	-2.98546
H	-2.42219	-1.65064	3.37507

Catalyst (H, 4)-Enamine protonation TS (pro-S) alternative conformation
Electronic energy (Hartrees): -1870.608838
Free Energy at 1M concentration and 253 K (Hartrees): -1870.071832
Coordinates:

P	0.35585	-0.66214	-0.35352
O	1.34567	-0.04758	-1.50310
O	1.17481	-0.60152	1.06411
O	-0.04675	-2.08096	-0.59074
O	-0.77448	0.41125	-0.27488

C	2.62352	-0.54408	-1.45865
C	3.09361	-1.40620	-2.56173
C	4.34158	-1.88190	-2.56297
H	4.67363	-2.51294	-3.37802
C	5.25671	-1.57159	-1.44438
C	6.61563	-2.16052	-1.42211
H	6.95131	-2.78511	-2.24064
C	7.43305	-1.92591	-0.39008
H	8.42533	-2.35792	-0.37394
C	6.97994	-1.08891	0.73708
H	7.63826	-0.92259	1.58005
C	5.75610	-0.54790	0.72501
H	5.43771	0.04182	1.57394
C	4.84523	-0.77181	-0.43096
C	3.47438	-0.17341	-0.47475
C	1.90052	0.54590	1.26975
C	1.50831	1.44032	2.37763
C	2.21031	2.54633	2.63631
H	1.91054	3.19267	3.45191
C	3.37364	2.90152	1.79731
C	4.10176	4.16650	2.04971
H	3.80749	4.80919	2.87017
C	5.12380	4.52205	1.26415
H	5.65428	5.44735	1.44836
C	5.52248	3.66134	0.13443
H	6.33491	3.96727	-0.51210
C	4.87484	2.51400	-0.09958
H	5.16944	1.91304	-0.94913
C	3.75639	2.08609	0.78524
C	3.01921	0.80430	0.55184
H	-1.79086	-2.45317	-0.65162
C	-2.96671	-3.43836	0.72201
C	-3.49555	-1.34491	-0.37618
C	-2.07055	-4.51581	0.85911
C	-4.12157	-3.34981	1.53947
C	-4.63499	-1.21147	0.48521
C	-2.34641	-5.50520	1.78546
H	-1.17172	-4.53797	0.25197
C	-4.37670	-4.38242	2.46840
C	-4.94211	-2.18979	1.38709
H	-5.23956	-0.31983	0.39560
C	-3.50502	-5.44825	2.58760
H	-1.65755	-6.33679	1.89719
H	-5.26483	-4.31989	3.09084
H	-5.82972	-2.09353	2.00686
H	-3.70484	-6.23873	3.30365
C	-3.09135	-0.34161	-1.31575
C	-4.00090	0.85062	-1.57682
H	-5.02759	0.53295	-1.81302
H	-3.62500	1.38050	-2.46333
N	-4.03738	1.70878	-0.39696
N	-2.72616	-2.45062	-0.20492
C	-4.78052	2.89198	-0.37962
C	-4.54911	3.82786	0.64869
C	-5.78152	3.17391	-1.32645
C	-5.29635	4.99667	0.72748
H	-3.77705	3.62251	1.38656
C	-6.52969	4.34858	-1.23052
H	-5.97519	2.48599	-2.14149
C	-6.29809	5.26840	-0.20980

H	-5.09446	5.70227	1.52879
H	-7.29741	4.54474	-1.97417
H	-6.88082	6.18176	-0.14572
H	-3.13190	1.79146	0.05022
H	-1.76457	0.11767	-0.70359
Cl	-2.44524	-1.01178	-2.87169
H	0.64677	1.19452	2.98546
H	2.42219	-1.65064	-3.37507

Catalyst (H, 4)-Enamine protonation TS (pro-S) alternative conformation
Electronic energy (Hartrees): -1870.60653
Free Energy at 1M concentration and 253 K (Hartrees): -1870.070243
Coordinates:

P	0.11510	-0.70536	-0.18447
O	1.02664	0.12078	-1.26424
O	1.07292	-1.02222	1.10621
O	-0.40563	-2.01001	-0.69489
O	-0.92551	0.36789	0.26156
C	2.26811	-0.42247	-1.47645
C	2.57209	-1.01488	-2.79461
C	3.78107	-1.52463	-3.04399
H	3.99082	-1.95593	-4.01494
C	4.81927	-1.52507	-1.99184
C	6.13359	-2.15446	-2.25714
H	6.34699	-2.57887	-3.23033
C	7.06153	-2.20387	-1.29550
H	8.02113	-2.66533	-1.48979
C	6.77529	-1.64219	0.03826
H	7.52234	-1.70675	0.81888
C	5.59306	-1.06698	0.28736
H	5.39763	-0.68432	1.27993
C	4.56082	-0.97408	-0.78124
C	3.23315	-0.32980	-0.53326
C	1.88763	0.01298	1.49369
C	1.66411	0.63131	2.81611
C	2.45690	1.61600	3.24610
H	2.28071	2.06000	4.21801
C	3.55186	2.11648	2.38933
C	4.37978	3.25618	2.84719
H	4.20909	3.69552	3.82219
C	5.33967	3.74960	2.05775
H	5.94310	4.58347	2.39248
C	5.56980	3.16840	0.72162
H	6.33219	3.58843	0.07823
C	4.83108	2.13538	0.30004
H	5.00190	1.74334	-0.69334
C	3.78057	1.55191	1.17912
C	2.94419	0.39205	0.73653
H	-2.15471	-2.25206	-0.69626
C	-3.30712	-3.52946	0.43371
C	-3.78355	-1.19650	-0.02303
C	-2.46116	-4.63403	0.21409
C	-4.40782	-3.62815	1.32202
C	-4.87307	-1.27199	0.90699
C	-2.73527	-5.82707	0.85735
H	-1.60025	-4.52036	-0.43607
C	-4.66382	-4.86470	1.95471
C	-5.17849	-2.44574	1.53573
H	-5.47941	-0.39215	1.07231
C	-3.84236	-5.95114	1.72308

H	-2.08497	-6.68088	0.69426
H	-5.51157	-4.94335	2.62941
H	-6.03201	-2.49250	2.20687
H	-4.04086	-6.89917	2.21216
C	-3.35838	0.00130	-0.68592
C	-4.16348	1.27620	-0.54163
H	-4.52355	1.33775	0.49994
H	-5.05300	1.26925	-1.18982
N	-3.34587	2.42855	-0.86842
H	-2.35239	2.29104	-0.74044
C	-3.79762	3.72812	-0.70169
C	-2.87746	4.79508	-0.78118
C	-5.15426	4.03483	-0.48085
C	-3.30216	6.11093	-0.65018
H	-1.82660	4.57451	-0.95246
C	-5.56599	5.36258	-0.35711
H	-5.89004	3.24144	-0.41087
C	-4.65190	6.41127	-0.43798
H	-2.57053	6.91195	-0.71483
H	-6.61931	5.57249	-0.19028
H	-4.97989	7.44093	-0.33718
H	-1.99140	0.20399	-0.07992
N	-3.06557	-2.33577	-0.20411
Cl	-2.85120	-0.26892	-2.41080
H	0.85296	0.28063	3.44152
H	1.81009	-1.03244	-3.56345

Catalyst (H, 4)-Enamine protonation TS (pro-S) alternative conformation
Electronic energy (Hartrees): -1870.6062
Free Energy at 1M concentration and 253 K (Hartrees): -1870.070016
Coordinates:

P	0.16892	-0.71492	-0.07534
O	0.99855	0.22783	-1.12813
O	1.20325	-1.10558	1.13282
O	-0.32306	-1.99445	-0.67861
O	-0.88216	0.26555	0.51337
C	2.24063	-0.25717	-1.44925
C	2.48854	-0.72906	-2.82642
C	3.69537	-1.17892	-3.17973
H	3.86379	-1.52193	-4.19301
C	4.78845	-1.23418	-2.18621
C	6.10276	-1.79862	-2.57084
H	6.27453	-2.13443	-3.58598
C	7.08219	-1.89859	-1.66585
H	8.04179	-2.31257	-1.94744
C	6.85351	-1.45807	-0.27661
H	7.64302	-1.56376	0.45629
C	5.67162	-0.94218	0.08056
H	5.51992	-0.64899	1.11053
C	4.58116	-0.79330	-0.92185
C	3.25219	-0.21306	-0.55250
C	2.00883	-0.08000	1.56260
C	1.84107	0.41977	2.94219
C	2.62989	1.38935	3.41202
H	2.49499	1.74579	4.42570
C	3.66343	1.99230	2.54501
C	4.48477	3.11504	3.05382
H	4.35560	3.46681	4.06987
C	5.38737	3.70119	2.26020
H	5.98598	4.52261	2.63234

C	5.56008	3.23980	0.86977
H	6.27533	3.73475	0.22551
C	4.82695	2.22306	0.40144
H	4.95404	1.91993	-0.62892
C	3.84113	1.53697	1.28139
C	3.01292	0.39242	0.78661
H	-2.02546	-2.21940	-0.64839
C	-3.18245	-3.60189	0.34699
C	-3.70977	-1.25736	0.05110
C	-2.29834	-4.66425	0.07248
C	-4.30910	-3.79751	1.18582
C	-4.82694	-1.43033	0.93231
C	-2.56044	-5.90993	0.61156
H	-1.42009	-4.47691	-0.53605
C	-4.55087	-5.08620	1.71225
C	-5.11965	-2.65629	1.46089
H	-5.46425	-0.58215	1.14105
C	-3.69202	-6.12933	1.42612
H	-1.88163	-6.73185	0.40621
H	-5.41777	-5.23910	2.34872
H	-5.99325	-2.77572	2.09627
H	-3.87956	-7.11748	1.83329
C	-3.30063	0.00370	-0.50277
C	-4.18314	1.21971	-0.31129
H	-4.52270	1.22277	0.73348
H	-5.08775	1.17514	-0.94639
N	-3.42907	2.43611	-0.54844
N	-2.95255	-2.35732	-0.18887
C	-4.04032	3.68045	-0.59262
C	-5.33381	3.90242	-0.08276
C	-3.33836	4.77864	-1.13240
C	-5.89380	5.18041	-0.11337
H	-5.90288	3.08217	0.34092
C	-3.90976	6.04434	-1.15746
H	-2.33744	4.62327	-1.52797
C	-5.19453	6.26067	-0.64814
H	-6.89400	5.32588	0.28634
H	-3.34622	6.87136	-1.58103
H	-5.63812	7.25097	-0.66987
H	-2.63982	2.32273	-1.16907
H	-1.95004	0.19825	0.12916
Cl	-2.76576	-0.12846	-2.24703
H	1.68685	-0.70661	-3.55367
H	1.07455	-0.00626	3.57704

Catalyst (H, 4)-Enamine protonation TS (pro-S) alternative conformation
Electronic energy (Hartrees): -1870.604907
Free Energy at 1M concentration and 253 K (Hartrees): -1870.067442
Coordinates:

P	0.42233	-0.95892	0.01104
O	-0.64398	-0.82000	1.25431
O	-0.45904	-1.07042	-1.36366
O	1.31324	-2.15802	0.12590
O	1.10246	0.43262	-0.03481
C	-1.79133	-1.56342	1.13648
C	-2.05023	-2.62276	2.13233
C	-3.18400	-3.32728	2.09330
H	-3.35959	-4.10107	2.83035
C	-4.18417	-3.06849	1.03676
C	-5.40859	-3.89901	0.96539

H	-5.58696	-4.66988	1.70483
C	-6.29807	-3.70235	-0.01357
H	-7.19185	-4.31060	-0.06571
C	-6.05767	-2.66431	-1.03384
H	-6.77103	-2.52546	-1.83600
C	-4.95706	-1.90537	-0.97636
H	-4.79194	-1.16670	-1.74897
C	-3.97135	-2.09019	0.12403
C	-2.73639	-1.25029	0.21787
C	-1.40357	-0.07991	-1.47161
C	-1.20631	0.99526	-2.46499
C	-2.09123	1.99005	-2.56744
H	-1.93143	2.77662	-3.29440
C	-3.26429	2.03684	-1.66920
C	-4.19712	3.18493	-1.73991
H	-4.04467	3.96501	-2.47541
C	-5.22554	3.26857	-0.88946
H	-5.90381	4.11061	-0.93855
C	-5.43163	2.21996	0.12757
H	-6.25378	2.30505	0.82645
C	-4.59999	1.17381	0.19474
H	-4.75794	0.42972	0.96363
C	-3.46604	1.05128	-0.76135
C	-2.51438	-0.10121	-0.70180
H	2.95471	-1.54517	0.52755
C	4.65030	-1.72833	-0.61084
C	4.06027	0.19275	0.74087
C	4.31415	-3.02832	-1.03848
C	5.79672	-1.07029	-1.12593
C	5.18848	0.88309	0.19377
C	5.13626	-3.66553	-1.94888
H	3.40366	-3.48595	-0.66664
C	6.61884	-1.75651	-2.04819
C	6.03306	0.26557	-0.68643
H	5.39630	1.89006	0.53079
C	6.29683	-3.03756	-2.45055
H	4.88095	-4.66526	-2.28630
H	7.50133	-1.25684	-2.43748
H	6.90964	0.79091	-1.05649
H	6.92899	-3.56139	-3.16008
C	3.08651	0.80381	1.60630
C	3.29565	2.24027	2.10387
H	4.33095	2.41879	2.42418
H	2.68808	2.37115	3.00128
N	2.95035	3.28652	1.15566
N	3.85723	-1.07702	0.30437
C	1.63597	3.58045	0.77700
C	1.41992	4.38425	-0.35895
C	0.51608	3.14551	1.50734
C	0.13326	4.74250	-0.74596
H	2.27514	4.72821	-0.93716
C	-0.76871	3.51244	1.10897
H	0.63512	2.49625	2.36669
C	-0.97592	4.31349	-0.01317
H	-0.00239	5.36157	-1.62870
H	-1.61771	3.15280	1.68402
H	-1.97951	4.59596	-0.31446
H	3.62426	3.43962	0.41934
H	1.94338	0.68642	0.72260
Cl	2.55824	-0.29894	2.95960

H	-0.33574	0.97887	-3.10823
H	-1.31606	-2.82577	2.90171

Catalyst (H, 4)-Enamine protonation TS (pro-S) alternative conformation
Electronic energy (Hartrees): -1870.604909
Free Energy at 1M concentration and 253 K (Hartrees): -1870.06743
Coordinates:

P	0.42390	-0.95759	0.00572
O	-0.64081	-0.82009	1.25050
O	-0.45910	-1.06808	-1.36796
O	1.31538	-2.15647	0.11850
O	1.10378	0.43416	-0.03948
C	-1.78774	-1.56429	1.13375
C	-2.04441	-2.62443	2.12919
C	-3.17764	-3.32981	2.09124
H	-3.35164	-4.10417	2.82804
C	-4.17940	-3.07122	1.03618
C	-5.40324	-3.90266	0.96594
H	-5.58002	-4.67405	1.70518
C	-6.29413	-3.70618	-0.01173
H	-7.18749	-4.31510	-0.06305
C	-6.05587	-2.66744	-1.03173
H	-6.77037	-2.52875	-1.83289
C	-4.95580	-1.90767	-0.97526
H	-4.79225	-1.16851	-1.74769
C	-3.96855	-2.09224	0.12376
C	-2.73417	-1.25136	0.21653
C	-1.40465	-0.07829	-1.47371
C	-1.21008	0.99747	-2.46692
C	-2.09652	1.99101	-2.56797
H	-1.93876	2.77800	-3.29490
C	-3.26845	2.03593	-1.66822
C	-4.20301	3.18267	-1.73743
H	-4.05270	3.96307	-2.47300
C	-5.23033	3.26469	-0.88554
H	-5.90985	4.10575	-0.93354
C	-5.43347	2.21567	0.13159
H	-6.25471	2.29949	0.83166
C	-4.60029	1.17070	0.19739
H	-4.75607	0.42629	0.96638
C	-3.46760	1.04991	-0.76034
C	-2.51443	-0.10137	-0.70252
H	2.95626	-1.54386	0.52439
C	4.65447	-1.72372	-0.61063
C	4.05988	0.19455	0.74310
C	4.32052	-3.02337	-1.04100
C	5.80124	-1.06370	-1.12235
C	5.18860	0.88683	0.19954
C	5.14508	-3.65828	-1.95081
H	3.40975	-3.48254	-0.67172
C	6.62588	-1.74758	-2.04409
C	6.03548	0.27163	-0.68008
H	5.39504	1.89325	0.53903
C	6.30599	-3.02832	-2.44916
H	4.89147	-4.65772	-2.29034
H	7.50866	-1.24640	-2.43078
H	6.91235	0.79837	-1.04744
H	6.94010	-3.55036	-3.15826
C	3.08368	0.80321	1.60737
C	3.28930	2.23925	2.10731

H	4.32333	2.41950	2.43076
H	2.67892	2.36780	3.00314
N	2.94416	3.28599	1.15962
N	3.85897	-1.07473	0.30412
C	1.62992	3.57851	0.77935
C	1.41454	4.38402	-0.35551
C	0.50943	3.14026	1.50684
C	0.12802	4.74128	-0.74386
H	2.27019	4.73019	-0.93175
C	-0.77521	3.50616	1.10710
H	0.62789	2.48896	2.36473
C	-0.98172	4.30936	-0.01363
H	-0.00710	5.36183	-1.62564
H	-1.62466	3.14406	1.67995
H	-1.98520	4.59115	-0.31592
H	3.61892	3.44109	0.42449
H	1.94244	0.68686	0.72074
Cl	2.55403	-0.30220	2.95799
H	-0.34036	0.98246	-3.11130
H	-1.30905	-2.82732	2.89745

Catalyst (H, 4)-Enamine protonation TS (pro-S) alternative conformation
Electronic energy (Hartrees): -1870.599133
Free Energy at 1M concentration and 253 K (Hartrees): -1870.063597
Coordinates:

P	0.32424	0.76302	-0.35828
O	1.18643	0.59849	1.04202
O	1.31005	0.22902	-1.56901
O	-0.03432	2.17802	-0.67093
O	-0.81573	-0.25483	-0.14904
C	2.47545	1.05876	0.95479
C	2.85799	2.26106	1.72222
C	4.11420	2.71334	1.68807
H	4.38143	3.59611	2.25558
C	5.12884	2.02851	0.85947
C	6.50188	2.57787	0.77636
H	6.77207	3.45603	1.34960
C	7.41320	1.99366	-0.00879
H	8.41587	2.39668	-0.07004
C	7.05108	0.80585	-0.80524
H	7.78713	0.35204	-1.45632
C	5.81495	0.29837	-0.73411
H	5.56645	-0.55836	-1.34571
C	4.79744	0.91679	0.15932
C	3.40997	0.36655	0.26437
C	2.00023	-0.92080	-1.27793
C	1.68278	-2.14945	-2.03332
C	2.35986	-3.27774	-1.80570
H	2.11613	-4.17132	-2.36686
C	3.41822	-3.31458	-0.77483
C	4.11525	-4.58862	-0.48340
H	3.87762	-5.48061	-1.04966
C	5.03895	-4.64390	0.48193
H	5.54665	-5.57569	0.69526
C	5.35898	-3.43819	1.26932
H	6.09056	-3.49594	2.06497
C	4.73891	-2.28084	1.01117
H	4.97284	-1.41706	1.61851
C	3.73165	-2.19338	-0.08164
C	3.02750	-0.91098	-0.39697

H	-4.45242	-1.70995	-1.49617
C	-4.71662	-3.07778	0.04652
C	-3.81993	-0.82676	0.19218
C	-5.24326	-4.09734	-0.76383
C	-4.56086	-3.26403	1.44217
C	-3.63963	-1.01139	1.59842
C	-5.61377	-5.29663	-0.18009
H	-5.35480	-3.93795	-1.83220
C	-4.94703	-4.50027	2.00466
C	-4.00393	-2.18421	2.19518
H	-3.17486	-0.21791	2.16702
C	-5.46660	-5.50176	1.20614
H	-6.02200	-6.08788	-0.80052
H	-4.82725	-4.64989	3.07345
H	-3.85478	-2.31544	3.26295
H	-5.76178	-6.44929	1.64417
C	-3.31741	0.34528	-0.49937
C	-3.66529	1.71059	0.10508
H	-3.58685	1.62250	1.20525
H	-4.72041	1.92732	-0.10794
N	-2.83818	2.75439	-0.43113
N	-4.34508	-1.86244	-0.49425
C	-3.07430	4.09018	-0.13156
C	-2.06823	5.03151	-0.44442
C	-4.26942	4.55957	0.44648
C	-2.26715	6.38411	-0.20471
H	-1.13474	4.67035	-0.86564
C	-4.45528	5.92515	0.67427
H	-5.05872	3.86833	0.72274
C	-3.46451	6.84946	0.35253
H	-1.47565	7.08634	-0.45372
H	-5.38930	6.26194	1.11740
H	-3.61478	7.90884	0.53637
H	-1.84615	2.52033	-0.54925
H	-2.04341	0.12557	-0.39352
Cl	-3.68461	0.31232	-2.28156
H	0.89733	-2.13300	-2.77824
H	2.11519	2.77603	2.31813

Catalyst (H, 4)-Enamine protonation TS (pro-S) alternative conformation
Electronic energy (Hartrees): -1870.597556
Free Energy at 1M concentration and 253 K (Hartrees): -1870.06068
Coordinates:

P	0.26075	-0.03767	-1.80817
O	0.87997	0.56044	-0.36448
O	1.30087	-1.23260	-2.26245
O	0.21412	1.00235	-2.86472
O	-1.04387	-0.68594	-1.29550
C	2.21697	0.84861	-0.42686
C	2.65338	2.25830	-0.38263
C	3.95373	2.56201	-0.40521
H	4.26218	3.59951	-0.37109
C	4.96635	1.48889	-0.50467
C	6.40254	1.83960	-0.59476
H	6.71314	2.87612	-0.55121
C	7.32183	0.87814	-0.73274
H	8.37101	1.13546	-0.79986
C	6.90810	-0.53609	-0.80754
H	7.65581	-1.30719	-0.94149
C	5.61369	-0.86466	-0.72274

H	5.33224	-1.90598	-0.80288
C	4.58034	0.19025	-0.53784
C	3.12744	-0.14850	-0.43142
C	1.76324	-2.02721	-1.24266
C	1.37603	-3.45238	-1.22920
C	1.84379	-4.27307	-0.28555
H	1.55080	-5.31563	-0.28900
C	2.73468	-3.75650	0.77389
C	3.18873	-4.66031	1.85602
H	2.90214	-5.70462	1.84870
C	3.95187	-4.19041	2.84837
H	4.28174	-4.85119	3.63962
C	4.33443	-2.76609	2.87610
H	4.93147	-2.38712	3.69557
C	3.93430	-1.94201	1.90075
H	4.20609	-0.89683	1.95789
C	3.11254	-2.45544	0.77009
C	2.65631	-1.55808	-0.33790
H	-5.08406	-0.37980	-1.80150
C	-5.86649	-1.22374	-0.07662
C	-3.99857	0.31786	-0.25601
C	-6.85387	-1.97788	-0.73342
C	-5.74587	-1.25440	1.33475
C	-3.87045	0.29985	1.16711
C	-7.71291	-2.76105	0.01760
H	-6.93118	-1.94411	-1.81612
C	-6.64003	-2.06334	2.07029
C	-4.72069	-0.45396	1.92631
H	-3.07482	0.89792	1.59485
C	-7.60852	-2.80634	1.42238
H	-8.47549	-3.34790	-0.48420
H	-6.55162	-2.09116	3.15221
H	-4.62094	-0.45242	3.00814
H	-8.29171	-3.42660	1.99285
C	-3.04260	0.98047	-1.12616
C	-2.41432	2.31830	-0.68475
H	-3.20487	3.03587	-0.44087
H	-1.85817	2.70418	-1.54908
N	-1.55760	2.12865	0.48011
N	-4.99338	-0.42646	-0.78847
C	-1.01517	3.23622	1.15361
C	0.06056	3.00748	2.03572
C	-1.50691	4.54566	1.02676
C	0.61766	4.05125	2.76355
H	0.45671	1.99994	2.12728
C	-0.94444	5.58421	1.77235
H	-2.31518	4.76846	0.33953
C	0.11711	5.35162	2.64378
H	1.45167	3.84783	3.42991
H	-1.34018	6.58944	1.65485
H	0.55328	6.16618	3.21333
H	-0.84052	1.42555	0.31232
H	-2.07896	0.14396	-1.23948
Cl	-3.67403	1.15166	-2.82492
H	0.71086	-3.83409	-1.99334
H	1.91505	3.04822	-0.32938

Catalyst (H, 4)-Enamine protonation TS (pro-R) lowest free energy conformation
Electronic energy (Hartrees): -1870.614699
Free Energy at 1M concentration and 253 K (Hartrees): -1870.077307

Coordinates:

P	-0.01004	-0.96174	-1.17581
O	1.35609	-1.41517	-1.95809
O	0.46593	-0.17167	0.19775
O	-0.87479	-2.09461	-0.72725
O	-0.60226	0.07594	-2.15805
C	2.32672	-1.94206	-1.14383
C	2.68310	-3.36725	-1.29564
C	3.65172	-3.90593	-0.55073
H	3.90246	-4.95233	-0.67300
C	4.35988	-3.08589	0.45420
C	5.38387	-3.71139	1.32265
H	5.64034	-4.75585	1.19583
C	5.99423	-2.99015	2.26890
H	6.74123	-3.44846	2.90408
C	5.64101	-1.57072	2.46000
H	6.12167	-0.99936	3.24366
C	4.72101	-0.99429	1.67777
H	4.46127	0.04068	1.85401
C	4.05552	-1.77346	0.59764
C	3.02853	-1.15279	-0.29691
C	1.48265	0.72825	0.00497
C	1.20268	2.16549	0.19204
C	2.17413	3.06903	0.04301
H	1.95409	4.12008	0.18331
C	3.53648	2.63903	-0.33635
C	4.59014	3.65454	-0.56498
H	4.37355	4.70551	-0.41902
C	5.81270	3.27849	-0.95511
H	6.58165	4.02139	-1.12338
C	6.11376	1.85052	-1.17111
H	7.09871	1.55536	-1.50926
C	5.17073	0.92425	-0.96292
H	5.40820	-0.11402	-1.15118
C	3.81402	1.32244	-0.49749
C	2.74242	0.30765	-0.24986
H	-3.21822	0.29402	0.54719
C	-2.71189	2.07447	1.58243
C	-2.96086	1.76476	-0.80743
C	-2.73448	1.50305	2.86841
C	-2.39635	3.44620	1.40839
C	-2.64136	3.14706	-1.00015
C	-2.44327	2.29569	3.96409
H	-2.97238	0.45038	2.98435
C	-2.10544	4.22666	2.54840
C	-2.37918	3.95112	0.07118
H	-2.61528	3.52737	-2.01192
C	-2.12795	3.66112	3.80912
H	-2.45642	1.85718	4.95693
H	-1.86166	5.27690	2.41613
H	-2.14256	5.00006	-0.08577
H	-1.90210	4.26471	4.68197
C	-3.09586	0.78871	-1.85987
H	-1.76279	0.37783	-2.01214
C	-3.97419	-0.45105	-1.62565
H	-3.91544	-1.08649	-2.51833
H	-5.01904	-0.14135	-1.51848
N	-3.55349	-1.17846	-0.41383
N	-3.00177	1.31686	0.47223
C	-4.41068	-2.17157	0.13272

C	-5.79901	-2.00197	0.24260
C	-3.82526	-3.34133	0.64732
C	-6.57949	-2.98520	0.85205
H	-6.27708	-1.10502	-0.13765
C	-4.61387	-4.31112	1.26042
H	-2.75157	-3.47496	0.55041
C	-5.99658	-4.14311	1.36641
H	-7.65356	-2.83819	0.92524
H	-4.14301	-5.20979	1.64919
H	-6.60971	-4.90429	1.83889
H	-2.60815	-1.56906	-0.54991
Cl	-3.50561	1.50030	-3.47084
H	2.15710	-3.97733	-2.01894
H	0.20427	2.48611	0.45553

Catalyst (H, 4)-Enamine protonation TS (pro-R) alternative conformation
Electronic energy (Hartrees): -1870.613767
Free Energy at 1M concentration and 253 K (Hartrees): -1870.075549
Coordinates:

P	-0.41838	-0.56695	0.04189
O	0.66778	0.62209	0.32384
O	0.42513	-1.93711	-0.22312
O	-1.29259	-0.31284	-1.14576
O	-1.12655	-0.69576	1.42752
C	1.66889	0.68629	-0.60945
C	1.72051	1.83992	-1.52980
C	2.70012	1.93979	-2.43166
H	2.72761	2.79106	-3.10046
C	3.72361	0.87813	-2.53810
C	4.76571	0.96503	-3.58700
H	4.80185	1.82191	-4.24822
C	5.66489	-0.01532	-3.72399
H	6.42517	0.04836	-4.49173
C	5.61505	-1.19411	-2.83825
H	6.33159	-1.99460	-2.97009
C	4.68078	-1.28080	-1.88419
H	4.64919	-2.16551	-1.26274
C	3.69256	-0.18371	-1.69662
C	2.64281	-0.24992	-0.63265
C	1.54516	-2.10859	0.55440
C	1.58457	-3.25808	1.48096
C	2.66730	-3.49247	2.22605
H	2.68041	-4.33973	2.90042
C	3.83035	-2.58472	2.15301
C	4.99518	-2.81214	3.03932
H	5.01190	-3.66380	3.70802
C	6.02989	-1.96545	3.02011
H	6.87985	-2.13143	3.66936
C	6.00958	-0.79336	2.12488
H	6.83902	-0.09795	2.13309
C	4.96483	-0.58151	1.31612
H	4.96214	0.29957	0.68901
C	3.81961	-1.53328	1.29879
C	2.64128	-1.32533	0.39853
H	-2.98769	-0.24876	-0.83188
C	-4.67051	-1.34878	-1.26140
C	-4.26389	-0.10450	0.78786
C	-4.21686	-1.70225	-2.54677
C	-5.87492	-1.88977	-0.74796
C	-5.45749	-0.68715	1.33292

C	-4.97659	-2.56820	-3.31354
H	-3.27455	-1.30146	-2.90609
C	-6.62862	-2.76459	-1.55954
C	-6.23340	-1.52493	0.58709
H	-5.73599	-0.41492	2.34101
C	-6.18925	-3.09768	-2.82742
H	-4.63018	-2.84387	-4.30483
H	-7.55517	-3.17519	-1.16823
H	-7.15113	-1.92672	1.00859
H	-6.77162	-3.77124	-3.44752
C	-3.36543	0.75876	1.49126
H	-2.08479	-0.14488	1.52195
C	-2.86544	2.02122	0.79553
H	-3.63736	2.80241	0.86367
H	-2.72889	1.80067	-0.27453
N	-1.63903	2.52156	1.39100
N	-3.93928	-0.47716	-0.48492
C	-0.92377	3.56572	0.82109
C	-1.46583	4.40214	-0.17268
C	0.39178	3.82188	1.26337
C	-0.71293	5.45421	-0.69678
H	-2.47222	4.23416	-0.53958
C	1.13018	4.86940	0.72854
H	0.82495	3.18044	2.02606
C	0.58698	5.69893	-0.25858
H	-1.15468	6.08474	-1.46397
H	2.14264	5.03999	1.08537
H	1.16704	6.51659	-0.67455
H	-1.06782	1.81916	1.83920
Cl	-3.84157	1.10967	3.20044
H	0.96017	2.60796	-1.47273
H	0.72901	-3.91763	1.55259

Catalyst (H, 4)-Enamine protonation TS (pro-R) alternative conformation
Electronic energy (Hartrees): -1870.613767
Free Energy at 1M concentration and 253 K (Hartrees): -1870.075547
Coordinates:

P	0.41833	-0.56683	-0.04196
O	-0.66785	0.62217	-0.32388
O	-0.42514	-1.93702	0.22307
O	1.29257	-0.31276	1.14565
O	1.12637	-0.69565	-1.42768
C	-1.66892	0.68636	0.60944
C	-1.72050	1.83998	1.52981
C	-2.70009	1.93985	2.43168
H	-2.72754	2.79112	3.10050
C	-3.72360	0.87822	2.53812
C	-4.76570	0.96514	3.58704
H	-4.80182	1.82202	4.24825
C	-5.66490	-0.01519	3.72402
H	-6.42518	0.04851	4.49177
C	-5.61509	-1.19398	2.83828
H	-6.33165	-1.99445	2.97012
C	-4.68083	-1.28069	1.88422
H	-4.64926	-2.16539	1.26277
C	-3.69258	-0.18362	1.69664
C	-2.64284	-0.24984	0.63266
C	-1.54520	-2.10852	-0.55440
C	-1.58465	-3.25803	-1.48093
C	-2.66740	-3.49241	-2.22601

H	-2.68053	-4.33967	-2.90038
C	-3.83042	-2.58464	-2.15298
C	-4.99526	-2.81202	-3.03929
H	-5.01201	-3.66369	-3.70799
C	-6.02995	-1.96531	-3.02008
H	-6.87991	-2.13127	-3.66933
C	-6.00962	-0.79322	-2.12486
H	-6.83904	-0.09779	-2.13306
C	-4.96486	-0.58139	-1.31610
H	-4.96215	0.29968	-0.68898
C	-3.81966	-1.53319	-1.29877
C	-2.64132	-1.32526	-0.39851
H	2.98765	-0.24862	0.83181
C	4.67036	-1.34875	1.26144
C	4.26382	-0.10470	-0.78799
C	4.21674	-1.70199	2.54688
C	5.87471	-1.88993	0.74806
C	5.45730	-0.68763	-1.33302
C	4.97645	-2.56787	3.31377
H	3.27448	-1.30108	2.90621
C	6.62838	-2.76466	1.55975
C	6.23315	-1.52537	-0.58709
H	5.73579	-0.41562	-2.34117
C	6.18905	-3.09750	2.82770
H	4.63005	-2.84334	4.30512
H	7.55490	-3.17537	1.16846
H	7.15081	-1.92734	-1.00858
H	6.77142	-3.77098	3.44790
C	3.36555	0.75865	-1.49149
H	2.08457	-0.14499	-1.52221
C	2.86562	2.02121	-0.79589
H	3.63776	2.80221	-0.86375
H	2.72862	1.80066	0.27411
N	1.63955	2.52197	-1.39176
N	3.93921	-0.47713	0.48487
C	0.92412	3.56578	-0.82135
C	1.46613	4.40191	0.17269
C	-0.39153	3.82186	-1.26337
C	0.71310	5.45365	0.69726
H	2.47258	4.23398	0.53944
C	-1.13004	4.86908	-0.72810
H	-0.82469	3.18060	-2.02621
C	-0.58688	5.69833	0.25927
H	1.15482	6.08396	1.46466
H	-2.14257	5.03962	-1.08475
H	-1.16704	6.51574	0.67560
H	1.06829	1.81965	-1.84004
Cl	3.84184	1.10943	-3.20065
H	-0.96013	2.60800	1.47274
H	-0.72912	-3.91762	-1.55256

Catalyst (H, 4)-Enamine protonation TS (pro-R) alternative conformation
Electronic energy (Hartrees): -1870.61233
Free Energy at 1M concentration and 253 K (Hartrees): -1870.074224
Coordinates:

P	-0.14347	0.76094	0.00601
O	-0.91268	-0.60821	0.47560
O	-1.28596	1.83082	-0.44868
O	0.78552	0.53980	-1.14730
O	0.48066	1.27536	1.33732

C	-1.87316	-1.03195	-0.40641
C	-1.65657	-2.29063	-1.14773
C	-2.58274	-2.74180	-1.99748
H	-2.41179	-3.66631	-2.53490
C	-3.82272	-1.96962	-2.22436
C	-4.81177	-2.44413	-3.21961
H	-4.64555	-3.37256	-3.75172
C	-5.91297	-1.72704	-3.46750
H	-6.63443	-2.07382	-4.19603
C	-6.14144	-0.45334	-2.75904
H	-7.02329	0.13347	-2.98178
C	-5.25688	-0.01762	-1.85444
H	-5.43398	0.93090	-1.36577
C	-4.04206	-0.81935	-1.54250
C	-3.04010	-0.36019	-0.53039
C	-2.41458	1.85684	0.33383
C	-2.72280	3.09203	1.08262
C	-3.83101	3.17790	1.82236
H	-4.04325	4.09044	2.36552
C	-4.74915	2.02476	1.92210
C	-5.93755	2.10696	2.80245
H	-6.15430	3.02187	3.33978
C	-6.74653	1.05121	2.93995
H	-7.61381	1.11165	3.58469
C	-6.45130	-0.20468	2.22481
H	-7.09653	-1.06317	2.36011
C	-5.38329	-0.28821	1.42300
H	-5.17398	-1.22789	0.93023
C	-4.49077	0.88823	1.23143
C	-3.29389	0.82587	0.33410
H	2.47621	0.82771	-0.83810
C	3.96115	2.09247	-1.48070
C	3.72581	1.19643	0.76600
C	3.48076	2.12319	-2.80410
C	5.05234	2.90731	-1.09078
C	4.79860	2.05544	1.18029
C	4.10428	2.94422	-3.72687
H	2.62396	1.51356	-3.07217
C	5.66900	3.72849	-2.05923
C	5.44075	2.85821	0.28376
H	5.09995	2.02364	2.21773
C	5.20524	3.74546	-3.36135
H	3.73721	2.97101	-4.74822
H	6.50944	4.34931	-1.76179
H	6.27198	3.47634	0.61286
H	5.68211	4.37852	-4.10244
C	2.95780	0.34400	1.62082
H	1.52147	0.95783	1.53059
C	2.62249	-1.06958	1.17864
H	2.21088	-1.02734	0.16600
H	1.81856	-1.46189	1.82036
N	3.79164	-1.94572	1.16606
N	3.36535	1.27464	-0.54693
C	3.64934	-3.29912	0.85299
C	2.51696	-3.80509	0.18775
C	4.69085	-4.19355	1.17264
C	2.44551	-5.15680	-0.15182
H	1.69023	-3.15016	-0.06204
C	4.60409	-5.53810	0.83231
H	5.57238	-3.81701	1.68646

C	3.48072	-6.03489	0.16392
H	1.56052	-5.52317	-0.66548
H	5.42176	-6.20495	1.09254
H	3.41554	-7.08549	-0.10083
H	4.40473	-1.78129	1.95522
Cl	3.47345	0.36038	3.36128
H	-0.74265	-2.85128	-0.99833
H	-2.04574	3.93502	1.02731

Catalyst (H, 4)-Enamine protonation TS (pro-R) alternative conformation
Electronic energy (Hartrees): -1870.61233
Free Energy at 1M concentration and 253 K (Hartrees): -1870.074222
Coordinates:

P	-0.14317	-0.76130	-0.00524
O	-0.91228	0.60788	-0.47496
O	-1.28571	-1.83099	0.44975
O	0.78596	-0.54004	1.14794
O	0.48078	-1.27588	-1.33656
C	-1.87294	1.03169	0.40682
C	-1.65648	2.29044	1.14806
C	-2.58292	2.74181	1.99741
H	-2.41208	3.66640	2.53473
C	-3.82303	1.96974	2.22401
C	-4.81241	2.44451	3.21880
H	-4.64633	3.37305	3.75078
C	-5.91374	1.72752	3.46645
H	-6.63545	2.07449	4.19464
C	-6.14203	0.45369	2.75817
H	-7.02399	-0.13304	2.98073
C	-5.25717	0.01773	1.85399
H	-5.43414	-0.93090	1.36546
C	-4.04219	0.81934	1.54229
C	-3.03993	0.35996	0.53057
C	-2.41420	-1.85728	-0.33294
C	-2.72225	-3.09271	-1.08140
C	-3.83027	-3.17875	-1.82144
H	-4.04240	-4.09144	-2.36440
C	-4.74833	-2.02558	-1.92178
C	-5.93651	-2.10798	-2.80242
H	-6.15317	-3.02304	-3.33954
C	-6.74542	-1.05222	-2.94044
H	-7.61254	-1.11281	-3.58540
C	-6.45032	0.20387	-2.22560
H	-7.09546	1.06236	-2.36132
C	-5.38250	0.28758	-1.42352
H	-5.17328	1.22739	-0.93097
C	-4.49010	-0.88886	-1.23138
C	-3.29348	-0.82629	-0.33372
H	2.47633	-0.82744	0.83804
C	3.96150	-2.09194	1.48057
C	3.72587	-1.19606	-0.76616
C	3.48123	-2.12259	2.80401
C	5.05272	-2.90671	1.09059
C	4.79872	-2.05496	-1.18050
C	4.10490	-2.94352	3.72677
H	2.62442	-1.51300	3.07212
C	5.66952	-3.72779	2.05903
C	5.44100	-2.85765	-0.28399
H	5.09997	-2.02320	-2.21797
C	5.20588	-3.74471	3.36119

H	3.73793	-2.97027	4.74816
H	6.50997	-4.34857	1.76155
H	6.27224	-3.47574	-0.61313
H	5.68286	-4.37768	4.10228
C	2.95756	-0.34386	-1.62096
H	1.52153	-0.95796	-1.53029
C	2.62193	1.06964	-1.17871
H	2.21027	1.02730	-0.16609
H	1.81794	1.46179	-1.82047
N	3.79090	1.94600	-1.16602
N	3.36552	-1.27423	0.54680
C	3.64836	3.29940	-0.85316
C	2.51552	3.80535	-0.18870
C	4.69002	4.19386	-1.17220
C	2.44378	5.15708	0.15073
H	1.68868	3.15038	0.06062
C	4.60297	5.53843	-0.83204
H	5.57192	3.81733	-1.68541
C	3.47913	6.03521	-0.16440
H	1.55844	5.52344	0.66381
H	5.42077	6.20531	-1.09178
H	3.41373	7.08582	0.10024
H	4.40445	1.78140	-1.95480
Cl	3.47302	-0.36011	-3.36146
H	-0.74245	2.85099	0.99891
H	-2.04526	-3.93573	-1.02563

Catalyst (H, 4)-Enamine protonation TS (pro-R) alternative conformation
Electronic energy (Hartrees): -1870.610845
Free Energy at 1M concentration and 253 K (Hartrees): -1870.07385
Coordinates:

P	0.58824	0.52694	-0.11762
O	1.66776	0.85109	1.07068
O	1.35190	-0.41845	-1.21457
O	0.09549	1.74057	-0.84109
O	-0.45069	-0.32467	0.66911
C	2.92242	1.17843	0.62152
C	3.42179	2.54960	0.84900
C	4.65174	2.89519	0.46027
H	5.00474	3.90421	0.63367
C	5.51711	1.91432	-0.22754
C	6.85262	2.32889	-0.71598
H	7.20948	3.33535	-0.53575
C	7.62252	1.46333	-1.38399
H	8.59764	1.76761	-1.74188
C	7.13934	0.09479	-1.64819
H	7.75779	-0.59303	-2.21038
C	5.93656	-0.29180	-1.20719
H	5.59428	-1.29211	-1.43514
C	5.08020	0.64834	-0.43299
C	3.73415	0.24080	0.07913
C	2.10067	-1.43104	-0.66925
C	1.67779	-2.82794	-0.89433
C	2.39882	-3.83999	-0.40522
H	2.07698	-4.85954	-0.57760
C	3.61452	-3.57011	0.39088
C	4.36780	-4.69803	0.98617
H	4.05167	-5.71818	0.80688
C	5.43983	-4.46176	1.74982
H	5.98875	-5.28653	2.18567

C	5.86961	-3.07526	2.01278
H	6.72394	-2.89065	2.65127
C	5.19936	-2.04897	1.47603
H	5.51885	-1.04105	1.70362
C	4.02356	-2.29483	0.59658
C	3.25968	-1.16682	-0.02297
H	-1.60561	2.02466	-0.77448
C	-2.69380	3.55508	0.07306
C	-3.27830	1.20747	0.10854
C	-1.80204	4.55138	-0.37088
C	-3.78352	3.88388	0.91896
C	-4.36299	1.51788	0.99248
C	-2.01891	5.86295	0.00936
H	-0.95391	4.26595	-0.98417
C	-3.97973	5.23556	1.27974
C	-4.60951	2.80882	1.36621
H	-5.02228	0.71612	1.29545
C	-3.11290	6.21234	0.82925
H	-1.33425	6.63511	-0.32768
H	-4.81856	5.49069	1.92111
H	-5.46021	3.03393	2.00403
H	-3.26623	7.24912	1.11049
C	-2.91586	-0.13360	-0.26392
H	-1.51967	-0.26963	0.32166
C	-3.79221	-1.27790	0.17887
H	-3.89375	-1.21334	1.27015
H	-3.26543	-2.22226	-0.02422
N	-5.12403	-1.25694	-0.42709
N	-2.50804	2.24481	-0.30162
C	-6.01602	-2.31671	-0.24393
C	-5.89150	-3.23183	0.81709
C	-7.10899	-2.45651	-1.12241
C	-6.83819	-4.24296	0.98958
H	-5.05857	-3.16047	1.50782
C	-8.04258	-3.46983	-0.94098
H	-7.21797	-1.75664	-1.94775
C	-7.91815	-4.37384	0.11847
H	-6.71999	-4.93852	1.81611
H	-8.87423	-3.55562	-1.63506
H	-8.64791	-5.16507	0.25759
H	-5.09721	-0.92063	-1.38236
Cl	-2.47277	-0.29585	-2.03039
H	2.78633	3.27751	1.33734
H	0.77831	-3.03106	-1.46168

Catalyst (H, 4)-Enamine protonation TS (pro-R) alternative conformation
Electronic energy (Hartrees): -1870.610846
Free Energy at 1M concentration and 253 K (Hartrees): -1870.073843
Coordinates:

P	-0.58731	0.51927	0.12380
O	-1.66427	0.84480	-1.06647
O	-1.35405	-0.42568	1.21897
O	-0.09467	1.73218	0.84850
O	0.45239	-0.33319	-0.66101
C	-2.91898	1.17460	-0.61931
C	-3.41531	2.54686	-0.84733
C	-4.64516	2.89482	-0.46035
H	-4.99590	3.90458	-0.63410
C	-5.51351	1.91553	0.22605
C	-6.84896	2.33265	0.71253

H	-7.20356	3.33985	0.53190
C	-7.62158	1.46847	1.37923
H	-8.59666	1.77462	1.73569
C	-7.14149	0.09893	1.64396
H	-7.76216	-0.58778	2.20510
C	-5.93878	-0.28995	1.20473
H	-5.59881	-1.29099	1.43303
C	-5.07938	0.64865	0.43198
C	-3.73334	0.23855	-0.07817
C	-2.10400	-1.43662	0.67215
C	-1.68427	-2.83444	0.89758
C	-2.40662	-3.84497	0.40725
H	-2.08705	-4.86521	0.57991
C	-3.62066	-3.57253	-0.39055
C	-4.37529	-4.69885	-0.98721
H	-4.06142	-5.71966	-0.80767
C	-5.44575	-4.46029	-1.75239
H	-5.99565	-5.28389	-2.18924
C	-5.87242	-3.07287	-2.01569
H	-6.72545	-2.88644	-2.65542
C	-5.20094	-2.04802	-1.47771
H	-5.51811	-1.03942	-1.70557
C	-4.02690	-2.29639	-0.59656
C	-3.26171	-1.17000	0.02438
H	1.60506	2.02102	0.77923
C	2.68646	3.55344	-0.07344
C	3.27697	1.20728	-0.10812
C	1.79347	4.54791	0.37213
C	3.77262	3.88411	-0.92316
C	4.35810	1.51955	-0.99582
C	2.00564	5.85960	-0.01035
H	0.94806	4.26098	0.98847
C	3.96411	5.23586	-1.28615
C	4.60004	2.81068	-1.37177
H	5.01854	0.71920	-1.29996
C	3.09612	6.21090	-0.83409
H	1.32001	6.63034	0.32794
H	4.80023	5.49245	-1.93046
H	5.44813	3.03730	-2.01252
H	3.24582	7.24774	-1.11704
C	2.91920	-0.13423	0.26690
H	1.52145	-0.27464	-0.31542
C	3.79745	-1.27671	-0.17676
H	3.89608	-1.21318	-1.26836
H	3.27374	-2.22226	0.02876
N	5.13073	-1.25136	0.42583
N	2.50537	2.24313	0.30337
C	6.02529	-2.30876	0.24153
C	5.90078	-3.22530	-0.81826
C	7.12083	-2.44455	1.11745
C	6.84993	-4.23389	-0.99208
H	5.06593	-3.15702	-1.50698
C	8.05686	-3.45538	0.93472
H	7.22986	-1.74353	1.94180
C	7.93240	-4.36081	-0.12351
H	6.73167	-4.93062	-1.81762
H	8.89048	-3.53808	1.62682
H	8.66408	-5.15008	-0.26365
H	5.10537	-0.91409	1.38079
Cl	2.48108	-0.29557	2.03464

H	-2.77766	3.27359	-1.33461
H	-0.78600	-3.03948	1.46622

Catalyst (H, 4)-Enamine protonation TS (pro-R) alternative conformation
Electronic energy (Hartrees): -1870.611471
Free Energy at 1M concentration and 253 K (Hartrees): -1870.073543
Coordinates:

P	-0.33442	-0.13217	-0.32987
O	-1.37888	0.47986	-1.42818
O	-1.21238	-0.63649	0.95017
O	0.67397	0.86064	0.15944
O	0.20906	-1.39425	-1.06629
C	-2.32376	1.31986	-0.89536
C	-2.28507	2.75217	-1.25299
C	-3.20550	3.59356	-0.77518
H	-3.16531	4.64145	-1.04555
C	-4.25720	3.10320	0.14025
C	-5.23078	4.05791	0.71874
H	-5.19659	5.10438	0.44214
C	-6.15451	3.63261	1.58713
H	-6.86457	4.33125	2.01053
C	-6.19981	2.21203	1.98226
H	-6.93484	1.87934	2.70385
C	-5.32824	1.34004	1.46206
H	-5.36547	0.30886	1.78618
C	-4.31459	1.78993	0.46883
C	-3.33249	0.83544	-0.13483
C	-2.37633	-1.29351	0.63523
C	-2.50467	-2.71856	1.00106
C	-3.63341	-3.38453	0.74511
H	-3.71300	-4.42922	1.01910
C	-4.75817	-2.70773	0.06657
C	-5.97810	-3.47512	-0.27539
H	-6.06147	-4.51773	0.00530
C	-6.98077	-2.88491	-0.93451
H	-7.87119	-3.44685	-1.18534
C	-6.87060	-1.46947	-1.33508
H	-7.67575	-1.00491	-1.88966
C	-5.77513	-0.76551	-1.02689
H	-5.70741	0.26326	-1.35366
C	-4.66343	-1.39726	-0.26408
C	-3.43011	-0.63166	0.10133
H	2.35928	0.13904	0.42044
C	3.60483	-0.65706	1.84806
C	3.42104	-1.38045	-0.47371
C	3.20596	0.25135	2.84558
C	4.50786	-1.70469	2.15076
C	4.30450	-2.47243	-0.15610
C	3.72110	0.12132	4.12450
H	2.49663	1.03496	2.59914
C	5.01762	-1.80613	3.46113
C	4.82979	-2.61377	1.09157
H	4.56144	-3.15930	-0.95010
C	4.63325	-0.90429	4.43798
H	3.41517	0.82266	4.89478
H	5.71529	-2.60674	3.69080
H	5.51928	-3.42841	1.29698
H	5.02964	-0.98730	5.44466
C	2.79170	-1.16936	-1.72560
H	1.26782	-1.38582	-1.31744

C	2.66313	0.22347	-2.41895
H	1.64857	0.33120	-2.81584
H	3.32681	0.20227	-3.29082
N	2.95739	1.38857	-1.63390
N	3.10550	-0.54896	0.56855
C	4.23431	1.82781	-1.31705
C	5.39530	1.20669	-1.81561
C	4.38395	2.95091	-0.47707
C	6.65520	1.71077	-1.49219
H	5.31822	0.32953	-2.44861
C	5.64684	3.43706	-0.16015
H	3.49564	3.44217	-0.08695
C	6.79746	2.82472	-0.66586
H	7.53626	1.21548	-1.89161
H	5.73239	4.30606	0.48680
H	7.78208	3.20784	-0.41771
H	2.18744	1.76126	-1.09452
Cl	3.13664	-2.42055	-2.98421
H	-1.50645	3.12027	-1.90904
H	-1.67784	-3.22470	1.48306

Catalyst (H, 4)-Enamine protonation TS (pro-R) alternative conformation
Electronic energy (Hartrees): -1870.610105
Free Energy at 1M concentration and 253 K (Hartrees): -1870.072258
Coordinates:

P	0.46757	-0.81146	-0.21048
O	-0.40951	-1.44059	1.02195
O	-0.58775	-0.33104	-1.36489
O	1.41486	-1.78059	-0.84971
O	1.07386	0.43218	0.49575
C	-1.50767	-2.17501	0.65014
C	-1.54392	-3.60868	1.00307
C	-2.61604	-4.35104	0.71690
H	-2.62736	-5.40077	0.98287
C	-3.77041	-3.75062	0.01760
C	-4.92439	-4.60054	-0.35663
H	-4.93924	-5.64852	-0.08407
C	-5.95171	-4.07857	-1.03485
H	-6.79382	-4.70073	-1.30923
C	-5.93387	-2.65614	-1.42471
H	-6.75716	-2.24629	-1.99553
C	-4.89876	-1.87813	-1.08740
H	-4.89758	-0.84546	-1.40843
C	-3.76197	-2.43417	-0.30238
C	-2.59504	-1.58487	0.09688
C	-1.61525	0.43844	-0.88281
C	-1.68772	1.86033	-1.27443
C	-2.69148	2.62780	-0.84295
H	-2.74449	3.66419	-1.15033
C	-3.70916	2.07449	0.07422
C	-4.75880	2.96080	0.62700
H	-4.80122	4.00236	0.33340
C	-5.65512	2.48005	1.49522
H	-6.42063	3.12893	1.90073
C	-5.59542	1.06647	1.91399
H	-6.31148	0.68945	2.63280
C	-4.65294	0.25613	1.41809
H	-4.61421	-0.77037	1.75707
C	-3.66501	0.76792	0.42984
C	-2.59900	-0.11416	-0.13857

H	3.02821	-1.14176	-0.87477
C	4.70022	-1.89231	0.05137
C	4.14619	0.45347	-0.17703
C	4.35386	-3.22806	-0.23605
C	5.83143	-1.59533	0.85394
C	5.25910	0.76474	0.67097
C	5.15098	-4.24583	0.25301
H	3.45662	-3.42698	-0.81219
C	6.62850	-2.66115	1.32912
C	6.07621	-0.22178	1.14848
H	5.47126	1.80095	0.89581
C	6.29660	-3.96767	1.02967
H	4.88821	-5.27703	0.03816
H	7.49935	-2.43448	1.93764
H	6.93773	0.03747	1.75826
H	6.90932	-4.78402	1.39794
C	3.20727	1.43326	-0.65820
H	1.98280	0.94860	0.02012
C	3.53833	2.91536	-0.44674
H	4.44975	3.18932	-0.99742
H	3.75419	3.06976	0.61660
N	2.52296	3.86675	-0.83569
N	3.93301	-0.86127	-0.43673
C	1.35479	4.12589	-0.11862
C	1.10079	3.60178	1.16047
C	0.40620	5.00617	-0.67820
C	-0.05879	3.95751	1.84995
H	1.78223	2.89004	1.60998
C	-0.74213	5.35620	0.02168
H	0.59020	5.42372	-1.66540
C	-0.98833	4.83566	1.29557
H	-0.23647	3.52617	2.83119
H	-1.45382	6.03873	-0.43514
H	-1.89023	5.10295	1.83717
H	2.43239	3.98473	-1.83450
Cl	2.70747	1.14251	-2.38870
H	-0.69535	-4.05888	1.50247
H	-0.92870	2.27835	-1.92365

Catalyst (H, 4)-Enamine protonation TS (pro-R) alternative conformation
Electronic energy (Hartrees): -1870.608972
Free Energy at 1M concentration and 253 K (Hartrees): -1870.071875
Coordinates:

P	0.19013	0.23886	-1.27244
O	0.74405	0.38327	0.27005
O	1.32305	-0.58560	-2.11239
O	-0.02289	1.55579	-1.94463
O	-1.04681	-0.68778	-1.07003
C	2.03422	0.83985	0.34077
C	2.28655	2.19182	0.87830
C	3.53435	2.65810	0.97228
H	3.70654	3.65151	1.36756
C	4.67281	1.83292	0.51482
C	6.04630	2.38523	0.56275
H	6.22073	3.37525	0.96548
C	7.07667	1.66507	0.10661
H	8.07981	2.07011	0.14070
C	6.84795	0.32124	-0.45762
H	7.68361	-0.24659	-0.84601
C	5.61190	-0.18949	-0.50156

H	5.46586	-1.16676	-0.94145
C	4.45801	0.58528	0.03107
C	3.06644	0.03667	0.00045
C	1.93435	-1.61161	-1.43396
C	1.74876	-2.99042	-1.92967
C	2.36143	-4.01462	-1.33108
H	2.21523	-5.01987	-1.70646
C	3.21268	-3.78186	-0.14627
C	3.82930	-4.93359	0.55183
H	3.68930	-5.93819	0.17242
C	4.55913	-4.73861	1.65513
H	5.00798	-5.58096	2.16552
C	4.74272	-3.37586	2.18898
H	5.31414	-3.22791	3.09629
C	4.19443	-2.32778	1.56335
H	4.32206	-1.34186	1.98910
C	3.40693	-2.52278	0.31468
C	2.78875	-1.36789	-0.41030
H	-1.58097	2.37152	-1.46280
C	-1.83209	3.58565	0.17615
C	-3.16671	1.64459	-0.39206
C	-0.77056	4.44112	-0.17534
C	-2.50834	3.74171	1.41183
C	-3.83241	1.76541	0.87260
C	-0.40741	5.45370	0.69426
H	-0.24131	4.27425	-1.10751
C	-2.11949	4.79370	2.26991
C	-3.52628	2.78834	1.72404
H	-4.57327	1.02206	1.13182
C	-1.08571	5.64029	1.91646
H	0.41440	6.11241	0.43093
H	-2.64046	4.91898	3.21487
H	-4.06029	2.88510	2.66561
H	-0.78862	6.44503	2.58100
C	-3.37007	0.54868	-1.29437
H	-2.05294	-0.16948	-1.16737
C	-4.50204	-0.43619	-1.03240
H	-4.62746	-1.05942	-1.92900
H	-5.46039	0.08001	-0.87261
N	-4.19204	-1.22976	0.15229
N	-2.21780	2.57419	-0.67351
C	-5.05025	-2.23515	0.60362
C	-6.38701	-2.34055	0.17955
C	-4.57350	-3.16246	1.55223
C	-7.21485	-3.33506	0.70366
H	-6.78311	-1.65605	-0.56196
C	-5.40666	-4.15187	2.05956
H	-3.54137	-3.09265	1.88746
C	-6.73836	-4.24702	1.64329
H	-8.24398	-3.39696	0.36028
H	-5.01207	-4.85588	2.78718
H	-7.38713	-5.02060	2.04152
H	-3.21059	-1.47551	0.21055
Cl	-3.29281	1.00872	-3.04325
H	1.45477	2.80577	1.19975
H	1.11342	-3.17019	-2.78765

Catalyst (H, 4)-Enamine protonation TS (pro-R) alternative conformation
Electronic energy (Hartrees): -1870.609079
Free Energy at 1M concentration and 253 K (Hartrees): -1870.071807

Coordinates:

P	-0.26088	-0.21568	-0.11593
O	0.80693	1.01976	-0.23996
O	0.60631	-1.57907	0.13815
O	-1.06714	-0.40597	-1.36928
O	-1.04923	0.10654	1.17892
C	1.90238	0.74440	-1.01910
C	2.07201	1.47676	-2.29031
C	3.14349	1.26145	-3.05750
H	3.25471	1.80548	-3.98727
C	4.15771	0.26475	-2.65517
C	5.30619	-0.01535	-3.54765
H	5.42375	0.53601	-4.47229
C	6.20146	-0.95206	-3.21715
H	7.03953	-1.15715	-3.87067
C	6.04186	-1.71955	-1.96749
H	6.75694	-2.49293	-1.71816
C	5.01078	-1.47128	-1.15151
H	4.90160	-2.06450	-0.25379
C	4.02317	-0.40901	-1.48737
C	2.86493	-0.10428	-0.58970
C	1.62533	-1.44867	1.04888
C	1.53927	-2.19368	2.32111
C	2.52494	-2.11614	3.21877
H	2.44841	-2.66781	4.14753
C	3.70144	-1.26031	2.95890
C	4.75509	-1.12713	3.99142
H	4.68321	-1.68459	4.91709
C	5.79829	-0.31598	3.78652
H	6.56696	-0.21809	4.54223
C	5.89774	0.45950	2.53553
H	6.73252	1.13224	2.38659
C	4.95410	0.34297	1.59395
H	5.03632	0.94052	0.69619
C	3.80309	-0.58044	1.79134
C	2.73720	-0.73622	0.75232
H	-2.69484	-0.94162	-1.05271
C	-3.85941	-2.63476	-0.87071
C	-4.07014	-0.60135	0.44655
C	-3.21251	-3.30795	-1.92436
C	-4.84093	-3.29336	-0.08979
C	-5.03269	-1.27612	1.26898
C	-3.56348	-4.61725	-2.20349
H	-2.44200	-2.79424	-2.49042
C	-5.17961	-4.62649	-0.40591
C	-5.41008	-2.55837	0.99723
H	-5.46638	-0.72600	2.09226
C	-4.55321	-5.28065	-1.45073
H	-3.06616	-5.13982	-3.01480
H	-5.93589	-5.13019	0.18949
H	-6.16305	-3.04140	1.61451
H	-4.81660	-6.30588	-1.68915
C	-3.56192	0.72114	0.64898
H	-2.13230	0.40865	1.00764
C	-3.46012	1.65195	-0.58275
H	-4.32654	2.32246	-0.57712
H	-3.56350	1.02387	-1.47417
N	-2.25375	2.43049	-0.72657
N	-3.53582	-1.32753	-0.57979
C	-2.11393	3.79060	-0.49017

C	-3.07253	4.55677	0.19919
C	-0.95083	4.44083	-0.95280
C	-2.87371	5.92360	0.39209
H	-3.95759	4.08487	0.60921
C	-0.76356	5.80204	-0.74526
H	-0.19350	3.85920	-1.47130
C	-1.72593	6.56274	-0.07563
H	-3.62990	6.49108	0.92833
H	0.14376	6.27334	-1.11416
H	-1.57912	7.62623	0.08406
H	-1.53568	2.00099	-1.29046
Cl	-4.25349	1.57297	2.08798
H	1.32284	2.19316	-2.60122
H	0.67178	-2.80862	2.52465

Catalyst (H, 4)-Enamine protonation TS (pro-R) alternative conformation
Electronic energy (Hartrees): -1870.607973
Free Energy at 1M concentration and 253 K (Hartrees): -1870.07087
Coordinates:

P	-0.05404	1.05595	-0.05087
O	0.96461	1.43606	1.17322
O	0.85481	0.40310	-1.24547
O	-0.80373	2.21575	-0.62250
O	-0.88991	-0.07884	0.62360
C	2.18054	1.94502	0.78795
C	2.51627	3.33079	1.17302
C	3.70729	3.85046	0.86595
H	3.93848	4.86810	1.15539
C	4.69518	3.05111	0.11235
C	5.98529	3.66201	-0.28323
H	6.21956	4.67734	0.01169
C	6.86532	2.96375	-1.00849
H	7.80712	3.41149	-1.29841
C	6.54931	1.58543	-1.42832
H	7.25601	1.03450	-2.03550
C	5.38894	1.02212	-1.07227
H	5.17002	0.01946	-1.41346
C	4.41161	1.77323	-0.23693
C	3.10975	1.16518	0.18298
C	1.70517	-0.57997	-0.80852
C	1.43443	-1.98148	-1.18725
C	2.24262	-2.96020	-0.77266
H	2.03165	-3.98445	-1.05418
C	3.40225	-2.65104	0.09074
C	4.25340	-3.75179	0.59821
H	4.05020	-4.77494	0.30724
C	5.27448	-3.48760	1.42030
H	5.89495	-4.29285	1.79194
C	5.55024	-2.09893	1.83503
H	6.36406	-1.89518	2.51898
C	4.79044	-1.09617	1.37918
H	4.99705	-0.08960	1.71665
C	3.66972	-1.36854	0.43826
C	2.80530	-0.26694	-0.08905
H	-2.55976	1.95996	-0.80641
C	-4.13030	3.01877	-0.00607
C	-3.96967	0.60510	-0.16182
C	-3.54834	4.26910	-0.29294
C	-5.33949	2.93590	0.73029
C	-5.16563	0.50747	0.62161

C	-4.18863	5.41904	0.13075
H	-2.60076	4.30323	-0.82007
C	-5.97109	4.13167	1.13883
C	-5.82880	1.62973	1.03188
H	-5.56143	-0.47322	0.84758
C	-5.40696	5.35670	0.84010
H	-3.74387	6.38554	-0.08503
H	-6.90128	4.06973	1.69639
H	-6.75592	1.53483	1.59098
H	-5.89365	6.27318	1.15716
C	-3.17609	-0.51089	-0.59000
H	-1.91840	-0.25921	0.15745
C	-3.62024	-1.93369	-0.31956
H	-4.34598	-2.28251	-1.06847
H	-4.12735	-1.96019	0.66070
N	-2.46611	-2.81525	-0.32967
N	-3.52179	1.86038	-0.42718
C	-2.55434	-4.16800	-0.03928
C	-3.78003	-4.85937	-0.00206
C	-1.37009	-4.89658	0.20184
C	-3.80749	-6.23099	0.25433
H	-4.71108	-4.33151	-0.17629
C	-1.41404	-6.26015	0.46133
H	-0.41595	-4.37559	0.17833
C	-2.63366	-6.94470	0.48884
H	-4.76633	-6.74235	0.27498
H	-0.48562	-6.79453	0.64441
H	-2.66483	-8.01050	0.69128
H	-1.60451	-2.37007	-0.04296
Cl	-2.53611	-0.33117	-2.28196
H	1.79139	3.92750	1.71210
H	0.57364	-2.21538	-1.80104

Catalyst (H, 4)-Enamine protonation TS (pro-R) alternative conformation
Electronic energy (Hartrees): -1870.607284
Free Energy at 1M concentration and 253 K (Hartrees): -1870.070599
Coordinates:

P	0.07142	1.03739	-0.05905
O	1.12757	1.42235	1.13175
O	0.91506	0.22205	-1.20390
O	-0.56184	2.22030	-0.72527
O	-0.86019	0.04568	0.69163
C	2.37373	1.80950	0.70459
C	2.81508	3.19117	0.98301
C	4.03791	3.59622	0.63155
H	4.34696	4.61245	0.84268
C	4.95559	2.67155	-0.06543
C	6.28301	3.15331	-0.51258
H	6.59514	4.16734	-0.29558
C	7.10079	2.33943	-1.18848
H	8.06994	2.69281	-1.51617
C	6.67882	0.96132	-1.50255
H	7.33594	0.31570	-2.07086
C	5.48387	0.51425	-1.09908
H	5.18738	-0.49166	-1.36345
C	4.57425	1.39606	-0.31670
C	3.23556	0.91987	0.15453
C	1.69426	-0.78516	-0.69489
C	1.32179	-2.18709	-0.97207
C	2.06174	-3.18942	-0.49174

H	1.77623	-4.21322	-0.69962
C	3.24896	-2.90361	0.34183
C	4.02527	-4.02356	0.92216
H	3.74707	-5.04765	0.70623
C	5.07129	-3.77533	1.71746
H	5.63704	-4.59443	2.14232
C	5.44933	-2.38399	2.03003
H	6.28278	-2.19087	2.69309
C	4.75791	-1.36441	1.50753
H	5.03908	-0.35372	1.77065
C	3.61082	-1.62213	0.59464
C	2.82247	-0.50168	-0.00700
H	-2.28637	2.12004	-0.77482
C	-3.73112	3.35931	0.00811
C	-3.84475	0.94434	-0.09669
C	-3.01029	4.52943	-0.30386
C	-4.94228	3.42940	0.74340
C	-5.04561	0.99658	0.68164
C	-3.51534	5.75255	0.09545
H	-2.06549	4.44233	-0.82979
C	-5.43318	4.69791	1.12683
C	-5.57805	2.19496	1.06853
H	-5.55130	0.07166	0.92364
C	-4.73276	5.84350	0.80446
H	-2.96396	6.65788	-0.13870
H	-6.36408	4.75423	1.68381
H	-6.51058	2.21753	1.62616
H	-5.11123	6.81581	1.10244
C	-3.18385	-0.27026	-0.49044
H	-1.89338	-0.14556	0.22736
C	-3.83103	-1.60900	-0.20339
H	-4.68708	-1.81222	-0.87419
H	-4.22381	-1.57445	0.82168
N	-2.83276	-2.66029	-0.27182
N	-3.25639	2.13170	-0.38713
C	-3.15590	-4.00180	-0.12856
C	-4.38607	-4.43042	0.40396
C	-2.20677	-4.98094	-0.49007
C	-4.64566	-5.79161	0.57017
H	-5.13980	-3.70514	0.69065
C	-2.48066	-6.33207	-0.32165
H	-1.25009	-4.66413	-0.89922
C	-3.70346	-6.75364	0.21127
H	-5.60292	-6.09774	0.98388
H	-1.73139	-7.06449	-0.60991
H	-3.91417	-7.81025	0.34181
H	-2.08623	-2.47156	-0.92596
Cl	-2.56986	-0.21287	-2.21301
H	2.14217	3.87868	1.47963
H	0.44360	-2.40163	-1.56762

Catalyst (H, 4)-Enamine protonation TS (pro-R) alternative conformation
Electronic energy (Hartrees): -1870.607283
Free Energy at 1M concentration and 253 K (Hartrees): -1870.070597
Coordinates:

P	0.07136	1.03911	-0.05917
O	1.12723	1.42408	1.13187
O	0.91582	0.22617	-1.20510
O	-0.56355	2.22192	-0.72400
O	-0.85871	0.04525	0.69058

C	2.37374	1.81104	0.70553
C	2.81546	3.19226	0.98558
C	4.03865	3.59722	0.63531
H	4.34794	4.61311	0.84766
C	4.95643	2.67292	-0.06202
C	6.28429	3.15467	-0.50787
H	6.59665	4.16837	-0.28965
C	7.10220	2.34119	-1.18409
H	8.07167	2.69456	-1.51084
C	6.67992	0.96355	-1.49983
H	7.33716	0.31829	-2.06842
C	5.48458	0.51649	-1.09754
H	5.18790	-0.48903	-1.36314
C	4.57479	1.39782	-0.31480
C	3.23565	0.92161	0.15516
C	1.69451	-0.78198	-0.69723
C	1.32176	-2.18345	-0.97633
C	2.06104	-3.18658	-0.49663
H	1.77532	-4.21005	-0.70586
C	3.24779	-2.90212	0.33809
C	4.02335	-4.02299	0.91765
H	3.74498	-5.04674	0.70034
C	5.06890	-3.77601	1.71395
H	5.63411	-4.59578	2.13824
C	5.44717	-2.38515	2.02838
H	6.28025	-2.19306	2.69223
C	4.75643	-1.36474	1.50660
H	5.03775	-0.35444	1.77106
C	3.60988	-1.62105	0.59266
C	2.82228	-0.49966	-0.00819
H	-2.28755	2.11978	-0.77469
C	-3.73407	3.35745	0.00746
C	-3.84487	0.94237	-0.09707
C	-3.01451	4.52837	-0.30446
C	-4.94551	3.42622	0.74241
C	-5.04590	0.99328	0.68107
C	-3.52112	5.75095	0.09449
H	-2.06942	4.44228	-0.83004
C	-5.43803	4.69422	1.12547
C	-5.57988	2.19109	1.06763
H	-5.55048	0.06777	0.92314
C	-4.73887	5.84059	0.80310
H	-2.97073	6.65690	-0.13963
H	-6.36914	4.74954	1.68221
H	-6.51255	2.21265	1.62508
H	-5.11858	6.81250	1.10081
C	-3.18270	-0.27165	-0.49050
H	-1.89197	-0.14633	0.22674
C	-3.82899	-1.61079	-0.20333
H	-4.68636	-1.81343	-0.87267
H	-4.21966	-1.57753	0.82258
N	-2.83039	-2.66148	-0.27469
N	-3.25779	2.13035	-0.38748
C	-3.15241	-4.00295	-0.12864
C	-4.38130	-4.43137	0.40703
C	-2.20363	-4.98215	-0.49089
C	-4.63973	-5.79243	0.57609
H	-5.13512	-3.70609	0.69349
C	-2.47642	-6.33316	-0.31964
H	-1.24804	-4.66547	-0.90266

C	-3.69776	-6.75452	0.21678
H	-5.59600	-6.09842	0.99222
H	-1.72741	-7.06564	-0.60841
H	-3.90759	-7.81103	0.34953
H	-2.08658	-2.47287	-0.93195
Cl	-2.56949	-0.21434	-2.21350
H	2.14250	3.87953	1.48248
H	0.44388	-2.39701	-1.57269

Catalyst (H, 4)-Enamine protonation TS (pro-R) alternative conformation
Electronic energy (Hartrees): -1870.605546
Free Energy at 1M concentration and 253 K (Hartrees): -1870.067661
Coordinates:

P	0.22682	-0.73111	0.38823
O	-0.68876	0.16061	-0.63841
O	-0.79953	-1.47301	1.42093
O	1.19076	0.08312	1.18975
O	0.80963	-1.83214	-0.54756
C	-1.64026	0.92030	-0.00713
C	-1.49398	2.38951	0.00688
C	-2.42110	3.15600	0.58598
H	-2.30051	4.23224	0.58894
C	-3.58820	2.53760	1.24930
C	-4.57539	3.39383	1.94652
H	-4.46514	4.47109	1.93529
C	-5.60553	2.84054	2.59527
H	-6.32561	3.46695	3.10577
C	-5.75600	1.37342	2.62985
H	-6.57874	0.93154	3.17705
C	-4.87264	0.59060	1.99941
H	-4.99021	-0.48281	2.06009
C	-3.73873	1.19094	1.24448
C	-2.74065	0.34068	0.52309
C	-1.94916	-1.96381	0.85097
C	-2.16875	-3.42433	0.85231
C	-3.28904	-3.94180	0.34221
H	-3.43567	-5.01474	0.35026
C	-4.31109	-3.05796	-0.25563
C	-5.51597	-3.64845	-0.88303
H	-5.66672	-4.72075	-0.86865
C	-6.42146	-2.86063	-1.47251
H	-7.30077	-3.29312	-1.93216
C	-6.21764	-1.40017	-1.51130
H	-6.94238	-0.77071	-2.01144
C	-5.13538	-0.85578	-0.94308
H	-4.99582	0.21481	-1.00691
C	-4.13399	-1.71482	-0.25302
C	-2.91814	-1.13241	0.39741
H	2.81227	0.02919	0.54704
C	4.58264	-0.17193	1.56650
C	4.08218	-1.13039	-0.61172
C	4.15239	0.63929	2.63475
C	5.82895	-0.84315	1.62717
C	5.31540	-1.86171	-0.52800
C	4.97968	0.79800	3.73248
H	3.17226	1.10159	2.58543
C	6.65090	-0.65257	2.75880
C	6.15692	-1.70389	0.53397
H	5.57282	-2.51307	-1.35134
C	6.23636	0.16184	3.79613

H	4.65165	1.42033	4.55943
H	7.60970	-1.16165	2.80196
H	7.10415	-2.23657	0.55521
H	6.87107	0.30527	4.66451
C	3.12325	-1.23386	-1.67006
H	1.81881	-1.64960	-0.97812
C	2.60899	-0.00874	-2.46035
H	1.58881	-0.22998	-2.78050
H	3.21740	0.08835	-3.37542
N	2.59604	1.21584	-1.68527
N	3.78261	-0.33183	0.45594
C	1.85110	2.33698	-2.08277
C	0.79571	2.27874	-3.00868
C	2.15783	3.58160	-1.49944
C	0.07172	3.42956	-3.32302
H	0.52709	1.34242	-3.48192
C	1.43198	4.72192	-1.82426
H	2.96845	3.64225	-0.77671
C	0.37854	4.65808	-2.74015
H	-0.74100	3.35707	-4.04065
H	1.69214	5.66750	-1.35657
H	-0.18805	5.54828	-2.99400
H	3.51252	1.47598	-1.34439
Cl	3.48461	-2.53638	-2.87452
H	-0.62877	2.84740	-0.45053
H	-1.41618	-4.07890	1.27329

Catalyst (H, 4)-Enamine protonation TS (pro-R) alternative conformation
Electronic energy (Hartrees): -1870.595427
Free Energy at 1M concentration and 253 K (Hartrees): -1870.059019
Coordinates:

P	-0.01071	1.15328	-0.48668
O	-1.15062	0.90299	-1.64331
O	-0.74943	0.89913	0.96351
O	0.59030	2.51198	-0.48749
O	0.93540	-0.06663	-0.70851
C	-2.37629	1.45215	-1.36215
C	-2.86905	2.56059	-2.20450
C	-4.07671	3.08872	-1.98940
H	-4.42525	3.90122	-2.61482
C	-4.92344	2.58453	-0.88787
C	-6.23247	3.22241	-0.61663
H	-6.58442	4.03076	-1.24550
C	-6.98435	2.80486	0.40740
H	-7.94010	3.27255	0.60573
C	-6.50744	1.71429	1.27890
H	-7.11089	1.39681	2.11955
C	-5.32861	1.12783	1.04014
H	-4.98984	0.34656	1.70699
C	-4.49327	1.55872	-0.11435
C	-3.17472	0.91585	-0.40953
C	-1.52977	-0.22869	1.00345
C	-1.09590	-1.36262	1.84363
C	-1.82859	-2.47688	1.90632
H	-1.49531	-3.30306	2.52224
C	-3.07485	-2.59155	1.11972
C	-3.84629	-3.85559	1.14764
H	-3.51887	-4.67902	1.77018
C	-4.94863	-3.98686	0.40215
H	-5.51084	-4.91157	0.42196

C	-5.39457	-2.87663	-0.46088
H	-6.27462	-2.99837	-1.07921
C	-4.70783	-1.72851	-0.49046
H	-5.04079	-0.93846	-1.14972
C	-3.49656	-1.55345	0.35704
C	-2.70968	-0.28040	0.34484
H	2.41030	-1.28852	0.67067
C	2.73765	-3.29223	0.37890
C	3.49384	-1.45519	-1.03632
C	2.08677	-3.69840	1.55693
C	3.22192	-4.24967	-0.54623
C	3.96668	-2.41973	-1.98032
C	1.92252	-5.04991	1.80648
H	1.72342	-2.95405	2.25863
C	3.04681	-5.62003	-0.25619
C	3.84601	-3.75752	-1.73479
H	4.43505	-2.05077	-2.88208
C	2.40471	-6.01666	0.90224
H	1.41756	-5.36606	2.71372
H	3.42019	-6.35605	-0.96214
H	4.23262	-4.47478	-2.45358
H	2.27027	-7.07129	1.11783
C	3.46104	-0.02840	-1.21026
H	2.05562	0.09477	-1.09970
C	4.01481	0.83822	-0.07051
H	5.10950	0.85738	-0.15854
H	3.79937	0.32557	0.88850
N	3.51387	2.18547	-0.10397
N	2.91227	-1.95535	0.09308
C	3.99648	3.16009	0.76201
C	5.18063	3.01409	1.50962
C	3.27299	4.36695	0.88342
C	5.63309	4.05391	2.32494
H	5.75482	2.09522	1.46112
C	3.73363	5.38741	1.70402
H	2.34356	4.47311	0.33195
C	4.92176	5.24660	2.43096
H	6.55319	3.91817	2.88791
H	3.15602	6.30499	1.78042
H	5.27785	6.04861	3.07015
Cl	4.05574	0.53399	-2.81228
H	-2.24745	2.94415	-3.00360
H	-0.17675	-1.28802	2.41021
H	2.52670	2.29285	-0.33951

Catalyst (H, 4)-Product (S) lowest free energy conformation
Electronic energy (Hartrees): -1870.653123
Free Energy at 1M concentration and 253 K (Hartrees): -1870.112577
Coordinates:

P	0.22274	0.06669	1.24688
O	1.52838	0.45891	2.13775
O	0.74811	-0.27649	-0.27206
O	-0.54344	1.41841	1.01301
O	-0.50672	-1.04770	1.90136
C	2.48387	1.20896	1.49646
C	2.76464	2.56829	2.00125
C	3.71545	3.31790	1.43859
H	3.90942	4.31259	1.82033
C	4.48055	2.80421	0.28401
C	5.48386	3.67150	-0.37568

H	5.68376	4.66323	0.01069
C	6.14551	3.23501	-1.45253
H	6.87752	3.86997	-1.93473
C	5.86921	1.89185	-1.99598
H	6.39050	1.55454	-2.88261
C	4.96850	1.09824	-1.40489
H	4.76521	0.12831	-1.83792
C	4.24726	1.55469	-0.18463
C	3.24132	0.68437	0.50276
C	1.81247	-1.14023	-0.30136
C	1.62115	-2.49377	-0.86033
C	2.64271	-3.35220	-0.91531
H	2.48939	-4.34181	-1.32753
C	3.96990	-2.96298	-0.39344
C	5.07739	-3.94655	-0.39484
H	4.92620	-4.93546	-0.80953
C	6.26789	-3.61900	0.11875
H	7.07605	-4.33898	0.11900
C	6.47983	-2.27951	0.69954
H	7.43902	-2.02911	1.13408
C	5.48774	-1.38150	0.70158
H	5.65952	-0.41434	1.15437
C	4.16598	-1.71867	0.10585
C	3.04070	-0.73263	0.09019
H	-1.50071	1.33376	0.60803
C	-3.02476	1.63575	-1.36524
C	-4.02423	1.05943	0.66483
C	-1.84017	1.87738	-2.10563
C	-4.29318	1.73744	-2.01209
C	-5.31967	1.14139	0.09210
C	-1.92338	2.21103	-3.43958
H	-0.88232	1.78245	-1.60631
C	-4.34165	2.08565	-3.38552
C	-5.44811	1.48170	-1.23022
H	-6.18750	0.94958	0.71181
C	-3.17885	2.31865	-4.08491
H	-1.01503	2.39177	-4.00613
H	-5.30898	2.16206	-3.87406
H	-6.42949	1.55375	-1.69099
H	-3.22022	2.58303	-5.13697
C	-3.82283	0.64011	2.10359
C	-4.17054	-0.84245	2.39683
H	-5.22787	-1.01912	2.19115
H	-4.02855	-0.99628	3.47208
N	-3.34158	-1.75792	1.65618
N	-2.92939	1.31032	-0.03695
C	-3.66643	-2.32320	0.42662
C	-2.61760	-2.79590	-0.39155
C	-4.98848	-2.49966	-0.02458
C	-2.88589	-3.41858	-1.60448
H	-1.59511	-2.65818	-0.05402
C	-5.24134	-3.12139	-1.24748
H	-5.82628	-2.17134	0.58133
C	-4.20041	-3.58657	-2.05021
H	-2.05628	-3.77124	-2.21193
H	-6.27219	-3.24780	-1.56863
H	-4.40687	-4.07111	-2.99929
H	-2.34266	-1.65009	1.82459
H	-2.78862	0.82939	2.38894
Cl	-4.82934	1.70691	3.21335

H	0.64867	-2.78907	-1.23253
H	2.19654	2.95727	2.83676

Catalyst (H, 4)-Product (S) alternative conformation
Electronic energy (Hartrees): -1870.652976
Free Energy at 1M concentration and 253 K (Hartrees): -1870.111181
Coordinates:

P	-0.18620	-0.36812	-1.51147
O	0.20046	-0.23041	0.07886
O	1.15217	-0.81642	-2.31621
O	-0.73844	0.86971	-2.11464
O	-1.11152	-1.64385	-1.48758
C	1.29874	0.55908	0.30018
C	1.12686	1.84495	1.00387
C	2.17537	2.64289	1.21943
H	2.03485	3.58489	1.73491
C	3.51806	2.24845	0.74333
C	4.66259	3.17053	0.92497
H	4.52259	4.11842	1.42966
C	5.87320	2.83773	0.46427
H	6.70890	3.51278	0.59615
C	6.07141	1.55450	-0.23659
H	7.04939	1.30118	-0.62511
C	5.04508	0.71197	-0.40255
H	5.20872	-0.21326	-0.93865
C	3.69801	1.05371	0.12939
C	2.53361	0.13013	-0.04124
C	2.00919	-1.66113	-1.65353
C	2.27171	-2.98935	-2.24394
C	3.13646	-3.82913	-1.67046
H	3.31711	-4.79934	-2.11642
C	3.82294	-3.44870	-0.41950
C	4.72426	-4.41771	0.24603
H	4.91033	-5.38518	-0.20359
C	5.30973	-4.10385	1.40637
H	5.96661	-4.81328	1.89295
C	5.04998	-2.79526	2.03517
H	5.50697	-2.55781	2.98725
C	4.24178	-1.91038	1.43990
H	4.04648	-0.96932	1.93552
C	3.60810	-2.22929	0.13020
C	2.70478	-1.25521	-0.56209
H	-2.02367	-1.49448	-1.03826
C	-3.76722	-2.55215	0.47147
C	-4.20562	-0.33343	-0.10241
C	-2.93709	-3.69582	0.34783
C	-4.92928	-2.61598	1.29961
C	-5.37417	-0.31766	0.70227
C	-3.25742	-4.85295	1.02284
H	-2.05540	-3.63566	-0.28054
C	-5.22961	-3.82402	1.97958
C	-5.72704	-1.44829	1.39530
H	-5.97636	0.58188	0.74307
C	-4.40945	-4.92095	1.84338
H	-2.61928	-5.72592	0.92569
H	-6.11555	-3.86675	2.60700
H	-6.61755	-1.46107	2.01805
H	-4.64255	-5.84365	2.36555
C	-3.73089	0.90635	-0.83478
C	-3.02635	1.89091	0.11057

H	-2.36673	1.29204	0.76528
H	-3.77613	2.36330	0.75330
N	-2.29806	2.89120	-0.63996
N	-3.43685	-1.40822	-0.20924
C	-1.59903	3.90254	0.01980
C	-0.54317	4.54587	-0.66010
C	-1.89790	4.32211	1.32915
C	0.17841	5.56584	-0.05266
H	-0.29536	4.22374	-1.66810
C	-1.16709	5.35081	1.92559
H	-2.69512	3.84689	1.89025
C	-0.12470	5.98058	1.24815
H	0.99039	6.03822	-0.59916
H	-1.41760	5.65453	2.93869
H	0.44262	6.77572	1.72145
H	-1.74490	2.45720	-1.37599
H	-3.04149	0.61289	-1.62558
Cl	-5.12435	1.71481	-1.69751
H	1.76028	-3.28179	-3.15224
H	0.14696	2.13469	1.35677

Catalyst (H, 4)-Product (S) alternative conformation
Electronic energy (Hartrees): -1870.652981
Free Energy at 1M concentration and 253 K (Hartrees): -1870.111173
Coordinates:

P	0.18533	-0.38974	1.48877
O	-1.14644	-0.84818	2.29848
O	-0.20960	-0.24935	-0.09886
O	1.11937	-1.65881	1.45671
O	0.73204	0.84945	2.09434
C	-2.00950	-1.68651	1.63553
C	-2.27322	-3.01640	2.22157
C	-3.14684	-3.84923	1.65165
H	-3.32839	-4.82059	2.09467
C	-3.84202	-3.45954	0.40850
C	-4.75456	-4.42062	-0.25308
H	-4.94210	-5.38892	0.19414
C	-5.34882	-4.09831	-1.40657
H	-6.01428	-4.80180	-1.89008
C	-5.08729	-2.78849	-2.03206
H	-5.55146	-2.54427	-2.97889
C	-4.26875	-1.91081	-1.44033
H	-4.07239	-0.96879	-1.93370
C	-3.62555	-2.23882	-0.13759
C	-2.71222	-1.27206	0.55173
C	-1.30750	0.54287	-0.31156
C	-1.13685	1.83138	-1.01073
C	-2.18484	2.63276	-1.21599
H	-2.04552	3.57649	-1.72863
C	-3.52511	2.24011	-0.73162
C	-4.66822	3.16638	-0.90046
H	-4.52929	4.11592	-1.40233
C	-5.87613	2.83546	-0.43138
H	-6.71075	3.51365	-0.55378
C	-6.07271	1.55004	0.26592
H	-7.04832	1.29820	0.66135
C	-5.04769	0.70371	0.42028
H	-5.20988	-0.22308	0.95412
C	-3.70388	1.04341	-0.12110
C	-2.54099	0.11563	0.03675

H	2.03510	-1.49726	1.01960
C	3.81085	-2.53293	-0.47094
C	4.22009	-0.31164	0.11413
C	2.99052	-3.68510	-0.36150
C	4.98475	-2.58354	-1.28326
C	5.39872	-0.28240	-0.67527
C	3.33165	-4.83746	-1.03450
H	2.09962	-3.63542	0.25469
C	5.30655	-3.78704	-1.96144
C	5.77205	-1.40784	-1.36591
H	5.99233	0.62319	-0.70603
C	4.49562	-4.89238	-1.83900
H	2.70084	-5.71688	-0.94809
H	6.20153	-3.81949	-2.57653
H	6.67094	-1.41029	-1.97667
H	4.74517	-5.81157	-2.35974
C	3.72506	0.92131	0.84446
C	3.01988	1.90124	-0.10531
H	2.37167	1.29757	-0.76708
H	3.77084	2.38187	-0.74054
N	2.27606	2.89317	0.64079
N	3.46009	-1.39399	0.20809
C	1.57566	3.90165	-0.02151
C	0.51215	4.53797	0.65311
C	1.88049	4.32564	-1.32806
C	-0.21085	5.55559	0.04345
H	0.25990	4.21255	1.65896
C	1.14823	5.35203	-1.92670
H	2.68368	3.85580	-1.88517
C	0.09841	5.97495	-1.25444
H	-1.02873	6.02249	0.58585
H	1.40360	5.65940	-2.93749
H	-0.46991	6.76834	-1.72948
H	1.72064	2.45272	1.37117
H	3.03078	0.61876	1.62744
Cl	5.10114	1.74219	1.72334
H	-0.15830	2.12050	-1.36830
H	-1.75584	-3.31545	3.12434

Catalyst (H, 4)-Product (S) alternative conformation
Electronic energy (Hartrees): -1870.65487
Free Energy at 1M concentration and 253 K (Hartrees): -1870.111152
Coordinates:

P	0.73170	-2.73334	1.98113
O	-0.16910	-1.34886	2.02996
O	0.55408	-3.34506	0.46659
O	0.39172	-3.76384	2.97273
O	2.17925	-2.09471	2.09557
C	-1.40257	-1.46377	1.44484
C	-2.60214	-1.41143	2.30415
C	-3.82253	-1.44483	1.76488
H	-4.69341	-1.39927	2.40681
C	-3.98472	-1.54785	0.30027
C	-5.34449	-1.60989	-0.28305
H	-6.21488	-1.54063	0.35754
C	-5.50374	-1.75047	-1.60295
H	-6.49790	-1.79554	-2.02848
C	-4.32470	-1.86209	-2.48193
H	-4.46106	-2.00541	-3.54609
C	-3.09155	-1.80252	-1.96594

H	-2.24509	-1.91634	-2.62883
C	-2.89494	-1.60345	-0.50337
C	-1.52762	-1.50161	0.09961
C	0.64748	-2.43387	-0.55715
C	1.74043	-2.58716	-1.53806
C	1.86825	-1.72601	-2.55040
H	2.67981	-1.84586	-3.25719
C	0.93715	-0.58711	-2.67665
C	1.16980	0.43950	-3.71821
H	2.00288	0.34047	-4.40292
C	0.36308	1.50212	-3.80573
H	0.53881	2.25680	-4.56126
C	-0.76838	1.65025	-2.87123
H	-1.41884	2.51160	-2.94892
C	-0.99196	0.72328	-1.93293
H	-1.82702	0.85061	-1.26086
C	-0.10398	-0.46389	-1.81943
C	-0.30456	-1.49082	-0.74950
H	2.29786	-1.15107	1.76495
C	3.50931	0.13166	-0.13403
C	2.03882	1.48075	1.06640
C	4.27639	-1.06134	-0.20491
C	3.63579	1.10542	-1.17318
C	2.11212	2.49575	0.07791
C	5.12073	-1.27296	-1.27206
H	4.17709	-1.79272	0.58944
C	4.50173	0.84706	-2.26636
C	2.88250	2.29634	-1.04032
H	1.57141	3.42333	0.21102
C	5.23126	-0.31876	-2.31328
H	5.70745	-2.18501	-1.31937
H	4.58298	1.58687	-3.05776
H	2.93887	3.05422	-1.81694
H	5.89702	-0.51125	-3.14867
C	1.32059	1.69942	2.38195
C	0.07101	2.59123	2.37871
H	0.28340	3.54647	1.89824
H	-0.17331	2.82387	3.42360
N	-1.03279	1.92647	1.70359
N	2.68208	0.32863	0.93914
C	-1.97755	2.60302	0.91785
C	-3.30640	2.14140	0.90236
C	-1.64044	3.67837	0.07818
C	-4.25253	2.70699	0.05396
H	-3.59558	1.33426	1.56927
C	-2.60034	4.25097	-0.75483
H	-0.62507	4.05728	0.05067
C	-3.90883	3.76873	-0.78455
H	-5.26754	2.32006	0.05965
H	-2.31232	5.07803	-1.39770
H	-4.64668	4.21426	-1.44357
H	-1.46197	1.23251	2.30052
H	1.09237	0.72959	2.82328
Cl	2.53593	2.47762	3.53272
H	2.44613	-3.40116	-1.43183
H	-2.48920	-1.33587	3.37809

Catalyst (H, 4)-Product (S) alternative conformation
Electronic energy (Hartrees): -1870.652411
Free Energy at 1M concentration and 253 K (Hartrees): -1870.110088

Coordinates:

P	0.80072	1.45833	-2.25509
O	-0.21720	2.68398	-1.91466
O	0.21691	0.11731	-1.49342
O	2.06696	1.73082	-1.33307
O	0.98466	1.32106	-3.70987
C	-0.67064	2.72709	-0.61891
C	-0.27673	3.87512	0.22208
C	-0.72638	3.98506	1.47366
H	-0.42800	4.83312	2.07744
C	-1.60978	2.94591	2.03969
C	-2.06132	3.05649	3.44605
H	-1.76567	3.90740	4.04715
C	-2.83731	2.10771	3.97940
H	-3.16785	2.18971	5.00676
C	-3.23394	0.93870	3.17351
H	-3.84775	0.16605	3.61782
C	-2.82519	0.82465	1.90450
H	-3.10493	-0.05763	1.34609
C	-1.99253	1.89020	1.28120
C	-1.54680	1.80624	-0.14727
C	-1.12832	-0.08636	-1.65630
C	-1.58085	-1.27170	-2.41128
C	-2.88358	-1.53067	-2.54581
H	-3.20153	-2.40892	-3.09372
C	-3.88677	-0.61103	-1.97051
C	-5.32910	-0.87370	-2.18006
H	-5.64852	-1.75874	-2.71613
C	-6.24318	-0.01863	-1.70944
H	-7.29708	-0.21091	-1.86367
C	-5.81770	1.19832	-0.99226
H	-6.56195	1.89943	-0.63715
C	-4.51729	1.44254	-0.79290
H	-4.22818	2.35353	-0.28641
C	-3.48810	0.48158	-1.27555
C	-2.02819	0.71615	-1.04344
H	2.58212	0.91544	-1.04137
C	4.85333	0.40447	0.10662
C	3.50256	-1.48728	-0.01637
C	5.06585	1.77113	-0.21338
C	5.85226	-0.30207	0.84690
C	4.44239	-2.25701	0.71684
C	6.22338	2.39920	0.18994
H	4.30652	2.30209	-0.77594
C	7.03213	0.37672	1.24604
C	5.60399	-1.66449	1.14431
H	4.23678	-3.30228	0.91382
C	7.21363	1.70213	0.92383
H	6.38060	3.44413	-0.05917
H	7.78635	-0.16810	1.80707
H	6.34122	-2.23103	1.70690
H	8.11791	2.21861	1.23039
C	2.17884	-2.07896	-0.45698
C	1.18124	-2.17888	0.69861
H	1.09512	-1.16491	1.10934
H	1.59456	-2.82304	1.49347
N	-0.11970	-2.60476	0.23382
N	3.69674	-0.20875	-0.30515
C	-1.20319	-2.71084	1.10885
C	-2.40915	-3.26183	0.63181

C	-1.15427	-2.25598	2.43758
C	-3.52428	-3.34911	1.45543
H	-2.46079	-3.61343	-0.39570
C	-2.28097	-2.35550	3.25607
H	-0.24148	-1.83202	2.84079
C	-3.47241	-2.89562	2.77734
H	-4.44294	-3.77396	1.06064
H	-2.21886	-2.00045	4.28112
H	-4.34494	-2.96371	3.41886
H	-0.08025	-3.33699	-0.46402
H	1.74554	-1.47512	-1.24957
Cl	2.43131	-3.73908	-1.21138
H	-0.84971	-1.94248	-2.84435
H	0.38092	4.63420	-0.18205

Catalyst (H, 4)-Product (S) alternative conformation
Electronic energy (Hartrees): -1870.646636
Free Energy at 1M concentration and 253 K (Hartrees): -1870.106684
Coordinates:

P	-0.24423	-0.39096	-1.03422
O	-1.22552	0.82450	-1.53088
O	-1.09634	-1.30747	0.01660
O	0.78777	0.22054	0.00212
O	0.32278	-1.08240	-2.21236
C	-1.98819	1.39734	-0.54289
C	-1.74955	2.81398	-0.19986
C	-2.48411	3.41699	0.73788
H	-2.29689	4.45597	0.97917
C	-3.53056	2.66416	1.46006
C	-4.29108	3.32304	2.54698
H	-4.10735	4.36307	2.78642
C	-5.20584	2.63604	3.23931
H	-5.75999	3.11785	4.03448
C	-5.45276	1.21471	2.93116
H	-6.17746	0.65827	3.51148
C	-4.77501	0.61103	1.94791
H	-4.95580	-0.43820	1.75777
C	-3.77664	1.36959	1.14508
C	-3.00863	0.72142	0.03572
C	-2.36689	-1.60641	-0.40425
C	-2.69939	-3.00929	-0.72431
C	-3.93657	-3.34199	-1.10134
H	-4.16679	-4.37460	-1.33258
C	-4.97985	-2.30280	-1.22824
C	-6.33012	-2.67275	-1.71180
H	-6.56350	-3.70626	-1.93573
C	-7.26627	-1.73211	-1.87623
H	-8.25234	-2.00318	-2.23082
C	-6.95396	-0.31937	-1.58838
H	-7.70921	0.43973	-1.74625
C	-5.73883	0.02535	-1.14666
H	-5.52631	1.07035	-0.96617
C	-4.69501	-1.01302	-0.92645
C	-3.33079	-0.65749	-0.42437
H	1.66845	0.56724	-0.42680
C	3.38769	2.39310	-0.54538
C	4.03005	0.29802	-1.35095
C	2.36431	3.22940	-0.03207
C	4.70609	2.91474	-0.70820
C	5.36608	0.73966	-1.53156

C	2.64569	4.53651	0.29673
H	1.37167	2.81350	0.09700
C	4.96159	4.26613	-0.36099
C	5.69529	2.03363	-1.21250
H	6.10222	0.05404	-1.93263
C	3.95080	5.06099	0.13065
H	1.85969	5.17429	0.68948
H	5.96597	4.66013	-0.48863
H	6.71203	2.39389	-1.34469
H	4.15088	6.09441	0.39584
C	3.60527	-1.13048	-1.64006
C	3.93633	-2.10411	-0.49340
H	5.01910	-2.08913	-0.32443
H	3.67189	-3.12134	-0.82234
N	3.25644	-1.69317	0.71706
N	3.08772	1.09856	-0.87534
C	3.46943	-2.33648	1.93605
C	2.59415	-2.06057	3.00660
C	4.53675	-3.22474	2.15655
C	2.79001	-2.64588	4.25101
H	1.76233	-1.37961	2.84429
C	4.72462	-3.79969	3.41496
H	5.21832	-3.47720	1.35174
C	3.86009	-3.51947	4.47098
H	2.09914	-2.41756	5.05817
H	5.55713	-4.48279	3.56119
H	4.01071	-3.97455	5.44476
H	2.30416	-1.37298	0.58696
H	2.53664	-1.15398	-1.86793
Cl	4.41040	-1.75392	-3.15548
H	-1.93511	-3.77235	-0.64855
H	-0.97659	3.36806	-0.71692

Catalyst (H, 4)-Product (S) alternative conformation
Electronic energy (Hartrees): -1870.647109
Free Energy at 1M concentration and 253 K (Hartrees): -1870.105481
Coordinates:

P	0.08374	-0.25539	1.36129
O	-1.23070	-0.63803	2.24506
O	-0.39503	-0.11788	-0.20202
O	0.93819	-1.58264	1.29317
O	0.73706	0.94711	1.92737
C	-2.15580	-1.45471	1.64133
C	-2.43643	-2.76561	2.26130
C	-3.36426	-3.57584	1.74695
H	-3.55757	-4.53361	2.21394
C	-4.10384	-3.18091	0.53122
C	-5.07756	-4.12006	-0.07214
H	-5.27627	-5.07474	0.39892
C	-5.71320	-3.79500	-1.20260
H	-6.42312	-4.48268	-1.64379
C	-5.43730	-2.50427	-1.86082
H	-5.93606	-2.25862	-2.78952
C	-4.56367	-1.64618	-1.32144
H	-4.35889	-0.71883	-1.83862
C	-3.87237	-1.97648	-0.04421
C	-2.89537	-1.03195	0.58597
C	-1.46761	0.71885	-0.36520
C	-1.27976	1.99510	-1.08233
C	-2.30747	2.82995	-1.25529

H	-2.15645	3.76393	-1.78218
C	-3.64005	2.48922	-0.71345
C	-4.75697	3.45224	-0.84923
H	-4.60663	4.38960	-1.37031
C	-5.95523	3.16950	-0.32692
H	-6.77068	3.87439	-0.42549
C	-6.16659	1.90115	0.39675
H	-7.13310	1.68851	0.83523
C	-5.16552	1.02197	0.52173
H	-5.33704	0.10874	1.07556
C	-3.83446	1.30746	-0.07932
C	-2.69920	0.34097	0.04164
H	1.87024	-1.43846	0.89903
C	3.58769	-2.39456	-0.72485
C	4.25499	-0.41898	0.32017
C	2.58881	-3.38246	-0.92382
C	4.82921	-2.50510	-1.42280
C	5.51971	-0.46830	-0.32749
C	2.82319	-4.43324	-1.78307
H	1.64891	-3.28752	-0.39149
C	5.03727	-3.59982	-2.29963
C	5.79961	-1.49848	-1.18823
H	6.25685	0.29838	-0.11784
C	4.05272	-4.54577	-2.47601
H	2.05552	-5.18625	-1.93307
H	5.98424	-3.67820	-2.82658
H	6.76102	-1.55670	-1.69179
H	4.21553	-5.38273	-3.14796
C	3.91295	0.71296	1.26357
C	4.17482	2.12112	0.69720
H	5.25205	2.26022	0.54208
H	3.87262	2.84994	1.45303
N	3.44930	2.35304	-0.54013
N	3.33667	-1.35435	0.13235
C	2.45832	3.33742	-0.66648
C	2.29450	3.99530	-1.89875
C	1.59599	3.66576	0.39423
C	1.30556	4.96174	-2.06290
H	2.95358	3.74746	-2.72847
C	0.62229	4.64830	0.22019
H	1.63563	3.11098	1.32388
C	0.46736	5.30645	-1.00106
H	1.20024	5.45586	-3.02495
H	-0.03762	4.88266	1.05091
H	-0.29954	6.06432	-1.12707
H	4.01561	2.25269	-1.37004
H	2.87547	0.63558	1.58782
Cl	4.92583	0.52588	2.79079
H	-0.30502	2.24936	-1.47671
H	-1.88680	-3.06908	3.14332

Catalyst (H, 4)-Product (S) alternative conformation
Electronic energy (Hartrees): -1870.643471
Free Energy at 1M concentration and 253 K (Hartrees): -1870.104089
Coordinates:

P	0.50261	-0.80480	0.17423
O	1.49080	-0.00102	1.20926
O	1.43982	-1.42758	-1.00906
O	-0.25499	0.28239	-0.69303
O	-0.32583	-1.76090	0.94144

C	2.43732	0.78359	0.59890
C	2.34766	2.24978	0.75060
C	3.25367	3.04559	0.17767
H	3.17301	4.11930	0.29351
C	4.34553	2.46583	-0.63242
C	5.30536	3.36124	-1.31860
H	5.23018	4.43472	-1.19715
C	6.26686	2.84849	-2.09368
H	6.96664	3.50369	-2.59622
C	6.36829	1.38841	-2.27810
H	7.13440	0.98169	-2.92564
C	5.50944	0.56995	-1.65920
H	5.58878	-0.49507	-1.82992
C	4.45289	1.12169	-0.76729
C	3.48054	0.22978	-0.06078
C	2.60148	-2.00796	-0.56551
C	2.77359	-3.46573	-0.72877
C	3.90712	-4.06048	-0.34826
H	4.01952	-5.13016	-0.47451
C	4.99276	-3.26786	0.26572
C	6.21749	-3.94844	0.74610
H	6.33321	-5.01713	0.61445
C	7.18471	-3.24699	1.34660
H	8.07873	-3.74535	1.69851
C	7.03030	-1.79326	1.54418
H	7.80683	-1.23561	2.05174
C	5.93002	-1.16758	1.11004
H	5.82916	-0.10561	1.28832
C	4.85793	-1.92752	0.41032
C	3.62090	-1.25231	-0.09344
H	-1.06116	0.69798	-0.21348
C	-2.22306	2.88835	0.30626
C	-3.35958	0.97484	1.02012
C	-1.08296	3.46067	-0.31572
C	-3.26340	3.74223	0.78280
C	-4.44510	1.75463	1.49379
C	-0.98301	4.82814	-0.44260
H	-0.30669	2.80082	-0.68685
C	-3.12781	5.14702	0.63562
C	-4.38994	3.12199	1.37736
H	-5.29401	1.25928	1.94528
C	-2.00854	5.67928	0.03720
H	-0.10796	5.26140	-0.91759
H	-3.92148	5.79178	1.00285
H	-5.20898	3.73887	1.73771
H	-1.90842	6.75464	-0.07224
C	-3.34184	-0.54457	1.07205
C	-3.74214	-1.17250	-0.27964
H	-3.73217	-2.25936	-0.17357
H	-2.93163	-0.91954	-0.97542
N	-5.01586	-0.72075	-0.80955
N	-2.30139	1.52716	0.44022
C	-6.09478	-1.57197	-1.07152
C	-7.00341	-1.22860	-2.09052
C	-6.33266	-2.74349	-0.33125
C	-8.10781	-2.03030	-2.36149
H	-6.83280	-0.32644	-2.67454
C	-7.43564	-3.54508	-0.62251
H	-5.67593	-3.01224	0.48803
C	-8.33233	-3.20123	-1.63455

H	-8.79136	-1.74080	-3.15504
H	-7.59738	-4.44634	-0.03748
H	-9.19044	-3.82974	-1.85021
H	-4.93038	0.02253	-1.48782
H	-2.33374	-0.89304	1.31030
Cl	-4.38989	-1.18038	2.42225
H	1.97595	-4.05441	-1.16387
H	1.54016	2.68081	1.32888

Catalyst (H, 4)-Product (S) alternative conformation
Electronic energy (Hartrees): -1870.643105
Free Energy at 1M concentration and 253 K (Hartrees): -1870.103401
Coordinates:

P	-0.44066	-0.78524	-0.52399
O	-1.43474	0.26035	-1.30595
O	-1.33152	-1.55030	0.60897
O	0.46940	0.07064	0.45057
O	0.24733	-1.63575	-1.52053
C	-2.26120	0.98309	-0.48216
C	-2.06258	2.44309	-0.38296
C	-2.85114	3.18110	0.40186
H	-2.69142	4.25016	0.46810
C	-3.91952	2.53900	1.19607
C	-4.74394	3.35753	2.11479
H	-4.58977	4.42765	2.17665
C	-5.68001	2.77857	2.87425
H	-6.28074	3.37726	3.54668
C	-5.88750	1.31950	2.80869
H	-6.63083	0.85452	3.44340
C	-5.15173	0.57108	1.97864
H	-5.30542	-0.49943	1.96663
C	-4.12738	1.20317	1.10265
C	-3.29225	0.39096	0.16320
C	-2.56948	-1.95670	0.17910
C	-2.85058	-3.40411	0.09411
C	-4.05660	-3.83881	-0.27984
H	-4.24830	-4.90308	-0.33769
C	-5.11658	-2.87351	-0.63834
C	-6.42852	-3.36769	-1.11652
H	-6.62328	-4.43174	-1.16774
C	-7.37740	-2.50244	-1.48949
H	-8.33528	-2.86447	-1.84011
C	-7.11684	-1.05140	-1.43782
H	-7.88108	-0.35745	-1.76340
C	-5.93684	-0.59393	-1.00313
H	-5.76111	0.47321	-0.99772
C	-4.88083	-1.54205	-0.55311
C	-3.55512	-1.05839	-0.05431
H	1.28130	0.47766	-0.01871
C	2.54285	2.64339	-0.44624
C	3.46573	0.75849	-1.47433
C	1.53568	3.20107	0.38449
C	3.54444	3.49788	-1.00053
C	4.49918	1.53913	-2.05204
C	1.52621	4.55390	0.63850
H	0.78770	2.54145	0.81005
C	3.50490	4.88816	-0.71714
C	4.53187	2.89322	-1.81423
H	5.23578	1.06151	-2.68474
C	2.51393	5.40574	0.08458

H	0.75311	4.97578	1.27356
H	4.26840	5.53257	-1.14394
H	5.31304	3.50938	-2.25172
H	2.48603	6.46954	0.29863
C	3.34619	-0.75240	-1.65107
C	3.71719	-1.53024	-0.38281
H	3.55489	-2.59304	-0.58622
H	2.98593	-1.23483	0.38800
N	5.08694	-1.31319	0.03065
N	2.53186	1.29898	-0.70107
C	5.60376	-1.89508	1.18571
C	6.84174	-1.44455	1.68671
C	4.95135	-2.94311	1.86025
C	7.40528	-2.02517	2.81642
H	7.35950	-0.63745	1.17305
C	5.53296	-3.52110	2.98940
H	3.99231	-3.30843	1.51079
C	6.75796	-3.07206	3.47960
H	8.36035	-1.65712	3.18141
H	5.01044	-4.33011	3.49265
H	7.20052	-3.52484	4.36107
H	5.44902	-0.38940	-0.16177
H	2.30752	-1.00014	-1.88583
Cl	4.31117	-1.35014	-3.07827
H	-2.07345	-4.11650	0.34036
H	-1.27143	2.91658	-0.95044

Catalyst (H, 4)-Product (R) lowest free energy conformation
Electronic energy (Hartrees): -1870.654723
Free Energy at 1M concentration and 253 K (Hartrees): -1870.113225
Coordinates:

P	-0.25226	-1.20698	1.08856
O	-1.60540	-1.21932	1.99503
O	-0.63641	-0.54527	-0.36527
O	0.39033	-2.51819	0.82402
O	0.56025	-0.13713	1.90973
C	-2.74924	-1.57153	1.32356
C	-3.44299	-2.81436	1.71786
C	-4.58690	-3.16881	1.12751
H	-5.08421	-4.08267	1.42809
C	-5.15792	-2.33306	0.05132
C	-6.39006	-2.77253	-0.64345
H	-6.89194	-3.68219	-0.33780
C	-6.88700	-2.04953	-1.65253
H	-7.78670	-2.37212	-2.16047
C	-6.20223	-0.81727	-2.08668
H	-6.59617	-0.25023	-2.92028
C	-5.09133	-0.40842	-1.46275
H	-4.59361	0.48417	-1.81656
C	-4.54313	-1.18267	-0.31501
C	-3.30788	-0.74337	0.40793
C	-1.37840	0.60484	-0.28559
C	-0.76931	1.87414	-0.72979
C	-1.46141	3.01394	-0.66574
H	-1.00071	3.93914	-0.98910
C	-2.84029	3.01661	-0.13274
C	-3.57552	4.29594	-0.00477
H	-3.11848	5.22118	-0.33311
C	-4.80579	4.31463	0.51906
H	-5.34177	5.25022	0.61349

C	-5.43158	3.06173	0.98288
H	-6.41838	3.08762	1.42690
C	-4.77829	1.89958	0.86782
H	-5.24776	0.99774	1.23684
C	-3.42118	1.85607	0.25800
C	-2.66950	0.57025	0.11362
H	1.54881	-0.00981	1.62688
C	3.20639	1.74152	0.94181
C	4.01228	-0.44939	0.97574
C	2.13745	2.64312	1.17695
C	4.45524	2.24340	0.46700
C	5.28830	-0.02287	0.52262
C	2.30492	3.98775	0.92934
H	1.19935	2.25164	1.55422
C	4.59428	3.63350	0.22459
C	5.50199	1.30736	0.26839
H	6.07748	-0.75412	0.39429
C	3.53814	4.48834	0.44752
H	1.48226	4.67372	1.10729
H	5.54596	4.00987	-0.13999
H	6.46815	1.65608	-0.08589
H	3.64858	5.55137	0.25743
C	3.71653	-1.91204	1.22227
H	2.74256	-2.01297	1.69894
C	3.74738	-2.80517	-0.04282
H	3.56385	-3.83258	0.28856
H	4.74543	-2.78927	-0.48380
N	2.75242	-2.41810	-1.01009
N	3.02020	0.40354	1.18090
C	2.96223	-1.59823	-2.11294
C	4.23158	-1.38013	-2.68380
C	1.84750	-0.98660	-2.72682
C	4.37165	-0.57871	-3.81661
H	5.11419	-1.84638	-2.25956
C	2.00372	-0.19460	-3.85824
H	0.86175	-1.13420	-2.29659
C	3.26653	0.02317	-4.41674
H	5.36391	-0.43263	-4.23588
H	1.12528	0.26330	-4.30596
H	3.38417	0.64419	-5.29910
H	1.79787	-2.49313	-0.66884
Cl	4.92443	-2.58417	2.43691
H	-3.02209	-3.43938	2.49523
H	0.24555	1.87867	-1.10750

Catalyst (H, 4)-Product (R) alternative conformation
Electronic energy (Hartrees): -1870.65455
Free Energy at 1M concentration and 253 K (Hartrees): -1870.113201
Coordinates:

P	0.06753	-0.47734	-1.59893
O	1.45992	-0.27669	-2.41838
O	0.41124	-0.37615	0.00414
O	-0.65755	-1.75390	-1.81786
O	-0.64809	0.85894	-2.02881
C	2.53727	-0.93887	-1.88558
C	3.15518	-2.02797	-2.66868
C	4.23069	-2.66959	-2.20547
H	4.67287	-3.46607	-2.79102
C	4.79892	-2.31353	-0.88883
C	5.94828	-3.07969	-0.35403

H	6.39494	-3.87186	-0.94202
C	6.43767	-2.79884	0.85826
H	7.27643	-3.36038	1.24898
C	5.82663	-1.73210	1.67349
H	6.21250	-1.52630	2.66362
C	4.79131	-1.03028	1.19801
H	4.34481	-0.27059	1.82502
C	4.25455	-1.30744	-0.16290
C	3.10180	-0.53377	-0.72267
C	1.24201	0.66071	0.34451
C	0.70762	1.75030	1.18529
C	1.48544	2.77697	1.53630
H	1.07838	3.57272	2.14786
C	2.88777	2.83839	1.07326
C	3.72284	4.01305	1.41447
H	3.31850	4.80784	2.02894
C	4.97878	4.09866	0.96331
H	5.58786	4.95813	1.21201
C	5.53428	3.02820	0.11359
H	6.54402	3.11496	-0.26654
C	4.79015	1.96037	-0.19730
H	5.21112	1.20006	-0.84120
C	3.40092	1.83387	0.32203
C	2.54767	0.64938	-0.00724
H	-1.60723	0.96645	-1.66034
C	-2.98247	2.48520	-0.22231
C	-4.00158	0.46096	-0.78042
C	-1.89583	3.38185	-0.38854
C	-4.04553	2.83157	0.66487
C	-5.10616	0.74672	0.06556
C	-1.86639	4.56527	0.31605
H	-1.09652	3.11257	-1.07004
C	-3.98465	4.05841	1.37291
C	-5.12025	1.91366	0.78437
H	-5.92716	0.04223	0.12698
C	-2.91380	4.90737	1.20441
H	-1.02981	5.24559	0.18865
H	-4.79575	4.31488	2.04870
H	-5.95039	2.14800	1.44520
H	-2.87026	5.84438	1.75070
C	-3.94582	-0.83212	-1.56714
H	-3.04506	-0.85242	-2.17999
C	-4.00779	-2.09491	-0.69226
H	-4.09966	-2.97250	-1.35016
H	-4.91220	-2.05634	-0.08001
N	-2.83902	-2.11607	0.15677
N	-2.98862	1.30204	-0.91742
C	-2.78074	-2.81571	1.36193
C	-3.89310	-3.43118	1.96351
C	-1.53910	-2.88249	2.03019
C	-3.76381	-4.07473	3.19610
H	-4.85935	-3.42980	1.47093
C	-1.42406	-3.53594	3.25031
H	-0.67588	-2.40124	1.57874
C	-2.53589	-4.13647	3.85092
H	-4.63924	-4.54353	3.63799
H	-0.45509	-3.57318	3.74142
H	-2.44263	-4.64424	4.80574
H	-1.96728	-2.10767	-0.37094
Cl	-5.34472	-0.86903	-2.75679

H	2.73417	-2.30458	-3.62694
H	-0.32224	1.71532	1.51743

Catalyst (H, 4)-Product (R) alternative conformation
Electronic energy (Hartrees): -1870.654552
Free Energy at 1M concentration and 253 K (Hartrees): -1870.113176
Coordinates:

P	0.06359	-0.47958	-1.61422
O	0.40261	-0.37980	-0.01001
O	1.45654	-0.26624	-2.42937
O	-0.66141	0.85222	-2.04220
O	-0.65141	-1.76108	-1.83725
C	1.22641	0.66086	0.33538
C	0.68351	1.74439	1.17828
C	1.45484	2.77361	1.53587
H	1.04157	3.56482	2.14903
C	2.85838	2.84402	1.07776
C	3.68631	4.02147	1.42653
H	3.27588	4.81164	2.04294
C	4.94328	4.11533	0.97990
H	5.54714	4.97683	1.23414
C	5.50703	3.05122	0.12763
H	6.51755	3.14465	-0.24879
C	4.76937	1.98093	-0.19013
H	5.19634	1.22545	-0.83576
C	3.37912	1.84520	0.32416
C	2.53314	0.65763	-0.01241
C	2.53660	-0.92391	-1.89644
C	3.16293	-2.00649	-2.68200
C	4.24107	-2.64347	-2.21845
H	4.68939	-3.43527	-2.80574
C	4.80389	-2.28892	-0.89909
C	5.95702	-3.04963	-0.36455
H	6.41031	-3.83644	-0.95464
C	6.44186	-2.77018	0.84991
H	7.28357	-3.32752	1.24027
C	5.82177	-1.71086	1.66796
H	6.20387	-1.50649	2.65985
C	4.78291	-1.01405	1.19280
H	4.32972	-0.26001	1.82185
C	4.25147	-1.28902	-0.17065
C	3.09573	-0.51987	-0.73057
H	-1.62006	0.95284	-1.67073
C	-3.00014	2.46769	-0.23348
C	-4.00736	0.43327	-0.77588
C	-1.92008	3.37063	-0.40855
C	-4.06225	2.81195	0.65569
C	-5.11052	0.71625	0.07288
C	-1.89629	4.55834	0.28902
H	-1.12121	3.10284	-1.09112
C	-4.00732	4.04334	1.35629
C	-5.13012	1.88739	0.78471
H	-5.92632	0.00641	0.14152
C	-2.94295	4.89861	1.17896
H	-1.06480	5.24356	0.15471
H	-4.81776	4.29819	2.03346
H	-5.95940	2.11992	1.44726
H	-2.90395	5.83915	1.71950
C	-3.94562	-0.86478	-1.55399
H	-3.04997	-0.88103	-2.17442

C	-3.98776	-2.12174	-0.66914
H	-4.08012	-3.00484	-1.31955
H	-4.88540	-2.08568	-0.04685
N	-2.80934	-2.12760	0.16653
N	-3.00091	1.28061	-0.92201
C	-2.73251	-2.81261	1.37907
C	-3.83318	-3.43019	1.99963
C	-1.48330	-2.86126	2.03468
C	-3.68538	-4.05813	3.23820
H	-4.80454	-3.44261	1.51731
C	-1.34983	-3.49939	3.26101
H	-0.62923	-2.37790	1.56854
C	-2.45011	-4.10198	3.88050
H	-4.55207	-4.52899	3.69488
H	-0.37541	-3.52278	3.74213
H	-2.34245	-4.59771	4.84013
H	-1.94374	-2.11860	-0.37100
Cl	-5.35422	-0.92429	-2.73117
H	-0.34734	1.70275	1.50660
H	2.74594	-2.28216	-3.64231

Catalyst (H, 4)-Product (R) alternative conformation
Electronic energy (Hartrees): -1870.654015
Free Energy at 1M concentration and 253 K (Hartrees): -1870.111157
Coordinates:

P	0.35186	0.51046	-1.94219
O	0.17606	0.30416	-0.32392
O	-0.84133	-0.31227	-2.67652
O	1.60475	-0.36513	-2.35110
O	0.40949	1.96716	-2.21657
C	-0.23419	-0.94086	0.07962
C	0.67473	-1.74256	0.92284
C	0.29951	-2.94319	1.37006
H	0.97742	-3.52132	1.98562
C	-1.02595	-3.48936	1.01016
C	-1.40822	-4.84717	1.46176
H	-0.73247	-5.42512	2.07987
C	-2.58832	-5.36634	1.10648
H	-2.86632	-6.35913	1.43598
C	-3.51047	-4.59239	0.25375
H	-4.45514	-5.02689	-0.04697
C	-3.17695	-3.36325	-0.15670
H	-3.85993	-2.82410	-0.79916
C	-1.87989	-2.75883	0.25317
C	-1.48418	-1.38475	-0.18631
C	-2.07660	-0.03310	-2.14573
C	-3.03681	0.75869	-2.94069
C	-4.23273	1.07415	-2.43651
H	-4.92544	1.66030	-3.02742
C	-4.59743	0.65329	-1.06720
C	-5.89041	1.08321	-0.48677
H	-6.58487	1.66674	-1.07825
C	-6.20222	0.76051	0.77289
H	-7.14496	1.07987	1.19795
C	-5.25639	-0.02037	1.59227
H	-5.50472	-0.25716	2.61877
C	-4.08866	-0.41999	1.07586
H	-3.39999	-0.96662	1.70550
C	-3.73662	-0.09160	-0.33267
C	-2.42979	-0.50801	-0.92873

H	2.39694	-0.12171	-1.75896
C	4.32860	-1.05438	-0.33466
C	3.84703	1.17222	0.15788
C	4.05213	-2.21591	-1.10142
C	5.33774	-1.10868	0.67520
C	4.83823	1.20069	1.17352
C	4.75468	-3.37774	-0.86937
H	3.27901	-2.17147	-1.86065
C	6.04548	-2.31988	0.88427
C	5.57364	0.07039	1.42583
H	5.00463	2.11861	1.72453
C	5.75889	-3.43306	0.12711
H	4.53632	-4.26394	-1.45731
H	6.81342	-2.35256	1.65213
H	6.33725	0.06647	2.19907
H	6.30136	-4.35872	0.29196
C	2.94001	2.36238	-0.08416
H	2.46425	2.25105	-1.05767
C	1.86494	2.48284	1.00994
H	2.30219	2.96914	1.89089
H	1.58915	1.45855	1.30356
N	0.70436	3.21451	0.52923
N	3.61125	0.09045	-0.57294
C	-0.52110	3.04062	1.17329
C	-0.62475	2.64627	2.51933
C	-1.71199	3.27359	0.45284
C	-1.87768	2.49353	3.11664
H	0.27011	2.46371	3.10517
C	-2.95151	3.12110	1.06106
H	-1.64505	3.56325	-0.59231
C	-3.04964	2.72693	2.39985
H	-1.93077	2.18752	4.15832
H	-3.85259	3.30512	0.48164
H	-4.02028	2.60499	2.87031
H	0.60074	3.13973	-0.47965
Cl	3.91455	3.90469	-0.22549
H	1.65081	-1.35205	1.18142
H	-2.76620	1.08914	-3.93541

Catalyst (H, 4)-Product (R) alternative conformation
Electronic energy (Hartrees): -1870.64996
Free Energy at 1M concentration and 253 K (Hartrees): -1870.108703
Coordinates:

P	-0.31819	0.72102	0.38034
O	0.70668	1.58712	1.30981
O	0.54703	0.12048	-0.87219
O	-1.22840	1.75553	-0.39313
O	-1.01744	-0.27751	1.22209
C	1.72203	2.24790	0.66241
C	1.78431	3.71817	0.78911
C	2.78609	4.40629	0.23716
H	2.81696	5.48385	0.34077
C	3.83239	3.70523	-0.53379
C	4.89889	4.48684	-1.20150
H	4.93465	5.56357	-1.09099
C	5.82079	3.86716	-1.94562
H	6.59889	4.43921	-2.43434
C	5.77115	2.40295	-2.11579
H	6.50494	1.91068	-2.74095
C	4.81520	1.68672	-1.51279

H	4.78481	0.61825	-1.67653
C	3.80130	2.35594	-0.65105
C	2.72677	1.57810	0.04505
C	1.66377	-0.56761	-0.47505
C	1.72384	-2.02596	-0.69427
C	2.78991	-2.72552	-0.29834
H	2.81882	-3.79590	-0.46066
C	3.91687	-2.04386	0.37281
C	5.06369	-2.83699	0.87161
H	5.09027	-3.90901	0.72059
C	6.07006	-2.23305	1.51274
H	6.90732	-2.81356	1.87810
C	6.03675	-0.77481	1.73545
H	6.84470	-0.29592	2.27348
C	5.00718	-0.04793	1.28555
H	4.99307	1.01615	1.47976
C	3.89359	-0.69971	0.54432
C	2.73087	0.08864	0.02990
H	-2.21345	1.47144	-0.39133
C	-4.58040	2.04425	0.32115
C	-4.32254	-0.08554	-0.59025
C	-4.00688	3.30007	0.64739
C	-5.95263	1.79790	0.63186
C	-5.67713	-0.40814	-0.31118
C	-4.77515	4.26866	1.25423
H	-2.96163	3.47195	0.41518
C	-6.71515	2.81842	1.25523
C	-6.47906	0.52611	0.29410
H	-6.06160	-1.38026	-0.59538
C	-6.13716	4.02998	1.55970
H	-4.33225	5.22774	1.50450
H	-7.75881	2.62621	1.48814
H	-7.51895	0.30302	0.51717
H	-6.72372	4.80800	2.03833
C	-3.39075	-1.10854	-1.20578
H	-2.47186	-0.63069	-1.54139
C	-3.05564	-2.25572	-0.24455
H	-3.99029	-2.72603	0.10388
H	-2.57432	-1.77663	0.61011
N	-2.13911	-3.19931	-0.84655
N	-3.80217	1.09334	-0.28580
C	-1.25612	-3.99352	-0.10548
C	-0.72045	-3.59062	1.13264
C	-0.82480	-5.21613	-0.65515
C	0.21771	-4.39318	1.78041
H	-0.98679	-2.63253	1.56279
C	0.11685	-6.00268	0.00161
H	-1.22931	-5.54089	-1.61155
C	0.64693	-5.60119	1.22923
H	0.62431	-4.05616	2.73033
H	0.43309	-6.93906	-0.45028
H	1.38014	-6.21522	1.74270
H	-2.53508	-3.67891	-1.64447
Cl	-4.15578	-1.77254	-2.75001
H	1.01259	4.24083	1.34000
H	0.90233	-2.52725	-1.18742

Catalyst (H, 4)-Product (R) alternative conformation
Electronic energy (Hartrees): -1870.649452
Free Energy at 1M concentration and 253 K (Hartrees): -1870.108278

Coordinates:

P	-0.28175	-0.41706	2.27798
O	-0.30661	-0.65252	0.64051
O	-1.76220	0.10234	2.72099
O	0.50614	0.93200	2.52674
O	0.22401	-1.65549	2.90381
C	-0.88741	0.36428	-0.07289
C	-0.04675	1.15033	-0.99734
C	-0.57182	2.15946	-1.69570
H	0.05849	2.73392	-2.36317
C	-2.00142	2.50252	-1.54304
C	-2.55889	3.66684	-2.26885
H	-1.92905	4.24699	-2.93179
C	-3.84145	4.00884	-2.10620
H	-4.24764	4.86106	-2.63557
C	-4.70430	3.23334	-1.19465
H	-5.73580	3.52864	-1.05189
C	-4.21211	2.17693	-0.53707
H	-4.85731	1.63738	0.14311
C	-2.79478	1.76527	-0.72822
C	-2.21794	0.59064	-0.00296
C	-2.80681	-0.53743	2.10354
C	-3.72402	-1.34931	2.92870
C	-4.78407	-1.94519	2.37725
H	-5.44773	-2.53708	2.99530
C	-5.03647	-1.81957	0.92691
C	-6.17605	-2.54141	0.31502
H	-6.84404	-3.12769	0.93376
C	-6.38375	-2.47461	-1.00422
H	-7.21585	-3.00359	-1.45066
C	-5.47068	-1.69135	-1.85783
H	-5.63047	-1.66155	-2.92797
C	-4.44112	-1.03125	-1.31489
H	-3.77127	-0.48538	-1.96523
C	-4.21432	-1.06736	0.15641
C	-3.07565	-0.33195	0.79179
H	1.44100	0.95365	2.11023
C	2.85194	2.40053	0.59309
C	3.69981	0.25433	0.94876
C	1.86570	3.37857	0.88435
C	3.86776	2.69283	-0.36628
C	4.74881	0.47478	0.01912
C	1.89095	4.59309	0.23513
H	1.09535	3.14390	1.61051
C	3.86761	3.95645	-1.01050
C	4.82531	1.68142	-0.62952
H	5.46411	-0.31371	-0.16886
C	2.89611	4.88666	-0.71786
H	1.13027	5.33584	0.45536
H	4.64277	4.17332	-1.74021
H	5.61274	1.86967	-1.35427
H	2.89668	5.85030	-1.21768
C	3.48691	-1.08674	1.62484
H	2.88017	-0.95505	2.52064
C	2.78543	-2.11659	0.72667
H	1.82632	-1.67520	0.45729
H	2.56130	-3.00425	1.33372
N	3.53918	-2.41558	-0.47404
N	2.79806	1.18451	1.22574
C	2.93667	-2.80418	-1.67585

C	1.58949	-2.54019	-1.98519
C	3.72886	-3.44726	-2.64746
C	1.07145	-2.90220	-3.22874
H	0.93708	-2.06158	-1.26401
C	3.19816	-3.80324	-3.88234
H	4.77159	-3.66213	-2.42373
C	1.86217	-3.53291	-4.18840
H	0.02693	-2.68994	-3.44153
H	3.83510	-4.29937	-4.60958
H	1.44738	-3.81332	-5.15128
H	4.40301	-2.90722	-0.28358
Cl	5.08345	-1.76518	2.23054
H	1.00148	0.90459	-1.10923
H	-3.53667	-1.45762	3.98946

Catalyst (H, 4)-Product (R) alternative conformation
Electronic energy (Hartrees): -1870.647925
Free Energy at 1M concentration and 253 K (Hartrees): -1870.107106
Coordinates:

P	-0.25345	-0.06403	0.25038
O	-1.26404	-0.67164	1.38111
O	-1.18503	0.53900	-0.95978
O	0.72591	-1.01200	-0.32862
O	0.31002	1.16220	1.06939
C	-2.24576	-1.47745	0.86433
C	-2.21489	-2.92317	1.16427
C	-3.17179	-3.73036	0.69913
H	-3.13744	-4.78856	0.92658
C	-4.25734	-3.18746	-0.14449
C	-5.27531	-4.10252	-0.71038
H	-5.24683	-5.15941	-0.47617
C	-6.23247	-3.62821	-1.51480
H	-6.97509	-4.29788	-1.92892
C	-6.27106	-2.19260	-1.85214
H	-7.03444	-1.81975	-2.52283
C	-5.35925	-1.35619	-1.34268
H	-5.39344	-0.31250	-1.62443
C	-4.30665	-1.86157	-0.41919
C	-3.27927	-0.94798	0.17186
C	-2.32317	1.19605	-0.55947
C	-2.45365	2.63340	-0.87485
C	-3.55837	3.30390	-0.53926
H	-3.63925	4.35713	-0.77804
C	-4.65510	2.61878	0.17507
C	-5.84457	3.38910	0.60613
H	-5.92723	4.44194	0.36634
C	-6.82042	2.78875	1.29563
H	-7.68852	3.35270	1.61170
C	-6.71029	1.35875	1.64059
H	-7.49242	0.88503	2.21979
C	-5.64249	0.65229	1.25161
H	-5.57293	-0.38820	1.53829
C	-4.56240	1.29651	0.45463
C	-3.36065	0.52850	-0.00033
H	1.27451	1.42668	0.81274
C	2.89362	2.76255	-0.61457
C	3.83682	1.30397	0.94342
C	1.72922	3.34173	-1.17858
C	4.17732	3.12414	-1.12261
C	5.14571	1.60153	0.48766

C	1.84348	4.25084	-2.20630
H	0.76247	3.04294	-0.79066
C	4.25939	4.06705	-2.17922
C	5.30793	2.50469	-0.53344
H	5.99258	1.11523	0.95188
C	3.11516	4.61960	-2.70903
H	0.94968	4.68893	-2.63974
H	5.23797	4.34143	-2.56320
H	6.30074	2.74843	-0.90177
H	3.18312	5.33857	-3.51952
C	3.55480	0.27925	2.02636
H	2.60453	0.52383	2.50132
C	3.48110	-1.15932	1.50039
H	2.67112	-1.16816	0.76683
H	3.17010	-1.81218	2.33090
N	4.72163	-1.58281	0.88148
N	2.76180	1.86530	0.41156
C	4.75928	-2.59575	-0.09048
C	3.67414	-2.88084	-0.93931
C	5.95330	-3.32208	-0.25768
C	3.80058	-3.86392	-1.92067
H	2.73624	-2.34317	-0.85050
C	6.06413	-4.29732	-1.24392
H	6.79892	-3.11239	0.39431
C	4.98674	-4.57971	-2.08587
H	2.94966	-4.06840	-2.56476
H	6.99822	-4.84268	-1.34896
H	5.07025	-5.34322	-2.85297
H	5.47541	-1.66441	1.55296
Cl	4.79362	0.39971	3.37979
H	-1.41274	-3.32897	1.76755
H	-1.65089	3.14508	-1.38963

Catalyst (H, 4)-Product (R) alternative conformation
Electronic energy (Hartrees): -1870.647512
Free Energy at 1M concentration and 253 K (Hartrees): -1870.106698
Coordinates:

P	-0.21323	-1.29004	-0.59511
O	-0.98823	-0.07308	-1.37062
O	-1.23785	-1.86960	0.54281
O	0.85296	-0.58375	0.34268
O	0.30832	-2.26090	-1.57956
C	-1.67758	0.79305	-0.55909
C	-1.23675	2.20036	-0.49525
C	-1.87939	3.07718	0.27949
H	-1.54089	4.10519	0.31980
C	-3.03354	2.64170	1.09322
C	-3.69487	3.60421	2.00414
H	-3.35581	4.63173	2.04904
C	-4.71128	3.20797	2.77758
H	-5.19273	3.91147	3.44450
C	-5.17107	1.80681	2.73465
H	-5.97921	1.48758	3.38013
C	-4.58409	0.93009	1.91161
H	-4.92260	-0.09718	1.91538
C	-3.47190	1.36142	1.02128
C	-2.79795	0.40213	0.09093
C	-2.52895	-2.03952	0.11242
C	-3.07701	-3.40869	0.03447
C	-4.34470	-3.61027	-0.33374

```
H         -4.73348       -4.61966       -0.38582
C         -5.20552       -2.46418       -0.69330
C         -6.58923       -2.70447       -1.16399
H         -6.98118       -3.71299       -1.20864
C         -7.35991       -1.67764       -1.53780
H         -8.37053       -1.85429       -1.88261
C         -6.83036       -0.30150       -1.49509
H         -7.45155        0.52244       -1.82185
C         -5.58330       -0.07260       -1.06732
H         -5.20955        0.94230       -1.06932
C         -4.72286       -1.20056       -0.61556
C         -3.32797       -0.97303       -0.12251
H          1.81342       -0.99337        0.29762
C          3.85584       -1.78253        1.50841
C          4.11779       -1.09611       -0.70839
C          2.98531       -2.06723        2.59087
C          5.26547       -1.93106        1.67817
C          5.52754       -1.21199       -0.61622
C          3.50550       -2.48926        3.79399
H          1.91709       -1.94352        2.44792
C          5.76669       -2.36879        2.93097
C          6.08983       -1.62427        0.56699
H          6.13762       -0.98751       -1.48234
C          4.90308       -2.64250        3.96731
H          2.83790       -2.70779        4.62173
H          6.83979       -2.48249        3.05686
H          7.16810       -1.72115        0.66140
H          5.29052       -2.97670        4.92468
C          3.42005       -0.60248       -1.96396
H          2.41437       -1.02672       -1.99561
C          3.38171        0.93537       -2.07660
H          2.88917        1.19307       -3.02769
H          4.41265        1.30254       -2.13680
N          2.74052        1.50933       -0.91368
N          3.32483       -1.37002        0.31676
C          2.70249        2.88491       -0.69249
C          3.39988        3.81146       -1.48697
C          1.94898        3.36946        0.39712
C          3.34186        5.17509       -1.19152
H          3.97663        3.47785       -2.34260
C          1.88866        4.72970        0.66994
H          1.41709        2.65840        1.02451
C          2.58763        5.64865       -0.12008
H          3.88802        5.87257       -1.82097
H          1.29438        5.07571        1.51146
H          2.54207        6.71102        0.09721
H          1.91744        1.01641       -0.58721
Cl         4.23039       -1.24971       -3.46503
H         -0.38961        2.52234       -1.08665
H         -2.44685       -4.25345        0.28211
```

Catalyst (H, 4)-Product (R) alternative conformation
Electronic energy (Hartrees): -1870.647238
Free Energy at 1M concentration and 253 K (Hartrees): -1870.106005
Coordinates:

```
P         -0.40058       -1.27934        0.21449
O         -1.07555       -0.50419       -1.07353
O         -1.46928       -1.19061        1.44551
O          0.71651       -0.30491        0.75518
O          0.02020       -2.63317       -0.20767
```

C	-1.77426	0.64297	-0.78343
C	-1.30311	1.91871	-1.36095
C	-1.96511	3.05446	-1.12921
H	-1.60595	3.98119	-1.55928
C	-3.17023	3.05063	-0.27519
C	-3.85563	4.32684	0.03363
H	-3.49533	5.25493	-0.39255
C	-4.92082	4.33923	0.84191
H	-5.42016	5.27248	1.06835
C	-5.41067	3.08249	1.43910
H	-6.25899	3.10316	2.11106
C	-4.80194	1.92331	1.16272
H	-5.16461	1.01896	1.63221
C	-3.63465	1.88670	0.23906
C	-2.93918	0.60331	-0.09331
C	-2.74830	-1.53562	1.09206
C	-3.33321	-2.77417	1.64457
C	-4.58816	-3.12051	1.34648
H	-5.00527	-4.03074	1.75912
C	-5.39512	-2.28019	0.43689
C	-6.76232	-2.70811	0.06053
H	-7.18351	-3.61367	0.47922
C	-7.48186	-1.97949	-0.79950
H	-8.48038	-2.29355	-1.07489
C	-6.91166	-0.75242	-1.38719
H	-7.49064	-0.18055	-2.10092
C	-5.67931	-0.35416	-1.05071
H	-5.27374	0.53508	-1.51391
C	-4.87718	-1.13509	-0.06938
C	-3.50056	-0.70777	0.33314
H	1.69424	-0.66145	0.69892
C	3.93947	-0.46277	1.76125
C	3.89862	-1.47059	-0.34535
C	3.22394	0.07677	2.86059
C	5.36654	-0.48640	1.79637
C	5.31563	-1.51919	-0.38748
C	3.90890	0.56915	3.94929
H	2.14035	0.09295	2.82145
C	6.03953	0.02890	2.93343
C	6.03670	-1.03161	0.67332
H	5.80568	-1.95418	-1.24962
C	5.32414	0.54636	3.98927
H	3.35795	0.98085	4.78935
H	7.12556	0.00798	2.95454
H	7.12303	-1.05932	0.66306
H	5.84281	0.94044	4.85769
C	3.03843	-1.94489	-1.49889
H	2.06584	-2.26148	-1.11593
C	2.85201	-0.88901	-2.62326
H	2.21184	-1.34321	-3.38676
H	3.81659	-0.68992	-3.09220
N	2.26588	0.34733	-2.15889
N	3.24500	-0.95816	0.68858
C	2.98574	1.48299	-1.77995
C	4.20991	1.83525	-2.37786
C	2.44697	2.34873	-0.80736
C	4.86139	3.01241	-2.01290
H	4.64502	1.20748	-3.14823
C	3.10108	3.52727	-0.46313
H	1.52436	2.07136	-0.30611

C	4.31611	3.87221	-1.05918
H	5.80316	3.26260	-2.49413
H	2.66069	4.17486	0.29033
H	4.82683	4.78976	-0.78499
H	1.36992	0.24472	-1.70257
Cl	3.73838	-3.45367	-2.26667
H	-0.42000	1.93216	-1.98571
H	-2.74126	-3.40190	2.29844

Catalyst (H, 4)-Product (R) alternative conformation
Electronic energy (Hartrees): -1870.645336
Free Energy at 1M concentration and 253 K (Hartrees): -1870.105055
Coordinates:

P	0.48131	-0.52282	-0.72776
O	1.16685	-0.08441	0.69925
O	1.68683	-0.98879	-1.72387
O	0.06487	0.80091	-1.48610
O	-0.58192	-1.51540	-0.45360
C	2.30663	0.67162	0.58285
C	2.28616	2.06261	1.07855
C	3.37891	2.82554	0.99963
H	3.34821	3.84461	1.36484
C	4.61688	2.28982	0.39607
C	5.80182	3.16652	0.25092
H	5.77321	4.18494	0.61798
C	6.91110	2.70551	-0.33658
H	7.77596	3.34742	-0.44360
C	6.95503	1.32460	-0.85359
H	7.84801	0.96563	-1.34889
C	5.89193	0.52244	-0.72348
H	5.93982	-0.47763	-1.13271
C	4.66024	1.00875	-0.04316
C	3.45843	0.13185	0.12084
C	2.62026	-1.80424	-1.13628
C	2.73449	-3.20187	-1.59992
C	3.66156	-4.01266	-1.08368
H	3.73460	-5.03475	-1.43408
C	4.56619	-3.52613	-0.02117
C	5.54634	-4.45325	0.59023
H	5.62594	-5.47293	0.23427
C	6.33612	-4.03592	1.58535
H	7.05171	-4.71417	2.03193
C	6.22455	-2.65350	2.08804
H	6.84958	-2.32992	2.91037
C	5.34558	-1.80633	1.53996
H	5.26460	-0.80581	1.94266
C	4.48309	-2.24372	0.40799
C	3.50028	-1.31409	-0.23245
H	-0.85404	1.15742	-1.20307
C	-2.03868	3.12732	-0.12985
C	-3.41078	1.29892	-0.60072
C	-0.75938	3.73479	-0.21085
C	-3.11275	3.83576	0.48846
C	-4.53329	1.94480	-0.01391
C	-0.55925	4.99109	0.31697
H	0.04388	3.19143	-0.69572
C	-2.87253	5.12833	1.02059
C	-4.37759	3.19535	0.52847
H	-5.48664	1.43377	-0.00733
C	-1.61963	5.69340	0.93897

H	0.42248	5.45081	0.25455
H	-3.69327	5.66306	1.49066
H	-5.21934	3.70778	0.98682
H	-1.44079	6.68270	1.34863
C	-3.52015	-0.09745	-1.17777
H	-2.60874	-0.33060	-1.72606
C	-3.71717	-1.21439	-0.13267
H	-2.76306	-1.27764	0.40212
H	-3.82519	-2.16159	-0.66720
N	-4.81673	-1.02574	0.79934
N	-2.21756	1.87268	-0.65428
C	-5.91958	-1.88582	0.88116
C	-6.41172	-2.59687	-0.22726
C	-6.59013	-2.01779	2.11200
C	-7.52832	-3.42103	-0.09220
H	-5.94223	-2.48565	-1.19740
C	-7.71056	-2.83418	2.23001
H	-6.21992	-1.47705	2.98072
C	-8.18869	-3.54917	1.12984
H	-7.88914	-3.96149	-0.96306
H	-8.20651	-2.91834	3.19317
H	-9.05960	-4.19001	1.22346
H	-4.51510	-0.69979	1.70657
Cl	-4.85666	-0.11268	-2.43690
H	1.37924	2.46395	1.51235
H	2.06452	-3.56871	-2.36709

Catalyst (H, 4)-Product (R) alternative conformation
Electronic energy (Hartrees): -1870.645306
Free Energy at 1M concentration and 253 K (Hartrees): -1870.105047
Coordinates:

P	-0.48117	-0.49527	0.66113
O	-1.65829	-0.99767	1.67367
O	-1.20426	-0.03524	-0.74019
O	0.58689	-1.46960	0.34392
O	-0.06264	0.81712	1.43731
C	-2.59220	-1.81513	1.08966
C	-2.67306	-3.22470	1.52310
C	-3.59602	-4.03977	1.00619
H	-3.64391	-5.07096	1.33346
C	-4.53036	-3.54490	-0.02635
C	-5.50515	-4.47532	-0.64121
H	-5.55889	-5.50462	-0.30895
C	-6.32151	-4.04928	-1.61085
H	-7.03266	-4.73020	-2.06047
C	-6.24585	-2.65313	-2.08116
H	-6.89319	-2.32150	-2.88277
C	-5.37218	-1.80291	-1.52948
H	-5.31841	-0.79139	-1.90850
C	-4.47882	-2.25137	-0.42619
C	-3.49855	-1.31990	0.21529
C	-2.35258	0.70039	-0.58285
C	-2.36327	2.10215	-1.04756
C	-3.46493	2.84672	-0.92780
H	-3.45705	3.87393	-1.27073
C	-4.68131	2.27937	-0.30936
C	-5.87528	3.13480	-0.11837
H	-5.86967	4.16144	-0.46296
C	-6.96432	2.64429	0.48296
H	-7.83575	3.27064	0.62368

C	-6.97674	1.25168	0.96946
H	-7.85312	0.86844	1.47613
C	-5.90537	0.46860	0.79779
H	-5.92958	-0.54102	1.18497
C	-4.69633	0.98837	0.10182
C	-3.48583	0.13352	-0.10773
H	0.85520	1.17723	1.15594
C	2.05149	3.14243	0.08097
C	3.41468	1.31376	0.57478
C	0.77300	3.75317	0.14767
C	3.13249	3.84451	-0.53249
C	4.54391	1.95316	-0.00610
C	0.58035	5.00701	-0.38862
H	-0.03539	3.21398	0.62869
C	2.89980	5.13454	-1.07408
C	4.39602	3.20070	-0.55770
H	5.49578	1.43930	-0.00089
C	1.64768	5.70314	-1.00579
H	-0.40066	5.46957	-0.33694
H	3.72562	5.66460	-1.54052
H	5.24296	3.70825	-1.01190
H	1.47470	6.69053	-1.42251
C	3.51539	-0.07851	1.16316
H	2.59415	-0.30650	1.69720
C	3.73119	-1.20406	0.13171
H	2.78802	-1.26895	-0.42191
H	3.82587	-2.14697	0.67638
N	4.85053	-1.02649	-0.77926
N	2.22260	1.89082	0.61454
C	5.94844	-1.89543	-0.83221
C	6.41614	-2.59627	0.29306
C	6.63863	-2.04760	-2.04973
C	7.52829	-3.43068	0.18714
H	5.93104	-2.46909	1.25360
C	7.75443	-2.87395	-2.13870
H	6.28721	-1.51506	-2.93123
C	8.20812	-3.57903	-1.02189
H	7.87000	-3.96310	1.07057
H	8.26582	-2.97397	-3.09223
H	9.07529	-4.22780	-1.09287
H	4.56680	-0.71067	-1.69587
Cl	4.82898	-0.08149	2.44658
H	-1.98119	-3.59729	2.26775
H	-1.47205	2.52650	-1.49188

Catalyst (Ph, 5)-THF complex
Electronic energy (Hartrees): -876.474353
Free Energy at 1M concentration and 253 K (Hartrees): -875.921717
Coordinates:

P	0.81834	-1.11517	-0.31813
O	0.90693	0.15055	0.71813
O	-0.70932	-1.15256	-0.89936
O	1.79043	-1.07439	-1.43432
O	0.93470	-2.31495	0.70083
C	0.53907	1.32996	0.12678
C	1.57857	2.33326	-0.22904
C	1.21456	3.46090	-0.86015
H	1.95802	4.20418	-1.12396
C	-0.20003	3.70567	-1.20976
C	-0.56882	4.93540	-1.94912

H	0.18844	5.66863	-2.19749
C	-1.83746	5.14484	-2.31578
H	-2.10534	6.04285	-2.85737
C	-2.87370	4.14511	-1.99656
H	-3.89407	4.30874	-2.31852
C	-2.55248	3.03341	-1.32487
H	-3.32532	2.30330	-1.12737
C	-1.15086	2.80071	-0.87910
C	-0.76403	1.56932	-0.12545
C	-1.72178	-0.70969	-0.07590
C	-2.88272	-1.61219	0.17041
C	-3.92676	-1.17727	0.89221
H	-4.77886	-1.82522	1.06449
C	-3.92075	0.17363	1.48352
C	-5.03896	0.59755	2.35812
H	-5.87043	-0.07168	2.54133
C	-5.02609	1.80770	2.92684
H	-5.84284	2.11625	3.56654
C	-3.89223	2.72179	2.69356
H	-3.87754	3.69189	3.17344
C	-2.88021	2.34990	1.90121
H	-2.04936	3.02860	1.76182
C	-2.89378	1.01715	1.23676
C	-1.78376	0.58508	0.33372
C	-2.93220	-2.95920	-0.43690
C	-4.04265	-3.31455	-1.35677
C	-1.99328	-3.87850	-0.15339
C	-4.09711	-4.53287	-1.90951
C	-2.05518	-5.22145	-0.76719
C	-3.05374	-5.53208	-1.60223
H	-4.90502	-4.78843	-2.58290
H	-1.29203	-5.95358	-0.53683
C	3.00556	2.08503	0.08457
C	3.38311	1.69141	1.31556
C	4.04151	2.27005	-0.96505
C	4.80879	1.44799	1.61687
C	5.33178	2.04453	-0.68847
C	5.73431	1.61570	0.66548
H	5.10170	1.13025	2.60916
H	6.08277	2.17298	-1.45714
H	1.85552	-2.72771	0.69633
O	3.37375	-3.20271	0.72496
C	4.14598	-2.45418	1.69871
C	4.03734	-3.16588	-0.57214
C	5.48381	-2.15441	1.02123
H	3.60043	-1.53467	1.94505
H	4.23545	-3.06292	2.60326
C	5.07750	-2.05486	-0.45674
H	4.49791	-4.14673	-0.74374
H	3.27914	-2.96520	-1.33002
H	6.18136	-2.98630	1.16994
H	5.95453	-1.24943	1.41177
H	5.91453	-2.19085	-1.14654
H	4.60358	-1.09141	-0.66978
H	-3.10074	-6.51560	-2.05161
H	6.77836	1.43319	0.88453
H	-4.80907	-2.58574	-1.58991
H	-1.19837	-3.65870	0.54434
H	3.75590	2.57481	-1.96412
H	2.65442	1.56473	2.10660

Catalyst (Ph, 5)-Quinoline complex
Electronic energy (Hartrees): -1582.958483
Free Energy at 1M concentration and 253 K (Hartrees): -1582.368328
Coordinates:

P	-0.15920	-0.16265	-1.62772
O	-0.31600	0.63907	-0.18784
O	1.44039	-0.29645	-1.91149
O	-0.84162	0.48627	-2.76542
O	-0.62290	-1.60339	-1.18077
C	0.43852	1.78135	-0.11449
C	-0.23629	3.10559	-0.12193
C	0.50850	4.22287	-0.11049
H	0.03292	5.19683	-0.11237
C	1.98472	4.14350	-0.07197
C	2.78753	5.38824	-0.09936
H	2.29540	6.35248	-0.12773
C	4.12351	5.32723	-0.09113
H	4.70894	6.23735	-0.11243
C	4.80427	4.01865	-0.06279
H	5.88578	3.97623	-0.07331
C	4.08898	2.88820	-0.03383
H	4.60882	1.93945	-0.03083
C	2.60194	2.93981	-0.01818
C	1.78086	1.69413	0.01092
C	2.20152	-0.58695	-0.80326
C	2.82054	-1.93392	-0.68108
C	3.53723	-2.23509	0.41415
H	3.99654	-3.21167	0.51521
C	3.70429	-1.24951	1.50047
C	4.44943	-1.62924	2.72328
H	4.90196	-2.60994	2.80221
C	4.56344	-0.76518	3.73704
H	5.10621	-1.04455	4.63079
C	3.93829	0.56723	3.64580
H	4.01528	1.25397	4.47889
C	3.26780	0.92224	2.54380
H	2.79855	1.89587	2.51261
C	3.15596	-0.01721	1.39353
C	2.40539	0.34996	0.15467
C	2.62639	-2.94319	-1.74871
C	2.87986	-2.58292	-3.16748
C	2.20335	-4.18767	-1.45359
C	2.68288	-3.48275	-4.13900
C	1.98916	-5.17798	-2.52942
C	2.21441	-4.84257	-3.80491
H	2.87161	-3.21768	-5.17130
H	1.64543	-6.17418	-2.28258
C	-1.71574	3.19400	-0.13500
C	-2.45536	2.48135	0.73660
C	-2.40238	4.07711	-1.11289
C	-3.92943	2.57772	0.71903
C	-3.73910	4.15451	-1.13075
C	-4.53954	3.36940	-0.16989
H	-4.51409	2.00182	1.42456
H	-4.23671	4.79238	-1.84970
H	-1.43574	-1.58775	-0.56284
C	-3.90414	-0.92333	-0.06298
C	-2.76424	-2.03620	1.64231
C	-3.88334	-0.31199	-1.34476

C	-5.12400	-0.93667	0.68507
C	-3.94095	-2.10824	2.43535
C	-5.02866	0.26723	-1.84447
H	-2.96412	-0.28091	-1.91927
C	-6.28426	-0.33476	0.13541
C	-5.10313	-1.55790	1.95750
H	-3.90033	-2.58231	3.40807
C	-6.23641	0.25781	-1.10565
H	-4.99993	0.74556	-2.81836
H	-7.20476	-0.34667	0.71241
H	-6.01300	-1.58722	2.55102
H	-7.12405	0.72452	-1.52130
N	-2.75678	-1.48228	0.43522
C	-1.46795	-2.54219	2.16667
C	-0.29535	-1.92519	2.02026
H	0.61842	-2.34406	2.42542
H	-0.24719	-0.98172	1.48855
Cl	-1.56170	-4.05493	3.08065
H	-5.61973	3.43464	-0.18785
H	2.05610	-5.56662	-4.59366
H	3.23765	-1.59329	-3.42263
H	1.99407	-4.47441	-0.43010
H	-1.98467	1.84083	1.47244
H	-1.82486	4.65148	-1.82646

Catalyst (Ph, 5)-Enamine protonation TS (pro-S) lowest free energy conformation
Electronic energy (Hartrees): -1870.568227
Free Energy at 1M concentration and 253 K (Hartrees): -1869.863166
Coordinates:

P	-0.53158	0.71259	-0.30161
O	-1.71420	0.38042	-1.37828
O	-0.87633	-0.16760	1.03885
O	-0.43664	2.14354	0.11209
O	0.71080	0.06389	-0.98916
C	-2.98022	0.38154	-0.84357
C	-3.92078	1.48033	-1.19559
C	-5.15079	1.49277	-0.65730
H	-5.84745	2.28557	-0.90438
C	-5.57472	0.43402	0.28161
C	-6.91996	0.49924	0.89911
H	-7.60171	1.29816	0.63504
C	-7.29800	-0.42590	1.78743
H	-8.28041	-0.37623	2.23903
C	-6.37192	-1.50991	2.16504
H	-6.67582	-2.24286	2.90128
C	-5.15658	-1.58085	1.60988
H	-4.48747	-2.37183	1.92021
C	-4.73143	-0.57651	0.59601
C	-3.38137	-0.63320	-0.04085
C	-1.22790	-1.46916	0.77860
C	-0.27420	-2.56043	1.10840
C	-0.60891	-3.83266	0.83588
H	0.07333	-4.64034	1.07473
C	-1.90196	-4.15135	0.19559
C	-2.22514	-5.55759	-0.14069
H	-1.53492	-6.35116	0.11744
C	-3.36878	-5.85372	-0.76707
H	-3.60297	-6.88130	-1.01360
C	-4.30893	-4.77900	-1.13657
H	-5.22244	-5.02317	-1.66336

C	-4.02908	-3.50612	-0.83362
H	-4.72342	-2.73389	-1.13556
C	-2.77474	-3.16249	-0.10874
C	-2.43879	-1.75057	0.24502
C	1.04483	-2.24005	1.70524
C	1.13040	-1.33986	2.88443
C	2.17974	-2.73734	1.17722
C	2.32008	-1.03147	3.41437
C	3.49123	-2.38926	1.76241
C	3.55745	-1.58230	2.82690
H	2.37802	-0.37907	4.27598
H	4.39813	-2.78829	1.32705
C	-3.49927	2.57239	-2.10507
C	-2.91325	2.30036	-3.28656
C	-3.71802	3.99032	-1.71611
C	-2.48405	3.39824	-4.17785
C	-3.32791	4.98169	-2.52692
C	-2.68033	4.67150	-3.81704
H	-2.01104	3.16882	-5.12396
H	-3.48322	6.01305	-2.23760
H	1.22933	2.70799	0.51593
C	2.19391	2.76224	2.33448
C	3.08592	1.87307	0.26440
C	1.11508	3.37958	2.99528
C	3.34247	2.34948	3.05518
C	4.22701	1.39791	0.99228
C	1.19920	3.59845	4.35855
H	0.22979	3.64772	2.42836
C	3.39916	2.59833	4.44369
C	4.35898	1.66005	2.32540
H	4.97709	0.83411	0.45617
C	2.34430	3.21678	5.08763
H	0.36749	4.06851	4.87415
H	4.28004	2.28448	4.99646
H	5.24821	1.32928	2.85540
H	2.39043	3.40216	6.15578
C	2.86264	1.59730	-1.12255
C	3.95665	0.91430	-1.93035
H	4.92519	1.42062	-1.80692
H	3.69746	0.98933	-2.99569
N	4.09327	-0.47111	-1.49083
N	2.14058	2.53882	0.97749
C	5.04590	-1.32781	-2.04640
C	4.91646	-2.71692	-1.84594
C	6.15937	-0.86049	-2.76806
C	5.87165	-3.59950	-2.33488
H	4.05559	-3.09572	-1.30182
C	7.11417	-1.75795	-3.24895
H	6.28207	0.19885	-2.96242
C	6.98459	-3.12941	-3.03862
H	5.74366	-4.66531	-2.16639
H	7.96541	-1.37182	-3.80318
H	7.72941	-3.82053	-3.42000
H	3.19849	-0.91353	-1.31666
H	1.63200	0.69689	-1.04170
Cl	2.12615	2.97189	-2.04271
H	-2.36596	5.47612	-4.46915
H	4.51738	-1.32726	3.25671
H	2.14941	-3.38229	0.30792
H	0.22926	-0.93969	3.33175

H	-4.18197	4.22934	-0.76724
H	-2.75994	1.27781	-3.60860

Catalyst (Ph, 5)-Enamine protonation TS (pro-S) alternative conformation
Electronic energy (Hartrees): -1870.567301
Free Energy at 1M concentration and 253 K (Hartrees): -1869.862175
Coordinates:

P	-0.15456	0.11509	-0.23963
O	1.31970	0.49514	0.35078
O	-0.02520	-1.33691	-0.97952
O	-0.52582	1.03090	-1.43203
O	-1.08822	0.11701	0.93926
C	2.41164	0.05255	-0.35837
C	3.40196	1.05230	-0.83833
C	4.54402	0.64545	-1.41165
H	5.26537	1.37333	-1.76615
C	4.80617	-0.78989	-1.62345
C	6.03205	-1.21297	-2.33986
H	6.75455	-0.47467	-2.66507
C	6.25101	-2.50780	-2.59088
H	7.14611	-2.81590	-3.11566
C	5.26495	-3.51927	-2.16721
H	5.43536	-4.56339	-2.39612
C	4.15710	-3.14757	-1.51544
H	3.43669	-3.90578	-1.23997
C	3.91448	-1.71255	-1.19679
C	2.69457	-1.27157	-0.45068
C	0.49332	-2.31667	-0.17189
C	-0.36986	-3.45671	0.25034
C	0.13569	-4.38375	1.08462
H	-0.45972	-5.23828	1.38354
C	1.51759	-4.27113	1.59351
C	2.02337	-5.26353	2.57059
H	1.39130	-6.08232	2.89138
C	3.26169	-5.15170	3.06300
H	3.62962	-5.87623	3.77801
C	4.12611	-4.03267	2.64273
H	5.11924	-3.93498	3.06199
C	3.68078	-3.13462	1.75644
H	4.32341	-2.30927	1.48054
C	2.31610	-3.26316	1.17559
C	1.79668	-2.27779	0.18163
C	-1.75127	-3.60126	-0.28541
C	-2.02766	-3.33764	-1.72397
C	-2.76825	-3.99426	0.50835
C	-3.26833	-3.46129	-2.21140
C	-4.12905	-4.16025	-0.04337
C	-4.37059	-3.89011	-1.33004
H	-3.46227	-3.26849	-3.25865
H	-4.93332	-4.49057	0.60098
C	3.12966	2.49532	-0.71352
C	3.85938	3.26138	0.11633
C	2.10102	3.12482	-1.57521
C	3.63723	4.72153	0.16529
C	1.91523	4.45004	-1.54184
C	2.72331	5.28765	-0.63210
H	4.22224	5.33270	0.84022
H	1.17069	4.91041	-2.17832
H	-5.37033	-3.99889	-1.73022
H	2.56354	6.35762	-0.60148

H	-1.37783	1.74760	-1.23513
C	-2.54143	2.63164	-0.83844
C	-2.05090	3.27295	0.46410
H	-1.89834	2.48268	1.19943
H	-2.83747	3.94102	0.86186
N	-0.78631	3.97795	0.29857
H	-0.90434	4.78793	-0.29884
C	-0.00744	4.27688	1.43362
C	0.43387	5.59036	1.66305
C	0.40544	3.26400	2.31786
C	1.28521	5.88169	2.72780
H	0.11533	6.38311	0.98954
C	1.23748	3.57001	3.39132
H	0.10638	2.23647	2.14100
C	1.68933	4.87506	3.60431
H	1.62328	6.90378	2.87543
H	1.55277	2.77178	4.05789
H	2.34770	5.10139	4.43739
C	-3.65047	1.73083	-0.74560
C	-4.78858	1.69307	-1.61803
C	-4.60705	-0.13003	0.48787
C	-5.79928	0.80006	-1.41057
H	-4.83406	2.41294	-2.42293
C	-4.48707	-1.02891	1.56503
C	-5.76484	-0.13393	-0.32847
H	-6.66594	0.80556	-2.06641
C	-5.53203	-1.89253	1.84042
H	-3.58122	-1.02519	2.16266
C	-6.80288	-1.04331	-0.03264
C	-6.69416	-1.90554	1.04244
H	-5.44853	-2.57871	2.67757
H	-7.68998	-1.04871	-0.65960
H	-7.49835	-2.59682	1.27164
N	-3.60776	0.78032	0.23260
H	-2.68802	0.60860	0.69274
Cl	-2.71996	3.89192	-2.13429
H	-1.22434	-3.06437	-2.39571
H	-2.61193	-4.18202	1.56330
H	1.51709	2.51843	-2.25504
H	4.62615	2.82200	0.74288

Catalyst (Ph, 5)-Enamine protonation TS (pro-S) alternative conformation
Electronic energy (Hartrees): -1870.561197
Free Energy at 1M concentration and 253 K (Hartrees): -1869.856683
Coordinates:

P	0.09820	0.07307	-0.45362
O	-1.09685	-0.95697	-0.01495
O	-0.60111	1.54945	-0.56409
O	0.74629	-0.20502	-1.77093
O	0.99915	0.05480	0.81583
C	-2.28612	-0.77496	-0.67607
C	-2.74102	-1.80920	-1.64363
C	-3.89739	-1.63122	-2.30203
H	-4.24241	-2.37481	-3.01113
C	-4.71495	-0.42084	-2.07721
C	-5.96089	-0.22477	-2.85459
H	-6.28911	-0.98152	-3.55629
C	-6.68468	0.88831	-2.69605
H	-7.59385	1.03097	-3.26576
C	-6.23915	1.93184	-1.75373

H	-6.81792	2.84020	-1.64644
C	-5.11810	1.76295	-1.04313
H	-4.80018	2.55213	-0.37537
C	-4.31704	0.51593	-1.18476
C	-3.06326	0.30099	-0.40187
C	-1.40340	1.86244	0.50321
C	-0.92086	2.84543	1.50659
C	-1.69284	3.13863	2.56537
H	-1.35357	3.85229	3.30724
C	-3.00216	2.47652	2.74803
C	-3.81407	2.78135	3.94917
H	-3.47100	3.51790	4.66512
C	-4.97240	2.14663	4.15715
H	-5.56369	2.36916	5.03599
C	-5.44121	1.13012	3.19681
H	-6.36811	0.60320	3.38343
C	-4.71840	0.85006	2.10624
H	-5.07158	0.08349	1.43019
C	-3.44249	1.57058	1.84248
C	-2.61704	1.27989	0.63138
C	0.42615	3.44788	1.35843
C	0.83474	4.04717	0.06111
C	1.31705	3.40127	2.36751
C	2.07527	4.52158	-0.10437
C	2.68479	3.92991	2.18073
C	3.04567	4.45879	1.00605
H	2.36933	4.95441	-1.05172
H	3.39691	3.87843	2.99409
C	-1.90896	-3.00831	-1.90905
C	-1.40845	-3.74252	-0.89610
C	-1.60012	-3.40334	-3.30830
C	-0.55208	-4.91427	-1.17440
C	-0.82427	-4.46614	-3.55536
C	-0.27342	-5.25704	-2.43694
H	-0.15026	-5.49452	-0.35431
H	-0.59559	-4.74625	-4.57552
H	2.55573	-0.02202	-1.69322
C	3.73901	1.63552	-1.99803
C	3.92739	0.05792	-0.16636
C	3.06341	2.01450	-3.17358
C	4.74640	2.46554	-1.44464
C	4.92737	0.90366	0.41686
C	3.41231	3.19978	-3.79516
H	2.26358	1.38611	-3.55089
C	5.08671	3.66189	-2.11312
C	5.33063	2.04724	-0.21097
H	5.38373	0.60350	1.35008
C	4.43121	4.02420	-3.27409
H	2.88823	3.50126	-4.69672
H	5.86090	4.29625	-1.69105
H	6.11146	2.66027	0.23123
H	4.69019	4.94778	-3.78119
C	3.33049	-1.06987	0.48684
C	3.77911	-1.57373	1.85173
H	4.22961	-0.74035	2.40367
H	4.54130	-2.35423	1.76976
N	2.66463	-2.08394	2.65469
H	1.87066	-1.45446	2.60345
C	2.25335	-3.42720	2.59408
C	0.88255	-3.73051	2.51046

C	3.16937	-4.48881	2.68934
C	0.44511	-5.05180	2.52807
H	0.16576	-2.92038	2.40072
C	2.72183	-5.80830	2.68407
H	4.23111	-4.28228	2.78248
C	1.35952	-6.10370	2.60838
H	-0.61928	-5.25869	2.45529
H	3.44862	-6.61337	2.75324
H	1.01753	-7.13398	2.61011
H	2.05135	-0.44513	0.68415
N	3.40901	0.46371	-1.35657
Cl	2.89842	-2.43296	-0.62001
H	4.04923	4.83919	0.86588
H	0.35480	-6.11433	-2.64142
H	-1.98836	-2.82331	-4.13623
H	-1.63066	-3.49441	0.13441
H	0.12839	4.10798	-0.75727
H	1.05796	2.94526	3.31525

Catalyst (Ph, 5)-Enamine protonation TS (pro-S) alternative conformation
Electronic energy (Hartrees): -1870.55445
Free Energy at 1M concentration and 253 K (Hartrees): -1869.846355
Coordinates:

P	0.11361	0.86984	0.88622
O	-1.34724	1.52393	0.69610
O	-0.04647	-0.51860	1.71177
O	1.03107	1.80311	1.57844
O	0.52313	0.42158	-0.58887
C	-2.53011	0.90911	0.84482
C	-3.72818	1.79432	0.88083
C	-4.90674	1.31237	1.30614
H	-5.78061	1.95304	1.33194
C	-5.04659	-0.09076	1.73725
C	-6.33497	-0.55455	2.30439
H	-7.16924	0.13049	2.39122
C	-6.47439	-1.82014	2.71288
H	-7.41568	-2.15679	3.12778
C	-5.34205	-2.75903	2.60836
H	-5.45488	-3.77734	2.95745
C	-4.17667	-2.35153	2.09320
H	-3.35271	-3.05143	2.04264
C	-4.01389	-0.95228	1.60944
C	-2.72645	-0.48816	1.01308
C	-0.51559	-1.54139	0.93240
C	0.41458	-2.62187	0.50600
C	-0.01874	-3.56498	-0.34665
H	0.64094	-4.36635	-0.65867
C	-1.38826	-3.51246	-0.89344
C	-1.82480	-4.53606	-1.87133
H	-1.16005	-5.34616	-2.14425
C	-3.03733	-4.45972	-2.42879
H	-3.35295	-5.20489	-3.14741
C	-3.94099	-3.34769	-2.08133
H	-4.90831	-3.27286	-2.56107
C	-3.55898	-2.42205	-1.19408
H	-4.22556	-1.59646	-0.98688
C	-2.22719	-2.51425	-0.53276
C	-1.78002	-1.50195	0.47486
C	1.81278	-2.64296	0.99903
C	2.09335	-2.47527	2.44846

C	2.84366	-2.80804	0.14691
C	3.35272	-2.50602	2.90118
C	4.23101	-2.84747	0.65271
C	4.47173	-2.71316	1.96153
H	3.55376	-2.38650	3.95792
H	5.05184	-2.98830	-0.03851
C	-3.62791	3.20935	0.44177
C	-2.99613	3.52964	-0.70455
C	-4.23302	4.29392	1.25964
C	-2.89933	4.93935	-1.13645
C	-4.14432	5.56965	0.86301
C	-3.44508	5.90812	-0.39260
H	-2.38699	5.18521	-2.05765
H	-4.58132	6.35684	1.46375
H	2.74717	2.16286	0.76417
C	4.45377	1.24001	1.41294
C	3.74069	1.48413	-0.91724
C	4.18512	1.48088	2.77095
C	5.58268	0.47268	1.03861
C	4.85694	0.62964	-1.28567
C	5.05409	0.98918	3.73507
H	3.28883	2.02680	3.04526
C	6.45503	0.00532	2.03724
C	5.74312	0.18363	-0.36221
H	5.01050	0.40338	-2.33263
C	6.19995	0.26091	3.37634
H	4.83923	1.17293	4.78353
H	7.32368	-0.57717	1.74165
H	6.59615	-0.41299	-0.67470
H	6.87351	-0.10898	4.14236
C	2.86587	1.99276	-1.85932
C	2.97525	1.72297	-3.35297
H	4.00175	1.89588	-3.70055
H	2.36178	2.45661	-3.87983
N	2.60915	0.38717	-3.81573
N	3.62141	1.74839	0.43395
C	1.30325	-0.10638	-3.78547
C	1.09802	-1.49871	-3.85462
C	0.17447	0.73133	-3.74200
C	-0.18584	-2.02910	-3.90078
H	1.95990	-2.16216	-3.88312
C	-1.10809	0.18538	-3.78124
H	0.29043	1.80614	-3.66333
C	-1.30424	-1.19228	-3.86533
H	-0.31101	-3.10639	-3.96549
H	-1.96323	0.85470	-3.74098
H	-2.30622	-1.60695	-3.90465
H	3.30262	-0.32062	-3.61820
H	1.30598	0.85842	-0.99369
Cl	1.85299	3.39201	-1.41126
H	-3.37512	6.94094	-0.70857
H	5.48693	-2.75123	2.33405
H	-2.56253	2.76163	-1.33395
H	-4.73736	4.05962	2.18878
H	2.67359	-2.90853	-0.91832
H	1.27918	-2.34270	3.14973

Catalyst (Ph, 5)-Enamine protonation TS (pro-R) lowest free energy conformation
Electronic energy (Hartrees): -1870.572849
Free Energy at 1M concentration and 253 K (Hartrees): -1869.867372

Coordinates:

P	0.13959	0.05130	0.23577
O	-1.31458	0.66678	-0.18694
O	-0.11136	-1.48633	0.71710
O	0.84417	0.76243	1.34470
O	0.85868	0.01616	-1.15291
C	-2.40437	0.23220	0.53289
C	-3.24840	1.24362	1.22671
C	-4.37610	0.86096	1.84615
H	-4.99405	1.59228	2.35486
C	-4.76559	-0.56125	1.88719
C	-5.96376	-0.97167	2.65602
H	-6.57191	-0.22701	3.15444
C	-6.29859	-2.26345	2.74050
H	-7.17324	-2.56246	3.30362
C	-5.46874	-3.28672	2.07745
H	-5.73238	-4.33261	2.16813
C	-4.38754	-2.92572	1.37674
H	-3.78178	-3.69585	0.91833
C	-4.01859	-1.48866	1.24682
C	-2.82568	-1.05792	0.45555
C	-0.78329	-2.23468	-0.21351
C	-0.04027	-3.24751	-1.00714
C	-0.68684	-3.94258	-1.95708
H	-0.16098	-4.68854	-2.54172
C	-2.11683	-3.68969	-2.23216
C	-2.79413	-4.43098	-3.32150
H	-2.25619	-5.18477	-3.88292
C	-4.07153	-4.17114	-3.61901
H	-4.56466	-4.71223	-4.41624
C	-4.80831	-3.13465	-2.87202
H	-5.83409	-2.91093	-3.13539
C	-4.20800	-2.46270	-1.88290
H	-4.76134	-1.68849	-1.36935
C	-2.79703	-2.76423	-1.51476
C	-2.10288	-2.03214	-0.41158
C	1.40734	-3.46073	-0.76611
C	1.90963	-3.65707	0.61880
C	2.28779	-3.44067	-1.78534
C	3.21918	-3.81198	0.84780
C	3.73294	-3.61119	-1.52692
C	4.17470	-3.79003	-0.27701
H	3.58097	-3.95470	1.85781
H	4.43341	-3.58661	-2.35156
C	-2.85812	2.67007	1.22871
C	-3.67871	3.60898	0.72209
C	-1.56294	3.08231	1.82244
C	-3.28737	5.03393	0.75180
C	-1.21032	4.37326	1.84559
C	-2.11687	5.39694	1.28918
H	-3.95064	5.78300	0.33915
H	-0.26680	4.67139	2.28416
H	2.57790	0.75501	1.19219
C	4.17031	-0.38030	1.82453
C	3.93441	0.66094	-0.36131
C	3.61503	-0.60648	3.09798
C	5.37745	-1.01457	1.44298
C	5.14025	-0.00808	-0.76559
C	4.27416	-1.44448	3.98071
H	2.67141	-0.13767	3.35751

C	6.02863	-1.85494	2.37035
C	5.83704	-0.78848	0.10763
H	5.48770	0.14705	-1.77698
C	5.48705	-2.06711	3.62482
H	3.84545	-1.62614	4.96140
H	6.95492	-2.34043	2.07646
H	6.76115	-1.26258	-0.21235
H	5.98858	-2.71831	4.33309
C	3.09311	1.42903	-1.22143
H	1.84816	0.52456	-1.22116
C	2.49217	2.74965	-0.74251
H	3.22279	3.55197	-0.91345
H	2.32371	2.69126	0.34386
N	1.27869	3.07395	-1.46190
N	3.53185	0.44877	0.92709
C	0.64996	4.30574	-1.40291
C	1.21883	5.42605	-0.76744
C	-0.60250	4.46366	-2.03483
C	0.57060	6.66130	-0.80448
H	2.16310	5.33799	-0.24201
C	-1.23648	5.69857	-2.05923
H	-1.06361	3.60290	-2.51280
C	-0.65363	6.81597	-1.45197
H	1.03314	7.51100	-0.30911
H	-2.19915	5.78925	-2.55543
H	-1.15271	7.77948	-1.47110
H	0.69681	2.29064	-1.72126
Cl	3.66931	1.56402	-2.92639
H	5.23307	-3.91252	-0.08650
H	-1.83119	6.44041	1.31597
H	-4.63100	3.33384	0.28506
H	-0.91279	2.34435	2.26938
H	1.21720	-3.68645	1.45067
H	1.95485	-3.27566	-2.80277

Catalyst (Ph, 5)-Enamine protonation TS (pro-R) alternative conformation
Electronic energy (Hartrees): -1870.572085
Free Energy at 1M concentration and 253 K (Hartrees): -1869.866751
Coordinates:

P	0.19645	-0.09392	-0.16038
O	1.28300	0.84054	0.62688
O	1.04363	-1.31092	-0.84137
O	-0.30833	0.67781	-1.41355
O	-0.82744	-0.52010	0.84633
C	2.40113	1.13701	-0.11169
C	2.57594	2.51511	-0.63901
C	3.64094	2.79817	-1.40454
H	3.77787	3.79772	-1.80090
C	4.63715	1.75333	-1.72330
C	5.78314	2.07956	-2.60370
H	5.90998	3.08679	-2.98081
C	6.66479	1.13190	-2.93930
H	7.50074	1.37129	-3.58382
C	6.49348	-0.24651	-2.44309
H	7.19835	-1.01370	-2.73670
C	5.46473	-0.55139	-1.64373
H	5.34786	-1.57403	-1.31178
C	4.49136	0.49844	-1.23523
C	3.33357	0.18532	-0.34437
C	2.00857	-1.86039	-0.03314

C	1.81815	-3.24076	0.48646
C	2.75740	-3.78135	1.28058
H	2.63757	-4.78919	1.66106
C	3.96361	-3.01358	1.65271
C	4.95731	-3.61320	2.57371
H	4.81812	-4.62188	2.94249
C	6.02914	-2.91135	2.95647
H	6.75350	-3.34999	3.63059
C	6.22091	-1.53166	2.47163
H	7.07911	-0.96118	2.80279
C	5.33221	-0.98222	1.63580
H	5.48316	0.03871	1.31234
C	4.14938	-1.76080	1.17535
C	3.14396	-1.17305	0.23976
C	0.59509	-4.00549	0.13894
C	0.14411	-4.08666	-1.27516
C	-0.13407	-4.61506	1.09390
C	-0.97671	-4.74660	-1.59152
C	-1.36729	-5.34797	0.73837
C	-1.76809	-5.40906	-0.53660
H	-1.30114	-4.80356	-2.62252
H	-1.94803	-5.83103	1.51348
C	1.55932	3.55479	-0.35737
C	1.14972	3.79300	0.90289
C	0.95273	4.32457	-1.47374
C	0.11076	4.80812	1.17056
C	0.00278	5.23566	-1.22749
C	-0.43694	5.49081	0.15888
H	-0.20857	4.99417	2.18756
H	-0.44583	5.78710	-2.04358
H	-2.16437	0.67251	0.92516
C	-2.65902	2.43161	1.86538
C	-3.11590	1.87388	-0.45592
C	-2.11028	2.02976	3.09762
C	-3.18426	3.73659	1.70169
C	-3.60923	3.20892	-0.64023
C	-2.10863	2.92138	4.15582
H	-1.68433	1.03603	3.19150
C	-3.17580	4.61742	2.80424
C	-3.65853	4.08864	0.40024
H	-3.96165	3.48578	-1.62364
C	-2.64811	4.21614	4.01728
H	-1.68277	2.61675	5.10677
H	-3.58014	5.61776	2.67844
H	-4.06070	5.08621	0.24467
H	-2.63993	4.89787	4.86132
N	-2.67826	1.56123	0.79747
C	-2.94181	0.89894	-1.48750
C	-3.28913	-0.55774	-1.23046
H	-2.78682	-0.87617	-0.31565
H	-2.86129	-1.16973	-2.03999
N	-4.72705	-0.76330	-1.07777
H	-5.25838	-0.31393	-1.81375
C	-5.24456	-2.00789	-0.71217
C	-6.59962	-2.28939	-0.98054
C	-4.48480	-2.98063	-0.03524
C	-7.17413	-3.48853	-0.57602
H	-7.19908	-1.54925	-1.50603
C	-5.07713	-4.17699	0.37073
H	-3.43626	-2.80923	0.18160

C	-6.41911	-4.44612	0.10694
H	-8.22115	-3.67656	-0.79838
H	-4.47335	-4.90906	0.89986
H	-6.86834	-5.38223	0.42305
H	-1.41187	0.82206	-1.51236
Cl	-3.52875	1.41899	-3.12321
H	-2.67199	-5.94388	-0.79790
H	-1.20370	6.22936	0.35405
H	0.72672	-3.62256	-2.06094
H	0.15821	-4.55970	2.13543
H	1.57838	3.25218	1.73769
H	1.26282	4.13833	-2.49442

Catalyst (Ph, 5)-Product (S) lowest free energy conformation
Electronic energy (Hartrees): -1870.612622
Free Energy at 1M concentration and 253 K (Hartrees): -1869.904323
Coordinates:

P	0.24183	-0.66184	-1.08113
O	1.70219	-0.60171	-1.79160
O	0.28819	0.32765	0.23018
O	0.17316	-2.06802	-0.36933
O	-0.80882	-0.32812	-2.07299
C	2.76866	-0.61677	-0.92480
C	3.65800	-1.80909	-0.88984
C	4.68941	-1.83904	-0.02979
H	5.35038	-2.69753	0.00164
C	4.93778	-0.71058	0.88913
C	6.04647	-0.79493	1.86824
H	6.69388	-1.66292	1.88374
C	6.25139	0.19819	2.73958
H	7.06054	0.13462	3.45563
C	5.36648	1.37788	2.73667
H	5.52323	2.16771	3.45999
C	4.36602	1.46670	1.85278
H	3.71657	2.33060	1.88817
C	4.14300	0.38372	0.85483
C	3.03270	0.45690	-0.14286
C	0.84437	1.56029	-0.00264
C	-0.00314	2.78238	0.10472
C	0.54308	3.98217	-0.15807
H	-0.04893	4.88567	-0.07135
C	1.96436	4.09908	-0.54300
C	2.52573	5.42741	-0.88312
H	1.90194	6.31116	-0.83158
C	3.80443	5.54129	-1.25778
H	4.21297	6.51192	-1.50772
C	4.65991	4.34243	-1.34166
H	5.68902	4.43901	-1.66293
C	4.16395	3.13890	-1.03203
H	4.80420	2.27120	-1.11885
C	2.74964	2.99913	-0.58939
C	2.17210	1.66857	-0.23904
H	-0.57045	-2.11138	0.35199
C	-1.33493	-1.60825	2.71048
C	-2.98805	-2.19595	1.17228
C	0.04041	-1.41484	2.99484
C	-2.30452	-1.36064	3.72778
C	-4.01009	-1.98109	2.13334
C	0.42847	-0.98117	4.24271
H	0.76421	-1.60095	2.21056

C	-1.87065	-0.91645	5.00236
C	-3.66762	-1.57023	3.39565
H	-5.04428	-2.15655	1.86204
C	-0.53014	-0.72833	5.25358
H	1.48220	-0.82911	4.45613
H	-2.61261	-0.72554	5.77260
H	-4.43176	-1.39563	4.14811
H	-0.20242	-0.38499	6.22985
C	-3.32009	-2.65490	-0.23136
C	-4.27636	-1.73389	-1.00033
H	-5.19550	-1.61799	-0.41885
H	-4.55614	-2.23496	-1.94076
N	-3.64279	-0.44410	-1.19789
N	-1.70754	-2.02670	1.46006
C	-4.35885	0.61029	-1.78168
C	-3.66608	1.53305	-2.59119
C	-5.73321	0.81131	-1.56461
C	-4.32681	2.62426	-3.14463
H	-2.60610	1.37762	-2.76972
C	-6.38347	1.90924	-2.12860
H	-6.29935	0.12194	-0.94697
C	-5.69180	2.82502	-2.92024
H	-3.76848	3.32253	-3.76253
H	-7.44567	2.04521	-1.94290
H	-6.20496	3.67641	-3.35630
H	-2.69857	-0.51150	-1.57832
H	-2.39432	-2.79002	-0.79117
Cl	-4.06940	-4.32884	-0.14594
C	-1.42728	2.69373	0.52092
C	-1.79912	1.92015	1.55992
C	-2.46200	3.50270	-0.17778
C	-3.21448	1.85977	1.97998
H	-1.06852	1.35624	2.12513
C	-3.74078	3.45409	0.21558
H	-2.19043	4.12642	-1.02015
C	-4.13685	2.58603	1.34048
H	-3.50165	1.23632	2.81518
H	-4.49030	4.04069	-0.29949
H	-5.17350	2.54231	1.64825
C	3.40099	-2.95892	-1.78953
C	3.18385	-2.73305	-3.24191
C	3.34844	-4.21556	-1.30674
C	2.93036	-3.76498	-4.05626
H	3.24302	-1.73295	-3.65269
C	3.07007	-5.35155	-2.21007
H	3.48864	-4.41045	-0.25045
C	2.86877	-5.13741	-3.51528
H	2.77603	-3.59681	-5.11427
H	3.02569	-6.35691	-1.81165
H	2.66324	-5.96861	-4.17732

Catalyst (Ph, 5)-Product (S) alternative conformation
Electronic energy (Hartrees): -1870.602158
Free Energy at 1M concentration and 253 K (Hartrees): -1869.89482
Coordinates:

P	-0.51747	-0.64921	0.72315
O	-0.90434	0.46254	-0.42460
O	-1.87899	-0.94541	1.56496
O	0.57359	-0.23317	1.63258
O	-0.28551	-1.92564	-0.17858

C	-1.49404	1.57840	0.10903
C	-0.71998	2.84371	0.20416
C	-1.27691	3.91116	0.79827
H	-0.72688	4.84184	0.87628
C	-2.64661	3.83656	1.35077
C	-3.22443	5.01447	2.03880
H	-2.65826	5.93488	2.10990
C	-4.44639	4.94621	2.57780
H	-4.86669	5.80699	3.08170
C	-5.22115	3.69338	2.49796
H	-6.20143	3.63896	2.95366
C	-4.71080	2.62476	1.87512
H	-5.28869	1.71083	1.84685
C	-3.36373	2.69282	1.24593
C	-2.76971	1.51471	0.54717
C	-3.05575	-0.91441	0.85180
C	-3.84191	-2.17092	0.70889
C	-4.99071	-2.15768	0.01506
H	-5.57198	-3.06582	-0.09663
C	-5.48082	-0.91328	-0.60926
C	-6.72571	-0.93976	-1.41221
H	-7.29136	-1.85817	-1.50891
C	-7.15552	0.16870	-2.02367
H	-8.06419	0.14767	-2.61129
C	-6.38481	1.42079	-1.91061
H	-6.72563	2.30890	-2.42695
C	-5.26200	1.45678	-1.18389
H	-4.70278	2.38115	-1.13738
C	-4.78576	0.23904	-0.47015
C	-3.53588	0.25205	0.34971
H	0.55147	-1.79543	-0.75936
C	1.55119	-0.95436	-2.96284
C	3.08779	-1.39438	-1.26266
C	0.20036	-0.90468	-3.39175
C	2.58796	-0.55754	-3.86055
C	4.17526	-1.01620	-2.09562
C	-0.09984	-0.47306	-4.66452
H	-0.57827	-1.19430	-2.69594
C	2.24359	-0.12413	-5.16609
C	3.92119	-0.60640	-3.37999
H	5.17981	-1.06092	-1.69707
C	0.92513	-0.08176	-5.55949
H	-1.13559	-0.42950	-4.98722
H	3.03653	0.17689	-5.84509
H	4.73486	-0.31218	-4.03763
H	0.66573	0.25462	-6.55854
C	3.30661	-1.79499	0.18175
C	3.68728	-0.62170	1.10740
H	3.87136	-1.02344	2.10656
H	2.78250	-0.00785	1.18571
N	4.81961	0.17692	0.66691
N	1.83345	-1.37564	-1.68915
C	5.97692	0.36363	1.43273
C	6.68117	1.57690	1.31808
C	6.48937	-0.63044	2.28399
C	7.85663	1.78865	2.03182
H	6.29282	2.35820	0.66747
C	7.66059	-0.39997	3.00442
H	5.98963	-1.58926	2.36164
C	8.35551	0.80427	2.88757

H	8.37890	2.73558	1.92562
H	8.03715	-1.18216	3.65790
H	9.26865	0.97215	3.44961
H	4.54066	1.02191	0.19141
H	2.39027	-2.23670	0.57181
Cl	4.55632	-3.13675	0.25727
C	-3.35185	-3.43945	1.29795
C	-2.96916	-3.50015	2.58740
C	-3.28162	-4.66769	0.46387
C	-2.47601	-4.76984	3.16087
H	-3.03048	-2.62817	3.22678
C	-2.83449	-5.81577	0.98762
H	-3.58004	-4.63677	-0.57667
C	-2.41155	-5.86935	2.40125
H	-2.16834	-4.80771	4.19789
H	-2.77926	-6.70688	0.37575
H	-2.05044	-6.79957	2.82051
C	0.66001	2.92132	-0.33082
C	1.75687	3.46365	0.51247
C	0.94010	2.49892	-1.57861
C	3.00637	3.52877	0.03590
H	1.55346	3.79448	1.52328
C	2.31903	2.58128	-2.10259
H	0.16031	2.11101	-2.22253
C	3.30036	3.06932	-1.33626
H	3.80531	3.91437	0.65601
H	2.53170	2.25178	-3.11064
H	4.31137	3.12627	-1.71843

Catalyst (Ph, 5)-Product (S) alternative conformation
Electronic energy (Hartrees): -1870.602681
Free Energy at 1M concentration and 253 K (Hartrees): -1869.893829
Coordinates:

P	-0.31573	0.60487	-0.87749
O	-1.76082	0.57245	-1.62360
O	-0.46886	-0.27915	0.49824
O	-0.17930	2.05141	-0.25787
O	0.74584	0.14487	-1.80060
C	-2.86400	0.60365	-0.80273
C	-3.76300	1.78825	-0.85371
C	-4.84633	1.83202	-0.06122
H	-5.51346	2.68579	-0.09405
C	-5.14421	0.72657	0.87014
C	-6.31255	0.82878	1.77564
H	-6.96558	1.69128	1.72614
C	-6.56477	-0.14114	2.66048
H	-7.41741	-0.06440	3.32278
C	-5.67284	-1.31217	2.74687
H	-5.86823	-2.08206	3.48222
C	-4.61836	-1.41693	1.93002
H	-3.96569	-2.27291	2.03250
C	-4.34145	-0.36153	0.91563
C	-3.17176	-0.45470	-0.01129
C	-0.98932	-1.52871	0.28656
C	-0.11940	-2.72683	0.44877
C	-0.63367	-3.94420	0.20377
H	-0.02454	-4.83185	0.32694
C	-2.03915	-4.09910	-0.22728
C	-2.56478	-5.44766	-0.54373
H	-1.93038	-6.31842	-0.43383

C	-3.82408	-5.59614	-0.96867
H	-4.20647	-6.58174	-1.20075
C	-4.69306	-4.41549	-1.13359
H	-5.70509	-4.54067	-1.49647
C	-4.22978	-3.19352	-0.84644
H	-4.87815	-2.34017	-0.99366
C	-2.83951	-3.01461	-0.34620
C	-2.29938	-1.66348	-0.01547
H	0.69157	2.10924	0.27216
C	1.89754	1.93767	2.56035
C	3.28974	2.05735	0.69172
C	0.58561	1.80908	3.08538
C	3.00891	2.00049	3.45560
C	4.44116	2.14715	1.51632
C	0.39602	1.74151	4.44714
H	-0.24579	1.74549	2.39316
C	2.77682	1.93257	4.85312
C	4.29824	2.12165	2.88120
H	5.42198	2.23957	1.06671
C	1.49530	1.80468	5.33843
H	-0.60821	1.63517	4.84603
H	3.62560	1.97905	5.52984
H	5.16710	2.18609	3.53079
H	1.32170	1.74937	6.40865
C	3.37291	2.05659	-0.81908
C	4.67316	1.51040	-1.43503
H	5.48643	2.21487	-1.23135
H	4.53899	1.49136	-2.52214
N	5.04252	0.19274	-0.93080
N	2.07071	1.97946	1.20485
C	4.85138	-0.97114	-1.69913
C	5.85404	-1.95575	-1.72107
C	3.66412	-1.19337	-2.41505
C	5.67428	-3.13587	-2.43839
H	6.77895	-1.79008	-1.17196
C	3.50276	-2.37453	-3.13842
H	2.84412	-0.48584	-2.37144
C	4.49794	-3.35280	-3.15799
H	6.46235	-3.88389	-2.43928
H	2.57230	-2.53215	-3.67629
H	4.35777	-4.27159	-3.71895
H	5.93969	0.19236	-0.46666
H	2.50965	1.52191	-1.21676
Cl	3.17410	3.78820	-1.41948
C	1.29918	-2.58650	0.87148
C	1.64617	-1.75489	1.87441
C	2.36083	-3.38523	0.20240
C	3.06027	-1.61785	2.27994
H	0.89852	-1.18990	2.41591
C	3.63680	-3.27605	0.59125
H	2.11024	-4.05713	-0.60884
C	4.00599	-2.33951	1.66998
H	3.32764	-0.93670	3.07690
H	4.40329	-3.86726	0.10845
H	5.04219	-2.24004	1.96606
C	-3.45958	2.91690	-1.76565
C	-3.16652	2.65747	-3.19895
C	-3.43338	4.18450	-1.31033
C	-2.87052	3.67046	-4.02268
H	-3.20394	1.64796	-3.58872

C	-3.10776	5.29950	-2.22401
H	-3.62980	4.40360	-0.26782
C	-2.83765	5.05521	-3.51140
H	-2.66068	3.47786	-5.06689
H	-3.08458	6.31395	-1.84743
H	-2.59750	5.87101	-4.18092

Catalyst (Ph, 5)-Product (R) lowest free energy conformation
Electronic energy (Hartrees): -1870.60687
Free Energy at 1M concentration and 253 K (Hartrees): -1869.898373
Coordinates:

P	-0.04494	0.33950	0.12392
O	-0.71445	-1.09926	0.50113
O	-1.25704	1.29111	-0.42531
O	0.76257	0.11107	-1.21535
O	0.71017	0.86055	1.28900
C	-1.56141	-1.60414	-0.45507
C	-1.15208	-2.80952	-1.22439
C	-1.94574	-3.26343	-2.20768
H	-1.66458	-4.14302	-2.77521
C	-3.22077	-2.58447	-2.52083
C	-4.05736	-3.07612	-3.64030
H	-3.74873	-3.94396	-4.20983
C	-5.19621	-2.44958	-3.95450
H	-5.80585	-2.80886	-4.77351
C	-5.61985	-1.25928	-3.19307
H	-6.53256	-0.74513	-3.46534
C	-4.87424	-0.80961	-2.17715
H	-5.19442	0.07681	-1.64628
C	-3.61835	-1.51219	-1.79676
C	-2.76688	-1.02963	-0.66923
C	-2.42197	1.20178	0.29719
C	-2.84637	2.35051	1.14028
C	-3.98448	2.26637	1.84828
H	-4.30788	3.09864	2.46296
C	-4.80754	1.04099	1.80954
C	-6.03277	0.95633	2.63816
H	-6.34310	1.80245	3.23860
C	-6.75987	-0.16559	2.65108
H	-7.65393	-0.22678	3.25807
C	-6.33796	-1.32876	1.84867
H	-6.91823	-2.24187	1.88157
C	-5.23577	-1.26129	1.09312
H	-4.93499	-2.13616	0.53325
C	-4.43177	-0.00856	1.04254
C	-3.19889	0.09631	0.20435
C	-2.01217	3.57185	1.21374
C	-1.60076	4.26816	-0.03179
C	-1.62221	4.06851	2.40340
C	-0.85917	5.38137	0.03157
C	-0.78898	5.28732	2.46639
C	-0.42640	5.91077	1.33972
H	-0.58005	5.90186	-0.87457
H	-0.47380	5.67438	3.42674
C	0.11641	-3.50927	-0.90865
C	0.42193	-3.84080	0.36062
C	1.06386	-3.86590	-1.99655
C	1.67914	-4.55441	0.66496
C	2.20390	-4.50971	-1.71567
C	2.52396	-4.87548	-0.32120

H	1.91390	-4.81673	1.68844
H	2.89563	-4.76075	-2.50928
C	2.87416	2.67917	-1.52258
C	4.28015	0.97527	-0.77829
C	1.62682	3.07291	-2.07564
C	3.88039	3.67147	-1.30536
C	5.32460	1.90385	-0.53231
C	1.40321	4.39147	-2.40036
H	0.86364	2.32329	-2.24564
C	3.60581	5.02534	-1.62840
C	5.12125	3.23777	-0.78080
H	6.27355	1.54391	-0.15068
C	2.39113	5.37855	-2.16873
H	0.45238	4.67877	-2.83833
H	4.37448	5.77224	-1.45074
H	5.90580	3.96521	-0.59076
H	2.18604	6.41400	-2.42182
C	4.57068	-0.49729	-0.56855
H	5.46825	-0.59160	0.04340
C	3.46089	-1.36036	0.03843
H	3.82352	-2.38841	0.05468
H	2.58024	-1.34998	-0.61046
N	3.14659	-0.92047	1.38319
N	3.09459	1.35646	-1.23338
C	3.20691	-1.78717	2.47823
C	4.15361	-2.82322	2.57564
C	2.30576	-1.60120	3.54508
C	4.17689	-3.65554	3.69442
H	4.88234	-2.97709	1.78566
C	2.34991	-2.42990	4.66088
H	1.56373	-0.81106	3.47200
C	3.28009	-3.46974	4.74593
H	4.91418	-4.45257	3.74279
H	1.63914	-2.26920	5.46721
H	3.30584	-4.11933	5.61521
H	2.37945	-0.25338	1.43564
Cl	5.04455	-1.21332	-2.20593
H	0.18214	6.80472	1.38413
H	3.44719	-5.39433	-0.09825
H	-1.91783	3.89182	-0.99641
H	-1.90093	3.57665	3.32745
H	-0.25582	-3.61189	1.17368
H	0.84291	-3.59442	-3.02124
H	1.70781	0.52286	-1.22559

Catalyst (Ph, 5)-Product (R) alternative conformation
Electronic energy (Hartrees): -1870.60382
Free Energy at 1M concentration and 253 K (Hartrees): -1869.894861
Coordinates:

P	0.67237	-1.61572	0.78868
O	1.79861	-1.31075	-0.37470
O	0.65900	-0.28988	1.75581
O	0.87726	-2.82757	1.59611
O	-0.65644	-1.58454	-0.07076
C	3.02820	-1.02006	0.16237
C	4.12353	-2.02548	0.08827
C	5.29661	-1.76442	0.68829
H	6.10798	-2.48131	0.63910
C	5.50395	-0.49838	1.42157
C	6.78756	-0.25798	2.12122

H	7.58193	-0.99209	2.06817
C	6.97062	0.86510	2.82369
H	7.90827	1.03894	3.33564
C	5.89004	1.86522	2.91393
H	6.03677	2.76194	3.50210
C	4.72874	1.66020	2.28176
H	3.94291	2.39701	2.38024
C	4.52055	0.42970	1.47035
C	3.23538	0.17686	0.75359
C	0.92282	0.92134	1.15719
C	-0.14219	1.96275	1.13276
C	0.12438	3.17048	0.60795
H	-0.63659	3.94236	0.59588
C	1.45052	3.47216	0.04006
C	1.69610	4.79650	-0.57705
H	0.92002	5.55168	-0.57176
C	2.87230	5.06179	-1.15393
H	3.04824	6.02715	-1.61048
C	3.92748	4.03257	-1.18600
H	4.86807	4.24402	-1.67798
C	3.71800	2.83602	-0.62517
H	4.49737	2.08961	-0.69033
C	2.42423	2.53426	0.04968
C	2.16369	1.20724	0.68818
H	-0.70431	-1.02568	-0.90674
C	-0.84588	1.10794	-2.53994
C	-2.74191	-0.17883	-2.12094
C	0.56768	1.18080	-2.62688
C	-1.63611	2.22822	-2.93938
C	-3.59951	0.89352	-2.47940
C	1.16465	2.32253	-3.11411
H	1.15098	0.31711	-2.32301
C	-0.98588	3.40162	-3.39922
C	-3.04580	2.08810	-2.86782
H	-4.67140	0.75596	-2.43693
C	0.38701	3.44373	-3.49255
H	2.24528	2.36890	-3.20644
H	-1.58870	4.25672	-3.69188
H	-3.67919	2.92816	-3.13932
H	0.88090	4.33861	-3.85799
C	-3.26142	-1.55461	-1.74139
H	-2.41823	-2.24173	-1.74225
C	-3.96664	-1.68572	-0.39094
H	-3.26573	-1.29479	0.35553
H	-4.08921	-2.76039	-0.18109
N	-5.21450	-0.95081	-0.32550
N	-1.42225	-0.05759	-2.11531
C	-5.98205	-1.00905	0.85287
C	-5.50068	-1.56434	2.04936
C	-7.27764	-0.46017	0.83848
C	-6.29563	-1.55428	3.19769
H	-4.50837	-1.99772	2.09764
C	-8.06291	-0.46504	1.98502
H	-7.65563	-0.02057	-0.08147
C	-7.57731	-1.00938	3.17799
H	-5.90038	-1.98369	4.11390
H	-9.06028	-0.03570	1.94836
H	-8.18997	-1.00749	4.07385
H	-5.78399	-1.11034	-1.14952
Cl	-4.36011	-2.16916	-3.08864

C	3.92625	-3.30707	-0.63067
C	3.38928	-3.32975	-1.86561
C	4.32855	-4.58904	0.00582
C	3.19595	-4.61275	-2.57295
H	3.10778	-2.41172	-2.36666
C	4.15230	-5.74887	-0.63926
H	4.75462	-4.59297	1.00135
C	3.55844	-5.76150	-1.99091
H	2.76222	-4.61979	-3.56457
H	4.44155	-6.68059	-0.17049
H	3.41782	-6.70150	-2.50868
C	-1.48699	1.68465	1.69158
C	-1.62511	1.12186	3.05944
C	-2.59869	1.98346	0.99147
C	-2.83858	0.93342	3.59290
H	-0.74518	0.88564	3.64417
C	-3.93484	1.76954	1.58222
H	-2.53042	2.39704	-0.00605
C	-4.04828	1.28421	2.82315
H	-2.93618	0.53315	4.59372
H	-4.82108	2.01777	1.01254
H	-5.02648	1.14415	3.26392

Catalyst (4-i-Pr, 6)-THF complex
Electronic energy (Hartrees): -876.463823
Free Energy at 1M concentration and 253 K (Hartrees): -875.741327
Coordinates:

P	0.00231	-0.89518	-0.18491
O	0.84362	0.21592	0.67867
O	-1.36783	-0.16846	-0.70080
O	0.74060	-1.47833	-1.32799
O	-0.43692	-1.87082	0.97507
C	1.05926	1.37424	-0.02066
C	2.42248	1.68762	-0.53259
C	2.61102	2.80686	-1.25125
H	3.59723	3.05938	-1.62321
C	1.48319	3.71274	-1.54929
C	1.70932	4.91070	-2.39132
H	2.70329	5.14094	-2.75438
C	0.68724	5.71181	-2.70962
H	0.85328	6.58565	-3.32633
C	-0.67305	5.39860	-2.23364
H	-1.50035	6.03693	-2.51599
C	-0.88720	4.32225	-1.46827
H	-1.89799	4.09942	-1.15516
C	0.24563	3.43857	-1.07626
C	0.03626	2.23203	-0.21991
C	-1.93073	0.77921	0.12551
C	-3.35462	0.61493	0.53341
C	-3.95637	1.56719	1.26188
H	-4.99583	1.45809	1.55037
C	-3.20835	2.75880	1.70435
C	-3.86261	3.74977	2.59027
H	-4.89432	3.61409	2.89018
C	-3.18175	4.81515	3.02556
H	-3.66002	5.53793	3.67393
C	-1.77229	5.00241	2.63293
H	-1.22050	5.85594	3.00522
C	-1.17420	4.11556	1.82907
H	-0.13376	4.26234	1.57150

C	-1.92715	2.93920	1.31252
C	-1.28674	1.94304	0.40040
C	-4.13598	-0.55490	0.08288
C	-5.32982	-0.35872	-0.77293
C	-3.79310	-1.79686	0.45848
C	-6.04959	-1.41068	-1.18091
C	-4.58436	-2.95934	0.00747
C	-5.66615	-2.78779	-0.77816
H	-6.91668	-1.24825	-1.80873
H	-4.27796	-3.94761	0.32470
C	3.56575	0.77929	-0.26638
C	3.79984	0.30569	0.97034
C	4.49471	0.41228	-1.36451
C	4.97167	-0.55391	1.23530
C	5.55789	-0.36343	-1.12456
C	5.82470	-0.87549	0.24495
H	5.13940	-0.90070	2.24733
H	6.22470	-0.61117	-1.94001
H	0.12893	-2.70615	1.01618
O	1.17705	-3.88568	1.12214
C	2.24682	-3.51602	2.03070
C	1.73361	-4.29331	-0.16218
C	3.54032	-3.96061	1.34799
H	2.21859	-2.42888	2.17650
H	2.06175	-4.00542	2.99133
C	3.18199	-3.81498	-0.13925
H	1.66100	-5.38578	-0.22995
H	1.13962	-3.82463	-0.94781
H	3.75893	-5.00728	1.58719
H	4.39778	-3.35987	1.65879
H	3.82451	-4.40208	-0.80054
H	3.22541	-2.76714	-0.45307
H	-5.62028	0.63945	-1.07676
H	-2.95246	-1.96128	1.11697
H	4.31409	0.76867	-2.37102
H	3.15930	0.57756	1.79991
C	7.05662	-1.71404	0.53063
H	7.05894	-2.03032	1.59824
C	8.33129	-0.89489	0.30029
H	9.22411	-1.49070	0.58686
H	8.42964	-0.60012	-0.76624
H	8.31192	0.02301	0.92612
C	7.07070	-2.99237	-0.31595
H	7.14869	-2.75855	-1.39857
H	7.93696	-3.62657	-0.03050
H	6.14548	-3.57792	-0.14154
C	-6.49740	-3.96422	-1.25313
H	-7.33129	-3.58570	-1.88504
C	-5.66919	-4.91496	-2.12616
H	-4.85843	-5.39550	-1.53847
H	-6.32106	-5.71150	-2.54424
H	-5.21674	-4.35660	-2.97351
C	-7.13213	-4.70988	-0.07281
H	-7.72642	-4.00504	0.54730
H	-7.81452	-5.50267	-0.44702
H	-6.35696	-5.18454	0.56548

Catalyst (4-i-Pr, 6)-Quinoline complex
Electronic energy (Hartrees): -1582.948819
Free Energy at 1M concentration and 253 K (Hartrees): -1582.187912

Coordinates:

P	0.30856	-0.39695	-1.18470
O	-0.54319	0.55244	-0.13043
O	1.69607	0.39717	-1.50397
O	-0.40922	-0.72693	-2.43136
O	0.72013	-1.56866	-0.20825
C	-0.59610	1.87665	-0.48263
C	-1.89514	2.47323	-0.90059
C	-1.93708	3.77061	-1.24887
H	-2.87330	4.22832	-1.54615
C	-0.71445	4.60041	-1.22550
C	-0.77900	6.01413	-1.66399
H	-1.72260	6.44460	-1.97558
C	0.32765	6.76482	-1.67928
H	0.27945	7.79712	-2.00101
C	1.62027	6.18509	-1.26784
H	2.51534	6.79286	-1.29983
C	1.68887	4.91060	-0.86631
H	2.65040	4.50121	-0.58675
C	0.46166	4.06970	-0.81915
C	0.50982	2.64733	-0.37112
C	2.29555	0.95919	-0.40144
C	3.55017	0.36098	0.12813
C	4.11788	0.88207	1.22813
H	5.03372	0.45813	1.62363
C	3.49896	2.02668	1.92550
C	4.10905	2.53740	3.17516
H	5.02785	2.10302	3.54916
C	3.52581	3.53294	3.85042
H	3.97090	3.90320	4.76492
C	2.26593	4.12046	3.35848
H	1.78649	4.91039	3.92220
C	1.70994	3.67098	2.22761
H	0.77288	4.10173	1.90283
C	2.36197	2.58290	1.44715
C	1.75598	2.05305	0.18792
C	4.15046	-0.81932	-0.53695
C	4.36724	-0.81442	-2.00373
C	4.48038	-1.91525	0.16981
C	4.87302	-1.88994	-2.61794
C	5.04155	-3.10217	-0.50677
C	5.22989	-3.10551	-1.83946
H	5.02358	-1.86367	-3.68929
H	5.28934	-3.96991	0.09183
C	-3.13227	1.65070	-0.94492
C	-3.45138	0.82031	0.06538
C	-4.05120	1.75188	-2.10683
C	-4.68566	0.01080	0.01183
C	-5.17669	1.02984	-2.14870
C	-5.51959	0.10125	-1.04018
H	-4.91014	-0.64497	0.84362
H	-5.83421	1.12739	-3.00258
H	-0.00896	-1.82518	0.45773
C	-2.38620	-2.74458	0.91714
C	-1.15447	-2.15573	2.80833
C	-2.48822	-2.81012	-0.49768
C	-3.46878	-3.22754	1.71857
C	-2.17815	-2.63177	3.67115
C	-3.62188	-3.33335	-1.07773
H	-1.68340	-2.43477	-1.11937

C	-4.62189	-3.75647	1.08535
C	-3.32332	-3.15347	3.12513
H	-2.04551	-2.56415	4.74371
C	-4.69541	-3.81041	-0.28739
H	-3.69246	-3.37453	-2.15997
H	-5.44065	-4.11684	1.70186
H	-4.12666	-3.51106	3.76367
H	-5.57980	-4.21515	-0.76961
N	-1.25401	-2.22394	1.48587
C	0.06298	-1.51139	3.36816
C	0.63875	-0.41511	2.87545
H	1.52616	0.00958	3.32982
H	0.21023	0.07013	2.00599
Cl	0.72267	-2.27723	4.82090
H	4.12623	0.06639	-2.58572
H	4.31593	-1.95443	1.23988
H	-2.82619	0.74704	0.94637
H	-3.81283	2.40919	-2.93368
C	-6.80031	-0.71055	-1.07942
H	-6.83461	-1.40287	-0.20783
C	-6.87437	-1.57538	-2.34401
H	-7.74200	-2.26611	-2.27954
H	-6.99177	-0.94956	-3.25371
H	-5.95342	-2.18445	-2.44944
C	-8.02213	0.20819	-0.97667
H	-8.95266	-0.39772	-0.94128
H	-7.96604	0.81248	-0.04594
H	-8.07863	0.89570	-1.84755
C	5.79478	-4.32982	-2.53386
H	5.99662	-5.12964	-1.78593
C	7.12892	-4.00548	-3.21537
H	7.57122	-4.93002	-3.64412
H	6.99252	-3.26751	-4.03411
H	7.84431	-3.59084	-2.47321
C	4.78836	-4.89963	-3.53992
H	5.18021	-5.84286	-3.97694
H	3.82768	-5.12668	-3.02990
H	4.59583	-4.18171	-4.36525

Catalyst (4-i-Pr, 6)-Enamine protonation TS (pro-S) lowest free energy conformation
Electronic energy (Hartrees): -1870.559201
Free Energy at 1M concentration and 253 K (Hartrees): -1869.683299
Coordinates:

P	-0.87672	0.26094	-0.09130
O	-2.12573	-0.03834	-1.09522
O	-0.60765	-1.13265	0.72841
O	-1.12938	1.33476	0.91319
O	0.30426	0.46496	-1.09394
C	-3.20509	-0.58481	-0.44641
C	-4.39743	0.25960	-0.16758
C	-5.43198	-0.26372	0.50872
H	-6.30509	0.34121	0.72467
C	-5.38919	-1.66125	0.98774
C	-6.52403	-2.20038	1.77288
H	-7.39828	-1.58814	1.95624
C	-6.47338	-3.44395	2.26162
H	-7.30246	-3.83602	2.83644
C	-5.28072	-4.28061	2.03152
H	-5.23620	-5.27777	2.45022

C	-4.25208	-3.80541	1.31997
H	-3.37885	-4.42920	1.18616
C	-4.30598	-2.43493	0.74057
C	-3.17303	-1.88013	-0.06012
C	-0.74669	-2.28888	-0.00149
C	0.45562	-3.12751	-0.27515
C	0.30522	-4.28904	-0.93748
H	1.16058	-4.92662	-1.12811
C	-1.01653	-4.71172	-1.44006
C	-1.13373	-5.95965	-2.23045
H	-0.26287	-6.58098	-2.39886
C	-2.31400	-6.32324	-2.74271
H	-2.39797	-7.23407	-3.32136
C	-3.50261	-5.47586	-2.53320
H	-4.45015	-5.76319	-2.97048
C	-3.40830	-4.34981	-1.81679
H	-4.28893	-3.73338	-1.69785
C	-2.10809	-3.94783	-1.21138
C	-1.97710	-2.70375	-0.39568
C	1.81170	-2.68427	0.15351
C	2.03241	-2.12328	1.51150
C	2.86613	-2.79898	-0.67716
C	3.25450	-1.73531	1.89719
C	4.21885	-2.40372	-0.23885
C	4.41742	-1.88444	0.98674
H	3.39419	-1.34197	2.89603
H	5.04614	-2.55376	-0.91989
C	-4.41597	1.68387	-0.57414
C	-4.09476	2.04705	-1.82801
C	-4.74445	2.73414	0.42027
C	-4.04775	3.47381	-2.20603
C	-4.69779	4.02724	0.08011
C	-4.32517	4.43110	-1.30168
H	-3.77264	3.73106	-3.22133
H	-4.92865	4.77757	0.82501
H	0.29569	2.28609	1.46793
C	1.56812	1.89034	3.03579
C	2.20887	2.35426	0.74409
C	0.52797	1.74870	3.97334
C	2.90954	1.61487	3.40082
C	3.56774	2.05626	1.09745
C	0.83352	1.35278	5.26351
H	-0.49568	1.92607	3.66089
C	3.18708	1.22314	4.72773
C	3.90136	1.72507	2.37742
H	4.32200	2.12759	0.32863
C	2.16457	1.09529	5.64924
H	0.03301	1.23527	5.98738
H	4.21585	1.01404	5.00718
H	4.94041	1.53778	2.63448
H	2.38244	0.78961	6.66736
C	1.79030	2.61577	-0.59507
C	2.83933	2.69724	-1.69714
H	3.65245	3.38676	-1.43807
H	2.36296	3.10443	-2.59958
N	3.39546	1.36895	-1.91298
N	1.29127	2.28190	1.74481
C	4.64015	1.08766	-2.45961
C	4.89878	-0.22454	-2.90937
C	5.68477	2.02959	-2.53258

C	6.15562	-0.58588	-3.37679
H	4.09750	-0.95890	-2.88015
C	6.94303	1.65086	-3.00231
H	5.52189	3.05810	-2.23052
C	7.19642	0.34647	-3.42280
H	6.32249	-1.60668	-3.70964
H	7.73232	2.39676	-3.04345
H	8.17813	0.06294	-3.78797
H	2.69506	0.65257	-2.05330
H	0.92682	1.35115	-0.87756
Cl	0.50732	3.87661	-0.76625
H	2.74391	-3.18543	-1.68146
H	1.21208	-2.04781	2.21323
H	-5.00333	2.45940	1.43532
H	-3.86085	1.30163	-2.57816
C	5.80526	-1.51417	1.47571
H	5.71584	-1.01553	2.46628
C	6.66240	-2.76879	1.67651
H	6.83036	-3.29469	0.71233
H	7.64847	-2.49182	2.10681
H	6.15900	-3.46449	2.38160
C	6.50257	-0.52380	0.53284
H	7.43586	-0.14712	1.00267
H	6.76768	-1.00417	-0.43218
H	5.84426	0.34280	0.32837
C	-4.24681	5.89644	-1.68421
H	-3.95889	5.99306	-2.75548
C	-3.17051	6.61619	-0.86358
H	-3.06470	7.66561	-1.21243
H	-3.43027	6.62761	0.21625
H	-2.19080	6.10766	-0.99178
C	-5.60980	6.57882	-1.52252
H	-5.55617	7.62609	-1.88936
H	-6.37752	6.04131	-2.11934
H	-5.92757	6.59408	-0.45844

Catalyst (4-i-Pr, 6)-Enamine protonation TS (pro-S) alternative conformation
Electronic energy (Hartrees): -1870.558925
Free Energy at 1M concentration and 253 K (Hartrees): -1869.682848
Coordinates:

P	-0.88429	0.29213	-0.01257
O	-2.15382	0.08665	-1.01393
O	-0.65434	-1.15002	0.73072
O	-1.08848	1.32096	1.04848
O	0.29162	0.50714	-1.01895
C	-3.24312	-0.45987	-0.38198
C	-4.40570	0.40510	-0.04438
C	-5.45196	-0.12379	0.60947
H	-6.30409	0.49454	0.86718
C	-5.44963	-1.54651	1.00969
C	-6.59529	-2.09319	1.77365
H	-7.44914	-1.46571	1.99728
C	-6.57956	-3.36255	2.19361
H	-7.41648	-3.76017	2.75307
C	-5.41455	-4.22100	1.90871
H	-5.39773	-5.24036	2.27237
C	-4.37654	-3.73894	1.21561
H	-3.52360	-4.38051	1.04116
C	-4.39238	-2.33794	0.71177
C	-3.24876	-1.77505	-0.06777

C	-0.83578	-2.26050	-0.05840
C	0.33782	-3.11792	-0.39195
C	0.14498	-4.23781	-1.11246
H	0.97863	-4.88899	-1.34796
C	-1.19429	-4.59344	-1.62063
C	-1.35764	-5.79334	-2.47466
H	-0.50813	-6.43110	-2.68537
C	-2.55340	-6.09290	-2.99242
H	-2.67107	-6.96861	-3.61755
C	-3.71302	-5.22185	-2.72542
H	-4.67303	-5.45622	-3.16709
C	-3.57704	-4.13899	-1.95144
H	-4.43683	-3.50294	-1.79074
C	-2.25943	-3.80993	-1.33960
C	-2.08225	-2.61593	-0.46022
C	1.71197	-2.73591	0.03862
C	1.96712	-2.24964	1.41926
C	2.75129	-2.83743	-0.81264
C	3.20461	-1.91366	1.80472
C	4.12042	-2.50139	-0.37593
C	4.35022	-2.04878	0.87045
H	3.36894	-1.57398	2.81939
H	4.93373	-2.63993	-1.07598
C	-4.37971	1.85185	-0.36424
C	-4.03850	2.28109	-1.59186
C	-4.68372	2.84995	0.69056
C	-3.94002	3.72587	-1.87973
C	-4.59401	4.15950	0.43055
C	-4.19454	4.63460	-0.91795
H	-3.64577	4.03568	-2.87402
H	-4.80862	4.87183	1.21729
H	0.37065	2.19999	1.62165
C	1.65682	1.69619	3.14682
C	2.27406	2.25766	0.87065
C	0.62774	1.53285	4.09304
C	2.99650	1.37152	3.47600
C	3.63042	1.91031	1.18666
C	0.94312	1.06718	5.35731
H	-0.39591	1.74901	3.80606
C	3.28450	0.90887	4.77761
C	3.97517	1.50848	2.44321
H	4.37428	2.00143	0.40982
C	2.27325	0.76016	5.70838
H	0.15103	0.93305	6.08754
H	4.31201	0.66230	5.02982
H	5.01309	1.28410	2.67383
H	2.49904	0.40007	6.70679
C	1.84249	2.59385	-0.44758
C	2.87755	2.70310	-1.56028
H	3.71239	3.35735	-1.27927
H	2.39981	3.16747	-2.43391
N	3.39517	1.37339	-1.85044
N	1.37016	2.15789	1.88141
C	4.62310	1.08780	-2.43144
C	4.84060	-0.20723	-2.94786
C	5.69002	2.00606	-2.47806
C	6.07984	-0.57651	-3.45443
H	4.02147	-0.92217	-2.93928
C	6.93023	1.61946	-2.98773
H	5.55824	3.02264	-2.12484

C	7.14316	0.33103	-3.47463
H	6.21509	-1.58394	-3.83845
H	7.73745	2.34681	-3.00738
H	8.11106	0.04133	-3.87053
H	2.67431	0.68224	-2.01189
H	0.94152	1.36488	-0.77352
Cl	0.58941	3.89242	-0.53930
H	2.60453	-3.16903	-1.83312
H	1.15921	-2.18752	2.13651
H	-4.96009	2.52263	1.68518
H	-3.82398	1.57669	-2.38606
C	5.75446	-1.74099	1.35595
H	5.69243	-1.28777	2.37016
C	6.57793	-3.02720	1.48468
H	6.71747	-3.51130	0.49442
H	7.57734	-2.79867	1.91266
H	6.06448	-3.74118	2.16379
C	6.46730	-0.72673	0.45080
H	7.41629	-0.39782	0.92503
H	6.70727	-1.16831	-0.53902
H	5.83071	0.16591	0.29564
C	-4.06921	6.12323	-1.17915
H	-4.32940	6.67646	-0.24953
C	-5.04971	6.58420	-2.26453
H	-5.01194	7.68991	-2.36540
H	-4.80124	6.13220	-3.24815
H	-6.08654	6.29630	-1.98782
C	-2.62730	6.50338	-1.53842
H	-2.53647	7.60687	-1.62888
H	-1.93372	6.16245	-0.74003
H	-2.31926	6.04390	-2.50157

Catalyst (4-i-Pr, 6)-Enamine protonation TS (pro-S) alternative conformation
Electronic energy (Hartrees): -1870.557134
Free Energy at 1M concentration and 253 K (Hartrees): -1869.681193
Coordinates:

P	-0.73843	0.05261	0.09526
O	-1.93260	0.13187	-1.01543
O	-1.01948	-1.31257	0.94862
O	-0.72004	1.20060	1.04980
O	0.53611	-0.19749	-0.77130
C	-3.17978	0.02534	-0.45023
C	-4.02088	1.24245	-0.29344
C	-5.21666	1.14285	0.30860
H	-5.83979	2.02085	0.43387
C	-5.69194	-0.15522	0.83161
C	-6.99190	-0.22707	1.53872
H	-7.61113	0.65667	1.63003
C	-7.40549	-1.38203	2.07064
H	-8.35457	-1.43271	2.58850
C	-6.56221	-2.58763	1.96674
H	-6.89156	-3.51333	2.42085
C	-5.38875	-2.53515	1.32608
H	-4.77709	-3.42611	1.28495
C	-4.92921	-1.26571	0.69832
C	-3.62522	-1.17954	-0.02556
C	-1.49341	-2.38032	0.22357
C	-0.61631	-3.56688	0.01931
C	-1.10817	-4.64628	-0.61336
H	-0.48713	-5.52153	-0.76509

C	-2.47850	-4.64268	-1.16256
C	-2.96628	-5.82121	-1.91670
H	-2.34311	-6.70167	-2.01296
C	-4.17605	-5.80247	-2.48552
H	-4.53042	-6.66333	-3.03767
C	-5.02484	-4.60156	-2.37659
H	-5.99249	-4.58287	-2.86128
C	-4.59576	-3.53383	-1.69397
H	-5.22630	-2.65637	-1.65152
C	-3.26556	-3.55140	-1.02375
C	-2.76817	-2.38018	-0.24012
C	0.81224	-3.52475	0.42860
C	1.20238	-2.99554	1.75973
C	1.77934	-3.90937	-0.42554
C	2.49335	-2.85810	2.08460
C	3.20245	-3.78907	-0.05490
C	3.55903	-3.26700	1.13199
H	2.75347	-2.45733	3.05568
H	3.95031	-4.11088	-0.76850
C	-3.51794	2.56384	-0.73813
C	-2.99294	2.72567	-1.96528
C	-3.55452	3.72118	0.18918
C	-2.44602	4.03337	-2.38023
C	-3.05692	4.90542	-0.18476
C	-2.46525	5.08234	-1.53728
H	-2.02365	4.12580	-3.37306
H	-3.08597	5.73354	0.51131
H	0.76082	2.11985	1.27601
C	2.03208	2.16550	2.89230
C	2.67677	1.93993	0.56637
C	0.99038	2.32877	3.82561
C	3.36872	1.98318	3.32700
C	4.02958	1.71990	0.99500
C	1.29176	2.33091	5.17575
H	-0.02979	2.42478	3.46919
C	3.64174	2.00006	4.71186
C	4.35751	1.76506	2.31896
H	4.77954	1.52396	0.24243
C	2.61913	2.17479	5.62526
H	0.49143	2.45128	5.89936
H	4.66683	1.86561	5.04554
H	5.39232	1.63072	2.62290
H	2.83344	2.18432	6.68898
C	2.25360	1.84768	-0.79717
C	3.29504	1.65841	-1.88966
H	4.08014	2.42797	-1.84115
H	2.79947	1.77734	-2.86388
N	3.92079	0.35177	-1.73656
N	1.76059	2.16776	1.54350
C	4.99578	-0.06359	-2.52109
C	5.42081	-1.40477	-2.43442
C	5.70746	0.80834	-3.36473
C	6.51824	-1.85271	-3.15805
H	4.88187	-2.08574	-1.78258
C	6.81473	0.34683	-4.07912
H	5.39615	1.84081	-3.47503
C	7.23118	-0.97966	-3.98611
H	6.82139	-2.89261	-3.07190
H	7.34857	1.03912	-4.72459
H	8.08994	-1.33048	-4.54941

H	3.26509	-0.38224	-1.49883
H	1.27418	0.61684	-0.77548
Cl	1.02039	3.08559	-1.27901
H	1.53394	-4.27480	-1.41518
H	0.44413	-2.72086	2.48170
H	-3.96844	3.60799	1.18349
H	-2.96408	1.90288	-2.66892
C	5.02339	-3.09571	1.48626
H	5.66103	-3.47166	0.65428
C	5.36428	-1.61448	1.67707
H	4.80980	-1.18465	2.53807
H	6.45302	-1.49345	1.86089
H	5.10334	-1.04581	0.75952
C	5.38628	-3.90909	2.73355
H	6.47893	-3.84494	2.92383
H	4.85026	-3.53113	3.62978
H	5.12306	-4.97765	2.58077
C	-1.89177	6.42093	-1.96100
H	-1.49480	6.35099	-2.99895
C	-0.72007	6.82476	-1.05869
H	-0.25934	7.76428	-1.43178
H	-1.05624	6.98803	-0.01284
H	0.05751	6.03176	-1.06608
C	-2.97763	7.50271	-1.96720
H	-2.56300	8.45525	-2.36088
H	-3.81903	7.19340	-2.62363
H	-3.36860	7.68455	-0.94363

Catalyst (4-i-Pr, 6)-Enamine protonation TS (pro-S) alternative conformation
Electronic energy (Hartrees): -1870.556772
Free Energy at 1M concentration and 253 K (Hartrees): -1869.681015
Coordinates:

P	-0.74084	0.07404	0.16245
O	-1.94357	0.23812	-0.92964
O	-1.02890	-1.34009	0.92926
O	-0.70120	1.15793	1.18876
O	0.52281	-0.13443	-0.73032
C	-3.18801	0.10386	-0.36412
C	-4.02388	1.31318	-0.13153
C	-5.21847	1.18124	0.46728
H	-5.83981	2.05145	0.64477
C	-5.69685	-0.14381	0.91381
C	-6.99400	-0.25289	1.62137
H	-7.60871	0.62635	1.76926
C	-7.41063	-1.43631	2.08386
H	-8.35764	-1.51429	2.60214
C	-6.57350	-2.63706	1.90255
H	-6.90518	-3.58731	2.30082
C	-5.40271	-2.55072	1.26067
H	-4.79543	-3.44020	1.16211
C	-4.94007	-1.24729	0.70944
C	-3.63938	-1.12300	-0.01454
C	-1.51647	-2.35480	0.14017
C	-0.65127	-3.53306	-0.14687
C	-1.15749	-4.56555	-0.84340
H	-0.54558	-5.43465	-1.05561
C	-2.53145	-4.51498	-1.38095
C	-3.03512	-5.63857	-2.20528
H	-2.42074	-6.51650	-2.36217
C	-4.24848	-5.57290	-2.76288

H	-4.61448	-6.39355	-3.36630
C	-5.08539	-4.37398	-2.57128
H	-6.05603	-4.31580	-3.04680
C	-4.64189	-3.35590	-1.82497
H	-5.26396	-2.47738	-1.72218
C	-3.30745	-3.42800	-1.16688
C	-2.79408	-2.31389	-0.31331
C	0.78079	-3.52967	0.25249
C	1.18641	-3.08995	1.61131
C	1.73745	-3.86926	-0.63202
C	2.48120	-2.98817	1.93431
C	3.16438	-3.78902	-0.26547
C	3.53534	-3.34846	0.94970
H	2.75282	-2.65273	2.92679
H	3.90339	-4.07353	-1.00374
C	-3.52234	2.65814	-0.50399
C	-2.99042	2.88592	-1.71784
C	-3.57917	3.76946	0.47787
C	-2.45818	4.21907	-2.06494
C	-3.09850	4.97797	0.16340
C	-2.50347	5.22828	-1.17317
H	-2.03002	4.36443	-3.04813
H	-3.14265	5.77303	0.89715
H	0.79593	2.03440	1.45748
C	2.08233	1.95714	3.06071
C	2.70385	1.88293	0.71902
C	1.05042	2.06544	4.01274
C	3.42116	1.73451	3.46939
C	4.05825	1.62284	1.11867
C	1.36384	1.97331	5.35693
H	0.02807	2.19373	3.67315
C	3.70664	1.65498	4.84959
C	4.39879	1.57653	2.43950
H	4.79891	1.47049	0.34706
C	2.69375	1.77592	5.78230
H	0.57112	2.05088	6.09464
H	4.73339	1.48935	5.16350
H	5.43505	1.41319	2.72368
H	2.91750	1.71134	6.84216
C	2.26629	1.88522	-0.64334
C	3.29426	1.75739	-1.75714
H	4.08765	2.51505	-1.66899
H	2.78930	1.94298	-2.71605
N	3.90858	0.43785	-1.69494
N	1.79885	2.05319	1.71787
C	4.96488	0.06170	-2.52375
C	5.37982	-1.28535	-2.52493
C	5.66720	0.97686	-3.32846
C	6.45814	-1.69804	-3.29668
H	4.84832	-1.99952	-1.90315
C	6.75547	0.55004	-4.09196
H	5.36295	2.01645	-3.37069
C	7.16164	-0.78286	-4.08646
H	6.75385	-2.74347	-3.27857
H	7.28237	1.27526	-4.70623
H	8.00552	-1.10623	-4.68743
H	3.24819	-0.30299	-1.49407
H	1.27323	0.66866	-0.69035
Cl	1.04222	3.16495	-1.02950
H	1.48097	-4.16866	-1.64086

H	0.43659	-2.85311	2.35513
H	-3.99607	3.60325	1.46343
H	-2.95006	2.10059	-2.46241
C	5.00412	-3.21907	1.30352
H	5.63133	-3.54997	0.44488
C	5.36613	-1.75751	1.58495
H	4.82255	-1.37581	2.47509
H	6.45746	-1.66263	1.76875
H	5.10752	-1.12879	0.70671
C	5.36399	-4.11439	2.49433
H	6.45863	-4.07680	2.68025
H	4.83901	-3.78678	3.41655
H	5.08526	-5.16760	2.27636
C	-1.96133	6.60593	-1.50347
H	-2.11950	7.27573	-0.62922
C	-2.70959	7.23114	-2.68733
H	-2.36930	8.27735	-2.84177
H	-2.52889	6.66057	-3.62293
H	-3.80127	7.24843	-2.48137
C	-0.45116	6.56116	-1.76553
H	-0.06238	7.58922	-1.92694
H	0.07333	6.12747	-0.88752
H	-0.21734	5.95015	-2.66314

Catalyst (4-i-Pr, 6)-Enamine protonation TS (pro-R) lowest free energy conformation
Electronic energy (Hartrees): -1870.563001
Free Energy at 1M concentration and 253 K (Hartrees): -1869.686153
Coordinates:

P	0.08248	0.23376	0.01059
O	1.21253	1.20899	-0.63615
O	-1.14065	1.19572	0.50701
O	0.54401	-0.56248	1.18765
O	-0.40045	-0.56078	-1.24951
C	1.49889	2.30706	0.13845
C	2.79559	2.37369	0.86778
C	3.06680	3.44404	1.63206
H	4.00669	3.50891	2.16813
C	2.08551	4.53775	1.77673
C	2.39008	5.67978	2.67022
H	3.34567	5.73454	3.17684
C	1.48595	6.64660	2.85824
H	1.70896	7.47947	3.51253
C	0.17517	6.57293	2.18655
H	-0.56199	7.34569	2.36278
C	-0.10809	5.54847	1.37388
H	-1.08603	5.50445	0.91455
C	0.90069	4.48100	1.12617
C	0.61421	3.32899	0.21822
C	-1.48623	2.19628	-0.36902
C	-2.81153	2.14781	-1.05006
C	-3.15753	3.15114	-1.87758
H	-4.12503	3.15144	-2.36569
C	-2.22600	4.26236	-2.15502
C	-2.60705	5.31175	-3.12932
H	-3.58020	5.28629	-3.60379
C	-1.75040	6.29338	-3.43018
H	-2.02921	7.05840	-4.14328
C	-0.41678	6.32870	-2.80134
H	0.28061	7.11306	-3.06596

C	-0.06503	5.38863	-1.91663
H	0.92655	5.42274	-1.48588
C	-1.01946	4.30690	-1.54708
C	-0.65809	3.25317	-0.55321
C	-3.73870	1.00205	-0.83469
C	-3.96236	0.45441	0.52853
C	-4.39786	0.44672	-1.87003
C	-4.79197	-0.57858	0.71952
C	-5.34141	-0.66747	-1.65361
C	-5.53055	-1.17531	-0.42118
H	-4.94844	-0.95296	1.72315
H	-5.88264	-1.05707	-2.50559
C	3.76099	1.25031	0.80407
C	4.13051	0.71880	-0.37426
C	4.33800	0.70015	2.05590
C	5.09653	-0.39770	-0.42139
C	5.21027	-0.31284	2.01348
C	5.61593	-0.90407	0.71193
H	5.38640	-0.78987	-1.38666
H	5.61756	-0.69802	2.93915
H	-0.35781	-2.01956	1.44640
C	-2.01068	-2.61553	2.50731
C	-1.41358	-3.33961	0.26044
C	-1.68729	-1.82134	3.62340
C	-3.22937	-3.33458	2.46667
C	-2.65755	-4.05905	0.20984
C	-2.57158	-1.76151	4.68704
H	-0.76246	-1.25364	3.62179
C	-4.10351	-3.25986	3.57074
C	-3.50787	-4.07267	1.27390
H	-2.87892	-4.61267	-0.69123
C	-3.78078	-2.48401	4.66932
H	-2.32787	-1.14425	5.54629
H	-5.03815	-3.81264	3.53682
H	-4.42555	-4.65283	1.22477
H	-4.45843	-2.42523	5.51469
C	-0.50274	-3.20415	-0.82678
H	-0.56237	-1.62958	-1.09315
C	1.00176	-3.36620	-0.60630
H	1.24443	-4.43699	-0.61832
H	1.25342	-2.98770	0.39610
N	1.77124	-2.71702	-1.64239
N	-1.14890	-2.68666	1.43317
C	3.05234	-3.06580	-2.02308
C	3.78376	-4.09586	-1.40065
C	3.65892	-2.37578	-3.09537
C	5.06195	-4.42587	-1.85172
H	3.36652	-4.63050	-0.55470
C	4.93588	-2.71061	-3.52633
H	3.10908	-1.57639	-3.58619
C	5.65333	-3.74237	-2.91219
H	5.60476	-5.22236	-1.34934
H	5.37574	-2.15922	-4.35315
H	6.65173	-4.00039	-3.25034
H	1.40456	-1.84889	-2.00271
Cl	-1.01704	-4.01994	-2.35333
H	3.74464	1.11230	-1.30646
H	4.04634	1.11014	3.01489
H	-3.47659	0.90186	1.38577
H	-4.24828	0.80429	-2.88126

C	6.60800	-2.04963	0.66012
H	6.74432	-2.38347	-0.39285
C	6.09646	-3.25752	1.45435
H	6.76077	-4.13152	1.28439
H	6.07104	-3.04083	2.54323
H	5.07247	-3.52926	1.12332
C	7.98126	-1.60153	1.17105
H	8.71567	-2.42805	1.06259
H	8.34356	-0.73526	0.57704
H	7.93369	-1.30787	2.24132
C	-6.52750	-2.29003	-0.16520
H	-6.40639	-2.64485	0.88259
C	-6.29198	-3.49960	-1.08143
H	-6.89602	-4.36284	-0.72940
H	-5.22370	-3.79376	-1.06916
H	-6.58341	-3.27484	-2.12911
C	-7.96293	-1.77264	-0.30662
H	-8.68530	-2.57750	-0.05241
H	-8.13105	-0.92270	0.38905
H	-8.16116	-1.43036	-1.34497

Catalyst (4-i-Pr, 6)-Enamine protonation TS (pro-R) alternative conformation
Electronic energy (Hartrees): -1870.559583
Free Energy at 1M concentration and 253 K (Hartrees): -1869.683533
Coordinates:

P	0.01229	0.16079	0.16709
O	1.32302	0.96501	-0.38135
O	-1.14905	1.26317	0.49109
O	0.20927	-0.60914	1.43480
O	-0.41416	-0.65119	-1.09730
C	1.66297	2.07776	0.35260
C	3.00516	2.14753	0.99920
C	3.33660	3.23427	1.71776
H	4.30900	3.29980	2.19258
C	2.38798	4.35347	1.87491
C	2.74339	5.50326	2.73899
H	3.70221	5.53034	3.24167
C	1.88129	6.51232	2.90347
H	2.14111	7.35136	3.53592
C	0.56844	6.47998	2.23141
H	-0.13266	7.29038	2.38403
C	0.24057	5.44840	1.44482
H	-0.73537	5.43561	0.97800
C	1.20040	4.33086	1.23019
C	0.86059	3.17094	0.35351
C	-1.28387	2.26495	-0.43467
C	-2.53230	2.35067	-1.24407
C	-2.68209	3.36950	-2.10949
H	-3.59042	3.46507	-2.69286
C	-1.61156	4.36803	-2.29696
C	-1.77797	5.43824	-3.30816
H	-2.69714	5.51226	-3.87601
C	-0.79350	6.31604	-3.52683
H	-0.91552	7.09638	-4.26689
C	0.46847	6.21393	-2.77073
H	1.27144	6.91176	-2.97049
C	0.62674	5.25430	-1.85191
H	1.57186	5.18432	-1.33110
C	-0.47312	4.28954	-1.57169
C	-0.32958	3.21669	-0.54144

C	-3.59745	1.31981	-1.11200
C	-4.03190	0.84970	0.22845
C	-4.19312	0.80303	-2.20381
C	-4.98777	-0.07964	0.34612
C	-5.26796	-0.19998	-2.07247
C	-5.64871	-0.64067	-0.85854
H	-5.30028	-0.39453	1.33324
H	-5.74478	-0.56549	-2.97233
C	4.00720	1.07560	0.78380
C	5.23431	1.37248	0.31783
C	3.67583	-0.33713	1.08397
C	6.23016	0.30608	0.09208
C	4.57440	-1.30544	0.87175
C	5.92126	-0.98135	0.33961
H	7.20726	0.58163	-0.28275
H	4.31840	-2.32763	1.11681
H	-0.91965	-1.86229	1.65332
C	-2.76053	-2.24544	2.49669
C	-1.90874	-3.19370	0.42659
C	-2.53722	-1.38510	3.58845
C	-4.01370	-2.88343	2.32808
C	-3.18620	-3.82003	0.23432
C	-3.55491	-1.18195	4.50393
H	-1.58014	-0.88218	3.68107
C	-5.02622	-2.66305	3.28574
C	-4.17894	-3.68958	1.15992
H	-3.32226	-4.42487	-0.65085
C	-4.80164	-1.82397	4.36131
H	-3.38893	-0.51417	5.34367
H	-5.98680	-3.15324	3.15468
H	-5.12502	-4.20405	1.01341
H	-5.58445	-1.65227	5.09277
C	-0.82058	-3.22526	-0.50043
H	-0.66462	-1.70467	-0.88965
C	0.58142	-3.55602	0.00883
H	0.67171	-4.64675	0.12615
H	0.70313	-3.10845	1.00373
N	1.61901	-3.08770	-0.89261
N	-1.76531	-2.46095	1.56944
C	2.80030	-3.77794	-1.10707
C	3.31086	-4.69890	-0.17137
C	3.54146	-3.54471	-2.28397
C	4.50875	-5.36823	-0.42135
H	2.78840	-4.87139	0.76327
C	4.73152	-4.22128	-2.51943
H	3.16329	-2.83510	-3.01594
C	5.22940	-5.14240	-1.59278
H	4.88296	-6.06982	0.31957
H	5.27719	-4.02710	-3.43905
H	6.16243	-5.66447	-1.77915
H	1.30413	-2.50706	-1.65518
Cl	-1.16850	-4.12624	-2.03386
H	5.50555	2.39515	0.08491
H	2.71750	-0.59189	1.50859
H	-3.59853	1.27393	1.12488
H	-3.89298	1.10919	-3.19838
C	6.92127	-2.09675	0.10536
H	6.42555	-3.07212	0.30551
C	8.10515	-1.98623	1.07251
H	8.78688	-2.85330	0.93959

H	8.67999	-1.05233	0.89510
H	7.74006	-1.98904	2.12190
C	7.40228	-2.12495	-1.35150
H	7.97947	-3.05491	-1.54167
H	6.53443	-2.10406	-2.04305
H	8.05392	-1.25419	-1.57629
C	-6.77262	-1.64641	-0.69478
H	-6.81488	-1.96607	0.37034
C	-6.54354	-2.91009	-1.53656
H	-7.26632	-3.69886	-1.23760
H	-6.68080	-2.70374	-2.61902
H	-5.51770	-3.29983	-1.38048
C	-8.12376	-1.00634	-1.03083
H	-8.94565	-1.72989	-0.84319
H	-8.29237	-0.11512	-0.38904
H	-8.16095	-0.69296	-2.09618

Catalyst (4-i-Pr, 6)-Enamine protonation TS (pro-R) alternative conformation
Electronic energy (Hartrees): -1870.557963
Free Energy at 1M concentration and 253 K (Hartrees): -1869.681216
Coordinates:

P	-0.01859	0.17625	-0.01381
O	-1.20656	1.16080	0.50684
O	1.23676	1.12605	-0.45450
O	-0.37391	-0.66940	-1.19414
O	0.37316	-0.56503	1.30731
C	-1.41599	2.26291	-0.28559
C	-2.66101	2.35789	-1.09747
C	-2.86008	3.43476	-1.87481
H	-3.76291	3.52126	-2.46852
C	-1.85489	4.51363	-1.94368
C	-2.07865	5.66524	-2.84874
H	-2.99230	5.73409	-3.42610
C	-1.15293	6.62355	-2.95951
H	-1.31677	7.46350	-3.62221
C	0.10142	6.53146	-2.18950
H	0.85729	7.29810	-2.30182
C	0.31123	5.49817	-1.36578
H	1.25015	5.44137	-0.83235
C	-0.72364	4.43871	-1.20595
C	-0.51820	3.27599	-0.28969
C	1.53775	2.13815	0.42407
C	2.84131	2.11522	1.14501
C	3.12094	3.09745	2.02148
H	4.07352	3.11826	2.53779
C	2.14986	4.17841	2.28434
C	2.45707	5.20913	3.30353
H	3.39934	5.18336	3.83672
C	1.57167	6.17480	3.57163
H	1.79650	6.92642	4.31734
C	0.28033	6.21213	2.85956
H	-0.44020	6.98476	3.09518
C	-0.00446	5.28895	1.93383
H	-0.96568	5.32439	1.43875
C	0.98242	4.22405	1.60417
C	0.69221	3.18535	0.57251
C	3.84590	1.05211	0.86868
C	4.14476	0.64585	-0.52802
C	4.53195	0.47274	1.87072
C	5.09815	-0.25758	-0.78771

C	5.59973	-0.50556	1.58458
C	5.90199	-0.85114	0.31870
H	5.27923	-0.53033	-1.81791
H	6.16456	-0.90973	2.41588
C	-3.66535	1.26781	-1.08299
C	-4.13662	0.77531	0.07573
C	-4.18941	0.72832	-2.36235
C	-5.16995	-0.28048	0.07437
C	-5.12094	-0.23151	-2.36356
C	-5.64704	-0.77270	-1.08349
H	-5.54841	-0.63490	1.02352
H	-5.48988	-0.60803	-3.30866
H	0.47537	-2.17375	-1.20401
C	2.21135	-2.98091	-1.95269
C	1.18894	-3.52592	0.18652
C	2.17309	-2.19246	-3.11821
C	3.30817	-3.83984	-1.70006
C	2.30929	-4.38429	0.45731
C	3.21934	-2.27445	-4.02093
H	1.33758	-1.51807	-3.27561
C	4.34895	-3.91226	-2.64948
C	3.29831	-4.55846	-0.46284
H	2.32176	-4.91461	1.39889
C	4.30889	-3.13845	-3.79483
H	3.19870	-1.65952	-4.91539
H	5.18873	-4.57444	-2.45841
H	4.10712	-5.25414	-0.25564
H	5.11747	-3.18980	-4.51649
C	0.14590	-3.22055	1.10966
H	0.37156	-1.66027	1.26462
C	-1.31554	-3.25285	0.66587
H	-1.67682	-4.28867	0.72297
H	-1.36980	-2.94685	-0.38800
N	-2.16086	-2.42021	1.49768
N	1.18704	-2.90800	-1.03274
C	-3.43458	-2.76043	1.91414
C	-4.19479	-3.76338	1.28223
C	-4.01725	-2.06774	2.99686
C	-5.48150	-4.06154	1.72967
H	-3.79206	-4.29586	0.42788
C	-5.30433	-2.36930	3.42402
H	-3.44467	-1.28960	3.49581
C	-6.05200	-3.37172	2.79792
H	-6.04724	-4.83679	1.21949
H	-5.72673	-1.81720	4.25940
H	-7.05717	-3.60450	3.13441
H	-1.69674	-1.69155	2.01817
Cl	0.33048	-3.97452	2.74176
H	-3.79276	1.16550	1.02567
H	-3.81358	1.10558	-3.30537
H	3.60904	1.09493	-1.35432
H	4.33172	0.73138	2.90321
C	-6.71819	-1.84619	-1.08071
H	-6.94425	-2.15125	-0.03434
C	-6.24438	-3.10067	-1.82429
H	-6.98107	-3.92128	-1.69031
H	-6.12983	-2.90424	-2.91127
H	-5.26824	-3.44085	-1.42071
C	-8.02139	-1.31281	-1.68487
H	-8.81777	-2.08406	-1.61281

H	-8.35846	-0.41291	-1.12710
H	-7.88556	-1.04238	-2.75371
C	7.07994	-1.78595	0.06948
H	7.92860	-1.43948	0.70151
C	7.58860	-1.77207	-1.37985
H	8.52984	-2.35750	-1.45550
H	6.84497	-2.22497	-2.06951
H	7.80772	-0.73151	-1.70147
C	6.73557	-3.21752	0.49261
H	7.62071	-3.87776	0.37091
H	6.42746	-3.24992	1.55902
H	5.91027	-3.61125	-0.13097

Catalyst (4-i-Pr, 6)-Enamine protonation TS (pro-R) alternative conformation
Electronic energy (Hartrees): -1870.554606
Free Energy at 1M concentration and 253 K (Hartrees): -1869.677934
Coordinates:

P	-0.07722	0.15918	0.21264
O	1.24950	0.99151	-0.25193
O	-1.28324	1.22798	0.46507
O	0.05800	-0.61800	1.48278
O	-0.39258	-0.65399	-1.08621
C	1.50727	2.13026	0.47376
C	2.80230	2.25656	1.20366
C	3.06032	3.37643	1.90122
H	3.99628	3.48285	2.43779
C	2.07852	4.47650	1.95374
C	2.35212	5.66669	2.79258
H	3.27623	5.73685	3.35282
C	1.45875	6.65929	2.86299
H	1.65883	7.52790	3.47698
C	0.19234	6.56857	2.11199
H	-0.53527	7.36625	2.18857
C	-0.06154	5.50003	1.34772
H	-1.00568	5.44534	0.82231
C	0.93495	4.39931	1.23764
C	0.67807	3.19897	0.38725
C	-1.40432	2.21701	-0.47615
C	-2.63494	2.27833	-1.31338
C	-2.75419	3.25477	-2.23084
H	-3.65091	3.33480	-2.83414
C	-1.67670	4.24436	-2.42769
C	-1.80714	5.26794	-3.49101
H	-2.70095	5.30798	-4.10115
C	-0.82129	6.14571	-3.70419
H	-0.91695	6.89203	-4.48224
C	0.40599	6.09089	-2.88816
H	1.21022	6.78915	-3.08123
C	0.53130	5.17385	-1.92196
H	1.45122	5.13793	-1.35436
C	-0.57063	4.20934	-1.65071
C	-0.46030	3.18004	-0.57364
C	-3.73772	1.29849	-1.11819
C	-4.20568	0.95538	0.24859
C	-4.35279	0.73355	-2.17299
C	-5.23440	0.11747	0.42801
C	-5.50105	-0.17309	-1.97753
C	-5.95132	-0.46421	-0.74245
H	-5.53871	-0.11245	1.43944
H	-5.99698	-0.57362	-2.85338

C	3.83838	1.19930	1.10114
C	5.09413	1.50692	0.72729
C	3.50920	-0.21094	1.41504
C	6.12356	0.45437	0.61512
C	4.43686	-1.16803	1.29989
C	5.81710	-0.83208	0.87103
H	7.12378	0.73907	0.31579
H	4.17685	-2.18946	1.54687
H	-0.88039	-2.05271	1.50275
C	-2.73859	-2.71885	2.07809
C	-1.48318	-3.48185	0.13863
C	-2.80641	-1.84012	3.17582
C	-3.84005	-3.54549	1.74960
C	-2.60562	-4.30995	-0.21009
C	-3.96187	-1.80142	3.93724
H	-1.96241	-1.19277	3.39078
C	-4.99577	-3.49332	2.55637
C	-3.71247	-4.36254	0.58170
H	-2.52759	-4.91415	-1.10267
C	-5.05958	-2.63135	3.63599
H	-4.02154	-1.11686	4.77775
H	-5.83963	-4.12989	2.30544
H	-4.52807	-5.03204	0.32111
H	-5.95518	-2.58719	4.24690
C	-0.31756	-3.29884	-0.66177
H	-0.44737	-1.74210	-0.96847
C	1.07725	-3.34035	-0.02927
H	1.37517	-4.38724	0.10752
H	1.02238	-2.88114	0.96478
N	2.08505	-2.68065	-0.84561
N	-1.60310	-2.76757	1.29813
C	3.23259	-3.31826	-1.30428
C	3.88155	-4.32212	-0.56037
C	3.79893	-2.93315	-2.53577
C	5.03208	-4.93551	-1.05427
H	3.50253	-4.60511	0.41602
C	4.95012	-3.54863	-3.01374
H	3.31173	-2.15851	-3.12298
C	5.57583	-4.56265	-2.28290
H	5.51366	-5.70678	-0.45902
H	5.35904	-3.23637	-3.97096
H	6.47430	-5.04155	-2.65883
H	1.71880	-1.97512	-1.46913
Cl	-0.32743	-4.19372	-2.23163
H	5.36595	2.52759	0.48658
H	2.52579	-0.47221	1.77129
H	-3.73184	1.39775	1.11537
H	-4.02937	0.94730	-3.18433
C	6.85239	-1.93358	0.75270
H	6.35575	-2.91431	0.92415
C	7.93848	-1.78333	1.82355
H	8.64608	-2.63812	1.77016
H	8.50919	-0.84052	1.68362
H	7.47715	-1.77672	2.83431
C	7.46835	-1.97940	-0.65163
H	8.06672	-2.90746	-0.77303
H	6.66854	-1.97467	-1.42009
H	8.13235	-1.10693	-0.82833
C	-7.19184	-1.33674	-0.59536
H	-7.94527	-0.98665	-1.33668

C	-7.86369	-1.23321	0.78196
H	-8.83694	-1.76888	0.76845
H	-7.23081	-1.68988	1.57195
H	-8.06274	-0.16985	1.03464
C	-6.86463	-2.79870	-0.91517
H	-7.78447	-3.41940	-0.86239
H	-6.44546	-2.89202	-1.93936
H	-6.12707	-3.19246	-0.18949

Catalyst (4-i-Pr, 6)-Product (S) lowest free energy conformation
Electronic energy (Hartrees): -1870.602174
Free Energy at 1M concentration and 253 K (Hartrees): -1869.723004
Coordinates:

P	-0.71565	0.25301	-0.29150
O	-2.17811	0.41659	-0.98775
O	-0.78577	-1.12013	0.60401
O	-0.65758	1.35501	0.83572
O	0.34518	0.25483	-1.32483
C	-3.25703	0.00007	-0.24189
C	-4.24050	1.01470	0.22253
C	-5.31077	0.62378	0.93304
H	-6.03551	1.35376	1.27501
C	-5.50932	-0.79855	1.27546
C	-6.66950	-1.19465	2.10756
H	-7.39001	-0.45137	2.42565
C	-6.83142	-2.47064	2.47237
H	-7.67858	-2.75843	3.08149
C	-5.84760	-3.48793	2.05794
H	-5.97134	-4.51491	2.37676
C	-4.79904	-3.14186	1.30235
H	-4.07913	-3.90250	1.03310
C	-4.62132	-1.73088	0.85902
C	-3.46282	-1.31823	0.00893
C	-1.18607	-2.20136	-0.13871
C	-0.18196	-3.21041	-0.56737
C	-0.56579	-4.22689	-1.35523
H	0.15530	-4.96771	-1.68078
C	-1.97142	-4.34547	-1.79931
C	-2.36242	-5.45170	-2.70379
H	-1.63126	-6.19084	-3.00688
C	-3.61877	-5.54130	-3.15297
H	-3.90262	-6.34905	-3.81514
C	-4.61915	-4.53170	-2.75811
H	-5.62878	-4.59809	-3.14261
C	-4.27824	-3.53085	-1.93812
H	-5.02315	-2.79027	-1.68030
C	-2.89190	-3.43348	-1.40525
C	-2.48431	-2.32590	-0.49021
H	0.27624	1.44422	1.26443
C	1.99191	0.60951	2.95779
C	2.75368	2.17060	1.39871
C	0.91345	-0.13346	3.50128
C	3.30130	0.44939	3.50386
C	4.08495	2.05002	1.87147
C	1.13509	-0.99185	4.55520
H	-0.07203	-0.02170	3.06436
C	3.49264	-0.43873	4.59291
C	4.35001	1.19625	2.91227
H	4.86813	2.63114	1.40500
C	2.42911	-1.14367	5.10960

H	0.30798	-1.56270	4.96585
H	4.49097	-0.55590	5.00498
H	5.36278	1.08268	3.28916
H	2.58008	-1.82519	5.94093
C	2.38277	3.06554	0.23105
C	2.65815	2.44468	-1.14117
H	2.27128	3.12947	-1.91184
H	2.04303	1.54320	-1.18350
N	4.05464	2.10665	-1.32792
N	1.75467	1.47410	1.92057
C	4.45688	1.14243	-2.26564
C	5.79043	1.16202	-2.71652
C	3.60318	0.12851	-2.73688
C	6.25973	0.19657	-3.60092
H	6.46079	1.94018	-2.35752
C	4.08838	-0.82848	-3.62829
H	2.56906	0.07168	-2.41566
C	5.41196	-0.81078	-4.06669
H	7.29487	0.23531	-3.92924
H	3.41099	-1.60174	-3.98055
H	5.77575	-1.56335	-4.75913
H	4.65252	2.92429	-1.33419
H	1.32081	3.30300	0.29435
Cl	3.22999	4.69410	0.37803
C	1.23736	-3.06337	-0.16977
C	1.57037	-2.83605	1.11251
C	2.30813	-3.11765	-1.19480
C	2.97816	-2.60792	1.49115
H	0.81226	-2.80590	1.88545
C	3.58452	-2.90759	-0.85161
H	2.05961	-3.30899	-2.23124
C	3.94811	-2.61612	0.55988
H	3.20963	-2.42088	2.53039
H	4.35062	-2.94635	-1.61456
C	-4.01757	2.45105	-0.06719
C	-3.71546	2.89233	-1.45031
C	-4.04485	3.36447	0.91973
C	-3.45000	4.17767	-1.70923
H	-3.71529	2.17527	-2.26173
C	-3.75655	4.78469	0.63429
H	-4.24443	3.06856	1.94252
C	-3.46124	5.18551	-0.61615
H	-3.22975	4.47663	-2.72573
H	-3.77360	5.49103	1.45495
C	5.38459	-2.32207	0.94517
H	5.44614	-2.09838	2.03448
C	5.90523	-1.08596	0.20373
H	6.91094	-0.80857	0.58487
H	5.98240	-1.27841	-0.88689
H	5.22211	-0.22633	0.36824
C	6.28006	-3.53705	0.68135
H	6.32265	-3.77559	-0.40266
H	7.31187	-3.33336	1.03923
H	5.89104	-4.42197	1.22906
C	-3.13893	6.63949	-0.90239
H	-3.19871	7.23256	0.03811
C	-4.15410	7.24347	-1.87922
H	-5.18389	7.12491	-1.47925
H	-4.09602	6.75212	-2.87355
H	-3.95597	8.32875	-2.01021

C	-1.70898	6.78580	-1.43533
H	-0.98752	6.34046	-0.71699
H	-1.45717	7.86083	-1.55817
H	-1.59376	6.28185	-2.41840

Catalyst (4-i-Pr, 6)-Product (S) alternative conformation
Electronic energy (Hartrees): -1870.6019
Free Energy at 1M concentration and 253 K (Hartrees): -1869.72236
Coordinates:

P	0.69916	-0.23664	-0.29764
O	1.10094	1.05148	0.63545
O	2.06441	-0.65073	-1.08195
O	-0.39265	0.02161	-1.26417
O	0.47848	-1.35765	0.78754
C	1.71643	2.04381	-0.08534
C	0.94321	3.25068	-0.47506
C	1.53222	4.19793	-1.22243
H	0.97912	5.08190	-1.51812
C	2.93064	4.03612	-1.67613
C	3.54409	5.07238	-2.53905
H	2.98747	5.96481	-2.79696
C	4.78637	4.90971	-3.00639
H	5.23273	5.66694	-3.63794
C	5.54749	3.69049	-2.67480
H	6.54368	3.55449	-3.07567
C	5.00517	2.75009	-1.89267
H	5.57389	1.85447	-1.68276
C	3.63609	2.93053	-1.33694
C	3.00799	1.89819	-0.45895
C	3.22136	-0.54873	-0.34524
C	3.91208	-1.79310	0.08390
C	5.05607	-1.70803	0.78126
H	5.56470	-2.60832	1.10645
C	5.61420	-0.39191	1.15415
C	6.84883	-0.32761	1.97071
H	7.36745	-1.23774	2.24537
C	7.32675	0.85338	2.37606
H	8.22772	0.89868	2.97429
C	6.61854	2.09770	2.02282
H	6.99704	3.04865	2.37521
C	5.50535	2.05172	1.28191
H	4.99126	2.97641	1.05856
C	4.97798	0.74744	0.79290
C	3.74055	0.66737	-0.04207
H	-0.46722	-1.34552	1.21127
C	-2.06295	-0.37534	2.90873
C	-2.98263	-1.78021	1.28518
C	-0.90890	0.19687	3.50159
C	-3.35334	-0.05845	3.42978
C	-4.29790	-1.50375	1.73694
C	-1.04154	1.03968	4.58275
H	0.06428	-0.03253	3.08279
C	-3.45318	0.81501	4.54256
C	-4.47485	-0.64887	2.79586
H	-5.13787	-1.96798	1.23944
C	-2.31860	1.34867	5.11103
H	-0.15674	1.47844	5.03345
H	-4.43743	1.05381	4.93545
H	-5.47319	-0.41604	3.15600
H	-2.39919	2.01681	5.96275

C	-2.70032	-2.67953	0.09641
C	-2.88619	-1.99220	-1.26066
H	-2.58686	-2.70278	-2.04658
H	-2.16127	-1.17552	-1.27919
N	-4.22717	-1.47511	-1.44313
N	-1.91656	-1.23200	1.84899
C	-4.49913	-0.38738	-2.28811
C	-5.81574	-0.21882	-2.75720
C	-3.53121	0.56914	-2.64472
C	-6.15749	0.87108	-3.55112
H	-6.57381	-0.95085	-2.48606
C	-3.88945	1.65215	-3.44746
H	-2.50632	0.48659	-2.30098
C	-5.19584	1.81929	-3.90609
H	-7.18253	0.97567	-3.89644
H	-3.12473	2.37708	-3.71318
H	-5.46001	2.66848	-4.52841
H	-4.92007	-2.20942	-1.52035
H	-1.67372	-3.04011	0.16329
Cl	-3.73141	-4.20218	0.18679
C	3.28486	-3.10960	-0.18099
C	3.05572	-3.97382	0.82393
C	2.80160	-3.44298	-1.54276
C	2.29601	-5.21748	0.58361
H	3.37026	-3.74357	1.83463
C	2.10382	-4.56336	-1.75926
H	3.00732	-2.77568	-2.37042
C	1.81633	-5.50054	-0.64159
H	2.10425	-5.87632	1.42133
H	1.74928	-4.77941	-2.75860
C	-0.48250	3.36299	-0.08817
C	-0.88665	3.12547	1.31837
C	-1.43248	3.59384	-1.01209
C	-2.18011	3.09694	1.65570
H	-0.13363	2.97618	2.08222
C	-2.86147	3.56787	-0.63717
H	-1.16900	3.74346	-2.05207
C	-3.23146	3.30133	0.63054
H	-2.45508	2.92935	2.68754
H	-3.60253	3.73312	-1.40777
C	0.97714	-6.74237	-0.87163
H	0.87882	-7.31408	0.07880
C	-0.44051	-6.36353	-1.31654
H	-0.42828	-5.85094	-2.30185
H	-1.06969	-7.27525	-1.40013
H	-0.90662	-5.68805	-0.56736
C	1.64293	-7.67501	-1.88961
H	2.67086	-7.93166	-1.55502
H	1.06154	-8.61759	-1.97776
H	1.69903	-7.20103	-2.89238
C	-4.69022	3.18888	1.02795
H	-4.75148	2.94114	2.11118
C	-5.38540	2.05182	0.26937
H	-4.80926	1.10925	0.38228
H	-6.40457	1.88810	0.67932
H	-5.47460	2.28958	-0.81126
C	-5.42628	4.51830	0.82638
H	-5.46633	4.79512	-0.24858
H	-6.46598	4.43825	1.20940
H	-4.91068	5.32709	1.38710

Catalyst (4-i-Pr, 6)-Product (S) alternative conformation
Electronic energy (Hartrees): -1870.596432
Free Energy at 1M concentration and 253 K (Hartrees): -1869.71744
Coordinates:

P	-0.71283	0.24974	-0.39358
O	-2.22532	0.20703	-0.98973
O	-0.56790	-1.06211	0.58334
O	-0.69013	1.40479	0.68007
O	0.26764	0.32279	-1.50041
C	-3.19860	-0.27584	-0.14662
C	-4.25228	0.65623	0.33553
C	-5.22919	0.19648	1.13337
H	-6.00233	0.86665	1.49131
C	-5.25267	-1.21762	1.55692
C	-6.31053	-1.68450	2.48336
H	-7.08789	-1.00431	2.80876
C	-6.30922	-2.94628	2.92508
H	-7.08155	-3.28555	3.60326
C	-5.24566	-3.87576	2.50188
H	-5.23562	-4.88867	2.88341
C	-4.28852	-3.46601	1.66157
H	-3.50567	-4.16021	1.38907
C	-4.29417	-2.07358	1.13190
C	-3.24073	-1.59122	0.18611
C	-0.88959	-2.23197	-0.05339
C	0.19169	-3.17810	-0.43552
C	-0.12266	-4.29194	-1.11641
H	0.65124	-4.99439	-1.40304
C	-1.52597	-4.57810	-1.48634
C	-1.84215	-5.78864	-2.27965
H	-1.05430	-6.47721	-2.55877
C	-3.10107	-6.03571	-2.65718
H	-3.33040	-6.91979	-3.23796
C	-4.17998	-5.09747	-2.29398
H	-5.19330	-5.29293	-2.62041
C	-3.90657	-4.00333	-1.57399
H	-4.70996	-3.31880	-1.33711
C	-2.51575	-3.72914	-1.12094
C	-2.18383	-2.51615	-0.31659
H	0.27091	1.57100	0.98289
C	2.16766	1.27566	2.72808
C	2.70159	2.48272	0.80507
C	1.27450	0.39689	3.39264
C	3.38704	1.65606	3.36653
C	3.91596	2.93324	1.38560
C	1.59556	-0.09002	4.63990
H	0.36192	0.10240	2.88638
C	3.68181	1.14268	4.65495
C	4.24703	2.52997	2.65485
H	4.56923	3.59176	0.82582
C	2.80387	0.28410	5.27724
H	0.91771	-0.77403	5.14128
H	4.61236	1.43153	5.13569
H	5.17385	2.85851	3.11758
H	3.03650	-0.11308	6.26043
C	2.29974	2.83497	-0.60941
C	3.45697	2.95805	-1.62015
H	4.00924	3.88094	-1.41696
H	3.01718	3.06630	-2.61846

N	4.38317	1.83842	-1.56723
N	1.85206	1.71759	1.47310
C	4.34119	0.76288	-2.46678
C	5.54145	0.11422	-2.80931
C	3.13618	0.29113	-3.01149
C	5.53954	-0.96327	-3.68975
H	6.47954	0.46645	-2.38450
C	3.15114	-0.78572	-3.89760
H	2.18372	0.71304	-2.71464
C	4.34368	-1.41813	-4.24982
H	6.48001	-1.44571	-3.94155
H	2.20633	-1.14038	-4.29943
H	4.34168	-2.25862	-4.93673
H	5.32626	2.11079	-1.33301
H	1.56999	2.10111	-0.95219
Cl	1.39430	4.43950	-0.60314
C	1.60403	-2.87755	-0.09363
C	1.94172	-2.46723	1.14269
C	2.66340	-3.00009	-1.12479
C	3.33869	-2.11178	1.46090
H	1.19778	-2.39178	1.92588
C	3.93088	-2.68480	-0.83440
H	2.41443	-3.33417	-2.12430
C	4.29798	-2.20191	0.51978
H	3.57068	-1.77111	2.46178
H	4.69027	-2.78722	-1.59800
C	-4.18999	2.09302	-0.02311
C	-4.01234	2.49965	-1.43808
C	-4.23707	3.04175	0.92916
C	-3.86988	3.79021	-1.75996
H	-4.00097	1.75173	-2.22107
C	-4.08697	4.46761	0.57421
H	-4.34458	2.77333	1.97315
C	-3.89811	4.83829	-0.70578
H	-3.73587	4.06335	-2.79851
H	-4.11225	5.20371	1.36808
C	5.73803	-1.83004	0.81466
H	6.33680	-1.93741	-0.11711
C	6.34922	-2.77377	1.85665
H	7.42630	-2.53995	1.99587
H	5.83580	-2.67370	2.83668
H	6.26403	-3.82668	1.51230
C	5.85916	-0.36787	1.25976
H	5.39545	-0.21509	2.25652
H	6.92919	-0.07674	1.32215
H	5.35994	0.29740	0.52569
C	-3.70693	6.29947	-1.06375
H	-3.76224	6.92476	-0.14408
C	-4.81724	6.78353	-2.00311
H	-5.81167	6.60232	-1.54185
H	-4.71272	7.87465	-2.18412
H	-4.77350	6.25849	-2.98080
C	-2.32338	6.53160	-1.68201
H	-2.22104	5.99516	-2.64919
H	-2.16402	7.61633	-1.86125
H	-1.53191	6.17483	-0.98843

Catalyst (4-i-Pr, 6)-Product (S) alternative conformation
Electronic energy (Hartrees): -1870.595102
Free Energy at 1M concentration and 253 K (Hartrees): -1869.715248

Coordinates:

P	0.71271	-0.23218	-1.95694
O	-0.55950	0.20294	-0.96598
O	1.96882	0.76719	-1.61352
O	0.45641	-0.17224	-3.40349
O	1.03684	-1.65105	-1.34787
C	-0.77505	1.55635	-0.87453
C	-2.08923	2.13076	-1.29954
C	-2.29095	3.45595	-1.18636
H	-3.23234	3.89549	-1.49520
C	-1.24511	4.33836	-0.63717
C	-1.47349	5.80117	-0.58219
H	-2.40937	6.21938	-0.93146
C	-0.52526	6.61205	-0.10132
H	-0.69262	7.68066	-0.06221
C	0.75676	6.05321	0.36726
H	1.52846	6.71653	0.73606
C	0.97022	4.73300	0.32611
H	1.92653	4.34847	0.65448
C	-0.08714	3.81442	-0.17966
C	0.12261	2.33708	-0.22851
C	2.19683	1.01398	-0.28269
C	3.52427	0.67157	0.30796
C	3.73895	0.89358	1.61477
H	4.69416	0.64404	2.06243
C	2.68633	1.48307	2.46267
C	2.91345	1.62632	3.91935
H	3.84354	1.29018	4.36064
C	1.96899	2.16553	4.69698
H	2.13438	2.26718	5.76175
C	0.69873	2.62545	4.10540
H	-0.06123	3.06355	4.73954
C	0.48607	2.50401	2.79023
H	-0.45110	2.85363	2.38159
C	1.52618	1.90334	1.91021
C	1.30001	1.71986	0.44407
H	0.62430	-1.99659	-0.48217
C	1.05721	-2.92433	1.89492
C	-1.04769	-3.36138	1.00472
C	2.35363	-2.38144	1.72353
C	0.72406	-3.56788	3.12459
C	-1.44284	-4.05599	2.17793
C	3.27098	-2.44452	2.74878
H	2.60089	-1.94333	0.76515
C	1.68895	-3.61258	4.16268
C	-0.56829	-4.14378	3.23008
H	-2.42928	-4.50211	2.22665
C	2.93493	-3.05440	3.98105
H	4.26444	-2.02929	2.60995
H	1.42948	-4.09679	5.09996
H	-0.85193	-4.65392	4.14657
H	3.66912	-3.09047	4.77968
C	-2.01426	-3.15236	-0.14118
C	-3.23145	-2.31258	0.26849
H	-3.83010	-2.88014	0.98891
H	-3.86002	-2.14784	-0.61800
N	-2.75703	-1.09737	0.89274
N	0.15246	-2.82216	0.86997
C	-3.62847	-0.18655	1.48902
C	-3.09946	1.03578	1.94657

C	-5.00031	-0.43147	1.66731
C	-3.91374	1.98724	2.54545
H	-2.03594	1.21422	1.82379
C	-5.80739	0.52851	2.28125
H	-5.44720	-1.35129	1.30603
C	-5.27949	1.74141	2.72032
H	-3.47905	2.92496	2.88069
H	-6.86765	0.32396	2.40142
H	-5.91779	2.48377	3.18829
H	-1.98609	-0.66260	0.39759
H	-1.48403	-2.67952	-0.96809
Cl	-2.59981	-4.75094	-0.79940
C	-3.20474	1.25528	-1.75227
C	-4.44354	1.40732	-1.24489
C	-2.97623	0.19333	-2.76081
C	-5.54008	0.50504	-1.64658
H	-4.64954	2.16492	-0.49898
C	-3.96901	-0.62645	-3.12648
H	-2.01374	0.09185	-3.23332
C	-5.32289	-0.48100	-2.53698
H	-6.51431	0.64463	-1.19661
H	-3.77799	-1.39233	-3.86761
C	4.64045	0.17978	-0.53804
C	5.96518	0.84619	-0.46550
C	4.48474	-0.86726	-1.36592
C	6.98527	0.41553	-1.21628
H	6.11025	1.69589	0.19019
C	5.60954	-1.34381	-2.19605
H	3.54595	-1.39431	-1.42550
C	6.81033	-0.73987	-2.13471
H	7.94008	0.92062	-1.14945
H	5.44589	-2.18991	-2.85187
C	-6.42774	-1.43392	-2.95135
H	-6.02059	-2.16055	-3.68899
C	-6.94171	-2.24792	-1.75671
H	-7.40826	-1.59060	-0.99266
H	-7.69952	-2.98528	-2.09740
H	-6.10565	-2.80745	-1.28717
C	-7.57445	-0.68675	-3.64317
H	-8.32204	-1.41197	-4.02962
H	-8.08495	0.00427	-2.93922
H	-7.18341	-0.10094	-4.50247
C	7.96373	-1.23075	-2.98855
H	7.63636	-2.09938	-3.60350
C	8.42575	-0.14039	-3.96186
H	7.57170	0.19877	-4.58644
H	8.84187	0.73361	-3.41728
H	9.21122	-0.54175	-4.63727
C	9.12552	-1.71316	-2.11270
H	8.77260	-2.50014	-1.41224
H	9.92563	-2.14859	-2.74868
H	9.55882	-0.87665	-1.52439

Catalyst (4-i-Pr, 6)-Product (S) alternative conformation
Electronic energy (Hartrees): -1870.589722
Free Energy at 1M concentration and 253 K (Hartrees): -1869.709696
Coordinates:

P	-0.16898	0.19357	-0.37246
O	1.06744	1.14040	-0.91292
O	-1.23280	1.17212	0.37997

O	0.32963	-0.58069	0.92278
O	-0.67122	-0.62211	-1.49821
C	1.57024	2.01506	0.02045
C	2.98940	1.88745	0.47935
C	3.40770	2.66885	1.49493
H	4.41573	2.57739	1.88128
C	2.51878	3.68395	2.09458
C	2.99753	4.49730	3.23687
H	3.99526	4.34830	3.63071
C	2.19987	5.41873	3.78752
H	2.55016	6.01150	4.62262
C	0.83653	5.62489	3.26304
H	0.19010	6.36110	3.72315
C	0.39679	4.89868	2.22885
H	-0.61245	5.05415	1.87131
C	1.28127	3.88513	1.59168
C	0.80944	3.05017	0.45102
C	-1.44609	2.37488	-0.24308
C	-2.75999	2.63938	-0.88668
C	-2.97493	3.81602	-1.49427
H	-3.92667	4.01725	-1.97271
C	-1.92144	4.85072	-1.52367
C	-2.17246	6.13031	-2.22693
H	-3.13442	6.31850	-2.68740
C	-1.21252	7.05821	-2.29824
H	-1.39676	7.99222	-2.81319
C	0.10545	6.80649	-1.68625
H	0.88525	7.55290	-1.76632
C	0.34168	5.65588	-1.04551
H	1.32395	5.48699	-0.62623
C	-0.72571	4.62351	-0.93129
C	-0.49050	3.33059	-0.21872
H	0.64156	-1.53479	0.76333
C	2.60976	-3.16266	1.02230
C	0.80361	-3.96076	-0.22709
C	3.12418	-2.19980	1.93019
C	3.45883	-4.21337	0.56051
C	1.57374	-5.04866	-0.70749
C	4.43914	-2.26789	2.33120
H	2.46829	-1.40629	2.27204
C	4.81015	-4.25240	0.99286
C	2.89019	-5.15866	-0.32639
H	1.12197	-5.76720	-1.37778
C	5.29122	-3.29585	1.85679
H	4.83213	-1.52257	3.01592
H	5.45575	-5.04609	0.62742
H	3.50383	-5.97389	-0.70069
H	6.32697	-3.32524	2.18017
C	-0.66646	-3.73151	-0.55993
C	-1.52728	-3.82448	0.70778
H	-1.10160	-3.11902	1.42206
H	-1.41574	-4.83133	1.14466
N	-2.91364	-3.46712	0.46481
N	1.31509	-3.05168	0.59437
C	-3.70707	-2.99471	1.52497
C	-5.00707	-3.49539	1.70809
C	-3.24973	-1.98294	2.38924
C	-5.82260	-3.00664	2.72646
H	-5.37538	-4.27564	1.04574
C	-4.06610	-1.51648	3.41666

H	-2.27496	-1.53173	2.23291
C	-5.35526	-2.02271	3.59841
H	-6.82530	-3.40857	2.84311
H	-3.69395	-0.73195	4.06975
H	-5.98738	-1.64812	4.39735
H	-3.38910	-4.17016	-0.08779
H	-0.79503	-2.74840	-1.02433
Cl	-1.29099	-4.91901	-1.80477
C	-3.81130	1.59946	-0.89841
C	-4.23514	1.03734	0.24579
C	-4.40296	1.15982	-2.18480
C	-5.27927	-0.00613	0.22217
H	-3.82705	1.34752	1.20015
C	-5.33480	0.19989	-2.20797
H	-4.06942	1.60319	-3.11489
C	-5.80263	-0.42702	-0.94349
H	-5.61318	-0.42411	1.16142
H	-5.74617	-0.11428	-3.15833
C	3.96242	0.98183	-0.21107
C	3.56155	-0.36862	-0.68317
C	5.23531	1.37644	-0.42642
C	4.44235	-1.18121	-1.27660
H	2.56404	-0.73479	-0.52518
C	6.21284	0.47472	-1.06977
H	5.57076	2.36439	-0.13752
C	5.84626	-0.75551	-1.47386
H	4.12281	-2.16520	-1.59659
H	7.22997	0.82146	-1.19793
C	-6.85157	-1.52177	-0.96398
H	-7.05667	-1.86905	0.07371
C	-6.35575	-2.73764	-1.75493
H	-5.38110	-3.08421	-1.35122
H	-7.08446	-3.57137	-1.66606
H	-6.22914	-2.49192	-2.83058
C	-8.17543	-0.99956	-1.53224
H	-8.06499	-0.70034	-2.59614
H	-8.95477	-1.78854	-1.46620
H	-8.52055	-0.12118	-0.94586
C	6.83484	-1.71583	-2.10623
H	6.30646	-2.66041	-2.36477
C	7.95395	-2.08423	-1.12488
H	7.51883	-2.49614	-0.18932
H	8.61146	-2.86001	-1.57201
H	8.57396	-1.19777	-0.87313
C	7.40677	-1.15014	-3.41177
H	6.58060	-0.88576	-4.10602
H	8.02089	-0.24446	-3.22179
H	8.04612	-1.91157	-3.90738

Catalyst (4-i-Pr, 6)-Product (S) alternative conformation
Electronic energy (Hartrees): -1870.564519
Free Energy at 1M concentration and 253 K (Hartrees): -1869.676621
Coordinates:

P	-0.13387	0.31220	0.29402
O	0.82756	1.23488	-0.69911
O	-1.13234	1.38149	1.07072
O	0.67266	-0.34542	1.38216
O	-0.92798	-0.55381	-0.66871
C	1.43160	2.27871	-0.04465
C	2.90171	2.24987	0.18874

C	3.47205	3.23827	0.89792
H	4.54268	3.24464	1.06608
C	2.65948	4.35360	1.42732
C	3.30397	5.40785	2.24460
H	4.36846	5.37006	2.43999
C	2.57436	6.41020	2.74602
H	3.04614	7.17929	3.34383
C	1.12179	6.46574	2.49424
H	0.53409	7.26972	2.91821
C	0.53056	5.52127	1.75345
H	-0.53874	5.57171	1.59729
C	1.33314	4.41380	1.16668
C	0.69831	3.34652	0.33939
C	-1.58067	2.42808	0.30292
C	-3.02651	2.51074	-0.04361
C	-3.47213	3.55182	-0.76768
H	-4.52378	3.63537	-1.01659
C	-2.54376	4.59851	-1.23769
C	-3.05473	5.70523	-2.07999
H	-4.10907	5.76419	-2.32022
C	-2.21676	6.63505	-2.54985
H	-2.59090	7.44220	-3.16632
C	-0.77706	6.55607	-2.24031
H	-0.10050	7.29935	-2.64207
C	-0.30622	5.56337	-1.47672
H	0.75681	5.51511	-1.28432
C	-1.23061	4.53646	-0.92022
C	-0.73337	3.42052	-0.05994
H	-2.11940	-3.16275	0.56841
C	-3.20555	-3.10342	-1.17315
C	-0.81561	-2.90063	-0.94399
C	-4.45156	-3.27793	-0.54680
C	-3.10693	-2.96431	-2.58077
C	-0.68887	-2.81942	-2.35292
C	-5.59452	-3.30123	-1.32218
H	-4.50166	-3.38733	0.53177
C	-4.29898	-2.98298	-3.34188
C	-1.80562	-2.83365	-3.14381
H	0.29759	-2.70644	-2.78036
C	-5.52156	-3.14613	-2.72323
H	-6.56035	-3.43438	-0.84643
H	-4.23353	-2.86829	-4.41953
H	-1.70882	-2.74693	-4.22206
H	-6.43249	-3.15920	-3.31213
C	0.33767	-3.02208	0.02249
C	1.72140	-2.59254	-0.51351
H	1.54323	-1.70537	-1.13189
H	2.14683	-3.36679	-1.15741
N	2.64105	-2.22449	0.52577
N	-2.04196	-3.09584	-0.44103
C	3.56285	-3.05152	1.15063
C	4.25678	-2.54483	2.27049
C	3.88823	-4.34703	0.70642
C	5.24639	-3.29215	2.89654
H	4.01306	-1.54923	2.63183
C	4.88719	-5.08376	1.34162
H	3.36239	-4.78734	-0.13317
C	5.57915	-4.56987	2.43745
H	5.76461	-2.86924	3.75325
H	5.11960	-6.07879	0.97072

H	6.35429	-5.15118	2.92660
H	2.29881	-1.42024	1.05125
H	0.13054	-2.41260	0.90518
Cl	0.35000	-4.77647	0.60381
C	3.74254	1.17439	-0.39338
C	3.59815	0.81203	-1.68068
C	4.79486	0.52014	0.42336
C	4.48175	-0.20877	-2.27792
H	2.86328	1.29257	-2.31434
C	5.59733	-0.40440	-0.11776
H	4.91166	0.77993	1.46804
C	5.45208	-0.78683	-1.54728
H	4.34600	-0.46267	-3.32182
H	6.35314	-0.87328	0.49875
C	-3.97124	1.45723	0.40381
C	-3.97226	1.00721	1.81996
C	-4.84923	0.90688	-0.45752
C	-4.83229	0.06749	2.23095
H	-3.29050	1.45070	2.53441
C	-5.80545	-0.11956	0.00309
H	-4.86622	1.20344	-1.49914
C	-5.79839	-0.52320	1.28084
H	-4.83273	-0.25325	3.26465
H	-6.51459	-0.54370	-0.69562
C	6.39308	-1.79412	-2.18052
H	6.15365	-1.90812	-3.26198
C	-6.51192	-3.24848	2.59852
H	-5.88916	-2.91172	3.45488
C	-7.47909	-4.33387	3.05239
H	-6.90398	-5.18412	3.47423
H	-8.16380	-3.93968	3.83322
H	-8.07294	-4.70112	2.18899
C	-7.24560	-2.05564	1.98951
H	-7.91468	-1.59505	2.74931
H	-6.51064	-1.28172	1.62997
H	-7.86196	-2.39294	1.12787
C	7.84735	-1.31712	-2.09274
H	8.51028	-2.01540	-2.64696
H	7.94582	-0.30942	-2.55028
H	8.19117	-1.26861	-1.03780
C	6.22869	-3.17392	-1.53872
H	5.18102	-3.52023	-1.66180
H	6.89457	-3.90979	-2.03770
H	6.48006	-3.14509	-0.45726

Catalyst (4-i-Pr, 6)-Product (S) alternative conformation
Electronic energy (Hartrees): -1870.504779
Free Energy at 1M concentration and 253 K (Hartrees): -1869.619304
Coordinates:

P	0.56942	0.94569	1.20885
O	1.68125	1.28490	2.33711
O	1.46254	0.82772	-0.16223
O	-0.54035	1.90963	1.06234
O	0.12789	-0.51951	1.64164
C	2.98784	1.41072	1.91656
C	3.55333	2.77171	1.74639
C	4.87775	2.92401	1.87045
H	5.32207	3.89691	1.69197
C	5.75256	1.73704	1.76976
C	7.22062	1.92721	1.81440

H	7.63594	2.88366	2.10702
C	8.03912	0.92506	1.48662
H	9.11176	1.06649	1.51681
C	7.48202	-0.36249	1.04068
H	8.15074	-1.14997	0.71694
C	6.15750	-0.54858	0.99917
H	5.80360	-1.48061	0.59770
C	5.22100	0.53033	1.43171
C	3.72157	0.34769	1.48485
C	2.13018	-0.36769	-0.24062
C	1.83536	-1.23317	-1.39485
C	2.29562	-2.48987	-1.41914
H	2.11642	-3.12347	-2.27901
C	3.03183	-3.01659	-0.24081
C	3.34775	-4.45996	-0.15320
H	3.09324	-5.12258	-0.97094
C	3.93285	-4.95411	0.94412
H	4.15556	-6.01117	1.01051
C	4.26406	-4.06358	2.07484
H	4.71835	-4.47521	2.96675
C	3.99762	-2.75418	2.00162
H	4.23504	-2.11304	2.84110
C	3.38127	-2.18482	0.77665
C	3.03812	-0.73602	0.69410
H	-0.72848	-0.84976	1.18846
C	-1.84024	-3.01394	0.50757
C	-3.17467	-1.11852	0.22538
C	-0.56685	-3.56724	0.80078
C	-2.91783	-3.88261	0.15844
C	-4.30053	-1.91682	-0.10806
C	-0.37692	-4.92937	0.73181
H	0.23975	-2.89881	1.07937
C	-2.68910	-5.28127	0.10366
C	-4.16930	-3.28273	-0.13140
H	-5.23752	-1.43136	-0.34641
C	-1.44173	-5.79389	0.38006
H	0.60040	-5.34799	0.95179
H	-3.51147	-5.93656	-0.16957
H	-5.01554	-3.91448	-0.38793
H	-1.26952	-6.86444	0.32974
C	-3.25136	0.39505	0.28986
C	-3.75330	0.91548	1.65082
H	-3.78569	2.00606	1.61034
H	-2.96605	0.66091	2.37220
N	-5.02957	0.37628	2.08378
N	-2.00467	-1.65338	0.54584
C	-6.16376	1.15958	2.32578
C	-7.12200	0.70353	3.25072
C	-6.40739	2.36984	1.65300
C	-8.28026	1.43413	3.49664
H	-6.94774	-0.23056	3.78109
C	-7.56581	3.09869	1.91893
H	-5.70984	2.72793	0.90490
C	-8.51174	2.64334	2.83766
H	-9.00171	1.05804	4.21692
H	-7.73074	4.03221	1.38793
H	-9.41241	3.21618	3.03393
H	-4.94590	-0.40007	2.72403
H	-2.26274	0.82628	0.13089
Cl	-4.27718	1.03546	-1.08070

C	1.16661	-0.57900	-2.53422
C	1.93726	0.09656	-3.40412
C	-0.24308	-0.15615	-2.35380
C	1.44072	1.34333	-4.02714
H	2.99653	-0.12103	-3.47673
C	-0.68234	0.99026	-2.88650
H	-0.89519	-0.72966	-1.71138
C	0.22626	1.83077	-3.70716
H	2.12506	1.92094	-4.63590
H	-1.69523	1.31380	-2.68872
C	2.84453	3.62821	0.76145
C	1.50246	4.18215	1.06392
C	3.24127	3.56580	-0.52892
C	0.64183	4.44445	0.06605
H	1.18887	4.30635	2.09264
C	2.29564	3.86133	-1.62258
H	4.18281	3.09924	-0.79414
C	1.02931	4.18963	-1.34226
H	-0.35912	4.78878	0.29113
H	2.59005	3.66631	-2.64492
C	-0.18513	3.22784	-4.14127
H	0.66122	3.71951	-4.67142
C	-2.46023	-2.83949	-3.12134
H	-2.26988	-1.92828	-3.72784
C	-1.35480	3.15810	-5.12866
H	-1.07923	2.52804	-6.00139
H	-2.25857	2.72783	-4.64688
H	-1.60138	4.17576	-5.49974
C	-0.53995	4.12718	-2.93475
H	-0.71885	5.16540	-3.29156
H	0.26320	4.16546	-2.13598
H	-1.48023	3.77189	-2.45790
C	-3.69331	-3.56439	-3.64331
H	-4.56770	-2.88316	-3.58887
H	-3.54111	-3.87441	-4.69894
H	-3.90306	-4.45938	-3.02025
C	-1.23083	-3.73857	-3.12981
H	-0.38021	-3.19708	-2.67068
H	-1.42075	-4.65818	-2.53835
H	-0.96353	-4.01685	-4.17112

Catalyst (4-i-Pr, 6)-Product (R) lowest free energy conformation
Electronic energy (Hartrees): -1870.600558
Free Energy at 1M concentration and 253 K (Hartrees): -1869.721393
Coordinates:

P	0.53129	0.02291	-0.06500
O	0.39978	1.51238	0.59625
O	2.04350	-0.07849	-0.67426
O	-0.30523	0.05050	-1.40131
O	0.17330	-1.00038	0.94731
C	0.92612	2.53308	-0.15946
C	0.00657	3.53304	-0.76655
C	0.50923	4.49428	-1.55789
H	-0.14606	5.23424	-2.00270
C	1.96144	4.57363	-1.81925
C	2.48496	5.61381	-2.73526
H	1.80987	6.32808	-3.19017
C	3.79283	5.67425	-3.00722
H	4.17383	6.43345	-3.67804
C	4.71945	4.69866	-2.40281

H	5.77408	4.74062	-2.64289
C	4.25798	3.75847	-1.57011
H	4.95363	3.04153	-1.15544
C	2.80882	3.69495	-1.23491
C	2.26701	2.65273	-0.31293
C	3.01159	0.37327	0.18734
C	3.88412	-0.60768	0.87931
C	4.81762	-0.17413	1.74009
H	5.45406	-0.88340	2.25684
C	4.97608	1.27073	2.00926
C	5.98826	1.72519	2.99083
H	6.63571	1.00605	3.47698
C	6.10578	3.02461	3.28322
H	6.84491	3.35512	4.00148
C	5.21893	4.01082	2.63801
H	5.30438	5.05851	2.89612
C	4.30388	3.61213	1.74694
H	3.65023	4.35194	1.30550
C	4.18034	2.17283	1.38650
C	3.16882	1.70003	0.39299
C	3.67696	-2.05350	0.65468
C	3.70800	-2.59698	-0.72310
C	3.38586	-2.87301	1.67935
C	3.42585	-3.88474	-0.94775
C	3.05606	-4.29109	1.42939
C	3.05119	-4.78109	0.17373
H	3.47194	-4.26905	-1.95797
H	2.78750	-4.91595	2.27115
C	-1.45029	3.48533	-0.49256
C	-1.90945	3.39534	0.76817
C	-2.42342	3.57522	-1.60863
C	-3.36129	3.40611	1.04094
C	-3.73918	3.58173	-1.36286
C	-4.24754	3.50388	0.03255
H	-3.69435	3.34948	2.06977
H	-4.43057	3.64873	-2.19263
H	-0.97481	-0.73357	-1.50347
C	-1.14780	-3.11474	-2.16842
C	-3.07819	-2.24897	-1.18874
C	0.14094	-2.89130	-2.72151
C	-1.68740	-4.43904	-2.17698
C	-3.67694	-3.53396	-1.15958
C	0.84693	-3.94056	-3.26424
H	0.54992	-1.88774	-2.71714
C	-0.92113	-5.50052	-2.72466
C	-2.98334	-4.61780	-1.63859
H	-4.67671	-3.64448	-0.75494
C	0.32177	-5.25556	-3.25995
H	1.82192	-3.75704	-3.70435
H	-1.33613	-6.50447	-2.71669
H	-3.42211	-5.61172	-1.61837
H	0.90482	-6.06828	-3.68149
C	-3.88385	-1.06418	-0.69340
H	-4.81353	-1.43879	-0.26369
C	-3.18511	-0.14846	0.32757
H	-3.89208	0.63212	0.61913
H	-2.34683	0.35634	-0.15280
N	-2.72825	-0.89033	1.47442
N	-1.85443	-2.05678	-1.66019
C	-3.44137	-1.00177	2.65733

C	-4.80236	-0.65459	2.76655
C	-2.78806	-1.50153	3.80415
C	-5.47186	-0.79424	3.98267
H	-5.34546	-0.27668	1.90642
C	-3.47056	-1.64010	5.00622
H	-1.73699	-1.76926	3.73461
C	-4.81989	-1.28678	5.11178
H	-6.52070	-0.51469	4.03948
H	-2.93998	-2.02393	5.87363
H	-5.34855	-1.39495	6.05345
H	-1.72380	-1.04435	1.51734
Cl	-4.41540	-0.05455	-2.13802
H	3.96948	-1.95368	-1.55421
H	3.33586	-2.49405	2.69300
H	-1.22432	3.35138	1.60575
H	-2.07209	3.62950	-2.63153
C	-5.73643	3.56688	0.32024
H	-5.91310	3.49081	1.41698
C	2.64566	-6.21208	-0.12043
H	2.70099	-6.38488	-1.21831
C	1.19401	-6.47345	0.30080
H	0.87279	-7.47858	-0.04636
H	1.08289	-6.42841	1.40496
H	0.52183	-5.71632	-0.15607
C	3.60251	-7.21049	0.54138
H	3.54726	-7.14516	1.64876
H	3.34153	-8.24610	0.23547
H	4.64649	-7.00629	0.22073
C	-6.48139	2.39807	-0.33455
H	-7.55965	2.43920	-0.07055
H	-6.38874	2.43075	-1.44057
H	-6.07793	1.43258	0.03370
C	-6.31926	4.90905	-0.13620
H	-6.24879	5.02169	-1.23900
H	-7.38807	4.98007	0.15857
H	-5.77071	5.74615	0.34640

Catalyst (4-i-Pr, 6)-Product (R) alternative conformation
Electronic energy (Hartrees): -1870.600516
Free Energy at 1M concentration and 253 K (Hartrees): -1869.721357
Coordinates:

P	0.51959	-0.01010	-0.07770
O	0.58252	1.49631	0.55549
O	2.01815	-0.33570	-0.64140
O	-0.27160	0.11142	-1.43540
O	-0.00223	-0.95369	0.94072
C	1.28618	2.41314	-0.18926
C	0.55206	3.52337	-0.85469
C	1.22751	4.39077	-1.62547
H	0.70829	5.20802	-2.11277
C	2.68699	4.25769	-1.81124
C	3.40011	5.19974	-2.70518
H	2.85895	5.99870	-3.19672
C	4.71450	5.06677	-2.91166
H	5.23404	5.75465	-3.56621
C	5.45870	3.97428	-2.25736
H	6.51851	3.85959	-2.44515
C	4.82536	3.12108	-1.44419
H	5.38852	2.31541	-0.99304
C	3.36781	3.27284	-1.18059

C	2.63587	2.33283	-0.27990
C	3.01412	-0.02763	0.25149
C	3.71555	-1.12327	0.96635
C	4.66885	-0.82625	1.86311
H	5.18363	-1.61851	2.39444
C	5.01840	0.58159	2.14799
C	6.04598	0.88811	3.17017
H	6.56519	0.08456	3.67762
C	6.33562	2.15787	3.47321
H	7.08559	2.38042	4.22123
C	5.62426	3.25982	2.79847
H	5.84820	4.28494	3.06419
C	4.69730	2.99467	1.87070
H	4.17381	3.81940	1.40666
C	4.38402	1.58732	1.49958
C	3.35447	1.26275	0.46638
C	3.32641	-2.52687	0.71408
C	3.31833	-3.05117	-0.67167
C	2.91526	-3.31848	1.71949
C	2.88420	-4.29082	-0.92266
C	2.41974	-4.68190	1.44032
C	2.38123	-5.15044	0.17707
H	2.90322	-4.66349	-1.93811
H	2.06152	-5.28158	2.26688
C	-0.91056	3.68290	-0.67018
C	-1.45237	3.68817	0.56045
C	-1.79242	3.88097	-1.84662
C	-2.90148	3.91263	0.74070
C	-3.10589	4.08036	-1.68426
C	-3.70204	4.11225	-0.32499
H	-3.30006	3.93120	1.74660
H	-3.73193	4.22161	-2.55628
H	-1.04707	-0.56752	-1.54348
C	-1.55839	-2.90234	-2.20184
C	-3.35368	-1.75437	-1.25601
C	-0.23987	-2.87714	-2.72904
C	-2.29024	-4.13089	-2.21895
C	-4.13971	-2.93444	-1.23955
C	0.31188	-4.02334	-3.25421
H	0.31454	-1.94607	-2.71736
C	-1.68113	-5.29778	-2.74908
C	-3.60844	-4.11139	-1.70608
H	-5.15156	-2.89283	-0.85234
C	-0.40485	-5.24458	-3.25854
H	1.31200	-3.99066	-3.67434
H	-2.24228	-6.22802	-2.74821
H	-4.19185	-5.02803	-1.69483
H	0.05828	-6.13770	-3.66602
C	-3.97832	-0.46486	-0.76100
H	-4.96860	-0.69674	-0.36816
C	-3.17698	0.30649	0.30431
H	-3.76197	1.18178	0.59977
H	-2.25740	0.68432	-0.14161
N	-2.86834	-0.52404	1.43876
N	-2.10735	-1.74883	-1.70681
C	-3.66301	-0.61976	2.56849
C	-4.97690	-0.11371	2.62430
C	-3.15058	-1.26599	3.71393
C	-5.73641	-0.24376	3.78735
H	-5.41245	0.38187	1.76304

C	-3.92184	-1.39231	4.86246
H	-2.13764	-1.65898	3.68650
C	-5.22322	-0.88187	4.91516
H	-6.74541	0.16011	3.80300
H	-3.49908	-1.89207	5.73009
H	-5.82118	-0.98187	5.81535
H	-1.89407	-0.80424	1.51638
Cl	-4.30056	0.64623	-2.19124
H	3.67514	-2.43438	-1.48726
H	2.89330	-2.95139	2.73848
H	-0.83165	3.56832	1.43977
H	-1.37644	3.85822	-2.84620
C	-5.18361	4.40065	-0.16487
H	-5.63440	4.54425	-1.17187
C	1.81524	-6.51910	-0.14725
H	1.86885	-6.68092	-1.24691
C	0.33593	-6.61436	0.24712
H	-0.09492	-7.56924	-0.12244
H	0.21186	-6.57390	1.35006
H	-0.23480	-5.77644	-0.20666
C	2.63692	-7.63273	0.51234
H	2.57158	-7.57702	1.61970
H	2.26109	-8.62591	0.18597
H	3.70283	-7.54846	0.21036
C	-5.41286	5.70021	0.61675
H	-6.49503	5.95117	0.62836
H	-5.06229	5.60370	1.66624
H	-4.86828	6.53801	0.13089
C	-5.92121	3.22734	0.49136
H	-5.54016	3.03537	1.51670
H	-7.00785	3.44882	0.55541
H	-5.79675	2.31009	-0.12083

Catalyst (4-i-Pr, 6)-Product (S) alternative conformation
Electronic energy (Hartrees): -1870.599159
Free Energy at 1M concentration and 253 K (Hartrees): -1869.71898
Coordinates:

P	0.24141	0.15712	0.11735
O	-0.34559	1.58521	0.63982
O	1.79437	0.40496	-0.35197
O	-0.42522	-0.10939	-1.28654
O	0.03109	-0.86155	1.17406
C	-0.04974	2.65631	-0.16356
C	-1.14072	3.31234	-0.93575
C	-0.84572	4.34456	-1.74208
H	-1.62815	4.83835	-2.30685
C	0.54396	4.82594	-1.87480
C	0.84738	5.93440	-2.80990
H	0.04921	6.41295	-3.36371
C	2.10783	6.34765	-2.97693
H	2.32839	7.15637	-3.66165
C	3.20393	5.69220	-2.23969
H	4.22503	6.01416	-2.39887
C	2.94034	4.69388	-1.38877
H	3.76532	4.21209	-0.88242
C	1.53813	4.24384	-1.16489
C	1.21416	3.13277	-0.21860
C	2.54282	1.18604	0.49580
C	3.77652	0.61767	1.11305
C	4.53296	1.39176	1.90863

H	5.43528	0.99481	2.35962
C	4.14647	2.78795	2.19207
C	4.94990	3.59544	3.13940
H	5.82699	3.16895	3.61015
C	4.59517	4.85430	3.41865
H	5.18210	5.44318	4.11163
C	3.39647	5.44144	2.79029
H	3.10451	6.45476	3.03421
C	2.66707	4.72185	1.92977
H	1.78152	5.16575	1.49457
C	3.06376	3.32819	1.58924
C	2.27227	2.50825	0.62514
C	4.22894	-0.75682	0.78666
C	5.61578	-0.98394	0.30971
C	3.40822	-1.81101	0.92708
C	6.03790	-2.21813	0.01142
C	3.86839	-3.17279	0.58735
C	5.12199	-3.38146	0.14458
H	7.05457	-2.35973	-0.33120
H	3.17574	-3.99710	0.70477
C	-2.54131	2.83278	-0.83472
C	-3.12269	2.64238	0.36287
C	-3.33527	2.59591	-2.06589
C	-4.53964	2.23128	0.45175
C	-4.61817	2.22394	-1.98601
C	-5.27070	2.04401	-0.66518
H	-4.98120	2.10771	1.43197
H	-5.17883	2.05684	-2.89709
H	-0.79178	-1.07402	-1.39439
C	-0.08638	-3.41927	-1.75050
C	-2.33028	-3.15814	-1.17063
C	1.15585	-2.82908	-2.10537
C	-0.18264	-4.84380	-1.67438
C	-2.50481	-4.56265	-1.08764
C	2.24684	-3.62629	-2.36585
H	1.23082	-1.75003	-2.16480
C	0.96508	-5.63412	-1.94242
C	-1.43947	-5.39451	-1.32998
H	-3.47710	-4.96256	-0.82263
C	2.15687	-5.03723	-2.28091
H	3.19150	-3.16371	-2.63354
H	0.88343	-6.71546	-1.87673
H	-1.54993	-6.47330	-1.26064
H	3.03141	-5.64683	-2.48425
C	-3.51728	-2.26074	-0.88097
H	-4.35095	-2.89002	-0.56786
C	-3.27384	-1.16718	0.17723
H	-4.21486	-0.63555	0.33829
H	-2.56822	-0.43772	-0.21954
N	-2.76766	-1.71885	1.40721
N	-1.16406	-2.61554	-1.48882
C	-3.56470	-2.07049	2.48391
C	-4.96315	-2.21075	2.38617
C	-2.95775	-2.31939	3.73351
C	-5.71784	-2.57197	3.50253
H	-5.46810	-2.04283	1.44073
C	-3.72251	-2.68483	4.83429
H	-1.88001	-2.21244	3.82460
C	-5.11180	-2.81343	4.73420
H	-6.79557	-2.66789	3.39876

H	-3.22736	-2.86585	5.78471
H	-5.70526	-3.09686	5.59756
H	-1.78622	-1.52482	1.58890
Cl	-4.09768	-1.46738	-2.43443
H	6.29406	-0.14749	0.19484
H	2.40907	-1.68551	1.31424
H	-2.57749	2.82879	1.27973
H	-2.87648	2.71825	-3.03918
C	-6.74542	1.68919	-0.60319
H	-7.14375	1.61904	-1.63969
C	5.59558	-4.78182	-0.19527
H	4.75241	-5.50150	-0.08948
C	6.69624	-5.22918	0.77247
H	6.98617	-6.28035	0.55979
H	7.59746	-4.58666	0.67723
H	6.32688	-5.17629	1.81904
C	6.07917	-4.86697	-1.64817
H	6.99885	-4.26399	-1.80211
H	6.30350	-5.92269	-1.91128
H	5.29228	-4.49823	-2.33821
C	-7.54992	2.78304	0.11031
H	-8.63635	2.56074	0.04343
H	-7.27022	2.85131	1.18301
H	-7.36739	3.76677	-0.37289
C	-6.97131	0.32298	0.05431
H	-6.60486	0.31642	1.10271
H	-8.05437	0.07619	0.05814
H	-6.44542	-0.46649	-0.52246

Catalyst (4-i-Pr, 6)-Product (S) alternative conformation
Electronic energy (Hartrees): -1870.605525
Free Energy at 1M concentration and 253 K (Hartrees): -1869.718315
Coordinates:

P	-0.62577	0.23311	-0.05065
O	-0.21279	1.57414	-0.89503
O	-1.99650	0.59484	0.76146
O	0.39431	-0.26158	0.90052
O	-1.02345	-0.71611	-1.24919
C	-0.20849	2.71567	-0.13264
C	1.08648	3.35805	0.22745
C	1.08271	4.44137	1.02191
H	2.01349	4.93046	1.28506
C	-0.18531	4.99815	1.53559
C	-0.15641	6.16438	2.44895
H	0.78760	6.62270	2.71651
C	-1.29508	6.65512	2.94944
H	-1.27258	7.50607	3.61792
C	-2.58512	6.03030	2.60179
H	-3.50135	6.41909	3.02712
C	-2.62276	4.97981	1.77393
H	-3.57866	4.52516	1.55197
C	-1.36647	4.43956	1.18491
C	-1.37914	3.26707	0.26018
C	-2.91569	1.35823	0.08141
C	-4.23823	0.76550	-0.25872
C	-5.14115	1.50544	-0.92461
H	-6.11414	1.09427	-1.16741
C	-4.83139	2.89214	-1.32654
C	-5.82329	3.66756	-2.10761
H	-6.78112	3.22908	-2.35876

C	-5.54027	4.91159	-2.50793
H	-6.26520	5.47667	-3.07947
C	-4.23383	5.51361	-2.18203
H	-4.00292	6.51333	-2.52679
C	-3.33285	4.82364	-1.47321
H	-2.37386	5.27857	-1.26517
C	-3.64367	3.44819	-0.99484
C	-2.65813	2.65825	-0.19733
H	-0.53450	-1.62013	-1.24249
C	-0.57854	-4.17233	-1.03048
C	1.52433	-3.16397	-1.05924
C	-1.97861	-4.06024	-1.22712
C	-0.01592	-5.44073	-0.69286
C	2.15614	-4.38107	-0.69262
C	-2.77588	-5.17826	-1.12212
H	-2.39423	-3.08878	-1.47311
C	-0.86955	-6.56653	-0.57300
C	1.38797	-5.50217	-0.50590
H	3.22860	-4.39903	-0.55473
C	-2.22162	-6.43875	-0.79516
H	-3.84325	-5.10111	-1.29778
H	-0.43686	-7.52892	-0.31447
H	1.84672	-6.44616	-0.22442
H	-2.87205	-7.30402	-0.71356
C	2.31259	-1.89923	-1.32883
H	1.62236	-1.13538	-1.67931
C	3.10390	-1.31734	-0.16141
H	2.38236	-1.20520	0.65191
H	3.41488	-0.30224	-0.44664
N	4.22616	-2.13824	0.25839
N	0.21004	-3.06532	-1.19904
C	5.04932	-1.65597	1.29005
C	4.62866	-0.65946	2.18715
C	6.34101	-2.19434	1.43959
C	5.48519	-0.22027	3.19767
H	3.64149	-0.22031	2.09976
C	7.18559	-1.74480	2.44802
H	6.67685	-2.96544	0.74994
C	6.76592	-0.75144	3.33744
H	5.14106	0.55508	3.87663
H	8.18088	-2.17143	2.53722
H	7.42752	-0.39913	4.12246
H	4.77287	-2.45692	-0.53432
Cl	3.42823	-2.18687	-2.77671
C	2.37231	2.83190	-0.29382
C	2.52345	2.55576	-1.60121
C	3.53302	2.66291	0.61617
C	3.83122	2.11570	-2.12921
H	1.70521	2.69689	-2.29633
C	4.71919	2.26924	0.13810
H	3.42392	2.85246	1.67678
C	4.89349	1.99093	-1.31211
H	3.92031	1.91810	-3.19015
H	5.55075	2.15188	0.82074
C	-4.56242	-0.62364	0.15038
C	-4.28349	-1.07294	1.53887
C	-5.10514	-1.49500	-0.72178
C	-4.54145	-2.33236	1.90807
H	-3.88259	-0.37935	2.26708
C	-5.40263	-2.88100	-0.30577

H	-5.30949	-1.20282	-1.74449
C	-5.12101	-3.28765	0.94013
H	-4.33558	-2.65114	2.92136
H	-5.83589	-3.57243	-1.01654
C	6.24663	1.58614	-1.86528
H	6.17678	1.43944	-2.96682
C	-4.79335	-5.75808	2.84633
H	-5.04402	-5.03264	3.64953
C	-3.28447	-5.81388	2.64073
H	-2.90386	-4.84869	2.24474
H	-2.78897	-6.02410	3.61111
H	-3.03150	-6.63042	1.93413
C	-5.53205	-5.38598	1.56181
H	-5.24720	-6.08497	0.74543
H	-5.32106	-4.32298	1.24825
H	-6.62391	-5.49425	1.73704
C	7.28569	2.68684	-1.62402
H	7.47137	2.83198	-0.53860
H	8.24603	2.41691	-2.11300
H	6.93243	3.64584	-2.05995
C	6.70989	0.25570	-1.26477
H	5.96031	-0.53378	-1.47824
H	7.67422	-0.05396	-1.72082
H	6.84966	0.33852	-0.16632

Catalyst (4-i-Pr, 6)-Product (S) alternative conformation
Electronic energy (Hartrees): -1870.594037
Free Energy at 1M concentration and 253 K (Hartrees): -1869.71577
Coordinates:

P	-0.03255	0.48109	-0.06436
O	1.51782	0.04670	-0.32482
O	0.02277	1.85188	0.81127
O	-0.65044	-0.52219	0.97306
O	-0.71848	0.58244	-1.37930
C	2.48599	0.49836	0.54430
C	3.37365	-0.51465	1.17401
C	4.44379	-0.12458	1.87992
H	5.08866	-0.86259	2.34373
C	4.70045	1.30790	2.11986
C	5.82607	1.71211	2.99442
H	6.49374	0.96361	3.40280
C	6.02095	3.00194	3.28713
H	6.84252	3.29584	3.92758
C	5.10550	4.02694	2.75270
H	5.25178	5.06658	3.01601
C	4.08912	3.67286	1.95792
H	3.41567	4.44064	1.60202
C	3.88037	2.24419	1.58969
C	2.76870	1.82488	0.67919
C	0.62438	2.85748	0.10457
C	-0.21991	3.88911	-0.55293
C	0.36210	4.82320	-1.31972
H	-0.24119	5.57257	-1.81890
C	1.82570	4.81271	-1.53438
C	2.43954	5.82164	-2.42884
H	1.82865	6.59669	-2.87479
C	3.74950	5.77824	-2.69335
H	4.19613	6.51480	-3.34866
C	4.58723	4.71518	-2.10737
H	5.64139	4.66892	-2.34864

C	4.04475	3.80172	-1.29411
H	4.67421	3.01502	-0.90142
C	2.59571	3.85826	-0.95723
C	1.96463	2.85289	-0.04795
H	-1.61920	-0.80773	0.72187
C	-3.92196	-1.15079	1.58811
C	-3.54329	-1.93392	-0.58360
C	-3.46106	-0.40471	2.70409
C	-5.23653	-1.70819	1.61104
C	-4.82461	-2.53536	-0.62470
C	-4.28577	-0.21911	3.79060
H	-2.46382	0.02024	2.67464
C	-6.05592	-1.50426	2.75142
C	-5.65428	-2.42721	0.46580
H	-5.13694	-3.05502	-1.52053
C	-5.59032	-0.77122	3.81844
H	-3.93413	0.36124	4.63807
H	-7.05608	-1.92830	2.76113
H	-6.64314	-2.87777	0.45145
H	-6.22211	-0.61025	4.68631
C	-2.57801	-1.91145	-1.76176
H	-2.25885	-0.87435	-1.88479
C	-1.32320	-2.78270	-1.56705
H	-1.57024	-3.82162	-1.79799
H	-1.04436	-2.74435	-0.50105
N	-0.26029	-2.30418	-2.43242
N	-3.10864	-1.29411	0.49675
C	0.93379	-3.01008	-2.59954
C	0.98224	-4.41421	-2.64862
C	2.13488	-2.29658	-2.78438
C	2.18995	-5.07352	-2.87358
H	0.07307	-4.99411	-2.52972
C	3.33104	-2.96575	-3.01949
H	2.11807	-1.21249	-2.72412
C	3.37393	-4.36142	-3.06291
H	2.19958	-6.15988	-2.90360
H	4.24255	-2.38930	-3.15476
H	4.31106	-4.88154	-3.23445
H	-0.12533	-1.30189	-2.33351
Cl	-3.39825	-2.33375	-3.33438
C	3.05015	-1.95208	1.07790
C	1.93454	-2.43142	1.65008
C	3.98790	-2.88872	0.41756
C	1.65125	-3.87984	1.63958
H	1.25911	-1.77071	2.17747
C	3.72858	-4.20254	0.39981
H	4.88560	-2.51167	-0.05642
C	2.50791	-4.74024	1.05498
H	0.76274	-4.23489	2.14487
H	4.42538	-4.87362	-0.08640
C	-1.69659	3.80376	-0.45020
C	-2.52391	3.78522	-1.68124
C	-2.29320	3.61689	0.74079
C	-3.83608	3.53032	-1.61147
H	-2.06341	3.94525	-2.64820
C	-3.74020	3.33633	0.81876
H	-1.71558	3.62279	1.65701
C	-4.48542	3.27080	-0.30157
H	-4.41921	3.50020	-2.52329
H	-4.17806	3.14747	1.79038

C	2.28842	-6.24071	1.11364
H	3.08282	-6.74464	0.51952
C	2.40918	-6.75277	2.55385
H	1.60434	-6.33407	3.19504
H	2.33609	-7.86117	2.57187
H	3.39404	-6.46353	2.97933
C	0.94250	-6.64857	0.50113
H	0.86748	-6.27522	-0.53856
H	0.85826	-7.75590	0.47465
H	0.09124	-6.24653	1.08980
C	-5.95749	2.90918	-0.25446
H	-6.36635	2.92306	-1.28910
C	-6.75964	3.93170	0.55942
H	-6.59484	4.95341	0.15529
H	-7.84464	3.70267	0.49236
H	-6.46225	3.91543	1.62939
C	-6.15948	1.48890	0.28879
H	-5.84024	1.41692	1.35010
H	-7.23102	1.20377	0.22331
H	-5.57069	0.76411	-0.31352

Catalyst (3,5-(i-Pr)2, 7)-THF complex
Electronic energy (Hartrees): -876.458087
Free Energy at 1M concentration and 253 K (Hartrees): -875.565037
Coordinates:

P	0.04819	-0.79180	-0.12307
O	0.91594	0.33315	0.69253
O	-1.34990	-0.08076	-0.58965
O	0.72706	-1.37912	-1.30072
O	-0.30381	-1.76616	1.06703
C	1.11799	1.46557	-0.05037
C	2.45815	1.73222	-0.63335
C	2.62953	2.79490	-1.43449
H	3.60268	3.00315	-1.86443
C	1.50091	3.70112	-1.73255
C	1.70359	4.84516	-2.65169
H	2.68097	5.03399	-3.07812
C	0.68065	5.64869	-2.96112
H	0.82957	6.48333	-3.63401
C	-0.65784	5.39198	-2.39724
H	-1.48674	6.03207	-2.67076
C	-0.85103	4.36448	-1.56220
H	-1.84725	4.18223	-1.18318
C	0.28406	3.47910	-1.18172
C	0.09649	2.32221	-0.25390
C	-1.87154	0.90540	0.21895
C	-3.28467	0.77640	0.68011
C	-3.84060	1.75812	1.40729
H	-4.86916	1.67587	1.74000
C	-3.05974	2.95230	1.78104
C	-3.66384	3.97900	2.66192
H	-4.68182	3.86407	3.01301
C	-2.95440	5.05130	3.02940
H	-3.39596	5.80016	3.67418
C	-1.56331	5.21152	2.56574
H	-0.98793	6.07196	2.88223
C	-1.01067	4.29230	1.76580
H	0.01743	4.42072	1.45439
C	-1.79628	3.10613	1.32610
C	-1.20537	2.07158	0.42458

C	-4.10818	-0.38575	0.27474
C	-5.39650	-0.16826	-0.42540
C	-3.70397	-1.63797	0.53534
C	-6.15152	-1.21044	-0.81695
C	-4.51116	-2.80526	0.09929
C	-5.67512	-2.59164	-0.54062
C	3.59962	0.84086	-0.33144
C	3.95447	0.59493	0.94035
C	4.38200	0.24077	-1.43699
C	5.14646	-0.23756	1.24770
C	5.44692	-0.53970	-1.18067
C	5.84337	-0.77920	0.23222
H	0.18498	-2.64740	0.98739
O	1.16438	-3.88357	0.86697
C	2.44760	-3.46741	1.40207
C	1.33319	-4.39583	-0.48535
C	3.50043	-3.97785	0.41782
H	2.45432	-2.37293	1.46982
H	2.54371	-3.88361	2.40910
C	2.73303	-3.96221	-0.91313
H	1.22511	-5.48692	-0.44740
H	0.54775	-3.96554	-1.10848
H	3.80673	-4.99710	0.67792
H	4.39395	-3.34867	0.40981
H	3.16321	-4.62819	-1.66594
H	2.68154	-2.94868	-1.31961
H	-5.71778	0.84685	-0.62659
H	-2.78977	-1.80298	1.08361
H	4.07692	0.43442	-2.45848
H	3.39233	1.04083	1.75162
H	6.71577	-1.38607	0.43825
H	-6.27553	-3.43140	-0.86660
C	5.60267	-0.43327	2.68242
H	6.53567	-1.04059	2.70445
C	5.93283	0.91217	3.33962
H	6.36796	0.74584	4.34819
H	6.67923	1.46223	2.72733
H	5.02357	1.54035	3.44883
C	4.55417	-1.19805	3.49563
H	4.90202	-1.32726	4.54273
H	3.58225	-0.66069	3.50850
H	4.40731	-2.20756	3.06027
C	6.24550	-1.14681	-2.31909
H	5.80140	-0.84778	-3.29540
C	7.68974	-0.63298	-2.30889
H	8.23004	-0.97010	-1.39907
H	8.23352	-1.01023	-3.20120
H	7.69871	0.47760	-2.34172
C	6.20711	-2.67773	-2.26082
H	6.74903	-3.10572	-3.13100
H	6.67808	-3.05766	-1.32959
H	5.15600	-3.03109	-2.30301
C	-4.02189	-4.21798	0.35857
H	-4.75905	-4.95298	-0.03641
C	-2.69652	-4.47723	-0.36735
H	-1.88950	-3.82712	0.02893
H	-2.39077	-5.53714	-0.23642
H	-2.81268	-4.28075	-1.45465
C	-3.88489	-4.48403	1.86203
H	-3.09497	-3.84678	2.31312

H	-4.84917	-4.27865	2.37432
H	-3.61949	-5.54863	2.03631
C	-7.46617	-0.97762	-1.53764
H	-7.64903	0.11510	-1.64900
C	-8.63963	-1.54780	-0.73296
H	-9.60026	-1.29219	-1.22902
H	-8.56968	-2.65301	-0.64873
H	-8.64929	-1.10947	0.28797
C	-7.42821	-1.57131	-2.95038
H	-7.33023	-2.67714	-2.91914
H	-8.36200	-1.31551	-3.49519
H	-6.57001	-1.15040	-3.51685

Catalyst (3,5-(i-Pr)2, 7)-Quinoline complex
Electronic energy (Hartrees): -1582.941784
Free Energy at 1M concentration and 253 K (Hartrees): -1582.012193
Coordinates:

P	-0.20719	0.18571	0.97916
O	0.39003	0.99626	-0.33287
O	-1.62675	0.90483	1.33166
O	0.69625	0.18033	2.15011
O	-0.61927	-1.18779	0.33364
C	0.40778	2.35639	-0.14619
C	1.69825	3.03872	0.11969
C	1.70833	4.35187	0.39434
H	2.64443	4.85896	0.59832
C	0.44626	5.12282	0.42153
C	0.47461	6.56342	0.76561
H	1.41786	7.05671	0.96478
C	-0.66433	7.26100	0.83494
H	-0.64235	8.31312	1.08813
C	-1.95599	6.59607	0.57805
H	-2.87527	7.16234	0.65482
C	-1.99314	5.29643	0.26199
H	-2.95284	4.82553	0.09664
C	-0.73287	4.51241	0.15369
C	-0.74654	3.05967	-0.19038
C	-2.37064	1.27203	0.23398
C	-3.56920	0.47513	-0.12309
C	-4.30242	0.81347	-1.19317
H	-5.16464	0.21851	-1.47217
C	-3.91570	1.96759	-2.03050
C	-4.71948	2.30175	-3.22914
H	-5.61416	1.73649	-3.45907
C	-4.34274	3.29987	-4.03485
H	-4.93002	3.54188	-4.91125
C	-3.11669	4.06462	-3.74039
H	-2.80442	4.85574	-4.40991
C	-2.38515	3.77401	-2.65843
H	-1.47754	4.33368	-2.47984
C	-2.80725	2.68686	-1.73229
C	-2.00944	2.34081	-0.51570
C	-3.87011	-0.75382	0.63387
C	-4.16972	-0.67234	2.07964
C	-3.75002	-1.95394	0.04564
C	-4.32156	-1.79169	2.81250
C	-3.89309	-3.20160	0.83795
C	-4.17440	-3.11338	2.15126
C	2.95787	2.26551	0.11080
C	3.29993	1.53380	-0.96211

C	3.82748	2.25690	1.30907
C	4.55184	0.73472	-0.96709
C	4.94728	1.51239	1.33868
C	5.32228	0.72235	0.13579
H	0.14211	-1.75957	-0.04427
C	2.24702	-3.21206	0.17497
C	1.09660	-3.30725	-1.85745
C	2.44920	-2.57521	1.42800
C	3.08254	-4.31267	-0.19711
C	1.87097	-4.41802	-2.28615
C	3.45008	-3.02066	2.26280
H	1.83009	-1.73070	1.71608
C	4.09780	-4.74604	0.69262
C	2.85031	-4.90922	-1.45982
H	1.68823	-4.85198	-3.26062
C	4.27958	-4.10906	1.89886
H	3.60477	-2.52636	3.21654
H	4.72922	-5.58118	0.40223
H	3.46237	-5.75122	-1.77191
H	5.06181	-4.43793	2.57609
N	1.26807	-2.74901	-0.66290
C	0.09002	-2.68377	-2.75461
C	-0.19929	-1.38227	-2.79184
H	-0.95054	-0.99396	-3.46901
H	0.30986	-0.68373	-2.13892
Cl	-0.73479	-3.78983	-3.86446
H	-4.25773	0.30395	2.53966
H	-3.48804	-2.01372	-1.00395
H	2.66302	1.53288	-1.83827
H	3.53064	2.83699	2.17481
H	6.23280	0.13709	0.15281
H	-4.27131	-4.01266	2.74733
C	-3.66670	-4.55298	0.18771
H	-3.82862	-5.36479	0.93222
C	-4.61158	-1.72927	4.30006
H	-4.69443	-2.76529	4.69703
C	5.80889	1.47222	2.58640
H	5.37434	2.13476	3.36875
C	4.96188	-0.04792	-2.20095
H	5.94145	-0.54725	-2.02516
C	5.85067	0.05625	3.17175
H	6.32848	-0.65689	2.46688
H	6.42599	0.05363	4.12204
H	4.81900	-0.29219	3.39085
C	7.22190	1.99070	2.29611
H	7.17053	3.01191	1.86135
H	7.80744	2.04443	3.23869
H	7.75743	1.32454	1.58717
C	3.94683	-1.15193	-2.50957
H	2.94270	-0.72931	-2.72443
H	4.27857	-1.74375	-3.38906
H	3.87312	-1.83863	-1.64277
C	5.14491	0.88241	-3.40548
H	4.18180	1.34777	-3.70421
H	5.87181	1.68575	-3.15896
H	5.54178	0.30963	-4.27070
C	-5.95037	-1.03495	4.57829
H	-6.76496	-1.54038	4.01663
H	-6.18928	-1.09124	5.66177
H	-5.91775	0.03438	4.27991

C	-3.46661	-1.04837	5.06049
H	-3.37380	0.02066	4.77374
H	-3.65057	-1.10525	6.15458
H	-2.50620	-1.56298	4.84260
C	-4.66086	-4.78673	-0.95537
H	-4.49904	-4.06424	-1.78324
H	-4.54098	-5.81515	-1.35804
H	-5.70184	-4.67927	-0.58225
C	-2.22056	-4.67783	-0.30712
H	-2.04233	-5.69515	-0.71598
H	-2.00722	-3.93505	-1.10297
H	-1.51296	-4.51151	0.53343

Catalyst (3,5-(i-Pr)2, 7)-Enamine protonation TS (pro-S) lowest free energy conformation
Electronic energy (Hartrees): -1870.553523
Free Energy at 1M concentration and 253 K (Hartrees): -1869.508189
Coordinates:

P	-0.70738	0.01550	-0.20290
O	-1.94944	0.24199	0.83851
O	-0.60655	1.39421	-1.07961
O	-0.88126	-1.12781	-1.14669
O	0.52258	-0.02193	0.75725
C	-3.07446	0.74948	0.23859
C	-4.22654	-0.14740	-0.00669
C	-5.31446	0.31859	-0.63415
H	-6.15086	-0.34084	-0.83580
C	-5.37338	1.72930	-1.07592
C	-6.57011	2.22087	-1.79733
H	-7.41317	1.56202	-1.96459
C	-6.61425	3.47913	-2.24793
H	-7.48875	3.83656	-2.77617
C	-5.46565	4.38075	-2.03835
H	-5.49980	5.39136	-2.42455
C	-4.38089	3.94902	-1.38464
H	-3.54296	4.62184	-1.26274
C	-4.32687	2.56091	-0.85025
C	-3.12665	2.05205	-0.11925
C	-0.73517	2.54727	-0.34215
C	0.46507	3.38894	-0.10578
C	0.36152	4.49484	0.64720
H	1.23174	5.11568	0.82686
C	-0.93584	4.87802	1.24338
C	-1.01728	6.07858	2.10751
H	-0.13969	6.69172	2.27101
C	-2.17337	6.40942	2.69223
H	-2.23102	7.28589	3.32469
C	-3.37116	5.57329	2.48790
H	-4.29821	5.83461	2.98178
C	-3.31039	4.48948	1.70562
H	-4.19639	3.88028	1.58958
C	-2.03910	4.12378	1.02254
C	-1.94292	2.91741	0.14618
C	1.76534	2.98845	-0.68758
C	1.85972	2.71214	-2.13963
C	2.84569	2.81159	0.09201
C	3.00566	2.26733	-2.68267
C	4.12238	2.32252	-0.48588
C	4.18563	2.05692	-1.80487
C	-4.12731	-1.56554	0.37618

C	-4.08061	-1.91578	1.66994
C	-3.92871	-2.58985	-0.67072
C	-3.85158	-3.33023	2.06152
C	-3.68349	-3.87099	-0.33691
C	-3.66049	-4.25160	1.09865
H	0.40416	-2.41327	-1.19501
C	1.91499	-2.74573	-2.55826
C	2.15908	-2.58768	-0.15330
C	1.04974	-2.76783	-3.66823
C	3.32008	-2.81243	-2.73635
C	3.58288	-2.57139	-0.32454
C	1.58614	-2.89431	-4.93704
H	-0.01835	-2.66508	-3.51061
C	3.83275	-2.95111	-4.04450
C	4.13375	-2.69930	-1.56681
H	4.20359	-2.46583	0.55404
C	2.97892	-2.99883	-5.13046
H	0.92257	-2.91008	-5.79599
H	4.90874	-3.00786	-4.18225
H	5.21448	-2.72121	-1.67871
H	3.37820	-3.10244	-6.13400
C	1.51580	-2.43358	1.11370
C	2.34761	-2.43754	2.38902
H	3.06844	-3.26745	2.40107
H	1.67290	-2.59607	3.24187
N	3.07544	-1.17660	2.48486
N	1.40693	-2.65294	-1.28314
C	3.97608	-0.90303	3.51330
C	4.31391	0.43941	3.77912
C	4.60160	-1.91023	4.27015
C	5.26256	0.75828	4.74229
H	3.81942	1.22767	3.21705
C	5.55299	-1.57556	5.23491
H	4.34858	-2.95266	4.11188
C	5.89689	-0.24694	5.47870
H	5.50455	1.80210	4.92282
H	6.02432	-2.37124	5.80562
H	6.63647	0.00341	6.23248
H	2.51164	-0.37893	2.21608
H	0.94838	-1.05130	0.92323
Cl	-0.00826	-3.39026	1.29451
H	2.77761	2.98851	1.15907
H	0.98641	2.87356	-2.76045
H	-3.94123	-2.28310	-1.70916
H	-4.19144	-1.15750	2.43555
H	-3.48100	-5.28614	1.36484
H	5.10434	1.68562	-2.23886
C	5.32039	2.11036	0.41987
H	5.04347	2.39491	1.45808
C	6.49485	3.00815	0.01273
H	7.32105	2.90854	0.74874
H	6.88229	2.73282	-0.99106
H	6.17235	4.07121	-0.00551
C	5.73761	0.63494	0.45070
H	6.13476	0.30812	-0.53369
H	6.52264	0.47736	1.22075
H	4.86513	0.00089	0.70951
C	3.08611	1.97288	-4.16815
H	2.11823	2.22436	-4.65794
C	3.33440	0.48159	-4.40891

H	2.53301	-0.11260	-3.92187
H	3.31675	0.26088	-5.49746
H	4.31678	0.16528	-3.99823
C	4.16439	2.82805	-4.84323
H	5.17758	2.56577	-4.47189
H	4.14117	2.66922	-5.94252
H	3.97577	3.90475	-4.64429
C	-3.82086	-3.72787	3.52534
H	-3.63756	-4.82225	3.61717
C	-2.67942	-3.01865	4.26372
H	-2.82935	-1.91835	4.27556
H	-2.62499	-3.37793	5.31352
H	-1.70964	-3.24341	3.77217
C	-5.16901	-3.44112	4.19595
H	-5.38467	-2.35166	4.21325
H	-5.98447	-3.96018	3.64809
H	-5.16084	-3.81587	5.24177
C	-3.40427	-4.92303	-1.39320
H	-3.22957	-5.90145	-0.89331
C	-4.60458	-5.10586	-2.32995
H	-4.79079	-4.18856	-2.92783
H	-4.41601	-5.94907	-3.02823
H	-5.51566	-5.34143	-1.73927
C	-2.13173	-4.58989	-2.18282
H	-1.88909	-5.41849	-2.88168
H	-2.25939	-3.65768	-2.77260
H	-1.27446	-4.46058	-1.48806

Catalyst (3,5-(i-Pr)2, 7)-Enamine protonation TS (pro-S) alternative conformation
Electronic energy (Hartrees): -1870.553251
Free Energy at 1M concentration and 253 K (Hartrees): -1869.50801
Coordinates:

P	0.68152	-0.01577	0.13784
O	1.88700	0.23183	-0.94113
O	0.56055	1.37586	0.99362
O	0.91944	-1.13510	1.09604
O	-0.57052	-0.11176	-0.78778
C	3.01314	0.77521	-0.37614
C	4.19025	-0.09109	-0.13911
C	5.27839	0.41036	0.46017
H	6.13328	-0.22627	0.65739
C	5.31265	1.82937	0.87755
C	6.51147	2.36072	1.56651
H	7.37317	1.72472	1.72703
C	6.53457	3.62687	1.99606
H	7.41067	4.01336	2.50062
C	5.36084	4.49765	1.79563
H	5.37862	5.51497	2.16489
C	4.27383	4.02987	1.17115
H	3.41785	4.68071	1.05566
C	4.24223	2.63232	0.66023
C	3.04000	2.08329	-0.03741
C	0.64176	2.52003	0.23544
C	-0.58461	3.32930	0.01731
C	-0.52618	4.42588	-0.75424
H	-1.41451	5.02475	-0.91916
C	0.74600	4.83084	-1.38872
C	0.77644	6.01935	-2.27259
H	-0.11997	6.60786	-2.42393

C	1.90949	6.36963	-2.89017
H	1.92988	7.23722	-3.53699
C	3.13256	5.56696	-2.70225
H	4.04072	5.84357	-3.22220
C	3.11783	4.49443	-1.90242
H	4.02154	3.90956	-1.79836
C	1.87276	4.10789	-1.18343
C	1.82805	2.91354	-0.28694
C	-1.85959	2.91265	0.64345
C	-1.90320	2.64687	2.09945
C	-2.96468	2.72117	-0.09733
C	-3.02713	2.19349	2.68375
C	-4.21885	2.23171	0.52851
C	-4.23511	1.97554	1.85113
C	4.11427	-1.51877	-0.49247
C	4.00936	-1.89385	-1.77597
C	3.99772	-2.52900	0.58034
C	3.79483	-3.32003	-2.13115
C	3.76656	-3.82131	0.28137
C	3.67707	-4.22809	-1.14429
H	-0.33973	-2.46240	1.25880
C	-1.78163	-2.68918	2.71227
C	-2.14892	-2.62633	0.31885
C	-0.85865	-2.70261	3.77478
C	-3.17657	-2.67977	2.96680
C	-3.56026	-2.55432	0.56224
C	-1.32965	-2.73682	5.07496
H	0.20288	-2.66184	3.55695
C	-3.62237	-2.71991	4.30589
C	-4.04755	-2.59494	1.83667
H	-4.22227	-2.46992	-0.28810
C	-2.71324	-2.75446	5.34650
H	-0.62136	-2.74385	5.89751
H	-4.69064	-2.71531	4.50258
H	-5.12070	-2.56703	2.00563
H	-3.06107	-2.78318	6.37385
C	-1.56900	-2.53297	-0.98437
C	-2.46355	-2.57153	-2.21640
H	-3.19607	-3.38946	-2.16106
H	-1.83360	-2.77337	-3.09394
N	-3.17642	-1.30291	-2.32676
N	-1.34018	-2.67424	1.40938
C	-4.10315	-1.04974	-3.33737
C	-4.43742	0.28789	-3.63065
C	-4.75662	-2.07193	-4.04895
C	-5.40865	0.58836	-4.57700
H	-3.92311	1.08683	-3.10237
C	-5.73051	-1.75560	-4.99740
H	-4.50785	-3.11174	-3.86829
C	-6.06998	-0.43123	-5.26866
H	-5.64751	1.62886	-4.77955
H	-6.22320	-2.56246	-5.53331
H	-6.82724	-0.19518	-6.00940
H	-2.59267	-0.50540	-2.10355
H	-0.99478	-1.15833	-0.88230
Cl	-0.06705	-3.51561	-1.20256
H	-2.93528	2.89240	-1.16702
H	-1.01289	2.82378	2.68975
H	4.05643	-2.20356	1.61147
H	4.05987	-1.14718	-2.55914

H	3.50858	-5.27109	-1.38326
H	-5.13817	1.61005	2.32307
C	-5.44926	2.01675	-0.33229
H	-5.20708	2.28650	-1.38320
C	-6.60222	2.92806	0.10485
H	-7.45529	2.82457	-0.59928
H	-6.95447	2.66873	1.12573
H	-6.27230	3.98897	0.09701
C	-5.87830	0.54443	-0.33063
H	-6.24549	0.23299	0.67019
H	-6.68937	0.38360	-1.07250
H	-5.01982	-0.09966	-0.60984
C	-3.08117	1.90048	4.17082
H	-4.08850	1.50246	4.42536
C	-2.87905	3.17649	4.99618
H	-3.63073	3.94085	4.70432
H	-3.01071	2.95456	6.07672
H	-1.86208	3.59613	4.84310
C	-2.06212	0.82399	4.56097
H	-1.02093	1.19621	4.45717
H	-2.22277	0.50994	5.61431
H	-2.18835	-0.06277	3.90820
C	3.69512	-3.74415	-3.58439
H	3.53326	-4.84388	-3.64814
C	2.49912	-3.07436	-4.27147
H	2.62279	-1.97146	-4.31112
H	2.39583	-3.45465	-5.31013
H	1.56302	-3.31085	-3.72339
C	4.99809	-3.43847	-4.33155
H	5.18668	-2.34486	-4.37803
H	5.85385	-3.92925	-3.82034
H	4.94258	-3.83141	-5.36921
C	3.56705	-4.86009	1.36833
H	3.39061	-5.85122	0.89480
C	4.81751	-4.99808	2.24520
H	5.01195	-4.06599	2.81699
H	4.68494	-5.83250	2.96652
H	5.70269	-5.22337	1.61263
C	2.32865	-4.54193	2.21625
H	2.14061	-5.36395	2.93929
H	2.46455	-3.59742	2.78426
H	1.43469	-4.44291	1.56390

Catalyst (3,5-(i-Pr)2, 7)-Enamine protonation TS (pro-S) alternative conformation
Electronic energy (Hartrees): -1870.553065
Free Energy at 1M concentration and 253 K (Hartrees): -1869.507869
Coordinates:

P	0.70271	0.02292	0.12949
O	1.91826	0.32370	-0.92388
O	0.53500	1.39301	1.01189
O	0.95432	-1.11152	1.06572
O	-0.53337	-0.08235	-0.81682
C	3.01418	0.90211	-0.33424
C	4.21390	0.07423	-0.07404
C	5.27799	0.61278	0.53573
H	6.14757	0.00292	0.75235
C	5.26138	2.03357	0.94757
C	6.43450	2.60576	1.64799
H	7.31603	1.99981	1.81683

C	6.40982	3.87174	2.07778
H	7.26719	4.28806	2.59071
C	5.20847	4.70126	1.86681
H	5.18726	5.71816	2.23706
C	4.14423	4.19643	1.23206
H	3.26720	4.81722	1.11008
C	4.16594	2.79905	0.71976
C	2.99068	2.20929	0.00883
C	0.57672	2.55208	0.27319
C	-0.67703	3.32207	0.06686
C	-0.65615	4.42974	-0.69104
H	-1.56369	5.00168	-0.84654
C	0.60091	4.88488	-1.32196
C	0.59004	6.08336	-2.19274
H	-0.32618	6.64224	-2.33762
C	1.71015	6.47921	-2.80647
H	1.70038	7.35402	-3.44374
C	2.96032	5.71724	-2.62726
H	3.85825	6.03076	-3.14400
C	2.98285	4.63619	-1.83917
H	3.90620	4.08168	-1.74087
C	1.75210	4.19924	-1.12462
C	1.74905	2.99441	-0.24157
C	-1.93591	2.85700	0.69315
C	-1.95870	2.55028	2.14184
C	-3.04411	2.65939	-0.04164
C	-3.06381	2.04969	2.72331
C	-4.27986	2.12309	0.58272
C	-4.27512	1.82678	1.89702
C	4.17805	-1.36119	-0.39909
C	4.13047	-1.76496	-1.67701
C	4.02452	-2.34822	0.69109
C	3.93734	-3.19974	-2.00858
C	3.80920	-3.64615	0.41371
C	3.77615	-4.08519	-1.00744
H	-0.25535	-2.48880	1.16926
C	-1.67190	-2.79935	2.62927
C	-2.07065	-2.65715	0.24416
C	-0.73387	-2.83819	3.67799
C	-3.06334	-2.81896	2.90123
C	-3.47982	-2.61701	0.50696
C	-1.18728	-2.92107	4.98225
H	0.32464	-2.78105	3.44674
C	-3.49057	-2.90976	4.24389
C	-3.94985	-2.70872	1.78532
H	-4.15411	-2.51519	-0.33175
C	-2.56694	-2.96516	5.27075
H	-0.46837	-2.94736	5.79514
H	-4.55602	-2.92691	4.45474
H	-5.02105	-2.70306	1.96838
H	-2.90053	-3.03197	6.30106
C	-1.50723	-2.51006	-1.06112
C	-2.41480	-2.52396	-2.28399
H	-3.13518	-3.35349	-2.24553
H	-1.79220	-2.69021	-3.17411
N	-3.14649	-1.26273	-2.34822
N	-1.24812	-2.73046	1.32262
C	-4.08544	-0.99217	-3.34305
C	-4.43399	0.34935	-3.59930
C	-4.73794	-2.00185	-4.07312

C	-5.41824	0.66500	-4.52712
H	-3.92060	1.13932	-3.05682
C	-5.72467	-1.67040	-5.00290
H	-4.47891	-3.04370	-3.92083
C	-6.07840	-0.34266	-5.23705
H	-5.66811	1.70812	-4.70080
H	-6.21645	-2.46785	-5.55352
H	-6.84576	-0.09480	-5.96339
H	-2.57080	-0.46444	-2.10747
H	-0.94712	-1.12983	-0.92671
Cl	0.00861	-3.45992	-1.32632
H	-3.03046	2.86097	-1.10628
H	-1.06810	2.73494	2.72928
H	4.04075	-1.99941	1.71694
H	4.20669	-1.03489	-2.47361
H	3.61765	-5.13230	-1.23164
H	-5.16436	1.42714	2.36750
C	-5.51614	1.90925	-0.27000
H	-5.29171	2.21366	-1.31533
C	-6.68048	2.78567	0.20653
H	-7.54020	2.68577	-0.48999
H	-7.01492	2.49105	1.22382
H	-6.37064	3.85251	0.22485
C	-5.91807	0.42975	-0.30606
H	-6.26909	0.08325	0.68901
H	-6.73376	0.27500	-1.04415
H	-5.05108	-0.19014	-0.61199
C	-3.09393	1.71110	4.20141
H	-4.08584	1.27429	4.45287
C	-2.92353	2.96810	5.06228
H	-3.70216	3.71626	4.80063
H	-3.03644	2.71046	6.13701
H	-1.92208	3.42440	4.91162
C	-2.03785	0.65618	4.55084
H	-1.00995	1.06493	4.45219
H	-2.18038	0.30460	5.59489
H	-2.13978	-0.21372	3.87168
C	3.90915	-3.65809	-3.45465
H	3.75065	-4.75921	-3.50037
C	2.74931	-3.00621	-4.21675
H	2.87540	-1.90461	-4.27783
H	2.69769	-3.41226	-5.24950
H	1.78705	-3.22968	-3.71057
C	5.24780	-3.36864	-4.14279
H	5.43913	-2.27619	-4.20383
H	6.07674	-3.84759	-3.57887
H	5.24374	-3.78451	-5.17296
C	3.57503	-4.63995	1.53546
H	3.65433	-4.12582	2.52000
C	2.16243	-5.22953	1.45202
H	2.02677	-5.81600	0.51862
H	1.97629	-5.89706	2.32021
H	1.40873	-4.41406	1.47580
C	4.63614	-5.74595	1.52188
H	4.50595	-6.40738	2.40493
H	4.55943	-6.36619	0.60396
H	5.65217	-5.29897	1.57031

Catalyst (3,5-(i-Pr)2, 7)-Enamine protonation TS (pro-S) alternative conformation

Electronic energy (Hartrees): -1870.552638
Free Energy at 1M concentration and 253 K (Hartrees): -1869.50736
Coordinates:

P	0.68788	0.05723	0.12834
O	1.87862	0.29947	-0.96830
O	0.51501	1.48194	0.91795
O	0.97318	-1.00502	1.13736
O	-0.56332	-0.13041	-0.78425
C	2.98392	0.91679	-0.43904
C	4.20075	0.11508	-0.17718
C	5.27194	0.69079	0.38461
H	6.15622	0.10195	0.59990
C	5.24757	2.12759	0.73648
C	6.42826	2.74197	1.38662
H	7.31859	2.15223	1.56626
C	6.39960	4.02645	1.75739
H	7.26253	4.47369	2.23367
C	5.18683	4.83507	1.53065
H	5.16326	5.86821	1.85264
C	4.11567	4.29197	0.94071
H	3.23078	4.89883	0.80521
C	4.14087	2.87220	0.49481
C	2.95756	2.23976	-0.16351
C	0.54480	2.59063	0.10536
C	-0.71504	3.33771	-0.14251
C	-0.70550	4.39788	-0.96542
H	-1.61848	4.95151	-1.15237
C	0.54560	4.82361	-1.62746
C	0.52261	5.96903	-2.56673
H	-0.39790	6.51288	-2.73903
C	1.63699	6.33519	-3.20858
H	1.61838	7.17117	-3.89581
C	2.89277	5.59249	-2.99213
H	3.78596	5.88052	-3.53145
C	2.92623	4.55947	-2.14241
H	3.85355	4.01714	-2.01789
C	1.70201	4.15753	-1.39650
C	1.71103	3.00651	-0.44390
C	-1.96908	2.89962	0.51077
C	-1.99553	2.70153	1.97793
C	-3.06924	2.63030	-0.21299
C	-3.09843	2.23360	2.59024
C	-4.30040	2.12269	0.44324
C	-4.30067	1.92934	1.77658
C	4.18558	-1.32880	-0.46497
C	4.12008	-1.76673	-1.73128
C	4.08826	-2.29181	0.65241
C	3.96799	-3.21304	-2.02208
C	3.91077	-3.60446	0.41102
C	3.86036	-4.07931	-0.99504
H	-0.23962	-2.36638	1.36054
C	-1.66655	-2.58619	2.83030
C	-2.04312	-2.65040	0.43838
C	-0.74101	-2.51139	3.88802
C	-3.05993	-2.62461	3.09074
C	-3.45553	-2.62647	0.68491
C	-1.20656	-2.50665	5.19062
H	0.31704	-2.43436	3.66361
C	-3.49995	-2.62260	4.43233
C	-3.93701	-2.62866	1.96217

H	-4.12271	-2.60841	-0.16534
C	-2.58736	-2.57107	5.46918
H	-0.49637	-2.44584	6.00930
H	-4.56691	-2.65460	4.63358
H	-5.00989	-2.63802	2.13490
H	-2.93084	-2.56784	6.49840
C	-1.47127	-2.59352	-0.87047
C	-2.36778	-2.72223	-2.09484
H	-3.06685	-3.56529	-1.99943
H	-1.73364	-2.93768	-2.96610
N	-3.13085	-1.48889	-2.25672
N	-1.22990	-2.61238	1.52591
C	-4.07288	-1.31756	-3.27044
C	-4.45913	-0.00836	-3.62232
C	-4.69212	-2.39515	-3.92890
C	-5.44705	0.21157	-4.57359
H	-3.97179	0.83284	-3.13571
C	-5.68327	-2.15962	-4.88296
H	-4.40364	-3.41563	-3.70272
C	-6.07402	-0.86281	-5.21221
H	-5.72607	1.23196	-4.82189
H	-6.14869	-3.00826	-5.37707
H	-6.84430	-0.68962	-5.95688
H	-2.57762	-0.65999	-2.07389
H	-0.94844	-1.19460	-0.83038
Cl	0.06784	-3.52488	-1.05226
H	-3.05167	2.75213	-1.28969
H	-1.11018	2.94078	2.55353
H	4.11887	-1.91702	1.66789
H	4.15308	-1.05667	-2.54954
H	3.73241	-5.13682	-1.18732
H	-5.18659	1.55188	2.27102
C	-5.52541	1.81963	-0.39857
H	-5.29897	2.04913	-1.46246
C	-6.71139	2.70457	0.00301
H	-7.56286	2.53503	-0.69016
H	-7.04861	2.47960	1.03702
H	-6.42352	3.77605	-0.05620
C	-5.89602	0.33336	-0.32549
H	-6.24518	0.05480	0.69135
H	-6.70409	0.10643	-1.05322
H	-5.01431	-0.28888	-0.58079
C	-3.13465	2.01064	4.08994
H	-4.12513	1.58799	4.36967
C	-2.97688	3.33144	4.85210
H	-3.75784	4.05246	4.52857
H	-3.09558	3.15690	5.94280
H	-1.97696	3.78074	4.67307
C	-2.07416	0.99289	4.52452
H	-2.21732	0.72475	5.59295
H	-2.17103	0.07140	3.91638
H	-1.04813	1.39779	4.39521
C	3.91709	-3.68056	-3.46459
H	4.04904	-2.80218	-4.13464
C	5.05952	-4.65509	-3.77695
H	4.94652	-5.60237	-3.20837
H	5.06783	-4.89499	-4.86164
H	6.03586	-4.19309	-3.51655
C	2.55481	-4.29836	-3.80150
H	2.36827	-5.21287	-3.19949

H	1.74608	-3.56352	-3.60199
H	2.51575	-4.56979	-4.87800
C	3.73786	-4.59994	1.54214
H	3.60432	-5.61747	1.11256
C	4.98277	-4.65022	2.43610
H	5.13581	-3.68616	2.96596
H	4.87358	-5.45518	3.19390
H	5.88271	-4.87052	1.82289
C	2.47919	-4.29160	2.36316
H	2.31504	-5.08617	3.12183
H	2.57287	-3.31750	2.88797
H	1.58926	-4.25770	1.69901

Catalyst (3,5-(i-Pr)2, 7)-Enamine protonation TS (pro-S) alternative conformation
Electronic energy (Hartrees): -1870.552608
Free Energy at 1M concentration and 253 K (Hartrees): -1869.507357
Coordinates:

P	0.70570	0.10173	0.11221
O	1.89904	0.43022	-0.95827
O	0.47537	1.48685	0.95580
O	1.01848	-0.99161	1.07848
O	-0.53005	-0.08982	-0.82102
C	2.96556	1.08739	-0.39773
C	4.21367	0.33771	-0.12863
C	5.25396	0.95533	0.44611
H	6.15941	0.40272	0.66932
C	5.16215	2.38549	0.81271
C	6.30990	3.04532	1.47718
H	7.22666	2.49591	1.65175
C	6.21879	4.32105	1.86734
H	7.05772	4.80147	2.35406
C	4.96941	5.07405	1.64854
H	4.89510	6.09946	1.98709
C	3.92743	4.48967	1.04594
H	3.01484	5.05557	0.91791
C	4.02203	3.08001	0.57729
C	2.87328	2.40237	-0.09790
C	0.44694	2.62286	0.18167
C	-0.84856	3.31780	-0.03406
C	-0.89434	4.40099	-0.82562
H	-1.83263	4.91875	-0.98826
C	0.33010	4.90226	-1.48496
C	0.24632	6.07052	-2.39211
H	-0.69984	6.57529	-2.54307
C	1.33728	6.50559	-3.03124
H	1.27427	7.35819	-3.69503
C	2.62801	5.81677	-2.84385
H	3.50249	6.16084	-3.38097
C	2.71631	4.76375	-2.02311
H	3.66879	4.26206	-1.91920
C	1.51815	4.28513	-1.28038
C	1.58852	3.11044	-0.36024
C	-2.07511	2.80556	0.61944
C	-2.06720	2.53760	2.07602
C	-3.17962	2.53223	-0.09653
C	-3.13930	1.99778	2.68364
C	-4.37998	1.95163	0.55651
C	-4.34634	1.69163	1.87803
C	4.25559	-1.10745	-0.40424

C	4.26252	-1.55701	-1.66784
C	4.12536	-2.06262	0.71711
C	4.15479	-3.00868	-1.95143
C	3.98432	-3.37885	0.48179
C	4.00856	-3.86730	-0.92284
H	-0.12204	-2.42171	1.21536
C	-1.50767	-2.77303	2.69558
C	-1.93305	-2.71087	0.31164
C	-0.56024	-2.73739	3.73597
C	-2.89384	-2.85915	2.98107
C	-3.34018	-2.73870	0.58674
C	-0.99804	-2.81214	5.04606
H	0.49183	-2.63049	3.49330
C	-3.30474	-2.93932	4.32949
C	-3.79415	-2.82314	1.87136
H	-4.02573	-2.69277	-0.24771
C	-2.37108	-2.92115	5.34858
H	-0.27212	-2.78118	5.85253
H	-4.36604	-3.00724	4.55075
H	-4.86261	-2.86907	2.06451
H	-2.69224	-2.98016	6.38331
C	-1.38922	-2.56575	-1.00203
C	-2.30576	-2.65340	-2.21503
H	-2.98349	-3.51641	-2.15003
H	-1.68402	-2.80960	-3.10753
N	-3.09972	-1.43173	-2.30030
N	-1.09895	-2.71319	1.38364
C	-4.06363	-1.23179	-3.28795
C	-4.47697	0.08491	-3.57475
C	-4.67949	-2.28967	-3.98068
C	-5.48783	0.32990	-4.49532
H	-3.99285	0.91209	-3.06161
C	-5.69346	-2.02909	-4.90368
H	-4.37129	-3.31422	-3.80447
C	-6.11107	-0.72575	-5.16783
H	-5.78774	1.35544	-4.69289
H	-6.15589	-2.86294	-5.42502
H	-6.89919	-0.53299	-5.88862
H	-2.56121	-0.60048	-2.08679
H	-0.89519	-1.15689	-0.90209
Cl	0.17167	-3.44164	-1.26152
H	-3.18764	2.70539	-1.16619
H	-1.18124	2.78322	2.64792
H	4.10009	-1.67819	1.72997
H	4.31771	-0.85305	-2.49016
H	3.90772	-4.92741	-1.11291
H	-5.20921	1.26071	2.36952
C	-5.61304	1.65350	-0.27533
H	-5.41552	1.94082	-1.33087
C	-6.81486	2.48293	0.19219
H	-7.67584	2.32170	-0.49115
H	-7.12320	2.19904	1.22072
H	-6.55892	3.56404	0.17909
C	-5.94033	0.15533	-0.26801
H	-6.26222	-0.18181	0.74004
H	-6.75542	-0.05956	-0.99156
H	-5.04673	-0.42821	-0.56870
C	-3.13677	1.69846	4.17050
H	-4.10303	1.22026	4.44545
C	-3.01975	2.98520	4.99562

H	-3.83704	3.68686	4.72329
H	-3.10867	2.75155	6.07805
H	-2.04367	3.48585	4.82125
C	-2.02602	0.70674	4.53502
H	-2.13894	0.37791	5.59005
H	-2.09294	-0.18577	3.88134
H	-1.02073	1.16238	4.41184
C	4.19279	-3.49108	-3.38942
H	4.34584	-2.61695	-4.06054
C	5.36873	-4.44680	-3.62688
H	5.24148	-5.39048	-3.05529
H	5.44238	-4.69705	-4.70676
H	6.32025	-3.96389	-3.31722
C	2.86337	-4.13819	-3.79501
H	2.65818	-5.04773	-3.19150
H	2.03219	-3.41526	-3.65332
H	2.89116	-4.42473	-4.86795
C	3.78078	-4.34559	1.63272
H	3.80806	-3.79451	2.59993
C	2.40659	-5.01855	1.54050
H	2.32538	-5.64206	0.62493
H	2.23961	-5.66695	2.42689
H	1.60733	-4.24789	1.52156
C	4.90315	-5.38842	1.67992
H	4.79026	-6.02676	2.58212
H	4.88340	-6.04190	0.78224
H	5.89068	-4.88222	1.73472

Catalyst (3,5-(i-Pr)2, 7)-Enamine protonation TS (pro-S) alternative conformation
Electronic energy (Hartrees): -1870.550767
Free Energy at 1M concentration and 253 K (Hartrees): -1869.506182
Coordinates:

P	0.62173	-0.01183	0.18352
O	1.79613	0.04352	-0.95609
O	0.59709	1.47602	0.87082
O	0.85189	-1.01215	1.26877
O	-0.66472	-0.16348	-0.68176
C	2.96314	0.60638	-0.50562
C	4.11674	-0.27109	-0.20385
C	5.25000	0.25714	0.27683
H	6.08832	-0.38464	0.52253
C	5.35674	1.71567	0.49994
C	6.60612	2.28053	1.06042
H	7.45066	1.63464	1.26624
C	6.69640	3.58964	1.31760
H	7.60944	4.00079	1.72848
C	5.54703	4.47614	1.05492
H	5.61982	5.53112	1.28582
C	4.41467	3.97786	0.54516
H	3.57857	4.64421	0.38259
C	4.30712	2.52828	0.22480
C	3.05343	1.94478	-0.34265
C	0.69024	2.51897	-0.01991
C	-0.50392	3.36771	-0.26081
C	-0.44264	4.35707	-1.16541
H	-1.30795	4.98405	-1.34750
C	0.80461	4.60959	-1.91775
C	0.83636	5.67696	-2.94455
H	-0.03975	6.28871	-3.12058

C	1.94547	5.88848	-3.66091
H	1.96678	6.66756	-4.41196
C	3.14010	5.05232	-3.43855
H	4.02758	5.21521	-4.03641
C	3.12430	4.08875	-2.51033
H	4.00533	3.47499	-2.38059
C	1.90766	3.85922	-1.68376
C	1.86194	2.78724	-0.64423
C	-1.74665	3.11709	0.50228
C	-1.69426	3.06055	1.98075
C	-2.91138	2.89070	-0.12742
C	-2.79495	2.77102	2.69850
C	-4.14073	2.57201	0.64660
C	-4.07231	2.51487	1.98918
C	3.96937	-1.72831	-0.35430
C	3.81291	-2.27151	-1.57050
C	3.83898	-2.57384	0.85104
C	3.52916	-3.72200	-1.71883
C	3.54557	-3.88312	0.74025
C	3.40116	-4.47883	-0.61271
H	-0.46551	-2.21939	1.61325
C	-1.91766	-2.17371	3.07711
C	-2.28214	-2.44947	0.70054
C	-0.99647	-2.09253	4.13846
C	-3.31061	-2.06270	3.31724
C	-3.68773	-2.27589	0.91759
C	-1.46960	-1.93296	5.42835
H	0.06580	-2.13782	3.92460
C	-3.75842	-1.89816	4.64652
C	-4.17621	-2.10069	2.18078
H	-4.34189	-2.27877	0.05622
C	-2.85281	-1.84152	5.68890
H	-0.76358	-1.86933	6.25037
H	-4.82512	-1.81396	4.83357
H	-5.24631	-1.99263	2.33619
H	-3.20218	-1.71586	6.70841
C	-1.69861	-2.57421	-0.60183
C	-2.59236	-2.84868	-1.80520
H	-3.34297	-3.61710	-1.57536
H	-1.96600	-3.25373	-2.61129
N	-3.27640	-1.62083	-2.21582
N	-1.47590	-2.36396	1.78827
C	-4.08908	-1.61204	-3.35620
C	-4.21662	-0.42780	-4.10774
C	-4.82181	-2.74104	-3.76550
C	-5.06913	-0.36728	-5.20453
H	-3.63198	0.44513	-3.82759
C	-5.66959	-2.66929	-4.87077
H	-4.73679	-3.67538	-3.22181
C	-5.80732	-1.48683	-5.59667
H	-5.14793	0.56097	-5.76380
H	-6.22601	-3.55503	-5.16530
H	-6.46832	-1.43955	-6.45620
H	-2.66058	-0.81644	-2.16772
H	-1.11551	-1.21961	-0.68408
Cl	-0.21354	-3.61099	-0.64060
H	-2.95019	2.90835	-1.20966
H	-0.75230	3.25554	2.47793
H	3.93930	-2.11173	1.82520
H	3.87309	-1.64304	-2.45073

H	3.18281	-5.53617	-0.70035
H	-4.95521	2.27398	2.56880
C	-5.44974	2.28906	-0.06539
H	-6.25462	2.09606	0.67934
C	-5.33134	1.03273	-0.93250
H	-4.57999	1.17926	-1.73389
H	-5.02500	0.16735	-0.30789
H	-6.30989	0.79507	-1.40141
C	-5.89245	3.49518	-0.90148
H	-5.17863	3.69510	-1.72851
H	-6.89508	3.30513	-1.34059
H	-5.96082	4.39898	-0.25889
C	-2.75094	2.68611	4.21193
H	-3.76113	2.41221	4.58930
C	-2.38868	4.03862	4.83654
H	-3.10004	4.81925	4.49156
H	-2.45316	3.97465	5.94374
H	-1.35742	4.34502	4.56040
C	-1.78717	1.58791	4.67403
H	-0.73357	1.85062	4.44072
H	-1.88038	1.43385	5.77017
H	-2.03719	0.63585	4.16376
C	3.37148	-4.33794	-3.09634
H	3.16227	-5.42750	-3.00275
C	2.18576	-3.71636	-3.84402
H	2.35348	-2.63593	-4.03839
H	2.03894	-4.23061	-4.81771
H	1.25577	-3.83368	-3.24910
C	4.66480	-4.19754	-3.90689
H	4.89730	-3.13048	-4.10946
H	5.51328	-4.65089	-3.35094
H	4.56427	-4.72734	-4.87819
C	3.33190	-4.75097	1.96571
H	3.10160	-5.78921	1.63903
C	4.59866	-4.82233	2.82701
H	4.84677	-3.82985	3.25938
H	4.45106	-5.54235	3.66004
H	5.45647	-5.17222	2.21367
C	2.13109	-4.26293	2.78682
H	1.93135	-4.96528	3.62388
H	2.31881	-3.25384	3.21087
H	1.22378	-4.21988	2.14723

Catalyst (3,5-(i-Pr)2, 7)-Enamine protonation TS (pro-S) alternative conformation
Electronic energy (Hartrees): -1870.550469
Free Energy at 1M concentration and 253 K (Hartrees): -1869.506028
Coordinates:

P	0.63906	0.02234	0.18566
O	1.82880	0.17283	-0.92903
O	0.54010	1.48031	0.92876
O	0.89321	-1.01085	1.23380
O	-0.62585	-0.14748	-0.70786
C	2.95736	0.78222	-0.44201
C	4.14875	-0.04326	-0.13950
C	5.24852	0.53040	0.36596
H	6.11297	-0.07472	0.61415
C	5.27975	1.98744	0.61959
C	6.49315	2.60171	1.20657
H	7.36750	1.99490	1.40676

C	6.51399	3.90727	1.49480
H	7.40081	4.35449	1.92500
C	5.32332	4.74001	1.24051
H	5.33970	5.79147	1.49700
C	4.22288	4.19698	0.70713
H	3.35536	4.82366	0.55197
C	4.19267	2.75183	0.35158
C	2.97638	2.11894	-0.24421
C	0.58450	2.55805	0.07634
C	-0.64962	3.35489	-0.14147
C	-0.63428	4.37468	-1.01396
H	-1.52800	4.96565	-1.17832
C	0.60091	4.71145	-1.75326
C	0.58264	5.81030	-2.74666
H	-0.32231	6.38292	-2.90786
C	1.68207	6.09866	-3.45108
H	1.66686	6.90066	-4.17778
C	2.91624	5.31648	-3.24890
H	3.79640	5.54173	-3.83728
C	2.94559	4.32516	-2.35071
H	3.85571	3.75226	-2.23531
C	1.73931	4.01011	-1.53735
C	1.74386	2.90554	-0.53176
C	-1.87943	3.02268	0.61255
C	-1.81887	2.90201	2.08709
C	-3.03845	2.78276	-0.02312
C	-2.90378	2.53642	2.79388
C	-4.25202	2.38540	0.73955
C	-4.17422	2.26619	2.07755
C	4.06877	-1.50397	-0.30406
C	3.96001	-2.04269	-1.52731
C	3.93953	-2.36241	0.89275
C	3.72206	-3.49943	-1.69206
C	3.68490	-3.67670	0.76789
C	3.58287	-4.26639	-0.59436
H	-0.37198	-2.29837	1.51046
C	-1.81939	-2.36756	2.97642
C	-2.18221	-2.53439	0.58964
C	-0.89726	-2.32013	4.03898
C	-3.21405	-2.29988	3.22294
C	-3.59093	-2.40478	0.81574
C	-1.37095	-2.23348	5.33558
H	0.16551	-2.33576	3.82120
C	-3.66223	-2.21051	4.55926
C	-4.08074	-2.30154	2.08650
H	-4.24614	-2.38224	-0.04455
C	-2.75535	-2.18447	5.60179
H	-0.66494	-2.19590	6.15922
H	-4.73005	-2.15919	4.75170
H	-5.15269	-2.22500	2.24786
H	-3.10490	-2.11613	6.62669
C	-1.59624	-2.58406	-0.71627
C	-2.48263	-2.82133	-1.93286
H	-3.21957	-3.61289	-1.73981
H	-1.84737	-3.17820	-2.75457
N	-3.18776	-1.58841	-2.29014
N	-1.37637	-2.48204	1.67954
C	-3.98230	-1.53906	-3.44216
C	-4.12357	-0.32113	-4.13511
C	-4.68229	-2.66144	-3.92083

C	-4.95810	-0.22351	-5.24294
H	-3.56456	0.54907	-3.79935
C	-5.51222	-2.55209	-5.03656
H	-4.58576	-3.61997	-3.42303
C	-5.66369	-1.33763	-5.70460
H	-5.04832	0.73001	-5.75606
H	-6.04356	-3.43348	-5.38542
H	-6.31048	-1.26114	-6.57279
H	-2.58989	-0.77492	-2.19308
H	-1.04750	-1.21541	-0.74255
Cl	-0.08502	-3.57916	-0.79918
H	-3.08355	2.84833	-1.10326
H	-0.88363	3.11235	2.59061
H	4.00568	-1.90473	1.87278
H	4.01929	-1.40563	-2.40141
H	3.39159	-5.32696	-0.69622
H	-5.04520	1.96787	2.64844
C	-5.55602	2.09680	0.02052
H	-6.34889	1.83861	0.75838
C	-5.40352	0.89250	-0.91241
H	-4.66701	1.10729	-1.71207
H	-5.06063	0.00703	-0.33705
H	-6.37841	0.64550	-1.38412
C	-6.04357	3.33003	-0.74836
H	-5.34156	3.59730	-1.56646
H	-7.04194	3.13012	-1.19278
H	-6.13806	4.19569	-0.05833
C	-2.84930	2.38488	4.30183
H	-3.84426	2.04797	4.66854
C	-2.54677	3.72322	4.98586
H	-3.29568	4.48453	4.67927
H	-2.60174	3.60638	6.08926
H	-1.53251	4.08963	4.71988
C	-1.83388	1.31272	4.71224
H	-0.79416	1.63583	4.49290
H	-1.91827	1.10284	5.79980
H	-2.03852	0.37494	4.15741
C	3.62543	-4.11131	-3.07717
H	3.43972	-5.20610	-2.99587
C	2.45183	-3.51469	-3.86335
H	2.60001	-2.42979	-4.04814
H	2.35139	-4.02792	-4.84346
H	1.50486	-3.65701	-3.30215
C	4.94214	-3.93523	-3.84178
H	5.15627	-2.86151	-4.02912
H	5.78144	-4.37271	-3.25974
H	4.88759	-4.46023	-4.81935
C	3.47545	-4.53580	2.00035
H	3.61569	-3.92178	2.91864
C	2.04394	-5.08244	2.04316
H	1.84561	-5.75521	1.18192
H	1.88151	-5.65223	2.98284
H	1.31631	-4.24321	2.01836
C	4.50249	-5.67203	2.06179
H	4.39655	-6.22767	3.01797
H	4.36327	-6.38657	1.22326
H	5.53214	-5.25725	2.01414

Catalyst (3,5-(i-Pr)2, 7)-Enamine protonation TS (pro-S) alternative conformation

Electronic energy (Hartrees): -1870.550422
Free Energy at 1M concentration and 253 K (Hartrees): -1869.505791
Coordinates:

P	0.64984	0.10304	0.23160
O	1.86080	0.17328	-0.86844
O	0.55430	1.60007	0.88890
O	0.87014	-0.87096	1.34265
O	-0.60254	-0.10072	-0.67377
C	2.98881	0.80104	-0.40381
C	4.17876	-0.01298	-0.06819
C	5.28012	0.57859	0.41243
H	6.14502	-0.01723	0.68080
C	5.31476	2.04472	0.60530
C	6.52777	2.67997	1.17030
H	7.39859	2.07934	1.40203
C	6.55259	3.99712	1.39957
H	7.43910	4.45976	1.81379
C	5.36683	4.82234	1.10142
H	5.38646	5.88431	1.30987
C	4.26689	4.26015	0.58720
H	3.40282	4.88248	0.39835
C	4.23241	2.80052	0.29751
C	3.01647	2.14651	-0.27555
C	0.62465	2.62611	-0.02271
C	-0.59885	3.41422	-0.31330
C	-0.55945	4.38306	-1.24072
H	-1.44771	4.96434	-1.46014
C	0.69391	4.67276	-1.96934
C	0.70430	5.71575	-3.02130
H	-0.19282	6.28441	-3.23241
C	1.82011	5.95823	-3.71715
H	1.82586	6.71941	-4.48661
C	3.04381	5.18019	-3.44711
H	3.93736	5.36700	-4.02872
C	3.04730	4.23917	-2.49591
H	3.95033	3.66713	-2.33155
C	1.82245	3.97691	-1.69150
C	1.79792	2.92890	-0.62682
C	-1.84805	3.12159	0.42248
C	-1.83795	3.12051	1.90325
C	-2.97566	2.80047	-0.23258
C	-2.94006	2.79192	2.59874
C	-4.20652	2.43219	0.51593
C	-4.17750	2.42807	1.86090
C	4.10618	-1.47743	-0.19299
C	4.08132	-2.05469	-1.40331
C	3.91512	-2.30052	1.01974
C	3.88378	-3.51877	-1.53571
C	3.69739	-3.62536	0.92034
C	3.69436	-4.25700	-0.42371
H	-0.40355	-2.12279	1.58924
C	-1.93094	-2.22274	2.97268
C	-2.14361	-2.51653	0.57995
C	-1.07896	-2.04397	4.07903
C	-3.33777	-2.26975	3.14303
C	-3.56889	-2.49147	0.72596
C	-1.63061	-1.95318	5.34409
H	-0.00950	-1.96374	3.91763
C	-3.86656	-2.17531	4.44910
C	-4.13623	-2.38720	1.96376

H	-4.17532	-2.55278	-0.16759
C	-3.02593	-2.02859	5.53615
H	-0.97811	-1.81607	6.20068
H	-4.94385	-2.21470	4.58252
H	-5.21817	-2.39817	2.06503
H	-3.43714	-1.95647	6.53763
C	-1.48572	-2.57667	-0.69108
C	-2.29058	-2.91248	-1.94013
H	-2.97721	-3.75028	-1.75759
H	-1.59161	-3.24484	-2.71939
N	-3.06485	-1.74604	-2.36741
N	-1.40655	-2.35705	1.70800
C	-3.83528	-1.79244	-3.53552
C	-4.05670	-0.60941	-4.26733
C	-4.43648	-2.97905	-3.99360
C	-4.87253	-0.60899	-5.39320
H	-3.57363	0.31141	-3.94967
C	-5.24953	-2.96630	-5.12694
H	-4.27556	-3.91312	-3.46695
C	-5.48062	-1.78713	-5.83411
H	-5.02504	0.31963	-5.93638
H	-5.70349	-3.89618	-5.45882
H	-6.11333	-1.78608	-6.71592
H	-2.52572	-0.89096	-2.28663
H	-0.99149	-1.17268	-0.72570
Cl	0.08020	-3.48782	-0.66213
H	-2.97998	2.77513	-1.31539
H	-0.92371	3.38779	2.41988
H	3.91458	-1.81232	1.98631
H	4.18382	-1.44492	-2.29352
H	3.53366	-5.32463	-0.50110
H	-5.06099	2.14854	2.42064
C	-5.47006	2.03514	-0.22321
H	-6.28733	1.82677	0.50369
C	-5.24380	0.74955	-1.02329
H	-6.18621	0.43320	-1.51897
H	-4.46771	0.90247	-1.79938
H	-4.91582	-0.06620	-0.34535
C	-5.95678	3.17047	-1.13094
H	-5.23011	3.37386	-1.94580
H	-6.93149	2.89836	-1.58931
H	-6.09995	4.09810	-0.53643
C	-2.91187	2.77264	4.11482
H	-1.91045	3.09479	4.47988
C	-3.14348	1.35296	4.63945
H	-2.39134	0.66569	4.19854
H	-3.03062	1.32962	5.74429
H	-4.15944	0.98762	4.37878
C	-3.93712	3.75103	4.69872
H	-4.97472	3.44224	4.45122
H	-3.83643	3.79079	5.80438
H	-3.76010	4.77181	4.29732
C	3.88209	-4.14743	-2.91661
H	4.07932	-3.35697	-3.67437
C	4.99917	-5.18889	-3.05772
H	4.82292	-6.06002	-2.39180
H	5.04896	-5.55235	-4.10634
H	5.97946	-4.73268	-2.80201
C	2.51415	-4.75443	-3.25041
H	2.26362	-5.58593	-2.55804

H	1.72608	-3.97494	-3.17869
H	2.51564	-5.14789	-4.28928
C	3.43654	-4.47779	2.14760
H	3.28288	-5.53294	1.83017
C	4.63856	-4.46635	3.09973
H	4.80487	-3.45354	3.52414
H	4.46601	-5.17532	3.93743
H	5.55533	-4.78479	2.55882
C	2.15620	-4.03584	2.86824
H	1.93295	-4.72843	3.70748
H	2.26087	-3.00889	3.27754
H	1.29650	-4.05638	2.16536

Catalyst (3,5-(i-Pr)2, 7)-Enamine protonation TS (pro-S) alternative conformation
Electronic energy (Hartrees): -1870.550109
Free Energy at 1M concentration and 253 K (Hartrees): -1869.505633
Coordinates:

P	0.63207	0.05613	0.15995
O	1.79361	0.12624	-0.99210
O	0.55494	1.55933	0.80873
O	0.91024	-0.90574	1.26855
O	-0.65521	-0.16695	-0.68842
C	2.94019	0.75147	-0.57221
C	4.13663	-0.06594	-0.26874
C	5.25345	0.52389	0.17741
H	6.12342	-0.07387	0.42416
C	5.29906	1.99094	0.36171
C	6.53098	2.62451	0.88626
H	7.40645	2.02188	1.09401
C	6.56728	3.94261	1.10847
H	7.46764	4.40401	1.49314
C	5.37574	4.77066	0.84294
H	5.40539	5.83350	1.04566
C	4.25861	4.21002	0.36537
H	3.39131	4.83455	0.20017
C	4.21028	2.74921	0.08386
C	2.97488	2.09673	-0.44757
C	0.59359	2.58054	-0.11061
C	-0.63771	3.37238	-0.35864
C	-0.62826	4.33847	-1.29006
H	-1.52090	4.92420	-1.47752
C	0.59825	4.62144	-2.06522
C	0.57333	5.66080	-3.12056
H	-0.32941	6.23107	-3.30124
C	1.66394	5.89801	-3.85698
H	1.64386	6.65653	-4.62883
C	2.89470	5.11787	-3.62825
H	3.76730	5.30053	-4.24210
C	2.93005	4.18016	-2.67444
H	3.83716	3.60665	-2.54027
C	1.73415	3.92366	-1.82579
C	1.74545	2.87963	-0.75719
C	-1.86003	3.09196	0.42680
C	-1.78814	3.07740	1.90548
C	-3.02226	2.80327	-0.18173
C	-2.86835	2.76528	2.64463
C	-4.22900	2.45799	0.61604
C	-4.14281	2.44028	1.95868
C	4.05258	-1.53135	-0.37999

C	3.93696	-2.11479	-1.58226
C	3.94309	-2.34737	0.84770
C	3.71840	-3.57764	-1.69226
C	3.70596	-3.67042	0.77122
C	3.60143	-4.30854	-0.56563
H	-0.35687	-2.14824	1.65389
C	-1.79320	-2.12739	3.13387
C	-2.17263	-2.47825	0.76960
C	-0.86431	-1.97949	4.18124
C	-3.18679	-2.07069	3.38891
C	-3.58178	-2.35983	1.00023
C	-1.32899	-1.80851	5.47272
H	0.19629	-1.98319	3.95513
C	-3.62599	-1.89247	4.71929
C	-4.06295	-2.17439	2.26475
H	-4.24520	-2.41248	0.14772
C	-2.71176	-1.77078	5.74848
H	-0.61694	-1.69362	6.28390
H	-4.69313	-1.84978	4.91756
H	-5.13490	-2.10874	2.43085
H	-3.05449	-1.63500	6.76896
C	-1.59992	-2.61113	-0.53694
C	-2.49673	-2.95338	-1.72061
H	-3.21199	-3.74552	-1.46090
H	-1.86469	-3.35355	-2.52468
N	-3.23634	-1.76583	-2.15249
N	-1.35835	-2.33096	1.84466
C	-4.07365	-1.82125	-3.27346
C	-4.26065	-0.66620	-4.05729
C	-4.77501	-2.98726	-3.63083
C	-5.14022	-0.66925	-5.13423
H	-3.70153	0.23505	-3.81768
C	-5.65042	-2.97922	-4.71664
H	-4.64437	-3.90083	-3.06148
C	-5.84720	-1.82533	-5.47428
H	-5.26489	0.23801	-5.71901
H	-6.18173	-3.89251	-4.97061
H	-6.52951	-1.82780	-6.31831
H	-2.65179	-0.93714	-2.14031
H	-1.06941	-1.23681	-0.65974
Cl	-0.07365	-3.58671	-0.57081
H	-3.07455	2.79060	-1.26347
H	-0.84886	3.32238	2.38529
H	4.01486	-1.85462	1.80929
H	3.97875	-1.51030	-2.48097
H	3.42560	-5.37491	-0.62552
H	-5.00881	2.18118	2.55570
C	-5.53411	2.10521	-0.07152
H	-6.32199	1.90085	0.68821
C	-5.37646	0.83180	-0.90686
H	-4.64051	0.98601	-1.72103
H	-5.02944	-0.00408	-0.26364
H	-6.35020	0.54389	-1.35728
C	-6.03372	3.27037	-0.93324
H	-5.33805	3.47585	-1.77426
H	-7.03316	3.02966	-1.35441
H	-6.13009	4.18764	-0.31376
C	-2.80368	2.72401	4.15914
H	-3.79830	2.42277	4.55651
C	-2.48593	4.10594	4.74223

H	-3.23018	4.84946	4.38490
H	-2.53551	4.07021	5.85148
H	-1.47017	4.44312	4.44528
C	-1.79365	1.67606	4.63866
H	-0.75364	1.97118	4.38410
H	-1.86756	1.54956	5.73979
H	-2.01405	0.70138	4.15833
C	3.61244	-4.21277	-3.06611
H	3.76392	-3.42810	-3.84025
C	4.70559	-5.26709	-3.28027
H	4.56754	-6.13328	-2.59906
H	4.67730	-5.63574	-4.32787
H	5.70631	-4.82056	-3.09677
C	2.21790	-4.80616	-3.29894
H	2.00813	-5.63148	-2.58592
H	1.44538	-4.01753	-3.17594
H	2.14158	-5.20457	-4.33311
C	3.52204	-4.51375	2.01845
H	3.33621	-5.56886	1.71857
C	4.78686	-4.50996	2.88549
H	4.99234	-3.49674	3.29137
H	4.66520	-5.21262	3.73733
H	5.66089	-4.84092	2.28466
C	2.29881	-4.05452	2.82266
H	2.12539	-4.74168	3.67799
H	2.44280	-3.02745	3.21964
H	1.39305	-4.06711	2.17984

Catalyst (3,5-(i-Pr)2, 7)-Enamine protonation TS (pro-S) alternative conformation
Electronic energy (Hartrees): -1870.549975
Free Energy at 1M concentration and 253 K (Hartrees): -1869.505498
Coordinates:

P	0.64374	0.10077	0.15813
O	1.81014	0.31021	-0.97156
O	0.46421	1.55756	0.88775
O	0.97125	-0.90433	1.21321
O	-0.61890	-0.15263	-0.71879
C	2.90046	1.00366	-0.51033
C	4.15196	0.26795	-0.21972
C	5.21992	0.92473	0.25139
H	6.12880	0.38478	0.49105
C	5.15524	2.38436	0.48193
C	6.33476	3.09056	1.03352
H	7.25316	2.54908	1.22355
C	6.27058	4.39885	1.30225
H	7.13276	4.91327	1.70662
C	5.01979	5.14281	1.06189
H	4.96810	6.19679	1.30288
C	3.94910	4.51633	0.56040
H	3.03696	5.07877	0.41536
C	4.01234	3.06708	0.22632
C	2.83131	2.34209	-0.33472
C	0.42118	2.62436	0.02182
C	-0.86717	3.33427	-0.18319
C	-0.93498	4.34033	-1.06914
H	-1.86857	4.86880	-1.22435
C	0.26156	4.74704	-1.83629
C	0.15248	5.82759	-2.84384
H	-0.79114	6.33691	-2.99532

C	1.21736	6.17737	-3.57308
H	1.13586	6.96586	-4.31006
C	2.50402	5.48120	-3.38421
H	3.35641	5.75516	-3.99247
C	2.61529	4.50728	-2.47349
H	3.56318	3.99716	-2.36807
C	1.44763	4.12560	-1.63278
C	1.54367	3.03888	-0.61245
C	-2.05892	2.93230	0.59805
C	-1.96236	2.82871	2.07208
C	-3.21332	2.61582	-0.01221
C	-3.00862	2.40333	2.80329
C	-4.38516	2.15221	0.77782
C	-4.27393	2.04979	2.11481
C	4.17002	-1.19734	-0.35529
C	4.12959	-1.76703	-1.56879
C	4.06828	-2.03751	0.85721
C	3.99683	-3.23772	-1.70628
C	3.90530	-3.36820	0.75583
C	3.87550	-3.99056	-0.59482
H	-0.20636	-2.26477	1.50045
C	-1.61849	-2.42228	2.99267
C	-2.01377	-2.62307	0.61365
C	-0.68146	-2.30910	4.03711
C	-3.00974	-2.44462	3.26532
C	-3.42352	-2.58462	0.86590
C	-1.13535	-2.24695	5.34211
H	0.37564	-2.25566	3.79876
C	-3.43756	-2.37792	4.60961
C	-3.89538	-2.50806	2.14535
H	-4.09463	-2.60859	0.01811
C	-2.51480	-2.28722	5.63444
H	-0.41804	-2.15894	6.15203
H	-4.50262	-2.39554	4.82229
H	-4.96686	-2.50083	2.32656
H	-2.84879	-2.23652	6.66554
C	-1.44953	-2.64050	-0.70264
C	-2.34117	-2.93623	-1.90242
H	-3.02559	-3.76999	-1.69412
H	-1.70022	-3.25764	-2.73425
N	-3.12573	-1.74952	-2.25089
N	-1.19325	-2.51377	1.68817
C	-3.94267	-1.75275	-3.38817
C	-4.16774	-0.54876	-4.08334
C	-4.58430	-2.91656	-3.84928
C	-5.02698	-0.50559	-5.17562
H	-3.65499	0.35446	-3.76119
C	-5.43984	-2.86149	-4.94956
H	-4.42242	-3.86517	-3.34942
C	-5.67455	-1.66137	-5.61950
H	-5.18234	0.43845	-5.69067
H	-5.92504	-3.77419	-5.28488
H	-6.34081	-1.62729	-6.47558
H	-2.57584	-0.90135	-2.16796
H	-0.98104	-1.24103	-0.74199
Cl	0.12025	-3.53801	-0.81176
H	-3.28372	2.66870	-1.09168
H	-1.03238	3.10075	2.55532
H	4.08186	-1.55645	1.82814
H	4.16410	-1.14671	-2.45693

H	3.75574	-5.06243	-0.67760
H	-5.11371	1.70416	2.70542
C	-5.68335	1.77925	0.08764
H	-6.44444	1.48039	0.84338
C	-5.47702	0.57809	-0.83891
H	-6.44437	0.26875	-1.28885
H	-4.77082	0.82976	-1.65492
H	-5.07005	-0.28000	-0.26387
C	-6.25960	2.97401	-0.68063
H	-5.59142	3.27569	-1.51494
H	-7.25274	2.71047	-1.10302
H	-6.39235	3.83856	0.00448
C	-2.91519	2.26798	4.31087
H	-3.88080	1.87566	4.70022
C	-2.67935	3.62752	4.97917
H	-3.47782	4.34065	4.68201
H	-2.70601	3.51682	6.08425
H	-1.69392	4.05101	4.69067
C	-1.82984	1.26139	4.70800
H	-0.81591	1.64294	4.46330
H	-1.87855	1.05735	5.79885
H	-1.99069	0.30789	4.16568
C	3.98254	-3.85756	-3.09112
H	4.12458	-3.05488	-3.84840
C	5.13859	-4.84948	-3.27082
H	5.01808	-5.73171	-2.60705
H	5.17477	-5.20364	-4.32317
H	6.10533	-4.35355	-3.03851
C	2.63309	-4.52059	-3.39102
H	2.43497	-5.36245	-2.69417
H	1.81600	-3.77408	-3.29797
H	2.62409	-4.91248	-4.43045
C	3.73147	-4.21565	2.00177
H	3.80710	-3.57505	2.90943
C	2.34304	-4.86460	2.02758
H	2.21152	-5.56510	1.17571
H	2.20314	-5.42792	2.97472
H	1.55739	-4.08135	1.97325
C	4.83649	-5.27273	2.10733
H	4.75018	-5.81665	3.07215
H	4.76785	-6.01096	1.28059
H	5.83418	-4.78502	2.07237

Catalyst (3,5-(i-Pr)2, 7)-Enamine protonation TS (pro-S) alternative conformation
Electronic energy (Hartrees): -1870.548438
Free Energy at 1M concentration and 253 K (Hartrees): -1869.502239
Coordinates:

P	0.81054	-0.00513	-0.00181
O	1.99180	0.36372	-1.06820
O	0.51744	1.37495	0.83050
O	1.16419	-1.06934	0.98271
O	-0.40739	-0.26027	-0.94349
C	3.04141	1.03409	-0.49200
C	4.29456	0.29959	-0.20213
C	5.30986	0.92683	0.40652
H	6.21744	0.38555	0.64811
C	5.18668	2.35141	0.78615
C	6.30811	3.02167	1.48429
H	7.22955	2.48504	1.67340

C	6.18730	4.29100	1.88709
H	7.00702	4.77899	2.39841
C	4.93098	5.02631	1.64874
H	4.83221	6.04622	1.99752
C	3.91256	4.43248	1.01585
H	2.99386	4.98499	0.87381
C	4.04090	3.03035	0.53297
C	2.91859	2.34207	-0.17483
C	0.48063	2.51121	0.05833
C	-0.82532	3.17942	-0.18274
C	-0.87010	4.27643	-0.95705
H	-1.81246	4.78173	-1.13456
C	0.35841	4.81063	-1.58092
C	0.27204	5.99013	-2.47332
H	-0.67986	6.47988	-2.63714
C	1.36780	6.45387	-3.08338
H	1.30298	7.31457	-3.73644
C	2.66660	5.78555	-2.87872
H	3.54560	6.15267	-3.39272
C	2.75689	4.72272	-2.07096
H	3.71597	4.23668	-1.95439
C	1.55239	4.21240	-1.35990
C	1.62488	3.02833	-0.45270
C	-2.06597	2.63615	0.42522
C	-2.10010	2.31702	1.87260
C	-3.16506	2.42749	-0.31965
C	-3.22038	1.85548	2.46007
C	-4.41052	1.90958	0.29601
C	-4.43352	1.65484	1.61802
C	4.36055	-1.14499	-0.48355
C	4.28450	-1.59500	-1.74526
C	4.33076	-2.10411	0.64155
C	4.18129	-3.04818	-2.02298
C	4.19661	-3.42399	0.41281
C	4.13197	-3.91055	-0.98837
H	-0.06695	-2.34042	1.33479
C	-1.42038	-2.44361	2.88285
C	-1.91874	-2.65505	0.52059
C	-0.44228	-2.27402	3.88062
C	-2.79611	-2.49214	3.21891
C	-3.31722	-2.60263	0.83807
C	-0.83790	-2.19532	5.20366
H	0.59944	-2.18627	3.59189
C	-3.16177	-2.43515	4.58132
C	-3.73071	-2.55377	2.13814
H	-4.02795	-2.61585	0.02355
C	-2.19823	-2.29002	5.56135
H	-0.08776	-2.05946	5.97649
H	-4.21431	-2.49046	4.84462
H	-4.79308	-2.55766	2.36713
H	-2.48720	-2.23819	6.60592
C	-1.41216	-2.69450	-0.81417
C	-2.36968	-2.87056	-1.98421
H	-3.08743	-3.68373	-1.80295
H	-1.78553	-3.15926	-2.86899
N	-3.10577	-1.62615	-2.19065
N	-1.05181	-2.55820	1.56274
C	-4.05849	-1.48470	-3.19984
C	-4.41162	-0.18734	-3.62286
C	-4.72053	-2.57977	-3.78322

C	-5.41008	0.00635	-4.56890
H	-3.89343	0.66623	-3.19254
C	-5.72100	-2.37076	-4.73369
H	-4.45940	-3.59324	-3.49957
C	-6.07951	-1.08419	-5.13248
H	-5.66410	1.01842	-4.87203
H	-6.22027	-3.23233	-5.16884
H	-6.85801	-0.93171	-5.87312
H	-2.52402	-0.80549	-2.06968
H	-0.83000	-1.29571	-0.89138
Cl	0.09789	-3.66856	-1.01371
H	-3.14702	2.63729	-1.38262
H	-1.20668	2.47614	2.46419
H	4.36641	-1.72143	1.65393
H	4.26331	-0.89023	-2.56847
H	4.03706	-4.97329	-1.17078
H	-5.34982	1.30076	2.06628
C	-5.63666	1.68941	-0.57058
H	-5.37958	1.92170	-1.62705
C	-6.77498	2.63556	-0.17136
H	-7.62787	2.52075	-0.87394
H	-7.13357	2.42045	0.85763
H	-6.42668	3.68963	-0.21717
C	-6.09640	0.22585	-0.52833
H	-6.51472	-0.03667	0.46637
H	-6.87854	0.05147	-1.29771
H	-5.24226	-0.44924	-0.74060
C	-3.20226	1.55285	3.95548
H	-2.31639	0.91037	4.16157
C	-4.43221	0.77637	4.44978
H	-4.59513	-0.12854	3.82932
H	-4.27590	0.44732	5.49943
H	-5.34378	1.41091	4.41520
C	-3.05328	2.84788	4.76643
H	-3.90260	3.53450	4.56060
H	-3.03083	2.62070	5.85383
H	-2.10519	3.36597	4.51113
C	4.10133	-3.52696	-3.46027
H	4.18145	-2.64916	-4.13923
C	5.26597	-4.46406	-3.80342
H	5.20440	-5.41024	-3.22520
H	5.24720	-4.71188	-4.88620
H	6.23367	-3.96677	-3.57819
C	2.75067	-4.19313	-3.74885
H	2.61912	-5.11410	-3.14214
H	1.92360	-3.48826	-3.51794
H	2.68254	-4.46527	-4.82372
C	4.07601	-4.41555	1.55404
H	3.97333	-5.44075	1.13432
C	5.33403	-4.41375	2.43090
H	5.45937	-3.44070	2.95148
H	5.26430	-5.21608	3.19610
H	6.23287	-4.60632	1.80684
C	2.81787	-4.14553	2.38978
H	2.69035	-4.94151	3.15398
H	2.88494	-3.16624	2.90912
H	1.91877	-4.14434	1.73680

Catalyst (3,5-(i-Pr)2, 7)-Enamine protonation TS (pro-S) alternative conformation

Electronic energy (Hartrees): -1870.54877
Free Energy at 1M concentration and 253 K (Hartrees): -1869.502195
Coordinates:

P	0.79865	-0.07330	0.03009
O	2.00852	0.41521	-0.95136
O	0.30012	1.26110	0.84269
O	1.18537	-1.10527	1.03592
O	-0.31376	-0.43981	-1.00260
C	2.93032	1.22342	-0.33285
C	4.28001	0.68448	-0.01926
C	5.17227	1.46373	0.61137
H	6.15799	1.08146	0.85061
C	4.82379	2.84756	0.99591
C	5.81131	3.67446	1.72794
H	6.79555	3.27955	1.94729
C	5.49163	4.91102	2.12379
H	6.21352	5.51356	2.65983
C	4.14912	5.45344	1.84262
H	3.88971	6.44710	2.18460
C	3.25095	4.71694	1.17845
H	2.26611	5.12840	1.00389
C	3.60058	3.34954	0.70466
C	2.61741	2.50604	-0.03917
C	0.16857	2.38418	0.06217
C	-1.19120	2.91071	-0.23336
C	-1.31992	3.98994	-1.02438
H	-2.30090	4.39944	-1.23608
C	-0.13213	4.64393	-1.61035
C	-0.30727	5.79778	-2.52309
H	-1.29873	6.17865	-2.73459
C	0.75726	6.37271	-3.09244
H	0.62790	7.21450	-3.76039
C	2.11091	5.85331	-2.82174
H	2.96663	6.30971	-3.30243
C	2.28060	4.81507	-1.99499
H	3.28093	4.43868	-1.82947
C	1.10831	4.18241	-1.32977
C	1.26880	3.02271	-0.40293
C	-2.39305	2.27127	0.36057
C	-2.43358	1.98318	1.81432
C	-3.46867	1.98906	-0.39454
C	-3.54444	1.49549	2.39949
C	-4.69815	1.42914	0.21560
C	-4.73154	1.21098	1.54380
C	4.62058	-0.71598	-0.35787
C	4.41431	-1.18914	-1.59781
C	5.15486	-1.62035	0.68743
C	4.70078	-2.60835	-1.92695
C	5.41652	-2.91058	0.41210
C	5.17589	-3.41908	-0.96426
H	-0.03552	-2.38551	1.41808
C	-1.39811	-2.49103	2.95899
C	-1.87229	-2.77319	0.59862
C	-0.43263	-2.27046	3.95940
C	-2.77354	-2.58299	3.28776
C	-3.27344	-2.75606	0.90612
C	-0.83917	-2.18464	5.27866
H	0.60802	-2.15462	3.67509
C	-3.14869	-2.52122	4.64746
C	-3.69777	-2.69625	2.20237

H	-3.97746	-2.81550	0.08837
C	-2.19721	-2.32505	5.63025
H	-0.09941	-2.00994	6.05370
H	-4.19902	-2.61785	4.90728
H	-4.76100	-2.73469	2.42396
H	-2.49408	-2.27004	6.67243
C	-1.35173	-2.85108	-0.72961
C	-2.29799	-3.08567	-1.89838
H	-3.00074	-3.90611	-1.69355
H	-1.70278	-3.39306	-2.76942
N	-3.05740	-1.86420	-2.15143
N	-1.01716	-2.61925	1.64393
C	-4.02238	-1.78444	-3.15600
C	-4.39845	-0.51473	-3.63829
C	-4.67853	-2.91527	-3.67457
C	-5.41345	-0.38079	-4.57725
H	-3.88628	0.36613	-3.25921
C	-5.69550	-2.76651	-4.61863
H	-4.40048	-3.91005	-3.34490
C	-6.07709	-1.50604	-5.07520
H	-5.68511	0.61171	-4.92623
H	-6.18970	-3.65494	-5.00255
H	-6.86864	-1.40044	-5.81014
H	-2.48694	-1.03037	-2.07367
H	-0.75492	-1.45736	-0.87857
Cl	0.16287	-3.82863	-0.87560
H	-3.44929	2.18633	-1.45965
H	-1.55888	2.20120	2.41459
H	5.30988	-1.23168	1.68688
H	4.03528	-0.53118	-2.36985
H	5.38497	-4.45781	-1.18621
H	-5.63915	0.82973	1.98717
C	-5.90396	1.14503	-0.66131
H	-5.64199	1.36494	-1.71909
C	-7.08150	2.05671	-0.29688
H	-7.91927	1.89752	-1.00895
H	-7.44734	1.84852	0.73097
H	-6.77090	3.12169	-0.35867
C	-6.31100	-0.33275	-0.59470
H	-6.72175	-0.59319	0.40366
H	-7.08436	-0.54823	-1.36254
H	-5.43322	-0.97971	-0.79518
C	-3.54564	1.25846	3.90752
H	-2.65389	0.63940	4.15457
C	-4.77417	0.48967	4.41800
H	-4.91203	-0.44966	3.84491
H	-5.69379	1.10742	4.33092
H	-4.63498	0.21915	5.48661
C	-3.42846	2.58952	4.66482
H	-4.28066	3.25640	4.41146
H	-3.42721	2.41004	5.76134
H	-2.48089	3.10814	4.40942
C	4.45062	-3.13840	-3.32631
H	4.72469	-4.21632	-3.37834
C	2.96535	-3.03538	-3.69174
H	2.63022	-1.97691	-3.72283
H	2.78593	-3.49208	-4.68838
H	2.35324	-3.58383	-2.94531
C	5.32106	-2.40499	-4.35301
H	5.04446	-1.33153	-4.42087

H	6.39226	-2.48298	-4.06849
H	5.19452	-2.86529	-5.35616
C	5.93571	-3.83615	1.49613
H	6.05330	-3.27421	2.45020
C	4.94200	-4.97198	1.76521
H	4.83308	-5.62932	0.87664
H	5.29265	-5.58927	2.61972
H	3.94646	-4.55261	2.02573
C	7.31755	-4.38901	1.12941
H	7.72130	-4.99113	1.97122
H	7.26579	-5.03503	0.22754
H	8.02180	-3.55251	0.93241

Catalyst (3,5-(i-Pr)2, 7)-Enamine protonation TS (pro-S) alternative conformation
Electronic energy (Hartrees): -1870.54877
Free Energy at 1M concentration and 253 K (Hartrees): -1869.502192
Coordinates:

P	0.79864	-0.07335	0.02992
O	2.00856	0.41557	-0.95126
O	0.29999	1.26070	0.84301
O	1.18528	-1.10573	1.03536
O	-0.31374	-0.43947	-1.00296
C	2.93023	1.22373	-0.33248
C	4.28005	0.68492	-0.01911
C	5.17222	1.46416	0.61165
H	6.15803	1.08202	0.85071
C	4.82352	2.84783	0.99663
C	5.81096	3.67468	1.72883
H	6.79529	3.27989	1.94797
C	5.49107	4.91107	2.12509
H	6.21290	5.51358	2.66125
C	4.14844	5.45332	1.84420
H	3.88886	6.44682	2.18652
C	3.25034	4.71685	1.17988
H	2.26541	5.12818	1.00554
C	3.60020	3.34966	0.70564
C	2.61713	2.50622	-0.03840
C	0.16830	2.38404	0.06289
C	-1.19153	2.91056	-0.23241
C	-1.32036	3.99004	-1.02307
H	-2.30139	4.39953	-1.23459
C	-0.13265	4.64432	-1.60887
C	-0.30793	5.79844	-2.52123
H	-1.29943	6.17931	-2.73258
C	0.75654	6.37365	-3.09044
H	0.62709	7.21565	-3.75811
C	2.11024	5.85428	-2.81995
H	2.96591	6.31091	-3.30051
C	2.28005	4.81578	-1.99355
H	3.28042	4.43943	-1.82818
C	1.10783	4.18281	-1.32849
C	1.26846	3.02282	-0.40203
C	-2.39331	2.27082	0.36135
C	-2.43374	1.98214	1.81499
C	-3.46899	1.98889	-0.39379
C	-3.54454	1.49417	2.40004
C	-4.69842	1.42871	0.21621
C	-4.73170	1.21000	1.54433
C	4.62087	-0.71540	-0.35810

C	4.41454	-1.18833	-1.59811
C	5.15560	-1.61985	0.68690
C	4.70142	-2.60737	-1.92764
C	5.41766	-2.90993	0.41121
C	5.17697	-3.41818	-0.96522
H	-0.03537	-2.38625	1.41700
C	-1.39793	-2.49285	2.95786
C	-1.87212	-2.77363	0.59733
C	-0.43244	-2.27301	3.95842
C	-2.77337	-2.58492	3.28656
C	-3.27326	-2.75664	0.90483
C	-0.83899	-2.18792	5.27773
H	0.60823	-2.15714	3.67420
C	-3.14855	-2.52376	4.64629
C	-3.69760	-2.69756	2.20111
H	-3.97729	-2.81560	0.08704
C	-2.19707	-2.32827	5.62920
H	-0.09921	-2.01383	6.05288
H	-4.19891	-2.62027	4.90602
H	-4.76083	-2.73614	2.42268
H	-2.49395	-2.27377	6.67141
C	-1.35152	-2.85090	-0.73093
C	-2.29785	-3.08488	-1.89979
H	-3.00068	-3.90534	-1.69531
H	-1.70270	-3.39192	-2.77099
N	-3.05714	-1.86320	-2.15220
N	-1.01699	-2.62024	1.64272
C	-4.02262	-1.78295	-3.15624
C	-4.39857	-0.51301	-3.63802
C	-4.67940	-2.91345	-3.67475
C	-5.41403	-0.37852	-4.57640
H	-3.88594	0.36760	-3.25900
C	-5.69685	-2.76414	-4.61820
H	-4.40144	-3.90840	-3.34553
C	-6.07831	-1.50343	-5.07426
H	-5.68555	0.61415	-4.92500
H	-6.19155	-3.65232	-5.00206
H	-6.87023	-1.39741	-5.80874
H	-2.48648	-1.02951	-2.07449
H	-0.75470	-1.45719	-0.87944
Cl	0.16295	-3.82857	-0.87735
H	-3.44970	2.18660	-1.45881
H	-1.55900	2.19994	2.41530
H	5.31066	-1.23137	1.68642
H	4.03522	-0.53030	-2.36996
H	5.38636	-4.45679	-1.18746
H	-5.63928	0.82857	1.98761
C	-5.90429	1.14495	-0.66072
H	-5.64240	1.36525	-1.71843
C	-7.08179	2.05652	-0.29586
H	-7.91961	1.89762	-1.00793
H	-7.44756	1.84794	0.73194
H	-6.77117	3.12152	-0.35727
C	-6.31136	-0.33285	-0.59463
H	-6.72212	-0.59363	0.40363
H	-7.08473	-0.54804	-1.36255
H	-5.43360	-0.97976	-0.79534
C	-3.54562	1.25649	3.90796
H	-2.65380	0.63740	4.15469
C	-4.77404	0.48736	4.41820

H	-5.69372	1.10509	4.33148
H	-4.63475	0.21637	5.48667
H	-4.91188	-0.45170	3.84468
C	-3.42850	2.58723	4.66582
H	-4.28078	3.25414	4.41283
H	-3.42713	2.40728	5.76227
H	-2.48100	3.10605	4.41057
C	4.45124	-3.13718	-3.32709
H	4.72569	-4.21498	-3.37944
C	2.96586	-3.03461	-3.69226
H	2.63032	-1.97625	-3.72298
H	2.78645	-3.49109	-4.68901
H	2.35409	-3.58353	-2.94590
C	5.32124	-2.40319	-4.35376
H	5.04421	-1.32982	-4.42134
H	6.39251	-2.48083	-4.06941
H	5.19473	-2.86332	-5.35700
C	5.93739	-3.83557	1.49492
H	6.05496	-3.27382	2.44911
C	4.94415	-4.97184	1.76392
H	4.83528	-5.62899	0.87521
H	5.29520	-5.58921	2.61821
H	3.94851	-4.55289	2.02475
C	7.31937	-4.38784	1.12781
H	7.72351	-4.99001	1.96941
H	7.26767	-5.03367	0.22580
H	8.02328	-3.55103	0.93088

Catalyst (3,5-(i-Pr)2, 7)-Enamine protonation TS (pro-S) alternative conformation
Electronic energy (Hartrees): -1870.545982
Free Energy at 1M concentration and 253 K (Hartrees): -1869.500922
Coordinates:

P	0.71048	0.04038	0.12428
O	1.88529	0.24114	-0.99207
O	0.44021	1.52476	0.76181
O	1.07369	-0.86808	1.25311
O	-0.51791	-0.34915	-0.75278
C	2.98036	0.89313	-0.48546
C	4.19168	0.11261	-0.13509
C	5.24070	0.73079	0.42541
H	6.12145	0.16392	0.70462
C	5.19677	2.18514	0.69215
C	6.35337	2.84481	1.34139
H	7.24247	2.27386	1.57866
C	6.30433	4.14721	1.64032
H	7.14960	4.62754	2.11606
C	5.09233	4.93086	1.33581
H	5.05092	5.97935	1.60148
C	4.04307	4.34664	0.74584
H	3.15728	4.93684	0.55474
C	4.09258	2.90531	0.37728
C	2.93451	2.22700	-0.28043
C	0.50409	2.57803	-0.11957
C	-0.73767	3.34139	-0.41983
C	-0.68125	4.38322	-1.26712
H	-1.57326	4.95835	-1.48720
C	0.58958	4.76044	-1.91785
C	0.60849	5.87346	-2.89566
H	-0.29653	6.43086	-3.10319

C	1.74175	6.19326	-3.52910
H	1.75385	7.00541	-4.24449
C	2.97639	5.43105	-3.26482
H	3.88515	5.68049	-3.79732
C	2.97138	4.42746	-2.37994
H	3.88385	3.86920	-2.22031
C	1.72526	4.07860	-1.64288
C	1.69305	2.96631	-0.64643
C	-2.02834	2.95257	0.20551
C	-2.10048	2.69539	1.66430
C	-3.14127	2.82763	-0.53729
C	-3.25888	2.34782	2.25643
C	-4.43339	2.44688	0.08654
C	-4.48112	2.22385	1.41210
C	4.19088	-1.35153	-0.32335
C	3.95159	-1.88016	-1.53365
C	4.31856	-2.24110	0.85233
C	3.80774	-3.34796	-1.70436
C	4.16488	-3.57105	0.72516
C	3.90408	-4.14316	-0.62301
H	-0.14695	-2.04218	1.80525
C	-1.56260	-1.92789	3.30150
C	-1.95610	-2.55743	0.99667
C	-0.63235	-1.55467	4.28996
C	-2.94863	-1.97560	3.59320
C	-3.36705	-2.50895	1.24995
C	-1.08520	-1.26947	5.56537
H	0.41799	-1.48055	4.02821
C	-3.37242	-1.70359	4.91218
C	-3.83539	-2.25744	2.50774
H	-4.04164	-2.68173	0.42239
C	-2.45549	-1.35621	5.88596
H	-0.37296	-0.97586	6.33017
H	-4.43285	-1.75333	5.14280
H	-4.90544	-2.26423	2.69754
H	-2.78880	-1.13886	6.89540
C	-1.39464	-2.80250	-0.29638
C	-2.29173	-3.29038	-1.42603
H	-2.95580	-4.09779	-1.08828
H	-1.65368	-3.71704	-2.21173
N	-3.10617	-2.18212	-1.92829
N	-1.13637	-2.24949	2.03423
C	-3.97548	-2.36588	-3.00995
C	-4.23441	-1.28635	-3.87568
C	-4.63944	-3.58307	-3.24722
C	-5.14973	-1.40782	-4.91507
H	-3.69982	-0.35154	-3.72773
C	-5.55064	-3.69496	-4.29739
H	-4.45235	-4.44208	-2.61230
C	-5.82048	-2.61351	-5.13551
H	-5.33076	-0.55572	-5.56450
H	-6.05274	-4.64503	-4.45906
H	-6.53083	-2.70960	-5.95043
H	-2.56563	-1.32747	-2.00404
H	-0.89403	-1.39972	-0.58889
Cl	0.16579	-3.72074	-0.25632
H	-3.09867	2.99542	-1.60626
H	-1.20343	2.80663	2.26113
H	4.49387	-1.79870	1.82584
H	3.83829	-1.22863	-2.39152

H	3.78718	-5.21427	-0.72728
H	-5.42607	1.95966	1.86490
C	-5.68713	2.31650	-0.75880
H	-6.54818	2.01649	-0.11988
C	-5.51843	1.22557	-1.82083
H	-6.47809	1.06238	-2.35610
H	-5.22068	0.26807	-1.34144
H	-4.74641	1.51323	-2.56459
C	-6.05345	3.65822	-1.40313
H	-6.17372	4.43609	-0.61895
H	-5.26995	3.98766	-2.11816
H	-7.01539	3.56651	-1.95131
C	-3.27555	2.10160	3.76189
H	-2.43326	1.41467	4.00415
C	-4.55577	1.41987	4.26774
H	-5.42844	2.10335	4.19147
H	-4.43501	1.13187	5.33394
H	-4.75963	0.49747	3.68465
C	-3.05508	3.41499	4.52493
H	-3.86083	4.14234	4.28649
H	-3.05266	3.22791	5.62017
H	-2.07630	3.86571	4.25751
C	3.54134	-3.94010	-3.07576
H	3.45655	-5.04772	-3.00054
C	2.21494	-3.42679	-3.64946
H	2.24616	-2.32973	-3.81882
H	2.00148	-3.92648	-4.61838
H	1.38254	-3.65833	-2.95272
C	4.70249	-3.64298	-4.03115
H	4.79921	-2.55230	-4.21770
H	5.65507	-4.01980	-3.60087
H	4.53531	-4.15388	-5.00339
C	4.22650	-4.46916	1.94582
H	4.44710	-3.86180	2.85264
C	2.87551	-5.15352	2.18362
H	2.61580	-5.83167	1.34318
H	2.91008	-5.74887	3.12087
H	2.07559	-4.38899	2.28827
C	5.35242	-5.50100	1.81441
H	5.44411	-6.08430	2.75545
H	5.15564	-6.20776	0.98080
H	6.31990	-4.98691	1.62952

Catalyst (3,5-(i-Pr)2, 7)-Enamine protonation TS (pro-S) alternative conformation
Electronic energy (Hartrees): -1870.546316
Free Energy at 1M concentration and 253 K (Hartrees): -1869.500744
Coordinates:

P	0.72436	-0.04057	0.04671
O	1.88678	0.16202	-1.08262
O	0.57833	1.40602	0.80032
O	1.04670	-1.05674	1.09309
O	-0.54948	-0.26947	-0.82159
C	3.02703	0.72162	-0.56478
C	4.19255	-0.14646	-0.27547
C	5.29063	0.37754	0.28548
H	6.13495	-0.25821	0.52637
C	5.34500	1.81811	0.61657
C	6.55605	2.37410	1.26351
H	7.41433	1.73979	1.44739

C	6.59447	3.66044	1.62640
H	7.47947	4.06496	2.10039
C	5.42494	4.52913	1.39586
H	5.45422	5.56333	1.71399
C	4.32699	4.03896	0.80884
H	3.47411	4.68986	0.67410
C	4.27886	2.61772	0.36848
C	3.06494	2.04322	-0.28866
C	0.66666	2.51215	-0.01125
C	-0.54086	3.35958	-0.20879
C	-0.45834	4.44904	-0.99189
H	-1.32346	5.08577	-1.13664
C	0.80521	4.79642	-1.67345
C	0.84906	5.96619	-2.58174
H	-0.03005	6.58427	-2.71576
C	1.97280	6.26275	-3.24297
H	2.00300	7.11653	-3.90756
C	3.17141	5.41907	-3.07891
H	4.07125	5.65176	-3.63366
C	3.14406	4.36344	-2.25724
H	4.02874	3.74727	-2.17024
C	1.91018	4.03754	-1.49008
C	1.85407	2.86819	-0.56304
C	-1.82553	3.00142	0.44798
C	-1.84966	2.63446	1.88499
C	-2.97546	2.99644	-0.24780
C	-2.99821	2.29758	2.50162
C	-4.25919	2.63830	0.40645
C	-4.26131	2.30931	1.71063
C	4.08178	-1.59887	-0.49938
C	3.88324	-2.08299	-1.73455
C	4.02594	-2.50992	0.66395
C	3.62093	-3.52968	-1.94603
C	3.75157	-3.81729	0.49780
C	3.55767	-4.34527	-0.87674
H	-0.21585	-2.21786	1.54535
C	-1.56094	-2.17965	3.10663
C	-2.08281	-2.54497	0.77053
C	-0.57031	-1.96566	4.08371
C	-2.93470	-2.17406	3.45441
C	-3.47694	-2.43947	1.09066
C	-0.95362	-1.78646	5.40039
H	0.47105	-1.92565	3.78227
C	-3.28828	-2.01190	4.81168
C	-3.87892	-2.29026	2.38722
H	-4.19246	-2.48444	0.28095
C	-2.31329	-1.82230	5.77244
H	-0.19460	-1.61612	6.15756
H	-4.33990	-2.01990	5.08417
H	-4.93907	-2.25197	2.62324
H	-2.59213	-1.68919	6.81256
C	-1.58775	-2.68829	-0.56455
C	-2.55606	-3.00939	-1.69527
H	-3.25344	-3.80831	-1.40918
H	-1.97551	-3.39003	-2.54636
N	-3.32236	-1.81282	-2.05195
N	-1.20612	-2.39397	1.79577
C	-4.23919	-1.84566	-3.11029
C	-4.43834	-0.68891	-3.88711
C	-5.00792	-2.98594	-3.40618

C	-5.39509	-0.66145	-4.89589
H	-3.82501	0.18790	-3.69554
C	-5.95899	-2.94925	-4.42563
H	-4.87175	-3.90026	-2.83906
C	-6.16780	-1.79141	-5.17463
H	-5.52807	0.24756	-5.47606
H	-6.54202	-3.84240	-4.63358
H	-6.91035	-1.77166	-5.96594
H	-2.72992	-0.99002	-2.07825
H	-1.00063	-1.29911	-0.73916
Cl	-0.08616	-3.69387	-0.68683
H	-2.97001	3.24354	-1.30208
H	-0.92367	2.65220	2.44665
H	4.15971	-2.09722	1.65611
H	3.88925	-1.40905	-2.58254
H	3.35419	-5.40082	-1.00995
H	-5.19898	2.06086	2.18695
C	-5.55484	2.64792	-0.38389
H	-6.40304	2.34538	0.27083
C	-5.49915	1.63991	-1.53587
H	-6.48987	1.57462	-2.03414
H	-4.74577	1.94454	-2.29174
H	-5.23736	0.63135	-1.14922
C	-5.86846	4.05628	-0.90127
H	-5.09894	4.39880	-1.62529
H	-6.85739	4.06376	-1.40743
H	-5.90736	4.77282	-0.05301
C	-2.96213	1.92522	3.98041
H	-2.14182	1.18621	4.12598
C	-4.24836	1.25452	4.48555
H	-4.52016	0.39825	3.83351
H	-4.09321	0.86629	5.51488
H	-5.09142	1.97770	4.51260
C	-2.65123	3.15897	4.83860
H	-3.43390	3.93566	4.69962
H	-2.60931	2.87947	5.91314
H	-1.66623	3.59104	4.56313
C	3.40727	-4.07580	-3.34519
H	3.22241	-5.17281	-3.29986
C	2.17527	-3.44013	-4.00067
H	2.31528	-2.34836	-4.14797
H	1.98800	-3.90779	-4.99077
H	1.27845	-3.60415	-3.36700
C	4.65656	-3.86660	-4.20822
H	4.85994	-2.78584	-4.36414
H	5.53918	-4.33135	-3.71880
H	4.51786	-4.34634	-5.20060
C	3.60504	-4.75004	1.68489
H	3.37797	-5.77442	1.31505
C	4.90870	-4.84148	2.48720
H	5.15895	-3.86773	2.95914
H	4.80887	-5.60537	3.28761
H	5.74491	-5.14392	1.82107
C	2.43259	-4.32674	2.58020
H	2.28024	-5.07510	3.38680
H	2.62147	-3.33765	3.04862
H	1.49807	-4.26703	1.98260

Catalyst (3,5-(i-Pr)2, 7)-Enamine protonation TS (pro-S) alternative conformation

Electronic energy (Hartrees): -1870.545421
Free Energy at 1M concentration and 253 K (Hartrees): -1869.50017
Coordinates:

P	0.74072	0.08361	0.05064
O	1.89840	0.34782	-1.07093
O	0.44846	1.53952	0.74246
O	1.13654	-0.85712	1.14126
O	-0.49123	-0.29607	-0.82573
C	2.97028	1.04051	-0.56773
C	4.21478	0.30770	-0.23478
C	5.24930	0.96383	0.30791
H	6.15135	0.42782	0.58011
C	5.15554	2.41548	0.57538
C	6.29824	3.11827	1.20361
H	7.21383	2.58214	1.42050
C	6.20398	4.41667	1.50877
H	7.03918	4.92853	1.96914
C	4.95576	5.15248	1.23287
H	4.87815	6.19742	1.50438
C	3.91843	4.52910	0.66220
H	3.00610	5.08446	0.49335
C	4.01730	3.09216	0.28584
C	2.87387	2.37041	-0.35214
C	0.43832	2.61324	-0.11605
C	-0.84334	3.32904	-0.36102
C	-0.85844	4.38355	-1.19453
H	-1.77946	4.92602	-1.37414
C	0.37303	4.82102	-1.88280
C	0.31331	5.94621	-2.84491
H	-0.62042	6.46782	-3.01485
C	1.41073	6.32120	-3.51047
H	1.36539	7.14219	-4.21434
C	2.68415	5.60825	-3.29724
H	3.56371	5.90262	-3.85520
C	2.75017	4.59417	-2.42690
H	3.68985	4.07288	-2.30393
C	1.54467	4.18403	-1.65504
C	1.59171	3.05860	-0.67461
C	-2.09328	2.88258	0.30799
C	-2.09572	2.59409	1.76288
C	-3.23165	2.73339	-0.39065
C	-3.21544	2.19375	2.39466
C	-4.48304	2.29655	0.27819
C	-4.46732	2.04531	1.59941
C	4.25419	-1.15678	-0.39665
C	4.11079	-1.71066	-1.61048
C	4.29037	-2.01889	0.80504
C	3.99133	-3.18109	-1.75964
C	4.14223	-3.35079	0.69684
C	3.99096	-3.95282	-0.65479
H	-0.03587	-2.09013	1.65382
C	-1.39454	-2.07970	3.20416
C	-1.86668	-2.59115	0.88504
C	-0.43109	-1.74983	4.17618
C	-2.76814	-2.15934	3.54334
C	-3.26783	-2.57124	1.19130
C	-0.83843	-1.53919	5.48099
H	0.60778	-1.64972	3.87948
C	-3.14465	-1.96341	4.88995
C	-3.69168	-2.39182	2.47699

H	-3.97054	-2.70495	0.38010
C	-2.19509	-1.65844	5.84647
H	-0.10090	-1.27945	6.23398
H	-4.19507	-2.03782	5.15695
H	-4.75401	-2.41906	2.70457
H	-2.49245	-1.49964	6.87781
C	-1.35031	-2.76585	-0.43795
C	-2.28192	-3.21547	-1.55571
H	-2.92443	-4.04494	-1.23083
H	-1.66733	-3.59967	-2.38085
N	-3.12629	-2.09664	-1.98230
N	-1.01270	-2.32721	1.90676
C	-4.02800	-2.25128	-3.04326
C	-4.28858	-1.15956	-3.89255
C	-4.72269	-3.45280	-3.27205
C	-5.23503	-1.25360	-4.90710
H	-3.73179	-0.23638	-3.75210
C	-5.66338	-3.53820	-4.29809
H	-4.53732	-4.31966	-2.64722
C	-5.93447	-2.44403	-5.11948
H	-5.41718	-0.39235	-5.54400
H	-6.18890	-4.47657	-4.45349
H	-6.66851	-2.51906	-5.91541
H	-2.59151	-1.23762	-2.05188
H	-0.86629	-1.35047	-0.68651
Cl	0.22240	-3.66179	-0.49843
H	-3.23986	2.92307	-1.45674
H	-1.17902	2.72478	2.32478
H	4.38774	-1.55354	1.77886
H	4.04919	-1.07761	-2.48816
H	3.87997	-5.02513	-0.74381
H	-5.38311	1.74058	2.08544
C	-5.76681	2.14148	-0.51632
H	-6.58962	1.79983	0.15141
C	-5.60801	1.07878	-1.60785
H	-6.58362	0.89443	-2.10610
H	-4.87830	1.40804	-2.37634
H	-5.25874	0.12225	-1.16262
C	-6.20377	3.48299	-1.11541
H	-5.46253	3.85301	-1.85523
H	-7.18462	3.37027	-1.62478
H	-6.31593	4.24025	-0.31015
C	-3.15968	1.91391	3.89335
H	-2.28636	1.24964	4.08353
C	-4.39460	1.17947	4.43664
H	-4.59671	0.26661	3.83847
H	-4.21727	0.86787	5.48823
H	-5.29021	1.83678	4.41681
C	-2.94871	3.21657	4.67728
H	-3.78624	3.92253	4.48946
H	-2.89444	3.00548	5.76679
H	-1.99671	3.70463	4.38043
C	3.85021	-3.77922	-3.14700
H	3.90796	-2.96283	-3.90078
C	4.99537	-4.75209	-3.45530
H	4.95050	-5.64679	-2.79880
H	4.93355	-5.08829	-4.51238
H	5.97403	-4.24681	-3.30889
C	2.48604	-4.45575	-3.32562
H	2.36677	-5.31076	-2.62676

H	1.67293	-3.72173	-3.14143
H	2.38072	-4.83177	-4.36565
C	4.10083	-4.21991	1.93921
H	4.26105	-3.59336	2.84569
C	2.72671	-4.88206	2.09090
H	2.51679	-5.56865	1.24333
H	2.68629	-5.46371	3.03649
H	1.93313	-4.10482	2.12926
C	5.21959	-5.26773	1.91717
H	5.23511	-5.82613	2.87751
H	5.07432	-5.99498	1.09063
H	6.20472	-4.76998	1.78962

Catalyst (3,5-(i-Pr)2, 7)-Enamine protonation TS (pro-R) lowest free energy conformation
Electronic energy (Hartrees): -1870.554584
Free Energy at 1M concentration and 253 K (Hartrees): -1869.507261
Coordinates:

P	-0.21895	0.14632	0.22379
O	1.25371	0.69432	-0.25284
O	-1.00241	1.39898	0.90693
O	-0.17896	-0.98889	1.18914
O	-0.90538	-0.12500	-1.15943
C	1.84461	1.65768	0.53589
C	3.20329	1.39975	1.09976
C	3.82242	2.36253	1.80488
H	4.80140	2.18205	2.23454
C	3.16799	3.65953	2.05215
C	3.83862	4.66900	2.90467
H	4.82043	4.46875	3.31547
C	3.23411	5.83108	3.17283
H	3.72408	6.56741	3.79673
C	1.89116	6.10752	2.62958
H	1.39748	7.04080	2.86805
C	1.27983	5.20387	1.85506
H	0.28578	5.41975	1.48701
C	1.95292	3.91735	1.52203
C	1.29853	2.89808	0.64730
C	-1.05317	2.50305	0.09404
C	-2.32934	2.86055	-0.58064
C	-2.36266	3.91512	-1.41195
H	-3.28627	4.19170	-1.90735
C	-1.14001	4.69829	-1.68336
C	-1.18994	5.82227	-2.64722
H	-2.12716	6.09608	-3.11566
C	-0.07800	6.50204	-2.94594
H	-0.11539	7.32192	-3.65156
C	1.20665	6.12648	-2.32642
H	2.10766	6.66433	-2.59194
C	1.26201	5.11616	-1.45097
H	2.22063	4.84310	-1.03156
C	0.02700	4.37096	-1.08090
C	0.05849	3.24193	-0.10171
C	-3.54073	2.03473	-0.36407
C	-3.96230	1.68847	1.01310
C	-4.24451	1.56181	-1.40691
C	-5.02860	0.89535	1.22848
C	-5.42916	0.69181	-1.19235
C	-5.78789	0.37409	0.06485
C	3.88014	0.09333	0.90737

C	5.14733	0.03620	0.45961
C	3.20624	-1.15428	1.32934
C	5.87618	-1.25729	0.39735
C	3.83629	-2.34015	1.25832
C	5.23419	-2.38500	0.75357
H	-1.03710	-2.48301	0.82871
C	-2.63311	-3.38672	1.73519
C	-2.30703	-3.12394	-0.66395
C	-2.10144	-3.23431	3.02968
C	-3.95071	-3.87172	1.55013
C	-3.65274	-3.59136	-0.85520
C	-2.87875	-3.57977	4.12180
H	-1.10284	-2.82745	3.15017
C	-4.71418	-4.21837	2.68456
C	-4.42948	-3.95358	0.20438
H	-4.01689	-3.67590	-1.86889
C	-4.18627	-4.07787	3.95519
H	-2.47387	-3.45915	5.12182
H	-5.72445	-4.59149	2.54219
H	-5.43597	-4.32549	0.03135
H	-4.77822	-4.34359	4.82480
C	-1.43272	-2.67762	-1.69663
H	-1.25220	-1.14589	-1.35273
C	0.02994	-3.11916	-1.68917
H	0.10081	-4.09989	-2.17504
H	0.34264	-3.25959	-0.64169
N	0.88864	-2.18657	-2.39404
N	-1.87870	-3.04838	0.63304
C	2.22171	-2.44574	-2.68639
C	2.73484	-3.74564	-2.85217
C	3.09719	-1.35615	-2.87402
C	4.07062	-3.93706	-3.20262
H	2.09502	-4.61001	-2.71203
C	4.42084	-1.56165	-3.23964
H	2.72146	-0.34750	-2.72241
C	4.92491	-2.85498	-3.40724
H	4.44204	-4.95152	-3.32148
H	5.06527	-0.69936	-3.38873
H	5.96050	-3.01553	-3.68938
H	0.68935	-1.21427	-2.20328
Cl	-2.12086	-2.74016	-3.36261
H	-6.64551	-0.26397	0.24038
H	5.75531	-3.33354	0.73177
H	5.65810	0.94727	0.17347
H	2.22202	-1.08947	1.76632
H	-3.40323	2.09072	1.84836
H	-3.93234	1.79346	-2.41768
C	7.35147	-1.29413	0.03773
H	7.72075	-2.34437	0.05787
C	3.16226	-3.59608	1.78123
H	2.12156	-3.36246	2.10227
C	3.90116	-4.13566	3.01084
H	3.34993	-5.00110	3.43677
H	4.92860	-4.46579	2.74796
H	3.96560	-3.34792	3.79178
C	3.06094	-4.66778	0.69223
H	2.51363	-5.55374	1.07889
H	2.49696	-4.26884	-0.17230
H	4.06527	-4.99402	0.34911
C	7.60001	-0.76155	-1.37674

H	7.06642	-1.39183	-2.11422
H	7.25908	0.28993	-1.48059
H	8.68401	-0.80604	-1.61578
C	8.17599	-0.51125	1.06643
H	7.92198	0.56990	1.04442
H	7.98363	-0.90474	2.08762
H	9.26010	-0.62361	0.85119
C	-5.46953	0.52774	2.63258
H	-6.35696	-0.14051	2.57061
C	-5.89614	1.76832	3.42632
H	-6.68891	2.31815	2.87528
H	-6.30599	1.46489	4.41330
H	-5.03695	2.45127	3.59643
C	-4.37470	-0.25024	3.37027
H	-3.48443	0.38616	3.55975
H	-4.75945	-0.62221	4.34370
H	-4.06609	-1.12328	2.76163
C	-6.22198	0.16720	-2.37452
H	-7.06764	-0.46163	-2.01508
C	-6.83108	1.32112	-3.17882
H	-6.04044	1.95140	-3.63849
H	-7.47776	0.92034	-3.98838
H	-7.45767	1.95673	-2.51708
C	-5.34976	-0.72253	-3.26627
H	-5.96465	-1.17020	-4.07588
H	-4.52300	-0.14359	-3.72950
H	-4.91623	-1.54721	-2.66460

Catalyst (3,5-(i-Pr)2, 7)-Enamine protonation TS (pro-R) alternative conformation
Electronic energy (Hartrees): -1870.55481
Free Energy at 1M concentration and 253 K (Hartrees): -1869.507225
Coordinates:

P	-0.26820	0.24150	0.31527
O	1.20169	0.80141	-0.16800
O	-1.06705	1.49403	0.97455
O	-0.21672	-0.87740	1.29851
O	-0.94404	-0.05759	-1.06715
C	1.78582	1.77290	0.61558
C	3.14979	1.53198	1.17683
C	3.74657	2.49709	1.89905
H	4.72713	2.33081	2.33057
C	3.07070	3.78160	2.15435
C	3.71974	4.79286	3.02141
H	4.69928	4.60140	3.44169
C	3.09851	5.94584	3.29083
H	3.57273	6.68346	3.92529
C	1.75884	6.21062	2.73380
H	1.25182	7.13666	2.97246
C	1.16703	5.30531	1.94614
H	0.17520	5.51243	1.56713
C	1.85871	4.02885	1.61281
C	1.22481	3.00690	0.72712
C	-1.11716	2.58971	0.15110
C	-2.38466	2.92159	-0.55033
C	-2.42071	3.96985	-1.38863
H	-3.33798	4.22230	-1.90816
C	-1.20668	4.77387	-1.63863
C	-1.25797	5.89453	-2.60621
H	-2.19111	6.15168	-3.09192

C	-0.15227	6.59187	-2.88699
H	-0.19067	7.40937	-3.59532
C	1.12725	6.23907	-2.24392
H	2.02376	6.79127	-2.49485
C	1.18395	5.23173	-1.36512
H	2.13947	4.97576	-0.92834
C	-0.04478	4.46675	-1.01508
C	-0.01180	3.34013	-0.03277
C	-3.57671	2.06281	-0.36501
C	-4.03790	1.72477	1.00155
C	-4.20951	1.53305	-1.42531
C	-5.06678	0.88023	1.19154
C	-5.35067	0.60086	-1.24026
C	-5.74468	0.28960	0.00776
C	3.86248	0.24711	0.95302
C	5.14408	0.23618	0.54117
C	3.20573	-1.03448	1.29603
C	5.90658	-1.03352	0.45479
C	3.86682	-2.20150	1.19600
C	5.28271	-2.19123	0.74505
H	-0.99674	-2.41282	0.90056
C	-2.56669	-3.44081	1.71759
C	-2.12177	-3.15796	-0.66209
C	-2.13189	-3.23443	3.04024
C	-3.81718	-4.05249	1.45891
C	-3.40903	-3.74370	-0.92657
C	-2.93411	-3.65590	4.08721
H	-1.18659	-2.73178	3.21697
C	-4.60665	-4.47608	2.54803
C	-4.20776	-4.17780	0.08767
H	-3.70616	-3.85859	-1.95908
C	-4.17154	-4.28463	3.84721
H	-2.60286	-3.49500	5.10847
H	-5.56493	-4.94734	2.34852
H	-5.16354	-4.64191	-0.14103
H	-4.78400	-4.60962	4.68175
C	-1.23234	-2.65086	-1.64878
H	-1.19241	-1.08969	-1.29419
C	0.26512	-2.94469	-1.55308
H	0.45718	-3.93285	-1.98448
H	0.53656	-3.00502	-0.48673
N	1.06827	-1.96634	-2.26300
N	-1.78109	-3.03789	0.65908
C	2.39043	-2.17799	-2.64350
C	2.88429	-3.43418	-3.04201
C	3.26441	-1.07339	-2.69480
C	4.19980	-3.56778	-3.48128
H	2.24044	-4.30683	-3.02404
C	4.56750	-1.21692	-3.15542
H	2.90416	-0.10293	-2.36336
C	5.05183	-2.46601	-3.55217
H	4.55617	-4.54922	-3.78276
H	5.21254	-0.34300	-3.19363
H	6.07139	-2.57912	-3.90669
H	0.87339	-1.00983	-2.00185
Cl	-1.81085	-2.78606	-3.34941
H	-6.56679	-0.39807	0.16006
H	5.82881	-3.12405	0.70658
H	5.64290	1.16774	0.30122
H	2.20649	-1.01390	1.69968

H	-3.53256	2.17086	1.84977
H	-3.86280	1.75631	-2.42675
C	7.39083	-0.99123	0.13479
H	7.69552	0.06560	-0.03308
C	3.20695	-3.49507	1.63884
H	2.13903	-3.30731	1.89374
C	3.88353	-4.03935	2.90153
H	3.34647	-4.94240	3.26250
H	4.94177	-4.31219	2.70203
H	3.85592	-3.27470	3.70722
C	3.22037	-4.54240	0.52073
H	2.73396	-4.13328	-0.38495
H	4.25604	-4.84952	0.26444
H	2.65354	-5.44368	0.83753
C	8.22475	-1.51151	1.31288
H	7.96602	-0.95390	2.23849
H	8.04654	-2.59445	1.48346
H	9.30644	-1.36077	1.10930
C	7.71852	-1.75826	-1.15048
H	7.41486	-2.82379	-1.07594
H	7.19805	-1.28876	-2.00832
H	8.80983	-1.71494	-1.35357
C	-5.52136	0.52957	2.59552
H	-4.91390	1.08967	3.34196
C	-5.30961	-0.96090	2.87528
H	-5.57255	-1.19454	3.92895
H	-5.93402	-1.58979	2.20578
H	-4.24201	-1.22015	2.71926
C	-6.98404	0.93015	2.81827
H	-7.66611	0.34118	2.16934
H	-7.26826	0.75446	3.87778
H	-7.12049	2.01039	2.59715
C	-6.05098	-0.00505	-2.44160
H	-6.87591	-0.67205	-2.10340
C	-6.68430	1.08539	-3.31285
H	-5.90931	1.74750	-3.75402
H	-7.26445	0.62216	-4.13931
H	-7.37968	1.70222	-2.70418
C	-5.08358	-0.86794	-3.25856
H	-5.62958	-1.38009	-4.07925
H	-4.27182	-0.25416	-3.70314
H	-4.63012	-1.64301	-2.60726

Catalyst (3,5-(i-Pr)2, 7)-Enamine protonation TS (pro-R) alternative conformation
Electronic energy (Hartrees): -1870.554283
Free Energy at 1M concentration and 253 K (Hartrees): -1869.507185
Coordinates:

P	-0.21010	0.17793	0.21391
O	1.20960	0.84840	-0.26311
O	-1.10520	1.36280	0.88124
O	-0.07684	-0.94140	1.18908
O	-0.86011	-0.16595	-1.17187
C	1.71022	1.86657	0.51907
C	3.08290	1.73257	1.09087
C	3.61443	2.75204	1.78727
H	4.60390	2.66072	2.22100
C	2.84926	3.99014	2.01899
C	3.42659	5.06243	2.86302
H	4.42040	4.95210	3.27893

C	2.72269	6.17045	3.11723
H	3.14461	6.95269	3.73496
C	1.36300	6.32410	2.56701
H	0.78948	7.21349	2.79425
C	0.83521	5.36315	1.80006
H	-0.17234	5.48841	1.42672
C	1.61838	4.13643	1.48252
C	1.05811	3.05560	0.61624
C	-1.24821	2.45201	0.05907
C	-2.54951	2.69499	-0.61863
C	-2.67056	3.73490	-1.46025
H	-3.61417	3.92924	-1.95703
C	-1.51813	4.61593	-1.73853
C	-1.66131	5.72206	-2.71363
H	-2.61722	5.91019	-3.18653
C	-0.61072	6.49146	-3.01698
H	-0.71610	7.29822	-3.73080
C	0.69959	6.23325	-2.39118
H	1.55204	6.84361	-2.66023
C	0.83865	5.24000	-1.50555
H	1.81588	5.05409	-1.08142
C	-0.32916	4.39551	-1.13056
C	-0.20389	3.28272	-0.14038
C	-3.69022	1.77740	-0.38830
C	-4.08115	1.41809	0.99452
C	-4.35891	1.23960	-1.42275
C	-5.08447	0.55017	1.22354
C	-5.47451	0.28609	-1.19323
C	-5.80484	-0.04204	0.06911
C	3.86891	0.48741	0.91339
C	5.13185	0.53232	0.45223
C	3.30557	-0.80759	1.35487
C	5.96278	-0.69516	0.38975
C	4.03066	-1.93782	1.28420
C	5.42040	-1.87109	0.76097
H	-0.83706	-2.49732	0.86044
C	-2.35577	-3.50554	1.79083
C	-2.06607	-3.25015	-0.61423
C	-1.82905	-3.29671	3.07945
C	-3.63357	-4.09210	1.62147
C	-3.37541	-3.81731	-0.78919
C	-2.57008	-3.68787	4.18145
H	-0.86382	-2.81304	3.18776
C	-4.36009	-4.48348	2.76551
C	-4.11474	-4.22510	0.28041
H	-3.73855	-3.94229	-1.79901
C	-3.83600	-4.28773	4.03054
H	-2.16899	-3.52434	5.17690
H	-5.33955	-4.93496	2.63503
H	-5.09119	-4.67454	0.11981
H	-4.39966	-4.58825	4.90757
C	-1.23024	-2.76259	-1.65881
H	-1.14180	-1.20536	-1.33989
C	0.25687	-3.11305	-1.65609
H	0.38371	-4.09210	-2.13377
H	0.58171	-3.22573	-0.60899
N	1.05632	-2.13951	-2.37406
N	-1.63679	-3.12447	0.67904
C	2.39460	-2.33126	-2.69197
C	2.97539	-3.60408	-2.84619

C	3.20507	-1.19912	-2.91719
C	4.31161	-3.72863	-3.22427
H	2.38874	-4.49965	-2.67362
C	4.52997	-1.33889	-3.30854
H	2.77777	-0.20964	-2.77524
C	5.10031	-2.60553	-3.46703
H	4.73572	-4.72333	-3.33342
H	5.12394	-0.44604	-3.48503
H	6.13724	-2.71348	-3.76873
H	0.80683	-1.17776	-2.18941
Cl	-1.92459	-2.88977	-3.31851
H	-6.61238	-0.73957	0.25562
H	6.01544	-2.77398	0.73330
H	5.56217	1.47894	0.14704
H	2.32305	-0.82083	1.80099
H	-3.55304	1.87324	1.82275
H	-4.06876	1.48272	-2.43738
C	7.42404	-0.58621	-0.00945
H	7.66012	0.47771	-0.23366
C	3.46786	-3.24272	1.81772
H	2.41635	-3.09327	2.15338
C	4.26565	-3.71907	3.03663
H	3.79289	-4.62617	3.47008
H	5.31287	-3.96451	2.75926
H	4.27670	-2.92790	3.81668
C	3.43858	-4.32001	0.72994
H	2.96416	-5.24487	1.12177
H	2.83830	-3.96530	-0.12954
H	4.46173	-4.56868	0.37737
C	8.34534	-1.01222	1.14110
H	8.10383	-0.43195	2.05740
H	8.23718	-2.09530	1.36207
H	9.40496	-0.81189	0.87402
C	7.72639	-1.38641	-1.28033
H	7.49346	-2.46388	-1.14597
H	7.12983	-0.98552	-2.12371
H	8.79997	-1.28716	-1.54813
C	-5.49620	0.17231	2.63380
H	-6.32882	-0.56397	2.58337
C	-6.01867	1.38897	3.40698
H	-6.85206	1.86558	2.84775
H	-6.40350	1.07124	4.39952
H	-5.21557	2.13990	3.56420
C	-4.34476	-0.50549	3.38401
H	-3.50770	0.20184	3.56418
H	-4.70072	-0.89160	4.36288
H	-3.96729	-1.36063	2.78916
C	-6.23170	-0.30801	-2.36588
H	-7.02623	-0.99463	-1.99569
C	-6.92930	0.78933	-3.17746
H	-6.19022	1.47241	-3.64739
H	-7.54822	0.33356	-3.97960
H	-7.59837	1.38278	-2.51815
C	-5.30018	-1.13882	-3.25442
H	-5.88401	-1.63820	-4.05674
H	-4.52075	-0.50458	-3.72717
H	-4.80414	-1.92359	-2.64785

Catalyst (3,5-(i-Pr)2, 7)-Enamine protonation TS (pro-R) alternative conformation

Electronic energy (Hartrees): -1870.55413
Free Energy at 1M concentration and 253 K (Hartrees): -1869.50688
Coordinates:

P	-0.20963	0.19195	0.21027
O	1.28675	0.67471	-0.26597
O	-0.94793	1.48594	0.86484
O	-0.21955	-0.92546	1.19681
O	-0.89665	-0.07886	-1.17238
C	1.91281	1.61961	0.51790
C	3.25617	1.31096	1.09336
C	3.90671	2.25226	1.79945
H	4.87446	2.03592	2.23790
C	3.30259	3.57578	2.03405
C	4.00533	4.56229	2.88757
H	4.97413	4.32484	3.30929
C	3.44596	5.74957	3.14311
H	3.95933	6.46914	3.76775
C	2.12079	6.07708	2.58478
H	1.66318	7.03108	2.81295
C	1.48088	5.19435	1.80921
H	0.50014	5.44817	1.42978
C	2.10434	3.87958	1.49031
C	1.41739	2.88228	0.61544
C	-0.94234	2.58045	0.03758
C	-2.19403	2.98230	-0.65815
C	-2.17482	4.03221	-1.49567
H	-3.07955	4.33998	-2.00718
C	-0.91873	4.76509	-1.75484
C	-0.91167	5.88435	-2.72545
H	-1.83138	6.19290	-3.20682
C	0.23030	6.51692	-3.01436
H	0.23448	7.33339	-3.72490
C	1.49125	6.09349	-2.37732
H	2.41639	6.59284	-2.63497
C	1.49531	5.08702	-1.49572
H	2.43713	4.77791	-1.06350
C	0.22685	4.39456	-1.13639
C	0.20084	3.27178	-0.14978
C	-3.43661	2.19897	-0.46085
C	-3.88889	1.86420	0.90962
C	-4.13544	1.74320	-1.51508
C	-4.96994	1.08693	1.10787
C	-5.33607	0.89482	-1.32008
C	-5.71549	0.57426	-0.06788
C	3.88535	-0.02069	0.90907
C	5.15483	-0.12554	0.47633
C	3.16104	-1.24223	1.32409
C	5.83723	-1.44466	0.42718
C	3.74857	-2.45041	1.26412
C	5.15077	-2.54714	0.77918
H	-1.10085	-2.41137	0.84278
C	-2.72458	-3.26674	1.74587
C	-2.37237	-3.04047	-0.65318
C	-2.20012	-3.11180	3.04297
C	-4.05324	-3.71842	1.55542
C	-3.72695	-3.47891	-0.85031
C	-2.99628	-3.42032	4.13256
H	-1.19193	-2.73055	3.16718
C	-4.83586	-4.02813	2.68742
C	-4.52227	-3.80634	0.20665

H	-4.08390	-3.56905	-1.86601
C	-4.31562	-3.88419	3.96082
H	-2.59696	-3.29704	5.13449
H	-5.85469	-4.37545	2.54087
H	-5.53650	-4.15433	0.02910
H	-4.92241	-4.12123	4.82848
C	-1.48045	-2.62502	-1.68367
H	-1.26566	-1.09368	-1.35596
C	-0.02847	-3.10070	-1.66155
H	0.02244	-4.08804	-2.13590
H	0.27336	-3.23709	-0.61023
N	0.85702	-2.19665	-2.37059
N	-1.95259	-2.96069	0.64637
C	2.18099	-2.49639	-2.66729
C	2.64925	-3.80994	-2.85678
C	3.09307	-1.43431	-2.83704
C	3.97710	-4.04071	-3.21316
H	1.98005	-4.65423	-2.73210
C	4.40843	-1.67839	-3.20934
H	2.75240	-0.41636	-2.66619
C	4.86759	-2.98492	-3.40082
H	4.31332	-5.06498	-3.35110
H	5.08205	-0.83625	-3.34403
H	5.89658	-3.17582	-3.68823
H	0.68992	-1.21864	-2.17821
Cl	-2.15902	-2.68718	-3.35355
H	-6.57969	-0.05728	0.09354
H	5.63806	-3.51366	0.76831
H	5.70216	0.76537	0.19435
H	2.17461	-1.14100	1.74836
H	-3.33863	2.26005	1.75375
H	-3.80090	1.96236	-2.52222
C	7.31462	-1.53588	0.08637
H	7.64580	-2.59854	0.11556
C	3.02303	-3.67945	1.78209
H	1.98383	-3.40943	2.07855
C	3.71810	-4.23125	3.03159
H	3.13205	-5.07661	3.45130
H	4.74087	-4.59348	2.79378
H	3.78886	-3.43972	3.80815
C	2.91033	-4.75773	0.70049
H	2.31986	-5.61843	1.08068
H	2.38562	-4.34753	-0.18314
H	3.91029	-5.12539	0.38797
C	7.59946	-1.01920	-1.32716
H	7.05340	-1.63406	-2.06857
H	7.29688	0.04304	-1.43995
H	8.68415	-1.10275	-1.55219
C	8.15393	-0.77823	1.12199
H	7.93954	0.31126	1.09196
H	7.93467	-1.15952	2.14239
H	9.23588	-0.93072	0.92106
C	-5.44210	0.73020	2.50455
H	-6.33503	0.07084	2.42798
C	-5.87127	1.97898	3.28378
H	-6.64842	2.53333	2.71529
H	-6.30211	1.68485	4.26461
H	-5.00875	2.65462	3.46593
C	-4.37028	-0.05529	3.26757
H	-3.47918	0.57408	3.47567

H	-4.77953	-0.42231	4.23286
H	-4.05540	-0.93166	2.66729
C	-6.09697	0.39307	-2.53288
H	-5.63295	0.81507	-3.45189
C	-6.00730	-1.13206	-2.64815
H	-6.47836	-1.63099	-1.77464
H	-6.51835	-1.47633	-3.57239
H	-4.94230	-1.43792	-2.70859
C	-7.55677	0.86364	-2.51517
H	-8.05699	0.58355	-3.46674
H	-8.11806	0.40282	-1.67502
H	-7.59915	1.96923	-2.41352

Catalyst (3,5-(i-Pr)2, 7)-Enamine protonation TS (pro-R) alternative conformation
Electronic energy (Hartrees): -1870.554279
Free Energy at 1M concentration and 253 K (Hartrees): -1869.506737
Coordinates:

P	-0.12273	0.13137	0.13308
O	1.31121	0.77052	-0.33799
O	-0.98204	1.32191	0.83992
O	-0.01589	-1.01632	1.07701
O	-0.79562	-0.15376	-1.25660
C	1.83781	1.76960	0.45174
C	3.21070	1.60114	1.01278
C	3.76709	2.60038	1.71912
H	4.75777	2.48359	2.14411
C	3.03043	3.85121	1.97056
C	3.63591	4.90054	2.82370
H	4.62957	4.76380	3.23205
C	2.95772	6.02030	3.09546
H	3.40010	6.78577	3.71985
C	1.59843	6.20963	2.55570
H	1.04538	7.10812	2.79747
C	1.04554	5.26957	1.78059
H	0.03841	5.42101	1.41610
C	1.80030	4.03015	1.44315
C	1.21183	2.97129	0.56801
C	-1.11562	2.43002	0.04156
C	-2.42823	2.72717	-0.59271
C	-2.53691	3.78226	-1.41774
H	-3.48823	4.02039	-1.87942
C	-1.36345	4.62749	-1.71826
C	-1.49530	5.75012	-2.67597
H	-2.45573	5.97336	-3.12376
C	-0.42889	6.49128	-2.99435
H	-0.52604	7.31019	-3.69539
C	0.88692	6.18596	-2.40200
H	1.75096	6.77457	-2.68233
C	1.01591	5.17740	-1.53228
H	1.99593	4.95726	-1.13129
C	-0.16799	4.36309	-1.14203
C	-0.05357	3.23422	-0.16970
C	-3.59992	1.85915	-0.32380
C	-3.93960	1.48956	1.07005
C	-4.35355	1.38707	-1.33197
C	-4.97592	0.67198	1.33376
C	-5.51221	0.49717	-1.06302
C	-5.79009	0.15164	0.20736
C	3.97579	0.34693	0.81388

C	5.22758	0.38349	0.32243
C	3.42397	-0.93916	1.29649
C	6.06327	-0.84096	0.27533
C	4.16354	-2.06481	1.25716
C	5.53809	-2.00652	0.69956
H	-0.87851	-2.52078	0.70900
C	-2.39398	-3.46618	1.70559
C	-2.24570	-3.14465	-0.70364
C	-1.77028	-3.33964	2.96123
C	-3.71757	-3.96017	1.60353
C	-3.60058	-3.60953	-0.80839
C	-2.46216	-3.72260	4.09722
H	-0.76950	-2.92433	3.01858
C	-4.39188	-4.34635	2.78113
C	-4.29486	-4.00695	0.29547
H	-4.03525	-3.67476	-1.79523
C	-3.77307	-4.23302	4.01279
H	-1.98658	-3.62335	5.06803
H	-5.40630	-4.72718	2.70334
H	-5.30870	-4.38326	0.18706
H	-4.29662	-4.52905	4.91583
C	-1.43402	-2.69118	-1.78396
H	-1.17898	-1.16017	-1.42278
C	-0.00188	-3.20518	-1.89104
H	-0.01083	-4.13900	-2.47013
H	0.34278	-3.47025	-0.87978
N	0.89023	-2.26258	-2.53832
N	-1.72645	-3.09083	0.56077
C	2.23724	-2.53082	-2.73319
C	2.76326	-3.83630	-2.73561
C	3.11880	-1.45594	-2.97183
C	4.11892	-4.04878	-2.98667
H	2.11881	-4.68675	-2.54233
C	4.46323	-1.68419	-3.22976
H	2.73081	-0.44080	-2.94771
C	4.98026	-2.98394	-3.24236
H	4.50079	-5.06633	-2.98252
H	5.11204	-0.83410	-3.42199
H	6.03112	-3.16213	-3.44616
H	0.67451	-1.29008	-2.36857
Cl	-2.24529	-2.67069	-3.39613
H	-6.62918	-0.49882	0.42246
H	6.13790	-2.90751	0.68905
H	5.65071	1.32555	-0.00629
H	2.44235	-0.94797	1.74657
H	-3.34859	1.89906	1.87930
H	-4.10617	1.64076	-2.35522
C	7.51607	-0.73453	-0.15531
H	7.73579	0.32064	-0.43148
C	3.65811	-3.36792	1.85006
H	4.43714	-4.15147	1.71981
C	2.40274	-3.86485	1.12925
H	2.10898	-4.86320	1.51761
H	1.55470	-3.16665	1.27976
H	2.60742	-3.96301	0.04442
C	3.40747	-3.23254	3.35728
H	4.32528	-2.86513	3.86433
H	2.57492	-2.52686	3.56413
H	3.14501	-4.22192	3.78906
C	8.46135	-1.09766	0.99718

H	8.23041	-0.47685	1.88932
H	8.36683	-2.16998	1.27070
H	9.51423	-0.90108	0.70218
C	7.80806	-1.58803	-1.39362
H	7.59825	-2.66185	-1.20407
H	7.18902	-1.23837	-2.24350
H	8.87445	-1.48305	-1.68670
C	-5.33857	0.29018	2.75651
H	-6.20139	-0.41194	2.73448
C	-5.77673	1.51630	3.56605
H	-6.61721	2.03036	3.05245
H	-6.12531	1.20180	4.57291
H	-4.93888	2.23471	3.69105
C	-4.18321	-0.44205	3.44777
H	-3.31598	0.23248	3.60998
H	-4.51488	-0.83646	4.43183
H	-3.85745	-1.29613	2.82236
C	-6.37973	-0.00173	-2.20347
H	-7.19123	-0.65237	-1.80612
C	-7.05623	1.16913	-2.92526
H	-6.30630	1.82439	-3.41722
H	-7.75323	0.78613	-3.70097
H	-7.64229	1.77475	-2.20119
C	-5.56438	-0.85120	-3.18352
H	-6.23022	-1.27659	-3.96435
H	-4.77575	-0.24884	-3.68189
H	-5.08558	-1.69201	-2.64255

Catalyst (3,5-(i-Pr)2, 7)-Enamine protonation TS (pro-R) alternative conformation
Electronic energy (Hartrees): -1870.554505
Free Energy at 1M concentration and 253 K (Hartrees): -1869.506734
Coordinates:

P	-0.24811	0.28870	0.29312
O	1.24392	0.78815	-0.18993
O	-1.00304	1.57843	0.93127
O	-0.24289	-0.81802	1.29108
O	-0.92771	-0.00408	-1.08891
C	1.86221	1.74077	0.59060
C	3.21252	1.45004	1.16142
C	3.84103	2.39471	1.88387
H	4.81176	2.19303	2.32243
C	3.21354	3.70548	2.12907
C	3.89509	4.69474	2.99655
H	4.86340	4.46735	3.42488
C	3.31718	5.87219	3.25640
H	3.81509	6.59375	3.89118
C	1.99287	6.18621	2.68833
H	1.52047	7.13230	2.91928
C	1.37208	5.30097	1.90018
H	0.39180	5.54463	1.51294
C	2.01611	3.99717	1.57742
C	1.34929	2.99642	0.69176
C	-1.00243	2.66780	0.09786
C	-2.24998	3.04432	-0.61730
C	-2.23864	4.09052	-1.45886
H	-3.14050	4.37500	-1.98864
C	-0.99262	4.84679	-1.70049
C	-0.99264	5.96526	-2.67197
H	-1.91121	6.25684	-3.16593

C	0.14153	6.61810	-2.94611
H	0.14054	7.43402	-3.65729
C	1.40128	6.21791	-2.29188
H	2.32057	6.73385	-2.53750
C	1.41182	5.21215	-1.40952
H	2.35326	4.92059	-0.96455
C	0.15151	4.49686	-1.06672
C	0.13272	3.37389	-0.07984
C	-3.47312	2.22718	-0.44529
C	-3.95452	1.89687	0.91650
C	-4.11234	1.71918	-1.51293
C	-4.99988	1.07055	1.09535
C	-5.27123	0.81047	-1.34064
C	-5.67571	0.49359	-0.09566
C	3.87835	0.13870	0.94570
C	5.16177	0.07917	0.54364
C	3.17226	-1.11695	1.28688
C	5.87796	-1.21786	0.46762
C	3.79101	-2.30774	1.19616
C	5.21006	-2.35083	0.75738
H	-1.05323	-2.34450	0.90249
C	-2.64642	-3.32907	1.72740
C	-2.19689	-3.07223	-0.65436
C	-2.20578	-3.12462	3.04839
C	-3.91269	-3.90971	1.47362
C	-3.49856	-3.62747	-0.91385
C	-3.01857	-3.51671	4.09862
H	-1.24769	-2.64541	3.22130
C	-4.71252	-4.30438	2.56599
C	-4.30767	-4.03380	0.10354
H	-3.79916	-3.74256	-1.94527
C	-4.27219	-4.11443	3.86364
H	-2.68283	-3.35682	5.11858
H	-5.68291	-4.75175	2.37007
H	-5.27546	-4.47406	-0.12177
H	-4.89268	-4.41683	4.70073
C	-1.29556	-2.59530	-1.64502
H	-1.20755	-1.02918	-1.30346
C	0.19277	-2.93245	-1.54944
H	0.35509	-3.92873	-1.97430
H	0.46307	-2.99415	-0.48281
N	1.02497	-1.98351	-2.26622
N	-1.85190	-2.95259	0.66584
C	2.34089	-2.23975	-2.64249
C	2.79099	-3.51088	-3.04529
C	3.25393	-1.16698	-2.68488
C	4.10296	-3.68976	-3.47884
H	2.11556	-4.35950	-3.03523
C	4.55335	-1.35509	-3.14018
H	2.92716	-0.18583	-2.35029
C	4.99440	-2.61896	-3.54030
H	4.42527	-4.68188	-3.78368
H	5.22959	-0.50478	-3.17123
H	6.01092	-2.76710	-3.89060
H	0.86329	-1.02072	-2.00537
Cl	-1.88171	-2.72293	-3.34364
H	-6.50545	-0.18549	0.04772
H	5.72216	-3.30303	0.72713
H	5.69644	0.99092	0.30475
H	2.17130	-1.05869	1.68214

H	-3.44940	2.33242	1.77031
H	-3.75152	1.93304	-2.51217
C	7.36512	-1.23107	0.15888
H	7.70941	-0.18679	-0.01094
C	3.08072	-3.57467	1.63804
H	2.01697	-3.34795	1.87836
C	3.72226	-4.13413	2.91228
H	3.15041	-5.01643	3.27112
H	4.77317	-4.44355	2.72790
H	3.71024	-3.36430	3.71340
C	3.07127	-4.62922	0.52657
H	2.61575	-4.20804	-0.38955
H	4.09817	-4.97792	0.28894
H	2.46515	-5.50581	0.83988
C	8.17061	-1.77631	1.34543
H	7.92545	-1.20562	2.26676
H	7.95170	-2.85127	1.51904
H	9.25857	-1.66600	1.14955
C	7.67426	-2.01509	-1.12074
H	7.33170	-3.06861	-1.04405
H	7.17744	-1.53092	-1.98443
H	8.76789	-2.01217	-1.31578
C	-5.47645	0.72792	2.49390
H	-4.86965	1.27938	3.24725
C	-5.29074	-0.76528	2.77680
H	-5.57256	-0.99490	3.82646
H	-5.91419	-1.38515	2.09802
H	-4.22476	-1.03964	2.63678
C	-6.93561	1.15056	2.69850
H	-7.61850	0.57132	2.04167
H	-7.23535	0.98011	3.75458
H	-7.05317	2.23252	2.47492
C	-5.95389	0.23474	-2.56680
H	-5.48222	0.66319	-3.47890
C	-5.76618	-1.28428	-2.63881
H	-6.24500	-1.79127	-1.77416
H	-6.21299	-1.68076	-3.57531
H	-4.68275	-1.52508	-2.64387
C	-7.43947	0.61367	-2.61353
H	-7.88578	0.27980	-3.57452
H	-8.00231	0.13946	-1.78189
H	-7.55336	1.71660	-2.54284

Catalyst (3,5-(i-Pr)2, 7)-Enamine protonation TS (pro-R) alternative conformation
Electronic energy (Hartrees): -1870.552881
Free Energy at 1M concentration and 253 K (Hartrees): -1869.506313
Coordinates:

P	-0.07594	0.17321	0.06834
O	1.35679	0.81119	-0.39088
O	-0.96654	1.39118	0.69151
O	0.02185	-0.91679	1.08193
O	-0.69370	-0.20215	-1.32274
C	1.87896	1.76498	0.45178
C	3.19384	1.51434	1.09599
C	3.76015	2.46401	1.85577
H	4.72063	2.28482	2.32590
C	3.06846	3.74579	2.09274
C	3.66975	4.74729	3.00387
H	4.62969	4.55484	3.46672

C	3.02881	5.89232	3.26135
H	3.46803	6.62278	3.92845
C	1.71439	6.15797	2.64691
H	1.19094	7.07723	2.87620
C	1.16515	5.26192	1.81878
H	0.19060	5.46895	1.39739
C	1.87943	3.99523	1.49849
C	1.29425	2.98338	0.56689
C	-1.01140	2.50645	-0.10933
C	-2.26925	2.84106	-0.82729
C	-2.30158	3.90537	-1.64532
H	-3.21446	4.16399	-2.16955
C	-1.09604	4.73410	-1.84855
C	-1.14117	5.87153	-2.79674
H	-2.06353	6.11719	-3.30830
C	-0.04314	6.59865	-3.02763
H	-0.07692	7.42848	-3.72172
C	1.22202	6.26270	-2.34819
H	2.11338	6.83959	-2.55864
C	1.27250	5.24068	-1.48609
H	2.21846	4.99832	-1.02155
C	0.05060	4.44203	-1.19128
C	0.07717	3.29722	-0.23019
C	-3.47262	1.99962	-0.64357
C	-3.95484	1.69843	0.72349
C	-4.12380	1.48856	-1.70205
C	-5.01758	0.89566	0.91723
C	-5.30222	0.60798	-1.51300
C	-5.70656	0.31402	-0.26155
C	3.89062	0.23094	0.89380
C	5.02642	0.18168	0.18086
C	3.38400	-0.98923	1.55668
C	5.76763	-1.09932	0.02263
C	4.05888	-2.15016	1.46121
C	5.29007	-2.20169	0.63250
H	-0.94388	-2.37251	0.88413
C	-2.56035	-3.18257	1.85288
C	-2.22849	-3.09760	-0.55735
C	-2.02372	-2.95851	3.13497
C	-3.88677	-3.65576	1.69887
C	-3.58507	-3.54084	-0.71931
C	-2.80412	-3.22364	4.24703
H	-1.01694	-2.56471	3.22892
C	-4.65331	-3.91899	2.85365
C	-4.36994	-3.81241	0.36156
H	-3.94826	-3.69276	-1.72526
C	-4.12000	-3.70980	4.11250
H	-2.39502	-3.04966	5.23741
H	-5.67016	-4.28268	2.73591
H	-5.38459	-4.17247	0.21295
H	-4.71428	-3.91205	4.99744
C	-1.32531	-2.77791	-1.61285
H	-1.06668	-1.22287	-1.41873
C	0.09679	-3.32404	-1.53277
H	0.10054	-4.34977	-1.92712
H	0.36967	-3.39318	-0.46966
N	1.05340	-2.54343	-2.28804
N	-1.80282	-2.92768	0.73165
C	2.37656	-2.93345	-2.43458
C	2.83907	-4.21276	-2.07015

C	3.29942	-2.03196	-3.00610
C	4.17063	-4.57063	-2.28297
H	2.16515	-4.93296	-1.62070
C	4.61839	-2.40772	-3.22239
H	2.96051	-1.03847	-3.28895
C	5.07186	-3.68064	-2.86205
H	4.50060	-5.56313	-1.98752
H	5.30088	-1.70107	-3.68531
H	6.10537	-3.96717	-3.02828
H	0.88536	-1.54708	-2.30620
Cl	-2.00273	-2.94821	-3.27736
H	-6.55687	-0.33693	-0.10359
H	5.82391	-3.14037	0.54177
H	5.40380	1.09129	-0.26679
H	2.49514	-0.91834	2.16990
H	-3.44795	2.14905	1.56746
H	-3.77100	1.69527	-2.70564
C	7.07694	-1.18556	-0.74967
H	7.08054	-2.13947	-1.32239
C	3.63017	-3.38255	2.23747
H	4.34482	-4.20913	2.02779
C	2.24537	-3.87032	1.80297
H	1.97808	-4.79945	2.34992
H	1.46583	-3.10622	2.00428
H	2.25905	-4.10343	0.72064
C	3.67313	-3.12849	3.74959
H	4.68083	-2.76669	4.04651
H	2.91625	-2.37362	4.05129
H	3.46778	-4.07171	4.29950
C	7.26337	-0.05967	-1.77860
H	6.35553	0.04837	-2.40789
H	7.47688	0.90922	-1.27874
H	8.11888	-0.29792	-2.44616
C	8.26276	-1.21572	0.22140
H	8.29185	-0.28554	0.82900
H	8.18093	-2.08713	0.90548
H	9.21703	-1.30876	-0.33997
C	-5.54135	0.60064	2.31006
H	-6.38223	-0.12406	2.23448
C	-6.09581	1.86794	2.97072
H	-6.88330	2.31767	2.32878
H	-6.54985	1.61700	3.95311
H	-5.29315	2.61850	3.13304
C	-4.46597	-0.05015	3.18674
H	-3.65069	0.66491	3.42594
H	-4.91386	-0.40774	4.13832
H	-4.03102	-0.91932	2.65711
C	-6.02148	0.05720	-2.73011
H	-5.54772	0.46987	-3.64841
C	-5.88986	-1.46688	-2.80735
H	-6.35656	-1.95732	-1.92673
H	-6.38121	-1.84713	-3.72818
H	-4.81646	-1.74404	-2.85078
C	-7.49313	0.48916	-2.75663
H	-7.96321	0.17560	-3.71313
H	-8.06206	0.03161	-1.91983
H	-7.56679	1.59516	-2.68042

Catalyst (3,5-(i-Pr)2, 7)-Enamine protonation TS (pro-R) alternative conformation

Electronic energy (Hartrees): -1870.553815
Free Energy at 1M concentration and 253 K (Hartrees): -1869.506177
Coordinates:

P	-0.09777	0.17105	0.10779
O	1.36120	0.75551	-0.35589
O	-0.92401	1.39962	0.78831
O	-0.04105	-0.96640	1.06806
O	-0.76480	-0.11120	-1.28583
C	1.91546	1.73991	0.43333
C	3.27411	1.52467	1.01226
C	3.85880	2.50725	1.71898
H	4.83969	2.35683	2.15594
C	3.16569	3.78593	1.95396
C	3.79899	4.81774	2.80826
H	4.78194	4.64723	3.22963
C	3.15919	5.96335	3.06501
H	3.62183	6.71604	3.69026
C	1.81438	6.19900	2.50745
H	1.29184	7.11868	2.73711
C	1.23671	5.27495	1.73113
H	0.24029	5.46085	1.35338
C	1.94944	4.00662	1.41028
C	1.33308	2.96455	0.53416
C	-1.00518	2.50455	-0.02165
C	-2.29730	2.84513	-0.67640
C	-2.35667	3.89982	-1.50690
H	-3.29205	4.16904	-1.98374
C	-1.14956	4.70100	-1.79454
C	-1.22778	5.82310	-2.75874
H	-2.17350	6.07913	-3.22010
C	-0.13093	6.52330	-3.06627
H	-0.18895	7.34184	-3.77206
C	1.16504	6.17287	-2.45547
H	2.05364	6.72805	-2.72709
C	1.24573	5.16446	-1.57977
H	2.21181	4.91045	-1.16524
C	0.02780	4.39594	-1.20140
C	0.08804	3.26884	-0.22212
C	-3.50143	2.01723	-0.42447
C	-3.87073	1.65639	0.96434
C	-4.25394	1.56675	-1.44376
C	-4.92470	0.85675	1.21207
C	-5.43301	0.70272	-1.19298
C	-5.72994	0.35089	0.07317
C	3.99480	0.24250	0.82892
C	5.24905	0.22999	0.34294
C	3.39482	-1.01856	1.31986
C	6.03947	-1.02451	0.30791
C	4.09346	-2.17042	1.29321
C	5.47101	-2.16669	0.73995
H	-0.93901	-2.45570	0.71100
C	-2.48009	-3.35201	1.71341
C	-2.32014	-3.05458	-0.69817
C	-1.85505	-3.23163	2.96895
C	-3.81785	-3.80706	1.61383
C	-3.68700	-3.48335	-0.80076
C	-2.56031	-3.58112	4.10752
H	-0.84235	-2.84595	3.02415
C	-4.50550	-4.16021	2.79398
C	-4.39437	-3.84983	0.30530

H	-4.12087	-3.54835	-1.78781
C	-3.88590	-4.05232	4.02573
H	-2.08364	-3.48595	5.07821
H	-5.53079	-4.51114	2.71809
H	-5.41832	-4.19814	0.19823
H	-4.41986	-4.32272	4.93072
C	-1.49485	-2.63456	-1.78134
H	-1.18312	-1.10307	-1.43600
C	-0.08176	-3.19902	-1.88580
H	-0.12415	-4.13679	-2.45728
H	0.25393	-3.46837	-0.87259
N	0.84347	-2.29468	-2.54091
N	-1.80065	-3.00530	0.56648
C	2.18054	-2.61167	-2.72915
C	2.66335	-3.93337	-2.69629
C	3.09705	-1.57329	-2.99657
C	4.01132	-4.19718	-2.94025
H	1.99131	-4.75665	-2.48082
C	4.43330	-1.85269	-3.24661
H	2.74257	-0.54569	-3.00202
C	4.90733	-3.16863	-3.22349
H	4.35937	-5.22630	-2.90826
H	5.11012	-1.03020	-3.46130
H	5.95191	-3.38635	-3.42121
H	0.65866	-1.31306	-2.38803
Cl	-2.30761	-2.59632	-3.39274
H	-6.57854	-0.29023	0.27463
H	6.03718	-3.08925	0.73737
H	5.70711	1.15351	0.00842
H	2.41141	-0.98808	1.76518
H	-3.28576	2.05656	1.78261
H	-3.98500	1.81005	-2.46485
C	7.49509	-0.97538	-0.12320
H	7.75441	0.06916	-0.40537
C	3.53854	-3.44960	1.89385
H	4.29022	-4.26127	1.77563
C	2.27127	-3.90795	1.16843
H	1.93843	-4.89097	1.56432
H	1.44825	-3.17774	1.30532
H	2.48047	-4.02444	0.08624
C	3.28281	-3.29247	3.39813
H	4.20941	-2.95255	3.90835
H	2.47376	-2.55676	3.59328
H	2.98344	-4.26839	3.83647
C	8.42665	-1.36762	1.03093
H	8.21973	-0.73373	1.91974
H	8.29208	-2.43412	1.31031
H	9.48600	-1.21241	0.73443
C	7.75339	-1.84616	-1.35688
H	7.50514	-2.91051	-1.16039
H	7.14513	-1.47932	-2.20740
H	8.82224	-1.78158	-1.65268
C	-5.31858	0.48485	2.62912
H	-6.18750	-0.20914	2.59318
C	-5.76072	1.71932	3.42351
H	-6.58765	2.23715	2.89200
H	-6.12964	1.41389	4.42590
H	-4.91911	2.43152	3.55870
C	-4.18434	-0.25453	3.34727
H	-3.31697	0.41450	3.53010

H	-4.54160	-0.64678	4.32323
H	-3.84898	-1.11028	2.72970
C	-6.27625	0.23669	-2.36528
H	-5.87671	0.68734	-3.30086
C	-6.19767	-1.28357	-2.53433
H	-6.59158	-1.81032	-1.63924
H	-6.78619	-1.60083	-3.42144
H	-5.14227	-1.58346	-2.69722
C	-7.73078	0.70551	-2.23229
H	-8.29551	0.45654	-3.15602
H	-8.23395	0.21695	-1.37124
H	-7.76445	1.80716	-2.09184

Catalyst (3,5-(i-Pr)2, 7)-Enamine protonation TS (pro-R) alternative conformation
Electronic energy (Hartrees): -1870.553643
Free Energy at 1M concentration and 253 K (Hartrees): -1869.506113
Coordinates:

P	-0.18793	0.13576	0.15234
O	1.27456	0.69709	-0.32142
O	-0.95093	1.36195	0.90708
O	-0.12899	-1.03793	1.07043
O	-0.91015	-0.06983	-1.22371
C	1.88531	1.59242	0.52783
C	3.22123	1.25520	1.09291
C	3.87488	2.15647	1.84368
H	4.84489	1.91513	2.26398
C	3.26599	3.46321	2.15308
C	3.96889	4.40478	3.05562
H	4.94337	4.15146	3.45438
C	3.40245	5.57052	3.38434
H	3.91599	6.25718	4.04488
C	2.06911	5.91824	2.85843
H	1.60600	6.85332	3.14610
C	1.42856	5.07649	2.03900
H	0.44196	5.34327	1.68464
C	2.05947	3.78816	1.63813
C	1.37312	2.83773	0.71108
C	-0.98899	2.51071	0.15687
C	-2.26254	2.92076	-0.49104
C	-2.28613	4.01846	-1.26443
H	-3.20813	4.33130	-1.74096
C	-1.05474	4.80035	-1.49835
C	-1.09431	5.97492	-2.40039
H	-2.02996	6.28537	-2.84868
C	0.02507	6.65539	-2.66816
H	-0.00478	7.51228	-3.32875
C	1.30754	6.23064	-2.07651
H	2.21434	6.77015	-2.31790
C	1.35367	5.17432	-1.25655
H	2.31059	4.86678	-0.85770
C	0.11066	4.42656	-0.91989
C	0.13193	3.24576	-0.00342
C	-3.48188	2.09825	-0.31439
C	-3.91215	1.69880	1.04541
C	-4.18240	1.67262	-1.37928
C	-4.98641	0.90705	1.22320
C	-5.37273	0.80105	-1.20628
C	-5.74232	0.43786	0.03542
C	3.83834	-0.06663	0.85045

C	5.02998	-0.15718	0.23580
C	3.21540	-1.28146	1.42081
C	5.72185	-1.46910	0.10580
C	3.83721	-2.47330	1.34208
C	5.13410	-2.56297	0.62662
H	-1.03620	-2.47068	0.68001
C	-2.62438	-3.41397	1.56240
C	-2.30664	-3.07794	-0.82796
C	-2.08846	-3.29852	2.85923
C	-3.93790	-3.90555	1.36608
C	-3.64907	-3.54883	-1.03065
C	-2.85821	-3.68692	3.94204
H	-1.09311	-2.88676	2.98920
C	-4.69382	-4.29572	2.49160
C	-4.41999	-3.94910	0.01987
H	-4.01642	-3.60388	-2.04532
C	-4.16197	-4.19145	3.76397
H	-2.45048	-3.59554	4.94400
H	-5.70109	-4.67372	2.34112
H	-5.42430	-4.32275	-0.16183
H	-4.74789	-4.49073	4.62673
C	-1.43449	-2.59887	-1.84904
H	-1.25771	-1.08329	-1.45929
C	0.03054	-3.02613	-1.83840
H	0.11962	-4.01872	-2.30208
H	0.34456	-3.13312	-0.79022
N	0.87930	-2.10130	-2.56074
N	-1.87741	-3.03405	0.46943
C	2.23832	-2.31125	-2.73738
C	2.86860	-3.53764	-2.45537
C	3.02466	-1.26309	-3.26049
C	4.23003	-3.70607	-2.71225
H	2.30045	-4.36472	-2.04565
C	4.37537	-1.44863	-3.51801
H	2.55374	-0.30679	-3.47320
C	4.99508	-2.67376	-3.25025
H	4.69287	-4.66257	-2.48403
H	4.94978	-0.62724	-3.93788
H	6.05170	-2.81465	-3.45399
H	0.59795	-1.13212	-2.51071
Cl	-2.11607	-2.64087	-3.51904
H	-6.60399	-0.20242	0.18043
H	5.63037	-3.52385	0.55764
H	5.49687	0.74442	-0.13709
H	2.28926	-1.18298	1.96969
H	-3.35248	2.06062	1.89863
H	-3.86230	1.94267	-2.37803
C	7.10612	-1.59947	-0.51605
H	7.12426	-2.52761	-1.12997
C	3.28848	-3.70532	2.04058
H	3.98656	-4.55586	1.87590
C	1.93403	-4.12573	1.46490
H	1.58215	-5.05755	1.95665
H	1.17135	-3.33644	1.62243
H	2.03613	-4.32935	0.38002
C	3.19801	-3.49152	3.55705
H	4.18511	-3.17461	3.95667
H	2.44199	-2.71785	3.80922
H	2.90978	-4.44033	4.05793
C	7.47512	-0.44461	-1.45924

H	6.66430	-0.26820	-2.19455
H	7.66501	0.49173	-0.89240
H	8.39854	-0.69906	-2.02209
C	8.16759	-1.74075	0.58160
H	8.17481	-0.84075	1.23372
H	7.96137	-2.63440	1.20832
H	9.17467	-1.86515	0.12912
C	-5.43754	0.48590	2.60890
H	-6.33366	-0.16693	2.51566
C	-5.85049	1.69685	3.45404
H	-6.63273	2.28139	2.92408
H	-6.26941	1.35669	4.42511
H	-4.98263	2.35939	3.65756
C	-4.35597	-0.33794	3.31555
H	-3.45581	0.27620	3.53020
H	-4.74742	-0.74211	4.27336
H	-4.06117	-1.19112	2.67274
C	-6.15604	0.32096	-2.41334
H	-7.00924	-0.31407	-2.08384
C	-6.74968	1.50423	-3.18593
H	-5.95060	2.14372	-3.61739
H	-7.39071	1.13397	-4.01431
H	-7.37887	2.12234	-2.51023
C	-5.27894	-0.54469	-3.32392
H	-5.88698	-0.96137	-4.15496
H	-4.44318	0.04391	-3.75800
H	-4.85740	-1.39168	-2.74472

Catalyst (3,5-(i-Pr)2, 7)-Enamine protonation TS (pro-R) alternative conformation
Electronic energy (Hartrees): -1870.551874
Free Energy at 1M concentration and 253 K (Hartrees): -1869.503606
Coordinates:

P	0.16553	0.12732	0.03182
O	1.44775	1.00581	-0.45404
O	-0.96476	1.17977	0.57040
O	0.43883	-0.85271	1.12392
O	-0.32157	-0.45410	-1.34204
C	1.79534	2.02555	0.40023
C	3.09958	1.96482	1.10124
C	3.47280	2.95928	1.91769
H	4.42797	2.91555	2.42855
C	2.57887	4.11427	2.14099
C	2.97382	5.17431	3.09726
H	3.92685	5.11824	3.60849
C	2.15457	6.20409	3.33549
H	2.44349	6.97745	4.03546
C	0.84866	6.28352	2.65381
H	0.18203	7.10937	2.86630
C	0.48394	5.33376	1.78469
H	-0.48676	5.40228	1.31217
C	1.39587	4.19560	1.48717
C	1.01531	3.12499	0.51588
C	-1.15535	2.27064	-0.24268
C	-2.42176	2.39465	-1.01054
C	-2.59417	3.44199	-1.83451
H	-3.51873	3.55498	-2.38915
C	-1.53588	4.45902	-1.99362
C	-1.72942	5.57337	-2.95058
H	-2.65565	5.65982	-3.50504

C	-0.76079	6.47604	-3.13656
H	-0.90254	7.28875	-3.83721
C	0.51006	6.35891	-2.39746
H	1.29942	7.07907	-2.57039
C	0.69283	5.35946	-1.52684
H	1.64318	5.28147	-1.01685
C	-0.38826	4.36434	-1.28359
C	-0.21225	3.23825	-0.31710
C	-3.50035	1.39138	-0.84940
C	-4.00633	1.06429	0.50353
C	-4.07333	0.82325	-1.92219
C	-5.07830	0.26570	0.67280
C	-5.22284	-0.10178	-1.76718
C	-5.70163	-0.35161	-0.53457
C	3.98754	0.81623	0.87798
C	4.90606	0.85289	-0.09674
C	3.82144	-0.40458	1.69188
C	5.73889	-0.34048	-0.39139
C	4.56518	-1.49849	1.44223
C	5.55852	-1.45910	0.33737
H	-0.70757	-2.18091	1.19208
C	-2.38731	-2.72756	2.24047
C	-1.98629	-3.12130	-0.13100
C	-1.89770	-2.22410	3.46035
C	-3.69277	-3.26823	2.15529
C	-3.34662	-3.57181	-0.24290
C	-2.69737	-2.29587	4.58796
H	-0.90719	-1.78152	3.49275
C	-4.47067	-3.34958	3.32930
C	-4.14450	-3.67086	0.85757
H	-3.69401	-3.89039	-1.21514
C	-3.98232	-2.87039	4.53124
H	-2.32412	-1.90678	5.53027
H	-5.46512	-3.78312	3.27119
H	-5.14992	-4.07110	0.75711
H	-4.58900	-2.92944	5.42880
C	-1.04015	-3.05375	-1.19229
H	-0.71313	-1.45526	-1.29290
C	0.35150	-3.62606	-0.94323
H	0.31036	-4.71572	-1.08292
H	0.60372	-3.45061	0.11261
N	1.35986	-3.09857	-1.83787
N	-1.59749	-2.70122	1.11322
C	2.66367	-3.57769	-1.83563
C	3.05205	-4.72615	-1.11868
C	3.64184	-2.91537	-2.60632
C	4.36956	-5.18182	-1.17165
H	2.33479	-5.25887	-0.50505
C	4.94802	-3.38578	-2.65495
H	3.35477	-2.03439	-3.17487
C	5.32971	-4.52181	-1.93481
H	4.64232	-6.06466	-0.59946
H	5.67964	-2.86371	-3.26511
H	6.35346	-4.88029	-1.97020
H	1.25289	-2.12385	-2.08331
Cl	-1.66967	-3.53682	-2.81248
H	-6.55392	-1.00728	-0.42910
H	6.16197	-2.33721	0.14236
H	5.02523	1.75284	-0.68739
H	3.09737	-0.39962	2.49689

H	-3.52503	1.51662	1.36205
H	-3.70574	1.05403	-2.91444
C	6.79598	-0.28971	-1.47857
H	7.22559	-1.30518	-1.63343
C	4.44022	-2.74874	2.29242
H	5.08174	-3.54686	1.85698
C	3.00352	-3.28446	2.30587
H	2.97475	-4.28402	2.78941
H	2.32172	-2.60545	2.86035
H	2.63311	-3.38837	1.26873
C	4.94090	-2.49216	3.71817
H	5.98945	-2.12554	3.69257
H	4.30931	-1.73686	4.23285
H	4.91678	-3.43479	4.30558
C	6.20277	0.15902	-2.82112
H	5.29778	-0.43405	-3.06371
H	5.92459	1.23375	-2.80101
H	6.94685	0.00735	-3.63189
C	7.94692	0.63100	-1.06052
H	7.59250	1.67557	-0.92671
H	8.38821	0.27741	-0.10404
H	8.74354	0.62153	-1.83483
C	-5.61526	0.03494	2.08393
H	-4.77019	-0.32058	2.71480
C	-6.71748	-1.03262	2.16825
H	-6.38023	-1.98183	1.70624
H	-6.96156	-1.24272	3.23162
H	-7.64278	-0.68827	1.65816
C	-6.13985	1.34787	2.68570
H	-6.95557	1.76456	2.05631
H	-6.53179	1.17294	3.71060
H	-5.32857	2.10121	2.76376
C	-5.86961	-0.73260	-2.98686
H	-6.71575	-1.38528	-2.67431
C	-6.45179	0.34023	-3.91435
H	-5.65113	0.98057	-4.34131
H	-7.00256	-0.13954	-4.75141
H	-7.16448	0.98199	-3.35334
C	-4.87249	-1.62320	-3.73540
H	-5.37699	-2.12864	-4.58630
H	-4.02040	-1.03078	-4.13087
H	-4.48067	-2.40550	-3.05305

Catalyst (3,5-(i-Pr)2, 7)-Enamine protonation TS (pro-R) alternative conformation
Electronic energy (Hartrees): -1870.551607
Free Energy at 1M concentration and 253 K (Hartrees): -1869.503511
Coordinates:

P	-0.22626	0.20462	0.18952
O	1.22904	0.79594	-0.29558
O	-1.07307	1.44527	0.81606
O	-0.15781	-0.88815	1.20158
O	-0.87940	-0.14943	-1.19113
C	1.78466	1.78685	0.48466
C	3.15112	1.58741	1.05869
C	3.70319	2.56588	1.79942
H	4.68297	2.43067	2.24328
C	2.98452	3.82780	2.04942
C	3.58806	4.85422	2.93145
H	4.56375	4.68849	3.37118

C	2.93041	5.98932	3.19015
H	3.37138	6.73805	3.83548
C	1.59599	6.21927	2.60569
H	1.05947	7.13094	2.83503
C	1.04531	5.30010	1.80438
H	0.05652	5.48149	1.40477
C	1.77778	4.04324	1.48395
C	1.18871	3.00484	0.58710
C	-1.13462	2.53166	-0.01908
C	-2.40283	2.84034	-0.73156
C	-2.44814	3.88410	-1.57562
H	-3.36630	4.12390	-2.09946
C	-1.24570	4.70693	-1.81941
C	-1.30693	5.82208	-2.79280
H	-2.23882	6.05872	-3.29117
C	-0.21172	6.53995	-3.06294
H	-0.25733	7.35346	-3.77543
C	1.06614	6.21589	-2.40162
H	1.95443	6.78540	-2.64289
C	1.13153	5.21370	-1.51754
H	2.08584	4.97958	-1.06595
C	-0.08595	4.42523	-1.18077
C	-0.04311	3.30420	-0.19308
C	-3.59091	1.97684	-0.53755
C	-4.04078	1.63878	0.83269
C	-4.24248	1.45769	-1.59251
C	-5.07292	0.79777	1.03105
C	-5.38816	0.53628	-1.39775
C	-5.76512	0.21513	-0.14484
C	3.92168	0.33759	0.81874
C	5.21279	0.39109	0.44455
C	3.31004	-0.98059	1.09688
C	6.03030	-0.84140	0.34566
C	4.02216	-2.11971	1.00310
C	5.45405	-2.03265	0.59817
H	-0.96138	-2.42805	0.89143
C	-2.53807	-3.35691	1.81008
C	-2.18328	-3.17431	-0.59162
C	-2.03309	-3.13735	3.10562
C	-3.83555	-3.89361	1.62359
C	-3.50904	-3.69278	-0.78570
C	-2.81620	-3.46762	4.19821
H	-1.05090	-2.69205	3.22543
C	-4.60511	-4.22360	2.75900
C	-4.29050	-4.04111	0.27528
H	-3.85248	-3.82936	-1.80093
C	-4.10311	-4.01676	4.03099
H	-2.43225	-3.29530	5.19890
H	-5.59968	-4.63647	2.61586
H	-5.28119	-4.45299	0.10169
H	-4.69983	-4.26966	4.90115
C	-1.30168	-2.74262	-1.62471
H	-1.17484	-1.19254	-1.34081
C	0.16491	-3.16641	-1.57956
H	0.26256	-4.13009	-2.09632
H	0.43923	-3.34591	-0.52785
N	1.04106	-2.20653	-2.21815
N	-1.77922	-3.03169	0.70725
C	2.38640	-2.43206	-2.45883
C	2.95834	-3.71767	-2.48223

C	3.21916	-1.32683	-2.73593
C	4.30204	-3.88299	-2.81936
H	2.35913	-4.59040	-2.24755
C	4.55483	-1.50824	-3.06628
H	2.79794	-0.32610	-2.68563
C	5.11175	-2.79006	-3.11982
H	4.71780	-4.88703	-2.83994
H	5.16639	-0.63666	-3.28363
H	6.15402	-2.93252	-3.38596
H	0.78476	-1.23773	-2.09556
Cl	-1.96552	-2.86449	-3.29837
H	-6.58939	-0.46765	0.01683
H	6.05463	-2.92838	0.54376
H	5.67854	1.34863	0.24356
H	2.28727	-1.01648	1.43293
H	-3.53039	2.08604	1.67624
H	-3.90871	1.68188	-2.59882
C	7.51408	-0.72491	0.04118
H	7.77178	0.34795	-0.10174
C	3.35046	-3.44105	1.36477
H	2.38550	-3.49164	0.81612
C	3.04385	-3.49750	2.86818
H	2.54592	-4.45723	3.12409
H	3.98123	-3.41037	3.45875
H	2.35791	-2.67526	3.16181
C	4.15152	-4.68590	0.95507
H	3.53647	-5.59833	1.10839
H	4.42341	-4.63869	-0.11829
H	5.07533	-4.78383	1.56462
C	8.36200	-1.23208	1.21480
H	8.07263	-0.70527	2.14932
H	8.23073	-2.32501	1.36244
H	9.43730	-1.02989	1.02231
C	7.88581	-1.44729	-1.25790
H	7.66526	-2.53369	-1.19293
H	7.32158	-1.00931	-2.10635
H	8.96906	-1.31951	-1.46837
C	-5.54610	0.44237	2.42775
H	-6.38959	-0.27926	2.35199
C	-6.07566	1.67689	3.16685
H	-6.87939	2.16063	2.57146
H	-6.50237	1.37790	4.14802
H	-5.26590	2.41601	3.34479
C	-4.43780	-0.24750	3.23006
H	-3.59900	0.44871	3.44252
H	-4.84097	-0.62289	4.19470
H	-4.04743	-1.11086	2.65620
C	-6.09755	-0.03503	-2.61102
H	-5.64727	0.39845	-3.53153
C	-5.91043	-1.55330	-2.69552
H	-6.36184	-2.06481	-1.81892
H	-6.38529	-1.94617	-3.61969
H	-4.82756	-1.79232	-2.73585
C	-7.58419	0.34254	-2.62253
H	-8.05155	0.01369	-3.57522
H	-8.12780	-0.13709	-1.78127
H	-7.69761	1.44494	-2.54310

Catalyst (3,5-(i-Pr)2, 7)-Enamine protonation TS (pro-R) alternative conformation

Electronic energy (Hartrees): -1870.551531
Free Energy at 1M concentration and 253 K (Hartrees): -1869.503053
Coordinates:

P	0.19010	0.15026	0.01064
O	1.50397	0.97567	-0.48286
O	-0.90555	1.25009	0.52417
O	0.42304	-0.82119	1.11906
O	-0.30998	-0.43679	-1.35758
C	1.88785	1.98814	0.36455
C	3.18428	1.88156	1.07449
C	3.58897	2.86475	1.88959
H	4.53850	2.78756	2.40698
C	2.73807	4.05397	2.10117
C	3.16628	5.10281	3.05547
H	4.11270	5.01287	3.57408
C	2.38528	6.16430	3.28280
H	2.69844	6.92945	3.98135
C	1.08839	6.29023	2.59097
H	0.45227	7.14196	2.79470
C	0.69400	5.35088	1.72350
H	-0.26991	5.45410	1.24344
C	1.56391	4.17732	1.43802
C	1.14957	3.11698	0.46933
C	-1.04654	2.34043	-0.30002
C	-2.30129	2.50726	-1.07901
C	-2.42902	3.55660	-1.90845
H	-3.34415	3.70029	-2.47149
C	-1.33276	4.53316	-2.06386
C	-1.47801	5.64966	-3.02686
H	-2.39665	5.76827	-3.58796
C	-0.47499	6.51452	-3.21012
H	-0.58152	7.32881	-3.91517
C	0.78568	6.35312	-2.46201
H	1.60265	7.04235	-2.63271
C	0.92506	5.35146	-1.58593
H	1.86848	5.24016	-1.06928
C	-0.19422	4.39871	-1.34566
C	-0.06726	3.27167	-0.37255
C	-3.41443	1.54136	-0.92575
C	-3.94089	1.23226	0.42388
C	-3.99447	0.98730	-2.00251
C	-5.03241	0.45926	0.58633
C	-5.16156	0.08741	-1.85464
C	-5.65431	-0.15285	-0.62392
C	4.03099	0.70057	0.86060
C	4.94566	0.69488	-0.11839
C	3.82857	-0.50390	1.69042
C	5.73759	-0.52867	-0.40151
C	4.53463	-1.62480	1.45116
C	5.52348	-1.63185	0.34154
H	-0.76520	-2.11617	1.20526
C	-2.46539	-2.59385	2.25382
C	-2.06268	-3.04467	-0.10783
C	-1.96986	-2.07811	3.46606
C	-3.78491	-3.09977	2.17176
C	-3.43315	-3.46551	-0.21659
C	-2.77899	-2.10157	4.58915
H	-0.96733	-1.66329	3.49635
C	-4.57314	-3.13161	3.34100
C	-4.23895	-3.52123	0.88054

H	-3.78211	-3.80008	-1.18269
C	-4.07966	-2.63937	4.53575
H	-2.40088	-1.70231	5.52527
H	-5.57955	-3.53688	3.28450
H	-5.25267	-3.90071	0.78277
H	-4.69416	-2.66006	5.42968
C	-1.11373	-3.02131	-1.16670
H	-0.73387	-1.41696	-1.29311
C	0.26276	-3.62395	-0.90748
H	0.19361	-4.71455	-1.02784
H	0.51910	-3.43635	0.14528
N	1.28441	-3.13983	-1.81126
N	-1.66837	-2.61261	1.13122
C	2.57191	-3.66069	-1.81060
C	2.92988	-4.80844	-1.07683
C	3.56395	-3.04381	-2.60105
C	4.23184	-5.30659	-1.13235
H	2.20149	-5.30824	-0.44875
C	4.85413	-3.55620	-2.65185
H	3.29980	-2.16489	-3.18371
C	5.20594	-4.69089	-1.91460
H	4.48128	-6.18762	-0.54687
H	5.59638	-3.06851	-3.27747
H	6.21748	-5.08232	-1.95177
H	1.20612	-2.16666	-2.07277
Cl	-1.75448	-3.50835	-2.78113
H	-6.51488	-0.79688	-0.52355
H	6.09731	-2.53147	0.15537
H	5.09229	1.58341	-0.72003
H	3.10911	-0.46495	2.49863
H	-3.45832	1.67846	1.28487
H	-3.61454	1.20527	-2.99377
C	6.79308	-0.52517	-1.49147
H	7.18509	-1.55683	-1.63878
C	4.37366	-2.85919	2.31833
H	4.98712	-3.68317	1.89070
C	2.92062	-3.34826	2.34530
H	2.86181	-4.33859	2.84486
H	2.26307	-2.63903	2.89115
H	2.54301	-3.45692	1.31128
C	4.88926	-2.60023	3.73834
H	5.94878	-2.26749	3.70323
H	4.28449	-1.81865	4.24596
H	4.83815	-3.53393	4.33815
C	6.21381	-0.06595	-2.83661
H	5.28558	-0.62516	-3.07136
H	5.97801	1.01899	-2.82589
H	6.94900	-0.25405	-3.64784
C	7.97758	0.35649	-1.08316
H	7.66125	1.41408	-0.95624
H	8.40844	-0.00569	-0.12515
H	8.77123	0.31262	-1.85933
C	-5.59243	0.25210	1.99224
H	-4.76524	-0.12240	2.63559
C	-6.72539	-0.78345	2.06919
H	-6.40755	-1.74656	1.62273
H	-6.99249	-0.97562	3.13041
H	-7.63223	-0.41879	1.54035
C	-6.08811	1.58273	2.57974
H	-6.88380	2.01802	1.93741

H	-6.49792	1.42498	3.60043
H	-5.25746	2.31387	2.66438
C	-5.78775	-0.52572	-3.09388
H	-5.23878	-0.16738	-3.99286
C	-5.66701	-2.05363	-3.07850
H	-6.22004	-2.49346	-2.22154
H	-6.07653	-2.47807	-4.01984
H	-4.59834	-2.34432	-3.00633
C	-7.24813	-0.08676	-3.26002
H	-7.64556	-0.45140	-4.23123
H	-7.88562	-0.49182	-2.44590
H	-7.31543	1.02224	-3.25236

Catalyst (3,5-(i-Pr)2, 7)-Enamine protonation TS (pro-R) alternative conformation
Electronic energy (Hartrees): -1870.550919
Free Energy at 1M concentration and 253 K (Hartrees): -1869.502336
Coordinates:

P	0.11549	0.16239	0.09965
O	1.39979	1.03279	-0.39719
O	-1.02674	1.22030	0.59457
O	0.38458	-0.79006	1.21580
O	-0.34495	-0.45507	-1.26829
C	1.72486	2.09266	0.41584
C	3.03548	2.09079	1.10859
C	3.40004	3.13716	1.86253
H	4.36117	3.13892	2.36407
C	2.48630	4.28464	2.03658
C	2.86922	5.39910	2.93412
H	3.82818	5.38762	3.43715
C	2.03170	6.42326	3.12924
H	2.31180	7.23647	3.78631
C	0.71774	6.44238	2.45870
H	0.03658	7.26452	2.63669
C	0.36392	5.44297	1.64234
H	-0.61254	5.46809	1.17735
C	1.29595	4.30962	1.39242
C	0.92943	3.18629	0.47704
C	-1.22577	2.27571	-0.26177
C	-2.48836	2.35340	-1.04262
C	-2.66907	3.36806	-1.90498
H	-3.59098	3.44839	-2.46962
C	-1.62199	4.39068	-2.09743
C	-1.82350	5.46519	-3.09738
H	-2.74881	5.52092	-3.65730
C	-0.86320	6.36946	-3.31567
H	-1.01073	7.15296	-4.04770
C	0.40671	6.29310	-2.56977
H	1.19001	7.01301	-2.76926
C	0.59649	5.33002	-1.66051
H	1.54680	5.28028	-1.14703
C	-0.47614	4.33522	-1.38054
C	-0.29298	3.24993	-0.36934
C	-3.55240	1.33800	-0.85763
C	-4.05656	1.03603	0.50200
C	-4.11328	0.73338	-1.91700
C	-5.11490	0.22313	0.68884
C	-5.24850	-0.20538	-1.74177
C	-5.72579	-0.43295	-0.50439
C	3.94537	0.94855	0.93898

C	4.96274	1.01848	0.06956
C	3.71669	-0.29054	1.71076
C	5.84239	-0.15591	-0.15627
C	4.51813	-1.35737	1.54424
C	5.61795	-1.28461	0.54366
H	-0.72800	-2.15879	1.29957
C	-2.40705	-2.72123	2.33832
C	-1.97325	-3.13959	-0.02519
C	-1.94123	-2.19169	3.55625
C	-3.70216	-3.28492	2.24700
C	-3.32602	-3.61510	-0.14321
C	-2.75248	-2.26074	4.67615
H	-0.95937	-1.73075	3.59411
C	-4.49183	-3.36284	3.41282
C	-4.13328	-3.71275	0.94960
H	-3.65810	-3.95266	-1.11431
C	-4.02617	-2.85811	4.61371
H	-2.39660	-1.85115	5.61652
H	-5.47815	-3.81395	3.34933
H	-5.13109	-4.13047	0.84420
H	-4.64230	-2.91487	5.50495
C	-1.02341	-3.07035	-1.07901
H	-0.72633	-1.45217	-1.20864
C	0.38883	-3.59308	-0.82419
H	0.37130	-4.68065	-0.95064
H	0.64624	-3.39881	0.22822
N	1.38292	-3.04808	-1.73203
N	-1.60460	-2.69765	1.21932
C	2.65320	-3.61130	-1.85236
C	2.89713	-4.98583	-1.66089
C	3.74214	-2.79215	-2.21297
C	4.17696	-5.50984	-1.83134
H	2.08982	-5.65363	-1.38333
C	5.01318	-3.33044	-2.38597
H	3.57818	-1.72555	-2.34720
C	5.24729	-4.69354	-2.19441
H	4.33340	-6.57383	-1.67459
H	5.83627	-2.67947	-2.66408
H	6.24271	-5.10688	-2.32142
H	1.37415	-2.03732	-1.78307
Cl	-1.62795	-3.58725	-2.69589
H	-6.56818	-1.09890	-0.38484
H	6.25287	-2.14873	0.39462
H	5.13056	1.93215	-0.48740
H	2.90767	-0.31492	2.43102
H	-3.58514	1.51715	1.35019
H	-3.74659	0.94422	-2.91401
C	6.99005	-0.07599	-1.14532
H	7.46444	-1.07736	-1.25420
C	4.32143	-2.60097	2.39123
H	3.47774	-2.44646	3.10144
C	5.56788	-2.89147	3.23494
H	5.37266	-3.74385	3.92019
H	6.43687	-3.14830	2.59279
H	5.82575	-2.00363	3.85118
C	3.95583	-3.80537	1.52108
H	3.02707	-3.58708	0.95619
H	4.77038	-4.04601	0.80616
H	3.76900	-4.69574	2.15835
C	6.50209	0.34592	-2.53804

H	5.65351	-0.29087	-2.86241
H	6.17076	1.40566	-2.54560
H	7.32434	0.23276	-3.27636
C	8.07004	0.88362	-0.63511
H	7.67448	1.91806	-0.54615
H	8.43298	0.55205	0.36136
H	8.93367	0.89192	-1.33391
C	-5.64923	0.01484	2.10457
H	-4.79935	-0.31494	2.74302
C	-6.73580	-1.06669	2.21138
H	-7.66575	-0.74672	1.69392
H	-6.97726	-1.25784	3.27893
H	-6.38463	-2.02041	1.76949
C	-6.19358	1.33238	2.67821
H	-7.01437	1.72396	2.03936
H	-6.58440	1.17326	3.70611
H	-5.39348	2.09890	2.74155
C	-5.88366	-0.87432	-2.94721
H	-6.71927	-1.53354	-2.62041
C	-6.48247	0.16765	-3.89897
H	-5.69201	0.81161	-4.33932
H	-7.02409	-0.33979	-4.72562
H	-7.20647	0.80978	-3.35307
C	-4.87157	-1.76515	-3.67515
H	-5.36737	-2.29840	-4.51411
H	-4.02942	-1.16791	-4.08439
H	-4.46685	-2.52488	-2.97515

Catalyst (3,5-(i-Pr)2, 7)-Enamine protonation TS (pro-R) alternative conformation
Electronic energy (Hartrees): -1870.550476
Free Energy at 1M concentration and 253 K (Hartrees): -1869.502301
Coordinates:

P	0.08469	0.16190	0.08458
O	1.43566	0.93586	-0.39764
O	-0.94361	1.28764	0.66446
O	0.29048	-0.87211	1.13786
O	-0.47997	-0.32925	-1.29735
C	1.83768	1.98228	0.40063
C	3.17267	1.91945	1.05223
C	3.62401	2.96759	1.75769
H	4.59688	2.92518	2.23451
C	2.78677	4.16959	1.93382
C	3.26744	5.27992	2.78875
H	4.24500	5.22635	3.25166
C	2.49564	6.35221	2.99509
H	2.84702	7.16231	3.62101
C	1.15675	6.42832	2.38092
H	0.52805	7.28896	2.56968
C	0.71543	5.43280	1.60343
H	-0.27867	5.50082	1.18235
C	1.57480	4.24579	1.33873
C	1.11232	3.12743	0.46122
C	-1.12314	2.35300	-0.18180
C	-2.41464	2.50602	-0.90416
C	-2.57202	3.53341	-1.75691
H	-3.51177	3.67293	-2.27877
C	-1.47044	4.48463	-2.00577
C	-1.65045	5.56954	-2.99859
H	-2.59826	5.68851	-3.50879

C	-0.64302	6.40503	-3.27196
H	-0.77507	7.19614	-3.99874
C	0.65689	6.24150	-2.59478
H	1.47629	6.90513	-2.83927
C	0.82812	5.26838	-1.69259
H	1.79877	5.15271	-1.23010
C	-0.29428	4.35105	-1.35100
C	-0.13348	3.25919	-0.34364
C	-3.52237	1.54189	-0.68378
C	-3.93316	1.18399	0.69474
C	-4.18784	1.01351	-1.72419
C	-4.99350	0.38435	0.92288
C	-5.34428	0.10983	-1.50759
C	-5.72558	-0.17691	-0.24971
C	4.00944	0.71122	0.92728
C	5.16841	0.75335	0.25152
C	3.60481	-0.53553	1.61307
C	6.03603	-0.44643	0.16475
C	4.38580	-1.63156	1.56038
C	5.64637	-1.58186	0.77737
H	-0.75922	-2.26089	1.09877
C	-2.36861	-2.91608	2.18567
C	-2.09660	-3.10561	-0.22905
C	-1.81427	-2.51080	3.41443
C	-3.68053	-3.44356	2.12589
C	-3.46046	-3.55635	-0.30342
C	-2.55791	-2.66419	4.57193
H	-0.82080	-2.07428	3.43053
C	-4.40140	-3.60783	3.32717
C	-4.20025	-3.74304	0.82558
H	-3.86032	-3.79451	-1.27843
C	-3.85006	-3.22391	4.53612
H	-2.13501	-2.34969	5.52109
H	-5.40324	-4.02610	3.28464
H	-5.21215	-4.13176	0.74666
H	-4.41296	-3.34575	5.45562
C	-1.22270	-2.92022	-1.33557
H	-0.90535	-1.31688	-1.29716
C	0.20458	-3.45464	-1.24982
H	0.19455	-4.52343	-1.50520
H	0.53349	-3.38138	-0.20233
N	1.10845	-2.78377	-2.15833
N	-1.63820	-2.79870	1.02437
C	2.39642	-3.22688	-2.41511
C	2.89387	-4.45798	-1.94606
C	3.24481	-2.42813	-3.21138
C	4.18812	-4.86636	-2.27079
H	2.28037	-5.09526	-1.31913
C	4.52667	-2.85268	-3.53289
H	2.87498	-1.47637	-3.58491
C	5.01697	-4.07563	-3.06367
H	4.54822	-5.81841	-1.88963
H	5.14949	-2.22246	-4.16199
H	6.02255	-4.40018	-3.31146
H	0.95463	-1.79182	-2.27251
Cl	-1.94860	-3.25520	-2.95205
H	-6.58132	-0.81881	-0.09746
H	6.26573	-2.46801	0.72376
H	5.47602	1.67202	-0.23451
H	2.69361	-0.53796	2.19633

H	-3.38356	1.60362	1.52811
H	-3.88663	1.25492	-2.73596
C	7.34829	-0.36264	-0.59252
H	7.47942	0.67342	-0.97642
C	4.03237	-2.89553	2.32246
H	4.81597	-3.66253	2.13381
C	2.70447	-3.48782	1.84224
H	2.53129	-4.47455	2.32199
H	1.85311	-2.82161	2.09250
H	2.73893	-3.63717	0.74486
C	4.00716	-2.64368	3.83498
H	4.97768	-2.21556	4.16556
H	3.19182	-1.94173	4.11123
H	3.84721	-3.60028	4.37675
C	8.54367	-0.65232	0.32408
H	8.51790	0.02092	1.20759
H	8.53607	-1.70626	0.67407
H	9.49367	-0.47067	-0.22245
C	7.34681	-1.29218	-1.81012
H	7.24303	-2.35530	-1.50573
H	6.50587	-1.02270	-2.48195
H	8.29340	-1.17714	-2.37996
C	-5.40342	0.08941	2.36436
H	-4.50574	-0.29216	2.90102
C	-6.49209	-0.98673	2.49827
H	-6.19894	-1.90910	1.95830
H	-6.63442	-1.25139	3.56794
H	-7.46172	-0.62233	2.09584
C	-5.87243	1.37201	3.06825
H	-6.74135	1.81252	2.53339
H	-6.17107	1.15006	4.11524
H	-5.05757	2.12456	3.10649
C	-6.10445	-0.46070	-2.69119
H	-6.94268	-1.09998	-2.33295
C	-6.72562	0.65894	-3.53425
H	-5.94229	1.29099	-4.00363
H	-7.35550	0.22353	-4.33916
H	-7.37163	1.30071	-2.89761
C	-5.19755	-1.35252	-3.54535
H	-5.78271	-1.81283	-4.36981
H	-4.36065	-0.77148	-3.98732
H	-4.77894	-2.16949	-2.92216

Catalyst (3,5-(i-Pr)2, 7)-Enamine protonation TS (pro-R) alternative conformation
Electronic energy (Hartrees): -1870.550168
Free Energy at 1M concentration and 253 K (Hartrees): -1869.501766
Coordinates:

P	0.09632	0.18888	0.06661
O	1.48596	0.89158	-0.41542
O	-0.87443	1.36751	0.63819
O	0.24650	-0.85150	1.12230
O	-0.49141	-0.27709	-1.31573
C	1.93883	1.92018	0.37919
C	3.26892	1.79439	1.03276
C	3.76999	2.82224	1.73484
H	4.73866	2.73407	2.21401
C	2.99251	4.06419	1.90605
C	3.52585	5.15222	2.75845
H	4.49932	5.05257	3.22234

C	2.80680	6.26119	2.96131
H	3.19657	7.05499	3.58540
C	1.47355	6.40033	2.34597
H	0.88700	7.29077	2.53216
C	0.98513	5.42526	1.57072
H	-0.00439	5.54007	1.14904
C	1.78637	4.19743	1.30954
C	1.27109	3.10050	0.43438
C	-0.99822	2.43670	-0.21323
C	-2.27830	2.65072	-0.94094
C	-2.38421	3.68610	-1.79195
H	-3.31390	3.86969	-2.31805
C	-1.23739	4.58337	-2.03702
C	-1.36252	5.67689	-3.02886
H	-2.30287	5.84335	-3.53957
C	-0.31460	6.46150	-3.30076
H	-0.40664	7.25891	-4.02683
C	0.97518	6.23266	-2.62317
H	1.82682	6.85477	-2.86668
C	1.09719	5.25131	-1.72189
H	2.06068	5.08676	-1.25926
C	-0.06979	4.39086	-1.38163
C	0.03539	3.29236	-0.37408
C	-3.42675	1.73201	-0.73401
C	-3.85390	1.37278	0.63960
C	-4.10654	1.23777	-1.78245
C	-4.93492	0.59798	0.85569
C	-5.28371	0.36208	-1.57983
C	-5.67397	0.06553	-0.32537
C	4.04626	0.54560	0.91733
C	5.21869	0.53203	0.26354
C	3.57355	-0.67856	1.60048
C	6.03454	-0.70503	0.20069
C	4.30658	-1.80809	1.56915
C	5.58336	-1.81843	0.81139
H	-0.83830	-2.21697	1.07046
C	-2.46708	-2.82937	2.15223
C	-2.19609	-3.01867	-0.26303
C	-1.90467	-2.44193	3.38300
C	-3.79359	-3.31846	2.08862
C	-3.57060	-3.43641	-0.33982
C	-2.65632	-2.57321	4.53819
H	-0.89875	-2.03510	3.40263
C	-4.52338	-3.46018	3.28722
C	-4.31755	-3.60490	0.78687
H	-3.97319	-3.66690	-1.31536
C	-3.96478	-3.09298	4.49817
H	-2.22700	-2.27191	5.48876
H	-5.53757	-3.84712	3.24084
H	-5.33853	-3.96852	0.70514
H	-4.53430	-3.19722	5.41577
C	-1.31942	-2.85042	-1.36888
H	-0.95017	-1.24429	-1.31760
C	0.09385	-3.42067	-1.29137
H	0.05681	-4.48677	-1.55586
H	0.42634	-3.36505	-0.24388
N	1.01293	-2.76464	-2.19570
N	-1.73079	-2.73040	0.99268
C	2.29176	-3.23239	-2.45346
C	2.76317	-4.47710	-1.99379

C	3.15755	-2.44415	-3.24144
C	4.04973	-4.90853	-2.31951
H	2.13491	-5.10776	-1.37479
C	4.43120	-2.89142	-3.56406
H	2.80766	-1.48191	-3.60720
C	4.89582	-4.12798	-3.10423
H	4.38997	-5.87078	-1.94582
H	5.06822	-2.26762	-4.18541
H	5.89530	-4.47049	-3.35254
H	0.88055	-1.76914	-2.30510
Cl	-2.05804	-3.14902	-2.98663
H	-6.54073	-0.56229	-0.18366
H	6.16549	-2.73041	0.77718
H	5.57793	1.43423	-0.21789
H	2.65342	-0.63695	2.16775
H	-3.29804	1.77072	1.47938
H	-3.79577	1.47875	-2.79217
C	7.36785	-0.68141	-0.52360
H	7.54976	0.34440	-0.91423
C	3.88501	-3.04960	2.33366
H	4.63978	-3.85005	2.16788
C	2.54410	-3.59072	1.83008
H	2.31687	-4.56192	2.31887
H	1.71721	-2.88460	2.05105
H	2.59761	-3.75781	0.73596
C	3.83871	-2.78383	3.84335
H	4.81848	-2.39169	4.19084
H	3.04636	-2.04790	4.09710
H	3.62970	-3.72863	4.38912
C	8.52669	-1.00779	0.42697
H	8.50537	-0.32367	1.30221
H	8.46744	-2.05636	0.78803
H	9.49700	-0.87078	-0.09639
C	7.36173	-1.62362	-1.73144
H	7.20761	-2.67839	-1.41954
H	6.55093	-1.32851	-2.42866
H	8.32705	-1.55249	-2.27648
C	-5.36007	0.30061	2.29232
H	-4.47625	-0.11143	2.82944
C	-6.47971	-0.74522	2.41040
H	-6.20855	-1.67174	1.86629
H	-6.63777	-1.01328	3.47701
H	-7.43548	-0.35075	2.00313
C	-5.79652	1.58899	3.00663
H	-6.64968	2.05919	2.47165
H	-6.10711	1.36532	4.04976
H	-4.96102	2.31780	3.05689
C	-6.03297	-0.16653	-2.78952
H	-5.55324	0.22595	-3.71351
C	-5.95843	-1.69561	-2.86442
H	-6.44925	-2.16723	-1.98673
H	-6.45979	-2.05717	-3.78740
H	-4.89703	-2.01733	-2.90088
C	-7.48812	0.31855	-2.80344
H	-7.97925	0.01731	-3.75329
H	-8.06469	-0.11310	-1.95815
H	-7.52053	1.42690	-2.73281

Catalyst (3,5-(i-Pr)2, 7)-Enamine protonation TS (pro-R) alternative conformation

Electronic energy (Hartrees): -1870.549879
Free Energy at 1M concentration and 253 K (Hartrees): -1869.501527
Coordinates:

P	0.12704	0.25048	0.05820
O	1.38204	1.13306	-0.47920
O	-1.02527	1.30594	0.53371
O	0.42353	-0.67249	1.18761
O	-0.36290	-0.40201	-1.29428
C	1.71421	2.18295	0.34417
C	2.98445	2.13200	1.10532
C	3.32585	3.14482	1.91286
H	4.25153	3.10447	2.47566
C	2.43293	4.31291	2.06147
C	2.79723	5.39951	2.99999
H	3.73080	5.35561	3.54705
C	1.97412	6.43821	3.17821
H	2.24066	7.23089	3.86532
C	0.69361	6.50085	2.44854
H	0.02245	7.33385	2.61365
C	0.35657	5.52703	1.59505
H	-0.59724	5.58412	1.08818
C	1.27472	4.37860	1.36243
C	0.92444	3.27917	0.41188
C	-1.22519	2.35883	-0.32743
C	-2.48034	2.42124	-1.12025
C	-2.66696	3.43340	-1.98375
H	-3.58251	3.49771	-2.56059
C	-1.63201	4.47111	-2.16428
C	-1.83935	5.54584	-3.16277
H	-2.76068	5.59108	-3.73013
C	-0.88915	6.46326	-3.37058
H	-1.04100	7.24695	-4.10152
C	0.37537	6.40183	-2.61402
H	1.15002	7.13361	-2.80381
C	0.57058	5.43845	-1.70624
H	1.51596	5.40087	-1.18251
C	-0.49039	4.42803	-1.43937
C	-0.30045	3.34236	-0.43044
C	-3.52110	1.37973	-0.95671
C	-4.04235	1.06305	0.39318
C	-4.02785	0.74420	-2.02521
C	-5.05820	0.19421	0.56233
C	-5.10695	-0.25799	-1.86802
C	-5.59806	-0.50768	-0.63857
C	3.85430	0.95414	0.98537
C	4.73796	0.86561	-0.01763
C	3.71613	-0.15592	1.95002
C	5.55441	-0.36225	-0.19517
C	4.45752	-1.26998	1.82068
C	5.40283	-1.37902	0.67564
H	-0.59770	-2.13484	1.34786
C	-2.27471	-2.70639	2.37907
C	-1.78377	-3.20252	0.03653
C	-1.84807	-2.12043	3.58462
C	-3.55690	-3.29720	2.28396
C	-3.12732	-3.71524	-0.08407
C	-2.68404	-2.15785	4.68916
H	-0.87587	-1.63970	3.62744
C	-4.37223	-3.34026	3.43198
C	-3.95206	-3.78726	0.99439

H	-3.43524	-4.09936	-1.04575
C	-3.94484	-2.77838	4.62310
H	-2.35599	-1.70360	5.61917
H	-5.34952	-3.81019	3.36353
H	-4.93867	-4.23008	0.88606
H	-4.58164	-2.80928	5.50099
C	-0.83512	-3.15870	-1.00240
H	-0.66228	-1.38074	-1.19453
C	0.62210	-3.50288	-0.74428
H	0.73435	-4.59718	-0.80256
H	0.87029	-3.21298	0.28602
N	1.53314	-2.89681	-1.69043
N	-1.44628	-2.71127	1.27726
C	2.79134	-3.38662	-1.97686
C	3.39249	-4.41071	-1.21890
C	3.51756	-2.85147	-3.06145
C	4.67571	-4.85987	-1.52977
H	2.86587	-4.84925	-0.37945
C	4.79485	-3.30994	-3.35809
H	3.06439	-2.06978	-3.66655
C	5.39328	-4.31562	-2.59311
H	5.11741	-5.64448	-0.92103
H	5.33023	-2.87464	-4.19781
H	6.39386	-4.66603	-2.82486
H	1.18580	-2.13845	-2.25488
Cl	-1.38640	-3.76399	-2.60510
H	-6.39379	-1.22941	-0.53199
H	5.99596	-2.27812	0.56535
H	4.84129	1.68883	-0.71337
H	3.01570	-0.05037	2.77012
H	-3.61726	1.57526	1.24771
H	-3.64719	0.96364	-3.01588
C	6.57615	-0.45466	-1.31341
H	6.91466	-1.51000	-1.42257
C	4.35457	-2.37886	2.85125
H	3.61408	-2.10038	3.63493
C	5.69681	-2.59224	3.56021
H	5.58267	-3.34127	4.37270
H	6.47163	-2.95568	2.85231
H	6.04321	-1.63892	4.01377
C	3.85937	-3.67851	2.21305
H	2.88509	-3.50285	1.71110
H	4.59044	-4.06206	1.47086
H	3.71039	-4.45414	2.99415
C	5.98595	-0.02972	-2.66552
H	4.99517	-0.50048	-2.82220
H	5.86269	1.07226	-2.72495
H	6.66095	-0.34759	-3.48855
C	7.80814	0.38933	-0.97351
H	7.53657	1.46199	-0.86985
H	8.25650	0.04008	-0.01870
H	8.57253	0.28906	-1.77347
C	-5.61505	-0.03265	1.96653
H	-4.76493	-0.31329	2.62785
C	-6.64610	-1.16870	2.05546
H	-7.57536	-0.90443	1.50624
H	-6.90957	-1.36009	3.11774
H	-6.23161	-2.10744	1.63726
C	-6.24187	1.25816	2.51640
H	-7.06632	1.60149	1.85489

H	-6.64873	1.08563	3.53587
H	-5.48510	2.06632	2.59292
C	-5.63975	-0.97366	-3.09572
H	-5.11024	-0.59162	-3.99661
C	-5.36314	-2.47962	-3.02193
H	-5.89368	-2.94545	-2.16446
H	-5.69873	-2.97526	-3.95771
H	-4.27276	-2.65583	-2.91025
C	-7.13326	-0.69355	-3.30499
H	-7.46947	-1.13069	-4.26937
H	-7.74402	-1.13368	-2.48860
H	-7.31379	0.40220	-3.33934

Catalyst (3,5-(i-Pr)2, 7)-Enamine protonation TS (pro-R) alternative conformation
Electronic energy (Hartrees): -1870.550584
Free Energy at 1M concentration and 253 K (Hartrees): -1869.501346
Coordinates:

P	0.13676	0.18827	0.08202
O	1.45520	1.00076	-0.42236
O	-0.96771	1.29623	0.54982
O	0.36295	-0.75600	1.21348
O	-0.33835	-0.43361	-1.28116
C	1.82160	2.05235	0.38385
C	3.12739	2.00096	1.08387
C	3.52902	3.03509	1.83596
H	4.48704	3.00105	2.34229
C	2.66106	4.21900	1.99999
C	3.08290	5.32086	2.89561
H	4.03731	5.27276	3.40507
C	2.28624	6.37887	3.08104
H	2.59471	7.18287	3.73673
C	0.97855	6.44827	2.40161
H	0.33006	7.29802	2.57175
C	0.58995	5.46054	1.58685
H	-0.38160	5.52312	1.11514
C	1.47701	4.28944	1.34771
C	1.07118	3.17779	0.43477
C	-1.11361	2.35186	-0.31722
C	-2.36455	2.47613	-1.11084
C	-2.49767	3.49419	-1.97770
H	-3.41001	3.60770	-2.55204
C	-1.40937	4.47396	-2.16402
C	-1.55961	5.55220	-3.16897
H	-2.47756	5.64326	-3.73631
C	-0.56193	6.41620	-3.38263
H	-0.67206	7.20243	-4.11830
C	0.69813	6.29116	-2.62670
H	1.51144	6.97804	-2.82249
C	0.84185	5.32441	-1.71293
H	1.78554	5.23804	-1.19214
C	-0.27228	4.37480	-1.43796
C	-0.14126	3.28713	-0.42111
C	-3.46582	1.49942	-0.93722
C	-3.99402	1.21548	0.41741
C	-4.03264	0.90985	-2.00230
C	-5.07388	0.42916	0.59427
C	-5.18739	-0.00270	-1.83756
C	-5.68159	-0.22080	-0.60343
C	3.99452	0.82493	0.92079

C	5.01614	0.85450	0.05404
C	3.72202	-0.40048	1.70029
C	5.85721	-0.34983	-0.16022
C	4.48862	-1.49433	1.54451
C	5.59373	-1.46509	0.54754
H	-0.79543	-2.09543	1.31750
C	-2.50038	-2.58300	2.35013
C	-2.05768	-3.06214	-0.00172
C	-2.03109	-2.04147	3.56126
C	-3.81136	-3.10830	2.25785
C	-3.42288	-3.50366	-0.12034
C	-2.85600	-2.05905	4.67354
H	-1.03550	-1.61101	3.60044
C	-4.61558	-3.13379	3.41553
C	-4.24203	-3.55459	0.96583
H	-3.75477	-3.85690	-1.08571
C	-4.14725	-2.61637	4.61035
H	-2.49682	-1.63948	5.60827
H	-5.61535	-3.55409	3.35042
H	-5.24955	-3.94820	0.85993
H	-4.77464	-2.63257	5.49539
C	-1.10298	-3.04252	-1.04949
H	-0.75330	-1.40536	-1.20819
C	0.29906	-3.58304	-0.78039
H	0.26123	-4.67329	-0.87322
H	0.56097	-3.36201	0.26547
N	1.30469	-3.08698	-1.70440
N	-1.68625	-2.60834	1.23921
C	2.55256	-3.69916	-1.82794
C	2.74038	-5.08431	-1.65092
C	3.67424	-2.92108	-2.17814
C	3.99860	-5.65750	-1.82248
H	1.90531	-5.72242	-1.38590
C	4.92326	-3.50834	-2.35417
H	3.55383	-1.84727	-2.30110
C	5.10199	-4.88122	-2.17474
H	4.11161	-6.72854	-1.67685
H	5.77256	-2.88833	-2.62395
H	6.08035	-5.33301	-2.30336
H	1.33669	-2.07706	-1.76303
Cl	-1.71181	-3.57025	-2.65998
H	-6.53310	-0.87481	-0.49124
H	6.20087	-2.35044	0.40766
H	5.21702	1.75853	-0.50770
H	2.91067	-0.39269	2.41828
H	-3.52225	1.68963	1.26931
H	-3.65078	1.10756	-2.99705
C	7.01071	-0.31471	-1.14509
H	7.45260	-1.33177	-1.24472
C	4.24991	-2.72390	2.40124
H	3.40852	-2.53710	3.10638
C	5.48336	-3.04516	3.25288
H	5.25918	-3.88499	3.94473
H	6.34660	-3.33430	2.61675
H	5.76599	-2.16025	3.86245
C	3.85096	-3.92444	1.54028
H	2.93024	-3.68386	0.97157
H	4.65936	-4.19532	0.82918
H	3.63646	-4.80345	2.18460
C	6.54222	0.11263	-2.54284

H	5.67529	-0.49922	-2.86667
H	6.24472	1.18226	-2.55916
H	7.36357	-0.03171	-3.27670
C	8.11949	0.61287	-0.63742
H	7.75771	1.66036	-0.55782
H	8.46739	0.27714	0.36301
H	8.98573	0.58764	-1.33258
C	-5.63504	0.24432	2.00297
H	-4.80487	-0.10633	2.65595
C	-6.75501	-0.80375	2.09867
H	-6.42384	-1.77181	1.67310
H	-7.02267	-0.97723	3.16297
H	-7.66484	-0.46173	1.55992
C	-6.14953	1.58033	2.56153
H	-6.94827	1.99237	1.90775
H	-6.56110	1.43825	3.58382
H	-5.32854	2.32352	2.63428
C	-5.79964	-0.65390	-3.06435
H	-5.25357	-0.30732	-3.96969
C	-5.65427	-2.17885	-3.01376
H	-6.20103	-2.60773	-2.14728
H	-6.05579	-2.63143	-3.94539
H	-4.58104	-2.45031	-2.93433
C	-7.26633	-0.24249	-3.24494
H	-7.65425	-0.63486	-4.20916
H	-7.90020	-0.63984	-2.42424
H	-7.35160	0.86517	-3.26203

Catalyst (3,5-(i-Pr)2, 7)-Product (R) lowest free energy conformation
Electronic energy (Hartrees): -1870.595816
Free Energy at 1M concentration and 253 K (Hartrees): -1869.546102
Coordinates:

P	0.00197	0.34045	0.30066
O	-0.74669	1.65282	0.89107
O	1.44203	0.84369	-0.30757
O	-0.75496	-0.03780	-1.03124
O	0.07751	-0.69473	1.35734
C	-0.72807	2.75676	0.07740
C	-2.00449	3.23628	-0.52466
C	-1.98914	4.32319	-1.31345
H	-2.90778	4.69560	-1.75146
C	-0.72324	5.02338	-1.61012
C	-0.72687	6.17792	-2.53870
H	-1.65837	6.53295	-2.96186
C	0.41965	6.78521	-2.86150
H	0.41589	7.62735	-3.54141
C	1.69557	6.29866	-2.30471
H	2.62313	6.77723	-2.59141
C	1.70938	5.26045	-1.46094
H	2.66077	4.90477	-1.08983
C	0.43763	4.59592	-1.06181
C	0.42322	3.44042	-0.11367
C	2.12119	1.75054	0.46784
C	3.47015	1.38967	0.98162
C	4.19221	2.29955	1.65254
H	5.17910	2.04678	2.02352
C	3.63634	3.63762	1.93558
C	4.40836	4.59298	2.76366
H	5.39148	4.32463	3.12999
C	3.89392	5.78997	3.06499

H	4.45807	6.48670	3.67141
C	2.54969	6.15962	2.58287
H	2.12998	7.12123	2.84874
C	1.84640	5.30583	1.83010
H	0.85373	5.58921	1.50663
C	2.41759	3.98178	1.46000
C	1.65991	3.01753	0.60606
C	4.03769	0.04883	0.72578
C	5.30533	-0.08295	-0.02747
C	3.43566	-1.04996	1.20567
C	5.85206	-1.29231	-0.24594
C	4.02147	-2.39668	0.98349
C	5.16403	-2.50203	0.28009
C	-3.27853	2.50855	-0.28326
C	-3.61400	2.09499	0.95154
C	-4.19952	2.22788	-1.41297
C	-4.86113	1.32424	1.18641
C	-5.35095	1.56109	-1.21593
C	-5.68118	1.07730	0.14985
H	-0.66676	-1.02297	-1.33072
C	0.84280	-2.74540	-2.36604
C	-1.24702	-3.57531	-1.75243
C	1.74885	-1.65555	-2.44408
C	1.27666	-4.03394	-2.81087
C	-0.88204	-4.88231	-2.17107
C	3.02471	-1.84874	-2.92502
H	1.43461	-0.67504	-2.11370
C	2.59326	-4.19471	-3.31186
C	0.36314	-5.10802	-2.69649
H	-1.60046	-5.68797	-2.06942
C	3.45361	-3.12326	-3.36503
H	3.71071	-1.00829	-2.96496
H	2.90946	-5.17963	-3.64379
H	0.66207	-6.10231	-3.01693
H	4.46048	-3.25480	-3.74715
C	-2.65523	-3.39133	-1.22184
H	-3.01131	-4.36404	-0.88008
C	-2.86892	-2.36281	-0.10117
H	-3.93527	-2.36216	0.15335
H	-2.63471	-1.36120	-0.46351
N	-2.04279	-2.67152	1.03717
N	-0.41536	-2.54694	-1.84905
C	-2.42949	-3.46450	2.10460
C	-3.60609	-4.23755	2.10820
C	-1.59677	-3.50703	3.24421
C	-3.93405	-5.01594	3.21949
H	-4.27272	-4.23168	1.25146
C	-1.93655	-4.28963	4.33954
H	-0.69054	-2.90729	3.25134
C	-3.10907	-5.05351	4.34192
H	-4.85048	-5.60015	3.19931
H	-1.27957	-4.29996	5.20533
H	-3.37091	-5.66301	5.20097
H	-1.25974	-2.04467	1.20723
Cl	-3.75500	-2.98695	-2.64740
H	5.78745	0.81340	-0.39924
H	2.53681	-0.95283	1.79597
H	-2.97693	2.32961	1.79401
H	-3.93087	2.56594	-2.40646
H	5.60350	-3.47574	0.10529

H	-6.59409	0.51626	0.30392
C	-5.19803	0.81063	2.57407
H	-6.16981	0.26759	2.55194
C	-6.29958	1.30892	-2.37303
H	-5.89127	1.76273	-3.30426
C	3.35754	-3.63288	1.56473
H	3.97955	-4.53143	1.35156
C	7.16520	-1.41500	-0.99647
H	7.53885	-0.40362	-1.27464
C	8.24160	-2.06117	-0.11653
H	7.98491	-3.11424	0.12517
H	9.21975	-2.04992	-0.64313
H	8.35171	-1.49102	0.83075
C	6.97970	-2.19628	-2.30079
H	6.63176	-3.23233	-2.10313
H	6.23718	-1.68062	-2.94533
H	7.94058	-2.24538	-2.85609
C	1.98971	-3.87472	0.92093
H	1.52227	-4.79034	1.34177
H	1.30597	-3.01836	1.09117
H	2.11726	-4.02541	-0.16881
C	3.23369	-3.52899	3.08959
H	2.85779	-4.48812	3.50557
H	4.22811	-3.32200	3.53981
H	2.52945	-2.72189	3.38278
C	-5.35577	1.96878	3.56575
H	-6.11463	2.68912	3.19199
H	-5.69962	1.58218	4.54887
H	-4.39459	2.50425	3.71587
C	-4.13907	-0.18689	3.05728
H	-3.14190	0.29409	3.14721
H	-4.42516	-0.59859	4.04856
H	-4.06657	-1.03186	2.34253
C	-6.45287	-0.19200	-2.63719
H	-7.09795	-0.35893	-3.52607
H	-5.45838	-0.63839	-2.84362
H	-6.90797	-0.71069	-1.76701
C	-7.66166	1.96416	-2.11725
H	-8.31129	1.85170	-3.01142
H	-8.17469	1.49928	-1.24885
H	-7.53041	3.04944	-1.91855

Catalyst (3,5-(i-Pr)2, 7)-Product (R) alternative conformation
Electronic energy (Hartrees): -1870.5954
Free Energy at 1M concentration and 253 K (Hartrees): -1869.545982
Coordinates:

P	0.02202	0.29367	0.28483
O	-0.57784	1.66756	0.90486
O	1.51234	0.64693	-0.30613
O	-0.76521	0.03620	-1.05915
O	-0.02540	-0.77036	1.31400
C	-0.43604	2.77900	0.11368
C	-1.65018	3.40525	-0.48196
C	-1.51337	4.49571	-1.25387
H	-2.38436	4.97289	-1.68749
C	-0.17727	5.05737	-1.53767
C	-0.05126	6.21884	-2.44896
H	-0.93685	6.68027	-2.86806
C	1.15600	6.70104	-2.76132
H	1.24680	7.54830	-3.42871

C	2.36909	6.06904	-2.21025
H	3.34446	6.44667	-2.48895
C	2.26627	5.02351	-1.38174
H	3.17178	4.55990	-1.01501
C	0.92800	4.49704	-0.99399
C	0.78395	3.33541	-0.06429
C	2.28232	1.45801	0.48970
C	3.57851	0.93917	1.00241
C	4.39682	1.75114	1.68801
H	5.34728	1.38228	2.05700
C	3.99498	3.13933	1.98992
C	4.86797	3.98938	2.83245
H	5.81387	3.60667	3.19514
C	4.49110	5.23223	3.15114
H	5.12899	5.85205	3.76793
C	3.19803	5.75798	2.67408
H	2.88878	6.75674	2.95414
C	2.40459	5.00005	1.90836
H	1.45084	5.39824	1.58879
C	2.82375	3.62574	1.51863
C	1.96475	2.76607	0.64863
C	3.98930	-0.45382	0.72966
C	5.22040	-0.71872	-0.04805
C	3.28066	-1.48268	1.21927
C	5.62460	-1.98112	-0.28600
C	3.71358	-2.88408	0.98246
C	4.82081	-3.10944	0.25039
C	-2.99673	2.81813	-0.25252
C	-3.37852	2.42693	0.97609
C	-3.93840	2.65214	-1.38771
C	-4.70297	1.79392	1.19884
C	-5.15536	2.11019	-1.20158
C	-5.54171	1.65084	0.15781
H	-0.77731	-0.94532	-1.38067
C	0.58824	-2.82149	-2.34777
C	-1.59088	-3.43433	-1.79474
C	1.58739	-1.81778	-2.43728
C	0.92409	-4.16258	-2.71509
C	-1.33047	-4.78471	-2.14738
C	2.86181	-2.14373	-2.84272
H	1.34409	-0.79822	-2.17194
C	2.24503	-4.46202	-3.13536
C	-0.08797	-5.14435	-2.60133
H	-2.12282	-5.51813	-2.04780
C	3.19911	-3.47313	-3.19138
H	3.61702	-1.36574	-2.89128
H	2.48914	-5.48655	-3.40171
H	0.13297	-6.17508	-2.86486
H	4.21022	-3.71218	-3.50427
C	-2.99326	-3.09607	-1.32944
H	-3.47409	-4.02448	-1.01918
C	-3.13359	-2.06119	-0.20120
H	-4.20156	-1.94701	0.01917
H	-2.78045	-1.08750	-0.54236
N	-2.38441	-2.46574	0.95977
N	-0.66504	-2.49004	-1.89365
C	-2.87836	-3.24588	1.99102
C	-4.12850	-3.89192	1.93938
C	-2.08826	-3.40795	3.15006
C	-4.56708	-4.66490	3.01554

H	-4.76537	-3.78914	1.06647
C	-2.53873	-4.18348	4.21001
H	-1.12579	-2.90596	3.20066
C	-3.78308	-4.82155	4.15695
H	-5.53783	-5.14992	2.95278
H	-1.91198	-4.28790	5.09182
H	-4.13087	-5.42620	4.98849
H	-1.53559	-1.94034	1.15557
Cl	-3.97192	-2.55012	-2.79411
H	5.79604	0.12165	-0.41509
H	2.40892	-1.29097	1.82827
H	-2.72138	2.57849	1.82223
H	-3.63063	2.96998	-2.37651
H	5.15520	-4.12384	0.07031
H	-6.51048	1.19006	0.30296
C	-5.09649	1.29812	2.57795
H	-6.12421	0.87071	2.54841
C	-6.11901	1.96864	-2.36496
H	-5.65826	2.37985	-3.29147
C	2.94753	-4.04285	1.59688
H	3.44252	-5.00492	1.33410
C	6.90940	-2.27150	-1.04028
H	7.03048	-3.37306	-1.13856
C	6.87637	-1.69687	-2.46207
H	6.82073	-0.58797	-2.44822
H	7.79354	-1.99553	-3.01315
H	6.00086	-2.09414	-3.01435
C	8.12879	-1.75355	-0.26793
H	8.11352	-0.64543	-0.19224
H	8.14098	-2.18349	0.75656
H	9.06406	-2.05977	-0.78340
C	1.51833	-4.11388	1.05190
H	0.98978	-4.99482	1.47447
H	0.94251	-3.20206	1.30843
H	1.54605	-4.22675	-0.04863
C	2.94292	-3.94603	3.12687
H	2.47033	-4.85182	3.56333
H	3.98467	-3.88095	3.50761
H	2.37708	-3.05457	3.47144
C	-5.11998	2.44813	3.59118
H	-5.78988	3.25839	3.23179
H	-5.50734	2.08561	4.56727
H	-4.10344	2.86569	3.75052
C	-4.15889	0.17683	3.03952
H	-3.11293	0.53842	3.13487
H	-4.48941	-0.21787	4.02386
H	-4.18505	-0.65698	2.30843
C	-6.43338	0.49443	-2.63797
H	-7.08113	0.40270	-3.53580
H	-5.49183	-0.05920	-2.83291
H	-6.95483	0.02658	-1.77625
C	-7.40311	2.76689	-2.11229
H	-8.05710	2.72855	-3.00953
H	-7.96733	2.35753	-1.24769
H	-7.15580	3.83084	-1.90887

Catalyst (3,5-(i-Pr)2, 7)-Product (R) alternative conformation
Electronic energy (Hartrees): -1870.595464
Free Energy at 1M concentration and 253 K (Hartrees): -1869.545866
Coordinates:

P	-0.04385	0.32445	0.35590
O	-0.81898	1.61113	0.96849
O	1.37210	0.87309	-0.26912
O	-0.81242	-0.06323	-0.96681
O	0.07727	-0.71671	1.40245
C	-0.83154	2.72588	0.16936
C	-2.12089	3.17711	-0.42514
C	-2.13882	4.27590	-1.19677
H	-3.06830	4.62479	-1.63138
C	-0.89445	5.01774	-1.48292
C	-0.93374	6.18729	-2.39162
H	-1.87640	6.52336	-2.80542
C	0.19414	6.83167	-2.70793
H	0.16429	7.68485	-3.37330
C	1.48526	6.37118	-2.16432
H	2.39805	6.87990	-2.44651
C	1.53126	5.31954	-1.33846
H	2.49390	4.98399	-0.97789
C	0.27992	4.61344	-0.94562
C	0.30082	3.44198	-0.01685
C	2.04237	1.78101	0.51300
C	3.40630	1.44205	1.00111
C	4.11576	2.35459	1.68184
H	5.11373	2.11804	2.03344
C	3.53240	3.67296	2.00007
C	4.29246	4.62913	2.83830
H	5.28604	4.37646	3.18725
C	3.75430	5.80746	3.17028
H	4.30972	6.50478	3.78406
C	2.39602	6.15541	2.71179
H	1.95765	7.10148	3.00218
C	1.70335	5.30090	1.94999
H	0.70066	5.56756	1.64359
C	2.30013	3.99825	1.54633
C	1.55416	3.03400	0.68217
C	4.00301	0.12261	0.70534
C	5.25599	0.04054	-0.07869
C	3.44024	-1.00154	1.17385
C	5.82764	-1.14912	-0.33752
C	4.05414	-2.32746	0.90704
C	5.18173	-2.38778	0.17461
C	-3.36884	2.40150	-0.20409
C	-3.70254	1.96442	1.02279
C	-4.25997	2.09028	-1.34839
C	-4.92046	1.14148	1.23458
C	-5.38295	1.36850	-1.17268
C	-5.71316	0.86511	0.18363
H	-0.69924	-1.04047	-1.28120
C	0.84261	-2.68292	-2.39473
C	-1.18532	-3.61615	-1.72433
C	1.69362	-1.55091	-2.49061
C	1.32077	-3.94491	-2.86913
C	-0.77270	-4.90077	-2.16796
C	2.95874	-1.67831	-3.01974
H	1.34583	-0.59012	-2.13674
C	2.62439	-4.03810	-3.41834
C	0.46361	-5.06244	-2.73574
H	-1.44906	-5.74023	-2.05214
C	3.43033	-2.92628	-3.49082
H	3.60287	-0.80610	-3.07426

H	2.97448	-5.00346	-3.77294
H	0.79856	-6.03840	-3.07627
H	4.42680	-3.00593	-3.91242
C	-2.58529	-3.50520	-1.15300
H	-2.87902	-4.49368	-0.79753
C	-2.82354	-2.48530	-0.03052
H	-3.88563	-2.52584	0.23787
H	-2.63267	-1.47503	-0.39453
N	-1.97168	-2.76438	1.09645
N	-0.40588	-2.54881	-1.83440
C	-2.31765	-3.57307	2.16632
C	-3.46476	-4.38924	2.17973
C	-1.47250	-3.58719	3.29738
C	-3.75269	-5.18149	3.29231
H	-4.13979	-4.40651	1.32976
C	-1.77240	-4.38389	4.39424
H	-0.58901	-2.95442	3.29678
C	-2.91581	-5.19065	4.40643
H	-4.64696	-5.79930	3.27969
H	-1.10707	-4.37148	5.25360
H	-3.14637	-5.81107	5.26663
H	-1.21150	-2.10829	1.25965
Cl	-3.74593	-3.16866	-2.54841
H	5.70684	0.95720	-0.44005
H	2.55265	-0.94076	1.78584
H	-3.08453	2.21646	1.87459
H	-3.99302	2.44952	-2.33404
H	5.64111	-3.34577	-0.03286
H	-6.60520	0.26566	0.31888
C	-5.25853	0.60998	2.61521
H	-6.20524	0.02532	2.57505
C	-6.31701	1.06485	-2.32924
H	-7.16955	0.45508	-1.95618
C	3.43662	-3.59252	1.47716
H	4.07086	-4.47118	1.22143
C	7.12656	-1.22072	-1.11888
H	7.47134	-0.19368	-1.37617
C	8.23657	-1.86614	-0.28110
H	8.01015	-2.93172	-0.06548
H	9.20258	-1.81598	-0.82750
H	8.35374	-1.32131	0.68013
C	6.92967	-1.96908	-2.44053
H	6.60472	-3.01658	-2.26517
H	6.16513	-1.44943	-3.05539
H	7.87988	-1.98446	-3.01581
C	2.05380	-3.84964	0.87224
H	1.62312	-4.78841	1.28115
H	1.35574	-3.01607	1.09077
H	2.14742	-3.96554	-0.22504
C	3.36055	-3.52834	3.00726
H	3.01631	-4.50441	3.41109
H	4.36512	-3.31314	3.43026
H	2.65160	-2.74208	3.34277
C	-5.48356	1.75804	3.60569
H	-6.26731	2.44493	3.22049
H	-5.82595	1.35473	4.58259
H	-4.54952	2.33522	3.77198
C	-4.16571	-0.34152	3.11584
H	-3.19273	0.18274	3.22621
H	-4.45208	-0.76857	4.10053

H	-4.04198	-1.18024	2.40085
C	-6.90988	2.35156	-2.91636
H	-6.12114	2.98193	-3.37923
H	-7.66362	2.10245	-3.69353
H	-7.41491	2.93642	-2.11801
C	-5.61518	0.23726	-3.41205
H	-6.34475	-0.06277	-4.19420
H	-4.79800	0.81411	-3.89451
H	-5.18884	-0.68361	-2.96426

Catalyst (3,5-(i-Pr)2, 7)-Product (R) alternative conformation
Electronic energy (Hartrees): -1870.594517
Free Energy at 1M concentration and 253 K (Hartrees): -1869.545392
Coordinates:

P	0.00386	0.36066	0.39184
O	-0.66810	1.70010	1.01310
O	1.45992	0.80135	-0.22674
O	-0.79071	0.04395	-0.93551
O	0.03881	-0.69616	1.42884
C	-0.59371	2.81714	0.22044
C	-1.84340	3.36711	-0.37391
C	-1.77784	4.46555	-1.14283
H	-2.67774	4.88146	-1.58088
C	-0.48029	5.11259	-1.42376
C	-0.42915	6.28453	-2.32877
H	-1.34288	6.69250	-2.74284
C	0.74499	6.84218	-2.64165
H	0.78115	7.69707	-3.30445
C	1.99655	6.28311	-2.09792
H	2.94582	6.72171	-2.37746
C	1.96129	5.22848	-1.27541
H	2.89508	4.81946	-0.91488
C	0.65941	4.61827	-0.88638
C	0.59021	3.44470	0.03766
C	2.19730	1.65097	0.56072
C	3.53071	1.20543	1.04806
C	4.30783	2.05745	1.73341
H	5.28425	1.74309	2.08445
C	3.82829	3.41572	2.05674
C	4.65999	4.30678	2.89867
H	5.63057	3.97618	3.24716
C	4.21525	5.52243	3.23431
H	4.82304	6.17223	3.85062
C	2.88864	5.97685	2.77636
H	2.52537	6.95338	3.06958
C	2.13186	5.18147	2.01150
H	1.15330	5.52670	1.70554
C	2.62556	3.83758	1.60350
C	1.80781	2.93763	0.73488
C	4.02517	-0.15428	0.74528
C	5.27536	-0.32735	-0.02835
C	3.37213	-1.23509	1.19840
C	5.75443	-1.55572	-0.29389
C	3.88233	-2.60262	0.92347
C	5.00798	-2.74456	0.19935
C	-3.14235	2.67940	-0.16387
C	-3.51817	2.27307	1.06147
C	-4.02901	2.40508	-1.32045
C	-4.77163	1.50578	1.25778
C	-5.18179	1.72826	-1.16041

C	-5.55205	1.24287	0.19199
H	-0.74462	-0.93351	-1.26116
C	0.68835	-2.66196	-2.40119
C	-1.39194	-3.47947	-1.74129
C	1.60602	-1.58248	-2.48521
C	1.09012	-3.94463	-2.89084
C	-1.05575	-4.78200	-2.19824
C	2.86102	-1.77917	-3.01758
H	1.31706	-0.60675	-2.11993
C	2.38510	-4.10939	-3.44325
C	0.16821	-5.01089	-2.76871
H	-1.78120	-5.58066	-2.09129
C	3.25624	-3.04720	-3.50448
H	3.55656	-0.94675	-3.06254
H	2.67614	-5.08979	-3.80974
H	0.44462	-6.00129	-3.11992
H	4.24503	-3.18164	-3.93006
C	-2.78546	-3.29593	-1.17239
H	-3.12212	-4.26366	-0.79830
C	-2.98276	-2.24818	-0.06813
H	-4.05048	-2.21892	0.17877
H	-2.72173	-1.25540	-0.43621
N	-2.17423	-2.56848	1.07852
N	-0.55043	-2.45908	-1.83837
C	-2.57111	-3.38513	2.12339
C	-3.77244	-4.11994	2.11751
C	-1.72642	-3.49195	3.25007
C	-4.11155	-4.92408	3.20690
H	-4.45089	-4.06188	1.27226
C	-2.07849	-4.29869	4.32373
H	-0.80065	-2.92306	3.26429
C	-3.27470	-5.02501	4.31649
H	-5.04671	-5.47751	3.17973
H	-1.41171	-4.35886	5.17995
H	-3.54545	-5.65413	5.15846
H	-1.36631	-1.97493	1.25024
Cl	-3.92883	-2.94098	-2.57847
H	5.80009	0.55431	-0.37684
H	2.48697	-1.11052	1.80403
H	-2.89960	2.49720	1.92179
H	-3.73323	2.75220	-2.30221
H	5.39202	-3.73375	-0.01475
H	-6.46468	0.67372	0.31379
C	-5.13241	1.01486	2.64776
H	-4.37280	1.38546	3.37138
C	-6.11124	1.46204	-2.32959
H	-6.98596	0.87547	-1.97090
C	3.16249	-3.82043	1.47637
H	3.72883	-4.74370	1.21839
C	7.05119	-1.72152	-1.06441
H	7.48155	-0.72235	-1.30181
C	8.09641	-2.46870	-0.22780
H	7.78284	-3.51616	-0.03370
H	9.06921	-2.48723	-0.76405
H	8.24658	-1.95172	0.74416
C	6.80666	-2.43017	-2.39973
H	6.39111	-3.44846	-2.24454
H	6.09655	-1.83715	-3.01353
H	7.75861	-2.51800	-2.96556
C	1.76982	-3.96385	0.85665

H	1.26202	-4.86788	1.25522
H	1.13766	-3.07886	1.07358
H	1.86525	-4.08070	-0.24031
C	3.07666	-3.76357	3.00626
H	2.65412	-4.71349	3.39790
H	4.09066	-3.63030	3.44037
H	2.42724	-2.92780	3.34258
C	-5.10317	-0.51608	2.71785
H	-4.09600	-0.88359	2.43172
H	-5.31118	-0.85598	3.75475
H	-5.85842	-0.96518	2.03854
C	-6.48856	1.56605	3.10539
H	-7.31740	1.15887	2.48865
H	-6.67173	1.28963	4.16569
H	-6.49393	2.67454	3.03017
C	-6.65739	2.77138	-2.91167
H	-5.84341	3.38284	-3.35595
H	-7.40654	2.55258	-3.70228
H	-7.15668	3.36278	-2.11450
C	-5.42283	0.62301	-3.41221
H	-6.15598	0.33935	-4.19709
H	-4.59302	1.18444	-3.89126
H	-5.01533	-0.30630	-2.96513

Catalyst (3,5-(i-Pr)2, 7)-Product (R) alternative conformation
Electronic energy (Hartrees): -1870.594179
Free Energy at 1M concentration and 253 K (Hartrees): -1869.545305
Coordinates:

P	0.02796	0.32419	0.38693
O	-0.48167	1.72239	1.03287
O	1.52239	0.60409	-0.23180
O	-0.80378	0.12330	-0.94054
O	-0.05521	-0.74676	1.40651
C	-0.27112	2.83769	0.26223
C	-1.44366	3.54119	-0.32664
C	-1.24572	4.63945	-1.07249
H	-2.08836	5.16437	-1.50747
C	0.12012	5.13690	-1.33363
C	0.31300	6.31370	-2.21286
H	-0.54442	6.83435	-2.62100
C	1.54597	6.73677	-2.50982
H	1.68552	7.59535	-3.15395
C	2.72051	6.02380	-1.97443
H	3.71606	6.35411	-2.24166
C	2.55762	4.96340	-1.17497
H	3.43531	4.44054	-0.82078
C	1.19117	4.50147	-0.80311
C	0.97982	3.32462	0.09518
C	2.35682	1.33849	0.57394
C	3.62164	0.72037	1.05340
C	4.49646	1.45382	1.75751
H	5.42494	1.01260	2.10207
C	4.18705	2.85358	2.11135
C	5.11885	3.61565	2.97478
H	6.04031	3.16026	3.31582
C	4.82522	4.86827	3.33953
H	5.50586	5.42368	3.97178
C	3.56587	5.49278	2.89208
H	3.32399	6.49915	3.20873
C	2.71993	4.81480	2.10777

H	1.79214	5.28459	1.80966
C	3.04645	3.43065	1.66791
C	2.12674	2.65836	0.77863
C	3.94059	-0.68410	0.72407
C	5.13876	-0.99545	-0.08658
C	3.17517	-1.68429	1.18635
C	5.45493	-2.27034	-0.38348
C	3.51031	-3.09963	0.88417
C	4.58778	-3.36574	0.12201
C	-2.81410	3.00244	-0.14085
C	-3.24933	2.62678	1.07449
C	-3.70849	2.83981	-1.31223
C	-4.58383	2.00336	1.24553
C	-4.93004	2.29054	-1.17600
C	-5.37220	1.83759	0.16606
H	-0.87226	-0.84852	-1.27752
C	0.37072	-2.72992	-2.39762
C	-1.79174	-3.31257	-1.75466
C	1.39498	-1.75215	-2.49116
C	0.64557	-4.05835	-2.85153
C	-1.58925	-4.65050	-2.18694
C	2.63412	-2.09127	-2.98676
H	1.19981	-0.74185	-2.15945
C	1.92962	-4.37131	-3.36442
C	-0.38647	-5.01859	-2.72957
H	-2.39685	-5.36596	-2.07922
C	2.90896	-3.40777	-3.42593
H	3.41016	-1.33397	-3.03772
H	2.12551	-5.38628	-3.69859
H	-0.21039	-6.03956	-3.05706
H	3.89103	-3.65742	-3.81381
C	-3.16184	-2.97411	-1.20076
H	-3.60792	-3.89807	-0.83075
C	-3.24866	-1.91198	-0.09512
H	-4.30825	-1.76530	0.14519
H	-2.87719	-0.95342	-0.45919
N	-2.48733	-2.31832	1.05576
N	-0.84692	-2.38763	-1.85759
C	-2.95656	-3.12762	2.07498
C	-4.22963	-3.72991	2.05933
C	-2.11670	-3.36594	3.18523
C	-4.64078	-4.53549	3.12249
H	-4.90698	-3.56488	1.22746
C	-2.54137	-4.17216	4.23267
H	-1.13546	-2.89964	3.20759
C	-3.80772	-4.76780	4.21509
H	-5.62984	-4.98510	3.08841
H	-1.87565	-4.33613	5.07604
H	-4.13461	-5.39763	5.03635
H	-1.61052	-1.83199	1.22574
Cl	-4.24614	-2.49273	-2.61534
H	5.76194	-0.17951	-0.43041
H	2.32861	-1.46195	1.81936
H	-2.62031	2.76955	1.94462
H	-3.36157	3.16219	-2.28570
H	4.85082	-4.39128	-0.10673
H	-6.34450	1.37325	0.26930
C	-5.02010	1.54771	2.62587
H	-4.23490	1.82489	3.36382
C	-5.86366	2.13489	-2.36169

H	-6.80085	1.64048	-2.02244
C	2.67603	-4.23006	1.46152
H	3.10810	-5.21089	1.15998
C	6.70416	-2.61124	-1.17512
H	6.74713	-3.71252	-1.32701
C	6.68787	-1.96781	-2.56743
H	6.72281	-0.85997	-2.50075
H	7.56599	-2.31200	-3.15437
H	5.77102	-2.26561	-3.11497
C	7.96836	-2.21487	-0.40299
H	8.02900	-1.11333	-0.27308
H	7.96840	-2.69517	0.59896
H	8.87210	-2.55662	-0.95129
C	1.24286	-4.19007	0.92379
H	0.65630	-5.04214	1.32869
H	0.73256	-3.24659	1.20382
H	1.25861	-4.27906	-0.17929
C	2.68434	-4.19079	2.99420
H	2.16198	-5.08354	3.39967
H	3.72985	-4.20050	3.37002
H	2.17239	-3.28215	3.37608
C	-5.16682	0.02301	2.68432
H	-4.20339	-0.45564	2.41223
H	-5.43029	-0.29692	3.71494
H	-5.95661	-0.33288	1.98925
C	-6.31191	2.24700	3.06723
H	-7.17176	1.94051	2.43485
H	-6.54225	1.98690	4.12245
H	-6.19007	3.34934	3.00052
C	-6.25942	3.49987	-2.93743
H	-5.37826	4.02670	-3.36152
H	-7.01369	3.36859	-3.74243
H	-6.70758	4.13266	-2.14167
C	-5.25022	1.23808	-3.44317
H	-5.99814	1.03668	-4.23946
H	-4.36133	1.71552	-3.90705
H	-4.94503	0.26876	-2.99949

Catalyst (3,5-(i-Pr)2, 7)-Product (R) alternative conformation
Electronic energy (Hartrees): -1870.594302
Free Energy at 1M concentration and 253 K (Hartrees): -1869.544994
Coordinates:

P	0.10266	0.29682	0.20862
O	-0.71335	1.55823	0.82877
O	1.53319	0.88198	-0.34489
O	-0.60353	-0.05181	-1.16056
O	0.19335	-0.79197	1.20981
C	-0.80210	2.64167	-0.00813
C	-2.12143	2.99615	-0.60130
C	-2.19742	4.02135	-1.46533
H	-3.15028	4.30460	-1.89704
C	-0.98935	4.78634	-1.83646
C	-1.09030	5.87131	-2.84029
H	-2.05030	6.12519	-3.27242
C	0.00331	6.54033	-3.22000
H	-0.07144	7.33145	-3.95488
C	1.31797	6.19190	-2.64961
H	2.20314	6.71973	-2.98051
C	1.41942	5.21786	-1.73796
H	2.39830	4.96268	-1.35560

C	0.20644	4.48675	-1.27781
C	0.29104	3.39580	-0.25981
C	2.11641	1.87519	0.40383
C	3.47794	1.64334	0.95582
C	4.13200	2.64254	1.56529
H	5.12619	2.47917	1.96568
C	3.48418	3.95543	1.75370
C	4.18361	5.01551	2.51643
H	5.18229	4.84252	2.89787
C	3.58428	6.18974	2.74004
H	4.09554	6.96303	3.29873
C	2.21829	6.42990	2.23796
H	1.73005	7.37414	2.44197
C	1.57973	5.48076	1.54393
H	0.56990	5.67117	1.20604
C	2.24488	4.17940	1.25896
C	1.56139	3.11060	0.46761
C	4.11537	0.31768	0.82728
C	5.39191	0.17490	0.09092
C	3.55290	-0.75955	1.39457
C	5.97697	-1.02980	-0.03961
C	4.17281	-2.09832	1.25962
C	5.31685	-2.22149	0.55847
C	-3.34510	2.23693	-0.23592
C	-3.61566	1.95226	1.04987
C	-4.30122	1.80963	-1.28754
C	-4.84583	1.21036	1.42484
C	-5.43310	1.15673	-0.96638
C	-5.70748	0.84450	0.46006
H	-0.52768	-1.04438	-1.44705
C	1.01406	-2.85750	-2.20414
C	-1.16640	-3.56392	-1.77936
C	1.95808	-1.80090	-2.28925
C	1.44853	-4.19552	-2.46230
C	-0.80650	-4.91563	-2.01905
C	3.27183	-2.07173	-2.59748
H	1.63915	-0.78395	-2.10338
C	2.80817	-4.43708	-2.78653
C	0.48637	-5.22693	-2.35489
H	-1.56282	-5.68758	-1.93161
C	3.70451	-3.39581	-2.84835
H	3.98336	-1.25388	-2.64741
H	3.12720	-5.45802	-2.97631
H	0.78280	-6.25671	-2.53486
H	4.74392	-3.58841	-3.09244
C	-2.61835	-3.26690	-1.46253
H	-3.09573	-4.20140	-1.16552
C	-2.90355	-2.19471	-0.39354
H	-3.98952	-2.13351	-0.25883
H	-2.57867	-1.21767	-0.75161
N	-2.23484	-2.49892	0.84342
N	-0.28608	-2.57668	-1.86677
C	-2.74275	-3.29832	1.85185
C	-3.95666	-4.00341	1.73890
C	-2.01504	-3.40743	3.05712
C	-4.42138	-4.78026	2.80115
H	-4.54621	-3.94236	0.82971
C	-2.49093	-4.18757	4.10250
H	-1.08182	-2.85933	3.15536
C	-3.69952	-4.88356	3.98873

H	-5.36337	-5.31121	2.69058
H	-1.91243	-4.24998	5.02050
H	-4.06726	-5.49173	4.80901
H	-1.38494	-1.98079	1.05043
Cl	-3.45796	-2.80858	-3.03950
H	5.85113	1.05876	-0.33558
H	2.64532	-0.65021	1.97321
H	-2.94613	2.28790	1.83120
H	-4.07803	2.03595	-2.32318
H	5.77800	-3.19298	0.44184
H	-6.61417	0.31254	0.71822
C	-5.12734	0.87035	2.87636
H	-6.09596	0.32728	2.95796
C	-6.42511	0.76087	-2.04397
H	-6.02323	1.03166	-3.04644
C	3.50883	-3.29264	1.91958
H	2.61942	-2.94344	2.49004
C	7.30989	-1.16229	-0.75292
H	7.64839	-0.16417	-1.11202
C	8.38910	-1.68295	0.20339
H	8.16256	-2.71699	0.53969
H	9.37750	-1.68589	-0.30382
H	8.45893	-1.02256	1.09422
C	7.19029	-2.06369	-1.98597
H	6.89616	-3.09622	-1.70230
H	6.43481	-1.64975	-2.68596
H	8.16328	-2.10882	-2.52011
C	4.44500	-3.97121	2.92749
H	3.89571	-4.76125	3.48274
H	5.31327	-4.43996	2.41797
H	4.81742	-3.22607	3.66270
C	3.00154	-4.29086	0.87526
H	2.48609	-5.13905	1.37401
H	2.26960	-3.78894	0.21082
H	3.83609	-4.69161	0.26162
C	-5.25431	2.14074	3.72470
H	-6.03117	2.80896	3.29518
H	-5.55833	1.87661	4.76007
H	-4.29146	2.69200	3.77132
C	-4.04525	-0.05930	3.43546
H	-3.04989	0.43367	3.43494
H	-4.29518	-0.35445	4.47679
H	-3.99004	-0.98020	2.81981
C	-6.66166	-0.75353	-2.04943
H	-7.28726	-1.03703	-2.92252
H	-5.69362	-1.28749	-2.12764
H	-7.17818	-1.08576	-1.12438
C	-7.74541	1.51882	-1.86822
H	-8.43721	1.27706	-2.70320
H	-8.23812	1.24769	-0.91026
H	-7.55907	2.61416	-1.87826

Catalyst (3,5-(i-Pr)2, 7)-Product (R) alternative conformation
Electronic energy (Hartrees): -1870.594116
Free Energy at 1M concentration and 253 K (Hartrees): -1869.544212
Coordinates:

P	0.17669	0.28354	0.20783
O	-0.61822	1.55786	0.83074
O	1.63290	0.83756	-0.30976
O	-0.50670	-0.02332	-1.18302

O	0.21614	-0.82350	1.19218
C	-0.68115	2.64280	-0.00622
C	-1.98754	3.01513	-0.61489
C	-2.03691	4.02940	-1.49308
H	-2.98070	4.32349	-1.93731
C	-0.81144	4.76943	-1.85906
C	-0.88259	5.84527	-2.87514
H	-1.83305	6.11000	-3.32160
C	0.22622	6.49281	-3.24799
H	0.17317	7.27744	-3.99166
C	1.52808	6.12954	-2.65773
H	2.42554	6.63983	-2.98295
C	1.60279	5.16359	-1.73494
H	2.57265	4.89696	-1.33764
C	0.37279	4.45675	-1.28245
C	0.42792	3.37424	-0.25339
C	2.21901	1.82564	0.44321
C	3.56800	1.57340	1.01646
C	4.23197	2.56548	1.62678
H	5.21715	2.38715	2.04272
C	3.60591	3.89164	1.79544
C	4.31442	4.94512	2.55900
H	5.30456	4.75713	2.95532
C	3.73384	6.13193	2.76504
H	4.25170	6.90038	3.32436
C	2.37936	6.39284	2.24264
H	1.90585	7.34750	2.43244
C	1.73280	5.44980	1.54770
H	0.73126	5.65597	1.19476
C	2.37758	4.13403	1.28220
C	1.68514	3.07149	0.49005
C	4.18121	0.23411	0.90835
C	5.46943	0.06071	0.19906
C	3.58259	-0.82913	1.46530
C	6.02987	-1.15726	0.08338
C	4.17623	-2.18148	1.34805
C	5.33231	-2.33181	0.67257
C	-3.22763	2.28760	-0.24379
C	-3.52139	2.05480	1.04747
C	-4.17492	1.83871	-1.29455
C	-4.76556	1.34541	1.42968
C	-5.31955	1.21082	-0.96770
C	-5.61491	0.94742	0.46425
H	-0.45601	-1.01543	-1.47881
C	1.03980	-2.88460	-2.18304
C	-1.18431	-3.49638	-1.84424
C	2.02976	-1.86851	-2.23021
C	1.42643	-4.23998	-2.42584
C	-0.87460	-4.86188	-2.07375
C	3.34183	-2.19479	-2.48664
H	1.74681	-0.83884	-2.05614
C	2.78665	-4.53886	-2.69669
C	0.41646	-5.22845	-2.35882
H	-1.66620	-5.60072	-2.01774
C	3.72801	-3.53624	-2.72122
H	4.08821	-1.40747	-2.50732
H	3.06975	-5.57244	-2.87471
H	0.67471	-6.27008	-2.52929
H	4.76789	-3.77202	-2.92209
C	-2.63062	-3.12920	-1.57810

H	-3.17130	-4.04186	-1.32420
C	-2.89190	-2.06868	-0.49259
H	-3.97486	-1.94893	-0.38622
H	-2.50175	-1.10367	-0.81593
N	-2.28386	-2.44916	0.75662
N	-0.25871	-2.54903	-1.89606
C	-2.93236	-3.13376	1.77114
C	-4.20354	-3.71897	1.61331
C	-2.29440	-3.24934	3.02517
C	-4.81419	-4.37708	2.68160
H	-4.72474	-3.65786	0.66321
C	-2.91466	-3.91205	4.07628
H	-1.31637	-2.79468	3.15855
C	-4.18270	-4.48198	3.91939
H	-5.79899	-4.81324	2.53553
H	-2.40359	-3.97883	5.03312
H	-4.66434	-4.99634	4.74485
H	-1.42000	-1.97413	1.00561
Cl	-3.37920	-2.58204	-3.17280
H	5.95624	0.93253	-0.22153
H	2.66366	-0.69873	2.02151
H	-2.85761	2.40782	1.82739
H	-3.93776	2.03467	-2.33328
H	5.77483	-3.31343	0.56937
H	-6.53027	0.43491	0.72735
C	-5.05677	1.08964	2.89680
H	-4.24364	1.53615	3.51108
C	-6.31430	0.81370	-2.04276
H	-5.89454	1.04029	-3.04884
C	3.47208	-3.35918	1.99612
H	2.57680	-2.98939	2.54390
C	7.37177	-1.32261	-0.60590
H	7.73737	-0.33428	-0.96547
C	8.42289	-1.85939	0.37248
H	8.16816	-2.88580	0.71200
H	9.41953	-1.88739	-0.11755
H	8.49178	-1.19416	1.25977
C	7.25471	-2.23002	-1.83509
H	6.93586	-3.25472	-1.54981
H	6.51919	-1.80686	-2.55060
H	8.23539	-2.29780	-2.35254
C	4.36988	-4.05282	3.02838
H	3.79161	-4.82970	3.57266
H	5.24052	-4.54071	2.54135
H	4.73951	-3.31271	3.77003
C	2.96936	-4.35116	0.94354
H	2.42224	-5.18491	1.43280
H	2.26649	-3.83668	0.25750
H	3.81028	-4.77406	0.35394
C	-5.07291	-0.41180	3.20305
H	-4.09748	-0.85801	2.92137
H	-5.22735	-0.57960	4.29018
H	-5.88191	-0.92944	2.64514
C	-6.36531	1.75926	3.33501
H	-7.24181	1.29657	2.83408
H	-6.49436	1.65656	4.43362
H	-6.33971	2.84235	3.08858
C	-6.60513	-0.69099	-2.00630
H	-7.24243	-0.97525	-2.87064
H	-5.65854	-1.26234	-2.07281

H	-7.13170	-0.97972	-1.07245
C	-7.60924	1.62119	-1.90196
H	-8.30288	1.37729	-2.73479
H	-8.11763	1.39691	-0.93997
H	-7.38558	2.70875	-1.94423

Catalyst (3,5-(i-Pr)2, 7)-Product (R) alternative conformation
Electronic energy (Hartrees): -1870.593336
Free Energy at 1M concentration and 253 K (Hartrees): -1869.544032
Coordinates:

P	0.12419	0.28778	0.25885
O	-0.52973	1.63722	0.88390
O	1.62124	0.69314	-0.27869
O	-0.61233	0.04041	-1.11833
O	0.06842	-0.81255	1.24953
C	-0.45734	2.73722	0.06679
C	-1.70653	3.27200	-0.54124
C	-1.63389	4.31752	-1.38016
H	-2.53383	4.72562	-1.82539
C	-0.33037	4.92897	-1.71086
C	-0.27106	6.04069	-2.68845
H	-1.18192	6.42830	-3.12760
C	0.90710	6.56887	-3.03588
H	0.94929	7.37969	-3.75165
C	2.15483	6.03720	-2.45663
H	3.10710	6.45097	-2.76257
C	2.11218	5.03750	-1.56845
H	3.04271	4.64636	-1.18049
C	0.80617	4.46224	-1.14268
C	0.72969	3.34679	-0.15031
C	2.32467	1.58149	0.49783
C	3.63593	1.15629	1.05614
C	4.41275	2.04509	1.69165
H	5.37141	1.74039	2.09610
C	3.94563	3.42932	1.90377
C	4.77374	4.36970	2.69404
H	5.73649	4.05619	3.07819
C	4.33547	5.60907	2.93896
H	4.94075	6.29499	3.51747
C	3.01883	6.04101	2.43331
H	2.65994	7.03791	2.65458
C	2.26515	5.20088	1.71466
H	1.29321	5.53202	1.37431
C	2.75246	3.82831	1.40546
C	1.94006	2.87843	0.58480
C	4.08742	-0.24107	0.90571
C	5.33165	-0.54517	0.16392
C	3.38278	-1.24019	1.45634
C	5.73763	-1.81929	-0.00072
C	3.80846	-2.64948	1.29088
C	4.91680	-2.91769	0.57210
C	-3.01996	2.65388	-0.23090
C	-3.36681	2.38356	1.03971
C	-3.96902	2.33249	-1.32517
C	-4.66637	1.74352	1.35425
C	-5.16196	1.77032	-1.05923
C	-5.51315	1.45331	0.34896
H	-0.62316	-0.94324	-1.42964
C	0.78325	-2.86220	-2.22464
C	-1.43457	-3.43885	-1.80443

C	1.78594	-1.86253	-2.31661
C	1.14231	-4.22455	-2.47099
C	-1.15167	-4.81085	-2.03500
C	3.08443	-2.21026	-2.61141
H	1.52403	-0.82744	-2.14411
C	2.48878	-4.54681	-2.77864
C	0.12235	-5.19880	-2.36125
H	-1.95216	-5.53693	-1.94720
C	3.44444	-3.55965	-2.84049
H	3.83950	-1.43285	-2.66591
H	2.75055	-5.58650	-2.95372
H	0.36056	-6.24534	-2.53064
H	4.47454	-3.81542	-3.06608
C	-2.87101	-3.06180	-1.50232
H	-3.39344	-3.95965	-1.17016
C	-3.10864	-1.93935	-0.47663
H	-4.19013	-1.79458	-0.37702
H	-2.70195	-0.99816	-0.84746
N	-2.50422	-2.26078	0.78848
N	-0.49934	-2.50316	-1.89153
C	-3.10152	-3.00068	1.79347
C	-4.37353	-3.59140	1.66375
C	-2.41017	-3.16175	3.01425
C	-4.93025	-4.30519	2.72600
H	-4.93810	-3.48817	0.74251
C	-2.97806	-3.87850	4.05911
H	-1.43125	-2.70300	3.12464
C	-4.24483	-4.45871	3.92949
H	-5.91601	-4.74621	2.60244
H	-2.42563	-3.98232	4.98936
H	-4.68435	-5.01722	4.74976
H	-1.60988	-1.82857	1.00429
Cl	-3.68400	-2.63169	-3.10270
H	5.91527	0.27496	-0.23479
H	2.50050	-1.01671	2.04125
H	-2.69855	2.64272	1.85192
H	-3.68795	2.55772	-2.34690
H	5.23666	-3.94177	0.42895
H	-6.46456	0.98455	0.56132
C	-5.00816	1.42058	2.79683
H	-4.18563	1.78090	3.45371
C	-6.14152	1.47706	-2.17981
H	-5.69164	1.75652	-3.15925
C	2.99129	-3.75036	1.94212
H	2.17715	-3.28897	2.54442
C	7.04136	-2.15044	-0.70425
H	7.16141	-3.25585	-0.74245
C	7.05113	-1.64944	-2.15389
H	6.98769	-0.54183	-2.19908
H	7.98781	-1.96827	-2.65893
H	6.19797	-2.08260	-2.71364
C	8.24048	-1.59628	0.07471
H	8.22718	-0.48567	0.09259
H	8.22209	-1.97270	1.11997
H	9.18883	-1.93094	-0.39735
C	3.83909	-4.58579	2.90979
H	3.19041	-5.29598	3.46575
H	4.61220	-5.16939	2.36681
H	4.33921	-3.92285	3.64791
C	2.31640	-4.63473	0.89077

H	1.70511	-5.41989	1.38447
H	1.63818	-4.01501	0.26992
H	3.06698	-5.12583	0.23576
C	-5.11904	-0.09296	3.01118
H	-4.16700	-0.58039	2.71703
H	-5.30366	-0.31551	4.08368
H	-5.94689	-0.52632	2.41068
C	-6.28588	2.14004	3.24683
H	-7.17525	1.76090	2.70042
H	-6.44785	1.98042	4.33422
H	-6.19111	3.23232	3.06688
C	-6.46907	-0.01818	-2.24678
H	-7.09621	-0.23084	-3.13868
H	-5.53222	-0.60393	-2.33325
H	-7.01802	-0.35328	-1.34155
C	-7.41785	2.30984	-2.01708
H	-8.09267	2.14510	-2.88409
H	-7.96126	2.03335	-1.08857
H	-7.16378	3.39071	-1.97580

Catalyst (3,5-(i-Pr)2, 7)-Product (R) alternative conformation
Electronic energy (Hartrees): -1870.593168
Free Energy at 1M concentration and 253 K (Hartrees): -1869.543941
Coordinates:

P	0.00734	0.27225	0.29234
O	-0.74069	1.56162	0.93753
O	1.44880	0.80120	-0.28704
O	-0.75025	-0.04104	-1.05784
O	0.08076	-0.81988	1.29108
C	-0.73101	2.69139	0.15813
C	-2.01033	3.18041	-0.42977
C	-2.00019	4.28121	-1.19931
H	-2.92166	4.66061	-1.62552
C	-0.73753	4.98993	-1.48759
C	-0.74715	6.15993	-2.39657
H	-1.68084	6.51896	-2.81145
C	0.39661	6.77625	-2.71185
H	0.38851	7.62968	-3.37750
C	1.67538	6.28437	-2.16650
H	2.60080	6.77030	-2.44774
C	1.69443	5.23228	-1.34016
H	2.64799	4.87315	-0.97813
C	0.42558	4.55744	-0.94857
C	0.41689	3.38481	-0.02135
C	2.11954	1.68901	0.51822
C	3.46420	1.31653	1.03201
C	4.19282	2.21337	1.71185
H	5.17622	1.94785	2.08313
C	3.64177	3.54953	2.01361
C	4.41920	4.49191	2.85153
H	5.40456	4.21783	3.20754
C	3.90694	5.68417	3.17458
H	4.47512	6.37146	3.78803
C	2.55951	6.06118	2.70726
H	2.14166	7.01853	2.99090
C	1.85106	5.21917	1.94604
H	0.85624	5.50748	1.63374
C	2.41950	3.90107	1.55138
C	1.65717	2.95144	0.68427
C	4.00436	-0.03383	0.78429

C	5.22584	-0.20647	-0.03317
C	3.40473	-1.10821	1.31673
C	5.71405	-1.43676	-0.28468
C	3.92239	-2.47175	1.05671
C	5.00576	-2.62061	0.26826
C	-3.28529	2.45633	-0.18782
C	-3.62214	2.04530	1.04716
C	-4.21475	2.19441	-1.31431
C	-4.89294	1.31503	1.28810
C	-5.38417	1.55853	-1.11145
C	-5.72483	1.09221	0.25506
H	-0.69734	-1.02418	-1.36788
C	0.81222	-2.78594	-2.31420
C	-1.30060	-3.58343	-1.74069
C	1.71175	-1.69531	-2.43670
C	1.27081	-4.09883	-2.64875
C	-0.91699	-4.91236	-2.06029
C	3.00828	-1.91055	-2.84485
H	1.37392	-0.69609	-2.19775
C	2.61182	-4.28383	-3.07147
C	0.35430	-5.16696	-2.50617
H	-1.63860	-5.71292	-1.94222
C	3.46782	-3.21121	-3.16125
H	3.68391	-1.06494	-2.92158
H	2.95018	-5.28766	-3.31227
H	0.66998	-6.17921	-2.74357
H	4.49509	-3.36227	-3.47636
C	-2.72900	-3.35899	-1.28572
H	-3.13253	-4.31877	-0.96112
C	-2.95945	-2.31993	-0.17631
H	-4.03513	-2.27932	0.03048
H	-2.67281	-1.32844	-0.52883
N	-2.20198	-2.65430	1.00208
N	-0.46520	-2.56114	-1.86320
C	-2.67421	-3.42290	2.05274
C	-3.87465	-4.15673	1.99256
C	-1.91678	-3.47622	3.24299
C	-4.29824	-4.90639	3.09090
H	-4.48494	-4.14199	1.09514
C	-2.35101	-4.23061	4.32492
H	-0.99385	-2.90547	3.30092
C	-3.54696	-4.95426	4.26368
H	-5.23091	-5.46017	3.02046
H	-1.75068	-4.24917	5.23076
H	-3.88299	-5.54113	5.11261
H	-1.38322	-2.08074	1.19054
Cl	-3.74082	-2.92092	-2.76421
H	5.72782	0.67422	-0.41345
H	2.54188	-0.98015	1.95658
H	-2.97379	2.26187	1.88602
H	-3.93997	2.52348	-2.30834
H	5.39107	-3.60947	0.05567
H	-6.65583	0.56117	0.41205
C	-5.24333	0.81855	2.67842
H	-6.23742	0.31724	2.66279
C	-6.35938	1.31307	-2.24768
H	-7.25686	0.79033	-1.84876
C	3.22703	-3.66080	1.69450
H	2.42088	-3.29094	2.36670
C	7.00387	-1.63342	-1.06096

H	7.20030	-2.72344	-1.16620
C	6.91184	-1.05686	-2.47928
H	6.76529	0.04357	-2.45979
H	7.84577	-1.27659	-3.03928
H	6.06912	-1.52337	-3.02731
C	8.19527	-1.03455	-0.30351
H	8.10344	0.06951	-0.22238
H	8.25238	-1.46644	0.71865
H	9.14219	-1.27259	-0.83352
C	4.18962	-4.47778	2.56584
H	3.62774	-5.25852	3.12168
H	4.96561	-4.97781	1.94866
H	4.68801	-3.81628	3.30641
C	2.55292	-4.53995	0.63851
H	2.03681	-5.39574	1.12344
H	1.78961	-3.94436	0.09817
H	3.29338	-4.93497	-0.08902
C	-5.34577	1.98257	3.67062
H	-6.07335	2.73591	3.29972
H	-5.70273	1.61137	4.65499
H	-4.36135	2.47490	3.81756
C	-4.22497	-0.22267	3.15414
H	-3.20754	0.21543	3.23662
H	-4.52143	-0.62340	4.14687
H	-4.19518	-1.06759	2.43643
C	-6.84616	2.63272	-2.85928
H	-6.01441	3.17651	-3.35535
H	-7.63596	2.43273	-3.61450
H	-7.27900	3.28186	-2.06824
C	-5.74962	0.40050	-3.31722
H	-6.50801	0.16403	-4.09375
H	-4.87804	0.88198	-3.80907
H	-5.42111	-0.55211	-2.85317

Catalyst (3,5-(i-Pr)2, 7)-Product (R) alternative conformation
Electronic energy (Hartrees): -1870.59266
Free Energy at 1M concentration and 253 K (Hartrees): -1869.543293
Coordinates:

P	0.10297	0.24869	0.28779
O	-0.59718	1.57476	0.91386
O	1.58234	0.70480	-0.25818
O	-0.62913	-0.02679	-1.08685
O	0.08956	-0.85235	1.27913
C	-0.56861	2.67518	0.09460
C	-1.83946	3.16574	-0.50497
C	-1.80811	4.20646	-1.35242
H	-2.72489	4.58252	-1.79164
C	-0.52850	4.85934	-1.69648
C	-0.51378	5.96643	-2.68095
H	-1.44012	6.31872	-3.11749
C	0.64303	6.53418	-3.03748
H	0.65267	7.34173	-3.75809
C	1.91176	6.05110	-2.46130
H	2.84709	6.49705	-2.77420
C	1.90923	5.05587	-1.56705
H	2.85500	4.70091	-1.18107
C	0.62672	4.43672	-1.13161
C	0.59495	3.32463	-0.13302
C	2.25768	1.62151	0.51012
C	3.58894	1.24857	1.05801

C	4.33480	2.16736	1.68819
H	5.30808	1.90096	2.08469
C	3.81568	3.53251	1.90299
C	4.61120	4.50479	2.68822
H	5.58712	4.22868	3.06787
C	4.12697	5.72668	2.93444
H	4.70828	6.43576	3.50957
C	2.79241	6.10756	2.43470
H	2.39680	7.09031	2.65668
C	2.06794	5.23848	1.72039
H	1.08262	5.53204	1.38392
C	2.60551	3.88490	1.41084
C	1.82514	2.90317	0.59679
C	4.09627	-0.12855	0.90024
C	5.34342	-0.37886	0.14330
C	3.44085	-1.15705	1.45765
C	5.80424	-1.63387	-0.02331
C	3.92897	-2.54611	1.29208
C	5.04146	-2.76608	0.56338
C	-3.13091	2.51571	-0.17111
C	-3.46028	2.26239	1.10753
C	-4.08291	2.15523	-1.24957
C	-4.74752	1.60909	1.44662
C	-5.26835	1.58527	-0.96002
C	-5.59990	1.28795	0.45489
H	-0.61273	-1.01131	-1.39330
C	0.84261	-2.89470	-2.18318
C	-1.35923	-3.53191	-1.76697
C	1.81677	-1.86747	-2.27588
C	1.24029	-4.24683	-2.42595
C	-1.03738	-4.89618	-1.99359
C	3.12519	-2.17901	-2.56715
H	1.52560	-0.83961	-2.10722
C	2.59598	-4.53168	-2.73025
C	0.24755	-5.24908	-2.31619
H	-1.81791	-5.64381	-1.90678
C	3.52384	-3.51809	-2.79195
H	3.85763	-1.38040	-2.62198
H	2.88725	-5.56401	-2.90230
H	0.51517	-6.28893	-2.48288
H	4.56163	-3.74474	-3.01369
C	-2.80710	-3.19527	-1.47141
H	-3.29876	-4.09894	-1.10911
C	-3.08245	-2.04890	-0.48355
H	-4.16765	-1.92748	-0.39841
H	-2.69517	-1.10908	-0.87778
N	-2.48658	-2.31997	0.79752
N	-0.45047	-2.57059	-1.85372
C	-3.09049	-3.02265	1.82555
C	-4.35591	-3.62904	1.70523
C	-2.41357	-3.12514	3.06067
C	-4.92249	-4.29775	2.79134
H	-4.90776	-3.57345	0.77238
C	-2.99108	-3.79786	4.12930
H	-1.43983	-2.65382	3.16316
C	-4.25270	-4.39123	4.00965
H	-5.90342	-4.75118	2.67470
H	-2.45063	-3.85580	5.07049
H	-4.70010	-4.91439	4.84875
H	-1.59460	-1.87987	1.00581

Cl	-3.63520	-2.84644	-3.08493
H	5.88365	0.46528	-0.26637
H	2.55397	-0.97210	2.04939
H	-2.78910	2.54946	1.90791
H	-3.81161	2.35914	-2.27761
H	5.40672	-3.77495	0.42089
H	-6.54174	0.80559	0.68279
C	-5.07300	1.31375	2.89903
H	-4.24915	1.69814	3.54047
C	-6.27030	1.24237	-2.04628
H	-7.19114	0.83340	-1.57431
C	3.17116	-3.68093	1.95646
H	2.33994	-3.25565	2.56196
C	7.11016	-1.90868	-0.74686
H	7.28455	-3.00715	-0.77459
C	7.06762	-1.42627	-2.20213
H	6.93850	-0.32490	-2.25898
H	8.01304	-1.69632	-2.71920
H	6.23249	-1.91607	-2.74237
C	8.29471	-1.28622	0.00241
H	8.22876	-0.17739	0.00475
H	8.31275	-1.64745	1.05302
H	9.24961	-1.58222	-0.48211
C	4.06615	-4.46724	2.92278
H	3.45753	-5.20531	3.48762
H	4.86356	-5.01497	2.37755
H	4.53792	-3.77570	3.65329
C	2.53287	-4.60456	0.91603
H	1.96167	-5.41329	1.41959
H	1.82341	-4.02244	0.29372
H	3.30214	-5.06570	0.26095
C	-5.16439	-0.19555	3.14846
H	-4.20878	-0.67793	2.85847
H	-5.33830	-0.39582	4.22714
H	-5.99143	-0.65223	2.56444
C	-6.35507	2.02826	3.34441
H	-7.24442	1.62704	2.81399
H	-6.50607	1.89060	4.43638
H	-6.27452	3.11737	3.13997
C	-6.69104	2.48795	-2.83669
H	-5.84063	2.90138	-3.41913
H	-7.50826	2.23031	-3.54376
H	-7.06661	3.27023	-2.14283
C	-5.72527	0.15433	-2.97566
H	-6.49263	-0.12539	-3.72860
H	-4.81329	0.49849	-3.50813
H	-5.47907	-0.74974	-2.38145

Catalyst (2,4,6-c-Pent, 10)-THF complex
Electronic energy (Hartrees): -876.345273
Free Energy at 1M concentration and 253 K (Hartrees): -875.039661
Coordinates:

P	-0.16835	-0.45216	0.06895
O	0.91655	0.49208	0.88131
O	-1.19575	0.54145	-0.72506
O	0.58931	-1.06389	-1.17572
O	-0.77421	-1.39844	1.03120
C	1.30533	1.59144	0.15901
C	2.73112	1.71414	-0.25667
C	3.15808	2.83114	-0.87384

H	4.19680	2.92084	-1.18105
C	2.21105	3.92796	-1.18070
C	2.68174	5.11380	-1.94303
H	3.72423	5.19535	-2.23576
C	1.81970	6.08660	-2.27715
H	2.16368	6.95104	-2.83598
C	0.39311	5.97619	-1.89738
H	-0.30369	6.75467	-2.19126
C	-0.04093	4.91116	-1.20406
H	-1.09516	4.84030	-0.95969
C	0.91209	3.83512	-0.79736
C	0.45533	2.63671	-0.02120
C	-1.69948	1.48177	0.13312
C	-3.13135	1.41429	0.53331
C	-3.65740	2.37805	1.31192
H	-4.70606	2.33862	1.59507
C	-2.80457	3.48133	1.81608
C	-3.37778	4.48896	2.74598
H	-4.42570	4.43857	3.02597
C	-2.60195	5.46315	3.24658
H	-3.02095	6.19738	3.92716
C	-1.16833	5.53138	2.88249
H	-0.54159	6.30859	3.30804
C	-0.64175	4.62880	2.03895
H	0.41572	4.68439	1.80401
C	-1.49841	3.55692	1.45036
C	-0.93792	2.54005	0.50319
C	-3.98116	0.30134	0.03768
C	-4.37844	0.27480	-1.41620
C	-4.37200	-0.71015	0.86848
C	-5.09193	-0.77423	-1.88303
C	-5.20918	-1.81395	0.31957
C	-5.53769	-1.86436	-0.98472
H	-5.36429	-0.82109	-2.93358
H	-5.54579	-2.60128	0.98655
C	3.68266	0.59840	-0.01253
C	4.16112	0.34182	1.24105
C	4.15913	-0.22723	-1.17969
C	5.15389	-0.75701	1.42843
C	5.10268	-1.17124	-0.97381
C	5.62032	-1.46442	0.38457
H	5.51425	-0.97409	2.42975
H	5.48120	-1.75270	-1.80900
H	0.98489	-1.96271	-0.93854
O	1.54917	-3.32260	-0.31538
C	0.47915	-4.26233	-0.00832
C	2.31372	-3.04080	0.88673
C	0.79700	-4.80513	1.38529
H	-0.46852	-3.71551	-0.01296
H	0.47429	-5.02545	-0.79163
C	1.49808	-3.60760	2.04630
H	3.29018	-3.52819	0.79187
H	2.46000	-1.96047	0.94692
H	1.47396	-5.66459	1.32052
H	-0.10506	-5.11878	1.91673
H	2.12714	-3.88782	2.89559
H	0.75772	-2.87221	2.37045
C	3.60439	-0.01646	-2.59086
C	4.66290	0.55638	-3.54333
C	3.08612	-1.31432	-3.23709

H	2.75019	0.69207	-2.57362
C	4.49829	-0.19636	-4.85231
H	5.69447	0.39166	-3.16039
H	4.51735	1.64846	-3.69450
C	3.94696	-1.55614	-4.46753
H	2.02939	-1.17666	-3.55769
H	3.12047	-2.19112	-2.55406
H	3.76341	0.33373	-5.49801
H	5.46449	-0.28928	-5.39392
H	3.35647	-2.00217	-5.29693
H	4.78685	-2.23821	-4.20880
C	6.66497	-2.55074	0.58927
C	6.16004	-3.93359	0.15821
C	7.94827	-2.28234	-0.21363
H	6.94484	-2.61504	1.66612
C	7.33828	-4.60745	-0.52049
H	5.32429	-3.86068	-0.57174
H	5.81287	-4.52664	1.03232
C	8.14050	-3.47736	-1.13727
H	8.81478	-2.21029	0.48059
H	7.90528	-1.34011	-0.80356
H	7.95762	-5.13284	0.23999
H	7.00384	-5.33565	-1.29090
H	9.21373	-3.75009	-1.23380
H	7.73106	-3.23987	-2.14431
C	3.73143	1.14004	2.47547
C	3.07089	0.24819	3.53968
C	4.91194	1.84148	3.15930
H	3.00286	1.93706	2.22063
C	3.89657	0.40258	4.80937
H	3.01720	-0.82184	3.24079
H	2.03158	0.59805	3.72924
C	4.66245	1.70287	4.65029
H	4.96775	2.91279	2.86604
H	5.88437	1.36403	2.91036
H	3.25185	0.42249	5.71463
H	4.61550	-0.44249	4.89160
H	4.03426	2.55037	5.00403
H	5.61467	1.68590	5.22347
C	-3.98536	-0.77108	2.35000
C	-5.20962	-0.59640	3.25831
C	-3.33654	-2.11080	2.74246
H	-3.25677	0.02314	2.61636
C	-5.05035	-1.61243	4.37576
H	-6.15852	-0.80456	2.71574
H	-5.26145	0.43637	3.66737
C	-4.25604	-2.75356	3.76996
H	-2.34637	-1.92267	3.21448
H	-3.17432	-2.79184	1.87837
H	-4.47292	-1.16003	5.21234
H	-6.03578	-1.96052	4.75436
H	-3.67955	-3.30120	4.54671
H	-4.94873	-3.46123	3.26325
C	-6.36559	-3.01030	-1.54651
C	-5.65403	-4.36504	-1.41291
C	-7.72769	-3.15429	-0.84825
H	-6.55464	-2.82992	-2.62901
C	-6.71142	-5.34849	-0.94032
H	-4.83248	-4.33073	-0.66348
H	-5.22266	-4.68870	-2.38532

C	-7.72160	-4.51704	-0.17251
H	-8.54008	-3.12753	-1.60818
H	-7.92704	-2.34952	-0.10659
H	-7.20692	-5.81577	-1.82015
H	-6.27185	-6.14412	-0.30048
H	-8.72922	-4.98594	-0.19153
H	-7.38789	-4.40932	0.88336
C	-3.98913	1.40506	-2.37358
C	-3.10314	0.91934	-3.53268
C	-5.20455	2.09692	-3.00567
H	-3.42520	2.19852	-1.84197
C	-3.83035	1.28974	-4.81808
H	-2.90470	-0.17463	-3.50043
H	-2.12148	1.44275	-3.49867
C	-4.80590	2.38954	-4.44093
H	-5.45860	3.03600	-2.46673
H	-6.10455	1.44463	-3.00739
H	-3.12156	1.63131	-5.60327
H	-4.39501	0.40785	-5.19391
H	-4.29294	3.37560	-4.49223
H	-5.68891	2.39818	-5.11603

Catalyst (2,4,6-c-Pent, 10)-Quinoline complex
Electronic energy (Hartrees): -1582.829698
Free Energy at 1M concentration and 253 K (Hartrees): -1581.483353
Coordinates:

P	-0.33279	0.13126	-0.48498
O	0.45227	1.45013	-1.04790
O	-1.47021	0.69598	0.53997
O	0.62038	-0.57306	0.55314
O	-0.80314	-0.68063	-1.62757
C	0.67316	2.44132	-0.12381
C	2.06475	2.71956	0.30459
C	2.32145	3.76039	1.10913
H	3.33647	3.95717	1.43508
C	1.21660	4.58150	1.64269
C	1.51255	5.66265	2.61083
H	2.53807	5.89498	2.86991
C	0.51541	6.35234	3.17416
H	0.73276	7.14019	3.88373
C	-0.88522	6.02813	2.84554
H	-1.68849	6.56999	3.32798
C	-1.16359	5.05701	1.96830
H	-2.19922	4.81768	1.76991
C	-0.06444	4.30492	1.30163
C	-0.34556	3.22111	0.30923
C	-2.23858	1.69594	-0.00100
C	-3.66477	1.42337	-0.29599
C	-4.45110	2.40460	-0.76212
H	-5.49781	2.21167	-0.96711
C	-3.88419	3.73859	-1.05076
C	-4.73896	4.78177	-1.66281
H	-5.78551	4.58239	-1.85723
C	-4.22141	5.97279	-1.98215
H	-4.84638	6.73377	-2.43133
C	-2.79292	6.24866	-1.73698
H	-2.37539	7.20648	-2.01933
C	-2.01285	5.31512	-1.17993
H	-0.96319	5.53049	-1.03118
C	-2.58038	3.99638	-0.78872

C	-1.73319	2.94010	-0.15647
C	-4.20949	0.06822	-0.04952
C	-4.34865	-0.41655	1.36669
C	-4.49759	-0.77061	-1.08298
C	-4.71711	-1.69301	1.58845
C	-4.96934	-2.14680	-0.78448
C	-5.05018	-2.60139	0.47446
H	-4.78741	-2.06825	2.60218
H	-5.22369	-2.80430	-1.60614
C	3.15210	1.78275	-0.06273
C	3.65854	1.74743	-1.32569
C	3.70700	0.86504	0.99055
C	4.77839	0.81656	-1.62972
C	4.75157	0.07527	0.68348
C	5.31732	0.03983	-0.68067
H	5.17892	0.79009	-2.63547
H	5.18170	-0.57013	1.43843
H	0.94683	-1.48984	0.25540
C	3.12073	0.81301	2.40237
C	4.08869	1.38488	3.44593
C	2.76772	-0.61264	2.86550
H	2.18192	1.40185	2.46062
C	3.96984	0.48100	4.65997
H	5.14100	1.36541	3.08502
H	3.82613	2.43379	3.70558
C	3.60170	-0.88334	4.10869
H	1.68857	-0.66524	3.13296
H	2.96156	-1.38580	2.09165
H	3.15316	0.84829	5.32039
H	4.92018	0.44315	5.23520
H	3.03696	-1.48128	4.85629
H	4.52723	-1.43187	3.82523
C	6.49721	-0.86338	-0.99524
C	6.15981	-2.34583	-0.78323
C	7.71137	-0.55672	-0.10500
H	6.80875	-0.72994	-2.05692
C	7.30981	-2.93886	0.01379
H	5.21506	-2.47969	-0.21163
H	6.05697	-2.86862	-1.75765
C	7.91982	-1.77847	0.77601
H	8.61166	-0.41100	-0.74257
H	7.57982	0.35764	0.51502
H	8.06654	-3.36401	-0.68226
H	6.95777	-3.73617	0.70392
H	8.99824	-1.95245	0.98160
H	7.38269	-1.64274	1.74047
C	3.15114	2.65046	-2.45089
C	2.53901	1.83995	-3.60230
C	4.27055	3.50909	-3.06374
H	2.36965	3.35374	-2.09442
C	3.03229	2.49300	-4.88107
H	2.87310	0.77964	-3.58585
H	1.42820	1.85334	-3.55977
C	4.37346	3.10736	-4.52809
H	3.99455	4.58533	-2.99909
H	5.25039	3.38174	-2.55324
H	2.32486	3.29533	-5.18732
H	3.12912	1.75341	-5.70525
H	4.59931	3.98216	-5.17562
H	5.17450	2.34557	-4.65202

C	-4.32949	-0.37712	-2.55199
C	-5.67922	-0.31961	-3.27918
C	-3.45039	-1.36748	-3.33683
H	-3.85326	0.61920	-2.66130
C	-5.44609	-0.94889	-4.64133
H	-6.46190	-0.90141	-2.74358
H	-6.03451	0.72899	-3.38336
C	-4.32862	-1.95304	-4.43283
H	-2.60070	-0.82292	-3.80611
H	-3.02318	-2.17541	-2.70327
H	-5.11534	-0.16815	-5.36176
H	-6.36582	-1.43989	-5.02679
H	-3.75469	-2.11863	-5.37025
H	-4.75784	-2.92111	-4.09198
C	-5.47740	-4.02801	0.77175
C	-4.49520	-5.06134	0.19974
C	-6.86224	-4.36608	0.19550
H	-5.52231	-4.17617	1.87458
C	-5.35105	-6.15447	-0.41738
H	-3.84462	-4.62381	-0.58971
H	-3.84062	-5.47656	0.99702
C	-6.64375	-5.47296	-0.82490
H	-7.52145	-4.74410	1.00836
H	-7.36651	-3.49309	-0.27464
H	-5.56705	-6.93519	0.34536
H	-4.84746	-6.62296	-1.29062
H	-7.49281	-6.19024	-0.83632
H	-6.52587	-5.02920	-1.83832
C	-4.05904	0.49739	2.55826
C	-2.88523	-0.00956	3.41290
C	-5.26129	0.65038	3.50237
H	-3.80098	1.52129	2.21829
C	-3.44173	-0.25705	4.80694
H	-2.41932	-0.93505	3.00858
H	-2.09515	0.77266	3.46536
C	-4.69078	0.59854	4.90922
H	-5.78898	1.61370	3.32767
H	-5.99956	-0.17157	3.38085
H	-2.70329	0.01022	5.59357
H	-3.71740	-1.32962	4.91333
H	-4.41218	1.62466	5.23704
H	-5.41952	0.16750	5.62943
C	2.28077	-3.13998	-1.28418
C	1.81349	-3.84195	0.89120
C	2.03550	-2.23248	-2.34830
C	3.21155	-4.20948	-1.47659
C	2.73832	-4.91522	0.78524
C	2.70864	-2.38005	-3.54044
H	1.27672	-1.46718	-2.22802
C	3.87770	-4.33548	-2.72200
C	3.42706	-5.08962	-0.38871
H	2.87966	-5.58399	1.62451
C	3.63867	-3.43075	-3.73090
H	2.51101	-1.68851	-4.35359
H	4.58323	-5.15024	-2.85933
H	4.13675	-5.90567	-0.49577
H	4.15451	-3.52617	-4.68138
C	0.98061	-3.66140	2.10763
C	-0.26522	-3.18516	2.10917
H	-0.73945	-2.90631	1.17616

H	-0.82616	-3.06269	3.02722
Cl	1.72878	-4.15499	3.63303
N	1.61861	-2.97559	-0.09795

Catalyst (2,4,6-c-Pent, 10)-Enamine protonation TS (pro-S) lowest free energy conformation
Electronic energy (Hartrees): -1870.443224
Free Energy at 1M concentration and 253 K (Hartrees): -1868.980421
Coordinates:

P	0.17483	0.28492	-0.30260
O	-0.95017	1.29297	0.30945
O	1.47813	1.20925	-0.63497
O	-0.21009	-0.37939	-1.58586
O	0.50878	-0.62359	0.91266
C	-1.20514	2.36655	-0.50323
C	-2.51700	2.45205	-1.18537
C	-2.79512	3.50354	-1.96866
H	-3.75690	3.57383	-2.46349
C	-1.77791	4.55349	-2.19232
C	-2.06217	5.66092	-3.13368
H	-3.02508	5.72311	-3.62536
C	-1.12990	6.58661	-3.38392
H	-1.33801	7.39436	-4.07367
C	0.19087	6.50220	-2.73168
H	0.94853	7.24172	-2.95681
C	0.45676	5.50907	-1.87530
H	1.43969	5.45315	-1.42687
C	-0.58023	4.48941	-1.56107
C	-0.30974	3.37289	-0.60540
C	1.71810	2.24810	0.22876
C	2.96177	2.21841	1.03351
C	3.30796	3.28244	1.77155
H	4.21398	3.25913	2.36628
C	2.40939	4.45062	1.86153
C	2.75773	5.57117	2.76538
H	3.69790	5.56437	3.30286
C	1.91113	6.59328	2.92654
H	2.16562	7.41030	3.58928
C	0.61850	6.60110	2.21663
H	-0.07531	7.41671	2.37497
C	0.29617	5.59677	1.39350
H	-0.67123	5.61119	0.91063
C	1.24403	4.46959	1.17129
C	0.91582	3.34054	0.24549
C	3.77328	0.97872	1.08044
C	4.58807	0.58023	-0.11752
C	3.74012	0.16694	2.17521
C	5.29521	-0.56419	-0.07356
C	4.58100	-1.05862	2.19426
C	5.31873	-1.41331	1.13517
H	5.86418	-0.88425	-0.93694
H	4.58254	-1.67934	3.08188
C	-3.51535	1.37864	-0.97006
C	-4.13256	1.24032	0.23577
C	-3.78168	0.38315	-2.06330
C	-5.00999	0.06323	0.46884
C	-4.64546	-0.62407	-1.83267
C	-5.26865	-0.81958	-0.50513
H	-5.45121	-0.07389	1.44805
H	-4.87215	-1.32886	-2.62217

H	0.06760	-2.07539	-1.60529
C	1.66569	-3.14601	-2.33559
C	0.52936	-3.53724	-0.23181
C	1.76387	-2.36140	-3.50144
C	2.63011	-4.14615	-2.05522
C	1.51193	-4.53696	0.07499
C	2.81365	-2.58456	-4.37329
H	1.04022	-1.57141	-3.66945
C	3.67803	-4.36034	-2.97794
C	2.50078	-4.84346	-0.81570
H	1.43722	-5.07352	1.01090
C	3.77168	-3.58861	-4.11986
H	2.90527	-1.96932	-5.26299
H	4.41827	-5.12573	-2.76232
H	3.21502	-5.62831	-0.58110
H	4.58648	-3.74580	-4.81892
C	-0.46119	-3.06458	0.69227
C	-0.66124	-3.75577	2.03711
H	0.33232	-3.99773	2.43623
H	-1.19743	-4.70519	1.93829
N	-1.33864	-2.94051	3.03041
N	0.64516	-2.92770	-1.44024
C	-2.71495	-2.94215	3.27318
C	-3.57572	-3.98810	2.89669
C	-3.26513	-1.86085	3.98942
C	-4.93506	-3.92882	3.20195
H	-3.19947	-4.84050	2.34288
C	-4.62324	-1.80956	4.28116
H	-2.60973	-1.05433	4.31015
C	-5.47703	-2.84172	3.88601
H	-5.57849	-4.74459	2.88291
H	-5.01756	-0.95349	4.82158
H	-6.53862	-2.79916	4.10729
H	-0.92191	-2.02447	3.13287
H	0.10228	-1.69449	0.87694
Cl	-2.04341	-2.64421	-0.07876
C	4.58793	1.40668	-1.40388
C	5.99884	1.83387	-1.84354
C	3.97430	0.62977	-2.58027
H	3.99851	2.33724	-1.27396
C	6.10194	1.50365	-3.32541
H	6.79626	1.29140	-1.29065
H	6.15464	2.92254	-1.67736
C	5.09963	0.39141	-3.57264
H	3.51180	-0.33337	-2.27095
H	3.18521	1.24702	-3.06492
H	7.13117	1.18756	-3.60175
H	5.81920	2.39608	-3.92665
H	5.57550	-0.59365	-3.37102
H	4.72718	0.40663	-4.61982
C	2.85148	0.44245	3.39065
C	1.93058	-0.74174	3.73532
C	3.67805	0.73426	4.64863
H	2.18592	1.31551	3.22688
C	2.28226	-1.16837	5.15281
H	2.03178	-1.59888	3.03386
H	0.86956	-0.40742	3.70978
C	2.95910	0.03227	5.78691
H	4.70987	0.32658	4.56848
H	3.74804	1.82777	4.83840

H	2.99503	-2.02184	5.12012
H	1.37729	-1.47029	5.72346
H	3.66591	-0.27609	6.58743
H	2.18842	0.71027	6.21630
C	6.15304	-2.68163	1.16173
C	5.76212	-3.65245	0.03754
C	7.65042	-2.39244	0.99130
H	6.01742	-3.21174	2.13265
C	6.99656	-3.83219	-0.83281
H	4.91258	-3.28082	-0.57295
H	5.47111	-4.63386	0.47327
C	8.17502	-3.45988	0.04660
H	7.83077	-1.39200	0.53940
H	8.18093	-2.43438	1.96758
H	6.94603	-3.13748	-1.70059
H	7.08318	-4.87570	-1.20575
H	9.02958	-3.08570	-0.55782
H	8.49865	-4.34964	0.63099
C	-3.95937	2.24833	1.37568
C	-5.30417	2.74660	1.93326
C	-3.19136	1.64523	2.55894
H	-3.40320	3.15023	1.04220
C	-5.37085	2.28208	3.38073
H	-6.18267	2.37762	1.36001
H	-5.32982	3.85907	1.90819
C	-3.93055	2.08367	3.81099
H	-3.17729	0.53593	2.50903
H	-2.14009	2.00602	2.58434
H	-5.91356	1.31308	3.44082
H	-5.88526	3.02798	4.02455
H	-3.84786	1.32743	4.62071
H	-3.51418	3.05109	4.16982
C	-6.18555	-2.00137	-0.24211
C	-7.44098	-1.98871	-1.11217
C	-5.52672	-3.34891	-0.51118
H	-6.51709	-2.00437	0.82288
C	-7.81864	-3.45351	-1.33171
H	-7.25041	-1.50878	-2.09711
H	-8.26269	-1.43631	-0.60595
C	-6.68337	-4.30090	-0.76048
H	-4.88481	-3.31891	-1.41764
H	-4.91235	-3.67986	0.35105
H	-7.94024	-3.65786	-2.41824
H	-8.77412	-3.69232	-0.81531
H	-6.39002	-5.11217	-1.46172
H	-7.00318	-4.75417	0.20411
C	-3.10410	0.48817	-3.43006
C	-2.38409	-0.80673	-3.84991
C	-4.10716	0.81410	-4.54571
H	-2.33706	1.28921	-3.42923
C	-3.07891	-1.29689	-5.11064
H	-2.39423	-1.59256	-3.06331
H	-1.32133	-0.58172	-4.09045
C	-3.72602	-0.06861	-5.72196
H	-5.15206	0.58028	-4.24360
H	-4.05879	1.88903	-4.82666
H	-3.86490	-2.03672	-4.84168
H	-2.35970	-1.76801	-5.81523
H	-4.61397	-0.34016	-6.33300
H	-2.98638	0.46142	-6.36228

Catalyst (2,4,6-c-Pent, 10)-Enamine protonation TS (pro-S) alternative conformation
Electronic energy (Hartrees): -1870.439685
Free Energy at 1M concentration and 253 K (Hartrees): -1868.976329
Coordinates:

P	0.49147	0.36659	0.33761
O	1.71601	0.66467	-0.69418
O	-0.14856	1.82119	0.69967
O	0.91214	-0.25971	1.62724
O	-0.53642	-0.41239	-0.53798
C	2.65461	1.50068	-0.14393
C	3.98213	0.95079	0.21910
C	4.91854	1.75545	0.74111
H	5.89493	1.35982	0.99595
C	4.60667	3.17246	1.02476
C	5.61149	4.02245	1.70388
H	6.58700	3.62108	1.94892
C	5.31718	5.28770	2.02177
H	6.05146	5.90735	2.52011
C	3.98486	5.83873	1.70851
H	3.74495	6.85565	1.99114
C	3.07148	5.08143	1.08999
H	2.09263	5.49756	0.89232
C	3.39484	3.68303	0.69779
C	2.39520	2.81927	-0.00126
C	-0.05935	2.80218	-0.25720
C	-1.31846	3.34636	-0.81764
C	-1.29488	4.45959	-1.56430
H	-2.21456	4.84951	-1.98548
C	-0.00721	5.06659	-1.95288
C	0.00314	6.21978	-2.88247
H	-0.92882	6.67075	-3.20012
C	1.16193	6.70500	-3.33931
H	1.16810	7.54586	-4.02078
C	2.43484	6.08055	-2.93450
H	3.36760	6.45606	-3.33524
C	2.43711	5.04187	-2.09119
H	3.38202	4.58011	-1.84062
C	1.15812	4.51660	-1.53617
C	1.13121	3.37282	-0.56948
C	-2.57237	2.56813	-0.68028
C	-3.24385	2.46258	0.65955
C	-3.07256	1.87317	-1.74157
C	-4.32461	1.66979	0.78675
C	-4.32853	1.09949	-1.57301
C	-4.91317	0.97259	-0.37624
H	-4.79548	1.55024	1.75358
H	-4.74939	0.59580	-2.43350
C	4.26282	-0.48318	-0.02427
C	4.39910	-0.95558	-1.29303
C	4.30729	-1.43776	1.13481
C	4.51668	-2.42005	-1.51056
C	4.44416	-2.75587	0.89187
C	4.49102	-3.29020	-0.49062
H	4.58791	-2.78995	-2.52587
H	4.52611	-3.43590	1.72752
H	-0.04873	-1.53174	2.27093
C	-1.54254	-1.56940	3.69143
C	-1.57081	-2.70838	1.55420

C	-0.84683	-0.72699	4.57996
C	-2.87246	-1.97116	3.97383
C	-2.91747	-3.12364	1.82700
C	-1.47167	-0.30955	5.74090
H	0.15014	-0.39075	4.32044
C	-3.47797	-1.53113	5.17066
C	-3.52885	-2.78259	2.99899
H	-3.43554	-3.70032	1.07367
C	-2.78790	-0.71334	6.04507
H	-0.94207	0.34930	6.42201
H	-4.49741	-1.83908	5.38524
H	-4.54023	-3.12587	3.20023
H	-3.25927	-0.37216	6.96090
C	-0.91443	-2.92501	0.29874
C	-1.59123	-3.75994	-0.77589
H	-1.82974	-4.77550	-0.42173
H	-0.88122	-3.87633	-1.60712
N	-2.82573	-3.10734	-1.20133
N	-0.94768	-1.98918	2.52361
C	-3.55041	-3.57060	-2.30080
C	-4.56920	-2.75849	-2.83508
C	-3.32828	-4.83263	-2.87852
C	-5.33088	-3.18833	-3.91367
H	-4.75510	-1.78780	-2.38698
C	-4.10746	-5.25699	-3.95660
H	-2.54809	-5.48073	-2.49547
C	-5.10870	-4.44493	-4.48592
H	-6.10482	-2.53663	-4.30981
H	-3.91710	-6.23536	-4.38950
H	-5.70455	-4.78091	-5.32852
H	-2.77262	-2.09801	-1.14599
H	-0.72428	-1.45601	-0.21694
Cl	0.83352	-3.37653	0.43872
C	-2.71291	3.20038	1.88808
C	-3.75191	4.15285	2.50183
C	-2.28465	2.23378	3.00598
H	-1.83027	3.81879	1.62402
C	-3.69148	3.93062	4.00434
H	-4.78261	3.93771	2.14453
H	-3.51932	5.21174	2.25377
C	-3.20124	2.50678	4.18769
H	-1.23331	2.44600	3.30270
H	-2.34306	1.16494	2.70341
H	-2.95897	4.63565	4.45633
H	-4.68539	4.08003	4.47909
H	-2.66580	2.38736	5.15413
H	-4.06557	1.80682	4.16078
C	-6.12975	0.07910	-0.20475
C	-5.81788	-1.11573	0.70449
C	-7.32065	0.81766	0.42651
H	-6.45645	-0.31814	-1.19318
C	-6.61099	-0.90333	1.98230
H	-4.73361	-1.21283	0.92407
H	-6.15581	-2.05655	0.21554
C	-7.79891	-0.04740	1.58420
H	-8.13759	0.95724	-0.31500
H	-7.03690	1.82189	0.81067
H	-6.93769	-1.87037	2.42225
H	-5.98766	-0.35906	2.72567
H	-8.62871	-0.70420	1.24085

H	-8.15230	0.57218	2.43678
C	-2.36670	1.79369	-3.09876
C	-2.07908	0.34475	-3.53066
C	-3.20150	2.42755	-4.21811
H	-1.38557	2.31103	-3.08982
C	-2.81987	0.12689	-4.84175
H	-2.39386	-0.40723	-2.77771
H	-0.98680	0.21153	-3.69929
C	-3.04698	1.51124	-5.41930
H	-2.84275	3.45340	-4.45407
H	-4.27844	2.48877	-3.94709
H	-2.23509	-0.51136	-5.53898
H	-3.80144	-0.35653	-4.64142
H	-2.15602	1.81825	-6.01082
H	-3.94772	1.53638	-6.07011
C	4.22593	-0.95000	2.58244
C	3.14365	-1.66933	3.40796
C	5.55187	-1.14959	3.33016
H	3.98306	0.13097	2.62161
C	3.86855	-2.40050	4.52697
H	2.52708	-2.37492	2.81027
H	2.45944	-0.91710	3.85711
C	5.18069	-1.66218	4.71124
H	6.11905	-0.19575	3.40220
H	6.19947	-1.90255	2.82874
H	3.27157	-2.40436	5.46467
H	4.07185	-3.44986	4.21886
H	5.02961	-0.80201	5.40063
H	5.96730	-2.33057	5.12359
C	4.39031	-0.04657	-2.52438
C	5.66472	-0.18782	-3.36861
C	3.20619	-0.35090	-3.45698
H	4.32336	1.02625	-2.24676
C	5.20476	-0.17309	-4.81587
H	6.19859	-1.14186	-3.16841
H	6.37138	0.64823	-3.17216
C	3.80229	-0.75169	-4.79846
H	2.58747	0.56514	-3.58724
H	2.54185	-1.15539	-3.07034
H	5.16945	0.87575	-5.18564
H	5.88157	-0.76961	-5.46523
H	3.19542	-0.36484	-5.64560
H	3.85815	-1.86079	-4.86557
C	4.56487	-4.78544	-0.78394
C	4.34574	-5.70276	0.41673
C	3.50999	-5.24845	-1.79395
H	5.57224	-5.00376	-1.20651
C	3.95860	-7.04181	-0.18405
H	3.49256	-5.34481	1.03547
H	5.26093	-5.78722	1.04219
C	3.23340	-6.71952	-1.48659
H	3.87674	-5.12597	-2.83650
H	2.57034	-4.66456	-1.67370
H	4.87729	-7.63078	-0.40085
H	3.31073	-7.62089	0.50949
H	3.60886	-7.36922	-2.30728
H	2.13984	-6.88612	-1.36990

Catalyst (2,4,6-c-Pent, 10)-Enamine protonation TS (pro-S) alternative conformation

Electronic energy (Hartrees): -1870.439375
Free Energy at 1M concentration and 253 K (Hartrees): -1868.976119
Coordinates:

P	0.59216	0.32714	0.27542
O	1.72277	0.47959	-0.88794
O	0.05462	1.84128	0.57482
O	1.11428	-0.20459	1.57160
O	-0.53809	-0.47784	-0.42883
C	2.74555	1.30666	-0.49990
C	4.07265	0.71801	-0.20158
C	5.09396	1.51394	0.14473
H	6.06908	1.08835	0.35164
C	4.87965	2.96712	0.31157
C	5.98661	3.81908	0.80368
H	6.96243	3.38843	0.99095
C	5.78497	5.12267	1.02366
H	6.59403	5.74391	1.38551
C	4.45434	5.71541	0.78978
H	4.29139	6.76562	0.99490
C	3.44866	4.95618	0.33982
H	2.47477	5.40609	0.20072
C	3.66554	3.51178	0.05521
C	2.56137	2.64400	-0.45759
C	0.09326	2.74423	-0.46044
C	-1.18680	3.34321	-0.90657
C	-1.18547	4.39314	-1.73974
H	-2.12322	4.82880	-2.06521
C	0.07969	4.86767	-2.33251
C	0.04929	5.94001	-3.35384
H	-0.88459	6.42950	-3.60096
C	1.17278	6.30421	-3.98013
H	1.14891	7.08600	-4.72828
C	2.44474	5.62594	-3.66942
H	3.34446	5.90253	-4.20385
C	2.48430	4.65993	-2.74459
H	3.42275	4.15501	-2.56137
C	1.25075	4.27140	-2.00520
C	1.27092	3.21278	-0.94585
C	-2.46158	2.70719	-0.50131
C	-2.92593	2.82778	0.92152
C	-3.16098	1.93442	-1.37957
C	-4.00797	2.13074	1.31866
C	-4.38421	1.23385	-0.90826
C	-4.77990	1.30263	0.37205
H	-4.34098	2.18869	2.34792
H	-4.95110	0.63513	-1.61109
C	4.25171	-0.74890	-0.30319
C	4.23623	-1.36478	-1.51623
C	4.34679	-1.57305	0.94949
C	4.22907	-2.84879	-1.57340
C	4.36593	-2.91636	0.84859
C	4.23605	-3.59843	-0.46162
H	4.17383	-3.33092	-2.54159
H	4.48561	-3.50666	1.74570
H	0.09626	-1.29154	2.46913
C	-1.32698	-0.91260	3.91322
C	-1.60859	-2.30335	1.95232
C	-0.46368	-0.12348	4.69725
C	-2.69912	-1.03135	4.24803
C	-3.00345	-2.41863	2.26650

C	-0.96742	0.51968	5.81277
H	0.57080	-0.00910	4.39543
C	-3.17963	-0.36271	5.39526
C	-3.51762	-1.81249	3.37704
H	-3.63065	-2.98404	1.59114
C	-2.32609	0.39808	6.17081
H	-0.30726	1.13569	6.41533
H	-4.23110	-0.45320	5.65253
H	-4.57195	-1.92480	3.61509
H	-2.70034	0.91336	7.04932
C	-1.02352	-2.81555	0.74354
C	-1.80831	-3.80906	-0.10497
H	-2.14984	-4.66424	0.49722
H	-1.12788	-4.21334	-0.86755
N	-2.97001	-3.16029	-0.69999
N	-0.84949	-1.56738	2.80132
C	-3.80673	-3.84107	-1.58793
C	-4.70703	-3.10322	-2.38056
C	-3.81633	-5.24280	-1.69572
C	-5.58982	-3.74343	-3.24062
H	-4.70966	-2.02010	-2.30674
C	-4.71231	-5.87449	-2.56004
H	-3.12639	-5.84246	-1.11302
C	-5.60419	-5.13821	-3.33764
H	-6.27182	-3.14662	-3.84004
H	-4.70164	-6.95903	-2.62671
H	-6.29421	-5.63792	-4.00998
H	-2.77510	-2.20628	-0.97231
H	-0.76043	-1.49668	0.02438
Cl	0.67609	-3.41596	0.94875
C	-2.19555	3.71609	1.92810
C	-3.08959	4.82915	2.49421
C	-1.66179	2.92250	3.13185
H	-1.32767	4.21962	1.45402
C	-2.76318	4.90794	3.97535
H	-4.17082	4.59942	2.37469
H	-2.88623	5.80129	1.99386
C	-2.33069	3.50765	4.36662
H	-0.55973	3.05455	3.21161
H	-1.86846	1.83344	3.05606
H	-1.91951	5.61558	4.13478
H	-3.64113	5.24194	4.56964
H	-1.63753	3.52664	5.23527
H	-3.22706	2.90243	4.62717
C	-6.01491	0.56563	0.86057
C	-7.30162	1.08157	0.20323
C	-5.95849	-0.94731	0.59215
H	-6.11750	0.70930	1.96031
C	-8.14923	-0.14971	-0.06217
H	-7.09552	1.59113	-0.76404
H	-7.83593	1.79439	0.86856
C	-7.15637	-1.27355	-0.28878
H	-6.05140	-1.50078	1.55226
H	-5.01346	-1.26827	0.10466
H	-8.77082	-0.37630	0.83238
H	-8.81030	-0.00780	-0.94447
H	-7.59564	-2.26162	-0.03158
H	-6.84785	-1.27712	-1.35667
C	-2.73070	1.72914	-2.83554
C	-2.54989	0.25071	-3.21193

C	-3.74853	2.30611	-3.82170
H	-1.76109	2.21807	-3.05936
C	-3.37850	0.01381	-4.47012
H	-2.87121	-0.43722	-2.40686
H	-1.47750	0.04021	-3.42289
C	-3.69984	1.38860	-5.02842
H	-3.49019	3.35046	-4.10299
H	-4.77893	2.29592	-3.40328
H	-2.82507	-0.60194	-5.21179
H	-4.32623	-0.50324	-4.20386
H	-2.88430	1.71393	-5.71185
H	-4.66511	1.38877	-5.57961
C	4.45181	-0.92685	2.33219
C	3.41864	-1.46530	3.33876
C	5.83368	-1.14635	2.96362
H	4.28677	0.16747	2.27021
C	4.20913	-2.11388	4.46447
H	2.70238	-2.18889	2.89344
H	2.83064	-0.61851	3.75521
C	5.57869	-1.46297	4.42718
H	6.47047	-0.24054	2.86026
H	6.36870	-2.00352	2.49803
H	3.71582	-1.96177	5.44893
H	4.31024	-3.20465	4.27086
H	5.55783	-0.51875	5.01540
H	6.35941	-2.13734	4.84096
C	4.17435	-0.59593	-2.83800
C	5.35341	-0.92558	-3.76418
C	2.89198	-0.90702	-3.62748
H	4.20537	0.50257	-2.67933
C	4.76493	-1.03006	-5.16022
H	5.83645	-1.89175	-3.50227
H	6.13064	-0.13112	-3.72343
C	3.33427	-1.49379	-4.96005
H	2.32668	0.03448	-3.80866
H	2.21366	-1.61211	-3.09768
H	4.76655	-0.02712	-5.64202
H	5.33769	-1.74287	-5.79221
H	2.67973	-1.15427	-5.79184
H	3.30903	-2.60469	-4.90544
C	4.15991	-5.11671	-0.58680
C	4.00328	-5.87920	0.72673
C	2.96644	-5.58512	-1.42579
H	5.09417	-5.46877	-1.08104
C	3.44684	-7.23272	0.32442
H	3.25485	-5.38325	1.38433
H	4.97258	-5.98051	1.26157
C	2.60475	-6.98184	-0.92219
H	3.22630	-5.61091	-2.50662
H	2.09658	-4.90578	-1.28276
H	4.28599	-7.92089	0.07922
H	2.83945	-7.67691	1.14286
H	2.82551	-7.74826	-1.69706
H	1.52216	-7.02819	-0.67079

Catalyst (2,4,6-c-Pent, 10)-Enamine protonation TS (pro-S) alternative conformation
Electronic energy (Hartrees): -1870.438123
Free Energy at 1M concentration and 253 K (Hartrees): -1868.975949
Coordinates:

P	-0.09267	-0.08551	-0.38967
O	-1.40925	0.66915	0.24574
O	0.89294	1.08657	-0.96321
O	-0.39614	-1.04881	-1.49226
O	0.57367	-0.66092	0.88421
C	-1.82757	1.80543	-0.39810
C	-3.19110	1.82820	-0.97384
C	-3.65818	2.94026	-1.55696
H	-4.65859	2.95726	-1.97377
C	-2.80882	4.14638	-1.64205
C	-3.30693	5.34590	-2.35362
H	-4.30041	5.34922	-2.78458
C	-2.53099	6.42881	-2.47032
H	-2.89513	7.30417	-2.99247
C	-1.17115	6.42998	-1.89808
H	-0.54090	7.30220	-2.01466
C	-0.71332	5.35411	-1.24762
H	0.29507	5.37032	-0.85633
C	-1.57377	4.15047	-1.08557
C	-1.08492	2.93500	-0.36684
C	1.17049	2.05104	-0.02621
C	2.54372	2.15325	0.52209
C	2.81199	3.04215	1.48930
H	3.81922	3.13665	1.87823
C	1.73802	3.90714	2.02196
C	2.01800	4.80591	3.16475
H	3.01109	4.84586	3.59463
C	1.04310	5.56401	3.67782
H	1.24717	6.21613	4.51715
C	-0.31665	5.50743	3.10763
H	-1.10464	6.11122	3.53889
C	-0.57840	4.70639	2.06839
H	-1.58711	4.66506	1.67947
C	0.49898	3.86980	1.47523
C	0.22244	2.93703	0.34298
C	3.62768	1.35693	-0.09535
C	4.05295	1.68623	-1.49945
C	4.17737	0.28982	0.54531
C	4.91588	0.86760	-2.13109
C	5.18359	-0.53688	-0.16806
C	5.50766	-0.29776	-1.44678
H	5.19893	1.06565	-3.15787
H	5.62140	-1.38456	0.34169
C	-4.04097	0.62439	-0.85871
C	-4.64257	0.31393	0.32075
C	-4.15627	-0.30920	-2.02592
C	-5.38623	-0.96736	0.43851
C	-4.87163	-1.44128	-1.88734
C	-5.52615	-1.79759	-0.60981
H	-5.78898	-1.22563	1.40656
H	-4.96015	-2.12629	-2.72130
H	-0.11251	-2.72308	-0.72431
C	1.46351	-3.99744	-1.03978
C	0.36972	-3.59841	1.08236
C	1.52485	-3.70798	-2.41708
C	2.42484	-4.84477	-0.43223
C	1.35359	-4.42206	1.71416
C	2.51951	-4.29279	-3.17860
H	0.80936	-3.01141	-2.84186
C	3.42353	-5.42894	-1.24323

C	2.33010	-5.03329	0.97886
H	1.31185	-4.53773	2.78756
C	3.46465	-5.16591	-2.59840
H	2.57795	-4.06965	-4.23931
H	4.16049	-6.07983	-0.78155
H	3.05720	-5.67410	1.47045
H	4.23660	-5.61234	-3.21668
C	-0.59494	-2.80350	1.79676
C	-0.82069	-2.98594	3.30038
H	-1.00313	-4.03873	3.55396
H	-1.74124	-2.44402	3.55384
N	0.30511	-2.53549	4.10679
N	0.47261	-3.44482	-0.26236
C	0.50858	-1.18394	4.42717
C	1.50526	-0.85322	5.36425
C	-0.20432	-0.14204	3.81306
C	1.79224	0.47514	5.65874
H	2.06902	-1.65075	5.84353
C	0.09486	1.18625	4.11515
H	-0.96147	-0.35275	3.07063
C	1.09223	1.51037	5.03249
H	2.57349	0.70210	6.37894
H	-0.46547	1.97181	3.61630
H	1.32246	2.54722	5.25678
H	0.54941	-3.16327	4.85911
H	0.02385	-1.55436	1.41712
Cl	-2.20313	-2.71558	0.94857
C	3.77722	-0.13828	1.95745
C	3.36848	-1.61954	2.02574
C	4.93583	0.03996	2.94785
H	2.91661	0.45139	2.33844
C	4.37922	-2.31504	2.92272
H	3.31629	-2.11064	1.02947
H	2.36714	-1.70628	2.48972
C	4.97506	-1.22360	3.79138
H	4.77832	0.93532	3.58854
H	5.91146	0.15428	2.42510
H	3.89725	-3.10411	3.54020
H	5.17769	-2.77718	2.30185
H	4.34938	-1.08585	4.69952
H	6.01299	-1.47493	4.09957
C	3.51344	2.91731	-2.22957
C	4.61750	3.87779	-2.69019
C	2.69385	2.55326	-3.47829
H	2.85254	3.51391	-1.56771
C	4.14617	4.42161	-4.02643
H	5.59110	3.36194	-2.83462
H	4.76664	4.70144	-1.95791
C	3.32120	3.30678	-4.64408
H	1.63952	2.88417	-3.34449
H	2.68213	1.46067	-3.68665
H	3.50156	5.31319	-3.85996
H	5.00222	4.70229	-4.67753
H	2.54677	3.71215	-5.33077
H	3.99089	2.62329	-5.21174
C	6.43227	-1.22939	-2.21079
C	7.82705	-1.33847	-1.57429
C	5.86825	-2.65615	-2.28971
H	6.55663	-0.85251	-3.25118
C	8.15104	-2.82372	-1.51261

H	7.85145	-0.91683	-0.54542
H	8.58540	-0.79934	-2.18320
C	6.81166	-3.53631	-1.48732
H	5.86162	-2.99780	-3.34868
H	4.83073	-2.73344	-1.89682
H	8.71135	-3.12162	-2.42650
H	8.75708	-3.07183	-0.61441
H	6.88586	-4.55583	-1.92400
H	6.44947	-3.61506	-0.43838
C	-3.48227	-0.00497	-3.36391
C	-2.55526	-1.13577	-3.84518
C	-4.50392	0.23257	-4.48433
H	-2.85822	0.90979	-3.29269
C	-3.11216	-1.61272	-5.17841
H	-2.48287	-1.98134	-3.12646
H	-1.52869	-0.73606	-4.00321
C	-3.93960	-0.45893	-5.71285
H	-4.64813	1.31906	-4.67265
H	-5.49391	-0.21391	-4.24331
H	-2.29925	-1.88810	-5.88476
H	-3.76897	-2.49558	-5.01462
H	-3.28120	0.24522	-6.26845
H	-4.74764	-0.81725	-6.38683
C	-4.56583	1.21960	1.55281
C	-5.95263	1.64761	2.05262
C	-3.86310	0.53424	2.73510
H	-4.00898	2.15726	1.34341
C	-5.88540	1.57535	3.56804
H	-6.75624	0.96839	1.69413
H	-6.19937	2.67915	1.71810
C	-4.87085	0.48963	3.87384
H	-2.98438	1.14097	3.04720
H	-3.50337	-0.48955	2.49501
H	-5.52353	2.54671	3.97243
H	-6.87772	1.33930	4.00979
H	-4.38123	0.65934	4.85727
H	-5.37866	-0.50018	3.88440
C	-6.25183	-3.13575	-0.52361
C	-7.00680	-3.42365	0.77321
C	-7.31936	-3.29328	-1.60594
H	-5.49336	-3.93946	-0.66412
C	-7.99398	-4.51893	0.40887
H	-7.58918	-2.53259	1.09774
H	-6.31989	-3.74706	1.58540
C	-8.32164	-4.31378	-1.06816
H	-6.87364	-3.64330	-2.56248
H	-7.83820	-2.32372	-1.78066
H	-7.51605	-5.51309	0.55395
H	-8.90786	-4.46048	1.03939
H	-8.23013	-5.27617	-1.61792
H	-9.35932	-3.93163	-1.18639

Catalyst (2,4,6-c-Pent, 10)-Enamine protonation TS (pro-S) alternative conformation
Electronic energy (Hartrees): -1870.437633
Free Energy at 1M concentration and 253 K (Hartrees): -1868.974845
Coordinates:

P	0.34093	0.43803	0.36285
O	1.50837	0.92324	-0.66344
O	-0.44558	1.79320	0.80771

O	0.84556	-0.20647	1.61261
O	-0.60968	-0.40007	-0.54470
C	2.34999	1.84191	-0.08901
C	3.73917	1.43835	0.23548
C	4.58516	2.33023	0.77031
H	5.60341	2.04138	1.00391
C	4.11664	3.69126	1.10706
C	5.02903	4.62945	1.80047
H	6.05002	4.33779	2.01330
C	4.59496	5.83878	2.17135
H	5.26191	6.52276	2.68011
C	3.20043	6.23877	1.90308
H	2.84854	7.20909	2.22898
C	2.36923	5.40115	1.27235
H	1.34389	5.70492	1.10897
C	2.84646	4.06606	0.82068
C	1.94068	3.11562	0.10627
C	-0.49805	2.81243	-0.11020
C	-1.82452	3.21360	-0.63343
C	-1.95419	4.35256	-1.32880
H	-2.92195	4.63851	-1.72492
C	-0.75932	5.13201	-1.70713
C	-0.91012	6.31543	-2.58511
H	-1.89688	6.66100	-2.86743
C	0.17048	6.95817	-3.03909
H	0.05903	7.82104	-3.68293
C	1.51868	6.47856	-2.68346
H	2.38959	6.98213	-3.08291
C	1.66624	5.41374	-1.88695
H	2.66566	5.06130	-1.67305
C	0.47349	4.71282	-1.33412
C	0.60733	3.53580	-0.41749
C	-2.96228	2.26833	-0.53233
C	-3.60552	2.00820	0.80178
C	-3.37150	1.56581	-1.62756
C	-4.57660	1.07961	0.88442
C	-4.51038	0.62259	-1.49859
C	-5.06787	0.36276	-0.31079
H	-5.03602	0.85716	1.83477
H	-4.86137	0.10581	-2.38232
C	4.17468	0.04630	-0.02969
C	4.31147	-0.40864	-1.30496
C	4.38859	-0.89428	1.12274
C	4.63658	-1.83996	-1.53093
C	4.74278	-2.16986	0.87128
C	4.87785	-2.68025	-0.51442
H	4.67709	-2.20881	-2.54854
H	4.88767	-2.84275	1.70316
H	0.07001	-1.64436	2.14815
C	-1.39236	-2.01059	3.55577
C	-1.26300	-2.98354	1.34154
C	-0.83098	-1.13167	4.50208
C	-2.63774	-2.63912	3.80813
C	-2.52551	-3.62352	1.58188
C	-1.50032	-0.90421	5.69069
H	0.09472	-0.62042	4.26548
C	-3.29021	-2.39060	5.03504
C	-3.16894	-3.47086	2.77607
H	-2.95560	-4.21699	0.78746
C	-2.72939	-1.53773	5.96659

H	-1.07586	-0.21771	6.41662
H	-4.24446	-2.87280	5.22757
H	-4.11117	-3.98351	2.95101
H	-3.23783	-1.34424	6.90541
C	-0.59675	-3.00087	0.07249
C	-1.15377	-3.84196	-1.06432
H	-1.22228	-4.90833	-0.79587
H	-0.45003	-3.77546	-1.90622
N	-2.48317	-3.36120	-1.42878
N	-0.75005	-2.24739	2.36155
C	-3.14109	-3.83676	-2.56435
C	-4.27880	-3.14952	-3.02944
C	-2.73485	-4.99699	-3.24608
C	-4.97820	-3.60005	-4.14127
H	-4.60641	-2.25964	-2.50176
C	-3.45263	-5.44505	-4.35667
H	-1.86032	-5.54683	-2.91724
C	-4.57281	-4.75541	-4.81696
H	-5.84750	-3.04439	-4.48211
H	-3.11979	-6.34317	-4.86991
H	-5.12012	-5.10770	-5.68543
H	-2.59184	-2.36538	-1.28383
H	-0.63746	-1.48448	-0.31806
Cl	1.20256	-3.19023	0.17076
C	-3.16522	2.75282	2.06479
C	-4.32477	3.47035	2.76239
C	-2.56262	1.80946	3.10988
H	-2.40509	3.52017	1.81126
C	-4.70042	2.64257	3.98834
H	-5.20043	3.61121	2.09065
H	-3.98902	4.47802	3.09488
C	-3.63133	1.56888	4.16740
H	-1.68406	2.29913	3.58594
H	-2.21984	0.84831	2.66664
H	-4.75312	3.29140	4.89005
H	-5.69580	2.16881	3.84071
H	-3.18976	1.61845	5.18664
H	-4.07810	0.55960	4.03038
C	-6.14566	-0.70024	-0.18175
C	-5.65906	-1.88177	0.66603
C	-7.42230	-0.16912	0.48980
H	-6.42228	-1.09000	-1.18818
C	-6.44480	-1.83260	1.96471
H	-4.56655	-1.84424	0.86355
H	-5.88149	-2.83660	0.13949
C	-7.74685	-1.13126	1.62470
H	-8.26374	-0.13202	-0.23640
H	-7.28672	0.85498	0.90144
H	-6.62712	-2.85270	2.36674
H	-5.88465	-1.24054	2.72155
H	-8.49008	-1.88205	1.27576
H	-8.15700	-0.59516	2.50792
C	-2.67152	1.65069	-2.98790
C	-2.19783	0.27772	-3.49732
C	-3.59093	2.22666	-4.07130
H	-1.76702	2.29162	-2.95310
C	-2.90253	0.03958	-4.82488
H	-2.41400	-0.54964	-2.79052
H	-1.09714	0.29605	-3.66311
C	-3.31511	1.41200	-5.32274

H	-3.37949	3.30445	-4.24557
H	-4.66469	2.12071	-3.80139
H	-2.23700	-0.47184	-5.55355
H	-3.80982	-0.58263	-4.66073
H	-2.47429	1.87067	-5.88898
H	-4.21160	1.35351	-5.97726
C	4.21152	-0.44221	2.57386
C	3.24426	-1.33249	3.37491
C	5.54190	-0.44021	3.34050
H	3.80017	0.58637	2.61997
C	4.06160	-1.96229	4.49177
H	2.74725	-2.11294	2.75846
H	2.44997	-0.70057	3.82798
C	5.23931	-1.03089	4.70724
H	5.94724	0.59085	3.43709
H	6.30917	-1.06993	2.83797
H	3.46165	-2.07704	5.42053
H	4.43048	-2.96097	4.16955
H	4.94693	-0.21824	5.40875
H	6.11617	-1.57532	5.11983
C	4.12839	0.48563	-2.53355
C	5.37568	0.51793	-3.42817
C	2.96167	0.01781	-3.41945
H	3.92626	1.54010	-2.25215
C	4.86004	0.47301	-4.85577
H	6.03985	-0.35662	-3.25649
H	5.97145	1.44076	-3.25389
C	3.55234	-0.29331	-4.78703
H	2.21480	0.83712	-3.51843
H	2.43423	-0.87289	-3.01139
H	4.66593	1.50795	-5.21529
H	5.58561	-0.02303	-5.53627
H	2.86409	0.00850	-5.60607
H	3.75757	-1.38390	-4.86560
C	5.18553	-4.14147	-0.82244
C	6.39154	-4.31345	-1.74653
C	5.52304	-5.02180	0.37746
H	4.29336	-4.57944	-1.32675
C	6.93265	-5.71701	-1.47765
H	7.17582	-3.56216	-1.50120
H	6.10027	-4.19856	-2.81340
C	6.23557	-6.22434	-0.21727
H	4.61053	-5.32235	0.93701
H	6.23688	-4.50622	1.05748
H	6.71142	-6.39014	-2.33484
H	8.03371	-5.68135	-1.32538
H	5.48814	-7.00288	-0.48760
H	6.96375	-6.65558	0.50380

Catalyst (2,4,6-c-Pent, 10)-Enamine protonation TS (pro-S) alternative conformation
Electronic energy (Hartrees): -1870.437933
Free Energy at 1M concentration and 253 K (Hartrees): -1868.97449
Coordinates:

P	0.40356	0.45949	0.32332
O	1.49070	0.71644	-0.86167
O	-0.33906	1.89342	0.56367
O	1.00820	0.06291	1.63191
O	-0.61267	-0.52234	-0.32914
C	2.38759	1.69913	-0.52874

C	3.78915	1.32146	-0.22681
C	4.68712	2.27216	0.06815
H	5.71420	2.00151	0.28420
C	4.26747	3.68584	0.17120
C	5.24442	4.70992	0.60746
H	6.27383	4.43309	0.79836
C	4.85884	5.97975	0.77253
H	5.57310	6.72689	1.09363
C	3.45447	6.36359	0.53377
H	3.14356	7.38783	0.69452
C	2.56476	5.44760	0.13414
H	1.53486	5.74581	-0.00947
C	2.98525	4.03779	-0.08950
C	2.01323	2.99744	-0.54491
C	-0.44171	2.73623	-0.51652
C	-1.79808	3.09877	-0.98790
C	-1.96106	4.09325	-1.87139
H	-2.95416	4.34965	-2.22234
C	-0.78505	4.73497	-2.49022
C	-0.98242	5.74041	-3.55985
H	-1.98118	6.06719	-3.82159
C	0.07025	6.24421	-4.21183
H	-0.07540	6.97678	-4.99516
C	1.43219	5.78570	-3.88143
H	2.27740	6.17200	-4.43642
C	1.62213	4.88189	-2.91340
H	2.62759	4.53645	-2.71665
C	0.46483	4.34311	-2.14536
C	0.64963	3.35138	-1.03790
C	-2.94996	2.26279	-0.57999
C	-3.49332	2.37893	0.81510
C	-3.46150	1.32994	-1.43204
C	-4.49039	1.55865	1.19953
C	-4.57356	0.46150	-0.96660
C	-5.07096	0.56569	0.27515
H	-4.87735	1.61365	2.20980
H	-4.98731	-0.26992	-1.64843
C	4.17803	-0.10904	-0.24094
C	4.21635	-0.80902	-1.40790
C	4.45576	-0.81208	1.05773
C	4.49574	-2.26853	-1.36951
C	4.76456	-2.12241	1.03764
C	4.79038	-2.89839	-0.21970
H	4.43438	-2.81527	-2.29872
H	4.97445	-2.63971	1.96507
H	0.19064	-1.15837	2.52570
C	-1.24418	-1.00930	3.99723
C	-1.30277	-2.49777	2.08858
C	-0.54626	-0.01140	4.70463
C	-2.54570	-1.40079	4.39870
C	-2.62757	-2.89348	2.47257
C	-1.14071	0.57067	5.80905
H	0.42567	0.30871	4.34837
C	-3.11989	-0.79068	5.53543
C	-3.20904	-2.37697	3.59505
H	-3.15115	-3.59873	1.84185
C	-2.42733	0.17964	6.23384
H	-0.61116	1.34898	6.34964
H	-4.11750	-1.09057	5.84345
H	-4.20055	-2.70854	3.89166

H	-2.87513	0.65127	7.10240
C	-0.67986	-2.90550	0.85989
C	-1.31305	-4.01384	0.02858
H	-1.46451	-4.92782	0.62325
H	-0.61149	-4.27503	-0.77588
N	-2.60039	-3.57248	-0.49797
N	-0.67710	-1.60099	2.89156
C	-3.30856	-4.34004	-1.42546
C	-4.33720	-3.73717	-2.17537
C	-3.05981	-5.71070	-1.61549
C	-5.09313	-4.47917	-3.07376
H	-4.54381	-2.67994	-2.04029
C	-3.82942	-6.44599	-2.51861
H	-2.26818	-6.20476	-1.06353
C	-4.84885	-5.84388	-3.25391
H	-5.87899	-3.98495	-3.63838
H	-3.61870	-7.50404	-2.64866
H	-5.43969	-6.42274	-3.95654
H	-2.60837	-2.58175	-0.69805
H	-0.66318	-1.55204	0.12740
Cl	1.10769	-3.18590	0.98982
C	-2.92178	3.38251	1.81596
C	-3.97394	4.37695	2.32971
C	-2.32253	2.69295	3.05322
H	-2.11500	3.98681	1.35207
C	-3.72489	4.51104	3.82251
H	-5.01135	4.01191	2.16723
H	-3.87404	5.36280	1.82480
C	-3.11610	3.18940	4.25193
H	-1.25620	2.98927	3.16959
H	-2.36145	1.58347	2.99248
H	-2.99712	5.33215	4.00711
H	-4.66551	4.72233	4.37578
H	-2.46759	3.31513	5.14581
H	-3.92702	2.46535	4.48837
C	-6.20941	-0.32131	0.75159
C	-7.43440	-0.27516	-0.17680
C	-5.79211	-1.79426	0.85887
H	-6.53581	0.01434	1.76206
C	-7.57462	-1.66949	-0.76837
H	-7.34852	0.48847	-0.98086
H	-8.34390	-0.04108	0.42009
C	-6.91676	-2.60105	0.23140
H	-5.64816	-2.09081	1.91954
H	-4.84680	-1.99396	0.31171
H	-8.64095	-1.93803	-0.93016
H	-7.03338	-1.72262	-1.73874
H	-7.65448	-2.88394	1.01471
H	-6.53584	-3.52199	-0.25976
C	-2.93701	1.12622	-2.85772
C	-2.48746	-0.31507	-3.14172
C	-3.99288	1.46535	-3.91173
H	-2.05688	1.76468	-3.07565
C	-3.19233	-0.75135	-4.42180
H	-2.73353	-1.00957	-2.31681
H	-1.38464	-0.34798	-3.28900
C	-3.72462	0.51709	-5.06462
H	-3.91076	2.52516	-4.23824
H	-5.02384	1.28916	-3.53365
H	-2.49877	-1.28735	-5.10534

H	-4.04431	-1.42077	-4.17154
H	-2.94761	0.95176	-5.73195
H	-4.64704	0.31937	-5.65251
C	4.40323	-0.07918	2.39944
C	3.49525	-0.76726	3.43501
C	5.79226	0.03658	3.04324
H	4.00858	0.94917	2.27223
C	4.39426	-1.18321	4.58873
H	2.94280	-1.64182	3.02797
H	2.74560	-0.03832	3.81230
C	5.59561	-0.25972	4.52024
H	6.21811	1.05354	2.89780
H	6.50644	-0.70399	2.61963
H	3.87104	-1.09420	5.56548
H	4.72495	-2.23560	4.44591
H	5.37007	0.68462	5.06373
H	6.49716	-0.73400	4.96498
C	3.96509	-0.16465	-2.77361
C	5.14638	-0.34954	-3.73679
C	2.72909	-0.75522	-3.47253
H	3.80533	0.93071	-2.69183
C	4.53048	-0.65532	-5.09075
H	5.80341	-1.19544	-3.43976
H	5.77132	0.56908	-3.78728
C	3.21530	-1.34821	-4.78707
H	1.99531	0.05480	-3.68298
H	2.21218	-1.52971	-2.86345
H	4.33299	0.29528	-5.63425
H	5.19581	-1.29946	-5.70560
H	2.47747	-1.18919	-5.60311
H	3.39174	-2.43949	-4.66179
C	5.05964	-4.39688	-0.13360
C	5.18946	-5.14677	-1.45857
C	6.36665	-4.71593	0.59171
H	4.22281	-4.85684	0.44017
C	5.92032	-6.43035	-1.10479
H	5.82406	-4.57771	-2.17414
H	4.19590	-5.35496	-1.91194
C	6.80300	-6.09342	0.09447
H	6.22376	-4.72099	1.69433
H	7.14660	-3.96551	0.33025
H	5.18156	-7.21226	-0.82066
H	6.52433	-6.79702	-1.96322
H	6.67060	-6.85559	0.89348
H	7.87376	-6.07179	-0.20527

Catalyst (2,4,6-c-Pent, 10)-Enamine protonation TS (pro-S) alternative conformation
Electronic energy (Hartrees): -1870.435862
Free Energy at 1M concentration and 253 K (Hartrees): -1868.972774
Coordinates:

P	-0.12761	-0.21427	-0.33168
O	-1.44821	0.60784	0.19715
O	1.00442	0.89647	-0.72573
O	-0.37061	-1.08482	-1.52433
O	0.34911	-0.91541	0.96415
C	-1.68890	1.82033	-0.39728
C	-2.96063	2.00534	-1.13266
C	-3.25892	3.19367	-1.67484
H	-4.19026	3.32785	-2.21293

C	-2.31144	4.32135	-1.56090
C	-2.61587	5.60573	-2.23263
H	-3.54591	5.72962	-2.77341
C	-1.74179	6.61639	-2.17876
H	-1.96400	7.55358	-2.67250
C	-0.46558	6.45317	-1.45703
H	0.24741	7.26735	-1.43680
C	-0.18391	5.30014	-0.83978
H	0.76836	5.19754	-0.33745
C	-1.15787	4.17420	-0.86667
C	-0.86403	2.87309	-0.19227
C	1.26575	1.77617	0.29464
C	2.59035	1.74195	0.95579
C	2.82340	2.52464	2.02096
H	3.79361	2.51241	2.50386
C	1.77221	3.43771	2.51756
C	2.01562	4.24649	3.73355
H	2.96545	4.18065	4.24937
C	1.05981	5.05416	4.20550
H	1.23630	5.64053	5.09789
C	-0.24239	5.14163	3.51690
H	-1.01743	5.78393	3.91466
C	-0.46859	4.42423	2.41038
H	-1.43661	4.48894	1.93144
C	0.59111	3.53673	1.86189
C	0.35402	2.70626	0.64480
C	3.67297	0.91034	0.38009
C	4.26414	1.29971	-0.95381
C	4.09497	-0.22150	1.00942
C	5.15226	0.46052	-1.52763
C	5.15312	-1.04783	0.37627
C	5.63776	-0.75114	-0.83378
H	5.54716	0.64330	-2.51382
H	5.51384	-1.92590	0.89610
C	-3.90308	0.87225	-1.24709
C	-4.71397	0.53770	-0.20738
C	-3.92154	0.05447	-2.50594
C	-5.59997	-0.64681	-0.33628
C	-4.75962	-0.99705	-2.58980
C	-5.63251	-1.37689	-1.46102
H	-6.22982	-0.92496	0.49934
H	-4.79536	-1.58961	-3.49577
H	-0.20187	-2.76809	-0.88216
C	1.37333	-4.06244	-1.10532
C	-0.00676	-3.84889	0.87035
C	1.66163	-3.60255	-2.40580
C	2.20687	-5.02046	-0.47300
C	0.82939	-4.80952	1.52219
C	2.75985	-4.11881	-3.06770
H	1.03686	-2.82973	-2.84149
C	3.31222	-5.53599	-1.18622
C	1.88114	-5.38604	0.86652
H	0.59842	-5.09358	2.53998
C	3.58185	-5.09648	-2.46698
H	2.99706	-3.76025	-4.06450
H	3.94788	-6.27502	-0.70697
H	2.48947	-6.13408	1.36791
H	4.43361	-5.49197	-3.01064
C	-1.00490	-3.06289	1.54671
C	-1.40252	-3.36287	2.99995

H	-1.60351	-4.43134	3.15532
H	-2.35393	-2.85366	3.17842
N	-0.45831	-2.95021	4.01857
N	0.29001	-3.56192	-0.42254
C	-0.16777	-1.62332	4.33296
C	0.86997	-1.35238	5.24545
C	-0.88028	-0.54020	3.79093
C	1.19874	-0.04461	5.58282
H	1.41705	-2.18331	5.68546
C	-0.54521	0.76626	4.14348
H	-1.66153	-0.70716	3.06116
C	0.49367	1.03023	5.03490
H	2.01246	0.13460	6.28030
H	-1.10075	1.58551	3.69554
H	0.75041	2.05058	5.30031
H	0.25772	-3.61252	4.27198
H	-0.29580	-1.81768	1.35774
Cl	-2.49552	-2.78846	0.53473
C	3.51086	-0.71116	2.33671
C	3.01478	-2.16407	2.25526
C	4.55416	-0.66570	3.46186
H	2.64366	-0.09705	2.65834
C	3.88942	-2.98201	3.19108
H	3.04001	-2.57862	1.22403
H	1.96601	-2.22083	2.61086
C	4.45988	-1.99533	4.19293
H	4.35132	0.17833	4.15729
H	5.58627	-0.54376	3.06482
H	3.30311	-3.77862	3.69893
H	4.71685	-3.45281	2.61676
H	3.76926	-1.89771	5.05745
H	5.45461	-2.32693	4.56186
C	3.81291	2.59039	-1.66438
C	4.06014	3.86030	-0.84027
C	4.46830	2.90597	-3.01196
H	2.72195	2.51094	-1.85941
C	4.25894	5.00230	-1.83852
H	4.97027	3.74835	-0.20977
H	3.19155	4.09244	-0.19074
C	4.20200	4.38200	-3.22746
H	4.04246	2.28995	-3.83366
H	5.57199	2.77508	-2.96314
H	3.46570	5.77393	-1.72602
H	5.25014	5.47987	-1.67565
H	3.18570	4.51787	-3.65996
H	4.95462	4.83525	-3.90868
C	6.68139	-1.64336	-1.48473
C	6.16208	-2.28343	-2.77982
C	7.95961	-0.87378	-1.85335
H	6.96888	-2.46684	-0.79079
C	7.28181	-2.14580	-3.79746
H	5.25479	-1.76726	-3.16494
H	5.91076	-3.35377	-2.62053
C	8.07049	-0.92288	-3.36962
H	8.84233	-1.38349	-1.40695
H	7.95615	0.17901	-1.49400
H	7.93738	-3.04379	-3.75505
H	6.88343	-2.03048	-4.82878
H	9.13016	-0.99021	-3.69846
H	7.60946	-0.01081	-3.80892

C	-3.02040	0.39488	-3.69297
C	-2.15361	-0.79022	-4.15546
C	-3.82601	0.85375	-4.91594
H	-2.32194	1.21757	-3.43431
C	-2.54682	-1.07582	-5.59726
H	-2.27957	-1.69927	-3.52735
H	-1.07904	-0.50235	-4.12380
C	-3.15567	0.21240	-6.11815
H	-3.82354	1.96222	-5.00560
H	-4.88290	0.51006	-4.86761
H	-1.66899	-1.38287	-6.20615
H	-3.31063	-1.88423	-5.62316
H	-2.34900	0.87920	-6.49588
H	-3.88367	0.01584	-6.93481
C	-4.75100	1.32440	1.10431
C	-6.14526	1.89652	1.39970
C	-4.36680	0.45423	2.31116
H	-4.05187	2.18693	1.08964
C	-6.39437	1.64743	2.87750
H	-6.93980	1.39089	0.80866
H	-6.18838	2.98428	1.17211
C	-5.58495	0.41127	3.22040
H	-3.52300	0.93062	2.85728
H	-4.05408	-0.57441	2.02728
H	-6.02079	2.51198	3.46995
H	-7.47497	1.49574	3.08932
H	-5.29214	0.40435	4.29270
H	-6.18488	-0.49922	2.99984
C	-6.54305	-2.58557	-1.58988
C	-6.28697	-3.64238	-0.50226
C	-8.02933	-2.21030	-1.50017
H	-6.37465	-3.06867	-2.57907
C	-7.57873	-3.76420	0.29211
H	-5.44057	-3.38339	0.17023
H	-6.05662	-4.62071	-0.97963
C	-8.67657	-3.27792	-0.63499
H	-8.49783	-2.18242	-2.50820
H	-8.17856	-1.21816	-1.01953
H	-7.75946	-4.81124	0.61853
H	-7.53012	-3.10487	1.18709
H	-9.02201	-4.11942	-1.27569
H	-9.53946	-2.87237	-0.06353

Catalyst (2,4,6-c-Pent, 10)-Enamine protonation TS (pro-S) alternative conformation
Electronic energy (Hartrees): -1870.436548
Free Energy at 1M concentration and 253 K (Hartrees): -1868.972559
Coordinates:

P	-0.18816	0.41932	0.38306
O	0.95771	1.33312	-0.32783
O	-1.47701	1.40158	0.58456
O	0.16898	-0.09649	1.73895
O	-0.51382	-0.62545	-0.71997
C	1.23074	2.48544	0.36283
C	2.55060	2.62609	1.02053
C	2.86488	3.76615	1.65114
H	3.83779	3.88092	2.11478
C	1.86781	4.85402	1.75696
C	2.18392	6.06432	2.54973
H	3.15549	6.17049	3.01615

C	1.26979	7.02954	2.69662
H	1.50124	7.91271	3.27794
C	-0.06259	6.88779	2.07855
H	-0.80499	7.66254	2.22058
C	-0.35731	5.80121	1.35546
H	-1.34738	5.70681	0.92949
C	0.65885	4.73340	1.15551
C	0.35758	3.51576	0.34365
C	-1.67857	2.33878	-0.39721
C	-2.89163	2.22268	-1.23947
C	-3.21423	3.20828	-2.08857
H	-4.09693	3.12044	-2.71168
C	-2.30784	4.35910	-2.27208
C	-2.62577	5.38029	-3.29682
H	-3.55571	5.32548	-3.84914
C	-1.76400	6.37046	-3.54959
H	-1.99615	7.11440	-4.30071
C	-0.48378	6.43934	-2.82118
H	0.22438	7.22396	-3.05470
C	-0.18911	5.52567	-1.88927
H	0.77194	5.57926	-1.39711
C	-1.15632	4.44062	-1.56303
C	-0.86117	3.41445	-0.51332
C	-3.68063	0.96747	-1.21472
C	-4.52798	0.64501	-0.01866
C	-3.59524	0.07469	-2.23910
C	-5.18060	-0.53366	0.02459
C	-4.41133	-1.16456	-2.18844
C	-5.12629	-1.49578	-1.10302
H	-5.78393	-0.76592	0.89044
H	-4.40032	-1.82439	-3.04641
C	3.51985	1.51053	0.91392
C	4.07209	1.20408	-0.29120
C	3.72626	0.58934	2.08327
C	4.76511	-0.09683	-0.45764
C	4.40187	-0.56236	1.89692
C	4.92750	-0.94768	0.56463
H	5.10391	-0.38146	-1.44487
H	4.53681	-1.22877	2.73487
H	-0.23651	-1.75706	2.06120
C	-1.95012	-2.53747	2.89137
C	-0.85405	-3.31218	0.87335
C	-1.94219	-1.63932	3.97636
C	-3.04192	-3.41880	2.69176
C	-1.95220	-4.20966	0.66016
C	-3.01479	-1.63314	4.84909
H	-1.11517	-0.94606	4.08399
C	-4.11556	-3.39313	3.60854
C	-2.99049	-4.26739	1.54546
H	-1.93234	-4.86508	-0.19985
C	-4.10419	-2.51180	4.67228
H	-3.02137	-0.93245	5.67816
H	-4.95262	-4.06849	3.45540
H	-3.79867	-4.97521	1.38630
H	-4.93550	-2.48672	5.36906
C	0.19758	-3.07672	-0.07260
C	0.33839	-3.93405	-1.32513
H	-0.66999	-4.13126	-1.71222
H	0.78849	-4.90754	-1.10809
N	1.09921	-3.28997	-2.38403

N	-0.90470	-2.55240	1.99838
C	2.46557	-3.47372	-2.61520
C	3.17424	-4.62603	-2.23039
C	3.16076	-2.47979	-3.33198
C	4.52664	-4.76060	-2.54258
H	2.68054	-5.41966	-1.68093
C	4.50950	-2.62392	-3.63524
H	2.62420	-1.59106	-3.65354
C	5.20997	-3.76717	-3.24334
H	5.05167	-5.65660	-2.22225
H	5.01675	-1.83117	-4.17849
H	6.26505	-3.87720	-3.47253
H	0.80011	-2.33668	-2.54600
H	-0.21537	-1.71086	-0.46987
Cl	1.80840	-2.75238	0.68468
C	-4.63851	1.60493	1.16659
C	-6.08630	2.03376	1.45635
C	-4.09297	0.98370	2.46263
H	-4.06389	2.53464	0.97654
C	-6.26630	1.92036	2.96208
H	-6.82892	1.37951	0.95007
H	-6.26843	3.07797	1.11956
C	-5.27189	0.86781	3.41491
H	-3.32687	1.65683	2.90815
H	-3.62073	-0.00934	2.30005
H	-6.01936	2.89350	3.44163
H	-7.30692	1.63405	3.22804
H	-4.96036	1.03120	4.46933
H	-5.72970	-0.14188	3.32292
C	-5.94621	-2.78356	-1.10292
C	-5.15993	-4.00242	-1.58974
C	-6.47236	-3.21500	0.26428
H	-6.81645	-2.63763	-1.78247
C	-5.83658	-5.22110	-0.96977
H	-4.10587	-3.94617	-1.24120
H	-5.16897	-4.07106	-2.69944
C	-6.75002	-4.70443	0.13873
H	-7.39150	-2.65531	0.54369
H	-5.69343	-3.06983	1.04447
H	-6.43672	-5.75851	-1.73668
H	-5.07546	-5.92068	-0.56073
H	-7.81391	-4.86298	-0.14519
H	-6.55169	-5.22665	1.10023
C	-2.66251	0.26566	-3.43795
C	-1.72030	-0.93248	-3.65374
C	-3.44177	0.45066	-4.74531
H	-2.00923	1.15476	-3.31696
C	-2.01509	-1.47269	-5.04526
H	-1.84338	-1.73357	-2.89208
H	-0.66271	-0.58764	-3.61337
C	-2.67591	-0.33249	-5.79696
H	-3.51009	1.52531	-5.02317
H	-4.47390	0.04231	-4.67211
H	-1.08706	-1.80957	-5.55627
H	-2.72267	-2.32813	-4.97165
H	-1.89423	0.31684	-6.25032
H	-3.35021	-0.71011	-6.59593
C	3.95987	2.12128	-1.51268
C	5.32618	2.40003	-2.16067
C	3.07119	1.51221	-2.60740

H	3.53124	3.10889	-1.23695
C	5.29802	1.74453	-3.53289
H	6.18196	2.02158	-1.55946
H	5.46158	3.49746	-2.28764
C	3.83309	1.65546	-3.91498
H	2.09599	2.04312	-2.67099
H	2.86469	0.43752	-2.41571
H	5.87310	2.33682	-4.27724
H	5.73114	0.72229	-3.47307
H	3.52567	2.59581	-4.42398
H	3.64415	0.79424	-4.59206
C	3.21583	0.93797	3.48394
C	2.25546	-0.11902	4.05720
C	4.36457	1.08185	4.49456
H	2.66585	1.89921	3.47396
C	2.74888	-0.43806	5.46033
H	2.24105	-1.05001	3.44957
H	1.21706	0.27500	4.10425
C	4.21935	-0.06698	5.47905
H	4.26001	2.04481	5.04278
H	5.36965	1.06798	4.01787
H	2.20091	0.18732	6.19971
H	2.60046	-1.51123	5.70934
H	4.54825	0.22852	6.49890
H	4.82759	-0.93361	5.13881
C	5.53717	-2.31520	0.28673
C	6.93056	-2.23741	-0.33301
C	5.73038	-3.22891	1.49162
H	4.86533	-2.84024	-0.42805
C	7.58723	-3.58542	-0.04233
H	7.52021	-1.42584	0.14974
H	6.87890	-2.04775	-1.42698
C	6.72927	-4.27281	1.01933
H	4.77326	-3.69424	1.81320
H	6.19079	-2.67388	2.33859
H	7.62107	-4.20540	-0.96516
H	8.62486	-3.43663	0.32862
H	6.18408	-5.13002	0.56530
H	7.34943	-4.64365	1.86426

Catalyst (2,4,6-c-Pent, 10)-Enamine protonation TS (pro-S) alternative conformation
Electronic energy (Hartrees): -1870.435019
Free Energy at 1M concentration and 253 K (Hartrees): -1868.971403
Coordinates:

P	-0.04147	0.36457	0.30880
O	1.11435	1.24729	-0.42703
O	-1.31790	1.36827	0.49779
O	0.30427	-0.13605	1.67310
O	-0.37454	-0.68981	-0.77994
C	1.35869	2.45135	0.17980
C	2.66407	2.65728	0.84813
C	2.96008	3.84871	1.38502
H	3.92310	4.00906	1.85571
C	1.95759	4.93678	1.37238
C	2.25457	6.21512	2.05857
H	3.21600	6.36692	2.53333
C	1.33567	7.18607	2.09850
H	1.55316	8.11913	2.60208
C	0.01688	6.98381	1.46838

H	-0.72990	7.76533	1.52446
C	-0.26056	5.83518	0.84069
H	-1.24170	5.69934	0.40553
C	0.76144	4.75715	0.75953
C	0.47913	3.46897	0.05668
C	-1.55616	2.22139	-0.54941
C	-2.80109	2.04303	-1.33266
C	-3.12335	2.92656	-2.28739
H	-4.03509	2.79952	-2.85970
C	-2.20195	4.03270	-2.61933
C	-2.52421	4.93755	-3.74681
H	-3.45834	4.82657	-4.28339
C	-1.66144	5.89173	-4.11114
H	-1.89688	6.55057	-4.93698
C	-0.37718	6.03747	-3.40080
H	0.32962	6.79248	-3.72024
C	-0.07847	5.22980	-2.37681
H	0.88309	5.33687	-1.89389
C	-1.04421	4.18712	-1.93217
C	-0.73968	3.27653	-0.78470
C	-3.66446	0.86677	-1.06845
C	-4.45451	0.80804	0.20693
C	-3.68097	-0.19540	-1.92108
C	-5.16220	-0.30271	0.48649
C	-4.53058	-1.37200	-1.59771
C	-5.24963	-1.42940	-0.46440
H	-5.69956	-0.37946	1.42319
H	-4.53205	-2.19567	-2.29493
C	3.64082	1.54352	0.85128
C	4.23056	1.15750	-0.31201
C	3.79831	0.69701	2.08230
C	4.91190	-0.15875	-0.37384
C	4.45553	-0.47659	1.98949
C	5.01988	-0.95044	0.70200
H	5.28367	-0.50589	-1.32889
H	4.55462	-1.08929	2.87242
H	-0.18709	-1.79652	1.98642
C	-1.97931	-2.48984	2.72308
C	-0.81578	-3.32233	0.76729
C	-1.98391	-1.58915	3.80571
C	-3.09708	-3.32386	2.47013
C	-1.94956	-4.15392	0.48542
C	-3.09481	-1.53477	4.62719
H	-1.13387	-0.93087	3.95026
C	-4.20731	-3.25431	3.34060
C	-3.03228	-4.16528	1.31815
H	-1.92418	-4.79742	-0.38333
C	-4.20872	-2.37049	4.40226
H	-3.11153	-0.83049	5.45308
H	-5.06222	-3.89836	3.15526
H	-3.86758	-4.82748	1.10660
H	-5.06841	-2.31192	5.06160
C	0.29952	-3.13734	-0.11683
C	0.49053	-4.01737	-1.34703
H	-0.49702	-4.19776	-1.79088
H	0.90093	-4.99836	-1.08932
N	1.32721	-3.41206	-2.36999
N	-0.89082	-2.55617	1.88579
C	2.70313	-3.61666	-2.50978
C	3.38054	-4.74728	-2.01937

C	3.44510	-2.66767	-3.24045
C	4.74744	-4.90473	-2.24658
H	2.85251	-5.50340	-1.44952
C	4.80769	-2.83379	-3.45769
H	2.93347	-1.79740	-3.64297
C	5.47637	-3.95587	-2.96285
H	5.24756	-5.78213	-1.84523
H	5.35027	-2.07597	-4.01628
H	6.54184	-4.08307	-3.12533
H	1.04945	-2.46248	-2.58335
H	-0.06829	-1.78702	-0.53132
Cl	1.87179	-2.85279	0.73282
C	-4.44298	1.96117	1.21192
C	-5.85071	2.49298	1.53103
C	-3.81174	1.54954	2.55052
H	-3.86082	2.82015	0.81969
C	-5.92784	2.60833	3.04595
H	-6.65156	1.80906	1.17508
H	-6.01871	3.48507	1.05709
C	-4.92153	1.60971	3.58652
H	-3.01403	2.27477	2.82680
H	-3.35560	0.53713	2.51840
H	-5.63358	3.63519	3.35720
H	-6.95204	2.39173	3.41971
H	-4.53330	1.92257	4.57991
H	-5.40070	0.61041	3.68158
C	-6.11516	-2.62275	-0.07905
C	-7.56465	-2.23032	0.19751
C	-6.23376	-3.75213	-1.10012
H	-5.69532	-3.05355	0.85497
C	-8.38891	-3.50169	-0.00278
H	-7.89913	-1.45442	-0.52757
H	-7.68522	-1.83786	1.23087
C	-7.47022	-4.52292	-0.67115
H	-5.32926	-4.39849	-1.10614
H	-6.42253	-3.34827	-2.11941
H	-8.74647	-3.89134	0.97554
H	-9.26984	-3.29036	-0.64776
H	-7.18200	-5.30765	0.06309
H	-7.96454	-5.00499	-1.54255
C	-2.85054	-0.24675	-3.20617
C	-1.97449	-1.50892	-3.30295
C	-3.74024	-0.23744	-4.45556
H	-2.16095	0.61924	-3.28663
C	-2.42896	-2.26110	-4.54447
H	-2.04026	-2.16145	-2.40514
H	-0.90972	-1.21323	-3.43081
C	-3.11285	-1.22846	-5.42004
H	-3.78844	0.77792	-4.90650
H	-4.77759	-0.56725	-4.22551
H	-1.57136	-2.73347	-5.07090
H	-3.16018	-3.04903	-4.25828
H	-2.35342	-0.71009	-6.04658
H	-3.87643	-1.69648	-6.07836
C	4.16499	1.99594	-1.59154
C	5.55302	2.22119	-2.21395
C	3.30258	1.32696	-2.67182
H	3.73866	3.00277	-1.39191
C	5.55409	1.49075	-3.54806
H	6.38616	1.86569	-1.56851

H	5.70774	3.30809	-2.39707
C	4.09893	1.39641	-3.96466
H	2.33019	1.85382	-2.78923
H	3.08993	0.26447	-2.42572
H	6.15638	2.03422	-4.30799
H	5.97243	0.46874	-3.41891
H	3.81510	2.31286	-4.52806
H	3.91855	0.50408	-4.60249
C	3.26236	1.14921	3.44278
C	2.25835	0.15782	4.05626
C	4.38798	1.32919	4.47418
H	2.74088	2.12263	3.35515
C	2.68723	-0.05148	5.50004
H	2.25679	-0.82076	3.52829
H	1.22546	0.56817	4.02373
C	4.16898	0.26879	5.54112
H	4.30820	2.33627	4.94126
H	5.40468	1.23826	4.03187
H	2.13755	0.65672	6.15908
H	2.48941	-1.09230	5.83663
H	4.47579	0.63334	6.54539
H	4.75462	-0.64299	5.29074
C	5.61801	-2.34008	0.52789
C	7.03946	-2.31652	-0.02914
C	5.74034	-3.18954	1.78785
H	4.97107	-2.89405	-0.18842
C	7.65748	-3.65751	0.36183
H	7.62070	-1.48998	0.43819
H	7.04179	-2.18359	-1.13259
C	6.74005	-4.27328	1.41788
H	4.76153	-3.62120	2.09040
H	6.17345	-2.59727	2.62381
H	7.72116	-4.32639	-0.52455
H	8.68002	-3.50583	0.77153
H	6.20012	-5.14477	0.98524
H	7.31461	-4.60816	2.30881

Catalyst (2,4,6-c-Pent, 10)-Enamine protonation TS (pro-S) alternative conformation
Electronic energy (Hartrees): -1870.431969
Free Energy at 1M concentration and 253 K (Hartrees): -1868.969019
Coordinates:

P	-0.19036	-0.18983	-0.24171
O	-1.45582	0.74266	0.23580
O	0.96374	0.82003	-0.80589
O	-0.51768	-1.18045	-1.31483
O	0.31493	-0.75699	1.10769
C	-1.67694	1.88465	-0.49080
C	-2.96764	2.03003	-1.20123
C	-3.24661	3.15832	-1.86779
H	-4.19177	3.26336	-2.38799
C	-2.25805	4.25488	-1.91928
C	-2.54476	5.46322	-2.72628
H	-3.49097	5.55947	-3.24416
C	-1.63486	6.43841	-2.82334
H	-1.84428	7.31969	-3.41576
C	-0.33688	6.31048	-2.13407
H	0.40421	7.09283	-2.23594
C	-0.07053	5.22702	-1.39583
H	0.89741	5.14628	-0.92020

C	-1.08331	4.14469	-1.25427
C	-0.80807	2.92070	-0.44163
C	1.29902	1.80175	0.09259
C	2.64860	1.79166	0.70198
C	2.95606	2.68590	1.65447
H	3.94508	2.69101	2.09773
C	1.96157	3.69479	2.07679
C	2.28773	4.63382	3.17402
H	3.25665	4.59196	3.65569
C	1.38348	5.53116	3.58118
H	1.62045	6.21336	4.38728
C	0.05568	5.58775	2.93984
H	-0.67752	6.30430	3.28684
C	-0.24509	4.75189	1.93930
H	-1.23065	4.79760	1.49520
C	0.75694	3.76256	1.46119
C	0.43734	2.80357	0.36322
C	3.67375	0.85178	0.19137
C	4.22295	1.05334	-1.20077
C	4.07835	-0.21379	0.93706
C	5.05409	0.11458	-1.70100
C	5.07771	-1.15374	0.37044
C	5.52241	-1.02469	-0.88364
H	5.41388	0.16098	-2.71606
H	5.42649	-1.97778	0.97931
C	-3.94769	0.92400	-1.15782
C	-4.74613	0.74699	-0.07232
C	-4.02739	-0.03320	-2.30941
C	-5.67429	-0.41231	-0.04047
C	-4.89446	-1.06254	-2.24699
C	-5.73672	-1.29646	-1.04779
H	-6.30688	-0.54675	0.82797
H	-4.98120	-1.72269	-3.09792
H	-0.38785	-2.78038	-0.48701
C	1.12490	-4.15887	-0.61914
C	-0.15739	-3.66047	1.37080
C	1.37008	-3.86687	-1.97582
C	1.95028	-5.07042	0.08795
C	0.67161	-4.57213	2.09775
C	2.41690	-4.50294	-2.61633
H	0.75442	-3.12486	-2.47376
C	3.00228	-5.71171	-0.60376
C	1.67224	-5.26307	1.47343
H	0.47555	-4.72605	3.15033
C	3.22879	-5.43719	-1.93776
H	2.62120	-4.27424	-3.65767
H	3.63207	-6.41520	-0.06673
H	2.27569	-5.97110	2.03517
H	4.03962	-5.92912	-2.46508
C	-1.09449	-2.76032	1.99005
C	-1.43773	-2.87484	3.48331
H	-1.65480	-3.91131	3.77413
H	-2.36934	-2.32343	3.63689
N	-0.44477	-2.36672	4.40916
N	0.09261	-3.53692	0.04272
C	-0.11444	-1.01702	4.53735
C	0.96930	-0.65817	5.36159
C	-0.83196	0.00576	3.89432
C	1.33632	0.67350	5.51654
H	1.52027	-1.43994	5.87965

C	-0.45802	1.33804	4.06397
H	-1.64952	-0.23269	3.22660
C	0.62521	1.68723	4.86890
H	2.18440	0.91987	6.14962
H	-1.01837	2.10737	3.53999
H	0.91121	2.72662	4.99251
H	0.28399	-3.00602	4.68611
H	-0.34805	-1.57908	1.63077
Cl	-2.61511	-2.53536	1.01143
C	3.53149	-0.51619	2.33420
C	2.97994	-1.94687	2.44832
C	4.62078	-0.37862	3.40697
H	2.70106	0.16720	2.60987
C	3.86004	-2.68042	3.44691
H	2.94977	-2.48412	1.47548
H	1.94446	-1.91721	2.84387
C	4.50484	-1.60357	4.29974
H	4.47936	0.55214	3.99928
H	5.63976	-0.35139	2.96119
H	3.26539	-3.38482	4.06859
H	4.64673	-3.25081	2.90660
H	3.85168	-1.37198	5.16790
H	5.49980	-1.92890	4.67359
C	3.79104	2.26751	-2.04554
C	4.11916	3.61548	-1.39102
C	4.40142	2.39168	-3.44453
H	2.69074	2.21110	-2.18938
C	4.32056	4.62018	-2.52688
H	5.04901	3.54154	-0.78415
H	3.28670	3.96036	-0.74420
C	4.18217	3.84128	-3.82735
H	3.91902	1.70043	-4.16941
H	5.50048	2.22094	-3.42066
H	3.56326	5.43356	-2.47991
H	5.33549	5.07014	-2.45935
H	3.15450	3.96748	-4.23519
H	4.92217	4.17744	-4.58586
C	6.50368	-2.03182	-1.45958
C	5.90651	-2.80072	-2.64647
C	7.79293	-1.36672	-1.96702
H	6.78947	-2.77713	-0.68166
C	6.98658	-2.83414	-3.71447
H	5.00371	-2.29678	-3.05751
H	5.62228	-3.83248	-2.34834
C	7.83772	-1.60291	-3.46903
H	8.67415	-1.85556	-1.49506
H	7.84383	-0.27908	-1.73909
H	7.60939	-3.74732	-3.58723
H	6.54956	-2.82732	-4.73652
H	8.87929	-1.75401	-3.82660
H	7.39288	-0.73184	-3.99889
C	-3.16300	0.14726	-3.55805
C	-2.35196	-1.11020	-3.92037
C	-4.00158	0.50065	-4.79396
H	-2.42947	0.96760	-3.41491
C	-2.81511	-1.53825	-5.30494
H	-2.47762	-1.94050	-3.19124
H	-1.26892	-0.85740	-3.96296
C	-3.40400	-0.29376	-5.94208
H	-3.96407	1.59174	-5.00582

H	-5.06709	0.20648	-4.66885
H	-1.97421	-1.94224	-5.90932
H	-3.60475	-2.31630	-5.21100
H	-2.59317	0.29727	-6.42300
H	-4.17210	-0.55233	-6.70283
C	-4.72363	1.68598	1.13576
C	-6.08534	2.35134	1.38086
C	-4.35131	0.95199	2.43366
H	-3.98922	2.50888	1.00726
C	-6.30414	2.30975	2.88345
H	-6.91425	1.80588	0.87940
H	-6.09128	3.40034	1.01148
C	-5.54396	1.08949	3.36743
H	-3.46839	1.44424	2.89751
H	-4.10095	-0.11969	2.27441
H	-5.87375	3.22421	3.34882
H	-7.38425	2.24134	3.13690
H	-5.22156	1.20722	4.42472
H	-6.19212	0.18951	3.28129
C	-6.71786	-2.46067	-0.95372
C	-6.63690	-3.48976	-2.07803
C	-6.53319	-3.29748	0.31340
H	-7.74941	-2.03959	-0.94641
C	-7.31350	-4.72913	-1.51774
H	-5.57739	-3.74902	-2.29878
H	-7.14241	-3.13132	-3.00104
C	-7.09170	-4.68260	-0.00796
H	-7.06852	-2.84497	1.17643
H	-5.45289	-3.38674	0.56317
H	-8.40301	-4.69028	-1.73969
H	-6.88582	-5.65411	-1.96235
H	-8.05349	-4.84736	0.52555
H	-6.37021	-5.46949	0.30371

Catalyst (2,4,6-c-Pent, 10)-Enamine protonation TS (pro-S) alternative conformation
Electronic energy (Hartrees): -1870.432326
Free Energy at 1M concentration and 253 K (Hartrees): -1868.968756
Coordinates:

P	0.04305	-0.15219	-0.01589
O	-1.12130	0.86606	0.51399
O	1.20504	0.74406	-0.74686
O	-0.48970	-0.98539	-1.20840
O	0.56601	-0.91414	1.17049
C	-1.54703	1.78261	-0.41901
C	-2.97584	1.79856	-0.83816
C	-3.40659	2.72070	-1.71387
H	-4.44855	2.74338	-2.01333
C	-2.46826	3.69129	-2.30326
C	-2.93811	4.62681	-3.35104
H	-3.97626	4.62175	-3.65935
C	-2.08135	5.47924	-3.92342
H	-2.42440	6.16137	-4.69055
C	-0.66192	5.48203	-3.52129
H	0.03193	6.15659	-4.00620
C	-0.23017	4.64439	-2.57137
H	0.82027	4.64461	-2.31221
C	-1.17894	3.71272	-1.90129
C	-0.72541	2.77370	-0.83066
C	1.50247	1.95968	-0.17933

C	2.91761	2.25818	0.17829
C	3.26476	3.49876	0.55981
H	4.29772	3.72683	0.79712
C	2.24051	4.54284	0.73452
C	2.61933	5.86082	1.29426
H	3.65392	6.07339	1.53359
C	1.68442	6.79231	1.51070
H	1.96111	7.75461	1.92169
C	0.27005	6.50548	1.20375
H	-0.48518	7.25472	1.40325
C	-0.08019	5.31936	0.69305
H	-1.12610	5.12075	0.49890
C	0.95366	4.28396	0.41767
C	0.59300	2.96096	-0.17540
C	3.96224	1.20643	0.10203
C	4.39388	0.68374	-1.23966
C	4.58541	0.76266	1.23143
C	5.39595	-0.21280	-1.29770
C	5.74917	-0.15490	1.10175
C	6.16374	-0.59973	-0.09703
H	5.68909	-0.63132	-2.25235
H	6.26660	-0.44683	2.00313
C	-3.95403	0.83722	-0.27850
C	-4.44472	0.99315	0.98350
C	-4.56530	-0.18047	-1.19803
C	-5.58901	0.14558	1.42075
C	-5.64521	-0.86859	-0.78651
C	-6.19515	-0.69968	0.57441
H	-5.99880	0.28641	2.41288
H	-6.13594	-1.55707	-1.46216
H	-0.60405	-2.12141	-1.01994
C	-0.61219	-3.54989	-0.66215
C	-1.29018	-3.58076	0.70715
H	-0.62306	-3.11671	1.43260
H	-1.43046	-4.63234	1.01859
N	-2.54895	-2.83654	0.74506
H	-3.23028	-3.27858	0.13599
C	-3.08933	-2.62961	2.03774
C	-4.33217	-3.16877	2.39335
C	-2.38651	-1.86406	2.98372
C	-4.88268	-2.92577	3.65165
H	-4.85260	-3.80198	1.68083
C	-2.92278	-1.66330	4.25277
H	-1.43862	-1.41252	2.70709
C	-4.17738	-2.17753	4.59288
H	-5.85447	-3.34260	3.90124
H	-2.36849	-1.06842	4.97378
H	-4.59706	-1.99614	5.57768
C	0.77889	-3.90360	-0.68482
C	1.41268	-4.79347	-1.61169
C	2.91550	-3.61669	0.42057
C	2.73520	-5.10709	-1.48575
H	0.80262	-5.23776	-2.38523
C	3.63104	-3.03394	1.48104
C	3.53340	-4.56269	-0.43377
H	3.19171	-5.81043	-2.17710
C	4.93564	-3.42918	1.71152
H	3.12937	-2.30811	2.10961
C	4.87494	-4.92605	-0.18647
C	5.56201	-4.38094	0.88117

H	5.48258	-3.00360	2.54663
H	5.35059	-5.65295	-0.83854
H	6.58673	-4.67815	1.07818
N	1.58841	-3.30575	0.23335
H	1.20881	-2.48568	0.75423
Cl	-1.61235	-4.47464	-1.86687
C	-7.49218	-1.38953	0.96810
C	-8.67299	-0.88916	0.12070
C	-7.44204	-2.91473	0.78851
H	-7.72590	-1.18078	2.03760
C	-9.15567	-2.07873	-0.69319
H	-8.40400	-0.04170	-0.54847
H	-9.49371	-0.54895	0.79077
C	-8.73691	-3.30366	0.09504
H	-7.36036	-3.43040	1.76968
H	-6.58760	-3.23285	0.15351
H	-10.25671	-2.05053	-0.84303
H	-8.65065	-2.08357	-1.68460
H	-9.51334	-3.53839	0.85670
H	-8.59604	-4.18376	-0.56926
C	-4.03446	-0.39439	-2.61620
C	-3.63783	-1.85113	-2.89438
C	-5.06720	-0.00509	-3.68591
H	-3.12405	0.21588	-2.79133
C	-4.22359	-2.19999	-4.25266
H	-4.04321	-2.55068	-2.13212
H	-2.53233	-1.95961	-2.91305
C	-5.41928	-1.28380	-4.42910
H	-4.61101	0.71824	-4.39812
H	-5.98073	0.46573	-3.25986
H	-3.47438	-1.98887	-5.04771
H	-4.52199	-3.26951	-4.30497
H	-5.62272	-1.08691	-5.50396
H	-6.31714	-1.75060	-3.96703
C	-3.96042	2.10094	1.92361
C	-5.04600	3.12438	2.26124
C	-3.46576	1.58096	3.26574
H	-3.11843	2.67826	1.49034
C	-4.75006	3.60519	3.68229
H	-6.06322	2.67797	2.22959
H	-5.01821	3.97207	1.54158
C	-3.58594	2.76720	4.20564
H	-2.41552	1.22207	3.19930
H	-4.11118	0.76248	3.64546
H	-4.48086	4.68412	3.68439
H	-5.64433	3.45925	4.32741
H	-2.64812	3.36486	4.16808
H	-3.76226	2.43423	5.25159
C	3.73490	1.14325	-2.54164
C	4.73147	1.77517	-3.52731
C	3.04740	-0.01344	-3.28681
H	2.96671	1.91717	-2.34826
C	4.37258	1.21948	-4.89514
H	5.78487	1.50879	-3.29290
H	4.65182	2.88459	-3.52234
C	3.75005	-0.13856	-4.62994
H	1.97592	0.23536	-3.45635
H	3.08859	-0.97680	-2.73127
H	3.62335	1.88229	-5.38234
H	5.26773	1.13402	-5.54861

H	3.03942	-0.42185	-5.43637
H	4.55129	-0.90735	-4.56215
C	4.17286	1.21561	2.63645
C	3.85385	0.06195	3.58501
C	5.25850	2.04525	3.33345
H	3.25089	1.83398	2.61621
C	4.13741	0.61182	4.96944
H	4.51310	-0.81320	3.41416
H	2.79310	-0.25851	3.49114
C	5.29353	1.58520	4.79025
H	5.00709	3.12733	3.27612
H	6.26016	1.90167	2.87211
H	3.24291	1.15860	5.34229
H	4.39484	-0.19997	5.68396
H	5.19658	2.44605	5.48708
H	6.25387	1.06225	4.99523
C	7.37427	-1.51025	-0.27999
C	8.35252	-0.98061	-1.32904
C	8.25553	-1.72999	0.94936
H	7.00487	-2.50052	-0.62752
C	9.70922	-1.59855	-0.99466
H	8.42644	0.12848	-1.26298
H	8.03348	-1.26211	-2.35629
C	9.57936	-2.22311	0.39217
H	7.81315	-2.46695	1.65229
H	8.44159	-0.76790	1.47705
H	9.97614	-2.37797	-1.74179
H	10.49901	-0.81556	-0.99655
H	9.55519	-3.33211	0.30508
H	10.42537	-1.92904	1.05087

Catalyst (2,4,6-c-Pent, 10)-Enamine protonation TS (pro-S) alternative conformation
Electronic energy (Hartrees): -1870.428516
Free Energy at 1M concentration and 253 K (Hartrees): -1868.96599
Coordinates:

P	-0.08480	-0.26332	0.15578
O	1.11930	0.70920	-0.37725
O	-1.28662	0.68163	0.75684
O	0.38034	-1.02018	1.42733
O	-0.54572	-1.10473	-1.00194
C	1.49980	1.69210	0.50706
C	2.91275	1.76494	0.97138
C	3.29292	2.74005	1.81288
H	4.32486	2.80775	2.13887
C	2.31572	3.72715	2.30514
C	2.72427	4.73164	3.31391
H	3.74419	4.75434	3.67722
C	1.83406	5.61301	3.78255
H	2.13212	6.34576	4.52126
C	0.43909	5.58168	3.30249
H	-0.28179	6.28368	3.70133
C	0.06292	4.68153	2.38672
H	-0.97030	4.66036	2.06567
C	1.04933	3.71248	1.83595
C	0.65652	2.70078	0.81024
C	-1.53775	1.85932	0.09125
C	-2.92766	2.15759	-0.36008
C	-3.21560	3.35840	-0.89118
H	-4.22994	3.59165	-1.19488

C	-2.15634	4.35769	-1.10807
C	-2.46503	5.61817	-1.82205
H	-3.47514	5.82148	-2.15547
C	-1.49613	6.50819	-2.06252
H	-1.72107	7.42797	-2.58680
C	-0.11404	6.23250	-1.62562
H	0.67026	6.94598	-1.84321
C	0.17185	5.09931	-0.97414
H	1.19650	4.90574	-0.68478
C	-0.90215	4.11245	-0.67470
C	-0.61275	2.84556	0.06069
C	-4.02687	1.17665	-0.17899
C	-4.55867	0.91109	1.20307
C	-4.66200	0.64089	-1.26128
C	-5.67361	0.16841	1.34201
C	-5.93018	-0.10880	-1.05665
C	-6.43205	-0.31150	0.17119
H	-6.06140	-0.04418	2.33101
H	-6.49459	-0.42775	-1.92402
C	3.93700	0.83356	0.44853
C	4.42414	0.97271	-0.81577
C	4.55341	-0.16686	1.38159
C	5.56063	0.11565	-1.24869
C	5.60862	-0.88883	0.96108
C	6.17462	-0.72206	-0.39467
H	5.91199	0.21418	-2.26478
H	6.07714	-1.59674	1.63294
H	0.60413	-2.14518	1.29738
C	0.71666	-3.59844	0.99388
C	1.40987	-3.64259	-0.36708
H	0.74128	-3.20810	-1.10950
H	1.57882	-4.69688	-0.65472
N	2.65393	-2.87169	-0.40298
H	3.32047	-3.27449	0.24830
C	3.23385	-2.74379	-1.68946
C	4.49029	-3.29523	-1.97415
C	2.55122	-2.05006	-2.70307
C	5.06192	-3.14785	-3.23792
H	5.01361	-3.85124	-1.19971
C	3.11579	-1.93435	-3.97042
H	1.59399	-1.58832	-2.48068
C	4.37606	-2.47214	-4.24620
H	6.03849	-3.58082	-3.43635
H	2.57674	-1.39270	-4.74314
H	4.81568	-2.36419	-5.23312
C	-0.65557	-4.01926	1.01483
C	-1.25016	-4.92030	1.95665
C	-2.80225	-3.84352	-0.09324
C	-2.55661	-5.29557	1.83554
H	-0.62165	-5.32423	2.73735
C	-3.54643	-3.29972	-1.15479
C	-3.37886	-4.79996	0.77853
H	-2.98086	-6.00888	2.53717
C	-4.83546	-3.74893	-1.37140
H	-3.07797	-2.56004	-1.79282
C	-4.70641	-5.21869	0.54446
C	-5.41952	-4.71426	-0.52575
H	-5.40217	-3.36123	-2.21132
H	-5.14919	-5.95525	1.20878
H	-6.43104	-5.05706	-0.71701

N	-1.48704	-3.47996	0.08093
H	-1.13699	-2.67006	-0.47421
Cl	1.74263	-4.45199	2.23121
C	7.40587	-1.54127	-0.77313
C	8.08407	-1.17477	-2.09312
C	8.53239	-1.41364	0.25325
H	7.10621	-2.61139	-0.82105
C	9.48666	-1.74580	-1.98062
H	8.16882	-0.06977	-2.19725
H	7.54431	-1.59556	-2.96780
C	9.82694	-1.73222	-0.49287
H	8.38447	-2.11554	1.10275
H	8.58089	-0.37304	0.64655
H	9.49295	-2.79211	-2.35888
H	10.21256	-1.14300	-2.56865
H	10.22121	-2.72438	-0.18139
H	10.59545	-0.95800	-0.27660
C	4.05681	-0.32801	2.81949
C	3.62058	-1.75939	3.15163
C	5.12807	0.05832	3.85297
H	3.17104	0.31326	3.00658
C	4.14772	-2.04371	4.54680
H	4.04991	-2.50225	2.44684
H	2.51350	-1.84844	3.12893
C	5.38990	-1.18535	4.68928
H	4.73886	0.86820	4.50957
H	6.07174	0.42118	3.38897
H	3.39222	-1.73306	5.30227
H	4.38143	-3.12197	4.68236
H	5.58346	-0.92805	5.75316
H	6.26746	-1.73275	4.27929
C	3.92003	2.05007	-1.78148
C	5.00520	3.04639	-2.20012
C	3.36775	1.47950	-3.08091
H	3.10342	2.65819	-1.34171
C	4.70331	3.42473	-3.65086
H	6.02480	2.60884	-2.13799
H	4.97791	3.94504	-1.54506
C	3.48012	2.61861	-4.07668
H	2.31375	1.14549	-2.96410
H	3.98259	0.63096	-3.44430
H	4.50247	4.51441	-3.74334
H	5.56992	3.16452	-4.29786
H	2.57073	3.25669	-4.01230
H	3.58485	2.23694	-5.11552
C	-3.88849	1.48202	2.45409
C	-4.81580	2.39676	3.26784
C	-3.39673	0.38355	3.41145
H	-3.01118	2.10283	2.19191
C	-4.52395	2.08279	4.72437
H	-5.89006	2.19809	3.06381
H	-4.61701	3.46891	3.04836
C	-4.09268	0.62779	4.74307
H	-2.29569	0.47112	3.54856
H	-3.61133	-0.64404	3.04313
H	-3.68790	2.72305	5.08380
H	-5.41710	2.24964	5.36467
H	-3.41689	0.41830	5.60036
H	-4.99162	-0.02338	4.81851
C	-4.18776	0.87743	-2.69856

C	-4.00179	-0.41576	-3.49752
C	-5.17293	1.74395	-3.49961
H	-3.19986	1.38471	-2.72471
C	-4.39695	-0.06899	-4.91997
H	-4.66525	-1.22732	-3.13619
H	-2.94836	-0.76937	-3.45547
C	-5.48105	0.98449	-4.78481
H	-4.69967	2.72044	-3.74562
H	-6.11417	1.95275	-2.94470
H	-3.52234	0.36177	-5.45623
H	-4.76375	-0.96226	-5.47064
H	-5.49425	1.66253	-5.66555
H	-6.47123	0.48507	-4.69414
C	-7.80453	-0.92863	0.38361
C	-8.93964	-0.11553	-0.25305
C	-7.93729	-2.34081	-0.17352
H	-8.01524	-0.99075	1.47599
C	-9.91762	-1.12667	-0.85068
H	-8.57495	0.56589	-1.05267
H	-9.44604	0.50384	0.51967
C	-9.41859	-2.50852	-0.45116
H	-7.57662	-3.09766	0.55592
H	-7.38501	-2.45271	-1.12930
H	-10.95062	-0.95701	-0.47634
H	-9.91806	-1.03812	-1.95968
H	-9.93539	-2.83859	0.47724
H	-9.59804	-3.25506	-1.25509

Catalyst (2,4,6-c-Pent, 10)-Enamine protonation TS (pro-S) alternative conformation
Electronic energy (Hartrees): -1870.428176
Free Energy at 1M concentration and 253 K (Hartrees): -1868.962197
Coordinates:

P	-0.03579	0.32095	-0.19137
O	0.91914	1.54878	-0.65214
O	-1.41409	0.97431	0.37794
O	0.50212	-0.60370	0.82951
O	-0.34680	-0.28817	-1.62786
C	1.07170	2.50222	0.32310
C	2.38769	2.64960	0.98983
C	2.55578	3.57685	1.94270
H	3.52022	3.69433	2.42334
C	1.42239	4.42852	2.35997
C	1.59832	5.38891	3.47357
H	2.56288	5.49302	3.95476
C	0.56616	6.12871	3.89266
H	0.69520	6.82859	4.70813
C	-0.75590	5.98319	3.25435
H	-1.59271	6.56755	3.61468
C	-0.92451	5.12330	2.24322
H	-1.90884	5.01538	1.80789
C	0.22136	4.31567	1.74336
C	0.05943	3.34712	0.61662
C	-1.87073	2.10153	-0.26242
C	-3.19953	2.05107	-0.91700
C	-3.71851	3.15489	-1.47302
H	-4.69823	3.12194	-1.93560
C	-2.94299	4.41143	-1.49376
C	-3.48533	5.59574	-2.19859
H	-4.46341	5.55259	-2.66149

C	-2.76827	6.72215	-2.27017
H	-3.16455	7.58642	-2.78736
C	-1.42814	6.78602	-1.65692
H	-0.84319	7.69300	-1.73942
C	-0.93113	5.72384	-1.01269
H	0.06390	5.78647	-0.59307
C	-1.72936	4.47267	-0.89593
C	-1.20050	3.27449	-0.17449
C	-3.96188	0.78277	-0.90198
C	-4.53191	0.29830	0.39944
C	-4.06344	0.00933	-2.01655
C	-5.04018	-0.94738	0.46873
C	-4.71806	-1.31986	-1.91175
C	-5.14171	-1.80087	-0.73278
H	-5.43781	-1.31838	1.40583
H	-4.82467	-1.92296	-2.80452
C	3.52062	1.80117	0.55920
C	4.15184	2.04254	-0.62181
C	3.92352	0.61962	1.39497
C	5.19911	1.09848	-1.08775
C	4.88311	-0.20748	0.93607
C	5.53677	0.01771	-0.36840
H	5.68258	1.28602	-2.03771
H	5.19252	-1.05919	1.52907
C	-0.33521	-3.61422	-0.49626
H	-0.35890	-1.26492	-1.62667
Cl	-0.82885	-3.58253	-2.21059
C	-3.51724	0.43557	-3.38052
C	-2.57653	-0.61021	-4.00280
C	-4.64618	0.66343	-4.39301
H	-2.93861	1.38036	-3.31783
C	-3.19997	-1.02400	-5.32816
H	-2.42303	-1.49717	-3.35423
H	-1.57963	-0.15389	-4.19361
C	-4.13964	0.10373	-5.70982
H	-4.88861	1.74452	-4.48829
H	-5.57371	0.12038	-4.10532
H	-2.42630	-1.18662	-6.10952
H	-3.78281	-1.96169	-5.19042
H	-3.57466	0.89039	-6.25748
H	-4.97464	-0.26135	-6.34625
C	-4.58176	1.20722	1.62812
C	-6.00204	1.37520	2.19842
C	-3.70812	0.69531	2.77691
H	-4.22078	2.22672	1.37690
C	-5.93520	0.94322	3.65748
H	-6.76849	0.78232	1.65322
H	-6.30401	2.44496	2.14453
C	-4.47122	1.02964	4.04467
H	-2.70755	1.17978	2.77718
H	-3.57104	-0.40346	2.71830
H	-6.56285	1.59281	4.30518
H	-6.27971	-0.11077	3.75093
H	-4.22979	2.06827	4.36238
H	-4.22703	0.32812	4.86992
C	-5.77444	-3.17815	-0.62569
C	-7.04678	-3.30287	-1.47881
C	-4.82795	-4.30243	-1.07830
H	-6.05249	-3.37225	0.43511
C	-6.74580	-4.33970	-2.54941

H	-7.36008	-2.34062	-1.94105
H	-7.88523	-3.66733	-0.84447
C	-5.65191	-5.21605	-1.97034
H	-4.43013	-4.86772	-0.20840
H	-3.96493	-3.91275	-1.66094
H	-7.64875	-4.93549	-2.80476
H	-6.37036	-3.83335	-3.46609
H	-6.10945	-6.02374	-1.35694
H	-5.03110	-5.67121	-2.77230
C	3.81628	3.24052	-1.51285
C	5.05498	4.08547	-1.85720
C	3.19699	2.80445	-2.84725
H	3.09258	3.92732	-1.02485
C	5.19696	4.04894	-3.37222
H	5.98505	3.72147	-1.36812
H	4.89345	5.13722	-1.53094
C	3.82131	3.69611	-3.90553
H	2.09132	2.92296	-2.83520
H	3.43134	1.74323	-3.08482
H	5.54655	5.02522	-3.77251
H	5.92134	3.25561	-3.66110
H	3.21621	4.62322	-4.01674
H	3.88700	3.17922	-4.88738
C	3.28883	0.34666	2.75931
C	2.73014	-1.08056	2.89067
C	4.29378	0.52969	3.90605
H	2.44258	1.03946	2.94536
C	3.53188	-1.76102	3.98834
H	2.78644	-1.65726	1.94318
H	1.66342	-1.03735	3.20147
C	4.06820	-0.63681	4.85349
H	4.13358	1.49848	4.42807
H	5.34508	0.50057	3.54318
H	2.90109	-2.46037	4.57882
H	4.37958	-2.32458	3.53966
H	3.30591	-0.35773	5.61440
H	5.00847	-0.93393	5.36642
C	6.56951	-0.97879	-0.86227
C	6.21891	-1.59941	-2.20937
C	7.96314	-0.37873	-1.04460
H	6.65889	-1.81403	-0.12956
C	7.54592	-2.09121	-2.75986
H	5.79622	-0.84888	-2.91139
H	5.49135	-2.42832	-2.09538
C	8.61477	-1.16418	-2.18338
H	8.55431	-0.47128	-0.10730
H	7.91305	0.69671	-1.32286
H	7.72739	-3.13232	-2.41247
H	7.55002	-2.07450	-3.87140
H	9.47689	-1.75731	-1.80722
H	8.97849	-0.46564	-2.96877
C	0.95115	-4.01283	-0.20593
C	2.01275	-4.30518	-1.12523
C	3.28904	-4.61856	-0.69793
H	1.79262	-4.27246	-2.18465
C	2.54700	-4.42087	1.59170
C	4.36730	-4.92052	-1.60385
C	3.59510	-4.65788	0.72386
H	2.66965	-4.44177	2.66976
C	5.62172	-5.18646	-1.13683

H	4.16303	-4.91688	-2.67071
C	4.92567	-4.93281	1.16992
C	5.91887	-5.18319	0.26769
H	6.42093	-5.40133	-1.84073
H	5.12754	-4.93453	2.23824
H	6.93140	-5.38594	0.60057
N	1.30915	-4.14908	1.15025
H	0.56879	-3.87596	1.80920
C	-1.49216	-3.50620	0.46722
H	-1.90794	-4.50030	0.69815
H	-2.30505	-2.93718	-0.00947
N	-1.07403	-2.89610	1.73595
H	-0.56933	-2.02060	1.56929
C	-1.99609	-2.79823	2.79870
C	-3.27672	-3.37110	2.76486
C	-1.59862	-2.10892	3.96045
C	-4.12716	-3.26182	3.86761
H	-3.62430	-3.88838	1.87974
C	-2.45421	-2.00365	5.05019
H	-0.61269	-1.65420	3.99135
C	-3.72686	-2.58334	5.01577
H	-5.11685	-3.70715	3.81538
H	-2.12664	-1.46184	5.93319
H	-4.39423	-2.49758	5.86748

Catalyst (2,4,6-c-Pent, 10)-Enamine protonation TS (pro-S) alternative conformation
Electronic energy (Hartrees): -1870.415875
Free Energy at 1M concentration and 253 K (Hartrees): -1868.952399
Coordinates:

P	-0.29177	-0.39438	0.07367
O	-1.50450	0.66151	0.36670
O	1.03684	0.45399	-0.37995
O	-0.58208	-1.39175	-1.00358
O	-0.04193	-0.94036	1.50014
C	-1.47516	1.85081	-0.30729
C	-2.62466	2.17860	-1.17815
C	-2.70677	3.37685	-1.76669
H	-3.54548	3.60902	-2.41302
C	-1.63492	4.37176	-1.57386
C	-1.71133	5.66713	-2.28785
H	-2.56659	5.89461	-2.91207
C	-0.72434	6.55984	-2.16929
H	-0.78014	7.50498	-2.69374
C	0.45049	6.25368	-1.33446
H	1.25762	6.97126	-1.26205
C	0.52966	5.08782	-0.68332
H	1.41965	4.88540	-0.10758
C	-0.57354	4.08926	-0.77892
C	-0.51461	2.77084	-0.06284
C	1.43642	1.39652	0.53540
C	2.86713	1.42772	0.96824
C	3.25660	2.38285	1.83309
H	4.30798	2.56357	2.01702
C	2.26715	3.29168	2.44700
C	2.66532	4.18889	3.55515
H	3.67759	4.16306	3.93879
C	1.77303	5.03078	4.09007
H	2.06232	5.68361	4.90337
C	0.38959	5.07078	3.57463

H	-0.32962	5.74707	4.01814
C	0.02445	4.27031	2.56639
H	-0.99545	4.30164	2.20460
C	1.00886	3.33836	1.95995
C	0.62918	2.44113	0.83754
C	3.89079	0.77340	0.10617
C	4.12162	1.37000	-1.26343
C	4.57681	-0.35840	0.46982
C	4.90919	0.71920	-2.13951
C	5.48420	-0.99281	-0.52260
C	5.61782	-0.51517	-1.76651
H	5.04579	1.10382	-3.14219
H	6.00632	-1.90365	-0.25596
C	-3.65781	1.15064	-1.42193
C	-4.64631	0.93060	-0.51635
C	-3.55597	0.28892	-2.64491
C	-5.60125	-0.18331	-0.74811
C	-4.45056	-0.70230	-2.82409
C	-5.50429	-0.98715	-1.81837
H	-6.38328	-0.35665	-0.01953
H	-4.39656	-1.29542	-3.72584
H	-0.56114	-2.98233	-0.16830
C	0.96911	-4.34651	-0.12259
C	-0.52523	-3.84128	1.71263
C	1.36859	-4.05109	-1.44136
C	1.71978	-5.24536	0.67772
C	0.23042	-4.73619	2.53440
C	2.50014	-4.66190	-1.94915
H	0.80434	-3.32051	-2.01148
C	2.86030	-5.86437	0.11846
C	1.29375	-5.42854	2.02692
H	-0.07636	-4.88061	3.56155
C	3.24643	-5.57674	-1.17597
H	2.82335	-4.42646	-2.95843
H	3.43424	-6.55561	0.72903
H	1.83680	-6.12783	2.65712
H	4.12984	-6.04443	-1.59838
C	-1.52821	-2.94362	2.21976
C	-2.02255	-3.03667	3.67164
H	-2.27927	-4.06757	3.94961
H	-2.95970	-2.47512	3.72173
N	-1.12218	-2.52886	4.68787
N	-0.13815	-3.73303	0.41604
C	-0.79886	-1.18385	4.86049
C	0.20221	-0.83564	5.78901
C	-1.44249	-0.15225	4.15542
C	0.55968	0.49348	5.98578
H	0.70808	-1.62178	6.34588
C	-1.07435	1.17602	4.36350
H	-2.19418	-0.38184	3.41117
C	-0.07269	1.51495	5.27219
H	1.34404	0.73077	6.69946
H	-1.57269	1.95050	3.78733
H	0.21428	2.55105	5.42113
H	-0.45577	-3.17890	5.07351
H	-0.75281	-1.76402	1.93241
Cl	-2.93379	-2.72244	1.08441
C	4.39198	-1.15469	1.76795
C	3.04658	-1.86861	1.83409
C	4.56692	-0.38902	3.08479

H	5.15936	-1.96217	1.79798
C	2.60778	-1.79068	3.28542
H	3.13675	-2.92596	1.50615
H	2.31048	-1.36989	1.18166
C	3.24744	-0.52743	3.83277
H	4.87589	0.66044	2.95422
H	5.36484	-0.87401	3.69138
H	1.50479	-1.76534	3.37597
H	2.99511	-2.67409	3.83970
H	2.59056	0.34251	3.61964
H	3.40637	-0.59971	4.93042
C	3.46276	2.68565	-1.69053
C	4.44609	3.75403	-2.16019
C	2.45712	2.52048	-2.83157
H	2.91742	3.14861	-0.84719
C	3.61961	4.67787	-3.03966
H	5.27213	3.32635	-2.76633
H	4.88386	4.30463	-1.29879
C	2.49788	3.82390	-3.62796
H	1.43644	2.33483	-2.43164
H	2.72722	1.67309	-3.49839
H	3.18412	5.48907	-2.41592
H	4.24300	5.13410	-3.83912
H	1.52543	4.35851	-3.55485
H	2.70459	3.60857	-4.69932
C	6.46442	-1.23630	-2.80218
C	7.94294	-1.33073	-2.39649
C	5.98080	-2.67404	-3.05592
H	6.41220	-0.68546	-3.76864
C	8.37583	-2.75146	-2.71939
H	8.08925	-1.14593	-1.30956
H	8.55974	-0.59532	-2.95798
C	7.11525	-3.59086	-2.62602
H	5.78799	-2.81631	-4.14254
H	5.04582	-2.92485	-2.50783
H	8.77372	-2.79367	-3.75760
H	9.15487	-3.11112	-2.01275
H	7.17948	-4.49111	-3.27483
H	6.95831	-3.91053	-1.57203
C	-2.46525	0.51764	-3.69263
C	-1.62337	-0.73920	-3.97779
C	-3.03872	0.97684	-5.03841
H	-1.75544	1.30376	-3.36170
C	-1.74539	-1.01284	-5.47061
H	-1.94937	-1.62785	-3.39443
H	-0.55717	-0.54190	-3.72601
C	-2.19021	0.29604	-6.09675
H	-2.98935	2.08314	-5.14039
H	-4.09702	0.65973	-5.16697
H	-0.78104	-1.35895	-5.90172
H	-2.52324	-1.78913	-5.64471
H	-1.29909	0.92340	-6.32131
H	-2.76536	0.12401	-7.03225
C	-4.80105	1.76248	0.75849
C	-6.16807	2.45724	0.83965
C	-4.66497	0.90597	2.02708
H	-4.03498	2.56395	0.82400
C	-6.64047	2.28229	2.27301
H	-6.91320	1.99820	0.15409
H	-6.08504	3.53611	0.58262

C	-5.99618	0.99685	2.75655
H	-3.86575	1.32806	2.67524
H	-4.40704	-0.15424	1.81377
H	-6.27963	3.13389	2.89154
H	-7.74905	2.22752	2.33356
H	-5.85610	1.00467	3.85926
H	-6.63640	0.13148	2.47590
C	-6.51435	-2.11715	-1.98988
C	-6.26996	-3.05582	-3.16838
C	-6.56492	-3.05879	-0.78562
H	-7.52222	-1.65968	-2.11829
C	-7.06316	-4.30803	-2.83557
H	-5.19574	-3.33958	-3.23027
H	-6.60212	-2.60550	-4.12899
C	-7.09654	-4.39101	-1.31143
H	-7.22495	-2.65617	0.01340
H	-5.54342	-3.20931	-0.37117
H	-8.09862	-4.20800	-3.23009
H	-6.59128	-5.21096	-3.28081
H	-8.13801	-4.56006	-0.95989
H	-6.45758	-5.22785	-0.95317

Catalyst (2,4,6-c-Pent, 10)-Enamine protonation TS (pro-R) lowest free energy conformation
Electronic energy (Hartrees): -1870.449348
Free Energy at 1M concentration and 253 K (Hartrees): -1868.985921
Coordinates:

P	0.03352	0.49490	-0.10432
O	-1.12746	1.25871	0.74633
O	0.98053	1.65250	-0.74481
O	-0.61381	-0.13461	-1.37275
O	0.74154	-0.43263	0.83110
C	-1.68007	2.34696	0.11796
C	-3.10738	2.28986	-0.27900
C	-3.68660	3.35756	-0.84585
H	-4.72907	3.31813	-1.14044
C	-2.89845	4.57658	-1.11956
C	-3.53005	5.71260	-1.82962
H	-4.57644	5.66785	-2.10501
C	-2.81034	6.79659	-2.13789
H	-3.27231	7.62530	-2.65877
C	-1.37993	6.86008	-1.78303
H	-0.79718	7.72977	-2.05801
C	-0.80069	5.84171	-1.13677
H	0.25552	5.89999	-0.91102
C	-1.59550	4.64205	-0.75586
C	-0.97412	3.49251	-0.02981
C	1.32282	2.64323	0.14015
C	2.73257	2.75112	0.58606
C	3.10983	3.77387	1.36696
H	4.14210	3.86204	1.68591
C	2.11056	4.74603	1.85596
C	2.52512	5.80932	2.80002
H	3.56457	5.90593	3.08811
C	1.61754	6.65177	3.30457
H	1.92125	7.42702	3.99611
C	0.19635	6.51690	2.93318
H	-0.53676	7.18562	3.36555
C	-0.18834	5.56336	2.07700
H	-1.23953	5.46721	1.84135

C	0.81395	4.63943	1.47878
C	0.41328	3.57290	0.51061
C	3.71457	1.72514	0.16240
C	4.15508	1.68175	-1.27442
C	4.17840	0.79171	1.04066
C	4.99121	0.70395	-1.67409
C	5.14843	-0.22662	0.56327
C	5.52768	-0.28615	-0.72127
H	5.29576	0.64205	-2.71177
H	5.54570	-0.94545	1.26775
C	-3.88295	1.04432	-0.06888
C	-4.25328	0.64984	1.18121
C	-4.26403	0.20817	-1.25758
C	-5.03396	-0.60588	1.34193
C	-5.04090	-0.87483	-1.07191
C	-5.44160	-1.31365	0.28088
H	-5.30308	-0.93220	2.33878
H	-5.35065	-1.47183	-1.92053
H	0.12174	-2.03590	0.74669
C	-1.25335	-3.25789	1.65514
C	-0.63094	-3.30737	-0.69841
C	-1.07544	-2.65964	2.91676
C	-2.23961	-4.25534	1.46443
C	-1.63255	-4.31948	-0.89790
C	-1.88488	-3.05214	3.96917
H	-0.32936	-1.88049	3.03548
C	-3.03850	-4.64098	2.56052
C	-2.37621	-4.78752	0.14286
H	-1.75824	-4.71502	-1.89574
C	-2.86881	-4.04520	3.79758
H	-1.76094	-2.58023	4.93911
H	-3.79524	-5.40651	2.41390
H	-3.10244	-5.57795	-0.02780
H	-3.49277	-4.33898	4.63523
N	-0.48133	-2.86157	0.58587
C	0.12444	-2.67496	-1.72606
C	1.62881	-2.37630	-1.60765
H	1.84068	-1.92355	-0.63805
H	1.87012	-1.61114	-2.35213
N	2.52720	-3.49335	-1.82438
H	2.50117	-3.86290	-2.76449
C	2.91311	-4.41085	-0.85367
C	3.54526	-5.60449	-1.25910
C	2.78541	-4.16761	0.52720
C	4.04758	-6.50105	-0.32314
H	3.65614	-5.80944	-2.32147
C	3.29718	-5.07317	1.45460
H	2.30711	-3.26372	0.88282
C	3.93281	-6.24521	1.04572
H	4.53632	-7.40796	-0.66887
H	3.19428	-4.85138	2.51357
H	4.32852	-6.94470	1.77499
H	-0.38876	-1.18756	-1.57048
Cl	-0.25336	-3.24185	-3.40432
C	-3.77800	0.54525	-2.66724
C	-3.06085	-0.63306	-3.35208
C	-4.93248	0.95497	-3.59283
H	-3.05357	1.38555	-2.64263
C	-3.90177	-1.00354	-4.56331
H	-2.92080	-1.51192	-2.68466

H	-2.05481	-0.30814	-3.69962
C	-4.68097	0.24968	-4.91514
H	-4.96070	2.05779	-3.73327
H	-5.91854	0.63060	-3.19260
H	-3.26723	-1.33721	-5.41279
H	-4.60895	-1.81877	-4.29372
H	-4.06198	0.89945	-5.57286
H	-5.63283	0.00248	-5.43321
C	-3.90473	1.44274	2.44300
C	-5.14913	1.85237	3.24570
C	-3.01252	0.63119	3.39460
H	-3.36425	2.38229	2.20607
C	-4.82119	1.56679	4.70204
H	-6.04978	1.26974	2.95485
H	-5.38101	2.93056	3.10156
C	-3.80644	0.43928	4.67692
H	-2.08460	1.20338	3.61862
H	-2.71198	-0.35053	2.97071
H	-4.36203	2.46848	5.16472
H	-5.72913	1.28137	5.27627
H	-3.14721	0.46815	5.57138
H	-4.33602	-0.53855	4.64637
C	-6.30212	-2.55131	0.46125
C	-7.69735	-2.36971	-0.14629
C	-5.69559	-3.79783	-0.20222
H	-6.43347	-2.77430	1.54547
C	-8.05546	-3.71257	-0.75518
H	-7.69919	-1.60007	-0.94993
H	-8.43876	-2.07357	0.62756
C	-6.73333	-4.31750	-1.18800
H	-5.49818	-4.57370	0.56977
H	-4.74133	-3.58652	-0.73060
H	-8.52657	-4.35972	0.01763
H	-8.75093	-3.59840	-1.61475
H	-6.77775	-5.42817	-1.18527
H	-6.48326	-3.96436	-2.21319
C	3.75269	0.73748	2.51131
C	3.15138	-0.62349	2.90310
C	4.94308	0.97779	3.45170
H	2.98335	1.50152	2.74574
C	3.85686	-1.06002	4.17754
H	3.30334	-1.39266	2.11621
H	2.05859	-0.53734	3.08793
C	5.18594	-0.32974	4.18841
H	4.68005	1.76788	4.19007
H	5.86222	1.30387	2.91622
H	3.25698	-0.74846	5.06131
H	4.00156	-2.16202	4.20456
H	5.53893	-0.15031	5.22706
H	5.94777	-0.93212	3.64683
C	3.66632	2.71063	-2.29463
C	4.81311	3.49854	-2.94566
C	2.86490	2.06659	-3.43850
H	3.00755	3.46306	-1.81527
C	4.44911	3.61806	-4.41541
H	5.78852	2.97304	-2.85636
H	4.92175	4.50471	-2.48439
C	3.61114	2.39122	-4.72415
H	1.84705	2.51486	-3.48071
H	2.74584	0.96641	-3.32302

H	3.83892	4.53460	-4.57551
H	5.35508	3.66243	-5.05792
H	2.90984	2.58231	-5.56509
H	4.28008	1.54335	-4.99126
C	6.50188	-1.34563	-1.20721
C	7.84579	-1.30818	-0.46036
C	5.94708	-2.76584	-1.04117
H	6.70909	-1.18753	-2.28992
C	7.99787	-2.66106	0.22111
H	7.90915	-0.48589	0.28609
H	8.67404	-1.17060	-1.19050
C	7.10794	-3.61350	-0.55514
H	5.54303	-3.15717	-2.00020
H	5.14217	-2.80001	-0.27721
H	9.05545	-3.00304	0.21786
H	7.63825	-2.59196	1.27185
H	7.66620	-4.01876	-1.42796
H	6.75860	-4.45492	0.08225

Catalyst (2,4,6-c-Pent, 10)-Enamine protonation TS (pro-R) alternative conformation
Electronic energy (Hartrees): -1870.448997
Free Energy at 1M concentration and 253 K (Hartrees): -1868.985317
Coordinates:

P	0.04748	0.51427	0.08865
O	1.29816	1.16531	-0.72555
O	-0.88539	1.75536	0.57506
O	0.57677	-0.05081	1.44005
O	-0.63964	-0.44596	-0.82838
C	1.82943	2.30028	-0.16500
C	3.21284	2.25009	0.36615
C	3.78794	3.36470	0.83921
H	4.79572	3.32925	1.23706
C	3.02962	4.63092	0.89309
C	3.64826	5.82809	1.50785
H	4.67039	5.79015	1.86372
C	2.94633	6.95946	1.63102
H	3.39861	7.83300	2.08256
C	1.54710	7.01555	1.16768
H	0.97601	7.92618	1.29510
C	0.98018	5.94149	0.60622
H	-0.05578	5.99884	0.30087
C	1.75872	4.68485	0.42837
C	1.15243	3.47248	-0.20301
C	-1.15318	2.66055	-0.42073
C	-2.53474	2.75873	-0.95039
C	-2.83588	3.69741	-1.85964
H	-3.84748	3.78064	-2.24026
C	-1.78003	4.57936	-2.39846
C	-2.10847	5.53997	-3.47694
H	-3.12736	5.63119	-3.83241
C	-1.14798	6.29554	-4.01980
H	-1.38870	6.99564	-4.80938
C	0.24621	6.16576	-3.55551
H	1.02289	6.76195	-4.01696
C	0.55307	5.30498	-2.57815
H	1.58624	5.20833	-2.27291
C	-0.50959	4.48157	-1.93900
C	-0.19625	3.52066	-0.83753
C	-3.57535	1.82238	-0.46272

C	-4.07963	1.95709	0.94749
C	-4.04410	0.81745	-1.25544
C	-4.97759	1.06566	1.41009
C	-5.08333	-0.09771	-0.71850
C	-5.52076	0.00172	0.54479
H	-5.32928	1.13159	2.43252
H	-5.48531	-0.87111	-1.35937
C	3.93225	0.95585	0.43475
C	4.40227	0.35077	-0.69065
C	4.13574	0.30138	1.77338
C	5.13441	-0.93544	-0.56557
C	4.85803	-0.83338	1.84461
C	5.38255	-1.48720	0.63085
H	5.49383	-1.42445	-1.46153
H	5.03038	-1.30663	2.80347
H	-0.15643	-2.07478	-0.62231
C	1.10469	-3.49165	-1.40939
C	0.45115	-3.29752	0.92937
C	1.00193	-2.98053	-2.71704
C	1.98719	-4.56056	-1.12308
C	1.36968	-4.36293	1.22962
C	1.77601	-3.53623	-3.72149
H	0.34131	-2.14154	-2.90974
C	2.74772	-5.11577	-2.17286
C	2.07814	-4.97925	0.24312
H	1.45509	-4.68049	2.25918
C	2.64876	-4.60935	-3.45653
H	1.71010	-3.13286	-4.72735
H	3.42524	-5.93574	-1.95135
H	2.73688	-5.80762	0.49005
H	3.24506	-5.03273	-4.25805
N	0.36309	-2.93943	-0.38880
C	-0.28266	-2.54183	1.88494
C	-1.76023	-2.15554	1.69922
H	-1.92441	-1.80866	0.67766
H	-1.96091	-1.29282	2.34213
N	-2.73952	-3.17214	2.02883
H	-2.76805	-3.40511	3.01168
C	-3.14233	-4.20041	1.18355
C	-3.86968	-5.27998	1.72596
C	-2.93816	-4.17457	-0.20903
C	-4.38946	-6.27486	0.90639
H	-4.04059	-5.31592	2.79943
C	-3.46803	-5.17695	-1.01963
H	-2.38119	-3.36666	-0.66670
C	-4.19805	-6.23402	-0.47729
H	-4.95224	-7.08923	1.35464
H	-3.30301	-5.12405	-2.09252
H	-4.60710	-7.01036	-1.11582
H	0.31277	-1.08392	1.66355
Cl	0.02091	-2.99204	3.61179
C	3.52486	0.88041	3.05021
C	2.67060	-0.13777	3.82841
C	4.59665	1.38952	4.02323
H	2.85704	1.73474	2.81481
C	3.32858	-0.30155	5.19000
H	2.57949	-1.12001	3.31503
H	1.64285	0.26521	3.97017
C	4.13794	0.96322	5.40700
H	4.69991	2.49521	3.96380

H	5.59059	0.93434	3.81708
H	2.57325	-0.44097	5.99352
H	4.01022	-1.18051	5.17260
H	3.48568	1.75190	5.84354
H	5.00003	0.78164	6.08477
C	4.20168	0.92328	-2.09504
C	5.52911	1.14525	-2.83757
C	3.35832	-0.01794	-2.96987
H	3.68597	1.90541	-2.07124
C	5.33423	0.57411	-4.23345
H	6.38035	0.62611	-2.34619
H	5.77954	2.22757	-2.89159
C	4.26325	-0.49163	-4.09531
H	2.49894	0.54189	-3.40188
H	2.95017	-0.88436	-2.40499
H	4.97358	1.37469	-4.91677
H	6.27897	0.14763	-4.63490
H	3.69762	-0.62269	-5.04318
H	4.73333	-1.46047	-3.81635
C	6.17325	-2.77827	0.75468
C	5.46914	-3.95939	0.07274
C	7.57293	-2.67633	0.12706
H	6.29936	-3.03040	1.83201
C	6.53968	-4.69516	-0.71485
H	4.67974	-3.61553	-0.62936
H	5.00228	-4.63293	0.82355
C	7.59164	-3.65185	-1.03977
H	8.33824	-2.98637	0.87298
H	7.82580	-1.64858	-0.21551
H	6.98905	-5.49132	-0.08084
H	6.12355	-5.15225	-1.63890
H	8.59408	-4.11461	-1.16793
H	7.31142	-3.12088	-1.97640
C	-3.55729	0.58208	-2.68888
C	-3.02152	-0.84419	-2.90511
C	-4.68874	0.78413	-3.70713
H	-2.73414	1.27393	-2.96192
C	-3.71851	-1.39106	-4.14170
H	-3.22888	-1.50805	-2.03888
H	-1.92219	-0.83422	-3.06824
C	-4.99327	-0.58381	-4.29596
H	-4.34072	1.46015	-4.51983
H	-5.60302	1.23214	-3.25850
H	-3.07235	-1.23445	-5.03384
H	-3.93853	-2.47583	-4.03704
H	-5.29513	-0.50721	-5.36303
H	-5.81432	-1.06749	-3.72306
C	-3.58876	3.07158	1.87246
C	-4.72518	3.97203	2.38003
C	-2.86625	2.52547	3.11553
H	-2.87743	3.74006	1.34637
C	-4.41791	4.23833	3.84345
H	-5.71878	3.47979	2.30299
H	-4.76891	4.92470	1.80780
C	-3.65002	3.02022	4.32221
H	-1.83156	2.93353	3.15628
H	-2.79309	1.41531	3.12566
H	-3.77403	5.14151	3.93132
H	-5.34702	4.39001	4.43449
H	-2.97646	3.27454	5.16907

H	-4.36729	2.23488	4.64849
C	-6.56934	-0.95100	1.09294
C	-7.87692	-0.92884	0.28354
C	-6.08502	-2.40610	1.10394
H	-6.81307	-0.66755	2.14196
C	-8.07605	-2.33986	-0.25265
H	-7.86302	-0.19306	-0.55061
H	-8.72583	-0.66829	0.95404
C	-7.27041	-3.24359	0.66160
H	-5.73900	-2.70968	2.11599
H	-5.25499	-2.56293	0.38319
H	-9.15007	-2.62607	-0.25949
H	-7.67249	-2.40595	-1.28754
H	-7.88353	-3.52118	1.54748
H	-6.94280	-4.16688	0.13573

Catalyst (2,4,6-c-Pent, 10)-Enamine protonation TS (pro-R) alternative conformation
Electronic energy (Hartrees): -1870.450089
Free Energy at 1M concentration and 253 K (Hartrees): -1868.985279
Coordinates:

P	-0.05604	0.47438	0.20714
O	1.14912	1.24283	-0.57411
O	-1.11804	1.62206	0.65341
O	0.49224	-0.01975	1.57930
O	-0.62962	-0.55487	-0.71344
C	1.60794	2.37074	0.06152
C	2.99072	2.37416	0.59816
C	3.48157	3.48296	1.17040
H	4.49000	3.48848	1.56809
C	2.63399	4.68280	1.32336
C	3.15891	5.86237	2.04919
H	4.17625	5.86422	2.42041
C	2.37780	6.92827	2.25373
H	2.76161	7.78886	2.78623
C	0.98466	6.92814	1.76869
H	0.34987	7.78365	1.96032
C	0.50217	5.86977	1.10739
H	-0.53026	5.88151	0.78488
C	1.36918	4.68963	0.84013
C	0.85773	3.49771	0.09714
C	-1.37298	2.55786	-0.31749
C	-2.70475	2.57338	-0.96903
C	-3.03431	3.57977	-1.79179
H	-4.00929	3.59655	-2.26542
C	-2.03345	4.60387	-2.15324
C	-2.37775	5.64056	-3.15354
H	-3.37953	5.69106	-3.56180
C	-1.45229	6.51504	-3.56148
H	-1.70435	7.27061	-4.29433
C	-0.07903	6.44028	-3.02874
H	0.67297	7.13228	-3.38545
C	0.24142	5.51107	-2.12096
H	1.26270	5.45749	-1.76954
C	-0.78685	4.55460	-1.62583
C	-0.46255	3.51894	-0.59600
C	-3.63942	1.44343	-0.75413
C	-4.32289	1.29774	0.57500
C	-3.84707	0.51130	-1.72824
C	-5.13350	0.24323	0.78105

C	-4.78772	-0.61050	-1.46608
C	-5.40248	-0.74544	-0.28362
H	-5.61892	0.11526	1.73941
H	-4.98388	-1.32435	-2.25611
C	3.81397	1.14206	0.53998
C	4.31124	0.68206	-0.64160
C	4.11080	0.39798	1.81280
C	5.16727	-0.53165	-0.64006
C	4.94004	-0.66289	1.77351
C	5.49834	-1.15448	0.50000
H	5.55427	-0.90249	-1.58029
H	5.18038	-1.19697	2.68448
H	-0.04218	-2.16166	-0.44869
C	1.34800	-3.45557	-1.22148
C	0.71686	-3.26869	1.12262
C	1.17821	-2.98399	-2.53693
C	2.32344	-4.43939	-0.93077
C	1.74355	-4.22750	1.42986
C	1.96816	-3.50470	-3.54698
H	0.44740	-2.20706	-2.73218
C	3.09498	-4.96660	-1.98762
C	2.48657	-4.80323	0.44331
H	1.88595	-4.50124	2.46550
C	2.92439	-4.50430	-3.27984
H	1.84710	-3.13388	-4.56018
H	3.83999	-5.72526	-1.76426
H	3.22811	-5.55565	0.69843
H	3.52929	-4.90425	-4.08693
N	0.57558	-2.95200	-0.19920
C	-0.08601	-2.58057	2.07678
C	-1.58801	-2.33354	1.87617
H	-1.75281	-1.82013	0.93011
H	-1.90315	-1.63496	2.65735
N	-2.44103	-3.50785	1.92684
H	-2.31666	-4.06721	2.75987
C	-2.87089	-4.21691	0.80917
C	-3.28311	-5.55632	0.95769
C	-2.99435	-3.62389	-0.46307
C	-3.81220	-6.26414	-0.11698
H	-3.19603	-6.03305	1.93151
C	-3.52242	-4.34561	-1.53076
H	-2.70151	-2.59193	-0.61492
C	-3.93840	-5.66837	-1.37377
H	-4.12607	-7.29393	0.03162
H	-3.61642	-3.85848	-2.49774
H	-4.35197	-6.22227	-2.21038
H	0.35092	-1.06994	1.83624
Cl	0.22028	-3.05390	3.80059
C	3.47863	0.80363	3.14474
C	2.74623	-0.35667	3.84334
C	4.52063	1.32909	4.14167
H	2.72603	1.60521	2.99473
C	3.46198	-0.58491	5.16556
H	2.72553	-1.29051	3.23984
H	1.69268	-0.06431	4.05076
C	4.15152	0.72762	5.48699
H	4.50631	2.44010	4.18837
H	5.55006	1.00473	3.87200
H	2.75075	-0.87551	5.96891
H	4.22429	-1.38603	5.04522

H	3.44065	1.40151	6.01479
H	5.04913	0.56872	6.12307
C	4.02962	1.35210	-1.98790
C	5.31481	1.76560	-2.72276
C	3.26169	0.41414	-2.93379
H	3.42505	2.27485	-1.87287
C	5.14304	1.31027	-4.16331
H	6.22077	1.28536	-2.29371
H	5.46329	2.86700	-2.67786
C	4.18290	0.13668	-4.11064
H	2.34266	0.92361	-3.30063
H	2.95096	-0.53700	-2.44725
H	4.69110	2.13166	-4.76255
H	6.11468	1.01729	-4.61686
H	3.61177	0.03831	-5.05917
H	4.75064	-0.80258	-3.92915
C	6.42446	-2.35856	0.50010
C	5.84304	-3.54143	-0.28594
C	7.79588	-2.04733	-0.12120
H	6.59234	-2.69383	1.54870
C	6.97848	-4.08585	-1.13529
H	5.01698	-3.22022	-0.95569
H	5.45572	-4.32569	0.39951
C	7.90729	-2.90978	-1.36918
H	8.60069	-2.33312	0.59211
H	7.92857	-0.97205	-0.37379
H	7.51814	-4.87990	-0.57308
H	6.60437	-4.50433	-2.09496
H	8.95251	-3.24751	-1.53923
H	7.56036	-2.33293	-2.25501
C	-3.17226	0.57226	-3.10326
C	-2.33136	-0.67627	-3.40816
C	-4.19966	0.69801	-4.23944
H	-2.48154	1.43525	-3.18898
C	-2.66224	-1.07413	-4.83612
H	-2.57535	-1.52034	-2.73153
H	-1.24555	-0.45832	-3.31233
C	-4.06582	-0.55816	-5.08686
H	-3.95886	1.58751	-4.86350
H	-5.24371	0.81320	-3.87316
H	-1.95378	-0.57753	-5.53572
H	-2.60437	-2.17554	-4.97552
H	-4.22961	-0.34123	-6.16465
H	-4.80606	-1.31765	-4.75160
C	-4.11656	2.31113	1.70082
C	-5.42711	2.97944	2.14650
C	-3.49667	1.67097	2.95420
H	-3.43986	3.12882	1.37899
C	-5.38928	3.00192	3.66545
H	-6.32343	2.41144	1.81458
H	-5.50797	4.01208	1.74148
C	-4.52168	1.82390	4.06717
H	-2.56846	2.21801	3.23313
H	-3.22981	0.60060	2.81143
H	-4.91585	3.94690	4.01305
H	-6.40907	2.92035	4.10016
H	-4.03256	1.99859	5.04988
H	-5.14701	0.90577	4.12819
C	-6.40602	-1.86460	-0.05621
C	-6.02123	-2.76538	1.12764

C	-7.80784	-1.31617	0.24722
H	-6.47670	-2.50372	-0.96651
C	-7.25219	-2.86812	2.01565
H	-5.17594	-2.34495	1.71576
H	-5.72658	-3.77601	0.77235
C	-8.09417	-1.64639	1.70255
H	-8.55498	-1.83341	-0.39513
H	-7.89808	-0.22212	0.06564
H	-7.82373	-3.78690	1.75645
H	-6.97562	-2.90201	3.09180
H	-9.17482	-1.84735	1.86807
H	-7.77962	-0.79908	2.35043

Catalyst (2,4,6-c-Pent, 10)-Enamine protonation TS (pro-R) alternative conformation
Electronic energy (Hartrees): -1870.448131
Free Energy at 1M concentration and 253 K (Hartrees): -1868.985069
Coordinates:

P	0.00435	0.08130	0.04651
O	1.13024	1.02181	-0.66078
O	-1.13919	1.07121	0.66301
O	0.51972	-0.76188	1.16711
O	-0.60180	-0.66377	-1.18530
C	1.55999	2.04941	0.14270
C	2.95152	2.03639	0.65696
C	3.38325	3.03226	1.44394
H	4.39942	3.02573	1.82154
C	2.47438	4.13134	1.82522
C	2.94384	5.18026	2.75959
H	3.96077	5.16200	3.13147
C	2.11319	6.15227	3.15115
H	2.45645	6.91725	3.83559
C	0.72145	6.17697	2.66270
H	0.04743	6.95278	3.00240
C	0.28903	5.23722	1.81407
H	-0.74261	5.26156	1.48978
C	1.21017	4.16674	1.34318
C	0.75343	3.10542	0.39515
C	-1.45724	2.16003	-0.11107
C	-2.83227	2.27509	-0.65604
C	-3.19661	3.37878	-1.32580
H	-4.20099	3.46608	-1.72440
C	-2.21825	4.45255	-1.58609
C	-2.61140	5.61775	-2.41165
H	-3.62593	5.70589	-2.78013
C	-1.71452	6.56367	-2.70965
H	-2.00210	7.41371	-3.31480
C	-0.32558	6.44365	-2.22760
H	0.40164	7.20013	-2.49307
C	0.03846	5.39749	-1.47709
H	1.06885	5.31825	-1.15763
C	-0.95603	4.35227	-1.10888
C	-0.57909	3.17871	-0.26313
C	-3.79536	1.16057	-0.49367
C	-4.42938	0.91493	0.84606
C	-4.13593	0.37926	-1.55505
C	-5.34539	-0.06710	0.96652
C	-5.20400	-0.63852	-1.38972
C	-5.78065	-0.86047	-0.20003
H	-5.80983	-0.25986	1.92605

H	-5.53205	-1.19958	-2.25568
C	3.87037	0.93081	0.30341
C	4.48353	0.89277	-0.91173
C	4.20555	-0.09616	1.34533
C	5.50610	-0.15721	-1.16958
C	5.17936	-0.98790	1.08944
C	5.86790	-1.02981	-0.21817
H	6.01566	-0.17075	-2.12511
H	5.44761	-1.71862	1.83465
H	-0.40398	-2.16849	1.51934
C	-1.98579	-2.66354	2.73159
C	-1.68062	-3.35404	0.41697
C	-1.46266	-1.98477	3.84829
C	-3.27909	-3.23697	2.78177
C	-2.99744	-3.92820	0.45929
C	-2.22216	-1.89856	5.00225
H	-0.48620	-1.51898	3.77905
C	-4.02775	-3.13080	3.97261
C	-3.74939	-3.88051	1.59476
H	-3.35825	-4.42131	-0.43175
C	-3.50678	-2.47329	5.07203
H	-1.82342	-1.36956	5.86229
H	-5.02087	-3.56973	4.00755
H	-4.72899	-4.34913	1.61430
H	-4.08691	-2.39085	5.98516
C	-0.85409	-3.28024	-0.74396
H	-0.82946	-1.73511	-1.01485
C	0.64723	-3.55829	-0.64122
H	0.80546	-4.64180	-0.70846
H	1.00366	-3.23924	0.35031
N	1.37998	-2.92118	-1.71210
N	-1.24607	-2.76936	1.57351
C	2.64852	-3.27878	-2.13043
C	3.31310	-4.43904	-1.68977
C	3.29985	-2.45976	-3.07710
C	4.56231	-4.77421	-2.21265
H	2.86096	-5.08256	-0.94352
C	4.55422	-2.79337	-3.56883
H	2.79194	-1.56569	-3.42823
C	5.19933	-3.96029	-3.14782
H	5.04993	-5.68020	-1.86210
H	5.03120	-2.13718	-4.29206
H	6.17925	-4.22203	-3.53382
H	1.08090	-1.98703	-1.95221
Cl	-1.54036	-4.07233	-2.21478
C	-4.07096	1.75888	2.06914
C	-5.29483	2.43968	2.70716
C	-3.40625	0.92707	3.17343
H	-3.36214	2.56798	1.79865
C	-5.37969	1.92354	4.13682
H	-6.24305	2.23830	2.16208
H	-5.14343	3.54212	2.72452
C	-3.98530	1.43525	4.48096
H	-3.64723	-0.15210	3.07096
H	-2.30053	1.04216	3.15991
H	-6.09246	1.07058	4.18419
H	-5.71357	2.71840	4.83842
H	-4.01444	0.63618	5.25291
H	-3.37358	2.28628	4.85431
C	-3.47771	0.51069	-2.92951

C	-2.94807	-0.83233	-3.46102
C	-4.45955	1.04133	-3.98166
H	-2.60878	1.20216	-2.90411
C	-3.69875	-1.11480	-4.75382
H	-3.08225	-1.66803	-2.74165
H	-1.86064	-0.74785	-3.68235
C	-4.19778	0.23259	-5.23989
H	-5.51742	0.89212	-3.67094
H	-4.30009	2.12591	-4.16828
H	-4.56639	-1.77924	-4.54539
H	-3.04196	-1.59868	-5.50887
H	-5.11726	0.12661	-5.85545
H	-3.40484	0.72741	-5.84356
C	-6.91729	-1.85717	-0.04897
C	-8.18151	-1.40108	-0.79146
C	-6.57393	-3.25702	-0.58596
H	-7.17368	-1.95991	1.02995
C	-8.74575	-2.64376	-1.45755
H	-7.94946	-0.64736	-1.57663
H	-8.92184	-0.95823	-0.08990
C	-7.54741	-3.53487	-1.72134
H	-5.52620	-3.34341	-0.94457
H	-6.72877	-4.01187	0.21609
H	-9.28111	-2.39488	-2.39939
H	-9.44496	-3.15686	-0.76055
H	-7.08104	-3.25231	-2.69123
H	-7.84003	-4.60670	-1.75472
C	3.47765	-0.12661	2.69249
C	2.96304	-1.52226	3.06810
C	4.38782	0.30633	3.84106
H	2.60155	0.55482	2.66779
C	3.88426	-2.06893	4.15678
H	2.92093	-2.20906	2.19368
H	1.93307	-1.43891	3.47779
C	4.87628	-0.96685	4.51501
H	5.23984	0.93373	3.49717
H	3.79586	0.89880	4.57392
H	4.42476	-2.97000	3.79240
H	3.28951	-2.35415	5.05238
H	5.88783	-1.22842	4.13504
H	4.94073	-0.83045	5.61655
C	4.22474	1.93925	-1.99795
C	5.47143	2.75116	-2.36432
C	3.72914	1.33255	-3.30549
H	3.45346	2.67607	-1.69043
C	5.40058	2.99463	-3.87237
H	6.41234	2.21245	-2.11983
H	5.47742	3.71654	-1.81165
C	4.11230	2.34640	-4.36671
H	4.24170	0.37447	-3.53147
H	2.63033	1.16303	-3.28417
H	6.27405	2.52062	-4.37217
H	5.40375	4.08258	-4.10160
H	4.25394	1.86121	-5.35683
H	3.31343	3.11666	-4.44817
C	7.01666	-1.99857	-0.46496
C	8.27369	-1.56657	0.28785
C	6.71547	-3.42202	0.01969
H	7.24870	-2.03459	-1.55422
C	8.29315	-2.36454	1.58034

H	8.30087	-0.47223	0.48738
H	9.17064	-1.83355	-0.31433
C	7.50289	-3.63939	1.31159
H	5.62901	-3.59212	0.18414
H	7.06234	-4.15361	-0.74303
H	7.80448	-1.77819	2.38866
H	9.33557	-2.59491	1.89060
H	6.81671	-3.85607	2.15952
H	8.19911	-4.49857	1.19188

Catalyst (2,4,6-c-Pent, 10)-Enamine protonation TS (pro-R) alternative conformation
Electronic energy (Hartrees): -1870.448726
Free Energy at 1M concentration and 253 K (Hartrees): -1868.984965
Coordinates:

P	-0.02136	0.32570	0.28330
O	1.16439	1.00915	-0.59597
O	-1.21890	1.42779	0.39541
O	0.37520	-0.05819	1.67119
O	-0.49154	-0.82258	-0.66592
C	1.53324	2.24657	-0.12950
C	2.87332	2.41723	0.48174
C	3.25246	3.62059	0.93536
H	4.23220	3.74993	1.38088
C	2.32663	4.76976	0.86637
C	2.72598	6.06948	1.45357
H	3.70729	6.18790	1.89603
C	1.87529	7.10127	1.44373
H	2.16727	8.05011	1.87493
C	0.53026	6.94557	0.85787
H	-0.16160	7.77781	0.87423
C	0.16129	5.77725	0.32008
H	-0.83817	5.67878	-0.08195
C	1.10752	4.62883	0.29408
C	0.72162	3.31445	-0.30284
C	-1.42484	2.19204	-0.72726
C	-2.69999	2.04229	-1.46937
C	-2.98865	2.87504	-2.47992
H	-3.91810	2.76378	-3.02672
C	-2.00324	3.88044	-2.92302
C	-2.29866	4.72062	-4.10656
H	-3.25872	4.64438	-4.60183
C	-1.37986	5.57282	-4.57240
H	-1.59584	6.18454	-5.43879
C	-0.06150	5.66819	-3.91827
H	0.68812	6.33905	-4.31781
C	0.21382	4.91739	-2.84559
H	1.19848	4.98391	-2.40361
C	-0.81136	3.99051	-2.29045
C	-0.53665	3.15035	-1.08340
C	-3.61436	0.92138	-1.14890
C	-4.43019	0.96416	0.11139
C	-3.70934	-0.15432	-1.97895
C	-5.24086	-0.07332	0.40176
C	-4.68868	-1.22426	-1.66463
C	-5.40524	-1.20530	-0.53182
H	-5.81629	-0.07112	1.31962
H	-4.82024	-2.03780	-2.36734
C	3.79229	1.26039	0.58439
C	4.41355	0.76267	-0.51984

C	4.04694	0.63818	1.92857
C	5.34427	-0.38457	-0.36878
C	4.93601	-0.36935	2.02541
C	5.61572	-0.91220	0.83354
H	5.83087	-0.78515	-1.24903
H	5.14760	-0.81437	2.98963
H	-0.51714	-1.35212	2.40209
C	-2.18801	-1.55506	3.57639
C	-1.61225	-2.92896	1.64992
C	-1.81907	-0.51009	4.44422
C	-3.45633	-2.17254	3.69719
C	-2.89402	-3.56810	1.77840
C	-2.70780	-0.09815	5.42252
H	-0.85908	-0.02367	4.31414
C	-4.33670	-1.73229	4.70711
C	-3.76759	-3.20640	2.75866
H	-3.12832	-4.36118	1.08368
C	-3.96921	-0.70893	5.56195
H	-2.42837	0.71449	6.08588
H	-5.31005	-2.20647	4.79626
H	-4.72388	-3.71505	2.84525
H	-4.65236	-0.36955	6.33350
C	-0.68432	-3.16209	0.59462
H	-0.69905	-1.78127	-0.16676
C	0.82109	-3.25934	0.88617
H	1.04565	-4.27095	1.23899
H	1.05839	-2.57023	1.70584
N	1.65558	-2.97669	-0.26460
N	-1.32533	-1.97159	2.58454
C	2.51551	-3.87948	-0.87477
C	3.07917	-4.98120	-0.20329
C	2.88893	-3.66207	-2.21747
C	3.96885	-5.83015	-0.86091
H	2.84992	-5.16314	0.84098
C	3.79199	-4.50468	-2.85410
H	2.45078	-2.82710	-2.75840
C	4.34001	-5.60283	-2.18560
H	4.39021	-6.67089	-0.31609
H	4.06172	-4.30564	-3.88791
H	5.04331	-6.26149	-2.68494
H	1.30921	-2.24417	-0.86837
Cl	-1.18397	-4.42024	-0.59897
C	-2.84616	-0.32075	-3.23165
C	-2.11439	-1.67430	-3.27341
C	-3.68353	-0.23676	-4.51362
H	-2.05965	0.46034	-3.29677
C	-2.60141	-2.39348	-4.52263
H	-2.29416	-2.29696	-2.37104
H	-1.01684	-1.50677	-3.35363
C	-3.13420	-1.30815	-5.43870
H	-3.60264	0.76920	-4.98066
H	-4.75859	-0.44631	-4.31821
H	-1.78193	-2.96602	-5.00875
H	-3.42563	-3.09125	-4.25507
H	-2.29949	-0.88789	-6.04260
H	-3.91998	-1.70145	-6.11945
C	-4.35107	2.14726	1.07589
C	-5.70489	2.84450	1.27597
C	-3.85785	1.73112	2.47014
H	-3.65207	2.92107	0.69777

C	-5.79202	3.15952	2.75946
H	-6.55909	2.19094	0.99464
H	-5.77013	3.77565	0.67139
C	-4.96724	2.08821	3.44767
H	-2.93706	2.29921	2.73067
H	-3.61968	0.64914	2.53306
H	-5.34234	4.15807	2.95621
H	-6.84450	3.15293	3.11701
H	-4.55648	2.45160	4.41452
H	-5.60224	1.19378	3.63395
C	-6.42053	-2.29201	-0.22416
C	-7.60981	-2.25825	-1.19216
C	-5.83409	-3.71261	-0.29920
H	-6.81785	-2.14271	0.80560
C	-7.95843	-3.70986	-1.46555
H	-7.34316	-1.76664	-2.15422
H	-8.47511	-1.71872	-0.74892
C	-6.64752	-4.46336	-1.34423
H	-5.94777	-4.21730	0.68524
H	-4.75678	-3.72679	-0.57085
H	-8.67122	-4.07506	-0.69320
H	-8.40901	-3.83853	-2.47353
H	-6.81588	-5.52127	-1.04746
H	-6.11445	-4.43773	-2.32065
C	4.21870	1.34361	-1.92179
C	5.54827	1.74973	-2.58072
C	3.55182	0.33382	-2.86588
H	3.58157	2.25236	-1.90901
C	5.69655	0.88672	-3.82555
H	6.42690	1.62518	-1.91035
H	5.50457	2.82009	-2.88260
C	4.29167	0.44336	-4.18696
H	2.47127	0.55892	-3.00043
H	3.64575	-0.70650	-2.48650
H	6.16827	1.45125	-4.65879
H	6.31866	-0.00531	-3.59140
H	3.80852	1.21869	-4.82206
H	4.29947	-0.52611	-4.73090
C	3.32396	1.12325	3.18530
C	2.62040	-0.00975	3.95420
C	4.28328	1.79687	4.17673
H	2.53899	1.86340	2.92521
C	3.29099	-0.08691	5.31680
H	2.66414	-0.99133	3.43317
H	1.54922	0.24910	4.09901
C	3.88976	1.28678	5.55268
H	4.19805	2.90458	4.12711
H	5.34258	1.52018	3.97917
H	2.56378	-0.35318	6.11428
H	4.10209	-0.84764	5.29304
H	3.11897	1.95751	5.99314
H	4.76584	1.23522	6.23484
C	6.60260	-2.05635	0.98747
C	6.14512	-3.32394	0.25404
C	7.99932	-1.71187	0.44423
H	6.70928	-2.30778	2.06709
C	7.37952	-3.88069	-0.43281
H	5.37936	-3.09711	-0.51856
H	5.71974	-4.06630	0.96391
C	8.26592	-2.67909	-0.69964

H	8.75866	-1.87139	1.24178
H	8.08654	-0.65917	0.09533
H	7.90239	-4.58495	0.25155
H	7.11842	-4.40916	-1.37528
H	9.33771	-2.96978	-0.74660
H	7.97149	-2.20790	-1.66364

Catalyst (2,4,6-c-Pent, 10)-Enamine protonation TS (pro-R) alternative conformation
Electronic energy (Hartrees): -1870.449565
Free Energy at 1M concentration and 253 K (Hartrees): -1868.984541
Coordinates:

P	0.11722	0.45700	-0.19312
O	-0.99646	1.32549	0.61965
O	1.15988	1.53075	-0.82822
O	-0.55975	-0.13838	-1.46224
O	0.74724	-0.50479	0.76363
C	-1.49177	2.40291	-0.07359
C	-2.92180	2.39969	-0.46854
C	-3.44380	3.46598	-1.09220
H	-4.48839	3.46867	-1.38206
C	-2.58908	4.61822	-1.44390
C	-3.15748	5.74033	-2.22589
H	-4.20615	5.73871	-2.49622
C	-2.37761	6.75806	-2.60567
H	-2.79344	7.57651	-3.17900
C	-0.94390	6.76090	-2.25817
H	-0.31302	7.57527	-2.59037
C	-0.42150	5.75477	-1.54749
H	0.63740	5.76523	-1.32654
C	-1.28302	4.63175	-1.08703
C	-0.72653	3.50027	-0.28476
C	1.51351	2.54280	0.02809
C	2.89218	2.57501	0.57192
C	3.31292	3.64433	1.26300
H	4.32181	3.67219	1.65863
C	2.37056	4.73080	1.59899
C	2.82111	5.84684	2.46229
H	3.85447	5.90499	2.78099
C	1.95339	6.78422	2.85709
H	2.28323	7.59790	3.49004
C	0.53885	6.70112	2.44814
H	-0.16475	7.44617	2.79663
C	0.12220	5.70019	1.66413
H	-0.92623	5.64438	1.40550
C	1.08329	4.67020	1.18142
C	0.65047	3.55190	0.28636
C	3.77103	1.39285	0.40885
C	4.34581	1.08530	-0.94380
C	4.01608	0.55313	1.45571
C	5.09723	-0.02059	-1.09869
C	4.89060	-0.63084	1.24289
C	5.40746	-0.91070	0.03923
H	5.50310	-0.26509	-2.07128
H	5.11770	-1.27183	2.08541
C	-3.76674	1.21269	-0.18911
C	-4.12555	0.88919	1.08544
C	-4.24547	0.36461	-1.33526
C	-4.98690	-0.30133	1.31475
C	-5.09823	-0.64635	-1.08597

C	-5.48595	-1.00746	0.29290
H	-5.24760	-0.57241	2.33017
H	-5.48375	-1.24519	-1.90052
H	0.05358	-2.09502	0.70472
C	-1.40722	-3.15827	1.67072
C	-0.87577	-3.29123	-0.70046
C	-1.13135	-2.56320	2.91606
C	-2.48867	-4.06042	1.52861
C	-1.96989	-4.21155	-0.84995
C	-1.93892	-2.86216	3.99959
H	-0.31069	-1.85907	2.99775
C	-3.28456	-4.35308	2.65566
C	-2.71622	-4.59703	0.22268
H	-2.16712	-4.60635	-1.83631
C	-3.01848	-3.75874	3.87587
H	-1.73822	-2.39142	4.95712
H	-4.11531	-5.04442	2.54585
H	-3.51409	-5.32275	0.08901
H	-3.63978	-3.97924	4.73765
N	-0.63626	-2.85312	0.57090
C	-0.10290	-2.74578	-1.76693
C	1.41975	-2.56525	-1.68709
H	1.67175	-1.96061	-0.81709
H	1.71641	-1.97628	-2.56040
N	2.20721	-3.78488	-1.65404
H	2.00445	-4.42393	-2.41090
C	2.66955	-4.38993	-0.48903
C	2.99718	-5.76034	-0.50606
C	2.90790	-3.66651	0.69667
C	3.55436	-6.37530	0.61070
H	2.82081	-6.33630	-1.41179
C	3.46267	-4.29626	1.80810
H	2.68275	-2.60783	0.74492
C	3.79364	-5.65177	1.78091
H	3.80066	-7.43277	0.56412
H	3.64607	-3.71117	2.70541
H	4.22900	-6.13341	2.65055
H	-0.46368	-1.21705	-1.64073
Cl	-0.55413	-3.36215	-3.41199
C	-3.79418	0.62350	-2.77463
C	-3.14680	-0.61037	-3.43068
C	-4.96584	1.03729	-3.67596
H	-3.03925	1.43529	-2.80853
C	-3.86542	-0.83041	-4.75446
H	-3.23574	-1.52220	-2.80018
H	-2.06486	-0.43686	-3.61740
C	-5.19972	-0.11762	-4.63549
H	-4.68376	1.94233	-4.25903
H	-5.89162	1.27184	-3.10530
H	-3.27557	-0.37280	-5.57946
H	-4.00521	-1.91299	-4.96494
H	-5.55351	0.24398	-5.62528
H	-5.95869	-0.81163	-4.21283
C	-3.69355	1.70315	2.30793
C	-4.88676	2.21749	3.12857
C	-2.82555	0.87109	3.26448
H	-3.10539	2.59925	2.02282
C	-4.54044	1.95278	4.58483
H	-5.83190	1.68959	2.87737
H	-5.05051	3.30431	2.95794

C	-3.59691	0.76486	4.57007
H	-1.86153	1.39578	3.44911
H	-2.59150	-0.13889	2.86541
H	-4.01603	2.83714	5.01014
H	-5.44944	1.73991	5.18812
H	-2.91464	0.77894	5.44746
H	-4.18514	-0.17924	4.58165
C	-6.44642	-2.15636	0.54345
C	-7.84304	-1.86226	-0.01394
C	-5.98645	-3.47160	-0.10480
H	-6.55283	-2.33745	1.63820
C	-8.35436	-3.18605	-0.55022
H	-7.80582	-1.12800	-0.84928
H	-8.51747	-1.46544	0.77591
C	-7.11694	-3.92915	-1.01718
H	-5.81447	-4.23690	0.68365
H	-5.04830	-3.36154	-0.68949
H	-8.84541	-3.75667	0.26906
H	-9.07732	-3.03762	-1.38142
H	-7.26356	-5.02972	-0.96394
H	-6.88782	-3.64162	-2.06747
C	3.44732	0.77838	2.86134
C	2.58280	-0.39440	3.34726
C	4.55963	0.96607	3.90531
H	2.79918	1.67657	2.90836
C	2.99912	-0.66346	4.78283
H	2.74599	-1.31148	2.74533
H	1.50187	-0.13839	3.30240
C	4.43680	-0.19227	4.88374
H	4.40219	1.92354	4.45037
H	5.57786	0.99381	3.45820
H	2.36348	-0.06658	5.47398
H	2.90702	-1.74154	5.03806
H	4.68670	0.12227	5.92014
H	5.11960	-1.01547	4.57823
C	4.09251	1.99019	-2.14953
C	5.38962	2.55068	-2.75471
C	3.35500	1.25735	-3.28270
H	3.47353	2.86544	-1.86476
C	5.23845	2.42089	-4.26123
H	6.28672	1.97982	-2.42984
H	5.53900	3.61439	-2.46643
C	4.29841	1.24943	-4.47540
H	2.43094	1.81692	-3.55029
H	3.05725	0.22053	-3.01125
H	4.77693	3.34659	-4.67114
H	6.21874	2.24888	-4.75625
H	3.74378	1.34614	-5.43379
H	4.88154	0.30202	-4.48458
C	6.34369	-2.09423	-0.14495
C	5.82888	-3.09026	-1.19554
C	7.73789	-1.64819	-0.60904
H	6.46112	-2.64086	0.81917
C	6.98122	-3.34574	-2.15521
H	4.95960	-2.68887	-1.76161
H	5.51904	-4.04295	-0.71498
C	7.89468	-2.14100	-2.03794
H	8.51234	-2.13119	0.02757
H	7.88581	-0.54682	-0.55304
H	7.53285	-4.25956	-1.84114

H	6.62017	-3.47792	-3.19823
H	8.94973	-2.41082	-2.26074
H	7.56484	-1.35093	-2.74761

Catalyst (2,4,6-c-Pent, 10)-Enamine protonation TS (pro-R) alternative conformation
Electronic energy (Hartrees): -1870.448147
Free Energy at 1M concentration and 253 K (Hartrees): -1868.984284
Coordinates:

P	0.20287	0.17280	-0.30462
O	1.33770	1.16481	-0.91345
O	-1.00783	1.13080	0.23601
O	0.65623	-0.66251	0.85246
O	-0.29529	-0.58500	-1.57031
C	1.55630	2.30842	-0.18531
C	2.83921	2.46425	0.54065
C	3.13294	3.62305	1.14594
H	4.06425	3.73055	1.69031
C	2.14442	4.71862	1.17493
C	2.44700	5.95408	1.93382
H	3.41624	6.08283	2.39943
C	1.52348	6.91326	2.05288
H	1.74471	7.81448	2.60997
C	0.19248	6.73693	1.44309
H	-0.56146	7.50306	1.57061
C	-0.08950	5.62730	0.75079
H	-1.08435	5.50885	0.34454
C	0.94096	4.56639	0.57265
C	0.65851	3.32028	-0.20766
C	-1.41642	2.09108	-0.65663
C	-2.80268	2.03527	-1.17844
C	-3.24062	2.98596	-2.01736
H	-4.25820	2.95660	-2.38966
C	-2.33134	4.06053	-2.46566
C	-2.79431	5.04260	-3.47292
H	-3.80620	4.99342	-3.85556
C	-1.96366	5.99212	-3.91700
H	-2.30218	6.70832	-4.65452
C	-0.57862	6.05886	-3.41255
H	0.09458	6.81682	-3.79182
C	-0.15198	5.17957	-2.49864
H	0.87350	5.23290	-2.15770
C	-1.07273	4.13585	-1.97270
C	-0.62351	3.14808	-0.94619
C	-3.71168	0.95442	-0.72978
C	-4.21719	0.96041	0.68495
C	-4.06408	-0.05762	-1.56776
C	-5.03017	-0.03260	1.09463
C	-4.99192	-1.11059	-1.07914
C	-5.49088	-1.09175	0.16568
H	-5.35376	-0.05208	2.12531
H	-5.28390	-1.90100	-1.75876
C	3.73527	1.29858	0.69655
C	4.51580	0.87145	-0.33162
C	3.74302	0.55796	2.00322
C	5.33014	-0.36211	-0.15519
C	4.55470	-0.50467	2.14443
C	5.37493	-1.00419	1.02112
H	5.91934	-0.72610	-0.98725
H	4.59674	-1.02938	3.09011

H	-0.19740	-2.12236	0.96279
C	-1.86653	-2.87233	1.90571
C	-1.10648	-3.46266	-0.32374
C	-1.64238	-2.12436	3.07678
C	-3.03564	-3.65916	1.76695
C	-2.30377	-4.23810	-0.48485
C	-2.57293	-2.17782	4.09890
H	-0.75722	-1.50304	3.14556
C	-3.95570	-3.70537	2.83585
C	-3.21403	-4.34251	0.52511
H	-2.45179	-4.75051	-1.42460
C	-3.73106	-2.97331	3.98641
H	-2.40817	-1.59262	4.99833
H	-4.84715	-4.31770	2.73321
H	-4.09943	-4.95802	0.38977
H	-4.44665	-3.00491	4.80135
C	-0.13209	-3.22189	-1.34685
H	-0.28898	-1.70929	-1.52208
C	1.35712	-3.33561	-1.02285
H	1.64308	-4.39825	-0.97954
H	1.53389	-2.90524	-0.02841
N	2.16317	-2.65427	-2.02205
N	-0.95102	-2.83681	0.87706
C	3.42354	-3.08791	-2.40104
C	4.23013	-3.87126	-1.55553
C	3.94279	-2.71372	-3.65786
C	5.51301	-4.24687	-1.95426
H	3.85947	-4.16941	-0.58147
C	5.22921	-3.07830	-4.03344
H	3.32611	-2.12121	-4.32969
C	6.03002	-3.84992	-3.18614
H	6.11827	-4.85172	-1.28458
H	5.60651	-2.76396	-5.00289
H	7.03350	-4.13714	-3.48347
H	1.63986	-2.16972	-2.73578
Cl	-0.48439	-4.05699	-2.91920
C	-3.56120	-0.15722	-3.00945
C	-2.92365	-1.52028	-3.32318
C	-4.69863	0.03589	-4.02076
H	-2.78796	0.60806	-3.23046
C	-3.75819	-2.14841	-4.42882
H	-2.87613	-2.19140	-2.44027
H	-1.88529	-1.37174	-3.69440
C	-4.46498	-0.99366	-5.11231
H	-4.69194	1.06569	-4.44032
H	-5.69517	-0.14709	-3.56110
H	-3.12468	-2.71552	-5.14486
H	-4.51175	-2.83582	-3.98498
H	-3.80262	-0.56335	-5.89594
H	-5.42065	-1.32054	-5.57640
C	-3.83152	2.06502	1.67218
C	-5.05245	2.74921	2.31218
C	-2.96868	1.53453	2.82720
H	-3.25278	2.86412	1.16571
C	-4.97705	2.45738	3.80340
H	-6.02164	2.40222	1.89138
H	-4.99043	3.84920	2.15402
C	-3.51477	2.17484	4.09141
H	-1.89798	1.79970	2.68753
H	-3.04306	0.43037	2.92078

H	-5.34880	3.31463	4.40542
H	-5.58331	1.55525	4.04009
H	-2.98228	3.13252	4.28445
H	-3.39686	1.50473	4.97056
C	-6.46865	-2.18045	0.59612
C	-7.12889	-1.97340	1.95710
C	-7.65646	-2.32912	-0.35632
H	-5.92076	-3.14823	0.61830
C	-8.35785	-2.86498	1.93082
H	-7.46939	-0.91986	2.07105
H	-6.45063	-2.24215	2.79415
C	-8.78500	-2.94690	0.46753
H	-7.39917	-2.97724	-1.22239
H	-7.97762	-1.33123	-0.73159
H	-8.08872	-3.87870	2.30222
H	-9.16818	-2.44822	2.56778
H	-8.94424	-4.00782	0.17440
H	-9.73094	-2.38490	0.30594
C	4.60580	1.62198	-1.66264
C	6.05694	1.92975	-2.07020
C	3.98872	0.81790	-2.81211
H	4.07447	2.59661	-1.61804
C	6.31902	1.15400	-3.35244
H	6.80049	1.66211	-1.28788
H	6.16354	3.01813	-2.27722
C	4.95611	0.93089	-3.97798
H	2.98977	1.21648	-3.09371
H	3.86990	-0.24842	-2.53476
H	6.99084	1.71489	-4.03769
H	6.78219	0.17192	-3.11123
H	4.68586	1.81121	-4.60233
H	4.94490	0.01691	-4.60965
C	2.86524	0.98965	3.17919
C	1.98371	-0.14616	3.72827
C	3.69614	1.51060	4.35942
H	2.17380	1.80351	2.87977
C	2.39073	-0.34600	5.18027
H	2.08363	-1.09698	3.16043
H	0.91561	0.16333	3.69055
C	3.03082	0.96090	5.60900
H	3.71509	2.62240	4.37721
H	4.74573	1.14399	4.31957
H	1.51517	-0.59588	5.81800
H	3.13951	-1.16608	5.24880
H	2.24204	1.66619	5.95317
H	3.76646	0.80298	6.42719
C	6.27445	-2.21313	1.21407
C	7.40754	-1.91993	2.20857
C	5.51487	-3.43199	1.76431
H	6.73595	-2.50540	0.24291
C	7.48035	-3.12225	3.13493
H	7.20003	-1.01049	2.81557
H	8.37492	-1.77153	1.68074
C	6.08083	-3.70538	3.14878
H	5.71343	-4.31575	1.12007
H	4.41376	-3.28022	1.80969
H	8.19086	-3.87119	2.71988
H	7.80788	-2.83138	4.15657
H	6.09890	-4.79377	3.37399
H	5.46696	-3.18457	3.91627

Catalyst (2,4,6-c-Pent, 10)-Enamine protonation TS (pro-R) alternative conformation
Electronic energy (Hartrees): -1870.446599
Free Energy at 1M concentration and 253 K (Hartrees): -1868.982923
Coordinates:

P	-0.01166	0.25493	0.16039
O	1.23710	1.04670	-0.51791
O	-1.22750	1.33110	0.32440
O	0.27097	-0.31577	1.51130
O	-0.38811	-0.75292	-0.97340
C	1.53877	2.22701	0.11469
C	2.81555	2.34199	0.86006
C	3.13150	3.49228	1.47192
H	4.06500	3.58045	2.01592
C	2.19711	4.63575	1.43974
C	2.52158	5.86911	2.19280
H	3.45746	5.94682	2.73212
C	1.65882	6.89061	2.21351
H	1.89588	7.79074	2.76582
C	0.37419	6.78847	1.49541
H	-0.32978	7.60965	1.53601
C	0.07353	5.68081	0.80766
H	-0.88481	5.61886	0.30982
C	1.03657	4.54748	0.74788
C	0.72659	3.30004	-0.01459
C	-1.37123	2.22303	-0.71030
C	-2.61357	2.18330	-1.51991
C	-2.82843	3.11258	-2.46275
H	-3.73620	3.08649	-3.05476
C	-1.80301	4.13379	-2.75247
C	-2.01512	5.08923	-3.86422
H	-2.94053	5.07286	-4.42649
C	-1.06339	5.97301	-4.18190
H	-1.21837	6.66931	-4.99592
C	0.20806	5.99075	-3.43446
H	0.98462	6.69120	-3.71359
C	0.40768	5.13534	-2.42518
H	1.35896	5.14953	-1.91074
C	-0.65526	4.16786	-2.03593
C	-0.46322	3.20839	-0.90526
C	-3.60122	1.09820	-1.31142
C	-4.45768	1.10196	-0.07935
C	-3.74153	0.10094	-2.22591
C	-5.34799	0.10658	0.10336
C	-4.80524	-0.91653	-2.02888
C	-5.54707	-0.95654	-0.91172
H	-5.95717	0.10691	0.99605
H	-4.97193	-1.64549	-2.81121
C	3.74332	1.19002	0.92168
C	4.48557	0.83423	-0.16212
C	3.87658	0.41886	2.20452
C	5.42559	-0.30971	-0.04885
C	4.77494	-0.58255	2.27550
C	5.58586	-0.97228	1.10580
H	6.01064	-0.59588	-0.91378
H	4.89961	-1.13405	3.19899
H	-0.66542	-1.69626	1.96668
C	-2.42634	-2.09222	2.94503
C	-1.63549	-3.18243	0.91535

C	-2.17384	-1.16076	3.96966
C	-3.67161	-2.76178	2.87572
C	-2.89928	-3.86450	0.84158
C	-3.15809	-0.90738	4.90975
H	-1.22687	-0.63314	3.98785
C	-4.65032	-2.48622	3.85288
C	-3.85972	-3.67150	1.78773
H	-3.05068	-4.55270	0.02232
C	-4.40016	-1.56951	4.85796
H	-2.96959	-0.17981	5.69312
H	-5.60573	-3.00048	3.79669
H	-4.79531	-4.22008	1.72634
H	-5.15897	-1.35488	5.60315
C	-0.60672	-3.25452	-0.06662
H	-0.59852	-1.77501	-0.63403
C	0.86515	-3.36970	0.35635
H	1.08150	-4.41763	0.58775
H	1.00457	-2.79741	1.28155
N	1.79813	-2.92178	-0.65821
N	-1.46697	-2.35046	1.98898
C	2.74606	-3.71946	-1.28431
C	3.27857	-4.88484	-0.70039
C	3.24171	-3.32603	-2.54484
C	4.25738	-5.62395	-1.36413
H	2.95469	-5.20228	0.28476
C	4.23211	-4.06059	-3.18522
H	2.82846	-2.44066	-3.02129
C	4.74955	-5.22209	-2.60534
H	4.65203	-6.51743	-0.88752
H	4.59487	-3.72649	-4.15369
H	5.52125	-5.79639	-3.10779
H	1.48884	-2.12453	-1.19638
Cl	-0.95303	-4.34887	-1.45961
C	-2.84147	-0.03204	-3.45616
C	-2.19996	-1.42625	-3.57064
C	-3.61720	0.20156	-4.75834
H	-2.00335	0.69617	-3.43349
C	-2.68874	-2.02291	-4.88195
H	-2.45213	-2.09472	-2.71930
H	-1.09179	-1.32916	-3.60634
C	-3.10963	-0.84269	-5.73703
H	-3.44400	1.22790	-5.14982
H	-4.71177	0.06188	-4.61730
H	-1.89352	-2.61913	-5.37967
H	-3.56885	-2.67591	-4.69081
H	-2.22546	-0.44447	-6.28261
H	-3.89467	-1.13157	-6.46899
C	-4.32483	2.19159	0.98577
C	-5.63806	2.95124	1.23390
C	-3.88129	1.61919	2.34070
H	-3.57382	2.95039	0.68626
C	-5.77741	3.06660	2.74348
H	-6.52065	2.41189	0.82679
H	-5.61073	3.95994	0.76618
C	-5.01749	1.88431	3.31497
H	-2.96538	2.14568	2.69062
H	-3.65374	0.53338	2.29251
H	-5.30575	4.01270	3.09041
H	-6.84371	3.05161	3.05701
H	-4.63514	2.10274	4.33555

H	-5.68701	0.99690	3.35909
C	-6.65700	-1.99635	-0.77465
C	-6.22434	-3.40867	-1.17682
C	-7.20402	-2.16896	0.64059
H	-7.49747	-1.69000	-1.43794
C	-7.11442	-4.37233	-0.39694
H	-5.16216	-3.58228	-0.90228
H	-6.34082	-3.56589	-2.27141
C	-7.83978	-3.54861	0.66257
H	-7.94672	-1.38021	0.89045
H	-6.37658	-2.15152	1.38281
H	-7.85150	-4.85383	-1.07648
H	-6.49871	-5.16665	0.07884
H	-8.91794	-3.46559	0.40132
H	-7.74629	-4.01290	1.66863
C	4.41882	1.57425	-1.49944
C	5.79659	2.07768	-1.96331
C	3.88149	0.67333	-2.61923
H	3.75715	2.46401	-1.44869
C	6.09165	1.37894	-3.28293
H	6.60658	1.88195	-1.22675
H	5.75538	3.17673	-2.13381
C	4.74272	0.95992	-3.83590
H	2.81184	0.88875	-2.83334
H	3.97493	-0.40281	-2.35921
H	6.62611	2.05064	-3.98909
H	6.71358	0.47535	-3.09750
H	4.30094	1.80004	-4.41638
H	4.83247	0.06624	-4.49095
C	3.02306	0.74940	3.42915
C	2.27280	-0.47128	3.99205
C	3.86542	1.31629	4.58074
H	2.25135	1.50599	3.17644
C	2.80915	-0.69637	5.39702
H	2.38849	-1.38640	3.37070
H	1.18714	-0.24176	4.05839
C	3.35113	0.64792	5.84449
H	3.76035	2.42138	4.64781
H	4.94497	1.07615	4.45983
H	2.01391	-1.06026	6.08325
H	3.63556	-1.44026	5.36825
H	2.52623	1.25527	6.27887
H	4.15745	0.52851	6.60021
C	6.58175	-2.11234	1.22838
C	6.23738	-3.28945	0.30667
C	8.01762	-1.68530	0.88111
H	6.58236	-2.48970	2.27612
C	7.55178	-3.74460	-0.30203
H	5.55304	-2.98189	-0.51301
H	5.75769	-4.11631	0.87428
C	8.42921	-2.50767	-0.33111
H	8.69343	-1.92531	1.73187
H	8.11167	-0.59779	0.66661
H	8.01768	-4.51692	0.34926
H	7.40598	-4.16241	-1.32177
H	9.50729	-2.77580	-0.29518
H	8.22588	-1.93066	-1.26047

Catalyst (2,4,6-c-Pent, 10)-Enamine protonation TS (pro-R) alternative conformation

Electronic energy (Hartrees): -1870.446864
Free Energy at 1M concentration and 253 K (Hartrees): -1868.982617
Coordinates:

P	0.02310	0.28411	0.00251
O	1.17338	1.21566	-0.66962
O	-1.14475	1.29576	0.53055
O	0.48153	-0.52769	1.17113
O	-0.52233	-0.49499	-1.23612
C	1.54850	2.27729	0.11622
C	2.90190	2.28322	0.72291
C	3.31424	3.34510	1.42980
H	4.30274	3.35125	1.87469
C	2.39972	4.47921	1.67247
C	2.83488	5.59775	2.54032
H	3.83749	5.61043	2.94950
C	1.99053	6.59429	2.82666
H	2.30876	7.41007	3.46285
C	0.61655	6.57658	2.29011
H	-0.06999	7.37291	2.54724
C	0.21479	5.57350	1.50101
H	-0.80558	5.56831	1.14223
C	1.15306	4.47441	1.14350
C	0.73323	3.34679	0.25580
C	-1.43842	2.34142	-0.31094
C	-2.78381	2.40138	-0.93358
C	-3.13637	3.47033	-1.66306
H	-4.11926	3.51759	-2.11792
C	-2.16514	4.55060	-1.92337
C	-2.53790	5.66923	-2.81983
H	-3.53680	5.72437	-3.23451
C	-1.64163	6.61322	-3.12533
H	-1.91425	7.42904	-3.78230
C	-0.27259	6.53604	-2.58194
H	0.45608	7.28840	-2.85505
C	0.07275	5.53242	-1.76725
H	1.09061	5.48214	-1.40507
C	-0.92369	4.49307	-1.38738
C	-0.56912	3.36849	-0.46754
C	-3.72160	1.26216	-0.78934
C	-4.38355	1.00673	0.53317
C	-3.98959	0.44595	-1.84446
C	-5.22718	-0.03784	0.65311
C	-5.00598	-0.62561	-1.69036
C	-5.57008	-0.89958	-0.50448
H	-5.69442	-0.22640	1.60911
H	-5.28458	-1.20007	-2.56412
C	3.77611	1.09721	0.57662
C	4.34635	0.79199	-0.62076
C	4.00090	0.20154	1.76088
C	5.14349	-0.45852	-0.74367
C	4.79770	-0.87343	1.62400
C	5.38675	-1.23980	0.31836
H	5.56434	-0.71882	-1.70670
H	4.98054	-1.51175	2.47598
H	-0.35209	-2.00507	1.43297
C	-1.94562	-2.68184	2.53984
C	-1.42563	-3.31002	0.24726
C	-1.57299	-1.95618	3.68682
C	-3.16981	-3.39167	2.50687
C	-2.66879	-4.02974	0.20728

C	-2.41875	-1.94247	4.78224
H	-0.64689	-1.39368	3.68016
C	-4.00494	-3.36537	3.64355
C	-3.48890	-4.07816	1.29423
H	-2.91952	-4.54887	-0.70659
C	-3.63787	-2.64844	4.76744
H	-2.13907	-1.37152	5.66218
H	-4.94443	-3.91035	3.61585
H	-4.41001	-4.65223	1.25002
H	-4.28752	-2.62400	5.63608
C	-0.54544	-3.13148	-0.86201
H	-0.64352	-1.58363	-1.09447
C	0.96797	-3.27966	-0.67879
H	1.22412	-4.34712	-0.68822
H	1.23891	-2.88745	0.31076
N	1.71566	-2.61537	-1.72792
N	-1.12684	-2.69470	1.43062
C	2.79691	-3.16451	-2.39556
C	3.57433	-4.20467	-1.85177
C	3.16225	-2.65787	-3.66058
C	4.67326	-4.70528	-2.54950
H	3.33391	-4.61031	-0.87537
C	4.27224	-3.15158	-4.33399
H	2.55567	-1.87843	-4.11487
C	5.04236	-4.18207	-3.78739
H	5.25440	-5.50985	-2.10672
H	4.53029	-2.73356	-5.30344
H	5.90662	-4.56929	-4.31742
H	1.23853	-1.85864	-2.19486
Cl	-1.07486	-3.94605	-2.38738
C	-3.27973	0.57040	-3.19441
C	-2.65740	-0.75814	-3.65926
C	-4.23909	1.01678	-4.30443
H	-2.44928	1.30662	-3.15658
C	-3.32837	-1.11463	-4.97733
H	-2.78287	-1.58064	-2.92255
H	-1.56604	-0.62336	-3.83120
C	-3.87464	0.19030	-5.52503
H	-4.13065	2.10317	-4.51560
H	-5.30035	0.81712	-4.03732
H	-2.61091	-1.58149	-5.68647
H	-4.16829	-1.81982	-4.79077
H	-3.08116	0.71142	-6.10537
H	-4.75657	0.01810	-6.17929
C	-4.10094	1.88384	1.75404
C	-5.36981	2.52547	2.33949
C	-3.44216	1.08789	2.89094
H	-3.41737	2.71717	1.49373
C	-5.28588	2.32863	3.84517
H	-6.29856	2.04703	1.95992
H	-5.42067	3.60830	2.09062
C	-4.42504	1.09615	4.05000
H	-2.49965	1.58967	3.20468
H	-3.19306	0.04657	2.59602
H	-4.78816	3.20890	4.30919
H	-6.29334	2.19718	4.29605
H	-3.89888	1.12682	5.02863
H	-5.06161	0.18480	4.00757
C	-6.64184	-1.98294	-0.40482
C	-6.27652	-3.27482	-1.14002

C	-6.95398	-2.44622	1.01638
H	-7.57821	-1.58163	-0.85457
C	-7.04054	-4.39733	-0.44423
H	-5.18525	-3.46862	-1.06491
H	-6.55684	-3.21731	-2.21448
C	-7.57122	-3.82692	0.86756
H	-7.65069	-1.74806	1.52955
H	-6.01791	-2.54115	1.60821
H	-7.88628	-4.73954	-1.08029
H	-6.36808	-5.26191	-0.25297
H	-8.67886	-3.73647	0.81779
H	-7.30041	-4.47680	1.72828
C	4.20699	1.68770	-1.85495
C	5.56680	2.12102	-2.42678
C	3.45638	0.97933	-2.98983
H	3.65199	2.62141	-1.62545
C	5.48420	1.90038	-3.92904
H	6.40838	1.52270	-2.01553
H	5.77167	3.19086	-2.20181
C	4.45308	0.80534	-4.12406
H	2.61485	1.61892	-3.33844
H	3.03180	0.00299	-2.67648
H	5.13272	2.83252	-4.42457
H	6.46923	1.60830	-4.35335
H	3.95856	0.88563	-5.11622
H	4.94649	-0.18750	-4.04220
C	3.35060	0.47837	3.11709
C	2.54930	-0.71998	3.65557
C	4.38846	0.83216	4.19248
H	2.63902	1.32627	3.04905
C	3.23848	-1.15808	4.93816
H	2.47925	-1.56470	2.93527
H	1.51490	-0.39224	3.89832
C	3.97852	0.06737	5.44002
H	4.40417	1.92676	4.38856
H	5.41422	0.51800	3.89750
H	2.50545	-1.52382	5.68959
H	3.96751	-1.96814	4.71471
H	3.29035	0.69219	6.05162
H	4.86120	-0.21587	6.05341
C	6.28681	-2.46046	0.18337
C	7.64124	-2.22763	0.85026
C	5.70291	-3.70788	0.85802
H	6.45582	-2.68412	-0.89517
C	7.55128	-2.84840	2.23438
H	7.91260	-1.14999	0.90679
H	8.43028	-2.75523	0.26922
C	6.47776	-3.92778	2.15698
H	5.85025	-4.58992	0.19753
H	4.61244	-3.61161	1.05575
H	8.53011	-3.27655	2.54222
H	7.25720	-2.07226	2.97434
H	6.95147	-4.93425	2.15145
H	5.79876	-3.86254	3.03532

Catalyst (2,4,6-c-Pent, 10)-Enamine protonation TS (pro-R) alternative conformation
Electronic energy (Hartrees): -1870.443963
Free Energy at 1M concentration and 253 K (Hartrees): -1868.980613
Coordinates:

P	0.00809	0.18728	0.03356
O	1.14472	1.07369	-0.71837
O	-1.22689	1.19623	0.38698
O	0.44431	-0.44358	1.31585
O	-0.44630	-0.77044	-1.11219
C	1.42370	2.26111	-0.09025
C	2.74411	2.44045	0.55946
C	3.05006	3.60769	1.14287
H	4.01461	3.74122	1.61921
C	2.06064	4.70397	1.18052
C	2.38244	5.95636	1.90269
H	3.35467	6.08491	2.36205
C	1.47278	6.93211	1.99460
H	1.70816	7.84605	2.52451
C	0.13955	6.75991	1.38725
H	-0.60057	7.54338	1.48716
C	-0.15979	5.63485	0.72780
H	-1.15281	5.52110	0.31455
C	0.85245	4.55237	0.58747
C	0.54514	3.28632	-0.14569
C	-1.55244	2.09101	-0.60264
C	-2.89877	2.01245	-1.22043
C	-3.26574	2.91636	-2.14098
H	-4.25335	2.86978	-2.58561
C	-2.31974	3.96176	-2.57992
C	-2.70659	4.89229	-3.66534
H	-3.69189	4.82949	-4.11031
C	-1.83980	5.81160	-4.10331
H	-2.12263	6.48989	-4.89792
C	-0.48989	5.89537	-3.51417
H	0.21458	6.62666	-3.88901
C	-0.13233	5.06311	-2.52937
H	0.87068	5.12725	-2.12926
C	-1.09582	4.05471	-2.00930
C	-0.72410	3.11764	-0.90632
C	-3.85247	0.96280	-0.78900
C	-4.48586	1.05903	0.57176
C	-4.16598	-0.07961	-1.60796
C	-5.36589	0.11207	0.95178
C	-5.20380	-1.04934	-1.17378
C	-5.78160	-0.96414	0.03282
H	-5.80748	0.14674	1.93992
H	-5.51107	-1.83055	-1.85556
C	3.71643	1.32439	0.56951
C	4.38452	0.97163	-0.56153
C	3.94036	0.55558	1.83905
C	5.33014	-0.17431	-0.51419
C	4.85064	-0.43629	1.84987
C	5.61122	-0.80785	0.63718
H	5.80134	-0.48101	-1.43606
H	5.02491	-0.99335	2.76105
H	-0.35842	-1.90741	1.70244
C	-2.00660	-2.55117	2.74914
C	-1.21895	-3.44534	0.62785
C	-1.81639	-1.62305	3.78990
C	-3.15117	-3.38417	2.72887
C	-2.38452	-4.28524	0.59531
C	-2.76573	-1.53043	4.79300
H	-0.94811	-0.97365	3.77060
C	-4.09367	-3.27261	3.77253

C	-3.28507	-4.27449	1.61740
H	-2.50448	-4.94902	-0.24899
C	-3.90757	-2.35549	4.79088
H	-2.62921	-0.80403	5.58820
H	-4.97175	-3.91192	3.75685
H	-4.12982	-4.95741	1.59462
H	-4.63995	-2.26708	5.58652
C	-0.25387	-3.33548	-0.41627
H	-0.46409	-1.83751	-0.85209
C	1.23917	-3.33088	-0.08205
H	1.57251	-4.36693	0.06617
H	1.38075	-2.80189	0.86890
N	2.04555	-2.73115	-1.13177
N	-1.08371	-2.64851	1.73017
C	3.20396	-3.31156	-1.62737
C	4.00387	-4.16990	-0.84899
C	3.62290	-3.02565	-2.94293
C	5.15893	-4.73863	-1.38503
H	3.73906	-4.37383	0.18296
C	4.78812	-3.58165	-3.45641
H	3.01165	-2.37713	-3.56557
C	5.56397	-4.45465	-2.68844
H	5.75801	-5.39753	-0.76195
H	5.08410	-3.34150	-4.47414
H	6.47054	-4.89229	-3.09402
H	1.53977	-2.14651	-1.78106
Cl	-0.58122	-4.36600	-1.86538
C	-3.52230	-0.28558	-2.98169
C	-2.89121	-1.68104	-3.13320
C	-4.55091	-0.14342	-4.11257
H	-2.71320	0.45140	-3.16806
C	-3.43073	-2.26642	-4.42985
H	-3.14433	-2.35525	-2.28585
H	-1.78350	-1.61146	-3.18773
C	-4.72224	-1.52589	-4.72060
H	-4.15535	0.54865	-4.88919
H	-5.52836	0.25756	-3.76361
H	-2.70571	-2.08017	-5.25303
H	-3.60534	-3.36055	-4.33941
H	-4.91513	-1.46614	-5.81369
H	-5.57277	-2.05046	-4.23315
C	-4.13475	2.18644	1.54482
C	-5.36305	2.98631	2.01038
C	-3.44460	1.65697	2.81072
H	-3.44701	2.91739	1.07453
C	-5.21945	3.14057	3.51647
H	-6.31813	2.46405	1.78612
H	-5.39975	3.98345	1.51892
C	-4.37754	1.96272	3.97061
H	-2.48038	2.19131	2.96417
H	-3.22723	0.56927	2.75786
H	-4.68339	4.08845	3.74480
H	-6.20941	3.14836	4.02190
H	-3.81034	2.20274	4.89589
H	-5.03476	1.08604	4.16318
C	-6.89429	-1.91358	0.44394
C	-8.10676	-1.83414	-0.49806
C	-6.44557	-3.38154	0.46137
H	-7.24113	-1.65043	1.46898
C	-8.21591	-3.19250	-1.17234

H	-8.02009	-1.02514	-1.25685
H	-9.02842	-1.64793	0.09703
C	-7.54833	-4.17027	-0.22473
H	-6.30265	-3.73992	1.50347
H	-5.49578	-3.53124	-0.09685
H	-9.27564	-3.47037	-1.36007
H	-7.66493	-3.17609	-2.13876
H	-8.28661	-4.51659	0.53221
H	-7.14190	-5.04941	-0.77028
C	4.19900	1.70024	-1.89603
C	5.52642	2.20562	-2.48601
C	3.56576	0.78572	-2.95299
H	3.54217	2.58895	-1.79222
C	5.54668	1.75817	-3.94023
H	6.41128	1.78648	-1.96011
H	5.59313	3.31407	-2.42182
C	4.62647	0.55425	-4.01593
H	2.69265	1.29830	-3.41531
H	3.20830	-0.17677	-2.52957
H	5.14576	2.57078	-4.58585
H	6.57555	1.50066	-4.27301
H	4.17352	0.45393	-5.02597
H	5.19892	-0.36998	-3.78231
C	3.16587	0.87638	3.11819
C	2.46479	-0.35092	3.72844
C	4.07792	1.44944	4.21219
H	2.37275	1.62670	2.91983
C	3.09003	-0.56822	5.09751
H	2.55221	-1.26629	3.10285
H	1.38245	-0.13237	3.86113
C	3.64910	0.78104	5.50725
H	3.97244	2.55419	4.28399
H	5.14859	1.21352	4.02272
H	2.34227	-0.93623	5.83312
H	3.91870	-1.30602	5.01838
H	2.84872	1.38363	5.99126
H	4.50226	0.66940	6.21095
C	6.61077	-1.95773	0.71767
C	7.58613	-2.06983	-0.45381
C	7.53080	-1.86250	1.93585
H	6.03623	-2.90709	0.79396
C	8.74020	-2.90289	0.07441
H	7.97517	-1.06468	-0.73331
H	7.11729	-2.54606	-1.33936
C	8.79602	-2.63924	1.57640
H	7.04814	-2.29427	2.83964
H	7.79672	-0.80020	2.13743
H	8.53525	-3.98060	-0.11184
H	9.69459	-2.62362	-0.42268
H	8.83145	-3.60191	2.13221
H	9.69923	-2.04389	1.83429

Catalyst (2,4,6-c-Pent, 10)-Enamine protonation TS (pro-R) alternative conformation
Electronic energy (Hartrees): -1870.443412
Free Energy at 1M concentration and 253 K (Hartrees): -1868.980314
Coordinates:

P	-0.08064	0.24653	0.09356
O	1.08785	1.16829	-0.56075
O	-1.31972	1.24584	0.45912

O	0.30815	-0.45576	1.35437
O	-0.49811	-0.64428	-1.11757
C	1.39687	2.26648	0.20159
C	2.71519	2.32776	0.87708
C	3.04023	3.40124	1.61139
H	4.00359	3.45041	2.10594
C	2.07531	4.50696	1.78368
C	2.41488	5.64500	2.66859
H	3.38157	5.68619	3.15501
C	1.52801	6.62457	2.87424
H	1.77632	7.45475	3.52279
C	0.20233	6.56978	2.22917
H	-0.51924	7.35400	2.41868
C	-0.11282	5.54890	1.42368
H	-1.09946	5.51726	0.98129
C	0.87428	4.46697	1.15898
C	0.54849	3.31685	0.26223
C	-1.55392	2.25382	-0.44400
C	-2.83674	2.25979	-1.18826
C	-3.14007	3.28959	-1.99177
H	-4.07760	3.29646	-2.53601
C	-2.17279	4.38507	-2.19908
C	-2.48495	5.46329	-3.16546
H	-3.44027	5.47703	-3.67535
C	-1.58723	6.42183	-3.41640
H	-1.81542	7.20807	-4.12439
C	-0.27583	6.40121	-2.74168
H	0.45655	7.16498	-2.96943
C	0.01423	5.43464	-1.86313
H	0.99311	5.42573	-1.40341
C	-0.98743	4.37957	-1.54523
C	-0.69414	3.29404	-0.55919
C	-3.76618	1.11138	-1.07151
C	-4.55350	0.92132	0.19155
C	-3.90951	0.22565	-2.09408
C	-5.38363	-0.13567	0.29408
C	-4.91079	-0.86422	-1.97166
C	-5.58577	-1.07941	-0.83238
H	-5.94254	-0.27515	1.20853
H	-5.08493	-1.50228	-2.82819
C	3.66890	1.20315	0.73642
C	4.31666	0.97937	-0.43931
C	3.90481	0.28792	1.90277
C	5.25894	-0.16614	-0.53851
C	4.81592	-0.69652	1.78678
C	5.55871	-0.92481	0.52919
H	5.71473	-0.36568	-1.49670
H	5.00088	-1.35794	2.62312
H	-0.53117	-1.90510	1.70573
C	-2.22184	-2.51660	2.70449
C	-1.46263	-3.32554	0.53867
C	-1.97350	-1.69537	3.82060
C	-3.43164	-3.24510	2.60452
C	-2.69001	-4.06445	0.42991
C	-2.92754	-1.60772	4.81962
H	-1.05536	-1.12032	3.86478
C	-4.37995	-3.13933	3.64358
C	-3.61903	-4.03325	1.42629
H	-2.84071	-4.66179	-0.45793
C	-4.13438	-2.33005	4.73751

H	-2.74395	-0.96463	5.67472
H	-5.30830	-3.69812	3.56430
H	-4.52757	-4.62168	1.33537
H	-4.87013	-2.24537	5.53040
C	-0.46902	-3.22495	-0.48046
H	-0.58716	-1.71958	-0.87950
C	1.01423	-3.31879	-0.11305
H	1.28854	-4.37812	-0.02276
H	1.15740	-2.85951	0.87328
N	1.87861	-2.70004	-1.10169
N	-1.29353	-2.60846	1.69001
C	3.02631	-3.29454	-1.60167
C	3.74325	-4.27051	-0.88261
C	3.51997	-2.90519	-2.86422
C	4.88461	-4.85562	-1.43069
H	3.42488	-4.55739	0.11378
C	4.66990	-3.48217	-3.38836
H	2.97532	-2.16202	-3.44072
C	5.35889	-4.47457	-2.68466
H	5.41704	-5.60796	-0.85468
H	5.02343	-3.16221	-4.36493
H	6.25411	-4.92713	-3.09898
H	1.43632	-2.02021	-1.70264
Cl	-0.81593	-4.17450	-1.97941
C	-3.07348	0.28986	-3.37400
C	-2.37442	-1.04441	-3.69093
C	-3.93085	0.63670	-4.59758
H	-2.27087	1.05407	-3.30312
C	-2.91363	-1.50811	-5.03574
H	-2.54004	-1.82191	-2.91371
H	-1.27660	-0.88252	-3.77682
C	-3.43585	-0.25886	-5.71974
H	-3.82388	1.70925	-4.87115
H	-5.00887	0.42829	-4.41866
H	-2.12398	-2.00112	-5.64320
H	-3.75126	-2.22270	-4.87692
H	-2.60374	0.24491	-6.25993
H	-4.24896	-0.49973	-6.43834
C	-4.41445	1.88117	1.37424
C	-5.74660	2.53447	1.77638
C	-3.87142	1.17537	2.62607
H	-3.71721	2.70928	1.13383
C	-5.80789	2.46265	3.29381
H	-6.62361	2.00025	1.35080
H	-5.79301	3.59142	1.43333
C	-4.96357	1.26263	3.67975
H	-2.96285	1.70351	2.99231
H	-3.59713	0.11648	2.43473
H	-5.36224	3.38448	3.72924
H	-6.85349	2.35442	3.65518
H	-4.53636	1.37821	4.69939
H	-5.58772	0.34220	3.64850
C	-6.63080	-2.19100	-0.76829
C	-6.14693	-3.51848	-1.35712
C	-7.08938	-2.56095	0.64050
H	-7.52119	-1.85879	-1.34888
C	-6.94999	-4.61560	-0.66428
H	-5.06545	-3.66406	-1.14865
H	-6.30692	-3.55064	-2.45702
C	-7.64996	-3.96870	0.52710

H	-7.85714	-1.85172	1.01961
H	-6.22404	-2.58282	1.33797
H	-7.70292	-5.04229	-1.36283
H	-6.27592	-5.43289	-0.32665
H	-8.74523	-3.91925	0.33859
H	-7.46983	-4.54380	1.46161
C	4.11478	1.85518	-1.67982
C	5.43585	2.41523	-2.23411
C	3.45507	1.06877	-2.82100
H	3.46869	2.73146	-1.46485
C	5.42997	2.12819	-3.72860
H	6.32676	1.93856	-1.77123
H	5.51042	3.51003	-2.05174
C	4.49594	0.94792	-3.92114
H	2.58009	1.63735	-3.20847
H	3.09440	0.06720	-2.50236
H	5.02851	3.00996	-4.27576
H	6.45114	1.89927	-4.10353
H	4.02546	0.96491	-4.92805
H	5.06165	-0.00218	-3.80178
C	3.13609	0.44621	3.21518
C	2.44201	-0.85033	3.67101
C	4.05236	0.88179	4.36767
H	2.33961	1.21237	3.11387
C	3.08340	-1.24057	4.99332
H	2.52100	-1.67736	2.93163
H	1.36172	-0.65246	3.84423
C	3.64116	0.04728	5.56902
H	3.93725	1.96684	4.58278
H	5.12358	0.68385	4.14114
H	2.34535	-1.70523	5.68267
H	3.91409	-1.95739	4.81039
H	2.84364	0.57686	6.13615
H	4.50275	-0.15010	6.24302
C	6.56317	-2.07101	0.46903
C	7.49329	-2.07911	-0.74369
C	7.52822	-2.07654	1.65525
H	5.99373	-3.02589	0.48570
C	8.66305	-2.95522	-0.33270
H	7.87688	-1.05383	-0.94734
H	6.99003	-2.47499	-1.64967
C	8.77875	-2.81632	1.18277
H	7.07993	-2.58472	2.53678
H	7.80142	-1.03423	1.93604
H	8.44506	-4.01348	-0.59886
H	9.59867	-2.63768	-0.84250
H	8.83721	-3.82145	1.65532
H	9.69112	-2.24046	1.45252

Catalyst (2,4,6-c-Pent, 10)-Product (R) lowest free energy conformation
Electronic energy (Hartrees): -1870.48665
Free Energy at 1M concentration and 253 K (Hartrees): -1869.019359
Coordinates:

P	0.36758	0.21974	-0.05455
O	-0.78645	1.17526	0.58785
O	1.56564	1.20617	-0.54715
O	-0.17556	-0.28028	-1.44701
O	0.74633	-0.82381	0.92703
C	-1.03388	2.34820	-0.08334
C	-2.38075	2.56179	-0.67093

C	-2.67779	3.73359	-1.25155
H	-3.66181	3.89587	-1.67629
C	-1.65398	4.78932	-1.37322
C	-1.96740	6.03123	-2.11710
H	-2.95849	6.18537	-2.52550
C	-1.02671	6.96520	-2.29248
H	-1.25577	7.87127	-2.83844
C	0.33159	6.75600	-1.75676
H	1.09574	7.50389	-1.92507
C	0.62319	5.64011	-1.07850
H	1.63350	5.49668	-0.71983
C	-0.42227	4.60622	-0.84389
C	-0.12576	3.35436	-0.08240
C	1.94170	2.12689	0.39857
C	3.27456	2.00229	1.03589
C	3.71053	2.95706	1.87007
H	4.69409	2.87915	2.31896
C	2.82483	4.07954	2.24168
C	3.27639	5.06589	3.24993
H	4.27112	4.99547	3.67222
C	2.45673	6.04510	3.64661
H	2.78704	6.76424	4.38496
C	1.09318	6.13856	3.09150
H	0.42588	6.91777	3.43663
C	0.67685	5.25597	2.17612
H	-0.33624	5.32832	1.80415
C	1.58879	4.18152	1.69679
C	1.15536	3.19309	0.66162
C	4.11918	0.83069	0.71369
C	4.69318	0.71333	-0.66840
C	4.26760	-0.19390	1.59806
C	5.25060	-0.45236	-1.04878
C	4.96177	-1.42923	1.15586
C	5.39392	-1.58001	-0.10492
C	-3.39586	1.48049	-0.63914
C	-4.03060	1.14899	0.51937
C	-3.78277	0.79889	-1.92478
C	-5.11681	0.13686	0.48345
C	-4.81039	-0.07285	-1.91672
C	-5.50446	-0.43492	-0.66527
H	-0.19900	-1.30663	-1.54553
C	1.19286	-3.21212	-2.32877
C	-0.81764	-3.89623	-1.36844
C	2.02973	-2.16026	-2.78667
C	1.63670	-4.56308	-2.47967
C	-0.43581	-5.25949	-1.47079
C	3.24229	-2.44753	-3.37249
H	1.69112	-1.13803	-2.67504
C	2.89278	-4.82253	-3.08507
C	0.77916	-5.58804	-2.01520
H	-1.11671	-6.02643	-1.11884
C	3.67942	-3.78435	-3.52688
H	3.87435	-1.64003	-3.72704
H	3.21919	-5.85313	-3.19228
H	1.08951	-6.62588	-2.10074
H	4.64110	-3.98531	-3.98843
C	-2.20214	-3.59321	-0.82961
H	-2.57097	-4.48115	-0.31513
C	-2.34571	-2.38658	0.10372
H	-3.40477	-2.27283	0.34293

H	-2.04721	-1.47446	-0.41773
N	-1.56870	-2.57775	1.30975
N	-0.02480	-2.91178	-1.76954
C	-2.16927	-2.78042	2.54989
C	-3.41472	-3.41951	2.69112
C	-1.50874	-2.34051	3.71452
C	-3.99201	-3.57389	3.95115
H	-3.92890	-3.81115	1.81938
C	-2.08934	-2.51274	4.96562
H	-0.55164	-1.83775	3.61555
C	-3.34091	-3.12187	5.09822
H	-4.95983	-4.06197	4.03089
H	-1.56432	-2.15197	5.84612
H	-3.79521	-3.24450	6.07631
H	-0.70272	-2.04315	1.33423
Cl	-3.34435	-3.39179	-2.26575
H	-5.14624	-0.53051	-2.83787
H	-5.62487	-0.11809	1.40501
H	5.64081	-0.56236	-2.05296
H	5.08294	-2.23276	1.86522
C	-3.05801	1.10753	-3.23668
C	-2.54108	-0.12141	-3.98351
C	-3.93822	1.85125	-4.24230
H	-2.16483	1.74043	-3.05010
C	-2.40794	0.33545	-5.42520
H	-3.25742	-0.96679	-3.94750
H	-1.56445	-0.46559	-3.58589
C	-3.47531	1.40684	-5.62987
H	-3.82483	2.95078	-4.12089
H	-5.01261	1.59438	-4.11361
H	-1.39927	0.77882	-5.58057
H	-2.54313	-0.51320	-6.13054
H	-3.05751	2.26543	-6.19972
H	-4.33261	0.98457	-6.19900
C	-3.70841	1.79567	1.86813
C	-4.94144	2.44462	2.52040
C	-3.17200	0.76823	2.87473
H	-2.94329	2.59341	1.77435
C	-5.16563	1.72106	3.84117
H	-5.85293	2.39138	1.88540
H	-4.73535	3.51931	2.72337
C	-3.83003	1.09867	4.20136
H	-2.06490	0.82167	2.96029
H	-3.44976	-0.26853	2.58866
H	-5.51236	2.41850	4.63416
H	-5.92365	0.91839	3.70446
H	-3.20926	1.84012	4.75150
H	-3.96090	0.18985	4.82827
C	-6.64525	-1.43685	-0.70434
C	-6.38733	-2.67587	0.15525
C	-7.98088	-0.84846	-0.22325
H	-6.79126	-1.78927	-1.75090
C	-7.75956	-3.12893	0.61239
H	-5.78190	-2.43219	1.05354
H	-5.87141	-3.47343	-0.42199
C	-8.57023	-1.85239	0.76429
H	-8.66902	-0.71819	-1.08751
H	-7.86222	0.14152	0.26976
H	-8.21914	-3.77339	-0.16949
H	-7.70391	-3.69263	1.56895

H	-9.64752	-2.03704	0.56174
H	-8.45720	-1.46542	1.80138
C	4.66576	1.89370	-1.64130
C	6.06835	2.31294	-2.11063
C	3.84637	1.59634	-2.90584
H	4.21615	2.78735	-1.16027
C	5.95352	2.55612	-3.60636
H	6.82736	1.52131	-1.92941
H	6.40624	3.23530	-1.58907
C	4.81587	1.66861	-4.07470
H	3.05951	2.37319	-3.03305
H	3.34368	0.60820	-2.87302
H	5.69339	3.62181	-3.79246
H	6.90105	2.31172	-4.13363
H	4.32769	2.08224	-4.98356
H	5.20654	0.65169	-4.29993
C	3.73429	-0.15178	3.03301
C	2.72225	-1.27189	3.32036
C	4.86428	-0.30686	4.06357
H	3.21878	0.80392	3.25522
C	3.12320	-1.88062	4.65379
H	2.73481	-2.06220	2.53828
H	1.69076	-0.86373	3.38306
C	4.60960	-1.61614	4.79424
H	4.82120	0.53214	4.79355
H	5.87756	-0.30561	3.60423
H	2.57640	-1.36925	5.47695
H	2.90087	-2.96924	4.68761
H	4.90847	-1.54998	5.86280
H	5.18161	-2.43640	4.30726
C	6.05227	-2.86931	-0.57469
C	7.32965	-3.18914	0.20285
C	5.14479	-4.08862	-0.40257
H	6.30857	-2.77002	-1.65371
C	6.96918	-4.25073	1.23704
H	7.76911	-2.28852	0.68633
H	8.08778	-3.60737	-0.49614
C	5.59874	-4.80933	0.86137
H	5.28347	-4.77058	-1.27029
H	4.06794	-3.81329	-0.34713
H	7.73298	-5.05891	1.24570
H	6.93011	-3.79621	2.25135
H	5.65679	-5.90557	0.68368
H	4.87316	-4.62822	1.68450

Catalyst (2,4,6-c-Pent, 10)-Product (R) alternative conformation
Electronic energy (Hartrees): -1870.485444
Free Energy at 1M concentration and 253 K (Hartrees): -1869.018922
Coordinates:

P	0.05171	-0.11120	0.18465
O	-1.01151	1.03504	0.63932
O	1.43692	0.63559	-0.26447
O	-0.42842	-0.65558	-1.21609
O	0.16048	-1.09492	1.28886
C	-0.97193	2.22448	-0.04262
C	-2.19727	2.67823	-0.74508
C	-2.21851	3.87344	-1.35225
H	-3.11554	4.21032	-1.85915
C	-1.01237	4.72578	-1.36106
C	-1.01840	5.99885	-2.11785

H	-1.91492	6.32135	-2.63264
C	0.08446	6.75327	-2.17304
H	0.07995	7.68203	-2.72877
C	1.31538	6.31561	-1.48753
H	2.21102	6.91961	-1.55541
C	1.32953	5.17102	-0.79488
H	2.25059	4.85871	-0.32101
C	0.10229	4.33370	-0.70032
C	0.09267	3.05033	0.06481
C	1.90575	1.51661	0.67598
C	3.21792	1.26349	1.32019
C	3.65004	2.08001	2.29261
H	4.62313	1.92217	2.74372
C	2.83657	3.24005	2.71046
C	3.29193	4.09282	3.83237
H	4.21893	3.86800	4.34507
C	2.55943	5.14332	4.21804
H	2.89198	5.76523	5.03908
C	1.29050	5.45295	3.53145
H	0.69314	6.29314	3.86124
C	0.87373	4.69290	2.51212
H	-0.06967	4.92557	2.03618
C	1.69044	3.53653	2.05459
C	1.24355	2.66739	0.92613
C	4.14132	0.24858	0.76262
C	4.95870	0.60956	-0.44393
C	4.33351	-0.94937	1.37877
C	5.92129	-0.23439	-0.85709
C	5.44781	-1.82374	0.91908
C	6.20817	-1.49039	-0.13353
C	-3.40594	1.82120	-0.74090
C	-4.13849	1.65757	0.39583
C	-3.80739	1.10315	-1.99816
C	-5.30512	0.73567	0.37748
C	-4.91591	0.33841	-1.98693
C	-5.70071	0.13275	-0.75107
H	-0.46537	-1.68636	-1.23123
C	1.04020	-3.64003	-1.03654
C	-1.18775	-4.13382	-0.56764
C	2.00228	-2.73648	-1.56032
C	1.47831	-4.90040	-0.52377
C	-0.83177	-5.40764	-0.05737
C	3.33556	-3.07418	-1.56046
H	1.67301	-1.78275	-1.95463
C	2.86252	-5.21106	-0.52999
C	0.48925	-5.78048	-0.02555
H	-1.60841	-6.06676	0.31396
C	3.77379	-4.31441	-1.03624
H	4.05885	-2.37575	-1.96722
H	3.18692	-6.16647	-0.12738
H	0.78537	-6.74535	0.37707
H	4.83127	-4.55508	-1.03073
C	-2.65170	-3.73847	-0.58304
H	-3.22715	-4.51800	-0.08280
C	-2.97195	-2.38483	0.07359
H	-4.04668	-2.21601	0.00885
H	-2.49892	-1.57603	-0.48727
N	-2.53546	-2.38882	1.45024
N	-0.28219	-3.28504	-1.03178
C	-3.39063	-2.38291	2.54472

C	-4.76509	-2.67320	2.45662
C	-2.85644	-2.07423	3.81431
C	-5.57148	-2.62633	3.59386
H	-5.21373	-2.93845	1.50575
C	-3.67173	-2.03648	4.93828
H	-1.79798	-1.84066	3.89421
C	-5.04038	-2.30725	4.84224
H	-6.63126	-2.84580	3.49376
H	-3.23374	-1.78321	5.90009
H	-5.67537	-2.27085	5.72161
H	-1.60733	-2.00249	1.60665
Cl	-3.25752	-3.75444	-2.32082
H	-5.23638	-0.16430	-2.89043
H	-5.86470	0.57963	1.29062
H	6.51648	0.00934	-1.72800
H	5.66953	-2.73047	1.46670
C	-2.99644	1.22601	-3.28964
C	-2.48077	-0.13064	-3.79858
C	-3.81740	1.84333	-4.43251
H	-2.10508	1.86757	-3.13753
C	-2.81255	-0.18780	-5.28139
H	-2.96696	-0.98396	-3.28004
H	-1.38311	-0.22221	-3.64962
C	-3.98522	0.75372	-5.47932
H	-3.25404	2.69310	-4.87877
H	-4.80750	2.22697	-4.10075
H	-1.94280	0.17566	-5.87247
H	-3.06617	-1.22160	-5.60188
H	-3.99482	1.17620	-6.50740
H	-4.93692	0.20598	-5.30268
C	-3.81207	2.38305	1.70557
C	-5.00724	3.17803	2.25826
C	-3.39164	1.39945	2.80455
H	-2.98336	3.11028	1.57740
C	-5.13919	2.79163	3.72451
H	-5.95517	2.94214	1.72835
H	-4.83439	4.27262	2.16192
C	-4.48916	1.42703	3.85399
H	-2.43707	1.73850	3.26567
H	-3.23635	0.37147	2.41847
H	-4.58768	3.52454	4.35424
H	-6.20341	2.76284	4.04416
H	-4.07855	1.26846	4.87467
H	-5.23690	0.63311	3.63734
C	-6.95158	-0.72898	-0.76518
C	-8.05163	-0.12609	-1.63885
C	-6.70883	-2.14921	-1.29891
H	-7.36420	-0.82548	0.26606
C	-8.82243	-1.31410	-2.18039
H	-7.62729	0.44619	-2.49344
H	-8.71292	0.54650	-1.05022
C	-7.79302	-2.42010	-2.33792
H	-6.78805	-2.88511	-0.46895
H	-5.70916	-2.26767	-1.76562
H	-9.59484	-1.62502	-1.44234
H	-9.31344	-1.07371	-3.14823
H	-8.25557	-3.42019	-2.19082
H	-7.35242	-2.37117	-3.35853
C	4.71077	1.90096	-1.22438
C	5.97465	2.76361	-1.38811

C	4.17608	1.62762	-2.63595
H	3.96027	2.53843	-0.71562
C	6.20258	2.91258	-2.88590
H	6.87193	2.32732	-0.89711
H	5.80263	3.76874	-0.94207
C	4.85816	2.64189	-3.53595
H	3.07030	1.74127	-2.67609
H	4.43518	0.60365	-2.98545
H	6.57785	3.92690	-3.14245
H	6.93927	2.15235	-3.22839
H	4.26051	3.58023	-3.56058
H	4.97816	2.25370	-4.57054
C	3.47022	-1.40982	2.55147
C	2.88008	-2.80598	2.36890
C	4.23972	-1.47805	3.86660
H	2.60724	-0.72667	2.71036
C	2.56235	-3.27508	3.77849
H	3.60785	-3.51178	1.91866
H	1.97169	-2.79114	1.73352
C	3.54366	-2.55251	4.70188
H	4.22283	-0.49368	4.38375
H	5.29908	-1.77251	3.69997
H	1.51974	-2.98583	4.03827
H	2.66274	-4.37903	3.86410
H	2.99771	-2.08897	5.55262
H	4.29233	-3.26843	5.10651
C	7.37706	-2.36566	-0.55498
C	7.24838	-2.85923	-2.00488
C	8.71388	-1.61323	-0.46418
H	7.44245	-3.26063	0.10576
C	8.60379	-2.63116	-2.65392
H	6.47672	-2.29457	-2.57187
H	6.98135	-3.93756	-2.03857
C	9.23449	-1.48549	-1.88676
H	9.43643	-2.21055	0.13531
H	8.62116	-0.61131	0.01079
H	9.23084	-3.54321	-2.53999
H	8.50325	-2.38777	-3.73389
H	10.34425	-1.53855	-1.92054
H	8.90169	-0.51755	-2.32246

Catalyst (2,4,6-c-Pent, 10)-Product (R) alternative conformation
Electronic energy (Hartrees): -1870.485138
Free Energy at 1M concentration and 253 K (Hartrees): -1869.018895
Coordinates:

P	0.01370	-0.10695	0.16641
O	-1.06710	1.03075	0.60224
O	1.41675	0.65287	-0.19693
O	-0.40929	-0.61328	-1.26203
O	0.08299	-1.11304	1.25401
C	-0.96200	2.25028	-0.01664
C	-2.10941	2.73625	-0.82027
C	-2.08409	3.96710	-1.35020
H	-2.92271	4.32352	-1.93733
C	-0.89349	4.82500	-1.18473
C	-0.84303	6.14509	-1.85455
H	-1.69415	6.49982	-2.42246
C	0.25522	6.90269	-1.76546
H	0.29207	7.86600	-2.25756
C	1.42641	6.42120	-1.00894

H	2.32150	7.02804	-0.96245
C	1.38903	5.23351	-0.39416
H	2.27051	4.89123	0.13095
C	0.16249	4.39131	-0.45649
C	0.09346	3.06150	0.22337
C	1.85572	1.46508	0.81702
C	3.13438	1.15003	1.49921
C	3.53011	1.89160	2.54427
H	4.48097	1.69131	3.02494
C	2.70793	3.02916	3.00679
C	3.11887	3.79533	4.20558
H	4.01931	3.52370	4.74237
C	2.37876	4.82493	4.63124
H	2.67882	5.38339	5.50847
C	1.14567	5.19799	3.91143
H	0.54193	6.02077	4.27208
C	0.76922	4.51665	2.82306
H	-0.14813	4.79440	2.32093
C	1.59515	3.38509	2.32293
C	1.19265	2.60366	1.11683
C	4.07127	0.16650	0.90799
C	4.95781	0.61170	-0.21937
C	4.21010	-1.08399	1.42751
C	5.92134	-0.21743	-0.65934
C	5.32312	-1.94526	0.94104
C	6.13755	-1.54341	-0.04470
C	-3.27714	1.85286	-1.04323
C	-4.18022	1.62852	-0.04988
C	-3.44347	1.17177	-2.37321
C	-5.32206	0.71238	-0.30433
C	-4.50331	0.36155	-2.56371
C	-5.49060	0.11644	-1.49373
H	-0.47381	-1.64606	-1.31375
C	0.96629	-3.63858	-1.23669
C	-1.28787	-4.08584	-0.84239
C	1.97178	-2.72542	-1.65173
C	1.34748	-4.95841	-0.84172
C	-0.98905	-5.41855	-0.46257
C	3.29237	-3.11071	-1.65525
H	1.68602	-1.72629	-1.95734
C	2.71984	-5.31853	-0.84984
C	0.31618	-5.84478	-0.45150
H	-1.79588	-6.08013	-0.16814
C	3.67448	-4.41107	-1.24527
H	4.05018	-2.40330	-1.97448
H	3.00008	-6.32033	-0.53678
H	0.56782	-6.85623	-0.14420
H	4.72295	-4.68936	-1.24099
C	-2.73194	-3.62337	-0.79925
H	-3.34093	-4.43555	-0.40091
C	-2.97914	-2.35397	0.03084
H	-4.04929	-2.14675	0.03201
H	-2.50134	-1.50015	-0.45592
N	-2.49014	-2.53217	1.38324
N	-0.34223	-3.23470	-1.21331
C	-3.33219	-2.49439	2.49287
C	-4.67794	-2.90097	2.44098
C	-2.81877	-2.03955	3.72445
C	-5.48672	-2.81424	3.57361
H	-5.09365	-3.29800	1.52043

C	-3.63301	-1.97068	4.84893
H	-1.78296	-1.71421	3.77244
C	-4.97791	-2.34715	4.78488
H	-6.52617	-3.12373	3.50318
H	-3.21561	-1.60267	5.78243
H	-5.61315	-2.28032	5.66238
H	-1.57274	-2.11656	1.53515
Cl	-3.35912	-3.36444	-2.50786
H	-4.64747	-0.12546	-3.51935
H	-6.04298	0.54314	0.48486
H	6.56739	0.08768	-1.47312
H	5.49662	-2.90354	1.41282
C	-2.45669	1.39958	-3.52167
C	-1.82072	0.09705	-4.03762
C	-3.12772	2.07052	-4.72949
H	-1.62258	2.05529	-3.19977
C	-1.98298	0.10324	-5.55051
H	-2.31045	-0.80584	-3.61514
H	-0.74131	0.05146	-3.77523
C	-3.14215	1.03679	-5.84329
H	-2.52078	2.94524	-5.05386
H	-4.15739	2.43096	-4.51158
H	-1.05864	0.50350	-6.02313
H	-2.17959	-0.91834	-5.94227
H	-3.03388	1.51431	-6.84121
H	-4.09673	0.46712	-5.81600
C	-4.06512	2.26239	1.33867
C	-5.31300	3.07266	1.72389
C	-3.87664	1.19732	2.42827
H	-3.20305	2.95830	1.40596
C	-5.66474	2.65935	3.14467
H	-6.17670	2.86107	1.05706
H	-5.10920	4.16499	1.67578
C	-5.10993	1.25750	3.31400
H	-2.97537	1.43569	3.03609
H	-3.74508	0.17811	2.00954
H	-5.16634	3.34407	3.86623
H	-6.76277	2.68257	3.31597
H	-4.85779	1.04696	4.37593
H	-5.85930	0.51330	2.96471
C	-6.69096	-0.77170	-1.77437
C	-6.79203	-1.97522	-0.83844
C	-8.03022	-0.02979	-1.65893
H	-6.61893	-1.17140	-2.81177
C	-8.27161	-2.29959	-0.78253
H	-6.44875	-1.72542	0.18769
H	-6.20510	-2.83896	-1.21927
C	-8.98281	-0.96089	-0.91054
H	-8.42915	0.19421	-2.67279
H	-7.93769	0.93193	-1.10793
H	-8.54241	-2.95086	-1.64302
H	-8.53867	-2.81416	0.16605
H	-9.94659	-1.07421	-1.45288
H	-9.18564	-0.55166	0.10399
C	4.78784	1.97851	-0.88390
C	6.02937	2.86788	-0.73601
C	4.49352	1.87384	-2.38996
H	3.94679	2.54196	-0.43345
C	6.16735	3.59773	-2.05985
H	6.95084	2.27273	-0.55450

H	5.91016	3.58838	0.10283
C	5.59229	2.65317	-3.09942
H	3.50736	2.33968	-2.61244
H	4.46176	0.82441	-2.75748
H	5.56609	4.53372	-2.03433
H	7.22805	3.84672	-2.27990
H	5.19589	3.21071	-3.97551
H	6.38568	1.95162	-3.44052
C	3.28809	-1.62029	2.52063
C	2.68155	-2.98504	2.20106
C	3.99839	-1.81215	3.85647
H	2.43281	-0.93295	2.69991
C	2.29053	-3.55630	3.55336
H	3.41542	-3.66974	1.72897
H	1.80452	-2.90243	1.52879
C	3.24105	-2.92947	4.57400
H	3.98328	-0.87134	4.44923
H	5.05632	-2.12213	3.70984
H	1.24227	-3.26600	3.78830
H	2.36714	-4.66546	3.55550
H	2.66418	-2.51801	5.43111
H	3.95440	-3.69162	4.95741
C	7.29138	-2.41938	-0.50330
C	7.19452	-2.78154	-1.99337
C	8.64942	-1.72718	-0.30647
H	7.30497	-3.37027	0.07767
C	8.58659	-2.58054	-2.56825
H	6.48191	-2.12401	-2.53743
H	6.86681	-3.83466	-2.12990
C	9.23633	-1.52515	-1.69466
H	9.32296	-2.39004	0.28105
H	8.57213	-0.75588	0.23073
H	9.16228	-3.52977	-2.49389
H	8.54650	-2.25759	-3.63115
H	10.34286	-1.63047	-1.68852
H	8.96745	-0.51351	-2.07139

Catalyst (2,4,6-c-Pent, 10)-Product (R) alternative conformation
Electronic energy (Hartrees): -1870.485651
Free Energy at 1M concentration and 253 K (Hartrees): -1869.018544
Coordinates:

P	0.34934	0.17294	-0.11727
O	-0.79804	1.15837	0.49333
O	1.49833	1.12974	-0.76264
O	-0.24497	-0.47601	-1.42969
O	0.80175	-0.76819	0.93404
C	-1.12760	2.25058	-0.27371
C	-2.53041	2.39572	-0.73953
C	-2.89380	3.47898	-1.44175
H	-3.91994	3.59504	-1.77127
C	-1.89822	4.51062	-1.79202
C	-2.29155	5.64748	-2.65587
H	-3.31337	5.73910	-3.00267
C	-1.38588	6.56380	-3.01429
H	-1.67358	7.39337	-3.64711
C	0.01204	6.43952	-2.55932
H	0.74510	7.17197	-2.87182
C	0.37611	5.41914	-1.77411
H	1.41160	5.33462	-1.47227
C	-0.62683	4.40836	-1.34065

C	-0.24760	3.26342	-0.45876
C	1.88163	2.16253	0.05587
C	3.24165	2.15084	0.64688
C	3.67553	3.20501	1.35260
H	4.67705	3.20683	1.76728
C	2.76858	4.33693	1.63079
C	3.22409	5.44265	2.50461
H	4.23494	5.44785	2.89311
C	2.38797	6.43664	2.82214
H	2.72094	7.24281	3.46304
C	1.00322	6.42888	2.31395
H	0.32359	7.22234	2.59703
C	0.58323	5.43802	1.51894
H	-0.44480	5.43971	1.18299
C	1.51155	4.34214	1.12664
C	1.07233	3.22913	0.22930
C	4.11690	0.97973	0.42263
C	4.66451	0.74055	-0.95407
C	4.33409	0.06526	1.40747
C	5.27389	-0.43077	-1.22108
C	5.08605	-1.17281	1.08424
C	5.50499	-1.43583	-0.16257
C	-3.54230	1.36986	-0.39006
C	-3.99457	1.24428	0.88895
C	-4.11477	0.49926	-1.47416
C	-5.02352	0.21546	1.19377
C	-5.11853	-0.34532	-1.16846
C	-5.59100	-0.51164	0.22301
H	-0.20561	-1.50215	-1.42284
C	1.30990	-3.42593	-1.96785
C	-0.61261	-4.10166	-0.84029
C	2.03543	-2.40677	-2.63936
C	1.87243	-4.73872	-1.89430
C	-0.10619	-5.42098	-0.70532
C	3.25341	-2.69088	-3.21593
H	1.60739	-1.41368	-2.69822
C	3.13047	-4.99519	-2.49622
C	1.12505	-5.73350	-1.22098
H	-0.70798	-6.16856	-0.20076
C	3.80737	-3.99077	-3.14769
H	3.79872	-1.91186	-3.73807
H	3.54668	-5.99668	-2.43247
H	1.53025	-6.73752	-1.12887
H	4.77071	-4.18921	-3.60688
C	-2.02406	-3.83910	-0.35181
H	-2.31420	-4.64429	0.32365
C	-2.28816	-2.50029	0.34473
H	-3.35761	-2.42392	0.54337
H	-2.04153	-1.67058	-0.32187
N	-1.53076	-2.43579	1.57446
N	0.08028	-3.13633	-1.42950
C	-2.10982	-2.41572	2.83917
C	-3.43225	-2.82278	3.09344
C	-1.33129	-1.96702	3.92729
C	-3.96042	-2.74533	4.38231
H	-4.05472	-3.20322	2.29180
C	-1.86922	-1.90034	5.20615
H	-0.31112	-1.64507	3.74214
C	-3.19244	-2.28214	5.44909
H	-4.98906	-3.05536	4.54674

H	-1.24961	-1.53546	6.02107
H	-3.61194	-2.22116	6.44819
H	-0.64185	-1.94536	1.50048
Cl	-3.14965	-4.03348	-1.80417
H	-5.59050	-0.93627	-1.94231
H	-5.34946	0.08800	2.21834
H	5.64776	-0.63068	-2.21747
H	5.26459	-1.88665	1.87303
C	-3.60106	0.57887	-2.91199
C	-3.15952	-0.76232	-3.49325
C	-4.64132	1.12745	-3.89157
H	-2.71079	1.23911	-2.97552
C	-3.26319	-0.57943	-4.99599
H	-3.83563	-1.58803	-3.19239
H	-2.12253	-1.01958	-3.19163
C	-4.40553	0.40653	-5.21929
H	-4.51761	2.22611	-4.01208
H	-5.67941	0.93090	-3.54443
H	-2.31412	-0.14533	-5.38183
H	-3.45481	-1.54749	-5.50792
H	-4.14387	1.12966	-6.02235
H	-5.32464	-0.14349	-5.51936
C	-3.51091	2.13720	2.03641
C	-4.66097	2.88220	2.73342
C	-2.79073	1.32530	3.12099
H	-2.80430	2.91711	1.68598
C	-4.42503	2.72166	4.22708
H	-5.65650	2.46199	2.47402
H	-4.66623	3.95882	2.45405
C	-3.63399	1.43646	4.38015
H	-1.78471	1.76130	3.31224
H	-2.65261	0.26387	2.83429
H	-3.81943	3.57600	4.60298
H	-5.38307	2.67583	4.78885
H	-3.00019	1.45629	5.29307
H	-4.33227	0.57283	4.44131
C	-6.72261	-1.47480	0.54192
C	-8.01436	-1.11039	-0.20719
C	-6.38746	-2.92296	0.15951
H	-6.94533	-1.45632	1.63382
C	-8.32467	-2.28018	-1.12852
H	-7.93107	-0.16625	-0.78989
H	-8.84600	-0.98917	0.52211
C	-7.63079	-3.47912	-0.51209
H	-6.12568	-3.52619	1.05524
H	-5.54140	-2.97465	-0.55698
H	-9.41962	-2.44891	-1.21870
H	-7.90010	-2.08406	-2.13798
H	-8.29435	-3.94017	0.25281
H	-7.37340	-4.23863	-1.28191
C	4.55626	1.80689	-2.04626
C	5.92490	2.23105	-2.60473
C	3.71531	1.33991	-3.24266
H	4.08004	2.72742	-1.64906
C	5.76885	2.27061	-4.11640
H	6.72972	1.51111	-2.34103
H	6.22267	3.23011	-2.21711
C	4.65530	1.29129	-4.43613
H	2.90780	2.07926	-3.44307
H	3.23632	0.35386	-3.07531

H	5.46569	3.29303	-4.43375
H	6.71338	1.99134	-4.63163
H	4.13230	1.56625	-5.37755
H	5.07838	0.26773	-4.53844
C	3.83058	0.23941	2.84334
C	2.88735	-0.89263	3.27959
C	4.98939	0.25828	3.85299
H	3.26869	1.18632	2.97263
C	3.35611	-1.33699	4.65536
H	2.91866	-1.75917	2.58343
H	1.83820	-0.53095	3.33435
C	4.82778	-0.97825	4.72336
H	4.91225	1.16609	4.49219
H	5.99075	0.26810	3.36832
H	2.80104	-0.77420	5.43847
H	3.19801	-2.42684	4.80681
H	5.14748	-0.78303	5.76989
H	5.43452	-1.81293	4.30834
C	6.24997	-2.71937	-0.50034
C	7.59398	-2.81471	0.22137
C	5.47460	-3.97724	-0.10127
H	6.43413	-2.74994	-1.59804
C	7.38350	-3.71751	1.43034
H	7.98617	-1.81859	0.52449
H	8.33934	-3.28715	-0.45654
C	6.09002	-4.49367	1.19594
H	5.60341	-4.75058	-0.89029
H	4.38516	-3.78422	0.01787
H	8.24387	-4.41080	1.55503
H	7.29549	-3.10095	2.35171
H	6.29918	-5.58308	1.11654
H	5.38841	-4.33395	2.04397

Catalyst (2,4,6-c-Hex, 11)-THF complex
Electronic energy (Hartrees): -876.394046
Free Energy at 1M concentration and 253 K (Hartrees): -874.900124
Coordinates:

P	-0.11665	-0.73269	-0.42608
O	1.03423	0.05088	0.47279
O	-1.38921	0.27305	-0.66020
O	0.37796	-1.24430	-1.72079
O	-0.62849	-1.77334	0.64704
C	1.28885	1.29662	-0.03199
C	2.65587	1.62713	-0.52280
C	2.91336	2.85632	-1.00952
H	3.91428	3.11180	-1.34666
C	1.84758	3.88747	-1.04110
C	2.11477	5.21221	-1.65874
H	3.09094	5.42957	-2.08166
C	1.14840	6.14359	-1.70082
H	1.34291	7.10963	-2.15546
C	-0.18909	5.85358	-1.13356
H	-0.97097	6.60445	-1.18635
C	-0.43691	4.66345	-0.56328
H	-1.42806	4.46349	-0.16893
C	0.63317	3.62562	-0.49466
C	0.37523	2.28380	0.11155
C	-1.69603	1.01300	0.45210
C	-3.04753	0.87749	1.06390
C	-3.37255	1.61119	2.14516

H	-4.36894	1.54184	2.57329
C	-2.39351	2.55791	2.72953
C	-2.72836	3.29351	3.97656
H	-3.69298	3.14779	4.45334
C	-1.83746	4.13724	4.52139
H	-2.08105	4.67150	5.43411
C	-0.51546	4.33519	3.88373
H	0.20793	5.00328	4.34012
C	-0.20816	3.68399	2.75033
H	0.77344	3.82826	2.31119
C	-1.19721	2.75901	2.12144
C	-0.87114	2.00342	0.87159
C	-4.09329	0.06971	0.38469
C	-4.76851	0.64023	-0.83775
C	-4.48114	-1.14401	0.87667
C	-5.76286	-0.06243	-1.42336
C	-5.62725	-1.84891	0.23425
C	-6.23145	-1.35252	-0.86268
H	-6.25307	0.32300	-2.31143
H	-5.98329	-2.77767	0.66439
C	3.76846	0.65784	-0.34341
C	4.31322	0.44729	0.89350
C	4.34278	-0.04285	-1.54726
C	5.47798	-0.47451	1.02318
C	5.43707	-0.82021	-1.39180
C	6.03164	-1.06174	-0.05286
H	5.89687	-0.65977	2.00684
H	5.88485	-1.30639	-2.25073
H	-0.10255	-2.63429	0.64484
O	0.91029	-3.85785	0.72587
C	1.77568	-3.66662	1.87712
C	1.72016	-3.97200	-0.47479
C	3.21188	-3.56869	1.33466
H	1.45357	-2.74765	2.37699
H	1.63313	-4.50696	2.56408
C	2.99709	-3.20933	-0.14412
H	1.91318	-5.03503	-0.66957
H	1.15091	-3.53613	-1.29703
H	3.72198	-4.53364	1.42379
H	3.80457	-2.82688	1.87543
H	3.83383	-3.48988	-0.78843
H	2.81597	-2.13790	-0.25997
C	3.79979	1.14659	2.15770
C	3.24409	0.12560	3.16944
C	4.89390	2.01576	2.80829
H	2.96890	1.84002	1.92046
C	2.69902	0.83815	4.41395
H	4.03459	-0.59153	3.47948
H	2.42218	-0.45509	2.70011
C	4.34940	2.72641	4.05549
H	5.76954	1.39781	3.10148
H	5.24132	2.78033	2.07955
C	3.77800	1.71704	5.06068
H	2.35381	0.07979	5.14910
H	1.82361	1.46521	4.13342
H	5.16929	3.30053	4.53810
H	3.55574	3.44772	3.75934
H	3.33632	2.26597	5.91993
H	4.59800	1.07420	5.44989
C	3.70720	0.10507	-2.93006

C	4.62959	0.88708	-3.88481
C	3.32783	-1.26072	-3.54355
H	2.76159	0.67981	-2.84450
C	3.96387	1.06931	-5.25574
H	5.59579	0.35261	-4.01704
H	4.84785	1.88867	-3.45490
C	2.65371	-1.07087	-4.90789
H	4.22552	-1.90047	-3.68061
H	2.63318	-1.79825	-2.86595
C	3.56196	-0.28259	-5.86124
H	4.67169	1.58227	-5.94177
H	3.06304	1.71341	-5.15064
H	2.43331	-2.06621	-5.35014
H	1.68863	-0.53309	-4.77824
H	3.02565	-0.11004	-6.81900
H	4.47530	-0.87746	-6.08289
C	7.24087	-1.97097	0.10440
C	6.90851	-3.41257	-0.32874
C	8.44766	-1.42473	-0.68484
H	7.53816	-2.00913	1.17781
C	8.13194	-4.32626	-0.18193
H	6.56519	-3.43537	-1.38593
H	6.08926	-3.80992	0.30798
C	9.66457	-2.34595	-0.53216
H	8.20141	-1.33282	-1.76494
H	8.71107	-0.41288	-0.30666
C	9.32820	-3.77713	-0.96896
H	7.87930	-5.34056	-0.55912
H	8.40272	-4.41903	0.89292
H	10.49847	-1.95868	-1.15629
H	10.00271	-2.34774	0.52749
H	10.21086	-4.42987	-0.79692
H	9.09800	-3.79162	-2.05712
C	-3.78963	-1.80761	2.06988
C	-4.70375	-1.80398	3.30851
C	-3.34170	-3.24976	1.74592
H	-2.86862	-1.24849	2.33992
C	-3.98499	-2.42926	4.51312
H	-5.63785	-2.37163	3.10301
H	-4.98614	-0.75914	3.56134
C	-2.60196	-3.86162	2.94188
H	-4.21358	-3.89532	1.50876
H	-2.67639	-3.25315	0.85805
C	-3.49050	-3.84767	4.19383
H	-4.68391	-2.47045	5.37600
H	-3.12144	-1.79125	4.80402
H	-2.32036	-4.90968	2.70289
H	-1.66593	-3.29302	3.13744
H	-2.91123	-4.23707	5.05841
H	-4.36377	-4.51860	4.03756
C	-7.39004	-2.07845	-1.52867
C	-6.96044	-3.46626	-2.04692
C	-8.60031	-2.19636	-0.57978
H	-7.71882	-1.48370	-2.41090
C	-8.13336	-4.18151	-2.72976
H	-6.59089	-4.09961	-1.21117
H	-6.13262	-3.34911	-2.77990
C	-9.76646	-2.91659	-1.26893
H	-8.32484	-2.75747	0.33959
H	-8.93304	-1.18037	-0.27433

C	-9.33509	-4.29601	-1.78316
H	-7.81226	-5.19938	-3.03918
H	-8.43036	-3.62376	-3.64513
H	-10.60066	-3.03969	-0.54516
H	-10.13809	-2.30077	-2.11736
H	-10.18380	-4.76724	-2.32390
H	-9.07182	-4.94987	-0.92268
C	-4.35065	1.99279	-1.42358
C	-3.76258	1.83770	-2.84015
C	-5.51426	3.00461	-1.42274
H	-3.55599	2.44789	-0.80104
C	-3.30146	3.19551	-3.38636
H	-4.51731	1.40594	-3.53279
H	-2.89173	1.14779	-2.80937
C	-5.05115	4.36102	-1.97265
H	-6.35999	2.63593	-2.04228
H	-5.88389	3.14322	-0.38328
C	-4.45146	4.21162	-3.37752
H	-2.93369	3.06668	-4.42696
H	-2.45765	3.57976	-2.77135
H	-5.91808	5.05479	-2.01586
H	-4.29321	4.80355	-1.28888
H	-4.07082	5.19789	-3.71980
H	-5.24282	3.88479	-4.08749

Catalyst (2,4,6-c-Hex, 11)-Quinoline complex
Electronic energy (Hartrees): -1582.882763
Free Energy at 1M concentration and 253 K (Hartrees): -1581.347211
Coordinates:

P	0.32084	-0.05462	-1.03854
O	-0.87224	0.25367	0.07447
O	1.62188	0.86401	-0.67255
O	-0.07814	0.16902	-2.44108
O	0.71840	-1.50911	-0.57966
C	-1.05174	1.58229	0.36172
C	-2.37749	2.20710	0.13352
C	-2.54831	3.51641	0.37237
H	-3.52100	3.97363	0.23163
C	-1.43205	4.33039	0.89701
C	-1.60609	5.78830	1.08959
H	-2.54939	6.26044	0.84481
C	-0.59709	6.52849	1.56294
H	-0.72374	7.59456	1.70036
C	0.69460	5.89543	1.89408
H	1.51190	6.50267	2.26132
C	0.85834	4.57725	1.73425
H	1.81816	4.13465	1.96739
C	-0.25964	3.73969	1.22332
C	-0.09373	2.27381	1.01094
C	1.92160	0.92651	0.66664
C	3.22873	0.41415	1.14619
C	3.50552	0.41405	2.45907
H	4.47048	0.07058	2.81315
C	2.51483	0.92081	3.43003
C	2.78118	0.80974	4.88234
H	3.70892	0.37532	5.23346
C	1.87280	1.23689	5.76648
H	2.06549	1.15002	6.82795
C	0.60107	1.82100	5.29808
H	-0.13778	2.14730	6.01869

C	0.35725	1.93930	3.98805
H	-0.58985	2.35270	3.66724
C	1.36470	1.48261	2.99261
C	1.09598	1.55762	1.52746
C	4.28498	0.05488	0.17396
C	5.03097	1.15885	-0.52317
C	4.61720	-1.24467	-0.05810
C	6.03193	0.83822	-1.36449
C	5.77394	-1.54284	-0.94180
C	6.44118	-0.56568	-1.57549
H	6.57160	1.61594	-1.88894
H	6.08613	-2.56943	-1.07084
C	-3.55265	1.36562	-0.18580
C	-4.20548	0.69075	0.80149
C	-4.08712	1.34499	-1.58691
C	-5.47728	-0.00635	0.47823
C	-5.26527	0.73853	-1.82652
C	-6.00698	0.04145	-0.75212
H	-6.00174	-0.53465	1.26369
H	-5.68341	0.74100	-2.82330
H	-0.05182	-2.09223	-0.24450
C	-2.26948	-3.27351	-0.68472
C	-1.35125	-3.62909	1.43319
C	-2.21080	-2.61899	-1.94445
C	-3.34417	-4.17996	-0.41914
C	-2.38625	-4.54228	1.76911
C	-3.18047	-2.86658	-2.89014
H	-1.40851	-1.92430	-2.16411
C	-4.31821	-4.41771	-1.42150
C	-3.36969	-4.80678	0.84985
H	-2.39111	-5.00743	2.74642
C	-4.23498	-3.77564	-2.63578
H	-3.13171	-2.35782	-3.84761
H	-5.12803	-5.11123	-1.21260
H	-4.17554	-5.49596	1.08758
H	-4.98409	-3.95714	-3.40038
N	-1.29639	-3.03119	0.24750
C	-0.31237	-3.26186	2.43178
C	0.23204	-2.05180	2.56374
H	0.99021	-1.85224	3.31128
H	-0.09175	-1.24317	1.92027
Cl	0.17772	-4.56149	3.52769
C	4.67450	2.62958	-0.29896
C	5.86178	3.43152	0.27175
C	4.14267	3.28440	-1.58930
H	3.86674	2.71596	0.45334
C	5.45507	4.88954	0.52782
H	6.72350	3.41769	-0.42979
H	6.19103	2.97443	1.23038
C	3.73908	4.74204	-1.33041
H	4.91469	3.25863	-2.38878
H	3.25400	2.72436	-1.95279
C	4.91191	5.54368	-0.74996
H	6.33948	5.46002	0.88437
H	4.67977	4.92798	1.32488
H	3.41335	5.20766	-2.28529
H	2.87922	4.77298	-0.62527
H	4.56998	6.57482	-0.51630
H	5.72448	5.61536	-1.50607
C	3.85176	-2.41334	0.56175

C	3.38237	-3.42456	-0.50732
C	4.70397	-3.12561	1.62740
H	2.93296	-2.04863	1.06778
C	2.57782	-4.55835	0.13991
H	4.24672	-3.87310	-1.04086
H	2.75266	-2.91192	-1.26435
C	3.90989	-4.26581	2.27939
H	5.63139	-3.53736	1.17216
H	5.00139	-2.39966	2.41465
C	3.40049	-5.26220	1.22811
H	2.29130	-5.29711	-0.63906
H	1.64362	-4.15056	0.57992
H	4.56161	-4.79913	3.00424
H	3.04906	-3.84355	2.84213
H	2.76821	-6.02829	1.72623
H	4.26523	-5.78302	0.76123
C	7.61321	-0.87046	-2.49152
C	8.77198	-1.53670	-1.72144
C	7.17941	-1.73841	-3.69073
H	7.99984	0.08970	-2.90245
C	9.95477	-1.82363	-2.65544
H	8.43896	-2.49196	-1.26041
H	9.11163	-0.86244	-0.90519
C	8.36882	-2.02480	-4.61644
H	6.75490	-2.70504	-3.34201
H	6.39191	-1.20695	-4.26836
C	9.51913	-2.68637	-3.84675
H	10.74899	-2.35557	-2.08874
H	10.38177	-0.86511	-3.02436
H	8.04114	-2.69911	-5.43671
H	8.72327	-1.07559	-5.07530
H	10.38308	-2.82945	-4.53078
H	9.19902	-3.68837	-3.48507
C	-3.69729	0.63760	2.24390
C	-4.70207	1.26309	3.23021
C	-3.36420	-0.80834	2.65182
H	-2.75934	1.21622	2.35904
C	-4.15216	1.21361	4.66330
H	-5.67442	0.72630	3.19979
H	-4.88500	2.32323	2.94931
C	-2.81146	-0.85671	4.08154
H	-4.26459	-1.45691	2.58867
H	-2.60349	-1.21198	1.95463
C	-3.79834	-0.22365	5.07145
H	-4.91549	1.61614	5.36329
H	-3.24680	1.85584	4.73940
H	-2.62861	-1.91476	4.36819
H	-1.83905	-0.31820	4.12627
H	-3.34421	-0.21512	6.08555
H	-4.72524	-0.83676	5.11620
C	-3.32082	1.99769	-2.73365
C	-3.03202	1.00102	-3.87716
C	-4.06779	3.23429	-3.26911
H	-2.33208	2.34663	-2.36851
C	-2.22567	1.67744	-4.99254
H	-3.97508	0.60812	-4.31337
H	-2.45820	0.13682	-3.48369
C	-3.26956	3.90662	-4.39457
H	-5.07029	2.94691	-3.65523
H	-4.21394	3.96582	-2.44463

C	-2.96099	2.91487	-5.52462
H	-2.07204	0.95596	-5.82362
H	-1.22507	1.97283	-4.60680
H	-3.85690	4.75680	-4.80321
H	-2.31850	4.31387	-3.98590
H	-2.32911	3.41577	-6.28916
H	-3.90913	2.60277	-6.01537
C	-7.34632	-0.61536	-1.03741
C	-8.37170	0.41603	-1.55108
C	-7.18693	-1.77712	-2.03563
H	-7.75699	-1.04539	-0.09466
C	-9.71938	-0.25276	-1.84958
H	-8.00476	0.90820	-2.47767
H	-8.52217	1.20439	-0.78160
C	-8.53923	-2.43386	-2.33982
H	-6.73327	-1.42088	-2.98621
H	-6.51407	-2.54120	-1.59481
C	-9.55148	-1.40182	-2.85135
H	-10.41805	0.50172	-2.27093
H	-10.16283	-0.64032	-0.90600
H	-8.39817	-3.22283	-3.10962
H	-8.93392	-2.92013	-1.42058
H	-10.53332	-1.89799	-3.00801
H	-9.21117	-0.99799	-3.83033

Catalyst (2,4,6-c-Hex, 11)-Enamine protonation TS (pro-S) lowest free energy conformation
Electronic energy (Hartrees): -1870.491513
Free Energy at 1M concentration and 253 K (Hartrees): -1868.840412
Coordinates:

P	-0.03762	0.07961	0.29765
O	1.15171	1.00133	-0.32974
O	-1.36168	1.02828	0.43338
O	0.22148	-0.45177	1.66924
O	-0.26234	-0.94192	-0.84951
C	1.35891	2.16098	0.36651
C	2.64447	2.34960	1.07481
C	2.86476	3.47409	1.77016
H	3.81506	3.63075	2.26632
C	1.81900	4.51802	1.84323
C	2.03551	5.71835	2.68290
H	2.96329	5.84062	3.22788
C	1.08475	6.65474	2.77460
H	1.24300	7.53062	3.39043
C	-0.18784	6.49231	2.04512
H	-0.96032	7.24453	2.13989
C	-0.39156	5.41513	1.27797
H	-1.33958	5.30519	0.76807
C	0.66579	4.37767	1.14515
C	0.45986	3.16352	0.30117
C	-1.53378	1.97961	-0.53829
C	-2.76860	1.92964	-1.35839
C	-3.01602	2.89953	-2.25055
H	-3.92695	2.88022	-2.83686
C	-2.03840	3.98489	-2.46572
C	-2.27308	4.98127	-3.53610
H	-3.17988	4.94165	-4.12681
C	-1.36344	5.92995	-3.78258
H	-1.53409	6.65536	-4.56751
C	-0.11681	5.98156	-2.99559

H	0.62727	6.73554	-3.21793
C	0.10077	5.09204	-2.02024
H	1.03483	5.13324	-1.47625
C	-0.91602	4.04882	-1.71209
C	-0.69568	3.04030	-0.63194
C	-3.74306	0.82369	-1.17622
C	-4.63433	0.81621	0.03575
C	-3.86595	-0.16077	-2.11185
C	-5.58560	-0.13087	0.13899
C	-4.97807	-1.13858	-1.98677
C	-5.79398	-1.13686	-0.92412
H	-6.22677	-0.16196	1.00897
H	-5.13675	-1.86089	-2.77619
C	3.71377	1.33934	0.90173
C	4.38946	1.25079	-0.27918
C	4.01125	0.37040	2.01247
C	5.36100	0.14383	-0.47136
C	4.95453	-0.57003	1.81707
C	5.62357	-0.73269	0.50758
H	5.85215	0.03563	-1.42937
H	5.22655	-1.24266	2.61706
H	-0.23647	-2.14346	1.88302
C	-2.05681	-2.86285	2.50519
C	-0.78307	-3.64675	0.59634
C	-2.13954	-1.96081	3.58362
C	-3.14599	-3.71160	2.18608
C	-1.88756	-4.49412	0.24934
C	-3.29780	-1.92379	4.33820
H	-1.31086	-1.28743	3.77512
C	-4.30302	-3.66528	2.99444
C	-3.00757	-4.53877	1.02974
H	-1.80733	-5.12408	-0.62605
C	-4.38087	-2.78139	4.05351
H	-3.37498	-1.21725	5.15880
H	-5.13402	-4.32493	2.76160
H	-3.81806	-5.21468	0.77026
H	-5.27742	-2.73913	4.66315
C	0.35804	-3.41289	-0.23807
C	0.59667	-4.17422	-1.53378
H	-0.37756	-4.42751	-1.97094
H	1.12699	-5.11559	-1.36178
N	1.34174	-3.38427	-2.51068
N	-0.92162	-2.90897	1.72961
C	2.72422	-3.49470	-2.70226
C	3.40149	-4.72654	-2.66647
C	3.46276	-2.33884	-3.01830
C	4.76973	-4.78653	-2.92609
H	2.86041	-5.64209	-2.45072
C	4.82470	-2.41277	-3.29003
H	2.95247	-1.37979	-3.03548
C	5.49429	-3.63718	-3.24371
H	5.27175	-5.74950	-2.88477
H	5.37000	-1.50368	-3.52768
H	6.55948	-3.69282	-3.44444
H	1.02999	-2.42000	-2.52420
H	-0.00310	-2.04074	-0.59740
Cl	1.90498	-3.14215	0.65952
C	-4.47038	1.83652	1.16133
C	-5.74249	2.68706	1.35479
C	-4.06651	1.15087	2.48419

H	-3.66241	2.55130	0.91174
C	-5.53844	3.72085	2.47107
H	-6.61228	2.04621	1.61527
H	-5.97961	3.21939	0.40785
C	-3.86329	2.18847	3.59443
H	-4.84576	0.42718	2.80781
H	-3.12049	0.58697	2.34130
C	-5.12401	3.04234	3.78405
H	-6.48530	4.28015	2.63053
H	-4.75730	4.45187	2.16614
H	-3.62707	1.66512	4.54594
H	-3.00074	2.84390	3.34108
H	-4.92670	3.82068	4.55213
H	-5.95595	2.40194	4.15144
C	-2.90144	-0.30414	-3.28995
C	-2.32467	-1.73435	-3.39404
C	-3.58292	0.08265	-4.61548
H	-2.02923	0.37075	-3.15466
C	-1.31292	-1.82136	-4.54280
H	-3.12770	-2.47761	-3.58257
H	-1.83016	-2.01988	-2.44480
C	-2.58900	-0.01371	-5.78247
H	-4.44952	-0.58532	-4.81480
H	-3.96253	1.12468	-4.55391
C	-1.96559	-1.41447	-5.87129
H	-0.93563	-2.86369	-4.62216
H	-0.44471	-1.15881	-4.33145
H	-3.11718	0.21310	-6.73342
H	-1.78485	0.74347	-5.65022
H	-1.19775	-1.42368	-6.67442
H	-2.75141	-2.15387	-6.14129
C	-6.91315	-2.15669	-0.81183
C	-6.69473	-3.07531	0.40534
C	-8.28991	-1.46622	-0.73768
H	-6.91729	-2.80515	-1.71832
C	-7.82527	-4.10389	0.52886
H	-6.64501	-2.48021	1.34311
H	-5.73103	-3.61415	0.28758
C	-9.41497	-2.50178	-0.61273
H	-8.33825	-0.77821	0.13414
H	-8.45324	-0.86437	-1.65816
C	-9.19232	-3.41240	0.60157
H	-7.67061	-4.70934	1.44785
H	-7.80080	-4.79366	-0.34335
H	-10.38745	-1.97541	-0.50266
H	-9.46211	-3.11721	-1.53805
H	-9.99179	-4.18349	0.63303
H	-9.25798	-2.81224	1.53581
C	4.19311	2.25026	-1.42323
C	5.50789	2.98357	-1.75547
C	3.62617	1.56363	-2.68175
H	3.46951	3.04191	-1.13964
C	5.29174	4.00221	-2.88361
H	6.29409	2.26293	-2.06817
H	5.87248	3.51840	-0.85138
C	3.40496	2.58533	-3.80403
H	4.32135	0.77796	-3.04573
H	2.65625	1.07940	-2.44314
C	4.70653	3.32861	-4.13230
H	6.26377	4.47401	-3.14313

H	4.60443	4.80507	-2.53643
H	3.04368	2.05858	-4.71336
H	2.62254	3.31504	-3.49823
H	4.50213	4.10163	-4.90389
H	5.44811	2.61359	-4.55187
C	6.56566	-1.89766	0.26327
C	7.79679	-1.82037	1.18743
C	5.83159	-3.24233	0.44178
H	6.93682	-1.86106	-0.78717
C	8.74029	-3.00490	0.94015
H	7.48735	-1.82466	2.25508
H	8.34703	-0.87378	0.99406
C	6.78143	-4.42085	0.19464
H	5.41356	-3.32824	1.46833
H	4.98447	-3.30197	-0.27471
C	8.00588	-4.34064	1.11469
H	9.58690	-2.95561	1.65825
H	9.15991	-2.94087	-0.08799
H	6.24092	-5.37337	0.38267
H	7.11347	-4.42029	-0.86629
H	8.69986	-5.17455	0.87446
H	7.68566	-4.45548	2.17370
C	3.29840	0.46382	3.36118
C	2.73612	-0.89359	3.84365
C	4.22790	1.05898	4.43464
H	2.42580	1.14296	3.26713
C	1.96683	-0.71617	5.15803
H	3.55052	-1.62799	4.01772
H	2.06116	-1.32314	3.07867
C	3.48032	1.22658	5.76577
H	5.11108	0.40018	4.58789
H	4.59600	2.05358	4.10211
C	2.87114	-0.10235	6.23623
H	1.59997	-1.70588	5.50572
H	1.08158	-0.06338	4.99155
H	4.18661	1.59962	6.53823
H	2.67401	1.98368	5.64708
H	2.27516	0.07353	7.15739
H	3.68657	-0.81570	6.48793

Catalyst (2,4,6-c-Hex, 11)-Enamine protonation TS (pro-S) alternative conformation
Electronic energy (Hartrees): -1870.490626
Free Energy at 1M concentration and 253 K (Hartrees): -1868.83972
Coordinates:

P	-0.00559	0.13072	0.32600
O	1.16313	0.95217	-0.45920
O	-1.30293	1.12070	0.43706
O	0.32099	-0.26022	1.72999
O	-0.31060	-0.99851	-0.69454
C	1.42605	2.17675	0.09132
C	2.74970	2.41374	0.70924
C	3.02604	3.60467	1.25870
H	4.00307	3.79381	1.68690
C	2.00422	4.67459	1.26365
C	2.28545	5.95696	1.94860
H	3.24294	6.11889	2.42791
C	1.35676	6.91907	1.98153
H	1.56237	7.85510	2.48454
C	0.04438	6.70417	1.34161

H	-0.70931	7.47954	1.38963
C	-0.21806	5.55217	0.71366
H	-1.19386	5.40687	0.26919
C	0.81314	4.48252	0.64614
C	0.54174	3.18549	-0.04135
C	-1.51951	1.96354	-0.62198
C	-2.80795	1.86022	-1.34904
C	-3.08630	2.72120	-2.33847
H	-4.03542	2.66482	-2.85820
C	-2.09789	3.74131	-2.74194
C	-2.37533	4.61037	-3.90869
H	-3.31626	4.52616	-4.43816
C	-1.46283	5.49837	-4.31747
H	-1.66498	6.13056	-5.17242
C	-0.17125	5.60920	-3.61332
H	0.57309	6.31192	-3.96508
C	0.08567	4.83543	-2.55256
H	1.05031	4.91606	-2.06968
C	-0.93162	3.86321	-2.06604
C	-0.66729	2.98262	-0.88851
C	-3.80882	0.83707	-0.95390
C	-4.62058	1.04305	0.29610
C	-4.03039	-0.26084	-1.73058
C	-5.60272	0.16845	0.58721
C	-5.17106	-1.15456	-1.40433
C	-5.91897	-0.96230	-0.30777
H	-6.19007	0.28881	1.48807
H	-5.40994	-1.96835	-2.07408
C	3.78829	1.36237	0.60726
C	4.40358	1.11819	-0.58470
C	4.12114	0.52400	1.81048
C	5.34426	-0.02673	-0.68805
C	5.03805	-0.45374	1.68582
C	5.63908	-0.78647	0.37563
H	5.78555	-0.25990	-1.64808
H	5.33680	-1.03299	2.54696
H	-0.19198	-1.89440	2.15518
C	-2.01743	-2.45192	2.91260
C	-0.85540	-3.49867	1.05974
C	-2.01560	-1.43685	3.88887
C	-3.15724	-3.27273	2.72302
C	-2.00933	-4.32363	0.84538
C	-3.14083	-1.25929	4.67267
H	-1.14915	-0.79034	3.97834
C	-4.27993	-3.07865	3.55794
C	-3.10026	-4.22702	1.66137
H	-1.99133	-5.04849	0.04303
C	-4.27412	-2.08443	4.51738
H	-3.15245	-0.46636	5.41405
H	-5.15023	-3.71532	3.42724
H	-3.94911	-4.88712	1.50424
H	-5.14356	-1.93102	5.14811
C	0.25868	-3.40760	0.16288
C	0.41341	-4.31548	-1.04869
H	-0.58676	-4.57485	-1.41819
H	0.91476	-5.25410	-0.79494
N	1.14227	-3.66739	-2.13559
N	-0.91561	-2.63851	2.11036
C	2.51408	-3.83508	-2.35965
C	3.17047	-5.06650	-2.18639

C	3.26028	-2.74788	-2.85263
C	4.52577	-5.19139	-2.48754
H	2.62339	-5.93307	-1.82978
C	4.60889	-2.88763	-3.16193
H	2.76719	-1.78797	-2.97952
C	5.25776	-4.11062	-2.98045
H	5.01163	-6.15195	-2.33810
H	5.16027	-2.03001	-3.53708
H	6.31283	-4.21666	-3.21188
H	0.85179	-2.70355	-2.25285
H	-0.06989	-2.07472	-0.33343
Cl	1.85134	-3.10563	0.96471
C	-4.33941	2.20016	1.25377
C	-5.55984	3.13088	1.40591
C	-3.87046	1.68535	2.63097
H	-3.52325	2.83354	0.85543
C	-5.23621	4.29837	2.34867
H	-6.43367	2.57613	1.81038
H	-5.84188	3.53756	0.41023
C	-3.54852	2.85556	3.56796
H	-4.65306	1.05204	3.10231
H	-2.95918	1.06241	2.50768
C	-4.75749	3.78899	3.71521
H	-6.14630	4.92064	2.48774
H	-4.44992	4.94036	1.89347
H	-3.26706	2.45802	4.56687
H	-2.68038	3.42618	3.16972
H	-4.47535	4.65568	4.35076
H	-5.58636	3.24788	4.22252
C	-3.15174	-0.61358	-2.93069
C	-2.64007	-2.07041	-2.86151
C	-3.90403	-0.38432	-4.25455
H	-2.24713	0.03149	-2.94575
C	-1.71288	-2.36884	-4.04550
H	-3.48245	-2.79324	-2.89038
H	-2.09443	-2.24128	-1.91245
C	-2.99603	-0.69517	-5.45359
H	-4.80917	-1.02882	-4.30262
H	-4.23470	0.67414	-4.32067
C	-2.43788	-2.12379	-5.37609
H	-1.38264	-3.42880	-3.99591
H	-0.80761	-1.72521	-3.98680
H	-3.57754	-0.57957	-6.39338
H	-2.15450	0.03181	-5.48179
H	-1.72778	-2.28589	-6.21523
H	-3.26975	-2.85384	-5.48638
C	-7.08353	-1.87640	0.02836
C	-8.17816	-1.81867	-1.05662
C	-6.61180	-3.32635	0.25426
H	-7.54395	-1.52713	0.98041
C	-9.35430	-2.73499	-0.69425
H	-7.77015	-2.13087	-2.04259
H	-8.54973	-0.77529	-1.15421
C	-7.79258	-4.23655	0.61310
H	-6.11216	-3.72535	-0.65524
H	-5.87732	-3.34608	1.08466
C	-8.88056	-4.17644	-0.46667
H	-10.09876	-2.71981	-1.51914
H	-9.85437	-2.35663	0.22444
H	-7.43160	-5.28280	0.71302

H	-8.21958	-3.92726	1.59252
H	-9.74423	-4.80057	-0.15161
H	-8.48517	-4.59424	-1.41872
C	4.17014	1.97944	-1.82960
C	5.48029	2.63759	-2.30620
C	3.53181	1.16174	-2.97013
H	3.47477	2.81547	-1.60992
C	5.22875	3.51985	-3.53718
H	6.23795	1.86692	-2.56555
H	5.89681	3.26737	-1.48996
C	3.27564	2.04795	-4.19541
H	4.19465	0.32237	-3.26865
H	2.56597	0.73186	-2.63171
C	4.57310	2.71651	-4.66863
H	6.19550	3.93486	-3.89485
H	4.57297	4.37394	-3.25791
H	2.86225	1.42559	-5.01799
H	2.52174	2.82682	-3.94408
H	4.34600	3.39697	-5.51721
H	5.28114	1.93969	-5.03255
C	6.54601	-1.99490	0.22901
C	7.82290	-1.83793	1.07790
C	5.79686	-3.29028	0.60279
H	6.86487	-2.09301	-0.83459
C	8.73071	-3.06624	0.93076
H	7.56701	-1.70714	2.15171
H	8.38033	-0.93494	0.74614
C	6.71109	-4.51276	0.45463
H	5.42932	-3.24292	1.65094
H	4.91373	-3.41412	-0.06005
C	7.98121	-4.35326	1.29937
H	9.61283	-2.95229	1.59689
H	9.09968	-3.13566	-0.11628
H	6.16304	-5.42253	0.78132
H	6.98975	-4.64694	-0.61315
H	8.64672	-5.22659	1.12872
H	7.71216	-4.33305	2.37846
C	3.47274	0.78979	3.16872
C	2.91571	-0.49012	3.83462
C	4.45832	1.49069	4.12147
H	2.60557	1.46882	3.03262
C	2.20860	-0.14273	5.15000
H	3.72800	-1.21245	4.06114
H	2.20232	-0.99698	3.15658
C	3.77427	1.83001	5.45411
H	5.33830	0.83791	4.31394
H	4.82481	2.43129	3.65657
C	3.16866	0.57859	6.10668
H	1.84473	-1.07684	5.62960
H	1.32548	0.50179	4.94456
H	4.51995	2.27892	6.14486
H	2.97395	2.58288	5.28054
H	2.61706	0.87461	7.02471
H	3.98479	-0.11478	6.40719

Catalyst (2,4,6-c-Hex, 11)-Enamine protonation TS (pro-S) alternative conformation
Electronic energy (Hartrees): -1870.489376
Free Energy at 1M concentration and 253 K (Hartrees): -1868.839595
Coordinates:

P	-0.16593	-0.24082	-0.15702
O	-1.48432	0.47871	0.50578
O	0.86233	0.94142	-0.61456
O	-0.45769	-1.09922	-1.34463
O	0.45942	-0.94084	1.08234
C	-1.81987	1.72991	0.05463
C	-3.18270	1.93738	-0.49113
C	-3.58128	3.16233	-0.86124
H	-4.57767	3.31311	-1.26035
C	-2.65505	4.30719	-0.76483
C	-3.07054	5.63207	-1.28013
H	-4.06581	5.77099	-1.68372
C	-2.21610	6.66042	-1.25712
H	-2.51896	7.62701	-1.63846
C	-0.85278	6.47734	-0.72468
H	-0.15737	7.30677	-0.73315
C	-0.47139	5.28791	-0.24581
H	0.54282	5.17276	0.11188
C	-1.41867	4.13847	-0.24102
C	-1.01084	2.79362	0.26889
C	1.20727	1.78032	0.41351
C	2.62507	1.86079	0.83340
C	2.96516	2.62005	1.88634
H	4.00377	2.71273	2.18003
C	1.92668	3.36316	2.63197
C	2.28464	4.09198	3.86991
H	3.30714	4.08896	4.22643
C	1.34429	4.74956	4.55760
H	1.60536	5.27755	5.46558
C	-0.05393	4.75299	4.08430
H	-0.81175	5.27599	4.65316
C	-0.38612	4.10591	2.96121
H	-1.41941	4.10452	2.63922
C	0.65028	3.38043	2.18075
C	0.29667	2.61472	0.95135
C	3.66613	1.23124	-0.01268
C	3.93479	1.80723	-1.37837
C	4.34971	0.13050	0.40673
C	4.82655	1.19528	-2.17997
C	5.37064	-0.46697	-0.49260
C	5.57705	0.00578	-1.73081
H	5.02445	1.58398	-3.17036
H	5.94292	-1.31630	-0.14845
C	-4.10215	0.78580	-0.62864
C	-4.74226	0.26980	0.45759
C	-4.32892	0.18990	-1.98949
C	-5.65980	-0.88403	0.27070
C	-5.19952	-0.82854	-2.11675
C	-5.88455	-1.41031	-0.94151
H	-6.15543	-1.30702	1.13469
H	-5.40162	-1.24986	-3.09144
H	-0.17578	-2.81353	-0.76512
C	1.46323	-3.99021	-1.11986
C	0.21663	-3.88926	0.95345
C	1.63133	-3.49178	-2.42684
C	2.40391	-4.89120	-0.56045
C	1.15609	-4.80090	1.52990
C	2.72562	-3.90503	-3.16380
H	0.92202	-2.76516	-2.80911
C	3.50382	-5.29989	-1.34729

C	2.19260	-5.29786	0.79086
H	1.03533	-5.06990	2.56917
C	3.66305	-4.81497	-2.63070
H	2.87004	-3.51372	-4.16607
H	4.22676	-5.98940	-0.92075
H	2.88743	-6.00326	1.23888
H	4.51518	-5.12252	-3.22806
C	-0.77208	-3.16457	1.70652
C	-1.09466	-3.49876	3.16536
H	-1.40599	-4.54690	3.27079
H	-1.96473	-2.88970	3.44271
N	0.01383	-3.28132	4.08887
N	0.39504	-3.57802	-0.35668
C	0.27488	-2.03496	4.68017
C	1.11234	-1.98332	5.81159
C	-0.21488	-0.82734	4.15773
C	1.46109	-0.76513	6.38471
H	1.50660	-2.90933	6.22526
C	0.14091	0.38742	4.74321
H	-0.83135	-0.81865	3.27158
C	0.97995	0.43526	5.85459
H	2.11569	-0.75520	7.25189
H	-0.24582	1.30645	4.31184
H	1.25680	1.38634	6.29867
H	0.15410	-4.04879	4.73046
H	-0.10503	-1.87671	1.48557
Cl	-2.33658	-2.94898	0.79948
C	-4.56380	0.83199	1.86963
C	-5.89399	1.36685	2.43509
C	-3.96161	-0.22483	2.81617
H	-3.86300	1.69195	1.86895
C	-5.69151	1.94406	3.84361
H	-6.65587	0.55923	2.48371
H	-6.28407	2.16736	1.76926
C	-3.75218	0.35774	4.21890
H	-4.62326	-1.11439	2.89122
H	-2.98522	-0.56279	2.41683
C	-5.06960	0.90332	4.78545
H	-6.67300	2.26767	4.25193
H	-5.03257	2.83870	3.78995
H	-3.36213	-0.43631	4.89158
H	-2.99538	1.17212	4.17761
H	-4.87638	1.37265	5.77393
H	-5.78464	0.06516	4.93828
C	-6.81152	-2.60195	-1.10140
C	-8.01031	-2.25536	-2.00693
C	-6.04646	-3.82367	-1.64884
H	-7.22221	-2.88870	-0.10571
C	-8.94060	-3.46462	-2.16835
H	-7.66306	-1.93337	-3.01263
H	-8.58418	-1.41560	-1.55780
C	-6.98329	-5.02745	-1.81189
H	-5.58722	-3.58953	-2.63395
H	-5.22848	-4.09504	-0.94604
C	-8.17614	-4.67880	-2.71120
H	-9.76169	-3.20460	-2.87058
H	-9.39795	-3.71904	-1.18687
H	-6.41948	-5.87264	-2.26207
H	-7.35023	-5.35425	-0.81402
H	-8.86288	-5.55083	-2.76342

H	-7.81666	-4.46364	-3.74163
C	-3.60534	0.72616	-3.22499
C	-2.82383	-0.37624	-3.97203
C	-4.58671	1.43252	-4.18009
H	-2.85101	1.48008	-2.92062
C	-2.07498	0.21320	-5.17414
H	-3.50624	-1.17291	-4.33756
H	-2.09166	-0.84680	-3.28530
C	-3.84328	2.01442	-5.39043
H	-5.36315	0.72064	-4.53662
H	-5.09992	2.25896	-3.64197
C	-3.04415	0.92903	-6.12463
H	-1.55983	-0.60448	-5.72271
H	-1.29966	0.92830	-4.82008
H	-4.57904	2.46485	-6.09082
H	-3.15481	2.82069	-5.05348
H	-2.46780	1.39535	-6.95234
H	-3.74438	0.18842	-6.57003
C	4.10967	-0.52635	1.76580
C	3.80041	-2.03432	1.63182
C	5.32038	-0.30345	2.69165
H	3.22688	-0.07809	2.26828
C	3.56801	-2.66335	3.01017
H	4.63226	-2.57942	1.13952
H	2.89018	-2.17218	1.01009
C	5.07317	-0.92887	4.06996
H	6.23703	-0.74934	2.24674
H	5.49784	0.78621	2.81930
C	4.76031	-2.42409	3.94373
H	3.41162	-3.75611	2.90093
H	2.65440	-2.22649	3.45600
H	5.97753	-0.79532	4.70157
H	4.22566	-0.41039	4.57105
H	4.52494	-2.83473	4.94922
H	5.65283	-2.96094	3.55346
C	6.55449	-0.66295	-2.68065
C	7.99385	-0.63921	-2.12622
C	6.11222	-2.10470	-3.00587
H	6.56108	-0.09610	-3.63922
C	8.96582	-1.31413	-3.10264
H	8.04799	-1.16441	-1.14808
H	8.31323	0.41391	-1.96783
C	7.09354	-2.77459	-3.97533
H	6.04917	-2.71511	-2.07848
H	5.10334	-2.08476	-3.47189
C	8.52223	-2.74856	-3.41782
H	9.98180	-1.33524	-2.65293
H	9.02094	-0.72435	-4.04408
H	6.78320	-3.82878	-4.14057
H	7.06549	-2.25203	-4.95677
H	9.21526	-3.19043	-4.16561
H	8.57426	-3.36725	-2.49488
C	3.22897	3.07389	-1.86858
C	4.22743	4.21096	-2.16454
C	2.34655	2.79327	-3.10322
H	2.55921	3.46605	-1.07887
C	3.48376	5.48138	-2.60162
H	4.93736	3.91532	-2.96694
H	4.81695	4.43458	-1.24870
C	1.60327	4.06302	-3.53738

H	2.96445	2.42924	-3.95243
H	1.60100	2.00575	-2.86503
C	2.58694	5.20730	-3.81691
H	4.22373	6.26833	-2.86217
H	2.86372	5.86115	-1.75945
H	1.01765	3.84906	-4.45725
H	0.88827	4.36881	-2.74240
H	2.01602	6.12765	-4.06547
H	3.21887	4.94871	-4.69490

Catalyst (2,4,6-c-Hex, 11)-Enamine protonation TS (pro-S) alternative conformation
Electronic energy (Hartrees): -1870.491022
Free Energy at 1M concentration and 253 K (Hartrees): -1868.839587
Coordinates:

P	0.29338	0.45444	0.49260
O	1.54179	0.67621	-0.53108
O	-0.51575	1.86678	0.52877
O	0.69540	0.14251	1.89633
O	-0.59823	-0.57917	-0.25418
C	2.35172	1.71346	-0.14594
C	3.73561	1.41727	0.29690
C	4.54417	2.42088	0.66731
H	5.56409	2.21541	0.96901
C	4.04518	3.81180	0.68946
C	4.91142	4.89457	1.20955
H	5.91926	4.67404	1.53880
C	4.45122	6.14842	1.27928
H	5.08415	6.93876	1.66173
C	3.07401	6.45642	0.84828
H	2.70038	7.46907	0.92896
C	2.28509	5.48654	0.37142
H	1.27100	5.72915	0.08264
C	2.79297	4.09288	0.25738
C	1.93412	2.99329	-0.27563
C	-0.48243	2.63018	-0.60986
C	-1.75744	2.88541	-1.31979
C	-1.81940	3.84026	-2.25926
H	-2.74973	4.02583	-2.78302
C	-0.58827	4.51519	-2.71442
C	-0.65348	5.45581	-3.85671
H	-1.61057	5.72281	-4.28740
C	0.46873	5.97239	-4.36744
H	0.42005	6.65665	-5.20464
C	1.77862	5.59221	-3.80707
H	2.68525	5.98635	-4.24788
C	1.84857	4.74829	-2.77131
H	2.82328	4.45695	-2.40529
C	0.60621	4.19750	-2.16091
C	0.65158	3.26940	-0.98682
C	-2.90472	1.96622	-1.11644
C	-3.69687	2.02462	0.16147
C	-3.22322	1.04095	-2.06917
C	-4.71445	1.15985	0.33732
C	-4.40743	0.17173	-1.86046
C	-5.09823	0.19311	-0.71382
H	-5.29152	1.18066	1.25116
H	-4.70608	-0.50410	-2.64951
C	4.23332	0.01920	0.28346
C	4.48652	-0.62007	-0.89484

C	4.48451	-0.68915	1.58842
C	5.00450	-2.01205	-0.86181
C	5.01634	-1.92595	1.56643
C	5.26657	-2.63535	0.29641
H	5.18081	-2.52868	-1.79495
H	5.24954	-2.43643	2.49098
H	-0.13589	-1.18943	2.67109
C	-1.72752	-1.10149	3.96746
C	-1.51867	-2.57710	2.05758
C	-1.13752	-0.10319	4.76548
C	-3.05784	-1.52623	4.20757
C	-2.86690	-3.01456	2.28624
C	-1.86783	0.44584	5.80346
H	-0.13515	0.23657	4.53116
C	-3.77501	-0.94327	5.27469
C	-3.59460	-2.51558	3.32753
H	-3.28936	-3.74136	1.60741
C	-3.18870	0.02742	6.06470
H	-1.41888	1.21983	6.41824
H	-4.79530	-1.26753	5.45913
H	-4.60644	-2.87357	3.49883
H	-3.74454	0.47444	6.88240
C	-0.74819	-2.95859	0.91243
C	-1.26344	-4.04712	-0.01728
H	-1.49578	-4.97142	0.53266
H	-0.46196	-4.29800	-0.72687
N	-2.47540	-3.59430	-0.68702
N	-1.01891	-1.66677	2.93192
C	-3.20623	-4.42489	-1.53701
C	-4.25949	-3.86932	-2.28977
C	-2.96757	-5.80674	-1.64438
C	-5.04649	-4.66442	-3.11211
H	-4.45624	-2.80584	-2.21025
C	-3.77214	-6.59677	-2.46785
H	-2.15281	-6.26730	-1.09762
C	-4.81388	-6.04018	-3.20715
H	-5.85056	-4.20558	-3.68095
H	-3.56834	-7.66208	-2.53456
H	-5.43068	-6.66181	-3.84822
H	-2.43687	-2.62532	-0.97859
H	-0.67660	-1.59229	0.19818
Cl	1.02249	-3.17618	1.23244
C	-2.40426	0.84124	-3.34959
C	-1.92214	-0.61780	-3.50813
C	-3.21073	1.27596	-4.58874
H	-1.48134	1.45548	-3.32871
C	-1.08489	-0.77673	-4.78332
H	-2.77958	-1.32129	-3.55859
H	-1.29783	-0.90435	-2.63883
C	-2.37791	1.11019	-5.86717
H	-4.14030	0.67287	-4.68018
H	-3.50475	2.34287	-4.48811
C	-1.87874	-0.33294	-6.01851
H	-0.78933	-1.84175	-4.89889
H	-0.15594	-0.17125	-4.69837
H	-3.00085	1.37679	-6.74786
H	-1.50947	1.80500	-5.84003
H	-1.22822	-0.40344	-6.91660
H	-2.74558	-1.01356	-6.16883
C	-3.39522	3.05648	1.24665

C	-4.56761	4.04379	1.41793
C	-3.04712	2.39051	2.59781
H	-2.51689	3.66547	0.95166
C	-4.23976	5.09991	2.48217
H	-5.49362	3.50600	1.71703
H	-4.76552	4.55583	0.45093
C	-2.71265	3.45367	3.65142
H	-3.89526	1.77665	2.97020
H	-2.17510	1.71459	2.47677
C	-3.87338	4.44249	3.81928
H	-5.12179	5.75992	2.62782
H	-3.39387	5.73243	2.13332
H	-2.51310	2.95928	4.62485
H	-1.79199	4.00154	3.35229
H	-3.58130	5.22993	4.54683
H	-4.76016	3.91015	4.22862
C	-6.24289	-0.77872	-0.49221
C	-5.90838	-1.75193	0.65341
C	-7.56789	-0.04098	-0.21587
H	-6.39457	-1.39013	-1.41152
C	-7.05265	-2.74637	0.88007
H	-5.71987	-1.19416	1.59677
H	-4.98749	-2.31740	0.39704
C	-8.70926	-1.04102	0.01234
H	-7.47829	0.60929	0.68087
H	-7.82095	0.60537	-1.08435
C	-8.37195	-2.01228	1.15156
H	-6.80719	-3.39497	1.74844
H	-7.16348	-3.39896	-0.01401
H	-9.63781	-0.48608	0.26674
H	-8.89834	-1.61318	-0.92260
H	-9.19088	-2.75638	1.25341
H	-8.29675	-1.45221	2.10968
C	4.16133	-0.03208	2.93029
C	3.26099	-0.91726	3.82219
C	5.44896	0.34514	3.68831
H	3.59397	0.90528	2.75572
C	2.91281	-0.18625	5.12468
H	3.76766	-1.87061	4.08264
H	2.32678	-1.17446	3.28515
C	5.11435	1.06270	5.00385
H	6.04832	-0.56504	3.91040
H	6.07128	1.01838	3.06077
C	4.18701	0.21083	5.88232
H	2.29452	-0.85076	5.76594
H	2.31481	0.72344	4.89723
H	6.05500	1.27029	5.55777
H	4.62631	2.03763	4.78289
H	3.90855	0.78902	6.78946
H	4.72498	-0.70509	6.21224
C	5.78578	-4.06163	0.32966
C	4.76762	-5.04248	-0.28778
C	7.15709	-4.18086	-0.36657
H	5.92887	-4.36159	1.39259
C	5.29406	-6.48231	-0.23340
H	4.55893	-4.77596	-1.34685
H	3.80973	-4.98648	0.27435
C	7.67325	-5.62459	-0.31154
H	7.08636	-3.86568	-1.43034
H	7.88985	-3.51548	0.13995

C	6.65793	-6.59652	-0.92643
H	4.56909	-7.15713	-0.73737
H	5.38462	-6.80809	0.82625
H	8.63122	-5.69316	-0.87060
H	7.87261	-5.91103	0.74460
H	7.03611	-7.63614	-0.82165
H	6.54460	-6.38104	-2.01180
C	4.29413	0.04078	-2.26259
C	5.63100	0.14909	-3.02277
C	3.24997	-0.71711	-3.11050
H	3.92366	1.07967	-2.15525
C	5.42884	0.83424	-4.38150
H	6.07394	-0.85645	-3.18993
H	6.35150	0.74561	-2.42156
C	3.04889	-0.02945	-4.46692
H	3.57041	-1.76635	-3.28652
H	2.27699	-0.74749	-2.57587
C	4.37885	0.09528	-5.22203
H	6.39414	0.84967	-4.93178
H	5.10781	1.88792	-4.22565
H	2.33705	-0.62504	-5.07778
H	2.60710	0.98017	-4.31374
H	4.21098	0.65083	-6.16963
H	4.75607	-0.91836	-5.48179

Catalyst (2,4,6-c-Hex, 11)-Enamine protonation TS (pro-S) alternative conformation
Electronic energy (Hartrees): -1870.491022
Free Energy at 1M concentration and 253 K (Hartrees): -1868.839586
Coordinates:

P	0.29327	0.45471	0.49257
O	1.54154	0.67644	-0.53130
O	-0.51585	1.86704	0.52877
O	0.69550	0.14285	1.89625
O	-0.59845	-0.57895	-0.25402
C	2.35152	1.71369	-0.14630
C	3.73544	1.41749	0.29645
C	4.54407	2.42111	0.66666
H	5.56401	2.21563	0.96829
C	4.04512	3.81204	0.68876
C	4.91145	4.89482	1.20868
H	5.91934	4.67430	1.53780
C	4.45127	6.14869	1.27842
H	5.08426	6.93903	1.66074
C	3.07400	6.45668	0.84760
H	2.70040	7.46933	0.92830
C	2.28501	5.48679	0.37089
H	1.27088	5.72940	0.08225
C	2.79285	4.09312	0.25683
C	1.93392	2.99352	-0.27600
C	-0.48268	2.63038	-0.60991
C	-1.75777	2.88552	-1.31970
C	-1.81988	3.84035	-2.25919
H	-2.75028	4.02584	-2.78287
C	-0.58883	4.51532	-2.71450
C	-0.65423	5.45593	-3.85679
H	-1.61139	5.72289	-4.28737
C	0.46789	5.97256	-4.36766
H	0.41907	6.65681	-5.20485
C	1.77786	5.59244	-3.80744

H	2.68442	5.98663	-4.24836
C	1.84798	4.74853	-2.77169
H	2.82275	4.45723	-2.40579
C	0.60572	4.19769	-2.16113
C	0.65128	3.26961	-0.98703
C	-2.90496	1.96623	-1.11625
C	-3.69697	2.02454	0.16176
C	-3.22345	1.04091	-2.06893
C	-4.71441	1.15963	0.33773
C	-4.40752	0.17153	-1.86007
C	-5.09817	0.19282	-0.71334
H	-5.29136	1.18037	1.25164
H	-4.70616	-0.50435	-2.64907
C	4.23306	0.01938	0.28323
C	4.48612	-0.62014	-0.89497
C	4.48433	-0.68873	1.58831
C	5.00404	-2.01213	-0.86170
C	5.01612	-1.92555	1.56652
C	5.26620	-2.63522	0.29662
H	5.18025	-2.52896	-1.79475
H	5.24939	-2.43585	2.49115
H	-0.13547	-1.18930	2.67098
C	-1.72701	-1.10175	3.96748
C	-1.51799	-2.57729	2.05757
C	-1.13719	-0.10328	4.76541
C	-3.05720	-1.52682	4.20773
C	-2.86610	-3.01508	2.28636
C	-1.86753	0.44559	5.80347
H	-0.13493	0.23675	4.53097
C	-3.77440	-0.94404	5.27492
C	-3.59380	-2.51630	3.32775
H	-3.28845	-3.74197	1.60756
C	-3.18826	0.02682	6.06485
H	-1.41872	1.21971	6.41817
H	-4.79458	-1.26856	5.45948
H	-4.60553	-2.87454	3.49915
H	-3.74413	0.47370	6.88261
C	-0.74755	-2.95854	0.91232
C	-1.26267	-4.04706	-0.01747
H	-1.49479	-4.97149	0.53237
H	-0.46121	-4.29770	-0.72716
N	-2.47478	-3.59438	-0.68703
N	-1.01836	-1.66685	2.93187
C	-3.20534	-4.42492	-1.53731
C	-4.25881	-3.86945	-2.28984
C	-2.96617	-5.80664	-1.64520
C	-5.04551	-4.66451	-3.11250
H	-4.45596	-2.80607	-2.20988
C	-3.77045	-6.59665	-2.46899
H	-2.15125	-6.26711	-1.09861
C	-4.81239	-6.04015	-3.20807
H	-5.84975	-4.20575	-3.68116
H	-3.56626	-7.66185	-2.53610
H	-5.42896	-6.66176	-3.84939
H	-2.43653	-2.62531	-0.97832
H	-0.67644	-1.59215	0.19821
Cl	1.02321	-3.17570	1.23207
C	-2.40459	0.84127	-3.34944
C	-1.92227	-0.61771	-3.50796
C	-3.21126	1.27582	-4.58851

H	-1.48176	1.45564	-3.32868
C	-1.08515	-0.77658	-4.78324
H	-2.77962	-1.32133	-3.55829
H	-1.29782	-0.90413	-2.63872
C	-2.37857	1.11010	-5.86703
H	-4.14075	0.67260	-4.67982
H	-3.50541	2.34270	-4.48789
C	-1.87921	-0.33296	-6.01836
H	-0.78944	-1.84155	-4.89879
H	-0.15629	-0.17095	-4.69842
H	-3.00164	1.37658	-6.74766
H	-1.51022	1.80504	-5.84002
H	-1.22878	-0.40342	-6.91652
H	-2.74597	-1.01371	-6.16854
C	-3.39532	3.05644	1.24690
C	-4.56781	4.04362	1.41830
C	-3.04701	2.39052	2.59803
H	-2.51709	3.66553	0.95181
C	-4.23997	5.09978	2.48249
H	-5.49372	3.50572	1.71749
H	-4.76588	4.55562	0.45131
C	-2.71255	3.45373	3.65160
H	-3.89505	1.77657	2.97051
H	-2.17494	1.71469	2.47692
C	-3.87337	4.44242	3.81957
H	-5.12206	5.75969	2.62823
H	-3.39419	5.73239	2.13354
H	-2.51283	2.95936	4.62501
H	-1.79198	4.00169	3.35236
H	-3.58131	5.22990	4.54709
H	-4.76005	3.90998	4.22901
C	-6.24265	-0.77920	-0.49156
C	-5.90780	-1.75236	0.65399
C	-7.56772	-0.04167	-0.21501
H	-6.39436	-1.39062	-1.41086
C	-7.05187	-2.74699	0.88083
H	-5.71923	-1.19457	1.59733
H	-4.98685	-2.31768	0.39748
C	-8.70890	-1.04189	0.01337
H	-7.47809	0.60860	0.68172
H	-7.82102	0.60465	-1.08344
C	-8.37125	-2.01311	1.15253
H	-6.80617	-3.39556	1.74914
H	-7.16274	-3.39958	-0.01325
H	-9.63749	-0.48711	0.26792
H	-8.89803	-1.61408	-0.92154
H	-9.19004	-2.75734	1.25451
H	-8.29598	-1.45304	2.11064
C	4.16129	-0.03138	2.93008
C	3.26107	-0.91639	3.82228
C	5.44900	0.34600	3.68789
H	3.59389	0.90593	2.75538
C	2.91302	-0.18510	5.12464
H	3.76779	-1.86967	4.08289
H	2.32681	-1.17374	3.28540
C	5.11453	1.06385	5.00330
H	6.04839	-0.56413	3.91011
H	6.07126	1.01910	3.06013
C	4.18729	0.21217	5.88205
H	2.29481	-0.84948	5.76611

H	2.31497	0.72452	4.89705
H	6.05523	1.27157	5.55707
H	4.62645	2.03873	4.78217
H	3.90891	0.79056	6.78909
H	4.72530	-0.70366	6.21213
C	5.78530	-4.06152	0.33011
C	4.76699	-5.04242	-0.28700
C	7.15652	-4.18101	-0.36625
H	5.92850	-4.36127	1.39308
C	5.29333	-6.48229	-0.23239
H	4.55819	-4.77611	-1.34610
H	3.80918	-4.98624	0.27522
C	7.67258	-5.62476	-0.31098
H	7.08570	-3.86604	-1.43008
H	7.88940	-3.51558	0.14004
C	6.65711	-6.59673	-0.92556
H	4.56825	-7.15715	-0.73613
H	5.38400	-6.80785	0.82732
H	8.63048	-5.69352	-0.87015
H	7.87205	-5.91100	0.74519
H	7.03523	-7.63636	-0.82061
H	6.54368	-6.38147	-2.01096
C	4.29361	0.04043	-2.26283
C	5.63041	0.14857	-3.02314
C	3.24938	-0.71764	-3.11050
H	3.92315	1.07934	-2.15567
C	5.42815	0.83342	-4.38201
H	6.07335	-0.85701	-3.19012
H	6.35096	0.74522	-2.42212
C	3.04820	-0.03028	-4.46705
H	3.56978	-1.76693	-3.28631
H	2.27644	-0.74789	-2.57578
C	4.37809	0.09428	-5.22230
H	6.39340	0.84872	-4.93236
H	5.10713	1.88713	-4.22636
H	2.33631	-0.62602	-5.07773
H	2.60641	0.97937	-4.31407
H	4.21016	0.64961	-6.17001
H	4.75529	-0.91943	-5.48186

Catalyst (2,4,6-c-Hex, 11)-Enamine protonation TS (pro-S) alternative conformation
Electronic energy (Hartrees): -1870.491023
Free Energy at 1M concentration and 253 K (Hartrees): -1868.839571
Coordinates:

P	-0.29235	-0.45704	0.49258
O	-1.53964	-0.67819	-0.53258
O	0.51587	-1.86977	0.52962
O	-0.69574	-0.14484	1.89584
O	0.60079	0.57604	-0.25314
C	-2.35028	-1.71534	-0.14874
C	-3.73437	-1.41880	0.29321
C	-4.54383	-2.42235	0.66178
H	-5.56389	-2.21663	0.96280
C	-4.04554	-3.81352	0.68327
C	-4.91287	-4.89627	1.20158
H	-5.92102	-4.67554	1.52974
C	-4.45327	-6.15035	1.27105
H	-5.08700	-6.94067	1.65220
C	-3.07565	-6.45863	0.84158

H	-2.70253	-7.47147	0.92211
C	-2.28575	-5.48877	0.36633
H	-1.27139	-5.73161	0.07872
C	-2.79292	-4.09484	0.25250
C	-1.93300	-2.99527	-0.27879
C	0.48406	-2.63252	-0.60947
C	1.76000	-2.88719	-1.31791
C	1.82333	-3.84187	-2.25747
H	2.75431	-4.02700	-2.78024
C	0.59285	-4.51679	-2.71443
C	0.65968	-5.45710	-3.85687
H	1.61738	-5.72403	-4.28626
C	-0.46180	-5.97349	-4.36937
H	-0.41194	-6.65752	-5.20668
C	-1.77249	-5.59339	-3.81081
H	-2.67849	-5.98737	-4.25304
C	-1.84392	-4.74973	-2.77494
H	-2.81914	-4.45843	-2.41027
C	-0.60244	-4.19918	-2.16258
C	-0.64947	-3.27142	-0.98828
C	2.90647	-1.96717	-1.11351
C	3.69702	-2.02439	0.16547
C	3.22531	-1.04182	-2.06607
C	4.71364	-1.15869	0.34229
C	4.40842	-0.17140	-1.85609
C	5.09780	-0.19185	-0.70859
H	5.28954	-1.17866	1.25687
H	4.70731	0.50465	-2.64485
C	-4.23103	-0.02031	0.28164
C	-4.48277	0.62114	-0.89578
C	-4.48292	0.68601	1.58758
C	-5.00000	2.01334	-0.86082
C	-5.01419	1.92307	1.56723
C	-5.26283	2.63479	0.29822
H	-5.17514	2.53169	-1.79322
H	-5.24799	2.43208	2.49242
H	0.13277	1.18942	2.66935
C	1.72344	1.10595	3.96715
C	1.51319	2.57948	2.05582
C	1.13503	0.10673	4.76520
C	3.05255	1.53390	4.20824
C	2.86020	3.02021	2.28548
C	1.86568	-0.44005	5.80414
H	0.13368	-0.23553	4.53012
C	3.77006	0.95330	5.27641
C	3.58792	2.52382	3.32799
H	3.28177	3.74725	1.60634
C	3.18529	-0.01834	6.06642
H	1.41798	-1.21476	6.41891
H	4.78940	1.28008	5.46164
H	4.59877	2.88424	3.50003
H	3.74141	-0.46356	6.88491
C	0.74320	2.95784	0.90934
C	1.25719	4.04568	-0.02178
H	1.48682	4.97167	0.52651
H	0.45603	4.29332	-0.73288
N	2.47097	3.59414	-0.68915
N	1.01453	1.66890	2.93055
C	3.19921	4.42401	-1.54212
C	4.25495	3.86933	-2.29203

C	2.95530	5.80447	-1.65530
C	5.03907	4.66397	-3.11757
H	4.45594	2.80701	-2.20773
C	3.75705	6.59412	-2.48188
H	2.13864	6.26416	-1.11065
C	4.80111	6.03840	-3.21855
H	5.84512	4.20587	-3.68419
H	3.54919	7.65835	-2.55314
H	5.41568	6.65968	-3.86212
H	2.43527	2.62413	-0.97765
H	0.67590	1.59021	0.19727
Cl	-1.02834	3.17178	1.22676
C	2.40773	-0.84304	-3.34754
C	1.92426	0.61548	-3.50675
C	3.21624	-1.27699	-4.58563
H	1.48543	-1.45821	-3.32781
C	1.08857	0.77351	-4.78307
H	2.78100	1.31990	-3.55604
H	1.29842	0.90137	-2.63832
C	2.38496	-1.11207	-5.86515
H	4.14533	-0.67300	-4.67588
H	3.51117	-2.34361	-4.48457
C	1.88456	0.33056	-6.01717
H	0.79200	1.83821	-4.89905
H	0.16018	0.16702	-4.69937
H	3.00932	-1.37807	-6.74501
H	1.51717	-1.80774	-5.83915
H	1.23520	0.40042	-6.91615
H	2.75093	1.01205	-6.16629
C	3.39473	-3.05588	1.25082
C	4.56754	-4.04233	1.42409
C	3.04459	-2.38939	2.60122
H	2.51716	-3.66560	0.95507
C	4.23905	-5.09807	2.48850
H	5.49282	-3.50377	1.72402
H	4.76697	-4.55477	0.45760
C	2.70943	-3.45217	3.65499
H	3.89194	-1.77489	2.97435
H	2.17239	-1.71397	2.47883
C	3.87057	-4.44018	3.82482
H	5.12132	-5.75744	2.63561
H	3.39401	-5.73133	2.13894
H	2.50835	-2.95733	4.62789
H	1.78949	-4.00075	3.35499
H	3.57806	-5.22742	4.55241
H	4.75650	-3.90708	4.23498
C	6.24108	0.78131	-0.48568
C	5.90372	1.75466	0.65898
C	7.56647	0.04513	-0.20700
H	6.39349	1.39247	-1.40503
C	7.04659	2.75040	0.88707
H	5.71425	1.19711	1.60228
H	4.98265	2.31908	0.40088
C	8.70638	1.04651	0.02260
H	7.47621	-0.60488	0.68985
H	7.82158	-0.60128	-1.07483
C	8.36623	2.01782	1.16093
H	6.79908	3.39909	1.75478
H	7.15815	3.40275	-0.00709
H	9.63514	0.49269	0.27865

H	8.89629	1.61853	-0.91226
H	9.18420	2.76282	1.26381
H	8.29010	1.45802	2.11913
C	-4.16098	0.02664	2.92861
C	-3.26154	0.91028	3.82297
C	-5.44928	-0.35189	3.68482
H	-3.59341	-0.91038	2.75290
C	-2.91438	0.17683	5.12435
H	-3.76859	1.86302	4.08485
H	-2.32692	1.16873	3.28724
C	-5.11581	-1.07195	4.99928
H	-6.04875	0.55792	3.90810
H	-6.07114	-1.02388	3.05553
C	-4.18919	-0.22179	5.88015
H	-2.29672	0.84020	5.76738
H	-2.31606	-0.73234	4.89568
H	-6.05693	-1.28056	5.55201
H	-4.62761	-2.04647	4.77688
H	-3.91146	-0.80174	6.78638
H	-4.72745	0.69345	6.21144
C	-5.78112	4.06133	0.33345
C	-4.76166	5.04258	-0.28121
C	-7.15163	4.18273	-0.36396
H	-5.92511	4.35953	1.39675
C	-5.28718	6.48266	-0.22484
H	-4.55205	4.77778	-1.34052
H	-3.80441	4.98495	0.28179
C	-7.66687	5.62671	-0.30691
H	-7.08003	3.86938	-1.42821
H	-7.88536	3.51697	0.14063
C	-6.65025	6.59901	-0.91905
H	-4.56124	7.15786	-0.72687
H	-5.37861	6.80663	0.83529
H	-8.62421	5.69692	-0.86682
H	-7.86712	5.91141	0.74953
H	-7.02784	7.63869	-0.81283
H	-6.53596	6.38537	-2.00467
C	-4.28953	-0.03744	-2.26449
C	-5.62584	-0.14385	-3.02589
C	-3.24439	0.72151	-3.11026
H	-3.91955	-1.07664	-2.15863
C	-5.42292	-0.82663	-4.38569
H	-6.06826	0.86216	-3.19158
H	-6.34704	-0.74116	-2.42631
C	-3.04258	0.03625	-4.46776
H	-3.56420	1.77121	-3.28464
H	-2.27180	0.75052	-2.57484
C	-4.37200	-0.08657	-5.22409
H	-6.38780	-0.84069	-4.93672
H	-5.10242	-1.88071	-4.23149
H	-2.33006	0.63268	-5.07701
H	-2.60130	-0.97381	-4.31610
H	-4.20365	-0.64045	-6.17257
H	-4.74862	0.92769	-5.48228

Catalyst (2,4,6-c-Hex, 11)-Enamine protonation TS (pro-S) alternative conformation
Electronic energy (Hartrees): -1870.491023
Free Energy at 1M concentration and 253 K (Hartrees): -1868.839571
Coordinates:

P	-0.29227	-0.45720	0.49272
O	-1.53968	-0.67826	-0.53233
O	0.51562	-1.87009	0.52995
O	-0.69544	-0.14466	1.89596
O	0.60105	0.57553	-0.25328
C	-2.35040	-1.71534	-0.14845
C	-3.73446	-1.41866	0.29350
C	-4.54399	-2.42211	0.66214
H	-5.56403	-2.21628	0.96317
C	-4.04584	-3.81333	0.68367
C	-4.91325	-4.89597	1.20206
H	-5.92136	-4.67514	1.53026
C	-4.45377	-6.15009	1.27158
H	-5.08755	-6.94034	1.65278
C	-3.07619	-6.45852	0.84207
H	-2.70316	-7.47139	0.92263
C	-2.28622	-5.48876	0.36674
H	-1.27189	-5.73171	0.07910
C	-2.79327	-4.09479	0.25286
C	-1.93325	-2.99532	-0.27849
C	0.48382	-2.63281	-0.60915
C	1.75975	-2.88755	-1.31759
C	1.82302	-3.84225	-2.25713
H	2.75399	-4.02745	-2.77989
C	0.59249	-4.51710	-2.71409
C	0.65927	-5.45744	-3.85652
H	1.61695	-5.72442	-4.28590
C	-0.46224	-5.97378	-4.36900
H	-0.41242	-6.65782	-5.20630
C	-1.77291	-5.59360	-3.81044
H	-2.67893	-5.98755	-4.25265
C	-1.84429	-4.74991	-2.77458
H	-2.81949	-4.45856	-2.40990
C	-0.60277	-4.19941	-2.16225
C	-0.64975	-3.27162	-0.98798
C	2.90626	-1.96756	-1.11320
C	3.69675	-2.02471	0.16582
C	3.22520	-1.04231	-2.06583
C	4.71346	-1.15911	0.34258
C	4.40838	-0.17197	-1.85589
C	5.09776	-0.19242	-0.70839
H	5.28932	-1.17902	1.25719
H	4.70734	0.50400	-2.64468
C	-4.23102	-0.02013	0.28182
C	-4.48279	0.62121	-0.89565
C	-4.48278	0.68633	1.58770
C	-5.00002	2.01342	-0.86080
C	-5.01406	1.92338	1.56726
C	-5.26280	2.63497	0.29820
H	-5.17521	2.53167	-1.79323
H	-5.24778	2.43249	2.49242
H	0.13314	1.18991	2.66897
C	1.72385	1.10711	3.96677
C	1.51334	2.57998	2.05494
C	1.13566	0.10799	4.76511
C	3.05288	1.53541	4.20773
C	2.86025	3.02104	2.28448
C	1.86646	-0.43836	5.80418
H	0.13437	-0.23453	4.53017
C	3.77051	0.95528	5.27607

C	3.58806	2.52515	3.32717
H	3.28170	3.74792	1.60510
C	3.18597	-0.01628	6.06634
H	1.41893	-1.21301	6.41915
H	4.78976	1.28235	5.46121
H	4.59883	2.88585	3.49911
H	3.74219	-0.46114	6.88496
C	0.74329	2.95779	0.90833
C	1.25709	4.04534	-0.02325
H	1.48629	4.97168	0.52460
H	0.45596	4.29233	-0.73461
N	2.47112	3.59386	-0.69019
N	1.01483	1.66961	2.92999
C	3.19950	4.42363	-1.54313
C	4.25564	3.86896	-2.29248
C	2.95535	5.80401	-1.65678
C	5.03992	4.66354	-3.11793
H	4.45683	2.80669	-2.20781
C	3.75726	6.59360	-2.48326
H	2.13838	6.26368	-1.11258
C	4.80173	6.03789	-3.21938
H	5.84629	4.20545	-3.68411
H	3.54921	7.65776	-2.55489
H	5.41642	6.65912	-3.86286
H	2.43572	2.62375	-0.97839
H	0.67608	1.58987	0.19670
Cl	-1.02828	3.17164	1.22569
C	2.40769	-0.84358	-3.34735
C	1.92437	0.61498	-3.50674
C	3.21621	-1.27774	-4.58536
H	1.48532	-1.45864	-3.32759
C	1.08877	0.77297	-4.78312
H	2.78119	1.31931	-3.55606
H	1.29852	0.90102	-2.63838
C	2.38500	-1.11287	-5.86494
H	4.14537	-0.67386	-4.67563
H	3.51103	-2.34438	-4.48419
C	1.88477	0.32980	-6.01714
H	0.79232	1.83768	-4.89922
H	0.16031	0.16658	-4.69940
H	3.00938	-1.37903	-6.74474
H	1.51714	-1.80844	-5.83890
H	1.23546	0.39963	-6.91615
H	2.75122	1.01118	-6.16629
C	3.39425	-3.05597	1.25134
C	4.56691	-4.04254	1.42489
C	3.04411	-2.38918	2.60160
H	2.51663	-3.66563	0.95564
C	4.23818	-5.09804	2.48948
H	5.49224	-3.50405	1.72480
H	4.76635	-4.55519	0.45852
C	2.70870	-3.45170	3.65553
H	3.89153	-1.77474	2.97468
H	2.17204	-1.71363	2.47902
C	3.86967	-4.43986	3.82564
H	5.12036	-5.75750	2.63678
H	3.39309	-5.73125	2.13996
H	2.50759	-2.95664	4.62832
H	1.78869	-4.00020	3.35555
H	3.57698	-5.22692	4.55335

H	4.75564	-3.90681	4.23580
C	6.24117	0.78061	-0.48556
C	5.90393	1.75414	0.65899
C	7.56644	0.04426	-0.20676
H	6.39370	1.39164	-1.40497
C	7.04694	2.74973	0.88699
H	5.71434	1.19672	1.60235
H	4.98295	2.31867	0.40079
C	8.70650	1.04549	0.02275
H	7.47607	-0.60563	0.69017
H	7.82148	-0.60229	-1.07451
C	8.36646	2.01698	1.16096
H	6.79950	3.39855	1.75463
H	7.15862	3.40196	-0.00723
H	9.63517	0.49157	0.27888
H	8.89651	1.61738	-0.91217
H	9.18454	2.76187	1.26378
H	8.29022	1.45730	2.11922
C	-4.16067	0.02712	2.92877
C	-3.26112	0.91088	3.82289
C	-5.44886	-0.35134	3.68519
H	-3.59311	-0.90991	2.75309
C	-2.91378	0.17759	5.12432
H	-3.76816	1.86364	4.08473
H	-2.32659	1.16929	3.28702
C	-5.11521	-1.07122	4.99969
H	-6.04832	0.55850	3.90843
H	-6.07080	-1.02341	3.05605
C	-4.18849	-0.22096	5.88033
H	-2.29604	0.84104	5.76719
H	-2.31547	-0.73160	4.89567
H	-6.05626	-1.27978	5.55256
H	-4.62703	-2.04577	4.77734
H	-3.91063	-0.80080	6.78659
H	-4.72672	0.69432	6.21158
C	-5.78114	4.06150	0.33332
C	-4.76177	5.04272	-0.28152
C	-7.15171	4.18276	-0.36400
H	-5.92507	4.35979	1.39661
C	-5.28735	6.48278	-0.22527
H	-4.55221	4.77781	-1.34081
H	-3.80448	4.98520	0.28143
C	-7.66700	5.62672	-0.30706
H	-7.08017	3.86929	-1.42822
H	-7.88537	3.51702	0.14072
C	-6.65048	6.59899	-0.91939
H	-4.56148	7.15795	-0.72743
H	-5.37872	6.80687	0.83483
H	-8.62439	5.69682	-0.86692
H	-7.86719	5.91154	0.74935
H	-7.02810	7.63867	-0.81327
H	-6.53625	6.38522	-2.00500
C	-4.28958	-0.03748	-2.26431
C	-5.62589	-0.14391	-3.02571
C	-3.24441	0.72137	-3.11013
H	-3.91963	-1.07668	-2.15837
C	-5.42297	-0.82681	-4.38545
H	-6.06828	0.86210	-3.19149
H	-6.34711	-0.74115	-2.42609
C	-3.04261	0.03599	-4.46757

H	-3.56420	1.77106	-3.28460
H	-2.27183	0.75039	-2.57470
C	-4.37203	-0.08685	-5.22391
H	-6.38785	-0.84088	-4.93649
H	-5.10250	-1.88088	-4.23117
H	-2.33006	0.63235	-5.07686
H	-2.60135	-0.97407	-4.31583
H	-4.20368	-0.64082	-6.17233
H	-4.74861	0.92739	-5.48218

Catalyst (2,4,6-c-Hex, 11)-Enamine protonation TS (pro-S) alternative conformation
Electronic energy (Hartrees): -1870.489217
Free Energy at 1M concentration and 253 K (Hartrees): -1868.838965
Coordinates:

P	-0.12072	-0.12533	-0.34773
O	-1.47499	0.42450	0.39878
O	0.96464	1.09382	-0.36197
O	-0.32696	-0.58717	-1.75354
O	0.38068	-1.18232	0.67673
C	-1.75769	1.76255	0.29850
C	-3.07542	2.16194	-0.25118
C	-3.42646	3.45501	-0.28704
H	-4.39073	3.74646	-0.68706
C	-2.49113	4.49331	0.18821
C	-2.84662	5.92360	0.04238
H	-3.80622	6.20597	-0.37258
C	-1.98135	6.87267	0.41500
H	-2.24011	7.91769	0.30366
C	-0.66548	6.49854	0.96681
H	0.04027	7.27144	1.24275
C	-0.33909	5.20956	1.11364
H	0.64252	4.96156	1.49438
C	-1.30049	4.14022	0.72620
C	-0.95242	2.69293	0.86118
C	1.23031	1.59346	0.88673
C	2.60553	1.49029	1.42642
C	2.86355	1.90747	2.67538
H	3.87295	1.86785	3.06673
C	1.77635	2.45591	3.51424
C	2.03549	2.79243	4.93249
H	3.02220	2.64733	5.35436
C	1.04925	3.26891	5.70051
H	1.23782	3.50975	6.73869
C	-0.30143	3.46211	5.13720
H	-1.09790	3.83442	5.76843
C	-0.54368	3.17076	3.85393
H	-1.54454	3.30176	3.46346
C	0.54576	2.65303	2.98507
C	0.29005	2.28056	1.56415
C	3.70673	1.07811	0.52472
C	4.11534	2.00591	-0.58901
C	4.32229	-0.12869	0.66394
C	5.06334	1.60495	-1.45669
C	5.40923	-0.49391	-0.28113
C	5.74095	0.30142	-1.30913
H	5.36168	2.24977	-2.27297
H	5.92738	-1.43285	-0.15006
C	-4.00725	1.12505	-0.74896
C	-4.71416	0.34840	0.11903

C	-4.16543	0.93213	-2.23130
C	-5.63007	-0.68647	-0.42475
C	-5.03761	0.00871	-2.67816
C	-5.78976	-0.85594	-1.74610
H	-6.17511	-1.31800	0.26290
H	-5.19167	-0.12614	-3.74049
H	-0.13993	-2.41219	-1.67918
C	1.50032	-3.51397	-2.22076
C	0.06654	-3.95191	-0.31908
C	1.80252	-2.67023	-3.30783
C	2.36347	-4.58259	-1.87053
C	0.92647	-5.03593	0.04360
C	2.95202	-2.90777	-4.03844
H	1.14938	-1.83050	-3.52105
C	3.52323	-4.80174	-2.64686
C	2.01657	-5.34944	-0.71842
H	0.70402	-5.58327	0.94792
C	3.81410	-3.97726	-3.71607
H	3.19936	-2.25256	-4.86793
H	4.18651	-5.61952	-2.37988
H	2.65030	-6.18644	-0.43754
H	4.71162	-4.14159	-4.30340
C	-0.96801	-3.42337	0.52987
C	-1.44778	-4.15032	1.78987
H	-1.82871	-5.15069	1.54354
H	-2.30402	-3.58041	2.17338
N	-0.42723	-4.31777	2.81930
N	0.37400	-3.28727	-1.46325
C	-0.15625	-3.33563	3.78554
C	0.54545	-3.70589	4.94922
C	-0.49740	-1.98462	3.61331
C	0.90637	-2.75355	5.89625
H	0.82415	-4.74763	5.09461
C	-0.12996	-1.03967	4.57102
H	-1.00381	-1.65258	2.71966
C	0.57387	-1.40812	5.71587
H	1.45366	-3.06654	6.78118
H	-0.39796	0.00047	4.40680
H	0.86120	-0.66435	6.45247
H	-0.40576	-5.25104	3.20545
H	-0.24567	-2.16387	0.74838
Cl	-2.43374	-2.86899	-0.39882
C	-4.61133	0.49575	1.63868
C	-5.96623	0.88946	2.25870
C	-4.07749	-0.79668	2.28731
H	-3.90269	1.30414	1.91227
C	-5.83920	1.05219	3.78036
H	-6.73835	0.11983	2.04252
H	-6.30854	1.85198	1.81977
C	-3.94279	-0.62779	3.80522
H	-4.75280	-1.65435	2.07976
H	-3.08403	-1.03932	1.86189
C	-5.28440	-0.22326	4.43031
H	-6.83903	1.27724	4.20985
H	-5.16836	1.90932	4.01007
H	-3.60221	-1.58658	4.25228
H	-3.17500	0.14537	4.02996
H	-5.14322	-0.04850	5.51852
H	-6.01657	-1.05168	4.30874
C	-6.70523	-1.93186	-2.30163

C	-6.22299	-3.34027	-1.89654
C	-8.16924	-1.70621	-1.87135
H	-6.67694	-1.88109	-3.41377
C	-7.14480	-4.42106	-2.47545
H	-6.19880	-3.44362	-0.78975
H	-5.19121	-3.50354	-2.27767
C	-9.08236	-2.79418	-2.45112
H	-8.25898	-1.71587	-0.76330
H	-8.51422	-0.71355	-2.23442
C	-8.59940	-4.19254	-2.04514
H	-6.80936	-5.41864	-2.11876
H	-7.07797	-4.41475	-3.58571
H	-10.11778	-2.64133	-2.07769
H	-9.10363	-2.71341	-3.56027
H	-9.24760	-4.95740	-2.52430
H	-8.68597	-4.31220	-0.94266
C	-3.36821	1.76773	-3.23300
C	-2.55002	0.89141	-4.20580
C	-4.28798	2.72675	-4.01325
H	-2.62924	2.39560	-2.69492
C	-1.73040	1.76662	-5.16264
H	-3.21421	0.23580	-4.80828
H	-1.86054	0.23954	-3.63192
C	-3.47227	3.59673	-4.97956
H	-5.04715	2.15482	-4.59042
H	-4.82592	3.38863	-3.30009
C	-2.63930	2.73186	-5.93544
H	-1.18935	1.11597	-5.88293
H	-0.97261	2.34377	-4.58786
H	-4.16348	4.23501	-5.57081
H	-2.79919	4.26729	-4.40088
H	-2.01378	3.39107	-6.57489
H	-3.31744	2.15324	-6.60064
C	3.93982	-1.13335	1.75032
C	3.61396	-2.52457	1.16215
C	5.06030	-1.24120	2.80169
H	3.02241	-0.80512	2.28275
C	3.23379	-3.50587	2.27637
H	4.47898	-2.94725	0.61027
H	2.76717	-2.43710	0.44856
C	4.66806	-2.21993	3.91558
H	6.00566	-1.58583	2.32780
H	5.24789	-0.24256	3.25191
C	4.33504	-3.60065	3.33880
H	3.06260	-4.51352	1.84510
H	2.29220	-3.16688	2.74825
H	5.51038	-2.31582	4.63386
H	3.78824	-1.82436	4.47022
H	3.99386	-4.26744	4.15981
H	5.24879	-4.04824	2.88959
C	6.78672	-0.11597	-2.32741
C	8.16846	-0.32150	-1.67302
C	6.34333	-1.38196	-3.08946
H	6.89549	0.69942	-3.07809
C	9.21110	-0.73698	-2.71894
H	8.11926	-1.10556	-0.88666
H	8.49603	0.62595	-1.19221
C	7.39474	-1.79505	-4.12653
H	6.18028	-2.22742	-2.38572
H	5.38284	-1.18089	-3.61142

C	8.76564	-1.99874	-3.46943
H	10.18012	-0.93493	-2.21227
H	9.36711	0.09312	-3.44267
H	7.07678	-2.74202	-4.61344
H	7.47012	-1.01385	-4.91465
H	9.51476	-2.24260	-4.25307
H	8.71734	-2.85648	-2.76285
C	3.48819	3.39293	-0.74855
C	4.53680	4.51624	-0.62117
C	2.71704	3.51993	-2.07916
H	2.75753	3.57576	0.06323
C	3.86791	5.89387	-0.73317
H	5.31098	4.42661	-1.41354
H	5.04383	4.44007	0.36546
C	2.04866	4.89640	-2.18847
H	3.40152	3.38189	-2.94398
H	1.93335	2.73556	-2.13865
C	3.08173	6.02214	-2.04528
H	4.64767	6.68457	-0.69427
H	3.18158	6.04842	0.12879
H	1.54577	4.98283	-3.17562
H	1.27085	4.99874	-1.40024
H	2.55911	7.00259	-2.06449
H	3.78609	5.99259	-2.90557

Catalyst (2,4,6-c-Hex, 11)-Enamine protonation TS (pro-S) alternative conformation
Electronic energy (Hartrees): -1870.488353
Free Energy at 1M concentration and 253 K (Hartrees): -1868.838783
Coordinates:

P	-0.23739	-0.26785	-0.16843
O	-1.54515	0.17727	0.71676
O	0.91881	0.85639	0.08281
O	-0.49689	-0.37022	-1.63663
O	0.22534	-1.56262	0.55276
C	-1.70931	1.51654	0.96717
C	-2.94798	2.17539	0.49045
C	-3.20457	3.44893	0.81871
H	-4.10628	3.93178	0.46025
C	-2.22227	4.22931	1.59486
C	-2.46283	5.67007	1.83964
H	-3.38665	6.13059	1.51213
C	-1.53645	6.41119	2.45606
H	-1.71071	7.46529	2.62911
C	-0.26616	5.79832	2.88651
H	0.49247	6.41031	3.35723
C	-0.04418	4.49514	2.68198
H	0.90969	4.08051	2.97716
C	-1.07761	3.64762	2.02306
C	-0.84664	2.19257	1.76168
C	1.25155	0.99823	1.40572
C	2.64488	0.73094	1.82846
C	2.97077	0.80303	3.12872
H	3.99412	0.64154	3.44517
C	1.93998	1.13750	4.13502
C	2.26888	1.08492	5.57751
H	3.26425	0.80419	5.89850
C	1.33477	1.37588	6.49001
H	1.57394	1.33194	7.54462
C	-0.02703	1.75319	6.06263

H	-0.78065	1.97593	6.80680
C	-0.33163	1.81338	4.76107
H	-1.33897	2.07658	4.46532
C	0.69841	1.50102	3.73664
C	0.37230	1.52177	2.28253
C	3.69087	0.54139	0.79489
C	4.05376	1.70310	-0.09444
C	4.29813	-0.66487	0.61704
C	4.96463	1.51822	-1.06764
C	5.34692	-0.79553	-0.42843
C	5.64874	0.21987	-1.24900
H	5.22634	2.33525	-1.72577
H	5.86700	-1.73771	-0.53849
C	-3.85923	1.44771	-0.41868
C	-4.72913	0.51674	0.06007
C	-3.81111	1.75044	-1.89112
C	-5.61642	-0.19404	-0.89560
C	-4.66852	1.11682	-2.71291
C	-5.59606	0.08255	-2.20891
H	-6.29086	-0.95135	-0.52082
H	-4.68189	1.34794	-3.76960
H	-0.31124	-2.12401	-2.04446
C	1.30958	-3.03533	-2.90598
C	-0.06974	-3.97023	-1.15050
C	1.57949	-1.93221	-3.74008
C	2.18341	-4.15135	-2.87278
C	0.80238	-5.10237	-1.10612
C	2.70796	-1.95870	-4.53794
H	0.91902	-1.07207	-3.70641
C	3.32093	-4.14795	-3.71083
C	1.87040	-5.19692	-1.95381
H	0.60081	-5.87776	-0.38083
C	3.58071	-3.06774	-4.53137
H	2.93054	-1.10620	-5.17222
H	3.99245	-5.00150	-3.68754
H	2.51141	-6.07394	-1.92176
H	4.46166	-3.06340	-5.16509
C	-1.09272	-3.70065	-0.17566
C	-1.48251	-4.73330	0.88499
H	-1.71235	-5.70852	0.43465
H	-2.41402	-4.38089	1.34657
N	-0.44500	-4.95121	1.88633
N	0.20633	-3.02424	-2.08457
C	-0.29708	-4.12659	3.01217
C	0.52877	-4.55631	4.06941
C	-0.90167	-2.86348	3.11153
C	0.76332	-3.73657	5.16812
H	1.00785	-5.53145	4.00872
C	-0.65853	-2.05069	4.21808
H	-1.52459	-2.48843	2.31340
C	0.17534	-2.47114	5.25234
H	1.41382	-4.08998	5.96350
H	-1.12998	-1.07279	4.25770
H	0.36575	-1.82854	6.10638
H	-0.26689	-5.92498	2.08573
H	-0.39351	-2.53006	0.35555
Cl	-2.59283	-2.97410	-0.90763
C	-4.84080	0.17457	1.54698
C	-6.24688	0.49472	2.09148
C	-4.48524	-1.30254	1.80556

H	-4.13109	0.78072	2.14742
C	-6.33992	0.15997	3.58706
H	-7.02309	-0.08343	1.54510
H	-6.46136	1.57645	1.94940
C	-4.57139	-1.63021	3.30086
H	-5.16851	-1.97949	1.24908
H	-3.45345	-1.49844	1.45382
C	-5.96615	-1.30527	3.85131
H	-7.37763	0.34283	3.93993
H	-5.66115	0.82738	4.16259
H	-4.35691	-2.71004	3.45283
H	-3.80362	-1.04844	3.85712
H	-5.98124	-1.49574	4.94593
H	-6.71874	-1.97304	3.37708
C	-6.48633	-0.66067	-3.18835
C	-6.15442	-2.16719	-3.21387
C	-7.98033	-0.43136	-2.88068
H	-6.29564	-0.26380	-4.21128
C	-7.04758	-2.90633	-4.21839
H	-6.29681	-2.61760	-2.20732
H	-5.09025	-2.30846	-3.50397
C	-8.86558	-1.17798	-3.88698
H	-8.22851	-0.78214	-1.85533
H	-8.20824	0.65551	-2.93559
C	-8.53230	-2.67532	-3.90914
H	-6.82852	-3.99482	-4.17221
H	-6.82142	-2.55551	-5.24940
H	-9.93259	-1.04415	-3.60682
H	-8.72244	-0.74808	-4.90282
H	-9.15032	-3.17672	-4.68467
H	-8.78373	-3.12859	-2.92495
C	-2.83118	2.77906	-2.45994
C	-1.96649	2.20996	-3.60569
C	-3.56783	4.04899	-2.92565
H	-2.11639	3.09002	-1.67107
C	-0.96082	3.26096	-4.09370
H	-2.59643	1.89783	-4.46566
H	-1.41127	1.31932	-3.25134
C	-2.56867	5.10237	-3.42505
H	-4.28276	3.80501	-3.74194
H	-4.14686	4.47660	-2.07859
C	-1.68098	4.53939	-4.54368
H	-0.38119	2.84666	-4.94649
H	-0.24305	3.50146	-3.27834
H	-3.12610	5.98403	-3.80806
H	-1.93096	5.44267	-2.57919
H	-0.92628	5.30259	-4.83138
H	-2.30444	4.32019	-5.43833
C	3.93944	-1.90268	1.43955
C	3.54073	-3.09211	0.53856
C	5.10555	-2.29187	2.36796
H	3.05940	-1.70513	2.08669
C	3.19361	-4.32308	1.38437
H	4.36085	-3.36795	-0.15670
H	2.65684	-2.81027	-0.07288
C	4.73969	-3.51590	3.21621
H	6.01751	-2.51951	1.77377
H	5.34255	-1.44372	3.04587
C	4.34387	-4.69899	2.32567
H	2.97912	-5.18463	0.71920

H	2.28245	-4.11091	1.97637
H	5.61191	-3.80331	3.84175
H	3.89760	-3.26249	3.89801
H	4.02795	-5.54994	2.96678
H	5.22256	-5.02698	1.72782
C	6.67644	0.03599	-2.35134
C	6.03191	0.22698	-3.73844
C	7.87084	0.99301	-2.16635
H	7.07756	-1.00332	-2.31489
C	7.06760	0.03725	-4.85358
H	5.58709	1.24198	-3.82821
H	5.21763	-0.51659	-3.87301
C	8.90169	0.80159	-3.28663
H	7.53070	2.05130	-2.16887
H	8.35914	0.79245	-1.18781
C	8.25717	0.98767	-4.66646
H	6.58895	0.23721	-5.83634
H	7.42632	-1.01564	-4.85616
H	9.72077	1.54215	-3.16185
H	9.34676	-0.21535	-3.21620
H	9.01420	0.78667	-5.45465
H	7.91554	2.04012	-4.78003
C	3.41990	3.08537	0.08484
C	4.46954	4.16240	0.42377
C	2.59999	3.50420	-1.15486
H	2.72230	3.07513	0.94372
C	3.79066	5.52045	0.65678
H	5.21031	4.26566	-0.39841
H	5.01735	3.86945	1.34594
C	1.91732	4.85742	-0.91870
H	3.25452	3.58222	-2.04988
H	1.82147	2.74402	-1.37329
C	2.94826	5.93571	-0.55794
H	4.56819	6.29176	0.84442
H	3.14096	5.46471	1.55832
H	1.37495	5.16113	-1.83993
H	1.17029	4.76272	-0.10001
H	2.41875	6.88536	-0.32870
H	3.61605	6.11763	-1.42859

Catalyst (2,4,6-c-Hex, 11)-Enamine protonation TS (pro-S) alternative conformation
Electronic energy (Hartrees): -1870.489131
Free Energy at 1M concentration and 253 K (Hartrees): -1868.838771
Coordinates:

P	-0.25130	-0.25983	0.17333
O	-1.55646	0.64404	0.58863
O	0.73601	0.69850	-0.70479
O	-0.58034	-1.47794	-0.62784
O	0.44515	-0.49129	1.54169
C	-1.89478	1.68301	-0.24232
C	-3.24011	1.67809	-0.86340
C	-3.65663	2.72985	-1.58137
H	-4.63674	2.71856	-2.04401
C	-2.75303	3.87022	-1.82326
C	-3.18316	4.96462	-2.72376
H	-4.18150	4.96304	-3.14340
C	-2.33778	5.95341	-3.03178
H	-2.65107	6.74943	-3.69483
C	-0.96909	5.95294	-2.48322

H	-0.27922	6.73922	-2.76157
C	-0.57480	4.97638	-1.65847
H	0.44517	4.98339	-1.30025
C	-1.51331	3.88214	-1.28038
C	-1.09172	2.76476	-0.37834
C	1.13108	1.82384	-0.02729
C	2.57373	2.04404	0.21959
C	2.97162	3.10625	0.93746
H	4.02665	3.29464	1.09542
C	1.97174	4.04759	1.48635
C	2.40108	5.14345	2.38437
H	3.44676	5.25788	2.64139
C	1.49729	5.99173	2.88787
H	1.81075	6.78952	3.54863
C	0.06844	5.83995	2.54885
H	-0.65839	6.52269	2.96947
C	-0.32865	4.85857	1.73052
H	-1.38174	4.75192	1.50439
C	0.66547	3.91485	1.15699
C	0.24327	2.79201	0.27246
C	3.56296	1.17867	-0.46540
C	3.68551	1.26127	-1.96584
C	4.32834	0.29300	0.23072
C	4.54494	0.43838	-2.59531
C	5.30061	-0.55304	-0.50879
C	5.39073	-0.51237	-1.84658
H	4.63997	0.47221	-3.67279
H	5.93616	-1.22960	0.04444
C	-4.08671	0.47012	-0.76495
C	-4.80118	0.20527	0.36275
C	-4.15402	-0.46549	-1.93996
C	-5.64025	-1.02039	0.40920
C	-4.96683	-1.53554	-1.87193
C	-5.72925	-1.84809	-0.64230
H	-6.19191	-1.24573	1.31255
H	-5.06318	-2.19754	-2.72079
H	-0.16413	-2.89154	0.42056
C	1.49760	-4.08808	0.31426
C	0.41428	-3.35119	2.34918
C	1.53264	-4.04310	-1.09362
C	2.52489	-4.73699	1.04514
C	1.44703	-3.99485	3.09934
C	2.57899	-4.65785	-1.75543
H	0.75976	-3.49586	-1.62309
C	3.57337	-5.36260	0.33411
C	2.44662	-4.68149	2.46869
H	1.42078	-3.92076	4.17720
C	3.59968	-5.32625	-1.04614
H	2.61991	-4.61641	-2.83947
H	4.36154	-5.86048	0.89164
H	3.21144	-5.18491	3.05411
H	4.41112	-5.80015	-1.58880
C	-0.56602	-2.46721	2.92056
C	-0.70748	-2.30553	4.43600
H	-0.78698	-3.27699	4.94260
H	-1.65789	-1.78709	4.61654
N	0.41023	-1.59090	5.04114
N	0.47570	-3.47230	0.99830
C	0.48057	-0.19001	5.07560
C	1.45050	0.42543	5.89097

C	-0.34966	0.62996	4.29469
C	1.60137	1.80790	5.89827
H	2.10458	-0.19460	6.50073
C	-0.18866	2.01487	4.31083
H	-1.09229	0.19703	3.64068
C	0.78541	2.61843	5.10372
H	2.36559	2.25422	6.52873
H	-0.83772	2.62035	3.68462
H	0.90906	3.69694	5.10454
H	0.77501	-2.02668	5.87573
H	-0.02533	-1.28626	2.24999
Cl	-2.20760	-2.64398	2.15500
C	-4.78688	1.12477	1.58556
C	-6.19203	1.68461	1.88294
C	-4.22175	0.39730	2.82085
H	-4.13379	2.00510	1.41178
C	-6.15952	2.61552	3.10346
H	-6.91207	0.86108	2.07900
H	-6.55656	2.25664	1.00191
C	-4.18516	1.33248	4.03546
H	-4.83490	-0.49495	3.07139
H	-3.19181	0.05202	2.60331
C	-5.57995	1.89910	4.33106
H	-7.19203	2.95694	3.33193
H	-5.54617	3.51426	2.87221
H	-3.82015	0.76932	4.92129
H	-3.47432	2.16661	3.84510
H	-5.51182	2.61720	5.17631
H	-6.26034	1.07394	4.63666
C	-6.58258	-3.10126	-0.56266
C	-7.70971	-3.07330	-1.61435
C	-5.71637	-4.36703	-0.72149
H	-7.06761	-3.15668	0.43935
C	-8.56552	-4.34402	-1.53194
H	-7.28849	-2.99060	-2.63977
H	-8.35943	-2.18827	-1.43867
C	-6.57876	-5.63331	-0.64246
H	-5.18175	-4.35651	-1.69633
H	-4.95321	-4.39857	0.08679
C	-7.70066	-5.60140	-1.68820
H	-9.33119	-4.32350	-2.33710
H	-9.09717	-4.37488	-0.55539
H	-5.93870	-6.52452	-0.81857
H	-7.01859	-5.72394	0.37517
H	-8.33938	-6.50274	-1.56804
H	-7.26023	-5.62729	-2.70932
C	-3.34329	-0.20491	-3.21148
C	-2.48246	-1.41648	-3.63088
C	-4.25718	0.22846	-4.37289
H	-2.62818	0.62426	-3.03433
C	-1.64347	-1.07661	-4.86968
H	-3.11733	-2.29715	-3.86573
H	-1.80289	-1.70016	-2.80354
C	-3.42612	0.56124	-5.62032
H	-4.98121	-0.57943	-4.61829
H	-4.83616	1.12982	-4.07645
C	-2.54026	-0.62363	-6.02987
H	-1.06668	-1.97419	-5.18037
H	-0.91567	-0.27259	-4.62167
H	-4.10928	0.81531	-6.45903

H	-2.78855	1.45036	-5.41789
H	-1.90303	-0.32441	-6.88965
H	-3.18145	-1.47136	-6.35760
C	4.22797	0.12186	1.74669
C	3.95000	-1.34548	2.14164
C	5.50837	0.63556	2.43221
H	3.38050	0.71098	2.15532
C	3.86638	-1.49273	3.66587
H	4.74588	-2.02441	1.77082
H	2.98805	-1.67182	1.69139
C	5.40380	0.49446	3.95544
H	6.39605	0.06976	2.07354
H	5.66218	1.70726	2.18120
C	5.13039	-0.96070	4.35111
H	3.74024	-2.56240	3.93099
H	2.98109	-0.94049	4.03638
H	6.35551	0.82874	4.42149
H	4.58719	1.14628	4.33813
H	5.00195	-1.02232	5.45322
H	6.00080	-1.59392	4.07079
C	6.33094	-1.43184	-2.60516
C	7.80308	-1.19077	-2.21197
C	5.94123	-2.91069	-2.40454
H	6.24034	-1.21195	-3.69313
C	8.73649	-2.12471	-2.99304
H	7.95370	-1.36329	-1.12427
H	8.07841	-0.13649	-2.43303
C	6.88437	-3.83751	-3.18122
H	5.97487	-3.18330	-1.32696
H	4.90250	-3.06990	-2.76632
C	8.34583	-3.59359	-2.78470
H	9.78164	-1.97105	-2.64815
H	8.69405	-1.87648	-4.07645
H	6.61757	-4.89480	-2.96700
H	6.75993	-3.66803	-4.27341
H	9.00571	-4.23945	-3.40288
H	8.49497	-3.87244	-1.71825
C	2.86234	2.25674	-2.78833
C	3.75536	3.25543	-3.55084
C	1.90399	1.53821	-3.76284
H	2.23541	2.87598	-2.11903
C	2.89391	4.27359	-4.31299
H	4.41286	2.72618	-4.27392
H	4.40386	3.79901	-2.82954
C	1.04037	2.55504	-4.51999
H	2.47535	0.93198	-4.49873
H	1.23754	0.84856	-3.20425
C	1.91639	3.57078	-5.26600
H	3.55505	4.94817	-4.89826
H	2.32392	4.89803	-3.58970
H	0.39638	2.01894	-5.25007
H	0.37401	3.08654	-3.80556
H	1.26469	4.33101	-5.74781
H	2.48703	3.05174	-6.06726

Catalyst (2,4,6-c-Hex, 11)-Enamine protonation TS (pro-S) alternative conformation
Electronic energy (Hartrees): -1870.48426
Free Energy at 1M concentration and 253 K (Hartrees): -1868.831772
Coordinates:

P	-0.21731	-0.31127	0.07383
O	1.11595	0.56757	-0.31620
O	-1.34288	0.71745	0.68839
O	0.09362	-1.21581	1.29385
O	-0.72241	-1.01169	-1.15786
C	1.52624	1.47192	0.64082
C	2.93148	1.44635	1.14455
C	3.32178	2.32882	2.08063
H	4.35004	2.34420	2.42252
C	2.37323	3.31508	2.62533
C	2.78000	4.19804	3.74254
H	3.77772	4.12617	4.15745
C	1.91421	5.08479	4.24635
H	2.21001	5.72800	5.06503
C	0.54906	5.18240	3.69363
H	-0.15354	5.88748	4.11884
C	0.17733	4.39520	2.67752
H	-0.83470	4.46636	2.30011
C	1.13839	3.42093	2.09293
C	0.74674	2.52524	0.96717
C	-1.44873	1.94072	0.06931
C	-2.76802	2.37957	-0.45896
C	-2.92684	3.62982	-0.92444
H	-3.89829	3.96415	-1.27018
C	-1.77306	4.54323	-1.01251
C	-1.92137	5.86269	-1.66846
H	-2.88484	6.17420	-2.05243
C	-0.86367	6.67183	-1.79565
H	-0.97115	7.63441	-2.27876
C	0.45636	6.24987	-1.28798
H	1.31293	6.89965	-1.41319
C	0.59694	5.06427	-0.68416
H	1.57754	4.76462	-0.33814
C	-0.57591	4.16368	-0.51563
C	-0.44576	2.83558	0.15271
C	-3.93375	1.47657	-0.36168
C	-4.52738	1.20770	0.99334
C	-4.47921	0.90706	-1.47365
C	-5.56033	0.35343	1.09197
C	-5.59877	-0.05768	-1.30535
C	-6.10493	-0.33971	-0.09455
H	-6.02112	0.17364	2.05305
H	-6.01589	-0.53670	-2.18131
C	3.96291	0.58083	0.52506
C	4.46443	0.87498	-0.70901
C	4.61699	-0.48140	1.36349
C	5.64896	0.12226	-1.20406
C	5.72339	-1.09283	0.89905
C	6.29179	-0.76156	-0.42591
H	6.05321	0.36346	-2.17842
H	6.22756	-1.83128	1.50636
H	0.20200	-2.35007	1.01432
C	0.23763	-3.73145	0.54834
C	0.96569	-3.68385	-0.79933
H	0.28690	-3.26157	-1.53741
H	1.19276	-4.71760	-1.11756
N	2.16325	-2.85122	-0.78698
H	2.86730	-3.25488	-0.17866
C	2.72166	-2.51194	-2.03807
C	4.08096	-2.74801	-2.28179

C	1.95015	-1.90479	-3.04467
C	4.66743	-2.37750	-3.49084
H	4.66446	-3.23341	-1.50478
C	2.53788	-1.56470	-4.26054
H	0.91313	-1.65386	-2.84879
C	3.89661	-1.79221	-4.49472
H	5.72576	-2.56022	-3.65424
H	1.92986	-1.08827	-5.02508
H	4.34755	-1.50975	-5.44105
C	-1.13894	-4.14303	0.47197
C	-1.75684	-5.18145	1.23943
C	-3.22647	-3.85077	-0.71813
C	-3.03733	-5.57623	0.97804
H	-1.15898	-5.67949	1.98922
C	-3.93909	-3.17275	-1.72020
C	-3.81087	-4.95437	-0.04769
H	-3.47365	-6.40030	1.53617
C	-5.19864	-3.61376	-2.08078
H	-3.46746	-2.33091	-2.20388
C	-5.10137	-5.37970	-0.43393
C	-5.78237	-4.72829	-1.44522
H	-5.74033	-3.09615	-2.86605
H	-5.54660	-6.22912	0.07586
H	-6.76983	-5.06423	-1.74341
N	-1.93746	-3.47564	-0.40735
H	-1.55720	-2.60180	-0.82844
Cl	1.20528	-4.71203	1.73921
C	-4.02809	1.93045	2.24627
C	-5.11604	2.84314	2.84757
C	-3.50294	0.94131	3.30832
H	-3.18222	2.59795	1.98927
C	-4.57599	3.59453	4.07266
H	-6.00533	2.24980	3.15203
H	-5.44172	3.58370	2.08471
C	-2.95931	1.69751	4.52744
H	-4.31152	0.25570	3.64224
H	-2.68927	0.32012	2.87806
C	-4.03185	2.61841	5.12477
H	-5.39291	4.19945	4.52155
H	-3.76811	4.29174	3.75801
H	-2.63346	0.96593	5.29779
H	-2.07160	2.29912	4.23026
H	-3.59157	3.19522	5.96634
H	-4.86622	2.00457	5.53000
C	-4.02091	1.25448	-2.89569
C	-3.50592	0.02168	-3.66085
C	-5.15734	1.94426	-3.67755
H	-3.17137	1.96469	-2.87974
C	-3.05403	0.40222	-5.07712
H	-4.29865	-0.75223	-3.73887
H	-2.63500	-0.40441	-3.11917
C	-4.70096	2.32101	-5.09303
H	-6.04347	1.27694	-3.75074
H	-5.46822	2.86797	-3.14248
C	-4.18557	1.09080	-5.85003
H	-2.74350	-0.51547	-5.62155
H	-2.17339	1.07961	-5.02064
H	-5.55604	2.76205	-5.64904
H	-3.89798	3.08873	-5.03563
H	-3.80992	1.40489	-6.84752

H	-5.02029	0.37287	-6.00844
C	4.07663	-0.84542	2.74640
C	3.65485	-2.32361	2.81759
C	5.09352	-0.51164	3.85846
H	3.15908	-0.25952	2.95984
C	3.09492	-2.66972	4.20377
H	4.51147	-2.99553	2.59440
H	2.86649	-2.50026	2.06023
C	4.52395	-0.85766	5.23994
H	6.03944	-1.07599	3.70974
H	5.33608	0.57222	3.83087
C	4.10502	-2.33164	5.30725
H	2.85907	-3.75472	4.24410
H	2.15007	-2.10854	4.37643
H	5.29535	-0.65992	6.01498
H	3.64693	-0.20863	5.45682
H	3.64724	-2.53763	6.29857
H	5.00265	-2.98016	5.20278
C	3.94622	2.04041	-1.55880
C	5.04146	3.09891	-1.80211
C	3.36453	1.54085	-2.89237
H	3.12660	2.57850	-1.04622
C	4.49177	4.26730	-2.63215
H	5.90914	2.65908	-2.33842
H	5.40285	3.48822	-0.82527
C	2.82020	2.70999	-3.72338
H	4.13921	1.00407	-3.48112
H	2.53166	0.83732	-2.68545
C	3.90364	3.77019	-3.95940
H	5.31222	4.98694	-2.84129
H	3.70726	4.80301	-2.05320
H	2.46102	2.32694	-4.70273
H	1.95411	3.17110	-3.19831
H	3.46172	4.62912	-4.50866
H	4.71399	3.34095	-4.58888
C	7.64508	-1.31512	-0.84795
C	8.75049	-0.76538	0.07698
C	7.66890	-2.85823	-0.86416
H	7.87767	-0.97289	-1.88278
C	10.12932	-1.29852	-0.33033
H	8.55065	-1.05101	1.13330
H	8.76393	0.34515	0.02025
C	9.05402	-3.38448	-1.26285
H	7.40133	-3.27031	0.13217
H	6.93509	-3.23537	-1.60226
C	10.14341	-2.83114	-0.33736
H	10.89257	-0.92823	0.38748
H	10.39553	-0.91655	-1.34051
H	9.05479	-4.49438	-1.20658
H	9.27532	-3.09598	-2.31415
H	11.13661	-3.18716	-0.68610
H	9.98564	-3.21194	0.69580
C	-7.24377	-1.33209	0.05451
C	-6.89881	-2.43751	1.07407
C	-8.54458	-0.61604	0.46366
H	-7.42925	-1.82853	-0.92592
C	-8.04839	-3.44576	1.18704
H	-6.71159	-2.00560	2.08028
H	-5.97347	-2.96184	0.76170
C	-9.69970	-1.61888	0.59524

H	-8.40854	-0.08700	1.43262
H	-8.81047	0.14093	-0.30619
C	-9.35231	-2.74072	1.58377
H	-7.79399	-4.20852	1.95404
H	-8.18735	-3.96985	0.21642
H	-10.60862	-1.08679	0.94956
H	-9.92779	-2.05809	-0.40092
H	-10.17930	-3.48268	1.60163
H	-9.25063	-2.31692	2.60714

Catalyst (2,4,6-c-Hex, 11)-Enamine protonation TS (pro-R) lowest free energy conformation
Electronic energy (Hartrees): -1870.499174
Free Energy at 1M concentration and 253 K (Hartrees): -1868.847048
Coordinates:

P	0.05140	0.14798	0.01598
O	-1.20779	1.02503	0.57401
O	1.16179	1.18849	-0.55785
O	-0.37885	-0.58674	-1.28494
O	0.52141	-0.69795	1.15735
C	-1.58846	2.07851	-0.21787
C	-2.96720	2.09648	-0.76854
C	-3.37458	3.13659	-1.51069
H	-4.38440	3.16323	-1.90295
C	-2.45092	4.24759	-1.81323
C	-2.88167	5.33420	-2.72272
H	-3.88284	5.33321	-3.13555
C	-2.03441	6.31828	-3.04251
H	-2.34885	7.11039	-3.70970
C	-0.66314	6.31988	-2.49812
H	0.02543	7.10582	-2.78053
C	-0.26691	5.34707	-1.66941
H	0.75126	5.35445	-1.30364
C	-1.20779	4.26138	-1.27963
C	-0.78915	3.16110	-0.36041
C	1.41237	2.26506	0.25221
C	2.77479	2.43634	0.81291
C	3.07044	3.53260	1.52734
H	4.07226	3.67714	1.91388
C	2.02686	4.53672	1.81742
C	2.33643	5.68401	2.70127
H	3.33393	5.80611	3.10485
C	1.38433	6.57153	3.00866
H	1.61068	7.40833	3.65675
C	0.01825	6.40557	2.47631
H	-0.75317	7.11467	2.74769
C	-0.27020	5.37627	1.67156
H	-1.28429	5.26059	1.31260
C	0.78537	4.39657	1.29644
C	0.49083	3.24185	0.39578
C	3.82045	1.42731	0.51816
C	4.37464	1.33987	-0.87824
C	4.27054	0.57527	1.48337
C	5.28624	0.39080	-1.15951
C	5.31430	-0.42270	1.13036
C	5.77540	-0.54221	-0.12262
H	5.69907	0.31537	-2.15592
H	5.70612	-1.06953	1.90272
C	-3.91089	0.98743	-0.48122
C	-4.50070	0.86476	0.74145

C	-4.30804	0.06231	-1.59665
C	-5.57072	-0.15205	0.92564
C	-5.34304	-0.77715	-1.41161
C	-6.00922	-0.89916	-0.09751
H	-6.03627	-0.25639	1.89694
H	-5.67934	-1.40987	-2.22083
H	-0.26027	-2.24928	1.20701
C	-1.96602	-3.08522	1.97971
C	-1.01290	-3.54573	-0.20973
C	-1.86052	-2.37810	3.19254
C	-3.09143	-3.90197	1.71445
C	-2.16871	-4.34604	-0.50121
C	-2.86976	-2.49836	4.13114
H	-1.00341	-1.73349	3.35905
C	-4.09184	-4.01919	2.70350
C	-3.14609	-4.53239	0.43132
H	-2.22643	-4.81564	-1.47299
C	-3.98636	-3.32484	3.89433
H	-2.79903	-1.94413	5.06199
H	-4.94903	-4.65851	2.51239
H	-3.99323	-5.17256	0.19958
H	-4.76325	-3.41198	4.64674
N	-0.97504	-2.97752	1.02978
C	0.02588	-3.20724	-1.13046
C	1.47879	-3.18552	-0.68531
H	1.54570	-2.62855	0.24947
H	2.06713	-2.61430	-1.42049
N	2.01341	-4.53352	-0.48311
H	1.85755	-5.11646	-1.29715
C	3.27823	-4.73152	0.07968
C	4.03341	-5.85934	-0.29779
C	3.81194	-3.87436	1.05996
C	5.26474	-6.12559	0.29146
H	3.64171	-6.52713	-1.06194
C	5.04541	-4.15467	1.64572
H	3.26598	-2.99074	1.36738
C	5.78438	-5.27624	1.27094
H	5.82584	-7.00081	-0.02479
H	5.43401	-3.48276	2.40546
H	6.74805	-5.48091	1.72626
H	-0.24755	-1.68672	-1.28796
Cl	-0.12921	-3.97707	-2.76532
C	-3.54773	0.04549	-2.92017
C	-3.03003	-1.36654	-3.27336
C	-4.40821	0.60015	-4.07125
H	-2.64982	0.69346	-2.83643
C	-2.21463	-1.33034	-4.57141
H	-3.87139	-2.07923	-3.40832
H	-2.39684	-1.75320	-2.44964
C	-3.60539	0.63001	-5.37955
H	-5.31669	-0.02532	-4.21348
H	-4.73858	1.63294	-3.82952
C	-3.05185	-0.75991	-5.72470
H	-1.88993	-2.36092	-4.83013
H	-1.30240	-0.71076	-4.42526
H	-4.26270	0.97802	-6.20512
H	-2.76492	1.35261	-5.28490
H	-2.41899	-0.68647	-6.63507
H	-3.89478	-1.45003	-5.94916
C	-7.16588	-1.86430	0.09344

C	-8.35895	-1.47738	-0.80282
C	-6.72935	-3.31637	-0.17962
H	-7.51686	-1.81745	1.15031
C	-9.52753	-2.45273	-0.60909
H	-8.06074	-1.48067	-1.87401
H	-8.69936	-0.45070	-0.54522
C	-7.90065	-4.28665	0.01546
H	-6.34005	-3.42561	-1.21494
H	-5.91658	-3.59008	0.52148
C	-9.08856	-3.89866	-0.87399
H	-10.34857	-2.18501	-1.30845
H	-9.91987	-2.36546	0.42805
H	-7.57041	-5.31621	-0.24122
H	-8.21516	-4.28452	1.08240
H	-9.93922	-4.58349	-0.66870
H	-8.80688	-4.01324	-1.94395
C	-4.14988	1.76321	1.92859
C	-5.36891	2.58281	2.39752
C	-3.57033	0.93396	3.09202
H	-3.37471	2.50502	1.65256
C	-4.99393	3.48381	3.58227
H	-6.20172	1.91548	2.70651
H	-5.73127	3.21856	1.56035
C	-3.19896	1.83726	4.27427
H	-4.30267	0.17241	3.43689
H	-2.65878	0.39991	2.74944
C	-4.40797	2.66057	4.73736
H	-5.90048	4.01968	3.93700
H	-4.25328	4.24608	3.25363
H	-2.83818	1.20822	5.11640
H	-2.37181	2.51995	3.97840
H	-4.09315	3.34627	5.55309
H	-5.18846	1.98026	5.14387
C	3.75597	0.60720	2.92530
C	3.23895	-0.77061	3.39159
C	4.85251	1.11403	3.88176
H	2.89469	1.30029	3.01883
C	2.70357	-0.69156	4.82661
H	4.04588	-1.53185	3.36252
H	2.42501	-1.11295	2.72327
C	4.32847	1.18678	5.32276
H	5.73756	0.44141	3.84921
H	5.18266	2.12794	3.56845
C	3.78421	-0.17203	5.78407
H	2.37896	-1.70294	5.15340
H	1.81684	-0.02074	4.86084
H	5.15399	1.49920	5.99780
H	3.52454	1.95274	5.38941
H	3.35198	-0.06727	6.80236
H	4.61718	-0.90709	5.83972
C	3.95528	2.32416	-1.97133
C	5.14522	3.18121	-2.44902
C	3.29131	1.60584	-3.16576
H	3.20774	3.03833	-1.57308
C	4.69324	4.19204	-3.51238
H	5.94576	2.54067	-2.87854
H	5.57435	3.73404	-1.58493
C	2.83617	2.62147	-4.22136
H	3.99850	0.89054	-3.63844
H	2.40904	1.02803	-2.81857

C	4.01291	3.48611	-4.69329
H	5.57684	4.75555	-3.88186
H	3.98786	4.92265	-3.05827
H	2.40707	2.07931	-5.09131
H	2.03916	3.27167	-3.79692
H	3.64196	4.24867	-5.41137
H	4.75445	2.84880	-5.22352
C	6.80098	-1.60763	-0.46721
C	6.27074	-2.55656	-1.56159
C	8.13532	-0.97141	-0.90238
H	7.00883	-2.22673	0.43568
C	7.30652	-3.63799	-1.89211
H	6.03369	-1.99297	-2.48979
H	5.33576	-3.04412	-1.21559
C	9.16963	-2.05324	-1.24198
H	7.98702	-0.32127	-1.79212
H	8.53170	-0.33921	-0.07808
C	8.63813	-3.00768	-2.31963
H	6.92045	-4.27679	-2.71532
H	7.46904	-4.28678	-1.00393
H	10.10028	-1.56885	-1.60814
H	9.42247	-2.62979	-0.32494
H	9.38395	-3.81207	-2.49696
H	8.49922	-2.45295	-3.27368

Catalyst (2,4,6-c-Hex, 11)-Enamine protonation TS (pro-R) alternative conformation
Electronic energy (Hartrees): -1870.499031
Free Energy at 1M concentration and 253 K (Hartrees): -1868.846947
Coordinates:

P	0.04931	0.14349	-0.05322
O	-1.25226	0.95763	0.50129
O	1.17339	1.24020	-0.47527
O	-0.29542	-0.48517	-1.43317
O	0.46758	-0.78897	1.03943
C	-1.60206	2.07175	-0.21852
C	-2.94708	2.12329	-0.84565
C	-3.32841	3.22140	-1.51448
H	-4.31375	3.27110	-1.96280
C	-2.40704	4.36496	-1.66350
C	-2.80310	5.52361	-2.49668
H	-3.77977	5.54917	-2.96386
C	-1.95433	6.54065	-2.68029
H	-2.24327	7.38564	-3.29183
C	-0.61536	6.50758	-2.06159
H	0.07600	7.32224	-2.23463
C	-0.25098	5.46906	-1.30078
H	0.74535	5.45404	-0.87936
C	-1.19572	4.34379	-1.06145
C	-0.81241	3.17080	-0.21988
C	1.36416	2.24514	0.43671
C	2.69098	2.37946	1.08586
C	2.93172	3.41289	1.90661
H	3.90852	3.53248	2.35989
C	1.86005	4.37886	2.22250
C	2.10482	5.44862	3.21698
H	3.07696	5.54466	3.68444
C	1.12511	6.29767	3.54549
H	1.30402	7.07765	4.27426
C	-0.20737	6.16557	2.92571

H	-1.00227	6.84160	3.21327
C	-0.43705	5.20667	2.02126
H	-1.42830	5.11296	1.59789
C	0.65102	4.27268	1.62308
C	0.42246	3.19738	0.61167
C	3.76470	1.40785	0.76699
C	4.39813	1.44442	-0.59763
C	4.17164	0.48123	1.68135
C	5.33887	0.53234	-0.90425
C	5.24903	-0.47216	1.30684
C	5.78282	-0.47980	0.07725
H	5.80858	0.54576	-1.87797
H	5.60629	-1.17786	2.04358
C	-3.88424	0.97864	-0.72279
C	-4.53802	0.72620	0.44615
C	-4.20076	0.16269	-1.94480
C	-5.59971	-0.31437	0.46565
C	-5.23273	-0.70051	-1.90495
C	-5.97248	-0.95493	-0.65316
H	-6.11229	-0.52420	1.39412
H	-5.50805	-1.26278	-2.78726
H	-0.27610	-2.35498	0.92428
C	-1.98362	-3.30348	1.55216
C	-0.93320	-3.54257	-0.62836
C	-1.94807	-2.70539	2.82619
C	-3.07100	-4.12583	1.17077
C	-2.05594	-4.33813	-1.03857
C	-2.98649	-2.94150	3.70951
H	-1.12059	-2.05233	3.08420
C	-4.09981	-4.36698	2.10615
C	-3.06198	-4.63355	-0.16658
H	-2.06391	-4.71724	-2.05072
C	-4.06295	-3.78010	3.35729
H	-2.96962	-2.47095	4.68773
H	-4.92577	-5.01340	1.82328
H	-3.88389	-5.26741	-0.48897
H	-4.86293	-3.96057	4.06769
N	-0.96178	-3.08335	0.65612
C	0.13791	-3.10785	-1.46731
C	1.56773	-3.10316	-0.95158
H	1.57958	-2.62848	0.02987
H	2.18018	-2.46238	-1.60516
N	2.11599	-4.45596	-0.83892
H	2.01451	-4.96831	-1.70709
C	3.34805	-4.69027	-0.22095
C	4.13890	-5.77569	-0.64692
C	3.81199	-3.91571	0.85857
C	5.33680	-6.08132	-0.00987
H	3.80128	-6.37895	-1.48680
C	5.01236	-4.23519	1.49115
H	3.23761	-3.06580	1.20637
C	5.78669	-5.31466	1.06752
H	5.92692	-6.92181	-0.36509
H	5.34674	-3.62764	2.32708
H	6.72435	-5.54994	1.56076
H	-0.15323	-1.57775	-1.51407
Cl	0.07350	-3.73590	-3.16746
C	-3.35786	0.27753	-3.21233
C	-2.79605	-1.08975	-3.66098
C	-4.15140	0.92932	-4.36052

H	-2.47746	0.92355	-3.01052
C	-1.89875	-0.92310	-4.89320
H	-3.61579	-1.79417	-3.91756
H	-2.21168	-1.54675	-2.83705
C	-3.26644	1.09042	-5.60483
H	-5.03846	0.31067	-4.61970
H	-4.51344	1.93135	-4.04575
C	-2.66870	-0.25498	-6.04099
H	-1.54096	-1.92137	-5.22484
H	-1.00819	-0.31072	-4.62983
H	-3.87482	1.50770	-6.43581
H	-2.44591	1.80969	-5.38820
H	-1.97939	-0.08941	-6.89666
H	-3.48378	-0.92952	-6.38429
C	-7.11451	-1.95546	-0.65325
C	-6.79028	-3.17174	0.23440
C	-8.44288	-1.29782	-0.22463
H	-7.25459	-2.33326	-1.69153
C	-7.94238	-4.18374	0.22521
H	-6.59828	-2.85274	1.28208
H	-5.87523	-3.66850	-0.15108
C	-9.58748	-2.31916	-0.23622
H	-8.35741	-0.86840	0.79724
H	-8.68999	-0.46990	-0.92442
C	-9.25855	-3.52381	0.65471
H	-7.70410	-5.01577	0.92235
H	-8.05488	-4.61349	-0.79453
H	-10.51640	-1.83333	0.13264
H	-9.77196	-2.66338	-1.27773
H	-10.08133	-4.26734	0.58415
H	-9.18324	-3.19542	1.71485
C	-4.26741	1.50015	1.73709
C	-5.52399	2.25276	2.21940
C	-3.73900	0.55967	2.83911
H	-3.49097	2.27511	1.58259
C	-5.22979	3.02838	3.51088
H	-6.36149	1.54785	2.40973
H	-5.85037	2.96887	1.43391
C	-3.44817	1.33803	4.12788
H	-4.47623	-0.24184	3.06147
H	-2.80161	0.07348	2.49506
C	-4.69453	2.09487	4.60519
H	-6.16330	3.51417	3.86781
H	-4.48502	3.82892	3.30578
H	-3.12361	0.62815	4.91897
H	-2.61759	2.05698	3.95164
H	-4.43666	2.69453	5.50444
H	-5.48469	1.36712	4.89382
C	3.57511	0.38511	3.08847
C	3.05435	-1.03289	3.40672
C	4.60691	0.82106	4.14650
H	2.69889	1.05818	3.18936
C	2.43599	-1.08120	4.80937
H	3.87383	-1.77982	3.36166
H	2.28596	-1.32689	2.66550
C	3.99989	0.76615	5.55537
H	5.50275	0.16311	4.10953
H	4.93869	1.86128	3.93911
C	3.45134	-0.63256	5.86871
H	2.10916	-2.12006	5.03075

H	1.53821	-0.42544	4.84746
H	4.78027	1.02931	6.30138
H	3.18157	1.51505	5.63872
H	2.95956	-0.61876	6.86504
H	4.29118	-1.36063	5.91158
C	4.02545	2.51227	-1.62733
C	5.22676	3.41922	-1.96348
C	3.44076	1.88925	-2.91322
H	3.24572	3.18240	-1.21458
C	4.81939	4.51046	-2.96349
H	6.06007	2.82567	-2.39813
H	5.59803	3.90246	-1.03340
C	3.02976	2.98460	-3.90529
H	4.18440	1.22371	-3.40237
H	2.54954	1.27484	-2.66614
C	4.21749	3.89842	-4.23584
H	5.71340	5.11251	-3.23384
H	4.07830	5.19280	-2.49148
H	2.65864	2.51248	-4.84030
H	2.20016	3.58833	-3.47470
H	3.87558	4.71428	-4.90829
H	4.99747	3.31577	-4.77375
C	6.84347	-1.50056	-0.29526
C	6.39274	-2.35845	-1.49533
C	8.19034	-0.81483	-0.59577
H	7.00876	-2.19210	0.56272
C	7.46298	-3.39646	-1.85405
H	6.20112	-1.72044	-2.38505
H	5.44710	-2.88379	-1.24778
C	9.25979	-1.85232	-0.96377
H	8.08323	-0.09299	-1.43471
H	8.52818	-0.25073	0.30084
C	8.80691	-2.71750	-2.14749
H	7.13547	-3.96707	-2.74955
H	7.58403	-4.11665	-1.01566
H	10.20216	-1.32823	-1.23229
H	9.46842	-2.50194	-0.08529
H	9.57450	-3.49565	-2.34755
H	8.71471	-2.08522	-3.05796

Catalyst (2,4,6-c-Hex, 11)-Enamine protonation TS (pro-R) alternative conformation
Electronic energy (Hartrees): -1870.496679
Free Energy at 1M concentration and 253 K (Hartrees): -1868.845238
Coordinates:

P	0.06130	0.15661	0.04696
O	-1.19106	0.97130	0.70572
O	1.14212	1.25036	-0.48623
O	-0.39920	-0.48398	-1.29328
O	0.56965	-0.77033	1.10612
C	-1.60433	2.07923	0.01081
C	-2.99510	2.12191	-0.50772
C	-3.43135	3.21245	-1.15526
H	-4.44964	3.25620	-1.52336
C	-2.52672	4.35471	-1.39153
C	-2.98769	5.50028	-2.20929
H	-3.99859	5.51984	-2.59721
C	-2.15543	6.51199	-2.47832
H	-2.49168	7.34688	-3.07949
C	-0.77101	6.48640	-1.96902

H	-0.09446	7.29548	-2.21259
C	-0.34810	5.46104	-1.22089
H	0.67947	5.45062	-0.88256
C	-1.27155	4.34182	-0.88727
C	-0.82084	3.17920	-0.06503
C	1.41478	2.27498	0.38288
C	2.81060	2.46250	0.84683
C	3.12093	3.50131	1.63680
H	4.14607	3.66031	1.95049
C	2.06749	4.43534	2.08413
C	2.39924	5.51666	3.04002
H	3.41527	5.63575	3.39535
C	1.44469	6.34864	3.47100
H	1.68741	7.13746	4.17125
C	0.05482	6.18733	3.00189
H	-0.71635	6.85152	3.37029
C	-0.25418	5.21771	2.13311
H	-1.28313	5.10338	1.81818
C	0.80133	4.30083	1.62450
C	0.48266	3.21365	0.65159
C	3.87132	1.57022	0.32507
C	4.26676	1.68788	-1.12223
C	4.44850	0.62127	1.11413
C	5.15059	0.80929	-1.62976
C	5.43950	-0.30827	0.51006
C	5.75622	-0.24535	-0.79264
H	5.44690	0.87479	-2.66852
H	5.89660	-1.06596	1.13046
C	-3.91709	0.97819	-0.29699
C	-4.48436	0.74722	0.92044
C	-4.32245	0.14360	-1.47891
C	-5.53920	-0.29525	1.03672
C	-5.34581	-0.71988	-1.34827
C	-5.98765	-0.95931	-0.03819
H	-5.98686	-0.48570	2.00334
H	-5.68974	-1.28619	-2.20222
H	-0.21213	-2.33342	1.04644
C	-1.89535	-3.25793	1.77071
C	-0.96446	-3.52334	-0.46067
C	-1.78539	-2.65282	3.03699
C	-3.01137	-4.06876	1.45231
C	-2.11408	-4.31152	-0.80431
C	-2.77865	-2.86962	3.97552
H	-0.93693	-2.00943	3.24674
C	-3.99469	-4.28708	2.44108
C	-3.07571	-4.58985	0.12129
H	-2.17735	-4.69980	-1.81101
C	-3.88391	-3.69384	3.68499
H	-2.70360	-2.39433	4.94872
H	-4.84325	-4.92395	2.20783
H	-3.91707	-5.22113	-0.15216
H	-4.64780	-3.85903	4.43761
N	-0.91870	-3.05592	0.82084
C	0.06302	-3.10265	-1.35833
C	1.52246	-3.13091	-0.92830
H	1.61304	-2.65790	0.04928
H	2.10539	-2.50388	-1.62023
N	2.04828	-4.49745	-0.85720
H	2.00592	-4.95131	-1.76192
C	3.22628	-4.80066	-0.16516

C	4.09855	-5.78220	-0.67320
C	3.54251	-4.21101	1.07256
C	5.23585	-6.16341	0.03292
H	3.87560	-6.24423	-1.63241
C	4.68932	-4.59275	1.76574
H	2.87821	-3.47205	1.50492
C	5.54812	-5.56754	1.25596
H	5.89091	-6.92196	-0.38703
H	4.90934	-4.12594	2.72184
H	6.44149	-5.85692	1.80003
H	-0.23555	-1.57295	-1.38588
Cl	-0.10747	-3.72556	-3.05309
C	-3.58351	0.24541	-2.81074
C	-3.06107	-1.12694	-3.29091
C	-4.46626	0.88791	-3.89742
H	-2.68925	0.89187	-2.68576
C	-2.26545	-0.97267	-4.59258
H	-3.89933	-1.83232	-3.47475
H	-2.41318	-1.57798	-2.51263
C	-3.68378	1.03688	-5.20994
H	-5.37158	0.26804	-4.07903
H	-4.80154	1.89311	-3.56385
C	-3.12425	-0.31322	-5.68075
H	-1.93679	-1.97445	-4.94316
H	-1.35589	-0.35973	-4.40699
H	-4.35642	1.44795	-5.99309
H	-2.84785	1.75665	-5.06607
H	-2.50559	-0.15624	-6.59031
H	-3.96487	-0.98935	-5.95165
C	-7.13143	-1.94984	0.08984
C	-8.34480	-1.49967	-0.74762
C	-6.68744	-3.36827	-0.31595
H	-7.46239	-1.99808	1.15308
C	-9.50084	-2.50018	-0.61720
H	-8.06705	-1.40767	-1.82038
H	-8.68922	-0.50255	-0.39627
C	-7.84605	-4.36396	-0.18317
H	-6.31806	-3.38387	-1.36410
H	-5.85832	-3.69289	0.34289
C	-9.05444	-3.91341	-1.01360
H	-10.33745	-2.18187	-1.27571
H	-9.87396	-2.50642	0.43063
H	-7.51182	-5.36416	-0.53366
H	-8.13965	-4.45676	0.88567
H	-9.89489	-4.62215	-0.85215
H	-8.79268	-3.93277	-2.09452
C	-4.12519	1.55072	2.17133
C	-5.34643	2.31338	2.72329
C	-3.51716	0.63747	3.25441
H	-3.36305	2.32238	1.94500
C	-4.96406	3.11963	3.97214
H	-6.16638	1.61218	2.98867
H	-5.72923	3.01047	1.94620
C	-3.13900	1.44619	4.50127
H	-4.23419	-0.15996	3.54626
H	-2.60418	0.14642	2.85573
C	-4.35073	2.21259	5.04745
H	-5.87135	3.61287	4.38245
H	-4.23765	3.91578	3.69630
H	-2.75770	0.75541	5.28388

H	-2.32496	2.16190	4.25054
H	-4.03189	2.83344	5.91208
H	-5.11662	1.49095	5.40784
C	4.11812	0.46402	2.60009
C	3.62549	-0.95872	2.93072
C	5.33936	0.82361	3.46835
H	3.29729	1.14767	2.89834
C	3.28614	-1.08742	4.42036
H	4.39931	-1.71292	2.68257
H	2.72115	-1.18675	2.33358
C	5.00808	0.68795	4.96101
H	6.19896	0.16117	3.22547
H	5.64608	1.87212	3.26189
C	4.49658	-0.72127	5.28894
H	2.98144	-2.13394	4.63743
H	2.42965	-0.42288	4.66965
H	5.92119	0.89490	5.55952
H	4.23768	1.43939	5.24244
H	4.20567	-0.76567	6.36043
H	5.31087	-1.46098	5.12413
C	3.71459	2.80132	-2.01491
C	4.83205	3.74464	-2.50406
C	2.92011	2.23790	-3.21244
H	3.01376	3.43726	-1.43842
C	4.24719	4.88189	-3.35380
H	5.57965	3.18908	-3.11075
H	5.35768	4.18270	-1.62758
C	2.33190	3.37788	-4.05374
H	3.57352	1.61389	-3.85967
H	2.09085	1.59506	-2.85016
C	3.43523	4.33037	-4.53390
H	5.07531	5.51313	-3.74181
H	3.59553	5.52454	-2.72133
H	1.80676	2.95058	-4.93493
H	1.58511	3.94168	-3.45151
H	2.97333	5.17587	-5.08753
H	4.11295	3.79289	-5.23317
C	6.71127	-1.23993	-1.42762
C	8.10812	-1.19040	-0.77372
C	6.13473	-2.66900	-1.38309
H	6.84040	-0.97048	-2.50045
C	9.05830	-2.19599	-1.43780
H	8.04387	-1.42419	0.31100
H	8.53164	-0.16790	-0.87958
C	7.09185	-3.66113	-2.05507
H	5.96646	-2.98477	-0.33162
H	5.15585	-2.69548	-1.91020
C	8.47646	-3.61539	-1.39758
H	10.03411	-2.18479	-0.90626
H	9.24090	-1.89704	-2.49348
H	6.67933	-4.68830	-1.97141
H	7.18283	-3.41979	-3.13697
H	9.16017	-4.30729	-1.93467
H	8.39998	-3.95941	-0.34237

Catalyst (2,4,6-c-Hex, 11)-Enamine protonation TS (pro-R) alternative conformation
Electronic energy (Hartrees): -1870.496514
Free Energy at 1M concentration and 253 K (Hartrees): -1868.845135
Coordinates:

P	-0.06165	0.15395	0.02417
O	1.23648	0.90884	-0.61644
O	-1.15568	1.29283	0.41662
O	0.31389	-0.39462	1.43038
O	-0.52302	-0.84042	-0.99433
C	1.61996	2.06404	0.01608
C	2.97817	2.13730	0.61223
C	3.38891	3.27268	1.19621
H	4.38369	3.33739	1.62115
C	2.48595	4.43660	1.28817
C	2.91248	5.63980	2.03915
H	3.89978	5.68297	2.48203
C	2.07772	6.67515	2.17985
H	2.38860	7.55244	2.73230
C	0.72431	6.61880	1.59536
H	0.04406	7.44898	1.73574
C	0.33315	5.54059	0.90660
H	-0.67370	5.51046	0.51192
C	1.26157	4.39250	0.71485
C	0.84572	3.17211	-0.03931
C	-1.37020	2.24931	-0.54180
C	-2.73600	2.40772	-1.09707
C	-2.99178	3.38470	-1.98002
H	-3.99614	3.52438	-2.36257
C	-1.90603	4.27596	-2.43731
C	-2.17359	5.28261	-3.49000
H	-3.16713	5.37977	-3.90977
C	-1.18844	6.07371	-3.92902
H	-1.38417	6.80783	-4.69991
C	0.17162	5.94106	-3.37184
H	0.96854	6.57073	-3.74601
C	0.42242	5.03915	-2.41574
H	1.43112	4.94355	-2.03579
C	-0.66865	4.16994	-1.89861
C	-0.41521	3.15899	-0.82915
C	-3.83228	1.56194	-0.57121
C	-4.30672	1.78874	0.83895
C	-4.37349	0.56004	-1.31936
C	-5.22565	0.95483	1.35936
C	-5.40578	-0.31647	-0.70527
C	-5.79367	-0.15570	0.56949
H	-5.57885	1.09880	2.37211
H	-5.83524	-1.11525	-1.29298
C	3.89489	0.97147	0.55566
C	4.52987	0.63109	-0.60092
C	4.21817	0.23811	1.82719
C	5.57903	-0.42164	-0.56175
C	5.24015	-0.63765	1.83458
C	5.96031	-0.98682	0.59424
H	6.07710	-0.70100	-1.47972
H	5.52173	-1.13979	2.75049
H	0.21977	-2.40836	-0.79182
C	1.90804	-3.41429	-1.38509
C	0.89231	-3.49840	0.82372
C	1.85688	-2.90315	-2.69571
C	2.99482	-4.21950	-0.96688
C	2.01642	-4.27313	1.26829
C	2.87808	-3.20868	-3.57804
H	1.03144	-2.26030	-2.98408
C	4.00537	-4.53346	-1.90035

C	3.00491	-4.63571	0.40190
H	2.03786	-4.58419	2.30320
C	3.95275	-4.03223	-3.18774
H	2.84877	-2.80493	-4.58540
H	4.82994	-5.16793	-1.58794
H	3.82667	-5.25464	0.75240
H	4.73882	-4.26846	-3.89732
N	0.90289	-3.12516	-0.48933
C	-0.16482	-3.00484	1.64587
C	-1.60534	-3.05029	1.15713
H	-1.65054	-2.64154	0.14784
H	-2.21013	-2.37499	1.78147
N	-2.14186	-4.41451	1.15339
H	-2.15139	-4.80491	2.08812
C	-3.28157	-4.76105	0.41926
C	-4.19094	-5.69779	0.94647
C	-3.52068	-4.26262	-0.87465
C	-5.28981	-6.12338	0.20565
H	-4.02775	-6.08952	1.94803
C	-4.62990	-4.68741	-1.60291
H	-2.82452	-3.56236	-1.32109
C	-5.52606	-5.61711	-1.07351
H	-5.97513	-6.84550	0.64127
H	-4.79007	-4.29129	-2.60185
H	-6.38966	-5.94073	-1.64564
H	0.14415	-1.47311	1.58314
Cl	-0.07218	-3.50818	3.38569
C	3.39420	0.45217	3.09429
C	2.82811	-0.87347	3.64989
C	4.20863	1.17897	4.18109
H	2.51619	1.08949	2.85720
C	1.94788	-0.60812	4.87711
H	3.64584	-1.56351	3.94844
H	2.23032	-1.38597	2.86947
C	3.34143	1.43993	5.42098
H	5.09351	0.57228	4.47384
H	4.57538	2.15153	3.78904
C	2.73775	0.13620	5.96283
H	1.58639	-1.57587	5.28614
H	1.05897	-0.00897	4.57991
H	3.96416	1.91159	6.21122
H	2.52438	2.14907	5.16195
H	2.06063	0.37121	6.81194
H	3.55131	-0.51860	6.34561
C	7.09349	-1.99577	0.65013
C	6.74861	-3.26719	-0.14784
C	8.42204	-1.38140	0.16222
H	7.24281	-2.30255	1.71026
C	7.89217	-4.28670	-0.08172
H	6.54699	-3.01935	-1.21277
H	5.83414	-3.72777	0.28121
C	9.55811	-2.40992	0.23150
H	8.32795	-1.02270	-0.88579
H	8.68430	-0.50948	0.80031
C	9.20867	-3.67008	-0.57033
H	7.63874	-5.16257	-0.71707
H	8.01303	-4.64620	0.96389
H	10.48660	-1.95917	-0.18036
H	9.75196	-2.68316	1.29213
H	10.02592	-4.41449	-0.45773

H	9.12372	-3.41490	-1.64967
C	4.25263	1.32006	-1.93764
C	5.51254	2.02185	-2.48376
C	3.69855	0.31478	-2.96714
H	3.48737	2.11351	-1.82665
C	5.21295	2.71178	-3.82165
H	6.33907	1.29485	-2.63481
H	5.85686	2.78530	-1.75238
C	3.40332	1.00754	-4.30291
H	4.42183	-0.51082	-3.14204
H	2.75840	-0.13227	-2.58011
C	4.65373	1.71302	-4.84388
H	6.14816	3.15999	-4.22081
H	4.48050	3.53403	-3.66351
H	3.06062	0.24995	-5.04026
H	2.58427	1.74817	-4.16746
H	4.39342	2.25329	-5.77931
H	5.43124	0.95666	-5.09018
C	-3.96199	0.29089	-2.76863
C	-3.46851	-1.15612	-2.96603
C	-5.12816	0.59479	-3.72897
H	-3.11751	0.94424	-3.06882
C	-3.04691	-1.39629	-4.42045
H	-4.26412	-1.88409	-2.70860
H	-2.60234	-1.34701	-2.30302
C	-4.71486	0.34753	-5.18647
H	-6.00761	-0.04172	-3.48774
H	-5.43398	1.65784	-3.61730
C	-4.20195	-1.08568	-5.38126
H	-2.74268	-2.45818	-4.54296
H	-2.16993	-0.75833	-4.66798
H	-5.59033	0.51678	-5.84959
H	-3.92119	1.07060	-5.47745
H	-3.85141	-1.21059	-6.42827
H	-5.03279	-1.80526	-5.21003
C	-3.79505	2.96262	1.67668
C	-4.92926	3.94563	2.03046
C	-3.07453	2.48583	2.95592
H	-3.05697	3.55018	1.09538
C	-4.38227	5.13999	2.82534
H	-5.71493	3.44074	2.63333
H	-5.40074	4.31988	1.09553
C	-2.52353	3.68225	3.74226
H	-3.76927	1.91550	3.60960
H	-2.23249	1.81317	2.69010
C	-3.64296	4.67378	4.08719
H	-5.22487	5.80274	3.11797
H	-3.69014	5.73029	2.18487
H	-2.05270	3.31909	4.68106
H	-1.73880	4.19554	3.14309
H	-3.20479	5.55578	4.60181
H	-4.36388	4.19361	4.78496
C	-6.79163	-1.09568	1.22144
C	-8.14970	-1.08714	0.48875
C	-6.22688	-2.52717	1.31404
H	-6.97729	-0.74718	2.26276
C	-9.14448	-2.03601	1.17078
H	-8.02782	-1.40061	-0.57064
H	-8.56885	-0.05740	0.49557
C	-7.22890	-3.46202	2.00252

H	-6.00371	-2.92055	0.29957
H	-5.27889	-2.51986	1.89547
C	-8.57460	-3.45757	1.26723
H	-10.08931	-2.05889	0.58625
H	-9.38229	-1.65908	2.18988
H	-6.82199	-4.49472	2.01751
H	-7.37723	-3.14111	3.05707
H	-9.29338	-4.10437	1.81465
H	-8.44317	-3.87884	0.24598

Catalyst (2,4,6-c-Hex, 11)-Enamine protonation TS (pro-R) alternative conformation
Electronic energy (Hartrees): -1870.49701
Free Energy at 1M concentration and 253 K (Hartrees): -1868.844799
Coordinates:

P	0.20250	-0.04548	-0.40008
O	1.30748	0.93173	-1.07731
O	-1.06126	0.88050	0.06658
O	0.69071	-0.76919	0.81112
O	-0.23867	-0.92192	-1.61843
C	1.51271	2.10406	-0.39383
C	2.81236	2.33555	0.28677
C	3.07277	3.52870	0.84066
H	4.02669	3.70845	1.32143
C	2.03187	4.57291	0.88913
C	2.28823	5.82766	1.63310
H	3.26108	6.01056	2.07229
C	1.31682	6.73540	1.77490
H	1.50343	7.64976	2.32314
C	-0.01872	6.48432	1.20161
H	-0.81000	7.20835	1.34741
C	-0.25733	5.35884	0.51891
H	-1.25448	5.18369	0.13880
C	0.82399	4.35372	0.32087
C	0.58491	3.08496	-0.43314
C	-1.47972	1.82406	-0.83996
C	-2.90556	1.83120	-1.25355
C	-3.34939	2.76934	-2.10511
H	-4.39501	2.79858	-2.38792
C	-2.42500	3.78404	-2.64744
C	-2.90144	4.74919	-3.66460
H	-3.92846	4.71663	-4.00653
C	-2.06419	5.66370	-4.16664
H	-2.41222	6.36797	-4.91118
C	-0.66033	5.71009	-3.71348
H	0.01611	6.44192	-4.13582
C	-0.22217	4.84552	-2.79091
H	0.81491	4.88369	-2.48384
C	-1.14783	3.83799	-2.20747
C	-0.68571	2.86754	-1.17268
C	-3.86851	0.88189	-0.64282
C	-4.28807	1.07579	0.79094
C	-4.42901	-0.11449	-1.38272
C	-5.25939	0.29161	1.29682
C	-5.53537	-0.91302	-0.79549
C	-5.94797	-0.71649	0.46557
H	-5.56802	0.40382	2.32795
H	-6.04036	-1.64254	-1.41312
C	3.79391	1.23415	0.42359
C	4.55849	0.84012	-0.63178

C	4.00254	0.60989	1.77809
C	5.57201	-0.22874	-0.42558
C	5.00173	-0.27616	1.93803
C	5.80186	-0.75047	0.78846
H	6.15940	-0.56738	-1.26908
H	5.21696	-0.67854	2.91747
H	-0.16358	-2.18751	1.17375
C	-1.77457	-2.74008	2.31799
C	-1.23468	-3.58846	0.10138
C	-1.41855	-1.88084	3.37427
C	-2.98109	-3.47835	2.36339
C	-2.48561	-4.29739	0.11682
C	-2.25418	-1.77767	4.47171
H	-0.51007	-1.29540	3.29412
C	-3.79812	-3.36977	3.50931
C	-3.29836	-4.26589	1.21094
H	-2.74348	-4.88454	-0.75298
C	-3.44308	-2.52980	4.54913
H	-1.98730	-1.10553	5.28154
H	-4.71290	-3.95301	3.56235
H	-4.21339	-4.85209	1.21301
H	-4.08059	-2.44522	5.42302
C	-0.32807	-3.53283	-1.00015
H	-0.38254	-1.98937	-1.39214
C	1.17516	-3.68342	-0.75805
H	1.40972	-4.74864	-0.62662
H	1.43606	-3.17357	0.18196
N	1.94207	-3.18961	-1.87896
N	-0.94884	-2.86306	1.22293
C	3.26766	-3.50555	-2.11810
C	4.01611	-4.35761	-1.28420
C	3.89933	-2.97689	-3.26376
C	5.34394	-4.65789	-1.59229
H	3.57318	-4.77243	-0.38559
C	5.22809	-3.26574	-3.54394
H	3.32647	-2.34045	-3.93364
C	5.96824	-4.11113	-2.71113
H	5.89824	-5.31881	-0.93109
H	5.68803	-2.83152	-4.42775
H	7.00589	-4.33864	-2.93327
H	1.58490	-2.36126	-2.33232
Cl	-0.83308	-4.50596	-2.43658
C	-3.63223	2.12828	1.68631
C	-4.64998	3.15559	2.22141
C	-2.85952	1.47356	2.84974
H	-2.89769	2.71684	1.10603
C	-3.94254	4.22011	3.07291
H	-5.42714	2.65900	2.84126
H	-5.15751	3.65430	1.36702
C	-2.15184	2.53768	3.69749
H	-3.54846	0.89122	3.49949
H	-2.09851	0.77289	2.44758
C	-3.15325	3.57585	4.22165
H	-4.69979	4.91601	3.49363
H	-3.25195	4.81344	2.43330
H	-1.64813	2.04571	4.55734
H	-1.37039	3.04385	3.08788
H	-2.60365	4.36617	4.77668
H	-3.85853	3.08771	4.92976
C	-3.97724	-0.44035	-2.80585

C	-3.53314	-1.91228	-2.92757
C	-5.08782	-0.12581	-3.82715
H	-3.09235	0.16946	-3.08574
C	-3.06106	-2.22118	-4.35411
H	-4.36402	-2.60380	-2.67145
H	-2.70258	-2.10551	-2.21826
C	-4.61771	-0.43773	-5.25413
H	-6.00115	-0.72085	-3.60909
H	-5.35892	0.94997	-3.76537
C	-4.15730	-1.89623	-5.37729
H	-2.80053	-3.29836	-4.43000
H	-2.14628	-1.63103	-4.58259
H	-5.45341	-0.25599	-5.96362
H	-3.78168	0.24243	-5.52953
H	-3.76534	-2.07149	-6.40217
H	-5.02479	-2.57489	-5.22190
C	-7.13292	-1.47374	1.03902
C	-8.43238	-1.13323	0.28096
C	-6.88737	-2.99503	1.04513
H	-7.27536	-1.15792	2.09750
C	-9.62789	-1.88656	0.87866
H	-8.33935	-1.40067	-0.79439
H	-8.62523	-0.04014	0.34667
C	-8.08667	-3.74156	1.64184
H	-6.70436	-3.37176	0.01549
H	-5.99013	-3.21497	1.65509
C	-9.37739	-3.40000	0.88682
H	-10.53660	-1.66871	0.27723
H	-9.81215	-1.53265	1.91695
H	-7.90427	-4.83608	1.58100
H	-8.19866	-3.47285	2.71542
H	-10.23434	-3.91071	1.37629
H	-9.30813	-3.77280	-0.15891
C	3.12500	0.98451	2.97420
C	2.51879	-0.24944	3.68008
C	3.90248	1.84048	3.99270
H	2.26256	1.58781	2.62197
C	1.58585	0.18902	4.81665
H	3.31393	-0.89548	4.10935
H	1.94824	-0.86227	2.95544
C	2.98392	2.27870	5.14243
H	4.76005	1.26552	4.40636
H	4.30931	2.74587	3.49534
C	2.33437	1.06809	5.82782
H	1.18919	-0.71044	5.33498
H	0.72151	0.75098	4.39853
H	3.57764	2.84781	5.88963
H	2.19164	2.95410	4.75000
H	1.62097	1.42428	6.60173
H	3.11803	0.46469	6.33665
C	4.46569	1.49684	-2.01045
C	5.78520	2.20479	-2.37728
C	4.08481	0.47120	-3.09437
H	3.68031	2.27974	-2.02703
C	5.67953	2.87440	-3.75465
H	6.62927	1.48162	-2.39264
H	6.01289	2.98156	-1.61492
C	3.97250	1.14484	-4.46717
H	4.85142	-0.32859	-3.15677
H	3.11107	0.00020	-2.84030

C	5.27990	1.85930	-4.83415
H	6.66026	3.32455	-4.01988
H	4.92744	3.69308	-3.71585
H	3.74715	0.37432	-5.23564
H	3.13419	1.87602	-4.45909
H	5.14951	2.38700	-5.80329
H	6.09186	1.10934	-4.95915
C	6.87104	-1.81133	0.97968
C	8.03592	-1.26765	1.82843
C	6.29141	-3.08905	1.62010
H	7.28710	-2.09821	-0.01400
C	9.12583	-2.33429	2.00456
H	7.67170	-0.95009	2.83025
H	8.47866	-0.37948	1.32691
C	7.38135	-4.15592	1.77858
H	5.86181	-2.87097	2.62133
H	5.47499	-3.48951	0.98612
C	8.55093	-3.61987	2.61484
H	9.92303	-1.93769	2.66940
H	9.58644	-2.56378	1.01845
H	6.95062	-5.04952	2.27936
H	7.74952	-4.46900	0.77681
H	9.34950	-4.39093	2.66473
H	8.20538	-3.41735	3.65255

Catalyst (2,4,6-c-Hex, 11)-Enamine protonation TS (pro-R) alternative conformation
Electronic energy (Hartrees): -1870.49389
Free Energy at 1M concentration and 253 K (Hartrees): -1868.842985
Coordinates:

P	-0.05701	-0.23528	-0.05697
O	1.21917	0.65909	-0.53958
O	-1.24371	0.77996	0.44153
O	0.21383	-1.13413	1.11018
O	-0.51791	-0.89581	-1.38689
C	1.54786	1.67786	0.31963
C	2.88331	1.68504	0.96936
C	3.19313	2.65325	1.84500
H	4.17124	2.67273	2.31217
C	2.22031	3.71835	2.15867
C	2.54011	4.72732	3.19455
H	3.49363	4.69743	3.70701
C	1.65021	5.67624	3.50551
H	1.88290	6.41106	4.26536
C	0.34342	5.71813	2.82142
H	-0.38107	6.47480	3.09371
C	0.04963	4.81800	1.87618
H	-0.92351	4.85343	1.40446
C	1.03944	3.77126	1.50186
C	0.72715	2.74052	0.46898
C	-1.42986	1.89845	-0.32925
C	-2.76456	2.14581	-0.92879
C	-2.97561	3.25673	-1.65169
H	-3.95556	3.45830	-2.06801
C	-1.88302	4.22668	-1.86763
C	-2.09968	5.39958	-2.74545
H	-3.06186	5.55552	-3.21726
C	-1.10633	6.26889	-2.96241
H	-1.26437	7.12442	-3.60627
C	0.21176	6.05959	-2.33228

H	1.01672	6.75650	-2.52711
C	0.41503	5.00757	-1.53093
H	1.39458	4.86150	-1.09450
C	-0.68700	4.04447	-1.26303
C	-0.48372	2.85926	-0.38058
C	-3.89563	1.23154	-0.63925
C	-4.54312	1.27240	0.71852
C	-4.39173	0.40591	-1.60160
C	-5.63658	0.51825	0.94201
C	-5.64302	-0.34652	-1.32923
C	-6.24122	-0.29905	-0.12959
H	-6.10949	0.51993	1.91537
H	-6.09171	-0.92423	-2.12505
C	3.92198	0.69937	0.58868
C	4.66775	0.87757	-0.53585
C	4.27521	-0.39370	1.55009
C	5.89881	0.06252	-0.72110
C	5.42054	-1.07641	1.36945
C	6.30579	-0.81545	0.21400
H	6.49631	0.24471	-1.59970
H	5.70721	-1.85329	2.06609
H	-0.81358	-2.43604	1.16864
C	-2.62095	-3.00369	1.98335
C	-1.77400	-3.65368	-0.19667
C	-2.40188	-2.28327	3.17355
C	-3.85398	-3.66148	1.75300
C	-3.03633	-4.28044	-0.46177
C	-3.40228	-2.24023	4.12718
H	-1.46482	-1.75388	3.30802
C	-4.84420	-3.61720	2.75914
C	-4.01790	-4.30387	0.48643
H	-3.17390	-4.75690	-1.42220
C	-4.62428	-2.91509	3.92893
H	-3.24260	-1.67482	5.04010
H	-5.78373	-4.13767	2.59667
H	-4.95197	-4.81923	0.27870
H	-5.39247	-2.87722	4.69425
C	-0.69292	-3.51632	-1.12855
H	-0.65531	-2.01666	-1.31945
C	0.73173	-3.80646	-0.67018
H	0.94980	-4.87864	-0.81004
H	0.79705	-3.60388	0.40446
N	1.69996	-2.99075	-1.38460
N	-1.63722	-3.06320	1.02331
C	3.04406	-3.31948	-1.44388
C	3.64474	-4.18875	-0.51399
C	3.85100	-2.75766	-2.45452
C	4.99923	-4.50443	-0.62113
H	3.05864	-4.61345	0.29376
C	5.19575	-3.08709	-2.55194
H	3.40312	-2.07180	-3.16948
C	5.78311	-3.97379	-1.64357
H	5.44156	-5.17958	0.10662
H	5.78918	-2.65160	-3.35129
H	6.83306	-4.23574	-1.72712
H	1.33866	-2.51216	-2.19659
Cl	-1.00085	-4.25144	-2.75661
C	-3.98410	2.13944	1.84700
C	-5.01067	3.17853	2.34177
C	-3.47404	1.27643	3.01850

H	-3.11810	2.72513	1.48383
C	-4.40107	4.05723	3.44337
H	-5.91863	2.67928	2.74327
H	-5.32094	3.82615	1.49289
C	-2.86597	2.15745	4.11675
H	-4.30188	0.67502	3.45304
H	-2.69669	0.57320	2.65256
C	-3.87795	3.20216	4.60591
H	-5.17461	4.75869	3.82329
H	-3.56833	4.66285	3.02181
H	-2.55676	1.51697	4.97064
H	-1.95806	2.66913	3.72659
H	-3.38952	3.86194	5.35480
H	-4.73013	2.69010	5.10464
C	-3.71790	0.22553	-2.96083
C	-3.42905	-1.26189	-3.25530
C	-4.57176	0.83600	-4.08815
H	-2.73331	0.73974	-2.97712
C	-2.71682	-1.41650	-4.60451
H	-4.36778	-1.85476	-3.28441
H	-2.79283	-1.68552	-2.45246
C	-3.87119	0.67759	-5.44484
H	-5.56805	0.34419	-4.13263
H	-4.73316	1.91766	-3.89163
C	-3.54871	-0.79495	-5.73464
H	-2.56163	-2.49600	-4.81651
H	-1.71773	-0.92950	-4.56008
H	-4.53118	1.07287	-6.24666
H	-2.93169	1.27314	-5.45156
H	-2.98202	-0.86527	-6.68784
H	-4.49559	-1.36565	-5.85681
C	-7.54253	-1.03480	0.13656
C	-8.68581	-0.48486	-0.74030
C	-7.38572	-2.55522	-0.06218
H	-7.83136	-0.86849	1.19923
C	-10.00025	-1.22081	-0.44880
H	-8.44078	-0.59845	-1.81896
H	-8.82502	0.59858	-0.53273
C	-8.70273	-3.28405	0.23045
H	-7.06865	-2.78863	-1.10168
H	-6.60527	-2.93417	0.62608
C	-9.83940	-2.73446	-0.64071
H	-10.79020	-0.84632	-1.13481
H	-10.32447	-1.00889	0.59387
H	-8.57466	-4.36878	0.02582
H	-8.96456	-3.16750	1.30521
H	-10.78929	-3.24165	-0.36645
H	-9.62982	-2.95493	-1.71063
C	3.34972	-0.75060	2.70848
C	2.91707	-2.23113	2.65906
C	3.99594	-0.42106	4.06758
H	2.41857	-0.14986	2.63501
C	1.94966	-2.55073	3.80496
H	3.79497	-2.90738	2.73849
H	2.42014	-2.43836	1.69004
C	3.03540	-0.75092	5.21883
H	4.93703	-0.99794	4.20197
H	4.24768	0.66105	4.10722
C	2.58302	-2.21690	5.16211
H	1.68827	-3.63035	3.77512

H	1.01139	-1.96857	3.67830
H	3.54578	-0.56204	6.18762
H	2.14635	-0.08436	5.16567
H	1.84078	-2.40262	5.96791
H	3.45568	-2.88315	5.34019
C	4.33358	1.93528	-1.58892
C	5.44958	2.99222	-1.70656
C	4.05049	1.28041	-2.95511
H	3.41443	2.49105	-1.31858
C	5.08420	4.05067	-2.75614
H	6.41426	2.52198	-1.99394
H	5.59047	3.49186	-0.72325
C	3.69056	2.34249	-4.00239
H	4.93488	0.70800	-3.30970
H	3.20031	0.57047	-2.85580
C	4.79588	3.40079	-4.11615
H	5.92664	4.76731	-2.86332
H	4.19101	4.62139	-2.41789
H	3.54948	1.85147	-4.98918
H	2.73112	2.83250	-3.72417
H	4.47899	4.18500	-4.83680
H	5.72395	2.92977	-4.50859
C	7.63451	-1.56563	0.15081
C	8.39420	-1.44180	-1.19195
C	8.55704	-1.11246	1.30309
H	7.40107	-2.64310	0.30156
C	9.67891	-2.27805	-1.17130
H	8.66898	-0.38086	-1.38371
H	7.75446	-1.79444	-2.02708
C	9.85705	-1.92857	1.32563
H	8.79816	-0.03196	1.19076
H	8.04808	-1.24585	2.28176
C	10.59083	-1.84447	-0.01786
H	10.21826	-2.14631	-2.13393
H	9.42473	-3.35579	-1.06566
H	10.51950	-1.54072	2.12913
H	9.62542	-2.99153	1.55747
H	11.48521	-2.50330	0.01050
H	10.93865	-0.80174	-0.18808

Catalyst (2,4,6-c-Hex, 11)-Enamine protonation TS (pro-R) alternative conformation
Electronic energy (Hartrees): -1870.492228
Free Energy at 1M concentration and 253 K (Hartrees): -1868.84036
Coordinates:

P	0.17726	0.00302	-0.42706
O	1.31252	1.01255	-0.99880
O	-1.08883	0.90799	0.07170
O	0.62138	-0.81711	0.73830
O	-0.23850	-0.77340	-1.72089
C	1.51176	2.12786	-0.22411
C	2.79413	2.29068	0.50725
C	3.04931	3.43300	1.16161
H	3.99155	3.56419	1.67966
C	2.01742	4.48236	1.26296
C	2.26371	5.67144	2.11093
H	3.22452	5.80705	2.59180
C	1.29769	6.57732	2.29516
H	1.47674	7.44330	2.91922
C	-0.02215	6.38898	1.66432

H	-0.81010	7.10919	1.84327
C	-0.25115	5.32388	0.88789
H	-1.23787	5.19189	0.46549
C	0.82535	4.32380	0.64348
C	0.59605	3.12072	-0.21404
C	-1.47005	1.92257	-0.77243
C	-2.88450	1.98321	-1.21905
C	-3.29299	2.98803	-2.00999
H	-4.33033	3.05403	-2.31628
C	-2.34111	4.02646	-2.45058
C	-2.77718	5.07207	-3.40433
H	-3.79457	5.07994	-3.77528
C	-1.91519	6.00985	-3.81294
H	-2.23370	6.77305	-4.51118
C	-0.52414	6.00228	-3.31963
H	0.17257	6.75438	-3.66674
C	-0.12244	5.06461	-2.45360
H	0.90589	5.06505	-2.11618
C	-1.07607	4.02900	-1.97367
C	-0.65468	2.97681	-1.00353
C	-3.87624	1.00856	-0.70166
C	-4.33861	1.11633	0.72645
C	-4.42529	0.06986	-1.52076
C	-5.34834	0.33029	1.14353
C	-5.55704	-0.75402	-1.01954
C	-6.02241	-0.61962	0.23408
H	-5.69362	0.38475	2.16799
H	-6.01673	-1.45029	-1.70131
C	3.76573	1.17427	0.57488
C	4.55439	0.86550	-0.49111
C	3.93729	0.43627	1.87629
C	5.55499	-0.22612	-0.35251
C	4.92660	-0.46860	1.98428
C	5.75138	-0.85095	0.81793
H	6.16051	-0.49718	-1.20756
H	5.11579	-0.95364	2.93109
H	-0.24210	-2.25946	0.96513
C	-1.85725	-2.93646	2.03644
C	-1.28080	-3.57508	-0.24063
C	-1.52992	-2.16905	3.16996
C	-3.04768	-3.70081	2.00251
C	-2.52890	-4.28673	-0.30986
C	-2.37175	-2.19289	4.26753
H	-0.63650	-1.55567	3.15133
C	-3.86627	-3.73007	3.15102
C	-3.35182	-4.36897	0.77391
H	-2.77326	-4.78618	-1.23645
C	-3.53698	-2.98515	4.26893
H	-2.12665	-1.59248	5.13831
H	-4.76685	-4.33747	3.13816
H	-4.26607	-4.95321	0.71037
H	-4.17790	-3.00399	5.14421
C	-0.35092	-3.42891	-1.31290
H	-0.39258	-1.84912	-1.58056
C	1.14654	-3.60112	-1.04989
H	1.37769	-4.67405	-1.00174
H	1.38474	-3.17189	-0.06478
N	1.94077	-3.01800	-2.10689
N	-1.02063	-2.94380	0.94317
C	3.26688	-3.32679	-2.35095

C	3.99236	-4.25772	-1.58363
C	3.92438	-2.70555	-3.43394
C	5.32278	-4.54360	-1.89403
H	3.52981	-4.74648	-0.73335
C	5.25539	-2.98252	-3.71549
H	3.36978	-2.00644	-4.05490
C	5.97266	-3.90626	-2.94852
H	5.85869	-5.26642	-1.28442
H	5.73520	-2.47631	-4.54902
H	7.01215	-4.12401	-3.17175
H	1.59594	-2.15443	-2.50001
Cl	-0.82218	-4.27677	-2.83704
C	-3.67569	2.07289	1.71872
C	-4.67284	3.09118	2.30709
C	-2.95903	1.30089	2.84627
H	-2.90568	2.67863	1.20664
C	-3.95349	4.05964	3.25767
H	-5.48353	2.57651	2.86617
H	-5.13875	3.67301	1.48209
C	-2.23939	2.26943	3.79288
H	-3.68572	0.69501	3.43033
H	-2.21196	0.60485	2.41009
C	-3.21905	3.29952	4.37159
H	-4.69801	4.74758	3.71283
H	-3.22542	4.67576	2.68459
H	-1.77711	1.69411	4.62380
H	-1.42423	2.79275	3.24497
H	-2.65845	4.02376	5.00084
H	-3.96021	2.78375	5.02106
C	-3.93640	-0.16294	-2.95060
C	-3.49969	-1.62682	-3.16406
C	-5.01547	0.23321	-3.97719
H	-3.03950	0.45611	-3.16395
C	-2.98948	-1.83771	-4.59547
H	-4.34180	-2.32785	-2.98225
H	-2.69146	-1.87796	-2.44720
C	-4.50763	0.01899	-5.40926
H	-5.93879	-0.36747	-3.82692
H	-5.28027	1.30445	-3.84651
C	-4.05393	-1.43109	-5.62305
H	-2.73494	-2.90907	-4.74109
H	-2.06431	-1.24130	-4.75580
H	-5.32181	0.25789	-6.12679
H	-3.65954	0.70954	-5.61256
H	-3.63422	-1.53663	-6.64651
H	-4.93017	-2.11131	-5.54064
C	-7.21434	-1.39946	0.77794
C	-8.14939	-2.00134	-0.29720
C	-6.73678	-2.50491	1.73599
H	-7.82649	-0.67603	1.36376
C	-9.34743	-2.69957	0.35804
H	-7.60893	-2.75558	-0.90983
H	-8.51962	-1.19554	-0.96774
C	-7.92479	-3.23065	2.38194
H	-6.11381	-3.23752	1.17846
H	-6.11507	-2.06234	2.54370
C	-8.87604	-3.79733	1.32044
H	-9.98471	-3.15347	-0.43123
H	-9.96311	-1.95492	0.90912
H	-7.54547	-4.06142	3.01511

H	-8.47883	-2.52543	3.03991
H	-9.75854	-4.24900	1.82239
H	-8.35977	-4.59952	0.74824
C	3.03456	0.71758	3.07918
C	2.40442	-0.56559	3.66662
C	3.79384	1.47992	4.18231
H	2.18443	1.35517	2.75875
C	1.44868	-0.21509	4.81473
H	3.18538	-1.25149	4.05843
H	1.84658	-1.11179	2.88129
C	2.85205	1.82889	5.34373
H	4.63813	0.86572	4.56579
H	4.21758	2.42006	3.77096
C	2.17955	0.57098	5.91171
H	1.03468	-1.15112	5.24776
H	0.59766	0.38669	4.42567
H	3.43212	2.32934	6.14857
H	2.07314	2.54084	4.99119
H	1.45102	0.86760	6.69659
H	2.94766	-0.07888	6.38587
C	4.50167	1.63952	-1.80984
C	5.83574	2.36277	-2.08203
C	4.13949	0.71528	-2.98718
H	3.72354	2.42915	-1.77793
C	5.77006	3.14976	-3.39848
H	6.67398	1.63495	-2.13949
H	6.05068	3.06848	-1.25006
C	4.06721	1.50588	-4.29897
H	4.90066	-0.08423	-3.09975
H	3.15561	0.23483	-2.79827
C	5.38924	2.23571	-4.57104
H	6.76078	3.61094	-3.59998
H	5.02398	3.96977	-3.30724
H	3.85483	0.80685	-5.13638
H	3.23503	2.24232	-4.24807
H	5.28749	2.84632	-5.49382
H	6.19786	1.49093	-4.74070
C	6.80878	-1.93318	0.94386
C	7.95669	-1.47263	1.86203
C	6.20596	-3.25528	1.46087
H	7.24643	-2.13905	-0.06059
C	9.03530	-2.55939	1.97257
H	7.57088	-1.23750	2.87841
H	8.41683	-0.54912	1.44755
C	7.28493	-4.34086	1.55375
H	5.75431	-3.11857	2.46680
H	5.40220	-3.59409	0.77684
C	8.43795	-3.88696	2.45881
H	9.81910	-2.22686	2.68666
H	9.51760	-2.70887	0.98146
H	6.83674	-5.26975	1.96742
H	7.67448	-4.57166	0.53782
H	9.23017	-4.66615	2.46171
H	8.06940	-3.76968	3.50173

Catalyst (2,4,6-c-Hex, 11)-Enamine protonation TS (pro-R) alternative conformation
Electronic energy (Hartrees): -1870.4911
Free Energy at 1M concentration and 253 K (Hartrees): -1868.83966
Coordinates:

P	0.02214	-0.28162	-0.04152
O	1.30464	0.64255	-0.44125
O	-1.20606	0.69761	0.42212
O	0.25325	-1.22104	1.09780
O	-0.33832	-0.91325	-1.42194
C	1.61478	1.58386	0.50854
C	2.93324	1.53444	1.18760
C	3.20451	2.39488	2.18057
H	4.17559	2.38243	2.66233
C	2.20977	3.41156	2.57941
C	2.48548	4.30339	3.72901
H	3.41708	4.21584	4.27414
C	1.58281	5.21766	4.10155
H	1.78329	5.86683	4.94407
C	0.30639	5.33886	3.37065
H	-0.42815	6.06690	3.69004
C	0.05276	4.54583	2.32334
H	-0.89799	4.63705	1.81456
C	1.05657	3.53942	1.88369
C	0.78628	2.62418	0.73727
C	-1.33086	1.88507	-0.25422
C	-2.62145	2.20088	-0.91594
C	-2.78390	3.37886	-1.53837
H	-3.73387	3.62661	-1.99715
C	-1.67535	4.35325	-1.59215
C	-1.82762	5.60247	-2.37287
H	-2.75715	5.81247	-2.88725
C	-0.81539	6.47361	-2.45089
H	-0.92592	7.38453	-3.02487
C	0.45929	6.19145	-1.76262
H	1.28113	6.89091	-1.84615
C	0.60306	5.07108	-1.04571
H	1.55271	4.87430	-0.56548
C	-0.52108	4.10239	-0.93318
C	-0.37999	2.83814	-0.15392
C	-3.76599	1.26843	-0.78408
C	-4.45231	1.14353	0.54752
C	-4.20583	0.53714	-1.84515
C	-5.47805	0.28136	0.67273
C	-5.39486	-0.33883	-1.67426
C	-5.99776	-0.48201	-0.48139
H	-5.97166	0.15779	1.62811
H	-5.77028	-0.85599	-2.54181
C	4.01566	0.67441	0.65911
C	4.75287	1.07769	-0.41265
C	4.38524	-0.57983	1.39083
C	5.96836	0.30575	-0.78399
C	5.51791	-1.22266	1.05003
C	6.37252	-0.75543	-0.06305
H	6.55063	0.65390	-1.62174
H	5.81638	-2.11875	1.57860
H	-0.92903	-2.47427	1.21525
C	-2.76996	-2.77157	2.08039
C	-2.12534	-3.41138	-0.17761
C	-2.37545	-2.24142	3.32325
C	-4.11612	-3.14461	1.85140
C	-3.49626	-3.75979	-0.42774
C	-3.32011	-2.09743	4.32413
H	-1.34497	-1.93493	3.46869
C	-5.05441	-2.98455	2.89292

C	-4.43633	-3.65196	0.55389
H	-3.75022	-4.14162	-1.40633
C	-4.66347	-2.46897	4.11477
H	-3.02089	-1.68262	5.28170
H	-6.08746	-3.27084	2.71707
H	-5.45666	-3.97000	0.36091
H	-5.38853	-2.34524	4.91236
C	-1.06974	-3.45226	-1.14082
H	-0.74655	-1.95207	-1.33622
C	0.29743	-3.99169	-0.72606
H	0.28852	-5.08761	-0.81020
H	0.46424	-3.75327	0.33505
N	1.35850	-3.47226	-1.56542
N	-1.84575	-2.93787	1.07293
C	2.68604	-3.83113	-1.38386
C	3.08115	-4.89569	-0.55180
C	3.68721	-3.11998	-2.07802
C	4.42880	-5.23991	-0.44006
H	2.34285	-5.45012	0.01651
C	5.02244	-3.47924	-1.96118
H	3.39877	-2.28923	-2.71747
C	5.41052	-4.54485	-1.14246
H	4.70810	-6.06356	0.21171
H	5.76800	-2.92169	-2.51955
H	6.45589	-4.82319	-1.05484
H	1.21326	-2.52805	-1.89606
Cl	-1.55001	-4.09356	-2.76134
C	-4.02998	1.99043	1.74930
C	-5.17126	2.90970	2.22994
C	-3.51588	1.11443	2.90820
H	-3.19844	2.66551	1.46711
C	-4.70747	3.77726	3.40872
H	-6.05277	2.31218	2.54840
H	-5.48887	3.57310	1.39606
C	-3.05026	1.98382	4.08226
H	-4.31522	0.42957	3.26306
H	-2.66258	0.49643	2.56023
C	-4.17699	2.90996	4.55869
H	-5.56151	4.38741	3.77352
H	-3.90978	4.47459	3.06930
H	-2.73475	1.32702	4.92138
H	-2.17043	2.59097	3.77344
H	-3.79231	3.56845	5.36684
H	-5.00706	2.30058	4.97925
C	-3.52677	0.57644	-3.21507
C	-3.10503	-0.83246	-3.68013
C	-4.44125	1.23558	-4.26539
H	-2.59356	1.17610	-3.17350
C	-2.39895	-0.76673	-5.03967
H	-3.98244	-1.50707	-3.77138
H	-2.41725	-1.27520	-2.93450
C	-3.74448	1.29626	-5.63191
H	-5.39176	0.66787	-4.36742
H	-4.69332	2.26930	-3.94413
C	-3.29781	-0.09929	-6.08913
H	-2.14737	-1.79670	-5.37262
H	-1.44835	-0.19735	-4.94224
H	-4.44538	1.72089	-6.38237
H	-2.86088	1.96927	-5.57095
H	-2.74000	-0.01037	-7.04602

H	-4.19209	-0.73491	-6.27207
C	-7.21393	-1.37388	-0.25493
C	-8.47269	-0.51871	-0.00471
C	-7.49680	-2.39470	-1.38254
H	-7.00752	-1.95946	0.66869
C	-9.67689	-1.40426	0.34704
H	-8.70547	0.08471	-0.91012
H	-8.29910	0.18511	0.83770
C	-8.68054	-3.29468	-1.00999
H	-7.74782	-1.86852	-2.32975
H	-6.60077	-3.02732	-1.55922
C	-9.93566	-2.45479	-0.74072
H	-10.57964	-0.76653	0.46202
H	-9.49276	-1.91330	1.31882
H	-8.88337	-3.99926	-1.84508
H	-8.42809	-3.89529	-0.10837
H	-10.76161	-3.12337	-0.41586
H	-10.25416	-1.94852	-1.67854
C	3.49338	-1.14354	2.49308
C	3.02955	-2.57951	2.16938
C	4.19524	-1.09520	3.86401
H	2.57308	-0.52838	2.57794
C	2.10873	-3.11774	3.27112
H	3.89705	-3.26612	2.06769
H	2.48357	-2.58007	1.20426
C	3.27587	-1.64178	4.96514
H	5.13267	-1.69287	3.84463
H	4.46304	-0.04364	4.10647
C	2.80407	-3.06524	4.63751
H	1.83275	-4.16854	3.03728
H	1.17273	-2.51944	3.30680
H	3.82578	-1.65113	5.93070
H	2.39373	-0.97396	5.08093
H	2.09495	-3.40590	5.42230
H	3.67535	-3.75670	4.63948
C	4.41284	2.33007	-1.22534
C	5.54164	3.37750	-1.15654
C	4.08913	1.96902	-2.68788
H	3.51014	2.83333	-0.82680
C	5.16449	4.63179	-1.95720
H	6.49106	2.96640	-1.56151
H	5.71570	3.66429	-0.09640
C	3.71456	3.22529	-3.48586
H	4.95944	1.47736	-3.17409
H	3.23457	1.25777	-2.71352
C	4.82968	4.27703	-3.41242
H	6.01456	5.34722	-1.94041
H	4.28943	5.12986	-1.48372
H	3.54156	2.94740	-4.54775
H	2.76870	3.65461	-3.08676
H	4.50285	5.19475	-3.94703
H	5.74044	3.89045	-3.92067
C	7.67033	-1.51258	-0.32949
C	8.34666	-1.20114	-1.68566
C	8.67450	-1.26974	0.81633
H	7.41315	-2.59436	-0.33544
C	9.59745	-2.06679	-1.87884
H	8.65301	-0.13269	-1.73009
H	7.63964	-1.40027	-2.51890
C	9.94269	-2.11410	0.62667

H	8.94757	-0.19181	0.85692
H	8.21767	-1.54157	1.79244
C	10.59445	-1.84363	-0.73505
H	10.08172	-1.80243	-2.84352
H	9.30911	-3.14026	-1.92296
H	10.66762	-1.87314	1.43374
H	9.68684	-3.19351	0.70883
H	11.46315	-2.52383	-0.86754
H	10.97093	-0.79745	-0.76653

Catalyst (2,4,6-c-Pent, 10)-Product (R) lowest free energy conformation
Electronic energy (Hartrees): -1870.539098
Free Energy at 1M concentration and 253 K (Hartrees): -1868.883813
Coordinates:

P	0.09761	-0.18988	0.09625
O	-1.05472	0.81357	0.66405
O	1.45355	0.68747	-0.14551
O	-0.27455	-0.51761	-1.39715
O	0.20770	-1.32794	1.04079
C	-1.04687	2.09509	0.17777
C	-2.25039	2.59197	-0.53437
C	-2.30162	3.86698	-0.94718
H	-3.18323	4.23965	-1.45478
C	-1.14572	4.76454	-0.74708
C	-1.17113	6.13257	-1.31398
H	-2.05187	6.48998	-1.83293
C	-0.10364	6.92954	-1.19485
H	-0.12147	7.92749	-1.61344
C	1.10736	6.44629	-0.50439
H	1.97678	7.08714	-0.43327
C	1.13664	5.21647	0.02116
H	2.04466	4.87511	0.50017
C	-0.05248	4.32644	-0.08058
C	-0.03950	2.94397	0.48327
C	1.82028	1.42023	0.95298
C	3.11036	1.12719	1.62104
C	3.45548	1.80171	2.72757
H	4.41440	1.62012	3.19921
C	2.56135	2.84319	3.27535
C	2.91690	3.52968	4.53839
H	3.83066	3.27162	5.05912
C	2.10984	4.46980	5.04234
H	2.36935	4.96974	5.96655
C	0.85819	4.82283	4.34473
H	0.20072	5.57123	4.76800
C	0.53141	4.21409	3.19883
H	-0.40025	4.47375	2.71361
C	1.43074	3.18401	2.61330
C	1.08437	2.48316	1.34217
C	4.10187	0.24814	0.95967
C	4.94262	0.81862	-0.14749
C	4.30386	-1.03096	1.37667
C	5.93902	0.07296	-0.66004
C	5.44452	-1.79975	0.81022
C	6.23319	-1.28203	-0.14353
H	6.55084	0.46502	-1.46044
H	5.66160	-2.78454	1.20224
C	-3.39712	1.68350	-0.78732
C	-4.24072	1.32208	0.22066
C	-3.66174	1.20768	-2.19062

C	-5.42091	0.47560	-0.09621
C	-4.78046	0.50066	-2.43985
C	-5.70080	0.10565	-1.35511
H	-6.08279	0.17692	0.70466
H	-5.01655	0.19065	-3.44878
H	-0.37241	-1.53609	-1.55899
C	1.04734	-3.52166	-1.59601
C	-1.21149	-3.99080	-1.25882
C	2.05487	-2.58690	-1.95435
C	1.42979	-4.84732	-1.22419
C	-0.91307	-5.33388	-0.91735
C	3.38016	-2.95195	-1.90979
H	1.76638	-1.58331	-2.24216
C	2.80662	-5.18993	-1.19094
C	0.39515	-5.75181	-0.88793
H	-1.72119	-6.00892	-0.65917
C	3.76387	-4.25838	-1.51920
H	4.13767	-2.22067	-2.17121
H	3.08872	-6.19621	-0.89434
H	0.64632	-6.76962	-0.60197
H	4.81546	-4.52285	-1.47621
C	-2.65471	-3.52482	-1.19910
H	-3.27517	-4.36612	-0.88828
C	-2.89720	-2.33962	-0.24643
H	-3.96406	-2.11174	-0.23746
H	-2.39798	-1.45093	-0.63794
N	-2.42725	-2.65323	1.08328
N	-0.26330	-3.12621	-1.58838
C	-3.27226	-2.86638	2.16458
C	-4.61771	-3.25278	2.01847
C	-2.76356	-2.70345	3.47010
C	-5.42800	-3.43050	3.13946
H	-5.03323	-3.42721	1.03184
C	-3.58022	-2.89469	4.57791
H	-1.72734	-2.40051	3.59560
C	-4.92380	-3.25172	4.42680
H	-6.46614	-3.71901	2.99670
H	-3.16485	-2.74980	5.57169
H	-5.56015	-3.39168	5.29479
H	-1.50994	-2.27094	1.30219
Cl	-3.26118	-3.09064	-2.87685
C	-4.05815	1.78718	1.66677
C	-5.25638	2.63636	2.13750
C	-3.83833	0.58753	2.61022
H	-3.16567	2.43723	1.76695
C	-5.05861	3.10145	3.58672
H	-6.20254	2.05706	2.07483
H	-5.35716	3.52895	1.48218
C	-3.64203	1.05759	4.05646
H	-4.70331	-0.10920	2.57350
H	-2.93513	0.02677	2.29225
C	-4.83079	1.90715	4.52288
H	-5.95960	3.66073	3.91870
H	-4.18804	3.79166	3.64315
H	-3.54131	0.17210	4.71995
H	-2.70393	1.65061	4.13336
H	-4.63360	2.27971	5.55098
H	-5.74784	1.27848	4.55617
C	-6.94065	-0.70701	-1.68739
C	-6.91402	-2.08842	-1.00181

C	-8.22733	0.06294	-1.32392
H	-6.96287	-0.88599	-2.78629
C	-8.16901	-2.89900	-1.34800
H	-6.85311	-1.97548	0.10223
H	-6.02178	-2.65499	-1.34378
C	-9.47484	-0.75862	-1.67259
H	-8.24782	0.30213	-0.23833
H	-8.25763	1.02165	-1.88624
C	-9.44238	-2.12727	-0.98129
H	-8.14929	-3.86012	-0.79043
H	-8.17438	-3.13435	-2.43510
H	-10.38154	-0.20491	-1.34675
H	-9.53628	-0.89728	-2.77449
H	-10.33091	-2.71688	-1.29372
H	-9.49202	-1.99066	0.12153
C	-2.69966	1.52781	-3.33441
C	-2.25198	0.26434	-4.10280
C	-3.31567	2.55030	-4.30882
H	-1.77361	1.97999	-2.92268
C	-1.24178	0.62988	-5.19722
H	-3.11744	-0.24152	-4.58093
H	-1.78597	-0.45878	-3.40505
C	-2.31645	2.91478	-5.41573
H	-4.23921	2.13741	-4.77094
H	-3.59358	3.47440	-3.75843
C	-1.83552	1.66363	-6.16395
H	-0.96605	-0.28592	-5.76308
H	-0.31606	1.03686	-4.73363
H	-2.80310	3.60768	-6.13532
H	-1.44350	3.44205	-4.97134
H	-1.06324	1.95568	-6.90757
H	-2.68791	1.21024	-6.71625
C	4.66920	2.21048	-0.71888
C	5.88905	3.14454	-0.58685
C	4.19553	2.13033	-2.18343
H	3.85383	2.70176	-0.15400
C	5.55882	4.54188	-1.13029
H	6.76123	2.74062	-1.14437
H	6.17498	3.23113	0.48414
C	3.86913	3.52878	-2.72406
H	4.97781	1.66658	-2.82308
H	3.28259	1.49834	-2.24493
C	5.07469	4.46827	-2.58505
H	6.46699	5.18002	-1.07793
H	4.77315	5.01358	-0.49932
H	3.58603	3.45087	-3.79573
H	3.00034	3.95049	-2.17161
H	4.78669	5.48564	-2.92677
H	5.90281	4.11010	-3.23547
C	3.41745	-1.70219	2.42400
C	2.83850	-3.03536	1.90394
C	4.18790	-1.92943	3.73756
H	2.54580	-1.05679	2.66431
C	1.92417	-3.67697	2.95427
H	3.64808	-3.75429	1.65669
H	2.25803	-2.85429	0.97754
C	3.28223	-2.58200	4.79193
H	5.07226	-2.58051	3.56322
H	4.55111	-0.95410	4.12859
C	2.67951	-3.89493	4.27203

H	1.55766	-4.65450	2.57304
H	1.03937	-3.02710	3.13263
H	3.87629	-2.78891	5.70790
H	2.46424	-1.88013	5.06709
H	1.97921	-4.30299	5.03214
H	3.48993	-4.64096	4.11776
C	7.43771	-2.05680	-0.65153
C	7.35128	-2.30918	-2.17105
C	8.74456	-1.31947	-0.29668
H	7.47325	-3.05334	-0.15372
C	8.57881	-3.08340	-2.66823
H	7.28317	-1.34984	-2.72768
H	6.44262	-2.90261	-2.40065
C	9.96772	-2.09632	-0.80020
H	8.75292	-0.30239	-0.74644
H	8.81922	-1.20614	0.80695
C	9.87605	-2.34795	-2.31021
H	8.51653	-3.20053	-3.77156
H	8.58841	-4.09996	-2.21677
H	10.88814	-1.51351	-0.58097
H	10.04178	-3.06745	-0.26297
H	10.74551	-2.95986	-2.63328
H	9.91822	-1.37847	-2.85389

Catalyst (2,4,6-c-Pent, 10)-Product (R) alternative conformation
Electronic energy (Hartrees): -1870.539329
Free Energy at 1M concentration and 253 K (Hartrees): -1868.883711
Coordinates:

P	0.11180	-0.19687	0.14246
O	-1.03273	0.84620	0.65238
O	1.44381	0.66335	-0.24732
O	-0.31266	-0.68546	-1.29370
O	0.27625	-1.23015	1.19334
C	-1.07113	2.07012	0.03579
C	-2.32276	2.48211	-0.64647
C	-2.40989	3.69897	-1.20359
H	-3.32669	4.01091	-1.68950
C	-1.25275	4.61673	-1.17760
C	-1.32401	5.91031	-1.89519
H	-2.23453	6.20002	-2.40489
C	-0.26183	6.72266	-1.92353
H	-0.31339	7.66579	-2.45201
C	0.98833	6.33049	-1.24497
H	1.85169	6.98168	-1.29162
C	1.05959	5.16943	-0.58427
H	1.99408	4.89199	-0.11476
C	-0.12139	4.26518	-0.52349
C	-0.06149	2.95580	0.19120
C	1.84117	1.51715	0.74820
C	3.15876	1.31278	1.39565
C	3.53417	2.10919	2.40734
H	4.51191	1.99048	2.86002
C	2.64563	3.19565	2.87046
C	3.03596	4.02036	4.03694
H	3.97168	3.83118	4.54815
C	2.23310	5.00061	4.46554
H	2.51789	5.60151	5.31954
C	0.95176	5.26086	3.78123
H	0.29819	6.04311	4.14518
C	0.59351	4.52706	2.72124

H	-0.35921	4.72128	2.24642
C	1.48697	3.44937	2.21798
C	1.10471	2.60842	1.04607
C	4.13786	0.37545	0.79898
C	4.93040	0.82807	-0.39482
C	4.37262	-0.84746	1.34703
C	5.91605	0.03868	-0.86058
C	5.50037	-1.66469	0.82425
C	6.24506	-1.24955	-0.21144
H	6.49384	0.34623	-1.72096
H	5.74455	-2.59839	1.31330
C	-3.48717	1.56285	-0.68339
C	-4.24408	1.34868	0.43009
C	-3.86458	0.90547	-1.98251
C	-5.43396	0.46184	0.33360
C	-4.99213	0.17189	-2.03867
C	-5.81219	-0.07819	-0.83356
H	-6.02417	0.27490	1.22116
H	-5.30906	-0.26447	-2.97519
H	-0.40388	-1.71434	-1.33509
C	1.04220	-3.68492	-1.18904
C	-1.19271	-4.13649	-0.70361
C	2.01908	-2.78915	-1.69954
C	1.46177	-4.95050	-0.67463
C	-0.85758	-5.42217	-0.20953
C	3.35104	-3.13020	-1.66633
H	1.70284	-1.83150	-2.09471
C	2.84430	-5.27070	-0.65868
C	0.45745	-5.81825	-0.18511
H	-1.64311	-6.06898	0.16461
C	3.77188	-4.37501	-1.13729
H	4.08495	-2.42695	-2.04546
H	3.15507	-6.22989	-0.25426
H	0.73743	-6.78970	0.21324
H	4.82858	-4.62018	-1.10685
C	-2.64217	-3.68912	-0.65450
H	-3.23326	-4.48102	-0.19332
C	-2.87906	-2.37574	0.11243
H	-3.94676	-2.15788	0.10683
H	-2.39775	-1.55003	-0.41599
N	-2.38312	-2.49110	1.46427
N	-0.27370	-3.30731	-1.17648
C	-3.19136	-2.49730	2.59404
C	-4.57311	-2.76317	2.55757
C	-2.59680	-2.23515	3.84691
C	-5.32865	-2.73440	3.72991
H	-5.06637	-2.99435	1.61966
C	-3.36160	-2.21648	5.00629
H	-1.53180	-2.02184	3.88674
C	-4.73801	-2.45961	4.96214
H	-6.39550	-2.93350	3.67093
H	-2.87784	-1.99857	5.95479
H	-5.33351	-2.43776	5.86922
H	-1.44299	-2.12508	1.59771
Cl	-3.31303	-3.53718	-2.35823
C	-3.95491	2.02283	1.77350
C	-5.12557	2.92631	2.21096
C	-3.63968	0.97843	2.86189
H	-3.06884	2.68573	1.70497
C	-4.81838	3.60346	3.55380

H	-6.06311	2.33881	2.31367
H	-5.29505	3.71067	1.44130
C	-3.33306	1.65903	4.20119
H	-4.49516	0.28330	3.00050
H	-2.75670	0.37992	2.55616
C	-4.49359	2.56350	4.63450
H	-5.69896	4.20125	3.87319
H	-3.95843	4.29899	3.43448
H	-3.16369	0.88198	4.97722
H	-2.40169	2.26083	4.11094
H	-4.21864	3.08639	5.57558
H	-5.39391	1.94268	4.83778
C	-7.07273	-0.92226	-0.91835
C	-8.11608	-0.25148	-1.83415
C	-6.76183	-2.35346	-1.40301
H	-7.52879	-1.00964	0.09487
C	-9.39266	-1.09734	-1.92334
H	-7.70507	-0.11103	-2.85774
H	-8.37565	0.75103	-1.42919
C	-8.04289	-3.19190	-1.49463
H	-6.27405	-2.33357	-2.40138
H	-6.06741	-2.84691	-0.69178
C	-9.07774	-2.51902	-2.40464
H	-10.10107	-0.61860	-2.63316
H	-9.88407	-1.14016	-0.92640
H	-7.79535	-4.19620	-1.90091
H	-8.47274	-3.33086	-0.47817
H	-10.01170	-3.12106	-2.40544
H	-8.69169	-2.48375	-3.44724
C	-3.00882	1.07273	-3.23759
C	-2.60309	-0.28216	-3.85828
C	-3.72486	1.94575	-4.28624
H	-2.06045	1.58520	-2.97445
C	-1.70034	-0.06742	-5.07911
H	-3.49620	-0.85864	-4.18027
H	-2.06139	-0.89286	-3.10939
C	-2.83237	2.15698	-5.51720
H	-4.67784	1.46815	-4.60347
H	-3.96927	2.93624	-3.84600
C	-2.39629	0.81589	-6.12353
H	-1.45778	-1.05195	-5.53386
H	-0.74555	0.40698	-4.76166
H	-3.39200	2.73947	-6.28025
H	-1.93355	2.74602	-5.22968
H	-1.69814	1.00491	-6.96706
H	-3.28547	0.28400	-6.52804
C	4.62154	2.14835	-1.10198
C	5.83463	3.10032	-1.11273
C	4.09980	1.90813	-2.53231
H	3.81994	2.69094	-0.56498
C	5.47117	4.42872	-1.79100
H	6.69216	2.64523	-1.65333
H	6.15512	3.30356	-0.06762
C	3.74057	3.23809	-3.20812
H	4.86494	1.38392	-3.14541
H	3.19209	1.26684	-2.49406
C	4.93985	4.19568	-3.21205
H	6.37351	5.07545	-1.83838
H	4.70176	4.95963	-1.18764
H	3.42277	3.04353	-4.25494

H	2.88621	3.71053	-2.67458
H	4.62969	5.16833	-3.65082
H	5.74972	3.77559	-3.84825
C	3.53653	-1.40578	2.49707
C	2.95764	-2.79473	2.15278
C	4.35993	-1.47667	3.79619
H	2.66549	-0.74622	2.69804
C	2.09303	-3.32327	3.30328
H	3.76740	-3.52891	1.95634
H	2.34045	-2.72493	1.23533
C	3.50508	-2.01642	4.95199
H	5.24638	-2.13408	3.66039
H	4.72360	-0.46007	4.06136
C	2.90145	-3.38513	4.60600
H	1.72577	-4.34077	3.04820
H	1.20687	-2.66583	3.44291
H	4.13703	-2.11390	5.86076
H	2.68841	-1.29636	5.17993
H	2.23678	-3.71252	5.43408
H	3.71582	-4.13577	4.50358
C	7.43706	-2.06751	-0.67968
C	7.29552	-2.48284	-2.15877
C	8.74901	-1.28870	-0.45728
H	7.50141	-3.00436	-0.07940
C	8.51072	-3.29931	-2.61694
H	7.19699	-1.58959	-2.81236
H	6.38477	-3.10274	-2.28791
C	9.95971	-2.10852	-0.92124
H	8.73017	-0.32610	-1.01395
H	8.86437	-1.05646	0.62401
C	9.81335	-2.52226	-2.39069
H	8.40774	-3.53515	-3.69801
H	8.54729	-4.26115	-2.05942
H	10.88208	-1.50035	-0.80183
H	10.06349	-3.01556	-0.28586
H	10.67584	-3.16061	-2.67943
H	9.82541	-1.61693	-3.03683

Catalyst (2,4,6-c-Pent, 10)-Product (R) alternative conformation
Electronic energy (Hartrees): -1870.538474
Free Energy at 1M concentration and 253 K (Hartrees): -1868.883341
Coordinates:

P	0.15264	-0.14114	0.04065
O	-1.04838	0.80506	0.61129
O	1.44539	0.81001	-0.25837
O	-0.23004	-0.53656	-1.43345
O	0.35607	-1.24303	1.01260
C	-1.11989	2.08230	0.11675
C	-2.36798	2.51283	-0.56114
C	-2.49203	3.77649	-0.99281
H	-3.40645	4.09938	-1.47619
C	-1.37690	4.73197	-0.83754
C	-1.48433	6.08820	-1.42284
H	-2.39344	6.39271	-1.92636
C	-0.45621	6.93958	-1.33966
H	-0.53385	7.92875	-1.77201
C	0.79235	6.52894	-0.66941
H	1.62934	7.21396	-0.62662
C	0.89530	5.31050	-0.12687
H	1.82938	5.02348	0.33733

C	-0.24842	4.35935	-0.19063
C	-0.15143	2.98633	0.38841
C	1.80203	1.57682	0.82008
C	3.12826	1.37279	1.44854
C	3.46158	2.07316	2.54275
H	4.44444	1.95754	2.98507
C	2.51932	3.05927	3.11222
C	2.86565	3.77386	4.36216
H	3.80679	3.57491	4.85954
C	2.01631	4.66635	4.88313
H	2.26906	5.18671	5.79791
C	0.72781	4.93902	4.21708
H	0.03769	5.64929	4.65388
C	0.40873	4.30467	3.08297
H	-0.54884	4.50501	2.62044
C	1.35291	3.32665	2.47938
C	1.01551	2.59661	1.22270
C	4.15728	0.56624	0.75430
C	4.91682	1.19233	-0.38189
C	4.46221	-0.69485	1.16239
C	5.94757	0.51862	-0.92606
C	5.64145	-1.37641	0.56630
C	6.36329	-0.80136	-0.40890
H	6.49829	0.94342	-1.75506
H	5.94223	-2.33974	0.95383
C	-3.48586	1.55514	-0.74554
C	-4.28048	1.19779	0.30254
C	-3.77980	1.02547	-2.12273
C	-5.43734	0.29818	0.05502
C	-4.87698	0.26735	-2.30947
C	-5.74352	-0.12622	-1.18046
H	-6.05909	0.00151	0.88817
H	-5.13458	-0.08500	-3.29909
H	-0.31951	-1.56248	-1.55004
C	1.10921	-3.54881	-1.66311
C	-1.12478	-4.03150	-1.19969
C	2.09508	-2.60509	-2.05842
C	1.50976	-4.88881	-1.36870
C	-0.80625	-5.38361	-0.91652
C	3.41634	-2.98092	-2.13779
H	1.79370	-1.58873	-2.28110
C	2.88045	-5.24206	-1.46354
C	0.49911	-5.80294	-0.98949
H	-1.59738	-6.06624	-0.62760
C	3.81550	-4.30675	-1.83972
H	4.16013	-2.24682	-2.43016
H	3.17544	-6.26226	-1.23472
H	0.76672	-6.83006	-0.75676
H	4.86071	-4.58705	-1.91297
C	-2.56737	-3.58212	-1.06037
H	-3.15368	-4.42321	-0.68888
C	-2.77812	-2.37441	-0.13176
H	-3.84735	-2.16417	-0.07290
H	-2.31596	-1.48867	-0.57200
N	-2.23383	-2.64464	1.17903
N	-0.19842	-3.15418	-1.55850
C	-3.01893	-2.86320	2.30343
C	-4.35680	-3.29365	2.22893
C	-2.45433	-2.65850	3.57980
C	-5.10714	-3.47423	3.39048

H	-4.81303	-3.49920	1.26668
C	-3.21128	-2.85277	4.72870
H	-1.42340	-2.32141	3.64928
C	-4.54847	-3.25423	4.64858
H	-6.14120	-3.79734	3.30298
H	-2.75443	-2.67537	5.69871
H	-5.13829	-3.39644	5.54848
H	-1.31851	-2.23280	1.34734
Cl	-3.27923	-3.21596	-2.71472
C	-4.06374	1.71577	1.72593
C	-5.27826	2.52875	2.21837
C	-3.75612	0.55658	2.69451
H	-3.19676	2.40531	1.77199
C	-5.04508	3.04643	3.64430
H	-6.20064	1.90925	2.21049
H	-5.44194	3.39558	1.54153
C	-3.52581	1.07860	4.11774
H	-4.59043	-0.17737	2.71127
H	-2.84123	0.02670	2.35755
C	-4.73120	1.89207	4.60533
H	-5.95561	3.57812	3.99508
H	-4.20302	3.77335	3.64598
H	-3.36246	0.21872	4.80216
H	-2.61161	1.71216	4.14076
H	-4.51122	2.30404	5.61358
H	-5.61867	1.22721	4.69287
C	-6.95798	-1.00043	-1.44148
C	-6.85233	-2.35216	-0.70610
C	-8.26350	-0.26999	-1.06441
H	-7.00702	-1.22218	-2.53168
C	-8.08182	-3.22598	-0.98290
H	-6.76437	-2.19458	0.39063
H	-5.94664	-2.89384	-1.05319
C	-9.48587	-1.15399	-1.34236
H	-8.25992	0.00879	0.01191
H	-8.35115	0.66491	-1.65985
C	-9.37489	-2.49369	-0.60394
H	-8.00485	-4.16393	-0.39186
H	-8.11011	-3.50180	-2.06007
H	-10.40431	-0.62576	-1.00720
H	-9.57577	-1.33587	-2.43599
H	-10.24695	-3.13028	-0.86660
H	-9.39688	-2.31832	0.49429
C	-2.87168	1.34764	-3.30913
C	-2.39478	0.07876	-4.04979
C	-3.56414	2.30924	-4.29404
H	-1.95320	1.85332	-2.94548
C	-1.44016	0.44835	-5.19160
H	-3.25298	-0.48073	-4.47874
H	-1.87284	-0.59733	-3.34437
C	-2.62052	2.67626	-5.44798
H	-4.48478	1.84286	-4.70855
H	-3.86142	3.23952	-3.76422
C	-2.11198	1.42095	-6.17052
H	-1.14417	-0.47426	-5.73576
H	-0.51777	0.91066	-4.77571
H	-3.16132	3.32229	-6.17249
H	-1.75634	3.25509	-5.05343
H	-1.37967	1.71893	-6.95130
H	-2.96257	0.91248	-6.67564

C	4.51993	2.55588	-0.94973
C	5.67109	3.57866	-0.86980
C	3.99786	2.42974	-2.39444
H	3.69320	2.99048	-0.35596
C	5.21702	4.94366	-1.40628
H	6.54842	3.23609	-1.45935
H	5.99106	3.69426	0.18875
C	3.54794	3.79583	-2.92913
H	4.78753	2.02035	-3.06145
H	3.13268	1.73154	-2.41761
C	4.68407	4.82377	-2.84076
H	6.07650	5.64781	-1.39218
H	4.42336	5.36052	-0.74735
H	3.23137	3.68879	-3.98886
H	2.67137	4.15574	-2.34644
H	4.30762	5.81434	-3.17519
H	5.51108	4.52400	-3.52141
C	3.65092	-1.43438	2.22403
C	3.14322	-2.79365	1.69576
C	4.47249	-1.63076	3.51170
H	2.74782	-0.84918	2.49966
C	2.30924	-3.51499	2.76127
H	3.98965	-3.45181	1.40543
H	2.51972	-2.63398	0.79314
C	3.64452	-2.36135	4.57838
H	5.39362	-2.21709	3.30212
H	4.77984	-0.63942	3.91038
C	3.11909	-3.70378	4.05062
H	1.99794	-4.50850	2.37228
H	1.38850	-2.93079	2.98005
H	4.27763	-2.54207	5.47351
H	2.78857	-1.72240	4.88905
H	2.47318	-4.17373	4.82304
H	3.97421	-4.38813	3.85670
C	7.61456	-1.45847	-0.96578
C	8.69501	-1.61323	0.12460
C	7.30035	-2.81693	-1.62319
H	8.03769	-0.79949	-1.75756
C	9.96209	-2.26148	-0.44758
H	8.31860	-2.23922	0.96289
H	8.95507	-0.61284	0.53437
C	8.57296	-3.46185	-2.18655
H	6.84369	-3.51088	-0.88491
H	6.57880	-2.66776	-2.45449
C	9.64255	-3.61329	-1.09786
H	10.69812	-2.41338	0.37100
H	10.42245	-1.58393	-1.20003
H	8.32445	-4.46373	-2.59820
H	8.97141	-2.83979	-3.01810
H	10.56818	-4.03086	-1.54908
H	9.28749	-4.32679	-0.32187

Catalyst (2,4,6-c-Pent, 10)-Product (R) alternative conformation
Electronic energy (Hartrees): -1870.538565
Free Energy at 1M concentration and 253 K (Hartrees): -1868.883287
Coordinates:

P	0.16082	-0.15714	0.08923
O	-1.02367	0.82946	0.62530
O	1.43362	0.76721	-0.34761
O	-0.27614	-0.68371	-1.32994

O	0.40892	-1.17181	1.14231
C	-1.13313	2.05764	0.02414
C	-2.42423	2.42605	-0.60840
C	-2.57808	3.64032	-1.15662
H	-3.52380	3.91909	-1.60625
C	-1.45853	4.60325	-1.16385
C	-1.60462	5.89586	-1.87185
H	-2.54105	6.14967	-2.35286
C	-0.57815	6.75159	-1.92730
H	-0.68422	7.69384	-2.44919
C	0.70639	6.40911	-1.28731
H	1.54033	7.09582	-1.35551
C	0.84487	5.24950	-0.63483
H	1.80327	5.00990	-0.19376
C	-0.29484	4.29585	-0.54568
C	-0.15855	2.98524	0.15670
C	1.82618	1.63617	0.63682
C	3.17991	1.50356	1.22481
C	3.55091	2.30900	2.23112
H	4.55286	2.24428	2.63961
C	2.62225	3.33846	2.74297
C	3.01209	4.17259	3.90278
H	3.97563	4.02995	4.37606
C	2.17461	5.10412	4.37207
H	2.45920	5.71199	5.22116
C	0.85597	5.30099	3.73908
H	0.17594	6.04386	4.13561
C	0.49750	4.55842	2.68532
H	-0.48131	4.70527	2.24780
C	1.42753	3.53485	2.13781
C	1.04661	2.68482	0.97245
C	4.18896	0.64285	0.56752
C	4.89745	1.16663	-0.65037
C	4.52466	-0.57158	1.07960
C	5.91402	0.45323	-1.17000
C	5.68651	-1.29760	0.50186
C	6.36310	-0.81068	-0.55075
H	6.42913	0.80477	-2.05436
H	6.01268	-2.21863	0.96444
C	-3.55843	1.47064	-0.59466
C	-4.26530	1.24339	0.54847
C	-3.96449	0.79265	-1.87379
C	-5.42809	0.31761	0.50481
C	-5.06780	0.02106	-1.88081
C	-5.83229	-0.24524	-0.64281
H	-5.97627	0.11821	1.41630
H	-5.40524	-0.43388	-2.80125
H	-0.35623	-1.71407	-1.34644
C	1.09002	-3.70387	-1.30367
C	-1.11972	-4.15614	-0.71448
C	2.04957	-2.79890	-1.83180
C	1.51784	-5.00201	-0.88521
C	-0.77393	-5.46687	-0.29940
C	3.37155	-3.17002	-1.92101
H	1.72836	-1.81419	-2.14888
C	2.88831	-5.35200	-0.99392
C	0.53328	-5.87985	-0.37450
H	-1.54601	-6.12227	0.08764
C	3.79747	-4.45386	-1.50112
H	4.09517	-2.46468	-2.31655

H	3.20368	-6.33999	-0.67049
H	0.82220	-6.87395	-0.04425
H	4.84249	-4.73183	-1.58452
C	-2.56519	-3.71323	-0.58831
H	-3.12582	-4.50410	-0.08901
C	-2.77623	-2.39515	0.17453
H	-3.84498	-2.18462	0.21332
H	-2.32350	-1.57039	-0.37915
N	-2.22238	-2.49603	1.50470
N	-0.21807	-3.31213	-1.19560
C	-2.98496	-2.53153	2.66491
C	-4.35846	-2.83934	2.67952
C	-2.35162	-2.25479	3.89539
C	-5.07006	-2.83579	3.87937
H	-4.87919	-3.08310	1.75999
C	-3.07276	-2.26132	5.08256
H	-1.29273	-2.00978	3.89591
C	-4.44201	-2.54547	5.08932
H	-6.13189	-3.06695	3.85972
H	-2.56061	-2.03087	6.01301
H	-5.00339	-2.54318	6.01815
H	-1.28643	-2.10919	1.60235
Cl	-3.32194	-3.59217	-2.26001
C	-3.94659	1.93959	1.87403
C	-5.13075	2.80375	2.35288
C	-3.54839	0.91701	2.95552
H	-3.08900	2.63381	1.76458
C	-4.79448	3.50254	3.67750
H	-6.04135	2.18374	2.49783
H	-5.35976	3.57510	1.58548
C	-3.21374	1.61921	4.27687
H	-4.37077	0.19145	3.13290
H	-2.65600	0.35047	2.61774
C	-4.38864	2.48375	4.75088
H	-5.68269	4.07060	4.02846
H	-3.96598	4.22775	3.51871
H	-2.98459	0.85510	5.05042
H	-2.30974	2.25423	4.14513
H	-4.09583	3.02373	5.67680
H	-5.25655	1.83191	4.99414
C	-7.06434	-1.13368	-0.67158
C	-8.16612	-0.50824	-1.55010
C	-6.72201	-2.55681	-1.15828
H	-7.47666	-1.22853	0.35956
C	-9.41443	-1.39933	-1.58146
H	-7.80109	-0.36289	-2.59031
H	-8.44475	0.48820	-1.14286
C	-7.97471	-3.44107	-1.19054
H	-6.27794	-2.52778	-2.17656
H	-5.98046	-3.01854	-0.47369
C	-9.06879	-2.81380	-2.06319
H	-10.16689	-0.95255	-2.26635
H	-9.86416	-1.44977	-0.56539
H	-7.70796	-4.43977	-1.59847
H	-8.35780	-3.58543	-0.15629
H	-9.98001	-3.44832	-2.02105
H	-8.72661	-2.77513	-3.12089
C	-3.16565	0.98155	-3.16279
C	-2.72764	-0.36251	-3.78411
C	-3.95767	1.81263	-4.19077

H	-2.23013	1.53618	-2.94290
C	-1.88554	-0.12634	-5.04369
H	-3.60788	-0.98048	-4.06154
H	-2.12914	-0.93844	-3.05069
C	-3.12517	2.04390	-5.45950
H	-4.90290	1.29424	-4.46398
H	-4.22305	2.79801	-3.75086
C	-2.66002	0.71376	-6.06818
H	-1.62070	-1.10579	-5.49698
H	-0.93930	0.39134	-4.77147
H	-3.73857	2.59360	-6.20536
H	-2.24012	2.67218	-5.21572
H	-2.00587	0.91975	-6.94245
H	-3.54259	0.14081	-6.42871
C	4.46707	2.47108	-1.32296
C	5.60951	3.50547	-1.37166
C	3.89960	2.21393	-2.73276
H	3.65511	2.94978	-0.74286
C	5.12434	4.81435	-2.01077
H	6.47076	3.11838	-1.95742
H	5.96238	3.71650	-0.33858
C	3.41894	3.52416	-3.37068
H	4.67104	1.75285	-3.38721
H	3.04113	1.51032	-2.66516
C	4.54668	4.56431	-3.41069
H	5.97653	5.52335	-2.08707
H	4.34814	5.28183	-1.36516
H	3.06964	3.32158	-4.40588
H	2.55793	3.92773	-2.79353
H	4.14941	5.51868	-3.81831
H	5.35435	4.21183	-4.08928
C	3.76713	-1.21206	2.24073
C	3.25281	-2.61901	1.86676
C	4.64464	-1.27833	3.50458
H	2.86977	-0.60988	2.49803
C	2.47350	-3.24162	3.03123
H	4.09331	-3.29571	1.60309
H	2.58893	-2.54994	0.98180
C	3.87121	-1.90986	4.67075
H	5.56285	-1.87503	3.31213
H	4.95704	-0.25114	3.79346
C	3.33966	-3.30048	4.29621
H	2.15751	-4.27001	2.75225
H	1.55621	-2.64600	3.23319
H	4.54374	-1.99946	5.55075
H	3.02179	-1.25066	4.95597
H	2.73280	-3.69957	5.13713
H	4.19375	-3.99362	4.13111
C	7.59817	-1.50802	-1.09481
C	8.72333	-1.55218	-0.04021
C	7.27323	-2.92442	-1.60901
H	7.98171	-0.92306	-1.96137
C	9.97375	-2.24096	-0.60172
H	8.38814	-2.10039	0.86723
H	8.98809	-0.51601	0.26405
C	8.52921	-3.60878	-2.16305
H	6.85639	-3.54977	-0.79034
H	6.51594	-2.85981	-2.41915
C	9.64409	-3.64980	-1.11061
H	10.74390	-2.31044	0.19636

H	10.39555	-1.63262	-1.43191
H	8.27591	-4.64653	-2.46943
H	8.88594	-3.06358	-3.06455
H	10.55546	-4.09989	-1.55959
H	9.32956	-4.29113	-0.25793

Catalyst (2,4,6-c-Hept, 12)-THF complex
Electronic energy (Hartrees): -876.298399
Free Energy at 1M concentration and 253 K (Hartrees): -874.61768
Coordinates:

P	0.00734	-0.50134	0.05576
O	-1.04753	0.32687	0.99367
O	1.17788	0.54409	-0.37554
O	-0.56982	-1.13620	-1.14729
O	0.64238	-1.45436	1.14468
C	-1.39430	1.55299	0.48975
C	-2.78021	1.77243	0.01059
C	-3.15295	2.98596	-0.42007
H	-4.16206	3.14938	-0.77963
C	-2.17651	4.09138	-0.47572
C	-2.57715	5.39178	-1.06018
H	-3.59443	5.54591	-1.39807
C	-1.68263	6.37785	-1.18243
H	-1.97508	7.32620	-1.61427
C	-0.29044	6.17168	-0.74148
H	0.43450	6.96596	-0.86440
C	0.07970	5.00359	-0.20459
H	1.11295	4.86887	0.08458
C	-0.90838	3.90198	-0.04173
C	-0.51729	2.57887	0.53441
C	1.62846	1.38439	0.61275
C	3.06533	1.34032	0.97634
C	3.54801	2.18336	1.90102
H	4.60214	2.17253	2.15125
C	2.64794	3.13104	2.58819
C	3.17095	3.98122	3.68219
H	4.21589	3.92682	3.96126
C	2.35404	4.81669	4.33294
H	2.73633	5.43594	5.13406
C	0.92587	4.88857	3.96893
H	0.26612	5.55197	4.51314
C	0.44487	4.12917	2.97785
H	-0.60964	4.18182	2.74107
C	1.34668	3.21469	2.22651
C	0.83524	2.35907	1.11470
C	3.97908	0.41844	0.25997
C	4.34479	0.71137	-1.17022
C	4.47788	-0.69149	0.87261
C	5.18980	-0.11859	-1.81116
C	5.45207	-1.54173	0.14170
C	5.78824	-1.28547	-1.13157
H	5.44009	0.05989	-2.84925
H	5.89026	-2.39132	0.64804
C	-3.72980	0.63737	-0.07060
C	-4.32688	0.13565	1.04477
C	-4.07461	0.07703	-1.42178
C	-5.34696	-0.93530	0.89480
C	-5.05516	-0.83906	-1.51839
C	-5.72451	-1.37843	-0.31353
H	-5.81006	-1.34555	1.78295

H	-5.34586	-1.22024	-2.48802
H	0.31362	-2.40352	1.05818
C	3.74212	1.89560	-1.93149
C	4.85694	2.82961	-2.46078
C	2.83803	1.38416	-3.08267
H	3.12422	2.49976	-1.24159
C	4.35582	4.22824	-2.84755
H	5.37408	2.37514	-3.33364
H	5.62300	2.96250	-1.66487
C	1.63166	2.28802	-3.39756
H	3.43676	1.23452	-4.00760
H	2.42780	0.38183	-2.82621
C	1.89473	3.80421	-3.38182
H	5.22515	4.80481	-3.23212
H	3.99480	4.76315	-1.94163
H	1.24321	2.00263	-4.39977
H	0.82013	2.07252	-2.66797
C	4.05237	-1.13077	2.27553
C	3.52715	-2.58939	2.25146
C	5.22203	-0.93831	3.25990
H	3.22074	-0.49036	2.63288
C	2.64127	-2.91476	3.46769
H	4.36068	-3.32044	2.17628
H	2.90788	-2.72749	1.33864
C	4.85868	-1.28230	4.71387
H	6.10662	-1.53121	2.94016
H	5.52834	0.13006	3.23236
C	4.79336	-2.79955	4.95360
H	2.02623	-2.03323	3.75163
H	1.91527	-3.70402	3.17227
H	3.90999	-0.78684	5.01565
H	5.65957	-0.86790	5.36456
C	6.76275	-2.17561	-1.88543
C	8.13933	-2.19523	-1.17673
C	6.15209	-3.58035	-2.07769
H	6.90474	-1.73951	-2.89939
C	9.26910	-2.68420	-2.10006
H	8.10497	-2.80235	-0.24600
H	8.38892	-1.15382	-0.87347
C	7.04730	-4.54201	-2.87548
H	5.89519	-4.03694	-1.09707
H	5.19605	-3.46640	-2.63537
C	9.48756	-4.20758	-2.08850
H	10.22002	-2.20745	-1.77547
H	9.09798	-2.33972	-3.14310
H	6.41991	-5.40979	-3.17547
H	7.40792	-4.06679	-3.81361
C	-3.34349	0.54620	-2.68106
C	-2.83564	-0.64912	-3.52657
C	-4.25659	1.47201	-3.52005
H	-2.44633	1.11603	-2.36843
C	-1.69227	-0.27327	-4.47869
H	-3.66280	-1.10098	-4.11603
H	-2.46129	-1.44744	-2.85141
C	-3.50705	2.58812	-4.27425
H	-4.85330	0.87511	-4.24400
H	-5.00118	1.97063	-2.86049
C	-2.13595	2.21419	-4.86850
H	-1.40146	-1.18844	-5.03883
H	-0.79948	0.04087	-3.89415

H	-4.16506	2.95176	-5.09354
H	-3.35571	3.44587	-3.58249
C	-4.00690	0.63354	2.45607
C	-5.25898	1.26554	3.09753
C	-3.40774	-0.52006	3.29552
H	-3.25190	1.44237	2.41440
C	-5.00394	1.83916	4.50122
H	-6.09791	0.53855	3.14020
H	-5.59411	2.10171	2.44450
C	-2.62742	-0.01361	4.52116
H	-4.18972	-1.24854	3.60087
H	-2.68704	-1.07407	2.65603
C	-3.48254	0.18758	5.78531
H	-5.87030	2.48675	4.75920
H	-4.10221	2.48978	4.50946
H	-1.83669	-0.75691	4.76508
H	-2.08951	0.92933	4.28039
C	-6.81139	-2.43355	-0.44493
C	-8.02112	-1.84136	-1.19787
C	-6.23834	-3.70612	-1.11121
H	-7.16493	-2.70961	0.57453
C	-9.19247	-2.82320	-1.36184
H	-7.71631	-1.45597	-2.19498
H	-8.39133	-0.96638	-0.61883
C	-7.12830	-4.94127	-0.88909
H	-6.05027	-3.54623	-2.19517
H	-5.25679	-3.92598	-0.64048
C	-8.21688	-5.14250	-1.95765
H	-9.44669	-3.30753	-0.39398
H	-6.48184	-5.84634	-0.89552
H	-7.59565	-4.90994	0.11911
C	8.21801	-5.07544	-2.03362
H	10.12267	-4.47186	-1.21470
H	10.06723	-4.47821	-2.99815
H	7.90509	-5.21060	-0.97505
H	8.48647	-6.08880	-2.40477
C	3.40952	-3.42520	4.70114
H	5.55835	-3.32354	4.33980
H	5.06320	-2.99978	6.01367
H	2.77243	-3.25885	5.59732
H	3.54514	-4.52492	4.60675
C	3.26781	4.22102	-3.93456
H	1.76337	4.19325	-2.35026
H	1.10332	4.29026	-3.99358
H	3.17714	5.25963	-4.32115
H	3.56683	3.58295	-4.79436
C	-2.07459	0.81239	-5.49834
H	-1.89504	2.96339	-5.65408
H	-1.34868	2.32654	-4.09230
H	-3.03059	0.56021	-6.00690
H	-1.28533	0.82531	-6.28150
H	-10.08274	-2.23163	-1.66854
C	-8.92181	-3.86885	-2.45610
H	-9.89428	-4.17407	-2.90090
H	-8.33372	-3.41897	-3.28588
H	-7.76609	-5.65294	-2.83685
H	-8.98102	-5.83579	-1.54273
C	-4.90400	0.73651	5.56819
H	-3.56946	-0.78575	6.31617
H	-2.93355	0.87423	6.46636

H	-5.59428	-0.10138	5.32719
H	-5.25503	1.15228	6.53802
O	-0.36532	-3.84318	0.98420
C	-1.44362	-3.98286	1.94019
H	-1.39004	-3.11881	2.60744
H	-1.27326	-4.88829	2.53403
C	-0.85767	-4.21036	-0.32592
H	-0.24988	-3.68254	-1.06137
H	-0.74733	-5.29616	-0.45324
C	-2.75830	-4.05844	1.12867
C	-2.31815	-3.77338	-0.32304
H	-3.20061	-5.05571	1.21479
H	-3.50020	-3.33886	1.48376
H	-2.36785	-2.70717	-0.55409
H	-2.91038	-4.31822	-1.06279

Catalyst (2,4,6-c-Hept, 12)-Quinoline complex
Electronic energy (Hartrees): -1582.786798
Free Energy at 1M concentration and 253 K (Hartrees): -1581.067554
Coordinates:

P	0.19517	0.01240	-0.59324
O	-0.97203	0.01824	0.58213
O	1.39244	0.99909	-0.09640
O	-0.28043	0.40216	-1.93329
O	0.76894	-1.44274	-0.39028
C	-1.20520	1.24060	1.15840
C	-2.52992	1.88055	0.97943
C	-2.79727	3.05019	1.57603
H	-3.76310	3.52270	1.43963
C	-1.77058	3.72052	2.39657
C	-2.05464	5.05207	2.97935
H	-3.02115	5.51685	2.82891
C	-1.11692	5.68973	3.68752
H	-1.32384	6.66468	4.10961
C	0.20668	5.07080	3.88719
H	0.96803	5.60063	4.44504
C	0.47112	3.86537	3.37062
H	1.45800	3.44603	3.51029
C	-0.56608	3.13448	2.59167
C	-0.28963	1.79664	1.98270
C	1.80073	0.78081	1.19645
C	3.19226	0.33081	1.44579
C	3.60302	0.10197	2.70231
H	4.62592	-0.19769	2.89554
C	2.66721	0.25843	3.83423
C	3.09583	-0.11589	5.20130
H	4.10149	-0.47746	5.37617
C	2.24034	-0.01581	6.22458
H	2.55297	-0.29340	7.22291
C	0.86381	0.46356	5.99451
H	0.17092	0.52387	6.82386
C	0.46960	0.81293	4.76461
H	-0.55002	1.14244	4.61382
C	1.41666	0.72832	3.62051
C	0.99496	1.09204	2.23642
C	4.15097	0.22138	0.31959
C	4.66023	1.47735	-0.33558
C	4.58021	-0.99396	-0.12344
C	5.55721	1.37517	-1.33506
C	5.62111	-1.04850	-1.18222

C	6.08219	0.06558	-1.76957
H	5.90951	2.26575	-1.83975
H	6.00863	-2.01065	-1.48880
C	-3.54665	1.24610	0.11276
C	-4.34004	0.24591	0.58316
C	-3.75491	1.78321	-1.27405
C	-5.38947	-0.31699	-0.30645
C	-4.77172	1.30353	-2.01313
C	-5.61549	0.18869	-1.52826
H	-6.00446	-1.12973	0.05792
H	-4.97644	1.72103	-2.98950
H	0.07037	-2.16514	-0.20318
C	4.14991	2.86403	0.06624
C	5.32477	3.77172	0.50510
C	3.35331	3.49435	-1.10470
H	3.47816	2.77142	0.93960
C	4.88582	5.00466	1.30701
H	5.91872	4.10479	-0.37355
H	6.01053	3.18331	1.15434
C	2.19861	4.41496	-0.67003
H	4.03624	4.05422	-1.78036
H	2.90243	2.69221	-1.73101
C	2.48535	5.35550	0.51353
H	5.79777	5.59192	1.55101
H	4.44366	4.68613	2.27666
H	1.90139	5.02729	-1.54940
H	1.31925	3.78733	-0.40497
C	4.01404	-2.31906	0.39279
C	3.49810	-3.18153	-0.78909
C	5.07758	-3.04632	1.23879
H	3.14825	-2.12323	1.05817
C	2.52196	-4.28016	-0.33555
H	4.33736	-3.62980	-1.36303
H	2.94948	-2.51860	-1.49411
C	4.57702	-4.36468	1.85255
H	5.99603	-3.23192	0.64035
H	5.37465	-2.37712	2.07495
C	4.52271	-5.50213	0.81906
H	1.90357	-3.91937	0.50927
H	1.81116	-4.48902	-1.16507
H	3.59277	-4.22540	2.35103
H	5.29939	-4.65884	2.64509
C	7.12826	0.00341	-2.87064
C	8.42821	-0.65514	-2.34712
C	6.54320	-0.69897	-4.11456
H	7.36868	1.04835	-3.16852
C	9.64497	-0.34192	-3.23561
H	8.30386	-1.75344	-2.22755
H	8.64373	-0.24300	-1.33593
C	7.51526	-0.76592	-5.30339
H	6.19245	-1.72317	-3.86147
H	5.64635	-0.12772	-4.44214
C	9.86547	-1.33699	-4.38875
H	10.55789	-0.35815	-2.60060
H	9.57675	0.69067	-3.64181
H	6.92288	-1.04228	-6.20303
H	7.96502	0.22991	-5.50840
C	-2.88229	2.91483	-1.81914
C	-2.37527	2.61049	-3.25146
C	-3.65884	4.25294	-1.77605

H	-1.98424	3.00055	-1.17553
C	-1.13388	3.42507	-3.63950
H	-3.17333	2.79106	-4.00399
H	-2.11030	1.53448	-3.32822
C	-2.77957	5.48958	-1.50685
H	-4.22587	4.40232	-2.72084
H	-4.42561	4.22133	-0.97006
C	-1.38996	5.50067	-2.17050
H	-0.85100	3.14058	-4.67629
H	-0.27729	3.14676	-2.98649
H	-3.34165	6.39010	-1.83788
H	-2.63350	5.59030	-0.40863
C	-4.25581	-0.27315	2.02155
C	-5.53372	0.13103	2.78548
C	-3.99044	-1.79739	2.03547
H	-3.41113	0.20827	2.55385
C	-5.53808	-0.29995	4.26052
H	-6.44032	-0.26426	2.27809
H	-5.61239	1.24070	2.75802
C	-3.47042	-2.29202	3.39796
H	-4.89193	-2.37210	1.73278
H	-3.20227	-2.01206	1.28267
C	-4.56916	-2.69102	4.39802
H	-6.35413	0.25882	4.76885
H	-4.58940	-0.01245	4.76418
H	-2.84261	-3.19117	3.23086
H	-2.79787	-1.53659	3.85975
C	-6.72471	-0.37132	-2.40375
C	-7.83540	0.68490	-2.57081
C	-6.14700	-0.86047	-3.75298
H	-7.17852	-1.24591	-1.88450
C	-9.03064	0.19628	-3.40537
H	-7.42607	1.61985	-3.01210
H	-8.21475	0.94926	-1.55877
C	-7.09101	-1.83491	-4.47830
H	-5.88313	-0.00682	-4.41425
H	-5.20322	-1.40690	-3.54696
C	-8.11413	-1.15836	-5.40842
H	-9.38687	-0.79518	-3.04996
H	-6.47653	-2.52298	-5.09952
H	-7.62247	-2.48140	-3.74654
C	8.59910	-1.83821	-5.10500
H	10.41270	-2.22275	-3.99795
H	10.53220	-0.85395	-5.13633
H	8.18407	-2.71327	-4.55839
H	8.90558	-2.21904	-6.10388
C	3.19030	-5.60925	0.05581
H	5.36020	-5.40685	0.09371
H	4.68518	-6.46659	1.34826
H	2.46858	-6.18714	0.67390
H	3.36906	-6.20463	-0.86618
C	3.91317	5.92413	0.54975
H	2.26008	4.83160	1.46530
H	1.76689	6.20229	0.45412
H	3.87906	6.89297	1.09434
H	4.28594	6.14375	-0.47425
C	-1.36249	4.94527	-3.60435
H	-1.03720	6.55493	-2.19441
H	-0.66679	4.95365	-1.52882
H	-2.28626	5.22106	-4.15824

H	-0.51129	5.42189	-4.13790
H	-9.86467	0.91415	-3.24528
C	-8.71491	0.16799	-4.90981
H	-9.65899	0.34152	-5.47133
H	-8.03917	1.00861	-5.18072
H	-7.63180	-0.96759	-6.39213
H	-8.94068	-1.87916	-5.59277
C	-5.82346	-1.80148	4.42136
H	-4.89117	-3.73289	4.17932
H	-4.11933	-2.70278	5.41500
H	-6.53895	-2.14698	3.64325
H	-6.33187	-1.96155	5.39737
C	-1.95897	-3.51181	-0.98996
C	-1.12090	-4.09585	1.10859
C	-1.89880	-2.67223	-2.13371
C	-2.94942	-4.54243	-0.92958
C	-2.05397	-5.16047	1.22735
C	-2.79094	-2.85935	-3.16524
H	-1.16280	-1.87846	-2.19015
C	-3.85557	-4.70021	-2.00866
C	-2.96351	-5.36695	0.22138
H	-2.05065	-5.77549	2.11848
C	-3.77515	-3.87580	-3.10719
H	-2.74102	-2.20974	-4.03348
H	-4.60754	-5.48239	-1.95307
H	-3.70236	-6.15995	0.29965
H	-4.46922	-3.99888	-3.93283
N	-1.07080	-3.31693	0.03402
C	-0.20244	-3.76760	2.23078
C	0.10100	-2.53116	2.62583
H	-0.34425	-1.68173	2.12068
H	0.77931	-2.35350	3.45184
Cl	0.47632	-5.15792	3.08793

Catalyst (2,4,6-c-Hept, 12)-Enamine protonation TS (pro-S) lowest free energy conformation
Electronic energy (Hartrees): -1870.392126
Free Energy at 1M concentration and 253 K (Hartrees): -1868.641941
Coordinates:

P	-0.28614	-0.32169	0.17593
O	1.06793	0.53954	-0.18226
O	-1.38819	0.71722	0.81798
O	0.00275	-1.27452	1.36271
O	-0.80415	-0.97345	-1.07558
C	1.50594	1.38927	0.81152
C	2.92274	1.32800	1.27977
C	3.33736	2.15463	2.25637
H	4.37373	2.15198	2.57235
C	2.40892	3.12132	2.86764
C	2.84306	3.94179	4.02149
H	3.84209	3.82922	4.42409
C	2.00046	4.82261	4.57306
H	2.31621	5.42038	5.41829
C	0.63455	4.97747	4.03486
H	-0.04799	5.67989	4.49561
C	0.23823	4.24663	2.98638
H	-0.77251	4.36075	2.61592
C	1.17189	3.27709	2.35316
C	0.75231	2.44304	1.19202
C	-1.44550	1.96411	0.24118

C	-2.72897	2.44763	-0.33177
C	-2.84030	3.71320	-0.76733
H	-3.78646	4.07633	-1.15204
C	-1.66327	4.60138	-0.77928
C	-1.75226	5.93846	-1.40984
H	-2.69225	6.28427	-1.82164
C	-0.66919	6.72020	-1.48231
H	-0.73303	7.69492	-1.94846
C	0.61960	6.25152	-0.93739
H	1.49787	6.87883	-1.01988
C	0.70576	5.05143	-0.35257
H	1.66551	4.71912	0.02022
C	-0.49575	4.17882	-0.24663
C	-0.42293	2.82784	0.38479
C	-3.90922	1.56019	-0.33153
C	-4.58846	1.26326	0.97526
C	-4.37263	1.00464	-1.48635
C	-5.61892	0.40092	0.99121
C	-5.48836	0.02343	-1.40915
C	-6.07832	-0.27570	-0.24020
H	-6.13097	0.19207	1.92062
H	-5.82926	-0.45450	-2.31867
C	3.94296	0.52273	0.56396
C	4.37997	0.89994	-0.67363
C	4.66444	-0.56778	1.30759
C	5.55832	0.21131	-1.26562
C	5.76148	-1.12098	0.75624
C	6.25258	-0.70543	-0.57529
H	5.91178	0.52602	-2.23921
H	6.32355	-1.86563	1.30364
H	0.09646	-2.39722	0.99130
C	0.13475	-3.72533	0.44773
C	0.85442	-3.63381	-0.90322
H	0.14733	-3.26044	-1.64045
H	1.14034	-4.65388	-1.21693
N	1.99930	-2.73056	-0.91080
H	2.74719	-3.09783	-0.33292
C	2.49160	-2.33412	-2.17342
C	3.85370	-2.47748	-2.46801
C	1.64805	-1.76173	-3.14279
C	4.37074	-2.05523	-3.69191
H	4.49657	-2.93094	-1.71893
C	2.16853	-1.36623	-4.37223
H	0.60752	-1.57337	-2.90310
C	3.52888	-1.50762	-4.65940
H	5.43201	-2.16686	-3.89518
H	1.50432	-0.91633	-5.10557
H	3.92700	-1.18497	-5.61653
C	-1.23790	-4.15150	0.36231
C	-1.84251	-5.20842	1.11313
C	-3.31938	-3.87862	-0.84465
C	-3.11681	-5.61566	0.84199
H	-1.24033	-5.70721	1.85904
C	-4.03333	-3.22200	-1.86144
C	-3.88998	-4.99773	-0.18615
H	-3.54671	-6.44986	1.38990
C	-5.27788	-3.69141	-2.23885
H	-3.58139	-2.37659	-2.35617
C	-5.16667	-5.44954	-0.58759
C	-5.84864	-4.81475	-1.60821

H	-5.81680	-3.18693	-3.03456
H	-5.59876	-6.30896	-0.08335
H	-6.82490	-5.17176	-1.91849
N	-2.04305	-3.47819	-0.50805
H	-1.67381	-2.58149	-0.88881
Cl	1.12122	-4.72085	1.60847
C	-4.15698	1.93896	2.27941
C	-5.28202	2.84323	2.81993
C	-3.67778	0.88348	3.30787
H	-3.30638	2.61751	2.07680
C	-4.86148	3.61140	4.08017
H	-6.20935	2.26370	3.01778
H	-5.52995	3.59048	2.03413
C	-2.78667	1.49712	4.40412
H	-4.53728	0.34182	3.75921
H	-3.06846	0.12133	2.77434
C	-3.55564	2.03409	5.62573
H	-5.55551	4.46841	4.22176
H	-2.08433	0.71438	4.76594
H	-2.14793	2.30449	3.98351
C	-3.80744	1.36004	-2.86918
C	-3.19980	0.11641	-3.55462
C	-4.89719	2.04880	-3.71466
H	-2.98511	2.09358	-2.77404
C	-2.25889	0.47547	-4.72043
H	-3.99239	-0.58461	-3.89441
H	-2.58177	-0.40950	-2.80013
C	-4.39469	2.48038	-5.09895
H	-5.79489	1.40222	-3.81982
H	-5.22345	2.96402	-3.17329
C	-2.95961	0.59675	-6.08485
H	-1.48714	-0.32097	-4.80895
H	-1.70314	1.41387	-4.50338
H	-5.08823	3.24872	-5.50500
C	4.22815	-1.00727	2.70687
C	3.83657	-2.49964	2.71284
C	5.32315	-0.66642	3.74211
H	3.30980	-0.45657	2.99667
C	3.31983	-3.00040	4.07359
H	4.68093	-3.13898	2.37687
H	3.01883	-2.62981	1.97510
C	4.72907	-0.61243	5.15171
H	6.17342	-1.38079	3.69109
H	5.73276	0.34170	3.51342
C	4.45361	-3.19481	5.09468
H	2.84204	-3.98979	3.90799
H	2.53198	-2.32430	4.47233
H	5.21476	0.18560	5.75441
C	3.79803	2.09546	-1.43880
C	4.87995	3.16599	-1.69737
C	3.11767	1.61187	-2.73259
H	3.01288	2.59646	-0.84386
C	4.22808	4.52891	-1.95426
H	5.55486	2.88225	-2.53356
H	5.50700	3.26363	-0.78346
C	2.39338	2.72921	-3.50144
H	3.85196	1.10878	-3.39743
H	2.35685	0.85322	-2.45603
C	3.37325	3.64616	-4.25193
H	4.84909	5.35024	-1.53524

H	1.73383	2.24308	-4.25361
H	1.73363	3.31577	-2.82447
C	7.58769	-1.21983	-1.09633
C	8.71957	-0.74078	-0.16195
C	7.55682	-2.75554	-1.26770
H	7.77407	-0.78286	-2.10474
C	10.07483	-0.81929	-0.86927
H	8.72912	-1.30950	0.79359
H	8.54106	0.32733	0.09494
C	8.86761	-3.35308	-1.80814
H	7.28357	-3.25808	-0.31528
H	6.76435	-3.00387	-2.00110
C	9.97037	-3.40856	-0.73730
H	10.72450	0.03216	-0.57082
H	8.65223	-4.39566	-2.12976
H	9.21184	-2.80204	-2.71075
C	-7.22870	-1.26725	-0.17953
C	-6.89871	-2.40752	0.80896
C	-8.54030	-0.52894	0.17884
H	-7.35135	-1.72683	-1.18638
C	-7.97283	-3.50000	0.85560
H	-6.74261	-2.01276	1.83603
H	-5.93830	-2.87111	0.50327
C	-9.79366	-1.34957	-0.17843
H	-8.55498	-0.23776	1.25226
H	-8.58427	0.41298	-0.41262
C	-9.15731	-3.03380	1.70356
H	-7.52802	-4.39431	1.34376
H	-8.29321	-3.80568	-0.16377
H	-9.59491	-3.88488	2.26929
C	-4.32512	1.30206	-6.07864
C	-10.26554	-2.30795	0.92962
C	-4.89608	2.72725	5.33297
C	4.77812	-1.93826	5.92105
C	10.83128	-2.13625	-0.65818
C	3.89197	4.83270	-3.42054
H	4.05269	-1.86322	6.75920
H	5.78851	-2.06041	6.36834
H	4.15726	-3.99806	5.80434
H	5.37416	-3.55698	4.58676
H	3.12266	5.63450	-3.42368
H	4.79681	5.24202	-3.92002
H	2.85425	4.06142	-5.14344
H	4.23287	3.05870	-4.64241
H	-3.75114	1.19115	6.32430
H	-2.89277	2.74744	6.16303
H	-5.70538	1.96795	5.26057
H	-5.14714	3.36382	6.20935
H	-10.62773	-0.64464	-0.38868
H	-9.63205	-1.91843	-1.11987
H	-10.86362	-1.73314	1.66874
H	-10.94250	-3.06390	0.47546
H	-5.12863	0.56293	-5.86663
H	-4.51715	1.68185	-7.10585
H	-3.09785	-0.42172	-6.50959
H	-2.27713	1.14150	-6.77358
H	11.33722	-2.11040	0.33142
H	11.62481	-2.19172	-1.43387
H	9.53032	-3.62483	0.26082
H	10.64624	-4.26104	-0.96769

Catalyst (2,4,6-c-Hept, 12)-Enamine protonation TS (pro-S) alternative conformation
Electronic energy (Hartrees): -1870.393279
Free Energy at 1M concentration and 253 K (Hartrees): -1868.558108
Coordinates:

P	-0.06104	0.15715	0.38303
O	1.13427	1.09619	-0.20447
O	-1.35411	1.12766	0.61586
O	0.22127	-0.48560	1.70265
O	-0.34747	-0.76911	-0.83000
C	1.39181	2.19666	0.56782
C	2.71319	2.31479	1.22373
C	2.98936	3.38435	1.98305
H	3.96456	3.48913	2.44330
C	1.96888	4.43703	2.17993
C	2.24606	5.56956	3.09274
H	3.19930	5.63665	3.60222
C	1.31888	6.51241	3.29447
H	1.52145	7.33867	3.96360
C	0.01234	6.42442	2.61366
H	-0.74000	7.18076	2.79661
C	-0.24632	5.40902	1.78152
H	-1.21745	5.35178	1.30760
C	0.78330	4.36657	1.52744
C	0.51678	3.22145	0.60765
C	-1.51940	2.15231	-0.28064
C	-2.76361	2.18817	-1.08947
C	-3.01153	3.23991	-1.88405
H	-3.92554	3.28067	-2.46396
C	-2.01904	4.32325	-2.02295
C	-2.25055	5.40625	-3.00637
H	-3.16887	5.43318	-3.57974
C	-1.32311	6.35029	-3.19740
H	-1.49110	7.13865	-3.91970
C	-0.06050	6.31104	-2.43593
H	0.69772	7.06244	-2.61505
C	0.15417	5.34278	-1.53809
H	1.10049	5.31916	-1.01491
C	-0.88087	4.30077	-1.29151
C	-0.66230	3.20154	-0.30279
C	-3.72752	1.05857	-1.03304
C	-4.57792	0.86883	0.19418
C	-3.86912	0.20779	-2.09133
C	-5.49650	-0.11490	0.20448
C	-4.95577	-0.80559	-2.06138
C	-5.72308	-0.97379	-0.97608
H	-6.09447	-0.29247	1.08910
H	-5.13260	-1.41511	-2.93758
C	3.75021	1.29853	0.93270
C	4.34710	1.25562	-0.29280
C	4.08012	0.25703	1.96433
C	5.26379	0.13075	-0.60947
C	4.96796	-0.70636	1.65422
C	5.55380	-0.81020	0.29975
H	5.68154	0.05981	-1.60561
H	5.24385	-1.44703	2.39244
H	-0.24762	-2.18040	1.78469
C	-2.02401	-2.98130	2.44032
C	-0.84045	-3.59382	0.41451

C	-2.06596	-2.16667	3.58827
C	-3.11366	-3.82719	2.11483
C	-1.94887	-4.43320	0.06066
C	-3.18092	-2.21457	4.40480
H	-1.24659	-1.48372	3.78225
C	-4.22774	-3.86587	2.98210
C	-3.02460	-4.56022	0.89222
H	-1.90450	-4.99454	-0.86259
C	-4.26289	-3.07025	4.11104
H	-3.22609	-1.57390	5.28007
H	-5.06136	-4.51800	2.73789
H	-3.83832	-5.22840	0.62295
H	-5.12622	-3.09375	4.76783
C	0.25934	-3.29217	-0.45328
C	0.43586	-3.96952	-1.80479
H	-0.55924	-4.16827	-2.22340
H	0.94421	-4.93402	-1.71329
N	1.16946	-3.13683	-2.75189
N	-0.93221	-2.94173	1.60397
C	2.53275	-3.28164	-3.02868
C	3.18642	-4.52659	-3.04696
C	3.27687	-2.13785	-3.37552
C	4.53854	-4.60774	-3.37747
H	2.64264	-5.43583	-2.81331
C	4.62349	-2.23093	-3.70756
H	2.78062	-1.17215	-3.37521
C	5.27205	-3.46783	-3.70725
H	5.02213	-5.58101	-3.37534
H	5.17140	-1.32659	-3.95813
H	6.32612	-3.54086	-3.95525
H	0.89031	-2.16461	-2.69137
H	-0.10110	-1.88506	-0.68863
Cl	1.84752	-3.09375	0.38898
C	-4.39436	1.72534	1.44760
C	-5.68211	2.51262	1.76829
C	-3.91049	0.84481	2.62685
H	-3.62103	2.49164	1.25496
C	-5.53692	3.45060	2.97862
H	-6.54149	1.82726	1.93054
H	-5.93350	3.13657	0.88209
C	-3.22278	1.66654	3.73044
H	-4.74244	0.23963	3.04820
H	-3.16062	0.12157	2.23912
C	-5.56077	2.68547	4.31259
H	-6.40497	4.14548	2.96770
H	-4.62219	4.07693	2.89116
H	-2.48034	1.01567	4.24259
H	-2.63612	2.50154	3.28862
C	-2.96092	0.25803	-3.32430
C	-2.33841	-1.12662	-3.61243
C	-3.74117	0.81154	-4.53924
H	-2.10298	0.93094	-3.12057
C	-1.31611	-1.10308	-4.76044
H	-3.11942	-1.88299	-3.83578
H	-1.82172	-1.48149	-2.69841
C	-2.80456	1.39245	-5.61467
H	-4.41140	0.03776	-4.97324
H	-4.39758	1.63971	-4.19330
C	-2.29324	0.37363	-6.65073
H	-0.58971	-0.27025	-4.63572

H	-0.73460	-2.04924	-4.70896
H	-3.35591	2.18428	-6.16781
H	-1.93925	1.90653	-5.14178
C	-6.80174	-2.04481	-0.94209
C	-6.53571	-3.03316	0.22005
C	-8.19921	-1.37776	-0.88784
H	-6.75567	-2.61580	-1.89688
C	-7.00438	-4.46194	-0.10132
H	-6.99271	-2.67405	1.16801
H	-5.44059	-3.09212	0.39726
C	-9.36556	-2.27455	-0.43387
H	-8.43044	-0.98568	-1.90310
H	-8.18233	-0.49724	-0.20916
C	-8.52396	-4.64657	-0.02448
H	-6.63516	-4.77550	-1.10226
H	-6.54302	-5.15303	0.63724
H	-9.42606	-2.27167	0.67664
H	-10.30861	-1.80581	-0.79140
C	4.09238	2.30501	-1.38087
C	5.40386	3.03211	-1.73894
C	3.41306	1.65138	-2.60832
H	3.41480	3.09375	-0.99901
C	5.21382	4.15784	-2.76966
H	6.17060	2.31608	-2.10517
H	5.80986	3.48822	-0.80883
C	2.67781	2.67715	-3.48903
H	4.14240	1.06813	-3.21068
H	2.65193	0.92918	-2.24527
C	3.55170	3.33508	-4.57275
H	4.37671	4.82938	-2.47798
H	1.83666	2.16066	-4.00213
H	2.20675	3.46464	-2.86148
C	6.44175	-1.98884	-0.06289
C	7.69810	-2.00676	0.83947
C	5.62072	-3.29329	0.01050
H	6.77610	-1.87170	-1.11898
C	8.82450	-2.88054	0.25979
H	7.44739	-2.32763	1.87393
H	8.09011	-0.96746	0.91012
C	6.41687	-4.55074	-0.37182
H	5.17779	-3.42631	1.02136
H	4.76826	-3.20238	-0.69713
C	8.76848	-4.35860	0.68530
H	9.79975	-2.47050	0.60281
H	8.84059	-2.81228	-0.84977
H	5.68413	-5.36966	-0.54258
H	6.96323	-4.40308	-1.32847
C	3.44214	0.27358	3.35457
C	2.82298	-1.10393	3.70890
C	4.47614	0.74597	4.39447
H	2.61728	1.01313	3.35979
C	1.76028	-1.00569	4.81725
H	3.60439	-1.84178	3.99151
H	2.32284	-1.50662	2.80386
C	3.89493	0.89350	5.81003
H	5.35287	0.06186	4.41240
H	4.85542	1.74301	4.07977
C	3.69243	-0.46540	6.49928
H	1.03778	-1.83975	4.68113
H	1.16773	-0.07112	4.70920

H	4.62627	1.47629	6.41175
H	2.95047	1.48037	5.79381
C	-4.17887	2.21706	4.80391
H	-5.98559	3.35339	5.09368
H	-6.24889	1.81421	4.24868
H	-4.33873	1.43176	5.57509
H	-3.67265	3.06624	5.31317
C	-9.30900	-3.71691	-0.96287
H	-8.90012	-3.75335	-1.99594
H	-10.35101	-4.10152	-1.01637
H	-8.86593	-4.51527	1.02562
H	-8.75264	-5.69755	-0.30585
C	-1.99827	-1.04708	-6.13722
H	-1.33154	-1.53945	-6.87876
H	-2.93752	-1.64239	-6.13060
H	-1.36807	0.78602	-7.11003
H	-3.04283	0.29054	-7.46808
C	7.36885	-4.99504	0.75021
H	7.49689	-6.09793	0.68774
H	6.91523	-4.79579	1.74584
H	9.39502	-4.94351	-0.02325
H	9.23950	-4.46098	1.68750
C	2.31451	-1.10450	6.25099
H	2.38185	-2.17787	6.53409
H	1.57486	-0.63994	6.93936
H	4.49390	-1.17647	6.20169
H	3.80265	-0.32283	7.59666
H	6.13888	4.77491	-2.76374
C	5.01759	3.61389	-4.19439
H	5.62434	2.69459	-4.34712
H	5.41255	4.36369	-4.91444
H	3.55072	2.68463	-5.47478
H	3.06988	4.29244	-4.86967

Catalyst (2,4,6-c-Hept, 12)-Enamine protonation TS (pro-S) alternative conformation
Electronic energy (Hartrees): -1870.348044
Free Energy at 1M concentration and 253 K (Hartrees): -1868.513316
Coordinates:

P	0.16950	0.33239	-0.38887
O	-1.14363	0.58082	0.56864
O	1.23603	1.54722	-0.12439
O	-0.10454	0.32260	-1.85834
O	0.73484	-0.97712	0.23551
C	-1.57754	1.87683	0.52793
C	-2.94624	2.16135	0.01402
C	-3.42614	3.41917	0.04940
H	-4.42509	3.63224	-0.32027
C	-2.59827	4.52334	0.58868
C	-3.11025	5.91766	0.54433
H	-4.09873	6.12310	0.14458
C	-2.35164	6.92916	0.99498
H	-2.72535	7.94749	0.96051
C	-0.99694	6.66034	1.52906
H	-0.38024	7.48501	1.87192
C	-0.52742	5.40311	1.57816
H	0.47652	5.23158	1.95167
C	-1.37054	4.26445	1.10699
C	-0.87215	2.85249	1.15288
C	1.37452	1.84794	1.20406

C	2.70945	1.66780	1.83966
C	2.89667	2.03399	3.12124
H	3.86886	1.90802	3.58812
C	1.77816	2.60265	3.90908
C	1.97721	2.91833	5.34747
H	2.94774	2.76568	5.80996
C	0.95726	3.38903	6.08198
H	1.10272	3.61704	7.13303
C	-0.37380	3.58551	5.46392
H	-1.20033	3.94420	6.06900
C	-0.56235	3.30872	4.16343
H	-1.55223	3.43599	3.73852
C	0.57013	2.81293	3.32563
C	0.38659	2.49486	1.87255
C	3.81982	1.06107	1.05280
C	4.43918	1.76523	0.04994
C	4.20822	-0.37326	1.32271
C	5.40049	1.03806	-0.83365
C	5.14690	-0.95298	0.54675
C	5.74145	-0.24345	-0.60775
H	5.80249	1.52333	-1.70725
H	5.45607	-1.97128	0.74827
C	-3.77624	1.05661	-0.54419
C	-4.38320	0.03262	0.38994
C	-3.94641	0.93061	-1.89411
C	-5.06126	-1.00613	-0.15027
C	-4.75955	-0.19916	-2.42233
C	-5.32860	-1.11188	-1.60995
H	-5.42928	-1.79707	0.47473
H	-4.84271	-0.28585	-3.49558
H	0.20983	-1.27378	-2.54263
C	1.76296	-2.05375	-3.65082
C	0.55601	-3.19949	-1.89336
C	1.94756	-0.86471	-4.38257
C	2.63565	-3.15694	-3.83144
C	1.45982	-4.30182	-2.03156
C	2.97607	-0.79843	-5.30419
H	1.30077	-0.01751	-4.18304
C	3.66253	-3.06155	-4.79669
C	2.43894	-4.28797	-2.98431
H	1.32859	-5.16553	-1.39428
C	3.83057	-1.89959	-5.52440
H	3.13014	0.11916	-5.86339
H	4.32921	-3.90738	-4.93854
H	3.08740	-5.15155	-3.10543
H	4.62814	-1.82556	-6.25627
C	-0.43099	-3.07687	-0.85874
C	-0.71071	-4.20886	0.10428
H	0.23794	-4.72386	0.32796
H	-1.38617	-4.95858	-0.33504
N	-1.30435	-3.66850	1.31564
N	0.74607	-2.14718	-2.73042
C	-1.44577	-4.41104	2.47582
C	-1.37943	-5.81699	2.48342
C	-1.67907	-3.74614	3.69781
C	-1.55437	-6.52278	3.67423
H	-1.20101	-6.35940	1.56128
C	-1.85073	-4.46250	4.87472
H	-1.70936	-2.65983	3.71003
C	-1.79133	-5.85995	4.87726

H	-1.50123	-7.60818	3.65452
H	-2.02573	-3.92308	5.80171
H	-1.92397	-6.41706	5.79915
H	-1.14702	-2.67936	1.45183
H	0.23500	-1.93735	-0.16606
Cl	-1.98707	-2.33340	-1.42720
C	3.60056	-1.18009	2.47686
C	4.62752	-1.27368	3.63320
C	3.08895	-2.56565	1.99906
H	2.72414	-0.62880	2.86748
C	4.33314	-2.31685	4.72676
H	4.69801	-0.27511	4.11536
H	5.63868	-1.49779	3.22792
C	1.85080	-3.03786	2.77901
H	3.89335	-3.33126	2.05484
H	2.78936	-2.49982	0.92981
C	2.84587	-2.47365	5.08269
H	4.76455	-3.29775	4.42917
H	4.88305	-2.00864	5.64301
C	2.15131	-3.52257	4.20145
H	1.40952	-3.89297	2.22802
H	1.07955	-2.23733	2.80680
H	2.31275	-1.49896	5.04296
H	2.78300	-2.82956	6.13427
H	2.75617	-4.45483	4.16007
H	1.18496	-3.79238	4.67961
C	4.16878	3.27180	-0.17233
C	4.37249	3.79087	-1.62766
C	4.82609	4.15397	0.91817
H	3.07986	3.41588	-0.02701
C	5.78178	4.28731	-2.02614
H	3.66346	4.63145	-1.80174
H	4.05586	2.99971	-2.34162
C	6.36179	4.16918	0.86812
H	4.52542	3.77934	1.91598
H	4.42280	5.18864	0.86222
C	6.19075	5.57726	-1.30192
H	6.56465	3.51692	-1.89144
H	5.75222	4.50634	-3.11595
C	6.92992	5.32029	0.02195
H	6.75418	3.19741	0.50057
H	6.75191	4.28698	1.90285
H	5.30362	6.22369	-1.12521
H	6.87129	6.15772	-1.96274
H	7.99729	5.09720	-0.19606
H	6.90842	6.25674	0.62121
C	6.67577	-0.97701	-1.56326
C	5.91842	-2.10781	-2.31556
C	7.96858	-1.39707	-0.82925
H	7.00243	-0.26003	-2.35145
C	6.16166	-3.54397	-1.81712
H	6.17202	-2.05439	-3.39740
H	4.82421	-1.90988	-2.27198
C	8.94780	-2.17996	-1.71934
H	7.74146	-1.97219	0.09373
H	8.47997	-0.46764	-0.49293
C	7.45299	-4.15315	-2.38431
H	6.14840	-3.60451	-0.71000
H	5.31660	-4.17022	-2.17529
C	8.71588	-3.69515	-1.64802

H	9.97918	-1.98272	-1.35342
H	8.90468	-1.82630	-2.77293
H	7.54621	-3.93578	-3.47091
H	7.38723	-5.25853	-2.28409
H	8.67945	-4.03099	-0.58834
H	9.58719	-4.20213	-2.11680
C	-4.23036	0.17507	1.92039
C	-4.24783	-1.14904	2.74261
C	-5.11749	1.30290	2.50159
H	-3.19558	0.53173	2.08108
C	-5.61662	-1.67077	3.23548
H	-3.60260	-1.01167	3.63914
H	-3.75261	-1.94903	2.15052
C	-6.62283	1.00390	2.44780
H	-4.93845	2.23345	1.92818
H	-4.80271	1.53499	3.54247
C	-6.24964	-0.77021	4.30474
H	-6.33646	-1.84242	2.41226
H	-5.43578	-2.66670	3.69614
C	-7.15824	0.32304	3.71827
H	-6.86740	0.39166	1.55674
H	-7.17282	1.96213	2.32157
H	-5.46412	-0.31160	4.94413
H	-6.87020	-1.39984	4.97941
H	-8.15108	-0.11910	3.48269
H	-7.32317	1.09522	4.50122
C	-3.32888	1.89602	-2.91623
C	-4.44654	2.78519	-3.51634
C	-2.51635	1.12469	-3.98985
H	-2.62142	2.57710	-2.40415
C	-4.09673	3.54104	-4.81147
H	-4.74390	3.53077	-2.74735
H	-5.35180	2.17428	-3.72654
C	-1.30784	1.92632	-4.50475
H	-3.16342	0.81785	-4.84029
H	-2.11625	0.18745	-3.54496
C	-2.65968	4.08460	-4.86992
H	-4.29916	2.88812	-5.68870
H	-4.80032	4.39762	-4.90142
C	-1.67922	3.06193	-5.46517
H	-0.63970	1.22860	-5.05387
H	-0.71709	2.33128	-3.65420
H	-2.31716	4.43506	-3.87207
H	-2.65876	4.97320	-5.53841
H	-2.08896	2.63736	-6.40786
H	-0.74269	3.59611	-5.73654
C	-6.10009	-2.32991	-2.15198
C	-6.55159	-2.26973	-3.64007
C	-7.27032	-2.81004	-1.24929
H	-5.35945	-3.16001	-2.12004
C	-7.53401	-1.14250	-4.01103
H	-7.02008	-3.24049	-3.91498
H	-5.65773	-2.19629	-4.29568
C	-8.25343	-1.73026	-0.75591
H	-6.85855	-3.33292	-0.35926
H	-7.84947	-3.59578	-1.78220
C	-8.83071	-1.14281	-3.19211
H	-7.05033	-0.14683	-3.94551
H	-7.80699	-1.27848	-5.08017
C	-8.60697	-0.62732	-1.76323

H	-7.84756	-1.25183	0.15842
H	-9.19200	-2.23997	-0.44577
H	-9.30080	-2.15038	-3.19158
H	-9.54429	-0.45508	-3.69658
H	-7.82632	0.16412	-1.74700
H	-9.54298	-0.13967	-1.41293

Catalyst (2,4,6-c-Hept, 12)-Enamine protonation TS (pro-S) alternative conformation
Electronic energy (Hartrees): -1870.349711
Free Energy at 1M concentration and 253 K (Hartrees): -1868.512211
Coordinates:

P	-0.13326	-0.33683	0.02350
O	1.19090	-0.07730	0.93705
O	-1.25055	-1.01322	0.99358
O	0.04751	-1.28101	-1.12027
O	-0.56734	1.11975	-0.31306
C	1.58092	-1.15949	1.68126
C	2.89974	-1.77686	1.39899
C	3.36464	-2.75107	2.19356
H	4.33675	-3.19018	2.00440
C	2.53139	-3.27493	3.29447
C	3.00496	-4.43520	4.08360
H	3.97987	-4.86437	3.88829
C	2.22884	-4.96136	5.03700
H	2.57470	-5.81091	5.61156
C	0.89129	-4.39612	5.29756
H	0.25770	-4.84337	6.05260
C	0.45616	-3.34440	4.59411
H	-0.53878	-2.96418	4.78321
C	1.32163	-2.72465	3.55390
C	0.86566	-1.53977	2.76431
C	-1.32450	-0.51708	2.26960
C	-2.60338	0.09480	2.70587
C	-2.77671	0.43981	3.98879
H	-3.71415	0.87555	4.31263
C	-1.66476	0.30990	4.95095
C	-1.82827	0.82215	6.33089
H	-2.77894	1.23081	6.65030
C	-0.80123	0.78878	7.18638
H	-0.92079	1.16619	8.19371
C	0.50403	0.25480	6.75404
H	1.33925	0.25734	7.44250
C	0.66080	-0.21562	5.51135
H	1.63563	-0.57295	5.20810
C	-0.48265	-0.22071	4.55779
C	-0.33840	-0.75344	3.16735
C	-3.65544	0.39083	1.70210
C	-4.40117	-0.60418	1.13464
C	-3.88730	1.82434	1.30271
C	-5.40511	-0.23261	0.10641
C	-4.89180	2.11192	0.45572
C	-5.67535	1.04525	-0.19168
H	-5.92889	-1.00229	-0.42131
H	-5.08694	3.14003	0.17641
C	3.69076	-1.31990	0.22987
C	4.42564	-0.00728	0.29696
C	3.74108	-2.06671	-0.90853
C	5.15084	0.37773	-0.76904
C	4.56124	-1.59090	-2.05051

C	5.27914	-0.45900	-1.98476
H	5.65356	1.32503	-0.76252
H	4.53403	-2.17482	-2.95240
H	-0.43960	-0.58460	-2.61142
C	-2.17780	-0.70305	-3.71080
C	-0.82568	1.28185	-3.39780
C	-2.38986	-2.05554	-3.37841
C	-3.13341	0.01051	-4.47708
C	-1.81246	2.02805	-4.12310
C	-3.52808	-2.68955	-3.84089
H	-1.67693	-2.55729	-2.73412
C	-4.26596	-0.67911	-4.96385
C	-2.90106	1.40626	-4.66697
H	-1.65909	3.08862	-4.26970
C	-4.46305	-2.00984	-4.64968
H	-3.70422	-3.72677	-3.57338
H	-4.98849	-0.14070	-5.56991
H	-3.61577	1.98014	-5.25098
H	-5.34336	-2.53045	-5.01202
C	0.28879	1.86090	-2.70455
C	0.59941	3.33782	-2.82174
H	-0.35325	3.89551	-2.82151
H	1.11303	3.57082	-3.76634
N	1.43849	3.74639	-1.70914
N	-1.05208	-0.05103	-3.26604
C	1.78242	5.06917	-1.47729
C	1.68835	6.06049	-2.47214
C	2.26150	5.44515	-0.20488
C	2.08048	7.37135	-2.19875
H	1.31630	5.80968	-3.45942
C	2.64435	6.75452	0.05348
H	2.31408	4.69781	0.58221
C	2.56150	7.73366	-0.94163
H	2.00212	8.11767	-2.98500
H	3.00604	7.01351	1.04497
H	2.86084	8.75664	-0.73728
H	1.32932	3.17793	-0.88032
H	-0.20443	1.51281	-1.31792
Cl	1.81232	0.87631	-2.80389
C	-2.99229	2.96160	1.80248
C	-3.75707	3.81192	2.84724
C	-2.43571	3.79763	0.61894
H	-2.12045	2.52051	2.32365
C	-3.14239	5.18152	3.19052
H	-3.83378	3.22159	3.78534
H	-4.79904	3.99352	2.50424
C	-1.02499	4.34273	0.89019
H	-3.12567	4.62879	0.35555
H	-2.35805	3.15204	-0.28414
C	-1.60566	5.20102	3.24582
H	-3.51269	5.94497	2.47167
H	-3.53375	5.48622	4.18595
C	-0.98311	5.51511	1.87662
H	-0.61820	4.70794	-0.07476
H	-0.35224	3.52877	1.23913
H	-1.20131	4.25316	3.66279
H	-1.30041	6.01197	3.94265
H	-1.47483	6.40377	1.42365
H	0.08328	5.78734	2.03406
C	-4.25678	-2.08517	1.53052

C	-3.99282	-3.03112	0.31942
C	-5.31727	-2.53441	2.56856
H	-3.32261	-2.18685	2.11312
C	-5.18583	-3.79713	-0.28562
H	-3.23490	-3.78974	0.62028
H	-3.50872	-2.44782	-0.49465
C	-6.73660	-2.76360	2.02639
H	-5.37294	-1.75012	3.35619
H	-4.96636	-3.45312	3.08887
C	-5.81587	-4.79124	0.69795
H	-5.95406	-3.12671	-0.71327
H	-4.78989	-4.38000	-1.14606
C	-6.97557	-4.19049	1.50650
H	-6.99821	-2.02630	1.24690
H	-7.45803	-2.58728	2.85401
H	-5.04437	-5.20195	1.38548
H	-6.21545	-5.65663	0.12511
H	-7.89133	-4.18246	0.87555
H	-7.18082	-4.86122	2.36939
C	-6.71478	1.40927	-1.23986
C	-8.12387	0.85668	-0.87389
C	-6.18820	1.04386	-2.64371
H	-6.81613	2.51827	-1.23518
C	-8.58438	-0.40090	-1.63751
H	-8.87701	1.66203	-1.02179
H	-8.16137	0.63237	0.21557
C	-7.18217	1.36199	-3.77152
H	-5.87876	-0.02257	-2.68436
H	-5.26602	1.64033	-2.82223
C	-9.16637	-0.07278	-3.02169
H	-7.77594	-1.15536	-1.72188
H	-9.39429	-0.87532	-1.04127
C	-8.09604	0.17207	-4.09030
H	-6.60081	1.59489	-4.69004
H	-7.78439	2.26616	-3.53371
H	-9.85131	0.80118	-2.96293
H	-9.78018	-0.93860	-3.35302
H	-7.49260	-0.74994	-4.24172
H	-8.61373	0.37958	-5.05193
C	4.37121	0.87210	1.55704
C	4.09812	2.38349	1.28525
C	5.49359	0.53012	2.56695
H	3.46029	0.57251	2.10537
C	5.30376	3.34450	1.22833
H	3.40913	2.76686	2.07189
H	3.53283	2.47889	0.33265
C	6.89095	1.03605	2.18052
H	5.54203	-0.57717	2.66469
H	5.21972	0.90733	3.57698
C	6.04348	3.45330	2.56823
H	6.00842	3.09968	0.41106
H	4.90277	4.35219	0.98323
C	7.19656	2.44698	2.70754
H	7.03657	1.00430	1.08483
H	7.64901	0.34501	2.60967
H	5.33268	3.33819	3.41560
H	6.47273	4.47524	2.65815
H	8.08512	2.83636	2.16395
H	7.47460	2.38118	3.78217
C	3.00892	-3.40416	-1.07096

C	4.03865	-4.55873	-0.98638
C	2.17013	-3.43061	-2.37663
H	2.30134	-3.54303	-0.22958
C	3.56565	-5.93511	-1.48917
H	4.35152	-4.66552	0.07460
H	4.95555	-4.29635	-1.55894
C	0.89067	-4.27478	-2.24065
H	2.77604	-3.79115	-3.23630
H	1.85034	-2.39654	-2.63030
C	2.10184	-6.27101	-1.16012
H	3.74312	-6.01142	-2.58435
H	4.21674	-6.70603	-1.02157
C	1.13862	-5.78740	-2.25536
H	0.22862	-4.03664	-3.10079
H	0.33370	-3.99454	-1.32002
H	1.80814	-5.87490	-0.16381
H	2.01102	-7.37779	-1.10339
H	1.50677	-6.09759	-3.25785
H	0.15975	-6.29258	-2.10475
C	6.12685	0.06633	-3.15058
C	6.24179	-0.82749	-4.42067
C	7.51686	0.60312	-2.71852
H	5.57393	0.96566	-3.50401
C	7.00374	-2.16440	-4.28873
H	6.73934	-0.23390	-5.21938
H	5.22293	-1.03696	-4.81221
C	8.33786	-0.31316	-1.79262
H	7.39137	1.57979	-2.20367
H	8.12142	0.84254	-3.62033
C	8.41019	-2.05193	-3.68899
H	6.42135	-2.91533	-3.71979
H	7.10686	-2.58563	-5.31253
C	8.36546	-1.79936	-2.17535
H	7.95502	-0.22845	-0.75436
H	9.38201	0.06909	-1.77583
H	9.01095	-1.28059	-4.21731
H	8.92248	-3.02454	-3.85745
H	7.49599	-2.32017	-1.71759
H	9.27041	-2.25130	-1.71331

Catalyst (2,4,6-c-Hept, 12)-Enamine protonation TS (pro-S) alternative conformation
Electronic energy (Hartrees): -1870.343353
Free Energy at 1M concentration and 253 K (Hartrees): -1868.508099
Coordinates:

P	0.38179	0.09689	0.18342
O	1.62020	-0.36303	1.15583
O	-0.90439	-0.87577	0.49282
O	0.66490	-0.01876	-1.27746
O	0.06205	1.51678	0.74489
C	1.67972	-1.71806	1.32777
C	2.82912	-2.47160	0.75638
C	2.97410	-3.78200	1.03363
H	3.80917	-4.33785	0.61904
C	1.96295	-4.49512	1.84572
C	2.08323	-5.96364	2.03909
H	2.94373	-6.49667	1.64600
C	1.12406	-6.63918	2.69001
H	1.20951	-7.71259	2.82524
C	-0.06239	-5.92443	3.21046

H	-0.84964	-6.48062	3.70932
C	-0.17514	-4.59618	3.05010
H	-1.07175	-4.10521	3.41006
C	0.89714	-3.82397	2.35179
C	0.78828	-2.34301	2.13789
C	-1.19634	-0.93833	1.82833
C	-2.56754	-0.56970	2.27451
C	-2.88623	-0.63250	3.58099
H	-3.89590	-0.40284	3.90939
C	-1.88302	-1.09462	4.57179
C	-2.21204	-1.08554	6.02072
H	-3.18005	-0.72725	6.35776
C	-1.31313	-1.52015	6.91864
H	-1.55344	-1.51242	7.97696
C	0.01184	-2.00720	6.46870
H	0.73806	-2.34549	7.20086
C	0.31661	-2.02457	5.16077
H	1.29608	-2.37285	4.84889
C	-0.67913	-1.56178	4.15173
C	-0.36384	-1.57256	2.68983
C	-3.62822	-0.33144	1.25771
C	-4.12188	-1.37228	0.51738
C	-4.16653	1.06182	1.04276
C	-5.18426	-1.10802	-0.48966
C	-5.16282	1.24375	0.14686
C	-5.69837	0.12206	-0.66008
H	-5.55759	-1.93552	-1.08038
H	-5.57221	2.23073	-0.02552
C	3.74023	-1.81076	-0.21707
C	4.72796	-0.76494	0.26325
C	3.61944	-2.07763	-1.55681
C	5.35945	-0.02167	-0.67546
C	4.49906	-1.36207	-2.51858
C	5.29653	-0.35915	-2.11835
H	5.94070	0.83962	-0.40890
H	4.45494	-1.63129	-3.56974
H	0.41987	1.50060	-2.13933
C	-1.21909	2.18337	-3.18147
C	0.10242	3.46272	-1.61141
C	-1.41002	0.95926	-3.84985
C	-2.14172	3.24792	-3.33872
C	-0.84443	4.53259	-1.72374
C	-2.49312	0.81957	-4.69760
H	-0.71419	0.14830	-3.66760
C	-3.23204	3.07362	-4.21949
C	-1.91261	4.42925	-2.56863
H	-0.69630	5.41292	-1.11392
C	-3.39859	1.88221	-4.89945
H	-2.64877	-0.12321	-5.21278
H	-3.93761	3.88930	-4.34909
H	-2.61079	5.25693	-2.66079
H	-4.23764	1.75383	-5.57546
C	1.17376	3.45900	-0.65686
C	1.52751	4.74613	0.07682
H	1.54201	5.60252	-0.61067
H	2.54701	4.64754	0.47309
N	0.55543	5.00581	1.13738
N	-0.13277	2.35419	-2.35506
C	0.67268	6.13049	1.96127
C	0.16903	6.08230	3.27586

C	1.24122	7.33687	1.51241
C	0.20411	7.20487	4.09548
H	-0.24253	5.14988	3.65415
C	1.27363	8.45496	2.34564
H	1.65593	7.40900	0.51318
C	0.75378	8.40529	3.63839
H	-0.19301	7.13640	5.10456
H	1.71644	9.37494	1.97353
H	0.78452	9.27943	4.28089
H	0.32540	4.16115	1.64852
H	0.57256	2.39127	0.21424
Cl	2.67440	2.61896	-1.22917
C	2.58511	-3.05680	-2.13578
C	3.29161	-4.36419	-2.56617
C	1.77246	-2.40972	-3.28921
H	1.85928	-3.33027	-1.34644
C	2.45956	-5.33045	-3.43269
H	3.60884	-4.90710	-1.65096
H	4.22088	-4.12500	-3.12827
C	0.31379	-2.89909	-3.32226
H	2.25979	-2.58764	-4.27273
H	1.74358	-1.30799	-3.15481
C	0.96003	-5.38309	-3.08941
H	2.59834	-5.07723	-4.50663
H	2.88492	-6.34997	-3.30425
C	0.15162	-4.32614	-3.86020
H	-0.26569	-2.22369	-3.98728
H	-0.14373	-2.81974	-2.31230
H	0.78903	-5.30181	-1.99413
H	0.57660	-6.38071	-3.39615
H	0.41765	-4.35084	-4.93968
H	-0.92522	-4.59351	-3.79299
C	4.94354	-0.50382	1.77568
C	5.84916	0.70071	2.18365
C	5.32614	-1.76444	2.59331
H	3.94455	-0.20761	2.15749
C	7.37564	0.60138	1.93298
H	5.70674	0.87887	3.27295
H	5.46865	1.62328	1.69405
C	6.42608	-2.64289	1.97658
H	4.43488	-2.39479	2.76958
H	5.62767	-1.46913	3.62134
C	8.05280	-0.69240	2.40252
H	7.85055	1.44378	2.48178
H	7.63474	0.77369	0.87213
C	7.68209	-1.88986	1.51449
H	6.01173	-3.20567	1.11306
H	6.72311	-3.39794	2.73702
H	7.84401	-0.89030	3.47534
H	9.15126	-0.53811	2.32178
H	7.55794	-1.56548	0.45835
H	8.52976	-2.60960	1.51606
C	6.15269	0.39851	-3.12685
C	7.66864	0.22686	-2.81673
C	5.64734	1.85224	-3.25598
H	5.99655	-0.06188	-4.12976
C	8.37586	1.43046	-2.16550
H	8.20212	-0.03655	-3.75684
H	7.81485	-0.65596	-2.15475
C	6.45824	2.69425	-4.25436

H	5.60881	2.35423	-2.26543
H	4.59689	1.81050	-3.62134
C	8.77988	2.50306	-3.18899
H	7.76834	1.87471	-1.35146
H	9.30890	1.05390	-1.69184
C	7.62565	3.42948	-3.58378
H	5.78181	3.46498	-4.68455
H	6.82222	2.07302	-5.10200
H	9.22057	2.03613	-4.09686
H	9.57572	3.13271	-2.73455
H	7.26766	3.99063	-2.69265
H	8.02060	4.18137	-4.30121
C	-3.64756	-2.82384	0.67108
C	-2.93102	-3.27433	-0.62948
C	-4.82268	-3.74721	1.13479
H	-2.88818	-2.89524	1.47303
C	-2.07164	-4.54139	-0.46699
H	-2.25637	-2.46264	-0.97513
H	-3.65108	-3.41192	-1.46103
C	-5.17327	-4.91742	0.19458
H	-5.74971	-3.15004	1.28090
H	-4.59399	-4.16131	2.14170
C	-2.66457	-5.62103	0.45070
H	-1.08046	-4.24878	-0.05514
H	-1.88990	-4.97961	-1.47169
C	-4.10381	-6.03122	0.09653
H	-5.42505	-4.53275	-0.81544
H	-6.11381	-5.38127	0.56484
H	-2.60899	-5.29802	1.51098
H	-2.01909	-6.52314	0.37588
H	-4.11862	-6.45752	-0.93019
H	-4.39187	-6.85329	0.78793
C	-6.79518	0.38653	-1.68289
C	-8.08294	-0.42954	-1.36942
C	-6.22640	0.23838	-3.11049
H	-7.08693	1.45693	-1.58547
C	-8.33265	-1.67275	-2.24449
H	-8.96486	0.24472	-1.44310
H	-8.06517	-0.75443	-0.30506
C	-7.26835	0.47952	-4.21383
H	-5.73290	-0.74833	-3.24793
H	-5.42678	1.00110	-3.22473
C	-8.97922	-1.32430	-3.59464
H	-7.40739	-2.26652	-2.39249
H	-9.04314	-2.32958	-1.69635
C	-7.97772	-0.81359	-4.63542
H	-6.73871	0.87647	-5.10744
H	-8.00838	1.25071	-3.90631
H	-9.79778	-0.58381	-3.45978
H	-9.44671	-2.24695	-4.00271
H	-8.53492	-0.61287	-5.57631
H	-7.23195	-1.60619	-4.86517
C	-3.60431	2.26249	1.81055
C	-4.56641	2.73363	2.93081
C	-3.08183	3.37436	0.86201
H	-2.69802	1.93213	2.35889
C	-5.78255	3.57349	2.50006
H	-3.98827	3.31149	3.68467
H	-4.94268	1.84123	3.47679
C	-2.85926	4.72734	1.55945

H	-3.71464	3.51572	-0.03501
H	-2.10378	3.03673	0.45830
C	-5.47789	4.81615	1.64069
H	-6.51848	2.93159	1.97277
H	-6.28844	3.91498	3.42982
C	-4.13923	5.50442	1.96293
H	-2.26264	5.37008	0.87707
H	-2.23272	4.56238	2.46052
H	-6.29143	5.55398	1.81494
H	-5.54172	4.57216	0.56139
H	-4.10444	5.73468	3.05038
H	-4.13098	6.48574	1.43955

Catalyst (2,4,6-c-Hept, 12)-Enamine protonation TS (pro-S) alternative conformation
Electronic energy (Hartrees): -1870.342242
Free Energy at 1M concentration and 253 K (Hartrees): -1868.506908
Coordinates:

P	0.40994	0.26589	0.35960
O	1.60323	0.00154	1.45271
O	-0.77718	-0.83870	0.62110
O	0.83112	0.11548	-1.06431
O	-0.11615	1.66360	0.81009
C	1.80685	-1.32877	1.69237
C	3.07112	-1.96441	1.23153
C	3.34878	-3.23413	1.58586
H	4.26851	-3.70436	1.25275
C	2.37296	-4.02668	2.36760
C	2.65063	-5.45982	2.64582
H	3.59394	-5.90187	2.33943
C	1.73298	-6.21676	3.26628
H	1.93402	-7.26487	3.46337
C	0.43619	-5.62839	3.66817
H	-0.31417	-6.25301	4.14228
C	0.17975	-4.33207	3.43053
H	-0.79209	-3.93783	3.70355
C	1.20131	-3.46765	2.76515
C	0.93498	-2.02162	2.46686
C	-1.16799	-0.87962	1.93233
C	-2.60434	-0.65784	2.25283
C	-3.01905	-0.69834	3.53290
H	-4.07208	-0.57734	3.77078
C	-2.05334	-0.99259	4.62038
C	-2.49800	-0.95632	6.03766
H	-3.52479	-0.69991	6.28052
C	-1.63139	-1.24017	7.02333
H	-1.95631	-1.21254	8.05848
C	-0.22729	-1.58726	6.70208
H	0.47084	-1.80345	7.50443
C	0.18284	-1.62836	5.42381
H	1.21777	-1.87235	5.20639
C	-0.77385	-1.33333	4.31858
C	-0.34130	-1.37291	2.88743
C	-3.59903	-0.60281	1.14699
C	-3.90794	-1.73402	0.43998
C	-4.27126	0.70319	0.80136
C	-4.90102	-1.65222	-0.66435
C	-5.19911	0.71735	-0.18219
C	-5.53087	-0.50158	-0.95696
H	-5.12587	-2.54902	-1.22848

H	-5.70257	1.63767	-0.44848
C	3.95556	-1.24259	0.27675
C	4.75930	-0.04734	0.75168
C	3.95076	-1.58217	-1.05203
C	5.31171	0.75164	-0.19204
C	4.75885	-0.77836	-2.00504
C	5.35594	0.36099	-1.62141
H	5.74805	1.70221	0.06026
H	4.80604	-1.08951	-3.04166
H	0.50628	1.54877	-2.03784
C	-1.10529	1.96852	-3.24742
C	-0.09220	3.47686	-1.65119
C	-1.08498	0.70106	-3.85978
C	-2.13719	2.89682	-3.53455
C	-1.15117	4.41045	-1.89783
C	-2.06849	0.38408	-4.77834
H	-0.31091	-0.00483	-3.58099
C	-3.12064	2.54309	-4.48473
C	-2.12275	4.13182	-2.81653
H	-1.16702	5.32932	-1.32827
C	-3.08048	1.31002	-5.10721
H	-2.06144	-0.59335	-5.25062
H	-3.90949	3.25405	-4.71329
H	-2.90835	4.85756	-3.00915
H	-3.83814	1.04330	-5.83676
C	0.88289	3.65223	-0.61320
C	1.00936	5.00609	0.07353
H	0.97693	5.82502	-0.65752
H	1.99512	5.05745	0.55494
N	-0.07521	5.18693	1.03713
N	-0.12284	2.31523	-2.34907
C	-0.16494	6.35159	1.80730
C	-0.76778	6.29742	3.07924
C	0.28900	7.59910	1.34071
C	-0.93817	7.45001	3.83802
H	-1.09352	5.33800	3.47329
C	0.11555	8.74743	2.11310
H	0.77429	7.67938	0.37442
C	-0.50138	8.68887	3.36211
H	-1.40776	7.37615	4.81510
H	0.47328	9.69866	1.72827
H	-0.63118	9.58686	3.95762
H	-0.24464	4.34250	1.57180
H	0.33591	2.56322	0.26750
Cl	2.52004	2.98422	-1.01165
C	3.10109	-2.72329	-1.63471
C	4.00913	-3.93487	-1.95129
C	2.28380	-2.25431	-2.86912
H	2.37077	-3.05912	-0.87433
C	3.37598	-5.05075	-2.80653
H	4.34239	-4.38210	-0.99116
H	4.92841	-3.59112	-2.47460
C	0.91170	-2.94455	-2.95735
H	2.85347	-2.41073	-3.81127
H	2.09596	-1.16249	-2.80295
C	1.87875	-5.29808	-2.54772
H	3.54775	-4.83380	-3.88364
H	3.92878	-5.99235	-2.59520
C	0.98344	-4.40430	-3.42230
H	0.28758	-2.38955	-3.69021

H	0.38516	-2.88250	-1.98069
H	1.62731	-5.18946	-1.47045
H	1.65757	-6.35343	-2.81908
H	1.31981	-4.44273	-4.48156
H	-0.04813	-4.81782	-3.40215
C	4.87103	0.29077	2.25992
C	5.60002	1.61328	2.65447
C	5.37413	-0.88162	3.14167
H	3.82652	0.46914	2.59000
C	7.13371	1.70030	2.45058
H	5.39977	1.80471	3.73234
H	5.12192	2.46451	2.12316
C	6.60124	-1.63503	2.60424
H	4.56201	-1.61339	3.30874
H	5.59230	-0.51456	4.16770
C	7.95058	0.52326	2.99923
H	7.47940	2.61818	2.97442
H	7.40383	1.86014	1.38941
C	7.76943	-0.74622	2.15309
H	6.29740	-2.28092	1.75303
H	6.96054	-2.31456	3.40783
H	7.72604	0.34236	4.07195
H	9.02303	0.81358	2.94929
H	7.64739	-0.48279	1.07982
H	8.70046	-1.35108	2.21595
C	6.08787	1.24491	-2.62345
C	5.11649	1.80021	-3.70614
C	7.35073	0.52440	-3.14682
H	6.46070	2.13597	-2.06954
C	5.18226	1.12982	-5.09108
H	5.29330	2.89213	-3.82545
H	4.06890	1.72318	-3.33894
C	8.14116	1.34884	-4.17577
H	7.10056	-0.47631	-3.56067
H	8.01628	0.33800	-2.27436
C	6.32842	1.67592	-5.95711
H	5.23846	0.02476	-5.01195
H	4.23031	1.35889	-5.61845
C	7.69221	1.06761	-5.61565
H	9.21260	1.06137	-4.09809
H	8.08050	2.43653	-3.95175
H	6.37934	2.78466	-5.88921
H	6.10719	1.43046	-7.01878
H	7.67805	-0.02755	-5.80920
H	8.44480	1.50710	-6.30599
C	-3.28876	-3.10909	0.72541
C	-2.41568	-3.54323	-0.48145
C	-4.38898	-4.13670	1.15207
H	-2.59873	-3.04640	1.58860
C	-1.43868	-4.69181	-0.17051
H	-1.80774	-2.67821	-0.82114
H	-3.04033	-3.80759	-1.35830
C	-4.52504	-5.38821	0.26245
H	-5.38587	-3.64438	1.18669
H	-4.20275	-4.46692	2.19796
C	-1.98580	-5.78186	0.76309
H	-0.52550	-4.26480	0.29985
H	-1.12277	-5.15957	-1.12750
C	-3.33433	-6.37440	0.32100
H	-4.72910	-5.08987	-0.78677

H	-5.43699	-5.93720	0.58464
H	-2.05884	-5.39797	1.80181
H	-1.24023	-6.60586	0.79764
H	-3.21241	-6.85387	-0.67466
H	-3.58849	-7.18609	1.03750
C	-6.55246	-0.42320	-2.08357
C	-7.76617	-1.36281	-1.82563
C	-5.84339	-0.58581	-3.44544
H	-6.96725	0.61037	-2.08253
C	-7.79794	-2.67382	-2.63423
H	-8.70631	-0.79956	-2.01734
H	-7.80945	-1.62299	-0.74435
C	-6.80089	-0.52841	-4.64588
H	-5.23504	-1.51586	-3.47513
H	-5.12418	0.25539	-3.54279
C	-8.35354	-2.47860	-4.05395
H	-6.80369	-3.16490	-2.66437
H	-8.47914	-3.37586	-2.10528
C	-7.32361	-1.91604	-5.03872
H	-6.23880	-0.12458	-5.51627
H	-7.64555	0.16906	-4.45343
H	-9.25695	-1.83043	-4.03756
H	-8.67847	-3.47031	-4.43767
H	-7.81178	-1.83397	-6.03421
H	-6.47798	-2.62963	-5.15215
C	-3.92184	2.00055	1.53743
C	-5.02569	2.40592	2.54718
C	-3.45407	3.11943	0.56849
H	-3.03372	1.81098	2.17503
C	-6.29142	3.06559	1.97044
H	-4.58751	3.08865	3.30764
H	-5.33861	1.50290	3.11530
C	-3.45611	4.52294	1.19830
H	-4.01795	3.13651	-0.38373
H	-2.41124	2.88553	0.26628
C	-6.06375	4.29076	1.06343
H	-6.89705	2.31132	1.42638
H	-6.91292	3.38886	2.83427
C	-4.84981	5.15539	1.44845
H	-2.88216	5.19931	0.52912
H	-2.89646	4.48207	2.15571
H	-6.97154	4.92995	1.12646
H	-6.00408	3.98582	-0.00049
H	-4.93770	5.44456	2.51870
H	-4.91286	6.10149	0.86718

Catalyst (2,4,6-c-Hept, 12)-Enamine protonation TS (pro-S) alternative conformation
Electronic energy (Hartrees): -1870.341369
Free Energy at 1M concentration and 253 K (Hartrees): -1868.50479
Coordinates:

P	0.53278	0.16819	0.20041
O	1.68521	-0.08504	1.33672
O	-0.57517	-1.04419	0.29753
O	1.01873	0.14251	-1.21101
O	-0.11796	1.48747	0.71567
C	2.06297	-1.39806	1.39516
C	3.43834	-1.78248	0.97217
C	3.85006	-3.05762	1.11689
H	4.85424	-3.34402	0.82188

C	2.92690	-4.09182	1.63522
C	3.36140	-5.51250	1.66922
H	4.37149	-5.78098	1.37465
C	2.50434	-6.47080	2.05344
H	2.82036	-7.50886	2.07216
C	1.11949	-6.11638	2.43696
H	0.42192	-6.89888	2.71804
C	0.72140	-4.83418	2.42124
H	-0.30790	-4.60634	2.67408
C	1.67184	-3.75202	2.02322
C	1.24846	-2.31457	1.97301
C	-0.96553	-1.33059	1.57671
C	-2.42488	-1.35529	1.87912
C	-2.84807	-1.71314	3.10676
H	-3.91030	-1.77589	3.32292
C	-1.87045	-2.09046	4.15509
C	-2.33793	-2.39348	5.53260
H	-3.39392	-2.32813	5.77682
C	-1.45571	-2.74859	6.48058
H	-1.79716	-2.96881	7.48693
C	-0.01235	-2.83585	6.15774
H	0.69677	-3.11143	6.93172
C	0.41873	-2.56880	4.91409
H	1.48083	-2.62483	4.69758
C	-0.55296	-2.19025	3.84767
C	-0.09910	-1.89608	2.45364
C	-3.41652	-1.14788	0.78529
C	-3.56955	-2.07934	-0.20760
C	-4.30763	0.06852	0.80920
C	-4.62682	-1.87733	-1.23728
C	-5.29230	0.17400	-0.10895
C	-5.47572	-0.83502	-1.18340
H	-4.74933	-2.62662	-2.01439
H	-5.94378	1.03130	-0.08730
C	4.32731	-0.78009	0.32468
C	4.90319	0.35886	1.14591
C	4.55195	-0.83377	-1.02663
C	5.48804	1.38067	0.47643
C	5.38302	0.22115	-1.66285
C	5.78847	1.29872	-0.97279
H	5.76790	2.29255	0.97379
H	5.61581	0.13720	-2.71743
H	0.07111	1.26596	-2.21249
C	-1.77994	1.14016	-3.11808
C	-0.95734	2.99544	-1.80141
C	-1.54342	-0.15090	-3.62459
C	-3.03639	1.76967	-3.29863
C	-2.25326	3.60094	-1.89207
C	-2.53992	-0.78699	-4.34087
H	-0.59591	-0.63030	-3.41239
C	-4.01547	1.10962	-4.07309
C	-3.23812	3.02281	-2.64226
H	-2.42885	4.52073	-1.35037
C	-3.77313	-0.14958	-4.58751
H	-2.36635	-1.78927	-4.71990
H	-4.96162	1.60823	-4.25883
H	-4.20451	3.51129	-2.73482
H	-4.53473	-0.65416	-5.17286
C	0.09901	3.50675	-0.97615
C	-0.02501	4.89798	-0.36832

H	-0.45197	5.61034	-1.08810
H	0.98443	5.26111	-0.13458
N	-0.87128	4.85966	0.82427
N	-0.79218	1.80343	-2.42766
C	-1.01203	5.99882	1.62446
C	-1.29068	5.85002	2.99716
C	-0.92244	7.30140	1.10123
C	-1.50189	6.96032	3.80634
H	-1.33470	4.85095	3.42368
C	-1.13272	8.40779	1.92464
H	-0.69356	7.45580	0.05264
C	-1.42792	8.25212	3.27820
H	-1.71650	6.81407	4.86142
H	-1.05970	9.40370	1.49599
H	-1.58859	9.11822	3.91222
H	-0.71605	4.01704	1.36617
H	0.00712	2.40151	0.03445
Cl	1.75837	3.25255	-1.65644
C	3.94938	-1.90790	-1.94617
C	5.04890	-2.92112	-2.34515
C	3.25018	-1.27153	-3.17705
H	3.17468	-2.47193	-1.39227
C	4.69724	-3.88695	-3.49349
H	5.30668	-3.52745	-1.45173
H	5.97586	-2.37931	-2.63519
C	2.01517	-2.06941	-3.62968
H	3.96014	-1.15081	-4.02438
H	2.89820	-0.24961	-2.91831
C	3.22758	-4.34167	-3.53109
H	4.98122	-3.42564	-4.46474
H	5.34084	-4.78761	-3.38608
C	2.34693	-3.38287	-4.34835
H	1.44182	-1.43760	-4.34136
H	1.34552	-2.27031	-2.76502
H	2.81869	-4.49102	-2.50826
H	3.19204	-5.33278	-4.03390
H	2.82507	-3.16245	-5.32787
H	1.38721	-3.89793	-4.57138
C	4.75443	0.38887	2.68908
C	5.24854	1.66455	3.44117
C	5.26659	-0.88656	3.40762
H	3.65937	0.39839	2.86891
C	6.77286	1.93364	3.51955
H	4.86977	1.61214	4.48634
H	4.75197	2.55860	3.00604
C	6.64248	-1.39626	2.95016
H	4.53588	-1.70951	3.30054
H	5.28746	-0.71737	4.50577
C	7.64129	0.75001	3.96508
H	6.92059	2.75128	4.25852
H	7.17415	2.33435	2.56935
C	7.74432	-0.32923	2.87659
H	6.54831	-1.87778	1.95369
H	6.96274	-2.19058	3.65942
H	7.28777	0.33220	4.93164
H	8.66510	1.14283	4.15067
H	7.74783	0.13714	1.86730
H	8.72418	-0.84501	2.97902
C	6.56875	2.42113	-1.64633
C	5.72046	3.12264	-2.74657

C	7.96291	1.91557	-2.08117
H	6.76192	3.20033	-0.87473
C	6.05580	2.75638	-4.20443
H	5.80700	4.22491	-2.62287
H	4.64305	2.90414	-2.57731
C	8.80813	2.98797	-2.78743
H	7.88155	1.00629	-2.71508
H	8.50720	1.60133	-1.16259
C	7.25669	3.54547	-4.74945
H	6.21013	1.66520	-4.33072
H	5.17384	3.02313	-4.82707
C	8.60925	2.97711	-4.30874
H	9.87999	2.76769	-2.58943
H	8.60320	3.99932	-2.37253
H	7.18461	4.61806	-4.46505
H	7.22130	3.50618	-5.86006
H	8.73560	1.94665	-4.70777
H	9.40744	3.60031	-4.76760
C	-2.74106	-3.36869	-0.29763
C	-1.86925	-3.33903	-1.58057
C	-3.65123	-4.63225	-0.14519
H	-2.03029	-3.42409	0.54727
C	-0.70596	-4.34823	-1.57254
H	-1.42241	-2.32996	-1.69600
H	-2.48551	-3.47728	-2.49146
C	-3.60931	-5.64022	-1.31081
H	-4.71272	-4.33078	-0.00590
H	-3.38519	-5.16906	0.79233
C	-1.02045	-5.70459	-0.92323
H	0.14977	-3.89912	-1.02081
H	-0.36817	-4.51257	-2.61879
C	-2.27277	-6.39984	-1.48272
H	-3.89416	-5.13926	-2.25899
H	-4.40802	-6.39434	-1.13704
H	-1.10455	-5.59429	0.17809
H	-0.14874	-6.37333	-1.09322
H	-2.11687	-6.61627	-2.56199
H	-2.36139	-7.38233	-0.96930
C	-6.63477	-0.76239	-2.17832
C	-7.15842	0.67991	-2.43164
C	-7.72030	-1.78685	-1.77177
H	-6.23258	-1.09811	-3.16141
C	-8.32986	1.12332	-1.53320
H	-7.45767	0.77268	-3.49904
H	-6.32554	1.40527	-2.31643
C	-9.01616	-1.69026	-2.59549
H	-7.94447	-1.70067	-0.68536
H	-7.29996	-2.80670	-1.91758
C	-9.69499	0.74232	-2.12616
H	-8.23010	0.72370	-0.50174
H	-8.29676	2.23184	-1.45208
C	-10.04191	-0.74122	-1.95737
H	-9.47651	-2.70193	-2.63104
H	-8.80424	-1.39570	-3.64655
H	-9.74554	1.02263	-3.20107
H	-10.47770	1.33487	-1.60435
H	-11.02707	-0.91750	-2.44144
H	-10.16803	-0.97834	-0.87813
C	-4.10857	1.18970	1.83542
C	-5.20732	1.18175	2.93020

C	-3.84957	2.55931	1.15307
H	-3.17145	0.98843	2.39452
C	-6.58364	1.76056	2.55330
H	-4.82914	1.72746	3.82219
H	-5.36231	0.13697	3.27452
C	-4.03019	3.76882	2.08644
H	-4.45044	2.70669	0.23492
H	-2.79587	2.55980	0.79841
C	-6.57832	3.17377	1.94029
H	-7.11685	1.06351	1.87439
H	-7.18889	1.79435	3.48577
C	-5.48710	4.10294	2.50018
H	-3.60884	4.65669	1.56826
H	-3.41479	3.61339	2.99856
H	-7.56578	3.63751	2.15575
H	-6.52254	3.12534	0.83454
H	-5.56156	4.11939	3.60963
H	-5.71838	5.13656	2.16077

Catalyst (2,4,6-c-Hept, 12)-Enamine protonation TS (pro-R) lowest free energy conformation
Electronic energy (Hartrees): -1870.398792
Free Energy at 1M concentration and 253 K (Hartrees): -1868.563728
Coordinates:

P	0.02620	0.16667	-0.02398
O	-1.20789	1.07442	0.53641
O	1.16922	1.18012	-0.58212
O	-0.42052	-0.54458	-1.33349
O	0.46633	-0.70392	1.10997
C	-1.56256	2.14182	-0.24874
C	-2.94928	2.21002	-0.77473
C	-3.33069	3.26285	-1.51331
H	-4.34429	3.32469	-1.89142
C	-2.37318	4.34185	-1.82749
C	-2.77621	5.44202	-2.73325
H	-3.77941	5.47185	-3.14003
C	-1.90126	6.40032	-3.05707
H	-2.19549	7.20243	-3.72152
C	-0.52751	6.36033	-2.52001
H	0.18233	7.12642	-2.80424
C	-0.15579	5.37462	-1.69515
H	0.86358	5.35131	-1.33307
C	-1.12617	4.31642	-1.30328
C	-0.73403	3.20111	-0.39123
C	1.44019	2.24740	0.23341
C	2.79952	2.37970	0.81181
C	3.11427	3.46422	1.53633
H	4.11229	3.57709	1.94284
C	2.09396	4.49330	1.81994
C	2.42021	5.62259	2.72070
H	3.41534	5.71587	3.13760
C	1.48566	6.52851	3.02807
H	1.72389	7.35181	3.68904
C	0.12229	6.40085	2.47859
H	-0.63571	7.12407	2.75052
C	-0.18069	5.38839	1.65804
H	-1.19344	5.30021	1.28783
C	0.85600	4.38854	1.28312
C	0.54348	3.24795	0.37084
C	3.81835	1.33462	0.54829

C	4.41005	1.22011	-0.83110
C	4.23160	0.49043	1.53738
C	5.33714	0.27353	-1.06556
C	5.29517	-0.50323	1.23683
C	5.80033	-0.63678	0.00250
H	5.78321	0.18427	-2.04704
H	5.66801	-1.13081	2.03435
C	-3.92739	1.13891	-0.45842
C	-4.44960	1.00945	0.79473
C	-4.41224	0.24243	-1.56301
C	-5.51052	-0.00468	1.03237
C	-5.45185	-0.57887	-1.32832
C	-6.02430	-0.72692	0.02662
H	-5.89942	-0.13233	2.03446
H	-5.85397	-1.18660	-2.12753
H	-0.30536	-2.26135	1.13029
C	-2.01755	-3.11781	1.86551
C	-1.03601	-3.53077	-0.32065
C	-1.92978	-2.43653	3.09440
C	-3.14025	-3.92638	1.56637
C	-2.18340	-4.33164	-0.64191
C	-2.95951	-2.56454	4.00957
H	-1.07204	-1.80089	3.29014
C	-4.16321	-4.05085	2.53102
C	-3.17272	-4.53784	0.27337
H	-2.22519	-4.78475	-1.62227
C	-4.07864	-3.37577	3.73453
H	-2.90323	-2.02735	4.95135
H	-5.02272	-4.67633	2.30781
H	-4.01528	-5.17586	0.01936
H	-4.87354	-3.46649	4.46744
N	-1.01368	-2.99018	0.93161
C	0.01200	-3.16895	-1.22183
C	1.45905	-3.16380	-0.75316
H	1.51483	-2.61991	0.19043
H	2.06482	-2.58899	-1.47126
N	1.97047	-4.51769	-0.55577
H	1.81651	-5.10607	-1.36521
C	3.18009	-4.77438	0.08959
C	3.83350	-6.00107	-0.14767
C	3.75302	-3.88795	1.02049
C	4.99590	-6.33623	0.53657
H	3.41080	-6.69357	-0.87226
C	4.91597	-4.24031	1.70478
H	3.29692	-2.92400	1.20955
C	5.54754	-5.46166	1.47635
H	5.47659	-7.28871	0.33014
H	5.33555	-3.54427	2.42520
H	6.45637	-5.72267	2.00897
H	-0.27677	-1.63942	-1.35760
Cl	-0.11894	-3.91026	-2.87202
C	-3.74349	0.23636	-2.93727
C	-3.22283	-1.17552	-3.28809
C	-4.70454	0.80393	-4.00840
H	-2.84610	0.88592	-2.89737
C	-2.43920	-1.22223	-4.60935
H	-4.05231	-1.91316	-3.32617
H	-2.54741	-1.51224	-2.47380
C	-3.95542	1.30578	-5.25602
H	-5.48135	0.05890	-4.28707

H	-5.24011	1.67667	-3.57363
C	-3.70664	0.23469	-6.33392
H	-4.55624	2.11702	-5.72270
H	-2.98941	1.77556	-4.96874
C	-7.16013	-1.70568	0.27646
C	-8.43384	-1.21192	-0.43796
C	-6.74199	-3.13162	-0.15222
H	-7.38060	-1.72234	1.36807
C	-9.65820	-2.11296	-0.20797
H	-8.25222	-1.09516	-1.52886
H	-8.67942	-0.20029	-0.04516
C	-7.62521	-4.22009	0.48100
H	-6.72721	-3.23620	-1.25868
H	-5.70363	-3.30257	0.19709
C	-9.59153	-3.40563	-1.03765
H	-10.55650	-1.54430	-0.53394
H	-9.79267	-2.33505	0.87314
C	-4.01112	1.87547	1.97915
C	-5.19657	2.70617	2.51090
C	-3.35802	0.98658	3.06467
H	-3.25876	2.61785	1.65396
C	-4.80995	3.65046	3.66165
H	-6.03394	2.05139	2.83335
H	-5.58217	3.32974	1.67401
C	-2.45835	1.78933	4.02045
H	-4.12182	0.41192	3.63205
H	-2.71009	0.23938	2.55811
C	-4.62104	2.89886	4.98980
H	-5.64404	4.37387	3.79384
H	-3.90589	4.24492	3.40453
C	3.66601	0.53555	2.96170
C	3.11637	-0.84316	3.38560
C	4.74112	1.06741	3.93805
H	2.79637	1.22151	2.99718
C	2.44101	-0.83216	4.76630
H	3.91205	-1.61608	3.37449
H	2.36037	-1.16165	2.64155
C	4.12825	1.62136	5.23728
H	5.50223	0.28807	4.16091
H	5.27935	1.90741	3.44552
C	3.46987	-0.82370	5.90882
H	1.84228	-1.76548	4.85237
H	1.72829	0.01632	4.85897
C	4.02230	2.17846	-1.96052
C	5.24509	3.01817	-2.40449
C	3.36829	1.40602	-3.12750
H	3.26105	2.88935	-1.58870
C	4.83601	4.30376	-3.14722
H	5.94670	2.41579	-3.02163
H	5.80236	3.32913	-1.49259
C	2.86204	2.31898	-4.25551
H	4.06674	0.64969	-3.54536
H	2.49776	0.84007	-2.73358
C	4.00975	2.82645	-5.14381
H	2.17861	1.71543	-4.89220
H	2.26173	3.16263	-3.84963
C	6.84274	-1.70107	-0.29876
C	6.33096	-2.64305	-1.41599
C	8.18835	-1.02921	-0.64094
H	7.00470	-2.31019	0.61921

C	7.10520	-3.97152	-1.46470
H	6.35430	-2.14179	-2.40788
H	5.26594	-2.88735	-1.20907
C	9.32177	-2.02623	-0.93322
H	8.07428	-0.33115	-1.49864
H	8.49653	-0.41252	0.23233
C	9.19189	-2.65943	-2.32865
H	10.27876	-1.46064	-0.90353
H	9.38384	-2.80403	-0.14124
C	8.35040	-3.94767	-2.36784
C	3.92498	0.57847	6.35177
C	4.65500	4.13986	-4.66722
C	-3.18944	2.38913	5.23540
C	-8.88142	-4.57615	-0.33447
C	-3.37004	-1.18098	-5.83243
H	-2.87776	0.58928	-6.98505
H	-4.60916	0.16229	-6.97966
H	-2.87643	-1.72432	-6.66781
H	-4.31148	-1.73472	-5.62337
H	-1.67824	-0.41270	-4.65354
H	-1.88620	-2.18583	-4.63590
H	-9.10804	-3.21209	-2.02023
H	-10.62988	-3.73125	-1.26676
H	-8.60438	-5.32383	-1.10970
H	-9.60394	-5.07611	0.34721
H	-7.01950	-5.14700	0.58567
H	-7.91946	-3.93223	1.51369
H	-1.92437	2.59528	3.47073
H	-1.66448	1.11205	4.40600
H	-3.23400	1.62170	6.03905
H	-2.57333	3.22721	5.62880
H	-4.87913	3.58795	5.82358
H	-5.33653	2.05065	5.06203
H	4.87819	0.46649	6.91356
H	3.17935	0.98533	7.06960
H	4.80528	2.40857	5.63584
H	3.16414	2.13392	5.02722
H	4.35893	-1.43334	5.63560
H	3.01723	-1.32476	6.79242
H	7.39691	-4.29221	-0.44170
H	6.42481	-4.76270	-1.84950
H	8.99691	-4.80681	-2.08388
H	8.03338	-4.12013	-3.41970
H	10.21023	-2.91110	-2.69790
H	8.77811	-1.92264	-3.05159
H	3.90947	4.73294	-2.70705
H	5.62732	5.06835	-2.98564
H	4.04168	4.99234	-5.03320
H	5.64933	4.22999	-5.15706
H	4.79367	2.04487	-5.25002
H	3.61158	2.99978	-6.16760

Catalyst (2,4,6-c-Hept, 12)-Enamine protonation TS (pro-R) alternative conformation
Electronic energy (Hartrees): -1870.398288
Free Energy at 1M concentration and 253 K (Hartrees): -1868.563418
Coordinates:

P	0.01195	0.15950	-0.02234
O	-1.27154	0.96412	0.58170
O	1.17649	1.25190	-0.33034

O	-0.33073	-0.33363	-1.45752
O	0.38413	-0.87904	0.98739
C	-1.57168	2.15143	-0.03658
C	-2.90567	2.30915	-0.67034
C	-3.24157	3.48015	-1.23196
H	-4.21631	3.60571	-1.68823
C	-2.28022	4.59984	-1.26052
C	-2.62334	5.84434	-1.98636
H	-3.59114	5.94791	-2.46118
C	-1.73714	6.84291	-2.06417
H	-1.98726	7.75077	-2.59773
C	-0.40969	6.70464	-1.43517
H	0.31185	7.50670	-1.52278
C	-0.09290	5.58741	-0.77068
H	0.89575	5.49754	-0.34046
C	-1.07910	4.47891	-0.65003
C	-0.74769	3.21822	0.07878
C	1.38718	2.16286	0.67131
C	2.71047	2.19778	1.34007
C	2.96735	3.13637	2.26379
H	3.94000	3.18128	2.73922
C	1.91862	4.09642	2.66279
C	2.17755	5.05199	3.76415
H	3.14421	5.07076	4.25204
C	1.21704	5.89213	4.16443
H	1.40605	6.58836	4.97130
C	-0.10833	5.86364	3.51649
H	-0.88822	6.53066	3.86058
C	-0.35037	5.00861	2.51616
H	-1.33674	4.98797	2.07204
C	0.71687	4.08718	2.04025
C	0.47279	3.12361	0.92552
C	3.76289	1.23135	0.94273
C	4.42203	1.37891	-0.40284
C	4.14492	0.22419	1.77979
C	5.37710	0.50092	-0.75998
C	5.24296	-0.68391	1.35771
C	5.80797	-0.58401	0.14633
H	5.86935	0.59684	-1.71853
H	5.58975	-1.44278	2.04524
C	-3.87503	1.18421	-0.68309
C	-4.52355	0.79875	0.45233
C	-4.21328	0.52673	-1.99300
C	-5.58639	-0.23533	0.35854
C	-5.25049	-0.32981	-2.04605
C	-5.97057	-0.73946	-0.82391
H	-6.08480	-0.55937	1.26230
H	-5.54369	-0.77135	-2.98953
H	-0.33951	-2.43405	0.72282
C	-2.04195	-3.44479	1.25917
C	-0.99919	-3.46113	-0.93822
C	-1.99833	-2.98525	2.58928
C	-3.13566	-4.21601	0.79749
C	-2.12514	-4.20821	-1.42404
C	-3.03953	-3.30030	3.44429
H	-1.16507	-2.36954	2.91258
C	-4.16821	-4.54006	1.70298
C	-3.13003	-4.58731	-0.58386
H	-2.13644	-4.48444	-2.46894
C	-4.12591	-4.08491	3.00775

H	-3.01742	-2.93388	4.46602
H	-5.00221	-5.14209	1.35380
H	-3.95390	-5.18431	-0.96618
H	-4.92901	-4.32714	3.69592
N	-1.02285	-3.13577	0.38678
C	0.07427	-2.95196	-1.73044
C	1.50377	-3.01924	-1.21565
H	1.52026	-2.63891	-0.19306
H	2.12964	-2.33156	-1.80592
N	2.01973	-4.38573	-1.22622
H	1.88151	-4.83923	-2.12086
C	3.21869	-4.73871	-0.60766
C	3.87776	-5.91648	-1.01582
C	3.77492	-4.00323	0.45531
C	5.02921	-6.35177	-0.37062
H	3.46839	-6.49193	-1.84311
C	4.92581	-4.45657	1.09940
H	3.31486	-3.07727	0.77709
C	5.56311	-5.63006	0.70017
H	5.51471	-7.26278	-0.71009
H	5.33102	-3.87850	1.92464
H	6.46256	-5.96987	1.20344
H	-0.20387	-1.41180	-1.63350
Cl	0.00458	-3.40814	-3.48430
C	-3.39745	0.80281	-3.25610
C	-2.81819	-0.50558	-3.83846
C	-4.24219	1.58969	-4.28565
H	-2.52016	1.42172	-2.97992
C	-1.88113	-0.27199	-5.03425
H	-3.62627	-1.20684	-4.13602
H	-2.24166	-1.02012	-3.04195
C	-3.36558	2.34673	-5.29985
H	-4.96410	0.92354	-4.80648
H	-4.84509	2.34935	-3.74106
C	-2.96429	1.53225	-6.54380
H	-3.92843	3.23919	-5.65145
H	-2.45228	2.74462	-4.80582
C	-7.10695	-1.74392	-0.92272
C	-6.70151	-3.06263	-0.23486
C	-8.41770	-1.13737	-0.36450
H	-7.26561	-1.97193	-2.00039
C	-7.76891	-4.16445	-0.31947
H	-6.43210	-2.88472	0.82869
H	-5.78691	-3.44019	-0.74002
C	-9.66684	-1.90393	-0.83329
H	-8.39208	-1.07425	0.74523
H	-8.50779	-0.09529	-0.74494
C	-8.93005	-3.91954	0.65765
H	-7.28140	-5.12310	-0.03684
H	-8.13613	-4.28603	-1.36169
C	-4.22296	1.38755	1.83280
C	-5.46835	2.09777	2.40163
C	-3.67809	0.27709	2.76344
H	-3.44825	2.17291	1.75509
C	-5.22094	2.76130	3.76683
H	-6.32949	1.40043	2.47925
H	-5.76650	2.89472	1.68480
C	-2.90088	0.84259	3.96447
H	-4.48992	-0.40140	3.10451
H	-2.96752	-0.34718	2.17950

C	-5.17139	1.73407	4.91021
H	-6.07234	3.44937	3.96220
H	-4.30072	3.38540	3.74714
C	3.50512	-0.00533	3.15386
C	2.96062	-1.44440	3.27816
C	4.51733	0.34129	4.27064
H	2.62218	0.65422	3.27042
C	2.21393	-1.70231	4.59666
H	3.77078	-2.19388	3.16463
H	2.25105	-1.62246	2.44634
C	3.82698	0.63159	5.61591
H	5.28128	-0.45876	4.38307
H	5.06320	1.26509	3.97633
C	3.18153	-1.90254	5.77469
H	1.62926	-2.64011	4.47116
H	1.48141	-0.89321	4.80931
C	4.06852	2.52356	-1.35659
C	5.29387	3.44302	-1.58119
C	3.48312	1.97106	-2.67499
H	3.27816	3.14696	-0.89878
C	4.89381	4.83901	-2.09418
H	6.03326	2.97166	-2.26466
H	5.80428	3.58675	-0.60259
C	3.00857	3.07005	-3.63896
H	4.21518	1.31322	-3.19064
H	2.60794	1.33154	-2.43369
C	4.18385	3.74554	-4.36440
H	2.36586	2.58611	-4.40677
H	2.37428	3.81765	-3.11386
C	6.88105	-1.56665	-0.29292
C	6.43174	-2.30058	-1.58010
C	8.22780	-0.83181	-0.45238
H	7.01645	-2.32713	0.50901
C	7.22876	-3.59296	-1.82767
H	6.48830	-1.63144	-2.46598
H	5.36304	-2.58690	-1.46680
C	9.38908	-1.75096	-0.86581
H	8.13754	0.00547	-1.17809
H	8.48902	-0.37606	0.52842
C	9.32739	-2.12980	-2.35507
H	10.33431	-1.19038	-0.69576
H	9.43175	-2.65551	-0.22081
C	8.51046	-3.39918	-2.65632
C	3.58699	-0.60645	6.49952
C	4.78188	4.95577	-3.62523
C	-3.77015	1.16318	5.19383
C	-10.07775	-3.07200	0.08076
C	-2.66010	0.03960	-6.32283
H	-2.07059	2.01528	-6.99633
H	-3.77990	1.60596	-7.29621
H	-2.05891	-0.31202	-7.18982
H	-3.60787	-0.54120	-6.35439
H	-1.13588	0.52263	-4.81141
H	-1.31029	-1.21117	-5.20034
H	-8.55637	-3.45174	1.59491
H	-9.35416	-4.90353	0.95525
H	-10.66985	-2.66985	0.93191
H	-10.75813	-3.73608	-0.49613
H	-10.52155	-1.19343	-0.87267
H	-9.53138	-2.27326	-1.87307

H	-2.32549	1.74671	3.66752
H	-2.14167	0.09146	4.27585
H	-3.89466	0.23525	5.79408
H	-3.21218	1.88326	5.83169
H	-5.52760	2.22668	5.84139
H	-5.88105	0.90043	4.71502
H	4.51233	-0.81494	7.08011
H	2.79822	-0.35048	7.24058
H	4.46669	1.33494	6.19301
H	2.86504	1.16539	5.45453
H	4.09481	-2.44168	5.43997
H	2.69284	-2.56627	6.52128
H	7.48224	-4.08689	-0.86521
H	6.57798	-4.30975	-2.37524
H	9.15907	-4.28864	-2.49857
H	8.24107	-3.38735	-3.73521
H	10.36420	-2.30267	-2.71821
H	8.93109	-1.28123	-2.95473
H	3.94131	5.17071	-1.62598
H	5.66315	5.56885	-1.75897
H	4.16834	5.85407	-3.85641
H	5.79450	5.14598	-4.04381
H	4.98685	3.00582	-4.57581
H	3.82745	4.10017	-5.35634

Catalyst (2,4,6-c-Hept, 12)-Enamine protonation TS (pro-R) alternative conformation
Electronic energy (Hartrees): -1870.398501
Free Energy at 1M concentration and 253 K (Hartrees): -1868.563374
Coordinates:

P	-0.05826	0.01508	0.28053
O	-1.17453	0.83099	1.13098
O	1.17875	1.03922	-0.01726
O	-0.54232	-0.47684	-1.04455
O	0.42172	-1.05927	1.30864
C	-1.45983	2.06898	0.61237
C	-2.79468	2.32167	0.01036
C	-3.10848	3.55378	-0.41765
H	-4.08701	3.75175	-0.83720
C	-2.10365	4.63389	-0.39227
C	-2.42084	5.94413	-1.00550
H	-3.40737	6.13006	-1.41169
C	-1.48737	6.89935	-1.07023
H	-1.71826	7.85476	-1.52336
C	-0.13395	6.64764	-0.54021
H	0.62540	7.41467	-0.62150
C	0.15953	5.47091	0.02459
H	1.16766	5.29952	0.37697
C	-0.87780	4.40810	0.13275
C	-0.57358	3.07958	0.74452
C	1.54412	1.86878	1.01466
C	2.95610	1.85271	1.47146
C	3.36155	2.70477	2.42553
H	4.39745	2.71493	2.74320
C	2.40264	3.63765	3.04916
C	2.83582	4.49817	4.17388
H	3.85819	4.45626	4.52850
C	1.96510	5.32846	4.75832
H	2.28159	5.95582	5.58155
C	0.56696	5.38478	4.28935

H	-0.13701	6.04551	4.77862
C	0.16753	4.61534	3.27010
H	-0.86653	4.65714	2.95381
C	1.13070	3.70472	2.59458
C	0.71158	2.84083	1.45186
C	3.93934	0.96222	0.80934
C	4.39689	1.28200	-0.58915
C	4.44141	-0.12746	1.45402
C	5.33467	0.50426	-1.16232
C	5.49615	-0.93526	0.78983
C	5.93987	-0.63282	-0.43981
H	5.65336	0.69793	-2.17854
H	5.92495	-1.77531	1.31968
C	-3.76901	1.21567	-0.15891
C	-4.43217	0.69268	0.91004
C	-4.10128	0.74271	-1.54939
C	-5.46256	-0.35453	0.67692
C	-5.13190	-0.10484	-1.71860
C	-5.83081	-0.70744	-0.56339
H	-5.95853	-0.80202	1.52887
H	-5.44208	-0.38924	-2.71437
H	0.22248	-1.85245	-1.62713
C	1.77686	-2.31362	-2.88630
C	1.20592	-3.48497	-0.83323
C	1.47827	-1.25750	-3.76760
C	2.92640	-3.11517	-3.08390
C	2.39411	-4.27585	-1.00361
C	2.31631	-1.01857	-4.84181
H	0.61817	-0.62937	-3.56483
C	3.75010	-2.85702	-4.20064
C	3.19330	-4.11592	-2.09647
H	2.61570	-5.02310	-0.25510
C	3.45154	-1.82261	-5.06867
H	2.09643	-0.19492	-5.51417
H	4.62975	-3.47516	-4.35759
H	4.06332	-4.75514	-2.22306
H	4.09374	-1.62309	-5.92017
C	0.32752	-3.54099	0.29182
H	0.49076	-2.08347	0.91366
C	-1.18687	-3.55623	0.07684
H	-1.50186	-4.57906	-0.17437
H	-1.42500	-2.92030	-0.78729
N	-1.90398	-3.12436	1.25913
N	0.95677	-2.56437	-1.80954
C	-3.12162	-3.62874	1.67255
C	-3.97090	-4.35591	0.81667
C	-3.55159	-3.40189	2.99738
C	-5.20087	-4.82554	1.27701
H	-3.68114	-4.53967	-0.21212
C	-4.79085	-3.85233	3.43242
H	-2.89625	-2.86840	3.68164
C	-5.63169	-4.57179	2.57768
H	-5.83134	-5.39464	0.60000
H	-5.09703	-3.64953	4.45532
H	-6.59687	-4.92976	2.92129
H	-1.37643	-2.59594	1.93666
Cl	0.79238	-4.76409	1.54121
C	3.78401	2.43041	-1.39445
C	4.85410	3.46390	-1.79955
C	2.99316	1.87288	-2.60364

H	3.07704	2.98640	-0.75282
C	4.26457	4.69101	-2.51604
H	5.63837	3.00148	-2.43621
H	5.35889	3.81930	-0.87399
C	1.92093	2.85548	-3.10656
H	3.67421	1.57309	-3.42949
H	2.46516	0.94978	-2.28189
C	2.42174	3.87993	-4.14159
H	5.05238	5.47547	-2.53654
H	3.40586	5.11133	-1.94751
H	1.10381	2.26888	-3.58096
H	1.44858	3.38881	-2.25245
C	3.96594	-0.56645	2.84089
C	3.43533	-2.02065	2.79547
C	5.10122	-0.37253	3.86675
H	3.12372	0.07340	3.17191
C	2.52889	-2.34837	3.99661
H	4.26683	-2.75462	2.72480
H	2.82683	-2.14295	1.87321
C	4.69770	-0.72968	5.30662
H	6.00197	-0.95454	3.57446
H	5.39885	0.69877	3.85578
C	4.64183	-2.24938	5.53286
H	1.92243	-1.46265	4.28567
H	1.79481	-3.12139	3.68666
H	3.73594	-0.24534	5.58369
H	5.47469	-0.31230	5.98384
C	7.04773	-1.43713	-1.10012
C	8.33457	-1.40180	-0.23970
C	6.55017	-2.86445	-1.39979
H	7.27644	-0.95854	-2.07856
C	9.58789	-1.79707	-1.04021
H	8.23240	-2.03957	0.66517
H	8.48571	-0.35816	0.11628
C	7.58300	-3.74939	-2.11388
H	6.20080	-3.36768	-0.47190
H	5.66937	-2.77425	-2.06693
C	9.89327	-3.30557	-1.04210
H	10.46639	-1.27844	-0.59732
H	9.51494	-1.42976	-2.08695
H	7.04372	-4.63953	-2.50593
H	8.01907	-3.22530	-2.99202
C	-3.31904	1.23239	-2.77081
C	-2.77565	0.05103	-3.60716
C	-4.18501	2.19909	-3.61350
H	-2.42347	1.78027	-2.41441
C	-1.87308	0.49651	-4.76959
H	-3.60185	-0.57436	-4.00613
H	-2.18059	-0.60952	-2.94376
C	-3.32806	3.13659	-4.48377
H	-4.91964	1.64201	-4.23488
H	-4.77883	2.83952	-2.92599
C	-2.95526	2.57435	-5.86843
H	-1.32935	-0.40263	-5.13321
H	-1.10505	1.22273	-4.42523
H	-3.89502	4.07952	-4.64612
H	-2.40306	3.43394	-3.94265
C	-4.20585	1.17060	2.34710
C	-5.49466	1.80941	2.90472
C	-3.68359	0.00307	3.21452

H	-3.44265	1.97189	2.37062
C	-5.33776	2.36238	4.33108
H	-6.34221	1.09154	2.87608
H	-5.77247	2.65751	2.24025
C	-2.99023	0.48529	4.50030
H	-4.49883	-0.71222	3.45548
H	-2.92526	-0.55496	2.62336
C	-3.93462	0.67488	5.70090
H	-6.21360	3.01713	4.53289
H	-4.43203	3.00200	4.41451
H	-2.22571	-0.26880	4.79024
H	-2.42896	1.42684	4.31365
C	-6.95916	-1.70199	-0.78483
C	-8.18239	-0.96043	-1.36098
C	-6.48466	-2.87199	-1.68092
H	-7.26033	-2.12222	0.20182
C	-9.40535	-1.86352	-1.58957
H	-7.91536	-0.44154	-2.30781
H	-8.47793	-0.16909	-0.63670
C	-7.40789	-4.09894	-1.58278
H	-6.37808	-2.55354	-2.74034
H	-5.47447	-3.18486	-1.34052
C	-8.57443	-4.10003	-2.58642
H	-9.62137	-2.47880	-0.68911
H	-6.80163	-5.01145	-1.77389
H	-7.80592	-4.20748	-0.55053
C	8.67922	-4.24426	-1.15676
H	10.43899	-3.56185	-0.10763
H	10.58792	-3.51410	-1.88512
H	8.25587	-4.43066	-0.14539
H	9.04728	-5.22853	-1.52063
C	3.27465	-2.88792	5.23009
H	5.43134	-2.75972	4.93880
H	4.88044	-2.45578	6.59923
H	2.61440	-2.75751	6.11540
H	3.42782	-3.98227	5.10468
C	3.86737	4.38085	-3.96896
H	1.73466	4.75425	-4.12201
H	2.33679	3.43119	-5.15562
H	3.97192	5.31210	-4.56768
H	4.57322	3.64837	-4.41807
C	-2.68302	1.06174	-5.94752
H	-3.77591	2.80958	-6.58110
H	-2.05511	3.11850	-6.22973
H	-3.64499	0.51485	-6.05701
H	-2.11637	0.87232	-6.88549
H	-10.28472	-1.20070	-1.74439
C	-9.25144	-2.74190	-2.84194
H	-10.26285	-2.94286	-3.25831
H	-8.69784	-2.19309	-3.63507
H	-8.20805	-4.49027	-3.56121
H	-9.34036	-4.81922	-2.22197
C	-5.32957	1.24559	5.38813
H	-4.07198	-0.30671	6.20536
H	-3.43023	1.34252	6.43335
H	-6.01082	0.41999	5.08641
H	-5.74540	1.65406	6.33516

Catalyst (2,4,6-c-Hept, 12)-Enamine protonation TS (pro-R) alternative conformation

Electronic energy (Hartrees): -1870.396609
Free Energy at 1M concentration and 253 K (Hartrees): -1868.562065
Coordinates:

P	0.01035	0.14017	0.06593
O	-1.22007	0.96748	0.74739
O	1.13497	1.21663	-0.41255
O	-0.46099	-0.42931	-1.30306
O	0.47609	-0.84661	1.08972
C	-1.59382	2.11605	0.09792
C	-2.98578	2.23387	-0.40550
C	-3.38082	3.36251	-1.01383
H	-4.39798	3.46003	-1.37431
C	-2.43153	4.47549	-1.21416
C	-2.84561	5.66452	-1.99387
H	-3.85335	5.73515	-2.38405
C	-1.97436	6.65237	-2.22633
H	-2.27630	7.51898	-2.80015
C	-0.59424	6.55809	-1.71289
H	0.11301	7.34938	-1.92566
C	-0.21353	5.49317	-0.99817
H	0.81068	5.43321	-0.65444
C	-1.17913	4.39789	-0.70870
C	-0.77335	3.18928	0.06798
C	1.42989	2.20105	0.49528
C	2.82796	2.33860	0.97002
C	3.15399	3.32451	1.81950
H	4.18044	3.44585	2.14579
C	2.11822	4.25733	2.30803
C	2.46407	5.27562	3.32603
H	3.47790	5.34883	3.69961
C	1.52462	6.10640	3.79123
H	1.77735	6.84874	4.53724
C	0.13761	6.00701	3.29687
H	-0.62180	6.66915	3.69235
C	-0.18347	5.09551	2.37143
H	-1.21059	5.02499	2.03806
C	0.85566	4.18095	1.82649
C	0.52157	3.15258	0.79724
C	3.87883	1.46729	0.39644
C	4.29507	1.67919	-1.03492
C	4.44872	0.47167	1.13088
C	5.20402	0.84897	-1.57913
C	5.46933	-0.39837	0.49087
C	5.80774	-0.25001	-0.79898
H	5.52233	0.99035	-2.60409
H	5.93385	-1.18192	1.07355
C	-3.95195	1.12345	-0.21393
C	-4.46904	0.84494	1.01655
C	-4.43319	0.35781	-1.41465
C	-5.51769	-0.20201	1.13771
C	-5.46298	-0.49671	-1.27403
C	-6.02705	-0.80891	0.05636
H	-5.90149	-0.44916	2.11922
H	-5.86427	-1.00935	-2.13766
H	-0.29159	-2.40352	0.92976
C	-1.98670	-3.35557	1.58568
C	-1.02640	-3.51643	-0.64284
C	-1.89608	-2.80779	2.87925
C	-3.10438	-4.13944	1.21101
C	-2.16854	-4.29155	-1.03748

C	-2.91652	-3.04373	3.78359
H	-1.04433	-2.18568	3.13492
C	-4.11740	-4.37722	2.16447
C	-3.14432	-4.60888	-0.14009
H	-2.21539	-4.63687	-2.06059
C	-4.02947	-3.83345	3.43265
H	-2.85775	-2.60830	4.77640
H	-4.97266	-4.98410	1.88152
H	-3.98195	-5.22713	-0.45259
H	-4.81710	-4.01045	4.15762
N	-0.99283	-3.11730	0.66210
C	0.00520	-3.04359	-1.50873
C	1.46310	-3.09900	-1.07571
H	1.55017	-2.70301	-0.06393
H	2.04721	-2.41972	-1.71541
N	1.99463	-4.46648	-1.10905
H	1.97762	-4.83519	-2.05272
C	3.16986	-4.81135	-0.42908
C	4.09852	-5.68084	-1.03093
C	3.42498	-4.37624	0.88411
C	5.23515	-6.09984	-0.34448
H	3.92292	-6.02328	-2.04832
C	4.57224	-4.79004	1.55706
H	2.70871	-3.73807	1.38908
C	5.48873	-5.65215	0.95285
H	5.93651	-6.76758	-0.83751
H	4.74440	-4.44206	2.57199
H	6.38198	-5.96864	1.48187
H	-0.29827	-1.51058	-1.45313
Cl	-0.15770	-3.56479	-3.23796
C	-3.77566	0.52603	-2.78416
C	-3.25192	-0.82757	-3.31422
C	-4.74777	1.21796	-3.76869
H	-2.88051	1.16983	-2.66969
C	-2.47590	-0.70162	-4.63491
H	-4.07885	-1.55787	-3.44205
H	-2.57092	-1.26275	-2.55313
C	-4.01016	1.88001	-4.94655
H	-5.52144	0.50852	-4.13517
H	-5.28667	2.02421	-3.22377
C	-3.76000	0.95658	-6.15313
H	-4.61979	2.73910	-5.30324
H	-3.04590	2.31745	-4.60703
C	-7.14959	-1.82447	0.19427
C	-8.43230	-1.26832	-0.45530
C	-6.71479	-3.18579	-0.39698
H	-7.36562	-1.96989	1.27717
C	-9.64406	-2.20605	-0.32826
H	-8.25653	-1.02378	-1.52579
H	-8.68936	-0.31223	0.05247
C	-7.58176	-4.35157	0.10808
H	-6.70232	-3.16166	-1.50816
H	-5.67319	-3.38228	-0.07151
C	-9.56386	-3.39311	-1.30206
H	-10.55086	-1.61546	-0.58423
H	-9.77157	-2.55339	0.72021
C	-4.03998	1.57741	2.29100
C	-5.23535	2.33154	2.90822
C	-3.37920	0.57952	3.27182
H	-3.29380	2.35823	2.05253

C	-4.86335	3.14267	4.16070
H	-6.06765	1.63663	3.14972
H	-5.62377	3.04250	2.14559
C	-2.49482	1.27977	4.31808
H	-4.13718	-0.06619	3.76586
H	-2.71861	-0.09557	2.68639
C	-4.67389	2.24680	5.39600
H	-5.70500	3.83885	4.36919
H	-3.96391	3.77065	3.97792
C	4.08199	0.19873	2.59272
C	3.56509	-1.24388	2.75716
C	5.29260	0.50597	3.50462
H	3.24533	0.85486	2.90443
C	3.10145	-1.57530	4.18381
H	4.33220	-1.97925	2.44268
H	2.70181	-1.37963	2.07629
C	4.88230	0.71504	4.97368
H	6.07004	-0.28466	3.42157
H	5.75918	1.45447	3.15618
C	4.28818	-1.83055	5.12738
H	2.50090	-2.50992	4.12979
H	2.43048	-0.78368	4.58291
C	3.74245	2.83760	-1.87157
C	4.87697	3.80883	-2.27938
C	2.94597	2.30376	-3.08168
H	3.02818	3.41898	-1.25915
C	4.34619	5.20530	-2.65509
H	5.49222	3.38838	-3.10435
H	5.55365	3.93919	-1.40538
C	2.26641	3.41322	-3.90020
H	3.59444	1.69198	-3.74494
H	2.15063	1.62399	-2.71011
C	3.26600	4.15928	-4.79878
H	1.51141	2.92874	-4.55750
H	1.71309	4.11708	-3.24031
C	6.78477	-1.19901	-1.47258
C	8.15134	-1.18894	-0.74394
C	6.15564	-2.60454	-1.57459
H	6.94683	-0.83703	-2.51236
C	9.28559	-1.76024	-1.61302
H	8.09567	-1.72923	0.22607
H	8.41293	-0.13249	-0.51130
C	7.04624	-3.62824	-2.29601
H	5.88479	-2.98590	-0.56653
H	5.20492	-2.51883	-2.14662
C	8.19319	-4.12519	-1.40122
H	6.41090	-4.50200	-2.55675
H	7.43063	-3.21788	-3.25497
C	9.47795	-3.28270	-1.49044
C	4.83934	-0.57076	5.81933
C	3.94586	5.36871	-4.13285
C	-3.23894	1.72475	5.59021
C	-8.83608	-4.62761	-0.74061
C	-3.41410	-0.50967	-5.83785
H	-2.93562	1.39608	-6.75647
H	-4.66469	0.96204	-6.79985
H	-2.92287	-0.93946	-6.73824
H	-4.35176	-1.09050	-5.69628
H	-1.71879	0.11088	-4.57974
H	-1.91888	-1.65111	-4.78710

H	-9.08659	-3.08087	-2.25673
H	-10.59878	-3.70377	-1.56498
H	-8.55206	-5.27675	-1.59776
H	-9.54959	-5.21272	-0.11995
H	-6.96381	-5.27632	0.10390
H	-7.87612	-4.18870	1.16766
H	-1.97104	2.15131	3.86761
H	-1.69262	0.57393	4.62735
H	-3.28054	0.86739	6.29731
H	-2.63361	2.51625	6.08400
H	-4.94244	2.83432	6.30118
H	-5.38174	1.38962	5.36710
H	5.86787	-0.79806	6.17606
H	4.22728	-0.36431	6.72464
H	5.61697	1.40213	5.44812
H	3.90130	1.23461	5.03594
H	5.11111	-2.34634	4.58539
H	3.95941	-2.53497	5.92261
H	9.13538	-1.48746	-2.68021
H	10.23975	-1.27707	-1.30798
H	10.07026	-3.62531	-2.36707
H	10.09171	-3.49481	-0.58763
H	8.44884	-5.16536	-1.70003
H	7.86168	-4.18501	-0.34150
H	3.48855	5.48664	-2.00554
H	5.14231	5.95157	-2.44024
H	3.26688	6.24627	-4.20896
H	4.85471	5.61771	-4.72343
H	4.04226	3.46047	-5.18051
H	2.72294	4.53086	-5.69531

Catalyst (2,4,6-c-Hept, 12)-Enamine protonation TS (pro-R) alternative conformation
Electronic energy (Hartrees): -1870.396245
Free Energy at 1M concentration and 253 K (Hartrees): -1868.561739
Coordinates:

P	0.00264	0.13028	-0.04019
O	1.28372	0.86892	-0.72960
O	-1.14057	1.25838	0.22668
O	0.37575	-0.26678	1.41734
O	-0.40409	-0.97355	-0.96435
C	1.60720	2.09921	-0.21651
C	2.95063	2.29431	0.38651
C	3.30442	3.50376	0.84697
H	4.28572	3.65602	1.28034
C	2.35194	4.63049	0.80051
C	2.71066	5.92254	1.42926
H	3.68577	6.05531	1.88126
C	1.82871	6.92778	1.44959
H	2.08976	7.87009	1.91364
C	0.49123	6.75081	0.85240
H	-0.22718	7.55932	0.89528
C	0.16157	5.59134	0.27213
H	-0.83447	5.47559	-0.13409
C	1.14186	4.47208	0.21732
C	0.79155	3.16079	-0.40508
C	-1.37998	2.10484	-0.82511
C	-2.74801	2.16877	-1.39294
C	-3.01953	3.01961	-2.39401
H	-4.02527	3.09031	-2.79181

C	-1.95320	3.87760	-2.94977
C	-2.23545	4.73996	-4.12002
H	-3.22477	4.75273	-4.56028
C	-1.26852	5.50404	-4.64011
H	-1.47473	6.13243	-5.49690
C	0.08578	5.48566	-4.05388
H	0.86826	6.09206	-4.49156
C	0.34926	4.71521	-2.99208
H	1.35375	4.70061	-2.58977
C	-0.72184	3.87830	-2.38789
C	-0.45228	3.00833	-1.20515
C	-3.83560	1.39262	-0.75487
C	-4.32629	1.81468	0.60467
C	-4.37293	0.29891	-1.36328
C	-5.27144	1.07569	1.21472
C	-5.43737	-0.46317	-0.66034
C	-5.84342	-0.12516	0.57296
H	-5.64210	1.36687	2.18923
H	-5.87798	-1.32298	-1.14596
C	3.90728	1.16321	0.48510
C	4.54457	0.68085	-0.61883
C	4.24744	0.61076	1.84231
C	5.59459	-0.35640	-0.44815
C	5.27434	-0.25194	1.95830
C	5.97937	-0.76984	0.76880
H	6.08279	-0.75854	-1.32572
H	5.57056	-0.61742	2.93279
H	0.32223	-2.51028	-0.59616
C	2.00280	-3.57855	-1.09255
C	1.00088	-3.43117	1.11947
C	1.94313	-3.20814	-2.44937
C	3.09649	-4.32890	-0.59759
C	2.12621	-4.15912	1.63508
C	2.96805	-3.58956	-3.29744
H	1.11185	-2.60474	-2.79989
C	4.11197	-4.72203	-1.49463
C	3.11016	-4.60846	0.80558
H	2.15115	-4.36580	2.69569
C	4.05386	-4.35407	-2.82627
H	2.93366	-3.29027	-4.34051
H	4.94643	-5.30686	-1.11824
H	3.93204	-5.19185	1.21240
H	4.84437	-4.64874	-3.50854
N	1.00112	-3.19961	-0.22652
C	-0.04926	-2.85454	1.89280
C	-1.49569	-2.95850	1.43194
H	-1.55286	-2.69932	0.37458
H	-2.09085	-2.19825	1.96070
N	-2.04135	-4.30708	1.63011
H	-2.06073	-4.54234	2.61565
C	-3.20247	-4.72692	0.96649
C	-4.17920	-5.46752	1.65737
C	-3.39464	-4.49783	-0.40804
C	-5.30184	-5.96134	0.99766
H	-4.05265	-5.65001	2.72219
C	-4.52904	-4.98165	-1.05531
H	-2.63816	-3.96826	-0.97631
C	-5.49359	-5.71535	-0.36255
H	-6.04108	-6.52672	1.55865
H	-4.65229	-4.79291	-2.11836

H	-6.37599	-6.08845	-0.87254
H	0.23962	-1.32694	1.66893
Cl	0.06901	-3.15038	3.67759
C	3.44730	1.00369	3.08446
C	2.86456	-0.24222	3.78763
C	4.30834	1.87179	4.03187
H	2.57150	1.60242	2.76272
C	1.93834	0.10669	4.96367
H	3.67048	-0.91874	4.14262
H	2.27905	-0.82368	3.04580
C	3.44729	2.72685	4.97940
H	5.02802	1.24804	4.60561
H	4.91442	2.57253	3.41654
C	3.04621	2.03270	6.29444
H	4.02212	3.64106	5.24529
H	2.53470	3.08895	4.45728
C	7.10063	-1.78132	0.94120
C	6.66988	-3.14377	0.36252
C	8.41698	-1.24238	0.32945
H	7.26278	-1.92551	2.03272
C	7.72013	-4.25271	0.52853
H	6.39600	-3.04663	-0.71026
H	5.75298	-3.46493	0.90117
C	9.65692	-1.98954	0.85101
H	8.38499	-1.26720	-0.78162
H	8.52601	-0.17499	0.62535
C	8.87852	-4.10493	-0.47109
H	7.21544	-5.22255	0.32526
H	8.09224	-4.29739	1.57514
C	4.24537	1.16458	-2.03973
C	5.49723	1.81506	-2.66348
C	3.68567	-0.00821	-2.88034
H	3.47846	1.96142	-2.02109
C	5.25614	2.37109	-4.07687
H	6.35130	1.10513	-2.68544
H	5.80403	2.66312	-2.01190
C	2.91629	0.47078	-4.12321
H	4.48810	-0.72251	-3.16595
H	2.96646	-0.57376	-2.24911
C	5.19646	1.25714	-5.13537
H	6.11430	3.03310	-4.32549
H	4.34223	3.00393	-4.10709
C	-3.92271	-0.19388	-2.74165
C	-3.41582	-1.64668	-2.65364
C	-5.07178	-0.02502	-3.76252
H	-3.06031	0.40454	-3.09660
C	-2.86734	-2.19059	-3.98151
H	-4.21146	-2.32326	-2.28312
H	-2.60017	-1.68242	-1.90480
C	-4.56993	-0.04151	-5.21786
H	-5.86204	-0.79220	-3.60971
H	-5.54748	0.96637	-3.59064
C	-3.99561	-2.58269	-4.94953
H	-2.28237	-3.10763	-3.74934
H	-2.16344	-1.46975	-4.45163
C	-3.80478	3.08062	1.29247
C	-4.94741	4.10501	1.49614
C	-3.07687	2.72537	2.60684
H	-3.05415	3.56467	0.64036
C	-4.42036	5.53945	1.69086

H	-5.60754	3.81190	2.34122
H	-5.57821	4.10878	0.57920
C	-2.42550	3.93920	3.28870
H	-3.76563	2.22035	3.31770
H	-2.27242	1.99459	2.38012
C	-3.45961	4.81340	4.01631
H	-1.71030	3.55238	4.04743
H	-1.83129	4.53640	2.56260
C	-6.86358	-0.96342	1.32486
C	-8.18977	-1.05757	0.53068
C	-6.25415	-2.34010	1.66462
H	-7.07647	-0.45274	2.29040
C	-9.37361	-1.49283	1.41229
H	-8.08853	-1.73435	-0.34546
H	-8.42837	-0.04614	0.13231
C	-7.19126	-3.24572	2.47896
H	-5.93361	-2.86561	0.73926
H	-5.33435	-2.17310	2.26832
C	-8.29481	-3.86541	1.60644
H	-6.57887	-4.07345	2.89733
H	-7.62113	-2.69902	3.34618
C	-9.57420	-3.01638	1.50418
C	-4.48916	-1.44040	-5.85582
C	-4.09058	5.91555	3.14710
C	3.78980	0.67900	-5.37341
C	10.04325	-3.23311	0.03060
C	2.72941	0.52856	6.21288
H	2.15870	2.56238	6.70501
H	3.86631	2.16830	7.03322
H	2.13219	0.26254	7.11248
H	3.67306	-0.05452	6.29103
H	1.19709	0.88355	4.67457
H	1.36210	-0.80895	5.21879
H	8.50611	-3.70689	-1.44051
H	9.28507	-5.11619	-0.69190
H	10.63612	-2.90957	-0.85290
H	10.71673	-3.86029	0.65491
H	10.52300	-1.29217	0.82918
H	9.52255	-2.27284	1.91758
H	2.35434	1.40408	-3.90006
H	2.14635	-0.29184	-4.37424
H	3.90511	-0.29679	-5.89438
H	3.23993	1.34948	-6.06967
H	5.55727	1.67091	-6.10246
H	5.89801	0.43474	-4.87427
H	-5.49620	-1.71717	-6.23794
H	-3.82041	-1.37387	-6.74188
H	-5.26653	0.56710	-5.83530
H	-3.58137	0.46120	-5.29667
H	-4.85627	-3.01028	-4.38974
H	-3.62554	-3.39894	-5.60788
H	-9.27694	-1.06658	2.43446
H	-10.30566	-1.05781	0.98931
H	-10.21402	-3.22546	2.38938
H	-10.14279	-3.35745	0.61137
H	-8.57573	-4.85047	2.03926
H	-7.90924	-4.07945	0.58570
H	-3.52914	5.71999	1.05076
H	-5.19686	6.24927	1.33025
H	-3.40498	6.79108	3.12806

H	-5.02391	6.25349	3.64852
H	-4.26240	4.18167	4.45570
H	-2.95753	5.30966	4.87559

Catalyst (2,4,6-c-Hept, 12)-Enamine protonation TS (pro-R) alternative conformation
Electronic energy (Hartrees): -1870.372036
Free Energy at 1M concentration and 253 K (Hartrees): -1868.535483
Coordinates:

P	0.18474	0.08194	0.08216
O	1.31640	0.86565	-0.78442
O	-1.11454	1.06232	0.21332
O	0.60091	-0.26900	1.47446
[truncated: 123,922 more chars]
